# Exploring the expressiveness of abstract metabolic networks

**DOI:** 10.1371/journal.pone.0281047

**Published:** 2023-02-09

**Authors:** Irene García, Bessem Chouaia, Mercè Llabrés, Marta Simeoni

**Affiliations:** 1 Mathematics and Computer Science Department, University of the Balearic Islands, Palma, Spain; 2 Dipartimento di Scienze Ambientali, Informatica e Statistica, Università Ca’ Foscari Venezia, Venice, Italy; 3 European Centre for Living Technology (ECLT), Venice, Italy; Cardiff’s Metropolitan University: Cardiff Metropolitan University, UNITED KINGDOM

## Abstract

Metabolism is characterised by chemical reactions linked to each other, creating a complex network structure. The whole metabolic network is divided into pathways of chemical reactions, such that every pathway is a metabolic function. A simplified representation of metabolism, which we call an *abstract metabolic network*, is a graph in which metabolic pathways are nodes and there is an edge between two nodes if their corresponding pathways share one or more compounds. The abstract metabolic network of a given organism results in a small network that requires low computational power to be analysed and makes it a suitable model to perform a large-scale comparison of organisms’ metabolism. To explore the potentials and limits of such a basic representation, we considered a comprehensive set of KEGG organisms, represented through their abstract metabolic network. We performed pairwise comparisons using graph kernel methods and analyse the results through exploratory data analysis and machine learning techniques. The results show that abstract metabolic networks discriminate macro evolutionary events, indicating that they are expressive enough to capture key steps in metabolism evolution.

## Introduction

Metabolism is the organism’s machinery that breaks down complex organic molecules to produce the energy and building blocks needed for the organism’s normal functioning. It comprises all chemical and physical processes that occur within the cells of living organisms and that allow for maintaining life. These processes interact with one another, creating a complex network structure. Each process, in turn, is made up of a pathway of chemical or physical reactions, and all the pathways together form the whole metabolic network. Even though all free-living organisms perform essential metabolic functions (e.g., glycolysis, ATP synthesis, nucleic acid synthesis and degradation), the underlying pathways and chemical reactions that carry them out may differ. The variety of habitats led organisms to diversity and adaptation and, in the process, to evolve their metabolic pathways to carry out similar metabolic functions under different ecological niches [1]. Metabolism is shaped not only by the cell’s genome but also by environmental conditions (e.g., nutrient availability, temperature, pH). A particular case is the metabolism of organisms in a mutualistic relationship, where one organism produces what is needed by the other and vice versa [2–5]. Hence, metabolism is not a static and preserved network during the organism’s life history, but a dynamic network that has evolved to adapt to different conditions for the organism’s survival. Consequently, two pivotal questions that need to be addressed arise: how did metabolism and, by extension, metabolic pathways evolve along the tree of life to adapt to changing conditions and habitats? And, what is the minimal metabolic information that makes it possible to recover and reconstruct metabolisms’ evolution?

Literature on metabolomics in the last two decades ranges from the analysis of single pathways [6–9], to the comparative analysis of a set of pathways [10], together with the metabolic networks dynamics [11, 12]. Several approaches analyse topological features of metabolic networks, such as the networks connectivity, centrality, strongly connected components or node degree [13–15]. Moreover, various approaches to metabolic network reconstruction, analysis and comparison have been proposed; see [16–19] for surveys on different approaches and tools.

Metabolism reconstruction and comparison can be automated thanks to the knowledge stored in metabolic databases such as BioCyc [20], BioModels [21] and KEGG (Kyoto Encyclopedia of Genes and Genomes) [22–24]. However, metabolism reconstruction generally requires human intervention since data repositories can be incomplete, heterogeneous and incoherent. Furthermore, the comparison of metabolic networks is computationally challenging due to the huge number of chemical reactions involved in metabolism. A crucial choice that greatly influences metabolism reconstruction and comparison is how a metabolic network is represented. On one hand, the more faithful the representation is, the more reliable the reconstructed metabolism and the comparison results will be. On the other hand, a fine-grain representation demands high computational costs. The approaches in the literature usually look for a compromise between representation accuracy and computational costs.

In this paper, we are interested in exploring the potential of a simplified representation of the metabolism as a graph, where nodes are metabolic pathways, and there is an edge between two nodes if their corresponding pathways share one or more compounds. We call it an *abstract metabolic network* (AMN, for short) since it does not include any information about the single biochemical reactions within each pathway. AMNs are a minimal description of the metabolism that is attractive for two main reasons. The first one is that they are small size graphs compared to other graph-based representations of the metabolism. A mean size metabolic network contains about one thousand reactions, which means having one thousand nodes in the corresponding graph, while the mean number of metabolic pathways is around a hundred, which becomes a hundred nodes in the corresponding AMN. This makes AMNs suitable for large-scale metabolism analysis, as they require a much lower computational effort. The second reason AMNs are interesting is that they allow for metabolism comparison from a novel perspective that can yield new insights in the field. Since AMNs do not take into account the biochemical reactions information, metabolism comparison through AMNs means comparing the topological organisation of the metabolism into functional units, which is rather different than performing the comparison through a detailed reaction-based representation. The effectiveness of AMNs needs to be investigated. However, while it seems reasonable to employ AMNs to verify whether the topological organisation of metabolic pathways played a role in evolution, their use for fine-grain metabolism comparison appears unsuitable.

Similar representations of the metabolism have already been introduced in the literature. In [25], a metabolic network is represented at two distinct levels: a structural level, which corresponds to AMNs, and a functional level, which represents the functional role of each pathway through their reactions. In [26, 27], the authors define the so-called NIP (Network of Interacting Pathways), which are similar to AMNs. However, unlike AMNs, NIP also includes currency compounds (except water) when considering the shared compounds between two pathways. Moreover, NIP edges are weighted with the number of shared compounds, so more information is considered w.r.t AMNs.

The goal of this paper is to explore the expressiveness of AMNs by performing an automatic large-scale metabolism analysis to test whether AMNs are able to discern well-known taxonomic groups. A positive answer would mean that AMNs allow for a first meaningful classification that requires low computational effort. It would also emphasise that evolutionary pressures affected not only genes and protein sequences, but also the topological organisation of the functional units in the cell.

To accomplish our goal, we consider a comprehensive set of organisms whose metabolic data is stored in the KEGG database, an extensive, reliable and widely used resource explicitly designed to present data in a standardised way, a key feature in our approach. We build the AMNs of the KEGG organisms and compare them in pairs by using graph kernels (see e.g. [28] for a survey). Comparing graphs is a difficult task, and graph kernels can be seen as a means to work around the complexity issue since they allow for a workable, although not exact, comparison. They have been widely used in a variety of contexts, and some approaches in the literature apply them to represent and compare metabolic networks, see e.g. [29–31]. Here we explore four different kernels: Vertex Histogram (VH), Shortest Path (SP), Weisfeiler-Lehman (WL) and Pyramid Match (PM). Each of them bases the comparison on specific topological characteristics: VH considers nodes only, SP looks at the shortest paths, WL is based on subtrees comparison, and PM considers the whole graph structure. The comparison results are analysed through exploratory data analysis and machine learning methods. We performed three main tests: the first involved the entire dataset, made of 7141 KEGG organisms. The second focused on Eukaryotes’ subset and also explored each eukaryotic kingdom separately. The third experiment focused on Prokaryotes.

The results demonstrate that AMNs are expressive enough to recognise key steps in metabolism evolution. The four considered graph kernels show similar patterns at higher taxonomical level, i.e. kingdoms, thus suggesting that the signals from the metabolisms’ comparison are clear and robust. In particular, AMNs are able to distinguish domains and kingdoms, although there is no clear separation between Bacteria and Archaea. Within Eukaryotes, we observe a good separation among kingdoms and within kingdoms, further evolutionary signals can be observed.

In addition to these results, the analysis highlights a new paraphyletic clade that we define, for the purpose of this study, as the *Protophotophytes*. They are the photosynthetic eukaryotes (macro and micro) whose photosynthetic capabilities derive from acquiring photosynthetic bacteria during the primary endosymbiotic event. The clade includes plants, green algae, and red algae.

## Methods

**Fig 1:**
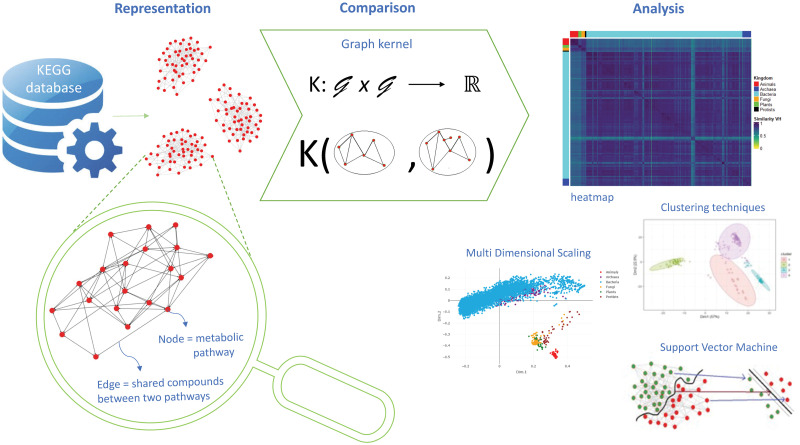
Overall view of the proposed methodology, composed by three steps: *Representation, comparison* and *analysis*.

This section illustrates the details of our methodology to automatically derive AMNs from KEGG metabolic data, compare the metabolisms of different organisms using graph kernels, and analyse the comparison results. Our analytical workflow is composed of three main steps: *representation*, *comparison* and *analysis*. The overall view of the approach is described in Fig 1.

In the *representation* step, we retrieve from KEGG the metabolic pathways of the considered organisms and build their AMN. The latter is defined as a graph where each node represents a metabolic pathway, and each edge between two nodes indicates that they share one or more metabolites. Nodes are labelled with the KEGG’s pathway identifiers, and edges are not labelled. This defines a very high-level and abstract view of the metabolism that does not include any information about the biochemical reactions within each pathway.

In the *comparison* step, the obtained graphs are pairwise compared using graph kernels. Each kernel produces a similarity matrix containing the comparison results. We explored the use of four graph kernels: Vertex Histogram (VH), Shortest Path (SP), Weisfeiler-Lehman (WL) and Pyramid Match (PM). Each of these kernels bases the comparison on specific topological characteristics, but all of them include nodes labels among the features taken into consideration. This means that all kernels evaluate, among the various aspects, the similarity of the sets of pathways present in the two graphs.

In the *analysis* step, we employ exploratory data analysis techniques as well as machine learning methods to analyse the similarity matrices obtained by the kernels. Exploratory data analysis comprises visualisation techniques such as Multi-Dimensional Scaling (MDS) and simple heatmaps to enhance a first qualitative view of the similarity matrices, while clustering techniques, such as k-means and hierarchical clustering, are used to classify the considered organisms and compare the obtained clusters with the taxonomic groups mention. In some experiments, we also used a Support Vector Machine (SVM) as a supervised prediction method to further support the clustering results.

We proceed now by detailing the techniques adopted for the representation, comparison and analysis steps.

### Metabolism representation: Building abstract metabolic networks from KEGG data

Our metabolism representation is based on the metabolic data stored in the KEGG database [22–24]. Data of the 7141 considered organisms was downloaded at the end of December 2021. The complete list of organisms can be found in the Supporting information, see S1 File.

We used KEGG as the sole source of metabolic data. Our approach strictly depends on the data representation and the knowledge available in KEGG, including data incompleteness, inconsistency and biases that could reflect negatively on our comparison method and the subsequent analyses. It is worth highlighting that biases exist in all metabolic databases and cannot be avoided while performing large-scale automated processes. Nonetheless, we can count on the fact that KEGG is a widely known database, whose content is constantly updated based on new knowledge.

The metabolic activity in KEGG is divided into various categories. Each category is then composed of various metabolic pathways. In order to consider the whole metabolic network, all categories and all the corresponding pathways are contemplated. The list of categories and corresponding pathways is available in the Supporting information S2 File.

KEGG associates to each metabolic function a unique *reference pathway* map that is not organism specific and contains integrated information from different organisms. A reference map can be expanded into organisms-specific pathways by combining the gene information in individual genomes. This representation of pathways plays an important role in avoiding incoherence in our metabolism representation and in automatising the whole process.

We represent the metabolic network of a given organism through a graph called *abstract metabolic network* (AMN), whose topology reflects the metabolic pathways stored in KEGG for that organism and their interconnections. Specifically, an AMN is an undirected graph in which each node identifies a metabolic pathway and an edge between two nodes indicates that the two corresponding pathways share one or more compounds among the ones defining their reactions network. Nodes are labelled with the KEGG pathway identifiers and edges are not labelled. Note that a shared compound *C* between two pathways may represent different situations:

- *C* is produced by one pathway and consumed by the other;
- *C* is a compound used in the shared part of two overlapping pathways;
- *C* is a compound present in both pathways, even though they express unrelated functions or work in different environments or conditions.

Ubiquitous compounds such as H_2_O, phosphate, ATP and ADP are not considered, since, though they are needed and assumed to be present everywhere, they are not “representative” compounds of any pathway and their consideration would only add noise to our representation.

Note that all AMNs share the same set of node labels, namely, the set of KEGG’s pathways identifiers. Moreover, in each AMN all labels have at most one occurrence, since there is at most one node for each pathway.

In this representation, all the reactions involved in the various pathway are omitted, and just the minimal information about the presence of pathways and their interconnections is taken into consideration. This clearly results in an abstract and coarse-grain view of the metabolism.

KEGG supplies two related representations for each pathway in its repository: a graphical representation (*pathway map*), showing the pathway content, and a textual one written in an XML format, a *KGML file*, where KGML stands for *KEGG Markup Language*. Such a file contains the information represented in the corresponding map.

KGML files only contain reactions information for pathways that include a chemical network; we call them *chemical pathways*. If a chemical pathway is present in the metabolism of an organism, its corresponding KGML file exists and contains one or more chemical reactions. The pathways based on physical mechanisms, for instance membrane pathways, do not contain information on reactions; we call them *physical pathways*. If a physical pathway is present in the metabolism of an organism, its corresponding KGML file exists but do not contain any chemical reactions.

To automatically reconstruct the metabolism of a specific organism as an AMN, it is necessary to download the KGML files of all pathways of the organism through the public KEGG’s APIs [32], and parse each file to extract the relevant information for our representation. The resulting graph is then stored locally. We implemented a specific command-line Java program that performs the entire job.

### Comparison through graph kernels

Graph kernels can be intuitively understood as functions measuring the similarity of pairs of graphs. Given two graphs in input, a graph kernel calculates a real number that measures their similarity according to some graph’s features. More in general, given a set of *n* graphs in input, the kernel’s output is an *n* × *n* similarity matrix where each cell (*i*, *j*) contains the result of the pairwise comparison between graphs *i* and *j*. In our case the matrix is normalised, that is, all similarity values are real numbers in the interval [0, 1], where 0 and 1 indicate the minimum and maximum similarity values, respectively. The matrix is symmetric and the main diagonal contains only 1 values, each cell being the comparison of a graph with itself.

We used four different kernels to compare the abstract representation of metabolisms, all implemented by the GraKel Python library [33]. Since nodes in AMNs are labelled with the KEGG pathways identifier, we decided to exploit such information in the comparison. For this reason, the selected kernels include node labels among the features taken into consideration for the comparison.

A formal introduction on graph kernels can be found, e.g., in [28, 34–38]. Here follows a brief description of the four kernels employed in this paper, while in the Supporting Information we provide their detailed mathematical formalisation, see S3 File.

- **Vertex Histogram**: This is the simplest kernel. It compares node-labelled graphs and it only considers the node labels of the graphs to compare. Given a set of node labels L, the vertex label histogram of a graph counts, for each label i∈L, the number of nodes in the graph that are labelled by *i*. In the particular case of a labelled graph being the AMN of a given organism, every node has only one label and different nodes have different labels. Therefore, this kernel only considers the presence or absence of pathways (node labels) in the two organisms under comparison and sum up the number of common pathways. Since only nodes and their labels, and not edges, are considered, the way metabolites are exchanged between pathways does not affect the comparison result.
- **Shortest-Path**: This kernel compares graphs in terms of their shortest paths. Intuitively, this means measuring how easy or difficult it is to connect, through shared compounds, couples of pathways in the two AMNs. It first computes the *shortest-paths graph* of every input graph, that is, a weighted graph with the same nodes as the original graph and with weighted edges corresponding to the shortest paths between each pair of nodes. The comparison is then performed by considering each pair of shortest paths in two given graphs and comparing their length and the labels of their endpoint nodes. The resulting scores are summed up to form the final similarity score.
- **Weisfeiler-Lehman (WL) subtree kernel**: Roughly speaking, this kernel compares the subtrees rooted on each node of the two graphs under consideration, taking into account also their node labels: the more the subtrees structures and labels coincide, the higher is their similarity. The fundamental idea of the Weisfeiler-Lehman algorithm is to replace the label of each node by a multiset of labels consisting of the original label of the node and the sorted set of labels of its neighbours. The resultant multiset is then compressed into a new short label, which reflects the knowledge of the node and its neighbourhood. This relabelling process is then repeated for *h* iterations. By performing this procedure simultaneously on all input graphs, it follows that two nodes from different graphs will get identical new labels if and only if they have identical multiset labels. The kernel function, in this case, compares the node labels of the graphs resulting after each iteration and summarises the comparison with a real number. It can be shown that this is equivalent to comparing the number of shared subtrees between the two input graphs (the kernel considers all subtrees up to height *h*).
- **Pyramid Match**: Intuitively, this kernel measures the similarity of the topological features (e.g. connectivity) of the nodes with the same label in the two compared graphs. Described at a high level, the Pyramid Match graph kernel first embeds the graph nodes into a vector space by considering the eigenvectors of the *k* largest in magnitude eigenvalues of the adjacency matrix of the graph. Thus, the nodes become points of a *k* dimensional hypercube. Next, to assess the similarity of two sets of nodes, the kernel maps these points into multi-resolution histograms and compares them with a weighted histogram intersection function. In the case of labelled graphs, the kernel restricts the matchings to occur only between nodes that share the same labels.

To conclude, we remark that the great advantage of graph kernels is that they provide a computationally feasible way to compare graphs. We exploit this characteristic throughout the paper to perform large-scale comparison of thousands of AMNs in a few minutes. However, we also want to point out that graph kernels do not provide insights on the agreements or disagreements of the input graphs, i.e, they do not allow for recovering the nodes and edges of the input graphs that are responsible for the obtained similarity result.

### Data visualisation with heatmaps and multi-dimensional scaling

A heatmap allows for a visual rendering of the similarity matrix produced by graph kernel. Each cell (*i*, *j*) in the heatmap shows the similarity value between the *i*-th and *j*-th AMNs, colour-coded so that darker colours correspond to a high degree of similarity (from dark blue to yellow). In our case the heatmaps are symmetric and cells in the main diagonal always show the darkest colour, resulting from comparing an organism with itself. As it is a challenge to annotate rows and columns with the KEGG identifier of each organism, a detailed view of the data is difficult to obtain. We used coloured bars on rows and columns to indicate the various taxonomic groups of organisms to ease this difficulty.

A different technique is Multi-dimensional Scaling (MDS), a classic multivariate data analysis technique that allows for obtaining a low-dimensional representation of the observed similarities [39]. Given a normalised similarity matrix **Q** produced by a graph kernel, we implemented a metric MDS by transforming **Q** into a distance matrix with elements

$$\begin{array}{c} d_{ij}^{2}=q_{ii}+q_{jj}-2q_{ij}=2\left(1-q_{ij}\right), \end{array}$$ (1)

where *q*_*ij*_ is the element in row *i* and column *j* in **Q** and *q*_*ii*_ = *q*_*jj*_ = 1.

The objective of the MDS is to represent the observed distances using a set of variables *y*_1_, …, *y*_*k*_, where *k* < *n*, such that the Euclidean distances between the coordinates of the elements for these variables are close to the original distances. In this way, the graphical representation in *k* dimensions will faithfully reproduce the observed structure. We executed the metric MDS using the *R* package [40], a language and environment for statistical computing.

### Clustering techniques

We used clustering techniques on the similarity matrices produced by graph kernels to identify patterns or groups of similar organisms. In particular, we used the *R* package to perform partitioning clustering methods, such as k-means, and agglomerative hierarchical clustering [41].

K-means clustering divides the dataset into *k* subsets, where *k* is pre-specified. The algorithm finds the best *k* groups, the best being those where the within sum of square (WSS) is the smallest possible and where the sum of squares between clusters is the largest. Each cluster is represented by the mean of the data points belonging to the cluster itself. We specified the number of clusters both according to the phylogeny and using the so-called Elbow method [39]. The Elbow method consists in plotting the value of the WSS produced by different values of *k*. The bend (elbow) location in the plot is generally considered an indicator of the appropriate number of clusters.

Agglomerative hierarchical clustering is an alternative approach that does not require specifying the number of clusters to be generated. We calculated the distance between hierarchical clusters with complete linkage: *d*(*C*, *C*′) = *max*{*d*(*a*, *b*)|*a* ∈ *C*, *b* ∈ *C*′}.

The result of hierarchical clustering is a tree-based representation of the objects; the visualisation is shown through a graph known as a dendrogram.

### Support Vector Machine

Support Vector Machine (SVM) is a well-known supervised learning method. The basic configuration works with two classes and considers linearly separable problems: data points that a hyperplane can separate. Considering data as n-dimensional labelled points, SVM searches the hyperplane separating points of different classes, maximising the distance between the boundary and data points. We use SVM as implemented in the *Scikit learn* library [42].

To evaluate the SVM prediction ability, we use cross-validation. Roughly speaking, given a dataset of known data (the *training set*), *k*-fold cross-validation divides the dataset into *k* partitions and, for *k* times, one partition at a time is used as a validation set, while the rest of the dataset remains as the training set. Prediction indexes are measured at each iteration and, in the end, the mean value of such measures will be the final prediction results. A modified version called *stratified* *k*-fold cross-validation allows for dealing with imbalanced datasets by preserving the imbalanced class distribution in each fold, thereby ensuring that each class is suitably represented. In this work we use stratified 5-fold cross validation.

Finally, we use *balanced accuracy* as a measure for prediction model evaluation, since it avoids inflated performance estimates on imbalanced datasets. It is defined as the fraction of the correct predictions, where each sample is weighted according to the inverse prevalence of its true class. Its values range between 0 and 1, with the best value 1. In our tests, however, we report the value as a percentage.

### Phylogenetic tree comparison

For the phylogenetic reconstruction of the *Pseudomonas* genus, the 121 complete genomes present in KEGG were downloaded from the NCBI database. Open reading frames (ORFs) were extracted, and all the nontruncated proteins longer than 50 residues were kept for the following analysis. Orthologs present in a single copy in all the genomes were then selected using OrthoFinder (ver 2.3.3) [43]. Three hundred and thirty proteins were kept for phylogenetic analysis. The amino acid sequences of the coding sequences (CDS) belonging to each ortholog family were aligned using MUSCLE (ver 3.8.31) [44]. For each of the aligned CDS, an evolutionary model was predicted using PartitionFinder 2 (ver 2.1.1) [45]; all the protein sequences were then concatenated and their phylogenetic tree constructed with iQtree 2 (ver 2.1.2) [46] using a partitioned Maximum Likelihood model that takes into account the evolutionary model predicted for each of the CDS. The phylogenetic trees were tested with 1,000 ultrafast bootstraps [47].

The distance matrices generated during the previous analyses were used to generate cladograms using the hierarchical clustering and converted to a phylogenetic object to be compared to the phylogenetic tree using the *R* package *ape* (ver 5.5) [40, 48]. To make it easier to visually compare the phylogenetic tree and the clustering based on the generated distance matrices, the connecting lines were coloured. The colours were assigned based on the position of the clusters within the phylogenetic tree. In particular, blue was assigned to the deepest branching cluster followed, in order, by turquoise, green, yellow, orange and finally red, which was assigned to the most recent branching cluster.

## Results

This section presents the results of comparing the metabolisms of different species, represented as AMNs, using graph kernels. The various experiments were carried out on the entire dataset as well as on different subsets of organisms. In particular, in the first experiment, we compared the metabolisms of the entire dataset, consisting of 7141 organisms, 687 Eukaryotes and 6454 Prokaryotes, stored in KEGG. In the second experiment, we considered all the Eukaryotes and later, we also focused on each kingdom separately: Animals, Plants and, by extension, Protophotophytes, Fungi and Protists. The third test focused on Prokaryotes.

The larger experiment, considering the whole dataset, ran in less than 86 seconds for VH and WL kernels, less than 7 minutes for SP and about 34 minutes for PM on a standard laptop, which reinforces the argument that AMNs and graph kernels are effective, from a computational point of view, to perform comparative analysis of large metabolomic datasets. The smaller experiments, i.e. Animals, Protophotophytes, Fungi and Protists, ran in a few seconds for all kernels.

### First experiment: Whole dataset analysis

This experiment considers the metabolism of 7141 organisms stored in the KEGG database. The set comprises 370 Animals, 127 Protophotophytes, 138 Fungi, 52 Protists, 6115 Bacteria and 339 Archaea. The AMNs of all organisms were pairwise compared using the VH, SP, WL and PM graph kernels.

**Fig 2:**
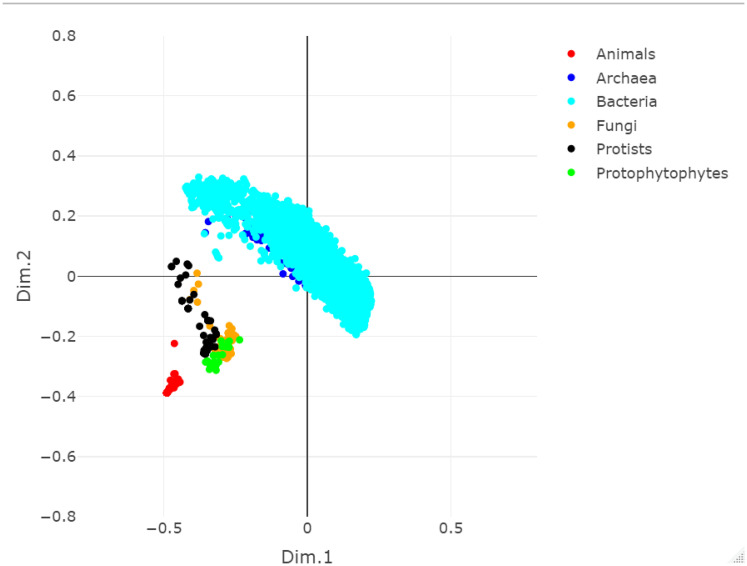
MDS plot of the VH similarity matrix of the whole dataset at the kingdom level.

Fig 2 shows the MDS plot of the VH similarity matrix. Looking at the plot, it is evident that the VH graph kernel is able to separate Eukaryotes and Prokaryotes. Within Eukaryotes, the various kingdoms can also be distinguished and Animals are clearly separated from the rest of the Eukaryotes. Instead, within Prokaryotes, Bacteria and Archaea cannot be clearly distinguished. A pattern similar to the one shown in Fig 2 can also be observed in the similarity matrices of the SP, WL and PM graph kernels, whose heatmaps and MDS plots can be found in the Supporting Information, see S4 File. However, VH for this experiment shows the best separation between domains and between Animals and the rest of the Eukaryotes kingdoms, suggesting that the simple comparison performed by the kernel gives already a clear signal at this level.

We also applied k-means clustering to the MDS data. The MDS transformation allows for a faithful representation of the original data and, at the same time, reduces the noise of the data themselves. Table 1 shows the 6-means clustering results (6 being the different kingdoms) obtained from the MDS data. Notice that all kernels separate Eukaryotes and Prokaryotes. In particular, Eukaryotes fall into two clusters, one homogeneous cluster for Animals and the other for the remaining kingdoms (up to some outliers in Protists or Fungi). At the same time, Prokaryotes are divided into the remaining clusters without a clear separation between Bacteria and Archaea. Further details on the organisms belonging to each cluster can be found in the Supporting information, see S4 File.

**Table 1:** Whole dataset experiment, kingdom level: Clustering results for kernels VH, SP, WL and PM starting from the MDS data.

Kingdom	VH clusters						SP clusters						WL clusters						PM clusters					
	1	2	3	4	5	6	1	2	3	4	5	6	1	2	3	4	5	6	1	2	3	4	5	6
Animals	0	0	1	0	0	369	1	0	4	0	0	365	0	0	19	0	0	351	3	0	0	367	0	0
Protophotophytes	0	0	127	0	0	0	0	0	127	0	0	0	0	0	127	0	0	0	127	0	0	0	0	0
Fungi	0	0	138	0	0	0	7	0	131	0	0	0	2	0	136	0	0	0	133	5	0	0	0	0
Protists	0	0	47	5	0	0	15	0	37	0	0	0	1	3	48	0	0	0	42	10	0	0	0	0
Bacteria	2501	1583	0	559	1472	0	683	1425	0	1695	2312	0	1683	430	0	2441	1561	0	0	632	2553	0	1528	1402
Archaea	0	100	0	13	226	0	60	96	0	180	3	0	291	4	0	0	44	0	0	12	5	0	174	148

However, at the kingdom level, only Animals are clearly separated, meaning that, within this wide dataset of organisms, their AMNs are significantly different w.r.t. to the others. It is also interesting to observe that the WL kernel is the only kernel able to cluster almost all Archaea within one group (cluster 1).

The SVM prediction analysis applied to the kernel data, see Table 2, improves the clustering results. Looking on the left side of the table (Discerning Eukaryotes/Prokaryotes), the high values of the balanced accuracy, above 98% for all kernels, confirms their ability in separating organisms based on the presence or absence of a nucleus. Furthermore, looking at column three of Table 2 (Discerning Kingdoms), it turns out that SVM analysis separated the different organisms also at the kingdom level, accurately assigning each organism to its kingdom, being the evaluation measure above 97% in all cases.

**Table 2:** Whole dataset test: Evaluation of the SVM results for discerning Eukaryotes/Prokaryotes and Kingdoms.

Kernel	Discerning Eukaryotes/Prokaryotes Balanced Accuracy	Discerning Kingdoms Balanced Accuracy
VH	99.93%	98.63%
SP	98.47%	97.42%
WL	99.05%	97.04%
PM	99.18%	97.36%

### Second experiment: Eukaryotes analysis

In this section, we present the analyses concerning the Eukaryotes domain. We first consider all the 687 Eukaryotes together and test whether AMNs, compared through the VH, SP, WL and PM graph kernels, allow for discerning the various kingdoms: Animals, Plants (more precisely Protophotophytes), Fungi and Protists. Subsequently, we consider each kingdom separately and evaluate the extent to which they can differentiate the organisms based on their respective phylum or class. We do not perform SVM prediction for these lower taxonomy level tests since some classes may contain a few organisms (sometimes just one), rendering the cross-validation process unusable.

#### Discerning the eukaryotic kingdoms

**Fig 3:**
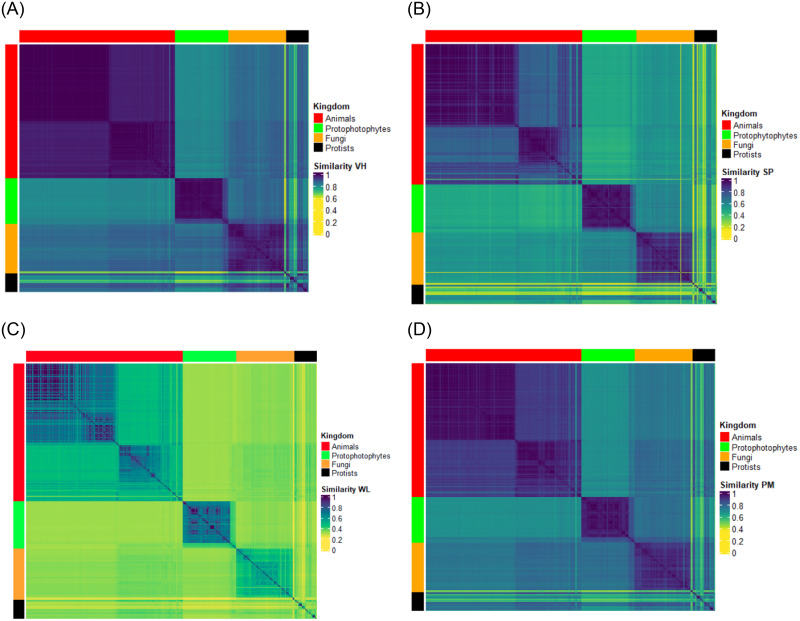
Heatmaps of the similarity matrices of KEGG Eukaryotes at the kingdom level: (a) Vertex Histogram; (b) Shortest Path; (c) Weisfeiler-Lehman; (d) Pyramid Match.

In this experiment, we consider all the Eukaryotes, represented through their AMNs, and use the VH, SP, WL and PM graph kernels to test if the Eukaryotes kingdoms can be distinguished. Fig 3 shows the heatmaps obtained by the four kernels for this test. We remind that, in the heatmaps colours range from dark blue, meaning high similarity, to yellow, indicating low similarity. The coloured bars beside rows and columns show the different kingdoms: Animals in red, Protophotophytes in green, Fungi in orange and Protists in black.

We observe a good separation among Animals, Protophotophytes, and Fungi in all heatmaps. Protists are apart, but they are generally closer to Fungi than to the rest of Eukaryotes. Also, in all heatmaps, Animals are well separated from the other kingdoms and show a further internal pattern that deserves further analysis. It is also worth noticing that all kernels highlight a pronounced difference between Animals and Protophotophytes. Animals are closer to Fungi than Protophotophytes, and, in turn, Protophotophytes are closer to Fungi than to Animals. The heatmaps view of the similarity matrices is also confirmed by the MDS plots that can be found in the Supporting Information, see S5 File.

Looking again at the heatmaps in Fig 3 we can also observe that the VH’s heatmap offers a more definite and compact view of the various taxonomic groups, while the other three kernels show higher intra- and intergroup variability. For instance, the internal pattern within the Animal kingdom is less evident in VH than in the other kernels. Such higher variability indicates a major ability in capturing the differences, which may also result in noise in identifying and separating the four kingdoms. Clearly, the different abilities of the four kernels are strictly related to the different features they involve in the comparison.

We applied a 4-means clustering analysis to the kernels results, four being the different kingdoms of the organisms considered in this test. Table 3 shows how the organisms of the four kingdoms are distributed into four clusters. Details on the organisms belonging to each cluster can be found in the Supporting Information, see S5 File.

**Table 3:** Eukaryotes test, kingdom level: Distribution of Eukaryotes into four groups for every graph kernel.

Kingdom	VH clusters				SP clusters				WL clusters				PM clusters			
	1	2	3	4	1	2	3	4	1	2	3	4	1	2	3	4
Animals	1	0	0	369	0	0	3	367	0	212	157	1	0	0	368	2
Protophotophytes	0	119	8	0	0	127	0	0	113	0	0	14	9	118	0	0
Fungi	5	0	133	0	131	0	7	0	0	0	0	138	132	0	0	6
Protists	21	0	31	0	14	0	35	3	0	0	0	52	26	0	1	25

Looking at Table 3 we can observe that Animals form homogeneous groups in all kernels, one cluster for VH, SP and PM (up to a few outliers), and two clusters for WL, meaning that for WL distinguishing the two subgroups of Animals is more relevant than distinguishing the other kingdoms. Morevover, in WL Animals are correctly split into Vertebrates and Invertebrates, up to a few outliers. Also, Protophotophytes form a big homogeneous group in all kernels, the outliers being Green and Red algae (i.e. unicellular organisms), which fall into another cluster in VH, WL and PM. Fungi are also grouped in one cluster by all kernels, with a few outliers (Microsporidians). However, Fungi always share their cluster with Protophotophytes (algae) and/or Protists. Accordingly, Protists are always mixed with Fungi and Protophotophytes (algae) and even split into two clusters in VH and PM, and three in SP.

Although all kernels give an interesting and meaningful classification, VH, SP and PM perform better at kingdom level: Animals, Protophotophytes and Fungi have their own cluster while Protists are split and mixed with Fungi and algae. However, WL has the advantage of keeping all Protists together in one group and offer the interesting viewpoint of evaluating more the Animals differences (Vertebrates vs. Invertebrates) than the kingdom boundaries.

The SVM prediction analysis also supports these results. In Table 4 we report the balanced accuracy obtained by each kernel: accuracy is above 95% in all cases.

**Table 4:** Eukaryotes test: Evaluation of SVM prediction results for discerning kingdoms.

Kernel	Balanced Accuracy
VH	96.93%
SP	96.61%
WL	95.68%
PM	96.26%

#### Discerning animals

**Fig 4:**
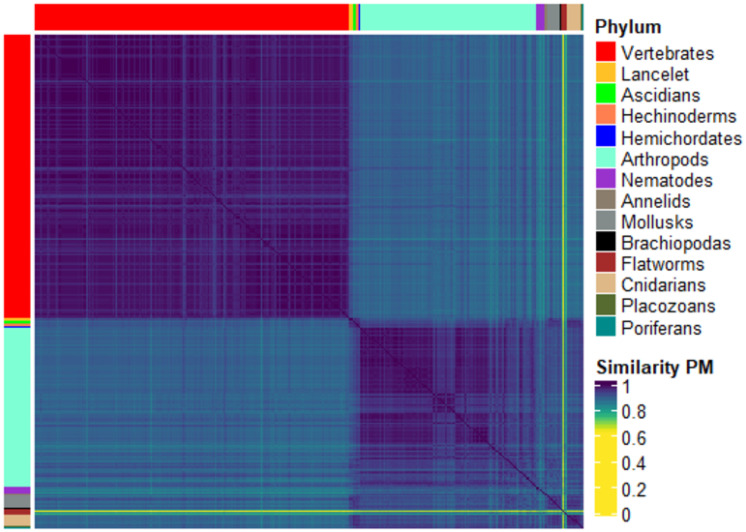
Heatmap of the PM similarity matrix of all KEGG Animals (left) and composition of clusters w.r.t. a total of four clusters for the PM graph kernel.

This experiment focuses on the Animals kingdom and tests whether AMNs, compared through the considered kernels methods, are useful to classify organisms at a lower taxonomic level, specifically at the Phylum level. Fig 4 shows the heatmap of the PM similarity matrix (on the left) and the corresponding 4-means clustering (on the right), *k* = 4 being the optimal number of clusters, as suggested by the Elbow method [39].

Looking at the heatmap in Fig 4, it is evident that the PM kernel does not allow for distinguishing the various Phyla, highlighted in the matrix by the two coloured bars on rows and columns. However, it separates Vertebrates and Invertebrates, and, within Invertebrates, almost all Arthropods are discernible. Finally, it can be noticed that two organisms appear to be far apart from all other Animals (see the light lines in the bottom part of the rows and, by symmetry, right part of the columns). This could be due to (temporarily) incomplete data for those organisms in the KEGG database. The k-means results reflect the main pattern of the heatmap. There are two homogeneous clusters: cluster 1 containing all and only Vertebrates and cluster 4 containing 97 Arthropods (almost all KEGG Insects) out of 119. Cluster 3 includes the remaining Animals, up to the two outliers that fall into cluster 2.

The other kernels also confirm the results shown here for the PM kernel. We refer to the Supporting Information for the VH, SP and WL heatmaps, MDS and k-means results, see S6 File.

In order to investigate whether AMNs are able to classify at a lower rank of taxonomy, we performed a further test focusing on Vertebrates only. The heatmaps obtained by the four kernels can be found in the Supporting Information, see S7 File. While VH, SP and PM do not provide any meaningful classification, WL is able to distinguish two main groups, roughly corresponding to Fishes and Mammals.

It is noteworthy that, at this level of taxonomy, the four kernels have different classification abilities. Nonetheless, there is a trend that using more features, like WL and PM, allows for a relatively better ability to differentiate between the groups. But, as evidenced by the different performance of the two kernels, the features considered by the kernels also affect their classification ability.

#### Discerning protophotophytes

**Fig 5:**
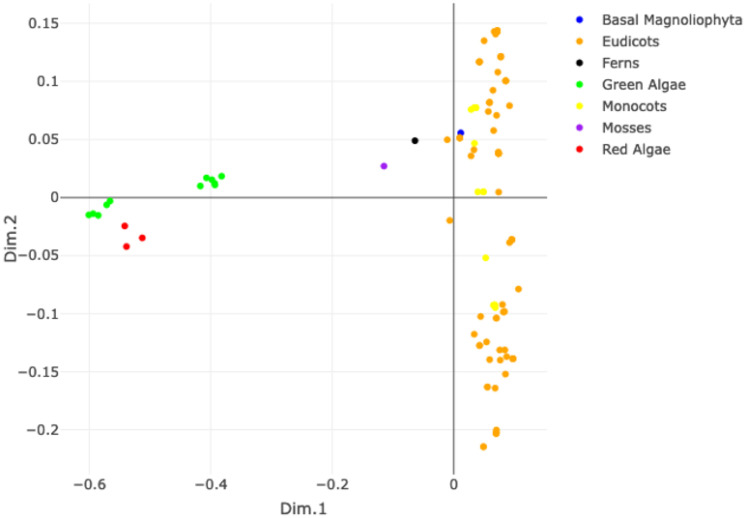
MDS plot of the SP similarity matrix of all KEGG Protophotophytes (left) and composition of clusters w.r.t. a total of three clusters for the SP graph kernel.

This experiment considers all the KEGG “Plants” (more precisely Protophotophytes). Their AMNs are compared using the four kernels to see whether the internal structure of the kingdom can be recognised. Fig 5 shows the MDS plot (on the left) and 3-means clustering (on the right) resulting from the SP graph kernel. The MDS highlights that Algae (red and green dots) are well separated from the other Protophotophytes, which instead appear to be rather mixed together. The k-means clustering also confirms this pattern: cluster 2 contains all Algae, while the other plants fall into the other two clusters without a clear separation. The other kernels show similar results, as illustrated in the Supporting Information, see S8 File.

#### Discerning Fungi

**Fig 6:**
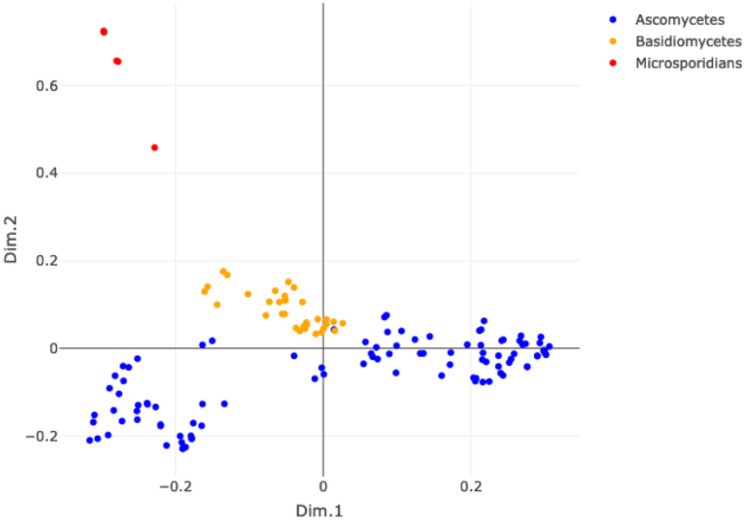
MDS plot of the WL similarity matrix of the KEGG Fungi (left) and composition of clusters w.r.t. a total of three clusters for the WL graph kernel.

This experiment considers the KEGG Fungi and examines whether their AMNs, compared through the kernel methods, can identify further differences inside the kingdom. Fig 6 shows the MDS plot (on the left side) obtained by the WL kernel and the Fungi classification at the phylum level (on the right side). We can observe that Microsporidians (red dots) are separated from the other taxa and that Ascomycetes (blue dots) form two separated groups.

On the right panel of Fig 6, the 3-means clustering confirms the MDS observations. Microsporidians form a homogeneous group (cluster 2) and Ascomycetes are divided in two different groups. Cluster 1 contains all Basidiomycetes and part of the Ascomycetes (namely Saccharomycetes, a class of Ascomycetes), while the rest of Ascomycetes fall in cluster 3. The other graph kernels show similar results, we refer to the Supporting Information for further details, see S9 File.

#### Discerning Protists

**Fig 7:**
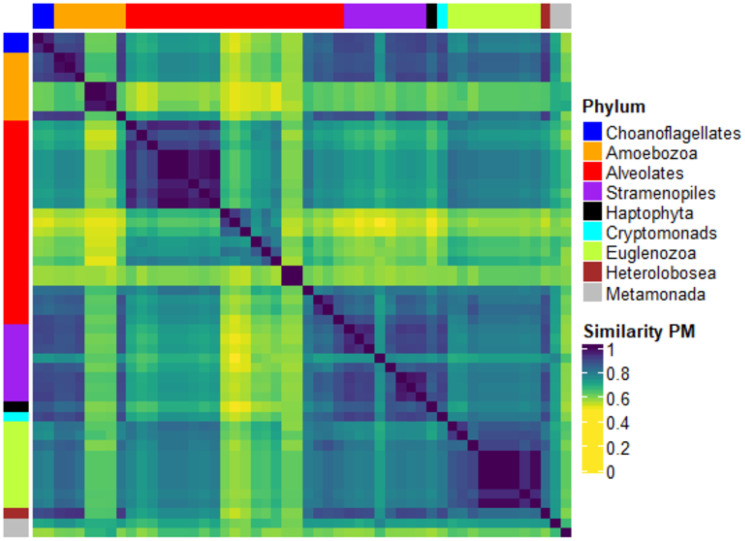
Heatmap of the PM similarity matrix of the KEGG Protists (left) and composition of clusters w.r.t. a total of four clusters for the PM graph kernel.

In this last experiment on the Eukaryotes domain, we consider the KEGG Protists. Fig 7 shows the heatmap (left) and k-means classification (right) obtained by the PM kernel.

We observe that the Protists similarity matrix does not contain a clear pattern. This is also reflected in the k-means clustering, where all Euglenozoa are grouped in a homogeneous cluster (cluster 1). This is true also for part of the Alveolates (cluster 4), but the remaining ones are divided into clusters 2 and 3. The other kernels show similar results, as illustrated in the Supporting Information, see S10 File.

### Third experiment: Prokaryotes analysis

**Fig 8:**
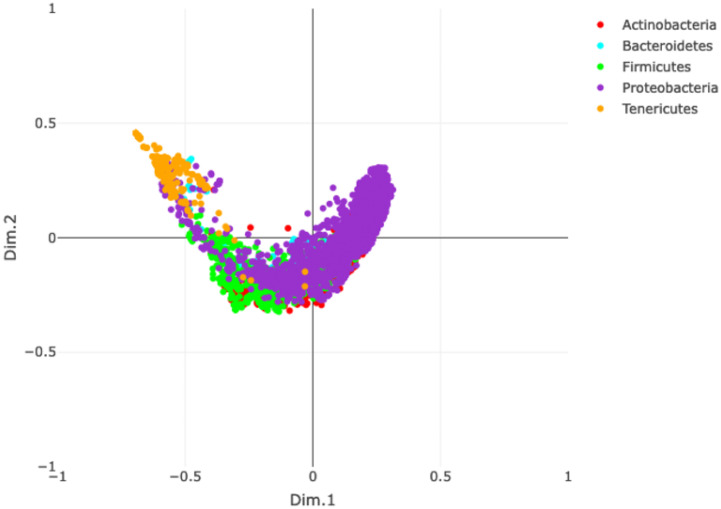
MDS plot of a selected set of bacteria at phylum level, obtained by the SP graph kernel.

When looking at Prokaryotes in Fig 2, showing the MDS plot of the whole dataset of organisms, it is evident that no clear pattern separates Bacteria from Archaea. In addition, even within Bacteria, no clear pattern separating the different clades at specific taxonomic levels (i.e., Phylum, order) were observed, as reflects Fig 8, which shows the MDS plot of the SP graph kernel for a selected set of Bacteria (i.e. all the Actinobacteria, Bacteroidetes, Firmicutes, Proteobacteria and Tenericutes). Looking at Fig 8, only Tenericutes appear to be slightly recognizable w.r.t the other clades. Similar plots were obtained by the other graph kernels, the complete analysis can be found in the Supporting Information, see S11 File.

#### *Pseudomonas* analysis at species level

**Fig 9:**
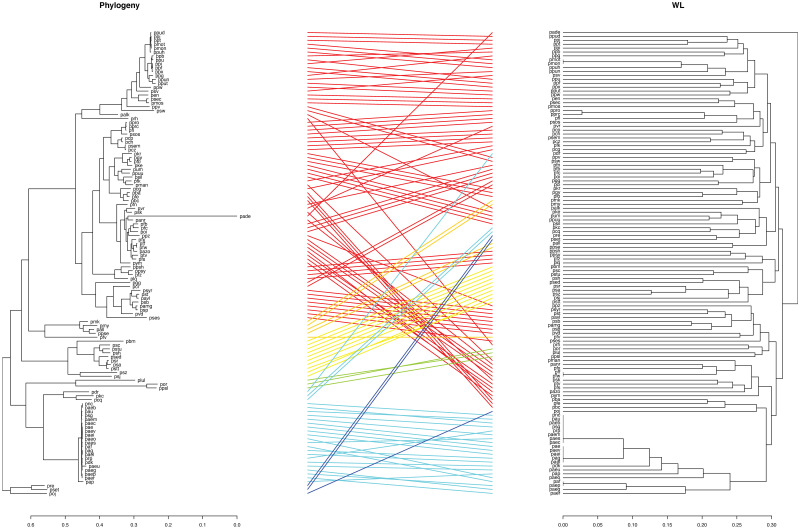
Comparison between *Pseudomonas* phylogeny and complete-linkage hierarchical clustering obtained with the WL graph kernel. The lines colour legend is as follows. The colours were assigned based on the position of the cluster within the phylogenetic tree. Blue was assigned to the deepest branching cluster followed in order by turquoise, green, yellow, orange and, finally, red which was assigned to the most recent branching cluster.

In order to analyse the usefulness of AMNs at a lower rank of taxonomy, we considered the bacteria in the genus *Pseudomonas*. More precisely, we compare directly the gene-based phylogeny of the 120 *Pseudomonas* strain available in KEGG with the dendrogram resulting from applying the complete-linkage hierarchical clustering to the kernels similarity matrices. The best result is shown in Fig 9: the gene-based phylogeny (below) is compared with the WL kernel dendrogram (above).

Looking at Fig 9, we observe that different clusters are relatively well-matched between the phylogeny and the clustering derived from the WL graph kernel. The species cluster of *P. aeruginosa* (turquoise lines) is relatively well-preserved in the complete linkage dendrogram, and the same is for clusters comprising respectively *P. stutzeri* (yellow lines) and *P. putida* (red lines next to the yellow ones).

The VH, SP and PM kernels results can be found in the Supporting Information, see S12 File. The various kernels perform differently in this experiment and even for the best case shown here, the comparison did not result in a perfect match between the WL dendrogram and the phylogeny. It is also noteworthy that VH gives the worst result in this test. This supports the fact that, at lower taxonomical levels, looking only at nodes and node labels does not allow for a meaningful comparison.

## Discussion

In this section, we discuss the results of the various experiments reported in the previous section from a biological and evolutionary viewpoint. We are aware that our analyses could be biased due to the use of KEGG. For instance, KEGG annotation and pathways reconstruction, and also how some pathways are included may be artefactual because of how KEGG assign genes/reactions to pathways. Nonetheless, the general agreement of all graph kernels on the results of the performed experiments suggests that the signal coming out of the metabolisms’ comparison is robust. In addition, we repeated the analyses after removing the pathways harbouring only one (analysis with threshold 1) or at maximum two reactions (analysis with threshold 2), see S13 File. Such changes affected the results of the experiments only in a marginal manner, and the clustering patterns were highly similar.This further confirms the approach’s robustness.

Graph kernels take as input two graphs and provide as output a real number measuring their similarity. They do not allow recovering the nodes and edges of the input graphs that are responsible for the similarity result. As such, the nature of the analyses does not allow us to identify the specific pathways within the AMNs that drive the clustering we observe. Nonetheless, it is still possible to link the clustering to macroevolutionary events. In this respect, as discussed below, it turns out that the clustering pattern is meaningful from the biological and evolutionary viewpoints, thus suggesting that both the AMN representation and the applied computational techniques are appropriate for comparing and analyzing the organisms’ metabolism. We proceed now by discussing the results obtained in every experiment reported in the previous section.

### First experiment: Whole dataset analysis

The clustering results obtained in the whole dataset experiment, see Table 1, show a clear separation between the AMNs of Prokaryotes and Eukaryotes. This separation is consistently observed with the different analyses. From an evolutionary viewpoint, these results are in accordance with the transition from a simple to a more complex cellular organization. This transition consists of the appearance of a defined nucleus and more developed organelles. It is noteworthy that this separation does not reflect the genic and genomic organization since some Archaea present genomes containing introns [49–51]. On the other hand, a defined nucleus and organelles led to a reorganization of parts of the metabolism (e.g., compartmentalization of specific metabolic pathways, transport of metabolites between the different compartments). This reorganization impacted the structure of the metabolic network at a higher level [52, 53]. The fact that there is no clear separation between Bacteria and Archaea suggests that the AMNs of both taxa present similar structuring, with the differences spreading along a continuum, probably impacted by the environment.

### Second experiment: Eukaryotes analysis

In this experiment, we first considered all the Eukaryotes together and then each kingdom separately. In the first case, we tested whether AMNs, compared through the VH, SP, WL and PM graph kernels, allow for discerning the various kingdoms: Animals, Plants (more precisely Protophotophytes), Fungi and Protists. As shown in Fig 3, there is a good separation among Animals, Protophotophytes, and Fungi in all heatmaps. Protists are apart, but they are generally closer to Fungi than to the rest of Eukaryotes. This clustering pattern fits the evolutionary pattern of the appearance of multicellularity, a milestone in the evolution of complex organisms [54, 55]. In addition to multicellularity, nutritional needs (i.e., autotrophy and heterotrophy) also influence the observed clustering patterns. Animal, Protists and Fungi are heterotrophs, while Protophotophytes are autotrophs. On the other hand, Fungi and Protists are mainly unicellular, with some species having a multicellular phase during their life cycle. Animals, on the contrary, are always multicellular. Animals have also undergone some physiological adaptation that further differentiated them from the other autotrophic Eukaryotes [56].

Protophotophytes can be either multicellular (e.g., land plants) or both (e.g., green algae) [57]. The difference between strictly multicellular plants and other photosynthetic eukaryotes is further detailed in Fig 5, where algae cluster separately from the other Protophotophytes. This clustering also reflects the evolutionary origin of land plants from a single clade of freshwater unicellular algae [58, 59].

The fact that some Protists are sometimes clustered with Protophotophytes can be retraced to secondary endosymbiotic events in which unicellular non-photosynthetic eukaryotes engulfed photosynthetic eukaryotes (namely ancestral algae) that later evolved into plastids. These plastids conferred onto the Protists that acquired them some metabolic pathways characteristic of photosynthetic organisms. The acquisition of these pathways sets these protists closer to Protophotophytes in terms of metabolic capabilities and network structure.

**Animal kingdom.** Focusing on Animals, we observe that the results shown in Fig 4 can be explained by the evolutionary paths taken by the different taxa, which reflect on the structure and organization of their respective AMNs. From the evolutionary point of view, Animals were separated along important evolutionary milestones. We observe that Vertebrates (i.e., Amphibians, Birds, Fishes, Mammals, and Reptiles) are clustered separately from the other groups, thus separating Metazoan with a skeleton from the rest of the animals. Skeleton represented a critical evolutionary step in response to predation and allowed those animals which had it to grow bigger [60]. To produce the building block of skeletons, vertebrates, unlike other Metazoans, can metabolise calcium phosphate, which sets them apart from other animals [61]. On the other hand, within the non-vertebrates, we observe that Insects are separated from the other organisms. This separation can be traced to the development of a rigid external hydrocarbon cuticle to support the body, thus allowing them to colonise dry land. Although insects share several traits with other arthropods (e.g., outer cuticle, process atmospheric oxygen), their ability to fly and the evolutionary and metabolic adaptation that comes with it sets them apart from the other non-vertebrates.

Test performed on Vertebrates only, (see S7 File, show that WL is able to distinguish two main groups, roughly corresponding to Fishes and Mammals.Fishes and Mammals usually live in completely different habitats (i.e. water and land, respectively), which impact their respective water metabolism and the physiological mechanisms they have evolved to maintain water homeostasis. Moreover, Mammals, in addition to Birds, are the only endotherms. The latter physiological feature requires the evolution of the pathways to metabolically produce heat, which are absent from fishes.

**Protophotophytes.** The analyses on this clade, see Fig 5 confirm the differences in network structure between Plants and algae already observed in the test considering all the Eukaryotes together. Although both groups are photoautotrophic organisms, land plants originated from a single clade of freshwater unicellular algae [58, 59]. In addition to their evolutionary origin, land plants further specialised and adapted to an environment where liquid water is less available [62–65]. We hypothesise that this adaptation reflects on the structure of their networks and separates them further from algae. In addition to adapting to the new environmental conditions, land plants evolved new features such as a vascular system to transport water and nutrients. They also evolved new pathways to interact with their environments, such as, among others, defense mechanisms against pathogens and herbivores but also systems to “talk” to other individuals or species using volatile compounds [66]. Such systems impacted the organization of their metabolic networks and set them apart from algae.

**Fungi kingdom.** Within fungi, we observed that Microsporidia are separated from the other fungal clades (See Fig 6). Indeed, Microsporidia are very peculiar within the fungal kingdom. These fungi are all obligate unicellular parasites of different animal hosts. They have reduced genomes indicating limited metabolic capabilities compared to other fungal clades. They do not possess mitochondrion. Such features set them apart from the other fungi and highly influence the structure of their metabolic network. It is also noteworthy to mention that although they are basal to the fungal kingdom, there is still a debate about their taxonomic affiliation and whether they have a protistan origin or not [67].

**Protist kingdom.** Finally, the analyses carried out on Protists did not show a clear clustering patterns except for Euglenozoa, see Fig 7. Chromoalveolata, Euglinids, and Kinoplastids are part of the Excavata and have similar evolutionary paths. The three taxa underwent a secondary endosymbiosis event in which their ancestors acquired a red alga for the Chromoalveolata and green algae for Euglinids and Kinoplastids. These algae conferred evolved into organelles similar to chloroplasts. The protists that acquired these new organelles thus became autotrophic. This secondary endosymbiotic event was later followed, in Alveolates and Euglenozoa, by a loss of the phototrophic organelle and a specialization toward intracellular parasitism with the evolution of metabolic pathways to adapt to this new environment, characterised by a richness in nutrients but at the same time the presence of an immune response against parasites.

### Third experiment: Prokaryotes analysis

In the first experiment, considering the whole dataset analysis, we did not observe a clear pattern separating Bacteria and Archaea. The absence of a phylogenetic signal in Prokaryotes can be attributed to the importance of ecological and environmental factors in shaping the prokaryotic metabolism and, by extension, the metabolic network structure [68–71]. The impact of the environment in shaping the metabolic network of prokaryotes manifests in the capacity of microbes sharing the same environment to exchange genetic material through horizontal gene transfer (HGT). This genic exchange transcends the kingdom barrier. It has been shown that Bacteria and Archaea sharing the same environment could exchange genetic material and display similar metabolic capabilities [72]. HGT is common between and within prokaryotic kingdoms, thus impacting the metabolic network structure of these organisms [73].

By focusing on a more limited microbial clade (i.e. the genus *Pseudomonas*) where the microorganisms are mesophilic and live in similar environments, it was possible to observe a clustering pattern similar, although not identical, to their evolution. (See Fig 9, where we show the comparison between the Pseudomonas phylogeny and the complete-linkage hierarchical clustering obtained with the WL graph kernel). This congruence, that we observe between kernels clustering and phylogeny, only strengthens the argument that, in prokaryotes, the environment has a stronger impact on network structure than evolution. Sequence-based phylogeny remains the best way to infer taxonomy and evolution within a given group of organisms.

We suppose that, in most cases, the non-perfect match between the phylogeny and the dendrogram is due to the differences in the nature of the data used by both approaches. Phylogeny analyses the sequence of the single copy genes present in all the organisms (i.e., core genes). These sequences represent a subset of the entire genomes. This subset also presents certain characteristics (e.g., selective pressure, rate of mutation) that can be different from the entire genomes or at least from other subsets of genes. This data allows for a fine reconstruction of the taxonomic and evolutionary relationships between the different organisms at a very detailed level (i.e., at the strain level). On the other hand, the AMNs use as starting data the pathways present in the organisms and their connections. Such data, as shown in the Results section, allows to observe patterns corresponding to macroevolutionary events but is not suitable to analyse finer evolutionary and taxonomic scales. Hence, as expected, we observe more differences rather than similarities. However, we consider that it is interesting to focus on the similarities observed, since it suggests that the organismal evolution that we observe through phylogenetic studies correlates with the organization and structure of the AMNs.

## Conclusion

In this paper, we compared the metabolism of a comprehensive set of organisms to explore the potentials of the so-called *abstract metabolic networks*, a simplified representation of metabolism. Our goal was to investigate the extent to which AMNs help discern the different taxonomic groups and capture evolutionary steps. We considered the metabolism of 7141 different species stored in the KEGG database and performed a large-scale comparison of their AMNs using graph kernels. Specifically, we employed the Vertex Histogram, Shortest Path, Weisfeiler-Lehman and the Pyramid Match graph kernels. We performed various experiments, first by considering the whole set of selected species, then by considering all the Eukaryotes and, within Eukaryotes, their kingdoms separately, and finally focusing on Prokaryotes. The comparison results were investigated through exploratory data analysis as well as machine learning techniques.

Looking at the experiments, we observe that all the results turned out to be biologically and evolutionary meaningful. Moreover, although performing differently, all the considered kernels reported a similar clustering pattern at higher taxonomic level, thus suggesting that such patterns are clear and robust. This allows us to state that AMNs, although being a straightforward representation of the metabolism, are able to reflect key evolutionary processes within the metabolism. However, it is also clear that, in general, they fail to capture fine-grain differences between species due to the lack of information on the reactions within each metabolic pathway.

Our abstraction of the metabolic pathway removes detailed information. This, in turn, makes it easier to cluster together organisms that, with a more detailed network, would have been segregated. Most organisms, in fact, are able to catabolise glucose and synthesise ribose and nucleotides. Nonetheless, the metabolic reactions that form these processes may differ from organism to organism. It would have been possible to differentiate between the organisms by comparing them through a detailed metabolic network representation, reporting the information on the reactions within each metabolic pathway. With less information reported on each pathway (higher level of abstraction), it is more challenging to differentiate organisms, even at higher taxonomic levels. It is surprising that with the highest level of abstraction (i.e. presence of the pathway and its link to other pathways), it is still possible to differentiate taxa up to the phylum and sometimes the class level. This suggests that evolution did not only impact the metabolism at the fine level (i.e. reaction) but also at a higher level that affected the pathways structure and their interaction with other pathways.

The effect of evolution on the higher levels of metabolic network organisation is only observable when the genetic exchange and, by extension, the exchange of functions is minimal. In the case of prokaryotes, where HGT is frequent, it is impossible to observe a relation between evolution and higher levels of metabolic network organisation. This is mainly due to the contribution of the environment in shaping these networks.

We conclude by drawing some considerations about the graph kernels explored in this study. Although all of them were effective in this setting, we try to highlight their similarities and differences. The VH graph kernel performed better at a high level of taxonomy, that is, with very distant evolutionary groups. Moreover, both the SP and PM graph kernels, although being conceptually different, consider the graph’s connectivity in the comparison and often obtained similar results. They performed better than VH when descending to lower levels of taxonomy, where the presence or absence of pathways is not enough to discriminate the different taxonomic groups. Finally, the WL graph kernel obtained better results at a lower taxonomy level. It captures the local similarities of the graphs under consideration by comparing their subtrees. This detailed comparison may add noise when evolutionarily distant species are compared, while it may reveal helpful information when comparing neighbouring species.

This exploratory investigation on the expressiveness of AMNs gives rise to new research questions that we would like to address for future work. The first question is: How to use AMNs to highlight the relevant topological similarities and differences among the metabolism of different species? Graph kernels allow for a fast and simple comparison of AMNs, but they do not provide insights into the nodes and edges that gave rise to the similarity results. A related question is: Are the concepts of *core* and *pan* metabolism, and by extension, core and pan genomes, of a set of species interesting and relevant if applied in the context of AMNs, i.e. at the level of topological organisation of metabolic networks? They correspond to finding the common subgraph (pathways and their connections) of all the considered species (core AMNs metabolism) and finding the overall graph that considers all pathways and connections of all the considered species (pan AMNs metabolism). A further question is: Is it possible to explore the pathways dependencies of two or more symbiotic species? Being able to study the interplay of their metabolisms in terms of the topological organisation of pathways would foster the comprehension of their mutual help and relationship.

To answer these questions graph kernels are not suitable, so a different comparison technique is needed. Since AMNs are graphs, any technique in the graph theory realm can be put into action. An alternative proposal that we would like to pursue is to use a graph database (see e.g. [74]) to store and compare AMNs. In a graph database setting, all the questions raised above and many more could be easily answered through specific queries. Moreover, a graph database usually provides a suitable visual rendering of graphs and, since AMNs are relatively small, looking at them on the screen would be feasible and effective.

## Supporting information

S1 File organisms.pdf. List of the considered KEGG organisms.
(PDF)

S2 File Metabolic_Categories_And_Pathways.pdf. List of the KEGG metabolic categories and corresponding pathways.
(PDF)

S3 File GraphKernels.pdf. Mathematical formalization of the graph kernels used in the paper.
(PDF)

S4 File WholeDatasetAnalysis.pdf. Complete set of analyses considering the whole set of KEGG organisms (first experiment).
(PDF)

S5 File EukaryotesAnalysis.pdf. Eukaryotes analyses at kingdom level (second experiment).
(PDF)

S6 File AnimalsAnalysis.pdf. Animals analyses at phylum level (second experiment).
(PDF)

S7 File VertebratesAnalysis.pdf. Vertebrates analyses at class level (second experiment).
(PDF)

S8 File ProtophotophytesAnalysis.pdf. Protophotophytes analyses at phylum level (second experiment).
(PDF)

S9 File FungiAnalysis.pdf. Fungi analyses at phylum level (second experiment).
(PDF)

S10 File ProtistsAnalysis.pdf. Protists analyses at phylum level (second experiment).
(PDF)

S11 File BacteriaAnalysis.pdf. Bacteria analyses at phylum level (third experiment). Only the phyla Actinobacteria, Bacteroidetes, Firmicutes, Proteobacteria and Tenericutes present in the list S1 File have been considered.
(PDF)

S12 File PseudomonasAnalysis.pdf. Pseudomonas analyses at species level (third experiment). Comparison between the Pseudomonas phylogenetic tree and the dendrograms resulting by using the VH, SP, WL and PM graph kernels.
(PDF)

S13 File ThresholdsAnalysis.pdf. Analyses of the considered experiments after removing the pathways harbouring only one reaction (threshold 1) or at most two reactions (threshold 2). The results can be directly compared with the ones presented in the Results section.
(PDF)
