# Supplementary material for: Exploring the expressiveness of abstract metabolic networks
Source: PLoS One. 2023 Feb 9;18(2):e0281047. doi: 10.1371/journal.pone.0281047 (PMC9910719; doi:10.1371/journal.pone.0281047)
Supplement: S1 File — List of the considered KEGG organisms. (PDF) [file pone.0281047.s001.pdf]

index	Kegg ID	ORGANISM	TAXONOMY
1	hsa	Homo sapiens (human)	"Eukaryotes;Animals;Vertebrates;Mammals"
2	ptr	Pan troglodytes (chimpanzee)	"Eukaryotes;Animals;Vertebrates;Mammals"
3	pps	Pan paniscus (bonobo)	"Eukaryotes;Animals;Vertebrates;Mammals"
4	ggo	Gorilla gorilla gorilla (western lowland gorilla)	"Eukaryotes;Animals;Vertebrates;Mammals"
5	pon	Pongo abelii (Sumatran orangutan)	"Eukaryotes;Animals;Vertebrates;Mammals"
6	nle	Nomascus leucogenys (northern white-cheeked gibbon)	"Eukaryotes;Animals;Vertebrates;Mammals"
7	mcc	Macaca mulatta (rhesus monkey)	"Eukaryotes;Animals;Vertebrates;Mammals"
8	mcf	Macaca fascicularis (crab-eating macaque)	"Eukaryotes;Animals;Vertebrates;Mammals"
9	csab	Chlorocebus sabaeus (green monkey)	"Eukaryotes;Animals;Vertebrates;Mammals"
10	caty	Cercocebus atys (sooty mangabey)	"Eukaryotes;Animals;Vertebrates;Mammals"
11	panu	Papio anubis (olive baboon)	"Eukaryotes;Animals;Vertebrates;Mammals"
12	rro	Rhinopithecus roxellana (golden snub-nosed monkey)	"Eukaryotes;Animals;Vertebrates;Mammals"
13	rbb	Rhinopithecus bieti (black snub-nosed monkey)	"Eukaryotes;Animals;Vertebrates;Mammals"
14	tfn	Trachypithecus francoisi (Francois's langur)	"Eukaryotes;Animals;Vertebrates;Mammals"
15	pteh	Piliocolobus tephrosceles (Ugandan red Colobus)	"Eukaryotes;Animals;Vertebrates;Mammals"
16	cjc	Callithrix jacchus (white-tufted-ear marmoset)	"Eukaryotes;Animals;Vertebrates;Mammals"
17	sbq	Saimiri boliviensis boliviensis (Bolivian squirrel monkey)	"Eukaryotes;Animals;Vertebrates;Mammals"
18	mmur	Microcebus murinus (gray mouse lemur)	"Eukaryotes;Animals;Vertebrates;Mammals"
19	mmu	Mus musculus (mouse)	"Eukaryotes;Animals;Vertebrates;Mammals"
20	mcal	Mus caroli (Ryukyu mouse)	"Eukaryotes;Animals;Vertebrates;Mammals"
21	mpah	Mus pahari (shrew mouse)	"Eukaryotes;Animals;Vertebrates;Mammals"
22	rno	Rattus norvegicus (rat)	"Eukaryotes;Animals;Vertebrates;Mammals"
23	mcoc	Mastomys coucha (southern multimammate mouse)	"Eukaryotes;Animals;Vertebrates;Mammals"
24	mun	Meriones unguiculatus (Mongolian gerbil)	"Eukaryotes;Animals;Vertebrates;Mammals"
25	cge	Cricetulus griseus (Chinese hamster)	"Eukaryotes;Animals;Vertebrates;Mammals"
26	pleu	Peromyscus leucopus (white-footed mouse)	"Eukaryotes;Animals;Vertebrates;Mammals"
27	ngi	Nannospalax galili (Upper Galilee mountains blind mole rat)	"Eukaryotes;Animals;Vertebrates;Mammals"
28	hgl	Heterocephalus glaber (naked mole rat)	"Eukaryotes;Animals;Vertebrates;Mammals"
29	ccan	Castor canadensis (American beaver)	"Eukaryotes;Animals;Vertebrates;Mammals"
30	ocu	Oryctolagus cuniculus (rabbit)	"Eukaryotes;Animals;Vertebrates;Mammals"
31	opi	Ochotona princeps (American pika)	"Eukaryotes;Animals;Vertebrates;Mammals"
32	tup	Tupaia chinensis (Chinese tree shrew)	"Eukaryotes;Animals;Vertebrates;Mammals"
33	cfa	Canis lupus familiaris (dog)	"Eukaryotes;Animals;Vertebrates;Mammals"
34	vvp	Vulpes vulpes (red fox)	"Eukaryotes;Animals;Vertebrates;Mammals"
35	vlg	Vulpes lagopus (Arctic fox)	"Eukaryotes;Animals;Vertebrates;Mammals"
36	aml	Ailuropoda melanoleuca (giant panda)	"Eukaryotes;Animals;Vertebrates;Mammals"
37	umr	Ursus maritimus (polar bear)	"Eukaryotes;Animals;Vertebrates;Mammals"
38	uah	Ursus arctos horribilis	"Eukaryotes;Animals;Vertebrates;Mammals"
39	oro	Odobenus rosmarus divergens (Pacific walrus)	"Eukaryotes;Animals;Vertebrates;Mammals"

40	elk	Enhydra lutris kenyoni (northern sea otter)	"Eukaryotes;Animals;Vertebrates;Mammals"
41	mpuf	Mustela putorius furo (domestic ferret)	"Eukaryotes;Animals;Vertebrates;Mammals"
42	eju	Eumetopias jubatus (Steller sea lion)	"Eukaryotes;Animals;Vertebrates;Mammals"
43	mlx	Mirounga leonina (Southern elephant seal)	"Eukaryotes;Animals;Vertebrates;Mammals"
44	fca	Felis catus (domestic cat)	"Eukaryotes;Animals;Vertebrates;Mammals"
45	pyu	Puma yagouaroundi (jaguarundi)	"Eukaryotes;Animals;Vertebrates;Mammals"
46	pbg	Prionailurus bengalensis (leopard cat)	"Eukaryotes;Animals;Vertebrates;Mammals"
47	ptg	Panthera tigris altaica (Amur tiger)	"Eukaryotes;Animals;Vertebrates;Mammals"
48	ppad	Panthera pardus (leopard)	"Eukaryotes;Animals;Vertebrates;Mammals"
49	aju	Acinonyx jubatus (cheetah)	"Eukaryotes;Animals;Vertebrates;Mammals"
50	hhv	Hyaena hyaena (striped hyena)	"Eukaryotes;Animals;Vertebrates;Mammals"
51	bta	Bos taurus (cow)	"Eukaryotes;Animals;Vertebrates;Mammals"
52	bom	Bos mutus (wild yak)	"Eukaryotes;Animals;Vertebrates;Mammals"
53	biu	Bos indicus (zebu cattle)	"Eukaryotes;Animals;Vertebrates;Mammals"
54	bbub	Bubalus bubalis (water buffalo)	"Eukaryotes;Animals;Vertebrates;Mammals"
55	chx	Capra hircus (goat)	"Eukaryotes;Animals;Vertebrates;Mammals"
56	oas	Ovis aries (sheep)	"Eukaryotes;Animals;Vertebrates;Mammals"
57	oda	Oryx dammah (scimitar-horned oryx)	"Eukaryotes;Animals;Vertebrates;Mammals"
58	ccad	Cervus canadensis (wapiti)	"Eukaryotes;Animals;Vertebrates;Mammals"
59	ssc	Sus scrofa (pig)	"Eukaryotes;Animals;Vertebrates;Mammals"
60	cfr	Camelus ferus (Wild Bactrian camel)	"Eukaryotes;Animals;Vertebrates;Mammals"
61	cbai	Camelus bactrianus (Bactrian camel)	"Eukaryotes;Animals;Vertebrates;Mammals"
62	cdk	Camelus dromedarius (Arabian camel)	"Eukaryotes;Animals;Vertebrates;Mammals"
63	bacu	Balaenoptera acutorostrata scammoni (minke whale)	"Eukaryotes;Animals;Vertebrates;Mammals"
64	lve	Lipotes vexillifer (Yangtze River dolphin)	"Eukaryotes;Animals;Vertebrates;Mammals"
65	oor	Orcinus orca (killer whale)	"Eukaryotes;Animals;Vertebrates;Mammals"
66	dle	Delphinapterus leucas (beluga whale)	"Eukaryotes;Animals;Vertebrates;Mammals"
67	pcad	Physeter catodon (sperm whale)	"Eukaryotes;Animals;Vertebrates;Mammals"
68	ecb	Equus caballus (horse)	"Eukaryotes;Animals;Vertebrates;Mammals"
69	epz	Equus przewalskii (Przewalski's horse)	"Eukaryotes;Animals;Vertebrates;Mammals"
70	eai	Equus asinus (ass)	"Eukaryotes;Animals;Vertebrates;Mammals"
71	myb	Myotis brandtii (Brandt's bat)	"Eukaryotes;Animals;Vertebrates;Mammals"
72	myd	Myotis davidii	"Eukaryotes;Animals;Vertebrates;Mammals"
73	mmyo	Myotis myotis	"Eukaryotes;Animals;Vertebrates;Mammals"
74	mna	Miniopterus natalensis	"Eukaryotes;Animals;Vertebrates;Mammals"
75	pkl	Pipistrellus kuhlii (Kuhl's pipistrelle)	"Eukaryotes;Animals;Vertebrates;Mammals"
76	hai	Hipposideros armiger (great roundleaf bat)	"Eukaryotes;Animals;Vertebrates;Mammals"
77	dro	Desmodus rotundus (common vampire bat)	"Eukaryotes;Animals;Vertebrates;Mammals"
78	shon	Sturnira hondurensis	"Eukaryotes;Animals;Vertebrates;Mammals"
79	ajm	Artibeus jamaicensis (Jamaican fruit-eating bat)	"Eukaryotes;Animals;Vertebrates;Mammals"
80	pdic	Phyllostomus discolor (pale spear-nosed bat)	"Eukaryotes;Animals;Vertebrates;Mammals"

81	mmf	Molossus molossus (Pallas's mastiff bat)	"Eukaryotes;Animals;Vertebrates;Mammals"
82	rfq	Rhinolophus ferrumequinum (greater horseshoe bat)	"Eukaryotes;Animals;Vertebrates;Mammals"
83	pale	Pteropus alecto (black flying fox)	"Eukaryotes;Animals;Vertebrates;Mammals"
84	pgig	Pteropus giganteus (Indian flying fox)	"Eukaryotes;Animals;Vertebrates;Mammals"
85	ray	Rousettus aegyptiacus (Egyptian rousette)	"Eukaryotes;Animals;Vertebrates;Mammals"
86	mjv	Manis javanica (Malayan pangolin)	"Eukaryotes;Animals;Vertebrates;Mammals"
87	tod	Talpa occidentalis (Iberian mole)	"Eukaryotes;Animals;Vertebrates;Mammals"
88	lav	Loxodonta africana (African savanna elephant)	"Eukaryotes;Animals;Vertebrates;Mammals"
89	tmu	Trichechus manatus latirostris (Florida manatee)	"Eukaryotes;Animals;Vertebrates;Mammals"
90	mdo	Monodelphis domestica (opossum)	"Eukaryotes;Animals;Vertebrates;Mammals"
91	gas	Gracilinanus agilis (Agile Gracile Mouse Opossum)	"Eukaryotes;Animals;Vertebrates;Mammals"
92	shr	Sarcophilus harrisii (Tasmanian devil)	"Eukaryotes;Animals;Vertebrates;Mammals"
93	pcw	Phascolarctos cinereus (koala)	"Eukaryotes;Animals;Vertebrates;Mammals"
94	oaa	Ornithorhynchus anatinus (platypus)	"Eukaryotes;Animals;Vertebrates;Mammals"
95	gga	Gallus gallus (chicken)	"Eukaryotes;Animals;Vertebrates;Birds"
96	pcoc	Phasianus colchicus (Ring-necked pheasant)	"Eukaryotes;Animals;Vertebrates;Birds"
97	mgp	Meleagris gallopavo (turkey)	"Eukaryotes;Animals;Vertebrates;Birds"
98	cjo	Coturnix japonica (Japanese quail)	"Eukaryotes;Animals;Vertebrates;Birds"
99	nmel	Numida meleagris (helmeted guineafowl)	"Eukaryotes;Animals;Vertebrates;Birds"
100	apla	Anas platyrhynchos (mallard)	"Eukaryotes;Animals;Vertebrates;Birds"
101	acyg	Anser cygnoides domesticus (swan goose)	"Eukaryotes;Animals;Vertebrates;Birds"
102	tgu	Taeniopygia guttata (zebra finch)	"Eukaryotes;Animals;Vertebrates;Birds"
103	lsr	Lonchura striata domestica (Bengalese finch)	"Eukaryotes;Animals;Vertebrates;Birds"
104	scan	Serinus canaria (common canary)	"Eukaryotes;Animals;Vertebrates;Birds"
105	pmoa	Passer montanus (Eurasian tree sparrow)	"Eukaryotes;Animals;Vertebrates;Birds"
106	otc	Onychostruthus taczanowskii (white-rumped snowfinch)	"Eukaryotes;Animals;Vertebrates;Birds"
107	pruf	Pyrgilauda ruficollis (rufous-necked snowfinch)	"Eukaryotes;Animals;Vertebrates;Birds"
108	gfr	Geospiza fortis (medium ground-finch)	"Eukaryotes;Animals;Vertebrates;Birds"
109	fab	Ficedula albicollis (collared flycatcher)	"Eukaryotes;Animals;Vertebrates;Birds"
110	phi	Pseudopodoces humilis (Tibetan ground-tit)	"Eukaryotes;Animals;Vertebrates;Birds"
111	pmaj	Parus major (Great Tit)	"Eukaryotes;Animals;Vertebrates;Birds"
112	ccae	Cyanistes caeruleus (blue tit)	"Eukaryotes;Animals;Vertebrates;Birds"
113	ccw	Corvus cornix (hooded crow)	"Eukaryotes;Animals;Vertebrates;Birds"
114	etl	Empidonax traillii (willow flycatcher)	"Eukaryotes;Animals;Vertebrates;Birds"
115	fpg	Falco peregrinus (peregrine falcon)	"Eukaryotes;Animals;Vertebrates;Birds"
116	fch	Falco cherrug (Saker falcon)	"Eukaryotes;Animals;Vertebrates;Birds"
117	clv	Columba livia (rock pigeon)	"Eukaryotes;Animals;Vertebrates;Birds"
118	egz	Egretta garzetta (little egret)	"Eukaryotes;Animals;Vertebrates;Birds"
119	nni	Nipponia nippon (crested ibis)	"Eukaryotes;Animals;Vertebrates;Birds"
120	acun	Athene cunicularia (burrowing owl)	"Eukaryotes;Animals;Vertebrates;Birds"
121	padl	Pygoscelis adeliae (Adelie penguin)	"Eukaryotes;Animals;Vertebrates;Birds"

122	aam	Apteryx mantelli mantelli (brown kiwi)	"Eukaryotes;Animals;Vertebrates;Birds"
123	arow	Apteryx rowi (Okarito brown kiwi)	"Eukaryotes;Animals;Vertebrates;Birds"
124	npd	Nothoprocta perdicaria	"Eukaryotes;Animals;Vertebrates;Birds"
125	dne	Dromaius novaehollandiae (emu)	"Eukaryotes;Animals;Vertebrates;Birds"
126	asn	Alligator sinensis (Chinese alligator)	"Eukaryotes;Animals;Vertebrates;Reptiles"
127	amj	Alligator mississippiensis (American alligator)	"Eukaryotes;Animals;Vertebrates;Reptiles"
128	cpoo	Crocodylus porosus (Australian saltwater crocodile)	"Eukaryotes;Animals;Vertebrates;Reptiles"
129	ggn	Gavialis gangeticus (Gharial)	"Eukaryotes;Animals;Vertebrates;Reptiles"
130	pss	Pelodiscus sinensis (Chinese soft-shelled turtle)	"Eukaryotes;Animals;Vertebrates;Reptiles"
131	cmy	Chelonia mydas (green sea turtle)	"Eukaryotes;Animals;Vertebrates;Reptiles"
132	cpic	Chrysemys picta (western painted turtle)	"Eukaryotes;Animals;Vertebrates;Reptiles"
133	tst	Trachemys scripta elegans	"Eukaryotes;Animals;Vertebrates;Reptiles"
134	cabi	Chelonoidis abingdonii (Abingdon island giant tortoise)	"Eukaryotes;Animals;Vertebrates;Reptiles"
135	acs	Anolis carolinensis (green anole)	"Eukaryotes;Animals;Vertebrates;Reptiles"
136	pvt	Pogona vitticeps (central bearded dragon)	"Eukaryotes;Animals;Vertebrates;Reptiles"
137	sund	Sceloporus undulatus (fence lizard)	"Eukaryotes;Animals;Vertebrates;Reptiles"
138	pbi	Python bivittatus (Burmese python)	"Eukaryotes;Animals;Vertebrates;Reptiles"
139	pmur	Protobothrops mucrosquamatus (Taiwan habu)	"Eukaryotes;Animals;Vertebrates;Reptiles"
140	tsr	Thamnophis sirtalis	"Eukaryotes;Animals;Vertebrates;Reptiles"
141	pgut	Pantherophis guttatus	"Eukaryotes;Animals;Vertebrates;Reptiles"
142	vko	Varanus komodoensis (Komodo dragon)	"Eukaryotes;Animals;Vertebrates;Reptiles"
143	pmua	Podarcis muralis (common wall lizard)	"Eukaryotes;Animals;Vertebrates;Reptiles"
144	zvi	Zootoca vivipara (common lizard)	"Eukaryotes;Animals;Vertebrates;Reptiles"
145	gja	Gekko japonicus	"Eukaryotes;Animals;Vertebrates;Reptiles"
146	xla	Xenopus laevis (African clawed frog)	"Eukaryotes;Animals;Vertebrates;Amphibians"
147	xtr	Xenopus tropicalis (western clawed frog)	"Eukaryotes;Animals;Vertebrates;Amphibians"
148	npr	Nanorana parkeri	"Eukaryotes;Animals;Vertebrates;Amphibians"
149	dre	Danio rerio (zebrafish)	"Eukaryotes;Animals;Vertebrates;Fishes"
150	srx	Sinocyclocheilus rhinocerous	"Eukaryotes;Animals;Vertebrates;Fishes"
151	sanh	Sinocyclocheilus anshuiensis	"Eukaryotes;Animals;Vertebrates;Fishes"
152	sgh	Sinocyclocheilus grahami	"Eukaryotes;Animals;Vertebrates;Fishes"
153	ccar	Cyprinus carpio (common carp)	"Eukaryotes;Animals;Vertebrates;Fishes"
154	caua	Carassius auratus (goldfish)	"Eukaryotes;Animals;Vertebrates;Fishes"
155	ipu	Ictalurus punctatus (channel catfish)	"Eukaryotes;Animals;Vertebrates;Fishes"
156	phyp	Pangasianodon hypophthalmus (striped catfish)	"Eukaryotes;Animals;Vertebrates;Fishes"
157	amex	Astyanax mexicanus (Mexican tetra)	"Eukaryotes;Animals;Vertebrates;Fishes"
158	eee	Electrophorus electricus (electric eel)	"Eukaryotes;Animals;Vertebrates;Fishes"
159	tru	Takifugu rubripes (torafugu)	"Eukaryotes;Animals;Vertebrates;Fishes"
160	tng	Tetraodon nigroviridis (spotted green pufferfish)	"Eukaryotes;Animals;Vertebrates;Fishes"
161	lco	Larimichthys crocea (large yellow croaker)	"Eukaryotes;Animals;Vertebrates;Fishes"
162	ncc	Notothenia coriiceps (black rockcod)	"Eukaryotes;Animals;Vertebrates;Fishes"

163	cgob	Cottoperca gobio	"Eukaryotes;Animals;Vertebrates;Fishes"
164	ely	Epinephelus lanceolatus (giant grouper)	"Eukaryotes;Animals;Vertebrates;Fishes"
165	plep	Plectropomus leopardus (leopard coralgrouper)	"Eukaryotes;Animals;Vertebrates;Fishes"
166	sluc	Sander lucioperca (pikeperch)	"Eukaryotes;Animals;Vertebrates;Fishes"
167	ecra	Etheostoma cragini (Arkansas darter)	"Eukaryotes;Animals;Vertebrates;Fishes"
168	pflv	Perca flavescens (yellow perch)	"Eukaryotes;Animals;Vertebrates;Fishes"
169	gat	Gasterosteus aculeatus (three-spined stickleback)	"Eukaryotes;Animals;Vertebrates;Fishes"
170	ppug	Pungitius pungitius (ninespine stickleback)	"Eukaryotes;Animals;Vertebrates;Fishes"
171	msam	Micropterus salmoides (largemouth bass)	"Eukaryotes;Animals;Vertebrates;Fishes"
172	cud	Cheilinus undulatus (humphead wrasse)	"Eukaryotes;Animals;Vertebrates;Fishes"
173	mze	Maylandia zebra (zebra mbuna)	"Eukaryotes;Animals;Vertebrates;Fishes"
174	onl	Oreochromis niloticus (Nile tilapia)	"Eukaryotes;Animals;Vertebrates;Fishes"
175	oau	Oreochromis aureus (blue tilapia)	"Eukaryotes;Animals;Vertebrates;Fishes"
176	ola	Oryzias latipes (Japanese medaka)	"Eukaryotes;Animals;Vertebrates;Fishes"
177	oml	Oryzias melastigma (Indian medaka)	"Eukaryotes;Animals;Vertebrates;Fishes"
178	xma	Xiphophorus maculatus (southern platyfish)	"Eukaryotes;Animals;Vertebrates;Fishes"
179	xco	Xiphophorus couchianus (Monterrey platyfish)	"Eukaryotes;Animals;Vertebrates;Fishes"
180	xhe	Xiphophorus hellerii (green swordtail)	"Eukaryotes;Animals;Vertebrates;Fishes"
181	pret	Poecilia reticulata (guppy)	"Eukaryotes;Animals;Vertebrates;Fishes"
182	cvg	Cyprinodon variegatus (sheepshead minnow)	"Eukaryotes;Animals;Vertebrates;Fishes"
183	ctul	Cyprinodon tularosa	"Eukaryotes;Animals;Vertebrates;Fishes"
184	nfu	Nothobranchius furzeri (turquoise killifish)	"Eukaryotes;Animals;Vertebrates;Fishes"
185	kmr	Kryptolebias marmoratus (mangrove rivulus)	"Eukaryotes;Animals;Vertebrates;Fishes"
186	alim	Austrofundulus limnaeus (annual killifish)	"Eukaryotes;Animals;Vertebrates;Fishes"
187	aoce	Amphiprion ocellaris (clown anemonefish)	"Eukaryotes;Animals;Vertebrates;Fishes"
188	csem	Cynoglossus semilaevis (tongue sole)	"Eukaryotes;Animals;Vertebrates;Fishes"
189	pov	Paralichthys olivaceus (Japanese flounder)	"Eukaryotes;Animals;Vertebrates;Fishes"
190	ssen	Solea senegalensis (Senegalese sole)	"Eukaryotes;Animals;Vertebrates;Fishes"
191	lcf	Lates calcarifer (barramundi perch)	"Eukaryotes;Animals;Vertebrates;Fishes"
192	sdu	Seriola dumerili (greater amberjack)	"Eukaryotes;Animals;Vertebrates;Fishes"
193	slal	Seriola lalandi dorsalis (Yellowtail amberjack)	"Eukaryotes;Animals;Vertebrates;Fishes"
194	xgl	Xiphias gladius (swordfish)	"Eukaryotes;Animals;Vertebrates;Fishes"
195	hcq	Hippocampus comes (tiger tail seahorse)	"Eukaryotes;Animals;Vertebrates;Fishes"
196	bpec	Boleophthalmus pectinirostris (great blue-spotted mudskipper)	"Eukaryotes;Animals;Vertebrates;Fishes"
197	malb	Monopterus albus (swamp eel)	"Eukaryotes;Animals;Vertebrates;Fishes"
198	sasa	Salmo salar (Atlantic salmon)	"Eukaryotes;Animals;Vertebrates;Fishes"
199	otw	Oncorhynchus tshawytscha (Chinook salmon)	"Eukaryotes;Animals;Vertebrates;Fishes"
200	omy	Oncorhynchus mykiss (rainbow trout)	"Eukaryotes;Animals;Vertebrates;Fishes"
201	salp	Salvelinus sp. IW2-2015 (Arctic char)	"Eukaryotes;Animals;Vertebrates;Fishes"
202	snh	Salvelinus namaycush (lake trout)	"Eukaryotes;Animals;Vertebrates;Fishes"
203	els	Esox lucius (northern pike)	"Eukaryotes;Animals;Vertebrates;Fishes"

204	sfm	Scleropages formosus (Asian bonytongue)	"Eukaryotes;Animals;Vertebrates;Fishes"
205	pki	Paramormyrops kingsleyae	"Eukaryotes;Animals;Vertebrates;Fishes"
206	aang	Anguilla anguilla (European eel)	"Eukaryotes;Animals;Vertebrates;Fishes"
207	loc	Lepisosteus oculatus (spotted gar)	"Eukaryotes;Animals;Vertebrates;Fishes"
208	pspa	Polyodon spathula (Mississippi paddlefish)	"Eukaryotes;Animals;Vertebrates;Fishes"
209	arut	Acipenser ruthenus (sterlet)	"Eukaryotes;Animals;Vertebrates;Fishes"
210	lcm	Latimeria chalumnae (coelacanth)	"Eukaryotes;Animals;Vertebrates;Fishes"
211	cmk	Callorhinchus milii (elephant shark)	"Eukaryotes;Animals;Vertebrates;Cartilaginous fishes"
212	rtp	Rhincodon typus (whale shark)	"Eukaryotes;Animals;Vertebrates;Cartilaginous fishes"
213	bfo	Branchiostoma floridae (Florida lancelet)	"Eukaryotes;Animals;Lancelets"
214	bbel	Branchiostoma belcheri (Belcher's lancelet)	"Eukaryotes;Animals;Lancelets"
215	cin	Ciona intestinalis (sea squirt)	"Eukaryotes;Animals;Ascidians"
216	sclv	Styela clava	"Eukaryotes;Animals;Ascidians"
217	spu	Strongylocentrotus purpuratus (purple sea urchin)	"Eukaryotes;Animals;Echinoderms"
218	aplc	Acanthaster planci (crown-of-thorns starfish)	"Eukaryotes;Animals;Echinoderms"
219	sko	Saccoglossus kowalevskii (acorn worm)	"Eukaryotes;Animals;Hemichordates"
220	dme	Drosophila melanogaster (fruit fly)	"Eukaryotes;Animals;Arthropods;Insects"
221	der	Drosophila erecta	"Eukaryotes;Animals;Arthropods;Insects"
222	dse	Drosophila sechellia	"Eukaryotes;Animals;Arthropods;Insects"
223	dsi	Drosophila simulans	"Eukaryotes;Animals;Arthropods;Insects"
224	dya	Drosophila yakuba	"Eukaryotes;Animals;Arthropods;Insects"
225	dan	Drosophila ananassae	"Eukaryotes;Animals;Arthropods;Insects"
226	dsr	Drosophila serrata	"Eukaryotes;Animals;Arthropods;Insects"
227	dpo	Drosophila pseudoobscura pseudoobscura	"Eukaryotes;Animals;Arthropods;Insects"
228	dpe	Drosophila persimilis	"Eukaryotes;Animals;Arthropods;Insects"
229	dmn	Drosophila miranda	"Eukaryotes;Animals;Arthropods;Insects"
230	dwi	Drosophila willistoni	"Eukaryotes;Animals;Arthropods;Insects"
231	dgr	Drosophila grimshawi	"Eukaryotes;Animals;Arthropods;Insects"
232	dmo	Drosophila mojavensis	"Eukaryotes;Animals;Arthropods;Insects"
233	daz	Drosophila arizonae	"Eukaryotes;Animals;Arthropods;Insects"
234	dnv	Drosophila navojoa	"Eukaryotes;Animals;Arthropods;Insects"
235	dhe	Drosophila hydei	"Eukaryotes;Animals;Arthropods;Insects"
236	dvi	Drosophila virilis	"Eukaryotes;Animals;Arthropods;Insects"
237	ccat	Ceratitis capitata (Mediterranean fruit fly)	"Eukaryotes;Animals;Arthropods;Insects"
238	bod	Bactrocera oleae (olive fruit fly)	"Eukaryotes;Animals;Arthropods;Insects"
239	mde	Musca domestica (house fly)	"Eukaryotes;Animals;Arthropods;Insects"
240	scac	Stomoxys calcitrans (stable fly)	"Eukaryotes;Animals;Arthropods;Insects"
241	lcq	Lucilia cuprina (Australian sheep blowfly)	"Eukaryotes;Animals;Arthropods;Insects"
242	aga	Anopheles gambiae (mosquito)	"Eukaryotes;Animals;Arthropods;Insects"
243	acoz	Anopheles coluzzii	"Eukaryotes;Animals;Arthropods;Insects"
244	aara	Anopheles arabiensis	"Eukaryotes;Animals;Arthropods;Insects"

245	aag	Aedes aegypti (yellow fever mosquito)	"Eukaryotes;Animals;Arthropods;Insects"
246	aalb	Aedes albopictus (Asian tiger mosquito)	"Eukaryotes;Animals;Arthropods;Insects"
247	cqu	Culex quinquefasciatus (southern house mosquito)	"Eukaryotes;Animals;Arthropods;Insects"
248	cpii	Culex pipiens pallens	"Eukaryotes;Animals;Arthropods;Insects"
249	ame	Apis mellifera (honey bee)	"Eukaryotes;Animals;Arthropods;Insects"
250	acer	Apis cerana (Asiatic honeybee)	"Eukaryotes;Animals;Arthropods;Insects"
251	bim	Bombus impatiens (common eastern bumble bee)	"Eukaryotes;Animals;Arthropods;Insects"
252	bbif	Bombus bifarius	"Eukaryotes;Animals;Arthropods;Insects"
253	bvk	Bombus vosnesenskii	"Eukaryotes;Animals;Arthropods;Insects"
254	bvan	Bombus vancouverensis nearcticus	"Eukaryotes;Animals;Arthropods;Insects"
255	bter	Bombus terrestris (buff-tailed bumblebee)	"Eukaryotes;Animals;Arthropods;Insects"
256	ccal	Ceratina calcarata (carpenter bee)	"Eukaryotes;Animals;Arthropods;Insects"
257	obb	Osmia bicornis bicornis	"Eukaryotes;Animals;Arthropods;Insects"
258	mgen	Megalopta genalis	"Eukaryotes;Animals;Arthropods;Insects"
259	nmea	Nomia melanderi (Alkali bee)	"Eukaryotes;Animals;Arthropods;Insects"
260	cgig	Colletes gigas	"Eukaryotes;Animals;Arthropods;Insects"
261	soc	Solenopsis invicta (red fire ant)	"Eukaryotes;Animals;Arthropods;Insects"
262	mpha	Monomorium pharaonis (pharaoh ant)	"Eukaryotes;Animals;Arthropods;Insects"
263	aec	Acromyrmex echinatior (Panamanian leafcutter ant)	"Eukaryotes;Animals;Arthropods;Insects"
264	acep	Atta cephalotes (leaf cutting ant)	"Eukaryotes;Animals;Arthropods;Insects"
265	pbar	Pogonomyrmex barbatus (red harvester ant)	"Eukaryotes;Animals;Arthropods;Insects"
266	vem	Vollenhovia emeryi	"Eukaryotes;Animals;Arthropods;Insects"
267	hst	Harpegnathos saltator (Jerdon's jumping ant)	"Eukaryotes;Animals;Arthropods;Insects"
268	dqu	Dinoponera quadriceps	"Eukaryotes;Animals;Arthropods;Insects"
269	cfo	Camponotus floridanus (Florida carpenter ant)	"Eukaryotes;Animals;Arthropods;Insects"
270	fex	Formica exsecta	"Eukaryotes;Animals;Arthropods;Insects"
271	lhu	Linepithema humile (Argentine ant)	"Eukaryotes;Animals;Arthropods;Insects"
272	pgc	Pseudomyrmex gracilis	"Eukaryotes;Animals;Arthropods;Insects"
273	obo	Ooceraea biroi (clonal raider ant)	"Eukaryotes;Animals;Arthropods;Insects"
274	pcf	Polistes canadensis	"Eukaryotes;Animals;Arthropods;Insects"
275	pfuc	Polistes fuscatus (common paper wasp)	"Eukaryotes;Animals;Arthropods;Insects"
276	vps	Vespula pensylvanica (western yellowjacket)	"Eukaryotes;Animals;Arthropods;Insects"
277	nvi	Nasonia vitripennis (jewel wasp)	"Eukaryotes;Animals;Arthropods;Insects"
278	csol	Ceratosolen solmsi marchali	"Eukaryotes;Animals;Arthropods;Insects"
279	tpre	Trichogramma pretiosum	"Eukaryotes;Animals;Arthropods;Insects"
280	mdl	Microplitis demolitor	"Eukaryotes;Animals;Arthropods;Insects"
281	cglo	Cotesia glomerata	"Eukaryotes;Animals;Arthropods;Insects"
282	fas	Fopius arisanus	"Eukaryotes;Animals;Arthropods;Insects"
283	dam	Diachasma alloeum	"Eukaryotes;Animals;Arthropods;Insects"
284	ccin	Cephus cinctus (wheat stem sawfly)	"Eukaryotes;Animals;Arthropods;Insects"
285	tca	Tribolium castaneum (red flour beetle)	"Eukaryotes;Animals;Arthropods;Insects"

286	dpa	Dendroctonus ponderosae (mountain pine beetle)	"Eukaryotes;Animals;Arthropods;Insects"
287	atd	Aethina tumida (small hive beetle)	"Eukaryotes;Animals;Arthropods;Insects"
288	agb	Anoplophora glabripennis (Asian longhorned beetle)	"Eukaryotes;Animals;Arthropods;Insects"
289	ldc	Leptinotarsa decemlineata (Colorado potato beetle)	"Eukaryotes;Animals;Arthropods;Insects"
290	nvl	Nicrophorus vespilloides	"Eukaryotes;Animals;Arthropods;Insects"
291	apln	Agrilus planipennis (emerald ash borer)	"Eukaryotes;Animals;Arthropods;Insects"
292	ppyr	Photinus pyralis (common eastern firefly)	"Eukaryotes;Animals;Arthropods;Insects"
293	otu	Onthophagus taurus	"Eukaryotes;Animals;Arthropods;Insects"
294	bmor	Bombyx mori (domestic silkworm)	"Eukaryotes;Animals;Arthropods;Insects"
295	bman	Bombyx mandarina (wild silkworm)	"Eukaryotes;Animals;Arthropods;Insects"
296	msex	Manduca sexta (tobacco hornworm)	"Eukaryotes;Animals;Arthropods;Insects"
297	dpl	Danaus plexippus (monarch butterfly)	"Eukaryotes;Animals;Arthropods;Insects"
298	bany	Bicyclus anynana (squinting bush brown)	"Eukaryotes;Animals;Arthropods;Insects"
299	pmac	Papilio machaon (common yellow swallowtail)	"Eukaryotes;Animals;Arthropods;Insects"
300	ppot	Papilio polytes (common Mormon)	"Eukaryotes;Animals;Arthropods;Insects"
301	pxu	Papilio xuthus (Asian swallowtail)	"Eukaryotes;Animals;Arthropods;Insects"
302	prap	Pieris rapae (cabbage white)	"Eukaryotes;Animals;Arthropods;Insects"
303	zce	Zerene cesonia (dogface butterfly)	"Eukaryotes;Animals;Arthropods;Insects"
304	haw	Helicoverpa armigera (cotton bollworm)	"Eukaryotes;Animals;Arthropods;Insects"
305	tnl	Trichoplusia ni (cabbage looper)	"Eukaryotes;Animals;Arthropods;Insects"
306	pxy	Plutella xylostella (diamondback moth)	"Eukaryotes;Animals;Arthropods;Insects"
307	api	Acyrthosiphon pisum (pea aphid)	"Eukaryotes;Animals;Arthropods;Insects"
308	dnx	Diuraphis noxia (Russian wheat aphid)	"Eukaryotes;Animals;Arthropods;Insects"
309	ags	Aphis gossypii (cotton aphid)	"Eukaryotes;Animals;Arthropods;Insects"
310	rmd	Rhopalosiphum maidis (corn leaf aphid)	"Eukaryotes;Animals;Arthropods;Insects"
311	btab	Bemisia tabaci (sweet potato whitefly)	"Eukaryotes;Animals;Arthropods;Insects"
312	dci	Diaphorina citri (Asian citrus psyllid)	"Eukaryotes;Animals;Arthropods;Insects"
313	clec	Cimex lectularius (bed bug)	"Eukaryotes;Animals;Arthropods;Insects"
314	hhal	Halyomorpha halys (brown marmorated stink bug)	"Eukaryotes;Animals;Arthropods;Insects"
315	nlu	Nilaparvata lugens (brown planthopper)	"Eukaryotes;Animals;Arthropods;Insects"
316	phu	Pediculus humanus corporis (human body louse)	"Eukaryotes;Animals;Arthropods;Insects"
317	foc	Frankliniella occidentalis (western flower thrips)	"Eukaryotes;Animals;Arthropods;Insects"
318	zne	Zootermopsis nevadensis	"Eukaryotes;Animals;Arthropods;Insects"
319	csec	Cryptotermes secundus	"Eukaryotes;Animals;Arthropods;Insects"
320	fcd	Folsomia candida	"Eukaryotes;Animals;Arthropods;Insects"
321	dpx	Daphnia pulex (common water flea)	"Eukaryotes;Animals;Arthropods;Crustaceans"
322	dmk	Daphnia magna	"Eukaryotes;Animals;Arthropods;Crustaceans"
323	pvm	Penaeus vannamei (Pacific white shrimp)	"Eukaryotes;Animals;Arthropods;Crustaceans"
324	pja	Penaeus japonicus	"Eukaryotes;Animals;Arthropods;Crustaceans"
325	hame	Homarus americanus (American lobster)	"Eukaryotes;Animals;Arthropods;Crustaceans"
326	hazt	Hyalella azteca	"Eukaryotes;Animals;Arthropods;Crustaceans"

327	eaf	Eurytemora affinis	"Eukaryotes;Animals;Arthropods;Crustaceans"
328	isc	Ixodes scapularis (black-legged tick)	"Eukaryotes;Animals;Arthropods;Chelicerates"
329	dsv	Dermacentor silvarum	"Eukaryotes;Animals;Arthropods;Chelicerates"
330	rsan	Rhipicephalus sanguineus (brown dog tick)	"Eukaryotes;Animals;Arthropods;Chelicerates"
331	rmp	Rhipicephalus microplus (southern cattle tick)	"Eukaryotes;Animals;Arthropods;Chelicerates"
332	vde	Varroa destructor (honeybee mite)	"Eukaryotes;Animals;Arthropods;Chelicerates"
333	vja	Varroa jacobsoni	"Eukaryotes;Animals;Arthropods;Chelicerates"
334	tut	Tetranychus urticae (two-spotted spider mite)	"Eukaryotes;Animals;Arthropods;Chelicerates"
335	dpte	Dermatophagoides pteronyssinus (European house dust mite)	"Eukaryotes;Animals;Arthropods;Chelicerates"
336	cscu	Centruroides sculpturatus (bark scorpion)	"Eukaryotes;Animals;Arthropods;Chelicerates"
337	ptep	Parasteatoda tepidariorum (common house spider)	"Eukaryotes;Animals;Arthropods;Chelicerates"
338	sdm	Stegodyphus dumicola	"Eukaryotes;Animals;Arthropods;Chelicerates"
339	cel	Caenorhabditis elegans (nematode)	"Eukaryotes;Animals;Nematodes"
340	cbr	Caenorhabditis briggsae	"Eukaryotes;Animals;Nematodes"
341	bmy	Brugia malayi (filaria)	"Eukaryotes;Animals;Nematodes"
342	loa	Loa loa (eye worm)	"Eukaryotes;Animals;Nematodes"
343	nai	Necator americanus	"Eukaryotes;Animals;Nematodes"
344	tsp	Trichinella spiralis	"Eukaryotes;Animals;Nematodes"
345	hro	Helobdella robusta	"Eukaryotes;Animals;Annelids"
346	lgi	Lottia gigantea (owl limpet)	"Eukaryotes;Animals;Mollusks"
347	pcan	Pomacea canaliculata (golden apple snail)	"Eukaryotes;Animals;Mollusks"
348	bgt	Biomphalaria glabrata (bloodfluke planorb)	"Eukaryotes;Animals;Mollusks"
349	gae	Gigantopelta aegis	"Eukaryotes;Animals;Mollusks"
350	crg	Crassostrea gigas (Pacific oyster)	"Eukaryotes;Animals;Mollusks"
351	myi	Mizuhopecten yessoensis (Yesso scallop)	"Eukaryotes;Animals;Mollusks"
352	pmax	Pecten maximus	"Eukaryotes;Animals;Mollusks"
353	obi	Octopus bimaculoides	"Eukaryotes;Animals;Mollusks"
354	osn	Octopus sinensis (East Asian common octopus)	"Eukaryotes;Animals;Mollusks"
355	lak	Lingula anatina	"Eukaryotes;Animals;Brachiopodas"
356	smm	Schistosoma mansoni	"Eukaryotes;Animals;Flatworms"
357	shx	Schistosoma haematobium	"Eukaryotes;Animals;Flatworms"
358	ovi	Opisthorchis viverrini	"Eukaryotes;Animals;Flatworms"
359	egl	Echinococcus granulosus	"Eukaryotes;Animals;Flatworms"
360	nve	Nematostella vectensis (starlet sea anemone)	"Eukaryotes;Animals;Cnidarians"
361	epa	Exaiptasia diaphana	"Eukaryotes;Animals;Cnidarians"
362	aten	Actinia tenebrosa (Australian red waratah sea anemone)	"Eukaryotes;Animals;Cnidarians"
363	adf	Acropora digitifera (stony coral)	"Eukaryotes;Animals;Cnidarians"
364	amil	Acropora millepora	"Eukaryotes;Animals;Cnidarians"
365	pdam	Pocillopora damicornis (lace coral)	"Eukaryotes;Animals;Cnidarians"
366	spis	Stylophora pistillata	"Eukaryotes;Animals;Cnidarians"
367	dgt	Dendronephthya gigantea (soft corals)	"Eukaryotes;Animals;Cnidarians"

368	hmg	Hydra vulgaris	"Eukaryotes;Animals;Cnidarians"
369	tad	Trichoplax adhaerens	"Eukaryotes;Animals;Placozoans"
370	aqu	Amphimedon queenslandica (sponge)	"Eukaryotes;Animals;Poriferans"
371	ath	Arabidopsis thaliana (thale cress)	"Eukaryotes;Plants;Eudicots;Mustard family"
372	aly	Arabidopsis lyrata (lyrate rockcress)	"Eukaryotes;Plants;Eudicots;Mustard family"
373	crb	Capsella rubella	"Eukaryotes;Plants;Eudicots;Mustard family"
374	csat	Camelina sativa (false flax)	"Eukaryotes;Plants;Eudicots;Mustard family"
375	eus	Eutrema salsugineum	"Eukaryotes;Plants;Eudicots;Mustard family"
376	brp	Brassica rapa (field mustard)	"Eukaryotes;Plants;Eudicots;Mustard family"
377	bna	Brassica napus (rape)	"Eukaryotes;Plants;Eudicots;Mustard family"
378	boe	Brassica oleracea (wild cabbage)	"Eukaryotes;Plants;Eudicots;Mustard family"
379	rsz	Raphanus sativus (radish)	"Eukaryotes;Plants;Eudicots;Mustard family"
380	thj	Tarenaya hassleriana (spider flower)	"Eukaryotes;Plants;Eudicots;Caper family"
381	cpap	Carica papaya (papaya)	"Eukaryotes;Plants;Eudicots;Papaya family"
382	cit	Citrus sinensis (Valencia orange)	"Eukaryotes;Plants;Eudicots;Rue family"
383	cic	Citrus clementina (mandarin orange)	"Eukaryotes;Plants;Eudicots;Rue family"
384	pvy	Pistacia vera (pistachio)	"Eukaryotes;Plants;Eudicots;Sumac family"
385	minc	Mangifera indica (mango)	"Eukaryotes;Plants;Eudicots;Sumac family"
386	tcc	Theobroma cacao (cacao)	"Eukaryotes;Plants;Eudicots;Mallow family"
387	gra	Gossypium raimondii	"Eukaryotes;Plants;Eudicots;Mallow family"
388	ghi	Gossypium hirsutum (upland cotton)	"Eukaryotes;Plants;Eudicots;Mallow family"
389	gab	Gossypium arboreum	"Eukaryotes;Plants;Eudicots;Mallow family"
390	dzi	Durio zibethinus (durian)	"Eukaryotes;Plants;Eudicots;Mallow family"
391	egr	Eucalyptus grandis (rose gum)	"Eukaryotes;Plants;Eudicots;Myrtle family"
392	gmx	Glycine max (soybean)	"Eukaryotes;Plants;Eudicots;Pea family"
393	gsj	Glycine soja (wild soybean)	"Eukaryotes;Plants;Eudicots;Pea family"
394	pvu	Phaseolus vulgaris (common bean)	"Eukaryotes;Plants;Eudicots;Pea family"
395	vra	Vigna radiata (mung bean)	"Eukaryotes;Plants;Eudicots;Pea family"
396	var	Vigna angularis (adzuki bean)	"Eukaryotes;Plants;Eudicots;Pea family"
397	vun	Vigna unguiculata (cowpea)	"Eukaryotes;Plants;Eudicots;Pea family"
398	ccaj	Cajanus cajan (pigeon pea)	"Eukaryotes;Plants;Eudicots;Pea family"
399	aprc	Abrus precatorius (Indian licorice)	"Eukaryotes;Plants;Eudicots;Pea family"
400	mtr	Medicago truncatula (barrel medic)	"Eukaryotes;Plants;Eudicots;Pea family"
401	cam	Cicer arietinum (chickpea)	"Eukaryotes;Plants;Eudicots;Pea family"
402	lja	Lotus japonicus	"Eukaryotes;Plants;Eudicots;Pea family"
403	adu	Arachis duranensis	"Eukaryotes;Plants;Eudicots;Pea family"
404	aip	Arachis ipaensis	"Eukaryotes;Plants;Eudicots;Pea family"
405	ahf	Arachis hypogaea (peanut)	"Eukaryotes;Plants;Eudicots;Pea family"
406	lang	Lupinus angustifolius (narrow-leaved blue lupine)	"Eukaryotes;Plants;Eudicots;Pea family"
407	fve	Fragaria vesca (woodland strawberry)	"Eukaryotes;Plants;Eudicots;Rose family"
408	rcn	Rosa chinensis (China rose)	"Eukaryotes;Plants;Eudicots;Rose family"

409	pper	Prunus persica (peach)	"Eukaryotes;Plants;Eudicots;Rose family"
410	pmum	Prunus mume (Japanese apricot)	"Eukaryotes;Plants;Eudicots;Rose family"
411	pavi	Prunus avium (sweet cherry)	"Eukaryotes;Plants;Eudicots;Rose family"
412	pdul	Prunus dulcis (almond)	"Eukaryotes;Plants;Eudicots;Rose family"
413	mdm	Malus domestica (apple)	"Eukaryotes;Plants;Eudicots;Rose family"
414	pxb	Pyrus x bretschneideri (Chinese white pear)	"Eukaryotes;Plants;Eudicots;Rose family"
415	zju	Ziziphus jujuba (Chinese jujube)	"Eukaryotes;Plants;Eudicots;Buckthorn family"
416	mnt	Morus notabilis	"Eukaryotes;Plants;Eudicots;Mulberry family"
417	csv	Cucumis sativus (cucumber)	"Eukaryotes;Plants;Eudicots;Cucumber family"
418	cmo	Cucumis melo (muskmelon)	"Eukaryotes;Plants;Eudicots;Cucumber family"
419	bhj	Benincasa hispida (wax gourd)	"Eukaryotes;Plants;Eudicots;Cucumber family"
420	mcha	Momordica charantia (bitter melon)	"Eukaryotes;Plants;Eudicots;Cucumber family"
421	cmax	Cucurbita maxima (winter squash)	"Eukaryotes;Plants;Eudicots;Cucumber family"
422	cmos	Cucurbita moschata (crookneck pumpkin)	"Eukaryotes;Plants;Eudicots;Cucumber family"
423	cpep	Cucurbita pepo subsp. pepo (vegetable marrow)	"Eukaryotes;Plants;Eudicots;Cucumber family"
424	rcu	Ricinus communis (castor bean)	"Eukaryotes;Plants;Eudicots;Spurge family"
425	jcu	Jatropha curcas	"Eukaryotes;Plants;Eudicots;Spurge family"
426	hbr	Hevea brasiliensis (rubber tree)	"Eukaryotes;Plants;Eudicots;Spurge family"
427	mesc	Manihot esculenta (cassava)	"Eukaryotes;Plants;Eudicots;Spurge family"
428	pop	Populus trichocarpa (black cottonwood)	"Eukaryotes;Plants;Eudicots;Willow family"
429	peu	Populus euphratica (Euphrates poplar)	"Eukaryotes;Plants;Eudicots;Willow family"
430	palz	Populus alba (white poplar)	"Eukaryotes;Plants;Eudicots;Willow family"
431	jre	Juglans regia (English walnut)	"Eukaryotes;Plants;Eudicots;Walnut family"
432	qsu	Quercus suber (cork oak)	"Eukaryotes;Plants;Eudicots;Beech family"
433	qlo	Quercus lobata (valley oak)	"Eukaryotes;Plants;Eudicots;Beech family"
434	twl	Tripterygium wilfordii	"Eukaryotes;Plants;Eudicots;Bittersweet family"
435	vvi	Vitis vinifera (wine grape)	"Eukaryotes;Plants;Eudicots;Grape family"
436	vri	Vitis riparia (riverbank grape)	"Eukaryotes;Plants;Eudicots;Grape family"
437	sly	Solanum lycopersicum (tomato)	"Eukaryotes;Plants;Eudicots;Nightshade family"
438	spen	Solanum pennellii	"Eukaryotes;Plants;Eudicots;Nightshade family"
439	sot	Solanum tuberosum (potato)	"Eukaryotes;Plants;Eudicots;Nightshade family"
440	cann	Capsicum annuum	"Eukaryotes;Plants;Eudicots;Nightshade family"
441	nta	Nicotiana tabacum (common tobacco)	"Eukaryotes;Plants;Eudicots;Nightshade family"
442	nsy	Nicotiana sylvestris	"Eukaryotes;Plants;Eudicots;Nightshade family"
443	nto	Nicotiana tomentosiformis	"Eukaryotes;Plants;Eudicots;Nightshade family"
444	nau	Nicotiana attenuata	"Eukaryotes;Plants;Eudicots;Nightshade family"
445	ini	Ipomoea nil (Japanese morning glory)	"Eukaryotes;Plants;Eudicots;Morning-glory family"
446	itr	Ipomoea triloba (trilobed morning glory)	"Eukaryotes;Plants;Eudicots;Morning-glory family"
447	sind	Sesamum indicum (sesame)	"Eukaryotes;Plants;Eudicots;Sesame family"
448	oeu	Olea europaea var. sylvestris (wild olive)	"Eukaryotes;Plants;Eudicots;Olive family"
449	egt	Erythranthe guttata (spotted monkey flower)	"Eukaryotes;Plants;Eudicots;Lopseed family"

450	sspl	Salvia splendens (scarlet sage)	"Eukaryotes;Plants;Eudicots;Mint family"
451	han	Helianthus annuus (common sunflower)	"Eukaryotes;Plants;Eudicots;Daisy family"
452	ecad	Erigeron canadensis (horseweed)	"Eukaryotes;Plants;Eudicots;Daisy family"
453	lsv	Lactuca sativa (garden lettuce)	"Eukaryotes;Plants;Eudicots;Daisy family"
454	ccav	Cynara cardunculus var. scolymus (artichoke)	"Eukaryotes;Plants;Eudicots;Daisy family"
455	dcr	Daucus carota (carrot)	"Eukaryotes;Plants;Eudicots;Parsley family"
456	csin	Camellia sinensis	"Eukaryotes;Plants;Eudicots;Tea family"
457	bvg	Beta vulgaris (sugar beet)	"Eukaryotes;Plants;Eudicots;Amaranth family"
458	soe	Spinacia oleracea (spinach)	"Eukaryotes;Plants;Eudicots;Amaranth family"
459	cqi	Chenopodium quinoa (quinoa)	"Eukaryotes;Plants;Eudicots;Amaranth family"
460	nnu	Nelumbo nucifera (sacred lotus)	"Eukaryotes;Plants;Eudicots;Lotus family"
461	ming	Macadamia integrifolia (macadamia nut)	"Eukaryotes;Plants;Eudicots;Protea family"
462	psom	Papaver somniferum (opium poppy)	"Eukaryotes;Plants;Eudicots;Poppy family"
463	ncol	Nymphaea colorata	"Eukaryotes;Plants;Eudicots;Water-lily family"
464	osa	Oryza sativa japonica (Japanese rice) (RefSeq)	"Eukaryotes;Plants;Monocots;Grass family"
465	dosa	Oryza sativa japonica (Japanese rice) (RAPDB)	"Eukaryotes;Plants;Monocots;Grass family"
466	obr	Oryza brachyantha (malo sina)	"Eukaryotes;Plants;Monocots;Grass family"
467	bdi	Brachypodium distachyon	"Eukaryotes;Plants;Monocots;Grass family"
468	ats	Aegilops tauschii (wheat D)	"Eukaryotes;Plants;Monocots;Grass family"
469	tdc	Triticum dicoccoides (wild emmer wheat)	"Eukaryotes;Plants;Monocots;Grass family"
470	sbi	Sorghum bicolor (sorghum)	"Eukaryotes;Plants;Monocots;Grass family"
471	zma	Zea mays (maize)	"Eukaryotes;Plants;Monocots;Grass family"
472	sita	Setaria italica (foxtail millet)	"Eukaryotes;Plants;Monocots;Grass family"
473	pvir	Panicum virgatum (switchgrass)	"Eukaryotes;Plants;Monocots;Grass family"
474	phai	Panicum hallii	"Eukaryotes;Plants;Monocots;Grass family"
475	pda	Phoenix dactylifera (date palm)	"Eukaryotes;Plants;Monocots;Palm family"
476	egu	Elaeis guineensis (African oil palm)	"Eukaryotes;Plants;Monocots;Palm family"
477	mus	Musa acuminata (wild Malaysian banana)	"Eukaryotes;Plants;Monocots;Banana family"
478	dct	Dendrobium catenatum	"Eukaryotes;Plants;Monocots;Orchid family"
479	peq	Phalaenopsis equestris	"Eukaryotes;Plants;Monocots;Orchid family"
480	aof	Asparagus officinalis (garden asparagus)	"Eukaryotes;Plants;Monocots;Asparagus family"
481	atr	Amborella trichopoda	"Eukaryotes;Plants;Basal Magnoliophyta;Amborella family"
482	smo	Selaginella moellendorffii	"Eukaryotes;Plants;Ferns"
483	ppp	Physcomitrium patens	"Eukaryotes;Plants;Mosses"
484	cre	Chlamydomonas reinhardtii	"Eukaryotes;Plants;Green algae"
485	vcn	Volvox carteri f. nagariensis	"Eukaryotes;Plants;Green algae"
486	mng	Monoraphidium neglectum	"Eukaryotes;Plants;Green algae"
487	csl	Coccomyxa subellipsoidea	"Eukaryotes;Plants;Green algae"
488	cvr	Chlorella variabilis	"Eukaryotes;Plants;Green algae"
489	apro	Auxenochlorella protothecoides	"Eukaryotes;Plants;Green algae"
490	olu	Ostreococcus lucimarinus	"Eukaryotes;Plants;Green algae"

491	ota	Ostreococcus tauri	"Eukaryotes;Plants;Green algae"
492	bpg	Bathycoccus prasinos	"Eukaryotes;Plants;Green algae"
493	mis	Micromonas commoda	"Eukaryotes;Plants;Green algae"
494	mpp	Micromonas pusilla	"Eukaryotes;Plants;Green algae"
495	cme	Cyanidioschyzon merolae	"Eukaryotes;Plants;Red algae"
496	gsl	Galdieria sulphuraria	"Eukaryotes;Plants;Red algae"
497	ccp	Chondrus crispus (carragheen)	"Eukaryotes;Plants;Red algae"
498	sce	Saccharomyces cerevisiae (budding yeast)	"Eukaryotes;Fungi;Ascomycetes;Saccharomycetes"
499	ago	Ashbya gossypii (Eremothecium gossypii)	"Eukaryotes;Fungi;Ascomycetes;Saccharomycetes"
500	erc	Eremothecium cymbalariae	"Eukaryotes;Fungi;Ascomycetes;Saccharomycetes"
501	kla	Kluyveromyces lactis	"Eukaryotes;Fungi;Ascomycetes;Saccharomycetes"
502	kmx	Kluyveromyces marxianus	"Eukaryotes;Fungi;Ascomycetes;Saccharomycetes"
503	lth	Lachancea thermotolerans	"Eukaryotes;Fungi;Ascomycetes;Saccharomycetes"
504	vpo	Vanderwaltozyma polyspora	"Eukaryotes;Fungi;Ascomycetes;Saccharomycetes"
505	zro	Zygosaccharomyces rouxii	"Eukaryotes;Fungi;Ascomycetes;Saccharomycetes"
506	cgr	Candida glabrata	"Eukaryotes;Fungi;Ascomycetes;Saccharomycetes"
507	ncs	Naumovozyma castellii	"Eukaryotes;Fungi;Ascomycetes;Saccharomycetes"
508	ndi	Naumovozyma dairenensis	"Eukaryotes;Fungi;Ascomycetes;Saccharomycetes"
509	tpf	Tetrapisispora phaffii	"Eukaryotes;Fungi;Ascomycetes;Saccharomycetes"
510	tbl	Tetrapisispora blattae	"Eukaryotes;Fungi;Ascomycetes;Saccharomycetes"
511	tdl	Torulaspora delbrueckii	"Eukaryotes;Fungi;Ascomycetes;Saccharomycetes"
512	tgb	Torulaspora globosa	"Eukaryotes;Fungi;Ascomycetes;Saccharomycetes"
513	kaf	Kazachstania africana	"Eukaryotes;Fungi;Ascomycetes;Saccharomycetes"
514	zmk	Zygotorulaspora mrakii	"Eukaryotes;Fungi;Ascomycetes;Saccharomycetes"
515	ppa	Komagataella phaffii	"Eukaryotes;Fungi;Ascomycetes;Saccharomycetes"
516	dha	Debaryomyces hansenii	"Eukaryotes;Fungi;Ascomycetes;Saccharomycetes"
517	pic	Scheffersomyces stipitis	"Eukaryotes;Fungi;Ascomycetes;Saccharomycetes"
518	pgu	Meyerozyma guilliermondii	"Eukaryotes;Fungi;Ascomycetes;Saccharomycetes"
519	spaa	Spathaspora passalidarum	"Eukaryotes;Fungi;Ascomycetes;Saccharomycetes"
520	lel	Lodderomyces elongisporus	"Eukaryotes;Fungi;Ascomycetes;Saccharomycetes"
521	cal	Candida albicans	"Eukaryotes;Fungi;Ascomycetes;Saccharomycetes"
522	ctp	Candida tropicalis	"Eukaryotes;Fungi;Ascomycetes;Saccharomycetes"
523	cot	Candida orthopsilosis	"Eukaryotes;Fungi;Ascomycetes;Saccharomycetes"
524	cdu	Candida dubliniensis	"Eukaryotes;Fungi;Ascomycetes;Saccharomycetes"
525	cten	Yamadazyma tenuis	"Eukaryotes;Fungi;Ascomycetes;Saccharomycetes"
526	yli	Yarrowia lipolytica	"Eukaryotes;Fungi;Ascomycetes;Saccharomycetes"
527	clu	Clavispora lusitaniae ATCC 42720	"Eukaryotes;Fungi;Ascomycetes;Saccharomycetes"
528	clus	Clavispora lusitaniae CBS 6936	"Eukaryotes;Fungi;Ascomycetes;Saccharomycetes"
529	caur	Candida auris	"Eukaryotes;Fungi;Ascomycetes;Saccharomycetes"
530	slb	Sugiyamaella lignohabitans	"Eukaryotes;Fungi;Ascomycetes;Saccharomycetes"
531	pkz	Pichia kudriavzevii	"Eukaryotes;Fungi;Ascomycetes;Saccharomycetes"

532	bnn	Brettanomyces nanus	"Eukaryotes;Fungi;Ascomycetes;Saccharomycetes"
533	bbrx	Brettanomyces bruxellensis	"Eukaryotes;Fungi;Ascomycetes;Saccharomycetes"
534	ncr	Neurospora crassa	"Eukaryotes;Fungi;Ascomycetes;Sordariomycetes"
535	nte	Neurospora tetrasperma	"Eukaryotes;Fungi;Ascomycetes;Sordariomycetes"
536	smp	Sordaria macrospora	"Eukaryotes;Fungi;Ascomycetes;Sordariomycetes"
537	pan	Podospora anserina	"Eukaryotes;Fungi;Ascomycetes;Sordariomycetes"
538	ttt	Thermothielavioides terrestris	"Eukaryotes;Fungi;Ascomycetes;Sordariomycetes"
539	mtm	Thermothelomyces thermophilus	"Eukaryotes;Fungi;Ascomycetes;Sordariomycetes"
540	cthr	Chaetomium thermophilum	"Eukaryotes;Fungi;Ascomycetes;Sordariomycetes"
541	mgr	Pyricularia oryzae	"Eukaryotes;Fungi;Ascomycetes;Sordariomycetes"
542	tmn	Phaeoacremonium minimum	"Eukaryotes;Fungi;Ascomycetes;Sordariomycetes"
543	ssck	Sporothrix schenckii	"Eukaryotes;Fungi;Ascomycetes;Sordariomycetes"
544	fgr	Fusarium graminearum	"Eukaryotes;Fungi;Ascomycetes;Sordariomycetes"
545	fpu	Fusarium pseudograminearum	"Eukaryotes;Fungi;Ascomycetes;Sordariomycetes"
546	fvr	Fusarium verticillioides	"Eukaryotes;Fungi;Ascomycetes;Sordariomycetes"
547	fox	Fusarium oxysporum	"Eukaryotes;Fungi;Ascomycetes;Sordariomycetes"
548	nhe	Fusarium vanettenii	"Eukaryotes;Fungi;Ascomycetes;Sordariomycetes"
549	tre	Trichoderma reesei QM6a	"Eukaryotes;Fungi;Ascomycetes;Sordariomycetes"
550	trr	Trichoderma reesei RUT C-30	"Eukaryotes;Fungi;Ascomycetes;Sordariomycetes"
551	maw	Metarhizium acridum	"Eukaryotes;Fungi;Ascomycetes;Sordariomycetes"
552	maj	Metarhizium robertsii	"Eukaryotes;Fungi;Ascomycetes;Sordariomycetes"
553	cmt	Cordyceps militaris	"Eukaryotes;Fungi;Ascomycetes;Sordariomycetes"
554	plj	Purpureocillium lilacinum	"Eukaryotes;Fungi;Ascomycetes;Sordariomycetes"
555	val	Verticillium alfalfae	"Eukaryotes;Fungi;Ascomycetes;Sordariomycetes"
556	vda	Verticillium dahliae	"Eukaryotes;Fungi;Ascomycetes;Sordariomycetes"
557	cfj	Colletotrichum fioriniae	"Eukaryotes;Fungi;Ascomycetes;Sordariomycetes"
558	sapo	Scedosporium apiospermum	"Eukaryotes;Fungi;Ascomycetes;Sordariomycetes"
559	ela	Eutypa lata	"Eukaryotes;Fungi;Ascomycetes;Sordariomycetes"
560	pfy	Pestalotiopsis fici	"Eukaryotes;Fungi;Ascomycetes;Sordariomycetes"
561	ssl	Sclerotinia sclerotiorum	"Eukaryotes;Fungi;Ascomycetes;Leotiomycetes"
562	bfu	Botrytis cinerea	"Eukaryotes;Fungi;Ascomycetes;Leotiomycetes"
563	mbe	Drepanopeziza brunnea	"Eukaryotes;Fungi;Ascomycetes;Leotiomycetes"
564	psco	Mollisia scopiformis	"Eukaryotes;Fungi;Ascomycetes;Leotiomycetes"
565	glz	Glarea lozoyensis	"Eukaryotes;Fungi;Ascomycetes;Leotiomycetes"
566	ani	Aspergillus nidulans	"Eukaryotes;Fungi;Ascomycetes;Eurotiomycetes"
567	afm	Aspergillus fumigatus	"Eukaryotes;Fungi;Ascomycetes;Eurotiomycetes"
568	act	Aspergillus clavatus	"Eukaryotes;Fungi;Ascomycetes;Eurotiomycetes"
569	nfi	Aspergillus fischeri	"Eukaryotes;Fungi;Ascomycetes;Eurotiomycetes"
570	aor	Aspergillus oryzae	"Eukaryotes;Fungi;Ascomycetes;Eurotiomycetes"
571	ang	Aspergillus niger	"Eukaryotes;Fungi;Ascomycetes;Eurotiomycetes"
572	afv	Aspergillus flavus	"Eukaryotes;Fungi;Ascomycetes;Eurotiomycetes"

573	pcs	Penicillium rubens	"Eukaryotes;Fungi;Ascomycetes;Eurotiomycetes"
574	pdp	Penicillium digitatum	"Eukaryotes;Fungi;Ascomycetes;Eurotiomycetes"
575	tmf	Talaromyces marneffei	"Eukaryotes;Fungi;Ascomycetes;Eurotiomycetes"
576	trg	Talaromyces rugulosus	"Eukaryotes;Fungi;Ascomycetes;Eurotiomycetes"
577	cim	Coccidioides immitis	"Eukaryotes;Fungi;Ascomycetes;Eurotiomycetes"
578	cpw	Coccidioides posadasii	"Eukaryotes;Fungi;Ascomycetes;Eurotiomycetes"
579	ure	Uncinocarpus reesii	"Eukaryotes;Fungi;Ascomycetes;Eurotiomycetes"
580	pbl	Paracoccidioides lutzii Pb01	"Eukaryotes;Fungi;Ascomycetes;Eurotiomycetes"
581	pbn	Paracoccidioides brasiliensis	"Eukaryotes;Fungi;Ascomycetes;Eurotiomycetes"
582	abe	Trichophyton benhamiae	"Eukaryotes;Fungi;Ascomycetes;Eurotiomycetes"
583	tve	Trichophyton verrucosum	"Eukaryotes;Fungi;Ascomycetes;Eurotiomycetes"
584	aje	Histoplasma mississippiense	"Eukaryotes;Fungi;Ascomycetes;Eurotiomycetes"
585	bgh	Blastomyces gilchristii	"Eukaryotes;Fungi;Ascomycetes;Eurotiomycetes"
586	pno	Parastagonospora nodorum	"Eukaryotes;Fungi;Ascomycetes;Dothideomycetes"
587	pte	Pyrenophora teres	"Eukaryotes;Fungi;Ascomycetes;Dothideomycetes"
588	bze	Bipolaris zeicola	"Eukaryotes;Fungi;Ascomycetes;Dothideomycetes"
589	bsc	Bipolaris sorokiniana	"Eukaryotes;Fungi;Ascomycetes;Dothideomycetes"
590	bor	Bipolaris oryzae	"Eukaryotes;Fungi;Ascomycetes;Dothideomycetes"
591	aalt	Alternaria alternata	"Eukaryotes;Fungi;Ascomycetes;Dothideomycetes"
592	ztr	Zymoseptoria tritici	"Eukaryotes;Fungi;Ascomycetes;Dothideomycetes"
593	pfj	Pseudocercospora fijiensis	"Eukaryotes;Fungi;Ascomycetes;Dothideomycetes"
594	bcom	Baudoinia panamericana	"Eukaryotes;Fungi;Ascomycetes;Dothideomycetes"
595	npa	Neofusicoccum parvum	"Eukaryotes;Fungi;Ascomycetes;Dothideomycetes"
596	tml	Tuber melanosporum	"Eukaryotes;Fungi;Ascomycetes;Pezizomycetes"
597	spo	Schizosaccharomyces pombe (fission yeast)	"Eukaryotes;Fungi;Ascomycetes;Schizosaccharomycetes"
598	cne	Cryptococcus neoformans var. neoformans JEC21	"Eukaryotes;Fungi;Basidiomycetes"
599	cnb	Cryptococcus neoformans var. neoformans B-3501A	"Eukaryotes;Fungi;Basidiomycetes"
600	cgi	Cryptococcus gattii	"Eukaryotes;Fungi;Basidiomycetes"
601	tms	Tremella mesenterica	"Eukaryotes;Fungi;Basidiomycetes"
602	tasa	Trichosporon asahii var. asahii	"Eukaryotes;Fungi;Basidiomycetes"
603	ppl	Postia placenta	"Eukaryotes;Fungi;Basidiomycetes"
604	tvs	Trametes versicolor	"Eukaryotes;Fungi;Basidiomycetes"
605	dsq	Dichomitus squalens	"Eukaryotes;Fungi;Basidiomycetes"
606	pco	Phanerochaete carnosa	"Eukaryotes;Fungi;Basidiomycetes"
607	shs	Stereum hirsutum	"Eukaryotes;Fungi;Basidiomycetes"
608	hir	Heterobasidion irregulare	"Eukaryotes;Fungi;Basidiomycetes"
609	psq	Punctularia strigosozonata	"Eukaryotes;Fungi;Basidiomycetes"
610	adl	Auricularia subglabra	"Eukaryotes;Fungi;Basidiomycetes"
611	fme	Fomitiporia mediterranea	"Eukaryotes;Fungi;Basidiomycetes"
612	gtr	Gloeophyllum trabeum	"Eukaryotes;Fungi;Basidiomycetes"
613	lbc	Laccaria bicolor	"Eukaryotes;Fungi;Basidiomycetes"

614	mpr	Moniliophthora perniciosa	"Eukaryotes;Fungi;Basidiomycetes"
615	mrr	Moniliophthora roreri	"Eukaryotes;Fungi;Basidiomycetes"
616	cci	Coprinopsis cinerea	"Eukaryotes;Fungi;Basidiomycetes"
617	scm	Schizophyllum commune	"Eukaryotes;Fungi;Basidiomycetes"
618	abp	Agaricus bisporus var. burnettii JB137-S8	"Eukaryotes;Fungi;Basidiomycetes"
619	abv	Agaricus bisporus var. bisporus H97	"Eukaryotes;Fungi;Basidiomycetes"
620	cput	Coniophora puteana	"Eukaryotes;Fungi;Basidiomycetes"
621	sla	Serpula lacrymans	"Eukaryotes;Fungi;Basidiomycetes"
622	wse	Wallemia mellicola	"Eukaryotes;Fungi;Basidiomycetes"
623	wic	Wallemia ichthyophaga	"Eukaryotes;Fungi;Basidiomycetes"
624	uma	Ustilago maydis	"Eukaryotes;Fungi;Basidiomycetes"
625	pfp	Pseudozyma flocculosa	"Eukaryotes;Fungi;Basidiomycetes"
626	mgl	Malassezia globosa	"Eukaryotes;Fungi;Basidiomycetes"
627	mrt	Malassezia restricta	"Eukaryotes;Fungi;Basidiomycetes"
628	msym	Malassezia sympodialis	"Eukaryotes;Fungi;Basidiomycetes"
629	pgr	Puccinia graminis	"Eukaryotes;Fungi;Basidiomycetes"
630	mlr	Melampsora larici-populina	"Eukaryotes;Fungi;Basidiomycetes"
631	ecu	Encephalitozoon cuniculi	"Eukaryotes;Fungi;Microsporidians"
632	ein	Encephalitozoon intestinalis	"Eukaryotes;Fungi;Microsporidians"
633	ehe	Encephalitozoon hellem	"Eukaryotes;Fungi;Microsporidians"
634	ero	Encephalitozoon romaleae	"Eukaryotes;Fungi;Microsporidians"
635	nce	Nosema ceranae	"Eukaryotes;Fungi;Microsporidians"
636	mbr	Monosiga brevicollis	"Eukaryotes;Protists;Choanoflagellates"
637	sre	Salpingoeca rosetta	"Eukaryotes;Protists;Choanoflagellates"
638	ddi	Dictyostelium discoideum (cellular slime mold)	"Eukaryotes;Protists;Amoebozoa;Dictyostelia"
639	dpp	Dictyostelium purpureum (cellular slime mold)	"Eukaryotes;Protists;Amoebozoa;Dictyostelia"
640	dfa	Cavenderia fasciculata (cellular slime mold)	"Eukaryotes;Protists;Amoebozoa;Dictyostelia"
641	ehi	Entamoeba histolytica	"Eukaryotes;Protists;Amoebozoa;Entamoeba"
642	edi	Entamoeba dispar	"Eukaryotes;Protists;Amoebozoa;Entamoeba"
643	eiv	Entamoeba invadens	"Eukaryotes;Protists;Amoebozoa;Entamoeba"
644	acan	Acanthamoeba castellanii	"Eukaryotes;Protists;Amoebozoa;Acanthamoeba"
645	pfa	Plasmodium falciparum 3D7	"Eukaryotes;Protists;Alveolates;Apicomplexans"
646	pfd	Plasmodium falciparum Dd2	"Eukaryotes;Protists;Alveolates;Apicomplexans"
647	pfh	Plasmodium falciparum HB3	"Eukaryotes;Protists;Alveolates;Apicomplexans"
648	pyo	Plasmodium yoelii	"Eukaryotes;Protists;Alveolates;Apicomplexans"
649	pcb	Plasmodium chabaudi	"Eukaryotes;Protists;Alveolates;Apicomplexans"
650	pbe	Plasmodium berghei	"Eukaryotes;Protists;Alveolates;Apicomplexans"
651	pkn	Plasmodium knowlesi	"Eukaryotes;Protists;Alveolates;Apicomplexans"
652	pvx	Plasmodium vivax	"Eukaryotes;Protists;Alveolates;Apicomplexans"
653	pcy	Plasmodium cynomolgi	"Eukaryotes;Protists;Alveolates;Apicomplexans"
654	tan	Theileria annulata	"Eukaryotes;Protists;Alveolates;Apicomplexans"

655	tpv	Theileria parva	"Eukaryotes;Protists;Alveolates;Apicomplexans"
656	tot	Theileria orientalis	"Eukaryotes;Protists;Alveolates;Apicomplexans"
657	beq	Theileria equi	"Eukaryotes;Protists;Alveolates;Apicomplexans"
658	bbo	Babesia bovis	"Eukaryotes;Protists;Alveolates;Apicomplexans"
659	bmic	Babesia microti	"Eukaryotes;Protists;Alveolates;Apicomplexans"
660	cpv	Cryptosporidium parvum	"Eukaryotes;Protists;Alveolates;Apicomplexans"
661	cho	Cryptosporidium hominis	"Eukaryotes;Protists;Alveolates;Apicomplexans"
662	tgo	Toxoplasma gondii	"Eukaryotes;Protists;Alveolates;Apicomplexans"
663	tet	Tetrahymena thermophila	"Eukaryotes;Protists;Alveolates;Ciliates"
664	ptm	Paramecium tetraurelia	"Eukaryotes;Protists;Alveolates;Ciliates"
665	smin	Breviolum minutum	"Eukaryotes;Protists;Alveolates;Dinoflagellates"
666	pti	Phaeodactylum tricornutum	"Eukaryotes;Protists;Stramenopiles;Diatoms"
667	fcy	Fragilariopsis cylindrus	"Eukaryotes;Protists;Stramenopiles;Diatoms"
668	tps	Thalassiosira pseudonana	"Eukaryotes;Protists;Stramenopiles;Diatoms"
669	ngd	Nannochloropsis gaditana	"Eukaryotes;Protists;Stramenopiles;Eustigmatophytes"
670	aaf	Aureococcus anophagefferens	"Eukaryotes;Protists;Stramenopiles;Pelagophytes"
671	pif	Phytophthora infestans	"Eukaryotes;Protists;Stramenopiles;Oomycetes"
672	psoj	Phytophthora sojae	"Eukaryotes;Protists;Stramenopiles;Oomycetes"
673	spar	Saprolegnia parasitica	"Eukaryotes;Protists;Stramenopiles;Oomycetes"
674	ehx	Emiliania huxleyi	"Eukaryotes;Protists;Haptophyta"
675	gtt	Guillardia theta	"Eukaryotes;Protists;Cryptomonads"
676	tbr	Trypanosoma brucei brucei	"Eukaryotes;Protists;Euglenozoa;Kinetoplasts"
677	tbg	Trypanosoma brucei gambiense	"Eukaryotes;Protists;Euglenozoa;Kinetoplasts"
678	tcr	Trypanosoma cruzi	"Eukaryotes;Protists;Euglenozoa;Kinetoplasts"
679	lma	Leishmania major	"Eukaryotes;Protists;Euglenozoa;Kinetoplasts"
680	lif	Leishmania infantum	"Eukaryotes;Protists;Euglenozoa;Kinetoplasts"
681	ldo	Leishmania donovani	"Eukaryotes;Protists;Euglenozoa;Kinetoplasts"
682	lmi	Leishmania mexicana	"Eukaryotes;Protists;Euglenozoa;Kinetoplasts"
683	lbz	Leishmania braziliensis	"Eukaryotes;Protists;Euglenozoa;Kinetoplasts"
684	lpan	Leishmania panamensis	"Eukaryotes;Protists;Euglenozoa;Kinetoplasts"
685	ngr	Naegleria gruberi	"Eukaryotes;Protists;Heterolobosea;Tetramitia"
686	tva	Trichomonas vaginalis	"Eukaryotes;Protists;Metamonada;Trichomonads"
687	gla	Giardia lamblia	"Eukaryotes;Protists;Metamonada;Diplomonads"
688	eco	Escherichia coli K-12 MG1655	"Prokaryotes;Bacteria;Gammaproteobacteria - Enterobacteria;Escherichia"
689	ecj	Escherichia coli K-12 W3110	"Prokaryotes;Bacteria;Gammaproteobacteria - Enterobacteria;Escherichia"
690	ecd	Escherichia coli K-12 DH10B	"Prokaryotes;Bacteria;Gammaproteobacteria - Enterobacteria;Escherichia"
691	ebw	Escherichia coli K-12 BW2952	"Prokaryotes;Bacteria;Gammaproteobacteria - Enterobacteria;Escherichia"
692	ecok	Escherichia coli K-12 MDS42	"Prokaryotes;Bacteria;Gammaproteobacteria - Enterobacteria;Escherichia"
693	ece	Escherichia coli O157:H7 EDL933 (EHEC)	"Prokaryotes;Bacteria;Gammaproteobacteria - Enterobacteria;Escherichia"
694	ecs	Escherichia coli O157:H7 Sakai (EHEC)	"Prokaryotes;Bacteria;Gammaproteobacteria - Enterobacteria;Escherichia"
695	ecf	Escherichia coli O157:H7 EC4115 (EHEC)	"Prokaryotes;Bacteria;Gammaproteobacteria - Enterobacteria;Escherichia"

696	etw	Escherichia coli O157:H7 TW14359 (EHEC)	"Prokaryotes;Bacteria;Gammaproteobacteria - Enterobacteria;Escherichia"
697	elx	Escherichia coli O157:H7 Xuzhou21 (EHEC)	"Prokaryotes;Bacteria;Gammaproteobacteria - Enterobacteria;Escherichia"
698	eoi	Escherichia coli O111:H- 11128 (EHEC)	"Prokaryotes;Bacteria;Gammaproteobacteria - Enterobacteria;Escherichia"
699	eoj	Escherichia coli O26:H11 11368 (EHEC)	"Prokaryotes;Bacteria;Gammaproteobacteria - Enterobacteria;Escherichia"
700	eoh	Escherichia coli O103:H2 12009 (EHEC)	"Prokaryotes;Bacteria;Gammaproteobacteria - Enterobacteria;Escherichia"
701	ecoo	Escherichia coli O145:H28 RM13514 (EHEC)	"Prokaryotes;Bacteria;Gammaproteobacteria - Enterobacteria;Escherichia"
702	ecoh	Escherichia coli O145:H28 RM13516 (EHEC)	"Prokaryotes;Bacteria;Gammaproteobacteria - Enterobacteria;Escherichia"
703	esl	Escherichia coli O104:H4 2011C-3493 (EAEC)	"Prokaryotes;Bacteria;Gammaproteobacteria - Enterobacteria;Escherichia"
704	eso	Escherichia coli O104:H4 2009EL-2071 (EAEC)	"Prokaryotes;Bacteria;Gammaproteobacteria - Enterobacteria;Escherichia"
705	esm	Escherichia coli O104:H4 2009EL-2050 (EAEC)	"Prokaryotes;Bacteria;Gammaproteobacteria - Enterobacteria;Escherichia"
706	eck	Escherichia coli 55989 (EAEC)	"Prokaryotes;Bacteria;Gammaproteobacteria - Enterobacteria;Escherichia"
707	ecg	Escherichia coli O127:H6 E2348/69 (EPEC)	"Prokaryotes;Bacteria;Gammaproteobacteria - Enterobacteria;Escherichia"
708	eok	Escherichia coli O55:H7 CB9615 (EPEC)	"Prokaryotes;Bacteria;Gammaproteobacteria - Enterobacteria;Escherichia"
709	elr	Escherichia coli O55:H7 RM12579 (EPEC)	"Prokaryotes;Bacteria;Gammaproteobacteria - Enterobacteria;Escherichia"
710	elh	Escherichia coli O78:H11:K80 H10407 (ETEC)	"Prokaryotes;Bacteria;Gammaproteobacteria - Enterobacteria;Escherichia"
711	ecw	Escherichia coli O139:H28 E24377A (ETEC)	"Prokaryotes;Bacteria;Gammaproteobacteria - Enterobacteria;Escherichia"
712	eun	Escherichia coli UMNK88 (ETEC, porcine)	"Prokaryotes;Bacteria;Gammaproteobacteria - Enterobacteria;Escherichia"
713	ecp	Escherichia coli O6:K15:H31 536 (UPEC)	"Prokaryotes;Bacteria;Gammaproteobacteria - Enterobacteria;Escherichia"
714	ena	Escherichia coli NA114 (UPEC)	"Prokaryotes;Bacteria;Gammaproteobacteria - Enterobacteria;Escherichia"
715	ecos	Escherichia coli O25b:K100:H4-ST131 EC958 (UPEC)	"Prokaryotes;Bacteria;Gammaproteobacteria - Enterobacteria;Escherichia"
716	ecv	Escherichia coli APEC O1 (APEC)	"Prokaryotes;Bacteria;Gammaproteobacteria - Enterobacteria;Escherichia"
717	ecoa	Escherichia coli APEC O78 (APEC)	"Prokaryotes;Bacteria;Gammaproteobacteria - Enterobacteria;Escherichia"
718	ecx	Escherichia coli O9 HS (commensal)	"Prokaryotes;Bacteria;Gammaproteobacteria - Enterobacteria;Escherichia"
719	ecm	Escherichia coli SMS-3-5 (environmental)	"Prokaryotes;Bacteria;Gammaproteobacteria - Enterobacteria;Escherichia"
720	ecy	Escherichia coli O152:H28 SE11 (commensal)	"Prokaryotes;Bacteria;Gammaproteobacteria - Enterobacteria;Escherichia"
721	ecr	Escherichia coli O8 IAI1 (commensal)	"Prokaryotes;Bacteria;Gammaproteobacteria - Enterobacteria;Escherichia"
722	ecq	Escherichia coli O81 ED1a (commensal)	"Prokaryotes;Bacteria;Gammaproteobacteria - Enterobacteria;Escherichia"
723	eum	Escherichia coli O17:K52:H18 UMN026 (ExPEC)	"Prokaryotes;Bacteria;Gammaproteobacteria - Enterobacteria;Escherichia"
724	ect	Escherichia coli O7:K1 IAI39 (ExPEC)	"Prokaryotes;Bacteria;Gammaproteobacteria - Enterobacteria;Escherichia"
725	eoc	Escherichia coli O7:K1 CE10	"Prokaryotes;Bacteria;Gammaproteobacteria - Enterobacteria;Escherichia"
726	ebr	Escherichia coli B REL606	"Prokaryotes;Bacteria;Gammaproteobacteria - Enterobacteria;Escherichia"
727	ebl	Escherichia coli BL21(DE3)	"Prokaryotes;Bacteria;Gammaproteobacteria - Enterobacteria;Escherichia"
728	ebe	Escherichia coli BL21(DE3)	"Prokaryotes;Bacteria;Gammaproteobacteria - Enterobacteria;Escherichia"
729	ebd	Escherichia coli BL21-Gold(DE3)pLysS AG	"Prokaryotes;Bacteria;Gammaproteobacteria - Enterobacteria;Escherichia"
730	eci	Escherichia coli O18:K1:H7 UTI89 (UPEC)	"Prokaryotes;Bacteria;Gammaproteobacteria - Enterobacteria;Escherichia"
731	eih	Escherichia coli O18:K1:H7 IHE3034 (ExPEC)	"Prokaryotes;Bacteria;Gammaproteobacteria - Enterobacteria;Escherichia"
732	ecz	Escherichia coli O45:K1:H7 S88 (ExPEC)	"Prokaryotes;Bacteria;Gammaproteobacteria - Enterobacteria;Escherichia"
733	ecc	Escherichia coli O6:K2:H1 CFT073 (UPEC)	"Prokaryotes;Bacteria;Gammaproteobacteria - Enterobacteria;Escherichia"
734	elo	Escherichia coli O44:H18 042 (EAEC)	"Prokaryotes;Bacteria;Gammaproteobacteria - Enterobacteria;Escherichia"
735	eln	Escherichia coli O83:H1 NRG 857C (AIEC)	"Prokaryotes;Bacteria;Gammaproteobacteria - Enterobacteria;Escherichia"
736	ese	Escherichia coli O150:H5 SE15 (commensal)	"Prokaryotes;Bacteria;Gammaproteobacteria - Enterobacteria;Escherichia"

737	ecl	Escherichia coli ATCC 8739	"Prokaryotes;Bacteria;Gammaproteobacteria - Enterobacteria;Escherichia"
738	eko	Escherichia coli KO11FL	"Prokaryotes;Bacteria;Gammaproteobacteria - Enterobacteria;Escherichia"
739	ekf	Escherichia coli KO11FL	"Prokaryotes;Bacteria;Gammaproteobacteria - Enterobacteria;Escherichia"
740	eab	Escherichia coli ABU 83972	"Prokaryotes;Bacteria;Gammaproteobacteria - Enterobacteria;Escherichia"
741	edh	Escherichia coli DH1	"Prokaryotes;Bacteria;Gammaproteobacteria - Enterobacteria;Escherichia"
742	edj	Escherichia coli DH1	"Prokaryotes;Bacteria;Gammaproteobacteria - Enterobacteria;Escherichia"
743	elu	Escherichia coli UM146	"Prokaryotes;Bacteria;Gammaproteobacteria - Enterobacteria;Escherichia"
744	elw	Escherichia coli W	"Prokaryotes;Bacteria;Gammaproteobacteria - Enterobacteria;Escherichia"
745	ell	Escherichia coli W	"Prokaryotes;Bacteria;Gammaproteobacteria - Enterobacteria;Escherichia"
746	elc	Escherichia coli clone D i14	"Prokaryotes;Bacteria;Gammaproteobacteria - Enterobacteria;Escherichia"
747	eld	Escherichia coli clone D i2	"Prokaryotes;Bacteria;Gammaproteobacteria - Enterobacteria;Escherichia"
748	elp	Escherichia coli P12b	"Prokaryotes;Bacteria;Gammaproteobacteria - Enterobacteria;Escherichia"
749	elf	Escherichia coli LF82	"Prokaryotes;Bacteria;Gammaproteobacteria - Enterobacteria;Escherichia"
750	ecol	Escherichia coli LY180	"Prokaryotes;Bacteria;Gammaproteobacteria - Enterobacteria;Escherichia"
751	ecoi	Escherichia coli O18:K1 PMV-1 (ExPEC)	"Prokaryotes;Bacteria;Gammaproteobacteria - Enterobacteria;Escherichia"
752	ecoj	Escherichia coli JJ1886	"Prokaryotes;Bacteria;Gammaproteobacteria - Enterobacteria;Escherichia"
753	efe	Escherichia fergusonii	"Prokaryotes;Bacteria;Gammaproteobacteria - Enterobacteria;Escherichia"
754	eal	Escherichia albertii	"Prokaryotes;Bacteria;Gammaproteobacteria - Enterobacteria;Escherichia"
755	ema	Escherichia marmotae	"Prokaryotes;Bacteria;Gammaproteobacteria - Enterobacteria;Escherichia"
756	esz	Escherichia sp. E4742	"Prokaryotes;Bacteria;Gammaproteobacteria - Enterobacteria;Escherichia"
757	sty	Salmonella enterica subsp. enterica serovar Typhi CT18	"Prokaryotes;Bacteria;Gammaproteobacteria - Enterobacteria;Salmonella"
758	stt	Salmonella enterica subsp. enterica serovar Typhi Ty2	"Prokaryotes;Bacteria;Gammaproteobacteria - Enterobacteria;Salmonella"
759	sex	Salmonella enterica subsp. enterica serovar Typhi P-stx-12	"Prokaryotes;Bacteria;Gammaproteobacteria - Enterobacteria;Salmonella"
760	sent	Salmonella enterica subsp. enterica serovar Typhi Ty21a	"Prokaryotes;Bacteria;Gammaproteobacteria - Enterobacteria;Salmonella"
761	stm	Salmonella enterica subsp. enterica serovar Typhimurium LT2	"Prokaryotes;Bacteria;Gammaproteobacteria - Enterobacteria;Salmonella"
762	seo	Salmonella enterica subsp. enterica serovar Typhimurium 14028S	"Prokaryotes;Bacteria;Gammaproteobacteria - Enterobacteria;Salmonella"
763	sev	Salmonella enterica subsp. enterica serovar Typhimurium D23580	"Prokaryotes;Bacteria;Gammaproteobacteria - Enterobacteria;Salmonella"
764	sey	Salmonella enterica subsp. enterica serovar Typhimurium SL1344	"Prokaryotes;Bacteria;Gammaproteobacteria - Enterobacteria;Salmonella"
765	sem	Salmonella enterica subsp. enterica serovar Typhimurium T000240	"Prokaryotes;Bacteria;Gammaproteobacteria - Enterobacteria;Salmonella"
766	sej	Salmonella enterica subsp. enterica serovar Typhimurium UK-1	"Prokaryotes;Bacteria;Gammaproteobacteria - Enterobacteria;Salmonella"
767	seb	Salmonella enterica subsp. enterica serovar Typhimurium ST4/74	"Prokaryotes;Bacteria;Gammaproteobacteria - Enterobacteria;Salmonella"
768	sef	Salmonella enterica subsp. enterica serovar Typhimurium 798	"Prokaryotes;Bacteria;Gammaproteobacteria - Enterobacteria;Salmonella"
769	setu	Salmonella enterica subsp. enterica serovar Typhimurium U288	"Prokaryotes;Bacteria;Gammaproteobacteria - Enterobacteria;Salmonella"
770	setc	Salmonella enterica subsp. enterica serovar Typhimurium var. 5-CFSAN001921	"Prokaryotes;Bacteria;Gammaproteobacteria - Enterobacteria;Salmonella"
771	senr	Salmonella enterica subsp. enterica serovar Typhimurium DT2	"Prokaryotes;Bacteria;Gammaproteobacteria - Enterobacteria;Salmonella"
772	send	Salmonella enterica subsp. enterica serovar Typhimurium DT104	"Prokaryotes;Bacteria;Gammaproteobacteria - Enterobacteria;Salmonella"
773	seni	Salmonella enterica subsp. enterica serovar Typhimurium 138736	"Prokaryotes;Bacteria;Gammaproteobacteria - Enterobacteria;Salmonella"
774	seen	Salmonella enterica subsp. enterica serovar 4,[5],12:i:- str. 08-1736	"Prokaryotes;Bacteria;Gammaproteobacteria - Enterobacteria;Salmonella"
775	spt	Salmonella enterica subsp. enterica serovar Paratyphi A ATCC9150	"Prokaryotes;Bacteria;Gammaproteobacteria - Enterobacteria;Salmonella"
776	sek	Salmonella enterica subsp. enterica serovar Paratyphi A AKU12601	"Prokaryotes;Bacteria;Gammaproteobacteria - Enterobacteria;Salmonella"

777	spq	Salmonella enterica subsp. enterica serovar Paratyphi B	"Prokaryotes;Bacteria;Gammaproteobacteria - Enterobacteria;Salmonella"
778	sei	Salmonella enterica subsp. enterica serovar Paratyphi C	"Prokaryotes;Bacteria;Gammaproteobacteria - Enterobacteria;Salmonella"
779	sec	Salmonella enterica subsp. enterica serovar Choleraesuis	"Prokaryotes;Bacteria;Gammaproteobacteria - Enterobacteria;Salmonella"
780	seh	Salmonella enterica subsp. enterica serovar Heidelberg SL476	"Prokaryotes;Bacteria;Gammaproteobacteria - Enterobacteria;Salmonella"
781	shb	Salmonella enterica subsp. enterica serovar Heidelberg B182	"Prokaryotes;Bacteria;Gammaproteobacteria - Enterobacteria;Salmonella"
782	senh	Salmonella enterica subsp. enterica serovar Heidelberg CFSAN002069	"Prokaryotes;Bacteria;Gammaproteobacteria - Enterobacteria;Salmonella"
783	seeh	Salmonella enterica subsp. enterica serovar Heidelberg 41578	"Prokaryotes;Bacteria;Gammaproteobacteria - Enterobacteria;Salmonella"
784	see	Salmonella enterica subsp. enterica serovar Newport SL254	"Prokaryotes;Bacteria;Gammaproteobacteria - Enterobacteria;Salmonella"
785	senn	Salmonella enterica subsp. enterica serovar Newport USMARC-S3124.1	"Prokaryotes;Bacteria;Gammaproteobacteria - Enterobacteria;Salmonella"
786	sew	Salmonella enterica subsp. enterica serovar Schwarzengrund	"Prokaryotes;Bacteria;Gammaproteobacteria - Enterobacteria;Salmonella"
787	sea	Salmonella enterica subsp. enterica serovar Agona SL483	"Prokaryotes;Bacteria;Gammaproteobacteria - Enterobacteria;Salmonella"
788	sens	Salmonella enterica subsp. enterica serovar Agona 24249	"Prokaryotes;Bacteria;Gammaproteobacteria - Enterobacteria;Salmonella"
789	sed	Salmonella enterica subsp. enterica serovar Dublin	"Prokaryotes;Bacteria;Gammaproteobacteria - Enterobacteria;Salmonella"
790	seg	Salmonella enterica subsp. enterica serovar Gallinarum 287/91	"Prokaryotes;Bacteria;Gammaproteobacteria - Enterobacteria;Salmonella"
791	sel	Salmonella enterica subsp. enterica serovar Gallinarum/pullorum RKS5078	"Prokaryotes;Bacteria;Gammaproteobacteria - Enterobacteria;Salmonella"
792	sega	Salmonella enterica subsp. enterica serovar Gallinarum/pullorum CDC1983-67	"Prokaryotes;Bacteria;Gammaproteobacteria - Enterobacteria;Salmonella"
793	set	Salmonella enterica subsp. enterica serovar Enteritidis P125109	"Prokaryotes;Bacteria;Gammaproteobacteria - Enterobacteria;Salmonella"
794	sena	Salmonella enterica subsp. enterica serovar Enteritidis EC20090135	"Prokaryotes;Bacteria;Gammaproteobacteria - Enterobacteria;Salmonella"
795	seno	Salmonella enterica subsp. enterica serovar Enteritidis EC20090193	"Prokaryotes;Bacteria;Gammaproteobacteria - Enterobacteria;Salmonella"
796	senv	Salmonella enterica subsp. enterica serovar Enteritidis EC20090332	"Prokaryotes;Bacteria;Gammaproteobacteria - Enterobacteria;Salmonella"
797	senq	Salmonella enterica subsp. enterica serovar Enteritidis EC20090531	"Prokaryotes;Bacteria;Gammaproteobacteria - Enterobacteria;Salmonella"
798	senl	Salmonella enterica subsp. enterica serovar Enteritidis OLF-SE1-1019-1	"Prokaryotes;Bacteria;Gammaproteobacteria - Enterobacteria;Salmonella"
799	senj	Salmonella enterica subsp. enterica serovar Javiana	"Prokaryotes;Bacteria;Gammaproteobacteria - Enterobacteria;Salmonella"
800	seec	Salmonella enterica subsp. enterica serovar Cubana	"Prokaryotes;Bacteria;Gammaproteobacteria - Enterobacteria;Salmonella"
801	seeb	Salmonella enterica subsp. enterica serovar Bareilly	"Prokaryotes;Bacteria;Gammaproteobacteria - Enterobacteria;Salmonella"
802	seep	Salmonella enterica subsp. enterica serovar Pullorum	"Prokaryotes;Bacteria;Gammaproteobacteria - Enterobacteria;Salmonella"
803	senb	Salmonella enterica subsp. enterica serovar Bovismorbificans	"Prokaryotes;Bacteria;Gammaproteobacteria - Enterobacteria;Salmonella"
804	sene	Salmonella enterica subsp. enterica serovar Thompson	"Prokaryotes;Bacteria;Gammaproteobacteria - Enterobacteria;Salmonella"
805	senc	Salmonella enterica subsp. enterica serovar Tennessee	"Prokaryotes;Bacteria;Gammaproteobacteria - Enterobacteria;Salmonella"
806	ses	Salmonella enterica subsp. arizonae	"Prokaryotes;Bacteria;Gammaproteobacteria - Enterobacteria;Salmonella"
807	sbg	Salmonella bongori NCTC 12419	"Prokaryotes;Bacteria;Gammaproteobacteria - Enterobacteria;Salmonella"
808	sbz	Salmonella bongori N268-08	"Prokaryotes;Bacteria;Gammaproteobacteria - Enterobacteria;Salmonella"
809	sbv	Salmonella bongori serovar 48:z41:--	"Prokaryotes;Bacteria;Gammaproteobacteria - Enterobacteria;Salmonella"
810	salz	Salmonella sp. SSDFZ69	"Prokaryotes;Bacteria;Gammaproteobacteria - Enterobacteria;Salmonella"
811	sfl	Shigella flexneri 301 (serotype 2a)	"Prokaryotes;Bacteria;Gammaproteobacteria - Enterobacteria;Shigella"
812	sfx	Shigella flexneri 2457T (serotype 2a)	"Prokaryotes;Bacteria;Gammaproteobacteria - Enterobacteria;Shigella"
813	sfv	Shigella flexneri 8401 (serotype 5b)	"Prokaryotes;Bacteria;Gammaproteobacteria - Enterobacteria;Shigella"
814	sfe	Shigella flexneri 2002017 (serotype Fxv)	"Prokaryotes;Bacteria;Gammaproteobacteria - Enterobacteria;Shigella"
815	sfn	Shigella flexneri 2003036	"Prokaryotes;Bacteria;Gammaproteobacteria - Enterobacteria;Shigella"

816	sfs	Shigella flexneri Shi06HN006 (serotype Yv)	"Prokaryotes;Bacteria;Gammaproteobacteria - Enterobacteria;Shigella"
817	sft	Shigella flexneri NCTC1 (serotype 2a)	"Prokaryotes;Bacteria;Gammaproteobacteria - Enterobacteria;Shigella"
818	ssn	Shigella sonnei Ss046	"Prokaryotes;Bacteria;Gammaproteobacteria - Enterobacteria;Shigella"
819	sbo	Shigella boydii Sb227 (serotype 4)	"Prokaryotes;Bacteria;Gammaproteobacteria - Enterobacteria;Shigella"
820	sbc	Shigella boydii CDC 3083-94 (serotype 18)	"Prokaryotes;Bacteria;Gammaproteobacteria - Enterobacteria;Shigella"
821	sdy	Shigella dysenteriae Sd197	"Prokaryotes;Bacteria;Gammaproteobacteria - Enterobacteria;Shigella"
822	sdz	Shigella dysenteriae 1617 (serotype 1)	"Prokaryotes;Bacteria;Gammaproteobacteria - Enterobacteria;Shigella"
823	shq	Shigella sp. PAMC 28760	"Prokaryotes;Bacteria;Gammaproteobacteria - Enterobacteria;Shigella"
824	enc	Enterobacter cloacae subsp. cloacae ATCC 13047	"Prokaryotes;Bacteria;Gammaproteobacteria - Enterobacteria;Enterobacter"
825	enl	Enterobacter cloacae subsp. dissolvens SDM	"Prokaryotes;Bacteria;Gammaproteobacteria - Enterobacteria;Enterobacter"
826	eclg	Enterobacter cloacae GGT036	"Prokaryotes;Bacteria;Gammaproteobacteria - Enterobacteria;Enterobacter"
827	ecle	Enterobacter cloacae ECNIH2	"Prokaryotes;Bacteria;Gammaproteobacteria - Enterobacteria;Enterobacter"
828	ecln	Enterobacter cloacae ECNIH4	"Prokaryotes;Bacteria;Gammaproteobacteria - Enterobacteria;Enterobacter"
829	ecli	Enterobacter cloacae ECNIH5	"Prokaryotes;Bacteria;Gammaproteobacteria - Enterobacteria;Enterobacter"
830	eclx	Enterobacter hormaechei subsp. xiangfangensis	"Prokaryotes;Bacteria;Gammaproteobacteria - Enterobacteria;Enterobacter"
831	ecly	Enterobacter hormaechei subsp. steigerwaltii	"Prokaryotes;Bacteria;Gammaproteobacteria - Enterobacteria;Enterobacter"
832	eclz	Enterobacter hormaechei subsp. hormaechei	"Prokaryotes;Bacteria;Gammaproteobacteria - Enterobacteria;Enterobacter"
833	eclo	Enterobacter hormaechei NCTC 9394	"Prokaryotes;Bacteria;Gammaproteobacteria - Enterobacteria;Enterobacter"
834	ehm	Enterobacter hormaechei CAV1176	"Prokaryotes;Bacteria;Gammaproteobacteria - Enterobacteria;Enterobacter"
835	exf	Enterobacter hormaechei subsp. xiangfangensis	"Prokaryotes;Bacteria;Gammaproteobacteria - Enterobacteria;Enterobacter"
836	ecla	Enterobacter hormaechei subsp. hoffmannii ECNIH3	"Prokaryotes;Bacteria;Gammaproteobacteria - Enterobacteria;Enterobacter"
837	eclc	Enterobacter hormaechei subsp. hoffmannii ECR091	"Prokaryotes;Bacteria;Gammaproteobacteria - Enterobacteria;Enterobacter"
838	eau	Enterobacter asburiae L1	"Prokaryotes;Bacteria;Gammaproteobacteria - Enterobacteria;Enterobacter"
839	ekb	Enterobacter kobei DSM 13645	"Prokaryotes;Bacteria;Gammaproteobacteria - Enterobacteria;Enterobacter"
840	eno	Enterobacter kobei ENHKU01	"Prokaryotes;Bacteria;Gammaproteobacteria - Enterobacteria;Enterobacter"
841	eec	Enterobacter ludwigii EcWSU1	"Prokaryotes;Bacteria;Gammaproteobacteria - Enterobacteria;Enterobacter"
842	elg	Enterobacter ludwigii EN-119	"Prokaryotes;Bacteria;Gammaproteobacteria - Enterobacteria;Enterobacter"
843	ecan	Enterobacter cancerogenus	"Prokaryotes;Bacteria;Gammaproteobacteria - Enterobacteria;Enterobacter"
844	ern	Enterobacter roggenkampii DSM 16690	"Prokaryotes;Bacteria;Gammaproteobacteria - Enterobacteria;Enterobacter"
845	ecls	Enterobacter roggenkampii 35734	"Prokaryotes;Bacteria;Gammaproteobacteria - Enterobacteria;Enterobacter"
846	echg	Enterobacter chengduensis	"Prokaryotes;Bacteria;Gammaproteobacteria - Enterobacteria;Enterobacter"
847	esh	Enterobacter sichuanensis	"Prokaryotes;Bacteria;Gammaproteobacteria - Enterobacteria;Enterobacter"
848	ent	Enterobacter sp. 638	"Prokaryotes;Bacteria;Gammaproteobacteria - Enterobacteria;Enterobacter"
849	eas	Enterobacter soli	"Prokaryotes;Bacteria;Gammaproteobacteria - Enterobacteria;Enterobacter"
850	enr	Enterobacter sp. R4-368	"Prokaryotes;Bacteria;Gammaproteobacteria - Enterobacteria;Enterobacter"
851	enx	Enterobacter sp. E20	"Prokaryotes;Bacteria;Gammaproteobacteria - Enterobacteria;Enterobacter"
852	enf	Enterobacter sp. FY-07	"Prokaryotes;Bacteria;Gammaproteobacteria - Enterobacteria;Enterobacter"
853	ebg	Enterobacter bugandensis	"Prokaryotes;Bacteria;Gammaproteobacteria - Enterobacteria;Enterobacter"
854	end	Enterobacter sp. ODB01	"Prokaryotes;Bacteria;Gammaproteobacteria - Enterobacteria;Enterobacter"
855	esa	Cronobacter sakazakii ATCC BAA-894	"Prokaryotes;Bacteria;Gammaproteobacteria - Enterobacteria;Cronobacter"
856	csk	Cronobacter sakazakii ES15	"Prokaryotes;Bacteria;Gammaproteobacteria - Enterobacteria;Cronobacter"

857	csz	Cronobacter sakazakii Sp291	"Prokaryotes;Bacteria;Gammaproteobacteria - Enterobacteria;Cronobacter"
858	csj	Cronobacter sakazakii ATCC 29544	"Prokaryotes;Bacteria;Gammaproteobacteria - Enterobacteria;Cronobacter"
859	ccon	Cronobacter condimenti	"Prokaryotes;Bacteria;Gammaproteobacteria - Enterobacteria;Cronobacter"
860	cdm	Cronobacter dublinensis	"Prokaryotes;Bacteria;Gammaproteobacteria - Enterobacteria;Cronobacter"
861	csi	Cronobacter malonaticus CMCC45402	"Prokaryotes;Bacteria;Gammaproteobacteria - Enterobacteria;Cronobacter"
862	cmj	Cronobacter malonaticus LMG 23826	"Prokaryotes;Bacteria;Gammaproteobacteria - Enterobacteria;Cronobacter"
863	cui	Cronobacter universalis	"Prokaryotes;Bacteria;Gammaproteobacteria - Enterobacteria;Cronobacter"
864	cmw	Cronobacter muytjensii	"Prokaryotes;Bacteria;Gammaproteobacteria - Enterobacteria;Cronobacter"
865	ctu	Cronobacter turicensis	"Prokaryotes;Bacteria;Gammaproteobacteria - Enterobacteria;Cronobacter"
866	kpn	Klebsiella pneumoniae subsp. pneumoniae MGH 78578 (serotype K52)	"Prokaryotes;Bacteria;Gammaproteobacteria - Enterobacteria;Klebsiella"
867	kpu	Klebsiella pneumoniae subsp. pneumoniae NTUH-K2044 (serotype K1)	"Prokaryotes;Bacteria;Gammaproteobacteria - Enterobacteria;Klebsiella"
868	kpm	Klebsiella pneumoniae subsp. pneumoniae HS11286	"Prokaryotes;Bacteria;Gammaproteobacteria - Enterobacteria;Klebsiella"
869	kpp	Klebsiella pneumoniae subsp. pneumoniae 1084 (serotype K1)	"Prokaryotes;Bacteria;Gammaproteobacteria - Enterobacteria;Klebsiella"
870	kph	Klebsiella pneumoniae subsp. pneumoniae KPNIH24	"Prokaryotes;Bacteria;Gammaproteobacteria - Enterobacteria;Klebsiella"
871	kpz	Klebsiella pneumoniae subsp. pneumoniae KPNIH27	"Prokaryotes;Bacteria;Gammaproteobacteria - Enterobacteria;Klebsiella"
872	kpv	Klebsiella pneumoniae subsp. pneumoniae KPNIH29	"Prokaryotes;Bacteria;Gammaproteobacteria - Enterobacteria;Klebsiella"
873	kpw	Klebsiella pneumoniae subsp. pneumoniae KPNIH30	"Prokaryotes;Bacteria;Gammaproteobacteria - Enterobacteria;Klebsiella"
874	kpy	Klebsiella pneumoniae subsp. pneumoniae KPNIH31	"Prokaryotes;Bacteria;Gammaproteobacteria - Enterobacteria;Klebsiella"
875	kpg	Klebsiella pneumoniae subsp. pneumoniae KPNIH32	"Prokaryotes;Bacteria;Gammaproteobacteria - Enterobacteria;Klebsiella"
876	kpc	Klebsiella pneumoniae subsp. pneumoniae KPNIH10	"Prokaryotes;Bacteria;Gammaproteobacteria - Enterobacteria;Klebsiella"
877	kpq	Klebsiella pneumoniae subsp. pneumoniae KPR0928	"Prokaryotes;Bacteria;Gammaproteobacteria - Enterobacteria;Klebsiella"
878	kpt	Klebsiella pneumoniae subsp. pneumoniae ATCC 43816 KPPR1	"Prokaryotes;Bacteria;Gammaproteobacteria - Enterobacteria;Klebsiella"
879	kpo	Klebsiella pneumoniae KCTC 2242	"Prokaryotes;Bacteria;Gammaproteobacteria - Enterobacteria;Klebsiella"
880	kpr	Klebsiella pneumoniae subsp. rhinoscleromatis SB3432	"Prokaryotes;Bacteria;Gammaproteobacteria - Enterobacteria;Klebsiella"
881	kpj	Klebsiella pneumoniae JM45	"Prokaryotes;Bacteria;Gammaproteobacteria - Enterobacteria;Klebsiella"
882	kpi	Klebsiella pneumoniae CG43	"Prokaryotes;Bacteria;Gammaproteobacteria - Enterobacteria;Klebsiella"
883	kpa	Klebsiella pneumoniae 30660/NJST258_1	"Prokaryotes;Bacteria;Gammaproteobacteria - Enterobacteria;Klebsiella"
884	kps	Klebsiella pneumoniae 30684/NJST258_2	"Prokaryotes;Bacteria;Gammaproteobacteria - Enterobacteria;Klebsiella"
885	kpx	Klebsiella pneumoniae PMK1	"Prokaryotes;Bacteria;Gammaproteobacteria - Enterobacteria;Klebsiella"
886	kpb	Klebsiella pneumoniae blaNDM-1	"Prokaryotes;Bacteria;Gammaproteobacteria - Enterobacteria;Klebsiella"
887	kpne	Klebsiella pneumoniae 32192	"Prokaryotes;Bacteria;Gammaproteobacteria - Enterobacteria;Klebsiella"
888	kpnu	Klebsiella pneumoniae 34618	"Prokaryotes;Bacteria;Gammaproteobacteria - Enterobacteria;Klebsiella"
889	kpnk	Klebsiella pneumoniae Kp52.145	"Prokaryotes;Bacteria;Gammaproteobacteria - Enterobacteria;Klebsiella"
890	kva	Klebsiella variicola At-22	"Prokaryotes;Bacteria;Gammaproteobacteria - Enterobacteria;Klebsiella"
891	kpe	Klebsiella variicola 342	"Prokaryotes;Bacteria;Gammaproteobacteria - Enterobacteria;Klebsiella"
892	kpk	Klebsiella variicola KP5-1	"Prokaryotes;Bacteria;Gammaproteobacteria - Enterobacteria;Klebsiella"
893	kvd	Klebsiella variicola DX120E	"Prokaryotes;Bacteria;Gammaproteobacteria - Enterobacteria;Klebsiella"
894	kvq	Klebsiella variicola DSM 15968	"Prokaryotes;Bacteria;Gammaproteobacteria - Enterobacteria;Klebsiella"
895	kox	Klebsiella michiganensis KCTC 1686	"Prokaryotes;Bacteria;Gammaproteobacteria - Enterobacteria;Klebsiella"
896	koe	Klebsiella michiganensis E718	"Prokaryotes;Bacteria;Gammaproteobacteria - Enterobacteria;Klebsiella"
897	koy	Klebsiella michiganensis HKOPL1	"Prokaryotes;Bacteria;Gammaproteobacteria - Enterobacteria;Klebsiella"

898	kom	Klebsiella michiganensis M1	"Prokaryotes;Bacteria;Gammaproteobacteria - Enterobacteria;Klebsiella"
899	kmi	Klebsiella michiganensis RC10	"Prokaryotes;Bacteria;Gammaproteobacteria - Enterobacteria;Klebsiella"
900	kok	Klebsiella oxytoca KONIH1	"Prokaryotes;Bacteria;Gammaproteobacteria - Enterobacteria;Klebsiella"
901	koc	Klebsiella oxytoca CAV1374	"Prokaryotes;Bacteria;Gammaproteobacteria - Enterobacteria;Klebsiella"
902	kqu	Klebsiella quasipneumoniae	"Prokaryotes;Bacteria;Gammaproteobacteria - Enterobacteria;Klebsiella"
903	eae	Klebsiella aerogenes KCTC 2190	"Prokaryotes;Bacteria;Gammaproteobacteria - Enterobacteria;Klebsiella"
904	ear	Klebsiella aerogenes EA1509E	"Prokaryotes;Bacteria;Gammaproteobacteria - Enterobacteria;Klebsiella"
905	kqv	Klebsiella quasivariicola	"Prokaryotes;Bacteria;Gammaproteobacteria - Enterobacteria;Klebsiella"
906	kll	Klebsiella sp. LTGPAF-6F	"Prokaryotes;Bacteria;Gammaproteobacteria - Enterobacteria;Klebsiella"
907	klw	Klebsiella huaxiensis	"Prokaryotes;Bacteria;Gammaproteobacteria - Enterobacteria;Klebsiella"
908	cro	Citrobacter rodentium	"Prokaryotes;Bacteria;Gammaproteobacteria - Enterobacteria;Citrobacter"
909	cko	Citrobacter koseri	"Prokaryotes;Bacteria;Gammaproteobacteria - Enterobacteria;Citrobacter"
910	cfd	Citrobacter freundii	"Prokaryotes;Bacteria;Gammaproteobacteria - Enterobacteria;Citrobacter"
911	cbra	Citrobacter braakii	"Prokaryotes;Bacteria;Gammaproteobacteria - Enterobacteria;Citrobacter"
912	cwe	Citrobacter werkmanii	"Prokaryotes;Bacteria;Gammaproteobacteria - Enterobacteria;Citrobacter"
913	cyo	Citrobacter youngae	"Prokaryotes;Bacteria;Gammaproteobacteria - Enterobacteria;Citrobacter"
914	cpot	Citrobacter portucalensis	"Prokaryotes;Bacteria;Gammaproteobacteria - Enterobacteria;Citrobacter"
915	cfq	Citrobacter freundii complex sp. CFNIH3	"Prokaryotes;Bacteria;Gammaproteobacteria - Enterobacteria;Citrobacter"
916	cama	Citrobacter amalonaticus Y19	"Prokaryotes;Bacteria;Gammaproteobacteria - Enterobacteria;Citrobacter"
917	caf	Citrobacter amalonaticus FDAARGOS_165	"Prokaryotes;Bacteria;Gammaproteobacteria - Enterobacteria;Citrobacter"
918	cif	Citrobacter sp. FDAARGOS_156	"Prokaryotes;Bacteria;Gammaproteobacteria - Enterobacteria;Citrobacter"
919	cfar	Citrobacter farmeri	"Prokaryotes;Bacteria;Gammaproteobacteria - Enterobacteria;Citrobacter"
920	cir	Citrobacter sp. CFNIH10	"Prokaryotes;Bacteria;Gammaproteobacteria - Enterobacteria;Citrobacter"
921	cie	Citrobacter sp. CRE-46	"Prokaryotes;Bacteria;Gammaproteobacteria - Enterobacteria;Citrobacter"
922	cpar	Citrobacter pasteurii	"Prokaryotes;Bacteria;Gammaproteobacteria - Enterobacteria;Citrobacter"
923	bfl	Candidatus Blochmannia floridanus	"Prokaryotes;Bacteria;Gammaproteobacteria - Enterobacteria;Blochmannia"
924	bpn	Candidatus Blochmannia pennsylvanicus	"Prokaryotes;Bacteria;Gammaproteobacteria - Enterobacteria;Blochmannia"
925	bva	Candidatus Blochmannia vafer	"Prokaryotes;Bacteria;Gammaproteobacteria - Enterobacteria;Blochmannia"
926	bchr	Candidatus Blochmannia chromaiodes	"Prokaryotes;Bacteria;Gammaproteobacteria - Enterobacteria;Blochmannia"
927	ben	Blochmannia endosymbiont of Camponotus (Colobopsis) obliquus 757	"Prokaryotes;Bacteria;Gammaproteobacteria - Enterobacteria;Blochmannia"
928	bed	Blochmannia endosymbiont of Polyrhachis (Hedomyrma) turneri 675	"Prokaryotes;Bacteria;Gammaproteobacteria - Enterobacteria;Blochmannia"
929	hde	Candidatus Hamiltonella defensa (Acyrthosiphon pisum)	"Prokaryotes;Bacteria;Gammaproteobacteria - Enterobacteria;Hamiltonella"
930	sect	Secondary endosymbiont of Ctenarytaina eucalypti	"Prokaryotes;Bacteria;Gammaproteobacteria - Enterobacteria;unclassified aphid symbionts"
931	sehc	Secondary endosymbiont of Heteropsylla cubana	"Prokaryotes;Bacteria;Gammaproteobacteria - Enterobacteria;unclassified aphid symbionts"
932	senm	Secondary endosymbiont of Trabutina mannipara	"Prokaryotes;Bacteria;Gammaproteobacteria - Enterobacteria;unclassified mealybug symbionts"
933	rip	Candidatus Riesia pediculicola	"Prokaryotes;Bacteria;Gammaproteobacteria - Enterobacteria;Riesia"
934	rig	Candidatus Riesia sp. GBBU	"Prokaryotes;Bacteria;Gammaproteobacteria - Enterobacteria;Riesia"
935	men	Candidatus Moranella endobia PCIT	"Prokaryotes;Bacteria;Gammaproteobacteria - Enterobacteria;Moranella"
936	meo	Candidatus Moranella endobia PCVAL	"Prokaryotes;Bacteria;Gammaproteobacteria - Enterobacteria;Moranella"
937	ebt	Shimwellia blattae	"Prokaryotes;Bacteria;Gammaproteobacteria - Enterobacteria;Shimwellia"

938	ror	Raoultella ornithinolytica B6	"Prokaryotes;Bacteria;Gammaproteobacteria - Enterobacteria;Raoultella"
939	ron	Raoultella ornithinolytica S12	"Prokaryotes;Bacteria;Gammaproteobacteria - Enterobacteria;Raoultella"
940	rpln	Raoultella planticola	"Prokaryotes;Bacteria;Gammaproteobacteria - Enterobacteria;Raoultella"
941	rao	Raoultella sp. X13	"Prokaryotes;Bacteria;Gammaproteobacteria - Enterobacteria;Raoultella"
942	rtg	Raoultella terrigena	"Prokaryotes;Bacteria;Gammaproteobacteria - Enterobacteria;Raoultella"
943	ree	Raoultella electrica	"Prokaryotes;Bacteria;Gammaproteobacteria - Enterobacteria;Raoultella"
944	cnt	Cedecea neteri SSMD04	"Prokaryotes;Bacteria;Gammaproteobacteria - Enterobacteria;Cedecea"
945	cem	Cedecea neteri M006	"Prokaryotes;Bacteria;Gammaproteobacteria - Enterobacteria;Cedecea"
946	cen	Cedecea neteri ND14a	"Prokaryotes;Bacteria;Gammaproteobacteria - Enterobacteria;Cedecea"
947	clap	Cedecea lapagei	"Prokaryotes;Bacteria;Gammaproteobacteria - Enterobacteria;Cedecea"
948	pge	Pluralibacter gergoviae	"Prokaryotes;Bacteria;Gammaproteobacteria - Enterobacteria;Pluralibacter"
949	esc	Enterobacter lignolyticus SCF1	"Prokaryotes;Bacteria;Gammaproteobacteria - Enterobacteria;Pluralibacter"
950	kle	Enterobacter lignolyticus G5	"Prokaryotes;Bacteria;Gammaproteobacteria - Enterobacteria;Pluralibacter"
951	ksa	Kosakonia sacchari	"Prokaryotes;Bacteria;Gammaproteobacteria - Enterobacteria;Kosakonia"
952	kor	Kosakonia oryzae	"Prokaryotes;Bacteria;Gammaproteobacteria - Enterobacteria;Kosakonia"
953	krd	Kosakonia radicincitans	"Prokaryotes;Bacteria;Gammaproteobacteria - Enterobacteria;Kosakonia"
954	kco	Kosakonia cowanii	"Prokaryotes;Bacteria;Gammaproteobacteria - Enterobacteria;Kosakonia"
955	kot	Kosakonia sp. CCTCC M2018092	"Prokaryotes;Bacteria;Gammaproteobacteria - Enterobacteria;Kosakonia"
956	kpse	Kosakonia pseudosacchari	"Prokaryotes;Bacteria;Gammaproteobacteria - Enterobacteria;Kosakonia"
957	kgo	Kluyvera genomosp. 3	"Prokaryotes;Bacteria;Gammaproteobacteria - Enterobacteria;Kluyvera"
958	kie	Kluyvera intermedia	"Prokaryotes;Bacteria;Gammaproteobacteria - Enterobacteria;Kluyvera"
959	kas	Kluyvera ascorbata	"Prokaryotes;Bacteria;Gammaproteobacteria - Enterobacteria;Kluyvera"
960	icp	Candidatus Ishikawaella capsulata	"Prokaryotes;Bacteria;Gammaproteobacteria - Enterobacteria;Ishikawaella"
961	lax	Leclercia adecarboxylata	"Prokaryotes;Bacteria;Gammaproteobacteria - Enterobacteria;Leclercia"
962	lei	Leclercia sp. LSNIH1	"Prokaryotes;Bacteria;Gammaproteobacteria - Enterobacteria;Leclercia"
963	leh	Leclercia sp. LSNIH3	"Prokaryotes;Bacteria;Gammaproteobacteria - Enterobacteria;Leclercia"
964	lee	Leclercia sp. W17	"Prokaryotes;Bacteria;Gammaproteobacteria - Enterobacteria;Leclercia"
965	ler	Leclercia sp. 1106151	"Prokaryotes;Bacteria;Gammaproteobacteria - Enterobacteria;Leclercia"
966	lea	Leclercia sp. J807	"Prokaryotes;Bacteria;Gammaproteobacteria - Enterobacteria;Leclercia"
967	laz	Lelliottia amnigena	"Prokaryotes;Bacteria;Gammaproteobacteria - Enterobacteria;Lelliottia"
968	lef	Lelliottia jeotgali	"Prokaryotes;Bacteria;Gammaproteobacteria - Enterobacteria;Lelliottia"
969	lni	Lelliottia nimipressuralis	"Prokaryotes;Bacteria;Gammaproteobacteria - Enterobacteria;Lelliottia"
970	lew	Lelliottia sp. WB101	"Prokaryotes;Bacteria;Gammaproteobacteria - Enterobacteria;Lelliottia"
971	buf	Buttiauxella sp. 3AFRM03	"Prokaryotes;Bacteria;Gammaproteobacteria - Enterobacteria;Buttiauxella"
972	bage	Buttiauxella agrestis	"Prokaryotes;Bacteria;Gammaproteobacteria - Enterobacteria;Buttiauxella"
973	sbw	Candidatus Tachikawaea gelatinosa	"Prokaryotes;Bacteria;Gammaproteobacteria - Enterobacteria;Tachikawaea"
974	den	Candidatus Doolittlea endobia	"Prokaryotes;Bacteria;Gammaproteobacteria - Enterobacteria;Doolittlea"
975	hed	Candidatus Hoaglandella endobia	"Prokaryotes;Bacteria;Gammaproteobacteria - Enterobacteria;Hoaglandella"
976	ged	Candidatus Gullanella endobia	"Prokaryotes;Bacteria;Gammaproteobacteria - Enterobacteria;Gullanella"
977	cmik	Candidatus Mikella endobia	"Prokaryotes;Bacteria;Gammaproteobacteria - Enterobacteria;Mikella"
978	ppet	Candidatus Purcelliella pentastirinorum	"Prokaryotes;Bacteria;Gammaproteobacteria - Enterobacteria;Purcelliella"

979	mety	Metakosakonia sp. MRY16-398	"Prokaryotes;Bacteria;Gammaproteobacteria - Enterobacteria;Metakosakonia"
980	ahn	Atlantibacter hermannii	"Prokaryotes;Bacteria;Gammaproteobacteria - Enterobacteria;Atlantibacter"
981	yre	Yokenella regensburgei	"Prokaryotes;Bacteria;Gammaproteobacteria - Enterobacteria;Yokenella"
982	sgoe	Scandinavium goeteborgense	"Prokaryotes;Bacteria;Gammaproteobacteria - Enterobacteria;Scandinavium"
983	kin	Phytobacter ursingii	"Prokaryotes;Bacteria;Gammaproteobacteria - Enterobacteria;Phytobacter"
984	pdz	Phytobacter diazotrophicus	"Prokaryotes;Bacteria;Gammaproteobacteria - Enterobacteria;Phytobacter"
985	ebf	Enterobacteriaceae bacterium FGI 57	"Prokaryotes;Bacteria;Gammaproteobacteria - Enterobacteria;unclassified Enterobacteriaceae"
986	ebc	Enterobacteriaceae bacterium ENNIH2	"Prokaryotes;Bacteria;Gammaproteobacteria - Enterobacteria;unclassified Enterobacteriaceae"
987	ebu	Enterobacteriaceae bacterium S05	"Prokaryotes;Bacteria;Gammaproteobacteria - Enterobacteria;unclassified Enterobacteriaceae"
988	psts	Plautia stali symbiont	"Prokaryotes;Bacteria;Gammaproteobacteria - Enterobacteria;unclassified Enterobacteriaceae"
989	izh	Jejubacter calystegiae	"Prokaryotes;Bacteria;Gammaproteobacteria - Enterobacteria;Izhakiella"
990	ype	Yersinia pestis CO92 (biovar Orientalis)	"Prokaryotes;Bacteria;Gammaproteobacteria - Enterobacteria;Yersinia"
991	ypk	Yersinia pestis KIM10+ (biovar Mediaevalis)	"Prokaryotes;Bacteria;Gammaproteobacteria - Enterobacteria;Yersinia"
992	yph	Yersinia pestis Harbin 35 (biovar Medievalis)	"Prokaryotes;Bacteria;Gammaproteobacteria - Enterobacteria;Yersinia"
993	ypa	Yersinia pestis Antiqua (biovar Antiqua)	"Prokaryotes;Bacteria;Gammaproteobacteria - Enterobacteria;Yersinia"
994	ypn	Yersinia pestis Nepal516 (biovar Antiqua)	"Prokaryotes;Bacteria;Gammaproteobacteria - Enterobacteria;Yersinia"
995	ypm	Yersinia pestis 91001 (biovar Microtus)	"Prokaryotes;Bacteria;Gammaproteobacteria - Enterobacteria;Yersinia"
996	ypp	Yersinia pestis Pestoides F (biovar Antiqua)	"Prokaryotes;Bacteria;Gammaproteobacteria - Enterobacteria;Yersinia"
997	ypg	Yersinia pestis Angola	"Prokaryotes;Bacteria;Gammaproteobacteria - Enterobacteria;Yersinia"
998	ypz	Yersinia pestis Z176003	"Prokaryotes;Bacteria;Gammaproteobacteria - Enterobacteria;Yersinia"
999	ypt	Yersinia pestis A1122	"Prokaryotes;Bacteria;Gammaproteobacteria - Enterobacteria;Yersinia"
1000	ypd	Yersinia pestis D106004	"Prokaryotes;Bacteria;Gammaproteobacteria - Enterobacteria;Yersinia"
1001	ypx	Yersinia pestis D182038	"Prokaryotes;Bacteria;Gammaproteobacteria - Enterobacteria;Yersinia"
1002	ypw	Yersinia pestis PBM19	"Prokaryotes;Bacteria;Gammaproteobacteria - Enterobacteria;Yersinia"
1003	ypj	Yersinia pestis Harbin35	"Prokaryotes;Bacteria;Gammaproteobacteria - Enterobacteria;Yersinia"
1004	ypv	Yersinia pestis Shasta	"Prokaryotes;Bacteria;Gammaproteobacteria - Enterobacteria;Yersinia"
1005	ypl	Yersinia pestis El Dorado	"Prokaryotes;Bacteria;Gammaproteobacteria - Enterobacteria;Yersinia"
1006	yps	Yersinia pseudotuberculosis IP 32953 (serotype I)	"Prokaryotes;Bacteria;Gammaproteobacteria - Enterobacteria;Yersinia"
1007	ypo	Yersinia pseudotuberculosis IP 32953 (serotype 1)	"Prokaryotes;Bacteria;Gammaproteobacteria - Enterobacteria;Yersinia"
1008	ypi	Yersinia pseudotuberculosis IP 31758 (serotype O:1b)	"Prokaryotes;Bacteria;Gammaproteobacteria - Enterobacteria;Yersinia"
1009	ypy	Yersinia pseudotuberculosis YPIII (serotype O:3)	"Prokaryotes;Bacteria;Gammaproteobacteria - Enterobacteria;Yersinia"
1010	ypb	Yersinia pseudotuberculosis PB1/+ (serotype 1B)	"Prokaryotes;Bacteria;Gammaproteobacteria - Enterobacteria;Yersinia"
1011	ypq	Yersinia pseudotuberculosis ATCC 6904	"Prokaryotes;Bacteria;Gammaproteobacteria - Enterobacteria;Yersinia"
1012	ypu	Yersinia pseudotuberculosis MD67	"Prokaryotes;Bacteria;Gammaproteobacteria - Enterobacteria;Yersinia"
1013	ypr	Yersinia pseudotuberculosis EP2/+	"Prokaryotes;Bacteria;Gammaproteobacteria - Enterobacteria;Yersinia"
1014	ypc	Yersinia pseudotuberculosis 1	"Prokaryotes;Bacteria;Gammaproteobacteria - Enterobacteria;Yersinia"
1015	ypf	Yersinia pseudotuberculosis PA3606	"Prokaryotes;Bacteria;Gammaproteobacteria - Enterobacteria;Yersinia"

1016	yen	Yersinia enterocolitica subsp. enterocolitica 8081 (serotype 0:8)	"Prokaryotes;Bacteria;Gammaproteobacteria - Enterobacteria;Yersinia"
1017	yep	Yersinia enterocolitica subsp. palearctica 105.5R(r) (serotype:O:9)	"Prokaryotes;Bacteria;Gammaproteobacteria - Enterobacteria;Yersinia"
1018	yey	Yersinia enterocolitica subsp. palearctica Y11 (serotype:0:3)	"Prokaryotes;Bacteria;Gammaproteobacteria - Enterobacteria;Yersinia"
1019	yel	Yersinia enterocolitica LC20	"Prokaryotes;Bacteria;Gammaproteobacteria - Enterobacteria;Yersinia"
1020	yew	Yersinia enterocolitica WA	"Prokaryotes;Bacteria;Gammaproteobacteria - Enterobacteria;Yersinia"
1021	yet	Yersinia enterocolitica 2516-87	"Prokaryotes;Bacteria;Gammaproteobacteria - Enterobacteria;Yersinia"
1022	yef	Yersinia enterocolitica FORC_002	"Prokaryotes;Bacteria;Gammaproteobacteria - Enterobacteria;Yersinia"
1023	yee	Yersinia enterocolitica (type O:5) YE53/03	"Prokaryotes;Bacteria;Gammaproteobacteria - Enterobacteria;Yersinia"
1024	ysi	Yersinia similis	"Prokaryotes;Bacteria;Gammaproteobacteria - Enterobacteria;Yersinia"
1025	yal	Yersinia aldovae	"Prokaryotes;Bacteria;Gammaproteobacteria - Enterobacteria;Yersinia"
1026	yfr	Yersinia frederiksenii	"Prokaryotes;Bacteria;Gammaproteobacteria - Enterobacteria;Yersinia"
1027	yin	Yersinia intermedia	"Prokaryotes;Bacteria;Gammaproteobacteria - Enterobacteria;Yersinia"
1028	ykr	Yersinia kristensenii	"Prokaryotes;Bacteria;Gammaproteobacteria - Enterobacteria;Yersinia"
1029	yro	Yersinia rohdei	"Prokaryotes;Bacteria;Gammaproteobacteria - Enterobacteria;Yersinia"
1030	yru	Yersinia ruckeri YRB	"Prokaryotes;Bacteria;Gammaproteobacteria - Enterobacteria;Yersinia"
1031	yrb	Yersinia ruckeri Big Creek 74 (serotype O2)	"Prokaryotes;Bacteria;Gammaproteobacteria - Enterobacteria;Yersinia"
1032	yak	Yersinia aleksiciae	"Prokaryotes;Bacteria;Gammaproteobacteria - Enterobacteria;Yersinia"
1033	yma	Yersinia massiliensis	"Prokaryotes;Bacteria;Gammaproteobacteria - Enterobacteria;Yersinia"
1034	yhi	Yersinia hibernica	"Prokaryotes;Bacteria;Gammaproteobacteria - Enterobacteria;Yersinia"
1035	yca	Yersinia canariae	"Prokaryotes;Bacteria;Gammaproteobacteria - Enterobacteria;Yersinia"
1036	ymo	Yersinia mollaretii	"Prokaryotes;Bacteria;Gammaproteobacteria - Enterobacteria;Yersinia"
1037	smar	Serratia marcescens SM39	"Prokaryotes;Bacteria;Gammaproteobacteria - Enterobacteria;Serratia"
1038	smac	Serratia marcescens subsp. marcescens Db11	"Prokaryotes;Bacteria;Gammaproteobacteria - Enterobacteria;Serratia"
1039	smw	Serratia marcescens WW4	"Prokaryotes;Bacteria;Gammaproteobacteria - Enterobacteria;Serratia"
1040	spe	Serratia proteamaculans	"Prokaryotes;Bacteria;Gammaproteobacteria - Enterobacteria;Serratia"
1041	srr	Serratia plymuthica AS9	"Prokaryotes;Bacteria;Gammaproteobacteria - Enterobacteria;Serratia"
1042	srl	Serratia plymuthica 4Rx13	"Prokaryotes;Bacteria;Gammaproteobacteria - Enterobacteria;Serratia"
1043	sry	Serratia plymuthica S13	"Prokaryotes;Bacteria;Gammaproteobacteria - Enterobacteria;Serratia"
1044	sply	Serratia plymuthica PRI-2C	"Prokaryotes;Bacteria;Gammaproteobacteria - Enterobacteria;Serratia"
1045	srs	Serratia sp. AS12	"Prokaryotes;Bacteria;Gammaproteobacteria - Enterobacteria;Serratia"
1046	sra	Serratia sp. AS13	"Prokaryotes;Bacteria;Gammaproteobacteria - Enterobacteria;Serratia"
1047	ssz	Serratia symbiotica	"Prokaryotes;Bacteria;Gammaproteobacteria - Enterobacteria;Serratia"
1048	smaf	Serratia sp. FGI94	"Prokaryotes;Bacteria;Gammaproteobacteria - Enterobacteria;Serratia"
1049	slq	Serratia liquefaciens	"Prokaryotes;Bacteria;Gammaproteobacteria - Enterobacteria;Serratia"
1050	serf	Serratia sp. FS14	"Prokaryotes;Bacteria;Gammaproteobacteria - Enterobacteria;Serratia"
1051	sers	Serratia sp. SCBI	"Prokaryotes;Bacteria;Gammaproteobacteria - Enterobacteria;Serratia"
1052	sfw	Serratia fonticola DSM 4576	"Prokaryotes;Bacteria;Gammaproteobacteria - Enterobacteria;Serratia"
1053	sfg	Serratia fonticola GS2	"Prokaryotes;Bacteria;Gammaproteobacteria - Enterobacteria;Serratia"
1054	srz	Serratia rubidaea	"Prokaryotes;Bacteria;Gammaproteobacteria - Enterobacteria;Serratia"
1055	sera	Serratia sp. ATCC 39006	"Prokaryotes;Bacteria;Gammaproteobacteria - Enterobacteria;Serratia"
1056	serq	Serratia sp. ATCC 39006	"Prokaryotes;Bacteria;Gammaproteobacteria - Enterobacteria;Serratia"

1057	serm	Serratia sp. MYb239	"Prokaryotes;Bacteria;Gammaproteobacteria - Enterobacteria;Serratia"
1058	squ	Serratia quinivorans	"Prokaryotes;Bacteria;Gammaproteobacteria - Enterobacteria;Serratia"
1059	sfj	Serratia ficaria	"Prokaryotes;Bacteria;Gammaproteobacteria - Enterobacteria;Serratia"
1060	sof	Serratia odorifera	"Prokaryotes;Bacteria;Gammaproteobacteria - Enterobacteria;Serratia"
1061	ssur	Serratia surfactantfaciens	"Prokaryotes;Bacteria;Gammaproteobacteria - Enterobacteria;Serratia"
1062	sfo	Chania multitudinisentens	"Prokaryotes;Bacteria;Gammaproteobacteria - Enterobacteria;Chania"
1063	rah	Rahnella sp. Y9602	"Prokaryotes;Bacteria;Gammaproteobacteria - Enterobacteria;Rahnella"
1064	raq	Rahnella aquatilis CIP 78.65 = ATCC 33071	"Prokaryotes;Bacteria;Gammaproteobacteria - Enterobacteria;Rahnella"
1065	raa	Rahnella aquatilis HX2	"Prokaryotes;Bacteria;Gammaproteobacteria - Enterobacteria;Rahnella"
1066	rox	Rahnella sp. ERMR1:05	"Prokaryotes;Bacteria;Gammaproteobacteria - Enterobacteria;Rahnella"
1067	gqu	Gibbsiella quercinecans	"Prokaryotes;Bacteria;Gammaproteobacteria - Enterobacteria;Gibbsiella"
1068	fsm	Candidatus Fukatsuia symbiotica	"Prokaryotes;Bacteria;Gammaproteobacteria - Enterobacteria;Fukatsuia"
1069	eame	Ewingella americana	"Prokaryotes;Bacteria;Gammaproteobacteria - Enterobacteria;Ewingella"
1070	rbad	Rouxiella badensis	"Prokaryotes;Bacteria;Gammaproteobacteria - Enterobacteria;Rouxiella"
1071	eca	Pectobacterium atrosepticum SCRI1043	"Prokaryotes;Bacteria;Gammaproteobacteria - Enterobacteria;Pectobacterium"
1072	patr	Pectobacterium atrosepticum JG10-08	"Prokaryotes;Bacteria;Gammaproteobacteria - Enterobacteria;Pectobacterium"
1073	pato	Pectobacterium atrosepticum 21A	"Prokaryotes;Bacteria;Gammaproteobacteria - Enterobacteria;Pectobacterium"
1074	pct	Pectobacterium carotovorum subsp. carotovorum PC1	"Prokaryotes;Bacteria;Gammaproteobacteria - Enterobacteria;Pectobacterium"
1075	pcc	Pectobacterium carotovorum subsp. carotovorum PCC21	"Prokaryotes;Bacteria;Gammaproteobacteria - Enterobacteria;Pectobacterium"
1076	pcv	Pectobacterium odoriferum	"Prokaryotes;Bacteria;Gammaproteobacteria - Enterobacteria;Pectobacterium"
1077	pwa	Pectobacterium parmentieri WPP163	"Prokaryotes;Bacteria;Gammaproteobacteria - Enterobacteria;Pectobacterium"
1078	ppar	Pectobacterium parmentieri RNS08.42.1A	"Prokaryotes;Bacteria;Gammaproteobacteria - Enterobacteria;Pectobacterium"
1079	pec	Pectobacterium parmentieri SCC3193	"Prokaryotes;Bacteria;Gammaproteobacteria - Enterobacteria;Pectobacterium"
1080	pws	Pectobacterium wasabiae	"Prokaryotes;Bacteria;Gammaproteobacteria - Enterobacteria;Pectobacterium"
1081	ppoa	Pectobacterium polaris	"Prokaryotes;Bacteria;Gammaproteobacteria - Enterobacteria;Pectobacterium"
1082	pbra	Pectobacterium brasiliense	"Prokaryotes;Bacteria;Gammaproteobacteria - Enterobacteria;Pectobacterium"
1083	ppuj	Pectobacterium punjabense	"Prokaryotes;Bacteria;Gammaproteobacteria - Enterobacteria;Pectobacterium"
1084	ddd	Dickeya dadantii 3937	"Prokaryotes;Bacteria;Gammaproteobacteria - Enterobacteria;Dickeya"
1085	dda	Dickeya paradisiaca	"Prokaryotes;Bacteria;Gammaproteobacteria - Enterobacteria;Dickeya"
1086	dze	Dickeya chrysanthemi	"Prokaryotes;Bacteria;Gammaproteobacteria - Enterobacteria;Dickeya"
1087	ddc	Dickeya zeae Ech586	"Prokaryotes;Bacteria;Gammaproteobacteria - Enterobacteria;Dickeya"
1088	dzc	Dickeya zeae EC1	"Prokaryotes;Bacteria;Gammaproteobacteria - Enterobacteria;Dickeya"
1089	dso	Dickeya solani IPO 2222	"Prokaryotes;Bacteria;Gammaproteobacteria - Enterobacteria;Dickeya"
1090	ced	Dickeya fangzhongdai ND14b	"Prokaryotes;Bacteria;Gammaproteobacteria - Enterobacteria;Dickeya"
1091	dfn	Dickeya fangzhongdai DSM 101947	"Prokaryotes;Bacteria;Gammaproteobacteria - Enterobacteria;Dickeya"
1092	ddq	Dickeya dianthicola	"Prokaryotes;Bacteria;Gammaproteobacteria - Enterobacteria;Dickeya"
1093	daq	Dickeya aquatica	"Prokaryotes;Bacteria;Gammaproteobacteria - Enterobacteria;Dickeya"
1094	dic	Dickeya poaceiphila	"Prokaryotes;Bacteria;Gammaproteobacteria - Enterobacteria;Dickeya"
1095	bgj	Brenneria goodwinii	"Prokaryotes;Bacteria;Gammaproteobacteria - Enterobacteria;Brenneria"
1096	brb	Brenneria rubrifaciens	"Prokaryotes;Bacteria;Gammaproteobacteria - Enterobacteria;Brenneria"
1097	bng	Brenneria nigrifluens	"Prokaryotes;Bacteria;Gammaproteobacteria - Enterobacteria;Brenneria"

1098	lbq	Lonsdalea britannica	"Prokaryotes;Bacteria;Gammaproteobacteria - Enterobacteria;Lonsdalea"
1099	lpop	Lonsdalea populi	"Prokaryotes;Bacteria;Gammaproteobacteria - Enterobacteria;Lonsdalea"
1100	sgl	Sodalis glossinidius	"Prokaryotes;Bacteria;Gammaproteobacteria - Enterobacteria;Sodalis"
1101	sod	Sodalis praecaptivus	"Prokaryotes;Bacteria;Gammaproteobacteria - Enterobacteria;Sodalis"
1102	pes	Candidatus Sodalis pierantonius	"Prokaryotes;Bacteria;Gammaproteobacteria - Enterobacteria;Sodalis"
1103	seny	Sodalis endosymbiont of Henestaris halophilus	"Prokaryotes;Bacteria;Gammaproteobacteria - Enterobacteria;Sodalis"
1104	eam	Erwinia amylovora CFBP1430	"Prokaryotes;Bacteria;Gammaproteobacteria - Enterobacteria;Erwinia"
1105	eay	Erwinia amylovora ATCC 49946	"Prokaryotes;Bacteria;Gammaproteobacteria - Enterobacteria;Erwinia"
1106	eta	Erwinia tasmaniensis	"Prokaryotes;Bacteria;Gammaproteobacteria - Enterobacteria;Erwinia"
1107	epy	Erwinia pyrifoliae Ep1/96	"Prokaryotes;Bacteria;Gammaproteobacteria - Enterobacteria;Erwinia"
1108	epr	Erwinia pyrifoliae DSM 12163	"Prokaryotes;Bacteria;Gammaproteobacteria - Enterobacteria;Erwinia"
1109	ebi	Erwinia billingiae	"Prokaryotes;Bacteria;Gammaproteobacteria - Enterobacteria;Erwinia"
1110	erj	Erwinia sp. Ejp617	"Prokaryotes;Bacteria;Gammaproteobacteria - Enterobacteria;Erwinia"
1111	ege	Erwinia gerundensis	"Prokaryotes;Bacteria;Gammaproteobacteria - Enterobacteria;Erwinia"
1112	epe	Erwinia persicina	"Prokaryotes;Bacteria;Gammaproteobacteria - Enterobacteria;Erwinia"
1113	ehd	Candidatus Erwinia haradaeae	"Prokaryotes;Bacteria;Gammaproteobacteria - Enterobacteria;Erwinia"
1114	erwi	Erwinia sp. J780	"Prokaryotes;Bacteria;Gammaproteobacteria - Enterobacteria;Erwinia"
1115	buc	Buchnera aphidicola APS (Acyrthosiphon pisum)	"Prokaryotes;Bacteria;Gammaproteobacteria - Enterobacteria;Buchnera"
1116	bap	Buchnera aphidicola 5A (Acyrthosiphon pisum)	"Prokaryotes;Bacteria;Gammaproteobacteria - Enterobacteria;Buchnera"
1117	bau	Buchnera aphidicola Tuc7 (Acyrthosiphon pisum)	"Prokaryotes;Bacteria;Gammaproteobacteria - Enterobacteria;Buchnera"
1118	baw	Buchnera aphidicola JF98 (Acyrthosiphon pisum)	"Prokaryotes;Bacteria;Gammaproteobacteria - Enterobacteria;Buchnera"
1119	bajc	Buchnera aphidicola JF99 (Acyrthosiphon pisum)	"Prokaryotes;Bacteria;Gammaproteobacteria - Enterobacteria;Buchnera"
1120	bua	Buchnera aphidicola LL01 (Acyrthosiphon pisum)	"Prokaryotes;Bacteria;Gammaproteobacteria - Enterobacteria;Buchnera"
1121	bup	Buchnera aphidicola TLW03 (Acyrthosiphon pisum)	"Prokaryotes;Bacteria;Gammaproteobacteria - Enterobacteria;Buchnera"
1122	bak	Buchnera aphidicola Ak (Acyrthosiphon kondoi)	"Prokaryotes;Bacteria;Gammaproteobacteria - Enterobacteria;Buchnera"
1123	buh	Buchnera aphidicola Ua (Uroleucon ambrosiae)	"Prokaryotes;Bacteria;Gammaproteobacteria - Enterobacteria;Buchnera"
1124	bapf	Buchnera aphidicola F009 (Myzus persicae)	"Prokaryotes;Bacteria;Gammaproteobacteria - Enterobacteria;Buchnera"
1125	bapg	Buchnera aphidicola G002 (Myzus persicae)	"Prokaryotes;Bacteria;Gammaproteobacteria - Enterobacteria;Buchnera"
1126	bapu	Buchnera aphidicola USDA (Myzus persicae)	"Prokaryotes;Bacteria;Gammaproteobacteria - Enterobacteria;Buchnera"
1127	bapw	Buchnera aphidicola W106 (Myzus persicae)	"Prokaryotes;Bacteria;Gammaproteobacteria - Enterobacteria;Buchnera"
1128	bas	Buchnera aphidicola Sg (Schizaphis graminum)	"Prokaryotes;Bacteria;Gammaproteobacteria - Enterobacteria;Buchnera"
1129	bab	Buchnera aphidicola Bp (Baizongia pistaciae)	"Prokaryotes;Bacteria;Gammaproteobacteria - Enterobacteria;Buchnera"
1130	bcc	Buchnera aphidicola BCc	"Prokaryotes;Bacteria;Gammaproteobacteria - Enterobacteria;Buchnera"
1131	baj	Buchnera aphidicola (Cinara tujafilina)	"Prokaryotes;Bacteria;Gammaproteobacteria - Enterobacteria;Buchnera"
1132	baph	Buchnera aphidicola (Aphis glycines)	"Prokaryotes;Bacteria;Gammaproteobacteria - Enterobacteria;Buchnera"
1133	wbr	Wigglesworthia glossinidia endosymbiont of Glossina brevipalpis	"Prokaryotes;Bacteria;Gammaproteobacteria - Enterobacteria;Wigglesworthia"
1134	wgl	Wigglesworthia glossinidia endosymbiont of Glossina morsitans morsitan	"Prokaryotes;Bacteria;Gammaproteobacteria - Enterobacteria;Wigglesworthia"
1135	pam	Pantoea ananatis LMG 20103	"Prokaryotes;Bacteria;Gammaproteobacteria - Enterobacteria;Pantoea"
1136	plf	Pantoea ananatis LMG 5342	"Prokaryotes;Bacteria;Gammaproteobacteria - Enterobacteria;Pantoea"
1137	paj	Pantoea ananatis AJ13355	"Prokaryotes;Bacteria;Gammaproteobacteria - Enterobacteria;Pantoea"

1138	paq	Pantoea ananatis PA13	"Prokaryotes;Bacteria;Gammaproteobacteria - Enterobacteria;Pantoea"
1139	pva	Pantoea vagans C9-1	"Prokaryotes;Bacteria;Gammaproteobacteria - Enterobacteria;Pantoea"
1140	pagg	Pantoea vagans FDAARGOS_160	"Prokaryotes;Bacteria;Gammaproteobacteria - Enterobacteria;Pantoea"
1141	pao	Pantoea sp. At-9b	"Prokaryotes;Bacteria;Gammaproteobacteria - Enterobacteria;Pantoea"
1142	kln	Pantoea rwandensis	"Prokaryotes;Bacteria;Gammaproteobacteria - Enterobacteria;Pantoea"
1143	pant	Pantoea sp. PSNIH1	"Prokaryotes;Bacteria;Gammaproteobacteria - Enterobacteria;Pantoea"
1144	panp	Pantoea sp. PSNIH2	"Prokaryotes;Bacteria;Gammaproteobacteria - Enterobacteria;Pantoea"
1145	hhs	Candidatus Pantoea carbekii	"Prokaryotes;Bacteria;Gammaproteobacteria - Enterobacteria;Pantoea"
1146	pck	Candidatus Pantoea carbekii US	"Prokaryotes;Bacteria;Gammaproteobacteria - Enterobacteria;Pantoea"
1147	pagc	Pantoea agglomerans C410P1	"Prokaryotes;Bacteria;Gammaproteobacteria - Enterobacteria;Pantoea"
1148	pstw	Pantoea stewartii subsp. stewartii	"Prokaryotes;Bacteria;Gammaproteobacteria - Enterobacteria;Pantoea"
1149	palh	Pantoea alhagi	"Prokaryotes;Bacteria;Gammaproteobacteria - Enterobacteria;Pantoea"
1150	pans	Pantoea sp. SO10	"Prokaryotes;Bacteria;Gammaproteobacteria - Enterobacteria;Pantoea"
1151	pey	Pantoea eucalypti	"Prokaryotes;Bacteria;Gammaproteobacteria - Enterobacteria;Pantoea"
1152	pdis	Pantoea dispersa	"Prokaryotes;Bacteria;Gammaproteobacteria - Enterobacteria;Pantoea"
1153	pgz	Mixta gaviniae	"Prokaryotes;Bacteria;Gammaproteobacteria - Enterobacteria;Mixta"
1154	pcd	Mixta calida	"Prokaryotes;Bacteria;Gammaproteobacteria - Enterobacteria;Mixta"
1155	mint	Mixta intestinalis	"Prokaryotes;Bacteria;Gammaproteobacteria - Enterobacteria;Mixta"
1156	mthi	Mixta theicola	"Prokaryotes;Bacteria;Gammaproteobacteria - Enterobacteria;Mixta"
1157	tci	Tatumella citrea	"Prokaryotes;Bacteria;Gammaproteobacteria - Enterobacteria;Tatumella"
1158	tpty	Tatumella ptyseos	"Prokaryotes;Bacteria;Gammaproteobacteria - Enterobacteria;Tatumella"
1159	plu	Photorhabdus laumondii subsp. laumondii TTO1	"Prokaryotes;Bacteria;Gammaproteobacteria - Enterobacteria;Photorhabdus"
1160	plum	Photorhabdus laumondii subsp. laumondii DSPV002N	"Prokaryotes;Bacteria;Gammaproteobacteria - Enterobacteria;Photorhabdus"
1161	pay	Photorhabdus asymbiotica	"Prokaryotes;Bacteria;Gammaproteobacteria - Enterobacteria;Photorhabdus"
1162	ptt	Photorhabdus thracensis	"Prokaryotes;Bacteria;Gammaproteobacteria - Enterobacteria;Photorhabdus"
1163	pmr	Proteus mirabilis HI4320	"Prokaryotes;Bacteria;Gammaproteobacteria - Enterobacteria;Proteus"
1164	pmib	Proteus mirabilis BB2000	"Prokaryotes;Bacteria;Gammaproteobacteria - Enterobacteria;Proteus"
1165	pvl	Proteus mirabilis CYPV1	"Prokaryotes;Bacteria;Gammaproteobacteria - Enterobacteria;Proteus"
1166	pvg	Proteus vulgaris	"Prokaryotes;Bacteria;Gammaproteobacteria - Enterobacteria;Proteus"
1167	phau	Proteus hauseri	"Prokaryotes;Bacteria;Gammaproteobacteria - Enterobacteria;Proteus"
1168	prot	Proteus sp. CD3	"Prokaryotes;Bacteria;Gammaproteobacteria - Enterobacteria;Proteus"
1169	pcol	Proteus columbae	"Prokaryotes;Bacteria;Gammaproteobacteria - Enterobacteria;Proteus"
1170	pcib	Proteus terrae subsp. cibarius	"Prokaryotes;Bacteria;Gammaproteobacteria - Enterobacteria;Proteus terrae"
1171	xbo	Xenorhabdus bovienii SS-2004	"Prokaryotes;Bacteria;Gammaproteobacteria - Enterobacteria;Xenorhabdus"
1172	xbv	Xenorhabdus bovienii CS03	"Prokaryotes;Bacteria;Gammaproteobacteria - Enterobacteria;Xenorhabdus"
1173	xne	Xenorhabdus nematophila ATCC 19061	"Prokaryotes;Bacteria;Gammaproteobacteria - Enterobacteria;Xenorhabdus"
1174	xnm	Xenorhabdus nematophila AN6/1	"Prokaryotes;Bacteria;Gammaproteobacteria - Enterobacteria;Xenorhabdus"
1175	xdo	Xenorhabdus doucetiae	"Prokaryotes;Bacteria;Gammaproteobacteria - Enterobacteria;Xenorhabdus"
1176	xpo	Xenorhabdus poinarii	"Prokaryotes;Bacteria;Gammaproteobacteria - Enterobacteria;Xenorhabdus"
1177	xho	Xenorhabdus hominickii	"Prokaryotes;Bacteria;Gammaproteobacteria - Enterobacteria;Xenorhabdus"
1178	psi	Providencia stuartii MRSN 2154	"Prokaryotes;Bacteria;Gammaproteobacteria - Enterobacteria;Providencia"

1179	psx	Providencia stuartii ATCC 33672	"Prokaryotes;Bacteria;Gammaproteobacteria - Enterobacteria;Providencia"
1180	psta	Providencia stuartii BE2467	"Prokaryotes;Bacteria;Gammaproteobacteria - Enterobacteria;Providencia"
1181	prg	Providencia rettgeri	"Prokaryotes;Bacteria;Gammaproteobacteria - Enterobacteria;Providencia"
1182	pala	Providencia alcalifaciens	"Prokaryotes;Bacteria;Gammaproteobacteria - Enterobacteria;Providencia"
1183	phei	Providencia heimbachae	"Prokaryotes;Bacteria;Gammaproteobacteria - Enterobacteria;Providencia"
1184	prq	Providencia huaxiensis	"Prokaryotes;Bacteria;Gammaproteobacteria - Enterobacteria;Providencia"
1185	prj	Providencia rustigianii	"Prokaryotes;Bacteria;Gammaproteobacteria - Enterobacteria;Providencia"
1186	pvc	Providencia vermicola	"Prokaryotes;Bacteria;Gammaproteobacteria - Enterobacteria;Providencia"
1187	mmk	Morganella morganii	"Prokaryotes;Bacteria;Gammaproteobacteria - Enterobacteria;Morganella"
1188	asy	Candidatus Arsenophonus lipoptenae	"Prokaryotes;Bacteria;Gammaproteobacteria - Enterobacteria;Arsenophonus"
1189	aen	Arsenophonus endosymbiont of Aleurodicus dispersus	"Prokaryotes;Bacteria;Gammaproteobacteria - Enterobacteria;Arsenophonus"
1190	ans	Arsenophonus nasoniae	"Prokaryotes;Bacteria;Gammaproteobacteria - Enterobacteria;Arsenophonus"
1191	eic	Edwardsiella ictaluri	"Prokaryotes;Bacteria;Gammaproteobacteria - Enterobacteria;Edwardsiella"
1192	etr	Edwardsiella tarda EIB202	"Prokaryotes;Bacteria;Gammaproteobacteria - Enterobacteria;Edwardsiella"
1193	etd	Edwardsiella tarda FL6-60	"Prokaryotes;Bacteria;Gammaproteobacteria - Enterobacteria;Edwardsiella"
1194	ete	Edwardsiella anguillarum	"Prokaryotes;Bacteria;Gammaproteobacteria - Enterobacteria;Edwardsiella"
1195	etc	Edwardsiella piscicida C07-087	"Prokaryotes;Bacteria;Gammaproteobacteria - Enterobacteria;Edwardsiella"
1196	edw	Edwardsiella sp. EA181011	"Prokaryotes;Bacteria;Gammaproteobacteria - Enterobacteria;Edwardsiella"
1197	edl	Edwardsiella sp. LADL05-105	"Prokaryotes;Bacteria;Gammaproteobacteria - Enterobacteria;Edwardsiella"
1198	eho	Edwardsiella hoshinae	"Prokaryotes;Bacteria;Gammaproteobacteria - Enterobacteria;Edwardsiella"
1199	hav	Hafnia alvei	"Prokaryotes;Bacteria;Gammaproteobacteria - Enterobacteria;Hafnia"
1200	hpar	Hafnia paralvei	"Prokaryotes;Bacteria;Gammaproteobacteria - Enterobacteria;Hafnia"
1201	opo	Obesumbacterium proteus	"Prokaryotes;Bacteria;Gammaproteobacteria - Enterobacteria;Obesumbacterium"
1202	lpv	Limnobaculum parvum	"Prokaryotes;Bacteria;Gammaproteobacteria - Enterobacteria;Limnobaculum"
1203	pfq	Pragia fontium	"Prokaryotes;Bacteria;Gammaproteobacteria - Enterobacteria;Pragia"
1204	prag	Jinshanibacter zhutongyuii	"Prokaryotes;Bacteria;Gammaproteobacteria - Enterobacteria;Jinshanibacter"
1205	lri	Leminorella richardii	"Prokaryotes;Bacteria;Gammaproteobacteria - Enterobacteria;Leminorella"
1206	pshi	Plesiomonas shigelloides	"Prokaryotes;Bacteria;Gammaproteobacteria - Enterobacteria;Plesiomonas"
1207	hin	Haemophilus influenzae Rd KW20 (serotype d)	"Prokaryotes;Bacteria;Gammaproteobacteria - Others;Haemophilus"
1208	hit	Haemophilus influenzae 86-028NP (nontypeable)	"Prokaryotes;Bacteria;Gammaproteobacteria - Others;Haemophilus"
1209	hip	Haemophilus influenzae PittEE (nontypeable)	"Prokaryotes;Bacteria;Gammaproteobacteria - Others;Haemophilus"
1210	hiq	Haemophilus influenzae PittGG (nontypeable)	"Prokaryotes;Bacteria;Gammaproteobacteria - Others;Haemophilus"
1211	hif	Haemophilus influenzae F3031 (nontypeable)	"Prokaryotes;Bacteria;Gammaproteobacteria - Others;Haemophilus"
1212	hil	Haemophilus influenzae F3047 (nontypeable)	"Prokaryotes;Bacteria;Gammaproteobacteria - Others;Haemophilus"
1213	hiu	Haemophilus influenzae 10810 (serotype b)	"Prokaryotes;Bacteria;Gammaproteobacteria - Others;Haemophilus"
1214	hie	Haemophilus influenzae R2846 (nontypeable)	"Prokaryotes;Bacteria;Gammaproteobacteria - Others;Haemophilus"
1215	hiz	Haemophilus influenzae R2866 (nontypeable)	"Prokaryotes;Bacteria;Gammaproteobacteria - Others;Haemophilus"
1216	hik	Haemophilus influenzae KR494 (serotype f)	"Prokaryotes;Bacteria;Gammaproteobacteria - Others;Haemophilus"
1217	hia	Haemophilus influenzae CGSHiCZ412602	"Prokaryotes;Bacteria;Gammaproteobacteria - Others;Haemophilus"
1218	hih	Haemophilus influenzae Hi375	"Prokaryotes;Bacteria;Gammaproteobacteria - Others;Haemophilus"
1219	hiw	Haemophilus influenzae 477	"Prokaryotes;Bacteria;Gammaproteobacteria - Others;Haemophilus"

1220	hic	Haemophilus influenzae C486	"Prokaryotes;Bacteria;Gammaproteobacteria - Others;Haemophilus"
1221	hix	Haemophilus influenzae 723	"Prokaryotes;Bacteria;Gammaproteobacteria - Others;Haemophilus"
1222	hpr	Haemophilus parainfluenzae	"Prokaryotes;Bacteria;Gammaproteobacteria - Others;Haemophilus"
1223	hdu	Haemophilus ducreyi	"Prokaryotes;Bacteria;Gammaproteobacteria - Others;Haemophilus"
1224	hay	Haemophilus sp. oral taxon 036	"Prokaryotes;Bacteria;Gammaproteobacteria - Others;Haemophilus"
1225	hpit	Haemophilus pittmaniae	"Prokaryotes;Bacteria;Gammaproteobacteria - Others;Haemophilus"
1226	hhz	Haemophilus haemolyticus	"Prokaryotes;Bacteria;Gammaproteobacteria - Others;Haemophilus"
1227	haeg	Haemophilus aegyptius	"Prokaryotes;Bacteria;Gammaproteobacteria - Others;Haemophilus"
1228	hpaa	Haemophilus parahaemolyticus	"Prokaryotes;Bacteria;Gammaproteobacteria - Others;Haemophilus"
1229	hap	Glaesserella parasuis SH0165	"Prokaryotes;Bacteria;Gammaproteobacteria - Others;Glaesserella"
1230	hpaz	Glaesserella parasuis ZJ0906	"Prokaryotes;Bacteria;Gammaproteobacteria - Others;Glaesserella"
1231	hpas	Glaesserella parasuis SH03	"Prokaryotes;Bacteria;Gammaproteobacteria - Others;Glaesserella"
1232	hpak	Glaesserella parasuis KL0318	"Prokaryotes;Bacteria;Gammaproteobacteria - Others;Glaesserella"
1233	gle	Glaesserella sp. 15-184	"Prokaryotes;Bacteria;Gammaproteobacteria - Others;Glaesserella"
1234	hso	Histophilus somni 129PT	"Prokaryotes;Bacteria;Gammaproteobacteria - Others;Histophilus"
1235	hsm	Histophilus somni 2336	"Prokaryotes;Bacteria;Gammaproteobacteria - Others;Histophilus"
1236	pmu	Pasteurella multocida subsp. multocida Pm70	"Prokaryotes;Bacteria;Gammaproteobacteria - Others;Pasteurella"
1237	pmv	Pasteurella multocida subsp. multocida HN06	"Prokaryotes;Bacteria;Gammaproteobacteria - Others;Pasteurella"
1238	pul	Pasteurella multocida subsp. multocida 3480	"Prokaryotes;Bacteria;Gammaproteobacteria - Others;Pasteurella"
1239	pmp	Pasteurella multocida 36950	"Prokaryotes;Bacteria;Gammaproteobacteria - Others;Pasteurella"
1240	pmul	Pasteurella multocida ATCC 43137	"Prokaryotes;Bacteria;Gammaproteobacteria - Others;Pasteurella"
1241	pdag	Pasteurella dagmatis	"Prokaryotes;Bacteria;Gammaproteobacteria - Others;Pasteurella"
1242	psky	Pasteurella skyensis	"Prokaryotes;Bacteria;Gammaproteobacteria - Others;Pasteurella"
1243	msu	Mannheimia succiniciproducens	"Prokaryotes;Bacteria;Gammaproteobacteria - Others;Basfia"
1244	bsun	Basfia succiniciproducens	"Prokaryotes;Bacteria;Gammaproteobacteria - Others;Basfia"
1245	mht	Mannheimia haemolytica USDA-ARS-USMARC-185	"Prokaryotes;Bacteria;Gammaproteobacteria - Others;Mannheimia"
1246	mhq	Mannheimia haemolytica USDA-ARS-USMARC-183	"Prokaryotes;Bacteria;Gammaproteobacteria - Others;Mannheimia"
1247	mhat	Mannheimia haemolytica USDA-ARS-USMARC-184	"Prokaryotes;Bacteria;Gammaproteobacteria - Others;Mannheimia"
1248	mhx	Mannheimia haemolytica M42548	"Prokaryotes;Bacteria;Gammaproteobacteria - Others;Mannheimia"
1249	mhae	Mannheimia haemolytica D153	"Prokaryotes;Bacteria;Gammaproteobacteria - Others;Mannheimia"
1250	mham	Mannheimia haemolytica D171	"Prokaryotes;Bacteria;Gammaproteobacteria - Others;Mannheimia"
1251	mhao	Mannheimia haemolytica D174	"Prokaryotes;Bacteria;Gammaproteobacteria - Others;Mannheimia"
1252	mhal	Mannheimia haemolytica USMARC_2286	"Prokaryotes;Bacteria;Gammaproteobacteria - Others;Mannheimia"
1253	mhaq	Mannheimia haemolytica 89010807N	"Prokaryotes;Bacteria;Gammaproteobacteria - Others;Mannheimia"
1254	mhay	Mannheimia haemolytica 89010807N lktA-	"Prokaryotes;Bacteria;Gammaproteobacteria - Others;Mannheimia"
1255	mvr	Mannheimia sp. USDA-ARS-USMARC-1261	"Prokaryotes;Bacteria;Gammaproteobacteria - Others;Mannheimia"
1256	mvi	Mannheimia varigena USDA-ARS-USMARC-1296	"Prokaryotes;Bacteria;Gammaproteobacteria - Others;Mannheimia"
1257	mvg	Mannheimia varigena USDA-ARS-USMARC-1312	"Prokaryotes;Bacteria;Gammaproteobacteria - Others;Mannheimia"
1258	mve	Mannheimia varigena USDA-ARS-USMARC-1388	"Prokaryotes;Bacteria;Gammaproteobacteria - Others;Mannheimia"
1259	mann	Mannheimia ovis	"Prokaryotes;Bacteria;Gammaproteobacteria - Others;Mannheimia"
1260	mgra	Mannheimia granulomatis	"Prokaryotes;Bacteria;Gammaproteobacteria - Others;Mannheimia"

1261	apl	Actinobacillus pleuropneumoniae L20 (serotype 5b)	"Prokaryotes;Bacteria;Gammaproteobacteria - Others;Actinobacillus"
1262	apj	Actinobacillus pleuropneumoniae JL03 (serotype 3)	"Prokaryotes;Bacteria;Gammaproteobacteria - Others;Actinobacillus"
1263	apa	Actinobacillus pleuropneumoniae AP76 (serotype 7)	"Prokaryotes;Bacteria;Gammaproteobacteria - Others;Actinobacillus"
1264	asu	Actinobacillus succinogenes	"Prokaryotes;Bacteria;Gammaproteobacteria - Others;Actinobacillus"
1265	asi	Actinobacillus suis H91-0380	"Prokaryotes;Bacteria;Gammaproteobacteria - Others;Actinobacillus"
1266	ass	Actinobacillus suis ATCC 33415	"Prokaryotes;Bacteria;Gammaproteobacteria - Others;Actinobacillus"
1267	aeu	Actinobacillus equuli subsp. equuli	"Prokaryotes;Bacteria;Gammaproteobacteria - Others;Actinobacillus"
1268	apor	Actinobacillus porcitonsillarum	"Prokaryotes;Bacteria;Gammaproteobacteria - Others;Actinobacillus"
1269	aio	Actinobacillus indolicus	"Prokaryotes;Bacteria;Gammaproteobacteria - Others;Actinobacillus"
1270	adp	Actinobacillus delphinicola	"Prokaryotes;Bacteria;Gammaproteobacteria - Others;Actinobacillus"
1271	alig	Actinobacillus lignieresii	"Prokaryotes;Bacteria;Gammaproteobacteria - Others;Actinobacillus"
1272	aap	Aggregatibacter aphrophilus NJ8700	"Prokaryotes;Bacteria;Gammaproteobacteria - Others;Aggregatibacter"
1273	aaz	Aggregatibacter aphrophilus W10433	"Prokaryotes;Bacteria;Gammaproteobacteria - Others;Aggregatibacter"
1274	aat	Aggregatibacter actinomycetemcomitans D11S-1	"Prokaryotes;Bacteria;Gammaproteobacteria - Others;Aggregatibacter"
1275	aao	Aggregatibacter actinomycetemcomitans ANH9381	"Prokaryotes;Bacteria;Gammaproteobacteria - Others;Aggregatibacter"
1276	aan	Aggregatibacter actinomycetemcomitans D7S-1	"Prokaryotes;Bacteria;Gammaproteobacteria - Others;Aggregatibacter"
1277	aah	Aggregatibacter actinomycetemcomitans HK1651	"Prokaryotes;Bacteria;Gammaproteobacteria - Others;Aggregatibacter"
1278	aacn	Aggregatibacter actinomycetemcomitans NUM4039	"Prokaryotes;Bacteria;Gammaproteobacteria - Others;Aggregatibacter"
1279	aact	Aggregatibacter actinomycetemcomitans 624	"Prokaryotes;Bacteria;Gammaproteobacteria - Others;Aggregatibacter"
1280	aseg	Aggregatibacter segnis	"Prokaryotes;Bacteria;Gammaproteobacteria - Others;Aggregatibacter"
1281	gan	Gallibacterium anatis	"Prokaryotes;Bacteria;Gammaproteobacteria - Others;Gallibacterium"
1282	bto	Bibersteinia trehalosi USDA-ARS-USMARC-192	"Prokaryotes;Bacteria;Gammaproteobacteria - Others;Bibersteinia"
1283	btre	Bibersteinia trehalosi USDA-ARS-USMARC-188	"Prokaryotes;Bacteria;Gammaproteobacteria - Others;Bibersteinia"
1284	btrh	Bibersteinia trehalosi USDA-ARS-USMARC-189	"Prokaryotes;Bacteria;Gammaproteobacteria - Others;Bibersteinia"
1285	btra	Bibersteinia trehalosi USDA-ARS-USMARC-190	"Prokaryotes;Bacteria;Gammaproteobacteria - Others;Bibersteinia"
1286	apag	Avibacterium paragallinarum	"Prokaryotes;Bacteria;Gammaproteobacteria - Others;Avibacterium"
1287	avt	Avibacterium volantium	"Prokaryotes;Bacteria;Gammaproteobacteria - Others;Avibacterium"
1288	rpne	Rodentibacter pneumotropicus	"Prokaryotes;Bacteria;Gammaproteobacteria - Others;Rodentibacter"
1289	rhey	Rodentibacter heylii	"Prokaryotes;Bacteria;Gammaproteobacteria - Others;Rodentibacter"
1290	ooi	Otariodibacter oris	"Prokaryotes;Bacteria;Gammaproteobacteria - Others;Otariodibacter"
1291	fcl	Frederiksenia canicola	"Prokaryotes;Bacteria;Gammaproteobacteria - Others;Frederiksenia"
1292	bhud	Bisgaardia hudsonensis	"Prokaryotes;Bacteria;Gammaproteobacteria - Others;Bisgaardia"
1293	paet	Pasteurella aerogenes	"Prokaryotes;Bacteria;Gammaproteobacteria - Others;unclassified Pasteurellaceae"
1294	xfa	Xylella fastidiosa 9a5c	"Prokaryotes;Bacteria;Gammaproteobacteria - Others;Xylella"
1295	xft	Xylella fastidiosa Temecula1	"Prokaryotes;Bacteria;Gammaproteobacteria - Others;Xylella"
1296	xfm	Xylella fastidiosa M12	"Prokaryotes;Bacteria;Gammaproteobacteria - Others;Xylella"
1297	xfn	Xylella fastidiosa M23	"Prokaryotes;Bacteria;Gammaproteobacteria - Others;Xylella"
1298	xff	Xylella fastidiosa subsp. fastidiosa GB514	"Prokaryotes;Bacteria;Gammaproteobacteria - Others;Xylella"
1299	xfl	Xylella fastidiosa MUL0034	"Prokaryotes;Bacteria;Gammaproteobacteria - Others;Xylella"
1300	xfs	Xylella fastidiosa subsp. sandyi Ann-1	"Prokaryotes;Bacteria;Gammaproteobacteria - Others;Xylella"
1301	xfh	Xylella fastidiosa Hib4	"Prokaryotes;Bacteria;Gammaproteobacteria - Others;Xylella"

1302	xtw	Xylella taiwanensis	"Prokaryotes;Bacteria;Gammaproteobacteria - Others;Xylella"
1303	xcc	Xanthomonas campestris pv. campestris ATCC 33913	"Prokaryotes;Bacteria;Gammaproteobacteria - Others;Xanthomonas"
1304	xcb	Xanthomonas campestris pv. campestris 8004	"Prokaryotes;Bacteria;Gammaproteobacteria - Others;Xanthomonas"
1305	xca	Xanthomonas campestris pv. campestris B100	"Prokaryotes;Bacteria;Gammaproteobacteria - Others;Xanthomonas"
1306	xcp	Xanthomonas campestris pv. raphani	"Prokaryotes;Bacteria;Gammaproteobacteria - Others;Xanthomonas"
1307	xcv	Xanthomonas campestris pv. vesicatoria	"Prokaryotes;Bacteria;Gammaproteobacteria - Others;Xanthomonas"
1308	xax	Xanthomonas axonopodis pv. citrumelo F1	"Prokaryotes;Bacteria;Gammaproteobacteria - Others;Xanthomonas"
1309	xac	Xanthomonas citri pv. citri 306	"Prokaryotes;Bacteria;Gammaproteobacteria - Others;Xanthomonas"
1310	xci	Xanthomonas citri subsp. citri Aw12879	"Prokaryotes;Bacteria;Gammaproteobacteria - Others;Xanthomonas"
1311	xct	Xanthomonas citri subsp. citri A306	"Prokaryotes;Bacteria;Gammaproteobacteria - Others;Xanthomonas"
1312	xcj	Xanthomonas citri subsp. citri UI6	"Prokaryotes;Bacteria;Gammaproteobacteria - Others;Xanthomonas"
1313	xcu	Xanthomonas citri pv. citri UI7	"Prokaryotes;Bacteria;Gammaproteobacteria - Others;Xanthomonas"
1314	xcn	Xanthomonas citri pv. citri NT17	"Prokaryotes;Bacteria;Gammaproteobacteria - Others;Xanthomonas"
1315	xcw	Xanthomonas citri pv. citri MN10	"Prokaryotes;Bacteria;Gammaproteobacteria - Others;Xanthomonas"
1316	xcr	Xanthomonas citri pv. citri MN11	"Prokaryotes;Bacteria;Gammaproteobacteria - Others;Xanthomonas"
1317	xcm	Xanthomonas citri pv. citri MN12	"Prokaryotes;Bacteria;Gammaproteobacteria - Others;Xanthomonas"
1318	xcf	Xanthomonas citri pv. citri mf20	"Prokaryotes;Bacteria;Gammaproteobacteria - Others;Xanthomonas"
1319	xfu	Xanthomonas citri pv. fuscans	"Prokaryotes;Bacteria;Gammaproteobacteria - Others;Xanthomonas"
1320	xao	Xanthomonas axonopodis Xac29-1	"Prokaryotes;Bacteria;Gammaproteobacteria - Others;Xanthomonas"
1321	xom	Xanthomonas oryzae pv. oryzae MAFF 311018	"Prokaryotes;Bacteria;Gammaproteobacteria - Others;Xanthomonas"
1322	xoo	Xanthomonas oryzae pv. oryzae KACC 10331	"Prokaryotes;Bacteria;Gammaproteobacteria - Others;Xanthomonas"
1323	xop	Xanthomonas oryzae pv. oryzae PXO99A	"Prokaryotes;Bacteria;Gammaproteobacteria - Others;Xanthomonas"
1324	xoy	Xanthomonas oryzae pv. oryzae PXO86	"Prokaryotes;Bacteria;Gammaproteobacteria - Others;Xanthomonas"
1325	xor	Xanthomonas oryzae pv. oryzicola BLS256	"Prokaryotes;Bacteria;Gammaproteobacteria - Others;Xanthomonas"
1326	xoz	Xanthomonas oryzae pv. oryzicola CFBP7342	"Prokaryotes;Bacteria;Gammaproteobacteria - Others;Xanthomonas"
1327	xal	Xanthomonas albilineans	"Prokaryotes;Bacteria;Gammaproteobacteria - Others;Xanthomonas"
1328	xsa	Xanthomonas sacchari	"Prokaryotes;Bacteria;Gammaproteobacteria - Others;Xanthomonas"
1329	xtn	Xanthomonas translucens	"Prokaryotes;Bacteria;Gammaproteobacteria - Others;Xanthomonas"
1330	xfr	Xanthomonas fragariae	"Prokaryotes;Bacteria;Gammaproteobacteria - Others;Xanthomonas"
1331	xve	Xanthomonas vesicatoria	"Prokaryotes;Bacteria;Gammaproteobacteria - Others;Xanthomonas"
1332	xpe	Xanthomonas perforans	"Prokaryotes;Bacteria;Gammaproteobacteria - Others;Xanthomonas"
1333	xhr	Xanthomonas hortorum	"Prokaryotes;Bacteria;Gammaproteobacteria - Others;Xanthomonas"
1334	xga	Xanthomonas hortorum pv. gardneri	"Prokaryotes;Bacteria;Gammaproteobacteria - Others;Xanthomonas"
1335	xph	Xanthomonas phaseoli	"Prokaryotes;Bacteria;Gammaproteobacteria - Others;Xanthomonas"
1336	xva	Xanthomonas vasicola	"Prokaryotes;Bacteria;Gammaproteobacteria - Others;Xanthomonas"
1337	xan	Xanthomonas sp. ISO98C4	"Prokaryotes;Bacteria;Gammaproteobacteria - Others;Xanthomonas"
1338	xar	Xanthomonas arboricola	"Prokaryotes;Bacteria;Gammaproteobacteria - Others;Xanthomonas"
1339	xhy	Xanthomonas hyacinthi	"Prokaryotes;Bacteria;Gammaproteobacteria - Others;Xanthomonas"
1340	xcz	Xanthomonas cucurbitae	"Prokaryotes;Bacteria;Gammaproteobacteria - Others;Xanthomonas"
1341	xth	Xanthomonas theicola	"Prokaryotes;Bacteria;Gammaproteobacteria - Others;Xanthomonas"
1342	sml	Stenotrophomonas maltophilia K279a	"Prokaryotes;Bacteria;Gammaproteobacteria - Others;Stenotrophomonas"

1343	smt	Stenotrophomonas maltophilia R551-3	"Prokaryotes;Bacteria;Gammaproteobacteria - Others;Stenotrophomonas"
1344	buj	Stenotrophomonas maltophilia JV3	"Prokaryotes;Bacteria;Gammaproteobacteria - Others;Stenotrophomonas"
1345	smz	Stenotrophomonas maltophilia D457	"Prokaryotes;Bacteria;Gammaproteobacteria - Others;Stenotrophomonas"
1346	sacz	Stenotrophomonas acidaminiphila	"Prokaryotes;Bacteria;Gammaproteobacteria - Others;Stenotrophomonas"
1347	stek	Stenotrophomonas sp. KCTC 12332	"Prokaryotes;Bacteria;Gammaproteobacteria - Others;Stenotrophomonas"
1348	srh	Stenotrophomonas rhizophila	"Prokaryotes;Bacteria;Gammaproteobacteria - Others;Stenotrophomonas"
1349	slm	Stenotrophomonas sp. LM091	"Prokaryotes;Bacteria;Gammaproteobacteria - Others;Stenotrophomonas"
1350	sten	Stenotrophomonas sp. WZN-1	"Prokaryotes;Bacteria;Gammaproteobacteria - Others;Stenotrophomonas"
1351	stem	Stenotrophomonas sp. MYb57	"Prokaryotes;Bacteria;Gammaproteobacteria - Others;Stenotrophomonas"
1352	stes	Stenotrophomonas sp. ASS1	"Prokaryotes;Bacteria;Gammaproteobacteria - Others;Stenotrophomonas"
1353	psu	Pseudoxanthomonas suwonensis 11-1	"Prokaryotes;Bacteria;Gammaproteobacteria - Others;Pseudoxanthomonas"
1354	psuw	Pseudoxanthomonas suwonensis J1	"Prokaryotes;Bacteria;Gammaproteobacteria - Others;Pseudoxanthomonas"
1355	psd	Pseudoxanthomonas spadix	"Prokaryotes;Bacteria;Gammaproteobacteria - Others;Pseudoxanthomonas"
1356	pmex	Pseudoxanthomonas mexicana	"Prokaryotes;Bacteria;Gammaproteobacteria - Others;Pseudoxanthomonas"
1357	lab	Lysobacter antibioticus 76	"Prokaryotes;Bacteria;Gammaproteobacteria - Others;Lysobacter"
1358	laq	Lysobacter antibioticus ATCC 29479	"Prokaryotes;Bacteria;Gammaproteobacteria - Others;Lysobacter"
1359	lcp	Lysobacter capsici	"Prokaryotes;Bacteria;Gammaproteobacteria - Others;Lysobacter"
1360	lgu	Lysobacter gummosus	"Prokaryotes;Bacteria;Gammaproteobacteria - Others;Lysobacter"
1361	lez	Lysobacter enzymogenes C3	"Prokaryotes;Bacteria;Gammaproteobacteria - Others;Lysobacter"
1362	lem	Lysobacter enzymogenes M497-1	"Prokaryotes;Bacteria;Gammaproteobacteria - Others;Lysobacter"
1363	lmb	Lysobacter maris	"Prokaryotes;Bacteria;Gammaproteobacteria - Others;Lysobacter"
1364	lyt	Lysobacter sp. TY2-98	"Prokaryotes;Bacteria;Gammaproteobacteria - Others;Lysobacter"
1365	lue	Lysobacter oculi	"Prokaryotes;Bacteria;Gammaproteobacteria - Others;Lysobacter"
1366	lyj	Lysobacter alkalisoli	"Prokaryotes;Bacteria;Gammaproteobacteria - Others;Lysobacter"
1367	lsol	Lysobacter soli	"Prokaryotes;Bacteria;Gammaproteobacteria - Others;Lysobacter"
1368	lum	Luteimonas chenhongjianii	"Prokaryotes;Bacteria;Gammaproteobacteria - Others;Luteimonas"
1369	lus	Luteimonas yindakuii	"Prokaryotes;Bacteria;Gammaproteobacteria - Others;Luteimonas"
1370	lug	Luteimonas granuli	"Prokaryotes;Bacteria;Gammaproteobacteria - Others;Luteimonas"
1371	thes	Thermomonas sp. SY21	"Prokaryotes;Bacteria;Gammaproteobacteria - Others;Thermomonas"
1372	theh	Thermomonas sp. HDW16	"Prokaryotes;Bacteria;Gammaproteobacteria - Others;Thermomonas"
1373	tcn	Thermomonas carbonis	"Prokaryotes;Bacteria;Gammaproteobacteria - Others;Thermomonas"
1374	tbv	Thermomonas brevis	"Prokaryotes;Bacteria;Gammaproteobacteria - Others;Thermomonas"
1375	xbc	Pseudolysobacter antarcticus	"Prokaryotes;Bacteria;Gammaproteobacteria - Others;Pseudolysobacter"
1376	fau	Frateuria aurantia	"Prokaryotes;Bacteria;Gammaproteobacteria - Others;Frateuria"
1377	rhd	Rhodanobacter denitrificans	"Prokaryotes;Bacteria;Gammaproteobacteria - Others;Rhodanobacter"
1378	rgl	Rhodanobacter glycinis	"Prokaryotes;Bacteria;Gammaproteobacteria - Others;Rhodanobacter"
1379	dji	Dyella jiangningensis	"Prokaryotes;Bacteria;Gammaproteobacteria - Others;Dyella"
1380	dja	Dyella japonica	"Prokaryotes;Bacteria;Gammaproteobacteria - Others;Dyella"
1381	dtx	Dyella thiooxydans	"Prokaryotes;Bacteria;Gammaproteobacteria - Others;Dyella"
1382	dye	Dyella sp. M7H15-1	"Prokaryotes;Bacteria;Gammaproteobacteria - Others;Dyella"
1383	dko	Dokdonella koreensis	"Prokaryotes;Bacteria;Gammaproteobacteria - Others;Dokdonella"

1384	lrz	Luteibacter rhizovicinus DSM 16549	"Prokaryotes;Bacteria;Gammaproteobacteria - Others;Luteibacter"
1385	lpy	Luteibacter pinisoli	"Prokaryotes;Bacteria;Gammaproteobacteria - Others;Luteibacter"
1386	xba	Ahniella affigens	"Prokaryotes;Bacteria;Gammaproteobacteria - Others;Ahniella"
1387	rbd	Aerosticca soli	"Prokaryotes;Bacteria;Gammaproteobacteria - Others;Aerosticca"
1388	vch	Vibrio cholerae O1 El Tor N16961	"Prokaryotes;Bacteria;Gammaproteobacteria - Others;Vibrio"
1389	vcf	Vibrio cholerae O1 El Tor FJ147	"Prokaryotes;Bacteria;Gammaproteobacteria - Others;Vibrio"
1390	vcs	Vibrio cholerae O1 El Tor MS6	"Prokaryotes;Bacteria;Gammaproteobacteria - Others;Vibrio"
1391	vce	Vibrio cholerae O1 2010EL-1786	"Prokaryotes;Bacteria;Gammaproteobacteria - Others;Vibrio"
1392	vcq	Vibrio cholerae O1 2012EL-2176	"Prokaryotes;Bacteria;Gammaproteobacteria - Others;Vibrio"
1393	vcj	Vibrio cholerae O1 MJ-1236	"Prokaryotes;Bacteria;Gammaproteobacteria - Others;Vibrio"
1394	vci	Vibrio cholerae O1 IEC224	"Prokaryotes;Bacteria;Gammaproteobacteria - Others;Vibrio"
1395	vco	Vibrio cholerae O1 O395	"Prokaryotes;Bacteria;Gammaproteobacteria - Others;Vibrio"
1396	vcr	Vibrio cholerae O1 O395	"Prokaryotes;Bacteria;Gammaproteobacteria - Others;Vibrio"
1397	vcm	Vibrio cholerae O1 M66-2	"Prokaryotes;Bacteria;Gammaproteobacteria - Others;Vibrio"
1398	vcl	Vibrio cholerae O1 LMA3984-4	"Prokaryotes;Bacteria;Gammaproteobacteria - Others;Vibrio"
1399	vcx	Vibrio cholerae O49 1154-74	"Prokaryotes;Bacteria;Gammaproteobacteria - Others;Vibrio"
1400	vcz	Vibrio cholerae O27 10432-62	"Prokaryotes;Bacteria;Gammaproteobacteria - Others;Vibrio"
1401	vvu	Vibrio vulnificus CMCP6	"Prokaryotes;Bacteria;Gammaproteobacteria - Others;Vibrio"
1402	vvy	Vibrio vulnificus YJ016	"Prokaryotes;Bacteria;Gammaproteobacteria - Others;Vibrio"
1403	vvm	Vibrio vulnificus MO6-24/O	"Prokaryotes;Bacteria;Gammaproteobacteria - Others;Vibrio"
1404	vvl	Vibrio vulnificus 93U204	"Prokaryotes;Bacteria;Gammaproteobacteria - Others;Vibrio"
1405	vpa	Vibrio parahaemolyticus RIMD 2210633	"Prokaryotes;Bacteria;Gammaproteobacteria - Others;Vibrio"
1406	vpb	Vibrio parahaemolyticus BB22OP	"Prokaryotes;Bacteria;Gammaproteobacteria - Others;Vibrio"
1407	vpk	Vibrio parahaemolyticus O1:K33 CDC_K4557	"Prokaryotes;Bacteria;Gammaproteobacteria - Others;Vibrio"
1408	vpf	Vibrio parahaemolyticus O1:Kuk FDA_R31	"Prokaryotes;Bacteria;Gammaproteobacteria - Others;Vibrio"
1409	vph	Vibrio parahaemolyticus UCM-V493	"Prokaryotes;Bacteria;Gammaproteobacteria - Others;Vibrio"
1410	vha	Vibrio campbellii	"Prokaryotes;Bacteria;Gammaproteobacteria - Others;Vibrio"
1411	vca	Vibrio campbellii	"Prokaryotes;Bacteria;Gammaproteobacteria - Others;Vibrio"
1412	vag	Vibrio alginolyticus	"Prokaryotes;Bacteria;Gammaproteobacteria - Others;Vibrio"
1413	vex	Vibrio antiquarius	"Prokaryotes;Bacteria;Gammaproteobacteria - Others;Vibrio"
1414	vdb	Vibrio diabolicus	"Prokaryotes;Bacteria;Gammaproteobacteria - Others;Vibrio"
1415	vhr	Vibrio harveyi	"Prokaryotes;Bacteria;Gammaproteobacteria - Others;Vibrio"
1416	vna	Vibrio natriegens	"Prokaryotes;Bacteria;Gammaproteobacteria - Others;Vibrio"
1417	vow	Vibrio owensii	"Prokaryotes;Bacteria;Gammaproteobacteria - Others;Vibrio"
1418	vro	Vibrio rotiferianus	"Prokaryotes;Bacteria;Gammaproteobacteria - Others;Vibrio"
1419	vsp	Vibrio atlanticus	"Prokaryotes;Bacteria;Gammaproteobacteria - Others;Vibrio"
1420	vej	Vibrio sp. EJY3	"Prokaryotes;Bacteria;Gammaproteobacteria - Others;Vibrio"
1421	vfu	Vibrio furnissii	"Prokaryotes;Bacteria;Gammaproteobacteria - Others;Vibrio"
1422	vni	Vibrio nigripulchritudo	"Prokaryotes;Bacteria;Gammaproteobacteria - Others;Vibrio"
1423	van	Vibrio anguillarum 775	"Prokaryotes;Bacteria;Gammaproteobacteria - Others;Vibrio"
1424	lag	Vibrio anguillarum M3	"Prokaryotes;Bacteria;Gammaproteobacteria - Others;Vibrio"

1425	vau	Vibrio anguillarum NB10	"Prokaryotes;Bacteria;Gammaproteobacteria - Others;Vibrio"
1426	vcy	Vibrio coralliilyticus RE98	"Prokaryotes;Bacteria;Gammaproteobacteria - Others;Vibrio"
1427	vct	Vibrio coralliilyticus OCN014	"Prokaryotes;Bacteria;Gammaproteobacteria - Others;Vibrio"
1428	vtu	Vibrio tubiashii	"Prokaryotes;Bacteria;Gammaproteobacteria - Others;Vibrio"
1429	vfl	Vibrio fluvialis	"Prokaryotes;Bacteria;Gammaproteobacteria - Others;Vibrio"
1430	vmi	Vibrio mimicus	"Prokaryotes;Bacteria;Gammaproteobacteria - Others;Vibrio"
1431	vbr	Vibrio breoganii	"Prokaryotes;Bacteria;Gammaproteobacteria - Others;Vibrio"
1432	vsc	Vibrio scophthalmi	"Prokaryotes;Bacteria;Gammaproteobacteria - Others;Vibrio"
1433	vga	Vibrio gazogenes	"Prokaryotes;Bacteria;Gammaproteobacteria - Others;Vibrio"
1434	vsh	Vibrio mediterranei	"Prokaryotes;Bacteria;Gammaproteobacteria - Others;Vibrio"
1435	vqi	Vibrio qinghaiensis	"Prokaryotes;Bacteria;Gammaproteobacteria - Others;Vibrio"
1436	vta	Vibrio tapetis subsp. tapetis	"Prokaryotes;Bacteria;Gammaproteobacteria - Others;Vibrio"
1437	vaf	Vibrio alfacsensis	"Prokaryotes;Bacteria;Gammaproteobacteria - Others;Vibrio"
1438	vnl	Vibrio neocaledonicus	"Prokaryotes;Bacteria;Gammaproteobacteria - Others;Vibrio"
1439	vcc	Vibrio cyclitrophicus	"Prokaryotes;Bacteria;Gammaproteobacteria - Others;Vibrio"
1440	vas	Vibrio astriarenae	"Prokaryotes;Bacteria;Gammaproteobacteria - Others;Vibrio"
1441	vaq	Vibrio aquimaris	"Prokaryotes;Bacteria;Gammaproteobacteria - Others;Vibrio"
1442	vsr	Vibrio spartinae	"Prokaryotes;Bacteria;Gammaproteobacteria - Others;Vibrio"
1443	vfi	Aliivibrio fischeri ES114	"Prokaryotes;Bacteria;Gammaproteobacteria - Others;Aliivibrio"
1444	vfm	Aliivibrio fischeri MJ11	"Prokaryotes;Bacteria;Gammaproteobacteria - Others;Aliivibrio"
1445	vsa	Aliivibrio salmonicida	"Prokaryotes;Bacteria;Gammaproteobacteria - Others;Aliivibrio"
1446	awd	Aliivibrio wodanis	"Prokaryotes;Bacteria;Gammaproteobacteria - Others;Aliivibrio"
1447	ppr	Photobacterium profundum	"Prokaryotes;Bacteria;Gammaproteobacteria - Others;Photobacterium"
1448	pgb	Photobacterium gaetbulicola	"Prokaryotes;Bacteria;Gammaproteobacteria - Others;Photobacterium"
1449	pds	Photobacterium damselae subsp. damselae	"Prokaryotes;Bacteria;Gammaproteobacteria - Others;Photobacterium"
1450	gho	Grimontia hollisae	"Prokaryotes;Bacteria;Gammaproteobacteria - Others;Grimontia"
1451	pmai	Paraphotobacterium marinum	"Prokaryotes;Bacteria;Gammaproteobacteria - Others;Paraphotobacterium"
1452	saly	Salinivibrio sp. YCSC6	"Prokaryotes;Bacteria;Gammaproteobacteria - Others;Salinivibrio"
1453	sks	Salinivibrio kushneri	"Prokaryotes;Bacteria;Gammaproteobacteria - Others;Salinivibrio"
1454	scot	Salinivibrio costicola	"Prokaryotes;Bacteria;Gammaproteobacteria - Others;Salinivibrio"
1455	pae	Pseudomonas aeruginosa PAO1	"Prokaryotes;Bacteria;Gammaproteobacteria - Others;Pseudomonas"
1456	paev	Pseudomonas aeruginosa PAO1-VE13	"Prokaryotes;Bacteria;Gammaproteobacteria - Others;Pseudomonas"
1457	paei	Pseudomonas aeruginosa PAO1-VE2	"Prokaryotes;Bacteria;Gammaproteobacteria - Others;Pseudomonas"
1458	pau	Pseudomonas aeruginosa UCBPP-PA14	"Prokaryotes;Bacteria;Gammaproteobacteria - Others;Pseudomonas"
1459	pap	Pseudomonas aeruginosa PA7	"Prokaryotes;Bacteria;Gammaproteobacteria - Others;Pseudomonas"
1460	pag	Pseudomonas aeruginosa LESB58	"Prokaryotes;Bacteria;Gammaproteobacteria - Others;Pseudomonas"
1461	paf	Pseudomonas aeruginosa M18	"Prokaryotes;Bacteria;Gammaproteobacteria - Others;Pseudomonas"
1462	pnc	Pseudomonas aeruginosa NCGM2.S1	"Prokaryotes;Bacteria;Gammaproteobacteria - Others;Pseudomonas"
1463	paeb	Pseudomonas aeruginosa NCGM 1900	"Prokaryotes;Bacteria;Gammaproteobacteria - Others;Pseudomonas"
1464	pdk	Pseudomonas aeruginosa DK2	"Prokaryotes;Bacteria;Gammaproteobacteria - Others;Pseudomonas"
1465	psg	Pseudomonas aeruginosa B136-33	"Prokaryotes;Bacteria;Gammaproteobacteria - Others;Pseudomonas"

1466	prp	Pseudomonas aeruginosa RP73	"Prokaryotes;Bacteria;Gammaproteobacteria - Others;Pseudomonas"
1467	paep	Pseudomonas aeruginosa PA1	"Prokaryotes;Bacteria;Gammaproteobacteria - Others;Pseudomonas"
1468	paer	Pseudomonas aeruginosa PA1R	"Prokaryotes;Bacteria;Gammaproteobacteria - Others;Pseudomonas"
1469	paem	Pseudomonas aeruginosa MTB-1	"Prokaryotes;Bacteria;Gammaproteobacteria - Others;Pseudomonas"
1470	pael	Pseudomonas aeruginosa LES431	"Prokaryotes;Bacteria;Gammaproteobacteria - Others;Pseudomonas"
1471	paes	Pseudomonas aeruginosa SCV20265	"Prokaryotes;Bacteria;Gammaproteobacteria - Others;Pseudomonas"
1472	paeu	Pseudomonas aeruginosa PA38182	"Prokaryotes;Bacteria;Gammaproteobacteria - Others;Pseudomonas"
1473	paeg	Pseudomonas aeruginosa YL84	"Prokaryotes;Bacteria;Gammaproteobacteria - Others;Pseudomonas"
1474	paec	Pseudomonas aeruginosa c7447m	"Prokaryotes;Bacteria;Gammaproteobacteria - Others;Pseudomonas"
1475	paeo	Pseudomonas aeruginosa PAO581	"Prokaryotes;Bacteria;Gammaproteobacteria - Others;Pseudomonas"
1476	pmy	Pseudomonas mendocina ymp	"Prokaryotes;Bacteria;Gammaproteobacteria - Others;Pseudomonas"
1477	pmk	Pseudomonas mendocina NK-01	"Prokaryotes;Bacteria;Gammaproteobacteria - Others;Pseudomonas"
1478	pre	Pseudomonas resinovorans	"Prokaryotes;Bacteria;Gammaproteobacteria - Others;Pseudomonas"
1479	ppse	Pseudomonas pseudoalcaligenes	"Prokaryotes;Bacteria;Gammaproteobacteria - Others;Pseudomonas"
1480	palc	Pseudomonas alcaligenes	"Prokaryotes;Bacteria;Gammaproteobacteria - Others;Pseudomonas"
1481	pcq	Pseudomonas citronellolis	"Prokaryotes;Bacteria;Gammaproteobacteria - Others;Pseudomonas"
1482	ppu	Pseudomonas putida KT2440	"Prokaryotes;Bacteria;Gammaproteobacteria - Others;Pseudomonas"
1483	ppf	Pseudomonas putida F1	"Prokaryotes;Bacteria;Gammaproteobacteria - Others;Pseudomonas"
1484	ppg	Pseudomonas putida GB-1	"Prokaryotes;Bacteria;Gammaproteobacteria - Others;Pseudomonas"
1485	ppw	Pseudomonas putida W619	"Prokaryotes;Bacteria;Gammaproteobacteria - Others;Pseudomonas"
1486	ppt	Pseudomonas putida S16	"Prokaryotes;Bacteria;Gammaproteobacteria - Others;Pseudomonas"
1487	ppb	Pseudomonas putida BIRD-1	"Prokaryotes;Bacteria;Gammaproteobacteria - Others;Pseudomonas"
1488	ppi	Pseudomonas putida ND6	"Prokaryotes;Bacteria;Gammaproteobacteria - Others;Pseudomonas"
1489	ppx	Pseudomonas putida DOT-T1E	"Prokaryotes;Bacteria;Gammaproteobacteria - Others;Pseudomonas"
1490	ppuh	Pseudomonas putida HB3267	"Prokaryotes;Bacteria;Gammaproteobacteria - Others;Pseudomonas"
1491	pput	Pseudomonas putida H8234	"Prokaryotes;Bacteria;Gammaproteobacteria - Others;Pseudomonas"
1492	ppun	Pseudomonas putida NBRC 14164	"Prokaryotes;Bacteria;Gammaproteobacteria - Others;Pseudomonas"
1493	ppud	Pseudomonas putida DLL-E4	"Prokaryotes;Bacteria;Gammaproteobacteria - Others;Pseudomonas"
1494	pfv	Pseudomonas fulva	"Prokaryotes;Bacteria;Gammaproteobacteria - Others;Pseudomonas"
1495	pmon	Pseudomonas monteilii SB3078	"Prokaryotes;Bacteria;Gammaproteobacteria - Others;Pseudomonas"
1496	pmot	Pseudomonas monteilii SB3101	"Prokaryotes;Bacteria;Gammaproteobacteria - Others;Pseudomonas"
1497	pmos	Pseudomonas soli	"Prokaryotes;Bacteria;Gammaproteobacteria - Others;Pseudomonas"
1498	ppj	Pseudomonas plecoglossicida	"Prokaryotes;Bacteria;Gammaproteobacteria - Others;Pseudomonas"
1499	por	Pseudomonas oryzihabitans	"Prokaryotes;Bacteria;Gammaproteobacteria - Others;Pseudomonas"
1500	pst	Pseudomonas syringae pv. tomato DC3000	"Prokaryotes;Bacteria;Gammaproteobacteria - Others;Pseudomonas"
1501	psb	Pseudomonas syringae pv. syringae B728a	"Prokaryotes;Bacteria;Gammaproteobacteria - Others;Pseudomonas"
1502	psyr	Pseudomonas syringae CC1557	"Prokaryotes;Bacteria;Gammaproteobacteria - Others;Pseudomonas"
1503	psp	Pseudomonas savastanoi pv. phaseolicola 1448A	"Prokaryotes;Bacteria;Gammaproteobacteria - Others;Pseudomonas"
1504	pamg	Pseudomonas amygdali	"Prokaryotes;Bacteria;Gammaproteobacteria - Others;Pseudomonas"
1505	pci	Pseudomonas cichorii	"Prokaryotes;Bacteria;Gammaproteobacteria - Others;Pseudomonas"
1506	pavl	Pseudomonas avellanae	"Prokaryotes;Bacteria;Gammaproteobacteria - Others;Pseudomonas"

1507	pvd	Pseudomonas viridiflava	"Prokaryotes;Bacteria;Gammaproteobacteria - Others;Pseudomonas"
1508	pfl	Pseudomonas protegens Pf-5	"Prokaryotes;Bacteria;Gammaproteobacteria - Others;Pseudomonas"
1509	pprc	Pseudomonas protegens CHA0	"Prokaryotes;Bacteria;Gammaproteobacteria - Others;Pseudomonas"
1510	ppro	Pseudomonas protegens Cab57	"Prokaryotes;Bacteria;Gammaproteobacteria - Others;Pseudomonas"
1511	pfo	Pseudomonas fluorescens Pf0-1	"Prokaryotes;Bacteria;Gammaproteobacteria - Others;Pseudomonas"
1512	pfs	Pseudomonas fluorescens SBW25	"Prokaryotes;Bacteria;Gammaproteobacteria - Others;Pseudomonas"
1513	pfe	Pseudomonas fluorescens F113	"Prokaryotes;Bacteria;Gammaproteobacteria - Others;Pseudomonas"
1514	pfc	Pseudomonas fluorescens A506	"Prokaryotes;Bacteria;Gammaproteobacteria - Others;Pseudomonas"
1515	pfn	Pseudomonas fluorescens UK4	"Prokaryotes;Bacteria;Gammaproteobacteria - Others;Pseudomonas"
1516	ppz	Pseudomonas poae	"Prokaryotes;Bacteria;Gammaproteobacteria - Others;Pseudomonas"
1517	pfb	Pseudomonas synxantha LBUM223	"Prokaryotes;Bacteria;Gammaproteobacteria - Others;Pseudomonas"
1518	pman	Pseudomonas mandelii	"Prokaryotes;Bacteria;Gammaproteobacteria - Others;Pseudomonas"
1519	ptv	Pseudomonas trivialis	"Prokaryotes;Bacteria;Gammaproteobacteria - Others;Pseudomonas"
1520	pcg	Pseudomonas corrugata	"Prokaryotes;Bacteria;Gammaproteobacteria - Others;Pseudomonas"
1521	pvr	Pseudomonas veronii	"Prokaryotes;Bacteria;Gammaproteobacteria - Others;Pseudomonas"
1522	pazo	Pseudomonas azotoformans	"Prokaryotes;Bacteria;Gammaproteobacteria - Others;Pseudomonas"
1523	poi	Pseudomonas orientalis	"Prokaryotes;Bacteria;Gammaproteobacteria - Others;Pseudomonas"
1524	pfw	Pseudomonas simiae PCL1751	"Prokaryotes;Bacteria;Gammaproteobacteria - Others;Pseudomonas"
1525	pff	Pseudomonas simiae PICF7	"Prokaryotes;Bacteria;Gammaproteobacteria - Others;Pseudomonas"
1526	pfx	Pseudomonas lurida	"Prokaryotes;Bacteria;Gammaproteobacteria - Others;Pseudomonas"
1527	pen	Pseudomonas entomophila	"Prokaryotes;Bacteria;Gammaproteobacteria - Others;Pseudomonas"
1528	psa	Pseudomonas stutzeri A1501	"Prokaryotes;Bacteria;Gammaproteobacteria - Others;Pseudomonas"
1529	psz	Pseudomonas stutzeri ATCC 17588	"Prokaryotes;Bacteria;Gammaproteobacteria - Others;Pseudomonas"
1530	psr	Pseudomonas stutzeri DSM 4166	"Prokaryotes;Bacteria;Gammaproteobacteria - Others;Pseudomonas"
1531	psc	Pseudomonas stutzeri CCUG 29243	"Prokaryotes;Bacteria;Gammaproteobacteria - Others;Pseudomonas"
1532	psj	Pseudomonas stutzeri DSM 10701	"Prokaryotes;Bacteria;Gammaproteobacteria - Others;Pseudomonas"
1533	psh	Pseudomonas stutzeri RCH2	"Prokaryotes;Bacteria;Gammaproteobacteria - Others;Pseudomonas"
1534	pstu	Pseudomonas stutzeri 19SMN4	"Prokaryotes;Bacteria;Gammaproteobacteria - Others;Pseudomonas"
1535	pstt	Pseudomonas stutzeri 28a24	"Prokaryotes;Bacteria;Gammaproteobacteria - Others;Pseudomonas"
1536	pbm	Pseudomonas balearica	"Prokaryotes;Bacteria;Gammaproteobacteria - Others;Pseudomonas"
1537	plul	Pseudomonas luteola	"Prokaryotes;Bacteria;Gammaproteobacteria - Others;Pseudomonas"
1538	pba	Pseudomonas brassicacearum subsp. brassicacearum NFM421	"Prokaryotes;Bacteria;Gammaproteobacteria - Others;Pseudomonas"
1539	pbc	Pseudomonas brassicacearum DF41	"Prokaryotes;Bacteria;Gammaproteobacteria - Others;Pseudomonas"
1540	ppuu	Pseudomonas sp. UW4	"Prokaryotes;Bacteria;Gammaproteobacteria - Others;Pseudomonas"
1541	pdr	Pseudomonas sp. ATCC 13867	"Prokaryotes;Bacteria;Gammaproteobacteria - Others;Pseudomonas"
1542	psv	Pseudomonas sp. VLB120	"Prokaryotes;Bacteria;Gammaproteobacteria - Others;Pseudomonas"
1543	psk	Pseudomonas sp. TKP	"Prokaryotes;Bacteria;Gammaproteobacteria - Others;Pseudomonas"
1544	pkc	Pseudomonas knackmussii	"Prokaryotes;Bacteria;Gammaproteobacteria - Others;Pseudomonas"
1545	pch	Pseudomonas chlororaphis PA23	"Prokaryotes;Bacteria;Gammaproteobacteria - Others;Pseudomonas"
1546	pcz	Pseudomonas chlororaphis PCL1606	"Prokaryotes;Bacteria;Gammaproteobacteria - Others;Pseudomonas"
1547	pcp	Pseudomonas chlororaphis subsp. aurantiaca	"Prokaryotes;Bacteria;Gammaproteobacteria - Others;Pseudomonas"

1548	pfz	Pseudomonas fragi	"Prokaryotes;Bacteria;Gammaproteobacteria - Others;Pseudomonas"
1549	plq	Pseudomonas lundensis	"Prokaryotes;Bacteria;Gammaproteobacteria - Others;Pseudomonas"
1550	palk	Pseudomonas alkylphenolica	"Prokaryotes;Bacteria;Gammaproteobacteria - Others;Pseudomonas"
1551	prh	Pseudomonas rhizosphaerae	"Prokaryotes;Bacteria;Gammaproteobacteria - Others;Pseudomonas"
1552	psw	Pseudomonas cremoricolorata	"Prokaryotes;Bacteria;Gammaproteobacteria - Others;Pseudomonas"
1553	ppv	Pseudomonas parafulva	"Prokaryotes;Bacteria;Gammaproteobacteria - Others;Pseudomonas"
1554	pses	Pseudomonas sp. StFLB209	"Prokaryotes;Bacteria;Gammaproteobacteria - Others;Pseudomonas"
1555	psem	Pseudomonas sp. MRSN12121	"Prokaryotes;Bacteria;Gammaproteobacteria - Others;Pseudomonas"
1556	psec	Pseudomonas sp. CCOS 191	"Prokaryotes;Bacteria;Gammaproteobacteria - Others;Pseudomonas"
1557	ppsy	Pseudomonas versuta	"Prokaryotes;Bacteria;Gammaproteobacteria - Others;Pseudomonas"
1558	psos	Pseudomonas sp. Os17	"Prokaryotes;Bacteria;Gammaproteobacteria - Others;Pseudomonas"
1559	pkr	Pseudomonas koreensis	"Prokaryotes;Bacteria;Gammaproteobacteria - Others;Pseudomonas"
1560	pfk	Pseudomonas frederiksbergensis	"Prokaryotes;Bacteria;Gammaproteobacteria - Others;Pseudomonas"
1561	panr	Pseudomonas antarctica	"Prokaryotes;Bacteria;Gammaproteobacteria - Others;Pseudomonas"
1562	ppsl	Pseudomonas psychrotolerans	"Prokaryotes;Bacteria;Gammaproteobacteria - Others;Pseudomonas"
1563	pset	Pseudomonas sp. TCU-HL1	"Prokaryotes;Bacteria;Gammaproteobacteria - Others;Pseudomonas"
1564	psil	Pseudomonas silesiensis	"Prokaryotes;Bacteria;Gammaproteobacteria - Others;Pseudomonas"
1565	pym	Pseudomonas yamanorum	"Prokaryotes;Bacteria;Gammaproteobacteria - Others;Pseudomonas"
1566	pade	Candidatus Pseudomonas adelgestsugas	"Prokaryotes;Bacteria;Gammaproteobacteria - Others;Pseudomonas"
1567	psed	Pseudomonas sp. R2A2	"Prokaryotes;Bacteria;Gammaproteobacteria - Others;Pseudomonas"
1568	pke	Pseudomonas kribbensis	"Prokaryotes;Bacteria;Gammaproteobacteria - Others;Pseudomonas"
1569	pall	Pseudomonas alcaliphila	"Prokaryotes;Bacteria;Gammaproteobacteria - Others;Pseudomonas"
1570	pum	Pseudomonas umsongensis	"Prokaryotes;Bacteria;Gammaproteobacteria - Others;Pseudomonas"
1571	poj	Pseudomonas otitidis	"Prokaryotes;Bacteria;Gammaproteobacteria - Others;Pseudomonas"
1572	pgg	Pseudomonas graminis	"Prokaryotes;Bacteria;Gammaproteobacteria - Others;Pseudomonas"
1573	ppsh	Pseudomonas psychrophila	"Prokaryotes;Bacteria;Gammaproteobacteria - Others;Pseudomonas"
1574	pgy	Pseudomonas glycinae	"Prokaryotes;Bacteria;Gammaproteobacteria - Others;Pseudomonas"
1575	avn	Azotobacter vinelandii DJ	"Prokaryotes;Bacteria;Gammaproteobacteria - Others;Azotobacter"
1576	avl	Azotobacter vinelandii CA	"Prokaryotes;Bacteria;Gammaproteobacteria - Others;Azotobacter"
1577	avd	Azotobacter vinelandii CA6	"Prokaryotes;Bacteria;Gammaproteobacteria - Others;Azotobacter"
1578	acx	Azotobacter chroococcum	"Prokaryotes;Bacteria;Gammaproteobacteria - Others;Azotobacter"
1579	pbb	Oblitimonas alkaliphila	"Prokaryotes;Bacteria;Gammaproteobacteria - Others;Oblitimonas"
1580	pagr	Permianibacter aggregans	"Prokaryotes;Bacteria;Gammaproteobacteria - Others;Permianibacter"
1581	emo	Entomomonas moraniae	"Prokaryotes;Bacteria;Gammaproteobacteria - Others;Entomomonas"
1582	par	Psychrobacter arcticus	"Prokaryotes;Bacteria;Gammaproteobacteria - Others;Psychrobacter"
1583	pcr	Psychrobacter cryohalolentis	"Prokaryotes;Bacteria;Gammaproteobacteria - Others;Psychrobacter"
1584	prw	Psychrobacter sp. PRwf-1	"Prokaryotes;Bacteria;Gammaproteobacteria - Others;Psychrobacter"
1585	pso	Psychrobacter sp. G	"Prokaryotes;Bacteria;Gammaproteobacteria - Others;Psychrobacter"
1586	pur	Psychrobacter urativorans	"Prokaryotes;Bacteria;Gammaproteobacteria - Others;Psychrobacter"
1587	pali	Psychrobacter alimentarius	"Prokaryotes;Bacteria;Gammaproteobacteria - Others;Psychrobacter"
1588	pspg	Psychrobacter sp. P2G3	"Prokaryotes;Bacteria;Gammaproteobacteria - Others;Psychrobacter"

1589	psyg	Psychrobacter sp. P11G5	"Prokaryotes;Bacteria;Gammaproteobacteria - Others;Psychrobacter"
1590	psyc	Psychrobacter sp. DAB_AL43B	"Prokaryotes;Bacteria;Gammaproteobacteria - Others;Psychrobacter"
1591	psya	Psychrobacter sp. AntiMn-1	"Prokaryotes;Bacteria;Gammaproteobacteria - Others;Psychrobacter"
1592	psyy	Psychrobacter sp. YP14	"Prokaryotes;Bacteria;Gammaproteobacteria - Others;Psychrobacter"
1593	psyp	Psychrobacter sp. PAMC27889	"Prokaryotes;Bacteria;Gammaproteobacteria - Others;Psychrobacter"
1594	acb	Acinetobacter baumannii ATCC 17978	"Prokaryotes;Bacteria;Gammaproteobacteria - Others;Acinetobacter"
1595	abm	Acinetobacter baumannii SDF	"Prokaryotes;Bacteria;Gammaproteobacteria - Others;Acinetobacter"
1596	aby	Acinetobacter baumannii AYE	"Prokaryotes;Bacteria;Gammaproteobacteria - Others;Acinetobacter"
1597	abc	Acinetobacter baumannii ACICU	"Prokaryotes;Bacteria;Gammaproteobacteria - Others;Acinetobacter"
1598	abn	Acinetobacter baumannii AB0057	"Prokaryotes;Bacteria;Gammaproteobacteria - Others;Acinetobacter"
1599	abb	Acinetobacter baumannii AB307-0294	"Prokaryotes;Bacteria;Gammaproteobacteria - Others;Acinetobacter"
1600	abx	Acinetobacter baumannii 1656-2	"Prokaryotes;Bacteria;Gammaproteobacteria - Others;Acinetobacter"
1601	abz	Acinetobacter baumannii MDR-ZJ06	"Prokaryotes;Bacteria;Gammaproteobacteria - Others;Acinetobacter"
1602	abr	Acinetobacter baumannii MDR-TJ	"Prokaryotes;Bacteria;Gammaproteobacteria - Others;Acinetobacter"
1603	abd	Acinetobacter baumannii TCDC-AB0715	"Prokaryotes;Bacteria;Gammaproteobacteria - Others;Acinetobacter"
1604	abh	Acinetobacter baumannii TYTH-1	"Prokaryotes;Bacteria;Gammaproteobacteria - Others;Acinetobacter"
1605	abad	Acinetobacter baumannii D1279779	"Prokaryotes;Bacteria;Gammaproteobacteria - Others;Acinetobacter"
1606	abj	Acinetobacter baumannii BJAB07104	"Prokaryotes;Bacteria;Gammaproteobacteria - Others;Acinetobacter"
1607	abab	Acinetobacter baumannii BJAB0715	"Prokaryotes;Bacteria;Gammaproteobacteria - Others;Acinetobacter"
1608	abaj	Acinetobacter baumannii BJAB0868	"Prokaryotes;Bacteria;Gammaproteobacteria - Others;Acinetobacter"
1609	abaz	Acinetobacter baumannii ZW85-1	"Prokaryotes;Bacteria;Gammaproteobacteria - Others;Acinetobacter"
1610	abk	Acinetobacter baumannii AbH12O-A2	"Prokaryotes;Bacteria;Gammaproteobacteria - Others;Acinetobacter"
1611	abau	Acinetobacter baumannii AB030	"Prokaryotes;Bacteria;Gammaproteobacteria - Others;Acinetobacter"
1612	abaa	Acinetobacter baumannii AB031	"Prokaryotes;Bacteria;Gammaproteobacteria - Others;Acinetobacter"
1613	abw	Acinetobacter baumannii AC29	"Prokaryotes;Bacteria;Gammaproteobacteria - Others;Acinetobacter"
1614	abal	Acinetobacter baumannii LAC-4	"Prokaryotes;Bacteria;Gammaproteobacteria - Others;Acinetobacter"
1615	acc	Acinetobacter pittii	"Prokaryotes;Bacteria;Gammaproteobacteria - Others;Acinetobacter"
1616	ano	Acinetobacter nosocomialis	"Prokaryotes;Bacteria;Gammaproteobacteria - Others;Acinetobacter"
1617	alc	Acinetobacter lactucae	"Prokaryotes;Bacteria;Gammaproteobacteria - Others;Acinetobacter"
1618	acal	Acinetobacter calcoaceticus	"Prokaryotes;Bacteria;Gammaproteobacteria - Others;Acinetobacter"
1619	acd	Acinetobacter oleivorans	"Prokaryotes;Bacteria;Gammaproteobacteria - Others;Acinetobacter"
1620	aci	Acinetobacter baylyi	"Prokaryotes;Bacteria;Gammaproteobacteria - Others;Acinetobacter"
1621	att	Acinetobacter sp. TTH0-4	"Prokaryotes;Bacteria;Gammaproteobacteria - Others;Acinetobacter"
1622	aei	Acinetobacter equi	"Prokaryotes;Bacteria;Gammaproteobacteria - Others;Acinetobacter"
1623	ajo	Acinetobacter johnsonii	"Prokaryotes;Bacteria;Gammaproteobacteria - Others;Acinetobacter"
1624	acw	Acinetobacter sp. DUT-2	"Prokaryotes;Bacteria;Gammaproteobacteria - Others;Acinetobacter"
1625	acv	Acinetobacter sp. TGL-Y2	"Prokaryotes;Bacteria;Gammaproteobacteria - Others;Acinetobacter"
1626	ahl	Acinetobacter haemolyticus	"Prokaryotes;Bacteria;Gammaproteobacteria - Others;Acinetobacter"
1627	ajn	Acinetobacter junii	"Prokaryotes;Bacteria;Gammaproteobacteria - Others;Acinetobacter"
1628	asol	Acinetobacter soli	"Prokaryotes;Bacteria;Gammaproteobacteria - Others;Acinetobacter"
1629	ala	Acinetobacter larvae	"Prokaryotes;Bacteria;Gammaproteobacteria - Others;Acinetobacter"

1630	asj	Acinetobacter schindleri	"Prokaryotes;Bacteria;Gammaproteobacteria - Others;Acinetobacter"
1631	aid	Acinetobacter indicus	"Prokaryotes;Bacteria;Gammaproteobacteria - Others;Acinetobacter"
1632	adv	Acinetobacter defluvii	"Prokaryotes;Bacteria;Gammaproteobacteria - Others;Acinetobacter"
1633	arj	Acinetobacter radioresistens	"Prokaryotes;Bacteria;Gammaproteobacteria - Others;Acinetobacter"
1634	awu	Acinetobacter wuhouensis	"Prokaryotes;Bacteria;Gammaproteobacteria - Others;Acinetobacter"
1635	acum	Acinetobacter cumulans	"Prokaryotes;Bacteria;Gammaproteobacteria - Others;Acinetobacter"
1636	agu	Acinetobacter guillouiae	"Prokaryotes;Bacteria;Gammaproteobacteria - Others;Acinetobacter"
1637	aug	Acinetobacter ursingii	"Prokaryotes;Bacteria;Gammaproteobacteria - Others;Acinetobacter"
1638	alw	Acinetobacter lwoffii	"Prokaryotes;Bacteria;Gammaproteobacteria - Others;Acinetobacter"
1639	ads	Acinetobacter dispersus	"Prokaryotes;Bacteria;Gammaproteobacteria - Others;Acinetobacter"
1640	aber	Acinetobacter bereziniae	"Prokaryotes;Bacteria;Gammaproteobacteria - Others;Acinetobacter"
1641	atn	Acinetobacter tandoii	"Prokaryotes;Bacteria;Gammaproteobacteria - Others;Acinetobacter"
1642	achi	Acinetobacter chinensis	"Prokaryotes;Bacteria;Gammaproteobacteria - Others;Acinetobacter"
1643	alj	Acinetobacter lanii	"Prokaryotes;Bacteria;Gammaproteobacteria - Others;Acinetobacter"
1644	mct	Moraxella catarrhalis BBH18	"Prokaryotes;Bacteria;Gammaproteobacteria - Others;Moraxella"
1645	mcs	Moraxella catarrhalis 25240	"Prokaryotes;Bacteria;Gammaproteobacteria - Others;Moraxella"
1646	mcat	Moraxella catarrhalis 25239	"Prokaryotes;Bacteria;Gammaproteobacteria - Others;Moraxella"
1647	moi	Moraxella ovis	"Prokaryotes;Bacteria;Gammaproteobacteria - Others;Moraxella"
1648	mos	Moraxella osloensis	"Prokaryotes;Bacteria;Gammaproteobacteria - Others;Moraxella"
1649	mbl	Moraxella bovoculi	"Prokaryotes;Bacteria;Gammaproteobacteria - Others;Moraxella"
1650	mboi	Moraxella bovis	"Prokaryotes;Bacteria;Gammaproteobacteria - Others;Moraxella"
1651	mcun	Moraxella cuniculi	"Prokaryotes;Bacteria;Gammaproteobacteria - Others;Moraxella"
1652	mnn	Moraxella nonliquefaciens	"Prokaryotes;Bacteria;Gammaproteobacteria - Others;Moraxella"
1653	mbah	Aquirhabdus parva	"Prokaryotes;Bacteria;Gammaproteobacteria - Others;Aquirhabdus"
1654	son	Shewanella oneidensis	"Prokaryotes;Bacteria;Gammaproteobacteria - Others;Shewanella"
1655	sdn	Shewanella denitrificans	"Prokaryotes;Bacteria;Gammaproteobacteria - Others;Shewanella"
1656	sfr	Shewanella frigidimarina	"Prokaryotes;Bacteria;Gammaproteobacteria - Others;Shewanella"
1657	saz	Shewanella amazonensis	"Prokaryotes;Bacteria;Gammaproteobacteria - Others;Shewanella"
1658	sbl	Shewanella baltica OS155	"Prokaryotes;Bacteria;Gammaproteobacteria - Others;Shewanella"
1659	sbm	Shewanella baltica OS185	"Prokaryotes;Bacteria;Gammaproteobacteria - Others;Shewanella"
1660	sbn	Shewanella baltica OS195	"Prokaryotes;Bacteria;Gammaproteobacteria - Others;Shewanella"
1661	sbp	Shewanella baltica OS223	"Prokaryotes;Bacteria;Gammaproteobacteria - Others;Shewanella"
1662	sbt	Shewanella baltica OS678	"Prokaryotes;Bacteria;Gammaproteobacteria - Others;Shewanella"
1663	sbs	Shewanella baltica OS117	"Prokaryotes;Bacteria;Gammaproteobacteria - Others;Shewanella"
1664	sbb	Shewanella baltica BA175	"Prokaryotes;Bacteria;Gammaproteobacteria - Others;Shewanella"
1665	slo	Shewanella loihica	"Prokaryotes;Bacteria;Gammaproteobacteria - Others;Shewanella"
1666	spc	Shewanella putrefaciens CN-32	"Prokaryotes;Bacteria;Gammaproteobacteria - Others;Shewanella"
1667	shp	Shewanella putrefaciens 200	"Prokaryotes;Bacteria;Gammaproteobacteria - Others;Shewanella"
1668	sse	Shewanella sediminis	"Prokaryotes;Bacteria;Gammaproteobacteria - Others;Shewanella"
1669	spl	Shewanella pealeana	"Prokaryotes;Bacteria;Gammaproteobacteria - Others;Shewanella"
1670	she	Shewanella sp. MR-4	"Prokaryotes;Bacteria;Gammaproteobacteria - Others;Shewanella"

1671	shm	Shewanella sp. MR-7	"Prokaryotes;Bacteria;Gammaproteobacteria - Others;Shewanella"
1672	shn	Shewanella sp. ANA-3	"Prokaryotes;Bacteria;Gammaproteobacteria - Others;Shewanella"
1673	shw	Shewanella sp. W3-18-1	"Prokaryotes;Bacteria;Gammaproteobacteria - Others;Shewanella"
1674	shl	Shewanella halifaxensis	"Prokaryotes;Bacteria;Gammaproteobacteria - Others;Shewanella"
1675	swd	Shewanella woodyi	"Prokaryotes;Bacteria;Gammaproteobacteria - Others;Shewanella"
1676	swp	Shewanella piezotolerans	"Prokaryotes;Bacteria;Gammaproteobacteria - Others;Shewanella"
1677	svo	Shewanella violacea	"Prokaryotes;Bacteria;Gammaproteobacteria - Others;Shewanella"
1678	shf	Shewanella sp. FDAARGOS_354	"Prokaryotes;Bacteria;Gammaproteobacteria - Others;Shewanella"
1679	sja	Shewanella japonica	"Prokaryotes;Bacteria;Gammaproteobacteria - Others;Shewanella"
1680	spsw	Shewanella psychrophila	"Prokaryotes;Bacteria;Gammaproteobacteria - Others;Shewanella"
1681	sbj	Shewanella bicestrii	"Prokaryotes;Bacteria;Gammaproteobacteria - Others;Shewanella"
1682	smav	Shewanella marisflavi	"Prokaryotes;Bacteria;Gammaproteobacteria - Others;Shewanella"
1683	shew	Shewanella sp. WE21	"Prokaryotes;Bacteria;Gammaproteobacteria - Others;Shewanella"
1684	salg	Shewanella algae	"Prokaryotes;Bacteria;Gammaproteobacteria - Others;Shewanella"
1685	slj	Shewanella livingstonensis	"Prokaryotes;Bacteria;Gammaproteobacteria - Others;Shewanella"
1686	smai	Shewanella maritima	"Prokaryotes;Bacteria;Gammaproteobacteria - Others;Shewanella"
1687	spol	Shewanella polaris	"Prokaryotes;Bacteria;Gammaproteobacteria - Others;Shewanella"
1688	sbk	Shewanella benthica	"Prokaryotes;Bacteria;Gammaproteobacteria - Others;Shewanella"
1689	skh	Shewanella khirikhana	"Prokaryotes;Bacteria;Gammaproteobacteria - Others;Shewanella"
1690	saes	Shewanella aestuarii	"Prokaryotes;Bacteria;Gammaproteobacteria - Others;Shewanella"
1691	ilo	Idiomarina loihiensis L2TR	"Prokaryotes;Bacteria;Gammaproteobacteria - Others;Idiomarina"
1692	ili	Idiomarina loihiensis GSL 199	"Prokaryotes;Bacteria;Gammaproteobacteria - Others;Idiomarina"
1693	ipi	Idiomarina piscisalsi	"Prokaryotes;Bacteria;Gammaproteobacteria - Others;Idiomarina"
1694	idi	Idiomarina sp. X4	"Prokaryotes;Bacteria;Gammaproteobacteria - Others;Idiomarina"
1695	idt	Idiomarina sp. OT37-5b	"Prokaryotes;Bacteria;Gammaproteobacteria - Others;Idiomarina"
1696	cps	Colwellia psychrerythraea	"Prokaryotes;Bacteria;Gammaproteobacteria - Others;Colwellia"
1697	com	Colwellia sp. MT41	"Prokaryotes;Bacteria;Gammaproteobacteria - Others;Colwellia"
1698	coz	Colwellia sp. PAMC 20917	"Prokaryotes;Bacteria;Gammaproteobacteria - Others;Colwellia"
1699	colw	Colwellia sp. PAMC 21821	"Prokaryotes;Bacteria;Gammaproteobacteria - Others;Colwellia"
1700	cola	Colwellia sp. Arc7-D	"Prokaryotes;Bacteria;Gammaproteobacteria - Others;Colwellia"
1701	cber	Colwellia beringensis	"Prokaryotes;Bacteria;Gammaproteobacteria - Others;Colwellia"
1702	cov	Colwellia sp. Arc7-635	"Prokaryotes;Bacteria;Gammaproteobacteria - Others;Colwellia"
1703	lsd	Litorilituus sediminis	"Prokaryotes;Bacteria;Gammaproteobacteria - Others;Litorilituus"
1704	tht	Thalassotalea sp. HSM 43	"Prokaryotes;Bacteria;Gammaproteobacteria - Others;Thalassotalea"
1705	thap	Thalassotalea sp. PS06	"Prokaryotes;Bacteria;Gammaproteobacteria - Others;Thalassotalea"
1706	pha	Pseudoalteromonas translucida TAC125	"Prokaryotes;Bacteria;Gammaproteobacteria - Others;Pseudoalteromonas"
1707	ptn	Pseudoalteromonas translucida KMM 520	"Prokaryotes;Bacteria;Gammaproteobacteria - Others;Pseudoalteromonas"
1708	pat	Pseudoalteromonas atlantica	"Prokaryotes;Bacteria;Gammaproteobacteria - Others;Pseudoalteromonas"
1709	psm	Pseudoalteromonas sp. SM9913	"Prokaryotes;Bacteria;Gammaproteobacteria - Others;Pseudoalteromonas"
1710	pseo	Pseudoalteromonas piratica OCN003	"Prokaryotes;Bacteria;Gammaproteobacteria - Others;Pseudoalteromonas"
1711	pia	Pseudoalteromonas issachenkonii	"Prokaryotes;Bacteria;Gammaproteobacteria - Others;Pseudoalteromonas"

1712	pphe	Pseudoalteromonas phenolica	"Prokaryotes;Bacteria;Gammaproteobacteria - Others;Pseudoalteromonas"
1713	pbw	Pseudoalteromonas sp. Bsw20308	"Prokaryotes;Bacteria;Gammaproteobacteria - Others;Pseudoalteromonas"
1714	prr	Pseudoalteromonas rubra	"Prokaryotes;Bacteria;Gammaproteobacteria - Others;Pseudoalteromonas"
1715	plz	Pseudoalteromonas luteoviolacea	"Prokaryotes;Bacteria;Gammaproteobacteria - Others;Pseudoalteromonas"
1716	paln	Pseudoalteromonas aliena	"Prokaryotes;Bacteria;Gammaproteobacteria - Others;Pseudoalteromonas"
1717	ppis	Pseudoalteromonas piscicida	"Prokaryotes;Bacteria;Gammaproteobacteria - Others;Pseudoalteromonas"
1718	pea	Pseudoalteromonas espejiana	"Prokaryotes;Bacteria;Gammaproteobacteria - Others;Pseudoalteromonas"
1719	pspo	Pseudoalteromonas spongiae	"Prokaryotes;Bacteria;Gammaproteobacteria - Others;Pseudoalteromonas"
1720	part	Pseudoalteromonas arctica	"Prokaryotes;Bacteria;Gammaproteobacteria - Others;Pseudoalteromonas"
1721	ptu	Pseudoalteromonas tunicata	"Prokaryotes;Bacteria;Gammaproteobacteria - Others;Pseudoalteromonas"
1722	png	Pseudoalteromonas nigrifaciens	"Prokaryotes;Bacteria;Gammaproteobacteria - Others;Pseudoalteromonas"
1723	ptd	Pseudoalteromonas tetraodonis	"Prokaryotes;Bacteria;Gammaproteobacteria - Others;Pseudoalteromonas"
1724	psen	Pseudoalteromonas sp. NC201	"Prokaryotes;Bacteria;Gammaproteobacteria - Others;Pseudoalteromonas"
1725	pdj	Pseudoalteromonas donghaensis	"Prokaryotes;Bacteria;Gammaproteobacteria - Others;Pseudoalteromonas"
1726	paga	Pseudoalteromonas agarivorans	"Prokaryotes;Bacteria;Gammaproteobacteria - Others;Pseudoalteromonas"
1727	pcar	Pseudoalteromonas carrageenovora	"Prokaryotes;Bacteria;Gammaproteobacteria - Others;Pseudoalteromonas"
1728	pmaa	Pseudoalteromonas marina	"Prokaryotes;Bacteria;Gammaproteobacteria - Others;Pseudoalteromonas"
1729	maq	Marinobacter hydrocarbonoclasticus VT8	"Prokaryotes;Bacteria;Gammaproteobacteria - Others;Marinobacter"
1730	mhc	Marinobacter hydrocarbonoclasticus ATCC 49840	"Prokaryotes;Bacteria;Gammaproteobacteria - Others;Marinobacter"
1731	mad	Marinobacter adhaerens	"Prokaryotes;Bacteria;Gammaproteobacteria - Others;Marinobacter"
1732	mbs	Marinobacter sp. BSs20148	"Prokaryotes;Bacteria;Gammaproteobacteria - Others;Marinobacter"
1733	msr	Marinobacter salarius	"Prokaryotes;Bacteria;Gammaproteobacteria - Others;Marinobacter"
1734	msx	Marinobacter similis	"Prokaryotes;Bacteria;Gammaproteobacteria - Others;Marinobacter"
1735	mpq	Marinobacter psychrophilus	"Prokaryotes;Bacteria;Gammaproteobacteria - Others;Marinobacter"
1736	mari	Marinobacter sp. CP1	"Prokaryotes;Bacteria;Gammaproteobacteria - Others;Marinobacter"
1737	mlq	Marinobacter sp. LQ44	"Prokaryotes;Bacteria;Gammaproteobacteria - Others;Marinobacter"
1738	msq	Marinobacter salinus	"Prokaryotes;Bacteria;Gammaproteobacteria - Others;Marinobacter"
1739	mara	Marinobacter sp. Arc7-DN-1	"Prokaryotes;Bacteria;Gammaproteobacteria - Others;Marinobacter"
1740	marj	Marinobacter sp. JH2	"Prokaryotes;Bacteria;Gammaproteobacteria - Others;Marinobacter"
1741	amc	Alteromonas mediterranea DE	"Prokaryotes;Bacteria;Gammaproteobacteria - Others;Alteromonas"
1742	amh	Alteromonas mediterranea 615	"Prokaryotes;Bacteria;Gammaproteobacteria - Others;Alteromonas"
1743	amaa	Alteromonas mediterranea DE1	"Prokaryotes;Bacteria;Gammaproteobacteria - Others;Alteromonas"
1744	amal	Alteromonas mediterranea U4	"Prokaryotes;Bacteria;Gammaproteobacteria - Others;Alteromonas"
1745	amae	Alteromonas mediterranea U7	"Prokaryotes;Bacteria;Gammaproteobacteria - Others;Alteromonas"
1746	amao	Alteromonas mediterranea U8	"Prokaryotes;Bacteria;Gammaproteobacteria - Others;Alteromonas"
1747	amad	Alteromonas mediterranea UM4b	"Prokaryotes;Bacteria;Gammaproteobacteria - Others;Alteromonas"
1748	amai	Alteromonas mediterranea UM7	"Prokaryotes;Bacteria;Gammaproteobacteria - Others;Alteromonas"
1749	amag	Alteromonas mediterranea MED64	"Prokaryotes;Bacteria;Gammaproteobacteria - Others;Alteromonas"
1750	amac	Alteromonas macleodii ATCC 27126	"Prokaryotes;Bacteria;Gammaproteobacteria - Others;Alteromonas"
1751	amb	Alteromonas macleodii Balearic Sea AD45	"Prokaryotes;Bacteria;Gammaproteobacteria - Others;Alteromonas"
1752	amg	Alteromonas macleodii English Channel 673	"Prokaryotes;Bacteria;Gammaproteobacteria - Others;Alteromonas"

1753	amk	Alteromonas macleodii Black Sea 11	"Prokaryotes;Bacteria;Gammaproteobacteria - Others;Alteromonas"
1754	alt	Alteromonas naphthalenivorans	"Prokaryotes;Bacteria;Gammaproteobacteria - Others;Alteromonas"
1755	aal	Alteromonas australica H 17	"Prokaryotes;Bacteria;Gammaproteobacteria - Others;Alteromonas"
1756	aaus	Alteromonas australica DE170	"Prokaryotes;Bacteria;Gammaproteobacteria - Others;Alteromonas"
1757	asp	Alteromonas stellipolaris LMG 21856	"Prokaryotes;Bacteria;Gammaproteobacteria - Others;Alteromonas"
1758	asq	Alteromonas stellipolaris LMG 21861	"Prokaryotes;Bacteria;Gammaproteobacteria - Others;Alteromonas"
1759	aaw	Alteromonas addita	"Prokaryotes;Bacteria;Gammaproteobacteria - Others;Alteromonas"
1760	alr	Alteromonas sp. RKMC-009	"Prokaryotes;Bacteria;Gammaproteobacteria - Others;Alteromonas"
1761	ale	Alteromonas sp. Mac1	"Prokaryotes;Bacteria;Gammaproteobacteria - Others;Alteromonas"
1762	alz	Alteromonas sp. Mac2	"Prokaryotes;Bacteria;Gammaproteobacteria - Others;Alteromonas"
1763	apel	Alteromonas pelagimontana	"Prokaryotes;Bacteria;Gammaproteobacteria - Others;Alteromonas"
1764	gag	Glaciecola sp. 4H-3-7+YE-5	"Prokaryotes;Bacteria;Gammaproteobacteria - Others;Glaciecola"
1765	gni	Glaciecola nitratireducens	"Prokaryotes;Bacteria;Gammaproteobacteria - Others;Glaciecola"
1766	gps	Paraglaciecola psychrophila	"Prokaryotes;Bacteria;Gammaproteobacteria - Others;Paraglaciecola"
1767	pmes	Paraglaciecola mesophila	"Prokaryotes;Bacteria;Gammaproteobacteria - Others;Paraglaciecola"
1768	lal	Lacimicrobium alkaliphilum	"Prokaryotes;Bacteria;Gammaproteobacteria - Others;Lacimicrobium"
1769	cate	Catenovulum sp. CCB-QB4	"Prokaryotes;Bacteria;Gammaproteobacteria - Others;Catenovulum"
1770	salh	Saliniradius amylolyticus	"Prokaryotes;Bacteria;Gammaproteobacteria - Others;Saliniradius"
1771	salm	Salinimonas sediminis	"Prokaryotes;Bacteria;Gammaproteobacteria - Others;Salinimonas"
1772	salk	Salinimonas sp. KX18D6	"Prokaryotes;Bacteria;Gammaproteobacteria - Others;Salinimonas"
1773	hmi	Hydrocarboniclastica marina	"Prokaryotes;Bacteria;Gammaproteobacteria - Others;Hydrocarboniclastica"
1774	pin	Psychromonas ingrahamii	"Prokaryotes;Bacteria;Gammaproteobacteria - Others;Psychromonas"
1775	psy	Psychromonas sp. CNPT3	"Prokaryotes;Bacteria;Gammaproteobacteria - Others;Psychromonas"
1776	fbl	Ferrimonas balearica	"Prokaryotes;Bacteria;Gammaproteobacteria - Others;Ferrimonas"
1777	fes	Ferrimonas lipolytica	"Prokaryotes;Bacteria;Gammaproteobacteria - Others;Ferrimonas"
1778	mvs	Moritella viscosa	"Prokaryotes;Bacteria;Gammaproteobacteria - Others;Moritella"
1779	mya	Moritella yayanosii	"Prokaryotes;Bacteria;Gammaproteobacteria - Others;Moritella"
1780	mmaa	Moritella marina	"Prokaryotes;Bacteria;Gammaproteobacteria - Others;Moritella"
1781	cja	Cellvibrio japonicus	"Prokaryotes;Bacteria;Gammaproteobacteria - Others;Cellvibrio"
1782	ceb	Cellvibrio sp. PSBB023	"Prokaryotes;Bacteria;Gammaproteobacteria - Others;Cellvibrio"
1783	cell	Cellvibrio sp. PSBB006	"Prokaryotes;Bacteria;Gammaproteobacteria - Others;Cellvibrio"
1784	cek	Cellvibrio sp. KY-YJ-3	"Prokaryotes;Bacteria;Gammaproteobacteria - Others;Cellvibrio"
1785	ceg	Cellvibrio sp. KY-GH-1	"Prokaryotes;Bacteria;Gammaproteobacteria - Others;Cellvibrio"
1786	sde	Saccharophagus degradans	"Prokaryotes;Bacteria;Gammaproteobacteria - Others;Saccharophagus"
1787	ttu	Teredinibacter turnerae	"Prokaryotes;Bacteria;Gammaproteobacteria - Others;Teredinibacter"
1788	saga	Simiduia agarivorans	"Prokaryotes;Bacteria;Gammaproteobacteria - Others;Simiduia"
1789	spoi	Spongiibacter sp. IMCC21906	"Prokaryotes;Bacteria;Gammaproteobacteria - Others;Spongiibacter"
1790	zal	Zhongshania aliphaticivorans	"Prokaryotes;Bacteria;Gammaproteobacteria - Others;Zhongshania"
1791	osg	Oceanicoccus sagamiensis	"Prokaryotes;Bacteria;Gammaproteobacteria - Others;Oceanicoccus"
1792	mthd	Microbulbifer thermotolerans	"Prokaryotes;Bacteria;Gammaproteobacteria - Others;Microbulbifer"
1793	micc	Microbulbifer aggregans	"Prokaryotes;Bacteria;Gammaproteobacteria - Others;Microbulbifer"

1794	maga	Microbulbifer agarilyticus	"Prokaryotes;Bacteria;Gammaproteobacteria - Others;Microbulbifer"
1795	mii	Microbulbifer sp. A4B17	"Prokaryotes;Bacteria;Gammaproteobacteria - Others;Microbulbifer"
1796	mict	Microbulbifer sp. THAF38	"Prokaryotes;Bacteria;Gammaproteobacteria - Others;Microbulbifer"
1797	mhyd	Microbulbifer hydrolyticus	"Prokaryotes;Bacteria;Gammaproteobacteria - Others;Microbulbifer"
1798	hja	Halioglobus japonicus	"Prokaryotes;Bacteria;Gammaproteobacteria - Others;Halioglobus"
1799	halc	Halioglobus maricola	"Prokaryotes;Bacteria;Gammaproteobacteria - Others;Halioglobus"
1800	kim	Kineobactrum salinum	"Prokaryotes;Bacteria;Gammaproteobacteria - Others;Kineobactrum"
1801	cbu	Coxiella burnetii RSA 493	"Prokaryotes;Bacteria;Gammaproteobacteria - Others;Coxiella"
1802	cbs	Coxiella burnetii RSA 331	"Prokaryotes;Bacteria;Gammaproteobacteria - Others;Coxiella"
1803	cbd	Coxiella burnetii Dugway 5J108-111	"Prokaryotes;Bacteria;Gammaproteobacteria - Others;Coxiella"
1804	cbg	Coxiella burnetii CbuG_Q212	"Prokaryotes;Bacteria;Gammaproteobacteria - Others;Coxiella"
1805	cbc	Coxiella burnetii CbuK_Q154	"Prokaryotes;Bacteria;Gammaproteobacteria - Others;Coxiella"
1806	cey	Coxiella mudrowiae	"Prokaryotes;Bacteria;Gammaproteobacteria - Others;Coxiella"
1807	cea	Coxiella endosymbiont of Amblyomma americanum	"Prokaryotes;Bacteria;Gammaproteobacteria - Others;Coxiella"
1808	cend	Coxiella endosymbiont of Amblyomma sculptum	"Prokaryotes;Bacteria;Gammaproteobacteria - Others;Coxiella"
1809	rvi	Candidatus Rickettsiella viridis	"Prokaryotes;Bacteria;Gammaproteobacteria - Others;Rickettsiella"
1810	alg	Aquicella lusitana	"Prokaryotes;Bacteria;Gammaproteobacteria - Others;Aquicella"
1811	asip	Aquicella siphonis	"Prokaryotes;Bacteria;Gammaproteobacteria - Others;Aquicella"
1812	lpn	Legionella pneumophila subsp. pneumophila Philadelphia 1 (serogroup 1)	"Prokaryotes;Bacteria;Gammaproteobacteria - Others;Legionella"
1813	lph	Legionella pneumophila subsp. pneumophila HL06041035 (serogroup 1)	"Prokaryotes;Bacteria;Gammaproteobacteria - Others;Legionella"
1814	lpo	Legionella pneumophila subsp. pneumophila Lorraine (serogroup 1)	"Prokaryotes;Bacteria;Gammaproteobacteria - Others;Legionella"
1815	lpu	Legionella pneumophila subsp. pneumophila LPE509	"Prokaryotes;Bacteria;Gammaproteobacteria - Others;Legionella"
1816	lpm	Legionella pneumophila subsp. pneumophila Thunder Bay	"Prokaryotes;Bacteria;Gammaproteobacteria - Others;Legionella"
1817	lpf	Legionella pneumophila Lens (serogroup 1)	"Prokaryotes;Bacteria;Gammaproteobacteria - Others;Legionella"
1818	lpp	Legionella pneumophila Paris (serogroup 1)	"Prokaryotes;Bacteria;Gammaproteobacteria - Others;Legionella"
1819	lpc	Legionella pneumophila Corby	"Prokaryotes;Bacteria;Gammaproteobacteria - Others;Legionella"
1820	lpa	Legionella pneumophila 2300/99 Alcoy (serogroup 1)	"Prokaryotes;Bacteria;Gammaproteobacteria - Others;Legionella"
1821	lpe	Legionella pneumophila subsp. pneumophila ATCC 43290 (serogroup 12)	"Prokaryotes;Bacteria;Gammaproteobacteria - Others;Legionella"
1822	llo	Legionella longbeachae	"Prokaryotes;Bacteria;Gammaproteobacteria - Others;Legionella"
1823	lfa	Legionella fallonii	"Prokaryotes;Bacteria;Gammaproteobacteria - Others;Legionella"
1824	lha	Legionella hackeliae	"Prokaryotes;Bacteria;Gammaproteobacteria - Others;Legionella"
1825	lok	Legionella oakridgensis	"Prokaryotes;Bacteria;Gammaproteobacteria - Others;Legionella"
1826	lcd	Legionella clemsonensis	"Prokaryotes;Bacteria;Gammaproteobacteria - Others;Legionella"
1827	les	Legionella endosymbiont of Polyplax serrata	"Prokaryotes;Bacteria;Gammaproteobacteria - Others;Legionella"
1828	lsh	Legionella sainthelensi	"Prokaryotes;Bacteria;Gammaproteobacteria - Others;Legionella"
1829	llg	Legionella lansingensis	"Prokaryotes;Bacteria;Gammaproteobacteria - Others;Legionella"
1830	lib	Legionella israelensis	"Prokaryotes;Bacteria;Gammaproteobacteria - Others;Legionella"
1831	lgt	Legionella geestiana	"Prokaryotes;Bacteria;Gammaproteobacteria - Others;Legionella"
1832	ljr	Legionella jordanis	"Prokaryotes;Bacteria;Gammaproteobacteria - Others;Legionella"

1833	lcj	Legionella cherrii	"Prokaryotes;Bacteria;Gammaproteobacteria - Others;Legionella"
1834	lwa	Legionella waltersii	"Prokaryotes;Bacteria;Gammaproteobacteria - Others;Legionella"
1835	lss	Legionella spiritensis	"Prokaryotes;Bacteria;Gammaproteobacteria - Others;Legionella"
1836	tmc	Tatlockia micdadei	"Prokaryotes;Bacteria;Gammaproteobacteria - Others;Tatlockia"
1837	mca	Methylococcus capsulatus	"Prokaryotes;Bacteria;Gammaproteobacteria - Others;Methylococcus"
1838	metu	Methylococcus sp. IM1	"Prokaryotes;Bacteria;Gammaproteobacteria - Others;Methylococcus"
1839	mmt	Methylomonas methanica	"Prokaryotes;Bacteria;Gammaproteobacteria - Others;Methylomonas"
1840	mdn	Methylomonas denitrificans	"Prokaryotes;Bacteria;Gammaproteobacteria - Others;Methylomonas"
1841	mdh	Methylomonas sp. DH-1	"Prokaryotes;Bacteria;Gammaproteobacteria - Others;Methylomonas"
1842	mko	Methylomonas koyamae	"Prokaryotes;Bacteria;Gammaproteobacteria - Others;Methylomonas"
1843	metl	Methylomonas sp. LW13	"Prokaryotes;Bacteria;Gammaproteobacteria - Others;Methylomonas"
1844	mah	Methylotuvimicrobium alcaliphilum	"Prokaryotes;Bacteria;Gammaproteobacteria - Others;Methylotuvimicrobium"
1845	mbur	Methylotuvimicrobium buryatense	"Prokaryotes;Bacteria;Gammaproteobacteria - Others;Methylotuvimicrobium"
1846	mpsy	Methylovulum psychrotolerans	"Prokaryotes;Bacteria;Gammaproteobacteria - Others;Methylovulum"
1847	mmai	Methylocaldum marinum	"Prokaryotes;Bacteria;Gammaproteobacteria - Others;Methylocaldum"
1848	mmob	Candidatus Methylospira mobilis	"Prokaryotes;Bacteria;Gammaproteobacteria - Others;Methylospira"
1849	mein	Methyloprofundus sp. INp10_methR40d	"Prokaryotes;Bacteria;Gammaproteobacteria - Others;Methyloprofundus"
1850	ftu	Francisella tularensis subsp. tularensis SCHU S4	"Prokaryotes;Bacteria;Gammaproteobacteria - Others;Francisella"
1851	ftq	Francisella tularensis subsp. tularensis SCHU S4 NR-28534	"Prokaryotes;Bacteria;Gammaproteobacteria - Others;Francisella"
1852	ftf	Francisella tularensis subsp. tularensis FSC198	"Prokaryotes;Bacteria;Gammaproteobacteria - Others;Francisella"
1853	ftw	Francisella tularensis subsp. tularensis WY96-3418	"Prokaryotes;Bacteria;Gammaproteobacteria - Others;Francisella"
1854	ftr	Francisella tularensis subsp. tularensis NE061598	"Prokaryotes;Bacteria;Gammaproteobacteria - Others;Francisella"
1855	ftt	Francisella tularensis subsp. tularensis TI0902	"Prokaryotes;Bacteria;Gammaproteobacteria - Others;Francisella"
1856	ftg	Francisella tularensis subsp. tularensis TIGB03	"Prokaryotes;Bacteria;Gammaproteobacteria - Others;Francisella"
1857	ftl	Francisella tularensis subsp. holarctica LVS	"Prokaryotes;Bacteria;Gammaproteobacteria - Others;Francisella"
1858	fth	Francisella tularensis subsp. holarctica OSU18	"Prokaryotes;Bacteria;Gammaproteobacteria - Others;Francisella"
1859	fta	Francisella tularensis subsp. holarctica FTNF002-00	"Prokaryotes;Bacteria;Gammaproteobacteria - Others;Francisella"
1860	fts	Francisella tularensis subsp. holarctica F92	"Prokaryotes;Bacteria;Gammaproteobacteria - Others;Francisella"
1861	fti	Francisella tularensis subsp. holarctica FSC200	"Prokaryotes;Bacteria;Gammaproteobacteria - Others;Francisella"
1862	fto	Francisella tularensis subsp. holarctica PHIT-FT049	"Prokaryotes;Bacteria;Gammaproteobacteria - Others;Francisella"
1863	ftc	Francisella tularensis subsp. holarctica FTT_1	"Prokaryotes;Bacteria;Gammaproteobacteria - Others;Francisella"
1864	ftv	Francisella tularensis subsp. holarctica VT68	"Prokaryotes;Bacteria;Gammaproteobacteria - Others;Francisella"
1865	ftz	Francisella tularensis subsp. holarctica 425	"Prokaryotes;Bacteria;Gammaproteobacteria - Others;Francisella"
1866	ftm	Francisella tularensis subsp. mediasiatica FSC147	"Prokaryotes;Bacteria;Gammaproteobacteria - Others;Francisella"
1867	ftn	Francisella tularensis subsp. novicida U112	"Prokaryotes;Bacteria;Gammaproteobacteria - Others;Francisella"
1868	ftx	Francisella tularensis subsp. novicida U112	"Prokaryotes;Bacteria;Gammaproteobacteria - Others;Francisella"
1869	ftd	Francisella tularensis subsp. novicida F6168	"Prokaryotes;Bacteria;Gammaproteobacteria - Others;Francisella"
1870	fty	Francisella tularensis subsp. novicida DPG 3A-IS	"Prokaryotes;Bacteria;Gammaproteobacteria - Others;Francisella"
1871	fcf	Francisella cf. novicida Fx1	"Prokaryotes;Bacteria;Gammaproteobacteria - Others;Francisella"
1872	fcn	Francisella hispaniensis 3523	"Prokaryotes;Bacteria;Gammaproteobacteria - Others;Francisella"
1873	fhi	Francisella hispaniensis FSC454	"Prokaryotes;Bacteria;Gammaproteobacteria - Others;Francisella"

1874	fph	Francisella philomiragia subsp. philomiragia ATCC 25017	"Prokaryotes;Bacteria;Gammaproteobacteria - Others;Francisella"
1875	fpt	Francisella philomiragia subsp. philomiragia ATCC 25015 O#319L	"Prokaryotes;Bacteria;Gammaproteobacteria - Others;Francisella"
1876	fpi	Francisella philomiragia O#319-029	"Prokaryotes;Bacteria;Gammaproteobacteria - Others;Francisella"
1877	fpm	Francisella philomiragia O#319-036	"Prokaryotes;Bacteria;Gammaproteobacteria - Others;Francisella"
1878	fpx	Francisella philomiragia O#319-067	"Prokaryotes;Bacteria;Gammaproteobacteria - Others;Francisella"
1879	fpz	Francisella philomiragia GA01-2794	"Prokaryotes;Bacteria;Gammaproteobacteria - Others;Francisella"
1880	fpj	Francisella philomiragia GA01-2801	"Prokaryotes;Bacteria;Gammaproteobacteria - Others;Francisella"
1881	frt	Francisella salina	"Prokaryotes;Bacteria;Gammaproteobacteria - Others;Francisella"
1882	fna	Francisella orientalis Toba 04	"Prokaryotes;Bacteria;Gammaproteobacteria - Others;Francisella"
1883	fnl	Francisella orientalis LADL--07-285A	"Prokaryotes;Bacteria;Gammaproteobacteria - Others;Francisella"
1884	frf	Francisella sp. FSC1006	"Prokaryotes;Bacteria;Gammaproteobacteria - Others;Francisella"
1885	fper	Francisella persica	"Prokaryotes;Bacteria;Gammaproteobacteria - Others;Francisella"
1886	fha	Francisella halioticida	"Prokaryotes;Bacteria;Gammaproteobacteria - Others;Francisella"
1887	frx	Francisella uliginis	"Prokaryotes;Bacteria;Gammaproteobacteria - Others;Francisella"
1888	frm	Francisella sp. MA067296	"Prokaryotes;Bacteria;Gammaproteobacteria - Others;Francisella"
1889	frc	Francisella frigiditurris	"Prokaryotes;Bacteria;Gammaproteobacteria - Others;Francisella"
1890	fad	Francisella adeliensis	"Prokaryotes;Bacteria;Gammaproteobacteria - Others;Francisella"
1891	fmi	Francisella marina	"Prokaryotes;Bacteria;Gammaproteobacteria - Others;Francisella"
1892	foo	Francisella opportunistica	"Prokaryotes;Bacteria;Gammaproteobacteria - Others;Francisella"
1893	fgu	Allofrancisella guangzhouensis	"Prokaryotes;Bacteria;Gammaproteobacteria - Others;Allofrancisella"
1894	afri	Allofrancisella frigidaquae	"Prokaryotes;Bacteria;Gammaproteobacteria - Others;Allofrancisella"
1895	aii	Allofrancisella inopinata	"Prokaryotes;Bacteria;Gammaproteobacteria - Others;Allofrancisella"
1896	tcx	Hydrogenovibrio crunogenus	"Prokaryotes;Bacteria;Gammaproteobacteria - Others;Hydrogenovibrio"
1897	htr	Hydrogenovibrio thermophilus	"Prokaryotes;Bacteria;Gammaproteobacteria - Others;Hydrogenovibrio"
1898	hmar	Hydrogenovibrio marinus	"Prokaryotes;Bacteria;Gammaproteobacteria - Others;Hydrogenovibrio"
1899	tcy	Thiomicrospira cyclica	"Prokaryotes;Bacteria;Gammaproteobacteria - Others;Thiomicrospira"
1900	tao	Thiomicrospira aerophila	"Prokaryotes;Bacteria;Gammaproteobacteria - Others;Thiomicrospira"
1901	thio	Thiomicrospira sp. S5	"Prokaryotes;Bacteria;Gammaproteobacteria - Others;Thiomicrospira"
1902	mej	Methylophaga nitratireducenticrescens	"Prokaryotes;Bacteria;Gammaproteobacteria - Others;Methylophaga"
1903	mec	Methylophaga frappieri	"Prokaryotes;Bacteria;Gammaproteobacteria - Others;Methylophaga"
1904	cyq	Cycloclasticus sp. P1	"Prokaryotes;Bacteria;Gammaproteobacteria - Others;Cycloclasticus"
1905	cza	Cycloclasticus zancles	"Prokaryotes;Bacteria;Gammaproteobacteria - Others;Cycloclasticus"
1906	cyy	Cycloclasticus sp. PY97N	"Prokaryotes;Bacteria;Gammaproteobacteria - Others;Cycloclasticus"
1907	psal	Piscirickettsia salmonis	"Prokaryotes;Bacteria;Gammaproteobacteria - Others;Piscirickettsia"
1908	thig	Thiomicrorhabdus sediminis	"Prokaryotes;Bacteria;Gammaproteobacteria - Others;Thiomicrorhabdus"
1909	tse	Thiosulfatimonas sediminis	"Prokaryotes;Bacteria;Gammaproteobacteria - Others;Thiosulfatimonas"
1910	tzo	Thiosulfativibrio zosterae	"Prokaryotes;Bacteria;Gammaproteobacteria - Others;Thiosulfativibrio"
1911	tig	Thioploca ingrica	"Prokaryotes;Bacteria;Gammaproteobacteria - Others;Thioploca"
1912	blep	Beggiatoa leptomitoformis	"Prokaryotes;Bacteria;Gammaproteobacteria - Others;Beggiatoa"
1913	this	Candidatus Thiothrix singaporensis	"Prokaryotes;Bacteria;Gammaproteobacteria - Others;Thiothrix"
1914	noc	Nitrosococcus oceani	"Prokaryotes;Bacteria;Gammaproteobacteria - Others;Nitrosococcus"

1915	nhl	Nitrosococcus halophilus	"Prokaryotes;Bacteria;Gammaproteobacteria - Others;Nitrosococcus"
1916	nwa	Nitrosococcus watsonii	"Prokaryotes;Bacteria;Gammaproteobacteria - Others;Nitrosococcus"
1917	nwr	Nitrosococcus wardiae	"Prokaryotes;Bacteria;Gammaproteobacteria - Others;Nitrosococcus"
1918	alv	Allochromatium vinosum	"Prokaryotes;Bacteria;Gammaproteobacteria - Others;Allochromatium"
1919	tvi	Thiocystis violascens	"Prokaryotes;Bacteria;Gammaproteobacteria - Others;Thiocystis"
1920	tmb	Thioflavicoccus mobilis	"Prokaryotes;Bacteria;Gammaproteobacteria - Others;Thioflavicoccus"
1921	mpur	Marichromatium purpuratum	"Prokaryotes;Bacteria;Gammaproteobacteria - Others;Marichromatium"
1922	tee	Candidatus Tenderia electrophaga	"Prokaryotes;Bacteria;Gammaproteobacteria - Others;Tenderia"
1923	ntt	Candidatus Nitrosoglobus terrae	"Prokaryotes;Bacteria;Gammaproteobacteria - Others;Nitrosoglobus"
1924	tsy	Candidatus Thiodictyon syntrophicum	"Prokaryotes;Bacteria;Gammaproteobacteria - Others;Thiodictyon"
1925	rhh	Rheinheimera sp. D18	"Prokaryotes;Bacteria;Gammaproteobacteria - Others;Rheinheimera"
1926	ttp	Thermochromatium tepidum	"Prokaryotes;Bacteria;Gammaproteobacteria - Others;Thermochromatium"
1927	ntg	Candidatus Nitrosacidococcus tergens	"Prokaryotes;Bacteria;Gammaproteobacteria - Others;Nitrosacidococcus"
1928	thip	Thiohalocapsa sp. PB-PSB1	"Prokaryotes;Bacteria;Gammaproteobacteria - Others;Thiohalocapsa"
1929	aeh	Alkalilimnicola ehrlichii	"Prokaryotes;Bacteria;Gammaproteobacteria - Others;Alkalilimnicola"
1930	hha	Halorhodospira halophila	"Prokaryotes;Bacteria;Gammaproteobacteria - Others;Halorhodospira"
1931	hhk	Halorhodospira halochloris	"Prokaryotes;Bacteria;Gammaproteobacteria - Others;Halorhodospira"
1932	hhc	Ectothiorhodospira haloalkaliphila	"Prokaryotes;Bacteria;Gammaproteobacteria - Others;Ectothiorhodospira"
1933	ebs	Ectothiorhodospira sp. BSL-9	"Prokaryotes;Bacteria;Gammaproteobacteria - Others;Ectothiorhodospira"
1934	tgr	Thioalkalivibrio sulfidiphilus	"Prokaryotes;Bacteria;Gammaproteobacteria - Others;Thioalkalivibrio"
1935	tkm	Thioalkalivibrio sp. K90mix	"Prokaryotes;Bacteria;Gammaproteobacteria - Others;Thioalkalivibrio"
1936	tni	Thioalkalivibrio nitratireducens	"Prokaryotes;Bacteria;Gammaproteobacteria - Others;Thioalkalivibrio"
1937	tti	Thioalkalivibrio paradoxus	"Prokaryotes;Bacteria;Gammaproteobacteria - Others;Thioalkalivibrio"
1938	tvr	Thioalkalivibrio versutus	"Prokaryotes;Bacteria;Gammaproteobacteria - Others;Thioalkalivibrio"
1939	ssal	Spiribacter salinus	"Prokaryotes;Bacteria;Gammaproteobacteria - Others;Spiribacter"
1940	spiu	Spiribacter curvatus	"Prokaryotes;Bacteria;Gammaproteobacteria - Others;Spiribacter"
1941	sros	Spiribacter roseus	"Prokaryotes;Bacteria;Gammaproteobacteria - Others;Spiribacter"
1942	spiz	Spiribacter sp. 2438	"Prokaryotes;Bacteria;Gammaproteobacteria - Others;Spiribacter"
1943	aprs	Acidihalobacter prosperus	"Prokaryotes;Bacteria;Gammaproteobacteria - Others;Acidihalobacter"
1944	hna	Halothiobacillus neapolitanus	"Prokaryotes;Bacteria;Gammaproteobacteria - Others;Halothiobacillus"
1945	haz	Halothiobacillus sp. LS2	"Prokaryotes;Bacteria;Gammaproteobacteria - Others;Halothiobacillus"
1946	wma	Wenzhouxiangella marina	"Prokaryotes;Bacteria;Gammaproteobacteria - Others;Wenzhouxiangella"
1947	woc	Woeseia oceani	"Prokaryotes;Bacteria;Gammaproteobacteria - Others;Woeseia"
1948	gai	Granulosicoccus antarcticus	"Prokaryotes;Bacteria;Gammaproteobacteria - Others;Granulosicoccus"
1949	ghl	Guyparkeria halophila	"Prokaryotes;Bacteria;Gammaproteobacteria - Others;Guyparkeria"
1950	ttc	Thiohalobacter thiocyanaticus	"Prokaryotes;Bacteria;Gammaproteobacteria - Others;Thiohalobacter"
1951	hch	Hahella chejuensis	"Prokaryotes;Bacteria;Gammaproteobacteria - Others;Hahella"
1952	hahe	Hahella sp. KA22	"Prokaryotes;Bacteria;Gammaproteobacteria - Others;Hahella"
1953	csa	Chromohalobacter salexigens	"Prokaryotes;Bacteria;Gammaproteobacteria - Others;Chromohalobacter"
1954	hel	Halomonas elongata	"Prokaryotes;Bacteria;Gammaproteobacteria - Others;Halomonas"
1955	hcs	Halomonas campaniensis	"Prokaryotes;Bacteria;Gammaproteobacteria - Others;Halomonas"

1956	hak	Halomonas sp. KO116	"Prokaryotes;Bacteria;Gammaproteobacteria - Others;Halomonas"
1957	ham	Halomonas sp. R57-5	"Prokaryotes;Bacteria;Gammaproteobacteria - Others;Halomonas"
1958	hhu	Halomonas huangheensis	"Prokaryotes;Bacteria;Gammaproteobacteria - Others;Halomonas"
1959	hco	Halomonas chromatireducens	"Prokaryotes;Bacteria;Gammaproteobacteria - Others;Halomonas"
1960	hsi	Halomonas aestuarii	"Prokaryotes;Bacteria;Gammaproteobacteria - Others;Halomonas"
1961	halo	Halomonas sp. 1513	"Prokaryotes;Bacteria;Gammaproteobacteria - Others;Halomonas"
1962	hhh	Halomonas hydrothermalis	"Prokaryotes;Bacteria;Gammaproteobacteria - Others;Halomonas"
1963	hbe	Halomonas beimenensis	"Prokaryotes;Bacteria;Gammaproteobacteria - Others;Halomonas"
1964	hag	Halomonas sp. GFAJ-1	"Prokaryotes;Bacteria;Gammaproteobacteria - Others;Halomonas"
1965	haf	Halomonas sp. SF2003	"Prokaryotes;Bacteria;Gammaproteobacteria - Others;Halomonas"
1966	halk	Halomonas alkaliphila	"Prokaryotes;Bacteria;Gammaproteobacteria - Others;Halomonas"
1967	hvn	Halomonas venusta	"Prokaryotes;Bacteria;Gammaproteobacteria - Others;Halomonas"
1968	hol	Halomonas olivaria	"Prokaryotes;Bacteria;Gammaproteobacteria - Others;Halomonas"
1969	hsr	Halomonas sulfidaeris	"Prokaryotes;Bacteria;Gammaproteobacteria - Others;Halomonas"
1970	hmd	Halomonas meridiana	"Prokaryotes;Bacteria;Gammaproteobacteria - Others;Halomonas"
1971	haxi	Halomonas axialensis	"Prokaryotes;Bacteria;Gammaproteobacteria - Others;Halomonas"
1972	htt	Halomonas titanicae	"Prokaryotes;Bacteria;Gammaproteobacteria - Others;Halomonas"
1973	hcam	Halomonas campisalis	"Prokaryotes;Bacteria;Gammaproteobacteria - Others;Halomonas"
1974	hpiz	Halomonas piezotolerans	"Prokaryotes;Bacteria;Gammaproteobacteria - Others;Halomonas"
1975	ple	Candidatus Portiera aleyrodidarum BT-B-HRs	"Prokaryotes;Bacteria;Gammaproteobacteria - Others;Portiera"
1976	ply	Candidatus Portiera aleyrodidarum BT-B-HRs	"Prokaryotes;Bacteria;Gammaproteobacteria - Others;Portiera"
1977	plr	Candidatus Portiera aleyrodidarum BT-QVLC	"Prokaryotes;Bacteria;Gammaproteobacteria - Others;Portiera"
1978	plo	Candidatus Portiera aleyrodidarum BT-QVLC	"Prokaryotes;Bacteria;Gammaproteobacteria - Others;Portiera"
1979	pld	Candidatus Portiera aleyrodidarum TV	"Prokaryotes;Bacteria;Gammaproteobacteria - Others;Portiera"
1980	plb	Candidatus Portiera aleyrodidarum TV-BCN	"Prokaryotes;Bacteria;Gammaproteobacteria - Others;Portiera"
1981	plc	Candidatus Portiera aleyrodidarum AD-CAI	"Prokaryotes;Bacteria;Gammaproteobacteria - Others;Portiera"
1982	pli	Candidatus Portiera aleyrodidarum AF-CAI	"Prokaryotes;Bacteria;Gammaproteobacteria - Others;Portiera"
1983	paly	Candidatus Portiera aleyrodidarum MED (Bemisia tabaci)	"Prokaryotes;Bacteria;Gammaproteobacteria - Others;Portiera"
1984	crp	Candidatus Carsonella ruddii PV	"Prokaryotes;Bacteria;Gammaproteobacteria - Others;Carsonella"
1985	cru	Candidatus Carsonella ruddii CE	"Prokaryotes;Bacteria;Gammaproteobacteria - Others;Carsonella"
1986	crc	Candidatus Carsonella ruddii CS	"Prokaryotes;Bacteria;Gammaproteobacteria - Others;Carsonella"
1987	crt	Candidatus Carsonella ruddii HT	"Prokaryotes;Bacteria;Gammaproteobacteria - Others;Carsonella"
1988	crh	Candidatus Carsonella ruddii HC	"Prokaryotes;Bacteria;Gammaproteobacteria - Others;Carsonella"
1989	crv	Candidatus Carsonella ruddii PC	"Prokaryotes;Bacteria;Gammaproteobacteria - Others;Carsonella"
1990	cri	Candidatus Carsonella ruddii DC	"Prokaryotes;Bacteria;Gammaproteobacteria - Others;Carsonella"
1991	eme	Candidatus Evansia muelleri	"Prokaryotes;Bacteria;Gammaproteobacteria - Others;Evansia"
1992	zpl	Zymobacter palmae	"Prokaryotes;Bacteria;Gammaproteobacteria - Others;Zymobacter"
1993	haa	Halotalea alkalilenta	"Prokaryotes;Bacteria;Gammaproteobacteria - Others;Halotalea"
1994	cmai	Cobetia marina	"Prokaryotes;Bacteria;Gammaproteobacteria - Others;Cobetia"
1995	kus	Kushneria konosiri	"Prokaryotes;Bacteria;Gammaproteobacteria - Others;Kushneria"
1996	kma	Kushneria marisflavi	"Prokaryotes;Bacteria;Gammaproteobacteria - Others;Kushneria"

1997	kuy	Kushneria phosphatilytica	"Prokaryotes;Bacteria;Gammaproteobacteria - Others;Kushneria"
1998	paur	Pistricoccus aurantiacus	"Prokaryotes;Bacteria;Gammaproteobacteria - Others;Pistricoccus"
1999	abo	Alcanivorax borkumensis	"Prokaryotes;Bacteria;Gammaproteobacteria - Others;Alcanivorax"
2000	adi	Alcanivorax dieselolei	"Prokaryotes;Bacteria;Gammaproteobacteria - Others;Alcanivorax"
2001	apac	Alcanivorax pacificus	"Prokaryotes;Bacteria;Gammaproteobacteria - Others;Alcanivorax"
2002	aln	Alcanivorax sp. NBRC 101098	"Prokaryotes;Bacteria;Gammaproteobacteria - Others;Alcanivorax"
2003	axe	Alcanivorax xenomutans	"Prokaryotes;Bacteria;Gammaproteobacteria - Others;Alcanivorax"
2004	kak	Ketobacter alkanivorans	"Prokaryotes;Bacteria;Gammaproteobacteria - Others;Ketobacter"
2005	kko	Kangiella koreensis	"Prokaryotes;Bacteria;Gammaproteobacteria - Others;Kangiella"
2006	kge	Kangiella geojedonensis	"Prokaryotes;Bacteria;Gammaproteobacteria - Others;Kangiella"
2007	ksd	Kangiella sediminilitoris	"Prokaryotes;Bacteria;Gammaproteobacteria - Others;Kangiella"
2008	kpd	Kangiella profundi	"Prokaryotes;Bacteria;Gammaproteobacteria - Others;Kangiella"
2009	mmw	Marinomonas sp. MWYL1	"Prokaryotes;Bacteria;Gammaproteobacteria - Others;Marinomonas"
2010	mme	Marinomonas mediterranea	"Prokaryotes;Bacteria;Gammaproteobacteria - Others;Marinomonas"
2011	mpc	Marinomonas posidonica	"Prokaryotes;Bacteria;Gammaproteobacteria - Others;Marinomonas"
2012	mpri	Marinomonas primoryensis	"Prokaryotes;Bacteria;Gammaproteobacteria - Others;Marinomonas"
2013	mard	Marinomonas arctica	"Prokaryotes;Bacteria;Gammaproteobacteria - Others;Marinomonas"
2014	tol	Thalassolituus oleivorans MIL-1	"Prokaryotes;Bacteria;Gammaproteobacteria - Others;Thalassolituus"
2015	tor	Thalassolituus oleivorans R6-15	"Prokaryotes;Bacteria;Gammaproteobacteria - Others;Thalassolituus"
2016	oai	Oleispira antarctica	"Prokaryotes;Bacteria;Gammaproteobacteria - Others;Oleispira"
2017	mars	Marinobacterium aestuarii	"Prokaryotes;Bacteria;Gammaproteobacteria - Others;Marinobacterium"
2018	bsan	Bacterioplanes sanyensis	"Prokaryotes;Bacteria;Gammaproteobacteria - Others;Bacterioplanes"
2019	ncu	Neptunomonas concharum	"Prokaryotes;Bacteria;Gammaproteobacteria - Others;Neptunomonas"
2020	nik	Nitrincola sp. KXZD1103	"Prokaryotes;Bacteria;Gammaproteobacteria - Others;Nitrincola"
2021	bmar	Bermanella marisrubri	"Prokaryotes;Bacteria;Gammaproteobacteria - Others;Bermanella"
2022	ajp	Amphritea japonica	"Prokaryotes;Bacteria;Gammaproteobacteria - Others;Amphritea"
2023	gsn	Gynuella sunshinyii	"Prokaryotes;Bacteria;Gammaproteobacteria - Others;Gynuella"
2024	rfo	Reinekea forsetii	"Prokaryotes;Bacteria;Gammaproteobacteria - Others;Reinekea"
2025	ome	Oleiphilus messinensis	"Prokaryotes;Bacteria;Gammaproteobacteria - Others;Oleiphilus"
2026	llp	Litoricola lipolytica	"Prokaryotes;Bacteria;Gammaproteobacteria - Others;Litoricola"
2027	aha	Aeromonas hydrophila subsp. hydrophila ATCC 7966	"Prokaryotes;Bacteria;Gammaproteobacteria - Others;Aeromonas"
2028	ahy	Aeromonas hydrophila ML09-119	"Prokaryotes;Bacteria;Gammaproteobacteria - Others;Aeromonas"
2029	ahd	Aeromonas hydrophila YL17	"Prokaryotes;Bacteria;Gammaproteobacteria - Others;Aeromonas"
2030	ahr	Aeromonas hydrophila subsp. hydrophila AL09-71	"Prokaryotes;Bacteria;Gammaproteobacteria - Others;Aeromonas"
2031	ahp	Aeromonas hydrophila pc104A	"Prokaryotes;Bacteria;Gammaproteobacteria - Others;Aeromonas"
2032	ahj	Aeromonas hydrophila J-1	"Prokaryotes;Bacteria;Gammaproteobacteria - Others;Aeromonas"
2033	ahh	Aeromonas hydrophila AL06-06	"Prokaryotes;Bacteria;Gammaproteobacteria - Others;Aeromonas"
2034	ahi	Aeromonas hydrophila AH10	"Prokaryotes;Bacteria;Gammaproteobacteria - Others;Aeromonas"
2035	aaj	Aeromonas hydrophila MX16A	"Prokaryotes;Bacteria;Gammaproteobacteria - Others;Aeromonas"
2036	asa	Aeromonas salmonicida subsp. salmonicida A449	"Prokaryotes;Bacteria;Gammaproteobacteria - Others;Aeromonas"
2037	aeo	Aeromonas salmonicida O23A	"Prokaryotes;Bacteria;Gammaproteobacteria - Others;Aeromonas"

2038	avr	Aeromonas veronii B565	"Prokaryotes;Bacteria;Gammaproteobacteria - Others;Aeromonas"
2039	avo	Aeromonas veronii TH0426	"Prokaryotes;Bacteria;Gammaproteobacteria - Others;Aeromonas"
2040	amed	Aeromonas media	"Prokaryotes;Bacteria;Gammaproteobacteria - Others;Aeromonas"
2041	asr	Aeromonas schubertii	"Prokaryotes;Bacteria;Gammaproteobacteria - Others;Aeromonas"
2042	adh	Aeromonas dhakensis	"Prokaryotes;Bacteria;Gammaproteobacteria - Others;Aeromonas"
2043	acav	Aeromonas caviae	"Prokaryotes;Bacteria;Gammaproteobacteria - Others;Aeromonas"
2044	aem	Aeromonas sp. CU5	"Prokaryotes;Bacteria;Gammaproteobacteria - Others;Aeromonas"
2045	aea	Aeromonas sp. ASNIH3	"Prokaryotes;Bacteria;Gammaproteobacteria - Others;Aeromonas"
2046	arv	Aeromonas rivipollensis	"Prokaryotes;Bacteria;Gammaproteobacteria - Others;Aeromonas"
2047	aes	Aeromonas sp. ASNIH5	"Prokaryotes;Bacteria;Gammaproteobacteria - Others;Aeromonas"
2048	ael	Aeromonas encheleia	"Prokaryotes;Bacteria;Gammaproteobacteria - Others;Aeromonas"
2049	tau	Tolumonas auensis	"Prokaryotes;Bacteria;Gammaproteobacteria - Others;Tolumonas"
2050	oce	Oceanimonas sp. GK1	"Prokaryotes;Bacteria;Gammaproteobacteria - Others;Oceanimonas"
2051	ocm	Oceanisphaera avium	"Prokaryotes;Bacteria;Gammaproteobacteria - Others;Oceanisphaera"
2052	opf	Oceanisphaera profunda	"Prokaryotes;Bacteria;Gammaproteobacteria - Others;Oceanisphaera"
2053	zdf	Zobellella denitrificans	"Prokaryotes;Bacteria;Gammaproteobacteria - Others;Zobellella"
2054	dno	Dichelobacter nodosus	"Prokaryotes;Bacteria;Gammaproteobacteria - Others;Dichelobacter"
2055	chj	Cardiobacterium hominis	"Prokaryotes;Bacteria;Gammaproteobacteria - Others;Cardiobacterium"
2056	gap	Gilliamella apicola	"Prokaryotes;Bacteria;Gammaproteobacteria - Others;Gilliamella"
2057	fpp	Frischella perrara	"Prokaryotes;Bacteria;Gammaproteobacteria - Others;Frischella"
2058	orb	Orbus sp. IPMB12	"Prokaryotes;Bacteria;Gammaproteobacteria - Others;Orbus"
2059	sdf	Steroidobacter denitrificans	"Prokaryotes;Bacteria;Gammaproteobacteria - Others;Steroidobacter"
2060	sok	Solimonas sp. K1W22B-7	"Prokaryotes;Bacteria;Gammaproteobacteria - Others;Solimonas"
2061	sini	Sinimarinibacterium sp. NLF-5-8	"Prokaryotes;Bacteria;Gammaproteobacteria - Others;Sinimarinibacterium"
2062	gbi	Immundisolibacter cernigliae	"Prokaryotes;Bacteria;Gammaproteobacteria - Others;Immundisolibacter"
2063	slim	Sulfuricaulis limicola	"Prokaryotes;Bacteria;Gammaproteobacteria - Others;Sulfuricaulis"
2064	sva	Sulfurifustis variabilis	"Prokaryotes;Bacteria;Gammaproteobacteria - Others;Sulfurifustis"
2065	acii	Acidiferrobacter sp. SPIII_3	"Prokaryotes;Bacteria;Gammaproteobacteria - Others;Acidiferrobacter"
2066	saln	Salinisphaera sp. LB1	"Prokaryotes;Bacteria;Gammaproteobacteria - Others;Salinisphaera"
2067	tbn	Thiolapillus brandeum	"Prokaryotes;Bacteria;Gammaproteobacteria - Others;Thiolapillus"
2068	seds	Sedimenticola thiotaurini	"Prokaryotes;Bacteria;Gammaproteobacteria - Others;Sedimenticola"
2069	tsn	Candidatus Thioglobus singularis PS1	"Prokaryotes;Bacteria;Gammaproteobacteria - Others;Thioglobus"
2070	thin	Candidatus Thioglobus sp. NP1	"Prokaryotes;Bacteria;Gammaproteobacteria - Others;Thioglobus"
2071	tho	Candidatus Thioglobus autotrophicus	"Prokaryotes;Bacteria;Gammaproteobacteria - Others;Thioglobus"
2072	pspi	Pseudohongiella spirulinae	"Prokaryotes;Bacteria;Gammaproteobacteria - Others;Pseudohongiella"
2073	eof	Endosymbiont of Sipalinus gigas	"Prokaryotes;Bacteria;Gammaproteobacteria - Others;Nardonella"
2074	rev	Candidatus Reidiella endopervernicosa	"Prokaryotes;Bacteria;Gammaproteobacteria - Others;Reidiella"
2075	rma	Candidatus Ruthia magnifica (Calyptogena magnifica)	"Prokaryotes;Bacteria;Gammaproteobacteria - Others;Ruthia"
2076	reo	Candidatus Ruthia endofausta	"Prokaryotes;Bacteria;Gammaproteobacteria - Others;Ruthia"
2077	vok	Candidatus Vesicomyosocius okutanii	"Prokaryotes;Bacteria;Gammaproteobacteria - Others;Vesicomyosocius"
2078	ebh	Endosymbiont of Bathymodiolus septemdierum	"Prokaryotes;Bacteria;Gammaproteobacteria - Others;unclassified Gammaproteobacteria"

2079	bci	Baumannia cicadellinicola Hc (Homalodisca coagulata)	"Prokaryotes;Bacteria;Gammaproteobacteria - Others;unclassified Gammaproteobacteria"
2080	bcib	Candidatus Baumannia cicadellinicola BGSS	"Prokaryotes;Bacteria;Gammaproteobacteria - Others;unclassified Gammaproteobacteria"
2081	bcig	Candidatus Baumannia cicadellinicola B-GSS	"Prokaryotes;Bacteria;Gammaproteobacteria - Others;unclassified Gammaproteobacteria"
2082	gpb	Gamma proteobacterium HdN1	"Prokaryotes;Bacteria;Gammaproteobacteria - Others;unclassified Gammaproteobacteria"
2083	enm	Endosymbiont of unidentified scaly snail isolate Monju	"Prokaryotes;Bacteria;Gammaproteobacteria - Others;unclassified Gammaproteobacteria"
2084	nme	Neisseria meningitidis MC58 (serogroup B)	"Prokaryotes;Bacteria;Betaproteobacteria;Neisseria"
2085	nmp	Neisseria meningitidis alpha710 (serogroup B)	"Prokaryotes;Bacteria;Betaproteobacteria;Neisseria"
2086	nmh	Neisseria meningitidis H44/76 (serogroup B)	"Prokaryotes;Bacteria;Betaproteobacteria;Neisseria"
2087	nmd	Neisseria meningitidis G2136 (serogroup B)	"Prokaryotes;Bacteria;Betaproteobacteria;Neisseria"
2088	nmm	Neisseria meningitidis M01-240149 (serogroup B)	"Prokaryotes;Bacteria;Betaproteobacteria;Neisseria"
2089	nms	Neisseria meningitidis M01-240355 (serogroup B)	"Prokaryotes;Bacteria;Betaproteobacteria;Neisseria"
2090	nmq	Neisseria meningitidis M04-240196 (serogroup B)	"Prokaryotes;Bacteria;Betaproteobacteria;Neisseria"
2091	nmz	Neisseria meningitidis NZ-05/33 (serogroup B)	"Prokaryotes;Bacteria;Betaproteobacteria;Neisseria"
2092	nma	Neisseria meningitidis Z2491 (serogroup A)	"Prokaryotes;Bacteria;Betaproteobacteria;Neisseria"
2093	nmw	Neisseria meningitidis WUE 2594 (serogroup A)	"Prokaryotes;Bacteria;Betaproteobacteria;Neisseria"
2094	nmx	Neisseria meningitidis 510612 (serogroup A)	"Prokaryotes;Bacteria;Betaproteobacteria;Neisseria"
2095	nmc	Neisseria meningitidis FAM18 (serogroup C)	"Prokaryotes;Bacteria;Betaproteobacteria;Neisseria"
2096	nmn	Neisseria meningitidis 053442 (serogroup C)	"Prokaryotes;Bacteria;Betaproteobacteria;Neisseria"
2097	nmt	Neisseria meningitidis 8013 (serogroup C)	"Prokaryotes;Bacteria;Betaproteobacteria;Neisseria"
2098	nmi	Neisseria meningitidis alpha14 (cnl strain)	"Prokaryotes;Bacteria;Betaproteobacteria;Neisseria"
2099	ngo	Neisseria gonorrhoeae FA 1090	"Prokaryotes;Bacteria;Betaproteobacteria;Neisseria"
2100	ngk	Neisseria gonorrhoeae NCCP11945	"Prokaryotes;Bacteria;Betaproteobacteria;Neisseria"
2101	nla	Neisseria lactamica	"Prokaryotes;Bacteria;Betaproteobacteria;Neisseria"
2102	nel	Neisseria elongata	"Prokaryotes;Bacteria;Betaproteobacteria;Neisseria"
2103	nwe	Neisseria weaveri	"Prokaryotes;Bacteria;Betaproteobacteria;Neisseria"
2104	nsi	Neisseria mucosa FDAARGOS_260	"Prokaryotes;Bacteria;Betaproteobacteria;Neisseria"
2105	nmj	Neisseria mucosa ATCC 19696	"Prokaryotes;Bacteria;Betaproteobacteria;Neisseria"
2106	nei	Neisseria chenwenguii	"Prokaryotes;Bacteria;Betaproteobacteria;Neisseria"
2107	nek	Neisseria sp. KEM232	"Prokaryotes;Bacteria;Betaproteobacteria;Neisseria"
2108	nfv	Neisseria flavescens	"Prokaryotes;Bacteria;Betaproteobacteria;Neisseria"
2109	nsf	Neisseria subflava	"Prokaryotes;Bacteria;Betaproteobacteria;Neisseria"
2110	nzl	Neisseria zalophi	"Prokaryotes;Bacteria;Betaproteobacteria;Neisseria"
2111	naq	Neisseria animalis	"Prokaryotes;Bacteria;Betaproteobacteria;Neisseria"
2112	nbl	Neisseria brasiliensis	"Prokaryotes;Bacteria;Betaproteobacteria;Neisseria"
2113	nzo	Neisseria zoodegmatis	"Prokaryotes;Bacteria;Betaproteobacteria;Neisseria"
2114	nci	Neisseria canis	"Prokaryotes;Bacteria;Betaproteobacteria;Neisseria"
2115	ncz	Neisseria cinerea	"Prokaryotes;Bacteria;Betaproteobacteria;Neisseria"
2116	nani	Neisseria animaloris	"Prokaryotes;Bacteria;Betaproteobacteria;Neisseria"
2117	nbc	Neisseria bacilliformis	"Prokaryotes;Bacteria;Betaproteobacteria;Neisseria"
2118	salv	Snodgrassella alvi	"Prokaryotes;Bacteria;Betaproteobacteria;Snodgrassella"
2119	kki	Kingella kingae	"Prokaryotes;Bacteria;Betaproteobacteria;Kingella"

2120	koa	Kingella oralis	"Prokaryotes;Bacteria;Betaproteobacteria;Kingella"
2121	vff	Vitreoscilla filiformis	"Prokaryotes;Bacteria;Betaproteobacteria;Vitreoscilla"
2122	vit	Vitreoscilla sp. C1	"Prokaryotes;Bacteria;Betaproteobacteria;Vitreoscilla"
2123	ecor	Eikenella corrodens	"Prokaryotes;Bacteria;Betaproteobacteria;Eikenella"
2124	eex	Eikenella exigua	"Prokaryotes;Bacteria;Betaproteobacteria;Eikenella"
2125	smur	Simonsiella muelleri	"Prokaryotes;Bacteria;Betaproteobacteria;Simonsiella"
2126	aff	Alysiella filiformis	"Prokaryotes;Bacteria;Betaproteobacteria;Alysiella"
2127	cste	Conchiformibius steedae	"Prokaryotes;Bacteria;Betaproteobacteria;Conchiformibius"
2128	nba	Neisseriaceae bacterium DSM 100970	"Prokaryotes;Bacteria;Betaproteobacteria;unclassified Neisseriaceae"
2129	cvi	Chromobacterium violaceum	"Prokaryotes;Bacteria;Betaproteobacteria;Chromobacterium"
2130	cvc	Chromobacterium vaccinii	"Prokaryotes;Bacteria;Betaproteobacteria;Chromobacterium"
2131	chro	Chromobacterium sp. ATCC 53434	"Prokaryotes;Bacteria;Betaproteobacteria;Chromobacterium"
2132	chri	Chromobacterium phragmitis IIBBL 112-1	"Prokaryotes;Bacteria;Betaproteobacteria;Chromobacterium"
2133	chrb	Chromobacterium phragmitis IIBBL 274-1	"Prokaryotes;Bacteria;Betaproteobacteria;Chromobacterium"
2134	crz	Chromobacterium rhizoryzae	"Prokaryotes;Bacteria;Betaproteobacteria;Chromobacterium"
2135	chrm	Chromobacterium paludis	"Prokaryotes;Bacteria;Betaproteobacteria;Chromobacterium"
2136	chae	Chromobacterium haemolyticum	"Prokaryotes;Bacteria;Betaproteobacteria;Chromobacterium"
2137	iod	Iodobacter sp. H11R3	"Prokaryotes;Bacteria;Betaproteobacteria;Iodobacter"
2138	ifl	Iodobacter fluviatilis	"Prokaryotes;Bacteria;Betaproteobacteria;Iodobacter"
2139	lhk	Laribacter hongkongensis	"Prokaryotes;Bacteria;Betaproteobacteria;Laribacter"
2140	pse	Pseudogulbenkiania sp. NH8B	"Prokaryotes;Bacteria;Betaproteobacteria;Pseudogulbenkiania"
2141	jeu	Jeongeupia sp. USM3	"Prokaryotes;Bacteria;Betaproteobacteria;Jeongeupia"
2142	aql	Aquaspirillum sp. LM1	"Prokaryotes;Bacteria;Betaproteobacteria;Aquaspirillum"
2143	amah	Aquitalea magnusonii	"Prokaryotes;Bacteria;Betaproteobacteria;Aquitalea"
2144	aqs	Aquitalea sp. USM4	"Prokaryotes;Bacteria;Betaproteobacteria;Aquitalea"
2145	dee	Deefgea sp. D17	"Prokaryotes;Bacteria;Betaproteobacteria;Deefgea"
2146	chiz	Chitinibacter sp. 2T18	"Prokaryotes;Bacteria;Betaproteobacteria;Chitinibacter"
2147	cfon	Chitinibacter fontanus	"Prokaryotes;Bacteria;Betaproteobacteria;Chitinibacter"
2148	rso	Ralstonia solanacearum GMI1000	"Prokaryotes;Bacteria;Betaproteobacteria;Ralstonia"
2149	rsc	Ralstonia solanacearum CFBP2957	"Prokaryotes;Bacteria;Betaproteobacteria;Ralstonia"
2150	rsl	Ralstonia solanacearum PSI07	"Prokaryotes;Bacteria;Betaproteobacteria;Ralstonia"
2151	rsn	Ralstonia solanacearum Po82	"Prokaryotes;Bacteria;Betaproteobacteria;Ralstonia"
2152	rsm	Ralstonia solanacearum CMR15	"Prokaryotes;Bacteria;Betaproteobacteria;Ralstonia"
2153	rse	Ralstonia solanacearum FQY_4	"Prokaryotes;Bacteria;Betaproteobacteria;Ralstonia"
2154	rsy	Ralstonia solanacearum UY031	"Prokaryotes;Bacteria;Betaproteobacteria;Ralstonia"
2155	rpi	Ralstonia pickettii 12J	"Prokaryotes;Bacteria;Betaproteobacteria;Ralstonia"
2156	rpf	Ralstonia pickettii 12D	"Prokaryotes;Bacteria;Betaproteobacteria;Ralstonia"
2157	rpj	Ralstonia pickettii DTP0602	"Prokaryotes;Bacteria;Betaproteobacteria;Ralstonia"
2158	rmn	Ralstonia mannitolilytica	"Prokaryotes;Bacteria;Betaproteobacteria;Ralstonia"
2159	rin	Ralstonia insidiosa	"Prokaryotes;Bacteria;Betaproteobacteria;Ralstonia"
2160	rpu	Ralstonia pseudosolanacearum	"Prokaryotes;Bacteria;Betaproteobacteria;Ralstonia"

2161	reh	Cupriavidus necator H16	"Prokaryotes;Bacteria;Betaproteobacteria;Cupriavidus"
2162	cnc	Cupriavidus necator N-1	"Prokaryotes;Bacteria;Betaproteobacteria;Cupriavidus"
2163	cuh	Cupriavidus necator NH9	"Prokaryotes;Bacteria;Betaproteobacteria;Cupriavidus"
2164	reu	Cupriavidus pinatubonensis JMP134	"Prokaryotes;Bacteria;Betaproteobacteria;Cupriavidus"
2165	rme	Cupriavidus metallidurans	"Prokaryotes;Bacteria;Betaproteobacteria;Cupriavidus"
2166	cti	Cupriavidus taiwanensis	"Prokaryotes;Bacteria;Betaproteobacteria;Cupriavidus"
2167	cbw	Cupriavidus basilensis	"Prokaryotes;Bacteria;Betaproteobacteria;Cupriavidus"
2168	cgd	Cupriavidus gilardii	"Prokaryotes;Bacteria;Betaproteobacteria;Cupriavidus"
2169	ccup	Cupriavidus sp. USMAHM13	"Prokaryotes;Bacteria;Betaproteobacteria;Cupriavidus"
2170	cup	Cupriavidus malaysiensis	"Prokaryotes;Bacteria;Betaproteobacteria;Cupriavidus"
2171	cuu	Cupriavidus sp. USMAA2-4	"Prokaryotes;Bacteria;Betaproteobacteria;Cupriavidus"
2172	cpau	Cupriavidus pauculus	"Prokaryotes;Bacteria;Betaproteobacteria;Cupriavidus"
2173	cox	Cupriavidus oxalaticus	"Prokaryotes;Bacteria;Betaproteobacteria;Cupriavidus"
2174	bma	Burkholderia mallei ATCC 23344	"Prokaryotes;Bacteria;Betaproteobacteria;Burkholderia"
2175	bmv	Burkholderia mallei SAVP1	"Prokaryotes;Bacteria;Betaproteobacteria;Burkholderia"
2176	bml	Burkholderia mallei NCTC 10229	"Prokaryotes;Bacteria;Betaproteobacteria;Burkholderia"
2177	bmn	Burkholderia mallei NCTC 10247	"Prokaryotes;Bacteria;Betaproteobacteria;Burkholderia"
2178	bmal	Burkholderia mallei 23344	"Prokaryotes;Bacteria;Betaproteobacteria;Burkholderia"
2179	bmae	Burkholderia mallei 6	"Prokaryotes;Bacteria;Betaproteobacteria;Burkholderia"
2180	bmaq	Burkholderia mallei BMQ	"Prokaryotes;Bacteria;Betaproteobacteria;Burkholderia"
2181	bmai	Burkholderia mallei 2000031063	"Prokaryotes;Bacteria;Betaproteobacteria;Burkholderia"
2182	bmaf	Burkholderia mallei FMH 23344	"Prokaryotes;Bacteria;Betaproteobacteria;Burkholderia"
2183	bmaz	Burkholderia mallei NCTC 10247	"Prokaryotes;Bacteria;Betaproteobacteria;Burkholderia"
2184	bmab	Burkholderia mallei 2002734299	"Prokaryotes;Bacteria;Betaproteobacteria;Burkholderia"
2185	bps	Burkholderia pseudomallei K96243	"Prokaryotes;Bacteria;Betaproteobacteria;Burkholderia"
2186	bpm	Burkholderia pseudomallei 1710b	"Prokaryotes;Bacteria;Betaproteobacteria;Burkholderia"
2187	bpl	Burkholderia pseudomallei 1106a	"Prokaryotes;Bacteria;Betaproteobacteria;Burkholderia"
2188	bpd	Burkholderia pseudomallei 668	"Prokaryotes;Bacteria;Betaproteobacteria;Burkholderia"
2189	bpr	Burkholderia pseudomallei MSHR346	"Prokaryotes;Bacteria;Betaproteobacteria;Burkholderia"
2190	bpse	Burkholderia pseudomallei MSHR305	"Prokaryotes;Bacteria;Betaproteobacteria;Burkholderia"
2191	bpsm	Burkholderia pseudomallei MSHR511	"Prokaryotes;Bacteria;Betaproteobacteria;Burkholderia"
2192	bpsu	Burkholderia pseudomallei MSHR146	"Prokaryotes;Bacteria;Betaproteobacteria;Burkholderia"
2193	bpsd	Burkholderia pseudomallei MSHR520	"Prokaryotes;Bacteria;Betaproteobacteria;Burkholderia"
2194	bpz	Burkholderia pseudomallei 1026b	"Prokaryotes;Bacteria;Betaproteobacteria;Burkholderia"
2195	bpq	Burkholderia pseudomallei BPC006	"Prokaryotes;Bacteria;Betaproteobacteria;Burkholderia"
2196	bpk	Burkholderia pseudomallei NCTC 13179	"Prokaryotes;Bacteria;Betaproteobacteria;Burkholderia"
2197	bpsh	Burkholderia pseudomallei HBPUB10134a	"Prokaryotes;Bacteria;Betaproteobacteria;Burkholderia"
2198	bpsa	Burkholderia pseudomallei NAU35A-3	"Prokaryotes;Bacteria;Betaproteobacteria;Burkholderia"
2199	bpso	Burkholderia pseudomallei A79A	"Prokaryotes;Bacteria;Betaproteobacteria;Burkholderia"
2200	but	Burkholderia pseudomallei TSV202	"Prokaryotes;Bacteria;Betaproteobacteria;Burkholderia"
2201	bte	Burkholderia thailandensis E264	"Prokaryotes;Bacteria;Betaproteobacteria;Burkholderia"

2202	btq	Burkholderia thailandensis 2002721723	"Prokaryotes;Bacteria;Betaproteobacteria;Burkholderia"
2203	btj	Burkholderia thailandensis E444	"Prokaryotes;Bacteria;Betaproteobacteria;Burkholderia"
2204	btz	Burkholderia thailandensis H0587	"Prokaryotes;Bacteria;Betaproteobacteria;Burkholderia"
2205	btd	Burkholderia thailandensis MSMB121	"Prokaryotes;Bacteria;Betaproteobacteria;Burkholderia"
2206	btv	Burkholderia thailandensis MSMB59	"Prokaryotes;Bacteria;Betaproteobacteria;Burkholderia"
2207	bthe	Burkholderia thailandensis E254	"Prokaryotes;Bacteria;Betaproteobacteria;Burkholderia"
2208	bthm	Burkholderia thailandensis USAMRU Malaysia #20	"Prokaryotes;Bacteria;Betaproteobacteria;Burkholderia"
2209	btha	Burkholderia thailandensis 2003015869	"Prokaryotes;Bacteria;Betaproteobacteria;Burkholderia"
2210	bthl	Burkholderia thailandensis 2002721643	"Prokaryotes;Bacteria;Betaproteobacteria;Burkholderia"
2211	bok	Burkholderia oklahomensis E0147	"Prokaryotes;Bacteria;Betaproteobacteria;Burkholderia"
2212	boc	Burkholderia oklahomensis C6786	"Prokaryotes;Bacteria;Betaproteobacteria;Burkholderia"
2213	buu	Burkholderia sp. BDU6	"Prokaryotes;Bacteria;Betaproteobacteria;Burkholderia"
2214	bvi	Burkholderia vietnamiensis G4	"Prokaryotes;Bacteria;Betaproteobacteria;Burkholderia"
2215	bve	Burkholderia vietnamiensis LMG 10929	"Prokaryotes;Bacteria;Betaproteobacteria;Burkholderia"
2216	bur	Burkholderia lata	"Prokaryotes;Bacteria;Betaproteobacteria;Burkholderia"
2217	bcn	Burkholderia cenocepacia AU 1054	"Prokaryotes;Bacteria;Betaproteobacteria;Burkholderia"
2218	bch	Burkholderia cenocepacia HI2424	"Prokaryotes;Bacteria;Betaproteobacteria;Burkholderia"
2219	bcm	Burkholderia cenocepacia MC0-3	"Prokaryotes;Bacteria;Betaproteobacteria;Burkholderia"
2220	bcj	Burkholderia cenocepacia J2315	"Prokaryotes;Bacteria;Betaproteobacteria;Burkholderia"
2221	bcen	Burkholderia cenocepacia DDS 22E-1	"Prokaryotes;Bacteria;Betaproteobacteria;Burkholderia"
2222	bcew	Burkholderia cenocepacia DWS 37E-2	"Prokaryotes;Bacteria;Betaproteobacteria;Burkholderia"
2223	bceo	Burkholderia cenocepacia H111	"Prokaryotes;Bacteria;Betaproteobacteria;Burkholderia"
2224	bam	Burkholderia ambifaria AMMD	"Prokaryotes;Bacteria;Betaproteobacteria;Burkholderia"
2225	bac	Burkholderia ambifaria MC40-6	"Prokaryotes;Bacteria;Betaproteobacteria;Burkholderia"
2226	bmj	Burkholderia multivorans ATCC 17616 (Tohoku)	"Prokaryotes;Bacteria;Betaproteobacteria;Burkholderia"
2227	bmu	Burkholderia multivorans ATCC 17616 (JGI)	"Prokaryotes;Bacteria;Betaproteobacteria;Burkholderia"
2228	bmk	Burkholderia multivorans DDS 15A-1	"Prokaryotes;Bacteria;Betaproteobacteria;Burkholderia"
2229	bmul	Burkholderia multivorans ATCC BAA-247	"Prokaryotes;Bacteria;Betaproteobacteria;Burkholderia"
2230	bct	Burkholderia cepacia GG4	"Prokaryotes;Bacteria;Betaproteobacteria;Burkholderia"
2231	bced	Burkholderia cepacia DDS 7H-2	"Prokaryotes;Bacteria;Betaproteobacteria;Burkholderia"
2232	bcep	Burkholderia cepacia ATCC 25416	"Prokaryotes;Bacteria;Betaproteobacteria;Burkholderia"
2233	bdl	Burkholderia dolosa	"Prokaryotes;Bacteria;Betaproteobacteria;Burkholderia"
2234	bpyr	Burkholderia pyrrocinia	"Prokaryotes;Bacteria;Betaproteobacteria;Burkholderia"
2235	bcon	Burkholderia contaminans	"Prokaryotes;Bacteria;Betaproteobacteria;Burkholderia"
2236	bub	Burkholderia ubonensis	"Prokaryotes;Bacteria;Betaproteobacteria;Burkholderia"
2237	bdf	Burkholderia diffusa	"Prokaryotes;Bacteria;Betaproteobacteria;Burkholderia"
2238	blat	Burkholderia latens	"Prokaryotes;Bacteria;Betaproteobacteria;Burkholderia"
2239	btei	Burkholderia territorii	"Prokaryotes;Bacteria;Betaproteobacteria;Burkholderia"
2240	bsem	Burkholderia seminalis	"Prokaryotes;Bacteria;Betaproteobacteria;Burkholderia"
2241	bpsl	Burkholderia pseudomultivorans	"Prokaryotes;Bacteria;Betaproteobacteria;Burkholderia"
2242	bmec	Burkholderia metallica	"Prokaryotes;Bacteria;Betaproteobacteria;Burkholderia"

2243	bstg	Burkholderia stagnalis	"Prokaryotes;Bacteria;Betaproteobacteria;Burkholderia"
2244	bstl	Burkholderia stabilis	"Prokaryotes;Bacteria;Betaproteobacteria;Burkholderia"
2245	bgl	Burkholderia glumae BGR1	"Prokaryotes;Bacteria;Betaproteobacteria;Burkholderia"
2246	bgu	Burkholderia glumae LMG 2196 = ATCC 33617	"Prokaryotes;Bacteria;Betaproteobacteria;Burkholderia"
2247	bug	Burkholderia sp. CCGE1001	"Prokaryotes;Bacteria;Betaproteobacteria;Burkholderia"
2248	bgf	Burkholderia sp. CCGE1003	"Prokaryotes;Bacteria;Betaproteobacteria;Burkholderia"
2249	bgd	Burkholderia gladioli BSR3	"Prokaryotes;Bacteria;Betaproteobacteria;Burkholderia"
2250	bgo	Burkholderia gladioli ATCC 10248	"Prokaryotes;Bacteria;Betaproteobacteria;Burkholderia"
2251	byi	Burkholderia sp. YI23	"Prokaryotes;Bacteria;Betaproteobacteria;Burkholderia"
2252	buk	Burkholderia sp. KJ006	"Prokaryotes;Bacteria;Betaproteobacteria;Burkholderia"
2253	bue	Burkholderia sp. RPE67	"Prokaryotes;Bacteria;Betaproteobacteria;Burkholderia"
2254	bul	Burkholderia sp. 2002721687	"Prokaryotes;Bacteria;Betaproteobacteria;Burkholderia"
2255	buq	Burkholderia sp. HB1	"Prokaryotes;Bacteria;Betaproteobacteria;Burkholderia"
2256	bgp	Burkholderia plantarii PG1	"Prokaryotes;Bacteria;Betaproteobacteria;Burkholderia"
2257	bpla	Burkholderia plantarii ATCC 43733	"Prokaryotes;Bacteria;Betaproteobacteria;Burkholderia"
2258	bud	Burkholderia sp. Bp5365	"Prokaryotes;Bacteria;Betaproteobacteria;Burkholderia"
2259	bum	Burkholderia sp. PAMC 26561	"Prokaryotes;Bacteria;Betaproteobacteria;Burkholderia"
2260	bui	Burkholderia sp. PAMC 28687	"Prokaryotes;Bacteria;Betaproteobacteria;Burkholderia"
2261	bxe	Paraburkholderia xenovorans LB400	"Prokaryotes;Bacteria;Betaproteobacteria;Paraburkholderia"
2262	bxb	Paraburkholderia xenovorans LB400	"Prokaryotes;Bacteria;Betaproteobacteria;Paraburkholderia"
2263	bph	Paraburkholderia phymatum	"Prokaryotes;Bacteria;Betaproteobacteria;Paraburkholderia"
2264	bge	Paraburkholderia atlantica CCGE1002	"Prokaryotes;Bacteria;Betaproteobacteria;Paraburkholderia"
2265	bpx	Paraburkholderia phenoliruptrix	"Prokaryotes;Bacteria;Betaproteobacteria;Paraburkholderia"
2266	bpy	Paraburkholderia phytofirmans PsJN	"Prokaryotes;Bacteria;Betaproteobacteria;Paraburkholderia"
2267	buz	Paraburkholderia phytofirmans OLGA172	"Prokaryotes;Bacteria;Betaproteobacteria;Paraburkholderia"
2268	bfn	Paraburkholderia fungorum	"Prokaryotes;Bacteria;Betaproteobacteria;Paraburkholderia"
2269	bcai	Paraburkholderia caribensis	"Prokaryotes;Bacteria;Betaproteobacteria;Paraburkholderia"
2270	pspw	Paraburkholderia sprentiae	"Prokaryotes;Bacteria;Betaproteobacteria;Paraburkholderia"
2271	para	Paraburkholderia sp. SOS3	"Prokaryotes;Bacteria;Betaproteobacteria;Paraburkholderia"
2272	parb	Paraburkholderia aromaticivorans	"Prokaryotes;Bacteria;Betaproteobacteria;Paraburkholderia"
2273	phs	Paraburkholderia hospita	"Prokaryotes;Bacteria;Betaproteobacteria;Paraburkholderia"
2274	pter	Paraburkholderia terrae	"Prokaryotes;Bacteria;Betaproteobacteria;Paraburkholderia"
2275	pgp	Paraburkholderia graminis	"Prokaryotes;Bacteria;Betaproteobacteria;Paraburkholderia"
2276	pcj	Paraburkholderia caledonica	"Prokaryotes;Bacteria;Betaproteobacteria;Paraburkholderia"
2277	pts	Paraburkholderia terricola	"Prokaryotes;Bacteria;Betaproteobacteria;Paraburkholderia"
2278	pcaf	Paraburkholderia caffeinilytica	"Prokaryotes;Bacteria;Betaproteobacteria;Paraburkholderia"
2279	pmeg	Paraburkholderia megapolitana	"Prokaryotes;Bacteria;Betaproteobacteria;Paraburkholderia"
2280	brh	Mycetohabitans rhizoxinica	"Prokaryotes;Bacteria;Betaproteobacteria;Mycetohabitans"
2281	pnu	Polynucleobacter asymbioticus	"Prokaryotes;Bacteria;Betaproteobacteria;Polynucleobacter"
2282	pne	Polynucleobacter necessarius	"Prokaryotes;Bacteria;Betaproteobacteria;Polynucleobacter"
2283	pdq	Polynucleobacter duraquae	"Prokaryotes;Bacteria;Betaproteobacteria;Polynucleobacter"

2284	poh	Polynucleobacter paneuropaeus	"Prokaryotes;Bacteria;Betaproteobacteria;Polynucleobacter"
2285	ppk	Pandoraea pnomenusa 3kgm	"Prokaryotes;Bacteria;Betaproteobacteria;Pandoraea"
2286	ppno	Pandoraea pnomenusa RB38	"Prokaryotes;Bacteria;Betaproteobacteria;Pandoraea"
2287	ppnm	Pandoraea pnomenusa DSM 16536	"Prokaryotes;Bacteria;Betaproteobacteria;Pandoraea"
2288	prb	Pandoraea pnomenusa	"Prokaryotes;Bacteria;Betaproteobacteria;Pandoraea"
2289	ppul	Pandoraea pulmonicola	"Prokaryotes;Bacteria;Betaproteobacteria;Pandoraea"
2290	pspu	Pandoraea sputorum	"Prokaryotes;Bacteria;Betaproteobacteria;Pandoraea"
2291	papi	Pandoraea apista	"Prokaryotes;Bacteria;Betaproteobacteria;Pandoraea"
2292	pve	Pandoraea vervacti	"Prokaryotes;Bacteria;Betaproteobacteria;Pandoraea"
2293	pox	Pandoraea oxalativorans	"Prokaryotes;Bacteria;Betaproteobacteria;Pandoraea"
2294	ptx	Pandoraea thiooxydans	"Prokaryotes;Bacteria;Betaproteobacteria;Pandoraea"
2295	pfg	Pandoraea faecigallinarum	"Prokaryotes;Bacteria;Betaproteobacteria;Pandoraea"
2296	pnr	Pandoraea norimbergensis	"Prokaryotes;Bacteria;Betaproteobacteria;Pandoraea"
2297	pand	Pandoraea sp. XY-2	"Prokaryotes;Bacteria;Betaproteobacteria;Pandoraea"
2298	pfib	Pandoraea fibrosis	"Prokaryotes;Bacteria;Betaproteobacteria;Pandoraea"
2299	plg	Paucimonas lemoignei	"Prokaryotes;Bacteria;Betaproteobacteria;Paucimonas"
2300	hyf	Ephemeroptericola cinctiostellae	"Prokaryotes;Bacteria;Betaproteobacteria;Ephemeroptericola"
2301	lmir	Lautropia mirabilis	"Prokaryotes;Bacteria;Betaproteobacteria;Lautropia"
2302	mcys	Mycoavidus cysteinexigens	"Prokaryotes;Bacteria;Betaproteobacteria;Mycoavidus"
2303	caba	Caballeronia sp. SBC2	"Prokaryotes;Bacteria;Betaproteobacteria;Caballeronia"
2304	buo	Caballeronia insecticola	"Prokaryotes;Bacteria;Betaproteobacteria;Caballeronia"
2305	limn	Limnobacter sp. SAORIC-580	"Prokaryotes;Bacteria;Betaproteobacteria;Limnobacter"
2306	cari	Chitinimonas arctica	"Prokaryotes;Bacteria;Betaproteobacteria;Chitinimonas"
2307	bpe	Bordetella pertussis Tohama I	"Prokaryotes;Bacteria;Betaproteobacteria;Bordetella"
2308	bpc	Bordetella pertussis CS	"Prokaryotes;Bacteria;Betaproteobacteria;Bordetella"
2309	bper	Bordetella pertussis 18323	"Prokaryotes;Bacteria;Betaproteobacteria;Bordetella"
2310	bpet	Bordetella pertussis B1917	"Prokaryotes;Bacteria;Betaproteobacteria;Bordetella"
2311	bpeu	Bordetella pertussis 137	"Prokaryotes;Bacteria;Betaproteobacteria;Bordetella"
2312	bpar	Bordetella parapertussis Bpp5	"Prokaryotes;Bacteria;Betaproteobacteria;Bordetella"
2313	bpa	Bordetella parapertussis 12822	"Prokaryotes;Bacteria;Betaproteobacteria;Bordetella"
2314	bbh	Bordetella bronchiseptica 253	"Prokaryotes;Bacteria;Betaproteobacteria;Bordetella"
2315	bbr	Bordetella bronchiseptica RB50	"Prokaryotes;Bacteria;Betaproteobacteria;Bordetella"
2316	bbm	Bordetella bronchiseptica MO149	"Prokaryotes;Bacteria;Betaproteobacteria;Bordetella"
2317	bbx	Bordetella bronchiseptica S798	"Prokaryotes;Bacteria;Betaproteobacteria;Bordetella"
2318	bpt	Bordetella petrii	"Prokaryotes;Bacteria;Betaproteobacteria;Bordetella"
2319	bav	Bordetella avium	"Prokaryotes;Bacteria;Betaproteobacteria;Bordetella"
2320	bho	Bordetella holmesii ATCC 51541	"Prokaryotes;Bacteria;Betaproteobacteria;Bordetella"
2321	bhm	Bordetella holmesii 44057	"Prokaryotes;Bacteria;Betaproteobacteria;Bordetella"
2322	bhz	Bordetella hinzii	"Prokaryotes;Bacteria;Betaproteobacteria;Bordetella"
2323	btrm	Bordetella trematum	"Prokaryotes;Bacteria;Betaproteobacteria;Bordetella"
2324	bbro	Bordetella bronchialis	"Prokaryotes;Bacteria;Betaproteobacteria;Bordetella"

2325	bfz	Bordetella flabilis	"Prokaryotes;Bacteria;Betaproteobacteria;Bordetella"
2326	bpdz	Bordetella pseudohinzii	"Prokaryotes;Bacteria;Betaproteobacteria;Bordetella"
2327	boh	Bordetella sp. H567	"Prokaryotes;Bacteria;Betaproteobacteria;Bordetella"
2328	bgm	Bordetella genomosp. 13	"Prokaryotes;Bacteria;Betaproteobacteria;Bordetella"
2329	boj	Bordetella sp. J329	"Prokaryotes;Bacteria;Betaproteobacteria;Bordetella"
2330	boz	Algicoccus marinus	"Prokaryotes;Bacteria;Betaproteobacteria;Bordetella"
2331	axy	Achromobacter xylosoxidans A8	"Prokaryotes;Bacteria;Betaproteobacteria;Achromobacter"
2332	axo	Achromobacter xylosoxidans NH44784-1996	"Prokaryotes;Bacteria;Betaproteobacteria;Achromobacter"
2333	axn	Achromobacter xylosoxidans NBRC 15126 = ATCC 27061	"Prokaryotes;Bacteria;Betaproteobacteria;Achromobacter"
2334	axx	Achromobacter xylosoxidans NCTC10807	"Prokaryotes;Bacteria;Betaproteobacteria;Achromobacter"
2335	adt	Achromobacter denitrificans	"Prokaryotes;Bacteria;Betaproteobacteria;Achromobacter"
2336	ais	Achromobacter insolitus	"Prokaryotes;Bacteria;Betaproteobacteria;Achromobacter"
2337	asw	Achromobacter spanius	"Prokaryotes;Bacteria;Betaproteobacteria;Achromobacter"
2338	achr	Achromobacter sp. AONIH1	"Prokaryotes;Bacteria;Betaproteobacteria;Achromobacter"
2339	achb	Achromobacter sp. B7	"Prokaryotes;Bacteria;Betaproteobacteria;Achromobacter"
2340	teq	Taylorella equigenitalis MCE9	"Prokaryotes;Bacteria;Betaproteobacteria;Taylorella"
2341	tea	Taylorella equigenitalis ATCC 35865	"Prokaryotes;Bacteria;Betaproteobacteria;Taylorella"
2342	teg	Taylorella equigenitalis 14/56	"Prokaryotes;Bacteria;Betaproteobacteria;Taylorella"
2343	tas	Taylorella asinigenitalis MCE3	"Prokaryotes;Bacteria;Betaproteobacteria;Taylorella"
2344	tat	Taylorella asinigenitalis 14/45	"Prokaryotes;Bacteria;Betaproteobacteria;Taylorella"
2345	put	Pusillimonas sp. T7-7	"Prokaryotes;Bacteria;Betaproteobacteria;Pusillimonas"
2346	pus	Pusillimonas thiosulfatoxidans	"Prokaryotes;Bacteria;Betaproteobacteria;Pusillimonas"
2347	pud	Pusillimonas sp. DMV24BSW_D	"Prokaryotes;Bacteria;Betaproteobacteria;Pusillimonas"
2348	aka	Advenella kashmirensis	"Prokaryotes;Bacteria;Betaproteobacteria;Advenella"
2349	amim	Advenella mimigardefordensis	"Prokaryotes;Bacteria;Betaproteobacteria;Advenella"
2350	cdn	Castellaniella defragrans	"Prokaryotes;Bacteria;Betaproteobacteria;Castellaniella"
2351	bpsi	Basilea psittacipulmonis	"Prokaryotes;Bacteria;Betaproteobacteria;Basilea"
2352	afa	Alcaligenes faecalis ZD02	"Prokaryotes;Bacteria;Betaproteobacteria;Alcaligenes"
2353	afq	Alcaligenes faecalis JQ135	"Prokaryotes;Bacteria;Betaproteobacteria;Alcaligenes"
2354	aaqu	Alcaligenes aquatilis	"Prokaryotes;Bacteria;Betaproteobacteria;Alcaligenes"
2355	phn	Paenalcaligenes hominis	"Prokaryotes;Bacteria;Betaproteobacteria;Paenalcaligenes"
2356	odi	Orrella dioscoreae	"Prokaryotes;Bacteria;Betaproteobacteria;Orrella"
2357	our	Oligella urethralis	"Prokaryotes;Bacteria;Betaproteobacteria;Oligella"
2358	pig	Pigmentiphaga sp. H8	"Prokaryotes;Bacteria;Betaproteobacteria;Pigmentiphaga"
2359	pacr	Pigmentiphaga aceris	"Prokaryotes;Bacteria;Betaproteobacteria;Pigmentiphaga"
2360	kgy	Kerstersia gyiorum	"Prokaryotes;Bacteria;Betaproteobacteria;Kerstersia"
2361	rfr	Rhodoferax ferrireducens	"Prokaryotes;Bacteria;Betaproteobacteria;Rhodoferax"
2362	rsb	Rhodoferax saidenbachensis	"Prokaryotes;Bacteria;Betaproteobacteria;Rhodoferax"
2363	rac	Rhodoferax antarcticus	"Prokaryotes;Bacteria;Betaproteobacteria;Rhodoferax"
2364	rhy	Rhodoferax koreense	"Prokaryotes;Bacteria;Betaproteobacteria;Rhodoferax"
2365	rhf	Rhodoferax sediminis CHu59-6-5	"Prokaryotes;Bacteria;Betaproteobacteria;Rhodoferax"

2366	rhg	Rhodoferax sediminis Gr-4	"Prokaryotes;Bacteria;Betaproteobacteria;Rhodoferax"
2367	pol	Polaromonas sp. JS666	"Prokaryotes;Bacteria;Betaproteobacteria;Polaromonas"
2368	pna	Polaromonas naphthalenivorans	"Prokaryotes;Bacteria;Betaproteobacteria;Polaromonas"
2369	pos	Polaromonas sp. SP1	"Prokaryotes;Bacteria;Betaproteobacteria;Polaromonas"
2370	poo	Polaromonas sp. Pch-P	"Prokaryotes;Bacteria;Betaproteobacteria;Polaromonas"
2371	aav	Acidovorax citrulli	"Prokaryotes;Bacteria;Betaproteobacteria;Acidovorax"
2372	ajs	Acidovorax sp. JS42	"Prokaryotes;Bacteria;Betaproteobacteria;Acidovorax"
2373	dia	Acidovorax ebreus	"Prokaryotes;Bacteria;Betaproteobacteria;Acidovorax"
2374	aaa	Acidovorax avenae	"Prokaryotes;Bacteria;Betaproteobacteria;Acidovorax"
2375	ack	Acidovorax sp. KKS102	"Prokaryotes;Bacteria;Betaproteobacteria;Acidovorax"
2376	acra	Acidovorax sp. RAC01	"Prokaryotes;Bacteria;Betaproteobacteria;Acidovorax"
2377	acid	Acidovorax carolinensis NA2	"Prokaryotes;Bacteria;Betaproteobacteria;Acidovorax"
2378	acip	Acidovorax carolinensis P4	"Prokaryotes;Bacteria;Betaproteobacteria;Acidovorax"
2379	acin	Acidovorax carolinensis NA3	"Prokaryotes;Bacteria;Betaproteobacteria;Acidovorax"
2380	acis	Acidovorax carolinensis P3	"Prokaryotes;Bacteria;Betaproteobacteria;Acidovorax"
2381	acio	Acidovorax sp. 1608163	"Prokaryotes;Bacteria;Betaproteobacteria;Acidovorax"
2382	amon	Acidovorax monticola	"Prokaryotes;Bacteria;Betaproteobacteria;Acidovorax"
2383	vei	Verminephrobacter eiseniae	"Prokaryotes;Bacteria;Betaproteobacteria;Verminephrobacter"
2384	dac	Delftia acidovorans	"Prokaryotes;Bacteria;Betaproteobacteria;Delftia"
2385	del	Delftia sp. Cs1-4	"Prokaryotes;Bacteria;Betaproteobacteria;Delftia"
2386	dts	Delftia tsuruhatensis	"Prokaryotes;Bacteria;Betaproteobacteria;Delftia"
2387	dhk	Delftia sp. HK171	"Prokaryotes;Bacteria;Betaproteobacteria;Delftia"
2388	dla	Delftia lacustris	"Prokaryotes;Bacteria;Betaproteobacteria;Delftia"
2389	vap	Variovorax paradoxus S110	"Prokaryotes;Bacteria;Betaproteobacteria;Variovorax"
2390	vpe	Variovorax paradoxus EPS	"Prokaryotes;Bacteria;Betaproteobacteria;Variovorax"
2391	vpd	Variovorax paradoxus B4	"Prokaryotes;Bacteria;Betaproteobacteria;Variovorax"
2392	vaa	Variovorax sp. PAMC 28711	"Prokaryotes;Bacteria;Betaproteobacteria;Variovorax"
2393	vbo	Variovorax boronicumulans	"Prokaryotes;Bacteria;Betaproteobacteria;Variovorax"
2394	vam	Variovorax sp. PMC12	"Prokaryotes;Bacteria;Betaproteobacteria;Variovorax"
2395	ctt	Comamonas thiooxydans	"Prokaryotes;Bacteria;Betaproteobacteria;Comamonas"
2396	ctes	Comamonas testosteroni TK102	"Prokaryotes;Bacteria;Betaproteobacteria;Comamonas"
2397	cke	Comamonas kerstersii	"Prokaryotes;Bacteria;Betaproteobacteria;Comamonas"
2398	cser	Comamonas serinivorans	"Prokaryotes;Bacteria;Betaproteobacteria;Comamonas"
2399	cof	Comamonas sp. NLF 7-7	"Prokaryotes;Bacteria;Betaproteobacteria;Comamonas"
2400	adn	Alicycliphilus denitrificans BC	"Prokaryotes;Bacteria;Betaproteobacteria;Alicycliphilus"
2401	adk	Alicycliphilus denitrificans K601	"Prokaryotes;Bacteria;Betaproteobacteria;Alicycliphilus"
2402	rta	Ramlibacter tataouinensis	"Prokaryotes;Bacteria;Betaproteobacteria;Ramlibacter"
2403	cbx	Candidatus Symbiobacter mobilis	"Prokaryotes;Bacteria;Betaproteobacteria;Symbiobacter"
2404	oto	Ottowia sp. oral taxon 894	"Prokaryotes;Bacteria;Betaproteobacteria;Ottowia"
2405	otk	Ottowia oryzae	"Prokaryotes;Bacteria;Betaproteobacteria;Ottowia"
2406	lim	Limnohabitans sp. 103DPR2	"Prokaryotes;Bacteria;Betaproteobacteria;Limnohabitans"

2407	lih	Limnohabitans sp. 63ED37-2	"Prokaryotes;Bacteria;Betaproteobacteria;Limnohabitans"
2408	hyr	Hydrogenophaga sp. RAC07	"Prokaryotes;Bacteria;Betaproteobacteria;Hydrogenophaga"
2409	hyb	Hydrogenophaga sp. PBC	"Prokaryotes;Bacteria;Betaproteobacteria;Hydrogenophaga"
2410	hyl	Hydrogenophaga sp. LPB0072	"Prokaryotes;Bacteria;Betaproteobacteria;Hydrogenophaga"
2411	hyc	Hydrogenophaga sp. PAMC20947	"Prokaryotes;Bacteria;Betaproteobacteria;Hydrogenophaga"
2412	hpse	Hydrogenophaga pseudoflava	"Prokaryotes;Bacteria;Betaproteobacteria;Hydrogenophaga"
2413	hyn	Hydrogenophaga sp. BPS33	"Prokaryotes;Bacteria;Betaproteobacteria;Hydrogenophaga"
2414	dpy	Diaphorobacter polyhydroxybutyrativorans	"Prokaryotes;Bacteria;Betaproteobacteria;Diaphorobacter"
2415	dih	Diaphorobacter sp. HDW4A	"Prokaryotes;Bacteria;Betaproteobacteria;Diaphorobacter"
2416	daer	Diaphorobacter aerolatus	"Prokaryotes;Bacteria;Betaproteobacteria;Diaphorobacter"
2417	drg	Diaphorobacter ruginosibacter	"Prokaryotes;Bacteria;Betaproteobacteria;Diaphorobacter"
2418	simp	Simplicispira suum	"Prokaryotes;Bacteria;Betaproteobacteria;Simplicispira"
2419	melm	Pulveribacter suum	"Prokaryotes;Bacteria;Betaproteobacteria;Melaminivora"
2420	mela	Melaminivora sp. SC2-9	"Prokaryotes;Bacteria;Betaproteobacteria;Melaminivora"
2421	cbaa	Serpentinomonas raichei	"Prokaryotes;Bacteria;Betaproteobacteria;Serpentinomonas"
2422	cbab	Serpentinomonas mccroryi	"Prokaryotes;Bacteria;Betaproteobacteria;Serpentinomonas"
2423	sthm	Schlegelella thermodepolymerans	"Prokaryotes;Bacteria;Betaproteobacteria;Schlegelella"
2424	mpt	Methylibium petroleiphilum	"Prokaryotes;Bacteria;Betaproteobacteria;Methylibium"
2425	metp	Methylibium sp. Pch-M	"Prokaryotes;Bacteria;Betaproteobacteria;Methylibium"
2426	har	Herminiimonas arsenicoxydans	"Prokaryotes;Bacteria;Betaproteobacteria;Herminiimonas"
2427	mms	Janthinobacterium sp. Marseille	"Prokaryotes;Bacteria;Betaproteobacteria;Janthinobacterium"
2428	jag	Janthinobacterium agaricidamnosum	"Prokaryotes;Bacteria;Betaproteobacteria;Janthinobacterium"
2429	jab	Janthinobacterium sp. B9-8	"Prokaryotes;Bacteria;Betaproteobacteria;Janthinobacterium"
2430	jaz	Janthinobacterium sp. 1_2014MBL_MicDiv	"Prokaryotes;Bacteria;Betaproteobacteria;Janthinobacterium"
2431	jal	Janthinobacterium sp. LM6	"Prokaryotes;Bacteria;Betaproteobacteria;Janthinobacterium"
2432	jsv	Janthinobacterium svalbardensis	"Prokaryotes;Bacteria;Betaproteobacteria;Janthinobacterium"
2433	jaj	Janthinobacterium sp. 17J80-10	"Prokaryotes;Bacteria;Betaproteobacteria;Janthinobacterium"
2434	jas	Janthinobacterium tructae	"Prokaryotes;Bacteria;Betaproteobacteria;Janthinobacterium"
2435	jlv	Janthinobacterium lividum	"Prokaryotes;Bacteria;Betaproteobacteria;Janthinobacterium"
2436	hse	Herbaspirillum seropedicae SmR1	"Prokaryotes;Bacteria;Betaproteobacteria;Herbaspirillum"
2437	hsz	Herbaspirillum seropedicae Z67	"Prokaryotes;Bacteria;Betaproteobacteria;Herbaspirillum"
2438	hht	Herbaspirillum hiltneri	"Prokaryotes;Bacteria;Betaproteobacteria;Herbaspirillum"
2439	hrb	Herbaspirillum rubrisubalbicans	"Prokaryotes;Bacteria;Betaproteobacteria;Herbaspirillum"
2440	hee	Herbaspirillum sp. meg3	"Prokaryotes;Bacteria;Betaproteobacteria;Herbaspirillum"
2441	hhf	Herbaspirillum huttiense	"Prokaryotes;Bacteria;Betaproteobacteria;Herbaspirillum"
2442	hfr	Herbaspirillum frisingense	"Prokaryotes;Bacteria;Betaproteobacteria;Herbaspirillum"
2443	zin	Candidatus Zinderia insecticola	"Prokaryotes;Bacteria;Betaproteobacteria;Zinderia"
2444	cfu	Collimonas fungivorans	"Prokaryotes;Bacteria;Betaproteobacteria;Collimonas"
2445	care	Collimonas arenae	"Prokaryotes;Bacteria;Betaproteobacteria;Collimonas"
2446	cpra	Collimonas pratensis	"Prokaryotes;Bacteria;Betaproteobacteria;Collimonas"
2447	mnr	Massilia sp. NR 4-1	"Prokaryotes;Bacteria;Betaproteobacteria;Massilia"

2448	masw	Massilia sp. WG5	"Prokaryotes;Bacteria;Betaproteobacteria;Massilia"
2449	mass	Massilia violaceinigra	"Prokaryotes;Bacteria;Betaproteobacteria;Massilia"
2450	masz	Massilia armeniaca	"Prokaryotes;Bacteria;Betaproteobacteria;Massilia"
2451	mtim	Massilia oculi	"Prokaryotes;Bacteria;Betaproteobacteria;Massilia"
2452	masy	Massilia sp. YMA4	"Prokaryotes;Bacteria;Betaproteobacteria;Massilia"
2453	mali	Massilia albidiflava	"Prokaryotes;Bacteria;Betaproteobacteria;Massilia"
2454	mum	Massilia umbonata	"Prokaryotes;Bacteria;Betaproteobacteria;Massilia"
2455	mfla	Massilia flava	"Prokaryotes;Bacteria;Betaproteobacteria;Massilia"
2456	mpli	Massilia plicata	"Prokaryotes;Bacteria;Betaproteobacteria;Massilia"
2457	ofo	Oxalobacter formigenes	"Prokaryotes;Bacteria;Betaproteobacteria;Oxalobacter"
2458	upv	Undibacterium parvum	"Prokaryotes;Bacteria;Betaproteobacteria;Undibacterium"
2459	upi	Undibacterium piscinae	"Prokaryotes;Bacteria;Betaproteobacteria;Undibacterium"
2460	nok	Noviherbaspirillum sp. UKPF54	"Prokaryotes;Bacteria;Betaproteobacteria;Noviherbaspirillum"
2461	dug	Duganella sp. AF9R3	"Prokaryotes;Bacteria;Betaproteobacteria;Duganella"
2462	sutt	Sutterella megalosphaeroides	"Prokaryotes;Bacteria;Betaproteobacteria;Sutterella"
2463	sutk	Sutterella faecalis	"Prokaryotes;Bacteria;Betaproteobacteria;Sutterella"
2464	lch	Leptothrix cholodnii	"Prokaryotes;Bacteria;Betaproteobacteria;Leptothrix"
2465	tin	Thiomonas intermedia	"Prokaryotes;Bacteria;Betaproteobacteria;Thiomonas"
2466	thi	Thiomonas arsenitoxydans	"Prokaryotes;Bacteria;Betaproteobacteria;Thiomonas"
2467	rge	Rubrivivax gelatinosus	"Prokaryotes;Bacteria;Betaproteobacteria;Rubrivivax"
2468	rbn	Rubrivivax benzoatilyticus	"Prokaryotes;Bacteria;Betaproteobacteria;Rubrivivax"
2469	rdp	Roseateles depolymerans	"Prokaryotes;Bacteria;Betaproteobacteria;Roseateles"
2470	pkt	Paucibacter sp. KCTC 42545	"Prokaryotes;Bacteria;Betaproteobacteria;Paucibacter"
2471	miu	Mitsuaria sp. 7	"Prokaryotes;Bacteria;Betaproteobacteria;Mitsuaria"
2472	rgu	Rhizobacter gummiphilus	"Prokaryotes;Bacteria;Betaproteobacteria;Rhizobacter"
2473	aon	Aquabacterium olei	"Prokaryotes;Bacteria;Betaproteobacteria;Aquabacterium"
2474	snn	Sphaerotilus natans subsp. sulfidivorans	"Prokaryotes;Bacteria;Betaproteobacteria;Sphaerotilus"
2475	xyk	Xylophilus rhododendri	"Prokaryotes;Bacteria;Betaproteobacteria;Xylophilus"
2476	bbag	Burkholderiales bacterium GJ-E10	"Prokaryotes;Bacteria;Betaproteobacteria;unclassified Burkholderiales"
2477	bbay	Burkholderiales bacterium YL45	"Prokaryotes;Bacteria;Betaproteobacteria;unclassified Burkholderiales"
2478	pbh	Polyangium brachysporum	"Prokaryotes;Bacteria;Betaproteobacteria;unclassified Burkholderiales"
2479	neu	Nitrosomonas europaea	"Prokaryotes;Bacteria;Betaproteobacteria;Nitrosomonas"
2480	net	Nitrosomonas eutropha	"Prokaryotes;Bacteria;Betaproteobacteria;Nitrosomonas"
2481	nit	Nitrosomonas sp. AL212	"Prokaryotes;Bacteria;Betaproteobacteria;Nitrosomonas"
2482	nii	Nitrosomonas sp. Is79A3	"Prokaryotes;Bacteria;Betaproteobacteria;Nitrosomonas"
2483	nco	Nitrosomonas communis	"Prokaryotes;Bacteria;Betaproteobacteria;Nitrosomonas"
2484	nur	Nitrosomonas ureae	"Prokaryotes;Bacteria;Betaproteobacteria;Nitrosomonas"
2485	nst	Nitrosomonas stercoris	"Prokaryotes;Bacteria;Betaproteobacteria;Nitrosomonas"
2486	nmu	Nitrosospira multiformis	"Prokaryotes;Bacteria;Betaproteobacteria;Nitrosospira"
2487	nlc	Nitrosospira lacus	"Prokaryotes;Bacteria;Betaproteobacteria;Nitrosospira"
2488	shd	Sulfuritalea hydrogenivorans	"Prokaryotes;Bacteria;Betaproteobacteria;Sulfuritalea"

2489	metr	Methyloversatilis sp. RAC08	"Prokaryotes;Bacteria;Betaproteobacteria;Methyloversatilis"
2490	doe	Denitratisoma oestradiolicum	"Prokaryotes;Bacteria;Betaproteobacteria;Denitratisoma"
2491	tbd	Thiobacillus denitrificans	"Prokaryotes;Bacteria;Betaproteobacteria;Thiobacillus"
2492	mfa	Methylobacillus flagellatus	"Prokaryotes;Bacteria;Betaproteobacteria;Methylobacillus"
2493	mmb	Methylotenera mobilis	"Prokaryotes;Bacteria;Betaproteobacteria;Methylotenera"
2494	meh	Methylotenera versatilis	"Prokaryotes;Bacteria;Betaproteobacteria;Methylotenera"
2495	mei	Methylovorus glucosetrophus	"Prokaryotes;Bacteria;Betaproteobacteria;Methylovorus"
2496	mep	Methylovorus sp. MP688	"Prokaryotes;Bacteria;Betaproteobacteria;Methylovorus"
2497	mbac	Candidatus Methylopumilus turicensis	"Prokaryotes;Bacteria;Betaproteobacteria;Methylopumilus"
2498	mbat	Candidatus Methylopumilus planktonicus	"Prokaryotes;Bacteria;Betaproteobacteria;Methylopumilus"
2499	meu	Methylophilus sp. TWE2	"Prokaryotes;Bacteria;Betaproteobacteria;Methylophilus"
2500	slt	Sideroxydans lithotrophicus	"Prokaryotes;Bacteria;Betaproteobacteria;Sideroxydans"
2501	gca	Gallionella capsiferriformans	"Prokaryotes;Bacteria;Betaproteobacteria;Gallionella"
2502	fam	Ferriphaselus amnicola	"Prokaryotes;Bacteria;Betaproteobacteria;Ferriphaselus"
2503	nim	Candidatus Nitrotoga sp. AM1P	"Prokaryotes;Bacteria;Betaproteobacteria;Nitrotoga"
2504	sdr	Sulfuricella denitrificans	"Prokaryotes;Bacteria;Betaproteobacteria;Sulfuricella"
2505	sulf	Sulfuriferula sp. AH1	"Prokaryotes;Bacteria;Betaproteobacteria;Sulfuriferula"
2506	splb	Sulfuriferula plumbiphila	"Prokaryotes;Bacteria;Betaproteobacteria;Sulfuriferula"
2507	sniv	Sulfuriferula nivalis	"Prokaryotes;Bacteria;Betaproteobacteria;Sulfuriferula"
2508	slac	Sulfurimicrobium lacus skT11	"Prokaryotes;Bacteria;Betaproteobacteria;Sulfurimicrobium"
2509	uru	Usitatibacter rugosus	"Prokaryotes;Bacteria;Betaproteobacteria;Usitatibacter"
2510	upl	Usitatibacter palustris	"Prokaryotes;Bacteria;Betaproteobacteria;Usitatibacter"
2511	eba	Aromatoleum aromaticum	"Prokaryotes;Bacteria;Betaproteobacteria;Aromatoleum"
2512	dsu	Azospira oryzae	"Prokaryotes;Bacteria;Betaproteobacteria;Azospira"
2513	rbu	Rugosibacter aromaticivorans	"Prokaryotes;Bacteria;Betaproteobacteria;Rugosibacter"
2514	otr	Oryzomicrobium terrae	"Prokaryotes;Bacteria;Betaproteobacteria;Oryzomicrobium"
2515	rbh	Rhodocyclaceae bacterium Thauera-like	"Prokaryotes;Bacteria;Betaproteobacteria;unclassified Rhodocyclaceae"
2516	dar	Dechloromonas aromatica	"Prokaryotes;Bacteria;Betaproteobacteria;Dechloromonas"
2517	dey	Dechloromonas sp. HYN0024	"Prokaryotes;Bacteria;Betaproteobacteria;Dechloromonas"
2518	azo	Azoarcus olearius BH72	"Prokaryotes;Bacteria;Betaproteobacteria;Azoarcus"
2519	aoa	Azoarcus olearius DQS4	"Prokaryotes;Bacteria;Betaproteobacteria;Azoarcus"
2520	aza	Azoarcus sp. KH32C	"Prokaryotes;Bacteria;Betaproteobacteria;Azoarcus"
2521	azi	Azoarcus sp. CIB	"Prokaryotes;Bacteria;Betaproteobacteria;Azoarcus"
2522	atw	Azoarcus pumilus	"Prokaryotes;Bacteria;Betaproteobacteria;Azoarcus"
2523	acom	Azoarcus communis	"Prokaryotes;Bacteria;Betaproteobacteria;Azoarcus"
2524	azd	Azoarcus sp. DN11	"Prokaryotes;Bacteria;Betaproteobacteria;Azoarcus"
2525	azr	Azoarcus sp. DD4	"Prokaryotes;Bacteria;Betaproteobacteria;Azoarcus"
2526	azq	Azoarcus sp. M9-3-2	"Prokaryotes;Bacteria;Betaproteobacteria;Azoarcus"
2527	tmz	Thauera sp. MZ1T	"Prokaryotes;Bacteria;Betaproteobacteria;Thauera"
2528	thu	Thauera humireducens	"Prokaryotes;Bacteria;Betaproteobacteria;Thauera"
2529	tcl	Thauera chlorobenzoica	"Prokaryotes;Bacteria;Betaproteobacteria;Thauera"

2530	thk	Thauera sp. K11	"Prokaryotes;Bacteria;Betaproteobacteria;Thauera"
2531	tak	Thauera aromatica	"Prokaryotes;Bacteria;Betaproteobacteria;Thauera"
2532	zpa	Zoogloeaceae bacteirum Par-f-2	"Prokaryotes;Bacteria;Betaproteobacteria;unclassified Zoogloeaceae"
2533	fpho	Fluviibacter phosphoraccumulans	"Prokaryotes;Bacteria;Betaproteobacteria;Fluviibacter"
2534	fmy	Ferrovum myxofaciens	"Prokaryotes;Bacteria;Betaproteobacteria;Ferrovum"
2535	app	Accumulibacter phosphatis	"Prokaryotes;Bacteria;Betaproteobacteria;Accumulibacter"
2536	tpn	Candidatus Tremblaya princeps PCIT	"Prokaryotes;Bacteria;Betaproteobacteria;Tremblaya"
2537	tpq	Candidatus Tremblaya princeps PCVAL	"Prokaryotes;Bacteria;Betaproteobacteria;Tremblaya"
2538	tpj	Candidatus Tremblaya phenacola	"Prokaryotes;Bacteria;Betaproteobacteria;Tremblaya"
2539	kci	Candidatus Kinetoplastibacterium crithidii (ex Angomonas deanei ATCC 30255)	"Prokaryotes;Bacteria;Betaproteobacteria;Kinetoplastibacterium"
2540	kct	Candidatus Kinetoplastibacterium crithidii TCC036E	"Prokaryotes;Bacteria;Betaproteobacteria;Kinetoplastibacterium"
2541	kbl	Candidatus Kinetoplastibacterium blastocrithidii (ex Strigomonas culicis)	"Prokaryotes;Bacteria;Betaproteobacteria;Kinetoplastibacterium"
2542	kbt	Candidatus Kinetoplastibacterium blastocrithidii TCC012E	"Prokaryotes;Bacteria;Betaproteobacteria;Kinetoplastibacterium"
2543	kde	Candidatus Kinetoplastibacterium desouzaii	"Prokaryotes;Bacteria;Betaproteobacteria;Kinetoplastibacterium"
2544	kga	Candidatus Kinetoplastibacterium galatii	"Prokaryotes;Bacteria;Betaproteobacteria;Kinetoplastibacterium"
2545	kon	Candidatus Kinetoplastibacterium oncopeltii	"Prokaryotes;Bacteria;Betaproteobacteria;Kinetoplastibacterium"
2546	kso	Candidatus Kinetoplastibacterium sorsogonicusi	"Prokaryotes;Bacteria;Betaproteobacteria;Kinetoplastibacterium"
2547	ssdc	Candidatus Profftella armatura	"Prokaryotes;Bacteria;Betaproteobacteria;Profftella"
2548	ndl	Candidatus Nasuia deltocephalinicola	"Prokaryotes;Bacteria;Betaproteobacteria;Nasuia"
2549	vfg	Candidatus Vidania fulgoroideae	"Prokaryotes;Bacteria;Betaproteobacteria;Vidania"
2550	bprc	Beta proteobacterium CB	"Prokaryotes;Bacteria;Betaproteobacteria;unclassified Betaproteobacteria"
2551	beb	Betaproteobacteria bacterium UKL13-2	"Prokaryotes;Bacteria;Betaproteobacteria;unclassified Betaproteobacteria"
2552	beba	Betaproteobacteria bacterium GR16-43	"Prokaryotes;Bacteria;Betaproteobacteria;unclassified Betaproteobacteria"
2553	hpy	Helicobacter pylori 26695	"Prokaryotes;Bacteria;Epsilonproteobacteria;Helicobacter"
2554	heo	Helicobacter pylori 26695	"Prokaryotes;Bacteria;Epsilonproteobacteria;Helicobacter"
2555	hpj	Helicobacter pylori J99	"Prokaryotes;Bacteria;Epsilonproteobacteria;Helicobacter"
2556	hpa	Helicobacter pylori HPAG1	"Prokaryotes;Bacteria;Epsilonproteobacteria;Helicobacter"
2557	hps	Helicobacter pylori Shi470	"Prokaryotes;Bacteria;Epsilonproteobacteria;Helicobacter"
2558	hhp	Helicobacter pylori Shi112	"Prokaryotes;Bacteria;Epsilonproteobacteria;Helicobacter"
2559	hhq	Helicobacter pylori Shi169	"Prokaryotes;Bacteria;Epsilonproteobacteria;Helicobacter"
2560	hhr	Helicobacter pylori Shi417	"Prokaryotes;Bacteria;Epsilonproteobacteria;Helicobacter"
2561	hpg	Helicobacter pylori G27	"Prokaryotes;Bacteria;Epsilonproteobacteria;Helicobacter"
2562	hpp	Helicobacter pylori P12	"Prokaryotes;Bacteria;Epsilonproteobacteria;Helicobacter"
2563	hpb	Helicobacter pylori B38	"Prokaryotes;Bacteria;Epsilonproteobacteria;Helicobacter"
2564	hpl	Helicobacter pylori B8	"Prokaryotes;Bacteria;Epsilonproteobacteria;Helicobacter"
2565	hpc	Helicobacter pylori PeCan4	"Prokaryotes;Bacteria;Epsilonproteobacteria;Helicobacter"
2566	hca	Helicobacter pylori PeCan18	"Prokaryotes;Bacteria;Epsilonproteobacteria;Helicobacter"
2567	hpm	Helicobacter pylori SJM180	"Prokaryotes;Bacteria;Epsilonproteobacteria;Helicobacter"
2568	hpe	Helicobacter pylori ELS37	"Prokaryotes;Bacteria;Epsilonproteobacteria;Helicobacter"
2569	hpo	Helicobacter pylori 35A	"Prokaryotes;Bacteria;Epsilonproteobacteria;Helicobacter"

2570	hpi	Helicobacter pylori 908	"Prokaryotes;Bacteria;Epsilonproteobacteria;Helicobacter"
2571	hpq	Helicobacter pylori 2017	"Prokaryotes;Bacteria;Epsilonproteobacteria;Helicobacter"
2572	hpw	Helicobacter pylori 2018	"Prokaryotes;Bacteria;Epsilonproteobacteria;Helicobacter"
2573	hpu	Helicobacter pylori Cuz20	"Prokaryotes;Bacteria;Epsilonproteobacteria;Helicobacter"
2574	hef	Helicobacter pylori F16	"Prokaryotes;Bacteria;Epsilonproteobacteria;Helicobacter"
2575	hpf	Helicobacter pylori F30	"Prokaryotes;Bacteria;Epsilonproteobacteria;Helicobacter"
2576	heq	Helicobacter pylori F32	"Prokaryotes;Bacteria;Epsilonproteobacteria;Helicobacter"
2577	hex	Helicobacter pylori F57	"Prokaryotes;Bacteria;Epsilonproteobacteria;Helicobacter"
2578	hpt	Helicobacter pylori Sat464	"Prokaryotes;Bacteria;Epsilonproteobacteria;Helicobacter"
2579	hpz	Helicobacter pylori 52	"Prokaryotes;Bacteria;Epsilonproteobacteria;Helicobacter"
2580	hpv	Helicobacter pylori v225d	"Prokaryotes;Bacteria;Epsilonproteobacteria;Helicobacter"
2581	hpx	Helicobacter pylori 83	"Prokaryotes;Bacteria;Epsilonproteobacteria;Helicobacter"
2582	hen	Helicobacter pylori SNT49	"Prokaryotes;Bacteria;Epsilonproteobacteria;Helicobacter"
2583	hph	Helicobacter pylori Lithuania75	"Prokaryotes;Bacteria;Epsilonproteobacteria;Helicobacter"
2584	heg	Helicobacter pylori Gambia94/24	"Prokaryotes;Bacteria;Epsilonproteobacteria;Helicobacter"
2585	hpn	Helicobacter pylori India7	"Prokaryotes;Bacteria;Epsilonproteobacteria;Helicobacter"
2586	hep	Helicobacter pylori Puno120	"Prokaryotes;Bacteria;Epsilonproteobacteria;Helicobacter"
2587	heu	Helicobacter pylori Puno135	"Prokaryotes;Bacteria;Epsilonproteobacteria;Helicobacter"
2588	hes	Helicobacter pylori SouthAfrica7	"Prokaryotes;Bacteria;Epsilonproteobacteria;Helicobacter"
2589	hpys	Helicobacter pylori SouthAfrica20	"Prokaryotes;Bacteria;Epsilonproteobacteria;Helicobacter"
2590	hcn	Helicobacter pylori HUP-B14	"Prokaryotes;Bacteria;Epsilonproteobacteria;Helicobacter"
2591	hpd	Helicobacter pylori 51	"Prokaryotes;Bacteria;Epsilonproteobacteria;Helicobacter"
2592	hey	Helicobacter pylori XZ274	"Prokaryotes;Bacteria;Epsilonproteobacteria;Helicobacter"
2593	her	Helicobacter pylori Rif1	"Prokaryotes;Bacteria;Epsilonproteobacteria;Helicobacter"
2594	hei	Helicobacter pylori Rif2	"Prokaryotes;Bacteria;Epsilonproteobacteria;Helicobacter"
2595	hpya	Helicobacter pylori Aklavik117	"Prokaryotes;Bacteria;Epsilonproteobacteria;Helicobacter"
2596	hpyk	Helicobacter pylori Aklavik86	"Prokaryotes;Bacteria;Epsilonproteobacteria;Helicobacter"
2597	hpyo	Helicobacter pylori OK113	"Prokaryotes;Bacteria;Epsilonproteobacteria;Helicobacter"
2598	hpyl	Helicobacter pylori OK310	"Prokaryotes;Bacteria;Epsilonproteobacteria;Helicobacter"
2599	hpyb	Helicobacter pylori oki102	"Prokaryotes;Bacteria;Epsilonproteobacteria;Helicobacter"
2600	hpyc	Helicobacter pylori oki112	"Prokaryotes;Bacteria;Epsilonproteobacteria;Helicobacter"
2601	hpyd	Helicobacter pylori oki128	"Prokaryotes;Bacteria;Epsilonproteobacteria;Helicobacter"
2602	hpye	Helicobacter pylori oki154	"Prokaryotes;Bacteria;Epsilonproteobacteria;Helicobacter"
2603	hpyf	Helicobacter pylori oki422	"Prokaryotes;Bacteria;Epsilonproteobacteria;Helicobacter"
2604	hpyg	Helicobacter pylori oki673	"Prokaryotes;Bacteria;Epsilonproteobacteria;Helicobacter"
2605	hpyh	Helicobacter pylori oki828	"Prokaryotes;Bacteria;Epsilonproteobacteria;Helicobacter"
2606	hpyj	Helicobacter pylori oki898	"Prokaryotes;Bacteria;Epsilonproteobacteria;Helicobacter"
2607	hpyr	Helicobacter pylori UM032	"Prokaryotes;Bacteria;Epsilonproteobacteria;Helicobacter"
2608	hpyi	Helicobacter pylori UM037	"Prokaryotes;Bacteria;Epsilonproteobacteria;Helicobacter"
2609	hpyu	Helicobacter pylori UM066	"Prokaryotes;Bacteria;Epsilonproteobacteria;Helicobacter"
2610	hpym	Helicobacter pylori UM299	"Prokaryotes;Bacteria;Epsilonproteobacteria;Helicobacter"

2611	hem	Helicobacter pylori UM298	"Prokaryotes;Bacteria;Epsilonproteobacteria;Helicobacter"
2612	heb	Helicobacter pylori BM012A	"Prokaryotes;Bacteria;Epsilonproteobacteria;Helicobacter"
2613	hez	Helicobacter pylori BM012S	"Prokaryotes;Bacteria;Epsilonproteobacteria;Helicobacter"
2614	hhe	Helicobacter hepaticus	"Prokaryotes;Bacteria;Epsilonproteobacteria;Helicobacter"
2615	hac	Helicobacter acinonychis	"Prokaryotes;Bacteria;Epsilonproteobacteria;Helicobacter"
2616	hms	Helicobacter mustelae	"Prokaryotes;Bacteria;Epsilonproteobacteria;Helicobacter"
2617	hfe	Helicobacter felis	"Prokaryotes;Bacteria;Epsilonproteobacteria;Helicobacter"
2618	hbi	Helicobacter bizzozeronii	"Prokaryotes;Bacteria;Epsilonproteobacteria;Helicobacter"
2619	hce	Helicobacter cetorum MIT 00-7128	"Prokaryotes;Bacteria;Epsilonproteobacteria;Helicobacter"
2620	hcm	Helicobacter cetorum MIT 99-5656	"Prokaryotes;Bacteria;Epsilonproteobacteria;Helicobacter"
2621	hcp	Helicobacter cinaedi PAGU611	"Prokaryotes;Bacteria;Epsilonproteobacteria;Helicobacter"
2622	hcb	Helicobacter cinaedi CCUG 18818 = ATCC BAA-847	"Prokaryotes;Bacteria;Epsilonproteobacteria;Helicobacter"
2623	hhm	Helicobacter heilmannii	"Prokaryotes;Bacteria;Epsilonproteobacteria;Helicobacter"
2624	hty	Helicobacter typhlonius	"Prokaryotes;Bacteria;Epsilonproteobacteria;Helicobacter"
2625	hbl	Helicobacter bilis	"Prokaryotes;Bacteria;Epsilonproteobacteria;Helicobacter"
2626	had	Helicobacter apodemus	"Prokaryotes;Bacteria;Epsilonproteobacteria;Helicobacter"
2627	het	Helicobacter sp. MIT 01-6242	"Prokaryotes;Bacteria;Epsilonproteobacteria;Helicobacter"
2628	hcl	Helicobacter cholecystus	"Prokaryotes;Bacteria;Epsilonproteobacteria;Helicobacter"
2629	hwi	Helicobacter winghamensis	"Prokaryotes;Bacteria;Epsilonproteobacteria;Helicobacter"
2630	wsu	Wolinella succinogenes	"Prokaryotes;Bacteria;Epsilonproteobacteria;Wolinella"
2631	tdn	Sulfurimonas denitrificans	"Prokaryotes;Bacteria;Epsilonproteobacteria;Sulfurimonas"
2632	sua	Sulfurimonas autotrophica	"Prokaryotes;Bacteria;Epsilonproteobacteria;Sulfurimonas"
2633	suln	Sulfurimonas sp. 1-1N	"Prokaryotes;Bacteria;Epsilonproteobacteria;Sulfurimonas"
2634	sulg	Sulfurimonas sp. GYSZ_1	"Prokaryotes;Bacteria;Epsilonproteobacteria;Sulfurimonas"
2635	sulc	Sulfurimonas sp. CVO	"Prokaryotes;Bacteria;Epsilonproteobacteria;Sulfurimonas"
2636	spal	Sulfurimonas paralvinellae	"Prokaryotes;Bacteria;Epsilonproteobacteria;Sulfurimonas"
2637	sku	Sulfuricurvum kujiense	"Prokaryotes;Bacteria;Epsilonproteobacteria;Sulfuricurvum"
2638	sulr	Candidatus Sulfuricurvum sp. RIFRC-1	"Prokaryotes;Bacteria;Epsilonproteobacteria;Sulfuricurvum"
2639	cje	Campylobacter jejuni subsp. jejuni NCTC 11168 = ATCC 700819	"Prokaryotes;Bacteria;Epsilonproteobacteria;Campylobacter"
2640	cjb	Campylobacter jejuni subsp. jejuni NCTC 11168-BN148	"Prokaryotes;Bacteria;Epsilonproteobacteria;Campylobacter"
2641	cjj	Campylobacter jejuni subsp. jejuni 81-176	"Prokaryotes;Bacteria;Epsilonproteobacteria;Campylobacter"
2642	cju	Campylobacter jejuni subsp. jejuni 81116	"Prokaryotes;Bacteria;Epsilonproteobacteria;Campylobacter"
2643	cjn	Campylobacter jejuni subsp. jejuni ICDCCJ07001	"Prokaryotes;Bacteria;Epsilonproteobacteria;Campylobacter"
2644	cji	Campylobacter jejuni subsp. jejuni IA3902	"Prokaryotes;Bacteria;Epsilonproteobacteria;Campylobacter"
2645	cjm	Campylobacter jejuni subsp. jejuni M1	"Prokaryotes;Bacteria;Epsilonproteobacteria;Campylobacter"
2646	cjs	Campylobacter jejuni subsp. jejuni S3	"Prokaryotes;Bacteria;Epsilonproteobacteria;Campylobacter"
2647	cjp	Campylobacter jejuni subsp. jejuni PT14	"Prokaryotes;Bacteria;Epsilonproteobacteria;Campylobacter"
2648	cjej	Campylobacter jejuni subsp. jejuni 00-2426	"Prokaryotes;Bacteria;Epsilonproteobacteria;Campylobacter"
2649	cjeu	Campylobacter jejuni subsp. jejuni 00-2538	"Prokaryotes;Bacteria;Epsilonproteobacteria;Campylobacter"
2650	cjen	Campylobacter jejuni subsp. jejuni 00-2544	"Prokaryotes;Bacteria;Epsilonproteobacteria;Campylobacter"
2651	cjei	Campylobacter jejuni subsp. jejuni 00-2425	"Prokaryotes;Bacteria;Epsilonproteobacteria;Campylobacter"

2652	cjer	Campylobacter jejuni subsp. jejuni R14	"Prokaryotes;Bacteria;Epsilonproteobacteria;Campylobacter"
2653	cjv	Campylobacter jejuni subsp. jejuni MTVDSCj20	"Prokaryotes;Bacteria;Epsilonproteobacteria;Campylobacter"
2654	cjy	Campylobacter jejuni subsp. jejuni YH001	"Prokaryotes;Bacteria;Epsilonproteobacteria;Campylobacter"
2655	cjq	Campylobacter jejuni subsp. jejuni 35925B2	"Prokaryotes;Bacteria;Epsilonproteobacteria;Campylobacter"
2656	cjl	Campylobacter jejuni subsp. jejuni 00-1597	"Prokaryotes;Bacteria;Epsilonproteobacteria;Campylobacter"
2657	cjw	Campylobacter jejuni subsp. jejuni 00-6200	"Prokaryotes;Bacteria;Epsilonproteobacteria;Campylobacter"
2658	cjr	Campylobacter jejuni RM1221	"Prokaryotes;Bacteria;Epsilonproteobacteria;Campylobacter"
2659	cjd	Campylobacter jejuni subsp. doylei 269.97	"Prokaryotes;Bacteria;Epsilonproteobacteria;Campylobacter"
2660	cjz	Campylobacter jejuni 32488	"Prokaryotes;Bacteria;Epsilonproteobacteria;Campylobacter"
2661	cjx	Campylobacter jejuni 4031	"Prokaryotes;Bacteria;Epsilonproteobacteria;Campylobacter"
2662	cff	Campylobacter fetus subsp. fetus 82-40	"Prokaryotes;Bacteria;Epsilonproteobacteria;Campylobacter"
2663	cft	Campylobacter fetus subsp. fetus 04/554	"Prokaryotes;Bacteria;Epsilonproteobacteria;Campylobacter"
2664	cfv	Campylobacter fetus subsp. venerealis cfvi03/293	"Prokaryotes;Bacteria;Epsilonproteobacteria;Campylobacter"
2665	cfx	Campylobacter fetus subsp. venerealis 97/608	"Prokaryotes;Bacteria;Epsilonproteobacteria;Campylobacter"
2666	cfz	Campylobacter fetus subsp. venerealis 84-112	"Prokaryotes;Bacteria;Epsilonproteobacteria;Campylobacter"
2667	camp	Campylobacter fetus subsp. testudinum 03-427	"Prokaryotes;Bacteria;Epsilonproteobacteria;Campylobacter"
2668	cfp	Campylobacter fetus subsp. testudinum pet-3	"Prokaryotes;Bacteria;Epsilonproteobacteria;Campylobacter"
2669	ccv	Campylobacter curvus	"Prokaryotes;Bacteria;Epsilonproteobacteria;Campylobacter"
2670	cha	Campylobacter hominis	"Prokaryotes;Bacteria;Epsilonproteobacteria;Campylobacter"
2671	cco	Campylobacter concisus 13826	"Prokaryotes;Bacteria;Epsilonproteobacteria;Campylobacter"
2672	ccoc	Campylobacter concisus ATCC 33237	"Prokaryotes;Bacteria;Epsilonproteobacteria;Campylobacter"
2673	cla	Campylobacter lari RM2100	"Prokaryotes;Bacteria;Epsilonproteobacteria;Campylobacter"
2674	clr	Campylobacter lari RM16701	"Prokaryotes;Bacteria;Epsilonproteobacteria;Campylobacter"
2675	clm	Campylobacter lari RM16712	"Prokaryotes;Bacteria;Epsilonproteobacteria;Campylobacter"
2676	clq	Campylobacter lari CCUG 22395	"Prokaryotes;Bacteria;Epsilonproteobacteria;Campylobacter"
2677	cln	Campylobacter lari NCTC 11845	"Prokaryotes;Bacteria;Epsilonproteobacteria;Campylobacter"
2678	cll	Campylobacter lari subsp. concheus LMG 11760	"Prokaryotes;Bacteria;Epsilonproteobacteria;Campylobacter"
2679	ccol	Campylobacter coli 76339	"Prokaryotes;Bacteria;Epsilonproteobacteria;Campylobacter"
2680	ccc	Campylobacter coli CVM N29710	"Prokaryotes;Bacteria;Epsilonproteobacteria;Campylobacter"
2681	ccq	Campylobacter coli 15-537360	"Prokaryotes;Bacteria;Epsilonproteobacteria;Campylobacter"
2682	ccf	Campylobacter coli RM1875	"Prokaryotes;Bacteria;Epsilonproteobacteria;Campylobacter"
2683	ccy	Campylobacter coli RM4661	"Prokaryotes;Bacteria;Epsilonproteobacteria;Campylobacter"
2684	ccoi	Campylobacter coli RM5611	"Prokaryotes;Bacteria;Epsilonproteobacteria;Campylobacter"
2685	ccof	Campylobacter coli FB1	"Prokaryotes;Bacteria;Epsilonproteobacteria;Campylobacter"
2686	ccoo	Campylobacter coli OR12	"Prokaryotes;Bacteria;Epsilonproteobacteria;Campylobacter"
2687	caj	Campylobacter iguaniorum	"Prokaryotes;Bacteria;Epsilonproteobacteria;Campylobacter"
2688	cis	Campylobacter insulaenigrae	"Prokaryotes;Bacteria;Epsilonproteobacteria;Campylobacter"
2689	cvo	Campylobacter volucris	"Prokaryotes;Bacteria;Epsilonproteobacteria;Campylobacter"
2690	cpel	Campylobacter peloridis	"Prokaryotes;Bacteria;Epsilonproteobacteria;Campylobacter"
2691	camr	Campylobacter sp. RM16704	"Prokaryotes;Bacteria;Epsilonproteobacteria;Campylobacter"
2692	csm	Campylobacter subantarcticus LMG 24374	"Prokaryotes;Bacteria;Epsilonproteobacteria;Campylobacter"

2693	csf	Campylobacter subantarcticus LMG 24377	"Prokaryotes;Bacteria;Epsilonproteobacteria;Campylobacter"
2694	cgra	Campylobacter gracilis	"Prokaryotes;Bacteria;Epsilonproteobacteria;Campylobacter"
2695	cure	Campylobacter ureolyticus	"Prokaryotes;Bacteria;Epsilonproteobacteria;Campylobacter"
2696	chyo	Campylobacter hyointestinalis	"Prokaryotes;Bacteria;Epsilonproteobacteria;Campylobacter"
2697	chv	Campylobacter helveticus	"Prokaryotes;Bacteria;Epsilonproteobacteria;Campylobacter"
2698	cspf	Campylobacter sputorum	"Prokaryotes;Bacteria;Epsilonproteobacteria;Campylobacter"
2699	cpin	Campylobacter pinnipediorum	"Prokaryotes;Bacteria;Epsilonproteobacteria;Campylobacter"
2700	ccun	Campylobacter cuniculorum	"Prokaryotes;Bacteria;Epsilonproteobacteria;Campylobacter"
2701	clx	Campylobacter lanienae	"Prokaryotes;Bacteria;Epsilonproteobacteria;Campylobacter"
2702	cavi	Campylobacter avium	"Prokaryotes;Bacteria;Epsilonproteobacteria;Campylobacter"
2703	chw	Campylobacter hepaticus	"Prokaryotes;Bacteria;Epsilonproteobacteria;Campylobacter"
2704	camz	Campylobacter sp. RM12175	"Prokaryotes;Bacteria;Epsilonproteobacteria;Campylobacter"
2705	camy	Campylobacter sp. RM6137	"Prokaryotes;Bacteria;Epsilonproteobacteria;Campylobacter"
2706	coj	Campylobacter ornithocola	"Prokaryotes;Bacteria;Epsilonproteobacteria;Campylobacter"
2707	cux	Campylobacter upsaliensis	"Prokaryotes;Bacteria;Epsilonproteobacteria;Campylobacter"
2708	crx	Campylobacter rectus	"Prokaryotes;Bacteria;Epsilonproteobacteria;Campylobacter"
2709	cgeo	Campylobacter geochelonis	"Prokaryotes;Bacteria;Epsilonproteobacteria;Campylobacter"
2710	cbla	Campylobacter blaseri	"Prokaryotes;Bacteria;Epsilonproteobacteria;Campylobacter"
2711	ccor	Campylobacter corcagiensis	"Prokaryotes;Bacteria;Epsilonproteobacteria;Campylobacter"
2712	carm	Campylobacter armoricus	"Prokaryotes;Bacteria;Epsilonproteobacteria;Campylobacter"
2713	cmuc	Campylobacter mucosalis	"Prokaryotes;Bacteria;Epsilonproteobacteria;Campylobacter"
2714	csho	Campylobacter showae	"Prokaryotes;Bacteria;Epsilonproteobacteria;Campylobacter"
2715	abu	Arcobacter butzleri RM4018	"Prokaryotes;Bacteria;Epsilonproteobacteria;Aliiarcobacter"
2716	abt	Arcobacter butzleri ED-1	"Prokaryotes;Bacteria;Epsilonproteobacteria;Aliiarcobacter"
2717	abl	Arcobacter butzleri 7h1h	"Prokaryotes;Bacteria;Epsilonproteobacteria;Aliiarcobacter"
2718	ask	Aliarcobacter skirrowii	"Prokaryotes;Bacteria;Epsilonproteobacteria;Aliiarcobacter"
2719	atp	Arcobacter trophiarum	"Prokaryotes;Bacteria;Epsilonproteobacteria;Aliiarcobacter"
2720	acib	Aliarcobacter cibarius	"Prokaryotes;Bacteria;Epsilonproteobacteria;Aliiarcobacter"
2721	acre	Arcobacter cryaerophilus	"Prokaryotes;Bacteria;Epsilonproteobacteria;Aliiarcobacter"
2722	alan	Aliarcobacter lanthieri	"Prokaryotes;Bacteria;Epsilonproteobacteria;Aliiarcobacter"
2723	apoc	Arcobacter porcinus	"Prokaryotes;Bacteria;Epsilonproteobacteria;Aliiarcobacter"
2724	afc	Aliarcobacter faecis	"Prokaryotes;Bacteria;Epsilonproteobacteria;Aliiarcobacter"
2725	ant	Arcobacter nitrofigilis	"Prokaryotes;Bacteria;Epsilonproteobacteria;Arcobacter"
2726	aell	Arcobacter ellisii	"Prokaryotes;Bacteria;Epsilonproteobacteria;Arcobacter"
2727	aaqi	Arcobacter aquimarinus	"Prokaryotes;Bacteria;Epsilonproteobacteria;Arcobacter"
2728	asui	Arcobacter suis	"Prokaryotes;Bacteria;Epsilonproteobacteria;Arcobacter"
2729	aclo	Arcobacter cloacae	"Prokaryotes;Bacteria;Epsilonproteobacteria;Arcobacter"
2730	aana	Arcobacter anaerophilus	"Prokaryotes;Bacteria;Epsilonproteobacteria;Arcobacter"
2731	avp	Arcobacter venerupis	"Prokaryotes;Bacteria;Epsilonproteobacteria;Arcobacter"
2732	adz	Arcobacter defluvii	"Prokaryotes;Bacteria;Epsilonproteobacteria;Arcobacter"
2733	alk	Poseidonibacter lekithochrous	"Prokaryotes;Bacteria;Epsilonproteobacteria;Poseidonibacter"

2734	alp	Poseidonibacter parvus	"Prokaryotes;Bacteria;Epsilonproteobacteria;Poseidonibacter"
2735	ahs	Malaciobacter halophilus	"Prokaryotes;Bacteria;Epsilonproteobacteria;Malaciobacter"
2736	amyt	Malaciobacter mytili	"Prokaryotes;Bacteria;Epsilonproteobacteria;Malaciobacter"
2737	amar	Malaciobacter marinus	"Prokaryotes;Bacteria;Epsilonproteobacteria;Malaciobacter"
2738	acaa	Malaciobacter canalis	"Prokaryotes;Bacteria;Epsilonproteobacteria;Malaciobacter"
2739	amol	Malaciobacter molluscorum	"Prokaryotes;Bacteria;Epsilonproteobacteria;Malaciobacter"
2740	apai	Malaciobacter pacificus	"Prokaryotes;Bacteria;Epsilonproteobacteria;Malaciobacter"
2741	hbv	Halarcobacter bivalviorum	"Prokaryotes;Bacteria;Epsilonproteobacteria;Halarcobacter"
2742	hebr	Halarcobacter ebronensis	"Prokaryotes;Bacteria;Epsilonproteobacteria;Halarcobacter"
2743	paco	Pseudoarcobacter acticola	"Prokaryotes;Bacteria;Epsilonproteobacteria;Pseudoarcobacter"
2744	arc	Arcobacter sp. L	"Prokaryotes;Bacteria;Epsilonproteobacteria;unclassified Arcobacter"
2745	sdl	Sulfurospirillum deleyianum	"Prokaryotes;Bacteria;Epsilonproteobacteria;Sulfurospirillum"
2746	sba	Sulfurospirillum barnesii	"Prokaryotes;Bacteria;Epsilonproteobacteria;Sulfurospirillum"
2747	smul	Sulfurospirillum multivorans	"Prokaryotes;Bacteria;Epsilonproteobacteria;Sulfurospirillum"
2748	shal	Sulfurospirillum halorespirans	"Prokaryotes;Bacteria;Epsilonproteobacteria;Sulfurospirillum"
2749	suls	Sulfurospirillum sp. SL2-1	"Prokaryotes;Bacteria;Epsilonproteobacteria;Sulfurospirillum"
2750	sulj	Sulfurospirillum sp. JPD-1	"Prokaryotes;Bacteria;Epsilonproteobacteria;Sulfurospirillum"
2751	sult	Sulfurospirillum sp. ACSTCE	"Prokaryotes;Bacteria;Epsilonproteobacteria;Sulfurospirillum"
2752	hyo	Hydrogenimonas sp. MAG	"Prokaryotes;Bacteria;Epsilonproteobacteria;Hydrogenimonas"
2753	nsa	Nitratifractor salsuginis	"Prokaryotes;Bacteria;Epsilonproteobacteria;Nitratifractor"
2754	sun	Sulfurovum sp. NBC37-1	"Prokaryotes;Bacteria;Epsilonproteobacteria;Sulfurovum"
2755	slh	Sulfurovum lithotrophicum	"Prokaryotes;Bacteria;Epsilonproteobacteria;Sulfurovum"
2756	nis	Nitratiruptor sp. SB155-2	"Prokaryotes;Bacteria;Epsilonproteobacteria;Nitratiruptor"
2757	nam	Nautilia profundicola	"Prokaryotes;Bacteria;Epsilonproteobacteria;Nautilia"
2758	nap	Nautilia sp. PV-1	"Prokaryotes;Bacteria;Epsilonproteobacteria;Nautilia"
2759	cmed	Caminibacter mediatlanticus	"Prokaryotes;Bacteria;Epsilonproteobacteria;Caminibacter"
2760	cpaf	Cetia pacifica	"Prokaryotes;Bacteria;Epsilonproteobacteria;Cetia"
2761	gsu	Geobacter sulfurreducens PCA	"Prokaryotes;Bacteria;Deltaproteobacteria;Geobacter"
2762	gsk	Geobacter sulfurreducens KN400	"Prokaryotes;Bacteria;Deltaproteobacteria;Geobacter"
2763	gme	Geobacter metallireducens	"Prokaryotes;Bacteria;Deltaproteobacteria;Geobacter"
2764	gur	Geobacter uraniireducens	"Prokaryotes;Bacteria;Deltaproteobacteria;Geobacter"
2765	glo	Geobacter lovleyi	"Prokaryotes;Bacteria;Deltaproteobacteria;Geobacter"
2766	gbm	Geobacter bemidjiensis	"Prokaryotes;Bacteria;Deltaproteobacteria;Geobacter"
2767	geo	Geobacter daltonii FRC-32	"Prokaryotes;Bacteria;Deltaproteobacteria;Geobacter"
2768	gem	Geobacter sp. M21	"Prokaryotes;Bacteria;Deltaproteobacteria;Geobacter"
2769	geb	Geobacter sp. M18	"Prokaryotes;Bacteria;Deltaproteobacteria;Geobacter"
2770	gpi	Geobacter pickeringii	"Prokaryotes;Bacteria;Deltaproteobacteria;Geobacter"
2771	gao	Geobacter anodireducens	"Prokaryotes;Bacteria;Deltaproteobacteria;Geobacter"
2772	gbn	Geobacter bremensis	"Prokaryotes;Bacteria;Deltaproteobacteria;Geobacter"
2773	gsb	Geoalkalibacter subterraneus	"Prokaryotes;Bacteria;Deltaproteobacteria;Geoalkalibacter"
2774	pca	Pelobacter carbinolicus	"Prokaryotes;Bacteria;Deltaproteobacteria;Syntrophotalea"

2775	pace	Syntrophotalea acetylenica	"Prokaryotes;Bacteria;Deltaproteobacteria;Syntrophotalea"
2776	pef	Syntrophotalea acetylenivorans	"Prokaryotes;Bacteria;Deltaproteobacteria;Syntrophotalea"
2777	ppd	Pelobacter propionicus	"Prokaryotes;Bacteria;Deltaproteobacteria;Pelobacter"
2778	des	Desulfuromonas soudanensis	"Prokaryotes;Bacteria;Deltaproteobacteria;Desulfuromonas"
2779	deu	Desulfuromonas sp. DDH964	"Prokaryotes;Bacteria;Deltaproteobacteria;Desulfuromonas"
2780	dvu	Desulfovibrio vulgaris Hildenborough	"Prokaryotes;Bacteria;Deltaproteobacteria;Desulfovibrio"
2781	dvl	Desulfovibrio vulgaris DP4	"Prokaryotes;Bacteria;Deltaproteobacteria;Desulfovibrio"
2782	dvm	Desulfovibrio vulgaris Miyazaki F	"Prokaryotes;Bacteria;Deltaproteobacteria;Desulfovibrio"
2783	dvg	Desulfovibrio vulgaris RCH1	"Prokaryotes;Bacteria;Deltaproteobacteria;Desulfovibrio"
2784	dde	Desulfovibrio alaskensis	"Prokaryotes;Bacteria;Deltaproteobacteria;Desulfovibrio"
2785	dds	Desulfovibrio desulfuricans ATCC 27774	"Prokaryotes;Bacteria;Deltaproteobacteria;Desulfovibrio"
2786	dma	Desulfovibrio magneticus	"Prokaryotes;Bacteria;Deltaproteobacteria;Desulfovibrio"
2787	dgg	Desulfovibrio gigas	"Prokaryotes;Bacteria;Deltaproteobacteria;Desulfovibrio"
2788	dfi	Desulfovibrio fairfieldensis	"Prokaryotes;Bacteria;Deltaproteobacteria;Desulfovibrio"
2789	dpg	Desulfovibrio piger	"Prokaryotes;Bacteria;Deltaproteobacteria;Desulfovibrio"
2790	def	Desulfovibrio sp. G11	"Prokaryotes;Bacteria;Deltaproteobacteria;Desulfovibrio"
2791	dtr	Candidatus Desulfovibrio trichonymphae	"Prokaryotes;Bacteria;Deltaproteobacteria;Desulfovibrio"
2792	dfl	Desulfovibrio ferrophilus	"Prokaryotes;Bacteria;Deltaproteobacteria;Desulfovibrio"
2793	dcb	Desulfovibrio carbinolicus	"Prokaryotes;Bacteria;Deltaproteobacteria;Desulfovibrio"
2794	dms	Desulfovibrio marinus	"Prokaryotes;Bacteria;Deltaproteobacteria;Desulfovibrio"
2795	dsd	Desulfovibrio sulfodismutans	"Prokaryotes;Bacteria;Deltaproteobacteria;Desulfovibrio"
2796	dsa	Desulfovibrio salexigens	"Prokaryotes;Bacteria;Deltaproteobacteria;Maridesulfovibrio"
2797	dhy	Desulfovibrio hydrothermalis	"Prokaryotes;Bacteria;Deltaproteobacteria;Maridesulfovibrio"
2798	daf	Desulfocurvibacter africanus subsp. africanus	"Prokaryotes;Bacteria;Deltaproteobacteria;Desulfocurvibacter"
2799	das	Pseudodesulfovibrio aespoeensis	"Prokaryotes;Bacteria;Deltaproteobacteria;Pseudodesulfovibrio"
2800	dpi	Pseudodesulfovibrio piezophilus	"Prokaryotes;Bacteria;Deltaproteobacteria;Pseudodesulfovibrio"
2801	dej	Pseudodesulfovibrio indicus	"Prokaryotes;Bacteria;Deltaproteobacteria;Pseudodesulfovibrio"
2802	pprf	Pseudodesulfovibrio profundus	"Prokaryotes;Bacteria;Deltaproteobacteria;Pseudodesulfovibrio"
2803	psel	Pseudodesulfovibrio cashew	"Prokaryotes;Bacteria;Deltaproteobacteria;Pseudodesulfovibrio"
2804	ddn	Pseudodesulfovibrio mercurii	"Prokaryotes;Bacteria;Deltaproteobacteria;Pseudodesulfovibrio"
2805	lip	Lawsonia intracellularis PHE/MN1-00	"Prokaryotes;Bacteria;Deltaproteobacteria;Lawsonia"
2806	lir	Lawsonia intracellularis N343	"Prokaryotes;Bacteria;Deltaproteobacteria;Lawsonia"
2807	dsx	Desulfolutivibrio sulfoxidireducens	"Prokaryotes;Bacteria;Deltaproteobacteria;Desulfolutivibrio"
2808	dba	Desulfomicrobium baculatum	"Prokaryotes;Bacteria;Deltaproteobacteria;Desulfomicrobium"
2809	doa	Desulfomicrobium orale	"Prokaryotes;Bacteria;Deltaproteobacteria;Desulfomicrobium"
2810	drt	Desulfohalobium retbaense	"Prokaryotes;Bacteria;Deltaproteobacteria;Desulfohalobium"
2811	dps	Desulfotalea psychrophila	"Prokaryotes;Bacteria;Deltaproteobacteria;Desulfotalea"
2812	dak	Desulfurivibrio alkaliphilus	"Prokaryotes;Bacteria;Deltaproteobacteria;Desulfurivibrio"
2813	dpr	Desulfobulbus propionicus	"Prokaryotes;Bacteria;Deltaproteobacteria;Desulfobulbus"
2814	deo	Desulfobulbus oralis	"Prokaryotes;Bacteria;Deltaproteobacteria;Desulfobulbus"
2815	dog	Desulfobulbus oligotrophicus	"Prokaryotes;Bacteria;Deltaproteobacteria;Desulfobulbus"

2816	dsf	Desulfocapsa sulfexigens	"Prokaryotes;Bacteria;Deltaproteobacteria;Desulfocapsa"
2817	dol	Desulfococcus oleovorans	"Prokaryotes;Bacteria;Deltaproteobacteria;Desulfococcus"
2818	dml	Desulfococcus multivorans	"Prokaryotes;Bacteria;Deltaproteobacteria;Desulfococcus"
2819	dal	Desulfatibacillum aliphaticivorans	"Prokaryotes;Bacteria;Deltaproteobacteria;Desulfatibacillum"
2820	dat	Desulfobacterium autotrophicum	"Prokaryotes;Bacteria;Deltaproteobacteria;Desulfobacterium"
2821	dto	Desulfobacula toluolica	"Prokaryotes;Bacteria;Deltaproteobacteria;Desulfobacula"
2822	dov	Desulfosarcina ovata subsp. sediminis	"Prokaryotes;Bacteria;Deltaproteobacteria;Desulfosarcina"
2823	dwd	Desulfosarcina widdelii	"Prokaryotes;Bacteria;Deltaproteobacteria;Desulfosarcina"
2824	dalk	Desulfosarcina alkanivorans	"Prokaryotes;Bacteria;Deltaproteobacteria;Desulfosarcina"
2825	ade	Anaeromyxobacter dehalogenans 2CP-C	"Prokaryotes;Bacteria;Deltaproteobacteria;Anaeromyxobacter"
2826	acp	Anaeromyxobacter dehalogenans 2CP-1	"Prokaryotes;Bacteria;Deltaproteobacteria;Anaeromyxobacter"
2827	afw	Anaeromyxobacter sp. Fw109-5	"Prokaryotes;Bacteria;Deltaproteobacteria;Anaeromyxobacter"
2828	ank	Anaeromyxobacter sp. K	"Prokaryotes;Bacteria;Deltaproteobacteria;Anaeromyxobacter"
2829	mxa	Myxococcus xanthus	"Prokaryotes;Bacteria;Deltaproteobacteria;Myxococcus"
2830	msd	Myxococcus stipitatus	"Prokaryotes;Bacteria;Deltaproteobacteria;Myxococcus"
2831	mym	Myxococcus hansupus	"Prokaryotes;Bacteria;Deltaproteobacteria;Myxococcus"
2832	mfb	Myxococcus fulvus	"Prokaryotes;Bacteria;Deltaproteobacteria;Myxococcus"
2833	ccx	Corallococcus coralloides	"Prokaryotes;Bacteria;Deltaproteobacteria;Corallococcus"
2834	mfu	Corallococcus macrosporus HW-1	"Prokaryotes;Bacteria;Deltaproteobacteria;Corallococcus"
2835	mmas	Corallococcus macrosporus DSM 14697	"Prokaryotes;Bacteria;Deltaproteobacteria;Corallococcus"
2836	sur	Stigmatella aurantiaca	"Prokaryotes;Bacteria;Deltaproteobacteria;Stigmatella"
2837	age	Archangium gephyra	"Prokaryotes;Bacteria;Deltaproteobacteria;Archangium"
2838	mbd	Melittangium boletus	"Prokaryotes;Bacteria;Deltaproteobacteria;Melittangium"
2839	cfus	Cystobacter fuscus	"Prokaryotes;Bacteria;Deltaproteobacteria;Cystobacter"
2840	vin	Vulgatibacter incomptus	"Prokaryotes;Bacteria;Deltaproteobacteria;Vulgatibacter"
2841	scl	Sorangium cellulosum So ce56	"Prokaryotes;Bacteria;Deltaproteobacteria;Sorangium"
2842	scu	Sorangium cellulosum So0157-2	"Prokaryotes;Bacteria;Deltaproteobacteria;Sorangium"
2843	ccro	Chondromyces crocatus	"Prokaryotes;Bacteria;Deltaproteobacteria;Chondromyces"
2844	samy	Sandaracinus amylolyticus	"Prokaryotes;Bacteria;Deltaproteobacteria;Sandaracinus"
2845	llu	Labilithrix luteola	"Prokaryotes;Bacteria;Deltaproteobacteria;Labilithrix"
2846	mrm	Minicystis rosea	"Prokaryotes;Bacteria;Deltaproteobacteria;Minicystis"
2847	hoh	Haliangium ochraceum	"Prokaryotes;Bacteria;Deltaproteobacteria;Haliangium"
2848	sat	Syntrophus aciditrophicus	"Prokaryotes;Bacteria;Deltaproteobacteria;Syntrophus"
2849	dao	Desulfobacca acetoxidans	"Prokaryotes;Bacteria;Deltaproteobacteria;Desulfobacca"
2850	dti	Desulfomonile tiedjei	"Prokaryotes;Bacteria;Deltaproteobacteria;Desulfomonile"
2851	sfu	Syntrophobacter fumaroxidans	"Prokaryotes;Bacteria;Deltaproteobacteria;Syntrophobacter"
2852	dax	Desulfoglaeba alkanexedens	"Prokaryotes;Bacteria;Deltaproteobacteria;Desulfoglaeba"
2853	dbr	Desulfarculus baarsii	"Prokaryotes;Bacteria;Deltaproteobacteria;Desulfarculus"
2854	hmr	Hippea maritima	"Prokaryotes;Bacteria;Deltaproteobacteria;Hippea"
2855	dav	Desulfurella acetivorans	"Prokaryotes;Bacteria;Deltaproteobacteria;Desulfurella"
2856	bsed	Bradymonas sediminis	"Prokaryotes;Bacteria;Deltaproteobacteria;Bradymonas"

2857	pcay	Persicimonas caeni	"Prokaryotes;Bacteria;Deltaproteobacteria;Persicimonas"
2858	rpr	Rickettsia prowazekii Madrid E	"Prokaryotes;Bacteria;Alphaproteobacteria;Rickettsia"
2859	rpo	Rickettsia prowazekii BuV67-CWPP	"Prokaryotes;Bacteria;Alphaproteobacteria;Rickettsia"
2860	rpw	Rickettsia prowazekii Chernikova	"Prokaryotes;Bacteria;Alphaproteobacteria;Rickettsia"
2861	rpz	Rickettsia prowazekii Dachau	"Prokaryotes;Bacteria;Alphaproteobacteria;Rickettsia"
2862	rpg	Rickettsia prowazekii GvV257	"Prokaryotes;Bacteria;Alphaproteobacteria;Rickettsia"
2863	rps	Rickettsia prowazekii Katsinyian	"Prokaryotes;Bacteria;Alphaproteobacteria;Rickettsia"
2864	rpv	Rickettsia prowazekii RpGvF24	"Prokaryotes;Bacteria;Alphaproteobacteria;Rickettsia"
2865	rpq	Rickettsia prowazekii Rp22	"Prokaryotes;Bacteria;Alphaproteobacteria;Rickettsia"
2866	rpl	Rickettsia prowazekii Breinl	"Prokaryotes;Bacteria;Alphaproteobacteria;Rickettsia"
2867	rpn	Rickettsia prowazekii NMRC Madrid E	"Prokaryotes;Bacteria;Alphaproteobacteria;Rickettsia"
2868	rty	Rickettsia typhi Wilmington	"Prokaryotes;Bacteria;Alphaproteobacteria;Rickettsia"
2869	rtt	Rickettsia typhi TH1527	"Prokaryotes;Bacteria;Alphaproteobacteria;Rickettsia"
2870	rtb	Rickettsia typhi B9991CWPP	"Prokaryotes;Bacteria;Alphaproteobacteria;Rickettsia"
2871	rcm	Rickettsia canadensis McKiel	"Prokaryotes;Bacteria;Alphaproteobacteria;Rickettsia"
2872	rcc	Rickettsia canadensis CA410	"Prokaryotes;Bacteria;Alphaproteobacteria;Rickettsia"
2873	rbe	Rickettsia bellii RML369-C	"Prokaryotes;Bacteria;Alphaproteobacteria;Rickettsia"
2874	rbo	Rickettsia bellii OSU 85-389	"Prokaryotes;Bacteria;Alphaproteobacteria;Rickettsia"
2875	rco	Rickettsia conorii	"Prokaryotes;Bacteria;Alphaproteobacteria;Rickettsia"
2876	rfe	Rickettsia felis	"Prokaryotes;Bacteria;Alphaproteobacteria;Rickettsia"
2877	rak	Rickettsia akari	"Prokaryotes;Bacteria;Alphaproteobacteria;Rickettsia"
2878	rri	Rickettsia rickettsii Sheila Smith	"Prokaryotes;Bacteria;Alphaproteobacteria;Rickettsia"
2879	rrj	Rickettsia rickettsii Iowa	"Prokaryotes;Bacteria;Alphaproteobacteria;Rickettsia"
2880	rra	Rickettsia rickettsii Arizona	"Prokaryotes;Bacteria;Alphaproteobacteria;Rickettsia"
2881	rrc	Rickettsia rickettsii Colombia	"Prokaryotes;Bacteria;Alphaproteobacteria;Rickettsia"
2882	rrh	Rickettsia rickettsii Hauke	"Prokaryotes;Bacteria;Alphaproteobacteria;Rickettsia"
2883	rrb	Rickettsia rickettsii Brazil	"Prokaryotes;Bacteria;Alphaproteobacteria;Rickettsia"
2884	rrn	Rickettsia rickettsii Hino	"Prokaryotes;Bacteria;Alphaproteobacteria;Rickettsia"
2885	rrp	Rickettsia rickettsii Hlp#2	"Prokaryotes;Bacteria;Alphaproteobacteria;Rickettsia"
2886	rrm	Rickettsia rickettsii Morgan	"Prokaryotes;Bacteria;Alphaproteobacteria;Rickettsia"
2887	rrr	Rickettsia rickettsii R	"Prokaryotes;Bacteria;Alphaproteobacteria;Rickettsia"
2888	rms	Rickettsia massiliae MTU5	"Prokaryotes;Bacteria;Alphaproteobacteria;Rickettsia"
2889	rmi	Rickettsia massiliae AZT80	"Prokaryotes;Bacteria;Alphaproteobacteria;Rickettsia"
2890	rpk	Rickettsia peacockii	"Prokaryotes;Bacteria;Alphaproteobacteria;Rickettsia"
2891	raf	Rickettsia africae	"Prokaryotes;Bacteria;Alphaproteobacteria;Rickettsia"
2892	rhe	Rickettsia heilongjiangensis	"Prokaryotes;Bacteria;Alphaproteobacteria;Rickettsia"
2893	rja	Rickettsia japonica	"Prokaryotes;Bacteria;Alphaproteobacteria;Rickettsia"
2894	rsv	Rickettsia slovaca 13-B	"Prokaryotes;Bacteria;Alphaproteobacteria;Rickettsia"
2895	rsw	Rickettsia slovaca D-CWPP	"Prokaryotes;Bacteria;Alphaproteobacteria;Rickettsia"
2896	rph	Rickettsia philipii	"Prokaryotes;Bacteria;Alphaproteobacteria;Rickettsia"
2897	rau	Rickettsia australis	"Prokaryotes;Bacteria;Alphaproteobacteria;Rickettsia"

2898	rmo	Rickettsia montanensis	"Prokaryotes;Bacteria;Alphaproteobacteria;Rickettsia"
2899	rpp	Rickettsia parkeri	"Prokaryotes;Bacteria;Alphaproteobacteria;Rickettsia"
2900	rre	Rickettsia rhipicephali	"Prokaryotes;Bacteria;Alphaproteobacteria;Rickettsia"
2901	ram	Rickettsia amblyommatis GAT-30V	"Prokaryotes;Bacteria;Alphaproteobacteria;Rickettsia"
2902	rab	Rickettsia amblyommatis Ac37	"Prokaryotes;Bacteria;Alphaproteobacteria;Rickettsia"
2903	rmc	Rickettsia monacensis	"Prokaryotes;Bacteria;Alphaproteobacteria;Rickettsia"
2904	ras	Rickettsia asiatica	"Prokaryotes;Bacteria;Alphaproteobacteria;Rickettsia"
2905	ric	Rickettsia sp. MEAM1 (Bemisia tabaci)	"Prokaryotes;Bacteria;Alphaproteobacteria;Rickettsia"
2906	ots	Orientia tsutsugamushi Boryong	"Prokaryotes;Bacteria;Alphaproteobacteria;Orientia"
2907	ott	Orientia tsutsugamushi Ikeda	"Prokaryotes;Bacteria;Alphaproteobacteria;Orientia"
2908	ptc	Candidatus Phycorickettsia trachydisci	"Prokaryotes;Bacteria;Alphaproteobacteria;Phycorickettsia"
2909	wol	Wolbachia endosymbiont of Drosophila melanogaster	"Prokaryotes;Bacteria;Alphaproteobacteria;Wolbachia"
2910	wri	Wolbachia sp. wRi	"Prokaryotes;Bacteria;Alphaproteobacteria;Wolbachia"
2911	wen	Wolbachia endosymbiont of Drosophila simulans wHa	"Prokaryotes;Bacteria;Alphaproteobacteria;Wolbachia"
2912	wed	Wolbachia endosymbiont of Drosophila simulans wNo	"Prokaryotes;Bacteria;Alphaproteobacteria;Wolbachia"
2913	wpi	Wolbachia endosymbiont of Culex quinquefasciatus Pel	"Prokaryotes;Bacteria;Alphaproteobacteria;Wolbachia"
2914	wbm	Wolbachia endosymbiont strain TRS of Brugia malayi	"Prokaryotes;Bacteria;Alphaproteobacteria;Wolbachia"
2915	woo	Wolbachia endosymbiont of Onchocerca ochengi	"Prokaryotes;Bacteria;Alphaproteobacteria;Wolbachia"
2916	wcl	Wolbachia endosymbiont of Cimex lectularius	"Prokaryotes;Bacteria;Alphaproteobacteria;Wolbachia"
2917	weo	Wolbachia endosymbiont of Folsomia candida	"Prokaryotes;Bacteria;Alphaproteobacteria;Wolbachia"
2918	wpp	Wolbachia pipientis	"Prokaryotes;Bacteria;Alphaproteobacteria;Wolbachia"
2919	ama	Anaplasma marginale St. Maries	"Prokaryotes;Bacteria;Alphaproteobacteria;Anaplasma"
2920	amf	Anaplasma marginale Florida	"Prokaryotes;Bacteria;Alphaproteobacteria;Anaplasma"
2921	amw	Anaplasma marginale Dawn	"Prokaryotes;Bacteria;Alphaproteobacteria;Anaplasma"
2922	amp	Anaplasma marginale Gypsy Plains	"Prokaryotes;Bacteria;Alphaproteobacteria;Anaplasma"
2923	acn	Anaplasma centrale	"Prokaryotes;Bacteria;Alphaproteobacteria;Anaplasma"
2924	aph	Anaplasma phagocytophilum HZ	"Prokaryotes;Bacteria;Alphaproteobacteria;Anaplasma"
2925	apy	Anaplasma phagocytophilum HZ2	"Prokaryotes;Bacteria;Alphaproteobacteria;Anaplasma"
2926	apd	Anaplasma phagocytophilum Dog2	"Prokaryotes;Bacteria;Alphaproteobacteria;Anaplasma"
2927	apha	Anaplasma phagocytophilum JM	"Prokaryotes;Bacteria;Alphaproteobacteria;Anaplasma"
2928	aoh	Anaplasma ovis	"Prokaryotes;Bacteria;Alphaproteobacteria;Anaplasma"
2929	eru	Ehrlichia ruminantium Welgevonden (South Africa)	"Prokaryotes;Bacteria;Alphaproteobacteria;Ehrlichia"
2930	erw	Ehrlichia ruminantium Welgevonden (France)	"Prokaryotes;Bacteria;Alphaproteobacteria;Ehrlichia"
2931	erg	Ehrlichia ruminantium Gardel	"Prokaryotes;Bacteria;Alphaproteobacteria;Ehrlichia"
2932	ecn	Ehrlichia canis	"Prokaryotes;Bacteria;Alphaproteobacteria;Ehrlichia"
2933	ech	Ehrlichia chaffeensis Arkansas	"Prokaryotes;Bacteria;Alphaproteobacteria;Ehrlichia"
2934	echa	Ehrlichia chaffeensis Heartland	"Prokaryotes;Bacteria;Alphaproteobacteria;Ehrlichia"
2935	echj	Ehrlichia chaffeensis Jax	"Prokaryotes;Bacteria;Alphaproteobacteria;Ehrlichia"
2936	echl	Ehrlichia chaffeensis Liberty	"Prokaryotes;Bacteria;Alphaproteobacteria;Ehrlichia"
2937	echs	Ehrlichia chaffeensis Osceola	"Prokaryotes;Bacteria;Alphaproteobacteria;Ehrlichia"
2938	echv	Ehrlichia chaffeensis Saint Vincent	"Prokaryotes;Bacteria;Alphaproteobacteria;Ehrlichia"

2939	echw	Ehrlichia chaffeensis Wakulla	"Prokaryotes;Bacteria;Alphaproteobacteria;Ehrlichia"
2940	echp	Ehrlichia chaffeensis West Paces	"Prokaryotes;Bacteria;Alphaproteobacteria;Ehrlichia"
2941	emr	Ehrlichia muris	"Prokaryotes;Bacteria;Alphaproteobacteria;Ehrlichia"
2942	ehh	Ehrlichia sp. HF	"Prokaryotes;Bacteria;Alphaproteobacteria;Ehrlichia"
2943	nse	Neorickettsia sennetsu	"Prokaryotes;Bacteria;Alphaproteobacteria;Neorickettsia"
2944	nri	Neorickettsia risticii	"Prokaryotes;Bacteria;Alphaproteobacteria;Neorickettsia"
2945	nhm	Neorickettsia helminthoeca	"Prokaryotes;Bacteria;Alphaproteobacteria;Neorickettsia"
2946	nef	Neorickettsia findlayensis	"Prokaryotes;Bacteria;Alphaproteobacteria;Neorickettsia"
2947	mmn	Candidatus Midichloria mitochondrii	"Prokaryotes;Bacteria;Alphaproteobacteria;Midichloria"
2948	fso	Candidatus Fokinia solitaria	"Prokaryotes;Bacteria;Alphaproteobacteria;Fokinia"
2949	rbt	Rickettsiales bacterium Ac37b	"Prokaryotes;Bacteria;Alphaproteobacteria;unclassified Rickettsiales"
2950	ren	Rickettsiales endosymbiont of Stachyamoeba lipophora	"Prokaryotes;Bacteria;Alphaproteobacteria;unclassified Rickettsiales"
2951	paca	Candidatus Paracaedibacter acanthamoebae	"Prokaryotes;Bacteria;Alphaproteobacteria;Paracaedibacter"
2952	caq	Candidatus Paracaedimonas acanthamoebae	"Prokaryotes;Bacteria;Alphaproteobacteria;Paracaedimonas"
2953	naf	Candidatus Nucleicultrix amoebiphila	"Prokaryotes;Bacteria;Alphaproteobacteria;Nucleicultrix"
2954	eaa	Endosymbiont of Acanthamoeba sp. UWC8	"Prokaryotes;Bacteria;Alphaproteobacteria;unclassified Holosporaceae"
2955	mlo	Mesorhizobium japonicum MAFF 303099	"Prokaryotes;Bacteria;Alphaproteobacteria;Mesorhizobium"
2956	mln	Mesorhizobium loti NZP2037	"Prokaryotes;Bacteria;Alphaproteobacteria;Mesorhizobium"
2957	mci	Mesorhizobium ciceri biovar biserrulae	"Prokaryotes;Bacteria;Alphaproteobacteria;Mesorhizobium"
2958	mop	Mesorhizobium opportunistum	"Prokaryotes;Bacteria;Alphaproteobacteria;Mesorhizobium"
2959	mam	Mesorhizobium australicum	"Prokaryotes;Bacteria;Alphaproteobacteria;Mesorhizobium"
2960	mamo	Mesorhizobium amorphae	"Prokaryotes;Bacteria;Alphaproteobacteria;Mesorhizobium"
2961	meso	Mesorhizobium sp. B7	"Prokaryotes;Bacteria;Alphaproteobacteria;Mesorhizobium"
2962	mesw	Mesorhizobium sp. WSM1497	"Prokaryotes;Bacteria;Alphaproteobacteria;Mesorhizobium"
2963	mesm	Mesorhizobium sp. M9A.F.Ca.ET.002.03.1.2	"Prokaryotes;Bacteria;Alphaproteobacteria;Mesorhizobium"
2964	mesp	Mesorhizobium sp. Pch-S	"Prokaryotes;Bacteria;Alphaproteobacteria;Mesorhizobium"
2965	mhua	Mesorhizobium huakuii	"Prokaryotes;Bacteria;Alphaproteobacteria;Mesorhizobium"
2966	mjr	Mesorhizobium jarvisii	"Prokaryotes;Bacteria;Alphaproteobacteria;Mesorhizobium"
2967	merd	Mesorhizobium erdmanii	"Prokaryotes;Bacteria;Alphaproteobacteria;Mesorhizobium"
2968	mes	Chelativorans sp. BNC1	"Prokaryotes;Bacteria;Alphaproteobacteria;Chelativorans"
2969	hoe	Hoeflea sp. IMCC20628	"Prokaryotes;Bacteria;Alphaproteobacteria;Hoeflea"
2970	aak	Aminobacter aminovorans	"Prokaryotes;Bacteria;Alphaproteobacteria;Aminobacter"
2971	amih	Aminobacter sp. MSH1	"Prokaryotes;Bacteria;Alphaproteobacteria;Aminobacter"
2972	pht	Phyllobacterium zundukense	"Prokaryotes;Bacteria;Alphaproteobacteria;Phyllobacterium"
2973	rpod	Roseitalea porphyridii	"Prokaryotes;Bacteria;Alphaproteobacteria;Roseitalea"
2974	niy	Nitratireductor sp. SY7	"Prokaryotes;Bacteria;Alphaproteobacteria;Nitratireductor"
2975	orm	Oricola thermophila	"Prokaryotes;Bacteria;Alphaproteobacteria;Oricola"
2976	pla	Parvibaculum lavamentivorans	"Prokaryotes;Bacteria;Alphaproteobacteria;Parvibaculum"
2977	pmob	Pyruvatibacter mobilis	"Prokaryotes;Bacteria;Alphaproteobacteria;Pyruvatibacter"
2978	rbs	Rhodobiaceae bacterium SMS8	"Prokaryotes;Bacteria;Alphaproteobacteria;unclassified Rhodobiaceae"
2979	sme	Sinorhizobium meliloti 1021	"Prokaryotes;Bacteria;Alphaproteobacteria;Sinorhizobium"

2980	smk	Sinorhizobium meliloti AK83	"Prokaryotes;Bacteria;Alphaproteobacteria;Sinorhizobium"
2981	smq	Sinorhizobium meliloti BL225C	"Prokaryotes;Bacteria;Alphaproteobacteria;Sinorhizobium"
2982	smx	Sinorhizobium meliloti SM11	"Prokaryotes;Bacteria;Alphaproteobacteria;Sinorhizobium"
2983	smi	Sinorhizobium meliloti Rm41	"Prokaryotes;Bacteria;Alphaproteobacteria;Sinorhizobium"
2984	smeg	Sinorhizobium meliloti GR4	"Prokaryotes;Bacteria;Alphaproteobacteria;Sinorhizobium"
2985	smel	Sinorhizobium meliloti 2011	"Prokaryotes;Bacteria;Alphaproteobacteria;Sinorhizobium"
2986	smer	Sinorhizobium meliloti RMO17	"Prokaryotes;Bacteria;Alphaproteobacteria;Sinorhizobium"
2987	smd	Sinorhizobium medicae	"Prokaryotes;Bacteria;Alphaproteobacteria;Sinorhizobium"
2988	rhi	Sinorhizobium fredii NGR234	"Prokaryotes;Bacteria;Alphaproteobacteria;Sinorhizobium"
2989	sfh	Sinorhizobium fredii HH103	"Prokaryotes;Bacteria;Alphaproteobacteria;Sinorhizobium"
2990	sfd	Sinorhizobium fredii USDA 257	"Prokaryotes;Bacteria;Alphaproteobacteria;Sinorhizobium"
2991	six	Sinorhizobium sp. RAC02	"Prokaryotes;Bacteria;Alphaproteobacteria;Sinorhizobium"
2992	same	Sinorhizobium americanum	"Prokaryotes;Bacteria;Alphaproteobacteria;Sinorhizobium"
2993	sino	Sinorhizobium sp. CCBAU 05631	"Prokaryotes;Bacteria;Alphaproteobacteria;Sinorhizobium"
2994	ead	Ensifer adhaerens OV14	"Prokaryotes;Bacteria;Alphaproteobacteria;Ensifer"
2995	eah	Ensifer adhaerens Casida A	"Prokaryotes;Bacteria;Alphaproteobacteria;Ensifer"
2996	esj	Ensifer sojae	"Prokaryotes;Bacteria;Alphaproteobacteria;Ensifer"
2997	eak	Ensifer alkalisoli	"Prokaryotes;Bacteria;Alphaproteobacteria;Ensifer"
2998	emx	Ensifer mexicanus	"Prokaryotes;Bacteria;Alphaproteobacteria;Ensifer"
2999	atu	Agrobacterium fabrum	"Prokaryotes;Bacteria;Alphaproteobacteria;Agrobacterium"
3000	ara	Agrobacterium radiobacter	"Prokaryotes;Bacteria;Alphaproteobacteria;Agrobacterium"
3001	ata	Agrobacterium tumefaciens S33	"Prokaryotes;Bacteria;Alphaproteobacteria;Agrobacterium"
3002	agr	Agrobacterium fabacearum H13-3	"Prokaryotes;Bacteria;Alphaproteobacteria;Agrobacterium"
3003	atf	Agrobacterium fabacearum Ach5	"Prokaryotes;Bacteria;Alphaproteobacteria;Agrobacterium"
3004	avi	Agrobacterium vitis	"Prokaryotes;Bacteria;Alphaproteobacteria;Agrobacterium"
3005	agc	Agrobacterium sp. RAC06	"Prokaryotes;Bacteria;Alphaproteobacteria;Agrobacterium"
3006	aro	Agrobacterium rhizogenes	"Prokaryotes;Bacteria;Alphaproteobacteria;Agrobacterium"
3007	agt	Agrobacterium sp. 33MFTa1.1	"Prokaryotes;Bacteria;Alphaproteobacteria;Agrobacterium"
3008	alf	Agrobacterium larrymoorei	"Prokaryotes;Bacteria;Alphaproteobacteria;Agrobacterium"
3009	ret	Rhizobium etli CFN 42	"Prokaryotes;Bacteria;Alphaproteobacteria;Rhizobium"
3010	rec	Rhizobium etli CIAT 652	"Prokaryotes;Bacteria;Alphaproteobacteria;Rhizobium"
3011	rel	Rhizobium etli bv. mimosae Mim1	"Prokaryotes;Bacteria;Alphaproteobacteria;Rhizobium"
3012	rep	Rhizobium etli bv. phaseoli IE4803	"Prokaryotes;Bacteria;Alphaproteobacteria;Rhizobium"
3013	rei	Rhizobium sp. IE4771	"Prokaryotes;Bacteria;Alphaproteobacteria;Rhizobium"
3014	rle	Rhizobium leguminosarum bv. viciae 3841	"Prokaryotes;Bacteria;Alphaproteobacteria;Rhizobium"
3015	rlt	Rhizobium leguminosarum bv. trifolii WSM2304	"Prokaryotes;Bacteria;Alphaproteobacteria;Rhizobium"
3016	rlg	Rhizobium leguminosarum bv. trifolii WSM1325	"Prokaryotes;Bacteria;Alphaproteobacteria;Rhizobium"
3017	rlb	Rhizobium leguminosarum bv. trifolii WSM1689	"Prokaryotes;Bacteria;Alphaproteobacteria;Rhizobium"
3018	rlu	Rhizobium leguminosarum bv. trifolii CB782	"Prokaryotes;Bacteria;Alphaproteobacteria;Rhizobium"
3019	rtr	Rhizobium tropici	"Prokaryotes;Bacteria;Alphaproteobacteria;Rhizobium"
3020	rir	Rhizobium pusense IRBG74	"Prokaryotes;Bacteria;Alphaproteobacteria;Rhizobium"

3021	rpus	Rhizobium pusense CFBP5875	"Prokaryotes;Bacteria;Alphaproteobacteria;Rhizobium"
3022	rhl	Rhizobium favelukesii	"Prokaryotes;Bacteria;Alphaproteobacteria;Rhizobium"
3023	rga	Rhizobium gallicum	"Prokaryotes;Bacteria;Alphaproteobacteria;Rhizobium"
3024	rhn	Rhizobium sp. N1341	"Prokaryotes;Bacteria;Alphaproteobacteria;Rhizobium"
3025	rpha	Rhizobium phaseoli	"Prokaryotes;Bacteria;Alphaproteobacteria;Rhizobium"
3026	rhx	Rhizobium sp. N731	"Prokaryotes;Bacteria;Alphaproteobacteria;Rhizobium"
3027	rhv	Rhizobium sp. S41	"Prokaryotes;Bacteria;Alphaproteobacteria;Rhizobium"
3028	rhk	Rhizobium sp. Kim5	"Prokaryotes;Bacteria;Alphaproteobacteria;Rhizobium"
3029	rez	Rhizobium esperanzae	"Prokaryotes;Bacteria;Alphaproteobacteria;Rhizobium"
3030	rjg	Rhizobium jaguaris	"Prokaryotes;Bacteria;Alphaproteobacteria;Rhizobium"
3031	rhr	Rhizobium sp. 11515TR	"Prokaryotes;Bacteria;Alphaproteobacteria;Rhizobium"
3032	rgr	Rhizobium grahamii	"Prokaryotes;Bacteria;Alphaproteobacteria;Rhizobium"
3033	rad	Rhizobium acidisoli	"Prokaryotes;Bacteria;Alphaproteobacteria;Rhizobium"
3034	roy	Rhizobium oryzihabitans	"Prokaryotes;Bacteria;Alphaproteobacteria;Rhizobium"
3035	rii	Rhizobium indicum	"Prokaryotes;Bacteria;Alphaproteobacteria;Rhizobium"
3036	ngl	Neorhizobium galegae bv. officinalis bv. officinalis HAMBI 1141	"Prokaryotes;Bacteria;Alphaproteobacteria;Neorhizobium"
3037	ngg	Neorhizobium galegae bv. orientalis HAMBI 540	"Prokaryotes;Bacteria;Alphaproteobacteria;Neorhizobium"
3038	neo	Neorhizobium sp. SOG26	"Prokaryotes;Bacteria;Alphaproteobacteria;Neorhizobium"
3039	nen	Neorhizobium sp. NCHU2750	"Prokaryotes;Bacteria;Alphaproteobacteria;Neorhizobium"
3040	rht	Pseudorhizobium banfieldiae	"Prokaryotes;Bacteria;Alphaproteobacteria;Pseudorhizobium"
3041	las	Candidatus Liberibacter asiaticus psy62	"Prokaryotes;Bacteria;Alphaproteobacteria;Liberibacter"
3042	laa	Candidatus Liberibacter asiaticus gxpsy	"Prokaryotes;Bacteria;Alphaproteobacteria;Liberibacter"
3043	lat	Candidatus Liberibacter asiaticus Ishi-1	"Prokaryotes;Bacteria;Alphaproteobacteria;Liberibacter"
3044	lso	Candidatus Liberibacter solanacearum	"Prokaryotes;Bacteria;Alphaproteobacteria;Liberibacter"
3045	lcc	Liberibacter crescens	"Prokaryotes;Bacteria;Alphaproteobacteria;Liberibacter"
3046	lar	Candidatus Liberibacter americanus	"Prokaryotes;Bacteria;Alphaproteobacteria;Liberibacter"
3047	lau	Candidatus Liberibacter africanus	"Prokaryotes;Bacteria;Alphaproteobacteria;Liberibacter"
3048	shz	Shinella sp. HZN7	"Prokaryotes;Bacteria;Alphaproteobacteria;Shinella"
3049	abaw	Georhizobium profundi	"Prokaryotes;Bacteria;Alphaproteobacteria;Georhizobium"
3050	kai	Kaistia sp. 32K	"Prokaryotes;Bacteria;Alphaproteobacteria;Kaistia"
3051	bme	Brucella melitensis bv. 1 16M	"Prokaryotes;Bacteria;Alphaproteobacteria;Brucella"
3052	bmel	Brucella melitensis bv. 1 16M	"Prokaryotes;Bacteria;Alphaproteobacteria;Brucella"
3053	bmi	Brucella melitensis ATCC 23457	"Prokaryotes;Bacteria;Alphaproteobacteria;Brucella"
3054	bmz	Brucella melitensis M28	"Prokaryotes;Bacteria;Alphaproteobacteria;Brucella"
3055	bmg	Brucella melitensis M5-90	"Prokaryotes;Bacteria;Alphaproteobacteria;Brucella"
3056	bmw	Brucella melitensis NI	"Prokaryotes;Bacteria;Alphaproteobacteria;Brucella"
3057	bmee	Brucella melitensis bv. 3 Ether	"Prokaryotes;Bacteria;Alphaproteobacteria;Brucella"
3058	bmf	Brucella abortus 2308	"Prokaryotes;Bacteria;Alphaproteobacteria;Brucella"
3059	bmb	Brucella abortus bv. 1 9-941	"Prokaryotes;Bacteria;Alphaproteobacteria;Brucella"
3060	bmc	Brucella abortus S19	"Prokaryotes;Bacteria;Alphaproteobacteria;Brucella"
3061	baa	Brucella abortus A13334	"Prokaryotes;Bacteria;Alphaproteobacteria;Brucella"

3062	babo	Brucella abortus bv. 2 86/8/59	"Prokaryotes;Bacteria;Alphaproteobacteria;Brucella"
3063	babr	Brucella abortus bv. 6 870	"Prokaryotes;Bacteria;Alphaproteobacteria;Brucella"
3064	babt	Brucella abortus 63 75	"Prokaryotes;Bacteria;Alphaproteobacteria;Brucella"
3065	babb	Brucella abortus BDW	"Prokaryotes;Bacteria;Alphaproteobacteria;Brucella"
3066	babu	Brucella abortus bv. 9 C68	"Prokaryotes;Bacteria;Alphaproteobacteria;Brucella"
3067	babs	Brucella abortus BER	"Prokaryotes;Bacteria;Alphaproteobacteria;Brucella"
3068	babc	Brucella abortus NCTC 10505	"Prokaryotes;Bacteria;Alphaproteobacteria;Brucella"
3069	bms	Brucella suis 1330	"Prokaryotes;Bacteria;Alphaproteobacteria;Brucella"
3070	bsi	Brucella suis 1330	"Prokaryotes;Bacteria;Alphaproteobacteria;Brucella"
3071	bsf	Brucella suis bv. 1	"Prokaryotes;Bacteria;Alphaproteobacteria;Brucella"
3072	bsui	Brucella suis bv. 2 Bs143CITA	"Prokaryotes;Bacteria;Alphaproteobacteria;Brucella"
3073	bsup	Brucella suis bv. 2 PT09143	"Prokaryotes;Bacteria;Alphaproteobacteria;Brucella"
3074	bsuv	Brucella suis bv. 2 PT09172	"Prokaryotes;Bacteria;Alphaproteobacteria;Brucella"
3075	bsuc	Brucella suis bv. 2 Bs364CITA	"Prokaryotes;Bacteria;Alphaproteobacteria;Brucella"
3076	bmt	Brucella suis ATCC 23445	"Prokaryotes;Bacteria;Alphaproteobacteria;Brucella"
3077	bsz	Brucella suis bv. 3	"Prokaryotes;Bacteria;Alphaproteobacteria;Brucella"
3078	bsv	Brucella suis VBI22	"Prokaryotes;Bacteria;Alphaproteobacteria;Brucella"
3079	bsw	Brucella suis ZW043	"Prokaryotes;Bacteria;Alphaproteobacteria;Brucella"
3080	bsg	Brucella suis ZW046	"Prokaryotes;Bacteria;Alphaproteobacteria;Brucella"
3081	bov	Brucella ovis	"Prokaryotes;Bacteria;Alphaproteobacteria;Brucella"
3082	bcs	Brucella canis ATCC 23365	"Prokaryotes;Bacteria;Alphaproteobacteria;Brucella"
3083	bsk	Brucella canis HSK A52141	"Prokaryotes;Bacteria;Alphaproteobacteria;Brucella"
3084	bol	Brucella canis Oliveri	"Prokaryotes;Bacteria;Alphaproteobacteria;Brucella"
3085	bcar	Brucella canis RM6/66	"Prokaryotes;Bacteria;Alphaproteobacteria;Brucella"
3086	bcas	Brucella canis SVA13	"Prokaryotes;Bacteria;Alphaproteobacteria;Brucella"
3087	bmr	Brucella microti	"Prokaryotes;Bacteria;Alphaproteobacteria;Brucella"
3088	bpp	Brucella pinnipedialis B2/94	"Prokaryotes;Bacteria;Alphaproteobacteria;Brucella"
3089	bpv	Brucella pinnipedialis 6/566	"Prokaryotes;Bacteria;Alphaproteobacteria;Brucella"
3090	bcet	Brucella ceti TE10759-12	"Prokaryotes;Bacteria;Alphaproteobacteria;Brucella"
3091	bcee	Brucella ceti TE28753-12	"Prokaryotes;Bacteria;Alphaproteobacteria;Brucella"
3092	bvl	Brucella vulpis	"Prokaryotes;Bacteria;Alphaproteobacteria;Brucella"
3093	bru	Brucella sp. 2002734562	"Prokaryotes;Bacteria;Alphaproteobacteria;Brucella"
3094	brj	Brucella sp. 09RB8471	"Prokaryotes;Bacteria;Alphaproteobacteria;Brucella"
3095	oin	Brucella intermedia	"Prokaryotes;Bacteria;Alphaproteobacteria;Brucella"
3096	oan	Ochrobactrum anthropi ATCC 49188	"Prokaryotes;Bacteria;Alphaproteobacteria;Brucella"
3097	oah	Brucella anthropi	"Prokaryotes;Bacteria;Alphaproteobacteria;Brucella"
3098	ops	Brucella pseudogrignonensis	"Prokaryotes;Bacteria;Alphaproteobacteria;Brucella"
3099	och	Ochrobactrum quorumnocens	"Prokaryotes;Bacteria;Alphaproteobacteria;Brucella"
3100	bja	Bradyrhizobium diazoefficiens USDA 110	"Prokaryotes;Bacteria;Alphaproteobacteria;Bradyrhizobium"
3101	bju	Bradyrhizobium japonicum USDA 6	"Prokaryotes;Bacteria;Alphaproteobacteria;Bradyrhizobium"
3102	bjp	Bradyrhizobium japonicum E109	"Prokaryotes;Bacteria;Alphaproteobacteria;Bradyrhizobium"

3103	bra	Bradyrhizobium sp. ORS 278	"Prokaryotes;Bacteria;Alphaproteobacteria;Bradyrhizobium"
3104	bbt	Bradyrhizobium sp. BTAi1	"Prokaryotes;Bacteria;Alphaproteobacteria;Bradyrhizobium"
3105	brs	Bradyrhizobium cosmicum	"Prokaryotes;Bacteria;Alphaproteobacteria;Bradyrhizobium"
3106	aol	Bradyrhizobium oligotrophicum	"Prokaryotes;Bacteria;Alphaproteobacteria;Bradyrhizobium"
3107	brc	Bradyrhizobium sp. CCGE-LA001	"Prokaryotes;Bacteria;Alphaproteobacteria;Bradyrhizobium"
3108	brad	Bradyrhizobium sp. BF49	"Prokaryotes;Bacteria;Alphaproteobacteria;Bradyrhizobium"
3109	bic	Bradyrhizobium icense	"Prokaryotes;Bacteria;Alphaproteobacteria;Bradyrhizobium"
3110	bro	Bradyrhizobium sp. ORS 285	"Prokaryotes;Bacteria;Alphaproteobacteria;Bradyrhizobium"
3111	brk	Bradyrhizobium sp. SK17	"Prokaryotes;Bacteria;Alphaproteobacteria;Bradyrhizobium"
3112	bot	Bradyrhizobium ottawaense	"Prokaryotes;Bacteria;Alphaproteobacteria;Bradyrhizobium"
3113	brq	Bradyrhizobium amphicarpaeae	"Prokaryotes;Bacteria;Alphaproteobacteria;Bradyrhizobium"
3114	bgq	Bradyrhizobium guangdongense	"Prokaryotes;Bacteria;Alphaproteobacteria;Bradyrhizobium"
3115	bgz	Bradyrhizobium guangzhouense	"Prokaryotes;Bacteria;Alphaproteobacteria;Bradyrhizobium"
3116	bsym	Bradyrhizobium symbiodeficiens	"Prokaryotes;Bacteria;Alphaproteobacteria;Bradyrhizobium"
3117	bbet	Bradyrhizobium betae	"Prokaryotes;Bacteria;Alphaproteobacteria;Bradyrhizobium"
3118	barh	Bradyrhizobium arachidis	"Prokaryotes;Bacteria;Alphaproteobacteria;Bradyrhizobium"
3119	bvz	Bradyrhizobium vignae	"Prokaryotes;Bacteria;Alphaproteobacteria;Bradyrhizobium"
3120	rpa	Rhodopseudomonas palustris CGA009	"Prokaryotes;Bacteria;Alphaproteobacteria;Rhodopseudomonas"
3121	rpb	Rhodopseudomonas palustris HaA2	"Prokaryotes;Bacteria;Alphaproteobacteria;Rhodopseudomonas"
3122	rpc	Rhodopseudomonas palustris BisB18	"Prokaryotes;Bacteria;Alphaproteobacteria;Rhodopseudomonas"
3123	rpd	Rhodopseudomonas palustris BisB5	"Prokaryotes;Bacteria;Alphaproteobacteria;Rhodopseudomonas"
3124	rpe	Rhodopseudomonas palustris BisA53	"Prokaryotes;Bacteria;Alphaproteobacteria;Rhodopseudomonas"
3125	rpt	Rhodopseudomonas palustris TIE-1	"Prokaryotes;Bacteria;Alphaproteobacteria;Rhodopseudomonas"
3126	rpx	Rhodopseudomonas palustris DX-1	"Prokaryotes;Bacteria;Alphaproteobacteria;Rhodopseudomonas"
3127	nwi	Nitrobacter winogradskyi	"Prokaryotes;Bacteria;Alphaproteobacteria;Nitrobacter"
3128	nha	Nitrobacter hamburgensis	"Prokaryotes;Bacteria;Alphaproteobacteria;Nitrobacter"
3129	oca	Afipia carboxidovorans OM5 (Mississippi)	"Prokaryotes;Bacteria;Alphaproteobacteria;Afipia"
3130	ocg	Afipia carboxidovorans OM5 (Goettingen)	"Prokaryotes;Bacteria;Alphaproteobacteria;Afipia"
3131	oco	Afipia carboxidovorans OM4	"Prokaryotes;Bacteria;Alphaproteobacteria;Afipia"
3132	bop	Bosea sp. PAMC 26642	"Prokaryotes;Bacteria;Alphaproteobacteria;Bosea"
3133	bos	Bosea sp. RAC05	"Prokaryotes;Bacteria;Alphaproteobacteria;Bosea"
3134	bvv	Bosea vaviloviae	"Prokaryotes;Bacteria;Alphaproteobacteria;Bosea"
3135	boi	Bosea sp. Tri-49	"Prokaryotes;Bacteria;Alphaproteobacteria;Bosea"
3136	bof	Bosea sp. F3-2	"Prokaryotes;Bacteria;Alphaproteobacteria;Bosea"
3137	vgo	Variibacter gotjawalensis	"Prokaryotes;Bacteria;Alphaproteobacteria;Variibacter"
3138	trb	Tardiphaga robiniae	"Prokaryotes;Bacteria;Alphaproteobacteria;Tardiphaga"
3139	bhe	Bartonella henselae Houston-1	"Prokaryotes;Bacteria;Alphaproteobacteria;Bartonella"
3140	bhn	Bartonella henselae BM1374163	"Prokaryotes;Bacteria;Alphaproteobacteria;Bartonella"
3141	bhs	Bartonella henselae BM1374165	"Prokaryotes;Bacteria;Alphaproteobacteria;Bartonella"
3142	bqu	Bartonella quintana Toulouse	"Prokaryotes;Bacteria;Alphaproteobacteria;Bartonella"
3143	bqr	Bartonella quintana RM-11	"Prokaryotes;Bacteria;Alphaproteobacteria;Bartonella"

3144	bbk	Bartonella bacilliformis	"Prokaryotes;Bacteria;Alphaproteobacteria;Bartonella"
3145	btr	Bartonella tribocorum CIP 105476	"Prokaryotes;Bacteria;Alphaproteobacteria;Bartonella"
3146	btx	Bartonella tribocorum BM1374166	"Prokaryotes;Bacteria;Alphaproteobacteria;Bartonella"
3147	bgr	Bartonella grahamii	"Prokaryotes;Bacteria;Alphaproteobacteria;Bartonella"
3148	bcd	Bartonella clarridgeiae	"Prokaryotes;Bacteria;Alphaproteobacteria;Bartonella"
3149	baus	Bartonella australis	"Prokaryotes;Bacteria;Alphaproteobacteria;Bartonella"
3150	bvn	Bartonella vinsonii subsp. berkhoffii	"Prokaryotes;Bacteria;Alphaproteobacteria;Bartonella"
3151	banc	Bartonella ancashensis	"Prokaryotes;Bacteria;Alphaproteobacteria;Bartonella"
3152	bapi	Bartonella apis	"Prokaryotes;Bacteria;Alphaproteobacteria;Bartonella"
3153	bart	Bartonella sp. JB15	"Prokaryotes;Bacteria;Alphaproteobacteria;Bartonella"
3154	bara	Bartonella sp. A1379B	"Prokaryotes;Bacteria;Alphaproteobacteria;Bartonella"
3155	barw	Bartonella sp. WD16.2	"Prokaryotes;Bacteria;Alphaproteobacteria;Bartonella"
3156	barr	Bartonella sp. Raccoon60	"Prokaryotes;Bacteria;Alphaproteobacteria;Bartonella"
3157	baro	Bartonella sp. 1-1C	"Prokaryotes;Bacteria;Alphaproteobacteria;Bartonella"
3158	barj	Bartonella sp. JB63	"Prokaryotes;Bacteria;Alphaproteobacteria;Bartonella"
3159	bez	Bartonella elizabethae	"Prokaryotes;Bacteria;Alphaproteobacteria;Bartonella"
3160	barn	Bartonella krasnovii OE 1-1	"Prokaryotes;Bacteria;Alphaproteobacteria;Bartonella"
3161	bky	Bartonella kosoy	"Prokaryotes;Bacteria;Alphaproteobacteria;Bartonella"
3162	bals	Bartonella alsatica	"Prokaryotes;Bacteria;Alphaproteobacteria;Bartonella"
3163	xau	Xanthobacter autotrophicus	"Prokaryotes;Bacteria;Alphaproteobacteria;Xanthobacter"
3164	azc	Azorhizobium caulinodans	"Prokaryotes;Bacteria;Alphaproteobacteria;Azorhizobium"
3165	sno	Starkeya novella	"Prokaryotes;Bacteria;Alphaproteobacteria;Starkeya"
3166	star	Starkeya sp. ORNL1	"Prokaryotes;Bacteria;Alphaproteobacteria;Starkeya"
3167	lne	Labrys sp. KNU-23	"Prokaryotes;Bacteria;Alphaproteobacteria;Labrys"
3168	anc	Ancylobacter sp. TS-1	"Prokaryotes;Bacteria;Alphaproteobacteria;Ancylobacter"
3169	apra	Ancylobacter pratisalsi	"Prokaryotes;Bacteria;Alphaproteobacteria;Ancylobacter"
3170	mea	Methylorubrum extorquens AM1	"Prokaryotes;Bacteria;Alphaproteobacteria;Methylorubrum"
3171	mdi	Methylorubrum extorquens DM4	"Prokaryotes;Bacteria;Alphaproteobacteria;Methylorubrum"
3172	mex	Methylorubrum extorquens PA1	"Prokaryotes;Bacteria;Alphaproteobacteria;Methylorubrum"
3173	mch	Methylorubrum extorquens CM4	"Prokaryotes;Bacteria;Alphaproteobacteria;Methylorubrum"
3174	mpo	Methylorubrum populi	"Prokaryotes;Bacteria;Alphaproteobacteria;Methylorubrum"
3175	mza	Methylorubrum zatmanii	"Prokaryotes;Bacteria;Alphaproteobacteria;Methylorubrum"
3176	mrd	Methylobacterium radiotolerans	"Prokaryotes;Bacteria;Alphaproteobacteria;Methylobacterium"
3177	met	Methylobacterium sp. 4-46	"Prokaryotes;Bacteria;Alphaproteobacteria;Methylobacterium"
3178	mno	Methylobacterium nodulans	"Prokaryotes;Bacteria;Alphaproteobacteria;Methylobacterium"
3179	mor	Methylobacterium oryzae	"Prokaryotes;Bacteria;Alphaproteobacteria;Methylobacterium"
3180	meta	Methylobacterium sp. AMS5	"Prokaryotes;Bacteria;Alphaproteobacteria;Methylobacterium"
3181	maqu	Methylobacterium aquaticum	"Prokaryotes;Bacteria;Alphaproteobacteria;Methylobacterium"
3182	mphy	Methylobacterium phyllosphaerae	"Prokaryotes;Bacteria;Alphaproteobacteria;Methylobacterium"
3183	mee	Methylobacterium currus	"Prokaryotes;Bacteria;Alphaproteobacteria;Methylobacterium"
3184	metd	Methylobacterium sp. DM1	"Prokaryotes;Bacteria;Alphaproteobacteria;Methylobacterium"

3185	metx	Methylobacterium sp. XJLW	"Prokaryotes;Bacteria;Alphaproteobacteria;Methylobacterium"
3186	mets	Methylobacterium durans	"Prokaryotes;Bacteria;Alphaproteobacteria;Methylobacterium"
3187	meti	Methylobacterium sp. 17Sr1-43	"Prokaryotes;Bacteria;Alphaproteobacteria;Methylobacterium"
3188	mmes	Methylobacterium mesophilicum	"Prokaryotes;Bacteria;Alphaproteobacteria;Methylobacterium"
3189	mtea	Methylobacterium terrae	"Prokaryotes;Bacteria;Alphaproteobacteria;Methylobacterium"
3190	moc	Microvirga ossetica	"Prokaryotes;Bacteria;Alphaproteobacteria;Microvirga"
3191	miv	Microvirga sp. 17 mud 1-3	"Prokaryotes;Bacteria;Alphaproteobacteria;Microvirga"
3192	mico	Microvirga thermotolerans	"Prokaryotes;Bacteria;Alphaproteobacteria;Microvirga"
3193	bid	Beijerinckia indica	"Prokaryotes;Bacteria;Alphaproteobacteria;Beijerinckia"
3194	msl	Methylocella silvestris	"Prokaryotes;Bacteria;Alphaproteobacteria;Methylocella"
3195	mtun	Methylocella tundrae	"Prokaryotes;Bacteria;Alphaproteobacteria;Methylocella"
3196	mlg	Methylovirgula ligni	"Prokaryotes;Bacteria;Alphaproteobacteria;Methylovirgula"
3197	rhj	Rhodoblastus sp. SSC7	"Prokaryotes;Bacteria;Alphaproteobacteria;Rhodoblastus"
3198	bbar	Beijerinckiaceae bacterium RH AL1	"Prokaryotes;Bacteria;Alphaproteobacteria;unclassified Beijerinckiaceae"
3199	chel	Chelatococcus sp. CO-6	"Prokaryotes;Bacteria;Alphaproteobacteria;Chelatococcus"
3200	cdq	Chelatococcus daeguensis	"Prokaryotes;Bacteria;Alphaproteobacteria;Chelatococcus"
3201	hdn	Hyphomicrobium denitrificans ATCC 51888	"Prokaryotes;Bacteria;Alphaproteobacteria;Hyphomicrobium"
3202	hdt	Hyphomicrobium denitrificans 1NES1	"Prokaryotes;Bacteria;Alphaproteobacteria;Hyphomicrobium"
3203	hmc	Hyphomicrobium sp. MC1	"Prokaryotes;Bacteria;Alphaproteobacteria;Hyphomicrobium"
3204	hni	Hyphomicrobium nitrativorans	"Prokaryotes;Bacteria;Alphaproteobacteria;Hyphomicrobium"
3205	rva	Rhodomicrobium vannielii	"Prokaryotes;Bacteria;Alphaproteobacteria;Rhodomicrobium"
3206	phl	Pelagibacterium halotolerans	"Prokaryotes;Bacteria;Alphaproteobacteria;Pelagibacterium"
3207	fil	Candidatus Filomicrobium marinum W	"Prokaryotes;Bacteria;Alphaproteobacteria;Filomicrobium"
3208	fiy	Candidatus Filomicrobium marinum Y	"Prokaryotes;Bacteria;Alphaproteobacteria;Filomicrobium"
3209	deq	Devosia sp. H5989	"Prokaryotes;Bacteria;Alphaproteobacteria;Devosia"
3210	dei	Devosia sp. I507	"Prokaryotes;Bacteria;Alphaproteobacteria;Devosia"
3211	dea	Devosia ginsengisoli	"Prokaryotes;Bacteria;Alphaproteobacteria;Devosia"
3212	bvr	Blastochloris viridis	"Prokaryotes;Bacteria;Alphaproteobacteria;Blastochloris"
3213	blag	Blastochloris tepida	"Prokaryotes;Bacteria;Alphaproteobacteria;Blastochloris"
3214	rhz	Rhodoplanes sp. Z2-YC6860	"Prokaryotes;Bacteria;Alphaproteobacteria;Rhodoplanes"
3215	mmyr	Maritalea myrionectae	"Prokaryotes;Bacteria;Alphaproteobacteria;Maritalea"
3216	yti	Youhaiella tibetensis	"Prokaryotes;Bacteria;Alphaproteobacteria;Youhaiella"
3217	mcg	Methyloceanibacter caenitepidi	"Prokaryotes;Bacteria;Alphaproteobacteria;Methyloceanibacter"
3218	metg	Methyloligella sp. GL2	"Prokaryotes;Bacteria;Alphaproteobacteria;Methyloligella"
3219	ntd	Novosphingobium tardaugens	"Prokaryotes;Bacteria;Alphaproteobacteria;Caenibius"
3220	msc	Methylocystis sp. SC2	"Prokaryotes;Bacteria;Alphaproteobacteria;Methylocystis"
3221	mbry	Methylocystis bryophila	"Prokaryotes;Bacteria;Alphaproteobacteria;Methylocystis"
3222	mros	Methylocystis rosea	"Prokaryotes;Bacteria;Alphaproteobacteria;Methylocystis"
3223	mhey	Methylocystis heyeri	"Prokaryotes;Bacteria;Alphaproteobacteria;Methylocystis"
3224	mpar	Methylocystis parvus	"Prokaryotes;Bacteria;Alphaproteobacteria;Methylocystis"
3225	mtw	Methylosinus trichosporium	"Prokaryotes;Bacteria;Alphaproteobacteria;Methylosinus"

3226	pleo	Pleomorphomonas sp. SM30	"Prokaryotes;Bacteria;Alphaproteobacteria;Pleomorphomonas"
3227	mey	Martelella endophytica	"Prokaryotes;Bacteria;Alphaproteobacteria;Martelella"
3228	maad	Martelella sp. AD-3	"Prokaryotes;Bacteria;Alphaproteobacteria;Martelella"
3229	mmed	Martelella mediterranea	"Prokaryotes;Bacteria;Alphaproteobacteria;Martelella"
3230	aua	Aureimonas sp. AU20	"Prokaryotes;Bacteria;Alphaproteobacteria;Aureimonas"
3231	aala	Aureimonas sp. OT7	"Prokaryotes;Bacteria;Alphaproteobacteria;Aureimonas"
3232	brn	Breoghania sp. L-A4	"Prokaryotes;Bacteria;Alphaproteobacteria;Breoghania"
3233	hci	Candidatus Hodgkinia cicadicola Dsem	"Prokaryotes;Bacteria;Alphaproteobacteria;Hodgkinia"
3234	hct	Candidatus Hodgkinia cicadicola TETULN	"Prokaryotes;Bacteria;Alphaproteobacteria;Hodgkinia"
3235	hcc	Candidatus Hodgkinia cicadicola TETUND1	"Prokaryotes;Bacteria;Alphaproteobacteria;Hodgkinia"
3236	hcd	Candidatus Hodgkinia cicadicola TETUND2	"Prokaryotes;Bacteria;Alphaproteobacteria;Hodgkinia"
3237	thd	Candidatus Tokpelaia hoelldoblerii	"Prokaryotes;Bacteria;Alphaproteobacteria;Tokpelaia"
3238	psin	Pseudorhodoplanes sinuspersici	"Prokaryotes;Bacteria;Alphaproteobacteria;Pseudorhodoplanes"
3239	hdi	Hartmannibacter diazotrophicus	"Prokaryotes;Bacteria;Alphaproteobacteria;Hartmannibacter"
3240	noh	Nordella sp. HKS 07	"Prokaryotes;Bacteria;Alphaproteobacteria;Nordella"
3241	rbm	Rhizobiales bacterium NRL2	"Prokaryotes;Bacteria;Alphaproteobacteria;unclassified Rhizobiales"
3242	psf	Pseudovibrio sp. FO-BEG1	"Prokaryotes;Bacteria;Alphaproteobacteria;Pseudovibrio"
3243	pphr	Pannonibacter phragmitetus	"Prokaryotes;Bacteria;Alphaproteobacteria;Pannonibacter"
3244	lap	Labrenzia sp. CP4	"Prokaryotes;Bacteria;Alphaproteobacteria;Labrenzia"
3245	lagg	Labrenzia aggregata	"Prokaryotes;Bacteria;Alphaproteobacteria;Labrenzia"
3246	labr	Labrenzia sp. VG12	"Prokaryotes;Bacteria;Alphaproteobacteria;Labrenzia"
3247	labp	Labrenzia sp. PHM005	"Prokaryotes;Bacteria;Alphaproteobacteria;Labrenzia"
3248	labt	Labrenzia sp. THAF35	"Prokaryotes;Bacteria;Alphaproteobacteria;Labrenzia"
3249	siw	Stappia indica	"Prokaryotes;Bacteria;Alphaproteobacteria;Stappia"
3250	ccr	Caulobacter vibrioides CB15	"Prokaryotes;Bacteria;Alphaproteobacteria;Caulobacter"
3251	ccs	Caulobacter vibrioides NA1000	"Prokaryotes;Bacteria;Alphaproteobacteria;Caulobacter"
3252	cak	Caulobacter sp. K31	"Prokaryotes;Bacteria;Alphaproteobacteria;Caulobacter"
3253	cse	Caulobacter segnis	"Prokaryotes;Bacteria;Alphaproteobacteria;Caulobacter"
3254	chq	Caulobacter henricii	"Prokaryotes;Bacteria;Alphaproteobacteria;Caulobacter"
3255	cmb	Caulobacter mirabilis	"Prokaryotes;Bacteria;Alphaproteobacteria;Caulobacter"
3256	cfh	Caulobacter flavus	"Prokaryotes;Bacteria;Alphaproteobacteria;Caulobacter"
3257	cauf	Caulobacter sp. FWC26	"Prokaryotes;Bacteria;Alphaproteobacteria;Caulobacter"
3258	pzu	Phenylobacterium zucineum	"Prokaryotes;Bacteria;Alphaproteobacteria;Phenylobacterium"
3259	phb	Phenylobacterium parvum	"Prokaryotes;Bacteria;Alphaproteobacteria;Phenylobacterium"
3260	bsb	Brevundimonas subvibrioides	"Prokaryotes;Bacteria;Alphaproteobacteria;Brevundimonas"
3261	brd	Brevundimonas sp. DS20	"Prokaryotes;Bacteria;Alphaproteobacteria;Brevundimonas"
3262	bne	Brevundimonas naejangsanensis	"Prokaryotes;Bacteria;Alphaproteobacteria;Brevundimonas"
3263	brg	Brevundimonas sp. GW460-12-10-14-LB2	"Prokaryotes;Bacteria;Alphaproteobacteria;Brevundimonas"
3264	brl	Brevundimonas sp. LM2	"Prokaryotes;Bacteria;Alphaproteobacteria;Brevundimonas"
3265	bvc	Brevundimonas vesicularis	"Prokaryotes;Bacteria;Alphaproteobacteria;Brevundimonas"
3266	bdm	Brevundimonas diminuta	"Prokaryotes;Bacteria;Alphaproteobacteria;Brevundimonas"

3267	brf	Brevundimonas sp. MF30-B	"Prokaryotes;Bacteria;Alphaproteobacteria;Brevundimonas"
3268	brev	Brevundimonas sp. SGAir0440	"Prokaryotes;Bacteria;Alphaproteobacteria;Brevundimonas"
3269	bmed	Brevundimonas mediterranea	"Prokaryotes;Bacteria;Alphaproteobacteria;Brevundimonas"
3270	bvy	Brevundimonas vancanneytii	"Prokaryotes;Bacteria;Alphaproteobacteria;Brevundimonas"
3271	aex	Asticcacaulis excentricus	"Prokaryotes;Bacteria;Alphaproteobacteria;Asticcacaulis"
3272	tsv	Terricaulis silvestris	"Prokaryotes;Bacteria;Alphaproteobacteria;Terricaulis"
3273	cbot	Caulobacteraceae bacterium	"Prokaryotes;Bacteria;Alphaproteobacteria;unclassified Caulobacteraceae"
3274	sil	Ruegeria pomeroyi	"Prokaryotes;Bacteria;Alphaproteobacteria;Ruegeria"
3275	sit	Ruegeria sp. TM1040	"Prokaryotes;Bacteria;Alphaproteobacteria;Ruegeria"
3276	rua	Ruegeria sp. AD91A	"Prokaryotes;Bacteria;Alphaproteobacteria;Ruegeria"
3277	rut	Ruegeria sp. THAF33	"Prokaryotes;Bacteria;Alphaproteobacteria;Ruegeria"
3278	rmb	Epibacterium mobile	"Prokaryotes;Bacteria;Alphaproteobacteria;Epibacterium"
3279	rsp	Rhodobacter sphaeroides 2.4.1	"Prokaryotes;Bacteria;Alphaproteobacteria;Luteovulum"
3280	rsh	Rhodobacter sphaeroides ATCC 17029	"Prokaryotes;Bacteria;Alphaproteobacteria;Luteovulum"
3281	rsq	Rhodobacter sphaeroides ATCC 17025	"Prokaryotes;Bacteria;Alphaproteobacteria;Luteovulum"
3282	rsk	Rhodobacter sphaeroides KD131	"Prokaryotes;Bacteria;Alphaproteobacteria;Luteovulum"
3283	rcp	Rhodobacter capsulatus	"Prokaryotes;Bacteria;Alphaproteobacteria;Rhodobacter"
3284	rhp	Rhodobacter sp. LPB0142	"Prokaryotes;Bacteria;Alphaproteobacteria;Rhodobacter"
3285	rbl	Rhodobacter blasticus	"Prokaryotes;Bacteria;Alphaproteobacteria;Rhodobacter"
3286	jan	Jannaschia sp. CCS1	"Prokaryotes;Bacteria;Alphaproteobacteria;Jannaschia"
3287	rde	Roseobacter denitrificans	"Prokaryotes;Bacteria;Alphaproteobacteria;Roseobacter"
3288	rli	Roseobacter litoralis	"Prokaryotes;Bacteria;Alphaproteobacteria;Roseobacter"
3289	rpon	Roseobacter ponti	"Prokaryotes;Bacteria;Alphaproteobacteria;Roseobacter"
3290	pde	Paracoccus denitrificans	"Prokaryotes;Bacteria;Alphaproteobacteria;Paracoccus"
3291	pami	Paracoccus aminophilus	"Prokaryotes;Bacteria;Alphaproteobacteria;Paracoccus"
3292	pye	Paracoccus yeei	"Prokaryotes;Bacteria;Alphaproteobacteria;Paracoccus"
3293	pcon	Paracoccus contaminans	"Prokaryotes;Bacteria;Alphaproteobacteria;Paracoccus"
3294	pzh	Paracoccus zhejiangensis	"Prokaryotes;Bacteria;Alphaproteobacteria;Paracoccus"
3295	paro	Paracoccus sp. BM15	"Prokaryotes;Bacteria;Alphaproteobacteria;Paracoccus"
3296	paru	Paracoccus jeotgali	"Prokaryotes;Bacteria;Alphaproteobacteria;Paracoccus"
3297	pamn	Paracoccus aminovorans	"Prokaryotes;Bacteria;Alphaproteobacteria;Paracoccus"
3298	pmut	Paracoccus mutanolyticus	"Prokaryotes;Bacteria;Alphaproteobacteria;Paracoccus"
3299	pars	Paracoccus suum	"Prokaryotes;Bacteria;Alphaproteobacteria;Paracoccus"
3300	parr	Paracoccus sp. Arc7-R13	"Prokaryotes;Bacteria;Alphaproteobacteria;Paracoccus"
3301	pkd	Paracoccus kondratievae	"Prokaryotes;Bacteria;Alphaproteobacteria;Paracoccus"
3302	ppan	Paracoccus pantotrophus	"Prokaryotes;Bacteria;Alphaproteobacteria;Paracoccus"
3303	dsh	Dinoroseobacter shibae	"Prokaryotes;Bacteria;Alphaproteobacteria;Dinoroseobacter"
3304	kvl	Ketogulonicigenium vulgare WSH-001	"Prokaryotes;Bacteria;Alphaproteobacteria;Ketogulonicigenium"
3305	kvu	Ketogulonicigenium vulgare Y25	"Prokaryotes;Bacteria;Alphaproteobacteria;Ketogulonicigenium"
3306	kro	Ketogulonicigenium robustum	"Prokaryotes;Bacteria;Alphaproteobacteria;Ketogulonicigenium"
3307	pga	Phaeobacter inhibens DSM 17395	"Prokaryotes;Bacteria;Alphaproteobacteria;Phaeobacter"

3308	pgl	Phaeobacter inhibens 2.10	"Prokaryotes;Bacteria;Alphaproteobacteria;Phaeobacter"
3309	pgd	Phaeobacter gallaeciensis DSM 26640	"Prokaryotes;Bacteria;Alphaproteobacteria;Phaeobacter"
3310	php	Phaeobacter porticola	"Prokaryotes;Bacteria;Alphaproteobacteria;Phaeobacter"
3311	ppic	Phaeobacter piscinae	"Prokaryotes;Bacteria;Alphaproteobacteria;Phaeobacter"
3312	phq	Phaeobacter sp. LSS9	"Prokaryotes;Bacteria;Alphaproteobacteria;Phaeobacter"
3313	oat	Octadecabacter antarcticus	"Prokaryotes;Bacteria;Alphaproteobacteria;Octadecabacter"
3314	oar	Octadecabacter arcticus	"Prokaryotes;Bacteria;Alphaproteobacteria;Octadecabacter"
3315	otm	Octadecabacter temperatus	"Prokaryotes;Bacteria;Alphaproteobacteria;Octadecabacter"
3316	oct	Octadecabacter sp. SW4	"Prokaryotes;Bacteria;Alphaproteobacteria;Octadecabacter"
3317	lmd	Leisingera methylohalidivorans	"Prokaryotes;Bacteria;Alphaproteobacteria;Leisingera"
3318	lej	Leisingera sp. NJS204	"Prokaryotes;Bacteria;Alphaproteobacteria;Leisingera"
3319	laqu	Leisingera aquaemixtae	"Prokaryotes;Bacteria;Alphaproteobacteria;Leisingera"
3320	red	Roseibacterium elongatum	"Prokaryotes;Bacteria;Alphaproteobacteria;Roseibacterium"
3321	ptp	Planktomarina temperata	"Prokaryotes;Bacteria;Alphaproteobacteria;Planktomarina"
3322	cid	Celeribacter indicus	"Prokaryotes;Bacteria;Alphaproteobacteria;Celeribacter"
3323	cmar	Celeribacter marinus	"Prokaryotes;Bacteria;Alphaproteobacteria;Celeribacter"
3324	ceh	Celeribacter ethanolicus	"Prokaryotes;Bacteria;Alphaproteobacteria;Celeribacter"
3325	cmag	Celeribacter manganoxidans	"Prokaryotes;Bacteria;Alphaproteobacteria;Celeribacter"
3326	malg	Marinovum algicola	"Prokaryotes;Bacteria;Alphaproteobacteria;Marinovum"
3327	con	Confluentimicrobium sp. EMB200-NS6	"Prokaryotes;Bacteria;Alphaproteobacteria;Actibacterium"
3328	rsu	Rhodovulum sulfidophilum	"Prokaryotes;Bacteria;Alphaproteobacteria;Rhodovulum"
3329	rhm	Rhodovulum sp. MB263	"Prokaryotes;Bacteria;Alphaproteobacteria;Rhodovulum"
3330	rhc	Rhodovulum sp. P5	"Prokaryotes;Bacteria;Alphaproteobacteria;Rhodovulum"
3331	hat	Halocynthiibacter arcticus	"Prokaryotes;Bacteria;Alphaproteobacteria;Halocynthiibacter"
3332	daa	Defluviimonas alba	"Prokaryotes;Bacteria;Alphaproteobacteria;Defluviimonas"
3333	ypac	Salipiger pacificus	"Prokaryotes;Bacteria;Alphaproteobacteria;Yangia"
3334	yan	Yangia sp. CCB-MM3	"Prokaryotes;Bacteria;Alphaproteobacteria;Salipiger"
3335	tpro	Salipiger profundus	"Prokaryotes;Bacteria;Alphaproteobacteria;Salipiger"
3336	suam	Sulfitobacter sp. AM1-D1	"Prokaryotes;Bacteria;Alphaproteobacteria;Sulfitobacter"
3337	spse	Sulfitobacter pseudonitzschiae	"Prokaryotes;Bacteria;Alphaproteobacteria;Sulfitobacter"
3338	sulz	Sulfitobacter sp. SK012	"Prokaryotes;Bacteria;Alphaproteobacteria;Sulfitobacter"
3339	suli	Sulfitobacter sp. JL08	"Prokaryotes;Bacteria;Alphaproteobacteria;Sulfitobacter"
3340	suld	Sulfitobacter sp. D7	"Prokaryotes;Bacteria;Alphaproteobacteria;Sulfitobacter"
3341	sdo	Sulfitobacter donghicola	"Prokaryotes;Bacteria;Alphaproteobacteria;Sulfitobacter"
3342	spot	Sulfitobacter pontiacus	"Prokaryotes;Bacteria;Alphaproteobacteria;Sulfitobacter"
3343	don	Marivivens sp. JLT3646	"Prokaryotes;Bacteria;Alphaproteobacteria;Donghicola"
3344	tom	Tateyamaria omphalii	"Prokaryotes;Bacteria;Alphaproteobacteria;Tateyamaria"
3345	paby	Pelagibaca abyssi	"Prokaryotes;Bacteria;Alphaproteobacteria;Pelagibaca"
3346	thw	Thioclava nitratireducens	"Prokaryotes;Bacteria;Alphaproteobacteria;Thioclava"
3347	tec	Thioclava electrotropha	"Prokaryotes;Bacteria;Alphaproteobacteria;Thioclava"
3348	rmm	Roseovarius mucosus	"Prokaryotes;Bacteria;Alphaproteobacteria;Roseovarius"

3349	rok	Roseovarius sp. AK1035	"Prokaryotes;Bacteria;Alphaproteobacteria;Roseovarius"
3350	rid	Roseovarius indicus	"Prokaryotes;Bacteria;Alphaproteobacteria;Roseovarius"
3351	rom	Roseovarius sp. MME-070	"Prokaryotes;Bacteria;Alphaproteobacteria;Roseovarius"
3352	roh	Roseovarius sp. THAF27	"Prokaryotes;Bacteria;Alphaproteobacteria;Roseovarius"
3353	lvs	Yoonia vestfoldensis	"Prokaryotes;Bacteria;Alphaproteobacteria;Yoonia"
3354	aht	Antarctobacter heliothermus	"Prokaryotes;Bacteria;Alphaproteobacteria;Antarctobacter"
3355	rbg	Rhodobaca barguzinensis	"Prokaryotes;Bacteria;Alphaproteobacteria;Rhodobaca"
3356	sagu	Sagittula sp. P11	"Prokaryotes;Bacteria;Alphaproteobacteria;Sagittula"
3357	thaa	Thalassococcus sp. S3	"Prokaryotes;Bacteria;Alphaproteobacteria;Thalassococcus"
3358	geh	Gemmobacter sp. HYN0069	"Prokaryotes;Bacteria;Alphaproteobacteria;Gemmobacter"
3359	taw	Tabrizicola piscis	"Prokaryotes;Bacteria;Alphaproteobacteria;Tabrizicola"
3360	salo	Silicimonas algicola	"Prokaryotes;Bacteria;Alphaproteobacteria;Silicimonas"
3361	sedi	Parasedimentitalea marina	"Prokaryotes;Bacteria;Alphaproteobacteria;Sedimentitalea"
3362	hml	Haematobacter massiliensis	"Prokaryotes;Bacteria;Alphaproteobacteria;Haematobacter"
3363	boo	Boseongicola sp. CCM32	"Prokaryotes;Bacteria;Alphaproteobacteria;Boseongicola"
3364	pseb	Pseudorhodobacter turbinis	"Prokaryotes;Bacteria;Alphaproteobacteria;Pseudorhodobacter"
3365	lit	Qingshengfaniella alkalisoli	"Prokaryotes;Bacteria;Alphaproteobacteria;Qingshengfaniella"
3366	ocd	Oceanicola sp. D3	"Prokaryotes;Bacteria;Alphaproteobacteria;Oceanicola"
3367	maru	Maribius sp. THAF1	"Prokaryotes;Bacteria;Alphaproteobacteria;Maribius"
3368	rot	Roseivivax sp. THAF197b	"Prokaryotes;Bacteria;Alphaproteobacteria;Roseivivax"
3369	ppru	Paraoceanicella profunda	"Prokaryotes;Bacteria;Alphaproteobacteria;Paraoceanicella"
3370	paed	Pseudohalocynthiibacter aestuariivivens	"Prokaryotes;Bacteria;Alphaproteobacteria;Pseudohalocynthiibacter"
3371	mon	Monaibacterium sp. ALG8	"Prokaryotes;Bacteria;Alphaproteobacteria;Monaibacterium"
3372	malu	Mameliella alba	"Prokaryotes;Bacteria;Alphaproteobacteria;Mameliella"
3373	tgl	Thalassobius gelatinovorus	"Prokaryotes;Bacteria;Alphaproteobacteria;Thalassobius"
3374	pamo	Profundibacter amoris	"Prokaryotes;Bacteria;Alphaproteobacteria;Profundibacter"
3375	pshq	Pseudopuniceibacterium antarcticum	"Prokaryotes;Bacteria;Alphaproteobacteria;Pseudopuniceibacterium"
3376	poz	Pontivivens sp. MT2928	"Prokaryotes;Bacteria;Alphaproteobacteria;Pontivivens"
3377	palw	Pseudooceanicola algae	"Prokaryotes;Bacteria;Alphaproteobacteria;Pseudooceanicola"
3378	ppaf	Pelagovum pacificum	"Prokaryotes;Bacteria;Alphaproteobacteria;Pelagovum"
3379	pgv	Polymorphum gilvum	"Prokaryotes;Bacteria;Alphaproteobacteria;Polymorphum"
3380	rbz	Rhodobacteraceae bacterium G7	"Prokaryotes;Bacteria;Alphaproteobacteria;unclassified Rhodobacteraceae"
3381	thas	Rhodobacteraceae bacterium SH-1	"Prokaryotes;Bacteria;Alphaproteobacteria;unclassified Rhodobacteraceae"
3382	faq	Rhodobacteraceae bacterium SC52	"Prokaryotes;Bacteria;Alphaproteobacteria;unclassified Rhodobacteraceae"
3383	hdh	Rhodobacteraceae bacterium RR4-56	"Prokaryotes;Bacteria;Alphaproteobacteria;unclassified Rhodobacteraceae"
3384	mmr	Maricaulis maris	"Prokaryotes;Bacteria;Alphaproteobacteria;Maricaulis"
3385	gak	Glycocaulis alkaliphilus	"Prokaryotes;Bacteria;Alphaproteobacteria;Glycocaulis"
3386	hyt	Hyphobacterium sp. CCMP332	"Prokaryotes;Bacteria;Alphaproteobacteria;Hyphobacterium"
3387	hne	Hyphomonas neptunium	"Prokaryotes;Bacteria;Alphaproteobacteria;Hyphomonas"
3388	hba	Hirschia baltica	"Prokaryotes;Bacteria;Alphaproteobacteria;Hirschia"
3389	hbc	Hyphomonadaceae bacterium UKL13-1	"Prokaryotes;Bacteria;Alphaproteobacteria;unclassified Hyphomonadaceae"

3390	zmo	Zymomonas mobilis subsp. mobilis ZM4	"Prokaryotes;Bacteria;Alphaproteobacteria;Zymomonas"
3391	zmn	Zymomonas mobilis subsp. mobilis NCIMB 11163	"Prokaryotes;Bacteria;Alphaproteobacteria;Zymomonas"
3392	zmm	Zymomonas mobilis subsp. mobilis ATCC 10988	"Prokaryotes;Bacteria;Alphaproteobacteria;Zymomonas"
3393	zmb	Zymomonas mobilis subsp. mobilis ATCC 29191	"Prokaryotes;Bacteria;Alphaproteobacteria;Zymomonas"
3394	zmi	Zymomonas mobilis subsp. mobilis CP4 = NRRL B-14023	"Prokaryotes;Bacteria;Alphaproteobacteria;Zymomonas"
3395	zmc	Zymomonas mobilis subsp. mobilis CP4 = NRRL B-14023	"Prokaryotes;Bacteria;Alphaproteobacteria;Zymomonas"
3396	zmr	Zymomonas mobilis subsp. mobilis NRRL B-12526	"Prokaryotes;Bacteria;Alphaproteobacteria;Zymomonas"
3397	zmp	Zymomonas mobilis subsp. pomaceae ATCC 29192	"Prokaryotes;Bacteria;Alphaproteobacteria;Zymomonas"
3398	nar	Novosphingobium aromaticivorans	"Prokaryotes;Bacteria;Alphaproteobacteria;Novosphingobium"
3399	npp	Novosphingobium sp. PP1Y	"Prokaryotes;Bacteria;Alphaproteobacteria;Novosphingobium"
3400	npn	Novosphingobium pentaromativorans	"Prokaryotes;Bacteria;Alphaproteobacteria;Novosphingobium"
3401	nre	Novosphingobium resinovorum	"Prokaryotes;Bacteria;Alphaproteobacteria;Novosphingobium"
3402	nov	Novosphingobium sp. P6W	"Prokaryotes;Bacteria;Alphaproteobacteria;Novosphingobium"
3403	not	Novosphingobium sp. THN1	"Prokaryotes;Bacteria;Alphaproteobacteria;Novosphingobium"
3404	nor	Novosphingobium sp. ABRDHK2	"Prokaryotes;Bacteria;Alphaproteobacteria;Novosphingobium"
3405	ngf	Novosphingobium ginsenosidimutans	"Prokaryotes;Bacteria;Alphaproteobacteria;Novosphingobium"
3406	nog	Novosphingobium sp. Gsoil 351	"Prokaryotes;Bacteria;Alphaproteobacteria;Novosphingobium"
3407	sal	Sphingopyxis alaskensis	"Prokaryotes;Bacteria;Alphaproteobacteria;Sphingopyxis"
3408	sphk	Sphingopyxis fribergensis	"Prokaryotes;Bacteria;Alphaproteobacteria;Sphingopyxis"
3409	sphp	Sphingopyxis sp. 113P3	"Prokaryotes;Bacteria;Alphaproteobacteria;Sphingopyxis"
3410	smag	Sphingopyxis macrogoltabida EY-1	"Prokaryotes;Bacteria;Alphaproteobacteria;Sphingopyxis"
3411	smaz	Sphingopyxis macrogoltabida 203	"Prokaryotes;Bacteria;Alphaproteobacteria;Sphingopyxis"
3412	ster	Sphingopyxis terrae	"Prokaryotes;Bacteria;Alphaproteobacteria;Sphingopyxis"
3413	sgi	Sphingopyxis granuli	"Prokaryotes;Bacteria;Alphaproteobacteria;Sphingopyxis"
3414	sphl	Sphingopyxis sp. LPB0140	"Prokaryotes;Bacteria;Alphaproteobacteria;Sphingopyxis"
3415	sphq	Sphingopyxis sp. QXT-31	"Prokaryotes;Bacteria;Alphaproteobacteria;Sphingopyxis"
3416	spho	Sphingopyxis sp. MG	"Prokaryotes;Bacteria;Alphaproteobacteria;Sphingopyxis"
3417	sphx	Sphingopyxis sp. PAMC25046	"Prokaryotes;Bacteria;Alphaproteobacteria;Sphingopyxis"
3418	sphu	Uncultured Sphingopyxis sp. UC10	"Prokaryotes;Bacteria;Alphaproteobacteria;Sphingopyxis"
3419	swi	Sphingomonas wittichii	"Prokaryotes;Bacteria;Alphaproteobacteria;Sphingomonas"
3420	sphd	Sphingomonas wittichii DC-6	"Prokaryotes;Bacteria;Alphaproteobacteria;Sphingomonas"
3421	sphm	Sphingomonas sp. MM-1	"Prokaryotes;Bacteria;Alphaproteobacteria;Sphingomonas"
3422	stax	Sphingomonas taxi	"Prokaryotes;Bacteria;Alphaproteobacteria;Sphingomonas"
3423	sphi	Sphingomonas hengshuiensis	"Prokaryotes;Bacteria;Alphaproteobacteria;Sphingomonas"
3424	ssan	Sphingomonas sanxanigenens	"Prokaryotes;Bacteria;Alphaproteobacteria;Sphingomonas"
3425	snj	Sphingomonas sp. NIC1	"Prokaryotes;Bacteria;Alphaproteobacteria;Sphingomonas"
3426	smy	Sphingomonas melonis	"Prokaryotes;Bacteria;Alphaproteobacteria;Sphingomonas"
3427	span	Sphingomonas panacis	"Prokaryotes;Bacteria;Alphaproteobacteria;Sphingomonas"
3428	skr	Sphingomonas koreensis	"Prokaryotes;Bacteria;Alphaproteobacteria;Sphingomonas"
3429	splm	Sphingomonas sp. LM7	"Prokaryotes;Bacteria;Alphaproteobacteria;Sphingomonas"
3430	splk	Sphingomonas sp. LK11	"Prokaryotes;Bacteria;Alphaproteobacteria;Sphingomonas"

3431	spkc	Sphingomonas sp. KC8	"Prokaryotes;Bacteria;Alphaproteobacteria;Sphingomonas"
3432	sphc	Sphingomonas sp. Cra20	"Prokaryotes;Bacteria;Alphaproteobacteria;Sphingomonas"
3433	sphf	Sphingomonas sp. FARSPH	"Prokaryotes;Bacteria;Alphaproteobacteria;Sphingomonas"
3434	spha	Sphingomonas sp. YZ-8	"Prokaryotes;Bacteria;Alphaproteobacteria;Sphingomonas"
3435	spau	Sphingomonas paucimobilis	"Prokaryotes;Bacteria;Alphaproteobacteria;Sphingomonas"
3436	sech	Sphingomonas echinoides	"Prokaryotes;Bacteria;Alphaproteobacteria;Sphingomonas"
3437	slut	Sphingomonas lutea	"Prokaryotes;Bacteria;Alphaproteobacteria;Sphingomonas"
3438	srhi	Sphingomonas rhizophila	"Prokaryotes;Bacteria;Alphaproteobacteria;Sphingomonas"
3439	sjp	Sphingobium japonicum	"Prokaryotes;Bacteria;Alphaproteobacteria;Sphingobium"
3440	sch	Sphingobium chlorophenolicum	"Prokaryotes;Bacteria;Alphaproteobacteria;Sphingobium"
3441	ssy	Sphingobium sp. SYK-6	"Prokaryotes;Bacteria;Alphaproteobacteria;Sphingobium"
3442	syb	Sphingobium sp. YBL2	"Prokaryotes;Bacteria;Alphaproteobacteria;Sphingobium"
3443	sbd	Sphingobium baderi	"Prokaryotes;Bacteria;Alphaproteobacteria;Sphingobium"
3444	spmi	Sphingobium sp. MI1205	"Prokaryotes;Bacteria;Alphaproteobacteria;Sphingobium"
3445	sphb	Sphingobium sp. EP60837	"Prokaryotes;Bacteria;Alphaproteobacteria;Sphingobium"
3446	sphr	Sphingobium sp. RAC03	"Prokaryotes;Bacteria;Alphaproteobacteria;Sphingobium"
3447	sinb	Sphingobium indicum	"Prokaryotes;Bacteria;Alphaproteobacteria;Sphingobium"
3448	spht	Sphingobium sp. TKS	"Prokaryotes;Bacteria;Alphaproteobacteria;Sphingobium"
3449	shyd	Sphingobium hydrophobicum	"Prokaryotes;Bacteria;Alphaproteobacteria;Sphingobium"
3450	sya	Sphingobium yanoikuyae	"Prokaryotes;Bacteria;Alphaproteobacteria;Sphingobium"
3451	sclo	Sphingobium cloacae	"Prokaryotes;Bacteria;Alphaproteobacteria;Sphingobium"
3452	spyg	Sphingobium sp. YG1	"Prokaryotes;Bacteria;Alphaproteobacteria;Sphingobium"
3453	sufl	Sphingobium fuliginis	"Prokaryotes;Bacteria;Alphaproteobacteria;Sphingobium"
3454	sami	Sphingobium amiense	"Prokaryotes;Bacteria;Alphaproteobacteria;Sphingobium"
3455	sbar	Sphingobium barthaii	"Prokaryotes;Bacteria;Alphaproteobacteria;Sphingobium"
3456	cij	Citromicrobium sp. JL477	"Prokaryotes;Bacteria;Alphaproteobacteria;Citromicrobium"
3457	sphg	Sphingorhabdus sp. M41	"Prokaryotes;Bacteria;Alphaproteobacteria;Sphingorhabdus"
3458	sfla	Sphingorhabdus sp. SMR4y	"Prokaryotes;Bacteria;Alphaproteobacteria;Sphingorhabdus"
3459	sphy	Sphingorhabdus sp. YGSMI21	"Prokaryotes;Bacteria;Alphaproteobacteria;Sphingorhabdus"
3460	blas	Blastomonas sp. RAC04	"Prokaryotes;Bacteria;Alphaproteobacteria;Blastomonas"
3461	bfw	Blastomonas fulva	"Prokaryotes;Bacteria;Alphaproteobacteria;Blastomonas"
3462	rdi	Rhizorhabdus dicambivorans	"Prokaryotes;Bacteria;Alphaproteobacteria;Rhizorhabdus"
3463	sphj	Tardibacter chloracetimidivorans	"Prokaryotes;Bacteria;Alphaproteobacteria;Tardibacter"
3464	spzr	Sphingosinithalassobacter sp. zrk23	"Prokaryotes;Bacteria;Alphaproteobacteria;Sphingosinithalassobacter"
3465	hgn	Hankyongella ginsenosidimutans	"Prokaryotes;Bacteria;Alphaproteobacteria;Hankyongella"
3466	palg	Parasphingopyxis algicola	"Prokaryotes;Bacteria;Alphaproteobacteria;Parasphingopyxis"
3467	smic	Sphingosinicella microcystinivorans	"Prokaryotes;Bacteria;Alphaproteobacteria;Sphingosinicella"
3468	sphs	Sphingosinicella sp. BN140058	"Prokaryotes;Bacteria;Alphaproteobacteria;Sphingosinicella"
3469	sand	Sandaracinobacter sp. M6	"Prokaryotes;Bacteria;Alphaproteobacteria;Sandaracinobacter"
3470	aay	Altererythrobacter atlanticus	"Prokaryotes;Bacteria;Alphaproteobacteria;Altererythrobacter"
3471	aep	Altererythrobacter epoxidivorans	"Prokaryotes;Bacteria;Alphaproteobacteria;Altererythrobacter"

3472	alb	Altererythrobacter sp. B11	"Prokaryotes;Bacteria;Alphaproteobacteria;Altererythrobacter"
3473	alh	Altererythrobacter sp. BO-6	"Prokaryotes;Bacteria;Alphaproteobacteria;Altererythrobacter"
3474	amx	Pelagerythrobacter marensis	"Prokaryotes;Bacteria;Alphaproteobacteria;Pelagerythrobacter"
3475	anh	Paraurantiacibacter namhicola	"Prokaryotes;Bacteria;Alphaproteobacteria;Paraurantiacibacter"
3476	ado	Tsuneonella dongtanensis	"Prokaryotes;Bacteria;Alphaproteobacteria;Tsuneonella"
3477	cna	Croceicoccus naphthovorans	"Prokaryotes;Bacteria;Alphaproteobacteria;Croceicoccus"
3478	cman	Croceicoccus marinus	"Prokaryotes;Bacteria;Alphaproteobacteria;Croceicoccus"
3479	ery	Aurantiacibacter atlanticus	"Prokaryotes;Bacteria;Alphaproteobacteria;Aurantiacibacter"
3480	egn	Aurantiacibacter gangjinensis	"Prokaryotes;Bacteria;Alphaproteobacteria;Aurantiacibacter"
3481	efv	Qipengyuania flava	"Prokaryotes;Bacteria;Alphaproteobacteria;Qipengyuania"
3482	eli	Erythrobacter litoralis HTCC2594	"Prokaryotes;Bacteria;Alphaproteobacteria;Erythrobacter"
3483	elq	Erythrobacter litoralis DSM 8509	"Prokaryotes;Bacteria;Alphaproteobacteria;Erythrobacter"
3484	erk	Erythrobacter sp. KY5	"Prokaryotes;Bacteria;Alphaproteobacteria;Erythrobacter"
3485	err	Erythrobacter aureus	"Prokaryotes;Bacteria;Alphaproteobacteria;Erythrobacter"
3486	erf	Erythrobacter sp. THAF29	"Prokaryotes;Bacteria;Alphaproteobacteria;Erythrobacter"
3487	emv	Erythrobacter mangrovi	"Prokaryotes;Bacteria;Alphaproteobacteria;Erythrobacter"
3488	pns	Erythrobacter neustonensis	"Prokaryotes;Bacteria;Alphaproteobacteria;Erythrobacter"
3489	porl	Porphyrobacter sp. LM 6	"Prokaryotes;Bacteria;Alphaproteobacteria;Porphyrobacter"
3490	phz	Porphyrobacter sp. HT-58-2	"Prokaryotes;Bacteria;Alphaproteobacteria;Porphyrobacter"
3491	pot	Porphyrobacter sp. YT40	"Prokaryotes;Bacteria;Alphaproteobacteria;Porphyrobacter"
3492	gox	Gluconobacter oxydans 621H	"Prokaryotes;Bacteria;Alphaproteobacteria;Gluconobacter"
3493	goh	Gluconobacter oxydans H24	"Prokaryotes;Bacteria;Alphaproteobacteria;Gluconobacter"
3494	goy	Gluconobacter oxydans DSM 3504	"Prokaryotes;Bacteria;Alphaproteobacteria;Gluconobacter"
3495	gal	Gluconobacter albidus	"Prokaryotes;Bacteria;Alphaproteobacteria;Gluconobacter"
3496	gti	Gluconobacter thailandicus	"Prokaryotes;Bacteria;Alphaproteobacteria;Gluconobacter"
3497	gbe	Granulibacter bethesdensis CGDNIH1	"Prokaryotes;Bacteria;Alphaproteobacteria;Granulobacter"
3498	gbh	Granulibacter bethesdensis CGDNIH2	"Prokaryotes;Bacteria;Alphaproteobacteria;Granulobacter"
3499	gbc	Granulibacter bethesdensis NIH3.1	"Prokaryotes;Bacteria;Alphaproteobacteria;Granulobacter"
3500	gbs	Granulibacter bethesdensis CGDNIH4	"Prokaryotes;Bacteria;Alphaproteobacteria;Granulobacter"
3501	acr	Acidiphilium cryptum	"Prokaryotes;Bacteria;Alphaproteobacteria;Acidiphilium"
3502	amv	Acidiphilium multivorum	"Prokaryotes;Bacteria;Alphaproteobacteria;Acidiphilium"
3503	gdi	Gluconacetobacter diazotrophicus PA1 5 (Brazil)	"Prokaryotes;Bacteria;Alphaproteobacteria;Gluconacetobacter"
3504	gdj	Gluconacetobacter diazotrophicus PA1 5 (JGI)	"Prokaryotes;Bacteria;Alphaproteobacteria;Gluconacetobacter"
3505	gxy	Komagataeibacter medellinensis	"Prokaryotes;Bacteria;Alphaproteobacteria;Komagataeibacter"
3506	gxl	Komagataeibacter xylinus	"Prokaryotes;Bacteria;Alphaproteobacteria;Komagataeibacter"
3507	kna	Komagataeibacter nataicola	"Prokaryotes;Bacteria;Alphaproteobacteria;Komagataeibacter"
3508	keu	Komagataeibacter europaeus	"Prokaryotes;Bacteria;Alphaproteobacteria;Komagataeibacter"
3509	ksc	Komagataeibacter saccharivorans	"Prokaryotes;Bacteria;Alphaproteobacteria;Komagataeibacter"
3510	kre	Komagataeibacter rhaeticus	"Prokaryotes;Bacteria;Alphaproteobacteria;Komagataeibacter"
3511	kha	Komagataeibacter hansenii	"Prokaryotes;Bacteria;Alphaproteobacteria;Komagataeibacter"
3512	apt	Acetobacter pasteurianus IFO 3283-01	"Prokaryotes;Bacteria;Alphaproteobacteria;Acetobacter"

3513	apw	Acetobacter pasteurianus IFO 3283-01-42C	"Prokaryotes;Bacteria;Alphaproteobacteria;Acetobacter"
3514	apf	Acetobacter pasteurianus IFO 3283-03	"Prokaryotes;Bacteria;Alphaproteobacteria;Acetobacter"
3515	apu	Acetobacter pasteurianus IFO 3283-07	"Prokaryotes;Bacteria;Alphaproteobacteria;Acetobacter"
3516	apg	Acetobacter pasteurianus IFO 3283-12	"Prokaryotes;Bacteria;Alphaproteobacteria;Acetobacter"
3517	apq	Acetobacter pasteurianus IFO 3283-22	"Prokaryotes;Bacteria;Alphaproteobacteria;Acetobacter"
3518	apx	Acetobacter pasteurianus IFO 3283-26	"Prokaryotes;Bacteria;Alphaproteobacteria;Acetobacter"
3519	apz	Acetobacter pasteurianus IFO 3283-32	"Prokaryotes;Bacteria;Alphaproteobacteria;Acetobacter"
3520	apk	Acetobacter pasteurianus 386B	"Prokaryotes;Bacteria;Alphaproteobacteria;Acetobacter"
3521	asz	Acetobacter senegalensis	"Prokaryotes;Bacteria;Alphaproteobacteria;Acetobacter"
3522	asv	Acetobacter oryzifermentans	"Prokaryotes;Bacteria;Alphaproteobacteria;Acetobacter"
3523	aace	Acetobacter aceti	"Prokaryotes;Bacteria;Alphaproteobacteria;Acetobacter"
3524	aper	Acetobacter persici	"Prokaryotes;Bacteria;Alphaproteobacteria;Acetobacter"
3525	apom	Acetobacter pomorum	"Prokaryotes;Bacteria;Alphaproteobacteria;Acetobacter"
3526	ato	Acetobacter tropicalis	"Prokaryotes;Bacteria;Alphaproteobacteria;Acetobacter"
3527	aasc	Acetobacter ascendens	"Prokaryotes;Bacteria;Alphaproteobacteria;Acetobacter"
3528	acet	Acetobacter sp. JWB	"Prokaryotes;Bacteria;Alphaproteobacteria;Acetobacter"
3529	aot	Acetobacter orientalis	"Prokaryotes;Bacteria;Alphaproteobacteria;Acetobacter"
3530	aoy	Acetobacter oryzoeni	"Prokaryotes;Bacteria;Alphaproteobacteria;Acetobacter"
3531	abg	Asaia bogorensis	"Prokaryotes;Bacteria;Alphaproteobacteria;Asaia"
3532	kba	Kozakia baliensis	"Prokaryotes;Bacteria;Alphaproteobacteria;Kozakia"
3533	rgi	Roseomonas gilardii	"Prokaryotes;Bacteria;Alphaproteobacteria;Roseomonas"
3534	ros	Roseomonas sp. FDAARGOS_362	"Prokaryotes;Bacteria;Alphaproteobacteria;Roseomonas"
3535	rmuc	Roseomonas mucosa	"Prokaryotes;Bacteria;Alphaproteobacteria;Roseomonas"
3536	nch	Neoasaia chiangmaiensis	"Prokaryotes;Bacteria;Alphaproteobacteria;Neoasaia"
3537	coq	Commensalibacter sp. AMU001	"Prokaryotes;Bacteria;Alphaproteobacteria;Commensalibacter"
3538	comm	Commensalibacter sp. ESL0284	"Prokaryotes;Bacteria;Alphaproteobacteria;Commensalibacter"
3539	shum	Stella humosa	"Prokaryotes;Bacteria;Alphaproteobacteria;Stella"
3540	svc	Stella vacuolata	"Prokaryotes;Bacteria;Alphaproteobacteria;Stella"
3541	ntn	Neokomagataea tanensis	"Prokaryotes;Bacteria;Alphaproteobacteria;Neokomagataea"
3542	neh	Oecophyllibacter saccharovorans	"Prokaryotes;Bacteria;Alphaproteobacteria;Neokomagataea"
3543	ssam	Swingsia samuiensis	"Prokaryotes;Bacteria;Alphaproteobacteria;Swingsia"
3544	swf	Swingsia sp. F3b2	"Prokaryotes;Bacteria;Alphaproteobacteria;Swingsia"
3545	bob	Bombella sp. ESL0368	"Prokaryotes;Bacteria;Alphaproteobacteria;Bombella"
3546	bomb	Bombella sp. KACC 21507	"Prokaryotes;Bacteria;Alphaproteobacteria;Bombella"
3547	rru	Rhodospirillum rubrum ATCC 11170	"Prokaryotes;Bacteria;Alphaproteobacteria;Rhodospirillum"
3548	rrf	Rhodospirillum rubrum F11	"Prokaryotes;Bacteria;Alphaproteobacteria;Rhodospirillum"
3549	rce	Rhodospirillum centenum	"Prokaryotes;Bacteria;Alphaproteobacteria;Rhodospirillum"
3550	rpm	Pararhodospirillum photometricum	"Prokaryotes;Bacteria;Alphaproteobacteria;Pararhodospirillum"
3551	mag	Magnetospirillum magneticum	"Prokaryotes;Bacteria;Alphaproteobacteria;Magnetospirillum"
3552	mgy	Magnetospirillum gryphiswaldense MSR-1 v2	"Prokaryotes;Bacteria;Alphaproteobacteria;Magnetospirillum"
3553	mgry	Magnetospirillum gryphiswaldense MSR-1	"Prokaryotes;Bacteria;Alphaproteobacteria;Magnetospirillum"

3554	magx	Magnetospirillum sp. XM-1	"Prokaryotes;Bacteria;Alphaproteobacteria;Magnetospirillum"
3555	magn	Magnetospirillum sp. ME-1	"Prokaryotes;Bacteria;Alphaproteobacteria;Magnetospirillum"
3556	azl	Azospirillum sp. B510	"Prokaryotes;Bacteria;Alphaproteobacteria;Azospirillum"
3557	ali	Azospirillum lipoferum	"Prokaryotes;Bacteria;Alphaproteobacteria;Azospirillum"
3558	abs	Azospirillum baldaniorum Sp245	"Prokaryotes;Bacteria;Alphaproteobacteria;Azospirillum"
3559	abq	Azospirillum brasilense Az39	"Prokaryotes;Bacteria;Alphaproteobacteria;Azospirillum"
3560	abf	Azospirillum brasilense Sp7	"Prokaryotes;Bacteria;Alphaproteobacteria;Azospirillum"
3561	ati	Azospirillum thiophilum	"Prokaryotes;Bacteria;Alphaproteobacteria;Azospirillum"
3562	ahu	Azospirillum humicireducens	"Prokaryotes;Bacteria;Alphaproteobacteria;Azospirillum"
3563	azt	Azospirillum sp. TSH58	"Prokaryotes;Bacteria;Alphaproteobacteria;Azospirillum"
3564	azm	Azospirillum ramasamyi	"Prokaryotes;Bacteria;Alphaproteobacteria;Azospirillum"
3565	azz	Azospirillum thermophilum	"Prokaryotes;Bacteria;Alphaproteobacteria;Azospirillum"
3566	aoz	Azospirillum oryzae	"Prokaryotes;Bacteria;Alphaproteobacteria;Azospirillum"
3567	tmo	Tistrella mobilis	"Prokaryotes;Bacteria;Alphaproteobacteria;Tistrella"
3568	thal	Candidatus Endolissoclinum faulkneri L2	"Prokaryotes;Bacteria;Alphaproteobacteria;Endolissoclinum"
3569	efk	Candidatus Endolissoclinum faulkneri L5	"Prokaryotes;Bacteria;Alphaproteobacteria;Endolissoclinum"
3570	txi	Thalassospira xiamenensis	"Prokaryotes;Bacteria;Alphaproteobacteria;Thalassospira"
3571	thac	Thalassospira marina	"Prokaryotes;Bacteria;Alphaproteobacteria;Thalassospira"
3572	tii	Thalassospira indica	"Prokaryotes;Bacteria;Alphaproteobacteria;Thalassospira"
3573	magq	Magnetospira sp. QH-2	"Prokaryotes;Bacteria;Alphaproteobacteria;Magnetospira"
3574	hjo	Haematospirillum jordaniae	"Prokaryotes;Bacteria;Alphaproteobacteria;Haematospirillum"
3575	nao	Nitrospirillum amazonense	"Prokaryotes;Bacteria;Alphaproteobacteria;Nitrospirillum"
3576	ncb	Niveispirillum cyanobacteriorum	"Prokaryotes;Bacteria;Alphaproteobacteria;Niveispirillum"
3577	fer	Ferrovibrio terrae	"Prokaryotes;Bacteria;Alphaproteobacteria;Ferrovibrio"
3578	htq	Hypericibacter terrae	"Prokaryotes;Bacteria;Alphaproteobacteria;Hypericibacter"
3579	hadh	Hypericibacter adhaerens	"Prokaryotes;Bacteria;Alphaproteobacteria;Hypericibacter"
3580	dex	Defluviicoccus sp. SSA4	"Prokaryotes;Bacteria;Alphaproteobacteria;Defluviicoccus"
3581	dvn	Defluviicoccus vanus	"Prokaryotes;Bacteria;Alphaproteobacteria;Defluviicoccus"
3582	skt	Skermanella sp. TT6	"Prokaryotes;Bacteria;Alphaproteobacteria;Skermanella"
3583	pbr	Parvularcula bermudensis	"Prokaryotes;Bacteria;Alphaproteobacteria;Parvularcula"
3584	mgm	Magnetococcus marinus	"Prokaryotes;Bacteria;Alphaproteobacteria;Magnetococcus"
3585	pub	Candidatus Pelagibacter ubique	"Prokaryotes;Bacteria;Alphaproteobacteria;Pelagibacter"
3586	pel	Candidatus Pelagibacter sp. IMCC9063	"Prokaryotes;Bacteria;Alphaproteobacteria;Pelagibacter"
3587	peg	Candidatus Pelagibacter giovannonii	"Prokaryotes;Bacteria;Alphaproteobacteria;Pelagibacter"
3588	apc	Alpha proteobacterium HIMB59	"Prokaryotes;Bacteria;Alphaproteobacteria;unclassified Pelagibacteraceae"
3589	apm	Alpha proteobacterium HIMB5	"Prokaryotes;Bacteria;Alphaproteobacteria;unclassified Pelagibacteraceae"
3590	ecog	Paremcibacter congregatus	"Prokaryotes;Bacteria;Alphaproteobacteria;Paremcibacter"
3591	mai	Micavibrio aeruginosavorus ARL-13	"Prokaryotes;Bacteria;Alphaproteobacteria;Micavibrio"
3592	man	Micavibrio aeruginosavorus EPB	"Prokaryotes;Bacteria;Alphaproteobacteria;Micavibrio"
3593	phr	Phreatobacter cathodiphilus	"Prokaryotes;Bacteria;Alphaproteobacteria;Phreatobacter"
3594	pstg	Phreatobacter stygius	"Prokaryotes;Bacteria;Alphaproteobacteria;Phreatobacter"

3595	apb	Candidatus Puniceispirillum marinum	"Prokaryotes;Bacteria;Alphaproteobacteria;Puniceispirillum"
3596	bba	Bdellovibrio bacteriovorus HD100	"Prokaryotes;Bacteria;Oligoflexia;Bdellovibrio"
3597	bbat	Bdellovibrio bacteriovorus Tiberius	"Prokaryotes;Bacteria;Oligoflexia;Bdellovibrio"
3598	bbw	Bdellovibrio bacteriovorus W	"Prokaryotes;Bacteria;Oligoflexia;Bdellovibrio"
3599	bbac	Bdellovibrio bacteriovorus 109J	"Prokaryotes;Bacteria;Oligoflexia;Bdellovibrio"
3600	bex	Bdellovibrio exovorus	"Prokaryotes;Bacteria;Oligoflexia;Bdellovibrio"
3601	bdq	Bdellovibrio sp. qaytius	"Prokaryotes;Bacteria;Oligoflexia;Bdellovibrio"
3602	bdc	Bdellovibrio sp. NC01	"Prokaryotes;Bacteria;Oligoflexia;Bdellovibrio"
3603	bdz	Bdellovibrio sp. ZAP7	"Prokaryotes;Bacteria;Oligoflexia;Bdellovibrio"
3604	bmx	Halobacteriovorax marinus	"Prokaryotes;Bacteria;Oligoflexia;Halobacteriovorax"
3605	hax	Halobacteriovorax sp. BALOs_7	"Prokaryotes;Bacteria;Oligoflexia;Halobacteriovorax"
3606	bsto	Bacteriovorax stolpii	"Prokaryotes;Bacteria;Oligoflexia;Bacteriovorax"
3607	sbf	Silvanigrellales bacterium RF1110005	"Prokaryotes;Bacteria;Oligoflexia;unclassified Silvanigrellales"
3608	afr	Acidithiobacillus ferrooxidans ATCC 23270	"Prokaryotes;Bacteria;Other proteobacteria;Acidithiobacillus"
3609	afe	Acidithiobacillus ferrooxidans ATCC 53993	"Prokaryotes;Bacteria;Other proteobacteria;Acidithiobacillus"
3610	acu	Acidithiobacillus caldus SM-1	"Prokaryotes;Bacteria;Other proteobacteria;Acidithiobacillus"
3611	acz	Acidithiobacillus caldus ATCC 51756	"Prokaryotes;Bacteria;Other proteobacteria;Acidithiobacillus"
3612	afi	Acidithiobacillus ferrivorans	"Prokaryotes;Bacteria;Other proteobacteria;Acidithiobacillus"
3613	afj	Acidithiobacillus ferridurans	"Prokaryotes;Bacteria;Other proteobacteria;Acidithiobacillus"
3614	atx	Acidithiobacillus thiooxidans	"Prokaryotes;Bacteria;Other proteobacteria;Acidithiobacillus"
3615	maes	Mariprofundus aestuarium	"Prokaryotes;Bacteria;Other proteobacteria;Mariprofundus"
3616	mfn	Mariprofundus ferrinatatus	"Prokaryotes;Bacteria;Other proteobacteria;Mariprofundus"
3617	htl	Hydrogenophilus thermoluteolus	"Prokaryotes;Bacteria;Other proteobacteria;Hydrogenophilus"
3618	bsu	Bacillus subtilis subsp. subtilis 168	"Prokaryotes;Bacteria;Firmicutes - Bacilli;Bacillus"
3619	bsr	Bacillus subtilis subsp. subtilis RO-NN-1	"Prokaryotes;Bacteria;Firmicutes - Bacilli;Bacillus"
3620	bsl	Bacillus subtilis subsp. subtilis BSP1	"Prokaryotes;Bacteria;Firmicutes - Bacilli;Bacillus"
3621	bsh	Bacillus subtilis subsp. subtilis 6051-HGW	"Prokaryotes;Bacteria;Firmicutes - Bacilli;Bacillus"
3622	bsy	Bacillus subtilis subsp. subtilis BAB-1	"Prokaryotes;Bacteria;Firmicutes - Bacilli;Bacillus"
3623	bsut	Bacillus subtilis subsp. subtilis AG1839	"Prokaryotes;Bacteria;Firmicutes - Bacilli;Bacillus"
3624	bsul	Bacillus subtilis subsp. subtilis JH642	"Prokaryotes;Bacteria;Firmicutes - Bacilli;Bacillus"
3625	bsus	Bacillus subtilis subsp. subtilis OH 131.1	"Prokaryotes;Bacteria;Firmicutes - Bacilli;Bacillus"
3626	bso	Bacillus subtilis subsp. natto BEST195	"Prokaryotes;Bacteria;Firmicutes - Bacilli;Bacillus"
3627	bsn	Bacillus subtilis BSn5	"Prokaryotes;Bacteria;Firmicutes - Bacilli;Bacillus"
3628	bsq	Bacillus subtilis QB928	"Prokaryotes;Bacteria;Firmicutes - Bacilli;Bacillus"
3629	bsx	Bacillus subtilis XF-1	"Prokaryotes;Bacteria;Firmicutes - Bacilli;Bacillus"
3630	bsp	Bacillus subtilis PY79	"Prokaryotes;Bacteria;Firmicutes - Bacilli;Bacillus"
3631	bss	Bacillus subtilis subsp. spizizenii W23	"Prokaryotes;Bacteria;Firmicutes - Bacilli;Bacillus"
3632	bst	Bacillus subtilis subsp. spizizenii TU-B-10	"Prokaryotes;Bacteria;Firmicutes - Bacilli;Bacillus"
3633	bli	Bacillus licheniformis ATCC 14580	"Prokaryotes;Bacteria;Firmicutes - Bacilli;Bacillus"
3634	bld	Bacillus licheniformis DSM 13 = ATCC 14580	"Prokaryotes;Bacteria;Firmicutes - Bacilli;Bacillus"
3635	blh	Bacillus paralicheniformis	"Prokaryotes;Bacteria;Firmicutes - Bacilli;Bacillus"

3636	bay	Bacillus velezensis FZB42	"Prokaryotes;Bacteria;Firmicutes - Bacilli;Bacillus"
3637	baq	Bacillus velezensis CAU B946	"Prokaryotes;Bacteria;Firmicutes - Bacilli;Bacillus"
3638	bya	Bacillus velezensis YAU B9601-Y2	"Prokaryotes;Bacteria;Firmicutes - Bacilli;Bacillus"
3639	bamp	Bacillus velezensis AS43.3	"Prokaryotes;Bacteria;Firmicutes - Bacilli;Bacillus"
3640	baml	Bacillus velezensis UCMB5036	"Prokaryotes;Bacteria;Firmicutes - Bacilli;Bacillus"
3641	bama	Bacillus velezensis UCMB5033	"Prokaryotes;Bacteria;Firmicutes - Bacilli;Bacillus"
3642	bamn	Bacillus velezensis UCMB5113	"Prokaryotes;Bacteria;Firmicutes - Bacilli;Bacillus"
3643	bamb	Bacillus velezensis NAU-B3	"Prokaryotes;Bacteria;Firmicutes - Bacilli;Bacillus"
3644	bamt	Bacillus velezensis TrigoCor1448	"Prokaryotes;Bacteria;Firmicutes - Bacilli;Bacillus"
3645	bamy	Bacillus velezensis SQR9	"Prokaryotes;Bacteria;Firmicutes - Bacilli;Bacillus"
3646	bmp	Bacillus velezensis JS25R	"Prokaryotes;Bacteria;Firmicutes - Bacilli;Bacillus"
3647	bao	Bacillus amyloliquefaciens DSM 7	"Prokaryotes;Bacteria;Firmicutes - Bacilli;Bacillus"
3648	baz	Bacillus amyloliquefaciens TA208	"Prokaryotes;Bacteria;Firmicutes - Bacilli;Bacillus"
3649	bql	Bacillus amyloliquefaciens LL3	"Prokaryotes;Bacteria;Firmicutes - Bacilli;Bacillus"
3650	bxh	Bacillus amyloliquefaciens XH7	"Prokaryotes;Bacteria;Firmicutes - Bacilli;Bacillus"
3651	bqy	Bacillus amyloliquefaciens Y2	"Prokaryotes;Bacteria;Firmicutes - Bacilli;Bacillus"
3652	bami	Bacillus amyloliquefaciens IT-45	"Prokaryotes;Bacteria;Firmicutes - Bacilli;Bacillus"
3653	bamc	Bacillus amyloliquefaciens CC178	"Prokaryotes;Bacteria;Firmicutes - Bacilli;Bacillus"
3654	bamf	Bacillus amyloliquefaciens LFB112	"Prokaryotes;Bacteria;Firmicutes - Bacilli;Bacillus"
3655	bsia	Bacillus siamensis	"Prokaryotes;Bacteria;Firmicutes - Bacilli;Bacillus"
3656	bae	Bacillus atrophaeus	"Prokaryotes;Bacteria;Firmicutes - Bacilli;Bacillus"
3657	bvm	Bacillus vallismortis	"Prokaryotes;Bacteria;Firmicutes - Bacilli;Bacillus"
3658	bson	Bacillus sonorensis	"Prokaryotes;Bacteria;Firmicutes - Bacilli;Bacillus"
3659	bht	Bacillus halotolerans	"Prokaryotes;Bacteria;Firmicutes - Bacilli;Bacillus"
3660	ban	Bacillus anthracis Ames	"Prokaryotes;Bacteria;Firmicutes - Bacilli;Bacillus"
3661	bar	Bacillus anthracis Ames Ancestor	"Prokaryotes;Bacteria;Firmicutes - Bacilli;Bacillus"
3662	bat	Bacillus anthracis Sterne	"Prokaryotes;Bacteria;Firmicutes - Bacilli;Bacillus"
3663	bah	Bacillus anthracis CDC 684	"Prokaryotes;Bacteria;Firmicutes - Bacilli;Bacillus"
3664	bai	Bacillus anthracis A0248	"Prokaryotes;Bacteria;Firmicutes - Bacilli;Bacillus"
3665	bax	Bacillus anthracis H9401	"Prokaryotes;Bacteria;Firmicutes - Bacilli;Bacillus"
3666	bant	Bacillus anthracis A16	"Prokaryotes;Bacteria;Firmicutes - Bacilli;Bacillus"
3667	banr	Bacillus anthracis A16R	"Prokaryotes;Bacteria;Firmicutes - Bacilli;Bacillus"
3668	bans	Bacillus anthracis SVA11	"Prokaryotes;Bacteria;Firmicutes - Bacilli;Bacillus"
3669	banh	Bacillus anthracis HYU01	"Prokaryotes;Bacteria;Firmicutes - Bacilli;Bacillus"
3670	banv	Bacillus anthracis Vollum	"Prokaryotes;Bacteria;Firmicutes - Bacilli;Bacillus"
3671	bce	Bacillus cereus ATCC 14579	"Prokaryotes;Bacteria;Firmicutes - Bacilli;Bacillus"
3672	bca	Bacillus cereus ATCC 10987	"Prokaryotes;Bacteria;Firmicutes - Bacilli;Bacillus"
3673	bcz	Bacillus cereus E33L	"Prokaryotes;Bacteria;Firmicutes - Bacilli;Bacillus"
3674	bcr	Bacillus cereus AH187	"Prokaryotes;Bacteria;Firmicutes - Bacilli;Bacillus"
3675	bcb	Bacillus cereus B4264	"Prokaryotes;Bacteria;Firmicutes - Bacilli;Bacillus"
3676	bcu	Bacillus cereus AH820	"Prokaryotes;Bacteria;Firmicutes - Bacilli;Bacillus"

3677	bcg	Bacillus cereus G9842	"Prokaryotes;Bacteria;Firmicutes - Bacilli;Bacillus"
3678	bcq	Bacillus cereus Q1	"Prokaryotes;Bacteria;Firmicutes - Bacilli;Bacillus"
3679	bcx	Bacillus cereus 03BB102	"Prokaryotes;Bacteria;Firmicutes - Bacilli;Bacillus"
3680	bal	Bacillus cereus biovar anthracis CI	"Prokaryotes;Bacteria;Firmicutes - Bacilli;Bacillus"
3681	bnc	Bacillus cereus NC7401	"Prokaryotes;Bacteria;Firmicutes - Bacilli;Bacillus"
3682	bcf	Bacillus cereus F837/76	"Prokaryotes;Bacteria;Firmicutes - Bacilli;Bacillus"
3683	bcer	Bacillus cereus FRI-35	"Prokaryotes;Bacteria;Firmicutes - Bacilli;Bacillus"
3684	bcef	Bacillus cereus FT9	"Prokaryotes;Bacteria;Firmicutes - Bacilli;Bacillus"
3685	bcy	Bacillus cytotoxicus	"Prokaryotes;Bacteria;Firmicutes - Bacilli;Bacillus"
3686	btk	Bacillus thuringiensis serovar konkukian 97-27	"Prokaryotes;Bacteria;Firmicutes - Bacilli;Bacillus"
3687	btl	Bacillus thuringiensis Al Hakam	"Prokaryotes;Bacteria;Firmicutes - Bacilli;Bacillus"
3688	btb	Bacillus thuringiensis BMB171	"Prokaryotes;Bacteria;Firmicutes - Bacilli;Bacillus"
3689	btt	Bacillus thuringiensis serovar kurstaki HD73	"Prokaryotes;Bacteria;Firmicutes - Bacilli;Bacillus"
3690	bthr	Bacillus thuringiensis serovar kurstaki YBT-1520	"Prokaryotes;Bacteria;Firmicutes - Bacilli;Bacillus"
3691	bthi	Bacillus thuringiensis serovar kurstaki HD-1	"Prokaryotes;Bacteria;Firmicutes - Bacilli;Bacillus"
3692	btc	Bacillus thuringiensis serovar chinensis CT-43	"Prokaryotes;Bacteria;Firmicutes - Bacilli;Bacillus"
3693	btf	Bacillus thuringiensis serovar finitimus YBT-020	"Prokaryotes;Bacteria;Firmicutes - Bacilli;Bacillus"
3694	btm	Bacillus thuringiensis MC28	"Prokaryotes;Bacteria;Firmicutes - Bacilli;Bacillus"
3695	btg	Bacillus thuringiensis Bt407	"Prokaryotes;Bacteria;Firmicutes - Bacilli;Bacillus"
3696	bti	Bacillus thuringiensis HD-771	"Prokaryotes;Bacteria;Firmicutes - Bacilli;Bacillus"
3697	btn	Bacillus thuringiensis HD-789	"Prokaryotes;Bacteria;Firmicutes - Bacilli;Bacillus"
3698	btht	Bacillus thuringiensis serovar thuringiensis IS5056	"Prokaryotes;Bacteria;Firmicutes - Bacilli;Bacillus"
3699	bthu	Bacillus thuringiensis YBT-1518	"Prokaryotes;Bacteria;Firmicutes - Bacilli;Bacillus"
3700	btw	Bacillus thuringiensis HD1011	"Prokaryotes;Bacteria;Firmicutes - Bacilli;Bacillus"
3701	bthy	Bacillus thuringiensis YWC2-8	"Prokaryotes;Bacteria;Firmicutes - Bacilli;Bacillus"
3702	bwe	Bacillus mycoides KBAB4	"Prokaryotes;Bacteria;Firmicutes - Bacilli;Bacillus"
3703	bww	Bacillus mycoides WSBC 10204	"Prokaryotes;Bacteria;Firmicutes - Bacilli;Bacillus"
3704	bmyo	Bacillus mycoides ATCC 6462	"Prokaryotes;Bacteria;Firmicutes - Bacilli;Bacillus"
3705	bty	Bacillus toyonensis	"Prokaryotes;Bacteria;Firmicutes - Bacilli;Bacillus"
3706	bmyc	Bacillus pseudomycoides 219298	"Prokaryotes;Bacteria;Firmicutes - Bacilli;Bacillus"
3707	bby	Bacillus bombysepticus	"Prokaryotes;Bacteria;Firmicutes - Bacilli;Bacillus"
3708	bwd	Bacillus wiedmannii	"Prokaryotes;Bacteria;Firmicutes - Bacilli;Bacillus"
3709	btro	Bacillus tropicus	"Prokaryotes;Bacteria;Firmicutes - Bacilli;Bacillus"
3710	bmob	Bacillus mobilis	"Prokaryotes;Bacteria;Firmicutes - Bacilli;Bacillus"
3711	bpu	Bacillus pumilus SAFR-032	"Prokaryotes;Bacteria;Firmicutes - Bacilli;Bacillus"
3712	bpum	Bacillus pumilus MTCC B6033	"Prokaryotes;Bacteria;Firmicutes - Bacilli;Bacillus"
3713	bpus	Bacillus pumilus SH-B9	"Prokaryotes;Bacteria;Firmicutes - Bacilli;Bacillus"
3714	bco	Bacillus cellulosilyticus	"Prokaryotes;Bacteria;Firmicutes - Bacilli;Bacillus"
3715	bjs	Bacillus sp. JS	"Prokaryotes;Bacteria;Firmicutes - Bacilli;Bacillus"
3716	baci	Bacillus sp. 1NLA3E	"Prokaryotes;Bacteria;Firmicutes - Bacilli;Bacillus"
3717	bif	Bacillus infantis	"Prokaryotes;Bacteria;Firmicutes - Bacilli;Bacillus"

3718	bmet	Bacillus methanolicus	"Prokaryotes;Bacteria;Firmicutes - Bacilli;Bacillus"
3719	gst	Bacillus sp. X1(2014)	"Prokaryotes;Bacteria;Firmicutes - Bacilli;Bacillus"
3720	bacw	Bacillus sp. WP8	"Prokaryotes;Bacteria;Firmicutes - Bacilli;Bacillus"
3721	bacp	Bacillus sp. Pc3	"Prokaryotes;Bacteria;Firmicutes - Bacilli;Bacillus"
3722	bacb	Bacillus sp. BH072	"Prokaryotes;Bacteria;Firmicutes - Bacilli;Bacillus"
3723	baco	Bacillus sp. OxB-1	"Prokaryotes;Bacteria;Firmicutes - Bacilli;Bacillus"
3724	bacy	Bacillus sp. YP1	"Prokaryotes;Bacteria;Firmicutes - Bacilli;Bacillus"
3725	bacl	Bacillus sp. BS34A	"Prokaryotes;Bacteria;Firmicutes - Bacilli;Bacillus"
3726	balm	Bacillus sp. LM 4-2	"Prokaryotes;Bacteria;Firmicutes - Bacilli;Bacillus"
3727	bsm	Bacillus smithii	"Prokaryotes;Bacteria;Firmicutes - Bacilli;Bacillus"
3728	bgy	Bacillus glycinifermentans	"Prokaryotes;Bacteria;Firmicutes - Bacilli;Bacillus"
3729	bwh	Bacillus weihaiensis	"Prokaryotes;Bacteria;Firmicutes - Bacilli;Bacillus"
3730	bxi	Bacillus xiamenensis	"Prokaryotes;Bacteria;Firmicutes - Bacilli;Bacillus"
3731	bhk	Bacillus horikoshii	"Prokaryotes;Bacteria;Firmicutes - Bacilli;Bacillus"
3732	bbev	Bacillus beveridgei	"Prokaryotes;Bacteria;Firmicutes - Bacilli;Bacillus"
3733	balt	Bacillus altitudinis	"Prokaryotes;Bacteria;Firmicutes - Bacilli;Bacillus"
3734	bacs	Bacillus sp. SDLI1	"Prokaryotes;Bacteria;Firmicutes - Bacilli;Bacillus"
3735	bsaf	Bacillus safensis	"Prokaryotes;Bacteria;Firmicutes - Bacilli;Bacillus"
3736	bit	Bacillus intestinalis	"Prokaryotes;Bacteria;Firmicutes - Bacilli;Bacillus"
3737	bacq	Bacillus sp. Y1	"Prokaryotes;Bacteria;Firmicutes - Bacilli;Bacillus"
3738	bcir	Bacillus circulans	"Prokaryotes;Bacteria;Firmicutes - Bacilli;Bacillus"
3739	bfd	Bacillus freudenreichii	"Prokaryotes;Bacteria;Firmicutes - Bacilli;Bacillus"
3740	bcoh	Bacillus cohnii	"Prokaryotes;Bacteria;Firmicutes - Bacilli;Bacillus"
3741	bda	Bacillus dafuensis	"Prokaryotes;Bacteria;Firmicutes - Bacilli;Bacillus"
3742	beo	Priestia filamentosa	"Prokaryotes;Bacteria;Firmicutes - Bacilli;Bacillus"
3743	bmq	Bacillus megaterium QM B1551	"Prokaryotes;Bacteria;Firmicutes - Bacilli;Priestia"
3744	bmd	Bacillus megaterium DSM 319	"Prokaryotes;Bacteria;Firmicutes - Bacilli;Priestia"
3745	bmh	Bacillus megaterium WSH-002	"Prokaryotes;Bacteria;Firmicutes - Bacilli;Priestia"
3746	bmeg	Bacillus megaterium NBRC 15308 = ATCC 14581	"Prokaryotes;Bacteria;Firmicutes - Bacilli;Priestia"
3747	bfx	Priestia flexa	"Prokaryotes;Bacteria;Firmicutes - Bacilli;Priestia"
3748	bck	Bacillus coagulans 2-6	"Prokaryotes;Bacteria;Firmicutes - Bacilli;Weizmannia"
3749	bag	Bacillus coagulans 36D1	"Prokaryotes;Bacteria;Firmicutes - Bacilli;Weizmannia"
3750	bcoa	Bacillus coagulans DSM 1 = ATCC 7050	"Prokaryotes;Bacteria;Firmicutes - Bacilli;Weizmannia"
3751	bha	Bacillus halodurans	"Prokaryotes;Bacteria;Firmicutes - Bacilli;Alkalihalobacillus"
3752	bcl	Bacillus clausii	"Prokaryotes;Bacteria;Firmicutes - Bacilli;Alkalihalobacillus"
3753	bpf	Bacillus pseudofirmus	"Prokaryotes;Bacteria;Firmicutes - Bacilli;Alkalihalobacillus"
3754	ble	Bacillus lehensis	"Prokaryotes;Bacteria;Firmicutes - Bacilli;Alkalihalobacillus"
3755	bkw	Alkalihalobacillus krulwichiae	"Prokaryotes;Bacteria;Firmicutes - Bacilli;Alkalihalobacillus"
3756	bgi	Alkalihalobacillus gibsonii	"Prokaryotes;Bacteria;Firmicutes - Bacilli;Alkalihalobacillus"
3757	bon	Bacillus oceanisediminis	"Prokaryotes;Bacteria;Firmicutes - Bacilli;Cytobacillus"
3758	bko	Cytobacillus kochii	"Prokaryotes;Bacteria;Firmicutes - Bacilli;Cytobacillus"

3759	oih	Oceanobacillus iheyensis	"Prokaryotes;Bacteria;Firmicutes - Bacilli;Oceanobacillus"
3760	ocn	Oceanobacillus zhaokaii	"Prokaryotes;Bacteria;Firmicutes - Bacilli;Oceanobacillus"
3761	ocb	Oceanobacillus sp. 143	"Prokaryotes;Bacteria;Firmicutes - Bacilli;Oceanobacillus"
3762	gka	Geobacillus kaustophilus	"Prokaryotes;Bacteria;Firmicutes - Bacilli;Geobacillus"
3763	gte	Geobacillus thermoleovorans CCB_US3_UF5	"Prokaryotes;Bacteria;Firmicutes - Bacilli;Geobacillus"
3764	gtk	Geobacillus thermoleovorans KCTC 3570	"Prokaryotes;Bacteria;Firmicutes - Bacilli;Geobacillus"
3765	gtm	Geobacillus thermocatenulatus	"Prokaryotes;Bacteria;Firmicutes - Bacilli;Geobacillus"
3766	gli	Geobacillus lituanicus	"Prokaryotes;Bacteria;Firmicutes - Bacilli;Geobacillus"
3767	gtn	Geobacillus thermodenitrificans	"Prokaryotes;Bacteria;Firmicutes - Bacilli;Geobacillus"
3768	gwc	Geobacillus sp. WCH70	"Prokaryotes;Bacteria;Firmicutes - Bacilli;Geobacillus"
3769	gyc	Geobacillus sp. Y412MC61	"Prokaryotes;Bacteria;Firmicutes - Bacilli;Geobacillus"
3770	gya	Geobacillus sp. Y412MC52	"Prokaryotes;Bacteria;Firmicutes - Bacilli;Geobacillus"
3771	gct	Geobacillus sp. C56-T3	"Prokaryotes;Bacteria;Firmicutes - Bacilli;Geobacillus"
3772	gmc	Geobacillus sp. Y4.1MC1	"Prokaryotes;Bacteria;Firmicutes - Bacilli;Geobacillus"
3773	ggh	Geobacillus sp. GHH01	"Prokaryotes;Bacteria;Firmicutes - Bacilli;Geobacillus"
3774	gjf	Geobacillus genomosp. 3	"Prokaryotes;Bacteria;Firmicutes - Bacilli;Geobacillus"
3775	gea	Geobacillus sp. 12AMOR1	"Prokaryotes;Bacteria;Firmicutes - Bacilli;Geobacillus"
3776	gel	Geobacillus sp. LC300	"Prokaryotes;Bacteria;Firmicutes - Bacilli;Geobacillus"
3777	gse	Geobacillus stearothermophilus	"Prokaryotes;Bacteria;Firmicutes - Bacilli;Geobacillus"
3778	gsr	Geobacillus subterraneus	"Prokaryotes;Bacteria;Firmicutes - Bacilli;Geobacillus"
3779	gej	Geobacillus sp. JS12	"Prokaryotes;Bacteria;Firmicutes - Bacilli;Geobacillus"
3780	gth	Parageobacillus thermoglucosidasius C56-YS93	"Prokaryotes;Bacteria;Firmicutes - Bacilli;Parageobacillus"
3781	ptl	Parageobacillus thermoglucosidasius DSM 2542	"Prokaryotes;Bacteria;Firmicutes - Bacilli;Parageobacillus"
3782	ptb	Parageobacillus toebii NBRC 107807	"Prokaryotes;Bacteria;Firmicutes - Bacilli;Parageobacillus"
3783	afl	Anoxybacillus flavithermus	"Prokaryotes;Bacteria;Firmicutes - Bacilli;Anoxybacillus"
3784	agn	Anoxybacillus gonensis	"Prokaryotes;Bacteria;Firmicutes - Bacilli;Anoxybacillus"
3785	anm	Anoxybacillus sp. B2M1	"Prokaryotes;Bacteria;Firmicutes - Bacilli;Anoxybacillus"
3786	aamy	Anoxybacillus amylolyticus	"Prokaryotes;Bacteria;Firmicutes - Bacilli;Anoxybacillus"
3787	anl	Anoxybacillus sp. B7M1	"Prokaryotes;Bacteria;Firmicutes - Bacilli;Anoxybacillus"
3788	and	Anoxybacillus sp. PDR2	"Prokaryotes;Bacteria;Firmicutes - Bacilli;Anoxybacillus"
3789	acai	Anoxybacillus caldiproteolyticus	"Prokaryotes;Bacteria;Firmicutes - Bacilli;Anoxybacillus"
3790	axl	Amphibacillus xylanus	"Prokaryotes;Bacteria;Firmicutes - Bacilli;Amphibacillus"
3791	lsp	Lysinibacillus sphaericus	"Prokaryotes;Bacteria;Firmicutes - Bacilli;Lysinibacillus"
3792	lgy	Lysinibacillus varians	"Prokaryotes;Bacteria;Firmicutes - Bacilli;Lysinibacillus"
3793	lfu	Lysinibacillus fusiformis	"Prokaryotes;Bacteria;Firmicutes - Bacilli;Lysinibacillus"
3794	lys	Lysinibacillus sp. YS11	"Prokaryotes;Bacteria;Firmicutes - Bacilli;Lysinibacillus"
3795	lyb	Lysinibacillus sp. B2A1	"Prokaryotes;Bacteria;Firmicutes - Bacilli;Lysinibacillus"
3796	lyz	Lysinibacillus sp. 2017	"Prokaryotes;Bacteria;Firmicutes - Bacilli;Lysinibacillus"
3797	lyg	Lysinibacillus sp. SGAir0095	"Prokaryotes;Bacteria;Firmicutes - Bacilli;Lysinibacillus"
3798	lpak	Lysinibacillus pakistanensis	"Prokaryotes;Bacteria;Firmicutes - Bacilli;Lysinibacillus"
3799	hhd	Halobacillus halophilus	"Prokaryotes;Bacteria;Firmicutes - Bacilli;Halobacillus"

3800	hmn	Halobacillus mangrovi	"Prokaryotes;Bacteria;Firmicutes - Bacilli;Halobacillus"
3801	hli	Halobacillus litoralis	"Prokaryotes;Bacteria;Firmicutes - Bacilli;Halobacillus"
3802	tap	Terribacillus goriensis	"Prokaryotes;Bacteria;Firmicutes - Bacilli;Terribacillus"
3803	vir	Virgibacillus sp. SK37	"Prokaryotes;Bacteria;Firmicutes - Bacilli;Virgibacillus"
3804	vhl	Virgibacillus halodenitrificans	"Prokaryotes;Bacteria;Firmicutes - Bacilli;Virgibacillus"
3805	vig	Virgibacillus sp. 6R	"Prokaryotes;Bacteria;Firmicutes - Bacilli;Virgibacillus"
3806	vil	Virgibacillus phasianinus	"Prokaryotes;Bacteria;Firmicutes - Bacilli;Virgibacillus"
3807	vne	Virgibacillus necropolis	"Prokaryotes;Bacteria;Firmicutes - Bacilli;Virgibacillus"
3808	vpn	Virgibacillus dokdonensis	"Prokaryotes;Bacteria;Firmicutes - Bacilli;Virgibacillus"
3809	vim	Virgibacillus sp. MSP4-1	"Prokaryotes;Bacteria;Firmicutes - Bacilli;Virgibacillus"
3810	lao	Lentibacillus amyloliquefaciens	"Prokaryotes;Bacteria;Firmicutes - Bacilli;Lentibacillus"
3811	fpn	Fictibacillus phosphorivorans	"Prokaryotes;Bacteria;Firmicutes - Bacilli;Fictibacillus"
3812	far	Fictibacillus arsenicus	"Prokaryotes;Bacteria;Firmicutes - Bacilli;Fictibacillus"
3813	sje	Salimicrobium jeotgali	"Prokaryotes;Bacteria;Firmicutes - Bacilli;Salimicrobium"
3814	apak	Aeribacillus pallidus	"Prokaryotes;Bacteria;Firmicutes - Bacilli;Aeribacillus"
3815	bsj	Peribacillus simplex	"Prokaryotes;Bacteria;Firmicutes - Bacilli;Peribacillus"
3816	bmur	Peribacillus muralis	"Prokaryotes;Bacteria;Firmicutes - Bacilli;Peribacillus"
3817	pbut	Peribacillus butanolivorans	"Prokaryotes;Bacteria;Firmicutes - Bacilli;Peribacillus"
3818	pasa	Peribacillus asahii	"Prokaryotes;Bacteria;Firmicutes - Bacilli;Peribacillus"
3819	aqt	Radiobacillus deserti	"Prokaryotes;Bacteria;Firmicutes - Bacilli;Aquibacillus"
3820	bthv	Caldibacillus thermoamylovorans	"Prokaryotes;Bacteria;Firmicutes - Bacilli;Caldibacillus"
3821	psyh	Psychrobacillus sp. AK 1817	"Prokaryotes;Bacteria;Firmicutes - Bacilli;Psychrobacillus"
3822	psyo	Psychrobacillus glaciei	"Prokaryotes;Bacteria;Firmicutes - Bacilli;Psychrobacillus"
3823	prd	Pradoshia sp. D12	"Prokaryotes;Bacteria;Firmicutes - Bacilli;Pradoshia"
3824	grc	Gracilibacillus sp. SCU50	"Prokaryotes;Bacteria;Firmicutes - Bacilli;Gracilibacillus"
3825	rue	Salicibibacter kimchii	"Prokaryotes;Bacteria;Firmicutes - Bacilli;Salicibibacter"
3826	sale	Salicibibacter halophilus	"Prokaryotes;Bacteria;Firmicutes - Bacilli;Salicibibacter"
3827	pof	Pontibacillus sp. HMF3514	"Prokaryotes;Bacteria;Firmicutes - Bacilli;Pontibacillus"
3828	nmk	Neobacillus mesonae	"Prokaryotes;Bacteria;Firmicutes - Bacilli;Neobacillus"
3829	ntm	Neobacillus thermocopriae	"Prokaryotes;Bacteria;Firmicutes - Bacilli;Neobacillus"
3830	meku	Metabacillus sp. KUDC1714	"Prokaryotes;Bacteria;Firmicutes - Bacilli;Metabacillus"
3831	aia	Anaerobacillus isosaccharinicus	"Prokaryotes;Bacteria;Firmicutes - Bacilli;Anaerobacillus"
3832	blen	Lederbergia lentus	"Prokaryotes;Bacteria;Firmicutes - Bacilli;Lederbergia"
3833	stea	Sporolactobacillus terrae	"Prokaryotes;Bacteria;Firmicutes - Bacilli;Sporolactobacillus"
3834	bse	Bacillus selenitireducens	"Prokaryotes;Bacteria;Firmicutes - Bacilli;unclassified Sporolactobacillaceae"
3835	sau	Staphylococcus aureus subsp. aureus N315 (MRSA/VSSA)	"Prokaryotes;Bacteria;Firmicutes - Bacilli;Staphylococcus"
3836	sav	Staphylococcus aureus subsp. aureus Mu50 (MRSA/VISA)	"Prokaryotes;Bacteria;Firmicutes - Bacilli;Staphylococcus"
3837	saw	Staphylococcus aureus subsp. aureus Mu3 (MRSA/hetero-VISA)	"Prokaryotes;Bacteria;Firmicutes - Bacilli;Staphylococcus"
3838	sah	Staphylococcus aureus subsp. aureus JH1 (MRSA/VSSA)	"Prokaryotes;Bacteria;Firmicutes - Bacilli;Staphylococcus"
3839	saj	Staphylococcus aureus subsp. aureus JH9 (MRSA/VISA)	"Prokaryotes;Bacteria;Firmicutes - Bacilli;Staphylococcus"
3840	sam	Staphylococcus aureus subsp. aureus MW2 (CA-MRSA)	"Prokaryotes;Bacteria;Firmicutes - Bacilli;Staphylococcus"

3841	sas	Staphylococcus aureus subsp. aureus MSSA476 (MSSA)	"Prokaryotes;Bacteria;Firmicutes - Bacilli;Staphylococcus"
3842	sar	Staphylococcus aureus subsp. aureus MRSA252 (MRSA)	"Prokaryotes;Bacteria;Firmicutes - Bacilli;Staphylococcus"
3843	sac	Staphylococcus aureus subsp. aureus COL (MRSA)	"Prokaryotes;Bacteria;Firmicutes - Bacilli;Staphylococcus"
3844	sax	Staphylococcus aureus subsp. aureus USA300_TCH1516 (CA-MRSA)	"Prokaryotes;Bacteria;Firmicutes - Bacilli;Staphylococcus"
3845	saa	Staphylococcus aureus subsp. aureus USA300_FPR3757 (CA-MRSA)	"Prokaryotes;Bacteria;Firmicutes - Bacilli;Staphylococcus"
3846	sao	Staphylococcus aureus subsp. aureus NCTC8325	"Prokaryotes;Bacteria;Firmicutes - Bacilli;Staphylococcus"
3847	sae	Staphylococcus aureus subsp. aureus Newman	"Prokaryotes;Bacteria;Firmicutes - Bacilli;Staphylococcus"
3848	sad	Staphylococcus aureus subsp. aureus ED98	"Prokaryotes;Bacteria;Firmicutes - Bacilli;Staphylococcus"
3849	suu	Staphylococcus aureus subsp. aureus M013 (CA-MRSA)	"Prokaryotes;Bacteria;Firmicutes - Bacilli;Staphylococcus"
3850	suv	Staphylococcus aureus subsp. aureus VC40	"Prokaryotes;Bacteria;Firmicutes - Bacilli;Staphylococcus"
3851	sue	Staphylococcus aureus subsp. aureus ED133	"Prokaryotes;Bacteria;Firmicutes - Bacilli;Staphylococcus"
3852	suj	Staphylococcus aureus subsp. aureus JKD6159 (CA-MRSA)	"Prokaryotes;Bacteria;Firmicutes - Bacilli;Staphylococcus"
3853	suk	Staphylococcus aureus subsp. aureus JKD6008 (MRSA/VISA)	"Prokaryotes;Bacteria;Firmicutes - Bacilli;Staphylococcus"
3854	suc	Staphylococcus aureus subsp. aureus ECT-R 2 (MSSA)	"Prokaryotes;Bacteria;Firmicutes - Bacilli;Staphylococcus"
3855	sut	Staphylococcus aureus subsp. aureus T0131 (MRSA)	"Prokaryotes;Bacteria;Firmicutes - Bacilli;Staphylococcus"
3856	suq	Staphylococcus aureus subsp. aureus TCH60	"Prokaryotes;Bacteria;Firmicutes - Bacilli;Staphylococcus"
3857	suz	Staphylococcus aureus subsp. aureus 11819-97 (CA-MRSA)	"Prokaryotes;Bacteria;Firmicutes - Bacilli;Staphylococcus"
3858	sud	Staphylococcus aureus subsp. aureus 71193 (MSSA)	"Prokaryotes;Bacteria;Firmicutes - Bacilli;Staphylococcus"
3859	sux	Staphylococcus aureus subsp. aureus HO 5096 0412 (MRSA)	"Prokaryotes;Bacteria;Firmicutes - Bacilli;Staphylococcus"
3860	suw	Staphylococcus aureus subsp. aureus TW20 (MRSA)	"Prokaryotes;Bacteria;Firmicutes - Bacilli;Staphylococcus"
3861	sug	Staphylococcus aureus subsp. aureus ST398 (MRSA)	"Prokaryotes;Bacteria;Firmicutes - Bacilli;Staphylococcus"
3862	suf	Staphylococcus aureus subsp. aureus LGA251 (MRSA)	"Prokaryotes;Bacteria;Firmicutes - Bacilli;Staphylococcus"
3863	saua	Staphylococcus aureus subsp. aureus 55/2053	"Prokaryotes;Bacteria;Firmicutes - Bacilli;Staphylococcus"
3864	saue	Staphylococcus aureus subsp. aureus 6850 (MSSA)	"Prokaryotes;Bacteria;Firmicutes - Bacilli;Staphylococcus"
3865	saun	Staphylococcus aureus subsp. aureus CN1 (CA-MRSA)	"Prokaryotes;Bacteria;Firmicutes - Bacilli;Staphylococcus"
3866	saus	Staphylococcus aureus subsp. aureus SA40 (CA-MRSA)	"Prokaryotes;Bacteria;Firmicutes - Bacilli;Staphylococcus"
3867	sauu	Staphylococcus aureus subsp. aureus SA957 (CA-MRSA)	"Prokaryotes;Bacteria;Firmicutes - Bacilli;Staphylococcus"
3868	saug	Staphylococcus aureus subsp. aureus SA268 (CA-MRSA)	"Prokaryotes;Bacteria;Firmicutes - Bacilli;Staphylococcus"
3869	sauz	Staphylococcus aureus subsp. aureus Z172 (MRSA/VISA)	"Prokaryotes;Bacteria;Firmicutes - Bacilli;Staphylococcus"
3870	saut	Staphylococcus aureus subsp. aureus ST228/10388 (MRSA)	"Prokaryotes;Bacteria;Firmicutes - Bacilli;Staphylococcus"
3871	sauj	Staphylococcus aureus subsp. aureus ST228/10497 (MRSA)	"Prokaryotes;Bacteria;Firmicutes - Bacilli;Staphylococcus"
3872	sauk	Staphylococcus aureus subsp. aureus ST228/15532 (MRSA)	"Prokaryotes;Bacteria;Firmicutes - Bacilli;Staphylococcus"
3873	sauq	Staphylococcus aureus subsp. aureus ST228/16035 (MRSA)	"Prokaryotes;Bacteria;Firmicutes - Bacilli;Staphylococcus"
3874	sauv	Staphylococcus aureus subsp. aureus ST228/18412 (MRSA)	"Prokaryotes;Bacteria;Firmicutes - Bacilli;Staphylococcus"
3875	sauw	Staphylococcus aureus subsp. aureus ST228/16125 (MRSA)	"Prokaryotes;Bacteria;Firmicutes - Bacilli;Staphylococcus"
3876	saux	Staphylococcus aureus subsp. aureus ST228/18341 (MRSA)	"Prokaryotes;Bacteria;Firmicutes - Bacilli;Staphylococcus"
3877	sauy	Staphylococcus aureus subsp. aureus ST228/18583 (MRSA)	"Prokaryotes;Bacteria;Firmicutes - Bacilli;Staphylococcus"
3878	sauf	Staphylococcus aureus subsp. aureus FDAARGOS_5	"Prokaryotes;Bacteria;Firmicutes - Bacilli;Staphylococcus"
3879	sab	Staphylococcus aureus RF122	"Prokaryotes;Bacteria;Firmicutes - Bacilli;Staphylococcus"
3880	suy	Staphylococcus aureus 04-02981 (MRSA)	"Prokaryotes;Bacteria;Firmicutes - Bacilli;Staphylococcus"
3881	saub	Staphylococcus aureus 08BA02176 (LA-MRSA)	"Prokaryotes;Bacteria;Firmicutes - Bacilli;Staphylococcus"

3882	saum	Staphylococcus aureus M1 (MRSA)	"Prokaryotes;Bacteria;Firmicutes - Bacilli;Staphylococcus"
3883	sauc	Staphylococcus aureus CA-347 (MRSA)	"Prokaryotes;Bacteria;Firmicutes - Bacilli;Staphylococcus"
3884	saur	Staphylococcus aureus Bmb9393 (MRSA)	"Prokaryotes;Bacteria;Firmicutes - Bacilli;Staphylococcus"
3885	saui	Staphylococcus aureus USA300-ISMMS1 (MRSA)	"Prokaryotes;Bacteria;Firmicutes - Bacilli;Staphylococcus"
3886	saud	Staphylococcus aureus 502A	"Prokaryotes;Bacteria;Firmicutes - Bacilli;Staphylococcus"
3887	sams	Staphylococcus aureus MS4	"Prokaryotes;Bacteria;Firmicutes - Bacilli;Staphylococcus"
3888	suh	Staphylococcus argenteus	"Prokaryotes;Bacteria;Firmicutes - Bacilli;Staphylococcus"
3889	ser	Staphylococcus epidermidis RP62A (MRSE)	"Prokaryotes;Bacteria;Firmicutes - Bacilli;Staphylococcus"
3890	sep	Staphylococcus epidermidis ATCC 12228	"Prokaryotes;Bacteria;Firmicutes - Bacilli;Staphylococcus"
3891	sepp	Staphylococcus epidermidis PM221	"Prokaryotes;Bacteria;Firmicutes - Bacilli;Staphylococcus"
3892	seps	Staphylococcus epidermidis SEI	"Prokaryotes;Bacteria;Firmicutes - Bacilli;Staphylococcus"
3893	sha	Staphylococcus haemolyticus JCSC1435	"Prokaryotes;Bacteria;Firmicutes - Bacilli;Staphylococcus"
3894	shh	Staphylococcus haemolyticus Sh29/312/L2	"Prokaryotes;Bacteria;Firmicutes - Bacilli;Staphylococcus"
3895	ssp	Staphylococcus saprophyticus	"Prokaryotes;Bacteria;Firmicutes - Bacilli;Staphylococcus"
3896	sca	Staphylococcus carnosus subsp. carnosus	"Prokaryotes;Bacteria;Firmicutes - Bacilli;Staphylococcus"
3897	slg	Staphylococcus lugdunensis HKU09-01	"Prokaryotes;Bacteria;Firmicutes - Bacilli;Staphylococcus"
3898	sln	Staphylococcus lugdunensis N920143	"Prokaryotes;Bacteria;Firmicutes - Bacilli;Staphylococcus"
3899	ssd	Staphylococcus pseudintermedius HKU10-03	"Prokaryotes;Bacteria;Firmicutes - Bacilli;Staphylococcus"
3900	sdt	Staphylococcus pseudintermedius ED99	"Prokaryotes;Bacteria;Firmicutes - Bacilli;Staphylococcus"
3901	sdp	Staphylococcus delphini	"Prokaryotes;Bacteria;Firmicutes - Bacilli;Staphylococcus"
3902	swa	Staphylococcus warneri	"Prokaryotes;Bacteria;Firmicutes - Bacilli;Staphylococcus"
3903	spas	Staphylococcus pasteuri	"Prokaryotes;Bacteria;Firmicutes - Bacilli;Staphylococcus"
3904	sxy	Staphylococcus xylosus HKUOPL8	"Prokaryotes;Bacteria;Firmicutes - Bacilli;Staphylococcus"
3905	sxl	Staphylococcus xylosus SMQ-121	"Prokaryotes;Bacteria;Firmicutes - Bacilli;Staphylococcus"
3906	sxo	Staphylococcus xylosus C2a	"Prokaryotes;Bacteria;Firmicutes - Bacilli;Staphylococcus"
3907	shu	Staphylococcus hyicus	"Prokaryotes;Bacteria;Firmicutes - Bacilli;Staphylococcus"
3908	scap	Staphylococcus capitis subsp. capitis	"Prokaryotes;Bacteria;Firmicutes - Bacilli;Staphylococcus"
3909	ssch	Staphylococcus schleiferi 1360-13	"Prokaryotes;Bacteria;Firmicutes - Bacilli;Staphylococcus"
3910	sscz	Staphylococcus schleiferi 2317-03	"Prokaryotes;Bacteria;Firmicutes - Bacilli;Staphylococcus"
3911	sagq	Staphylococcus agnetis	"Prokaryotes;Bacteria;Firmicutes - Bacilli;Staphylococcus"
3912	seqo	Staphylococcus equorum	"Prokaryotes;Bacteria;Firmicutes - Bacilli;Staphylococcus"
3913	ssif	Staphylococcus simulans	"Prokaryotes;Bacteria;Firmicutes - Bacilli;Staphylococcus"
3914	scv	Staphylococcus condimenti	"Prokaryotes;Bacteria;Firmicutes - Bacilli;Staphylococcus"
3915	spet	Staphylococcus pettenkoferi	"Prokaryotes;Bacteria;Firmicutes - Bacilli;Staphylococcus"
3916	slz	Staphylococcus lutrae	"Prokaryotes;Bacteria;Firmicutes - Bacilli;Staphylococcus"
3917	scoh	Staphylococcus cohnii	"Prokaryotes;Bacteria;Firmicutes - Bacilli;Staphylococcus"
3918	snl	Staphylococcus nepalensis	"Prokaryotes;Bacteria;Firmicutes - Bacilli;Staphylococcus"
3919	skl	Staphylococcus kloosii	"Prokaryotes;Bacteria;Firmicutes - Bacilli;Staphylococcus"
3920	sfq	Staphylococcus felis	"Prokaryotes;Bacteria;Firmicutes - Bacilli;Staphylococcus"
3921	shom	Staphylococcus hominis	"Prokaryotes;Bacteria;Firmicutes - Bacilli;Staphylococcus"
3922	smus	Staphylococcus muscae	"Prokaryotes;Bacteria;Firmicutes - Bacilli;Staphylococcus"

3923	scar	Staphylococcus caprae	"Prokaryotes;Bacteria;Firmicutes - Bacilli;Staphylococcus"
3924	schr	Staphylococcus chromogenes	"Prokaryotes;Bacteria;Firmicutes - Bacilli;Staphylococcus"
3925	sarl	Staphylococcus arlettae	"Prokaryotes;Bacteria;Firmicutes - Bacilli;Staphylococcus"
3926	spic	Staphylococcus piscifermentans	"Prokaryotes;Bacteria;Firmicutes - Bacilli;Staphylococcus"
3927	ssh	Staphylococcus schweitzeri	"Prokaryotes;Bacteria;Firmicutes - Bacilli;Staphylococcus"
3928	ssim	Staphylococcus simiae	"Prokaryotes;Bacteria;Firmicutes - Bacilli;Staphylococcus"
3929	sscu	Mammaliicoccus sciuri	"Prokaryotes;Bacteria;Firmicutes - Bacilli;Mammaliicoccus"
3930	sff	Mammaliicoccus fleurettii	"Prokaryotes;Bacteria;Firmicutes - Bacilli;Mammaliicoccus"
3931	sste	Mammaliicoccus stepanovicii	"Prokaryotes;Bacteria;Firmicutes - Bacilli;Mammaliicoccus"
3932	mlen	Mammaliicoccus lentus	"Prokaryotes;Bacteria;Firmicutes - Bacilli;Mammaliicoccus"
3933	mcl	Macrococcus caseolyticus	"Prokaryotes;Bacteria;Firmicutes - Bacilli;Macrococcus"
3934	mcak	Macrococcus canis	"Prokaryotes;Bacteria;Firmicutes - Bacilli;Macrococcus"
3935	macr	Macrococcus sp. IME1552	"Prokaryotes;Bacteria;Firmicutes - Bacilli;Macrococcus"
3936	shv	Salinicoccus halodurans	"Prokaryotes;Bacteria;Firmicutes - Bacilli;Salinicoccus"
3937	sbac	Abyssicoccus albus	"Prokaryotes;Bacteria;Firmicutes - Bacilli;Abyssicoccus"
3938	jea	Jeotgalicoccus sp. ATCC 8456	"Prokaryotes;Bacteria;Firmicutes - Bacilli;Jeotgalicoccus"
3939	lmo	Listeria monocytogenes EGD-e (serotype 1/2a)	"Prokaryotes;Bacteria;Firmicutes - Bacilli;Listeria"
3940	lmn	Listeria monocytogenes 08-5578 (serotype 1/2a)	"Prokaryotes;Bacteria;Firmicutes - Bacilli;Listeria"
3941	lmy	Listeria monocytogenes 08-5923 (serotype 1/2a)	"Prokaryotes;Bacteria;Firmicutes - Bacilli;Listeria"
3942	lmt	Listeria monocytogenes 10403S (serotype 1/2a)	"Prokaryotes;Bacteria;Firmicutes - Bacilli;Listeria"
3943	lmoc	Listeria monocytogenes SLCC5850 (serotype 1/2a)	"Prokaryotes;Bacteria;Firmicutes - Bacilli;Listeria"
3944	lmoe	Listeria monocytogenes La111 (serotype 1/2a)	"Prokaryotes;Bacteria;Firmicutes - Bacilli;Listeria"
3945	lmob	Listeria monocytogenes N53-1 (serotype 1/2a)	"Prokaryotes;Bacteria;Firmicutes - Bacilli;Listeria"
3946	lmod	Listeria monocytogenes EGD (serotype 1/2a)	"Prokaryotes;Bacteria;Firmicutes - Bacilli;Listeria"
3947	lmow	Listeria monocytogenes WSLC1001 (serotype 1/2a)	"Prokaryotes;Bacteria;Firmicutes - Bacilli;Listeria"
3948	lmoq	Listeria monocytogenes 6179 (serotype 1/2a)	"Prokaryotes;Bacteria;Firmicutes - Bacilli;Listeria"
3949	lmr	Listeria monocytogenes R479a (serotype 1/2a)	"Prokaryotes;Bacteria;Firmicutes - Bacilli;Listeria"
3950	lmom	Listeria monocytogenes Lm60 (serotype 1/2a)	"Prokaryotes;Bacteria;Firmicutes - Bacilli;Listeria"
3951	lmf	Listeria monocytogenes F2365 (serotype 4b)	"Prokaryotes;Bacteria;Firmicutes - Bacilli;Listeria"
3952	lmc	Listeria monocytogenes CLIP 80459 (serotype 4b)	"Prokaryotes;Bacteria;Firmicutes - Bacilli;Listeria"
3953	lmog	Listeria monocytogenes serotype 4b LL195	"Prokaryotes;Bacteria;Firmicutes - Bacilli;Listeria"
3954	lmp	Listeria monocytogenes 07PF0776 (serotype 4b)	"Prokaryotes;Bacteria;Firmicutes - Bacilli;Listeria"
3955	lmol	Listeria monocytogenes L312 (serotype 4b)	"Prokaryotes;Bacteria;Firmicutes - Bacilli;Listeria"
3956	lmoj	Listeria monocytogenes J1816 (serotype 4b)	"Prokaryotes;Bacteria;Firmicutes - Bacilli;Listeria"
3957	lmoz	Listeria monocytogenes J1-220 (serotype 4b)	"Prokaryotes;Bacteria;Firmicutes - Bacilli;Listeria"
3958	lmox	Listeria monocytogenes WSLC1042 (serotype 4b)	"Prokaryotes;Bacteria;Firmicutes - Bacilli;Listeria"
3959	lmh	Listeria monocytogenes HCC23 (serotype 4a)	"Prokaryotes;Bacteria;Firmicutes - Bacilli;Listeria"
3960	lmq	Listeria monocytogenes M7 (serotype 4a)	"Prokaryotes;Bacteria;Firmicutes - Bacilli;Listeria"
3961	lml	Listeria monocytogenes L99 (serotype 4a)	"Prokaryotes;Bacteria;Firmicutes - Bacilli;Listeria"
3962	lmg	Listeria monocytogenes FSL R2-561	"Prokaryotes;Bacteria;Firmicutes - Bacilli;Listeria"
3963	lms	Listeria monocytogenes Finland 1998	"Prokaryotes;Bacteria;Firmicutes - Bacilli;Listeria"

3964	lmj	Listeria monocytogenes J0161	"Prokaryotes;Bacteria;Firmicutes - Bacilli;Listeria"
3965	lmw	Listeria monocytogenes SLCC2755 (serotype 1/2b)	"Prokaryotes;Bacteria;Firmicutes - Bacilli;Listeria"
3966	lmx	Listeria monocytogenes SLCC2372 (serotype 1/2c)	"Prokaryotes;Bacteria;Firmicutes - Bacilli;Listeria"
3967	lmz	Listeria monocytogenes serotype 7 SLCC2482	"Prokaryotes;Bacteria;Firmicutes - Bacilli;Listeria"
3968	lmon	Listeria monocytogenes SLCC2376 (serotype 4c)	"Prokaryotes;Bacteria;Firmicutes - Bacilli;Listeria"
3969	lmos	Listeria monocytogenes SLCC7179 (serotype 3a)	"Prokaryotes;Bacteria;Firmicutes - Bacilli;Listeria"
3970	lmoo	Listeria monocytogenes SLCC2378 (serotype 4e)	"Prokaryotes;Bacteria;Firmicutes - Bacilli;Listeria"
3971	lmoy	Listeria monocytogenes SLCC2479 (serotype 3c)	"Prokaryotes;Bacteria;Firmicutes - Bacilli;Listeria"
3972	lmot	Listeria monocytogenes SLCC2540 (serotype 3b)	"Prokaryotes;Bacteria;Firmicutes - Bacilli;Listeria"
3973	lmoa	Listeria monocytogenes ATCC 19117 (serotype 4d)	"Prokaryotes;Bacteria;Firmicutes - Bacilli;Listeria"
3974	lmok	Listeria monocytogenes NE dc2014	"Prokaryotes;Bacteria;Firmicutes - Bacilli;Listeria"
3975	lmv	Listeria monocytogenes CFSAN006122	"Prokaryotes;Bacteria;Firmicutes - Bacilli;Listeria"
3976	lin	Listeria innocua (serotype 6a)	"Prokaryotes;Bacteria;Firmicutes - Bacilli;Listeria"
3977	lwe	Listeria welshimeri	"Prokaryotes;Bacteria;Firmicutes - Bacilli;Listeria"
3978	lsg	Listeria seeligeri	"Prokaryotes;Bacteria;Firmicutes - Bacilli;Listeria"
3979	liv	Listeria ivanovii subsp. ivanovii PAM 55	"Prokaryotes;Bacteria;Firmicutes - Bacilli;Listeria"
3980	lii	Listeria ivanovii subsp. ivanovii WSLC 3010	"Prokaryotes;Bacteria;Firmicutes - Bacilli;Listeria"
3981	liw	Listeria ivanovii WSLC3009	"Prokaryotes;Bacteria;Firmicutes - Bacilli;Listeria"
3982	lia	Listeria ivanovii subsp. londoniensis WSLC 30167	"Prokaryotes;Bacteria;Firmicutes - Bacilli;Listeria"
3983	lio	Listeria ivanovii subsp. londoniensis WSLC 30151	"Prokaryotes;Bacteria;Firmicutes - Bacilli;Listeria"
3984	lwi	Listeria weihenstephanensis	"Prokaryotes;Bacteria;Firmicutes - Bacilli;Listeria"
3985	lgz	Listeria grayi	"Prokaryotes;Bacteria;Firmicutes - Bacilli;Listeria"
3986	bths	Brochothrix thermosphacta	"Prokaryotes;Bacteria;Firmicutes - Bacilli;Brochothrix"
3987	esi	Exiguobacterium sibiricum	"Prokaryotes;Bacteria;Firmicutes - Bacilli;Exiguobacterium"
3988	eat	Exiguobacterium sp. AT1b	"Prokaryotes;Bacteria;Firmicutes - Bacilli;Exiguobacterium"
3989	ean	Exiguobacterium antarcticum	"Prokaryotes;Bacteria;Firmicutes - Bacilli;Exiguobacterium"
3990	exm	Exiguobacterium sp. MH3	"Prokaryotes;Bacteria;Firmicutes - Bacilli;Exiguobacterium"
3991	exu	Exiguobacterium sp. U13-1	"Prokaryotes;Bacteria;Firmicutes - Bacilli;Exiguobacterium"
3992	got	Gemella sp. oral taxon 928	"Prokaryotes;Bacteria;Firmicutes - Bacilli;Gemella"
3993	gmo	Gemella morbillorum	"Prokaryotes;Bacteria;Firmicutes - Bacilli;Gemella"
3994	geq	Gemella sp. ND 6198	"Prokaryotes;Bacteria;Firmicutes - Bacilli;Gemella"
3995	gsa	Gemella sanguinis	"Prokaryotes;Bacteria;Firmicutes - Bacilli;Gemella"
3996	gha	Gemella haemolysans	"Prokaryotes;Bacteria;Firmicutes - Bacilli;Gemella"
3997	bbe	Brevibacillus brevis	"Prokaryotes;Bacteria;Firmicutes - Bacilli;Brevibacillus"
3998	blr	Brevibacillus laterosporus	"Prokaryotes;Bacteria;Firmicutes - Bacilli;Brevibacillus"
3999	bfm	Brevibacillus formosus	"Prokaryotes;Bacteria;Firmicutes - Bacilli;Brevibacillus"
4000	bagr	Brevibacillus agri	"Prokaryotes;Bacteria;Firmicutes - Bacilli;Brevibacillus"
4001	brw	Brevibacillus sp. 7WMA2	"Prokaryotes;Bacteria;Firmicutes - Bacilli;Brevibacillus"
4002	pjd	Paenibacillus sp. JDR-2	"Prokaryotes;Bacteria;Firmicutes - Bacilli;Paenibacillus"
4003	gym	Paenibacillus sp. Y412MC10	"Prokaryotes;Bacteria;Firmicutes - Bacilli;Paenibacillus"
4004	ppy	Paenibacillus polymyxa E681	"Prokaryotes;Bacteria;Firmicutes - Bacilli;Paenibacillus"

4005	ppm	Paenibacillus polymyxa SC2	"Prokaryotes;Bacteria;Firmicutes - Bacilli;Paenibacillus"
4006	ppo	Paenibacillus polymyxa M1	"Prokaryotes;Bacteria;Firmicutes - Bacilli;Paenibacillus"
4007	ppol	Paenibacillus polymyxa CR1	"Prokaryotes;Bacteria;Firmicutes - Bacilli;Paenibacillus"
4008	ppq	Paenibacillus polymyxa SQR-21	"Prokaryotes;Bacteria;Firmicutes - Bacilli;Paenibacillus"
4009	ppoy	Paenibacillus polymyxa Sb3-1	"Prokaryotes;Bacteria;Firmicutes - Bacilli;Paenibacillus"
4010	pms	Paenibacillus mucilaginosus KNP414	"Prokaryotes;Bacteria;Firmicutes - Bacilli;Paenibacillus"
4011	pmq	Paenibacillus mucilaginosus 3016	"Prokaryotes;Bacteria;Firmicutes - Bacilli;Paenibacillus"
4012	pmw	Paenibacillus mucilaginosus K02	"Prokaryotes;Bacteria;Firmicutes - Bacilli;Paenibacillus"
4013	pta	Paenibacillus terrae	"Prokaryotes;Bacteria;Firmicutes - Bacilli;Paenibacillus"
4014	plv	Paenibacillus larvae	"Prokaryotes;Bacteria;Firmicutes - Bacilli;Paenibacillus"
4015	psab	Paenibacillus sabinae	"Prokaryotes;Bacteria;Firmicutes - Bacilli;Paenibacillus"
4016	pdu	Paenibacillus durus	"Prokaryotes;Bacteria;Firmicutes - Bacilli;Paenibacillus"
4017	pbd	Paenibacillus borealis	"Prokaryotes;Bacteria;Firmicutes - Bacilli;Paenibacillus"
4018	pgm	Paenibacillus graminis	"Prokaryotes;Bacteria;Firmicutes - Bacilli;Paenibacillus"
4019	pod	Paenibacillus odorifer	"Prokaryotes;Bacteria;Firmicutes - Bacilli;Paenibacillus"
4020	paen	Paenibacillus sp. FSL P4-0081	"Prokaryotes;Bacteria;Firmicutes - Bacilli;Paenibacillus"
4021	paef	Paenibacillus sp. FSL R5-0345	"Prokaryotes;Bacteria;Firmicutes - Bacilli;Paenibacillus"
4022	paeq	Paenibacillus sp. FSL R5-0912	"Prokaryotes;Bacteria;Firmicutes - Bacilli;Paenibacillus"
4023	pste	Paenibacillus stellifer	"Prokaryotes;Bacteria;Firmicutes - Bacilli;Paenibacillus"
4024	paea	Paenibacillus sp. FSL R7-0273	"Prokaryotes;Bacteria;Firmicutes - Bacilli;Paenibacillus"
4025	paee	Paenibacillus sp. FSL R7-0331	"Prokaryotes;Bacteria;Firmicutes - Bacilli;Paenibacillus"
4026	paeh	Paenibacillus sp. FSL H7-0357	"Prokaryotes;Bacteria;Firmicutes - Bacilli;Paenibacillus"
4027	paej	Paenibacillus sp. FSL H7-0737	"Prokaryotes;Bacteria;Firmicutes - Bacilli;Paenibacillus"
4028	pbj	Paenibacillus beijingensis	"Prokaryotes;Bacteria;Firmicutes - Bacilli;Paenibacillus"
4029	pih	Paenibacillus sp. IHBB 10380	"Prokaryotes;Bacteria;Firmicutes - Bacilli;Paenibacillus"
4030	pri	Paenibacillus riograndensis	"Prokaryotes;Bacteria;Firmicutes - Bacilli;Paenibacillus"
4031	ppeo	Paenibacillus peoriae	"Prokaryotes;Bacteria;Firmicutes - Bacilli;Paenibacillus"
4032	pnp	Paenibacillus naphthalenovorans	"Prokaryotes;Bacteria;Firmicutes - Bacilli;Paenibacillus"
4033	pow	Paenibacillus sp. 32O-W	"Prokaryotes;Bacteria;Firmicutes - Bacilli;Paenibacillus"
4034	pbv	Paenibacillus bovis	"Prokaryotes;Bacteria;Firmicutes - Bacilli;Paenibacillus"
4035	pxl	Paenibacillus xylanexedens	"Prokaryotes;Bacteria;Firmicutes - Bacilli;Paenibacillus"
4036	pyg	Paenibacillus yonginensis	"Prokaryotes;Bacteria;Firmicutes - Bacilli;Paenibacillus"
4037	pswu	Paenibacillus swuensis	"Prokaryotes;Bacteria;Firmicutes - Bacilli;Paenibacillus"
4038	pdh	Paenibacillus donghaensis	"Prokaryotes;Bacteria;Firmicutes - Bacilli;Paenibacillus"
4039	pib	Paenibacillus ihbetae	"Prokaryotes;Bacteria;Firmicutes - Bacilli;Paenibacillus"
4040	pcx	Paenibacillus crassostreae	"Prokaryotes;Bacteria;Firmicutes - Bacilli;Paenibacillus"
4041	pkb	Paenibacillus kribbensis	"Prokaryotes;Bacteria;Firmicutes - Bacilli;Paenibacillus"
4042	paih	Paenibacillus sp. IHB B 3084	"Prokaryotes;Bacteria;Firmicutes - Bacilli;Paenibacillus"
4043	pvo	Paenibacillus vortex	"Prokaryotes;Bacteria;Firmicutes - Bacilli;Paenibacillus"
4044	plw	Paenibacillus lautus	"Prokaryotes;Bacteria;Firmicutes - Bacilli;Paenibacillus"
4045	plen	Paenibacillus lentus	"Prokaryotes;Bacteria;Firmicutes - Bacilli;Paenibacillus"

4046	ppsc	Paenibacillus psychroresistens	"Prokaryotes;Bacteria;Firmicutes - Bacilli;Paenibacillus"
4047	plut	Paenibacillus lutimineralis	"Prokaryotes;Bacteria;Firmicutes - Bacilli;Paenibacillus"
4048	palb	Paenibacillus albus	"Prokaryotes;Bacteria;Firmicutes - Bacilli;Paenibacillus"
4049	pchi	Paenibacillus chitinolyticus	"Prokaryotes;Bacteria;Firmicutes - Bacilli;Paenibacillus"
4050	pbk	Paenibacillus baekrokdamisoli	"Prokaryotes;Bacteria;Firmicutes - Bacilli;Paenibacillus"
4051	pprt	Paenibacillus protaetiae	"Prokaryotes;Bacteria;Firmicutes - Bacilli;Paenibacillus"
4052	pbac	Paenibacillus barcinonensis	"Prokaryotes;Bacteria;Firmicutes - Bacilli;Paenibacillus"
4053	prz	Paenibacillus rhizovicinus	"Prokaryotes;Bacteria;Firmicutes - Bacilli;Paenibacillus"
4054	plyc	Paenibacillus lycopersici	"Prokaryotes;Bacteria;Firmicutes - Bacilli;Paenibacillus"
4055	tco	Thermobacillus composti	"Prokaryotes;Bacteria;Firmicutes - Bacilli;Thermobacillus"
4056	anx	Aneurinibacillus sp. XH2	"Prokaryotes;Bacteria;Firmicutes - Bacilli;Aneurinibacillus"
4057	asoc	Aneurinibacillus soli	"Prokaryotes;Bacteria;Firmicutes - Bacilli;Aneurinibacillus"
4058	coh	Cohnella candidum	"Prokaryotes;Bacteria;Firmicutes - Bacilli;Cohnella"
4059	cohn	Cohnella abietis	"Prokaryotes;Bacteria;Firmicutes - Bacilli;Cohnella"
4060	saca	Saccharibacillus brassicae	"Prokaryotes;Bacteria;Firmicutes - Bacilli;Saccharibacillus"
4061	aac	Alicyclobacillus acidocaldarius subsp. acidocaldarius DSM 446	"Prokaryotes;Bacteria;Firmicutes - Bacilli;Alicyclobacillus"
4062	aad	Alicyclobacillus acidocaldarius subsp. acidocaldarius Tc-4-1	"Prokaryotes;Bacteria;Firmicutes - Bacilli;Alicyclobacillus"
4063	bts	Kyrpidia tusciae	"Prokaryotes;Bacteria;Firmicutes - Bacilli;Kyrpidia"
4064	kyr	Kyrpidia spormannii	"Prokaryotes;Bacteria;Firmicutes - Bacilli;Kyrpidia"
4065	tum	Tumebacillus avium	"Prokaryotes;Bacteria;Firmicutes - Bacilli;Tumebacillus"
4066	tab	Tumebacillus algifaecis	"Prokaryotes;Bacteria;Firmicutes - Bacilli;Tumebacillus"
4067	eff	Effusibacillus sp. skT53	"Prokaryotes;Bacteria;Firmicutes - Bacilli;Effusibacillus"
4068	siv	Solibacillus silvestris StLB046	"Prokaryotes;Bacteria;Firmicutes - Bacilli;Solibacillus"
4069	ssil	Solibacillus silvestris DSM 12223	"Prokaryotes;Bacteria;Firmicutes - Bacilli;Solibacillus"
4070	sob	Solibacillus sp. R5-41	"Prokaryotes;Bacteria;Firmicutes - Bacilli;Solibacillus"
4071	pln	Planococcus sp. PAMC 21323	"Prokaryotes;Bacteria;Firmicutes - Bacilli;Planococcus"
4072	pku	Planococcus kocurii	"Prokaryotes;Bacteria;Firmicutes - Bacilli;Planococcus"
4073	prt	Planococcus rifietoensis	"Prokaryotes;Bacteria;Firmicutes - Bacilli;Planococcus"
4074	pll	Planococcus versutus	"Prokaryotes;Bacteria;Firmicutes - Bacilli;Planococcus"
4075	pana	Planococcus antarcticus	"Prokaryotes;Bacteria;Firmicutes - Bacilli;Planococcus"
4076	pdg	Planococcus donghaensis	"Prokaryotes;Bacteria;Firmicutes - Bacilli;Planococcus"
4077	phc	Planococcus halocryophilus	"Prokaryotes;Bacteria;Firmicutes - Bacilli;Planococcus"
4078	pmar	Planococcus sp. Y42	"Prokaryotes;Bacteria;Firmicutes - Bacilli;Planococcus"
4079	ppla	Planococcus plakortidis	"Prokaryotes;Bacteria;Firmicutes - Bacilli;Planococcus"
4080	pfae	Planococcus faecalis	"Prokaryotes;Bacteria;Firmicutes - Bacilli;Planococcus"
4081	plx	Planococcus sp. MB-3u-03	"Prokaryotes;Bacteria;Firmicutes - Bacilli;Planococcus"
4082	pmat	Planococcus maritimus	"Prokaryotes;Bacteria;Firmicutes - Bacilli;Planococcus"
4083	pdec	Planococcus dechangensis	"Prokaryotes;Bacteria;Firmicutes - Bacilli;Planococcus"
4084	jeo	Jeotgalibacillus malaysiensis	"Prokaryotes;Bacteria;Firmicutes - Bacilli;Jeotgalibacillus"
4085	kur	Kurthia sp. 11kri321	"Prokaryotes;Bacteria;Firmicutes - Bacilli;Kurthia"
4086	kzo	Kurthia zopfii	"Prokaryotes;Bacteria;Firmicutes - Bacilli;Kurthia"

4087	spsy	Sporosarcina psychrophila	"Prokaryotes;Bacteria;Firmicutes - Bacilli;Sporosarcina"
4088	spor	Sporosarcina sp. P33	"Prokaryotes;Bacteria;Firmicutes - Bacilli;Sporosarcina"
4089	spop	Sporosarcina sp. P37	"Prokaryotes;Bacteria;Firmicutes - Bacilli;Sporosarcina"
4090	sure	Sporosarcina ureae	"Prokaryotes;Bacteria;Firmicutes - Bacilli;Sporosarcina"
4091	spos	Sporosarcina sp. PTS2304	"Prokaryotes;Bacteria;Firmicutes - Bacilli;Sporosarcina"
4092	spae	Sporosarcina pasteurii	"Prokaryotes;Bacteria;Firmicutes - Bacilli;Sporosarcina"
4093	rst	Rummeliibacillus stabekisii	"Prokaryotes;Bacteria;Firmicutes - Bacilli;Rummeliibacillus"
4094	paek	Paenisporosarcina sp. K2R23-3	"Prokaryotes;Bacteria;Firmicutes - Bacilli;Paenisporosarcina"
4095	panc	Paenisporosarcina antarctica	"Prokaryotes;Bacteria;Firmicutes - Bacilli;Paenisporosarcina"
4096	pgq	Planomicrobium glaciei	"Prokaryotes;Bacteria;Firmicutes - Bacilli;Planomicrobium"
4097	play	Planomicrobium sp. Y50	"Prokaryotes;Bacteria;Firmicutes - Bacilli;Planomicrobium"
4098	vij	Viridibacillus sp. JNUCC-6	"Prokaryotes;Bacteria;Firmicutes - Bacilli;Viridibacillus"
4099	ntr	Novibacillus thermophilus	"Prokaryotes;Bacteria;Firmicutes - Bacilli;Novibacillus"
4100	lfb	Laceyella sacchari	"Prokaryotes;Bacteria;Firmicutes - Bacilli;Laceyella"
4101	tvu	Thermoactinomyces vulgaris	"Prokaryotes;Bacteria;Firmicutes - Bacilli;Thermoactinomyces"
4102	kpul	Kroppenstedtia pulmonis	"Prokaryotes;Bacteria;Firmicutes - Bacilli;Kroppenstedtia"
4103	keb	Kroppenstedtia eburnea	"Prokaryotes;Bacteria;Firmicutes - Bacilli;Kroppenstedtia"
4104	lla	Lactococcus lactis subsp. lactis Il1403	"Prokaryotes;Bacteria;Firmicutes - Bacilli;Lactococcus"
4105	llk	Lactococcus lactis subsp. lactis KF147	"Prokaryotes;Bacteria;Firmicutes - Bacilli;Lactococcus"
4106	llt	Lactococcus lactis subsp. lactis CV56	"Prokaryotes;Bacteria;Firmicutes - Bacilli;Lactococcus"
4107	lls	Lactococcus lactis subsp. lactis IO-1	"Prokaryotes;Bacteria;Firmicutes - Bacilli;Lactococcus"
4108	lld	Lactococcus lactis subsp. lactis KLDS 4.0325	"Prokaryotes;Bacteria;Firmicutes - Bacilli;Lactococcus"
4109	llx	Lactococcus lactis subsp. lactis NCDO 2118	"Prokaryotes;Bacteria;Firmicutes - Bacilli;Lactococcus"
4110	llj	Lactococcus lactis AI06	"Prokaryotes;Bacteria;Firmicutes - Bacilli;Lactococcus"
4111	llm	Lactococcus lactis subsp. cremoris MG1363	"Prokaryotes;Bacteria;Firmicutes - Bacilli;Lactococcus"
4112	llc	Lactococcus lactis subsp. cremoris SK11	"Prokaryotes;Bacteria;Firmicutes - Bacilli;Lactococcus"
4113	llr	Lactococcus lactis subsp. cremoris A76	"Prokaryotes;Bacteria;Firmicutes - Bacilli;Lactococcus"
4114	lln	Lactococcus lactis subsp. cremoris NZ9000	"Prokaryotes;Bacteria;Firmicutes - Bacilli;Lactococcus"
4115	lli	Lactococcus lactis subsp. cremoris UC509.9	"Prokaryotes;Bacteria;Firmicutes - Bacilli;Lactococcus"
4116	llw	Lactococcus lactis subsp. cremoris KW2	"Prokaryotes;Bacteria;Firmicutes - Bacilli;Lactococcus"
4117	lgr	Lactococcus garvieae ATCC 49156	"Prokaryotes;Bacteria;Firmicutes - Bacilli;Lactococcus"
4118	lgv	Lactococcus garvieae Lg2	"Prokaryotes;Bacteria;Firmicutes - Bacilli;Lactococcus"
4119	lpk	Lactococcus piscium	"Prokaryotes;Bacteria;Firmicutes - Bacilli;Lactococcus"
4120	lrn	Lactococcus raffinolactis	"Prokaryotes;Bacteria;Firmicutes - Bacilli;Lactococcus"
4121	lact	Lactococcus allomyrinae	"Prokaryotes;Bacteria;Firmicutes - Bacilli;Lactococcus"
4122	lack	Lactococcus sp. KACC 19320	"Prokaryotes;Bacteria;Firmicutes - Bacilli;Lactococcus"
4123	spy	Streptococcus pyogenes M1 GAS (serotype M1)	"Prokaryotes;Bacteria;Firmicutes - Bacilli;Streptococcus"
4124	spz	Streptococcus pyogenes MGAS5005 (serotype M1)	"Prokaryotes;Bacteria;Firmicutes - Bacilli;Streptococcus"
4125	spym	Streptococcus pyogenes M1 476 (serotype M1)	"Prokaryotes;Bacteria;Firmicutes - Bacilli;Streptococcus"
4126	spya	Streptococcus pyogenes A20 (serotype M1)	"Prokaryotes;Bacteria;Firmicutes - Bacilli;Streptococcus"
4127	spm	Streptococcus pyogenes MGAS8232 (serotype M18)	"Prokaryotes;Bacteria;Firmicutes - Bacilli;Streptococcus"

4128	spg	Streptococcus pyogenes MGAS315 (serotype M3)	"Prokaryotes;Bacteria;Firmicutes - Bacilli;Streptococcus"
4129	sps	Streptococcus pyogenes SSI-1 (serotype M3)	"Prokaryotes;Bacteria;Firmicutes - Bacilli;Streptococcus"
4130	sph	Streptococcus pyogenes MGAS10270 (serotype M2)	"Prokaryotes;Bacteria;Firmicutes - Bacilli;Streptococcus"
4131	spi	Streptococcus pyogenes MGAS10750 (serotype M4)	"Prokaryotes;Bacteria;Firmicutes - Bacilli;Streptococcus"
4132	spj	Streptococcus pyogenes MGAS2096 (serotype M12)	"Prokaryotes;Bacteria;Firmicutes - Bacilli;Streptococcus"
4133	spk	Streptococcus pyogenes MGAS9429 (serotype M12)	"Prokaryotes;Bacteria;Firmicutes - Bacilli;Streptococcus"
4134	spf	Streptococcus pyogenes Manfredo (serotype M5)	"Prokaryotes;Bacteria;Firmicutes - Bacilli;Streptococcus"
4135	spa	Streptococcus pyogenes MGAS10394 (serotype M6)	"Prokaryotes;Bacteria;Firmicutes - Bacilli;Streptococcus"
4136	spb	Streptococcus pyogenes MGAS6180 (serotype M28)	"Prokaryotes;Bacteria;Firmicutes - Bacilli;Streptococcus"
4137	stg	Streptococcus pyogenes MGAS15252 (serotype M59)	"Prokaryotes;Bacteria;Firmicutes - Bacilli;Streptococcus"
4138	stx	Streptococcus pyogenes MGAS1882 (serotype M59)	"Prokaryotes;Bacteria;Firmicutes - Bacilli;Streptococcus"
4139	soz	Streptococcus pyogenes NZ131 (serotype M49)	"Prokaryotes;Bacteria;Firmicutes - Bacilli;Streptococcus"
4140	stz	Streptococcus pyogenes Alab49 (serotype M53)	"Prokaryotes;Bacteria;Firmicutes - Bacilli;Streptococcus"
4141	spyh	Streptococcus pyogenes HSC5 (serotype M14)	"Prokaryotes;Bacteria;Firmicutes - Bacilli;Streptococcus"
4142	spyo	Streptococcus pyogenes STAB901 (serotype M44)	"Prokaryotes;Bacteria;Firmicutes - Bacilli;Streptococcus"
4143	spn	Streptococcus pneumoniae TIGR4 (virulent serotype 4)	"Prokaryotes;Bacteria;Firmicutes - Bacilli;Streptococcus"
4144	spd	Streptococcus pneumoniae D39 (virulent serotype 2)	"Prokaryotes;Bacteria;Firmicutes - Bacilli;Streptococcus"
4145	spr	Streptococcus pneumoniae R6 (avirulent serotype 2)	"Prokaryotes;Bacteria;Firmicutes - Bacilli;Streptococcus"
4146	spw	Streptococcus pneumoniae CGSP14 (serotype 14)	"Prokaryotes;Bacteria;Firmicutes - Bacilli;Streptococcus"
4147	sjj	Streptococcus pneumoniae JJA (serotype 14)	"Prokaryotes;Bacteria;Firmicutes - Bacilli;Streptococcus"
4148	snv	Streptococcus pneumoniae INV200 (serotype 14)	"Prokaryotes;Bacteria;Firmicutes - Bacilli;Streptococcus"
4149	spx	Streptococcus pneumoniae G54 (serotype 19F)	"Prokaryotes;Bacteria;Firmicutes - Bacilli;Streptococcus"
4150	snt	Streptococcus pneumoniae Taiwan19F-14 (serotype 19F)	"Prokaryotes;Bacteria;Firmicutes - Bacilli;Streptococcus"
4151	snd	Streptococcus pneumoniae ST556 (serotype 19F)	"Prokaryotes;Bacteria;Firmicutes - Bacilli;Streptococcus"
4152	spnn	Streptococcus pneumoniae A026 (serotype 19F)	"Prokaryotes;Bacteria;Firmicutes - Bacilli;Streptococcus"
4153	sne	Streptococcus pneumoniae ATCC 700669 (serotype 23F ST81 lineage)	"Prokaryotes;Bacteria;Firmicutes - Bacilli;Streptococcus"
4154	spv	Streptococcus pneumoniae Hungary19A 6 (serotype 19A)	"Prokaryotes;Bacteria;Firmicutes - Bacilli;Streptococcus"
4155	snc	Streptococcus pneumoniae TCH8431/19A (serotype 19A)	"Prokaryotes;Bacteria;Firmicutes - Bacilli;Streptococcus"
4156	snm	Streptococcus pneumoniae 70585 (serotype 5)	"Prokaryotes;Bacteria;Firmicutes - Bacilli;Streptococcus"
4157	spp	Streptococcus pneumoniae P1031 (serotype 1)	"Prokaryotes;Bacteria;Firmicutes - Bacilli;Streptococcus"
4158	sni	Streptococcus pneumoniae INV104 (serotype 1)	"Prokaryotes;Bacteria;Firmicutes - Bacilli;Streptococcus"
4159	spng	Streptococcus pneumoniae gamPNI0373 (virulent serotype 1)	"Prokaryotes;Bacteria;Firmicutes - Bacilli;Streptococcus"
4160	snb	Streptococcus pneumoniae 670-6B (serotype 6B)	"Prokaryotes;Bacteria;Firmicutes - Bacilli;Streptococcus"
4161	snp	Streptococcus pneumoniae AP200 (serotype 11A)	"Prokaryotes;Bacteria;Firmicutes - Bacilli;Streptococcus"
4162	snx	Streptococcus pneumoniae OXC141 (serotype 3)	"Prokaryotes;Bacteria;Firmicutes - Bacilli;Streptococcus"
4163	snu	Streptococcus pneumoniae SPNA45 (serotype 3)	"Prokaryotes;Bacteria;Firmicutes - Bacilli;Streptococcus"
4164	spne	Streptococcus pneumoniae SPN034156 (serotype 3)	"Prokaryotes;Bacteria;Firmicutes - Bacilli;Streptococcus"
4165	spnu	Streptococcus pneumoniae SPN034183 (serotype 3)	"Prokaryotes;Bacteria;Firmicutes - Bacilli;Streptococcus"
4166	spnm	Streptococcus pneumoniae SPN994038 (serotype 3)	"Prokaryotes;Bacteria;Firmicutes - Bacilli;Streptococcus"
4167	spno	Streptococcus pneumoniae SPN994039 (serotype 3)	"Prokaryotes;Bacteria;Firmicutes - Bacilli;Streptococcus"
4168	sag	Streptococcus agalactiae 2603 (serotype V)	"Prokaryotes;Bacteria;Firmicutes - Bacilli;Streptococcus"

4169	san	Streptococcus agalactiae NEM316 (serotype III)	"Prokaryotes;Bacteria;Firmicutes - Bacilli;Streptococcus"
4170	sak	Streptococcus agalactiae A909 (serotype Ia)	"Prokaryotes;Bacteria;Firmicutes - Bacilli;Streptococcus"
4171	sgc	Streptococcus agalactiae GD201008-001 (serotype Ia)	"Prokaryotes;Bacteria;Firmicutes - Bacilli;Streptococcus"
4172	sags	Streptococcus agalactiae SA20	"Prokaryotes;Bacteria;Firmicutes - Bacilli;Streptococcus"
4173	sagl	Streptococcus agalactiae 2-22 (serotype Ib)	"Prokaryotes;Bacteria;Firmicutes - Bacilli;Streptococcus"
4174	sagm	Streptococcus agalactiae 09mas018883	"Prokaryotes;Bacteria;Firmicutes - Bacilli;Streptococcus"
4175	sagi	Streptococcus agalactiae ILRI005	"Prokaryotes;Bacteria;Firmicutes - Bacilli;Streptococcus"
4176	sagr	Streptococcus agalactiae ILRI112	"Prokaryotes;Bacteria;Firmicutes - Bacilli;Streptococcus"
4177	sagp	Streptococcus agalactiae 138P	"Prokaryotes;Bacteria;Firmicutes - Bacilli;Streptococcus"
4178	sagc	Streptococcus agalactiae 138spar	"Prokaryotes;Bacteria;Firmicutes - Bacilli;Streptococcus"
4179	sagt	Streptococcus agalactiae COH1	"Prokaryotes;Bacteria;Firmicutes - Bacilli;Streptococcus"
4180	sage	Streptococcus agalactiae NGBS061	"Prokaryotes;Bacteria;Firmicutes - Bacilli;Streptococcus"
4181	sagg	Streptococcus agalactiae NGBS572	"Prokaryotes;Bacteria;Firmicutes - Bacilli;Streptococcus"
4182	sagn	Streptococcus agalactiae CNCTC 10/84	"Prokaryotes;Bacteria;Firmicutes - Bacilli;Streptococcus"
4183	smu	Streptococcus mutans UA159 (serotype c)	"Prokaryotes;Bacteria;Firmicutes - Bacilli;Streptococcus"
4184	smc	Streptococcus mutans NN2025 (serotype c)	"Prokaryotes;Bacteria;Firmicutes - Bacilli;Streptococcus"
4185	smut	Streptococcus mutans GS-5 (serotype c)	"Prokaryotes;Bacteria;Firmicutes - Bacilli;Streptococcus"
4186	smj	Streptococcus mutans LJ23 (serotype k)	"Prokaryotes;Bacteria;Firmicutes - Bacilli;Streptococcus"
4187	smua	Streptococcus mutans UA159-FR	"Prokaryotes;Bacteria;Firmicutes - Bacilli;Streptococcus"
4188	stc	Streptococcus thermophilus CNRZ1066	"Prokaryotes;Bacteria;Firmicutes - Bacilli;Streptococcus"
4189	stl	Streptococcus thermophilus LMG 18311	"Prokaryotes;Bacteria;Firmicutes - Bacilli;Streptococcus"
4190	ste	Streptococcus thermophilus LMD-9	"Prokaryotes;Bacteria;Firmicutes - Bacilli;Streptococcus"
4191	stn	Streptococcus thermophilus ND03	"Prokaryotes;Bacteria;Firmicutes - Bacilli;Streptococcus"
4192	stu	Streptococcus thermophilus JIM 8232	"Prokaryotes;Bacteria;Firmicutes - Bacilli;Streptococcus"
4193	stw	Streptococcus thermophilus MN-ZLW-002	"Prokaryotes;Bacteria;Firmicutes - Bacilli;Streptococcus"
4194	sthe	Streptococcus thermophilus ASCC 1275	"Prokaryotes;Bacteria;Firmicutes - Bacilli;Streptococcus"
4195	sths	Streptococcus thermophilus S9	"Prokaryotes;Bacteria;Firmicutes - Bacilli;Streptococcus"
4196	ssa	Streptococcus sanguinis	"Prokaryotes;Bacteria;Firmicutes - Bacilli;Streptococcus"
4197	ssb	Streptococcus suis BM407 (serotype 2)	"Prokaryotes;Bacteria;Firmicutes - Bacilli;Streptococcus"
4198	ssu	Streptococcus suis 05ZYH33 (serotype 2)	"Prokaryotes;Bacteria;Firmicutes - Bacilli;Streptococcus"
4199	ssv	Streptococcus suis 98HAH33 (serotype 2)	"Prokaryotes;Bacteria;Firmicutes - Bacilli;Streptococcus"
4200	ssi	Streptococcus suis P1/7 (serotype 2)	"Prokaryotes;Bacteria;Firmicutes - Bacilli;Streptococcus"
4201	sss	Streptococcus suis SC84 (serotype 2)	"Prokaryotes;Bacteria;Firmicutes - Bacilli;Streptococcus"
4202	ssf	Streptococcus suis A7 (serotype 2)	"Prokaryotes;Bacteria;Firmicutes - Bacilli;Streptococcus"
4203	ssw	Streptococcus suis GZ1 (serotype 2)	"Prokaryotes;Bacteria;Firmicutes - Bacilli;Streptococcus"
4204	sup	Streptococcus suis S735 (serotype 2)	"Prokaryotes;Bacteria;Firmicutes - Bacilli;Streptococcus"
4205	ssus	Streptococcus suis SC070731 (serotype 2)	"Prokaryotes;Bacteria;Firmicutes - Bacilli;Streptococcus"
4206	sst	Streptococcus suis ST3 (serotype 3)	"Prokaryotes;Bacteria;Firmicutes - Bacilli;Streptococcus"
4207	ssuy	Streptococcus suis YB51 (serotype 3)	"Prokaryotes;Bacteria;Firmicutes - Bacilli;Streptococcus"
4208	ssk	Streptococcus suis D12 (serotype 9)	"Prokaryotes;Bacteria;Firmicutes - Bacilli;Streptococcus"
4209	ssq	Streptococcus suis D9 (serotype 7)	"Prokaryotes;Bacteria;Firmicutes - Bacilli;Streptococcus"

4210	sui	Streptococcus suis JS14 (serotype 14)	"Prokaryotes;Bacteria;Firmicutes - Bacilli;Streptococcus"
4211	suo	Streptococcus suis SS12 (serotype 1/2)	"Prokaryotes;Bacteria;Firmicutes - Bacilli;Streptococcus"
4212	srp	Streptococcus suis ST1 (serotype 1)	"Prokaryotes;Bacteria;Firmicutes - Bacilli;Streptococcus"
4213	ssut	Streptococcus suis TL13 (serotype 16)	"Prokaryotes;Bacteria;Firmicutes - Bacilli;Streptococcus"
4214	ssui	Streptococcus suis T15	"Prokaryotes;Bacteria;Firmicutes - Bacilli;Streptococcus"
4215	sgo	Streptococcus gordonii	"Prokaryotes;Bacteria;Firmicutes - Bacilli;Streptococcus"
4216	sez	Streptococcus equi subsp. zooepidemicus MGCS10565	"Prokaryotes;Bacteria;Firmicutes - Bacilli;Streptococcus"
4217	seq	Streptococcus equi subsp. zooepidemicus H70	"Prokaryotes;Bacteria;Firmicutes - Bacilli;Streptococcus"
4218	sezo	Streptococcus equi subsp. zooepidemicus ATCC 35246	"Prokaryotes;Bacteria;Firmicutes - Bacilli;Streptococcus"
4219	sequ	Streptococcus equi subsp. zooepidemicus CY	"Prokaryotes;Bacteria;Firmicutes - Bacilli;Streptococcus"
4220	seu	Streptococcus equi subsp. equi 4047	"Prokaryotes;Bacteria;Firmicutes - Bacilli;Streptococcus"
4221	sub	Streptococcus uberis	"Prokaryotes;Bacteria;Firmicutes - Bacilli;Streptococcus"
4222	sds	Streptococcus dysgalactiae subsp. equisimilis GGS_124	"Prokaryotes;Bacteria;Firmicutes - Bacilli;Streptococcus"
4223	sdg	Streptococcus dysgalactiae subsp. equisimilis ATCC 12394	"Prokaryotes;Bacteria;Firmicutes - Bacilli;Streptococcus"
4224	sda	Streptococcus dysgalactiae subsp. equisimilis RE378	"Prokaryotes;Bacteria;Firmicutes - Bacilli;Streptococcus"
4225	sdc	Streptococcus dysgalactiae subsp. equisimilis AC-2713	"Prokaryotes;Bacteria;Firmicutes - Bacilli;Streptococcus"
4226	sdq	Streptococcus dysgalactiae subsp. equisimilis 167	"Prokaryotes;Bacteria;Firmicutes - Bacilli;Streptococcus"
4227	sga	Streptococcus gallolyticus UCN34	"Prokaryotes;Bacteria;Firmicutes - Bacilli;Streptococcus"
4228	sgg	Streptococcus gallolyticus subsp. gallolyticus ATCC BAA-2069	"Prokaryotes;Bacteria;Firmicutes - Bacilli;Streptococcus"
4229	sgt	Streptococcus gallolyticus subsp. gallolyticus ATCC 43143	"Prokaryotes;Bacteria;Firmicutes - Bacilli;Streptococcus"
4230	smb	Streptococcus mitis	"Prokaryotes;Bacteria;Firmicutes - Bacilli;Streptococcus"
4231	sor	Streptococcus oralis	"Prokaryotes;Bacteria;Firmicutes - Bacilli;Streptococcus"
4232	stk	Streptococcus parauberis	"Prokaryotes;Bacteria;Firmicutes - Bacilli;Streptococcus"
4233	stb	Streptococcus pasteurianus	"Prokaryotes;Bacteria;Firmicutes - Bacilli;Streptococcus"
4234	scp	Streptococcus parasanguinis ATCC 15912	"Prokaryotes;Bacteria;Firmicutes - Bacilli;Streptococcus"
4235	scf	Streptococcus parasanguinis FW213	"Prokaryotes;Bacteria;Firmicutes - Bacilli;Streptococcus"
4236	ssr	Streptococcus salivarius CCHSS3	"Prokaryotes;Bacteria;Firmicutes - Bacilli;Streptococcus"
4237	stf	Streptococcus salivarius 57.I	"Prokaryotes;Bacteria;Firmicutes - Bacilli;Streptococcus"
4238	stj	Streptococcus salivarius JIM8777	"Prokaryotes;Bacteria;Firmicutes - Bacilli;Streptococcus"
4239	strs	Streptococcus salivarius NCTC 8618	"Prokaryotes;Bacteria;Firmicutes - Bacilli;Streptococcus"
4240	ssah	Streptococcus salivarius HSISS4	"Prokaryotes;Bacteria;Firmicutes - Bacilli;Streptococcus"
4241	std	Streptococcus pseudopneumoniae	"Prokaryotes;Bacteria;Firmicutes - Bacilli;Streptococcus"
4242	smn	Streptococcus macedonicus	"Prokaryotes;Bacteria;Firmicutes - Bacilli;Streptococcus"
4243	sif	Streptococcus infantarius subsp. infantarius	"Prokaryotes;Bacteria;Firmicutes - Bacilli;Streptococcus"
4244	sie	Streptococcus intermedius JTH08	"Prokaryotes;Bacteria;Firmicutes - Bacilli;Streptococcus"
4245	sib	Streptococcus intermedius B196	"Prokaryotes;Bacteria;Firmicutes - Bacilli;Streptococcus"
4246	siu	Streptococcus intermedius C270	"Prokaryotes;Bacteria;Firmicutes - Bacilli;Streptococcus"
4247	sang	Streptococcus anginosus C1051	"Prokaryotes;Bacteria;Firmicutes - Bacilli;Streptococcus"
4248	sanc	Streptococcus anginosus C238	"Prokaryotes;Bacteria;Firmicutes - Bacilli;Streptococcus"
4249	sans	Streptococcus anginosus SA1	"Prokaryotes;Bacteria;Firmicutes - Bacilli;Streptococcus"
4250	scg	Streptococcus constellatus subsp. pharyngis C1050	"Prokaryotes;Bacteria;Firmicutes - Bacilli;Streptococcus"

4251	scon	Streptococcus constellatus subsp. pharyngis C232	"Prokaryotes;Bacteria;Firmicutes - Bacilli;Streptococcus"
4252	scos	Streptococcus constellatus subsp. pharyngis C818	"Prokaryotes;Bacteria;Firmicutes - Bacilli;Streptococcus"
4253	soi	Streptococcus cristatus	"Prokaryotes;Bacteria;Firmicutes - Bacilli;Streptococcus"
4254	sik	Streptococcus iniae SF1	"Prokaryotes;Bacteria;Firmicutes - Bacilli;Streptococcus"
4255	siq	Streptococcus iniae ISET0901	"Prokaryotes;Bacteria;Firmicutes - Bacilli;Streptococcus"
4256	sio	Streptococcus iniae ISNO	"Prokaryotes;Bacteria;Firmicutes - Bacilli;Streptococcus"
4257	siz	Streptococcus iniae SFST01-82	"Prokaryotes;Bacteria;Firmicutes - Bacilli;Streptococcus"
4258	slu	Streptococcus lutetiensis	"Prokaryotes;Bacteria;Firmicutes - Bacilli;Streptococcus"
4259	sig	Streptococcus sp. I-G2	"Prokaryotes;Bacteria;Firmicutes - Bacilli;Streptococcus"
4260	sip	Streptococcus sp. I-P16	"Prokaryotes;Bacteria;Firmicutes - Bacilli;Streptococcus"
4261	stv	Streptococcus sp. VT 162	"Prokaryotes;Bacteria;Firmicutes - Bacilli;Streptococcus"
4262	spat	Streptococcus pantholopis	"Prokaryotes;Bacteria;Firmicutes - Bacilli;Streptococcus"
4263	stra	Streptococcus sp. A12	"Prokaryotes;Bacteria;Firmicutes - Bacilli;Streptococcus"
4264	strn	Streptococcus sp. NPS 308	"Prokaryotes;Bacteria;Firmicutes - Bacilli;Streptococcus"
4265	ssob	Streptococcus sobrinus	"Prokaryotes;Bacteria;Firmicutes - Bacilli;Streptococcus"
4266	srq	Streptococcus ruminantium	"Prokaryotes;Bacteria;Firmicutes - Bacilli;Streptococcus"
4267	seqi	Streptococcus equinus	"Prokaryotes;Bacteria;Firmicutes - Bacilli;Streptococcus"
4268	ski	Streptococcus koreensis	"Prokaryotes;Bacteria;Firmicutes - Bacilli;Streptococcus"
4269	spei	Streptococcus periodonticum	"Prokaryotes;Bacteria;Firmicutes - Bacilli;Streptococcus"
4270	srat	Streptococcus ratti	"Prokaryotes;Bacteria;Firmicutes - Bacilli;Streptococcus"
4271	sgw	Streptococcus gwangjuense	"Prokaryotes;Bacteria;Firmicutes - Bacilli;Streptococcus"
4272	splr	Streptococcus pluranimalium	"Prokaryotes;Bacteria;Firmicutes - Bacilli;Streptococcus"
4273	strg	Streptococcus troglodytae	"Prokaryotes;Bacteria;Firmicutes - Bacilli;Streptococcus"
4274	ljo	Lactobacillus johnsonii NCC 533	"Prokaryotes;Bacteria;Firmicutes - Bacilli;Lactobacillus"
4275	ljf	Lactobacillus johnsonii FI9785	"Prokaryotes;Bacteria;Firmicutes - Bacilli;Lactobacillus"
4276	ljh	Lactobacillus johnsonii DPC 6026	"Prokaryotes;Bacteria;Firmicutes - Bacilli;Lactobacillus"
4277	ljn	Lactobacillus johnsonii N6.2	"Prokaryotes;Bacteria;Firmicutes - Bacilli;Lactobacillus"
4278	lac	Lactobacillus acidophilus NCFM	"Prokaryotes;Bacteria;Firmicutes - Bacilli;Lactobacillus"
4279	lad	Lactobacillus acidophilus La-14	"Prokaryotes;Bacteria;Firmicutes - Bacilli;Lactobacillus"
4280	laf	Lactobacillus acidophilus FSI4	"Prokaryotes;Bacteria;Firmicutes - Bacilli;Lactobacillus"
4281	ldb	Lactobacillus delbrueckii subsp. bulgaricus ATCC 11842	"Prokaryotes;Bacteria;Firmicutes - Bacilli;Lactobacillus"
4282	lbu	Lactobacillus delbrueckii subsp. bulgaricus ATCC BAA-365	"Prokaryotes;Bacteria;Firmicutes - Bacilli;Lactobacillus"
4283	lde	Lactobacillus delbrueckii subsp. bulgaricus ND02	"Prokaryotes;Bacteria;Firmicutes - Bacilli;Lactobacillus"
4284	ldl	Lactobacillus delbrueckii subsp. bulgaricus 2038	"Prokaryotes;Bacteria;Firmicutes - Bacilli;Lactobacillus"
4285	lga	Lactobacillus gasseri ATCC 33323	"Prokaryotes;Bacteria;Firmicutes - Bacilli;Lactobacillus"
4286	lhe	Lactobacillus helveticus DPC 4571	"Prokaryotes;Bacteria;Firmicutes - Bacilli;Lactobacillus"
4287	lhl	Lactobacillus helveticus H10	"Prokaryotes;Bacteria;Firmicutes - Bacilli;Lactobacillus"
4288	lhr	Lactobacillus helveticus R0052	"Prokaryotes;Bacteria;Firmicutes - Bacilli;Lactobacillus"
4289	lhv	Lactobacillus helveticus CNRZ32	"Prokaryotes;Bacteria;Firmicutes - Bacilli;Lactobacillus"
4290	lhh	Lactobacillus helveticus H9	"Prokaryotes;Bacteria;Firmicutes - Bacilli;Lactobacillus"
4291	lhd	Lactobacillus helveticus KLDS1.8701	"Prokaryotes;Bacteria;Firmicutes - Bacilli;Lactobacillus"

4292	lcr	Lactobacillus crispatus	"Prokaryotes;Bacteria;Firmicutes - Bacilli;Lactobacillus"
4293	lam	Lactobacillus amylovorus GRL 1112	"Prokaryotes;Bacteria;Firmicutes - Bacilli;Lactobacillus"
4294	lai	Lactobacillus amylovorus 30SC	"Prokaryotes;Bacteria;Firmicutes - Bacilli;Lactobacillus"
4295	lay	Lactobacillus amylovorus GRL1118	"Prokaryotes;Bacteria;Firmicutes - Bacilli;Lactobacillus"
4296	lke	Lactobacillus kefiranofaciens	"Prokaryotes;Bacteria;Firmicutes - Bacilli;Lactobacillus"
4297	law	Lactobacillus sp. wkB8	"Prokaryotes;Bacteria;Firmicutes - Bacilli;Lactobacillus"
4298	lae	Lactobacillus acetotolerans	"Prokaryotes;Bacteria;Firmicutes - Bacilli;Lactobacillus"
4299	lgl	Lactobacillus gallinarum	"Prokaryotes;Bacteria;Firmicutes - Bacilli;Lactobacillus"
4300	lje	Lactobacillus jensenii	"Prokaryotes;Bacteria;Firmicutes - Bacilli;Lactobacillus"
4301	lamy	Lactobacillus amylolyticus	"Prokaryotes;Bacteria;Firmicutes - Bacilli;Lactobacillus"
4302	lpw	Lactobacillus paragasseri	"Prokaryotes;Bacteria;Firmicutes - Bacilli;Lactobacillus"
4303	lkl	Lactobacillus kullabergensis	"Prokaryotes;Bacteria;Firmicutes - Bacilli;Lactobacillus"
4304	lapi	Lactobacillus apis	"Prokaryotes;Bacteria;Firmicutes - Bacilli;Lactobacillus"
4305	lhs	Lactobacillus helsingborgensis	"Prokaryotes;Bacteria;Firmicutes - Bacilli;Lactobacillus"
4306	lca	Lactobacillus paracasei ATCC 334	"Prokaryotes;Bacteria;Firmicutes - Bacilli;Lacticaseibacillus"
4307	lcz	Lacticaseibacillus paracasei Zhang	"Prokaryotes;Bacteria;Firmicutes - Bacilli;Lacticaseibacillus"
4308	lcs	Lactobacillus paracasei BD-II	"Prokaryotes;Bacteria;Firmicutes - Bacilli;Lacticaseibacillus"
4309	lce	Lactobacillus paracasei LC2W	"Prokaryotes;Bacteria;Firmicutes - Bacilli;Lacticaseibacillus"
4310	lcw	Lacticaseibacillus paracasei W56	"Prokaryotes;Bacteria;Firmicutes - Bacilli;Lacticaseibacillus"
4311	lcl	Lactobacillus paracasei LOCK919	"Prokaryotes;Bacteria;Firmicutes - Bacilli;Lacticaseibacillus"
4312	lpq	Lactobacillus paracasei N1115	"Prokaryotes;Bacteria;Firmicutes - Bacilli;Lacticaseibacillus"
4313	lpi	Lactobacillus paracasei subsp. paracasei 8700:2	"Prokaryotes;Bacteria;Firmicutes - Bacilli;Lacticaseibacillus"
4314	lpap	Lactobacillus paracasei subsp. paracasei JCM 8130	"Prokaryotes;Bacteria;Firmicutes - Bacilli;Lacticaseibacillus"
4315	lcb	Lactobacillus casei BL23	"Prokaryotes;Bacteria;Firmicutes - Bacilli;Lacticaseibacillus"
4316	lcx	Lactobacillus casei 12A	"Prokaryotes;Bacteria;Firmicutes - Bacilli;Lacticaseibacillus"
4317	lrh	Lactobacillus rhamnosus GG	"Prokaryotes;Bacteria;Firmicutes - Bacilli;Lacticaseibacillus"
4318	lrg	Lactobacillus rhamnosus GG	"Prokaryotes;Bacteria;Firmicutes - Bacilli;Lacticaseibacillus"
4319	lrl	Lactobacillus rhamnosus Lc 705	"Prokaryotes;Bacteria;Firmicutes - Bacilli;Lacticaseibacillus"
4320	lra	Lactobacillus rhamnosus ATCC 8530	"Prokaryotes;Bacteria;Firmicutes - Bacilli;Lacticaseibacillus"
4321	lro	Lactobacillus rhamnosus LOCK900	"Prokaryotes;Bacteria;Firmicutes - Bacilli;Lacticaseibacillus"
4322	lrc	Lactobacillus rhamnosus LOCK908	"Prokaryotes;Bacteria;Firmicutes - Bacilli;Lacticaseibacillus"
4323	lpl	Lactobacillus plantarum WCFS1	"Prokaryotes;Bacteria;Firmicutes - Bacilli;Lactiplantibacillus"
4324	lpj	Lactobacillus plantarum JDM1	"Prokaryotes;Bacteria;Firmicutes - Bacilli;Lactiplantibacillus"
4325	lpt	Lactobacillus plantarum ZJ316	"Prokaryotes;Bacteria;Firmicutes - Bacilli;Lactiplantibacillus"
4326	lps	Lactobacillus plantarum subsp. plantarum ST-III	"Prokaryotes;Bacteria;Firmicutes - Bacilli;Lactiplantibacillus"
4327	lpr	Lactobacillus plantarum subsp. plantarum P-8	"Prokaryotes;Bacteria;Firmicutes - Bacilli;Lactiplantibacillus"
4328	lpz	Lactobacillus plantarum 16	"Prokaryotes;Bacteria;Firmicutes - Bacilli;Lactiplantibacillus"
4329	lpb	Lactiplantibacillus plantarum B21	"Prokaryotes;Bacteria;Firmicutes - Bacilli;Lactiplantibacillus"
4330	lpx	Lactiplantibacillus paraplantarum	"Prokaryotes;Bacteria;Firmicutes - Bacilli;Lactiplantibacillus"
4331	lpg	Lactiplantibacillus pentosus	"Prokaryotes;Bacteria;Firmicutes - Bacilli;Lactiplantibacillus"
4332	lre	Lactobacillus reuteri DSM 20016	"Prokaryotes;Bacteria;Firmicutes - Bacilli;Limosilactobacillus"

4333	lrf	Lactobacillus reuteri JCM 1112	"Prokaryotes;Bacteria;Firmicutes - Bacilli;Limosilactobacillus"
4334	lru	Lactobacillus reuteri SD2112	"Prokaryotes;Bacteria;Firmicutes - Bacilli;Limosilactobacillus"
4335	lrt	Lactobacillus reuteri I5007	"Prokaryotes;Bacteria;Firmicutes - Bacilli;Limosilactobacillus"
4336	lrr	Lactobacillus reuteri TD1	"Prokaryotes;Bacteria;Firmicutes - Bacilli;Limosilactobacillus"
4337	lfe	Lactobacillus fermentum IFO 3956	"Prokaryotes;Bacteria;Firmicutes - Bacilli;Limosilactobacillus"
4338	lfr	Lactobacillus fermentum CECT 5716	"Prokaryotes;Bacteria;Firmicutes - Bacilli;Limosilactobacillus"
4339	lff	Lactobacillus fermentum F-6	"Prokaryotes;Bacteria;Firmicutes - Bacilli;Limosilactobacillus"
4340	lmu	Lactobacillus mucosae	"Prokaryotes;Bacteria;Firmicutes - Bacilli;Limosilactobacillus"
4341	lor	Limosilactobacillus oris	"Prokaryotes;Bacteria;Firmicutes - Bacilli;Limosilactobacillus"
4342	lva	Limosilactobacillus vaginalis	"Prokaryotes;Bacteria;Firmicutes - Bacilli;Limosilactobacillus"
4343	lfn	Limosilactobacillus frumenti	"Prokaryotes;Bacteria;Firmicutes - Bacilli;Limosilactobacillus"
4344	lpon	Limosilactobacillus pontis	"Prokaryotes;Bacteria;Firmicutes - Bacilli;Limosilactobacillus"
4345	lng	Liquorilactobacillus nagelii	"Prokaryotes;Bacteria;Firmicutes - Bacilli;Liquorilactobacillus"
4346	lhw	Liquorilactobacillus hordei	"Prokaryotes;Bacteria;Firmicutes - Bacilli;Liquorilactobacillus"
4347	lmal	Liquorilactobacillus mali	"Prokaryotes;Bacteria;Firmicutes - Bacilli;Liquorilactobacillus"
4348	lsn	Lactobacillus sanfranciscensis	"Prokaryotes;Bacteria;Firmicutes - Bacilli;Fructilactobacillus"
4349	lle	Fructilactobacillus lindneri	"Prokaryotes;Bacteria;Firmicutes - Bacilli;Fructilactobacillus"
4350	lfv	Fructilactobacillus fructivorans	"Prokaryotes;Bacteria;Firmicutes - Bacilli;Fructilactobacillus"
4351	lbh	Lactobacillus buchneri NRRL B-30929	"Prokaryotes;Bacteria;Firmicutes - Bacilli;Lentilactobacillus"
4352	lbn	Lactobacillus buchneri subsp. silagei CD034	"Prokaryotes;Bacteria;Firmicutes - Bacilli;Lentilactobacillus"
4353	lpar	Lactobacillus parabuchneri	"Prokaryotes;Bacteria;Firmicutes - Bacilli;Lentilactobacillus"
4354	lcu	Lentilactobacillus curieae	"Prokaryotes;Bacteria;Firmicutes - Bacilli;Lentilactobacillus"
4355	lkf	Lentilactobacillus kefiri	"Prokaryotes;Bacteria;Firmicutes - Bacilli;Lentilactobacillus"
4356	lhil	Lentilactobacillus hilgardii	"Prokaryotes;Bacteria;Firmicutes - Bacilli;Lentilactobacillus"
4357	lbr	Lactobacillus brevis ATCC 367	"Prokaryotes;Bacteria;Firmicutes - Bacilli;Levilactobacillus"
4358	lbk	Lactobacillus brevis KB290	"Prokaryotes;Bacteria;Firmicutes - Bacilli;Levilactobacillus"
4359	lko	Levilactobacillus koreensis	"Prokaryotes;Bacteria;Firmicutes - Bacilli;Levilactobacillus"
4360	lzy	Lactobacillus zymae	"Prokaryotes;Bacteria;Firmicutes - Bacilli;Levilactobacillus"
4361	lsua	Levilactobacillus suantsaii	"Prokaryotes;Bacteria;Firmicutes - Bacilli;Levilactobacillus"
4362	lji	Acetilactobacillus jinshanensis	"Prokaryotes;Bacteria;Firmicutes - Bacilli;Acetilactobacillus"
4363	lsl	Lactobacillus salivarius UCC118	"Prokaryotes;Bacteria;Firmicutes - Bacilli;Ligilactobacillus"
4364	lsi	Lactobacillus salivarius CECT 5713	"Prokaryotes;Bacteria;Firmicutes - Bacilli;Ligilactobacillus"
4365	lsj	Ligilactobacillus salivarius JCM1046	"Prokaryotes;Bacteria;Firmicutes - Bacilli;Ligilactobacillus"
4366	lrm	Lactobacillus ruminis	"Prokaryotes;Bacteria;Firmicutes - Bacilli;Ligilactobacillus"
4367	lagl	Ligilactobacillus agilis	"Prokaryotes;Bacteria;Firmicutes - Bacilli;Ligilactobacillus"
4368	laca	Lactobacillus acidipiscis	"Prokaryotes;Bacteria;Firmicutes - Bacilli;Ligilactobacillus"
4369	lani	Ligilactobacillus animalis	"Prokaryotes;Bacteria;Firmicutes - Bacilli;Ligilactobacillus"
4370	lbt	Loigolactobacillus backii	"Prokaryotes;Bacteria;Firmicutes - Bacilli;Loigolactobacillus"
4371	lcy	Lactobacillus coryniformis	"Prokaryotes;Bacteria;Firmicutes - Bacilli;Loigolactobacillus"
4372	lho	Lactobacillus hokkaidonensis	"Prokaryotes;Bacteria;Firmicutes - Bacilli;Paucilactobacillus"
4373	lol	Lactobacillus oligofermentans	"Prokaryotes;Bacteria;Firmicutes - Bacilli;Paucilactobacillus"

4374	lnn	Paucilactobacillus nenjiangensis	"Prokaryotes;Bacteria;Firmicutes - Bacilli;Paucilactobacillus"
4375	lku	Lactobacillus kunkeei	"Prokaryotes;Bacteria;Firmicutes - Bacilli;Apilactobacillus"
4376	lpd	Secundilactobacillus paracollinoides	"Prokaryotes;Bacteria;Firmicutes - Bacilli;Secundilactobacillus"
4377	lmae	Secundilactobacillus malefermentans	"Prokaryotes;Bacteria;Firmicutes - Bacilli;Secundilactobacillus"
4378	ppe	Pediococcus pentosaceus ATCC 25745	"Prokaryotes;Bacteria;Firmicutes - Bacilli;Pediococcus"
4379	ppen	Pediococcus pentosaceus SL4	"Prokaryotes;Bacteria;Firmicutes - Bacilli;Pediococcus"
4380	pce	Pediococcus claussenii	"Prokaryotes;Bacteria;Firmicutes - Bacilli;Pediococcus"
4381	pdm	Pediococcus damnosus	"Prokaryotes;Bacteria;Firmicutes - Bacilli;Pediococcus"
4382	paci	Pediococcus acidilactici	"Prokaryotes;Bacteria;Firmicutes - Bacilli;Pediococcus"
4383	pio	Pediococcus inopinatus	"Prokaryotes;Bacteria;Firmicutes - Bacilli;Pediococcus"
4384	lros	Furfurilactobacillus rossiae	"Prokaryotes;Bacteria;Firmicutes - Bacilli;Furfurilactobacillus"
4385	lgn	Companilactobacillus ginsenosidimutans	"Prokaryotes;Bacteria;Firmicutes - Bacilli;Companilactobacillus"
4386	lhi	Companilactobacillus heilongjiangensis	"Prokaryotes;Bacteria;Firmicutes - Bacilli;Companilactobacillus"
4387	lct	Companilactobacillus crustorum	"Prokaryotes;Bacteria;Firmicutes - Bacilli;Companilactobacillus"
4388	lalw	Companilactobacillus allii	"Prokaryotes;Bacteria;Firmicutes - Bacilli;Companilactobacillus"
4389	lali	Lactobacillus alimentarius	"Prokaryotes;Bacteria;Firmicutes - Bacilli;Companilactobacillus"
4390	lfm	Lactobacillus farciminis	"Prokaryotes;Bacteria;Firmicutes - Bacilli;Companilactobacillus"
4391	lzh	Companilactobacillus zhachilii	"Prokaryotes;Bacteria;Firmicutes - Bacilli;Companilactobacillus"
4392	lft	Companilactobacillus futsaii	"Prokaryotes;Bacteria;Firmicutes - Bacilli;Companilactobacillus"
4393	lsa	Lactobacillus sakei subsp. sakei	"Prokaryotes;Bacteria;Firmicutes - Bacilli;Latilactobacillus"
4394	lcv	Latilactobacillus curvatus	"Prokaryotes;Bacteria;Firmicutes - Bacilli;Latilactobacillus"
4395	lgm	Latilactobacillus graminis	"Prokaryotes;Bacteria;Firmicutes - Bacilli;Latilactobacillus"
4396	lah	Amylolactobacillus amylophilus	"Prokaryotes;Bacteria;Firmicutes - Bacilli;Amylolactobacillus"
4397	lbm	Bombilactobacillus bombi	"Prokaryotes;Bacteria;Firmicutes - Bacilli;Bombilactobacillus"
4398	lhb	Schleiferilactobacillus harbinensis	"Prokaryotes;Bacteria;Firmicutes - Bacilli;Schleiferilactobacillus"
4399	ldx	Lapidilactobacillus dextrinicus	"Prokaryotes;Bacteria;Firmicutes - Bacilli;Lapidilactobacillus"
4400	ooe	Oenococcus oeni	"Prokaryotes;Bacteria;Firmicutes - Bacilli;Oenococcus"
4401	oen	Oenococcus sp. UCMA 16435	"Prokaryotes;Bacteria;Firmicutes - Bacilli;Oenococcus"
4402	osi	Oenococcus sicerae	"Prokaryotes;Bacteria;Firmicutes - Bacilli;Oenococcus"
4403	lme	Leuconostoc mesenteroides subsp. mesenteroides ATCC 8293	"Prokaryotes;Bacteria;Firmicutes - Bacilli;Leuconostoc"
4404	lmm	Leuconostoc mesenteroides subsp. mesenteroides J18	"Prokaryotes;Bacteria;Firmicutes - Bacilli;Leuconostoc"
4405	lmk	Leuconostoc mesenteroides KFRI-MG	"Prokaryotes;Bacteria;Firmicutes - Bacilli;Leuconostoc"
4406	lci	Leuconostoc citreum	"Prokaryotes;Bacteria;Firmicutes - Bacilli;Leuconostoc"
4407	lki	Leuconostoc kimchii	"Prokaryotes;Bacteria;Firmicutes - Bacilli;Leuconostoc"
4408	lec	Leuconostoc sp. C2	"Prokaryotes;Bacteria;Firmicutes - Bacilli;Leuconostoc"
4409	lcn	Leuconostoc carnosum	"Prokaryotes;Bacteria;Firmicutes - Bacilli;Leuconostoc"
4410	lgs	Leuconostoc gelidum subsp. gasicomitatum	"Prokaryotes;Bacteria;Firmicutes - Bacilli;Leuconostoc"
4411	lge	Leuconostoc gelidum JB7	"Prokaryotes;Bacteria;Firmicutes - Bacilli;Leuconostoc"
4412	llf	Leuconostoc lactis	"Prokaryotes;Bacteria;Firmicutes - Bacilli;Leuconostoc"
4413	lgc	Leuconostoc garlicum	"Prokaryotes;Bacteria;Firmicutes - Bacilli;Leuconostoc"
4414	lsu	Leuconostoc mesenteroides subsp. suionicum	"Prokaryotes;Bacteria;Firmicutes - Bacilli;Leuconostoc"

4415	lpse	Leuconostoc pseudomesenteroides	"Prokaryotes;Bacteria;Firmicutes - Bacilli;Leuconostoc"
4416	wko	Weissella koreensis	"Prokaryotes;Bacteria;Firmicutes - Bacilli;Weissella"
4417	wce	Weissella ceti WS08	"Prokaryotes;Bacteria;Firmicutes - Bacilli;Weissella"
4418	wct	Weissella ceti WS74	"Prokaryotes;Bacteria;Firmicutes - Bacilli;Weissella"
4419	wci	Weissella ceti WS105	"Prokaryotes;Bacteria;Firmicutes - Bacilli;Weissella"
4420	wcb	Weissella cibaria	"Prokaryotes;Bacteria;Firmicutes - Bacilli;Weissella"
4421	wjo	Weissella jogaejeotgali	"Prokaryotes;Bacteria;Firmicutes - Bacilli;Weissella"
4422	wpa	Weissella paramesenteroides	"Prokaryotes;Bacteria;Firmicutes - Bacilli;Weissella"
4423	wcf	Weissella confusa	"Prokaryotes;Bacteria;Firmicutes - Bacilli;Weissella"
4424	wso	Weissella soli	"Prokaryotes;Bacteria;Firmicutes - Bacilli;Weissella"
4425	whe	Weissella hellenica	"Prokaryotes;Bacteria;Firmicutes - Bacilli;Weissella"
4426	wei	Weissella cryptocerci	"Prokaryotes;Bacteria;Firmicutes - Bacilli;Weissella"
4427	wdi	Weissella diestrammenae	"Prokaryotes;Bacteria;Firmicutes - Bacilli;Weissella"
4428	wvr	Weissella viridescens	"Prokaryotes;Bacteria;Firmicutes - Bacilli;Weissella"
4429	efa	Enterococcus faecalis V583	"Prokaryotes;Bacteria;Firmicutes - Bacilli;Enterococcus"
4430	efl	Enterococcus faecalis 62	"Prokaryotes;Bacteria;Firmicutes - Bacilli;Enterococcus"
4431	efi	Enterococcus faecalis OG1RF	"Prokaryotes;Bacteria;Firmicutes - Bacilli;Enterococcus"
4432	efd	Enterococcus faecalis D32	"Prokaryotes;Bacteria;Firmicutes - Bacilli;Enterococcus"
4433	efs	Enterococcus faecalis Symbioflor 1	"Prokaryotes;Bacteria;Firmicutes - Bacilli;Enterococcus"
4434	efn	Enterococcus faecalis DENG1	"Prokaryotes;Bacteria;Firmicutes - Bacilli;Enterococcus"
4435	efq	Enterococcus faecalis ATCC 29212	"Prokaryotes;Bacteria;Firmicutes - Bacilli;Enterococcus"
4436	ene	Enterococcus faecalis 7L76	"Prokaryotes;Bacteria;Firmicutes - Bacilli;Enterococcus"
4437	efc	Enterococcus faecium Aus0004	"Prokaryotes;Bacteria;Firmicutes - Bacilli;Enterococcus"
4438	efau	Enterococcus faecium Aus0085	"Prokaryotes;Bacteria;Firmicutes - Bacilli;Enterococcus"
4439	efu	Enterococcus faecium DO	"Prokaryotes;Bacteria;Firmicutes - Bacilli;Enterococcus"
4440	efm	Enterococcus faecium ATCC 8459 = NRRL B-2354	"Prokaryotes;Bacteria;Firmicutes - Bacilli;Enterococcus"
4441	eft	Enterococcus faecium T110	"Prokaryotes;Bacteria;Firmicutes - Bacilli;Enterococcus"
4442	ehr	Enterococcus hirae	"Prokaryotes;Bacteria;Firmicutes - Bacilli;Enterococcus"
4443	ecas	Enterococcus casseliflavus	"Prokaryotes;Bacteria;Firmicutes - Bacilli;Enterococcus"
4444	emu	Enterococcus mundtii	"Prokaryotes;Bacteria;Firmicutes - Bacilli;Enterococcus"
4445	edu	Enterococcus durans	"Prokaryotes;Bacteria;Firmicutes - Bacilli;Enterococcus"
4446	ega	Enterococcus gallinarum	"Prokaryotes;Bacteria;Firmicutes - Bacilli;Enterococcus"
4447	ess	Enterococcus silesiacus	"Prokaryotes;Bacteria;Firmicutes - Bacilli;Enterococcus"
4448	eth	Enterococcus thailandicus	"Prokaryotes;Bacteria;Firmicutes - Bacilli;Enterococcus"
4449	egv	Enterococcus gilvus	"Prokaryotes;Bacteria;Firmicutes - Bacilli;Enterococcus"
4450	eav	Enterococcus avium	"Prokaryotes;Bacteria;Firmicutes - Bacilli;Enterococcus"
4451	esg	Enterococcus saigonensis	"Prokaryotes;Bacteria;Firmicutes - Bacilli;Enterococcus"
4452	mps	Melissococcus plutonius ATCC 35311	"Prokaryotes;Bacteria;Firmicutes - Bacilli;Melissococcus"
4453	mpx	Melissococcus plutonius DAT561	"Prokaryotes;Bacteria;Firmicutes - Bacilli;Melissococcus"
4454	thl	Tetragenococcus halophilus NBRC 12172	"Prokaryotes;Bacteria;Firmicutes - Bacilli;Tetragenococcus"
4455	tey	Tetragenococcus halophilus	"Prokaryotes;Bacteria;Firmicutes - Bacilli;Tetragenococcus"

4456	too	Tetragenococcus osmophilus	"Prokaryotes;Bacteria;Firmicutes - Bacilli;Tetragenococcus"
4457	tkr	Tetragenococcus koreensis	"Prokaryotes;Bacteria;Firmicutes - Bacilli;Tetragenococcus"
4458	vte	Vagococcus teuberi	"Prokaryotes;Bacteria;Firmicutes - Bacilli;Vagococcus"
4459	vpi	Vagococcus penaei	"Prokaryotes;Bacteria;Firmicutes - Bacilli;Vagococcus"
4460	vac	Vagococcus sp. CF-49	"Prokaryotes;Bacteria;Firmicutes - Bacilli;Vagococcus"
4461	vao	Vagococcus zengguangii	"Prokaryotes;Bacteria;Firmicutes - Bacilli;Vagococcus"
4462	vah	Vagococcus coleopterorum	"Prokaryotes;Bacteria;Firmicutes - Bacilli;Vagococcus"
4463	vcp	Vagococcus carniphilus	"Prokaryotes;Bacteria;Firmicutes - Bacilli;Vagococcus"
4464	aur	Aerococcus urinae ACS-120-V-Col10a	"Prokaryotes;Bacteria;Firmicutes - Bacilli;Aerococcus"
4465	aun	Aerococcus urinae CCUG36881	"Prokaryotes;Bacteria;Firmicutes - Bacilli;Aerococcus"
4466	aui	Aerococcus urinaeequi	"Prokaryotes;Bacteria;Firmicutes - Bacilli;Aerococcus"
4467	asan	Aerococcus sanguinicola	"Prokaryotes;Bacteria;Firmicutes - Bacilli;Aerococcus"
4468	acg	Aerococcus christensenii	"Prokaryotes;Bacteria;Firmicutes - Bacilli;Aerococcus"
4469	avs	Aerococcus viridans	"Prokaryotes;Bacteria;Firmicutes - Bacilli;Aerococcus"
4470	auh	Aerococcus urinaehominis	"Prokaryotes;Bacteria;Firmicutes - Bacilli;Aerococcus"
4471	adc	Abiotrophia defectiva	"Prokaryotes;Bacteria;Firmicutes - Bacilli;Abiotrophia"
4472	abae	Suicoccus acidiformans	"Prokaryotes;Bacteria;Firmicutes - Bacilli;unclassified Aerococcaceae"
4473	crn	Carnobacterium sp. 17-4	"Prokaryotes;Bacteria;Firmicutes - Bacilli;Carnobacterium"
4474	cml	Carnobacterium maltaromaticum	"Prokaryotes;Bacteria;Firmicutes - Bacilli;Carnobacterium"
4475	caw	Carnobacterium inhibens	"Prokaryotes;Bacteria;Firmicutes - Bacilli;Carnobacterium"
4476	carc	Carnobacterium sp. CP1	"Prokaryotes;Bacteria;Firmicutes - Bacilli;Carnobacterium"
4477	cdj	Carnobacterium divergens	"Prokaryotes;Bacteria;Firmicutes - Bacilli;Carnobacterium"
4478	carn	Carnobacterium sp. PL17GRE32	"Prokaryotes;Bacteria;Firmicutes - Bacilli;Carnobacterium"
4479	marr	Marinilactibacillus sp. 15R	"Prokaryotes;Bacteria;Firmicutes - Bacilli;Marinilactibacillus"
4480	jep	Jeotgalibaca sp. PTS2502	"Prokaryotes;Bacteria;Firmicutes - Bacilli;Jeotgalibaca"
4481	jda	Jeotgalibaca dankookensis	"Prokaryotes;Bacteria;Firmicutes - Bacilli;Jeotgalibaca"
4482	jeh	Jeotgalibaca ciconiae	"Prokaryotes;Bacteria;Firmicutes - Bacilli;Jeotgalibaca"
4483	jar	Jeotgalibaca arthritidis	"Prokaryotes;Bacteria;Firmicutes - Bacilli;Jeotgalibaca"
4484	jpo	Jeotgalibaca porci	"Prokaryotes;Bacteria;Firmicutes - Bacilli;Jeotgalibaca"
4485	dpm	Dolosigranulum pigrum	"Prokaryotes;Bacteria;Firmicutes - Bacilli;Dolosigranulum"
4486	cac	Clostridium acetobutylicum ATCC 824	"Prokaryotes;Bacteria;Firmicutes - Clostridia;Clostridium"
4487	cae	Clostridium acetobutylicum DSM 1731	"Prokaryotes;Bacteria;Firmicutes - Clostridia;Clostridium"
4488	cay	Clostridium acetobutylicum EA 2018	"Prokaryotes;Bacteria;Firmicutes - Clostridia;Clostridium"
4489	cpe	Clostridium perfringens 13	"Prokaryotes;Bacteria;Firmicutes - Clostridia;Clostridium"
4490	cpf	Clostridium perfringens ATCC 13124	"Prokaryotes;Bacteria;Firmicutes - Clostridia;Clostridium"
4491	cpr	Clostridium perfringens SM101	"Prokaryotes;Bacteria;Firmicutes - Clostridia;Clostridium"
4492	ctc	Clostridium tetani E88	"Prokaryotes;Bacteria;Firmicutes - Clostridia;Clostridium"
4493	ctet	Clostridium tetani 12124569	"Prokaryotes;Bacteria;Firmicutes - Clostridia;Clostridium"
4494	cno	Clostridium novyi	"Prokaryotes;Bacteria;Firmicutes - Clostridia;Clostridium"
4495	cbo	Clostridium botulinum A ATCC 3502	"Prokaryotes;Bacteria;Firmicutes - Clostridia;Clostridium"
4496	cba	Clostridium botulinum A ATCC 19397	"Prokaryotes;Bacteria;Firmicutes - Clostridia;Clostridium"

4497	cbh	Clostridium botulinum A Hall	"Prokaryotes;Bacteria;Firmicutes - Clostridia;Clostridium"
4498	cby	Clostridium botulinum A2	"Prokaryotes;Bacteria;Firmicutes - Clostridia;Clostridium"
4499	cbl	Clostridium botulinum A3 Loch Maree	"Prokaryotes;Bacteria;Firmicutes - Clostridia;Clostridium"
4500	cbk	Clostridium botulinum B Eklund 17B (NRP)	"Prokaryotes;Bacteria;Firmicutes - Clostridia;Clostridium"
4501	cbb	Clostridium botulinum B1 Okra	"Prokaryotes;Bacteria;Firmicutes - Clostridia;Clostridium"
4502	cbi	Clostridium botulinum Ba4	"Prokaryotes;Bacteria;Firmicutes - Clostridia;Clostridium"
4503	cbn	Clostridium botulinum BKT015925	"Prokaryotes;Bacteria;Firmicutes - Clostridia;Clostridium"
4504	cbt	Clostridium botulinum E3	"Prokaryotes;Bacteria;Firmicutes - Clostridia;Clostridium"
4505	cbf	Clostridium botulinum F Langeland	"Prokaryotes;Bacteria;Firmicutes - Clostridia;Clostridium"
4506	cbm	Clostridium botulinum F 230613	"Prokaryotes;Bacteria;Firmicutes - Clostridia;Clostridium"
4507	cbj	Clostridium botulinum H04402 065	"Prokaryotes;Bacteria;Firmicutes - Clostridia;Clostridium"
4508	cbe	Clostridium beijerinckii NCIMB 8052	"Prokaryotes;Bacteria;Firmicutes - Clostridia;Clostridium"
4509	cbz	Clostridium beijerinckii ATCC 35702	"Prokaryotes;Bacteria;Firmicutes - Clostridia;Clostridium"
4510	cbei	Clostridium beijerinckii NCIMB 14988	"Prokaryotes;Bacteria;Firmicutes - Clostridia;Clostridium"
4511	ckl	Clostridium kluyveri DSM 555	"Prokaryotes;Bacteria;Firmicutes - Clostridia;Clostridium"
4512	ckr	Clostridium kluyveri NBRC 12016	"Prokaryotes;Bacteria;Firmicutes - Clostridia;Clostridium"
4513	clj	Clostridium ljungdahlii	"Prokaryotes;Bacteria;Firmicutes - Clostridia;Clostridium"
4514	ccb	Clostridium cellulovorans	"Prokaryotes;Bacteria;Firmicutes - Clostridia;Clostridium"
4515	cls	Clostridium sp. SY8519	"Prokaryotes;Bacteria;Firmicutes - Clostridia;Clostridium"
4516	clb	Clostridium sp. BNL1100	"Prokaryotes;Bacteria;Firmicutes - Clostridia;Clostridium"
4517	csr	Clostridium saccharoperbutylacetonicum	"Prokaryotes;Bacteria;Firmicutes - Clostridia;Clostridium"
4518	cpas	Clostridium pasteurianum BC1	"Prokaryotes;Bacteria;Firmicutes - Clostridia;Clostridium"
4519	cpat	Clostridium pasteurianum DSM 525 = ATCC 6013	"Prokaryotes;Bacteria;Firmicutes - Clostridia;Clostridium"
4520	cpae	Clostridium pasteurianum DSM 525 = ATCC 6013	"Prokaryotes;Bacteria;Firmicutes - Clostridia;Clostridium"
4521	csb	Clostridium saccharobutylicum	"Prokaryotes;Bacteria;Firmicutes - Clostridia;Clostridium"
4522	cah	Clostridium autoethanogenum	"Prokaryotes;Bacteria;Firmicutes - Clostridia;Clostridium"
4523	clt	Clostridium bornimense	"Prokaryotes;Bacteria;Firmicutes - Clostridia;Clostridium"
4524	cbv	Clostridium baratii	"Prokaryotes;Bacteria;Firmicutes - Clostridia;Clostridium"
4525	csq	Clostridium scatologenes	"Prokaryotes;Bacteria;Firmicutes - Clostridia;Clostridium"
4526	cld	Clostridium sporogenes	"Prokaryotes;Bacteria;Firmicutes - Clostridia;Clostridium"
4527	cace	Clostridium aceticum	"Prokaryotes;Bacteria;Firmicutes - Clostridia;Clostridium"
4528	cck	Clostridium carboxidivorans	"Prokaryotes;Bacteria;Firmicutes - Clostridia;Clostridium"
4529	cbut	Clostridium butyricum	"Prokaryotes;Bacteria;Firmicutes - Clostridia;Clostridium"
4530	ctyk	Clostridium tyrobutyricum	"Prokaryotes;Bacteria;Firmicutes - Clostridia;Clostridium"
4531	ceu	Clostridium estertheticum	"Prokaryotes;Bacteria;Firmicutes - Clostridia;Clostridium"
4532	ctae	Clostridium taeniosporum	"Prokaryotes;Bacteria;Firmicutes - Clostridia;Clostridium"
4533	cfm	Clostridium formicaceticum	"Prokaryotes;Bacteria;Firmicutes - Clostridia;Clostridium"
4534	cchv	Clostridium chauvoei	"Prokaryotes;Bacteria;Firmicutes - Clostridia;Clostridium"
4535	carg	Clostridium argentinense	"Prokaryotes;Bacteria;Firmicutes - Clostridia;Clostridium"
4536	cdrk	Clostridium drakei	"Prokaryotes;Bacteria;Firmicutes - Clostridia;Clostridium"
4537	cia	Clostridium isatidis	"Prokaryotes;Bacteria;Firmicutes - Clostridia;Clostridium"

4538	csep	Clostridium septicum	"Prokaryotes;Bacteria;Firmicutes - Clostridia;Clostridium"
4539	cdy	Clostridium diolis	"Prokaryotes;Bacteria;Firmicutes - Clostridia;Clostridium"
4540	ccoh	Clostridium cochlearium	"Prokaryotes;Bacteria;Firmicutes - Clostridia;Clostridium"
4541	cfer	Clostridium fermenticellae	"Prokaryotes;Bacteria;Firmicutes - Clostridia;Clostridium"
4542	amt	Alkaliphilus metalliredigens	"Prokaryotes;Bacteria;Firmicutes - Clostridia;Alkaliphilus"
4543	aoe	Alkaliphilus oremlandii	"Prokaryotes;Bacteria;Firmicutes - Clostridia;Alkaliphilus"
4544	asf	Candidatus Arthromitus sp. SFB-mouse-Japan	"Prokaryotes;Bacteria;Firmicutes - Clostridia;Arthromitus"
4545	asm	Candidatus Arthromitus sp. SFB-mouse-Yit	"Prokaryotes;Bacteria;Firmicutes - Clostridia;Arthromitus"
4546	aso	Candidatus Arthromitus sp. SFB-mouse-NL	"Prokaryotes;Bacteria;Firmicutes - Clostridia;Arthromitus"
4547	asb	Candidatus Arthromitus sp. SFB-rat-Yit	"Prokaryotes;Bacteria;Firmicutes - Clostridia;Arthromitus"
4548	gfe	Geosporobacter ferrireducens	"Prokaryotes;Bacteria;Firmicutes - Clostridia;Geosporobacter"
4549	hhw	Hathewaya histolytica	"Prokaryotes;Bacteria;Firmicutes - Clostridia;Hathewaya"
4550	cale	Caloramator sp. E03	"Prokaryotes;Bacteria;Firmicutes - Clostridia;Caloramator"
4551	crs	Crassaminicella sp. SY095	"Prokaryotes;Bacteria;Firmicutes - Clostridia;Crassaminicella"
4552	cazo	Caloranaerobacter azorensis	"Prokaryotes;Bacteria;Firmicutes - Clostridia;Caloranaerobacter"
4553	sarj	Sarcina sp. JB2	"Prokaryotes;Bacteria;Firmicutes - Clostridia;Sarcina"
4554	clo	Mageeibacillus indolicus	"Prokaryotes;Bacteria;Firmicutes - Clostridia;Mageeibacillus"
4555	fsa	Fastidiosipila sanguinis	"Prokaryotes;Bacteria;Firmicutes - Clostridia;Fastidiosipila"
4556	cth	Hungateiclostridium thermocellum ATCC 27405	"Prokaryotes;Bacteria;Firmicutes - Clostridia;Acetivibrio"
4557	ctx	Hungateiclostridium thermocellum DSM 1313	"Prokaryotes;Bacteria;Firmicutes - Clostridia;Acetivibrio"
4558	ccl	Hungateiclostridium clariflavum	"Prokaryotes;Bacteria;Firmicutes - Clostridia;Acetivibrio"
4559	hsc	Acetivibrio saccincola	"Prokaryotes;Bacteria;Firmicutes - Clostridia;Acetivibrio"
4560	ruk	Hungateiclostridiaceae bacterium KB18	"Prokaryotes;Bacteria;Firmicutes - Clostridia;unclassified Oscillospiraceae"
4561	rbp	Ruminococcaceae bacterium CPB6	"Prokaryotes;Bacteria;Firmicutes - Clostridia;unclassified Oscillospiraceae"
4562	cce	Ruminiclostridium cellulolyticum	"Prokaryotes;Bacteria;Firmicutes - Clostridia;Ruminiclostridium"
4563	css	Thermoclostridium stercorarium subsp. stercorarium DSM 8532	"Prokaryotes;Bacteria;Firmicutes - Clostridia;Thermoclostridium"
4564	csd	Thermoclostridium stercorarium subsp. stercorarium DSM 8532	"Prokaryotes;Bacteria;Firmicutes - Clostridia;Thermoclostridium"
4565	cthd	Pseudoclostridium thermosuccinogenes	"Prokaryotes;Bacteria;Firmicutes - Clostridia;Pseudoclostridium"
4566	esr	Eubacterium siraeum V10Sc8a	"Prokaryotes;Bacteria;Firmicutes - Clostridia;unclassified Ruminococcaceae"
4567	esu	Eubacterium siraeum 70/3	"Prokaryotes;Bacteria;Firmicutes - Clostridia;unclassified Ruminococcaceae"
4568	ccel	Clostridium cellulosi	"Prokaryotes;Bacteria;Firmicutes - Clostridia;unclassified Ruminococcaceae"
4569	fpla	Flavonifractor plautii	"Prokaryotes;Bacteria;Firmicutes - Clostridia;Flavonifractor"
4570	eha	Ethanoligenens harbinense	"Prokaryotes;Bacteria;Firmicutes - Clostridia;Ethanoligenens"
4571	ral	Ruminococcus albus	"Prokaryotes;Bacteria;Firmicutes - Clostridia;Ruminococcus"
4572	rch	Ruminococcus champanellensis	"Prokaryotes;Bacteria;Firmicutes - Clostridia;Ruminococcus"
4573	rum	Ruminococcus sp. SR1/5	"Prokaryotes;Bacteria;Firmicutes - Clostridia;Ruminococcus"
4574	rus	Ruminococcus bicirculans	"Prokaryotes;Bacteria;Firmicutes - Clostridia;Ruminococcus"
4575	ruj	Ruminococcus sp. JE7A12	"Prokaryotes;Bacteria;Firmicutes - Clostridia;Ruminococcus"
4576	fpr	Faecalibacterium prausnitzii L2-6	"Prokaryotes;Bacteria;Firmicutes - Clostridia;Faecalibacterium"
4577	fpa	Faecalibacterium prausnitzii SL3/3	"Prokaryotes;Bacteria;Firmicutes - Clostridia;Faecalibacterium"
4578	fpra	Faecalibacterium prausnitzii A2-165	"Prokaryotes;Bacteria;Firmicutes - Clostridia;Faecalibacterium"

4579	capr	Caproiciproducens sp. NJN-50	"Prokaryotes;Bacteria;Firmicutes - Clostridia;Caproiciproducens"
4580	ova	Oscillibacter valericigenes	"Prokaryotes;Bacteria;Firmicutes - Clostridia;Oscillibacter"
4581	obj	Oscillospiraceae bacterium J115	"Prokaryotes;Bacteria;Firmicutes - Clostridia;Dysosmobacter"
4582	bpb	Butyrivibrio proteoclasticus	"Prokaryotes;Bacteria;Firmicutes - Clostridia;Butyrivibrio"
4583	bfi	Butyrivibrio fibrisolvens	"Prokaryotes;Bacteria;Firmicutes - Clostridia;Butyrivibrio"
4584	bhu	Butyrivibrio hungatei	"Prokaryotes;Bacteria;Firmicutes - Clostridia;Butyrivibrio"
4585	cle	Cellulosilyticum lentocellum	"Prokaryotes;Bacteria;Firmicutes - Clostridia;Cellulosilyticum"
4586	cew	Cellulosilyticum sp. WCF-2	"Prokaryotes;Bacteria;Firmicutes - Clostridia;Cellulosilyticum"
4587	rho	Roseburia hominis	"Prokaryotes;Bacteria;Firmicutes - Clostridia;Roseburia"
4588	rix	Roseburia intestinalis XB6B4	"Prokaryotes;Bacteria;Firmicutes - Clostridia;Roseburia"
4589	rim	Roseburia intestinalis M50/1	"Prokaryotes;Bacteria;Firmicutes - Clostridia;Roseburia"
4590	coo	Coprococcus sp. ART55/1	"Prokaryotes;Bacteria;Firmicutes - Clostridia;Coprococcus"
4591	cct	Coprococcus catus	"Prokaryotes;Bacteria;Firmicutes - Clostridia;Coprococcus"
4592	rob	Blautia obeum	"Prokaryotes;Bacteria;Firmicutes - Clostridia;Blautia"
4593	byl	Blautia sp. YL58	"Prokaryotes;Bacteria;Firmicutes - Clostridia;Blautia"
4594	bhan	Blautia hansenii	"Prokaryotes;Bacteria;Firmicutes - Clostridia;Blautia"
4595	blau	Blautia argi	"Prokaryotes;Bacteria;Firmicutes - Clostridia;Blautia"
4596	bpro	Blautia producta	"Prokaryotes;Bacteria;Firmicutes - Clostridia;Blautia"
4597	blab	Blautia sp. SC05B48	"Prokaryotes;Bacteria;Firmicutes - Clostridia;Blautia"
4598	cpy	Lachnoclostridium phytofermentans	"Prokaryotes;Bacteria;Firmicutes - Clostridia;Lachnoclostridium"
4599	lacy	Lachnoclostridium sp. YL32	"Prokaryotes;Bacteria;Firmicutes - Clostridia;Lachnoclostridium"
4600	csci	Clostridium scindens	"Prokaryotes;Bacteria;Firmicutes - Clostridia;Lachnoclostridium"
4601	csh	Clostridium saccharolyticum WM1	"Prokaryotes;Bacteria;Firmicutes - Clostridia;Lacrimispora"
4602	cso	Clostridium cf. saccharolyticum K10	"Prokaryotes;Bacteria;Firmicutes - Clostridia;Lacrimispora"
4603	bprl	Anaerostipes hadrus	"Prokaryotes;Bacteria;Firmicutes - Clostridia;Anaerostipes"
4604	arf	Anaerostipes rhamnosivorans	"Prokaryotes;Bacteria;Firmicutes - Clostridia;Anaerostipes"
4605	acac	Anaerostipes caccae	"Prokaryotes;Bacteria;Firmicutes - Clostridia;Anaerostipes"
4606	hsd	Herbinix luporum	"Prokaryotes;Bacteria;Firmicutes - Clostridia;Herbinix"
4607	cpro	Anaerotignum propionicum	"Prokaryotes;Bacteria;Firmicutes - Clostridia;Anaerotignum"
4608	lua	Lachnoanaerobaculum umeaense	"Prokaryotes;Bacteria;Firmicutes - Clostridia;Lachnoanaerobaculum"
4609	ehl	Anaerobutyricum hallii	"Prokaryotes;Bacteria;Firmicutes - Clostridia;Anaerobutyricum"
4610	pxv	Pseudobutyrivibrio xylanivorans	"Prokaryotes;Bacteria;Firmicutes - Clostridia;Pseudobutyrivibrio"
4611	anr	Anaerocolumna sedimenticola	"Prokaryotes;Bacteria;Firmicutes - Clostridia;Anaerocolumna"
4612	acel	Anaerocolumna cellulosilytica	"Prokaryotes;Bacteria;Firmicutes - Clostridia;Anaerocolumna"
4613	eel	Eubacterium eligens	"Prokaryotes;Bacteria;Firmicutes - Clostridia;Lachnospira"
4614	rto	Ruminococcus torques	"Prokaryotes;Bacteria;Firmicutes - Clostridia;Mediterraneibacter"
4615	rgn	Ruminococcus gnavus	"Prokaryotes;Bacteria;Firmicutes - Clostridia;Mediterraneibacter"
4616	cbol	Enterocloster bolteae	"Prokaryotes;Bacteria;Firmicutes - Clostridia;Enterocloster"
4617	ere	Eubacterium rectale ATCC 33656	"Prokaryotes;Bacteria;Firmicutes - Clostridia;unclassified Lachnospiraceae"
4618	ert	Eubacterium rectale DSM 17629	"Prokaryotes;Bacteria;Firmicutes - Clostridia;unclassified Lachnospiraceae"
4619	era	Eubacterium rectale M104/1	"Prokaryotes;Bacteria;Firmicutes - Clostridia;unclassified Lachnospiraceae"

4620	lbw	Lachnospiraceae bacterium oral taxon 500	"Prokaryotes;Bacteria;Firmicutes - Clostridia;unclassified Lachnospiraceae"
4621	cdf	Clostridioides difficile 630	"Prokaryotes;Bacteria;Firmicutes - Clostridia;Clostridioides"
4622	pdc	Clostridioides difficile 630	"Prokaryotes;Bacteria;Firmicutes - Clostridia;Clostridioides"
4623	cdc	Clostridioides difficile CD196	"Prokaryotes;Bacteria;Firmicutes - Clostridia;Clostridioides"
4624	cdl	Clostridioides difficile R20291	"Prokaryotes;Bacteria;Firmicutes - Clostridia;Clostridioides"
4625	pdf	Clostridioides difficile 630Derm	"Prokaryotes;Bacteria;Firmicutes - Clostridia;Clostridioides"
4626	eac	Peptoclostridium acidaminophilum	"Prokaryotes;Bacteria;Firmicutes - Clostridia;Peptoclostridium"
4627	cst	Acetoanaerobium sticklandii	"Prokaryotes;Bacteria;Firmicutes - Clostridia;Acetoanaerobium"
4628	faa	Filifactor alocis	"Prokaryotes;Bacteria;Firmicutes - Clostridia;Filifactor"
4629	psor	Paeniclostridium sordellii	"Prokaryotes;Bacteria;Firmicutes - Clostridia;Paeniclostridium"
4630	roc	Romboutsia sp. CE17	"Prokaryotes;Bacteria;Firmicutes - Clostridia;Romboutsia"
4631	phx	Peptacetobacter hiranonis	"Prokaryotes;Bacteria;Firmicutes - Clostridia;Peptacetobacter"
4632	pbq	Peptostreptococcaceae bacterium oral taxon 929	"Prokaryotes;Bacteria;Firmicutes - Clostridia;unclassified Peptostreptococcaceae"
4633	sth	Symbiobacterium thermophilum	"Prokaryotes;Bacteria;Firmicutes - Clostridia;Symbiobacterium"
4634	swo	Syntrophomonas wolfei	"Prokaryotes;Bacteria;Firmicutes - Clostridia;Syntrophomonas"
4635	slp	Syntrophothermus lipocalidus	"Prokaryotes;Bacteria;Firmicutes - Clostridia;Syntrophothermus"
4636	salq	Candidatus Syntrophocurvum alkaliphilum	"Prokaryotes;Bacteria;Firmicutes - Clostridia;Syntrophocurvum"
4637	dsy	Desulfitobacterium hafniense Y51	"Prokaryotes;Bacteria;Firmicutes - Clostridia;Desulfitobacterium"
4638	dhd	Desulfitobacterium hafniense DCB-2	"Prokaryotes;Bacteria;Firmicutes - Clostridia;Desulfitobacterium"
4639	ddh	Desulfitobacterium dehalogenans	"Prokaryotes;Bacteria;Firmicutes - Clostridia;Desulfitobacterium"
4640	ddl	Desulfitobacterium dichloroeliminans	"Prokaryotes;Bacteria;Firmicutes - Clostridia;Desulfitobacterium"
4641	dmt	Desulfitobacterium metallireducens	"Prokaryotes;Bacteria;Firmicutes - Clostridia;Desulfitobacterium"
4642	drm	Desulfotomaculum reducens	"Prokaryotes;Bacteria;Firmicutes - Clostridia;Desulfotomaculum"
4643	dca	Desulfotomaculum nigrificans	"Prokaryotes;Bacteria;Firmicutes - Clostridia;Desulfotomaculum"
4644	dru	Desulfotomaculum ruminis	"Prokaryotes;Bacteria;Firmicutes - Clostridia;Desulfotomaculum"
4645	dfg	Desulfotomaculum ferrireducens	"Prokaryotes;Bacteria;Firmicutes - Clostridia;Desulfotomaculum"
4646	dae	Desulfofarcimen acetoxidans	"Prokaryotes;Bacteria;Firmicutes - Clostridia;Desulfofarcimen"
4647	dku	Desulfofundulus kuznetsovii	"Prokaryotes;Bacteria;Firmicutes - Clostridia;Desulfofundulus"
4648	dgi	Desulfallas gibsoniae	"Prokaryotes;Bacteria;Firmicutes - Clostridia;Desulfallas"
4649	pth	Pelotomaculum thermopropionicum	"Prokaryotes;Bacteria;Firmicutes - Clostridia;Pelotomaculum"
4650	dau	Candidatus Desulforudis audaxviator	"Prokaryotes;Bacteria;Firmicutes - Clostridia;Desulforudis"
4651	tjr	Thermincola potens	"Prokaryotes;Bacteria;Firmicutes - Clostridia;Thermincola"
4652	sgy	Syntrophobotulus glycolicus	"Prokaryotes;Bacteria;Firmicutes - Clostridia;Syntrophobotulus"
4653	dor	Desulfosporosinus orientis	"Prokaryotes;Bacteria;Firmicutes - Clostridia;Desulfosporosinus"
4654	dai	Desulfosporosinus acidiphilus	"Prokaryotes;Bacteria;Firmicutes - Clostridia;Desulfosporosinus"
4655	dmi	Desulfosporosinus meridiei	"Prokaryotes;Bacteria;Firmicutes - Clostridia;Desulfosporosinus"
4656	ded	Dehalobacter sp. DCA	"Prokaryotes;Bacteria;Firmicutes - Clostridia;Dehalobacter"
4657	dec	Dehalobacter sp. CF	"Prokaryotes;Bacteria;Firmicutes - Clostridia;Dehalobacter"
4658	drs	Dehalobacter restrictus	"Prokaryotes;Bacteria;Firmicutes - Clostridia;Dehalobacter"
4659	tfr	Thermoanaerosceptrum fracticalcis	"Prokaryotes;Bacteria;Firmicutes - Clostridia;Thermoanaerosceptrum"
4660	hmo	Heliobacterium modesticaldum	"Prokaryotes;Bacteria;Firmicutes - Clostridia;Heliobacterium"

4661	hcv	Heliorestis convoluta	"Prokaryotes;Bacteria;Firmicutes - Clostridia;Heliorestis"
4662	elm	Eubacterium callanderi	"Prokaryotes;Bacteria;Firmicutes - Clostridia;Eubacterium"
4663	emt	Eubacterium maltosivorans	"Prokaryotes;Bacteria;Firmicutes - Clostridia;Eubacterium"
4664	elim	Eubacterium limosum	"Prokaryotes;Bacteria;Firmicutes - Clostridia;Eubacterium"
4665	awo	Acetobacterium woodii	"Prokaryotes;Bacteria;Firmicutes - Clostridia;Acetobacterium"
4666	tmr	Thermaerobacter marianensis	"Prokaryotes;Bacteria;Firmicutes - Clostridia;Thermaerobacter"
4667	thef	Thermaerobacter sp. FW80	"Prokaryotes;Bacteria;Firmicutes - Clostridia;Thermaerobacter"
4668	thep	Thermaerobacter sp. PB12/4term	"Prokaryotes;Bacteria;Firmicutes - Clostridia;Thermaerobacter"
4669	say	Sulfobacillus acidophilus TPY	"Prokaryotes;Bacteria;Firmicutes - Clostridia;Sulfobacillus"
4670	sap	Sulfobacillus acidophilus DSM 10332	"Prokaryotes;Bacteria;Firmicutes - Clostridia;Sulfobacillus"
4671	sthr	Sulfobacillus thermotolerans	"Prokaryotes;Bacteria;Firmicutes - Clostridia;Sulfobacillus"
4672	cthm	Carboxydocella thermautotrophica	"Prokaryotes;Bacteria;Firmicutes - Clostridia;Carboxydocella"
4673	cmiu	Christensenella minuta	"Prokaryotes;Bacteria;Firmicutes - Clostridia;Christensenella"
4674	ibu	Intestinimonas butyriciproducens	"Prokaryotes;Bacteria;Firmicutes - Clostridia;Intestinimonas"
4675	mdv	Mogibacterium diversum	"Prokaryotes;Bacteria;Firmicutes - Clostridia;Mogibacterium"
4676	amij	Aminipila sp. JN-18	"Prokaryotes;Bacteria;Firmicutes - Clostridia;Aminipila"
4677	amic	Aminipila sp. CBA3637	"Prokaryotes;Bacteria;Firmicutes - Clostridia;Aminipila"
4678	abut	Aminipila butyrica	"Prokaryotes;Bacteria;Firmicutes - Clostridia;Aminipila"
4679	euu	Eubacterium sulci	"Prokaryotes;Bacteria;Firmicutes - Clostridia;unclassified Clostridiales"
4680	bprm	Butyrate-producing bacterium SM4/1	"Prokaryotes;Bacteria;Firmicutes - Clostridia;unclassified Clostridiales"
4681	bprs	Butyrate-producing bacterium SS3/4	"Prokaryotes;Bacteria;Firmicutes - Clostridia;unclassified Clostridiales"
4682	cbar	Petrocella atlantisensis	"Prokaryotes;Bacteria;Firmicutes - Clostridia;unclassified Clostridiales"
4683	tte	Caldanaerobacter subterraneus subsp. tengcongensis	"Prokaryotes;Bacteria;Firmicutes - Clostridia;Caldanaerobacter"
4684	tex	Thermoanaerobacter sp. X514	"Prokaryotes;Bacteria;Firmicutes - Clostridia;Thermoanaerobacter"
4685	thx	Thermoanaerobacter sp. X513	"Prokaryotes;Bacteria;Firmicutes - Clostridia;Thermoanaerobacter"
4686	tpd	Thermoanaerobacter pseudethanolicus	"Prokaryotes;Bacteria;Firmicutes - Clostridia;Thermoanaerobacter"
4687	tit	Thermoanaerobacter italicus	"Prokaryotes;Bacteria;Firmicutes - Clostridia;Thermoanaerobacter"
4688	tmt	Thermoanaerobacter mathranii	"Prokaryotes;Bacteria;Firmicutes - Clostridia;Thermoanaerobacter"
4689	tbo	Thermoanaerobacter brockii	"Prokaryotes;Bacteria;Firmicutes - Clostridia;Thermoanaerobacter"
4690	twi	Thermoanaerobacter wiegelii	"Prokaryotes;Bacteria;Firmicutes - Clostridia;Thermoanaerobacter"
4691	tki	Thermoanaerobacter kivui	"Prokaryotes;Bacteria;Firmicutes - Clostridia;Thermoanaerobacter"
4692	chy	Carboxydothermus hydrogenoformans	"Prokaryotes;Bacteria;Firmicutes - Clostridia;Carboxydothermus"
4693	mta	Moorella thermoacetica ATCC 39073	"Prokaryotes;Bacteria;Firmicutes - Clostridia;Moorella"
4694	mtho	Moorella thermoacetica DSM 521	"Prokaryotes;Bacteria;Firmicutes - Clostridia;Moorella"
4695	mthz	Moorella thermoacetica DSM 2955	"Prokaryotes;Bacteria;Firmicutes - Clostridia;Moorella"
4696	adg	Ammonifex degensii	"Prokaryotes;Bacteria;Firmicutes - Clostridia;Ammonifex"
4697	tpz	Thermacetogenium phaeum	"Prokaryotes;Bacteria;Firmicutes - Clostridia;Thermacetogenium"
4698	csc	Caldicellulosiruptor saccharolyticus	"Prokaryotes;Bacteria;Firmicutes - Clostridia;Caldicellulosiruptor"
4699	ate	Caldicellulosiruptor bescii	"Prokaryotes;Bacteria;Firmicutes - Clostridia;Caldicellulosiruptor"
4700	cob	Caldicellulosiruptor obsidiansis	"Prokaryotes;Bacteria;Firmicutes - Clostridia;Caldicellulosiruptor"
4701	chd	Caldicellulosiruptor hydrothermalis	"Prokaryotes;Bacteria;Firmicutes - Clostridia;Caldicellulosiruptor"

4702	cow	Caldicellulosiruptor owensensis	"Prokaryotes;Bacteria;Firmicutes - Clostridia;Caldicellulosiruptor"
4703	cki	Caldicellulosiruptor kristjanssonii	"Prokaryotes;Bacteria;Firmicutes - Clostridia;Caldicellulosiruptor"
4704	ckn	Caldicellulosiruptor kronotskyensis	"Prokaryotes;Bacteria;Firmicutes - Clostridia;Caldicellulosiruptor"
4705	clc	Caldicellulosiruptor lactoaceticus	"Prokaryotes;Bacteria;Firmicutes - Clostridia;Caldicellulosiruptor"
4706	ccha	Caldicellulosiruptor changbaiensis	"Prokaryotes;Bacteria;Firmicutes - Clostridia;Caldicellulosiruptor"
4707	ttm	Thermoanaerobacterium thermosaccharolyticum DSM 571	"Prokaryotes;Bacteria;Firmicutes - Clostridia;Thermoanaerobacterium"
4708	tto	Thermoanaerobacterium thermosaccharolyticum M0795	"Prokaryotes;Bacteria;Firmicutes - Clostridia;Thermoanaerobacterium"
4709	txy	Thermoanaerobacterium xylanolyticum	"Prokaryotes;Bacteria;Firmicutes - Clostridia;Thermoanaerobacterium"
4710	tsh	Thermoanaerobacterium saccharolyticum	"Prokaryotes;Bacteria;Firmicutes - Clostridia;Thermoanaerobacterium"
4711	tnr	Thermodesulfobium narugense	"Prokaryotes;Bacteria;Firmicutes - Clostridia;Thermodesulfobium"
4712	taci	Thermodesulfobium acidiphilum	"Prokaryotes;Bacteria;Firmicutes - Clostridia;Thermodesulfobium"
4713	mas	Mahella australiensis	"Prokaryotes;Bacteria;Firmicutes - Clostridia;Mahella"
4714	tep	Tepidanaerobacter acetatoxydans Re1	"Prokaryotes;Bacteria;Firmicutes - Clostridia;Tepidanaerobacter"
4715	tae	Tepidanaerobacter acetatoxydans Re1	"Prokaryotes;Bacteria;Firmicutes - Clostridia;Tepidanaerobacter"
4716	toc	Thermosediminibacter oceani	"Prokaryotes;Bacteria;Firmicutes - Clostridia;Thermosediminibacter"
4717	nth	Natranaerobius thermophilus	"Prokaryotes;Bacteria;Firmicutes - Clostridia;Natranaerobius"
4718	hor	Halothermothrix orenii	"Prokaryotes;Bacteria;Firmicutes - Clostridia;Halothermothrix"
4719	has	Halanaerobium hydrogeniformans	"Prokaryotes;Bacteria;Firmicutes - Clostridia;Halanaerobium"
4720	hpk	Halanaerobium praevalens	"Prokaryotes;Bacteria;Firmicutes - Clostridia;Halanaerobium"
4721	hals	Halocella sp. SP3-1	"Prokaryotes;Bacteria;Firmicutes - Clostridia;Halocella"
4722	aar	Acetohalobium arabaticum	"Prokaryotes;Bacteria;Firmicutes - Clostridia;Acetohalobium"
4723	hhl	Halobacteroides halobius	"Prokaryotes;Bacteria;Firmicutes - Clostridia;Halobacteroides"
4724	aft	Anoxybacter fermentans	"Prokaryotes;Bacteria;Firmicutes - Clostridia;Anoxybacter"
4725	fma	Finegoldia magna	"Prokaryotes;Bacteria;Firmicutes - Others;Finegoldia"
4726	apr	Anaerococcus prevotii	"Prokaryotes;Bacteria;Firmicutes - Others;Anaerococcus"
4727	pmic	Parvimonas micra	"Prokaryotes;Bacteria;Firmicutes - Others;Parvimonas"
4728	ped	Peptoniphilus sp. ING2-D1G	"Prokaryotes;Bacteria;Firmicutes - Others;Peptoniphilus"
4729	phar	Peptoniphilus harei	"Prokaryotes;Bacteria;Firmicutes - Others;Peptoniphilus"
4730	piv	Peptoniphilus ivorii	"Prokaryotes;Bacteria;Firmicutes - Others;Peptoniphilus"
4731	cad	Gottschalkia acidurici	"Prokaryotes;Bacteria;Firmicutes - Others;Gottschalkia"
4732	spoa	Tissierellia sp. JN-28	"Prokaryotes;Bacteria;Firmicutes - Others;Sporanaerobacter"
4733	kpar	Keratinibaculum paraultunense	"Prokaryotes;Bacteria;Firmicutes - Others;Keratinibaculum"
4734	vpr	Veillonella parvula	"Prokaryotes;Bacteria;Firmicutes - Others;Veillonella"
4735	vat	Veillonella atypica	"Prokaryotes;Bacteria;Firmicutes - Others;Veillonella"
4736	vrm	Veillonella rodentium	"Prokaryotes;Bacteria;Firmicutes - Others;Veillonella"
4737	vdn	Veillonella dispar	"Prokaryotes;Bacteria;Firmicutes - Others;Veillonella"
4738	vnk	Veillonella nakazawae	"Prokaryotes;Bacteria;Firmicutes - Others;Veillonella"
4739	med	Megasphaera elsdenii	"Prokaryotes;Bacteria;Firmicutes - Others;Megasphaera"
4740	mhw	Megasphaera hexanoica	"Prokaryotes;Bacteria;Firmicutes - Others;Megasphaera"
4741	meg	Megasphaera stantonii	"Prokaryotes;Bacteria;Firmicutes - Others;Megasphaera"
4742	dpn	Dialister pneumosintes	"Prokaryotes;Bacteria;Firmicutes - Others;Dialister"

4743	dho	Dialister hominis	"Prokaryotes;Bacteria;Firmicutes - Others;Dialister"
4744	ssg	Selenomonas sputigena	"Prokaryotes;Bacteria;Firmicutes - Others;Selenomonas"
4745	sri	Selenomonas ruminantium subsp. lactilytica	"Prokaryotes;Bacteria;Firmicutes - Others;Selenomonas"
4746	sele	Selenomonas sp. oral taxon 478	"Prokaryotes;Bacteria;Firmicutes - Others;Selenomonas"
4747	selo	Selenomonas sp. oral taxon 136	"Prokaryotes;Bacteria;Firmicutes - Others;Selenomonas"
4748	selt	Selenomonas sp. oral taxon 920	"Prokaryotes;Bacteria;Firmicutes - Others;Selenomonas"
4749	mhg	Megamonas hypermegale	"Prokaryotes;Bacteria;Firmicutes - Others;Megamonas"
4750	mfun	Megamonas funiformis	"Prokaryotes;Bacteria;Firmicutes - Others;Megamonas"
4751	puf	Pelosinus sp. UFO1	"Prokaryotes;Bacteria;Firmicutes - Others;Pelosinus"
4752	pft	Pelosinus fermentans	"Prokaryotes;Bacteria;Firmicutes - Others;Pelosinus"
4753	mana	Methylomusa anaerophila	"Prokaryotes;Bacteria;Firmicutes - Others;Methylomusa"
4754	sted	Sporomusa termitida	"Prokaryotes;Bacteria;Firmicutes - Others;Sporomusa"
4755	afn	Acidaminococcus fermentans	"Prokaryotes;Bacteria;Firmicutes - Others;Acidaminococcus"
4756	ain	Acidaminococcus intestini	"Prokaryotes;Bacteria;Firmicutes - Others;Acidaminococcus"
4757	pfac	Phascolarctobacterium faecium	"Prokaryotes;Bacteria;Firmicutes - Others;Phascolarctobacterium"
4758	erh	Erysipelothrix rhusiopathiae Fujisawa	"Prokaryotes;Bacteria;Firmicutes - Others;Erysipelothrix"
4759	ers	Erysipelothrix rhusiopathiae SY1027	"Prokaryotes;Bacteria;Firmicutes - Others;Erysipelothrix"
4760	erl	Erysipelothrix larvae	"Prokaryotes;Bacteria;Firmicutes - Others;Erysipelothrix"
4761	eri	Erysipelothrix piscisicarius	"Prokaryotes;Bacteria;Firmicutes - Others;Erysipelothrix"
4762	erd	Erysipelothrix sp. HDW6C	"Prokaryotes;Bacteria;Firmicutes - Others;Erysipelothrix"
4763	eio	Erysipelothrix inopinata	"Prokaryotes;Bacteria;Firmicutes - Others;Erysipelothrix"
4764	euc	Faecalitalea cylindroides	"Prokaryotes;Bacteria;Firmicutes - Others;Faecalitalea"
4765	fro	Faecalibaculum rodentium	"Prokaryotes;Bacteria;Firmicutes - Others;Faecalibaculum"
4766	aarg	Amedibacterium intestinale JCM 30884	"Prokaryotes;Bacteria;Firmicutes - Others;Absiella"
4767	absi	Amedibacterium intestinale 9CBEGH2	"Prokaryotes;Bacteria;Firmicutes - Others;Absiella"
4768	ciu	Clostridium innocuum	"Prokaryotes;Bacteria;Firmicutes - Others;Erysipelatoclostridium"
4769	erm	Erysipelatoclostridium ramosum	"Prokaryotes;Bacteria;Firmicutes - Others;Erysipelatoclostridium"
4770	fit	Faecalibacillus intestinalis	"Prokaryotes;Bacteria;Firmicutes - Others;Faecalibacillus"
4771	ebm	Intestinibaculum porci	"Prokaryotes;Bacteria;Firmicutes - Others;Intestinibaculum"
4772	erb	Erysipelotrichaceae bacterium I46	"Prokaryotes;Bacteria;Firmicutes - Others;unclassified Erysipelotrichaceae"
4773	tur	Turicibacter sp. H121	"Prokaryotes;Bacteria;Firmicutes - Others;Turicibacter"
4774	tsg	Turicibacter sanguinis	"Prokaryotes;Bacteria;Firmicutes - Others;Turicibacter"
4775	lpil	Limnochorda pilosa	"Prokaryotes;Bacteria;Firmicutes - Others;Limnochorda"
4776	mge	Mycoplasma genitalium G37	"Prokaryotes;Bacteria;Tenericutes;Mycoplasma"
4777	mgu	Mycoplasma genitalium M2288	"Prokaryotes;Bacteria;Tenericutes;Mycoplasma"
4778	mgc	Mycoplasma genitalium M2321	"Prokaryotes;Bacteria;Tenericutes;Mycoplasma"
4779	mgq	Mycoplasma genitalium M6282	"Prokaryotes;Bacteria;Tenericutes;Mycoplasma"
4780	mgx	Mycoplasma genitalium M6320	"Prokaryotes;Bacteria;Tenericutes;Mycoplasma"
4781	mpn	Mycoplasma pneumoniae M129	"Prokaryotes;Bacteria;Tenericutes;Mycoplasma"
4782	mpm	Mycoplasma pneumoniae 309	"Prokaryotes;Bacteria;Tenericutes;Mycoplasma"
4783	mpj	Mycoplasma pneumoniae FH	"Prokaryotes;Bacteria;Tenericutes;Mycoplasma"

4784	mpb	Mycoplasma pneumoniae M129-B7	"Prokaryotes;Bacteria;Tenericutes;Mycoplasma"
4785	mpe	Mycoplasma penetrans	"Prokaryotes;Bacteria;Tenericutes;Mycoplasma"
4786	mga	Mycoplasma gallisepticum R(low)	"Prokaryotes;Bacteria;Tenericutes;Mycoplasma"
4787	mgh	Mycoplasma gallisepticum R(high)	"Prokaryotes;Bacteria;Tenericutes;Mycoplasma"
4788	mgf	Mycoplasma gallisepticum F	"Prokaryotes;Bacteria;Tenericutes;Mycoplasma"
4789	mgn	Mycoplasma gallisepticum NC06_2006.080-5-2P	"Prokaryotes;Bacteria;Tenericutes;Mycoplasma"
4790	mgs	Mycoplasma gallisepticum NC95_13295-2-2P	"Prokaryotes;Bacteria;Tenericutes;Mycoplasma"
4791	mgt	Mycoplasma gallisepticum NY01_2001.047-5-1P	"Prokaryotes;Bacteria;Tenericutes;Mycoplasma"
4792	mgv	Mycoplasma gallisepticum VA94_7994-1-7P	"Prokaryotes;Bacteria;Tenericutes;Mycoplasma"
4793	mgw	Mycoplasma gallisepticum WI01_2001.043-13-2P	"Prokaryotes;Bacteria;Tenericutes;Mycoplasma"
4794	mgac	Mycoplasma gallisepticum CA06_2006.052-5-2P	"Prokaryotes;Bacteria;Tenericutes;Mycoplasma"
4795	mgan	Mycoplasma gallisepticum NC08_2008.031-4-3P	"Prokaryotes;Bacteria;Tenericutes;Mycoplasma"
4796	mgnc	Mycoplasma gallisepticum NC96_1596-4-2P	"Prokaryotes;Bacteria;Tenericutes;Mycoplasma"
4797	mgz	Mycoplasma gallisepticum S6	"Prokaryotes;Bacteria;Tenericutes;Mycoplasma"
4798	mmy	Mycoplasma mycoides subsp. mycoides SC PG1	"Prokaryotes;Bacteria;Tenericutes;Mycoplasma"
4799	mmym	Mycoplasma mycoides subsp. mycoides SC Gladysdale	"Prokaryotes;Bacteria;Tenericutes;Mycoplasma"
4800	mmyi	Mycoplasma mycoides subsp. mycoides izsam_mm5713	"Prokaryotes;Bacteria;Tenericutes;Mycoplasma"
4801	mml	Mycoplasma mycoides subsp. capri LC 95010	"Prokaryotes;Bacteria;Tenericutes;Mycoplasma"
4802	mcp	Mycoplasma capricolum subsp. capricolum ATCC 27343	"Prokaryotes;Bacteria;Tenericutes;Mycoplasma"
4803	mcac	Mycoplasma capricolum subsp. capripneumoniae 9231-Abomsa	"Prokaryotes;Bacteria;Tenericutes;Mycoplasma"
4804	mcap	Mycoplasma capricolum subsp. capripneumoniae F38	"Prokaryotes;Bacteria;Tenericutes;Mycoplasma"
4805	mcar	Mycoplasma capricolum subsp. capripneumoniae ILRI181	"Prokaryotes;Bacteria;Tenericutes;Mycoplasma"
4806	mcai	Mycoplasma capricolum subsp. capripneumoniae 87001	"Prokaryotes;Bacteria;Tenericutes;Mycoplasma"
4807	mlc	Mycoplasma leachii PG50	"Prokaryotes;Bacteria;Tenericutes;Mycoplasma"
4808	mlh	Mycoplasma leachii 99/014/6	"Prokaryotes;Bacteria;Tenericutes;Mycoplasma"
4809	mmo	Mycoplasma mobile	"Prokaryotes;Bacteria;Tenericutes;Mycoplasma"
4810	mhy	Mycoplasma hyopneumoniae 232	"Prokaryotes;Bacteria;Tenericutes;Mycoplasma"
4811	mhj	Mycoplasma hyopneumoniae J	"Prokaryotes;Bacteria;Tenericutes;Mycoplasma"
4812	mhp	Mycoplasma hyopneumoniae 7448	"Prokaryotes;Bacteria;Tenericutes;Mycoplasma"
4813	mhn	Mycoplasma hyopneumoniae 168	"Prokaryotes;Bacteria;Tenericutes;Mycoplasma"
4814	mhyl	Mycoplasma hyopneumoniae 168-L	"Prokaryotes;Bacteria;Tenericutes;Mycoplasma"
4815	mhyo	Mycoplasma hyopneumoniae 7422	"Prokaryotes;Bacteria;Tenericutes;Mycoplasma"
4816	mat	Mycoplasma arthritidis	"Prokaryotes;Bacteria;Tenericutes;Mycoplasma"
4817	mco	Mycoplasma conjunctivae	"Prokaryotes;Bacteria;Tenericutes;Mycoplasma"
4818	mho	Mycoplasma hominis ATCC 23114	"Prokaryotes;Bacteria;Tenericutes;Mycoplasma"
4819	mhom	Mycoplasma hominis ATCC 27545	"Prokaryotes;Bacteria;Tenericutes;Mycoplasma"
4820	mcd	Mycoplasma crocodyli	"Prokaryotes;Bacteria;Tenericutes;Mycoplasma"
4821	mhr	Mycoplasma hyorhinis HUB-1	"Prokaryotes;Bacteria;Tenericutes;Mycoplasma"
4822	mhh	Mycoplasma hyorhinis GDL-1	"Prokaryotes;Bacteria;Tenericutes;Mycoplasma"
4823	mhm	Mycoplasma hyorhinis MCLD	"Prokaryotes;Bacteria;Tenericutes;Mycoplasma"
4824	mhs	Mycoplasma hyorhinis SK76	"Prokaryotes;Bacteria;Tenericutes;Mycoplasma"

4825	mhv	Mycoplasma hyorhinis DBS 1050	"Prokaryotes;Bacteria;Tenericutes;Mycoplasma"
4826	mha	Mycoplasma haemofelis Langford 1	"Prokaryotes;Bacteria;Tenericutes;Mycoplasma"
4827	mhf	Mycoplasma haemofelis Ohio2	"Prokaryotes;Bacteria;Tenericutes;Mycoplasma"
4828	mss	Mycoplasma suis Illinois	"Prokaryotes;Bacteria;Tenericutes;Mycoplasma"
4829	msk	Mycoplasma suis KI3806	"Prokaryotes;Bacteria;Tenericutes;Mycoplasma"
4830	mpf	Mycoplasma putrefaciens KS1	"Prokaryotes;Bacteria;Tenericutes;Mycoplasma"
4831	mput	Mycoplasma putrefaciens Mput9231	"Prokaryotes;Bacteria;Tenericutes;Mycoplasma"
4832	mhe	Mycoplasma haemocanis	"Prokaryotes;Bacteria;Tenericutes;Mycoplasma"
4833	mwe	Mycoplasma wenyonii	"Prokaryotes;Bacteria;Tenericutes;Mycoplasma"
4834	mhl	Candidatus Mycoplasma haemolamae	"Prokaryotes;Bacteria;Tenericutes;Mycoplasma"
4835	mhb	Candidatus Mycoplasma haemominutum	"Prokaryotes;Bacteria;Tenericutes;Mycoplasma"
4836	mpv	Mycoplasma parvum	"Prokaryotes;Bacteria;Tenericutes;Mycoplasma"
4837	mov	Mycoplasma ovis	"Prokaryotes;Bacteria;Tenericutes;Mycoplasma"
4838	mbc	Mycoplasma bovoculi	"Prokaryotes;Bacteria;Tenericutes;Mycoplasma"
4839	mgj	Candidatus Mycoplasma girerdii	"Prokaryotes;Bacteria;Tenericutes;Mycoplasma"
4840	mfq	Mycoplasma flocculare	"Prokaryotes;Bacteria;Tenericutes;Mycoplasma"
4841	mcan	Mycoplasma canadense	"Prokaryotes;Bacteria;Tenericutes;Mycoplasma"
4842	myt	Mycoplasma yeatsii	"Prokaryotes;Bacteria;Tenericutes;Mycoplasma"
4843	mds	Mycoplasma dispar	"Prokaryotes;Bacteria;Tenericutes;Mycoplasma"
4844	myg	Mycoplasma sp. (ex Biomphalaria glabrata)	"Prokaryotes;Bacteria;Tenericutes;Mycoplasma"
4845	mpho	Mycoplasma phocidae	"Prokaryotes;Bacteria;Tenericutes;Mycoplasma"
4846	mhyv	Mycoplasma hyosynoviae	"Prokaryotes;Bacteria;Tenericutes;Mycoplasma"
4847	mclo	Mycoplasma cloacale	"Prokaryotes;Bacteria;Tenericutes;Mycoplasma"
4848	mamp	Mycoplasma amphoriforme	"Prokaryotes;Bacteria;Tenericutes;Mycoplasma"
4849	mans	Mycoplasma anserisalpingitidis	"Prokaryotes;Bacteria;Tenericutes;Mycoplasma"
4850	mphc	Mycoplasma phocicerebrale	"Prokaryotes;Bacteria;Tenericutes;Mycoplasma"
4851	miw	Mycoplasma iowae	"Prokaryotes;Bacteria;Tenericutes;Mycoplasma"
4852	mane	Mycoplasma anseris	"Prokaryotes;Bacteria;Tenericutes;Mycoplasma"
4853	mnh	Mycoplasma nasistruthionis	"Prokaryotes;Bacteria;Tenericutes;Mycoplasma"
4854	mnu	Mycoplasma neurolyticum	"Prokaryotes;Bacteria;Tenericutes;Mycoplasma"
4855	mstr	Mycoplasma struthionis	"Prokaryotes;Bacteria;Tenericutes;Mycoplasma"
4856	mcr	Mycoplasmopsis californica	"Prokaryotes;Bacteria;Tenericutes;Mycoplasmopsis"
4857	mcm	Mycoplasmopsis californicum	"Prokaryotes;Bacteria;Tenericutes;Mycoplasmopsis"
4858	mgb	Mycoplasmopsis gallinacea	"Prokaryotes;Bacteria;Tenericutes;Mycoplasmopsis"
4859	mgly	Mycoplasma glycophilum	"Prokaryotes;Bacteria;Tenericutes;Mycoplasmopsis"
4860	mcou	Mycoplasma columborale	"Prokaryotes;Bacteria;Tenericutes;Mycoplasmopsis"
4861	mcom	Mycoplasma columbinum	"Prokaryotes;Bacteria;Tenericutes;Mycoplasmopsis"
4862	mpu	Mycoplasma pulmonis	"Prokaryotes;Bacteria;Tenericutes;Mycoplasmopsis"
4863	msy	Mycoplasma synoviae 53	"Prokaryotes;Bacteria;Tenericutes;Mycoplasmopsis"
4864	mso	Mycoplasma synoviae ATCC 25204	"Prokaryotes;Bacteria;Tenericutes;Mycoplasmopsis"
4865	maa	Mycoplasma agalactiae PG2	"Prokaryotes;Bacteria;Tenericutes;Mycoplasmopsis"

4866	mal	Mycoplasma agalactiae 5632	"Prokaryotes;Bacteria;Tenericutes;Mycoplasmopsis"
4867	mfr	Mycoplasma fermentans JER	"Prokaryotes;Bacteria;Tenericutes;Mycoplasmopsis"
4868	mfm	Mycoplasma fermentans M64	"Prokaryotes;Bacteria;Tenericutes;Mycoplasmopsis"
4869	mfp	Mycoplasma fermentans PG18	"Prokaryotes;Bacteria;Tenericutes;Mycoplasmopsis"
4870	mbv	Mycoplasma bovis PG45	"Prokaryotes;Bacteria;Tenericutes;Mycoplasmopsis"
4871	mbh	Mycoplasma bovis Hubei-1	"Prokaryotes;Bacteria;Tenericutes;Mycoplasmopsis"
4872	mbi	Mycoplasma bovis HB0801	"Prokaryotes;Bacteria;Tenericutes;Mycoplasmopsis"
4873	mbq	Mycoplasma bovis CQ-W70	"Prokaryotes;Bacteria;Tenericutes;Mycoplasmopsis"
4874	mcy	Mycoplasma cynos	"Prokaryotes;Bacteria;Tenericutes;Mycoplasmopsis"
4875	mcas	Mycoplasma canis LV	"Prokaryotes;Bacteria;Tenericutes;Mycoplasmopsis"
4876	mck	Mycoplasma canis PG 14	"Prokaryotes;Bacteria;Tenericutes;Mycoplasmopsis"
4877	marg	Mycoplasma arginini	"Prokaryotes;Bacteria;Tenericutes;Mycoplasmopsis"
4878	mpul	Mycoplasma pullorum	"Prokaryotes;Bacteria;Tenericutes;Mycoplasmopsis"
4879	mbov	Mycoplasma bovigenitalium	"Prokaryotes;Bacteria;Tenericutes;Mycoplasmopsis"
4880	mboh	Mycoplasma bovirhinis	"Prokaryotes;Bacteria;Tenericutes;Mycoplasmopsis"
4881	mani	Mycoplasma anatis	"Prokaryotes;Bacteria;Tenericutes;Mycoplasmopsis"
4882	mphi	Mycoplasma phocirhinis	"Prokaryotes;Bacteria;Tenericutes;Mycoplasmopsis"
4883	uur	Ureaplasma parvum serovar 3 ATCC 700970	"Prokaryotes;Bacteria;Tenericutes;Ureaplasma"
4884	upa	Ureaplasma parvum serovar 3 ATCC 27815	"Prokaryotes;Bacteria;Tenericutes;Ureaplasma"
4885	upr	Ureaplasma parvum serovar 3 SV3F4	"Prokaryotes;Bacteria;Tenericutes;Ureaplasma"
4886	uue	Ureaplasma urealyticum	"Prokaryotes;Bacteria;Tenericutes;Ureaplasma"
4887	hcr	Candidatus Hepatoplasma crinochetorum	"Prokaryotes;Bacteria;Tenericutes;Hepatoplasma"
4888	poy	Onion yellows phytoplasma OY-M	"Prokaryotes;Bacteria;Tenericutes;Phytoplasma"
4889	ayw	Aster yellows witches'-broom phytoplasma AYWB	"Prokaryotes;Bacteria;Tenericutes;Phytoplasma"
4890	mbp	Maize bushy stunt phytoplasma	"Prokaryotes;Bacteria;Tenericutes;Phytoplasma"
4891	pml	Candidatus Phytoplasma mali	"Prokaryotes;Bacteria;Tenericutes;Phytoplasma"
4892	pal	Candidatus Phytoplasma australiense	"Prokaryotes;Bacteria;Tenericutes;Phytoplasma"
4893	nzs	Strawberry lethal yellows phytoplasma (CPA) NZSb11	"Prokaryotes;Bacteria;Tenericutes;Phytoplasma"
4894	psol	Candidatus Phytoplasma solani	"Prokaryotes;Bacteria;Tenericutes;Phytoplasma"
4895	pzi	Candidatus Phytoplasma ziziphi	"Prokaryotes;Bacteria;Tenericutes;Phytoplasma"
4896	acl	Acholeplasma laidlawii	"Prokaryotes;Bacteria;Tenericutes;Acholeplasma"
4897	abra	Acholeplasma brassicae	"Prokaryotes;Bacteria;Tenericutes;Acholeplasma"
4898	apal	Acholeplasma palmae	"Prokaryotes;Bacteria;Tenericutes;Acholeplasma"
4899	aoc	Acholeplasma oculi	"Prokaryotes;Bacteria;Tenericutes;Acholeplasma"
4900	aaxa	Acholeplasma axanthum	"Prokaryotes;Bacteria;Tenericutes;Acholeplasma"
4901	ahk	Acholeplasma hippikon	"Prokaryotes;Bacteria;Tenericutes;Acholeplasma"
4902	mfl	Mesoplasma florum L1	"Prokaryotes;Bacteria;Tenericutes;Mesoplasma"
4903	mfw	Mesoplasma florum W37	"Prokaryotes;Bacteria;Tenericutes;Mesoplasma"
4904	mchc	Mesoplasma chauliocola	"Prokaryotes;Bacteria;Tenericutes;Mesoplasma"
4905	mlac	Mesoplasma lactucae	"Prokaryotes;Bacteria;Tenericutes;Mesoplasma"
4906	ment	Mesoplasma entomophilum	"Prokaryotes;Bacteria;Tenericutes;Mesoplasma"

4907	msyr	Mesoplasma syrphidae	"Prokaryotes;Bacteria;Tenericutes;Mesoplasma"
4908	mtab	Mesoplasma tabanidae	"Prokaryotes;Bacteria;Tenericutes;Mesoplasma"
4909	mcol	Mesoplasma coleopterae	"Prokaryotes;Bacteria;Tenericutes;Mesoplasma"
4910	elj	Entomoplasma luminosum	"Prokaryotes;Bacteria;Tenericutes;Entomoplasma"
4911	esx	Entomoplasma somnilux	"Prokaryotes;Bacteria;Tenericutes;Entomoplasma"
4912	efr	Entomoplasma freundtii	"Prokaryotes;Bacteria;Tenericutes;Entomoplasma"
4913	eml	Entomoplasma melaleucae	"Prokaryotes;Bacteria;Tenericutes;Entomoplasma"
4914	scr	Spiroplasma chrysopicola	"Prokaryotes;Bacteria;Tenericutes;Spiroplasma"
4915	ssyr	Spiroplasma syrphidicola	"Prokaryotes;Bacteria;Tenericutes;Spiroplasma"
4916	sdi	Spiroplasma diminutum	"Prokaryotes;Bacteria;Tenericutes;Spiroplasma"
4917	stai	Spiroplasma taiwanense	"Prokaryotes;Bacteria;Tenericutes;Spiroplasma"
4918	sapi	Spiroplasma apis	"Prokaryotes;Bacteria;Tenericutes;Spiroplasma"
4919	smir	Spiroplasma mirum ATCC 29335	"Prokaryotes;Bacteria;Tenericutes;Spiroplasma"
4920	smia	Spiroplasma mirum ATCC 29335 SMCA	"Prokaryotes;Bacteria;Tenericutes;Spiroplasma"
4921	scq	Spiroplasma culicicola	"Prokaryotes;Bacteria;Tenericutes;Spiroplasma"
4922	ssab	Spiroplasma sabaudiense	"Prokaryotes;Bacteria;Tenericutes;Spiroplasma"
4923	satr	Spiroplasma atrichopogonis	"Prokaryotes;Bacteria;Tenericutes;Spiroplasma"
4924	seri	Spiroplasma eriocheiris	"Prokaryotes;Bacteria;Tenericutes;Spiroplasma"
4925	stur	Spiroplasma turonicum	"Prokaryotes;Bacteria;Tenericutes;Spiroplasma"
4926	sll	Spiroplasma litorale	"Prokaryotes;Bacteria;Tenericutes;Spiroplasma"
4927	skn	Spiroplasma kunkelii	"Prokaryotes;Bacteria;Tenericutes;Spiroplasma"
4928	scj	Spiroplasma cantharicola	"Prokaryotes;Bacteria;Tenericutes;Spiroplasma"
4929	shj	Spiroplasma helicoides	"Prokaryotes;Bacteria;Tenericutes;Spiroplasma"
4930	sck	Spiroplasma citri	"Prokaryotes;Bacteria;Tenericutes;Spiroplasma"
4931	sfz	Spiroplasma floricola	"Prokaryotes;Bacteria;Tenericutes;Spiroplasma"
4932	scou	Spiroplasma corruscae	"Prokaryotes;Bacteria;Tenericutes;Spiroplasma"
4933	scla	Spiroplasma clarkii	"Prokaryotes;Bacteria;Tenericutes;Spiroplasma"
4934	sprn	Spiroplasma sp. NBRC 100390	"Prokaryotes;Bacteria;Tenericutes;Spiroplasma"
4935	spit	Spiroplasma sp. TU-14	"Prokaryotes;Bacteria;Tenericutes;Spiroplasma"
4936	stab	Spiroplasma tabanidicola	"Prokaryotes;Bacteria;Tenericutes;Spiroplasma"
4937	sphh	Spiroplasma phoeniceum	"Prokaryotes;Bacteria;Tenericutes;Spiroplasma"
4938	smoo	Spiroplasma monobiae	"Prokaryotes;Bacteria;Tenericutes;Spiroplasma"
4939	salx	Spiroplasma alleghenense	"Prokaryotes;Bacteria;Tenericutes;Spiroplasma"
4940	sgq	Spiroplasma gladiatoris	"Prokaryotes;Bacteria;Tenericutes;Spiroplasma"
4941	schi	Spiroplasma chinense	"Prokaryotes;Bacteria;Tenericutes;Spiroplasma"
4942	mbj	Candidatus Izimaplasma sp. HR1	"Prokaryotes;Bacteria;Tenericutes;Izimaplasma"
4943	tbm	Tenericutes bacterium MO-XQ	"Prokaryotes;Bacteria;Tenericutes;unclassified Tenericutes"
4944	tbz	Tenericutes bacterium MZ-XQ	"Prokaryotes;Bacteria;Tenericutes;unclassified Tenericutes"
4945	mtu	Mycobacterium tuberculosis H37Rv	"Prokaryotes;Bacteria;Actinobacteria;Mycobacterium"
4946	mtv	Mycobacterium tuberculosis H37Rv	"Prokaryotes;Bacteria;Actinobacteria;Mycobacterium"
4947	mtc	Mycobacterium tuberculosis CDC1551	"Prokaryotes;Bacteria;Actinobacteria;Mycobacterium"

4948	mra	Mycobacterium tuberculosis H37Ra	"Prokaryotes;Bacteria;Actinobacteria;Mycobacterium"
4949	mtf	Mycobacterium tuberculosis F11	"Prokaryotes;Bacteria;Actinobacteria;Mycobacterium"
4950	mtb	Mycobacterium tuberculosis KZN 1435	"Prokaryotes;Bacteria;Actinobacteria;Mycobacterium"
4951	mtk	Mycobacterium tuberculosis KZN 4207	"Prokaryotes;Bacteria;Actinobacteria;Mycobacterium"
4952	mtz	Mycobacterium tuberculosis KZN 605	"Prokaryotes;Bacteria;Actinobacteria;Mycobacterium"
4953	mtg	Mycobacterium tuberculosis RGTB327	"Prokaryotes;Bacteria;Actinobacteria;Mycobacterium"
4954	mti	Mycobacterium tuberculosis RGTB423	"Prokaryotes;Bacteria;Actinobacteria;Mycobacterium"
4955	mte	Mycobacterium tuberculosis CCDC5079	"Prokaryotes;Bacteria;Actinobacteria;Mycobacterium"
4956	mtur	Mycobacterium tuberculosis CCDC5079	"Prokaryotes;Bacteria;Actinobacteria;Mycobacterium"
4957	mtl	Mycobacterium tuberculosis CCDC5180	"Prokaryotes;Bacteria;Actinobacteria;Mycobacterium"
4958	mto	Mycobacterium tuberculosis CTRI-2	"Prokaryotes;Bacteria;Actinobacteria;Mycobacterium"
4959	mtd	Mycobacterium tuberculosis UT205	"Prokaryotes;Bacteria;Actinobacteria;Mycobacterium"
4960	mtn	Mycobacterium tuberculosis Erdman = ATCC 35801	"Prokaryotes;Bacteria;Actinobacteria;Mycobacterium"
4961	mtj	Mycobacterium tuberculosis Beijing/NITR203	"Prokaryotes;Bacteria;Actinobacteria;Mycobacterium"
4962	mtub	Mycobacterium tuberculosis 7199-99	"Prokaryotes;Bacteria;Actinobacteria;Mycobacterium"
4963	mtuc	Mycobacterium tuberculosis CAS/NITR204	"Prokaryotes;Bacteria;Actinobacteria;Mycobacterium"
4964	mtue	Mycobacterium tuberculosis EAI5/NITR206	"Prokaryotes;Bacteria;Actinobacteria;Mycobacterium"
4965	mtx	Mycobacterium tuberculosis EAI5	"Prokaryotes;Bacteria;Actinobacteria;Mycobacterium"
4966	mtuh	Mycobacterium tuberculosis Haarlem/NITR202	"Prokaryotes;Bacteria;Actinobacteria;Mycobacterium"
4967	mtul	Mycobacterium tuberculosis Haarlem	"Prokaryotes;Bacteria;Actinobacteria;Mycobacterium"
4968	mtut	Mycobacterium tuberculosis BT1	"Prokaryotes;Bacteria;Actinobacteria;Mycobacterium"
4969	mtuu	Mycobacterium tuberculosis BT2	"Prokaryotes;Bacteria;Actinobacteria;Mycobacterium"
4970	mtq	Mycobacterium tuberculosis HKBS1	"Prokaryotes;Bacteria;Actinobacteria;Mycobacterium"
4971	mbo	Mycobacterium tuberculosis variant bovis AF2122/97	"Prokaryotes;Bacteria;Actinobacteria;Mycobacterium"
4972	mbb	Mycobacterium tuberculosis variant bovis BCG Pasteur 1173P2	"Prokaryotes;Bacteria;Actinobacteria;Mycobacterium"
4973	mbt	Mycobacterium tuberculosis variant bovis BCG Tokyo 172	"Prokaryotes;Bacteria;Actinobacteria;Mycobacterium"
4974	mbm	Mycobacterium tuberculosis variant bovis BCG Mexico	"Prokaryotes;Bacteria;Actinobacteria;Mycobacterium"
4975	mbk	Mycobacterium tuberculosis variant bovis BCG Korea 1168P	"Prokaryotes;Bacteria;Actinobacteria;Mycobacterium"
4976	mbx	Mycobacterium tuberculosis variant bovis BCG ATCC 35743	"Prokaryotes;Bacteria;Actinobacteria;Mycobacterium"
4977	maf	Mycobacterium tuberculosis variant africanum	"Prokaryotes;Bacteria;Actinobacteria;Mycobacterium"
4978	mmic	Mycobacterium tuberculosis variant microti	"Prokaryotes;Bacteria;Actinobacteria;Mycobacterium"
4979	mce	Mycobacterium canettii CIPT 140010059	"Prokaryotes;Bacteria;Actinobacteria;Mycobacterium"
4980	mcq	Mycobacterium canettii CIPT 140060008	"Prokaryotes;Bacteria;Actinobacteria;Mycobacterium"
4981	mcv	Mycobacterium canettii CIPT 140070008	"Prokaryotes;Bacteria;Actinobacteria;Mycobacterium"
4982	mcx	Mycobacterium canettii CIPT 140070010	"Prokaryotes;Bacteria;Actinobacteria;Mycobacterium"
4983	mcz	Mycobacterium canettii CIPT 140070017	"Prokaryotes;Bacteria;Actinobacteria;Mycobacterium"
4984	mle	Mycobacterium leprae TN	"Prokaryotes;Bacteria;Actinobacteria;Mycobacterium"
4985	mlb	Mycobacterium leprae Br4923	"Prokaryotes;Bacteria;Actinobacteria;Mycobacterium"
4986	mpa	Mycobacterium avium subsp. paratuberculosis K-10	"Prokaryotes;Bacteria;Actinobacteria;Mycobacterium"
4987	mao	Mycobacterium avium subsp. paratuberculosis MAP4	"Prokaryotes;Bacteria;Actinobacteria;Mycobacterium"
4988	mavi	Mycobacterium avium subsp. paratuberculosis E1	"Prokaryotes;Bacteria;Actinobacteria;Mycobacterium"

4989	mavu	Mycobacterium avium subsp. paratuberculosis E93	"Prokaryotes;Bacteria;Actinobacteria;Mycobacterium"
4990	mav	Mycobacterium avium 104	"Prokaryotes;Bacteria;Actinobacteria;Mycobacterium"
4991	mit	Mycobacterium intracellulare MOTT-02	"Prokaryotes;Bacteria;Actinobacteria;Mycobacterium"
4992	mia	Mycobacterium intracellulare ATCC 13950	"Prokaryotes;Bacteria;Actinobacteria;Mycobacterium"
4993	mid	Mycobacterium intracellulare subsp. intracellulare	"Prokaryotes;Bacteria;Actinobacteria;Mycobacterium"
4994	myo	Mycobacterium intracellulare subsp. yongonense	"Prokaryotes;Bacteria;Actinobacteria;Mycobacterium"
4995	mchi	Mycobacterium intracellulare subsp. chimaera	"Prokaryotes;Bacteria;Actinobacteria;Mycobacterium"
4996	mir	Mycobacterium paraintracellulare	"Prokaryotes;Bacteria;Actinobacteria;Mycobacterium"
4997	mmal	Mycobacterium marseillense	"Prokaryotes;Bacteria;Actinobacteria;Mycobacterium"
4998	mlp	Mycobacterium lepraemurium	"Prokaryotes;Bacteria;Actinobacteria;Mycobacterium"
4999	msa	Mycobacterium sp. JS623	"Prokaryotes;Bacteria;Actinobacteria;Mycobacterium"
5000	mul	Mycobacterium ulcerans	"Prokaryotes;Bacteria;Actinobacteria;Mycobacterium"
5001	mmc	Mycobacterium sp. MCS	"Prokaryotes;Bacteria;Actinobacteria;Mycobacterium"
5002	mkm	Mycobacterium sp. KMS	"Prokaryotes;Bacteria;Actinobacteria;Mycobacterium"
5003	mjl	Mycobacterium sp. JLS	"Prokaryotes;Bacteria;Actinobacteria;Mycobacterium"
5004	mmi	Mycobacterium marinum M	"Prokaryotes;Bacteria;Actinobacteria;Mycobacterium"
5005	mmae	Mycobacterium marinum E11	"Prokaryotes;Bacteria;Actinobacteria;Mycobacterium"
5006	mmm	Mycobacterium sp. MOTT36Y	"Prokaryotes;Bacteria;Actinobacteria;Mycobacterium"
5007	mli	Mycobacterium liflandii	"Prokaryotes;Bacteria;Actinobacteria;Mycobacterium"
5008	mkn	Mycobacterium kansasii ATCC 12478	"Prokaryotes;Bacteria;Actinobacteria;Mycobacterium"
5009	myv	Mycobacterium sp. VKM Ac-1817D	"Prokaryotes;Bacteria;Actinobacteria;Mycobacterium"
5010	mye	Mycobacterium sp. EPa45	"Prokaryotes;Bacteria;Actinobacteria;Mycobacterium"
5011	mhad	Mycobacterium haemophilum	"Prokaryotes;Bacteria;Actinobacteria;Mycobacterium"
5012	mdx	Mycobacterium dioxanotrophicus	"Prokaryotes;Bacteria;Actinobacteria;Mycobacterium"
5013	mshg	Mycobacterium shigaense	"Prokaryotes;Bacteria;Actinobacteria;Mycobacterium"
5014	mfj	Mycobacterium florentinum	"Prokaryotes;Bacteria;Actinobacteria;Mycobacterium"
5015	mgro	Mycobacterium grossiae	"Prokaryotes;Bacteria;Actinobacteria;Mycobacterium"
5016	mxe	Mycobacterium xenopi	"Prokaryotes;Bacteria;Actinobacteria;Mycobacterium"
5017	mnv	Mycobacterium noviomagense	"Prokaryotes;Bacteria;Actinobacteria;Mycobacterium"
5018	mpag	Mycobacterium paragordonae	"Prokaryotes;Bacteria;Actinobacteria;Mycobacterium"
5019	mnm	Mycobacterium novum	"Prokaryotes;Bacteria;Actinobacteria;Mycobacterium"
5020	mgor	Mycobacterium gordonae	"Prokaryotes;Bacteria;Actinobacteria;Mycobacterium"
5021	mcoo	Mycobacterium cookii	"Prokaryotes;Bacteria;Actinobacteria;Mycobacterium"
5022	msm	Mycolicibacterium smegmatis MC2 155	"Prokaryotes;Bacteria;Actinobacteria;Mycolicibacterium"
5023	msg	Mycolicibacterium smegmatis MC2 155	"Prokaryotes;Bacteria;Actinobacteria;Mycolicibacterium"
5024	msb	Mycolicibacterium smegmatis MC2 155	"Prokaryotes;Bacteria;Actinobacteria;Mycolicibacterium"
5025	msn	Mycolicibacterium smegmatis INHR1	"Prokaryotes;Bacteria;Actinobacteria;Mycolicibacterium"
5026	msh	Mycolicibacterium smegmatis INHR2	"Prokaryotes;Bacteria;Actinobacteria;Mycolicibacterium"
5027	mva	Mycolicibacterium vanbaalenii	"Prokaryotes;Bacteria;Actinobacteria;Mycolicibacterium"
5028	mgi	Mycolicibacterium gilvum PYR-GCK	"Prokaryotes;Bacteria;Actinobacteria;Mycolicibacterium"
5029	msp	Mycolicibacterium gilvum Spyr1	"Prokaryotes;Bacteria;Actinobacteria;Mycolicibacterium"

5030	mcb	Mycolicibacterium chubuense	"Prokaryotes;Bacteria;Actinobacteria;Mycolicibacterium"
5031	mne	Mycolicibacterium neoaurum VKM Ac-1815D	"Prokaryotes;Bacteria;Actinobacteria;Mycolicibacterium"
5032	myn	Mycolicibacterium neoaurum NRRL B-3805	"Prokaryotes;Bacteria;Actinobacteria;Mycolicibacterium"
5033	mgo	Mycolicibacterium goodii	"Prokaryotes;Bacteria;Actinobacteria;Mycolicibacterium"
5034	mft	Mycolicibacterium fortuitum	"Prokaryotes;Bacteria;Actinobacteria;Mycolicibacterium"
5035	mphl	Mycolicibacterium phlei	"Prokaryotes;Bacteria;Actinobacteria;Mycolicibacterium"
5036	mvq	Mycolicibacterium vaccae	"Prokaryotes;Bacteria;Actinobacteria;Mycolicibacterium"
5037	mll	Mycolicibacterium litorale	"Prokaryotes;Bacteria;Actinobacteria;Mycolicibacterium"
5038	mrh	Mycolicibacterium rhodesiae	"Prokaryotes;Bacteria;Actinobacteria;Mycolicibacterium"
5039	mthn	Mycolicibacterium thermoresistibile	"Prokaryotes;Bacteria;Actinobacteria;Mycolicibacterium"
5040	mhas	Mycolicibacterium hassiacum	"Prokaryotes;Bacteria;Actinobacteria;Mycolicibacterium"
5041	mdu	Mycolicibacterium duvalii	"Prokaryotes;Bacteria;Actinobacteria;Mycolicibacterium"
5042	mcht	Mycolicibacterium chitae	"Prokaryotes;Bacteria;Actinobacteria;Mycolicibacterium"
5043	mdr	Mycolicibacterium doricum	"Prokaryotes;Bacteria;Actinobacteria;Mycolicibacterium"
5044	mauu	Mycolicibacterium aurum	"Prokaryotes;Bacteria;Actinobacteria;Mycolicibacterium"
5045	mmag	Mycolicibacterium madagascariense	"Prokaryotes;Bacteria;Actinobacteria;Mycolicibacterium"
5046	mmor	Mycolicibacterium moriokaense	"Prokaryotes;Bacteria;Actinobacteria;Mycolicibacterium"
5047	mfx	Mycolicibacterium fallax	"Prokaryotes;Bacteria;Actinobacteria;Mycolicibacterium"
5048	maic	Mycolicibacterium aichiense	"Prokaryotes;Bacteria;Actinobacteria;Mycolicibacterium"
5049	mij	Mycolicibacterium insubricum	"Prokaryotes;Bacteria;Actinobacteria;Mycolicibacterium"
5050	malv	Mycolicibacterium alvei	"Prokaryotes;Bacteria;Actinobacteria;Mycolicibacterium"
5051	mty	Mycolicibacterium tokaiense	"Prokaryotes;Bacteria;Actinobacteria;Mycolicibacterium"
5052	mpsc	Mycolicibacterium psychrotolerans	"Prokaryotes;Bacteria;Actinobacteria;Mycolicibacterium"
5053	mab	Mycobacteroides abscessus ATCC 19977	"Prokaryotes;Bacteria;Actinobacteria;Mycobacteroides"
5054	mmv	Mycobacteroides abscessus subsp. massiliense GO 06	"Prokaryotes;Bacteria;Actinobacteria;Mycobacteroides"
5055	mabb	Mycobacteroides abscessus subsp. bolletii 50594	"Prokaryotes;Bacteria;Actinobacteria;Mycobacteroides"
5056	mabl	Mycobacteroides abscessus subsp. massiliense CCUG 48898 = JCM 15300	"Prokaryotes;Bacteria;Actinobacteria;Mycobacteroides"
5057	mche	Mycobacteroides chelonae	"Prokaryotes;Bacteria;Actinobacteria;Mycobacteroides"
5058	miz	Mycobacteroides immunogenum	"Prokaryotes;Bacteria;Actinobacteria;Mycobacteroides"
5059	mste	Mycobacterium stephanolepidis	"Prokaryotes;Bacteria;Actinobacteria;Mycobacteroides"
5060	msao	Mycobacteroides saopaulense	"Prokaryotes;Bacteria;Actinobacteria;Mycobacteroides"
5061	msal	Mycobacteroides salmoniphilum	"Prokaryotes;Bacteria;Actinobacteria;Mycobacteroides"
5062	mjd	Mycolicibacter sinensis	"Prokaryotes;Bacteria;Actinobacteria;Mycolicibacter"
5063	mter	Mycolicibacter terrae	"Prokaryotes;Bacteria;Actinobacteria;Mycolicibacter"
5064	mmin	Mycolicibacter minnesotensis	"Prokaryotes;Bacteria;Actinobacteria;Mycolicibacter"
5065	mhib	Mycolicibacter hiberniae	"Prokaryotes;Bacteria;Actinobacteria;Mycolicibacter"
5066	asd	Hoyosella subflava	"Prokaryotes;Bacteria;Actinobacteria;Hoyosella"
5067	mkr	Mycolicibacillus koreensis	"Prokaryotes;Bacteria;Actinobacteria;Mycolicibacillus"
5068	cgl	Corynebacterium glutamicum ATCC 13032 (Kyowa Hakko)	"Prokaryotes;Bacteria;Actinobacteria;Corynebacterium"
5069	cgb	Corynebacterium glutamicum ATCC 13032 (Bielefeld)	"Prokaryotes;Bacteria;Actinobacteria;Corynebacterium"

5070	cgu	Corynebacterium glutamicum K051	"Prokaryotes;Bacteria;Actinobacteria;Corynebacterium"
5071	cgt	Corynebacterium glutamicum R	"Prokaryotes;Bacteria;Actinobacteria;Corynebacterium"
5072	cgs	Corynebacterium glutamicum SCgG1	"Prokaryotes;Bacteria;Actinobacteria;Corynebacterium"
5073	cgg	Corynebacterium glutamicum SCgG2	"Prokaryotes;Bacteria;Actinobacteria;Corynebacterium"
5074	cgm	Corynebacterium glutamicum MB001	"Prokaryotes;Bacteria;Actinobacteria;Corynebacterium"
5075	cgj	Corynebacterium glutamicum ATCC 21831	"Prokaryotes;Bacteria;Actinobacteria;Corynebacterium"
5076	cgq	Corynebacterium glutamicum AR1	"Prokaryotes;Bacteria;Actinobacteria;Corynebacterium"
5077	cgx	Corynebacterium glutamicum B253	"Prokaryotes;Bacteria;Actinobacteria;Corynebacterium"
5078	cef	Corynebacterium efficiens	"Prokaryotes;Bacteria;Actinobacteria;Corynebacterium"
5079	cdi	Corynebacterium diphtheriae NCTC 13129	"Prokaryotes;Bacteria;Actinobacteria;Corynebacterium"
5080	cdp	Corynebacterium diphtheriae 241	"Prokaryotes;Bacteria;Actinobacteria;Corynebacterium"
5081	cdh	Corynebacterium diphtheriae INCA 402	"Prokaryotes;Bacteria;Actinobacteria;Corynebacterium"
5082	cdt	Corynebacterium diphtheriae HC01	"Prokaryotes;Bacteria;Actinobacteria;Corynebacterium"
5083	cde	Corynebacterium diphtheriae HC02	"Prokaryotes;Bacteria;Actinobacteria;Corynebacterium"
5084	cdr	Corynebacterium diphtheriae HC03	"Prokaryotes;Bacteria;Actinobacteria;Corynebacterium"
5085	cda	Corynebacterium diphtheriae HC04	"Prokaryotes;Bacteria;Actinobacteria;Corynebacterium"
5086	cdz	Corynebacterium diphtheriae 31A	"Prokaryotes;Bacteria;Actinobacteria;Corynebacterium"
5087	cdb	Corynebacterium diphtheriae BH8	"Prokaryotes;Bacteria;Actinobacteria;Corynebacterium"
5088	cds	Corynebacterium diphtheriae C7 (beta)	"Prokaryotes;Bacteria;Actinobacteria;Corynebacterium"
5089	cdd	Corynebacterium diphtheriae CDCE 8392	"Prokaryotes;Bacteria;Actinobacteria;Corynebacterium"
5090	cdw	Corynebacterium diphtheriae PW8	"Prokaryotes;Bacteria;Actinobacteria;Corynebacterium"
5091	cdv	Corynebacterium diphtheriae VA01	"Prokaryotes;Bacteria;Actinobacteria;Corynebacterium"
5092	cdip	Corynebacterium diphtheriae NCTC11397	"Prokaryotes;Bacteria;Actinobacteria;Corynebacterium"
5093	cjk	Corynebacterium jeikeium	"Prokaryotes;Bacteria;Actinobacteria;Corynebacterium"
5094	cur	Corynebacterium urealyticum DSM 7109	"Prokaryotes;Bacteria;Actinobacteria;Corynebacterium"
5095	cua	Corynebacterium urealyticum DSM 7111	"Prokaryotes;Bacteria;Actinobacteria;Corynebacterium"
5096	car	Corynebacterium aurimucosum	"Prokaryotes;Bacteria;Actinobacteria;Corynebacterium"
5097	ckp	Corynebacterium kroppenstedtii	"Prokaryotes;Bacteria;Actinobacteria;Corynebacterium"
5098	cpl	Corynebacterium pseudotuberculosis 3/99-5	"Prokaryotes;Bacteria;Actinobacteria;Corynebacterium"
5099	cpg	Corynebacterium pseudotuberculosis 316	"Prokaryotes;Bacteria;Actinobacteria;Corynebacterium"
5100	cpp	Corynebacterium pseudotuberculosis P54B96	"Prokaryotes;Bacteria;Actinobacteria;Corynebacterium"
5101	cpk	Corynebacterium pseudotuberculosis 1002	"Prokaryotes;Bacteria;Actinobacteria;Corynebacterium"
5102	cpq	Corynebacterium pseudotuberculosis C231	"Prokaryotes;Bacteria;Actinobacteria;Corynebacterium"
5103	cpx	Corynebacterium pseudotuberculosis I19	"Prokaryotes;Bacteria;Actinobacteria;Corynebacterium"
5104	cpz	Corynebacterium pseudotuberculosis PAT10	"Prokaryotes;Bacteria;Actinobacteria;Corynebacterium"
5105	cor	Corynebacterium pseudotuberculosis 267	"Prokaryotes;Bacteria;Actinobacteria;Corynebacterium"
5106	cop	Corynebacterium pseudotuberculosis 31	"Prokaryotes;Bacteria;Actinobacteria;Corynebacterium"
5107	cod	Corynebacterium pseudotuberculosis 1/06-A	"Prokaryotes;Bacteria;Actinobacteria;Corynebacterium"
5108	cos	Corynebacterium pseudotuberculosis 42/02-A	"Prokaryotes;Bacteria;Actinobacteria;Corynebacterium"
5109	coi	Corynebacterium pseudotuberculosis CIP 52.97	"Prokaryotes;Bacteria;Actinobacteria;Corynebacterium"
5110	coe	Corynebacterium pseudotuberculosis 258	"Prokaryotes;Bacteria;Actinobacteria;Corynebacterium"

5111	cou	Corynebacterium pseudotuberculosis Cp162	"Prokaryotes;Bacteria;Actinobacteria;Corynebacterium"
5112	cpse	Corynebacterium pseudotuberculosis 48252	"Prokaryotes;Bacteria;Actinobacteria;Corynebacterium"
5113	cpsu	Corynebacterium pseudotuberculosis CS_10	"Prokaryotes;Bacteria;Actinobacteria;Corynebacterium"
5114	cpsf	Corynebacterium pseudotuberculosis Ft_2193/67	"Prokaryotes;Bacteria;Actinobacteria;Corynebacterium"
5115	crd	Corynebacterium resistens	"Prokaryotes;Bacteria;Actinobacteria;Corynebacterium"
5116	cul	Corynebacterium ulcerans BR-AD22	"Prokaryotes;Bacteria;Actinobacteria;Corynebacterium"
5117	cuc	Corynebacterium ulcerans 809	"Prokaryotes;Bacteria;Actinobacteria;Corynebacterium"
5118	cue	Corynebacterium ulcerans 0102	"Prokaryotes;Bacteria;Actinobacteria;Corynebacterium"
5119	cun	Corynebacterium ulcerans 210932	"Prokaryotes;Bacteria;Actinobacteria;Corynebacterium"
5120	cus	Corynebacterium ulcerans FRC11	"Prokaryotes;Bacteria;Actinobacteria;Corynebacterium"
5121	cuq	Corynebacterium ulcerans 210931	"Prokaryotes;Bacteria;Actinobacteria;Corynebacterium"
5122	cuz	Corynebacterium ulcerans 05146	"Prokaryotes;Bacteria;Actinobacteria;Corynebacterium"
5123	cuj	Corynebacterium ulcerans 131002	"Prokaryotes;Bacteria;Actinobacteria;Corynebacterium"
5124	cva	Corynebacterium variabile	"Prokaryotes;Bacteria;Actinobacteria;Corynebacterium"
5125	chn	Corynebacterium halotolerans	"Prokaryotes;Bacteria;Actinobacteria;Corynebacterium"
5126	ccn	Corynebacterium callunae	"Prokaryotes;Bacteria;Actinobacteria;Corynebacterium"
5127	cter	Corynebacterium terpenotabidum	"Prokaryotes;Bacteria;Actinobacteria;Corynebacterium"
5128	cmd	Corynebacterium maris	"Prokaryotes;Bacteria;Actinobacteria;Corynebacterium"
5129	caz	Corynebacterium argentoratense	"Prokaryotes;Bacteria;Actinobacteria;Corynebacterium"
5130	cfn	Corynebacterium falsenii	"Prokaryotes;Bacteria;Actinobacteria;Corynebacterium"
5131	ccg	Corynebacterium casei	"Prokaryotes;Bacteria;Actinobacteria;Corynebacterium"
5132	cvt	Corynebacterium vitaeruminis	"Prokaryotes;Bacteria;Actinobacteria;Corynebacterium"
5133	cgy	Corynebacterium glyciniphilum	"Prokaryotes;Bacteria;Actinobacteria;Corynebacterium"
5134	cax	Corynebacterium atypicum	"Prokaryotes;Bacteria;Actinobacteria;Corynebacterium"
5135	cii	Corynebacterium imitans	"Prokaryotes;Bacteria;Actinobacteria;Corynebacterium"
5136	cuv	Corynebacterium ureicelerivorans	"Prokaryotes;Bacteria;Actinobacteria;Corynebacterium"
5137	coa	Corynebacterium sp. ATCC 6931	"Prokaryotes;Bacteria;Actinobacteria;Corynebacterium"
5138	cdo	Corynebacterium doosanense	"Prokaryotes;Bacteria;Actinobacteria;Corynebacterium"
5139	chm	Corynebacterium humireducens	"Prokaryotes;Bacteria;Actinobacteria;Corynebacterium"
5140	csx	Corynebacterium singulare	"Prokaryotes;Bacteria;Actinobacteria;Corynebacterium"
5141	cmq	Corynebacterium marinum	"Prokaryotes;Bacteria;Actinobacteria;Corynebacterium"
5142	cku	Corynebacterium kutscheri	"Prokaryotes;Bacteria;Actinobacteria;Corynebacterium"
5143	ccj	Corynebacterium camporealensis	"Prokaryotes;Bacteria;Actinobacteria;Corynebacterium"
5144	cmv	Corynebacterium mustelae	"Prokaryotes;Bacteria;Actinobacteria;Corynebacterium"
5145	cei	Corynebacterium epidermidicanis	"Prokaryotes;Bacteria;Actinobacteria;Corynebacterium"
5146	cted	Corynebacterium testudinoris	"Prokaryotes;Bacteria;Actinobacteria;Corynebacterium"
5147	cut	Corynebacterium uterequi	"Prokaryotes;Bacteria;Actinobacteria;Corynebacterium"
5148	clw	Corynebacterium lactis	"Prokaryotes;Bacteria;Actinobacteria;Corynebacterium"
5149	cdx	Corynebacterium deserti	"Prokaryotes;Bacteria;Actinobacteria;Corynebacterium"
5150	csp	Corynebacterium simulans	"Prokaryotes;Bacteria;Actinobacteria;Corynebacterium"
5151	csta	Corynebacterium stationis	"Prokaryotes;Bacteria;Actinobacteria;Corynebacterium"

5152	ccjz	Corynebacterium crudilactis	"Prokaryotes;Bacteria;Actinobacteria;Corynebacterium"
5153	cfk	Corynebacterium frankenforstense	"Prokaryotes;Bacteria;Actinobacteria;Corynebacterium"
5154	cpho	Corynebacterium phocae	"Prokaryotes;Bacteria;Actinobacteria;Corynebacterium"
5155	cfc	Corynebacterium flavescens	"Prokaryotes;Bacteria;Actinobacteria;Corynebacterium"
5156	cgv	Corynebacterium glaucum	"Prokaryotes;Bacteria;Actinobacteria;Corynebacterium"
5157	cstr	Corynebacterium striatum	"Prokaryotes;Bacteria;Actinobacteria;Corynebacterium"
5158	caqu	Corynebacterium aquilae	"Prokaryotes;Bacteria;Actinobacteria;Corynebacterium"
5159	csph	Corynebacterium sphenisci	"Prokaryotes;Bacteria;Actinobacteria;Corynebacterium"
5160	camg	Corynebacterium ammoniagenes	"Prokaryotes;Bacteria;Actinobacteria;Corynebacterium"
5161	cmin	Corynebacterium minutissimum	"Prokaryotes;Bacteria;Actinobacteria;Corynebacterium"
5162	cpeg	Corynebacterium pelargi	"Prokaryotes;Bacteria;Actinobacteria;Corynebacterium"
5163	cxe	Corynebacterium xerosis	"Prokaryotes;Bacteria;Actinobacteria;Corynebacterium"
5164	cee	Corynebacterium endometrii	"Prokaryotes;Bacteria;Actinobacteria;Corynebacterium"
5165	csan	Corynebacterium sanguinis	"Prokaryotes;Bacteria;Actinobacteria;Corynebacterium"
5166	cgk	Corynebacterium geronticis	"Prokaryotes;Bacteria;Actinobacteria;Corynebacterium"
5167	crf	Corynebacterium rouxii	"Prokaryotes;Bacteria;Actinobacteria;Corynebacterium"
5168	crl	Corynebacterium renale	"Prokaryotes;Bacteria;Actinobacteria;Corynebacterium"
5169	ccho	Corynebacterium choanis	"Prokaryotes;Bacteria;Actinobacteria;Corynebacterium"
5170	cpre	Corynebacterium provencense	"Prokaryotes;Bacteria;Actinobacteria;Corynebacterium"
5171	cpso	Corynebacterium pseudopelargi	"Prokaryotes;Bacteria;Actinobacteria;Corynebacterium"
5172	csur	Corynebacterium suranareeae	"Prokaryotes;Bacteria;Actinobacteria;Corynebacterium"
5173	bfv	Brevibacterium flavum	"Prokaryotes;Bacteria;Actinobacteria;Corynebacterium"
5174	nfa	Nocardia farcinica IFM 10152	"Prokaryotes;Bacteria;Actinobacteria;Nocardia"
5175	nfr	Nocardia farcinica NCTC11134	"Prokaryotes;Bacteria;Actinobacteria;Nocardia"
5176	ncy	Nocardia cyriacigeorgica	"Prokaryotes;Bacteria;Actinobacteria;Nocardia"
5177	nbr	Nocardia brasiliensis	"Prokaryotes;Bacteria;Actinobacteria;Nocardia"
5178	nno	Nocardia nova	"Prokaryotes;Bacteria;Actinobacteria;Nocardia"
5179	nsl	Nocardia mangyaensis	"Prokaryotes;Bacteria;Actinobacteria;Nocardia"
5180	nsr	Nocardia seriolae	"Prokaryotes;Bacteria;Actinobacteria;Nocardia"
5181	ntp	Nocardia terpenica	"Prokaryotes;Bacteria;Actinobacteria;Nocardia"
5182	noz	Nocardia sp. CS682	"Prokaryotes;Bacteria;Actinobacteria;Nocardia"
5183	nod	Nocardia otitidiscaviarum	"Prokaryotes;Bacteria;Actinobacteria;Nocardia"
5184	nah	Nocardia arthritidis	"Prokaryotes;Bacteria;Actinobacteria;Nocardia"
5185	nad	Nocardia asteroides	"Prokaryotes;Bacteria;Actinobacteria;Nocardia"
5186	nwl	Nocardia wallacei	"Prokaryotes;Bacteria;Actinobacteria;Nocardia"
5187	rha	Rhodococcus jostii	"Prokaryotes;Bacteria;Actinobacteria;Rhodococcus"
5188	rer	Rhodococcus erythropolis PR4	"Prokaryotes;Bacteria;Actinobacteria;Rhodococcus"
5189	rey	Rhodococcus erythropolis CCM2595	"Prokaryotes;Bacteria;Actinobacteria;Rhodococcus"
5190	reb	Rhodococcus erythropolis BG43	"Prokaryotes;Bacteria;Actinobacteria;Rhodococcus"
5191	rop	Rhodococcus opacus B4	"Prokaryotes;Bacteria;Actinobacteria;Rhodococcus"
5192	roa	Rhodococcus opacus PD630	"Prokaryotes;Bacteria;Actinobacteria;Rhodococcus"

5193	req	Rhodococcus hoagii	"Prokaryotes;Bacteria;Actinobacteria;Rhodococcus"
5194	rpy	Rhodococcus pyridinivorans	"Prokaryotes;Bacteria;Actinobacteria;Rhodococcus"
5195	rhb	Rhodococcus sp. B7740	"Prokaryotes;Bacteria;Actinobacteria;Rhodococcus"
5196	rav	Rhodococcus aetherivorans	"Prokaryotes;Bacteria;Actinobacteria;Rhodococcus"
5197	rfa	Rhodococcus fascians	"Prokaryotes;Bacteria;Actinobacteria;Rhodococcus"
5198	rhw	Rhodococcus sp. WMMA185	"Prokaryotes;Bacteria;Actinobacteria;Rhodococcus"
5199	rhs	Rhodococcus sp. PBTS 2	"Prokaryotes;Bacteria;Actinobacteria;Rhodococcus"
5200	rrz	Rhodococcus ruber	"Prokaryotes;Bacteria;Actinobacteria;Rhodococcus"
5201	rhu	Rhodococcus sp. PBTS 1	"Prokaryotes;Bacteria;Actinobacteria;Rhodococcus"
5202	rqi	Rhodococcus qingshengii	"Prokaryotes;Bacteria;Actinobacteria;Rhodococcus"
5203	rhq	Rhodococcus sp. p52	"Prokaryotes;Bacteria;Actinobacteria;Rhodococcus"
5204	rhod	Rhodococcus sp. 008	"Prokaryotes;Bacteria;Actinobacteria;Rhodococcus"
5205	rrt	Rhodococcus rhodochrous	"Prokaryotes;Bacteria;Actinobacteria;Rhodococcus"
5206	rby	Rhodococcus biphenylivorans	"Prokaryotes;Bacteria;Actinobacteria;Rhodococcus"
5207	rcr	Rhodococcus coprophilus	"Prokaryotes;Bacteria;Actinobacteria;Rhodococcus"
5208	rtm	Rhodococcus triatomae	"Prokaryotes;Bacteria;Actinobacteria;Rhodococcus"
5209	gbr	Gordonia bronchialis	"Prokaryotes;Bacteria;Actinobacteria;Gordonia"
5210	gpo	Gordonia polyisoprenivorans	"Prokaryotes;Bacteria;Actinobacteria;Gordonia"
5211	gor	Gordonia sp. KTR9	"Prokaryotes;Bacteria;Actinobacteria;Gordonia"
5212	goq	Gordonia phthalatica	"Prokaryotes;Bacteria;Actinobacteria;Gordonia"
5213	gta	Gordonia terrae	"Prokaryotes;Bacteria;Actinobacteria;Gordonia"
5214	goc	Gordonia sp. YC-JH1	"Prokaryotes;Bacteria;Actinobacteria;Gordonia"
5215	git	Gordonia iterans	"Prokaryotes;Bacteria;Actinobacteria;Gordonia"
5216	gru	Gordonia rubripertincta	"Prokaryotes;Bacteria;Actinobacteria;Gordonia"
5217	gom	Gordonia insulae	"Prokaryotes;Bacteria;Actinobacteria;Gordonia"
5218	gav	Gordonia alkanivorans	"Prokaryotes;Bacteria;Actinobacteria;Gordonia"
5219	god	Gordonia sp. 135	"Prokaryotes;Bacteria;Actinobacteria;Gordonia"
5220	tpr	Tsukamurella paurometabola	"Prokaryotes;Bacteria;Actinobacteria;Tsukamurella"
5221	tsm	Tsukamurella tyrosinosolvens	"Prokaryotes;Bacteria;Actinobacteria;Tsukamurella"
5222	srt	Segniliparus rotundus	"Prokaryotes;Bacteria;Actinobacteria;Segniliparus"
5223	dtm	Dietzia timorensis	"Prokaryotes;Bacteria;Actinobacteria;Dietzia"
5224	dit	Dietzia sp. oral taxon 368	"Prokaryotes;Bacteria;Actinobacteria;Dietzia"
5225	diz	Dietzia sp. JS16-p6b	"Prokaryotes;Bacteria;Actinobacteria;Dietzia"
5226	dpc	Dietzia psychralcaliphila	"Prokaryotes;Bacteria;Actinobacteria;Dietzia"
5227	dlu	Dietzia lutea	"Prokaryotes;Bacteria;Actinobacteria;Dietzia"
5228	cbq	Lawsonella clevelandensis	"Prokaryotes;Bacteria;Actinobacteria;Lawsonella"
5229	toy	Tomitella sp. HY188	"Prokaryotes;Bacteria;Actinobacteria;Tomitella"
5230	sco	Streptomyces coelicolor	"Prokaryotes;Bacteria;Actinobacteria;Streptomyces"
5231	salb	Streptomyces albidoflavus	"Prokaryotes;Bacteria;Actinobacteria;Streptomyces"
5232	sma	Streptomyces avermitilis	"Prokaryotes;Bacteria;Actinobacteria;Streptomyces"
5233	sgr	Streptomyces griseus	"Prokaryotes;Bacteria;Actinobacteria;Streptomyces"

5234	sgb	Streptomyces globisporus	"Prokaryotes;Bacteria;Actinobacteria;Streptomyces"
5235	scb	Streptomyces scabiei	"Prokaryotes;Bacteria;Actinobacteria;Streptomyces"
5236	ssx	Streptomyces sp. SirexAA-E	"Prokaryotes;Bacteria;Actinobacteria;Streptomyces"
5237	svl	Streptomyces violaceusniger	"Prokaryotes;Bacteria;Actinobacteria;Streptomyces"
5238	sct	Streptomyces cattleya NRRL 8057 = DSM 46488	"Prokaryotes;Bacteria;Actinobacteria;Streptomyces"
5239	scy	Streptomyces cattleya NRRL 8057 = DSM 46488	"Prokaryotes;Bacteria;Actinobacteria;Streptomyces"
5240	sfa	Streptomyces pratensis	"Prokaryotes;Bacteria;Actinobacteria;Streptomyces"
5241	sbh	Streptomyces bingchenggensis	"Prokaryotes;Bacteria;Actinobacteria;Streptomyces"
5242	shy	Streptomyces hygroscopicus subsp. jinggangensis 5008	"Prokaryotes;Bacteria;Actinobacteria;Streptomyces"
5243	sho	Streptomyces hygroscopicus subsp. jinggangensis TL01	"Prokaryotes;Bacteria;Actinobacteria;Streptomyces"
5244	sve	Streptomyces venezuelae	"Prokaryotes;Bacteria;Actinobacteria;Streptomyces"
5245	sdv	Streptomyces davaonensis	"Prokaryotes;Bacteria;Actinobacteria;Streptomyces"
5246	sals	Streptomyces albus DSM 41398	"Prokaryotes;Bacteria;Actinobacteria;Streptomyces"
5247	strp	Streptomyces sp. PAMC 26508	"Prokaryotes;Bacteria;Actinobacteria;Streptomyces"
5248	sfi	Streptomyces fulvissimus	"Prokaryotes;Bacteria;Actinobacteria;Streptomyces"
5249	sci	Streptomyces collinus	"Prokaryotes;Bacteria;Actinobacteria;Streptomyces"
5250	src	Streptomyces rapamycinicus	"Prokaryotes;Bacteria;Actinobacteria;Streptomyces"
5251	salu	Streptomyces albulus NK660	"Prokaryotes;Bacteria;Actinobacteria;Streptomyces"
5252	sall	Streptomyces albulus ZPM	"Prokaryotes;Bacteria;Actinobacteria;Streptomyces"
5253	slv	Streptomyces lividans	"Prokaryotes;Bacteria;Actinobacteria;Streptomyces"
5254	sgu	Streptomyces glaucescens	"Prokaryotes;Bacteria;Actinobacteria;Streptomyces"
5255	svt	Streptomyces vietnamensis	"Prokaryotes;Bacteria;Actinobacteria;Streptomyces"
5256	stre	Streptomyces sp. 769	"Prokaryotes;Bacteria;Actinobacteria;Streptomyces"
5257	scw	Streptomyces cyaneogriseus	"Prokaryotes;Bacteria;Actinobacteria;Streptomyces"
5258	sld	Streptomyces lydicus A02	"Prokaryotes;Bacteria;Actinobacteria;Streptomyces"
5259	slc	Streptomyces lydicus 103	"Prokaryotes;Bacteria;Actinobacteria;Streptomyces"
5260	sxi	Streptomyces xiamenensis	"Prokaryotes;Bacteria;Actinobacteria;Streptomyces"
5261	strm	Streptomyces sp. Mg1	"Prokaryotes;Bacteria;Actinobacteria;Streptomyces"
5262	strc	Streptomyces sp. CNQ-509	"Prokaryotes;Bacteria;Actinobacteria;Streptomyces"
5263	samb	Streptomyces ambofaciens	"Prokaryotes;Bacteria;Actinobacteria;Streptomyces"
5264	spri	Streptomyces pristinaespiralis	"Prokaryotes;Bacteria;Actinobacteria;Streptomyces"
5265	scz	Streptomyces sp. CFMR 7	"Prokaryotes;Bacteria;Actinobacteria;Streptomyces"
5266	scx	Streptomyces sp. CdTB01	"Prokaryotes;Bacteria;Actinobacteria;Streptomyces"
5267	srw	Streptomyces reticuli	"Prokaryotes;Bacteria;Actinobacteria;Streptomyces"
5268	strf	Streptomyces sp. 4F	"Prokaryotes;Bacteria;Actinobacteria;Streptomyces"
5269	sle	Streptomyces leeuwenhoekii	"Prokaryotes;Bacteria;Actinobacteria;Streptomyces"
5270	srn	Streptomyces rubrolavendulae	"Prokaryotes;Bacteria;Actinobacteria;Streptomyces"
5271	spav	Streptomyces parvulus	"Prokaryotes;Bacteria;Actinobacteria;Streptomyces"
5272	strt	Streptomyces sp. SAT1	"Prokaryotes;Bacteria;Actinobacteria;Streptomyces"
5273	sclf	Streptomyces clavuligerus	"Prokaryotes;Bacteria;Actinobacteria;Streptomyces"
5274	sgs	Streptomyces griseochromogenes	"Prokaryotes;Bacteria;Actinobacteria;Streptomyces"

5275	stsi	Streptomyces qaidamensis	"Prokaryotes;Bacteria;Actinobacteria;Streptomyces"
5276	sls	Streptomyces lincolnensis	"Prokaryotes;Bacteria;Actinobacteria;Streptomyces"
5277	snr	Streptomyces noursei	"Prokaryotes;Bacteria;Actinobacteria;Streptomyces"
5278	splu	Streptomyces pluripotens	"Prokaryotes;Bacteria;Actinobacteria;Streptomyces"
5279	strd	Streptomyces sp. CCM_MD2014	"Prokaryotes;Bacteria;Actinobacteria;Streptomyces"
5280	snw	Streptomyces niveus	"Prokaryotes;Bacteria;Actinobacteria;Streptomyces"
5281	sauo	Streptomyces autolyticus	"Prokaryotes;Bacteria;Actinobacteria;Streptomyces"
5282	ssia	Streptomyces alfalfae	"Prokaryotes;Bacteria;Actinobacteria;Streptomyces"
5283	svu	Streptomyces violaceoruber	"Prokaryotes;Bacteria;Actinobacteria;Streptomyces"
5284	spun	Streptomyces fodineus	"Prokaryotes;Bacteria;Actinobacteria;Streptomyces"
5285	sgv	Streptomyces gilvosporeus	"Prokaryotes;Bacteria;Actinobacteria;Streptomyces"
5286	smal	Streptomyces malaysiensis	"Prokaryotes;Bacteria;Actinobacteria;Streptomyces"
5287	slau	Streptomyces laurentii	"Prokaryotes;Bacteria;Actinobacteria;Streptomyces"
5288	salf	Streptomyces alboflavus	"Prokaryotes;Bacteria;Actinobacteria;Streptomyces"
5289	salj	Streptomyces albireticuli	"Prokaryotes;Bacteria;Actinobacteria;Streptomyces"
5290	slx	Streptomyces lavendulae subsp. lavendulae	"Prokaryotes;Bacteria;Actinobacteria;Streptomyces"
5291	stro	Streptomyces sp. MOE7	"Prokaryotes;Bacteria;Actinobacteria;Streptomyces"
5292	sfk	Streptomyces formicae	"Prokaryotes;Bacteria;Actinobacteria;Streptomyces"
5293	snz	Streptomyces nigra	"Prokaryotes;Bacteria;Actinobacteria;Streptomyces"
5294	sge	Streptomyces griseorubiginosus	"Prokaryotes;Bacteria;Actinobacteria;Streptomyces"
5295	srj	Streptomyces rochei	"Prokaryotes;Bacteria;Actinobacteria;Streptomyces"
5296	slk	Streptomyces lunaelactis	"Prokaryotes;Bacteria;Actinobacteria;Streptomyces"
5297	sky	Streptomyces koyangensis	"Prokaryotes;Bacteria;Actinobacteria;Streptomyces"
5298	sdx	Streptomyces dengpaensis	"Prokaryotes;Bacteria;Actinobacteria;Streptomyces"
5299	sgd	Streptomyces griseoviridis	"Prokaryotes;Bacteria;Actinobacteria;Streptomyces"
5300	sqz	Streptomyces qinzhouensis	"Prokaryotes;Bacteria;Actinobacteria;Streptomyces"
5301	scya	Streptomyces cyaneochromogenes	"Prokaryotes;Bacteria;Actinobacteria;Streptomyces"
5302	sast	Streptomyces asterosporus	"Prokaryotes;Bacteria;Actinobacteria;Streptomyces"
5303	snq	Streptomyces nodosus	"Prokaryotes;Bacteria;Actinobacteria;Streptomyces"
5304	stir	Streptomyces tirandamycinicus	"Prokaryotes;Bacteria;Actinobacteria;Streptomyces"
5305	ska	Streptomyces kanamyceticus	"Prokaryotes;Bacteria;Actinobacteria;Streptomyces"
5306	sgz	Streptomyces globosus	"Prokaryotes;Bacteria;Actinobacteria;Streptomyces"
5307	svn	Streptomyces vinaceus	"Prokaryotes;Bacteria;Actinobacteria;Streptomyces"
5308	snk	Streptomyces nitrosporeus	"Prokaryotes;Bacteria;Actinobacteria;Streptomyces"
5309	salw	Streptomyces alboniger	"Prokaryotes;Bacteria;Actinobacteria;Streptomyces"
5310	shaw	Streptomyces hawaiiensis	"Prokaryotes;Bacteria;Actinobacteria;Streptomyces"
5311	srk	Streptomyces rectiverticillatus	"Prokaryotes;Bacteria;Actinobacteria;Streptomyces"
5312	sfic	Streptomyces ficellus	"Prokaryotes;Bacteria;Actinobacteria;Streptomyces"
5313	sgal	Streptomyces galilaeus	"Prokaryotes;Bacteria;Actinobacteria;Streptomyces"
5314	sspo	Streptomyces spongiicola	"Prokaryotes;Bacteria;Actinobacteria;Streptomyces"
5315	svr	Streptomyces viridifaciens	"Prokaryotes;Bacteria;Actinobacteria;Streptomyces"

5316	spad	Streptomyces paludis	"Prokaryotes;Bacteria;Actinobacteria;Streptomyces"
5317	sfy	Streptomyces fagopyri	"Prokaryotes;Bacteria;Actinobacteria;Streptomyces"
5318	saqu	Streptomyces aquilus	"Prokaryotes;Bacteria;Actinobacteria;Streptomyces"
5319	sgf	Streptomyces griseofuscus	"Prokaryotes;Bacteria;Actinobacteria;Streptomyces"
5320	scav	Streptomyces cavourensis	"Prokaryotes;Bacteria;Actinobacteria;Streptomyces"
5321	sseo	Streptomyces seoulensis	"Prokaryotes;Bacteria;Actinobacteria;Streptomyces"
5322	ksk	Kitasatospora setae	"Prokaryotes;Bacteria;Actinobacteria;Kitasatospora"
5323	kab	Kitasatospora albolonga	"Prokaryotes;Bacteria;Actinobacteria;Kitasatospora"
5324	kau	Kitasatospora aureofaciens	"Prokaryotes;Bacteria;Actinobacteria;Kitasatospora"
5325	kit	Kitasatospora sp. MMS16-BH015	"Prokaryotes;Bacteria;Actinobacteria;Kitasatospora"
5326	stri	Streptacidiphilus bronchialis	"Prokaryotes;Bacteria;Actinobacteria;Streptacidiphilus"
5327	twh	Tropheryma whipplei Twist	"Prokaryotes;Bacteria;Actinobacteria;Tropheryma"
5328	tws	Tropheryma whipplei TW08/27	"Prokaryotes;Bacteria;Actinobacteria;Tropheryma"
5329	lxl	Luteimicrobium xylanilyticum	"Prokaryotes;Bacteria;Actinobacteria;Luteimicrobium"
5330	lxx	Leifsonia xyli subsp. xyli CTCB07	"Prokaryotes;Bacteria;Actinobacteria;Leifsonia"
5331	lxy	Leifsonia xyli subsp. cynodontis DSM 46306	"Prokaryotes;Bacteria;Actinobacteria;Leifsonia"
5332	leif	Leifsonia sp. PS1209	"Prokaryotes;Bacteria;Actinobacteria;Leifsonia"
5333	lse	Leifsonia shinshuensis	"Prokaryotes;Bacteria;Actinobacteria;Leifsonia"
5334	cmi	Clavibacter michiganensis subsp. michiganensis	"Prokaryotes;Bacteria;Actinobacteria;Clavibacter"
5335	cms	Clavibacter michiganensis subsp. sepedonicus	"Prokaryotes;Bacteria;Actinobacteria;Clavibacter"
5336	cmc	Clavibacter michiganensis subsp. nebraskensis	"Prokaryotes;Bacteria;Actinobacteria;Clavibacter"
5337	cmh	Clavibacter michiganensis subsp. insidiosus	"Prokaryotes;Bacteria;Actinobacteria;Clavibacter"
5338	ccap	Clavibacter michiganensis subsp. capsici	"Prokaryotes;Bacteria;Actinobacteria;Clavibacter"
5339	mts	Microbacterium testaceum	"Prokaryotes;Bacteria;Actinobacteria;Microbacterium"
5340	mim	Microbacterium sp. CGR1	"Prokaryotes;Bacteria;Actinobacteria;Microbacterium"
5341	mio	Microbacterium sp. No. 7	"Prokaryotes;Bacteria;Actinobacteria;Microbacterium"
5342	mix	Microbacterium sp. XT11	"Prokaryotes;Bacteria;Actinobacteria;Microbacterium"
5343	mip	Microbacterium sp. PAMC 28756	"Prokaryotes;Bacteria;Actinobacteria;Microbacterium"
5344	mcw	Microbacterium chocolatum	"Prokaryotes;Bacteria;Actinobacteria;Microbacterium"
5345	mpal	Microbacterium paludicola	"Prokaryotes;Bacteria;Actinobacteria;Microbacterium"
5346	mih	Microbacterium sp. BH-3-3-3	"Prokaryotes;Bacteria;Actinobacteria;Microbacterium"
5347	micr	Microbacterium sp. 1.5R	"Prokaryotes;Bacteria;Actinobacteria;Microbacterium"
5348	maur	Microbacterium aurum	"Prokaryotes;Bacteria;Actinobacteria;Microbacterium"
5349	mhos	Microbacterium hominis	"Prokaryotes;Bacteria;Actinobacteria;Microbacterium"
5350	mfol	Microbacterium foliorum	"Prokaryotes;Bacteria;Actinobacteria;Microbacterium"
5351	moo	Microbacterium oleivorans	"Prokaryotes;Bacteria;Actinobacteria;Microbacterium"
5352	mlv	Microbacterium lemovicicum	"Prokaryotes;Bacteria;Actinobacteria;Microbacterium"
5353	mwa	Microbacterium wangchenii	"Prokaryotes;Bacteria;Actinobacteria;Microbacterium"
5354	mprt	Microbacterium protaetiae	"Prokaryotes;Bacteria;Actinobacteria;Microbacterium"
5355	msed	Microbacterium sediminis	"Prokaryotes;Bacteria;Actinobacteria;Microbacterium"
5356	moy	Microbacterium oxydans	"Prokaryotes;Bacteria;Actinobacteria;Microbacterium"

5357	rla	Rhodoluna lacicola	"Prokaryotes;Bacteria;Actinobacteria;Rhodoluna"
5358	rpla	Candidatus Rhodoluna planktonica	"Prokaryotes;Bacteria;Actinobacteria;Rhodoluna"
5359	aqg	Candidatus Aquiluna sp. 15G-AUS-rot	"Prokaryotes;Bacteria;Actinobacteria;Aquiluna"
5360	rtx	Rathayibacter toxicus 70137	"Prokaryotes;Bacteria;Actinobacteria;Rathayibacter"
5361	rtc	Rathayibacter toxicus WAC3373	"Prokaryotes;Bacteria;Actinobacteria;Rathayibacter"
5362	rtn	Rathayibacter tritici	"Prokaryotes;Bacteria;Actinobacteria;Rathayibacter"
5363	rry	Rathayibacter rathayi	"Prokaryotes;Bacteria;Actinobacteria;Rathayibacter"
5364	ria	Rathayibacter iranicus	"Prokaryotes;Bacteria;Actinobacteria;Rathayibacter"
5365	rfs	Rathayibacter festucae	"Prokaryotes;Bacteria;Actinobacteria;Rathayibacter"
5366	rte	Rathayibacter tanaceti	"Prokaryotes;Bacteria;Actinobacteria;Rathayibacter"
5367	cum	Curtobacterium sp. MR_MD2014	"Prokaryotes;Bacteria;Actinobacteria;Curtobacterium"
5368	cub	Curtobacterium sp. BH-2-1-1	"Prokaryotes;Bacteria;Actinobacteria;Curtobacterium"
5369	cug	Curtobacterium sp. SGAir0471	"Prokaryotes;Bacteria;Actinobacteria;Curtobacterium"
5370	cqf	Curtobacterium flaccumfaciens	"Prokaryotes;Bacteria;Actinobacteria;Curtobacterium"
5371	mvd	Microterricola viridarii	"Prokaryotes;Bacteria;Actinobacteria;Microterricola"
5372	frp	Frondihabitans sp. PAMC 28766	"Prokaryotes;Bacteria;Actinobacteria;Frondihabitans"
5373	agy	Agromyces aureus	"Prokaryotes;Bacteria;Actinobacteria;Agromyces"
5374	agm	Agromyces badenianii	"Prokaryotes;Bacteria;Actinobacteria;Agromyces"
5375	agf	Agromyces protaetiae	"Prokaryotes;Bacteria;Actinobacteria;Agromyces"
5376	cart	Cryobacterium arcticum	"Prokaryotes;Bacteria;Actinobacteria;Cryobacterium"
5377	cry	Cryobacterium sp. LW097	"Prokaryotes;Bacteria;Actinobacteria;Cryobacterium"
5378	cphy	Cnuibacter physcomitrellae	"Prokaryotes;Bacteria;Actinobacteria;Cnuibacter"
5379	amin	Aurantimicrobium minutum	"Prokaryotes;Bacteria;Actinobacteria;Aurantimicrobium"
5380	aum	Aurantimicrobium sp. MWH-Mo1	"Prokaryotes;Bacteria;Actinobacteria;Aurantimicrobium"
5381	auw	Aurantimicrobium sp. MWH-Uga1	"Prokaryotes;Bacteria;Actinobacteria;Aurantimicrobium"
5382	psai	Pontimonas salivibrio	"Prokaryotes;Bacteria;Actinobacteria;Pontimonas"
5383	malk	Microcella alkaliphila	"Prokaryotes;Bacteria;Actinobacteria;Microcella"
5384	myl	Mycetocola zhujimingii	"Prokaryotes;Bacteria;Actinobacteria;Mycetocola"
5385	salc	Salinibacterium hongtaonis	"Prokaryotes;Bacteria;Actinobacteria;Salinibacterium"
5386	sala	Salinibacterium sp. UTAS2018	"Prokaryotes;Bacteria;Actinobacteria;Salinibacterium"
5387	sald	Salinibacterium sp. dk2585	"Prokaryotes;Bacteria;Actinobacteria;Salinibacterium"
5388	hum	Humibacter sp. BT305	"Prokaryotes;Bacteria;Actinobacteria;Humibacter"
5389	huw	Humibacter sp. WJ7-1	"Prokaryotes;Bacteria;Actinobacteria;Humibacter"
5390	gry	Gryllotalpicola protaetiae	"Prokaryotes;Bacteria;Actinobacteria;Gryllotalpicola"
5391	lyd	Protaetiibacter intestinalis	"Prokaryotes;Bacteria;Actinobacteria;Protaetiibacter"
5392	lyk	Lysinimonas sp. KACC 19322	"Prokaryotes;Bacteria;Actinobacteria;Lysinimonas"
5393	plap	Plantibacter sp. PA-3-X8	"Prokaryotes;Bacteria;Actinobacteria;Plantibacter"
5394	leu	Leucobacter muris	"Prokaryotes;Bacteria;Actinobacteria;Leucobacter"
5395	ltr	Leucobacter triazinivorans	"Prokaryotes;Bacteria;Actinobacteria;Leucobacter"
5396	leb	Leucobacter coleopterorum	"Prokaryotes;Bacteria;Actinobacteria;Leucobacter"
5397	ldn	Leucobacter denitrificans	"Prokaryotes;Bacteria;Actinobacteria;Leucobacter"

5398	agg	Agrococcus sp. SGAir0287	"Prokaryotes;Bacteria;Actinobacteria;Agrococcus"
5399	mant	Marisediminicola antarctica	"Prokaryotes;Bacteria;Actinobacteria;Marisediminicola"
5400	hea	Herbiconiux sp. SALV-R1	"Prokaryotes;Bacteria;Actinobacteria;Herbiconiux"
5401	frn	Frigoribacterium sp. NBH87	"Prokaryotes;Bacteria;Actinobacteria;Frigoribacterium"
5402	gln	Glaciihabitans sp. INWT7	"Prokaryotes;Bacteria;Actinobacteria;Glaciihabitans"
5403	chre	Chryseoglobus sp. 28M-23	"Prokaryotes;Bacteria;Actinobacteria;Chryseoglobus"
5404	agx	Agreia sp. COWG	"Prokaryotes;Bacteria;Actinobacteria;Agreia"
5405	art	Arthrobacter sp. FB24	"Prokaryotes;Bacteria;Actinobacteria;Arthrobacter"
5406	arr	Arthrobacter sp. Rue61a	"Prokaryotes;Bacteria;Actinobacteria;Arthrobacter"
5407	arm	Arthrobacter sp. PAMC 25486	"Prokaryotes;Bacteria;Actinobacteria;Arthrobacter"
5408	arl	Arthrobacter sp. LS16	"Prokaryotes;Bacteria;Actinobacteria;Arthrobacter"
5409	are	Arthrobacter sp. ERGS1:01	"Prokaryotes;Bacteria;Actinobacteria;Arthrobacter"
5410	aaq	Arthrobacter alpinus R3.8	"Prokaryotes;Bacteria;Actinobacteria;Arthrobacter"
5411	arw	Arthrobacter alpinus A3	"Prokaryotes;Bacteria;Actinobacteria;Arthrobacter"
5412	arh	Arthrobacter sp. Hiyo8	"Prokaryotes;Bacteria;Actinobacteria;Arthrobacter"
5413	ary	Arthrobacter sp. YC-RL1	"Prokaryotes;Bacteria;Actinobacteria;Arthrobacter"
5414	arz	Arthrobacter sp. ATCC 21022	"Prokaryotes;Bacteria;Actinobacteria;Arthrobacter"
5415	aru	Arthrobacter sp. U41	"Prokaryotes;Bacteria;Actinobacteria;Arthrobacter"
5416	arq	Arthrobacter sp. QXT-31	"Prokaryotes;Bacteria;Actinobacteria;Arthrobacter"
5417	arn	Arthrobacter sp. YN	"Prokaryotes;Bacteria;Actinobacteria;Arthrobacter"
5418	arx	Arthrobacter sp. ZXY-2	"Prokaryotes;Bacteria;Actinobacteria;Arthrobacter"
5419	acry	Arthrobacter crystallopoietes	"Prokaryotes;Bacteria;Actinobacteria;Arthrobacter"
5420	arth	Arthrobacter sp. PGP41	"Prokaryotes;Bacteria;Actinobacteria;Arthrobacter"
5421	artp	Arthrobacter sp. PAMC25564	"Prokaryotes;Bacteria;Actinobacteria;Arthrobacter"
5422	acit	Arthrobacter citreus	"Prokaryotes;Bacteria;Actinobacteria;Arthrobacter"
5423	ari	Psychromicrobium lacuslunae	"Prokaryotes;Bacteria;Actinobacteria;Psychromicrobium"
5424	aau	Paenarthrobacter aurescens	"Prokaryotes;Bacteria;Actinobacteria;Paenarthrobacter"
5425	pue	Paenarthrobacter ureafaciens	"Prokaryotes;Bacteria;Actinobacteria;Paenarthrobacter"
5426	ach	Pseudarthrobacter chlorophenolicus	"Prokaryotes;Bacteria;Actinobacteria;Pseudarthrobacter"
5427	apn	Pseudarthrobacter phenanthrenivorans	"Prokaryotes;Bacteria;Actinobacteria;Pseudarthrobacter"
5428	psul	Pseudarthrobacter sulfonivorans	"Prokaryotes;Bacteria;Actinobacteria;Pseudarthrobacter"
5429	psni	Pseudarthrobacter sp. NIBRBAC000502771	"Prokaryotes;Bacteria;Actinobacteria;Pseudarthrobacter"
5430	psey	Pseudarthrobacter sp. YJ56	"Prokaryotes;Bacteria;Actinobacteria;Pseudarthrobacter"
5431	aai	Glutamicibacter arilaitensis	"Prokaryotes;Bacteria;Actinobacteria;Glutamicibacter"
5432	gar	Glutamicibacter halophytocola	"Prokaryotes;Bacteria;Actinobacteria;Glutamicibacter"
5433	gcr	Glutamicibacter creatinolyticus	"Prokaryotes;Bacteria;Actinobacteria;Glutamicibacter"
5434	glu	Glutamicibacter sp. ZJUTW	"Prokaryotes;Bacteria;Actinobacteria;Glutamicibacter"
5435	rsa	Renibacterium salmoninarum	"Prokaryotes;Bacteria;Actinobacteria;Renibacterium"
5436	krh	Kocuria rhizophila	"Prokaryotes;Bacteria;Actinobacteria;Kocuria"
5437	kpl	Kocuria palustris	"Prokaryotes;Bacteria;Actinobacteria;Kocuria"
5438	kfv	Kocuria flava	"Prokaryotes;Bacteria;Actinobacteria;Kocuria"

5439	kii	Kocuria indica	"Prokaryotes;Bacteria;Actinobacteria;Kocuria"
5440	krs	Kocuria rosea	"Prokaryotes;Bacteria;Actinobacteria;Kocuria"
5441	kod	Kocuria sp. KD4	"Prokaryotes;Bacteria;Actinobacteria;Kocuria"
5442	kvr	Kocuria varians	"Prokaryotes;Bacteria;Actinobacteria;Kocuria"
5443	mlu	Micrococcus luteus	"Prokaryotes;Bacteria;Actinobacteria;Micrococcus"
5444	mick	Micrococcus sp. KBS0714	"Prokaryotes;Bacteria;Actinobacteria;Micrococcus"
5445	rmu	Rothia mucilaginosa	"Prokaryotes;Bacteria;Actinobacteria;Rothia"
5446	rdn	Rothia dentocariosa	"Prokaryotes;Bacteria;Actinobacteria;Rothia"
5447	raj	Rothia aeria	"Prokaryotes;Bacteria;Actinobacteria;Rothia"
5448	rter	Rothia terrae	"Prokaryotes;Bacteria;Actinobacteria;Rothia"
5449	rama	Rothia amarae	"Prokaryotes;Bacteria;Actinobacteria;Rothia"
5450	rkr	Rothia kristinae	"Prokaryotes;Bacteria;Actinobacteria;Rothia"
5451	satk	Sinomonas atrocyanea	"Prokaryotes;Bacteria;Actinobacteria;Sinomonas"
5452	nae	Neomicrococcus aestuarii	"Prokaryotes;Bacteria;Actinobacteria;Neomicrococcus"
5453	aul	Auritidibacter sp. NML130574	"Prokaryotes;Bacteria;Actinobacteria;Auritidibacter"
5454	cig	Citricoccus sp. SGAir0453	"Prokaryotes;Bacteria;Actinobacteria;Citricoccus"
5455	bcv	Beutenbergia cavernae	"Prokaryotes;Bacteria;Actinobacteria;Beutenbergia"
5456	bfa	Brachybacterium faecium	"Prokaryotes;Bacteria;Actinobacteria;Brachybacterium"
5457	brx	Brachybacterium sp. P6-10-X1	"Prokaryotes;Bacteria;Actinobacteria;Brachybacterium"
5458	brv	Brachybacterium avium	"Prokaryotes;Bacteria;Actinobacteria;Brachybacterium"
5459	bgg	Brachybacterium ginsengisoli	"Prokaryotes;Bacteria;Actinobacteria;Brachybacterium"
5460	brz	Brachybacterium vulturis	"Prokaryotes;Bacteria;Actinobacteria;Brachybacterium"
5461	bsau	Brachybacterium saurashtrense	"Prokaryotes;Bacteria;Actinobacteria;Brachybacterium"
5462	brr	Brachybacterium sp. SGAir0954	"Prokaryotes;Bacteria;Actinobacteria;Brachybacterium"
5463	dva	Dermabacter vaginalis	"Prokaryotes;Bacteria;Actinobacteria;Dermabacter"
5464	djj	Dermabacter jinjuensis	"Prokaryotes;Bacteria;Actinobacteria;Dermabacter"
5465	jde	Jonesia denitrificans	"Prokaryotes;Bacteria;Actinobacteria;Jonesia"
5466	kse	Kytococcus sedentarius	"Prokaryotes;Bacteria;Actinobacteria;Kytococcus"
5467	dni	Dermacoccus nishinomiyaensis	"Prokaryotes;Bacteria;Actinobacteria;Dermacoccus"
5468	day	Dermacoccus abyssi	"Prokaryotes;Bacteria;Actinobacteria;Dermacoccus"
5469	lmoi	Luteipulveratus mongoliensis	"Prokaryotes;Bacteria;Actinobacteria;Luteipulveratus"
5470	xce	Xylanimonas cellulosilytica	"Prokaryotes;Bacteria;Actinobacteria;Xylanimonas"
5471	xyl	Xylanimonas allomyrinae	"Prokaryotes;Bacteria;Actinobacteria;Xylanimonas"
5472	iva	Isoptericola variabilis	"Prokaryotes;Bacteria;Actinobacteria;Isoptericola"
5473	ido	Isoptericola dokdonensis	"Prokaryotes;Bacteria;Actinobacteria;Isoptericola"
5474	cet	Cellulosimicrobium sp. TH-20	"Prokaryotes;Bacteria;Actinobacteria;Cellulosimicrobium"
5475	cceu	Cellulosimicrobium cellulans	"Prokaryotes;Bacteria;Actinobacteria;Cellulosimicrobium"
5476	xya	Xylanimicrobium sp. FW10M-9	"Prokaryotes;Bacteria;Actinobacteria;Xylanimicrobium"
5477	ske	Sanguibacter keddieii	"Prokaryotes;Bacteria;Actinobacteria;Sanguibacter"
5478	sanw	Sanguibacter sp. HDW7	"Prokaryotes;Bacteria;Actinobacteria;Sanguibacter"
5479	cfl	Cellulomonas flavigena	"Prokaryotes;Bacteria;Actinobacteria;Cellulomonas"

5480	cfi	Cellulomonas fimi	"Prokaryotes;Bacteria;Actinobacteria;Cellulomonas"
5481	cga	Cellulomonas gilvus	"Prokaryotes;Bacteria;Actinobacteria;Cellulomonas"
5482	cez	Cellulomonas sp. PSBB021	"Prokaryotes;Bacteria;Actinobacteria;Cellulomonas"
5483	celz	Cellulomonas shaoxiangyii	"Prokaryotes;Bacteria;Actinobacteria;Cellulomonas"
5484	cej	Cellulomonas sp. JZ18	"Prokaryotes;Bacteria;Actinobacteria;Cellulomonas"
5485	celh	Cellulomonas sp. H30R-01	"Prokaryotes;Bacteria;Actinobacteria;Cellulomonas"
5486	oek	Oerskovia sp. KBS0722	"Prokaryotes;Bacteria;Actinobacteria;Oerskovia"
5487	psei	Pseudactinotalea sp. HY158	"Prokaryotes;Bacteria;Actinobacteria;Pseudactinotalea"
5488	ica	Intrasporangium calvum	"Prokaryotes;Bacteria;Actinobacteria;Intrasporangium"
5489	ars	Arsenicicoccus sp. oral taxon 190	"Prokaryotes;Bacteria;Actinobacteria;Arsenicicoccus"
5490	jte	Janibacter indicus	"Prokaryotes;Bacteria;Actinobacteria;Janibacter"
5491	jli	Janibacter limosus	"Prokaryotes;Bacteria;Actinobacteria;Janibacter"
5492	jme	Janibacter melonis	"Prokaryotes;Bacteria;Actinobacteria;Janibacter"
5493	teh	Tetrasphaera sp. HKS02	"Prokaryotes;Bacteria;Actinobacteria;Tetrasphaera"
5494	phw	Phycicoccus sp. HDW14	"Prokaryotes;Bacteria;Actinobacteria;Phycicoccus"
5495	pei	Phycicoccus endophyticus	"Prokaryotes;Bacteria;Actinobacteria;Phycicoccus"
5496	serj	Serinicoccus hydrothermalis	"Prokaryotes;Bacteria;Actinobacteria;Serinicoccus"
5497	serw	Ornithinimicrobium pratense	"Prokaryotes;Bacteria;Actinobacteria;Serinicoccus"
5498	orn	Ornithinimicrobium sp. AMA3305	"Prokaryotes;Bacteria;Actinobacteria;Ornithinimicrobium"
5499	orz	Ornithinimicrobium sp. H23M54	"Prokaryotes;Bacteria;Actinobacteria;Ornithinimicrobium"
5500	bly	Brevibacterium linens BS258	"Prokaryotes;Bacteria;Actinobacteria;Brevibacterium"
5501	blin	Brevibacterium aurantiacum	"Prokaryotes;Bacteria;Actinobacteria;Brevibacterium"
5502	bri	Brevibacterium sp. CS2	"Prokaryotes;Bacteria;Actinobacteria;Brevibacterium"
5503	blut	Brevibacterium luteolum	"Prokaryotes;Bacteria;Actinobacteria;Brevibacterium"
5504	bcau	Brevibacterium casei	"Prokaryotes;Bacteria;Actinobacteria;Brevibacterium"
5505	dco	Dermatophilus congolensis	"Prokaryotes;Bacteria;Actinobacteria;Dermatophilus"
5506	aus	Austwickia sp. Fred_18-Q3-R57-64_BATAC.85v2	"Prokaryotes;Bacteria;Actinobacteria;Austwickia"
5507	gez	Georgenia sp. Z294	"Prokaryotes;Bacteria;Actinobacteria;Georgenia"
5508	halt	Haloactinobacterium sp. RN3S43	"Prokaryotes;Bacteria;Actinobacteria;Haloactinobacterium"
5509	pac	Cutibacterium acnes KPA171202	"Prokaryotes;Bacteria;Actinobacteria;Cutibacterium"
5510	pak	Cutibacterium acnes SK137	"Prokaryotes;Bacteria;Actinobacteria;Cutibacterium"
5511	pav	Cutibacterium acnes TypeIA2 P.acn17	"Prokaryotes;Bacteria;Actinobacteria;Cutibacterium"
5512	pax	Cutibacterium acnes TypeIA2 P.acn31	"Prokaryotes;Bacteria;Actinobacteria;Cutibacterium"
5513	paz	Cutibacterium acnes TypeIA2 P.acn33	"Prokaryotes;Bacteria;Actinobacteria;Cutibacterium"
5514	paw	Cutibacterium acnes 266	"Prokaryotes;Bacteria;Actinobacteria;Cutibacterium"
5515	pad	Cutibacterium acnes subsp. defendens ATCC 11828	"Prokaryotes;Bacteria;Actinobacteria;Cutibacterium"
5516	pcn	Cutibacterium acnes 6609	"Prokaryotes;Bacteria;Actinobacteria;Cutibacterium"
5517	pacc	Cutibacterium acnes C1	"Prokaryotes;Bacteria;Actinobacteria;Cutibacterium"
5518	pach	Cutibacterium acnes HL096PA1	"Prokaryotes;Bacteria;Actinobacteria;Cutibacterium"
5519	pacn	Cutibacterium acnes hdn-1	"Prokaryotes;Bacteria;Actinobacteria;Cutibacterium"
5520	cacn	Cutibacterium acnes KCOM 1861 (= ChDC B594)	"Prokaryotes;Bacteria;Actinobacteria;Cutibacterium"

5521	pra	Cutibacterium avidum	"Prokaryotes;Bacteria;Actinobacteria;Cutibacterium"
5522	cgrn	Cutibacterium granulosum	"Prokaryotes;Bacteria;Actinobacteria;Cutibacterium"
5523	pfr	Propionibacterium freudenreichii subsp. shermanii CIRM-BIA1	"Prokaryotes;Bacteria;Actinobacteria;Propionibacterium"
5524	pfre	Propionibacterium freudenreichii subsp. freudenreichii DSM 20271	"Prokaryotes;Bacteria;Actinobacteria;Propionibacterium"
5525	prl	Propionibacterium sp. oral taxon 193	"Prokaryotes;Bacteria;Actinobacteria;Propionibacterium"
5526	pacd	Propionibacterium acidifaciens	"Prokaryotes;Bacteria;Actinobacteria;Propionibacterium"
5527	paus	Propionibacterium australiense	"Prokaryotes;Bacteria;Actinobacteria;Propionibacterium"
5528	ppc	Pseudopropionibacterium propionicum	"Prokaryotes;Bacteria;Actinobacteria;Arachnia"
5529	pbo	Acidipropionibacterium acidipropionici ATCC 4875	"Prokaryotes;Bacteria;Actinobacteria;Acidipropionibacterium"
5530	aaci	Acidipropionibacterium acidipropionici CGMCC 1.2230	"Prokaryotes;Bacteria;Actinobacteria;Acidipropionibacterium"
5531	acij	Acidipropionibacterium virtanenii	"Prokaryotes;Bacteria;Actinobacteria;Acidipropionibacterium"
5532	aji	Acidipropionibacterium jensenii	"Prokaryotes;Bacteria;Actinobacteria;Acidipropionibacterium"
5533	mph	Microlunatus phosphovorus	"Prokaryotes;Bacteria;Actinobacteria;Microlunatus"
5534	mik	Microlunatus sp. KUDC0627	"Prokaryotes;Bacteria;Actinobacteria;Microlunatus"
5535	micg	Microlunatus sp. Gsoil 973	"Prokaryotes;Bacteria;Actinobacteria;Microlunatus"
5536	tfl	Tessaracoccus flavus	"Prokaryotes;Bacteria;Actinobacteria;Tessaracoccus"
5537	tfa	Tessaracoccus flavescens	"Prokaryotes;Bacteria;Actinobacteria;Tessaracoccus"
5538	tes	Tessaracoccus aquimaris	"Prokaryotes;Bacteria;Actinobacteria;Tessaracoccus"
5539	tez	Tessaracoccus sp. T2.5-30	"Prokaryotes;Bacteria;Actinobacteria;Tessaracoccus"
5540	tdf	Tessaracoccus defluvii	"Prokaryotes;Bacteria;Actinobacteria;Tessaracoccus"
5541	tla	Tessaracoccus lapidicaptus	"Prokaryotes;Bacteria;Actinobacteria;Tessaracoccus"
5542	rain	Raineyella sp. CBA3103	"Prokaryotes;Bacteria;Actinobacteria;Raineyella"
5543	prv	Propioniciclava sp. HDW11	"Prokaryotes;Bacteria;Actinobacteria;Propioniciclava"
5544	nca	Nocardioides sp. JS614	"Prokaryotes;Bacteria;Actinobacteria;Nocardioides"
5545	ndk	Nocardioides dokdonensis	"Prokaryotes;Bacteria;Actinobacteria;Nocardioides"
5546	noy	Nocardioides euryhalodurans	"Prokaryotes;Bacteria;Actinobacteria;Nocardioides"
5547	noi	Nocardioides sp. dk3136	"Prokaryotes;Bacteria;Actinobacteria;Nocardioides"
5548	noo	Nocardioides sp. S-1144	"Prokaryotes;Bacteria;Actinobacteria;Nocardioides"
5549	ndp	Nocardioides daphniae	"Prokaryotes;Bacteria;Actinobacteria;Nocardioides"
5550	nsn	Nocardioides seonyuensis	"Prokaryotes;Bacteria;Actinobacteria;Nocardioides"
5551	nbe	Nocardioides baekrokdamisoli	"Prokaryotes;Bacteria;Actinobacteria;Nocardioides"
5552	nano	Nocardioides anomalus	"Prokaryotes;Bacteria;Actinobacteria;Nocardioides"
5553	nmes	Nocardioides mesophilus	"Prokaryotes;Bacteria;Actinobacteria;Nocardioides"
5554	psim	Pimelobacter simplex	"Prokaryotes;Bacteria;Actinobacteria;Pimelobacter"
5555	aer	Aeromicrobium erythreum	"Prokaryotes;Bacteria;Actinobacteria;Aeromicrobium"
5556	aez	Aeromicrobium chenweiae	"Prokaryotes;Bacteria;Actinobacteria;Aeromicrobium"
5557	aeb	Aeromicrobium sp. A1-2	"Prokaryotes;Bacteria;Actinobacteria;Aeromicrobium"
5558	aef	Aeromicrobium yanjiei	"Prokaryotes;Bacteria;Actinobacteria;Aeromicrobium"
5559	mgg	Micropruina glycogenica	"Prokaryotes;Bacteria;Actinobacteria;Micropruina"
5560	muz	Mumia sp. ZJ1417	"Prokaryotes;Bacteria;Actinobacteria;Mumia"
5561	kfl	Kribbella flavida	"Prokaryotes;Bacteria;Actinobacteria;Kribbella"

5562	kqi	Kribbella qitaiheensis	"Prokaryotes;Bacteria;Actinobacteria;Kribbella"
5563	tfu	Thermobifida fusca	"Prokaryotes;Bacteria;Actinobacteria;Thermobifida"
5564	nda	Nocardiopsis dassonvillei	"Prokaryotes;Bacteria;Actinobacteria;Nocardiopsis"
5565	nal	Nocardiopsis alba	"Prokaryotes;Bacteria;Actinobacteria;Nocardiopsis"
5566	ngv	Nocardiopsis gilva	"Prokaryotes;Bacteria;Actinobacteria;Nocardiopsis"
5567	nfe	Nocardiopsis flavescens	"Prokaryotes;Bacteria;Actinobacteria;Nocardiopsis"
5568	strr	Streptomonospora sp. M2	"Prokaryotes;Bacteria;Actinobacteria;Streptomonospora"
5569	tcu	Thermomonospora curvata	"Prokaryotes;Bacteria;Actinobacteria;Thermomonospora"
5570	actw	Actinomadura sp. WMMB499	"Prokaryotes;Bacteria;Actinobacteria;Actinomadura"
5571	sro	Streptosporangium roseum	"Prokaryotes;Bacteria;Actinobacteria;Streptosporangium"
5572	noa	Nonomuraea sp. ATCC 55076	"Prokaryotes;Bacteria;Actinobacteria;Nonomuraea"
5573	now	Nonomuraea sp. WYY166	"Prokaryotes;Bacteria;Actinobacteria;Nonomuraea"
5574	tbi	Thermobispora bispora	"Prokaryotes;Bacteria;Actinobacteria;Thermobispora"
5575	fra	Frankia casuarinae	"Prokaryotes;Bacteria;Actinobacteria;Frankia"
5576	fre	Frankia sp. EAN1pec	"Prokaryotes;Bacteria;Actinobacteria;Frankia"
5577	fri	Frankia inefficax	"Prokaryotes;Bacteria;Actinobacteria;Frankia"
5578	fal	Frankia alni	"Prokaryotes;Bacteria;Actinobacteria;Frankia"
5579	fsy	Candidatus Frankia datiscae	"Prokaryotes;Bacteria;Actinobacteria;Frankia"
5580	ace	Acidothermus cellulolyticus	"Prokaryotes;Bacteria;Actinobacteria;Acidothermus"
5581	nml	Nakamurella multipartita	"Prokaryotes;Bacteria;Actinobacteria;Nakamurella"
5582	nak	Nakamurella sp. s14-144	"Prokaryotes;Bacteria;Actinobacteria;Nakamurella"
5583	gob	Geodermatophilus obscurus	"Prokaryotes;Bacteria;Actinobacteria;Geodermatophilus"
5584	bsd	Blastococcus saxobsidens	"Prokaryotes;Bacteria;Actinobacteria;Blastococcus"
5585	mmar	Modestobacter marinus	"Prokaryotes;Bacteria;Actinobacteria;Modestobacter"
5586	kra	Kineococcus radiotolerans	"Prokaryotes;Bacteria;Actinobacteria;Kineococcus"
5587	sen	Saccharopolyspora erythraea	"Prokaryotes;Bacteria;Actinobacteria;Saccharopolyspora"
5588	sace	Saccharopolyspora coralli	"Prokaryotes;Bacteria;Actinobacteria;Saccharopolyspora"
5589	sacg	Saccharopolyspora sp. ASAGF58	"Prokaryotes;Bacteria;Actinobacteria;Saccharopolyspora"
5590	svi	Saccharomonospora viridis	"Prokaryotes;Bacteria;Actinobacteria;Saccharomonospora"
5591	sacc	Saccharomonospora xinjiangensis	"Prokaryotes;Bacteria;Actinobacteria;Saccharomonospora"
5592	amd	Amycolatopsis mediterranei U32	"Prokaryotes;Bacteria;Actinobacteria;Amycolatopsis"
5593	amn	Amycolatopsis mediterranei S699	"Prokaryotes;Bacteria;Actinobacteria;Amycolatopsis"
5594	amm	Amycolatopsis mediterranei S699	"Prokaryotes;Bacteria;Actinobacteria;Amycolatopsis"
5595	amz	Amycolatopsis mediterranei RB	"Prokaryotes;Bacteria;Actinobacteria;Amycolatopsis"
5596	aoi	Amycolatopsis keratiniphila	"Prokaryotes;Bacteria;Actinobacteria;Amycolatopsis"
5597	aja	Amycolatopsis japonica	"Prokaryotes;Bacteria;Actinobacteria;Amycolatopsis"
5598	amq	Amycolatopsis methanolica	"Prokaryotes;Bacteria;Actinobacteria;Amycolatopsis"
5599	amyc	Amycolatopsis sp. AA4	"Prokaryotes;Bacteria;Actinobacteria;Amycolatopsis"
5600	amyb	Amycolatopsis sp. BJA-103	"Prokaryotes;Bacteria;Actinobacteria;Amycolatopsis"
5601	aab	Amycolatopsis albispora	"Prokaryotes;Bacteria;Actinobacteria;Amycolatopsis"
5602	amyy	Amycolatopsis sp. YIM 10	"Prokaryotes;Bacteria;Actinobacteria;Amycolatopsis"

5603	aori	Amycolatopsis orientalis	"Prokaryotes;Bacteria;Actinobacteria;Amycolatopsis"
5604	pdx	Pseudonocardia dioxanivorans	"Prokaryotes;Bacteria;Actinobacteria;Pseudonocardia"
5605	psea	Pseudonocardia sp. AL041005-10	"Prokaryotes;Bacteria;Actinobacteria;Pseudonocardia"
5606	psee	Pseudonocardia sp. EC080625-04	"Prokaryotes;Bacteria;Actinobacteria;Pseudonocardia"
5607	pseh	Pseudonocardia sp. HH130629-09	"Prokaryotes;Bacteria;Actinobacteria;Pseudonocardia"
5608	pseq	Pseudonocardia sp. EC080610-09	"Prokaryotes;Bacteria;Actinobacteria;Pseudonocardia"
5609	pecq	Pseudonocardia sp EC080619-01	"Prokaryotes;Bacteria;Actinobacteria;Pseudonocardia"
5610	phh	Pseudonocardia sp. HH130630-07	"Prokaryotes;Bacteria;Actinobacteria;Pseudonocardia"
5611	paut	Pseudonocardia autotrophica	"Prokaryotes;Bacteria;Actinobacteria;Pseudonocardia"
5612	apre	Actinosynnema pretiosum	"Prokaryotes;Bacteria;Actinobacteria;Actinosynnema"
5613	ami	Actinosynnema mirum	"Prokaryotes;Bacteria;Actinobacteria;Actinosynnema"
5614	sesp	Saccharothrix espanaensis	"Prokaryotes;Bacteria;Actinobacteria;Saccharothrix"
5615	ssyi	Saccharothrix syringae	"Prokaryotes;Bacteria;Actinobacteria;Saccharothrix"
5616	kal	Kutzneria albida	"Prokaryotes;Bacteria;Actinobacteria;Kutzneria"
5617	kphy	Kibdelosporangium phytohabitans	"Prokaryotes;Bacteria;Actinobacteria;Kibdelosporangium"
5618	led	Lentzea guizhouensis	"Prokaryotes;Bacteria;Actinobacteria;Lentzea"
5619	ahm	Actinoalloteichus hymeniacidonis	"Prokaryotes;Bacteria;Actinobacteria;Actinoalloteichus"
5620	acti	Actinoalloteichus sp. GBA129-24	"Prokaryotes;Bacteria;Actinobacteria;Actinoalloteichus"
5621	acad	Actinoalloteichus fjordicus	"Prokaryotes;Bacteria;Actinobacteria;Actinoalloteichus"
5622	ahg	Actinoalloteichus hoggarensis	"Prokaryotes;Bacteria;Actinobacteria;Actinoalloteichus"
5623	acta	Actinoalloteichus sp. AHMU CJ021	"Prokaryotes;Bacteria;Actinobacteria;Actinoalloteichus"
5624	alo	Alloactinosynnema sp. L-07	"Prokaryotes;Bacteria;Actinobacteria;Alloactinosynnema"
5625	pmad	Prauserella marina	"Prokaryotes;Bacteria;Actinobacteria;Prauserella"
5626	stp	Salinispora tropica	"Prokaryotes;Bacteria;Actinobacteria;Salinispora"
5627	saq	Salinispora arenicola	"Prokaryotes;Bacteria;Actinobacteria;Salinispora"
5628	mau	Micromonospora aurantiaca	"Prokaryotes;Bacteria;Actinobacteria;Micromonospora"
5629	mil	Micromonospora sp. L5	"Prokaryotes;Bacteria;Actinobacteria;Micromonospora"
5630	micb	Micromonospora sp. B006	"Prokaryotes;Bacteria;Actinobacteria;Micromonospora"
5631	mtua	Micromonospora tulbaghiae	"Prokaryotes;Bacteria;Actinobacteria;Micromonospora"
5632	mich	Micromonospora sp. HM134	"Prokaryotes;Bacteria;Actinobacteria;Micromonospora"
5633	mtem	Micromonospora terminaliae	"Prokaryotes;Bacteria;Actinobacteria;Micromonospora"
5634	mcab	Micromonospora carbonacea	"Prokaryotes;Bacteria;Actinobacteria;Micromonospora"
5635	msag	Micromonospora sagamiensis	"Prokaryotes;Bacteria;Actinobacteria;Micromonospora"
5636	vma	Micromonospora maris	"Prokaryotes;Bacteria;Actinobacteria;Micromonospora"
5637	mcra	Micromonospora craniellae	"Prokaryotes;Bacteria;Actinobacteria;Micromonospora"
5638	ase	Actinoplanes sp. SE50/110	"Prokaryotes;Bacteria;Actinobacteria;Actinoplanes"
5639	ams	Actinoplanes missouriensis	"Prokaryotes;Bacteria;Actinobacteria;Actinoplanes"
5640	actn	Actinoplanes sp. N902-109	"Prokaryotes;Bacteria;Actinobacteria;Actinoplanes"
5641	afs	Actinoplanes friuliensis	"Prokaryotes;Bacteria;Actinobacteria;Actinoplanes"
5642	acts	Actinoplanes sp. SE50	"Prokaryotes;Bacteria;Actinobacteria;Actinoplanes"
5643	plk	Plantactinospora sp. KBS50	"Prokaryotes;Bacteria;Actinobacteria;Plantactinospora"

5644	plab	Plantactinospora sp. BC1	"Prokaryotes;Bacteria;Actinobacteria;Plantactinospora"
5645	plat	Plantactinospora sp. BB1	"Prokaryotes;Bacteria;Actinobacteria;Plantactinospora"
5646	pfla	Phytohabitans flavus	"Prokaryotes;Bacteria;Actinobacteria;Phytohabitans"
5647	psuu	Phytohabitans suffuscus	"Prokaryotes;Bacteria;Actinobacteria;Phytohabitans"
5648	ver	Verrucosispora sp. NA02020	"Prokaryotes;Bacteria;Actinobacteria;Verrucosispora"
5649	cai	Catenulispora acidiphila	"Prokaryotes;Bacteria;Actinobacteria;Catenulispora"
5650	sna	Stackebrandtia nassauensis	"Prokaryotes;Bacteria;Actinobacteria;Stackebrandtia"
5651	ahe	Arcanobacterium haemolyticum	"Prokaryotes;Bacteria;Actinobacteria;Arcanobacterium"
5652	arca	Arcanobacterium sp. 2701	"Prokaryotes;Bacteria;Actinobacteria;Arcanobacterium"
5653	mcu	Mobiluncus curtisii	"Prokaryotes;Bacteria;Actinobacteria;Mobiluncus"
5654	tpy	Trueperella pyogenes TP6375	"Prokaryotes;Bacteria;Actinobacteria;Trueperella"
5655	tpyo	Trueperella pyogenes TP8	"Prokaryotes;Bacteria;Actinobacteria;Trueperella"
5656	tbw	Trueperella bialowiezensis	"Prokaryotes;Bacteria;Actinobacteria;Trueperella"
5657	asg	Actinotignum schaalii	"Prokaryotes;Bacteria;Actinobacteria;Actinotignum"
5658	actt	Actinobaculum sp. 313	"Prokaryotes;Bacteria;Actinobacteria;Actinotignum"
5659	amy	Schaalia meyeri	"Prokaryotes;Bacteria;Actinobacteria;Schaalia"
5660	soo	Schaalia odontolytica	"Prokaryotes;Bacteria;Actinobacteria;Schaalia"
5661	acq	Actinomyces sp. oral taxon 414	"Prokaryotes;Bacteria;Actinobacteria;Actinomyces"
5662	aos	Actinomyces oris	"Prokaryotes;Bacteria;Actinobacteria;Actinomyces"
5663	ard	Actinomyces radicidentis	"Prokaryotes;Bacteria;Actinobacteria;Actinomyces"
5664	actp	Actinomyces gaoshouyii	"Prokaryotes;Bacteria;Actinobacteria;Actinomyces"
5665	actc	Actinomyces sp. Chiba101	"Prokaryotes;Bacteria;Actinobacteria;Actinomyces"
5666	acto	Actinomyces sp. oral taxon 897	"Prokaryotes;Bacteria;Actinobacteria;Actinomyces"
5667	ane	Actinomyces naeslundii	"Prokaryotes;Bacteria;Actinobacteria;Actinomyces"
5668	ahw	Actinomyces howellii	"Prokaryotes;Bacteria;Actinobacteria;Actinomyces"
5669	actz	Actinomyces sp. 432	"Prokaryotes;Bacteria;Actinobacteria;Actinomyces"
5670	air	Actinomyces israelii	"Prokaryotes;Bacteria;Actinobacteria;Actinomyces"
5671	asla	Actinomyces slackii	"Prokaryotes;Bacteria;Actinobacteria;Actinomyces"
5672	avc	Actinomyces viscosus	"Prokaryotes;Bacteria;Actinobacteria;Actinomyces"
5673	avu	Boudabousia tangfeifanii	"Prokaryotes;Bacteria;Actinobacteria;Boudabousia"
5674	fsl	Flaviflexus salsibiostraticola	"Prokaryotes;Bacteria;Actinobacteria;Flaviflexus"
5675	flh	Flaviflexus sp. H23T48	"Prokaryotes;Bacteria;Actinobacteria;Flaviflexus"
5676	wik	Winkia sp. C64	"Prokaryotes;Bacteria;Actinobacteria;Winkia"
5677	fvg	Fannyhessea vaginae	"Prokaryotes;Bacteria;Actinobacteria;Fannyhessea"
5678	blo	Bifidobacterium longum NCC2705	"Prokaryotes;Bacteria;Actinobacteria;Bifidobacterium"
5679	blj	Bifidobacterium longum DJO10A	"Prokaryotes;Bacteria;Actinobacteria;Bifidobacterium"
5680	bln	Bifidobacterium longum subsp. infantis ATCC 15697 (JGI)	"Prokaryotes;Bacteria;Actinobacteria;Bifidobacterium"
5681	blon	Bifidobacterium longum subsp. infantis ATCC 15697 (Tokyo)	"Prokaryotes;Bacteria;Actinobacteria;Bifidobacterium"
5682	blf	Bifidobacterium longum subsp. infantis 157F	"Prokaryotes;Bacteria;Actinobacteria;Bifidobacterium"
5683	bll	Bifidobacterium longum subsp. longum JDM301	"Prokaryotes;Bacteria;Actinobacteria;Bifidobacterium"
5684	blb	Bifidobacterium longum subsp. longum BBMN68	"Prokaryotes;Bacteria;Actinobacteria;Bifidobacterium"

5685	blm	Bifidobacterium longum subsp. longum JCM 1217	"Prokaryotes;Bacteria;Actinobacteria;Bifidobacterium"
5686	blk	Bifidobacterium longum subsp. longum KACC 91563	"Prokaryotes;Bacteria;Actinobacteria;Bifidobacterium"
5687	blg	Bifidobacterium longum subsp. longum F8	"Prokaryotes;Bacteria;Actinobacteria;Bifidobacterium"
5688	blz	Bifidobacterium longum subsp. longum GT15	"Prokaryotes;Bacteria;Actinobacteria;Bifidobacterium"
5689	blx	Bifidobacterium longum BXY01	"Prokaryotes;Bacteria;Actinobacteria;Bifidobacterium"
5690	bad	Bifidobacterium adolescentis ATCC 15703	"Prokaryotes;Bacteria;Actinobacteria;Bifidobacterium"
5691	badl	Bifidobacterium adolescentis 22L	"Prokaryotes;Bacteria;Actinobacteria;Bifidobacterium"
5692	bado	Bifidobacterium adolescentis BBMN23	"Prokaryotes;Bacteria;Actinobacteria;Bifidobacterium"
5693	bla	Bifidobacterium animalis subsp. lactis AD011	"Prokaryotes;Bacteria;Actinobacteria;Bifidobacterium"
5694	blc	Bifidobacterium animalis subsp. lactis Bl-04	"Prokaryotes;Bacteria;Actinobacteria;Bifidobacterium"
5695	blt	Bifidobacterium animalis subsp. lactis DSM 10140	"Prokaryotes;Bacteria;Actinobacteria;Bifidobacterium"
5696	bbb	Bifidobacterium animalis subsp. lactis BB-12	"Prokaryotes;Bacteria;Actinobacteria;Bifidobacterium"
5697	bbc	Bifidobacterium animalis subsp. lactis BLC1	"Prokaryotes;Bacteria;Actinobacteria;Bifidobacterium"
5698	bnm	Bifidobacterium animalis subsp. lactis CNCM I-2494	"Prokaryotes;Bacteria;Actinobacteria;Bifidobacterium"
5699	blv	Bifidobacterium animalis subsp. lactis V9	"Prokaryotes;Bacteria;Actinobacteria;Bifidobacterium"
5700	blw	Bifidobacterium animalis subsp. lactis B420	"Prokaryotes;Bacteria;Actinobacteria;Bifidobacterium"
5701	bls	Bifidobacterium animalis subsp. lactis Bi-07	"Prokaryotes;Bacteria;Actinobacteria;Bifidobacterium"
5702	bani	Bifidobacterium animalis subsp. lactis Bl12	"Prokaryotes;Bacteria;Actinobacteria;Bifidobacterium"
5703	banl	Bifidobacterium animalis subsp. lactis ATCC 27673	"Prokaryotes;Bacteria;Actinobacteria;Bifidobacterium"
5704	bni	Bifidobacterium animalis subsp. animalis ATCC 25527	"Prokaryotes;Bacteria;Actinobacteria;Bifidobacterium"
5705	banm	Bifidobacterium animalis RH	"Prokaryotes;Bacteria;Actinobacteria;Bifidobacterium"
5706	bde	Bifidobacterium dentium Bd1	"Prokaryotes;Bacteria;Actinobacteria;Bifidobacterium"
5707	bdn	Bifidobacterium dentium JCM 1195 = DSM 20436	"Prokaryotes;Bacteria;Actinobacteria;Bifidobacterium"
5708	bbp	Bifidobacterium bifidum PRL2010	"Prokaryotes;Bacteria;Actinobacteria;Bifidobacterium"
5709	bbi	Bifidobacterium bifidum S17	"Prokaryotes;Bacteria;Actinobacteria;Bifidobacterium"
5710	bbf	Bifidobacterium bifidum BGN4	"Prokaryotes;Bacteria;Actinobacteria;Bifidobacterium"
5711	bbv	Bifidobacterium breve ACS-071-V-Sch8b	"Prokaryotes;Bacteria;Actinobacteria;Bifidobacterium"
5712	bbru	Bifidobacterium breve UCC2003	"Prokaryotes;Bacteria;Actinobacteria;Bifidobacterium"
5713	bbre	Bifidobacterium breve 12L	"Prokaryotes;Bacteria;Actinobacteria;Bifidobacterium"
5714	bbrv	Bifidobacterium breve 689b	"Prokaryotes;Bacteria;Actinobacteria;Bifidobacterium"
5715	bbrj	Bifidobacterium breve JCM 7017	"Prokaryotes;Bacteria;Actinobacteria;Bifidobacterium"
5716	bbrc	Bifidobacterium breve JCM 7019	"Prokaryotes;Bacteria;Actinobacteria;Bifidobacterium"
5717	bbrn	Bifidobacterium breve NCFB 2258	"Prokaryotes;Bacteria;Actinobacteria;Bifidobacterium"
5718	bbrs	Bifidobacterium breve S27	"Prokaryotes;Bacteria;Actinobacteria;Bifidobacterium"
5719	bbrd	Bifidobacterium breve DSM 20213 = JCM 1192	"Prokaryotes;Bacteria;Actinobacteria;Bifidobacterium"
5720	bast	Bifidobacterium asteroides	"Prokaryotes;Bacteria;Actinobacteria;Bifidobacterium"
5721	btp	Bifidobacterium thermophilum	"Prokaryotes;Bacteria;Actinobacteria;Bifidobacterium"
5722	bcor	Bifidobacterium coryneforme	"Prokaryotes;Bacteria;Actinobacteria;Bifidobacterium"
5723	bka	Bifidobacterium kashiwanohense PV20-2	"Prokaryotes;Bacteria;Actinobacteria;Bifidobacterium"
5724	bks	Bifidobacterium kashiwanohense JCM 15439 = DSM 21854	"Prokaryotes;Bacteria;Actinobacteria;Bifidobacterium"
5725	bcat	Bifidobacterium catenulatum	"Prokaryotes;Bacteria;Actinobacteria;Bifidobacterium"

5726	bpsp	Bifidobacterium pseudolongum	"Prokaryotes;Bacteria;Actinobacteria;Bifidobacterium"
5727	bii	Bifidobacterium indicum	"Prokaryotes;Bacteria;Actinobacteria;Bifidobacterium"
5728	bang	Bifidobacterium angulatum	"Prokaryotes;Bacteria;Actinobacteria;Bifidobacterium"
5729	bpsc	Bifidobacterium pseudocatenulatum	"Prokaryotes;Bacteria;Actinobacteria;Bifidobacterium"
5730	bsca	Bifidobacterium scardovii	"Prokaryotes;Bacteria;Actinobacteria;Bifidobacterium"
5731	bact	Bifidobacterium actinocoloniiforme	"Prokaryotes;Bacteria;Actinobacteria;Bifidobacterium"
5732	bcho	Bifidobacterium choerinum	"Prokaryotes;Bacteria;Actinobacteria;Bifidobacterium"
5733	bgx	Bifidobacterium pullorum subsp. gallinarum	"Prokaryotes;Bacteria;Actinobacteria;Bifidobacterium"
5734	blem	Bifidobacterium lemurum	"Prokaryotes;Bacteria;Actinobacteria;Bifidobacterium"
5735	beu	Bifidobacterium eulemuris	"Prokaryotes;Bacteria;Actinobacteria;Bifidobacterium"
5736	gvg	Gardnerella vaginalis ATCC 14019	"Prokaryotes;Bacteria;Actinobacteria;Gardnerella"
5737	gva	Gardnerella vaginalis 409-05	"Prokaryotes;Bacteria;Actinobacteria;Gardnerella"
5738	gvh	Gardnerella vaginalis HMP9231	"Prokaryotes;Bacteria;Actinobacteria;Gardnerella"
5739	sij	Scardovia inopinata	"Prokaryotes;Bacteria;Actinobacteria;Scardovia"
5740	pdo	Parascardovia denticolens	"Prokaryotes;Bacteria;Actinobacteria;Parascardovia"
5741	aey	Actinopolyspora erythraea	"Prokaryotes;Bacteria;Actinobacteria;Actinopolyspora"
5742	plan	Candidatus Planktophila lacus	"Prokaryotes;Bacteria;Actinobacteria;Planktophila"
5743	plak	Candidatus Planktophila dulcis	"Prokaryotes;Bacteria;Actinobacteria;Planktophila"
5744	plim	Candidatus Planktophila limnetica	"Prokaryotes;Bacteria;Actinobacteria;Planktophila"
5745	psuf	Candidatus Planktophila sulfonica	"Prokaryotes;Bacteria;Actinobacteria;Planktophila"
5746	pvs	Candidatus Planktophila versatilis	"Prokaryotes;Bacteria;Actinobacteria;Planktophila"
5747	pvn	Candidatus Planktophila vernalis	"Prokaryotes;Bacteria;Actinobacteria;Planktophila"
5748	abam	Candidatus Nanopelagicus limnes	"Prokaryotes;Bacteria;Actinobacteria;Nanopelagicus"
5749	nhi	Candidatus Nanopelagicus hibericus	"Prokaryotes;Bacteria;Actinobacteria;Nanopelagicus"
5750	nab	Candidatus Nanopelagicus abundans	"Prokaryotes;Bacteria;Actinobacteria;Nanopelagicus"
5751	eke	Epidermidibacterium keratini	"Prokaryotes;Bacteria;Actinobacteria;Epidermidibacterium"
5752	abai	Actinobacteria bacterium	"Prokaryotes;Bacteria;Actinobacteria;unclassified Actinobacteria"
5753	rxy	Rubrobacter xylanophilus	"Prokaryotes;Bacteria;Actinobacteria;Rubrobacter"
5754	rrd	Rubrobacter radiotolerans	"Prokaryotes;Bacteria;Actinobacteria;Rubrobacter"
5755	rub	Rubrobacter sp. SCSIO 52909	"Prokaryotes;Bacteria;Actinobacteria;Rubrobacter"
5756	bsol	Baekduia soli	"Prokaryotes;Bacteria;Actinobacteria;Baekduia"
5757	cwo	Conexibacter woesei	"Prokaryotes;Bacteria;Actinobacteria;Conexibacter"
5758	afo	Acidimicrobium ferrooxidans	"Prokaryotes;Bacteria;Actinobacteria;Acidimicrobium"
5759	aym	Ilumatobacter coccineus	"Prokaryotes;Bacteria;Actinobacteria;Ilumatobacter"
5760	atq	Actinomarinicola tropica	"Prokaryotes;Bacteria;Actinobacteria;Actinomarinicola"
5761	ccu	Cryptobacterium curtum	"Prokaryotes;Bacteria;Actinobacteria;Cryptobacterium"
5762	shi	Slackia heliotrinireducens	"Prokaryotes;Bacteria;Actinobacteria;Slackia"
5763	ele	Eggerthella lenta	"Prokaryotes;Bacteria;Actinobacteria;Eggerthella"
5764	eyy	Eggerthella sp. YY7918	"Prokaryotes;Bacteria;Actinobacteria;Eggerthella"
5765	gpa	Gordonibacter pamelaeae	"Prokaryotes;Bacteria;Actinobacteria;Gordonibacter"
5766	aeq	Adlercreutzia equolifaciens	"Prokaryotes;Bacteria;Actinobacteria;Adlercreutzia"

5767	ddt	Denitrobacterium detoxificans	"Prokaryotes;Bacteria;Actinobacteria;Denitrobacterium"
5768	cbac	Berryella intestinalis	"Prokaryotes;Bacteria;Actinobacteria;Berryella"
5769	apv	Atopobium parvulum	"Prokaryotes;Bacteria;Actinobacteria;Lancefieldella"
5770	ols	Olsenella uli	"Prokaryotes;Bacteria;Actinobacteria;Olsenella"
5771	olo	Olsenella sp. oral taxon 807	"Prokaryotes;Bacteria;Actinobacteria;Olsenella"
5772	pcat	Parolsenella catena	"Prokaryotes;Bacteria;Actinobacteria;Parolsenella"
5773	cgo	Coriobacterium glomerans	"Prokaryotes;Bacteria;Actinobacteria;Coriobacterium"
5774	caer	Collinsella aerofaciens	"Prokaryotes;Bacteria;Actinobacteria;Collinsella"
5775	erz	Egibacter rhizosphaerae	"Prokaryotes;Bacteria;Actinobacteria;Egibacter"
5776	euz	Euzebya sp. DY32-46	"Prokaryotes;Bacteria;Actinobacteria;Euzebya"
5777	syn	Synechocystis sp. PCC 6803	"Prokaryotes;Bacteria;Cyanobacteria;Synechocystis"
5778	syz	Synechocystis sp. PCC 6803	"Prokaryotes;Bacteria;Cyanobacteria;Synechocystis"
5779	syy	Synechocystis sp. PCC 6803 GT-S	"Prokaryotes;Bacteria;Cyanobacteria;Synechocystis"
5780	syt	Synechocystis sp. PCC 6803 GT-I	"Prokaryotes;Bacteria;Cyanobacteria;Synechocystis"
5781	sys	Synechocystis sp. PCC 6803 PCC-N	"Prokaryotes;Bacteria;Cyanobacteria;Synechocystis"
5782	syq	Synechocystis sp. PCC 6803 PCC-P	"Prokaryotes;Bacteria;Cyanobacteria;Synechocystis"
5783	syj	Synechocystis sp. PCC 6714	"Prokaryotes;Bacteria;Cyanobacteria;Synechocystis"
5784	syo	Synechocystis sp. IPPAS B-1465	"Prokaryotes;Bacteria;Cyanobacteria;Synechocystis"
5785	syc	Synechococcus elongatus PCC6301	"Prokaryotes;Bacteria;Cyanobacteria;Synechococcus"
5786	syf	Synechococcus elongatus PCC7942	"Prokaryotes;Bacteria;Cyanobacteria;Synechococcus"
5787	syw	Synechococcus sp. WH8102	"Prokaryotes;Bacteria;Cyanobacteria;Synechococcus"
5788	syd	Synechococcus sp. CC9605	"Prokaryotes;Bacteria;Cyanobacteria;Synechococcus"
5789	sye	Synechococcus sp. CC9902	"Prokaryotes;Bacteria;Cyanobacteria;Synechococcus"
5790	syg	Synechococcus sp. CC9311	"Prokaryotes;Bacteria;Cyanobacteria;Synechococcus"
5791	syr	Synechococcus sp. RCC307	"Prokaryotes;Bacteria;Cyanobacteria;Synechococcus"
5792	syx	Synechococcus sp. WH 7803	"Prokaryotes;Bacteria;Cyanobacteria;Synechococcus"
5793	syp	Synechococcus sp. PCC7002	"Prokaryotes;Bacteria;Cyanobacteria;Synechococcus"
5794	cya	Synechococcus sp. JA-3-3Ab	"Prokaryotes;Bacteria;Cyanobacteria;Synechococcus"
5795	cyb	Synechococcus sp. JA-2-3B'a(2-13)	"Prokaryotes;Bacteria;Cyanobacteria;Synechococcus"
5796	syne	Synechococcus sp. PCC 6312	"Prokaryotes;Bacteria;Cyanobacteria;Synechococcus"
5797	synp	Synechococcus sp. PCC 7502	"Prokaryotes;Bacteria;Cyanobacteria;Synechococcus"
5798	synk	Synechococcus sp. KORDI-100	"Prokaryotes;Bacteria;Cyanobacteria;Synechococcus"
5799	synr	Synechococcus sp. KORDI-49	"Prokaryotes;Bacteria;Cyanobacteria;Synechococcus"
5800	synd	Synechococcus sp. KORDI-52	"Prokaryotes;Bacteria;Cyanobacteria;Synechococcus"
5801	syu	Synechococcus sp. UTEX 2973	"Prokaryotes;Bacteria;Cyanobacteria;Synechococcus"
5802	syh	Synechococcus sp. WH 8109	"Prokaryotes;Bacteria;Cyanobacteria;Synechococcus"
5803	synw	Synechococcus sp. WH 8103	"Prokaryotes;Bacteria;Cyanobacteria;Synechococcus"
5804	slw	Synechococcus lividus	"Prokaryotes;Bacteria;Cyanobacteria;Synechococcus"
5805	syv	Synechococcus sp. PCC 73109	"Prokaryotes;Bacteria;Cyanobacteria;Synechococcus"
5806	syl	Synechococcus sp. PCC 7003	"Prokaryotes;Bacteria;Cyanobacteria;Synechococcus"
5807	sync	Synechococcus sp. CB0101	"Prokaryotes;Bacteria;Cyanobacteria;Synechococcus"

5808	tel	Thermosynechococcus elongatus	"Prokaryotes;Bacteria;Cyanobacteria;Thermosynechococcus"
5809	thn	Thermosynechococcus sp. NK55	"Prokaryotes;Bacteria;Cyanobacteria;Thermosynechococcus"
5810	tvn	Thermosynechococcus vulcanus	"Prokaryotes;Bacteria;Cyanobacteria;Thermosynechococcus"
5811	thec	Thermosynechococcus sp. CL-1	"Prokaryotes;Bacteria;Cyanobacteria;Thermosynechococcus"
5812	cgc	Cyanobium gracile	"Prokaryotes;Bacteria;Cyanobacteria;Cyanobium"
5813	cyi	Cyanobium sp. NIES-981	"Prokaryotes;Bacteria;Cyanobacteria;Cyanobium"
5814	dsl	Dactylococcopsis salina	"Prokaryotes;Bacteria;Cyanobacteria;Dactylococcopsis"
5815	cmp	Chamaesiphon minutus	"Prokaryotes;Bacteria;Cyanobacteria;Chamaesiphon"
5816	lep	Leptolyngbya sp. PCC 7376	"Prokaryotes;Bacteria;Cyanobacteria;Leptolyngbya"
5817	len	Leptolyngbya sp. NIES-3755	"Prokaryotes;Bacteria;Cyanobacteria;Leptolyngbya"
5818	let	Leptolyngbya sp. O-77	"Prokaryotes;Bacteria;Cyanobacteria;Leptolyngbya"
5819	lbo	Leptolyngbya boryana	"Prokaryotes;Bacteria;Cyanobacteria;Leptolyngbya"
5820	hhg	Halomicronema hongdechloris	"Prokaryotes;Bacteria;Cyanobacteria;Halomicronema"
5821	pseu	Pseudanabaena sp. PCC 7367	"Prokaryotes;Bacteria;Cyanobacteria;Pseudanabaena"
5822	pser	Pseudanabaena sp. ABRG5-3	"Prokaryotes;Bacteria;Cyanobacteria;Pseudanabaena"
5823	pma	Prochlorococcus marinus subsp. marinus CCMP1375	"Prokaryotes;Bacteria;Cyanobacteria;Prochlorococcus"
5824	pmm	Prochlorococcus marinus subsp. pastoris CCMP1986	"Prokaryotes;Bacteria;Cyanobacteria;Prochlorococcus"
5825	pmt	Prochlorococcus marinus MIT 9313	"Prokaryotes;Bacteria;Cyanobacteria;Prochlorococcus"
5826	pmn	Prochlorococcus marinus NATL2A	"Prokaryotes;Bacteria;Cyanobacteria;Prochlorococcus"
5827	pmi	Prochlorococcus marinus MIT 9312	"Prokaryotes;Bacteria;Cyanobacteria;Prochlorococcus"
5828	pmb	Prochlorococcus marinus AS9601	"Prokaryotes;Bacteria;Cyanobacteria;Prochlorococcus"
5829	pmc	Prochlorococcus marinus MIT 9515	"Prokaryotes;Bacteria;Cyanobacteria;Prochlorococcus"
5830	pmf	Prochlorococcus marinus MIT 9303	"Prokaryotes;Bacteria;Cyanobacteria;Prochlorococcus"
5831	pmg	Prochlorococcus marinus MIT 9301	"Prokaryotes;Bacteria;Cyanobacteria;Prochlorococcus"
5832	pmh	Prochlorococcus marinus MIT 9215	"Prokaryotes;Bacteria;Cyanobacteria;Prochlorococcus"
5833	pmj	Prochlorococcus marinus MIT 9211	"Prokaryotes;Bacteria;Cyanobacteria;Prochlorococcus"
5834	pme	Prochlorococcus marinus NATL1A	"Prokaryotes;Bacteria;Cyanobacteria;Prochlorococcus"
5835	prc	Prochlorococcus sp. MIT 0604	"Prokaryotes;Bacteria;Cyanobacteria;Prochlorococcus"
5836	prm	Prochlorococcus sp. MIT 0801	"Prokaryotes;Bacteria;Cyanobacteria;Prochlorococcus"
5837	amr	Acaryochloris marina	"Prokaryotes;Bacteria;Cyanobacteria;Acaryochloris"
5838	theu	Thermoleptolyngbya sp. PKUAC-SCTA183	"Prokaryotes;Bacteria;Cyanobacteria;Thermoleptolyngbya"
5839	glp	Gloeocapsa sp. PCC 7428	"Prokaryotes;Bacteria;Cyanobacteria;Gloeocapsa"
5840	gen	Geminocystis sp. NIES-3709	"Prokaryotes;Bacteria;Cyanobacteria;Geminocystis"
5841	gee	Geminocystis sp. NIES-3708	"Prokaryotes;Bacteria;Cyanobacteria;Geminocystis"
5842	chon	Chondrocystis sp. NIES-4102	"Prokaryotes;Bacteria;Cyanobacteria;Chondrocystis"
5843	mar	Microcystis aeruginosa	"Prokaryotes;Bacteria;Cyanobacteria;Microcystis"
5844	mpk	Microcystis panniformis	"Prokaryotes;Bacteria;Cyanobacteria;Microcystis"
5845	miq	Microcystis sp. MC19	"Prokaryotes;Bacteria;Cyanobacteria;Microcystis"
5846	mvz	Microcystis viridis	"Prokaryotes;Bacteria;Cyanobacteria;Microcystis"
5847	can	Cyanobacterium aponinum	"Prokaryotes;Bacteria;Cyanobacteria;Cyanobacterium"
5848	csn	Cyanobacterium stanieri	"Prokaryotes;Bacteria;Cyanobacteria;Cyanobacterium"

5849	cyl	Cyanobacterium sp. HL-69	"Prokaryotes;Bacteria;Cyanobacteria;Cyanobacterium"
5850	hao	Halothece sp. PCC 7418	"Prokaryotes;Bacteria;Cyanobacteria;Halothece"
5851	enn	Euhalothece natronophila	"Prokaryotes;Bacteria;Cyanobacteria;Euhalothece"
5852	cyu	Candidatus Atelocyanobacterium thalassa	"Prokaryotes;Bacteria;Cyanobacteria;Atelocyanobacterium"
5853	cyt	Crocosphaera subtropica	"Prokaryotes;Bacteria;Cyanobacteria;Crocosphaera"
5854	cwa	Crocosphaera watsonii	"Prokaryotes;Bacteria;Cyanobacteria;Crocosphaera"
5855	cyp	Rippkaea orientalis PCC 8801	"Prokaryotes;Bacteria;Cyanobacteria;Rippkaea"
5856	cyh	Rippkaea orientalis PCC 8802	"Prokaryotes;Bacteria;Cyanobacteria;Rippkaea"
5857	cyc	Gloeothece citriformis	"Prokaryotes;Bacteria;Cyanobacteria;Gloeothece"
5858	cyj	Gloeothece verrucosa	"Prokaryotes;Bacteria;Cyanobacteria;Gloeothece"
5859	cyn	Cyanothece sp. PCC 7425	"Prokaryotes;Bacteria;Cyanobacteria;Cyanothece"
5860	ter	Trichodesmium erythraeum	"Prokaryotes;Bacteria;Cyanobacteria;Trichodesmium"
5861	mic	Microcoleus sp. PCC 7113	"Prokaryotes;Bacteria;Cyanobacteria;Microcoleus"
5862	arp	Arthrospira platensis	"Prokaryotes;Bacteria;Cyanobacteria;Arthrospira"
5863	pagh	Planktothrix agardhii	"Prokaryotes;Bacteria;Cyanobacteria;Planktothrix"
5864	oxy	Oxynema sp. AP17	"Prokaryotes;Bacteria;Cyanobacteria;Oxynema"
5865	lfs	Limnospira fusiformis	"Prokaryotes;Bacteria;Cyanobacteria;Limnospira"
5866	gei	Geitlerinema sp. PCC 7407	"Prokaryotes;Bacteria;Cyanobacteria;Geitlerinema"
5867	oac	Oscillatoria acuminata	"Prokaryotes;Bacteria;Cyanobacteria;Oscillatoria"
5868	oni	Oscillatoria nigro-viridis	"Prokaryotes;Bacteria;Cyanobacteria;Oscillatoria"
5869	mpro	Moorea producens	"Prokaryotes;Bacteria;Cyanobacteria;Moorea"
5870	cep	Crinalium epipsammum	"Prokaryotes;Bacteria;Cyanobacteria;Crinalium"
5871	gvi	Gloeobacter violaceus	"Prokaryotes;Bacteria;Cyanobacteria;Gloeobacter"
5872	glj	Gloeobacter kilaueensis	"Prokaryotes;Bacteria;Cyanobacteria;Gloeobacter"
5873	ana	Nostoc sp. PCC 7120	"Prokaryotes;Bacteria;Cyanobacteria;Nostoc"
5874	npu	Nostoc punctiforme	"Prokaryotes;Bacteria;Cyanobacteria;Nostoc"
5875	nos	Nostoc sp. PCC 7107	"Prokaryotes;Bacteria;Cyanobacteria;Nostoc"
5876	nop	Nostoc sp. PCC 7524	"Prokaryotes;Bacteria;Cyanobacteria;Nostoc"
5877	non	Nostoc sp. NIES-3756	"Prokaryotes;Bacteria;Cyanobacteria;Nostoc"
5878	nfl	Nostoc flagelliforme	"Prokaryotes;Bacteria;Cyanobacteria;Nostoc"
5879	noe	Nostoc sp. CENA543	"Prokaryotes;Bacteria;Cyanobacteria;Nostoc"
5880	nsh	Nostoc sphaeroides	"Prokaryotes;Bacteria;Cyanobacteria;Nostoc"
5881	ned	Nostoc edaphicum	"Prokaryotes;Bacteria;Cyanobacteria;Nostoc"
5882	ava	Trichormus variabilis	"Prokaryotes;Bacteria;Cyanobacteria;Trichormus"
5883	naz	'Nostoc azollae' 0708	"Prokaryotes;Bacteria;Cyanobacteria;Trichormus"
5884	anb	Anabaena sp. 90	"Prokaryotes;Bacteria;Cyanobacteria;Anabaena"
5885	acy	Anabaena cylindrica	"Prokaryotes;Bacteria;Cyanobacteria;Anabaena"
5886	awa	Anabaena sp. WA102	"Prokaryotes;Bacteria;Cyanobacteria;Anabaena"
5887	ann	Anabaena sp. YBS01	"Prokaryotes;Bacteria;Cyanobacteria;Anabaena"
5888	csg	Cylindrospermum stagnale	"Prokaryotes;Bacteria;Cyanobacteria;Cylindrospermum"
5889	calo	Calothrix sp. PCC 7507	"Prokaryotes;Bacteria;Cyanobacteria;Calothrix"

5890	calt	Calothrix sp. PCC 6303	"Prokaryotes;Bacteria;Cyanobacteria;Calothrix"
5891	calh	Calothrix sp. 336/3	"Prokaryotes;Bacteria;Cyanobacteria;Calothrix"
5892	riv	Rivularia sp. PCC 7116	"Prokaryotes;Bacteria;Cyanobacteria;Rivularia"
5893	fis	Fischerella sp. NIES-3754	"Prokaryotes;Bacteria;Cyanobacteria;Fischerella"
5894	nsp	Nodularia spumigena	"Prokaryotes;Bacteria;Cyanobacteria;Nodularia"
5895	dou	Dolichospermum sp. UHCC 0315A	"Prokaryotes;Bacteria;Cyanobacteria;Dolichospermum"
5896	dfs	Dolichospermum flos-aquae	"Prokaryotes;Bacteria;Cyanobacteria;Dolichospermum"
5897	ccur	Cylindrospermopsis curvispora	"Prokaryotes;Bacteria;Cyanobacteria;Cylindrospermopsis"
5898	toq	Tolypothrix sp. PCC 7910	"Prokaryotes;Bacteria;Cyanobacteria;Tolypothrix"
5899	ncn	Nostocales cyanobacterium HT-58-2	"Prokaryotes;Bacteria;Cyanobacteria;unclassified Nostocales"
5900	cthe	Chroococcidiopsis thermalis	"Prokaryotes;Bacteria;Cyanobacteria;Chroococcidiopsis"
5901	plp	Pleurocapsa sp. PCC 7327	"Prokaryotes;Bacteria;Cyanobacteria;Pleurocapsa"
5902	scs	Stanieria cyanosphaera	"Prokaryotes;Bacteria;Cyanobacteria;Stanieria"
5903	stan	Stanieria sp. NIES-3757	"Prokaryotes;Bacteria;Cyanobacteria;Stanieria"
5904	ceo	Cyanobacterium endosymbiont of Epithemia turgida	"Prokaryotes;Bacteria;Cyanobacteria;unclassified Cyanobacteria"
5905	cer	Cyanobacterium endosymbiont of Rhopalodia gibberula	"Prokaryotes;Bacteria;Cyanobacteria;unclassified Cyanobacteria"
5906	mbf	Candidatus Melainabacteria bacterium MEL.A1	"Prokaryotes;Bacteria;Cyanobacteria;unclassified Melainabacteria"
5907	det	Dehalococcoides mccartyi 195	"Prokaryotes;Bacteria;Chloroflexi;Dehalococcoides"
5908	deh	Dehalococcoides mccartyi CBDB1	"Prokaryotes;Bacteria;Chloroflexi;Dehalococcoides"
5909	deb	Dehalococcoides mccartyi BAV1	"Prokaryotes;Bacteria;Chloroflexi;Dehalococcoides"
5910	dev	Dehalococcoides mccartyi VS	"Prokaryotes;Bacteria;Chloroflexi;Dehalococcoides"
5911	deg	Dehalococcoides mccartyi GT	"Prokaryotes;Bacteria;Chloroflexi;Dehalococcoides"
5912	dmc	Dehalococcoides mccartyi BTF08	"Prokaryotes;Bacteria;Chloroflexi;Dehalococcoides"
5913	dmd	Dehalococcoides mccartyi DCMB5	"Prokaryotes;Bacteria;Chloroflexi;Dehalococcoides"
5914	dmg	Dehalococcoides mccartyi GY50	"Prokaryotes;Bacteria;Chloroflexi;Dehalococcoides"
5915	dmx	Dehalococcoides mccartyi CG1	"Prokaryotes;Bacteria;Chloroflexi;Dehalococcoides"
5916	dmy	Dehalococcoides mccartyi CG4	"Prokaryotes;Bacteria;Chloroflexi;Dehalococcoides"
5917	dmz	Dehalococcoides mccartyi CG5	"Prokaryotes;Bacteria;Chloroflexi;Dehalococcoides"
5918	duc	Dehalococcoides sp. UCH007	"Prokaryotes;Bacteria;Chloroflexi;Dehalococcoides"
5919	dly	Dehalogenimonas lykanthroporepellens	"Prokaryotes;Bacteria;Chloroflexi;Dehalogenimonas"
5920	dew	Dehalogenimonas sp. WBC-2	"Prokaryotes;Bacteria;Chloroflexi;Dehalogenimonas"
5921	dfo	Dehalogenimonas formicexedens	"Prokaryotes;Bacteria;Chloroflexi;Dehalogenimonas"
5922	rrs	Roseiflexus sp. RS-1	"Prokaryotes;Bacteria;Chloroflexi;Roseiflexus"
5923	rca	Roseiflexus castenholzii	"Prokaryotes;Bacteria;Chloroflexi;Roseiflexus"
5924	cau	Chloroflexus aurantiacus	"Prokaryotes;Bacteria;Chloroflexi;Chloroflexus"
5925	chl	Chloroflexus aurantiacus Y-400-fl	"Prokaryotes;Bacteria;Chloroflexi;Chloroflexus"
5926	cag	Chloroflexus aggregans	"Prokaryotes;Bacteria;Chloroflexi;Chloroflexus"
5927	hau	Herpetosiphon aurantiacus	"Prokaryotes;Bacteria;Chloroflexi;Herpetosiphon"
5928	tro	Thermomicrobium roseum	"Prokaryotes;Bacteria;Chloroflexi;Thermomicrobium"
5929	sti	Sphaerobacter thermophilus	"Prokaryotes;Bacteria;Chloroflexi;Sphaerobacterineae"
5930	atm	Anaerolinea thermophila	"Prokaryotes;Bacteria;Chloroflexi;Anaerolinea"

5931	abat	Brevefilum fermentans	"Prokaryotes;Bacteria;Chloroflexi;Brevefilum"
5932	psub	Pelolinea submarina	"Prokaryotes;Bacteria;Chloroflexi;Pelolinea"
5933	abao	Anaerolineaceae bacterium oral taxon 439	"Prokaryotes;Bacteria;Chloroflexi;unclassified Anaerolineaceae"
5934	cap	Caldilinea aerophila	"Prokaryotes;Bacteria;Chloroflexi;Caldilinea"
5935	pbf	Candidatus Promineofilum breve	"Prokaryotes;Bacteria;Chloroflexi;Promineofilum"
5936	kbs	Ktedonobacterales bacterium SCAWS-G2	"Prokaryotes;Bacteria;Chloroflexi;unclassified Ktedonobacterales"
5937	tbh	Tepidiforma bonchosmolovskayae	"Prokaryotes;Bacteria;Chloroflexi;Tepidiforma"
5938	ttr	Thermobaculum terrenum	"Prokaryotes;Bacteria;Chloroflexi;Thermobaculum"
5939	dra	Deinococcus radiodurans	"Prokaryotes;Bacteria;Deinococcus-Thermus;Deinococcus"
5940	dge	Deinococcus geothermalis	"Prokaryotes;Bacteria;Deinococcus-Thermus;Deinococcus"
5941	ddr	Deinococcus deserti	"Prokaryotes;Bacteria;Deinococcus-Thermus;Deinococcus"
5942	dmr	Deinococcus maricopensis	"Prokaryotes;Bacteria;Deinococcus-Thermus;Deinococcus"
5943	dpt	Deinococcus proteolyticus	"Prokaryotes;Bacteria;Deinococcus-Thermus;Deinococcus"
5944	dgo	Deinococcus gobiensis	"Prokaryotes;Bacteria;Deinococcus-Thermus;Deinococcus"
5945	dpd	Deinococcus peraridilitoris	"Prokaryotes;Bacteria;Deinococcus-Thermus;Deinococcus"
5946	dsw	Deinococcus swuensis	"Prokaryotes;Bacteria;Deinococcus-Thermus;Deinococcus"
5947	dch	Deinococcus soli	"Prokaryotes;Bacteria;Deinococcus-Thermus;Deinococcus"
5948	dab	Deinococcus actinosclerus	"Prokaryotes;Bacteria;Deinococcus-Thermus;Deinococcus"
5949	dpu	Deinococcus puniceus	"Prokaryotes;Bacteria;Deinococcus-Thermus;Deinococcus"
5950	dez	Deinococcus irradiatisoli	"Prokaryotes;Bacteria;Deinococcus-Thermus;Deinococcus"
5951	dwu	Deinococcus wulumuqiensis	"Prokaryotes;Bacteria;Deinococcus-Thermus;Deinococcus"
5952	dfc	Deinococcus ficus	"Prokaryotes;Bacteria;Deinococcus-Thermus;Deinococcus"
5953	dein	Deinococcus sp. AJ005	"Prokaryotes;Bacteria;Deinococcus-Thermus;Deinococcus"
5954	dga	Deinococcus grandis	"Prokaryotes;Bacteria;Deinococcus-Thermus;Deinococcus"
5955	tra	Truepera radiovictrix	"Prokaryotes;Bacteria;Deinococcus-Thermus;Truepera"
5956	tth	Thermus thermophilus HB27	"Prokaryotes;Bacteria;Deinococcus-Thermus;Thermus"
5957	ttj	Thermus thermophilus HB8	"Prokaryotes;Bacteria;Deinococcus-Thermus;Thermus"
5958	tts	Thermus thermophilus SG0.5JP17-16	"Prokaryotes;Bacteria;Deinococcus-Thermus;Thermus"
5959	ttl	Thermus thermophilus JL-18	"Prokaryotes;Bacteria;Deinococcus-Thermus;Thermus"
5960	tsc	Thermus scotoductus	"Prokaryotes;Bacteria;Deinococcus-Thermus;Thermus"
5961	thc	Thermus sp. CCB_US3_UF1	"Prokaryotes;Bacteria;Deinococcus-Thermus;Thermus"
5962	tos	Thermus oshimai	"Prokaryotes;Bacteria;Deinococcus-Thermus;Thermus"
5963	taq	Thermus aquaticus	"Prokaryotes;Bacteria;Deinococcus-Thermus;Thermus"
5964	tpar	Thermus parvatiensis	"Prokaryotes;Bacteria;Deinococcus-Thermus;Thermus"
5965	tbc	Thermus brockianus	"Prokaryotes;Bacteria;Deinococcus-Thermus;Thermus"
5966	mrb	Meiothermus ruber DSM 1279	"Prokaryotes;Bacteria;Deinococcus-Thermus;Meiothermus"
5967	mre	Meiothermus ruber DSM 1279	"Prokaryotes;Bacteria;Deinococcus-Thermus;Meiothermus"
5968	msv	Meiothermus silvanus	"Prokaryotes;Bacteria;Deinococcus-Thermus;Meiothermus"
5969	mtai	Meiothermus taiwanensis	"Prokaryotes;Bacteria;Deinococcus-Thermus;Meiothermus"
5970	opr	Oceanithermus profundus	"Prokaryotes;Bacteria;Deinococcus-Thermus;Oceanithermus"
5971	mhd	Marinithermus hydrothermalis	"Prokaryotes;Bacteria;Deinococcus-Thermus;Marinithermus"

5972	ccz	Chthonomonas calidirosea	"Prokaryotes;Bacteria;Other Terrabacteria group;Chthonomonas"
5973	fgi	Fimbriimonas ginsengisoli	"Prokaryotes;Bacteria;Other Terrabacteria group;Fimbriimonas"
5974	ctr	Chlamydia trachomatis D/UW-3/CX	"Prokaryotes;Bacteria;Chlamydiae;Chlamydia"
5975	ctd	Chlamydia trachomatis D-EC	"Prokaryotes;Bacteria;Chlamydiae;Chlamydia"
5976	ctf	Chlamydia trachomatis D-LC	"Prokaryotes;Bacteria;Chlamydiae;Chlamydia"
5977	ctrd	Chlamydia trachomatis D/SotonD1	"Prokaryotes;Bacteria;Chlamydiae;Chlamydia"
5978	ctro	Chlamydia trachomatis D/SotonD5	"Prokaryotes;Bacteria;Chlamydiae;Chlamydia"
5979	ctrt	Chlamydia trachomatis D/SotonD6	"Prokaryotes;Bacteria;Chlamydiae;Chlamydia"
5980	cta	Chlamydia trachomatis A/HAR-13	"Prokaryotes;Bacteria;Chlamydiae;Chlamydia"
5981	cty	Chlamydia trachomatis A2497	"Prokaryotes;Bacteria;Chlamydiae;Chlamydia"
5982	cra	Chlamydia trachomatis A2497	"Prokaryotes;Bacteria;Chlamydiae;Chlamydia"
5983	ctrq	Chlamydia trachomatis A/363	"Prokaryotes;Bacteria;Chlamydiae;Chlamydia"
5984	ctrx	Chlamydia trachomatis A/5291	"Prokaryotes;Bacteria;Chlamydiae;Chlamydia"
5985	ctrz	Chlamydia trachomatis A/7249	"Prokaryotes;Bacteria;Chlamydiae;Chlamydia"
5986	ctrp	Chlamydia trachomatis L1/1322/p2	"Prokaryotes;Bacteria;Chlamydiae;Chlamydia"
5987	ctlj	Chlamydia trachomatis L1/115	"Prokaryotes;Bacteria;Chlamydiae;Chlamydia"
5988	ctlx	Chlamydia trachomatis L1/224	"Prokaryotes;Bacteria;Chlamydiae;Chlamydia"
5989	ctll	Chlamydia trachomatis L1/440/LN	"Prokaryotes;Bacteria;Chlamydiae;Chlamydia"
5990	ctb	Chlamydia trachomatis 434/Bu	"Prokaryotes;Bacteria;Chlamydiae;Chlamydia"
5991	ctrr	Chlamydia trachomatis L2/25667R	"Prokaryotes;Bacteria;Chlamydiae;Chlamydia"
5992	ctlf	Chlamydia trachomatis L2/434/Bu(f)	"Prokaryotes;Bacteria;Chlamydiae;Chlamydia"
5993	ctli	Chlamydia trachomatis L2/434/Bu(i)	"Prokaryotes;Bacteria;Chlamydiae;Chlamydia"
5994	ctl	Chlamydia trachomatis L2b/UCH-1/proctitis	"Prokaryotes;Bacteria;Chlamydiae;Chlamydia"
5995	ctru	Chlamydia trachomatis L2b/UCH-2	"Prokaryotes;Bacteria;Chlamydiae;Chlamydia"
5996	ctrl	Chlamydia trachomatis L2b/LST	"Prokaryotes;Bacteria;Chlamydiae;Chlamydia"
5997	ctrv	Chlamydia trachomatis L2b/CV204	"Prokaryotes;Bacteria;Chlamydiae;Chlamydia"
5998	ctrm	Chlamydia trachomatis L2b/Ams1	"Prokaryotes;Bacteria;Chlamydiae;Chlamydia"
5999	ctla	Chlamydia trachomatis L2b/Ams2	"Prokaryotes;Bacteria;Chlamydiae;Chlamydia"
6000	ctlm	Chlamydia trachomatis L2b/Ams3	"Prokaryotes;Bacteria;Chlamydiae;Chlamydia"
6001	ctls	Chlamydia trachomatis L2b/Ams4	"Prokaryotes;Bacteria;Chlamydiae;Chlamydia"
6002	ctlz	Chlamydia trachomatis L2b/Ams5	"Prokaryotes;Bacteria;Chlamydiae;Chlamydia"
6003	ctlc	Chlamydia trachomatis L2b/Canada1	"Prokaryotes;Bacteria;Chlamydiae;Chlamydia"
6004	ctln	Chlamydia trachomatis L2b/Canada2	"Prokaryotes;Bacteria;Chlamydiae;Chlamydia"
6005	ctlb	Chlamydia trachomatis L2b/795	"Prokaryotes;Bacteria;Chlamydiae;Chlamydia"
6006	ctlq	Chlamydia trachomatis L2b/8200/07	"Prokaryotes;Bacteria;Chlamydiae;Chlamydia"
6007	cto	Chlamydia trachomatis L2c	"Prokaryotes;Bacteria;Chlamydiae;Chlamydia"
6008	ctrn	Chlamydia trachomatis L3/404/LN	"Prokaryotes;Bacteria;Chlamydiae;Chlamydia"
6009	ctj	Chlamydia trachomatis B/Jali20/OT	"Prokaryotes;Bacteria;Chlamydiae;Chlamydia"
6010	ctz	Chlamydia trachomatis B/TZ1A828/OT	"Prokaryotes;Bacteria;Chlamydiae;Chlamydia"
6011	ctg	Chlamydia trachomatis E/11023	"Prokaryotes;Bacteria;Chlamydiae;Chlamydia"
6012	ctk	Chlamydia trachomatis E/150	"Prokaryotes;Bacteria;Chlamydiae;Chlamydia"

6013	csw	Chlamydia trachomatis Sweden2	"Prokaryotes;Bacteria;Chlamydiae;Chlamydia"
6014	ces	Chlamydia trachomatis E/SW3	"Prokaryotes;Bacteria;Chlamydiae;Chlamydia"
6015	ctrb	Chlamydia trachomatis E/Bour	"Prokaryotes;Bacteria;Chlamydiae;Chlamydia"
6016	ctre	Chlamydia trachomatis E/SotonE4	"Prokaryotes;Bacteria;Chlamydiae;Chlamydia"
6017	ctrs	Chlamydia trachomatis E/SotonE8	"Prokaryotes;Bacteria;Chlamydiae;Chlamydia"
6018	ctec	Chlamydia trachomatis E/C599	"Prokaryotes;Bacteria;Chlamydiae;Chlamydia"
6019	cfs	Chlamydia trachomatis F/SW4	"Prokaryotes;Bacteria;Chlamydiae;Chlamydia"
6020	cfw	Chlamydia trachomatis F/SW5	"Prokaryotes;Bacteria;Chlamydiae;Chlamydia"
6021	ctfw	Chlamydia trachomatis F/SWFPminus	"Prokaryotes;Bacteria;Chlamydiae;Chlamydia"
6022	ctrf	Chlamydia trachomatis F/SotonF3	"Prokaryotes;Bacteria;Chlamydiae;Chlamydia"
6023	ctch	Chlamydia trachomatis F/11-96	"Prokaryotes;Bacteria;Chlamydiae;Chlamydia"
6024	ctn	Chlamydia trachomatis G/11074	"Prokaryotes;Bacteria;Chlamydiae;Chlamydia"
6025	ctq	Chlamydia trachomatis G/11222	"Prokaryotes;Bacteria;Chlamydiae;Chlamydia"
6026	ctv	Chlamydia trachomatis G/9301	"Prokaryotes;Bacteria;Chlamydiae;Chlamydia"
6027	ctw	Chlamydia trachomatis G/9768	"Prokaryotes;Bacteria;Chlamydiae;Chlamydia"
6028	ctrg	Chlamydia trachomatis G/SotonG1	"Prokaryotes;Bacteria;Chlamydiae;Chlamydia"
6029	ctri	Chlamydia trachomatis IU824	"Prokaryotes;Bacteria;Chlamydiae;Chlamydia"
6030	ctra	Chlamydia trachomatis IU888	"Prokaryotes;Bacteria;Chlamydiae;Chlamydia"
6031	ctrh	Chlamydia trachomatis Ia/SotonIa1	"Prokaryotes;Bacteria;Chlamydiae;Chlamydia"
6032	ctrj	Chlamydia trachomatis Ia/SotonIa3	"Prokaryotes;Bacteria;Chlamydiae;Chlamydia"
6033	ctrk	Chlamydia trachomatis K/SotonK1	"Prokaryotes;Bacteria;Chlamydiae;Chlamydia"
6034	ctjt	Chlamydia trachomatis J/6276tet1	"Prokaryotes;Bacteria;Chlamydiae;Chlamydia"
6035	ctcf	Chlamydia trachomatis RC-F/69	"Prokaryotes;Bacteria;Chlamydiae;Chlamydia"
6036	ctfs	Chlamydia trachomatis RC-F(s)/342	"Prokaryotes;Bacteria;Chlamydiae;Chlamydia"
6037	cthf	Chlamydia trachomatis RC-F(s)/852	"Prokaryotes;Bacteria;Chlamydiae;Chlamydia"
6038	ctcj	Chlamydia trachomatis RC-J/943	"Prokaryotes;Bacteria;Chlamydiae;Chlamydia"
6039	cthj	Chlamydia trachomatis RC-J/953	"Prokaryotes;Bacteria;Chlamydiae;Chlamydia"
6040	ctmj	Chlamydia trachomatis RC-J/966	"Prokaryotes;Bacteria;Chlamydiae;Chlamydia"
6041	cttj	Chlamydia trachomatis RC-J/971	"Prokaryotes;Bacteria;Chlamydiae;Chlamydia"
6042	ctjs	Chlamydia trachomatis RC-J(s)/122	"Prokaryotes;Bacteria;Chlamydiae;Chlamydia"
6043	ctrc	Chlamydia trachomatis RC-L2/55	"Prokaryotes;Bacteria;Chlamydiae;Chlamydia"
6044	ctrw	Chlamydia trachomatis RC-L2(s)/3	"Prokaryotes;Bacteria;Chlamydiae;Chlamydia"
6045	ctry	Chlamydia trachomatis RC-L2(s)/46	"Prokaryotes;Bacteria;Chlamydiae;Chlamydia"
6046	ctct	Chlamydia trachomatis C/TW-3	"Prokaryotes;Bacteria;Chlamydiae;Chlamydia"
6047	cmu	Chlamydia muridarum Nigg	"Prokaryotes;Bacteria;Chlamydiae;Chlamydia"
6048	cmur	Chlamydia muridarum Nigg CM972	"Prokaryotes;Bacteria;Chlamydiae;Chlamydia"
6049	cmn	Chlamydia muridarum Nigg 2 MCR	"Prokaryotes;Bacteria;Chlamydiae;Chlamydia"
6050	cmm	Chlamydia muridarum Nigg3 clone G0.1.1	"Prokaryotes;Bacteria;Chlamydiae;Chlamydia"
6051	cmg	Chlamydia muridarum Nigg3 clone G28.38.1	"Prokaryotes;Bacteria;Chlamydiae;Chlamydia"
6052	cmx	Chlamydia muridarum Nigg3	"Prokaryotes;Bacteria;Chlamydiae;Chlamydia"
6053	cmz	Chlamydia muridarum Nigg3 CMUT3-5	"Prokaryotes;Bacteria;Chlamydiae;Chlamydia"

6054	cpn	Chlamydia pneumoniae CWL029	"Prokaryotes;Bacteria;Chlamydiae;Chlamydia"
6055	cpa	Chlamydia pneumoniae AR39	"Prokaryotes;Bacteria;Chlamydiae;Chlamydia"
6056	cpj	Chlamydia pneumoniae J138	"Prokaryotes;Bacteria;Chlamydiae;Chlamydia"
6057	cpt	Chlamydia pneumoniae TW-183	"Prokaryotes;Bacteria;Chlamydiae;Chlamydia"
6058	clp	Chlamydia pneumoniae LPCoLN	"Prokaryotes;Bacteria;Chlamydiae;Chlamydia"
6059	cpm	Chlamydia pecorum E58	"Prokaryotes;Bacteria;Chlamydiae;Chlamydia"
6060	cpec	Chlamydia pecorum P787	"Prokaryotes;Bacteria;Chlamydiae;Chlamydia"
6061	cpeo	Chlamydia pecorum PV3056/3	"Prokaryotes;Bacteria;Chlamydiae;Chlamydia"
6062	cper	Chlamydia pecorum W73	"Prokaryotes;Bacteria;Chlamydiae;Chlamydia"
6063	chp	Chlamydia psittaci 6BC	"Prokaryotes;Bacteria;Chlamydiae;Chlamydia"
6064	chb	Chlamydia psittaci 6BC	"Prokaryotes;Bacteria;Chlamydiae;Chlamydia"
6065	chs	Chlamydia psittaci 01DC11	"Prokaryotes;Bacteria;Chlamydiae;Chlamydia"
6066	chi	Chlamydia psittaci 02DC15	"Prokaryotes;Bacteria;Chlamydiae;Chlamydia"
6067	cht	Chlamydia psittaci 08DC60	"Prokaryotes;Bacteria;Chlamydiae;Chlamydia"
6068	chc	Chlamydia psittaci C19/98	"Prokaryotes;Bacteria;Chlamydiae;Chlamydia"
6069	chr	Chlamydia psittaci RD1	"Prokaryotes;Bacteria;Chlamydiae;Chlamydia"
6070	cpsc	Chlamydia psittaci CP3	"Prokaryotes;Bacteria;Chlamydiae;Chlamydia"
6071	cpsn	Chlamydia psittaci NJ1	"Prokaryotes;Bacteria;Chlamydiae;Chlamydia"
6072	cpsb	Chlamydia psittaci 84/55	"Prokaryotes;Bacteria;Chlamydiae;Chlamydia"
6073	cpsg	Chlamydia psittaci GR9	"Prokaryotes;Bacteria;Chlamydiae;Chlamydia"
6074	cpsm	Chlamydia psittaci M56	"Prokaryotes;Bacteria;Chlamydiae;Chlamydia"
6075	cpsi	Chlamydia psittaci MN	"Prokaryotes;Bacteria;Chlamydiae;Chlamydia"
6076	cpsv	Chlamydia psittaci VS225	"Prokaryotes;Bacteria;Chlamydiae;Chlamydia"
6077	cpsw	Chlamydia psittaci WC	"Prokaryotes;Bacteria;Chlamydiae;Chlamydia"
6078	cpst	Chlamydia psittaci WS/RT/E30	"Prokaryotes;Bacteria;Chlamydiae;Chlamydia"
6079	cpsd	Chlamydia psittaci 01DC12	"Prokaryotes;Bacteria;Chlamydiae;Chlamydia"
6080	cpsa	Chlamydia psittaci Mat116	"Prokaryotes;Bacteria;Chlamydiae;Chlamydia"
6081	cav	Chlamydia avium	"Prokaryotes;Bacteria;Chlamydiae;Chlamydia"
6082	cca	Chlamydia caviae	"Prokaryotes;Bacteria;Chlamydiae;Chlamydia"
6083	cab	Chlamydia abortus S26/3	"Prokaryotes;Bacteria;Chlamydiae;Chlamydia"
6084	cabo	Chlamydia abortus AB7	"Prokaryotes;Bacteria;Chlamydiae;Chlamydia"
6085	cfe	Chlamydia felis Fe/C-56	"Prokaryotes;Bacteria;Chlamydiae;Chlamydia"
6086	cgz	Chlamydia gallinacea	"Prokaryotes;Bacteria;Chlamydiae;Chlamydia"
6087	chla	Chlamydia sp. S15-834C	"Prokaryotes;Bacteria;Chlamydiae;Chlamydia"
6088	pcu	Candidatus Protochlamydia amoebophila	"Prokaryotes;Bacteria;Chlamydiae;Protochlamydia"
6089	pnl	Candidatus Protochlamydia naegleriophila	"Prokaryotes;Bacteria;Chlamydiae;Protochlamydia"
6090	puv	Parachlamydia acanthamoebae	"Prokaryotes;Bacteria;Chlamydiae;Parachlamydia"
6091	ney	Neochlamydia sp. S13	"Prokaryotes;Bacteria;Chlamydiae;Neochlamydia"
6092	wch	Waddlia chondrophila	"Prokaryotes;Bacteria;Chlamydiae;Waddlia"
6093	sng	Simkania negevensis	"Prokaryotes;Bacteria;Chlamydiae;Simkania"
6094	ote	Opitutus terrae	"Prokaryotes;Bacteria;Verrucomicrobia;Opitutus"

6095	obg	Lacunisphaera limnophila	"Prokaryotes;Bacteria;Verrucomicrobia;Lacunisphaera"
6096	vbh	Nibricoccus aquaticus HZ-65	"Prokaryotes;Bacteria;Verrucomicrobia;Nibricoccus"
6097	obt	Opitutaceae bacterium TAV5	"Prokaryotes;Bacteria;Verrucomicrobia;unclassified Opitutaceae"
6098	caa	Coraliomargarita akajimensis	"Prokaryotes;Bacteria;Verrucomicrobia;Coraliomargarita"
6099	amu	Akkermansia muciniphila	"Prokaryotes;Bacteria;Verrucomicrobia;Akkermansia"
6100	agl	Akkermansia glycaniphila	"Prokaryotes;Bacteria;Verrucomicrobia;Akkermansia"
6101	roo	Roseimicrobium sp. ORNL1	"Prokaryotes;Bacteria;Verrucomicrobia;Roseimicrobium"
6102	luo	Luteolibacter luteus	"Prokaryotes;Bacteria;Verrucomicrobia;Luteolibacter"
6103	xii	Candidatus Xiphinematobacter sp. Idaho Grape	"Prokaryotes;Bacteria;Verrucomicrobia;Xiphinematobacter"
6104	min	Methylacidiphilum infernorum	"Prokaryotes;Bacteria;Verrucomicrobia;Methylacidiphilum"
6105	mkc	Methylacidiphilum kamchatkense	"Prokaryotes;Bacteria;Verrucomicrobia;Methylacidiphilum"
6106	meap	Methylacidimicrobium sp. AP8	"Prokaryotes;Bacteria;Verrucomicrobia;Methylacidimicrobium"
6107	psup	Candidatus Pinguicoccus supinus	"Prokaryotes;Bacteria;Verrucomicrobia;Pinguicoccus"
6108	vba	Verrucomicrobia bacterium IMCC26134	"Prokaryotes;Bacteria;Verrucomicrobia;unclassified Verrucomicrobia"
6109	vbs	Verrucomicrobia bacterium S94	"Prokaryotes;Bacteria;Verrucomicrobia;unclassified Verrucomicrobia"
6110	rba	Rhodopirellula baltica	"Prokaryotes;Bacteria;Planctomycetes;Rhodopirellula"
6111	psl	Pirellula staleyi	"Prokaryotes;Bacteria;Planctomycetes;Pirellula"
6112	pir	Pirellula sp. SH-Sr6A	"Prokaryotes;Bacteria;Planctomycetes;Pirellula"
6113	rul	Roseimaritima ulvae	"Prokaryotes;Bacteria;Planctomycetes;Roseimaritima"
6114	mff	Mariniblastus fucicola	"Prokaryotes;Bacteria;Planctomycetes;Mariniblastus"
6115	rol	Rosistilla oblonga	"Prokaryotes;Bacteria;Planctomycetes;Rosistilla"
6116	ahel	Aureliella helgolandensis	"Prokaryotes;Bacteria;Planctomycetes;Aureliella"
6117	lcre	Lignipirellula cremea	"Prokaryotes;Bacteria;Planctomycetes;Lignipirellula"
6118	aagg	Anatilimnocola aggregata	"Prokaryotes;Bacteria;Planctomycetes;Anatilimnocola"
6119	bvo	Bremerella volcania	"Prokaryotes;Bacteria;Planctomycetes;Bremerella"
6120	ttf	Thermogutta terrifontis	"Prokaryotes;Bacteria;Planctomycetes;Thermogutta"
6121	lpav	Lacipirellula parvula	"Prokaryotes;Bacteria;Planctomycetes;Lacipirellula"
6122	amuc	Aeoliella mucimassa	"Prokaryotes;Bacteria;Planctomycetes;Aeoliella"
6123	pnd	Pirellulimonas nuda	"Prokaryotes;Bacteria;Planctomycetes;Pirellulimonas"
6124	plm	Planctopirus limnophila	"Prokaryotes;Bacteria;Planctomycetes;Planctopirus"
6125	peh	Planctopirus ephydatiae	"Prokaryotes;Bacteria;Planctomycetes;Planctopirus"
6126	pbs	Rubinisphaera brasiliensis	"Prokaryotes;Bacteria;Planctomycetes;Rubinisphaera"
6127	pls	Planctomyces sp. SH-PL14	"Prokaryotes;Bacteria;Planctomycetes;Planctomyces"
6128	plh	Planctomyces sp. SH-PL62	"Prokaryotes;Bacteria;Planctomycetes;Planctomyces"
6129	fmr	Fuerstia marisgermanicae	"Prokaryotes;Bacteria;Planctomycetes;Fuerstia"
6130	gmr	Gimesia maris	"Prokaryotes;Bacteria;Planctomycetes;Gimesia"
6131	gim	Gimesia benthica	"Prokaryotes;Bacteria;Planctomycetes;Gimesia"
6132	mri	Maioricimonas rarisocia	"Prokaryotes;Bacteria;Planctomycetes;Maioricimonas"
6133	sdyn	Symmachiella dynata	"Prokaryotes;Bacteria;Planctomycetes;Symmachiella"
6134	plon	Polystyrenella longa	"Prokaryotes;Bacteria;Planctomycetes;Polystyrenella"
6135	ges	Gemmata sp. SH-PL17	"Prokaryotes;Bacteria;Planctomycetes;Gemmata"

6136	gog	Gemmata obscuriglobus	"Prokaryotes;Bacteria;Planctomycetes;Gemmata"
6137	gms	Gemmata massiliana	"Prokaryotes;Bacteria;Planctomycetes;Gemmata"
6138	tim	Tuwongella immobilis	"Prokaryotes;Bacteria;Planctomycetes;Tuwongella"
6139	lrs	Limnoglobus roseus	"Prokaryotes;Bacteria;Planctomycetes;Limnoglobus"
6140	ftj	Frigoriglobus tundricola	"Prokaryotes;Bacteria;Planctomycetes;Frigoriglobus"
6141	uli	Urbifossiella limnaea	"Prokaryotes;Bacteria;Planctomycetes;Urbifossiella"
6142	ipa	Isosphaera pallida	"Prokaryotes;Bacteria;Planctomycetes;Isosphaera"
6143	saci	Singulisphaera acidiphila	"Prokaryotes;Bacteria;Planctomycetes;Singulisphaera"
6144	pbor	Paludisphaera borealis	"Prokaryotes;Bacteria;Planctomycetes;Paludisphaera"
6145	agv	Aquisphaera giovannonii	"Prokaryotes;Bacteria;Planctomycetes;Aquisphaera"
6146	kst	Candidatus Kuenenia stuttgartiensis	"Prokaryotes;Bacteria;Planctomycetes;Kuenenia"
6147	broc	Candidatus Brocadia sp. Ega_18-Q3-R5-49_MAXAC.112v2	"Prokaryotes;Bacteria;Planctomycetes;Brocadia"
6148	phm	Phycisphaera mikurensis	"Prokaryotes;Bacteria;Planctomycetes;Phycisphaera"
6149	pcor	Poriferisphaera corsica	"Prokaryotes;Bacteria;Planctomycetes;Poriferisphaera"
6150	pbu	Sedimentisphaera cyanobacteriorum L21-RPul-D3	"Prokaryotes;Bacteria;Planctomycetes;Sedimentisphaera"
6151	pbp	Sedimentisphaera salicampi	"Prokaryotes;Bacteria;Planctomycetes;Sedimentisphaera"
6152	pbas	Limihaloglobus sulfuriphilus	"Prokaryotes;Bacteria;Planctomycetes;Limihaloglobus"
6153	alus	Anaerohalosphaera lusitana	"Prokaryotes;Bacteria;Planctomycetes;Anaerohalosphaera"
6154	vbl	Kiritimatiella glycovorans	"Prokaryotes;Bacteria;Kiritimatiellaeota;Kiritimatiella"
6155	vbc	Victivallales bacterium CCUG 44730	"Prokaryotes;Bacteria;Lentisphaerae;unclassified Victivallales"
6156	vai	Candidatus Vampirococcus archaeovorus	"Prokaryotes;Bacteria;Omnitrophica;Vampirococcus"
6157	bbu	Borreliella burgdorferi B31	"Prokaryotes;Bacteria;Spirochaetes;Borreliella"
6158	bbz	Borreliella burgdorferi ZS7	"Prokaryotes;Bacteria;Spirochaetes;Borreliella"
6159	bbn	Borreliella burgdorferi N40	"Prokaryotes;Bacteria;Spirochaetes;Borreliella"
6160	bbj	Borreliella burgdorferi JD1	"Prokaryotes;Bacteria;Spirochaetes;Borreliella"
6161	bbur	Borreliella burgdorferi CA382	"Prokaryotes;Bacteria;Spirochaetes;Borreliella"
6162	bga	Borreliella bavariensis	"Prokaryotes;Bacteria;Spirochaetes;Borreliella"
6163	bgb	Borreliella garinii BgVir	"Prokaryotes;Bacteria;Spirochaetes;Borreliella"
6164	bgn	Borreliella garinii NMJW1	"Prokaryotes;Bacteria;Spirochaetes;Borreliella"
6165	bgs	Borreliella garinii SZ	"Prokaryotes;Bacteria;Spirochaetes;Borreliella"
6166	bgc	Borreliella garinii CIP 103362	"Prokaryotes;Bacteria;Spirochaetes;Borreliella"
6167	baf	Borrelia afzelii (FLI)	"Prokaryotes;Bacteria;Spirochaetes;Borreliella"
6168	bafz	Borrelia afzelii (Maryland)	"Prokaryotes;Bacteria;Spirochaetes;Borreliella"
6169	bafh	Borreliella afzelii HLJ01	"Prokaryotes;Bacteria;Spirochaetes;Borreliella"
6170	baft	Borreliella afzelii Tom3107	"Prokaryotes;Bacteria;Spirochaetes;Borreliella"
6171	bafe	Borreliella afzelii K78	"Prokaryotes;Bacteria;Spirochaetes;Borreliella"
6172	bbs	Borreliella bissettii	"Prokaryotes;Bacteria;Spirochaetes;Borreliella"
6173	bvt	Borreliella valaisiana	"Prokaryotes;Bacteria;Spirochaetes;Borreliella"
6174	bchi	Borreliella chilensis	"Prokaryotes;Bacteria;Spirochaetes;Borreliella"
6175	bmay	Borreliella mayonii	"Prokaryotes;Bacteria;Spirochaetes;Borreliella"
6176	btu	Borrelia turicatae	"Prokaryotes;Bacteria;Spirochaetes;Borrelia"

6177	bhr	Borrelia hermsii DAH	"Prokaryotes;Bacteria;Spirochaetes;Borrelia"
6178	bhi	Borrelia hermsii CC1	"Prokaryotes;Bacteria;Spirochaetes;Borrelia"
6179	bdu	Borrelia duttonii	"Prokaryotes;Bacteria;Spirochaetes;Borrelia"
6180	bre	Borrelia recurrentis	"Prokaryotes;Bacteria;Spirochaetes;Borrelia"
6181	bcw	Borrelia crocidurae	"Prokaryotes;Bacteria;Spirochaetes;Borrelia"
6182	bmo	Borrelia miyamotoi LB-2001	"Prokaryotes;Bacteria;Spirochaetes;Borrelia"
6183	bmiy	Borrelia miyamotoi CT14D4	"Prokaryotes;Bacteria;Spirochaetes;Borrelia"
6184	bpak	Borrelia parkeri	"Prokaryotes;Bacteria;Spirochaetes;Borrelia"
6185	bane	Borrelia anserina	"Prokaryotes;Bacteria;Spirochaetes;Borrelia"
6186	btur	Borrelia turcica	"Prokaryotes;Bacteria;Spirochaetes;Borrelia"
6187	bmat	Borrelia maritima	"Prokaryotes;Bacteria;Spirochaetes;Borrelia"
6188	tpa	Treponema pallidum subsp. pallidum Nichols	"Prokaryotes;Bacteria;Spirochaetes;Treponema"
6189	tpw	Treponema pallidum subsp. pallidum Nichols	"Prokaryotes;Bacteria;Spirochaetes;Treponema"
6190	tpp	Treponema pallidum subsp. pallidum SS14	"Prokaryotes;Bacteria;Spirochaetes;Treponema"
6191	tpu	Treponema pallidum subsp. pallidum DAL-1	"Prokaryotes;Bacteria;Spirochaetes;Treponema"
6192	tph	Treponema pallidum subsp. pallidum Chicago	"Prokaryotes;Bacteria;Spirochaetes;Treponema"
6193	tpo	Treponema pallidum subsp. pallidum Mexico A	"Prokaryotes;Bacteria;Spirochaetes;Treponema"
6194	tpas	Treponema pallidum subsp. pallidum Sea 81-4	"Prokaryotes;Bacteria;Spirochaetes;Treponema"
6195	tpc	Treponema pallidum subsp. pertenue CDC2	"Prokaryotes;Bacteria;Spirochaetes;Treponema"
6196	tpg	Treponema pallidum subsp. pertenue Gauthier	"Prokaryotes;Bacteria;Spirochaetes;Treponema"
6197	tpm	Treponema pallidum subsp. pertenue SamoaD	"Prokaryotes;Bacteria;Spirochaetes;Treponema"
6198	tpb	Treponema pallidum Fribourg-Blanc	"Prokaryotes;Bacteria;Spirochaetes;Treponema"
6199	tde	Treponema denticola	"Prokaryotes;Bacteria;Spirochaetes;Treponema"
6200	tsu	Treponema succinifaciens	"Prokaryotes;Bacteria;Spirochaetes;Treponema"
6201	tbe	Treponema brennaborense	"Prokaryotes;Bacteria;Spirochaetes;Treponema"
6202	taz	Treponema azotonutricium	"Prokaryotes;Bacteria;Spirochaetes;Treponema"
6203	tpi	Treponema primitia	"Prokaryotes;Bacteria;Spirochaetes;Treponema"
6204	tpl	Treponema paraluiscuniculi	"Prokaryotes;Bacteria;Spirochaetes;Treponema"
6205	tped	Treponema pedis	"Prokaryotes;Bacteria;Spirochaetes;Treponema"
6206	scd	Treponema caldarium	"Prokaryotes;Bacteria;Spirochaetes;Treponema"
6207	tpk	Treponema putidum	"Prokaryotes;Bacteria;Spirochaetes;Treponema"
6208	trm	Treponema sp. OMZ 838	"Prokaryotes;Bacteria;Spirochaetes;Treponema"
6209	tphg	Treponema phagedenis	"Prokaryotes;Bacteria;Spirochaetes;Treponema"
6210	trz	Treponema sp. OMZ 804	"Prokaryotes;Bacteria;Spirochaetes;Treponema"
6211	trc	Treponema rectale	"Prokaryotes;Bacteria;Spirochaetes;Treponema"
6212	ssm	Sediminispirochaeta smaragdinae	"Prokaryotes;Bacteria;Spirochaetes;Sediminispirochaeta"
6213	sta	Spirochaeta thermophila DSM 6192	"Prokaryotes;Bacteria;Spirochaetes;Spirochaeta"
6214	stq	Spirochaeta thermophila DSM 6578	"Prokaryotes;Bacteria;Spirochaetes;Spirochaeta"
6215	sfc	Spirochaeta africana	"Prokaryotes;Bacteria;Spirochaetes;Spirochaeta"
6216	sper	Thiospirochaeta perfilievii	"Prokaryotes;Bacteria;Spirochaetes;Spirochaeta"
6217	sbu	Sphaerochaeta globosa	"Prokaryotes;Bacteria;Spirochaetes;Sphaerochaeta"

6218	scc	Sphaerochaeta coccoides	"Prokaryotes;Bacteria;Spirochaetes;Sphaerochaeta"
6219	sgp	Sphaerochaeta pleomorpha	"Prokaryotes;Bacteria;Spirochaetes;Sphaerochaeta"
6220	slr	Salinispira pacifica	"Prokaryotes;Bacteria;Spirochaetes;Salinispira"
6221	ock	Oceanispirochaeta crateris	"Prokaryotes;Bacteria;Spirochaetes;Oceanispirochaeta"
6222	lil	Leptospira interrogans serovar Lai 56601	"Prokaryotes;Bacteria;Spirochaetes;Leptospira"
6223	lie	Leptospira interrogans serovar Lai IPAV	"Prokaryotes;Bacteria;Spirochaetes;Leptospira"
6224	lic	Leptospira interrogans serovar Copenhageni	"Prokaryotes;Bacteria;Spirochaetes;Leptospira"
6225	lis	Leptospira interrogans serovar Linhai	"Prokaryotes;Bacteria;Spirochaetes;Leptospira"
6226	lbj	Leptospira borgpetersenii JB197	"Prokaryotes;Bacteria;Spirochaetes;Leptospira"
6227	lbl	Leptospira borgpetersenii L550	"Prokaryotes;Bacteria;Spirochaetes;Leptospira"
6228	lbi	Leptospira biflexa serovar Patoc Patoc 1 (Paris)	"Prokaryotes;Bacteria;Spirochaetes;Leptospira"
6229	lbf	Leptospira biflexa serovar Patoc Patoc 1 (Ames)	"Prokaryotes;Bacteria;Spirochaetes;Leptospira"
6230	lst	Leptospira santarosai	"Prokaryotes;Bacteria;Spirochaetes;Leptospira"
6231	laj	Leptospira tipperaryensis	"Prokaryotes;Bacteria;Spirochaetes;Leptospira"
6232	lmay	Leptospira mayottensis	"Prokaryotes;Bacteria;Spirochaetes;Leptospira"
6233	lkm	Leptospira kmetyi	"Prokaryotes;Bacteria;Spirochaetes;Leptospira"
6234	lwl	Leptospira weilii	"Prokaryotes;Bacteria;Spirochaetes;Leptospira"
6235	tpx	Turneriella parva	"Prokaryotes;Bacteria;Spirochaetes;Turneriella"
6236	bhy	Brachyspira hyodysenteriae WA1	"Prokaryotes;Bacteria;Spirochaetes;Brachyspira"
6237	bhd	Brachyspira hyodysenteriae ATCC 27164	"Prokaryotes;Bacteria;Spirochaetes;Brachyspira"
6238	brm	Brachyspira murdochii	"Prokaryotes;Bacteria;Spirochaetes;Brachyspira"
6239	bpo	Brachyspira pilosicoli 95/1000	"Prokaryotes;Bacteria;Spirochaetes;Brachyspira"
6240	bpj	Brachyspira pilosicoli B2904	"Prokaryotes;Bacteria;Spirochaetes;Brachyspira"
6241	bpip	Brachyspira pilosicoli P43/6/78	"Prokaryotes;Bacteria;Spirochaetes;Brachyspira"
6242	bpw	Brachyspira pilosicoli WesB	"Prokaryotes;Bacteria;Spirochaetes;Brachyspira"
6243	bip	Brachyspira intermedia	"Prokaryotes;Bacteria;Spirochaetes;Brachyspira"
6244	bhp	Brachyspira hampsonii	"Prokaryotes;Bacteria;Spirochaetes;Brachyspira"
6245	aba	Candidatus Koribacter versatilis	"Prokaryotes;Bacteria;Acidobacteria;Koribacter"
6246	aca	Acidobacterium capsulatum	"Prokaryotes;Bacteria;Acidobacteria;Acidobacterium"
6247	acm	Granulicella tundricola	"Prokaryotes;Bacteria;Acidobacteria;Granulicella"
6248	gma	Granulicella mallensis	"Prokaryotes;Bacteria;Acidobacteria;Granulicella"
6249	grw	Granulicella sp. WH15	"Prokaryotes;Bacteria;Acidobacteria;Granulicella"
6250	tsa	Terriglobus saanensis	"Prokaryotes;Bacteria;Acidobacteria;Terriglobus"
6251	trs	Terriglobus roseus	"Prokaryotes;Bacteria;Acidobacteria;Terriglobus"
6252	talb	Terriglobus albidus	"Prokaryotes;Bacteria;Acidobacteria;Terriglobus"
6253	abas	Acidisarcina polymorpha	"Prokaryotes;Bacteria;Acidobacteria;Acidisarcina"
6254	eda	Edaphobacter sp. 12200R-103	"Prokaryotes;Bacteria;Acidobacteria;Edaphobacter"
6255	sus	Candidatus Solibacter usitatus	"Prokaryotes;Bacteria;Acidobacteria;Solibacter"
6256	pfer	Paludibaculum fermentans	"Prokaryotes;Bacteria;Acidobacteria;Paludibaculum"
6257	ctm	Chloracidobacterium thermophilum	"Prokaryotes;Bacteria;Acidobacteria;Chloracidobacterium"
6258	abac	Luteitalea pratensis	"Prokaryotes;Bacteria;Acidobacteria;Luteitalea"

6259	thyd	Thermotomaculum hydrothermale	"Prokaryotes;Bacteria;Acidobacteria;Thermotomaculum"
6260	emi	Elusimicrobium minutum	"Prokaryotes;Bacteria;Elusimicrobia;Elusimicrobium"
6261	epo	Endomicrobium proavitum	"Prokaryotes;Bacteria;Elusimicrobia;Endomicrobium"
6262	eti	Candidatus Endomicrobium trichonymphae Rs-D17	"Prokaryotes;Bacteria;Elusimicrobia;Endomicrobium"
6263	rsd	Candidatus Endomicrobium trichonymphae Rs-D17	"Prokaryotes;Bacteria;Elusimicrobia;Endomicrobium"
6264	fnu	Fusobacterium nucleatum subsp. nucleatum ATCC 25586	"Prokaryotes;Bacteria;Fusobacteria;Fusobacterium"
6265	fnc	Fusobacterium nucleatum subsp. vincentii 3_1_36A2	"Prokaryotes;Bacteria;Fusobacteria;Fusobacterium"
6266	fnt	Fusobacterium nucleatum subsp. vincentii 3_1_27	"Prokaryotes;Bacteria;Fusobacteria;Fusobacterium"
6267	fus	Fusobacterium nucleatum subsp. animalis 4_8	"Prokaryotes;Bacteria;Fusobacteria;Fusobacterium"
6268	fne	Fusobacterium nucleatum subsp. animalis 7_1	"Prokaryotes;Bacteria;Fusobacteria;Fusobacterium"
6269	fhw	Fusobacterium hwasookii	"Prokaryotes;Bacteria;Fusobacteria;Fusobacterium"
6270	fpd	Fusobacterium pseudoperiodonticum	"Prokaryotes;Bacteria;Fusobacteria;Fusobacterium"
6271	fva	Fusobacterium varium	"Prokaryotes;Bacteria;Fusobacteria;Fusobacterium"
6272	ful	Fusobacterium ulcerans	"Prokaryotes;Bacteria;Fusobacteria;Fusobacterium"
6273	fmo	Fusobacterium mortiferum	"Prokaryotes;Bacteria;Fusobacteria;Fusobacterium"
6274	fgo	Fusobacterium gonidiaformans	"Prokaryotes;Bacteria;Fusobacteria;Fusobacterium"
6275	fnf	Fusobacterium necrophorum subsp. funduliforme	"Prokaryotes;Bacteria;Fusobacteria;Fusobacterium"
6276	fpei	Fusobacterium periodonticum	"Prokaryotes;Bacteria;Fusobacteria;Fusobacterium"
6277	ipo	Ilyobacter polytropus	"Prokaryotes;Bacteria;Fusobacteria;Ilyobacter"
6278	lba	Leptotrichia buccalis	"Prokaryotes;Bacteria;Fusobacteria;Leptotrichia"
6279	leo	Leptotrichia sp. oral taxon 212	"Prokaryotes;Bacteria;Fusobacteria;Leptotrichia"
6280	lot	Leptotrichia sp. oral taxon 847	"Prokaryotes;Bacteria;Fusobacteria;Leptotrichia"
6281	leq	Leptotrichia sp. oral taxon 498	"Prokaryotes;Bacteria;Fusobacteria;Leptotrichia"
6282	lhf	Leptotrichia hofstadii	"Prokaryotes;Bacteria;Fusobacteria;Leptotrichia"
6283	lsz	Leptotrichia shahii	"Prokaryotes;Bacteria;Fusobacteria;Leptotrichia"
6284	lhg	Leptotrichia hongkongensis	"Prokaryotes;Bacteria;Fusobacteria;Leptotrichia"
6285	lte	Leptotrichia trevisanii	"Prokaryotes;Bacteria;Fusobacteria;Leptotrichia"
6286	lwd	Leptotrichia wadei	"Prokaryotes;Bacteria;Fusobacteria;Leptotrichia"
6287	lgo	Pseudoleptotrichia goodfellowii	"Prokaryotes;Bacteria;Fusobacteria;Pseudoleptotrichia"
6288	str	Sebaldella termitidis	"Prokaryotes;Bacteria;Fusobacteria;Sebaldella"
6289	smf	Streptobacillus moniliformis	"Prokaryotes;Bacteria;Fusobacteria;Streptobacillus"
6290	sns	Sneathia vaginalis	"Prokaryotes;Bacteria;Fusobacteria;Sneathia"
6291	tai	Thermanaerovibrio acidaminovorans	"Prokaryotes;Bacteria;Synergistetes;Thermanaerovibrio"
6292	aco	Aminobacterium colombiense	"Prokaryotes;Bacteria;Synergistetes;Aminobacterium"
6293	tli	Thermovirga lienii	"Prokaryotes;Bacteria;Synergistetes;Thermovirga"
6294	amo	Acetomicrobium mobile	"Prokaryotes;Bacteria;Synergistetes;Anaerobaculum"
6295	sbr	Fretibacterium fastidiosum	"Prokaryotes;Bacteria;Synergistetes;Fretibacterium"
6296	cpor	Cloacibacillus porcorum	"Prokaryotes;Bacteria;Synergistetes;Cloacibacillus"
6297	fsu	Fibrobacter succinogenes	"Prokaryotes;Bacteria;Fibrobacteres;Fibrobacter"
6298	fsc	Fibrobacter succinogenes	"Prokaryotes;Bacteria;Fibrobacteres;Fibrobacter"
6299	gau	Gemmatimonas aurantiaca	"Prokaryotes;Bacteria;Gemmatimonadetes;Gemmatimonas"

6300	gph	Gemmatimonas phototrophica	"Prokaryotes;Bacteria;Gemmatimonadetes;Gemmatimonas"
6301	gba	Gemmatirosa kalamazoonesis	"Prokaryotes;Bacteria;Gemmatimonadetes;Gemmatirosa"
6302	bth	Bacteroides thetaiotaomicron VPI-5482	"Prokaryotes;Bacteria;Bacteroidetes;Bacteroides"
6303	btho	Bacteroides thetaiotaomicron 7330	"Prokaryotes;Bacteria;Bacteroidetes;Bacteroides"
6304	bfr	Bacteroides fragilis YCH46	"Prokaryotes;Bacteria;Bacteroidetes;Bacteroides"
6305	bfs	Bacteroides fragilis NCTC 9343	"Prokaryotes;Bacteria;Bacteroidetes;Bacteroides"
6306	bfg	Bacteroides fragilis 638R	"Prokaryotes;Bacteria;Bacteroidetes;Bacteroides"
6307	bfb	Bacteroides fragilis BOB25	"Prokaryotes;Bacteria;Bacteroidetes;Bacteroides"
6308	bhl	Bacteroides helcogenes	"Prokaryotes;Bacteria;Bacteroidetes;Bacteroides"
6309	bxy	Bacteroides xylanisolvens	"Prokaryotes;Bacteria;Bacteroidetes;Bacteroides"
6310	boa	Bacteroides ovatus	"Prokaryotes;Bacteria;Bacteroidetes;Bacteroides"
6311	bcel	Bacteroides cellulosilyticus	"Prokaryotes;Bacteria;Bacteroidetes;Bacteroides"
6312	bcac	Bacteroides caccae	"Prokaryotes;Bacteria;Bacteroidetes;Bacteroides"
6313	bcae	Bacteroides caecimuris	"Prokaryotes;Bacteria;Bacteroidetes;Bacteroides"
6314	bzg	Bacteroides zoogleoformans	"Prokaryotes;Bacteria;Bacteroidetes;Bacteroides"
6315	bhf	Bacteroides heparinolyticus	"Prokaryotes;Bacteria;Bacteroidetes;Bacteroides"
6316	bis	Bacteroides intestinalis	"Prokaryotes;Bacteria;Bacteroidetes;Bacteroides"
6317	bun	Bacteroides uniformis	"Prokaryotes;Bacteria;Bacteroidetes;Bacteroides"
6318	bvu	Bacteroides vulgatus	"Prokaryotes;Bacteria;Bacteroidetes;Phocaeicola"
6319	bsa	Phocaeicola salanitronis	"Prokaryotes;Bacteria;Bacteroidetes;Phocaeicola"
6320	bdo	Phocaeicola dorei HS1_L_1_B_010	"Prokaryotes;Bacteria;Bacteroidetes;Phocaeicola"
6321	bdh	Phocaeicola dorei HS1_L_3_B_079	"Prokaryotes;Bacteria;Bacteroidetes;Phocaeicola"
6322	pgi	Porphyromonas gingivalis W83	"Prokaryotes;Bacteria;Bacteroidetes;Porphyromonas"
6323	pgn	Porphyromonas gingivalis ATCC 33277	"Prokaryotes;Bacteria;Bacteroidetes;Porphyromonas"
6324	pgt	Porphyromonas gingivalis TDC60	"Prokaryotes;Bacteria;Bacteroidetes;Porphyromonas"
6325	pah	Porphyromonas asaccharolytica	"Prokaryotes;Bacteria;Bacteroidetes;Porphyromonas"
6326	pcre	Porphyromonas crevioricanis	"Prokaryotes;Bacteria;Bacteroidetes;Porphyromonas"
6327	pcag	Porphyromonas cangingivalis	"Prokaryotes;Bacteria;Bacteroidetes;Porphyromonas"
6328	pbt	Fermentimonas caenicola	"Prokaryotes;Bacteria;Bacteroidetes;Fermentimonas"
6329	pmuc	Petrimonas mucosa	"Prokaryotes;Bacteria;Bacteroidetes;Petrimonas"
6330	pet	Petrimonas sp. IBARAKI	"Prokaryotes;Bacteria;Bacteroidetes;Petrimonas"
6331	psac	Proteiniphilum saccharofermentans	"Prokaryotes;Bacteria;Bacteroidetes;Proteiniphilum"
6332	dys	Dysgonomonas sp. HDW5A	"Prokaryotes;Bacteria;Bacteroidetes;Dysgonomonas"
6333	ppn	Paludibacter propionicigenes	"Prokaryotes;Bacteria;Bacteroidetes;Paludibacter"
6334	pdi	Parabacteroides distasonis	"Prokaryotes;Bacteria;Bacteroidetes;Parabacteroides"
6335	parc	Parabacteroides sp. CT06	"Prokaryotes;Bacteria;Bacteroidetes;Parabacteroides"
6336	tfo	Tannerella forsythia	"Prokaryotes;Bacteria;Bacteroidetes;Tannerella"
6337	toh	Tannerella sp. oral taxon HOT-286	"Prokaryotes;Bacteria;Bacteroidetes;Tannerella"
6338	pary	Muribaculum intestinale	"Prokaryotes;Bacteria;Bacteroidetes;Muribaculum"
6339	dun	Duncaniella sp. B8	"Prokaryotes;Bacteria;Bacteroidetes;Duncaniella"
6340	bvs	Barnesiella viscericola	"Prokaryotes;Bacteria;Bacteroidetes;Barnesiella"

6341	copr	Coprobacter sp. 2CBH44	"Prokaryotes;Bacteria;Bacteroidetes;Coprobacter"
6342	osp	Odoribacter splanchnicus	"Prokaryotes;Bacteria;Bacteroidetes;Odoribacter"
6343	buy	Butyricimonas faecalis	"Prokaryotes;Bacteria;Bacteroidetes;Butyricimonas"
6344	aps	Candidatus Azobacteroides pseudotrichonymphae	"Prokaryotes;Bacteria;Bacteroidetes;Azobacteroides"
6345	pru	Prevotella ruminicola	"Prokaryotes;Bacteria;Bacteroidetes;Prevotella"
6346	pmz	Prevotella melaninogenica	"Prokaryotes;Bacteria;Bacteroidetes;Prevotella"
6347	pdn	Prevotella denticola	"Prokaryotes;Bacteria;Bacteroidetes;Prevotella"
6348	pit	Prevotella intermedia	"Prokaryotes;Bacteria;Bacteroidetes;Prevotella"
6349	pdt	Prevotella dentalis	"Prokaryotes;Bacteria;Bacteroidetes;Prevotella"
6350	pro	Prevotella sp. oral taxon 299	"Prokaryotes;Bacteria;Bacteroidetes;Prevotella"
6351	pfus	Prevotella fusca	"Prokaryotes;Bacteria;Bacteroidetes;Prevotella"
6352	peo	Prevotella enoeca	"Prokaryotes;Bacteria;Bacteroidetes;Prevotella"
6353	pje	Prevotella jejuni	"Prokaryotes;Bacteria;Bacteroidetes;Prevotella"
6354	poc	Prevotella oris	"Prokaryotes;Bacteria;Bacteroidetes;Prevotella"
6355	alq	Alloprevotella sp. E39	"Prokaryotes;Bacteria;Bacteroidetes;Alloprevotella"
6356	afd	Alistipes finegoldii	"Prokaryotes;Bacteria;Bacteroidetes;Alistipes"
6357	ash	Alistipes shahii	"Prokaryotes;Bacteria;Bacteroidetes;Alistipes"
6358	ald	Alistipes sp. dk3624	"Prokaryotes;Bacteria;Bacteroidetes;Alistipes"
6359	aok	Alistipes onderdonkii subsp. vulgaris	"Prokaryotes;Bacteria;Bacteroidetes;Alistipes"
6360	acou	Alistipes communis	"Prokaryotes;Bacteria;Bacteroidetes;Alistipes"
6361	ada	Alistipes dispar	"Prokaryotes;Bacteria;Bacteroidetes;Alistipes"
6362	ait	Alistipes indistinctus	"Prokaryotes;Bacteria;Bacteroidetes;Alistipes"
6363	rbc	Mucinivorans hirudinis	"Prokaryotes;Bacteria;Bacteroidetes;Mucinivorans"
6364	ttz	Tenuifilum thalassicum	"Prokaryotes;Bacteria;Bacteroidetes;Tenuifilum"
6365	blq	Salinivirga cyanobacteriivorans	"Prokaryotes;Bacteria;Bacteroidetes;Salinivirga"
6366	bacc	Bacteroidales bacterium CF	"Prokaryotes;Bacteria;Bacteroidetes;unclassified Bacteroidales"
6367	dori	Draconibacterium orientale	"Prokaryotes;Bacteria;Bacteroidetes;Draconibacterium"
6368	drc	Draconibacterium sp. M1	"Prokaryotes;Bacteria;Bacteroidetes;Draconibacterium"
6369	asx	Alkalitalea saponilacus	"Prokaryotes;Bacteria;Bacteroidetes;Alkalitalea"
6370	mbas	Labilibaculum antarcticum	"Prokaryotes;Bacteria;Bacteroidetes;Labilibaculum"
6371	sru	Salinibacter ruber DSM 13855	"Prokaryotes;Bacteria;Bacteroidetes;Salinibacter"
6372	srm	Salinibacter ruber M8	"Prokaryotes;Bacteria;Bacteroidetes;Salinibacter"
6373	rmr	Rhodothermus marinus DSM 4252	"Prokaryotes;Bacteria;Bacteroidetes;Rhodothermus"
6374	rmg	Rhodothermus marinus SG0.5JP17-172	"Prokaryotes;Bacteria;Bacteroidetes;Rhodothermus"
6375	rbar	Rhodothermaceae bacterium RA	"Prokaryotes;Bacteria;Bacteroidetes;unclassified Rhodothermaceae"
6376	cpi	Chitinophaga pinensis	"Prokaryotes;Bacteria;Bacteroidetes;Chitinophaga"
6377	cbae	Chitinophaga caeni	"Prokaryotes;Bacteria;Bacteroidetes;Chitinophaga"
6378	chit	Chitinophaga sp. XS-30	"Prokaryotes;Bacteria;Bacteroidetes;Chitinophaga"
6379	chih	Chitinophaga agri	"Prokaryotes;Bacteria;Bacteroidetes;Chitinophaga"
6380	nko	Niastella koreensis	"Prokaryotes;Bacteria;Bacteroidetes;Niastella"
6381	nso	Niabella soli	"Prokaryotes;Bacteria;Bacteroidetes;Niabella"

6382	nia	Niabella ginsenosidivorans	"Prokaryotes;Bacteria;Bacteroidetes;Niabella"
6383	fla	Flavisolibacter tropicus	"Prokaryotes;Bacteria;Bacteroidetes;Flavisolibacter"
6384	fgg	Flavisolibacter ginsenosidimutans	"Prokaryotes;Bacteria;Bacteroidetes;Flavisolibacter"
6385	arb	Arachidicoccus sp. BS20	"Prokaryotes;Bacteria;Bacteroidetes;Arachidicoccus"
6386	ark	Arachidicoccus soli	"Prokaryotes;Bacteria;Bacteroidetes;Arachidicoccus"
6387	agi	Arachidicoccus ginsenosidivorans	"Prokaryotes;Bacteria;Bacteroidetes;Arachidicoccus"
6388	arac	Arachidicoccus sp. B3-10	"Prokaryotes;Bacteria;Bacteroidetes;Arachidicoccus"
6389	fln	Filimonas lacunae	"Prokaryotes;Bacteria;Bacteroidetes;Filimonas"
6390	pseg	Paraflavitalea soli	"Prokaryotes;Bacteria;Bacteroidetes;Pseudoflavitalea"
6391	pgin	Panacibacter ginsenosidivorans	"Prokaryotes;Bacteria;Bacteroidetes;Panacibacter"
6392	pgo	Pseudobacter ginsenosidimutans	"Prokaryotes;Bacteria;Bacteroidetes;Pseudobacter"
6393	fls	Flavihumibacter sp. SB-02	"Prokaryotes;Bacteria;Bacteroidetes;Flavihumibacter"
6394	lacs	Lacibacter sp. S13-6-6	"Prokaryotes;Bacteria;Bacteroidetes;Lacibacter"
6395	hhy	Haliscomenobacter hydrossis	"Prokaryotes;Bacteria;Bacteroidetes;Haliscomenobacter"
6396	sgn	Saprospira grandis	"Prokaryotes;Bacteria;Bacteroidetes;Saprospira"
6397	phe	Pedobacter heparinus	"Prokaryotes;Bacteria;Bacteroidetes;Pedobacter"
6398	pep	Pedobacter sp. PACM 27299	"Prokaryotes;Bacteria;Bacteroidetes;Pedobacter"
6399	pcm	Pedobacter cryoconitis	"Prokaryotes;Bacteria;Bacteroidetes;Pedobacter"
6400	psty	Pedobacter steynii	"Prokaryotes;Bacteria;Bacteroidetes;Pedobacter"
6401	pgs	Pedobacter ginsengisoli	"Prokaryotes;Bacteria;Bacteroidetes;Pedobacter"
6402	pej	Pedobacter sp. CJ43	"Prokaryotes;Bacteria;Bacteroidetes;Pedobacter"
6403	pek	Pedobacter sp. KBS0701	"Prokaryotes;Bacteria;Bacteroidetes;Pedobacter"
6404	proe	Pedobacter roseus	"Prokaryotes;Bacteria;Bacteroidetes;Pedobacter"
6405	psn	Pseudopedobacter saltans	"Prokaryotes;Bacteria;Bacteroidetes;Pseudopedobacter"
6406	shg	Sphingobacterium sp. 21	"Prokaryotes;Bacteria;Bacteroidetes;Sphingobacterium"
6407	sht	Sphingobacterium sp. ML3W	"Prokaryotes;Bacteria;Bacteroidetes;Sphingobacterium"
6408	sphn	Sphingobacterium sp. B29	"Prokaryotes;Bacteria;Bacteroidetes;Sphingobacterium"
6409	smiz	Sphingobacterium mizutaii	"Prokaryotes;Bacteria;Bacteroidetes;Sphingobacterium"
6410	spsc	Sphingobacterium psychroaquaticum	"Prokaryotes;Bacteria;Bacteroidetes;Sphingobacterium"
6411	sphz	Sphingobacterium sp. CZ-2	"Prokaryotes;Bacteria;Bacteroidetes;Sphingobacterium"
6412	sphe	Sphingobacterium sp. dk4302	"Prokaryotes;Bacteria;Bacteroidetes;Sphingobacterium"
6413	spdr	Sphingobacterium sp. DR205	"Prokaryotes;Bacteria;Bacteroidetes;Sphingobacterium"
6414	sdj	Sphingobacterium daejeonense	"Prokaryotes;Bacteria;Bacteroidetes;Sphingobacterium"
6415	stha	Sphingobacterium thalpophilum	"Prokaryotes;Bacteria;Bacteroidetes;Sphingobacterium"
6416	scn	Solitalea canadensis	"Prokaryotes;Bacteria;Bacteroidetes;Solitalea"
6417	mup	Mucilaginibacter sp. PAMC 26640	"Prokaryotes;Bacteria;Bacteroidetes;Mucilaginibacter"
6418	muc	Mucilaginibacter xinganensis	"Prokaryotes;Bacteria;Bacteroidetes;Mucilaginibacter"
6419	mgot	Mucilaginibacter gotjawali	"Prokaryotes;Bacteria;Bacteroidetes;Mucilaginibacter"
6420	muh	Mucilaginibacter celer	"Prokaryotes;Bacteria;Bacteroidetes;Mucilaginibacter"
6421	mgin	Mucilaginibacter ginsenosidivorans	"Prokaryotes;Bacteria;Bacteroidetes;Mucilaginibacter"
6422	mgk	Mucilaginibacter ginsenosidivorax	"Prokaryotes;Bacteria;Bacteroidetes;Mucilaginibacter"

6423	mrub	Mucilaginibacter rubeus	"Prokaryotes;Bacteria;Bacteroidetes;Mucilaginibacter"
6424	mgos	Mucilaginibacter gossypii	"Prokaryotes;Bacteria;Bacteroidetes;Mucilaginibacter"
6425	agd	Anseongella ginsenosidimutans	"Prokaryotes;Bacteria;Bacteroidetes;Anseongella"
6426	oli	Olivibacter sp. LS-1	"Prokaryotes;Bacteria;Bacteroidetes;Olivibacter"
6427	sbx	Sphingobacteriaceae bacterium GW460-11-11-14-LB5	"Prokaryotes;Bacteria;Bacteroidetes;unclassified Sphingobacteriaceae"
6428	cmr	Cyclobacterium marinum	"Prokaryotes;Bacteria;Bacteroidetes;Cyclobacterium"
6429	camu	Cyclobacterium amurskyense	"Prokaryotes;Bacteria;Bacteroidetes;Cyclobacterium"
6430	bbd	Belliella baltica	"Prokaryotes;Bacteria;Bacteroidetes;Belliella"
6431	evi	Echinicola vietnamensis	"Prokaryotes;Bacteria;Bacteroidetes;Echinicola"
6432	est	Echinicola strongylocentroti	"Prokaryotes;Bacteria;Bacteroidetes;Echinicola"
6433	echi	Echinicola sp. LN3S3	"Prokaryotes;Bacteria;Bacteroidetes;Echinicola"
6434	alm	Algoriphagus sanaruensis	"Prokaryotes;Bacteria;Bacteroidetes;Algoriphagus"
6435	chu	Cytophaga hutchinsonii	"Prokaryotes;Bacteria;Bacteroidetes;Cytophaga"
6436	dfe	Dyadobacter fermentans	"Prokaryotes;Bacteria;Bacteroidetes;Dyadobacter"
6437	sli	Spirosoma linguale	"Prokaryotes;Bacteria;Bacteroidetes;Spirosoma"
6438	srd	Spirosoma radiotolerans	"Prokaryotes;Bacteria;Bacteroidetes;Spirosoma"
6439	smon	Spirosoma montaniterrae	"Prokaryotes;Bacteria;Bacteroidetes;Spirosoma"
6440	spir	Spirosoma pollinicola	"Prokaryotes;Bacteria;Bacteroidetes;Spirosoma"
6441	spik	Spirosoma sp. KCTC 42546	"Prokaryotes;Bacteria;Bacteroidetes;Spirosoma"
6442	spib	Spirosoma aureum	"Prokaryotes;Bacteria;Bacteroidetes;Spirosoma"
6443	lby	Leadbetterella byssophila	"Prokaryotes;Bacteria;Bacteroidetes;Leadbetterella"
6444	rsi	Runella slithyformis	"Prokaryotes;Bacteria;Bacteroidetes;Runella"
6445	run	Runella sp. HYN0085	"Prokaryotes;Bacteria;Bacteroidetes;Runella"
6446	rup	Runella sp. SP2	"Prokaryotes;Bacteria;Bacteroidetes;Runella"
6447	eol	Emticicia oligotrophica	"Prokaryotes;Bacteria;Bacteroidetes;Emticicia"
6448	fae	Fibrella aestuarina	"Prokaryotes;Bacteria;Bacteroidetes;Fibrella"
6449	fib	Fibrella sp. ES10-3-2-2	"Prokaryotes;Bacteria;Bacteroidetes;Fibrella"
6450	psez	Aquirufa nivalisilvae	"Prokaryotes;Bacteria;Bacteroidetes;Allopseudarcicella"
6451	als	Arcticibacterium luteifluviistationis	"Prokaryotes;Bacteria;Bacteroidetes;Arcticibacterium"
6452	rhoz	Rhodocytophaga sp. 172606-1	"Prokaryotes;Bacteria;Bacteroidetes;Rhodocytophaga"
6453	fli	Bernardetia litoralis	"Prokaryotes;Bacteria;Bacteroidetes;Flexibacter"
6454	hsw	Hymenobacter swuensis	"Prokaryotes;Bacteria;Bacteroidetes;Hymenobacter"
6455	hym	Hymenobacter sp. APR13	"Prokaryotes;Bacteria;Bacteroidetes;Hymenobacter"
6456	hyd	Hymenobacter sp. DG25B	"Prokaryotes;Bacteria;Bacteroidetes;Hymenobacter"
6457	hye	Hymenobacter sp. DG25A	"Prokaryotes;Bacteria;Bacteroidetes;Hymenobacter"
6458	hyg	Hymenobacter sedentarius	"Prokaryotes;Bacteria;Bacteroidetes;Hymenobacter"
6459	hyp	Hymenobacter sp. PAMC 26554	"Prokaryotes;Bacteria;Bacteroidetes;Hymenobacter"
6460	hyz	Hymenobacter sp. PAMC 26628	"Prokaryotes;Bacteria;Bacteroidetes;Hymenobacter"
6461	hnv	Hymenobacter nivis	"Prokaryotes;Bacteria;Bacteroidetes;Hymenobacter"
6462	hyh	Hymenobacter oligotrophus	"Prokaryotes;Bacteria;Bacteroidetes;Hymenobacter"
6463	hyj	Hymenobacter jejuensis	"Prokaryotes;Bacteria;Bacteroidetes;Hymenobacter"

6464	hqi	Hymenobacter qilianensis	"Prokaryotes;Bacteria;Bacteroidetes;Hymenobacter"
6465	hrs	Hymenobacter russus	"Prokaryotes;Bacteria;Bacteroidetes;Hymenobacter"
6466	pko	Pontibacter korlensis	"Prokaryotes;Bacteria;Bacteroidetes;Pontibacter"
6467	pact	Pontibacter actiniarum	"Prokaryotes;Bacteria;Bacteroidetes;Pontibacter"
6468	ruf	Rufibacter radiotolerans	"Prokaryotes;Bacteria;Bacteroidetes;Rufibacter"
6469	rti	Rufibacter tibetensis	"Prokaryotes;Bacteria;Bacteroidetes;Rufibacter"
6470	rud	Rufibacter sp. DG15C	"Prokaryotes;Bacteria;Bacteroidetes;Rufibacter"
6471	nib	Nibribacter ruber	"Prokaryotes;Bacteria;Bacteroidetes;Nibribacter"
6472	add	Adhaeribacter radiodurans	"Prokaryotes;Bacteria;Bacteroidetes;Adhaeribacter"
6473	aswu	Adhaeribacter swui	"Prokaryotes;Bacteria;Bacteroidetes;Adhaeribacter"
6474	mtt	Marivirga tractuosa	"Prokaryotes;Bacteria;Bacteroidetes;Marivirga"
6475	fpf	Roseivirga pacifica	"Prokaryotes;Bacteria;Bacteroidetes;Roseivirga"
6476	flm	Flammeovirga sp. MY04	"Prokaryotes;Bacteria;Bacteroidetes;Flammeovirga"
6477	fll	Flammeovirga pectinis	"Prokaryotes;Bacteria;Bacteroidetes;Flammeovirga"
6478	fbt	Flammeovirgaceae bacterium 311	"Prokaryotes;Bacteria;Bacteroidetes;unclassified Flammeovirgaceae"
6479	aas	Candidatus Amoebophilus asiaticus	"Prokaryotes;Bacteria;Bacteroidetes;Amoebophilus"
6480	che	Cardinium endosymbiont cEper1 of Encarsia pergandiella	"Prokaryotes;Bacteria;Bacteroidetes;Cardinium"
6481	cec	Cardinium endosymbiont of Sogatella furcifera	"Prokaryotes;Bacteria;Bacteroidetes;Cardinium"
6482	cher	Candidatus Cardinium hertigii	"Prokaryotes;Bacteria;Bacteroidetes;Cardinium"
6483	chk	Chryseolinea soli	"Prokaryotes;Bacteria;Bacteroidetes;Chryseolinea"
6484	gfo	Gramella forsetii	"Prokaryotes;Bacteria;Bacteroidetes;Gramella"
6485	grl	Gramella salexigens	"Prokaryotes;Bacteria;Bacteroidetes;Gramella"
6486	gfl	Gramella flava	"Prokaryotes;Bacteria;Bacteroidetes;Gramella"
6487	grs	Gramella fulva	"Prokaryotes;Bacteria;Bacteroidetes;Gramella"
6488	fps	Flavobacterium psychrophilum JIP02/86	"Prokaryotes;Bacteria;Bacteroidetes;Flavobacterium"
6489	fpc	Flavobacterium psychrophilum CSF259-93	"Prokaryotes;Bacteria;Bacteroidetes;Flavobacterium"
6490	fpy	Flavobacterium psychrophilum FPG101	"Prokaryotes;Bacteria;Bacteroidetes;Flavobacterium"
6491	fpo	Flavobacterium psychrophilum FPG3	"Prokaryotes;Bacteria;Bacteroidetes;Flavobacterium"
6492	fpq	Flavobacterium psychrophilum 950106-1/1	"Prokaryotes;Bacteria;Bacteroidetes;Flavobacterium"
6493	fpv	Flavobacterium psychrophilum V3-5	"Prokaryotes;Bacteria;Bacteroidetes;Flavobacterium"
6494	fpw	Flavobacterium psychrophilum V4-24	"Prokaryotes;Bacteria;Bacteroidetes;Flavobacterium"
6495	fpk	Flavobacterium psychrophilum v4-33	"Prokaryotes;Bacteria;Bacteroidetes;Flavobacterium"
6496	fpsz	Flavobacterium psychrophilum Z2	"Prokaryotes;Bacteria;Bacteroidetes;Flavobacterium"
6497	fjo	Flavobacterium johnsoniae UW101	"Prokaryotes;Bacteria;Bacteroidetes;Flavobacterium"
6498	fjg	Flavobacterium anhuiense	"Prokaryotes;Bacteria;Bacteroidetes;Flavobacterium"
6499	fbr	Flavobacterium branchiophilum	"Prokaryotes;Bacteria;Bacteroidetes;Flavobacterium"
6500	fco	Flavobacterium columnare	"Prokaryotes;Bacteria;Bacteroidetes;Flavobacterium"
6501	fin	Flavobacterium indicum	"Prokaryotes;Bacteria;Bacteroidetes;Flavobacterium"
6502	fgl	Flavobacterium gilvum	"Prokaryotes;Bacteria;Bacteroidetes;Flavobacterium"
6503	fcm	Flavobacterium commune	"Prokaryotes;Bacteria;Bacteroidetes;Flavobacterium"
6504	ffa	Flavobacterium faecale	"Prokaryotes;Bacteria;Bacteroidetes;Flavobacterium"

6505	fat	Flavobacterium arcticum	"Prokaryotes;Bacteria;Bacteroidetes;Flavobacterium"
6506	fki	Flavobacterium kingsejongi	"Prokaryotes;Bacteria;Bacteroidetes;Flavobacterium"
6507	fpal	Flavobacterium pallidum	"Prokaryotes;Bacteria;Bacteroidetes;Flavobacterium"
6508	fmg	Flavobacterium magnum	"Prokaryotes;Bacteria;Bacteroidetes;Flavobacterium"
6509	falb	Flavobacterium album	"Prokaryotes;Bacteria;Bacteroidetes;Flavobacterium"
6510	fcr	Flavobacterium crocinum	"Prokaryotes;Bacteria;Bacteroidetes;Flavobacterium"
6511	fse	Flavobacterium sediminis	"Prokaryotes;Bacteria;Bacteroidetes;Flavobacterium"
6512	fsn	Flavobacterium sangjuense	"Prokaryotes;Bacteria;Bacteroidetes;Flavobacterium"
6513	fnk	Flavobacterium nackdongense	"Prokaryotes;Bacteria;Bacteroidetes;Flavobacterium"
6514	fak	Flavobacterium alkalisoli	"Prokaryotes;Bacteria;Bacteroidetes;Flavobacterium"
6515	coc	Capnocytophaga ochracea	"Prokaryotes;Bacteria;Bacteroidetes;Capnocytophaga"
6516	ccm	Capnocytophaga canimorsus	"Prokaryotes;Bacteria;Bacteroidetes;Capnocytophaga"
6517	col	Capnocytophaga sp. oral taxon 323	"Prokaryotes;Bacteria;Bacteroidetes;Capnocytophaga"
6518	chg	Capnocytophaga haemolytica	"Prokaryotes;Bacteria;Bacteroidetes;Capnocytophaga"
6519	capn	Capnocytophaga endodontalis	"Prokaryotes;Bacteria;Bacteroidetes;Capnocytophaga"
6520	cgh	Capnocytophaga gingivalis	"Prokaryotes;Bacteria;Bacteroidetes;Capnocytophaga"
6521	clk	Capnocytophaga leadbetteri	"Prokaryotes;Bacteria;Bacteroidetes;Capnocytophaga"
6522	cspu	Capnocytophaga sputigena	"Prokaryotes;Bacteria;Bacteroidetes;Capnocytophaga"
6523	ccyn	Capnocytophaga cynodegmi	"Prokaryotes;Bacteria;Bacteroidetes;Capnocytophaga"
6524	caph	Capnocytophaga sp. H4358	"Prokaryotes;Bacteria;Bacteroidetes;Capnocytophaga"
6525	csto	Capnocytophaga stomatis	"Prokaryotes;Bacteria;Bacteroidetes;Capnocytophaga"
6526	capq	Capnocytophaga sp. H2931	"Prokaryotes;Bacteria;Bacteroidetes;Capnocytophaga"
6527	capf	Capnocytophaga sp. FDAARGOS_737	"Prokaryotes;Bacteria;Bacteroidetes;Capnocytophaga"
6528	rbi	Robiginitalea biformata	"Prokaryotes;Bacteria;Bacteroidetes;Robiginitalea"
6529	zpr	Zunongwangia profunda	"Prokaryotes;Bacteria;Bacteroidetes;Zunongwangia"
6530	cat	Croceibacter atlanticus	"Prokaryotes;Bacteria;Bacteroidetes;Croceibacter"
6531	fbc	Maribacter sp. HTCC2170	"Prokaryotes;Bacteria;Bacteroidetes;Maribacter"
6532	marm	Maribacter sp. 1_2014MBL_MicDiv	"Prokaryotes;Bacteria;Bacteroidetes;Maribacter"
6533	mart	Maribacter sp. T28	"Prokaryotes;Bacteria;Bacteroidetes;Maribacter"
6534	marb	Maribacter cobaltidurans	"Prokaryotes;Bacteria;Bacteroidetes;Maribacter"
6535	mare	Maribacter sp. MJ134	"Prokaryotes;Bacteria;Bacteroidetes;Maribacter"
6536	cao	Cellulophaga algicola	"Prokaryotes;Bacteria;Bacteroidetes;Cellulophaga"
6537	cly	Cellulophaga lytica DSM 7489	"Prokaryotes;Bacteria;Bacteroidetes;Cellulophaga"
6538	clh	Cellulophaga lytica HI1	"Prokaryotes;Bacteria;Bacteroidetes;Cellulophaga"
6539	cbal	Cellulophaga baltica NN016038	"Prokaryotes;Bacteria;Bacteroidetes;Cellulophaga"
6540	cbat	Cellulophaga baltica 18	"Prokaryotes;Bacteria;Bacteroidetes;Cellulophaga"
6541	kdi	Dokdonia sp. 4H-3-7-5	"Prokaryotes;Bacteria;Bacteroidetes;Dokdonia"
6542	dok	Dokdonia sp. MED134	"Prokaryotes;Bacteria;Bacteroidetes;Dokdonia"
6543	ddo	Dokdonia donghaensis	"Prokaryotes;Bacteria;Bacteroidetes;Dokdonia"
6544	dod	Dokdonia sp. Dokd-P16	"Prokaryotes;Bacteria;Bacteroidetes;Dokdonia"
6545	lan	Lacinutrix sp. 5H-3-7-4	"Prokaryotes;Bacteria;Bacteroidetes;Lacinutrix"

6546	lvn	Lacinutrix venerupis	"Prokaryotes;Bacteria;Bacteroidetes;Lacinutrix"
6547	laci	Lacinutrix sp. Bg11-31	"Prokaryotes;Bacteria;Bacteroidetes;Lacinutrix"
6548	zga	Zobellia galactanivorans	"Prokaryotes;Bacteria;Bacteroidetes;Zobellia"
6549	mrs	Muricauda ruestringensis	"Prokaryotes;Bacteria;Bacteroidetes;Muricauda"
6550	mlt	Muricauda lutaonensis	"Prokaryotes;Bacteria;Bacteroidetes;Muricauda"
6551	mut	Muricauda sp. 501str8	"Prokaryotes;Bacteria;Bacteroidetes;Muricauda"
6552	asl	Aequorivita sublithincola	"Prokaryotes;Bacteria;Bacteroidetes;Aequorivita"
6553	aev	Aequorivita sp. H23M31	"Prokaryotes;Bacteria;Bacteroidetes;Aequorivita"
6554	ptq	Psychroflexus torquis	"Prokaryotes;Bacteria;Bacteroidetes;Psychroflexus"
6555	ndo	Nonlabens dokdonensis	"Prokaryotes;Bacteria;Bacteroidetes;Nonlabens"
6556	nom	Nonlabens sp. MIC269	"Prokaryotes;Bacteria;Bacteroidetes;Nonlabens"
6557	nsd	Nonlabens sediminis	"Prokaryotes;Bacteria;Bacteroidetes;Nonlabens"
6558	nob	Nonlabens sp. MB-3u-79	"Prokaryotes;Bacteria;Bacteroidetes;Nonlabens"
6559	noj	Nonlabens ponticola	"Prokaryotes;Bacteria;Bacteroidetes;Nonlabens"
6560	pom	Polaribacter sp. MED152	"Prokaryotes;Bacteria;Bacteroidetes;Polaribacter"
6561	pob	Polaribacter vadi	"Prokaryotes;Bacteria;Bacteroidetes;Polaribacter"
6562	prn	Polaribacter reichenbachii	"Prokaryotes;Bacteria;Bacteroidetes;Polaribacter"
6563	pola	Polaribacter sp. BM10	"Prokaryotes;Bacteria;Bacteroidetes;Polaribacter"
6564	poa	Polaribacter sp. ALD11	"Prokaryotes;Bacteria;Bacteroidetes;Polaribacter"
6565	phal	Polaribacter haliotis	"Prokaryotes;Bacteria;Bacteroidetes;Polaribacter"
6566	myr	Myroides sp. A21	"Prokaryotes;Bacteria;Bacteroidetes;Myroides"
6567	mpw	Myroides profundi	"Prokaryotes;Bacteria;Bacteroidetes;Myroides"
6568	mod	Myroides odoratimimus	"Prokaryotes;Bacteria;Bacteroidetes;Myroides"
6569	myz	Myroides sp. ZB35	"Prokaryotes;Bacteria;Bacteroidetes;Myroides"
6570	win	Winogradskyella sp. PG-2	"Prokaryotes;Bacteria;Bacteroidetes;Winogradskyella"
6571	wij	Winogradskyella sp. J14-2	"Prokaryotes;Bacteria;Bacteroidetes;Winogradskyella"
6572	sze	Siansivirga zeaxanthinifaciens	"Prokaryotes;Bacteria;Bacteroidetes;Siansivirga"
6573	ahz	Algibacter alginicilyticus	"Prokaryotes;Bacteria;Bacteroidetes;Algibacter"
6574	syi	Sediminicola sp. YIK13	"Prokaryotes;Bacteria;Bacteroidetes;Sediminicola"
6575	tdi	Tenacibaculum dicentrarchi	"Prokaryotes;Bacteria;Bacteroidetes;Tenacibaculum"
6576	ten	Tenacibaculum todarodis	"Prokaryotes;Bacteria;Bacteroidetes;Tenacibaculum"
6577	tje	Tenacibaculum jejuense	"Prokaryotes;Bacteria;Bacteroidetes;Tenacibaculum"
6578	tmar	Tenacibaculum maritimum	"Prokaryotes;Bacteria;Bacteroidetes;Tenacibaculum"
6579	tmp	Tenacibaculum mesophilum	"Prokaryotes;Bacteria;Bacteroidetes;Tenacibaculum"
6580	lut	Lutibacter profundi	"Prokaryotes;Bacteria;Bacteroidetes;Lutibacter"
6581	lul	Urechidicola croceus	"Prokaryotes;Bacteria;Bacteroidetes;Urechidicola"
6582	wfu	Wenyingzhuangia fucanilytica	"Prokaryotes;Bacteria;Bacteroidetes;Wenyingzhuangia"
6583	for	Formosa sp. Hel1_33_131	"Prokaryotes;Bacteria;Bacteroidetes;Formosa"
6584	foh	Formosa sp. Hel3_A1_48	"Prokaryotes;Bacteria;Bacteroidetes;Formosa"
6585	fop	Formosa sediminum	"Prokaryotes;Bacteria;Bacteroidetes;Formosa"
6586	salt	Salegentibacter sp. T436	"Prokaryotes;Bacteria;Bacteroidetes;Salegentibacter"

6587	seon	Seonamhaeicola sp. S2-3	"Prokaryotes;Bacteria;Bacteroidetes;Seonamhaeicola"
6588	aalg	Arenibacter algicola	"Prokaryotes;Bacteria;Bacteroidetes;Arenibacter"
6589	oll	Olleya sp. Bg11-27	"Prokaryotes;Bacteria;Bacteroidetes;Olleya"
6590	oaq	Olleya aquimaris	"Prokaryotes;Bacteria;Bacteroidetes;Olleya"
6591	fek	Flavivirga eckloniae	"Prokaryotes;Bacteria;Bacteroidetes;Flavivirga"
6592	taj	Tamlana carrageenivorans	"Prokaryotes;Bacteria;Bacteroidetes;Tamlana"
6593	aue	Aureitalea sp. RR4-38	"Prokaryotes;Bacteria;Bacteroidetes;Aureitalea"
6594	spon	Flagellimonas maritima	"Prokaryotes;Bacteria;Bacteroidetes;Spongiibacterium"
6595	kos	Kordia sp. SMS9	"Prokaryotes;Bacteria;Bacteroidetes;Kordia"
6596	kan	Kordia antarctica	"Prokaryotes;Bacteria;Bacteroidetes;Kordia"
6597	marf	Mariniflexile sp. TRM1-10	"Prokaryotes;Bacteria;Bacteroidetes;Mariniflexile"
6598	aqb	Aquimarina sp. BL5	"Prokaryotes;Bacteria;Bacteroidetes;Aquimarina"
6599	aqa	Aquimarina sp. AD1	"Prokaryotes;Bacteria;Bacteroidetes;Aquimarina"
6600	aqd	Aquimarina sp. AD10	"Prokaryotes;Bacteria;Bacteroidetes;Aquimarina"
6601	emar	Euzebyella marina	"Prokaryotes;Bacteria;Bacteroidetes;Euzebyella"
6602	mur	Muriicola sp. MMS17-SY002	"Prokaryotes;Bacteria;Bacteroidetes;Muriicola"
6603	psyn	Psychroserpens sp. NJDZ02	"Prokaryotes;Bacteria;Bacteroidetes;Psychroserpens"
6604	afla	Antarcticibacterium flavum	"Prokaryotes;Bacteria;Bacteroidetes;Antarcticibacterium"
6605	anp	Antarcticibacterium arcticum	"Prokaryotes;Bacteria;Bacteroidetes;Antarcticibacterium"
6606	oci	Oceanihabitans sp. IOP_32	"Prokaryotes;Bacteria;Bacteroidetes;Oceanihabitans"
6607	mgel	Marinirhabdus gelatinilytica	"Prokaryotes;Bacteria;Bacteroidetes;Marinirhabdus"
6608	mesq	Mesoflavibacter sp. HG96	"Prokaryotes;Bacteria;Bacteroidetes;Mesoflavibacter"
6609	gaa	Galbibacter sp. bg1	"Prokaryotes;Bacteria;Bacteroidetes;Galbibacter"
6610	cagg	Costertonia aggregata	"Prokaryotes;Bacteria;Bacteroidetes;Costertonia"
6611	alti	Altibacter sp. ALE3EI	"Prokaryotes;Bacteria;Bacteroidetes;Altibacter"
6612	fba	Flavobacteriaceae bacterium 3519-10	"Prokaryotes;Bacteria;Bacteroidetes;unclassified Flavobacteriaceae"
6613	fbu	Flavobacteriaceae bacterium UJ101	"Prokaryotes;Bacteria;Bacteroidetes;unclassified Flavobacteriaceae"
6614	fbe	Flavobacteriaceae bacterium 10Alg115	"Prokaryotes;Bacteria;Bacteroidetes;unclassified Flavobacteriaceae"
6615	ran	Riemerella anatipestifer ATCC 11845 = DSM 15868	"Prokaryotes;Bacteria;Bacteroidetes;Riemerella"
6616	rai	Riemerella anatipestifer ATCC 11845 = DSM 15868	"Prokaryotes;Bacteria;Bacteroidetes;Riemerella"
6617	rar	Riemerella anatipestifer RA-GD	"Prokaryotes;Bacteria;Bacteroidetes;Riemerella"
6618	rag	Riemerella anatipestifer RA-CH-1	"Prokaryotes;Bacteria;Bacteroidetes;Riemerella"
6619	rae	Riemerella anatipestifer RA-CH-2	"Prokaryotes;Bacteria;Bacteroidetes;Riemerella"
6620	rat	Riemerella anatipestifer CH3	"Prokaryotes;Bacteria;Bacteroidetes;Riemerella"
6621	wvi	Weeksella virosa	"Prokaryotes;Bacteria;Bacteroidetes;Weeksella"
6622	orh	Ornithobacterium rhinotracheale DSM 15997	"Prokaryotes;Bacteria;Bacteroidetes;Ornithobacterium"
6623	ori	Ornithobacterium rhinotracheale ORT-UMN 88	"Prokaryotes;Bacteria;Bacteroidetes;Ornithobacterium"
6624	eao	Elizabethkingia anophelis NUHP1	"Prokaryotes;Bacteria;Bacteroidetes;Elizabethkingia"
6625	emn	Elizabethkingia anophelis FMS-007	"Prokaryotes;Bacteria;Bacteroidetes;Elizabethkingia"
6626	een	Elizabethkingia anophelis JM-87	"Prokaryotes;Bacteria;Bacteroidetes;Elizabethkingia"
6627	elb	Elizabethkingia miricola	"Prokaryotes;Bacteria;Bacteroidetes;Elizabethkingia"

6628	emg	Elizabethkingia meningoseptica	"Prokaryotes;Bacteria;Bacteroidetes;Elizabethkingia"
6629	ego	Elizabethkingia ursingii	"Prokaryotes;Bacteria;Bacteroidetes;Elizabethkingia"
6630	egm	Elizabethkingia bruuniana	"Prokaryotes;Bacteria;Bacteroidetes;Elizabethkingia"
6631	elz	Elizabethkingia sp. 2-6	"Prokaryotes;Bacteria;Bacteroidetes;Elizabethkingia"
6632	elt	Elizabethkingia sp. JS20170427COW	"Prokaryotes;Bacteria;Bacteroidetes;Elizabethkingia"
6633	chz	Chryseobacterium sp. StRB126	"Prokaryotes;Bacteria;Bacteroidetes;Chryseobacterium"
6634	cgn	Chryseobacterium gallinarum	"Prokaryotes;Bacteria;Bacteroidetes;Chryseobacterium"
6635	cih	Chryseobacterium sp. IHB B 17019	"Prokaryotes;Bacteria;Bacteroidetes;Chryseobacterium"
6636	chh	Chryseobacterium glaciei	"Prokaryotes;Bacteria;Bacteroidetes;Chryseobacterium"
6637	cio	Chryseobacterium indologenes	"Prokaryotes;Bacteria;Bacteroidetes;Chryseobacterium"
6638	chry	Chryseobacterium sp. T16E-39	"Prokaryotes;Bacteria;Bacteroidetes;Chryseobacterium"
6639	cpip	Chryseobacterium piperi	"Prokaryotes;Bacteria;Bacteroidetes;Chryseobacterium"
6640	chrs	Chryseobacterium sp. 3008163	"Prokaryotes;Bacteria;Bacteroidetes;Chryseobacterium"
6641	chrz	Chryseobacterium sp. 6424	"Prokaryotes;Bacteria;Bacteroidetes;Chryseobacterium"
6642	carh	Chryseobacterium arthrosphaerae	"Prokaryotes;Bacteria;Bacteroidetes;Chryseobacterium"
6643	csha	Chryseobacterium shandongense	"Prokaryotes;Bacteria;Bacteroidetes;Chryseobacterium"
6644	cnk	Chryseobacterium nakagawai	"Prokaryotes;Bacteria;Bacteroidetes;Chryseobacterium"
6645	cjt	Chryseobacterium joostei	"Prokaryotes;Bacteria;Bacteroidetes;Chryseobacterium"
6646	cil	Chryseobacterium indoltheticum	"Prokaryotes;Bacteria;Bacteroidetes;Chryseobacterium"
6647	ccau	Chryseobacterium carnipullorum	"Prokaryotes;Bacteria;Bacteroidetes;Chryseobacterium"
6648	cben	Chryseobacterium bernardetii	"Prokaryotes;Bacteria;Bacteroidetes;Chryseobacterium"
6649	cjg	Kaistella jeonii	"Prokaryotes;Bacteria;Bacteroidetes;Kaistella"
6650	ccas	Chryseobacterium carnis	"Prokaryotes;Bacteria;Bacteroidetes;Kaistella"
6651	cant	Kaistella antarctica	"Prokaryotes;Bacteria;Bacteroidetes;Kaistella"
6652	kda	Kaistella daneshvariae	"Prokaryotes;Bacteria;Bacteroidetes;Kaistella"
6653	clac	Chryseobacterium lactis	"Prokaryotes;Bacteria;Bacteroidetes;Epilithonimonas"
6654	eva	Epilithonimonas vandammei	"Prokaryotes;Bacteria;Bacteroidetes;Epilithonimonas"
6655	ctak	Chryseobacterium taklimakanense	"Prokaryotes;Bacteria;Bacteroidetes;Planobacterium"
6656	cnr	Cloacibacterium normanense	"Prokaryotes;Bacteria;Bacteroidetes;Cloacibacterium"
6657	ebv	Empedobacter brevis	"Prokaryotes;Bacteria;Bacteroidetes;Empedobacter"
6658	efal	Empedobacter falsenii	"Prokaryotes;Bacteria;Bacteroidetes;Empedobacter"
6659	este	Empedobacter stercoris	"Prokaryotes;Bacteria;Bacteroidetes;Empedobacter"
6660	bcad	Bergeyella cardium	"Prokaryotes;Bacteria;Bacteroidetes;Bergeyella"
6661	apib	Apibacter sp. B2966	"Prokaryotes;Bacteria;Bacteroidetes;Apibacter"
6662	civ	Cruoricaptor ignavus	"Prokaryotes;Bacteria;Bacteroidetes;Cruoricaptor"
6663	smg	Candidatus Sulcia muelleri GWSS	"Prokaryotes;Bacteria;Bacteroidetes;Sulcia"
6664	sms	Candidatus Sulcia muelleri SMDSEM	"Prokaryotes;Bacteria;Bacteroidetes;Sulcia"
6665	smh	Candidatus Sulcia muelleri DMIN	"Prokaryotes;Bacteria;Bacteroidetes;Sulcia"
6666	sum	Candidatus Sulcia muelleri CARI	"Prokaryotes;Bacteria;Bacteroidetes;Sulcia"
6667	smv	Candidatus Sulcia muelleri Sulcia-ALF	"Prokaryotes;Bacteria;Bacteroidetes;Sulcia"
6668	smub	Candidatus Sulcia muelleri BGSS	"Prokaryotes;Bacteria;Bacteroidetes;Sulcia"

6669	smum	Candidatus Sulcia muelleri ML	"Prokaryotes;Bacteria;Bacteroidetes;Sulcia"
6670	smue	Candidatus Sulcia muelleri TETUND	"Prokaryotes;Bacteria;Bacteroidetes;Sulcia"
6671	smup	Candidatus Sulcia muelleri PSPU	"Prokaryotes;Bacteria;Bacteroidetes;Sulcia"
6672	bbl	Blattabacterium sp. (Blattella germanica) Bge	"Prokaryotes;Bacteria;Bacteroidetes;Blattabacterium"
6673	bpi	Blattabacterium sp. BPLAN (Periplaneta americana)	"Prokaryotes;Bacteria;Bacteroidetes;Blattabacterium"
6674	bmm	Blattabacterium sp. (Mastotermes darwiniensis)	"Prokaryotes;Bacteria;Bacteroidetes;Blattabacterium"
6675	bcp	Blattabacterium Cpu (Cryptocercus punctulatus)	"Prokaryotes;Bacteria;Bacteroidetes;Blattabacterium"
6676	bbg	Blattabacterium sp. (Blaberus giganteus)	"Prokaryotes;Bacteria;Bacteroidetes;Blattabacterium"
6677	bbq	Blattabacterium sp. (Blatta orientalis)	"Prokaryotes;Bacteria;Bacteroidetes;Blattabacterium"
6678	blp	Blattabacterium cuenoti	"Prokaryotes;Bacteria;Bacteroidetes;Blattabacterium"
6679	blu	Blattabacterium sp. (Nauphoeta cinerea)	"Prokaryotes;Bacteria;Bacteroidetes;Blattabacterium"
6680	blck	Blattabacterium sp. (Cryptocercus kyebangensis)	"Prokaryotes;Bacteria;Bacteroidetes;Blattabacterium"
6681	fte	Fluviicola taffensis	"Prokaryotes;Bacteria;Bacteroidetes;Fluviicola"
6682	flu	Candidatus Fluviicola riflensis	"Prokaryotes;Bacteria;Bacteroidetes;Fluviicola"
6683	oho	Owenweeksia hongkongensis	"Prokaryotes;Bacteria;Bacteroidetes;Owenweeksia"
6684	ise	Ichthyobacterium seriolicida	"Prokaryotes;Bacteria;Bacteroidetes;Ichthyobacterium"
6685	elv	Candidatus Walczuchella monophlebidarum	"Prokaryotes;Bacteria;Bacteroidetes;Walczuchella"
6686	udi	Candidatus Uzinura diaspidicola	"Prokaryotes;Bacteria;Bacteroidetes;Uzinura"
6687	bbau	Bacteroidetes bacterium	"Prokaryotes;Bacteria;Bacteroidetes;unclassified Bacteroidetes"
6688	cte	Chlorobaculum tepidum	"Prokaryotes;Bacteria;Chlorobi;Chlorobaculum"
6689	cpc	Chlorobaculum parvum	"Prokaryotes;Bacteria;Chlorobi;Chlorobaculum"
6690	clz	Chlorobaculum limnaeum	"Prokaryotes;Bacteria;Chlorobi;Chlorobaculum"
6691	cch	Chlorobium chlorochromatii	"Prokaryotes;Bacteria;Chlorobi;Chlorobium"
6692	cph	Chlorobium phaeobacteroides DSM 266	"Prokaryotes;Bacteria;Chlorobi;Chlorobium"
6693	cpb	Chlorobium phaeobacteroides BS1	"Prokaryotes;Bacteria;Chlorobi;Chlorobium"
6694	cli	Chlorobium limicola	"Prokaryotes;Bacteria;Chlorobi;Chlorobium"
6695	pvi	Chlorobium phaeovibrioides	"Prokaryotes;Bacteria;Chlorobi;Chlorobium"
6696	plt	Chlorobium luteolum	"Prokaryotes;Bacteria;Chlorobi;Pelodictyon"
6697	pph	Pelodictyon phaeoclathratiforme	"Prokaryotes;Bacteria;Chlorobi;Pelodictyon"
6698	paa	Prosthecochloris aestuarii	"Prokaryotes;Bacteria;Chlorobi;Prosthecochloris"
6699	proc	Prosthecochloris sp. CIB 2401	"Prokaryotes;Bacteria;Chlorobi;Prosthecochloris"
6700	prs	Prosthecochloris sp. HL-130-GSB	"Prokaryotes;Bacteria;Chlorobi;Prosthecochloris"
6701	pros	Prosthecochloris sp. GSB1	"Prokaryotes;Bacteria;Chlorobi;Prosthecochloris"
6702	cts	Chloroherpeton thalassium	"Prokaryotes;Bacteria;Chlorobi;Chloroherpeton"
6703	ial	Ignavibacterium album	"Prokaryotes;Bacteria;Chlorobi;Ignavibacterium"
6704	mro	Melioribacter roseus	"Prokaryotes;Bacteria;Chlorobi;Melioribacter"
6705	cprv	Candidatus Cyclonatronum proteinivorum	"Prokaryotes;Bacteria;Balneolaeota;Cyclonatronum"
6706	caci	Candidatus Cloacimonas acidaminovorans	"Prokaryotes;Bacteria;Cloacimonetes;Cloacimonas"
6707	aae	Aquifex aeolicus	"Prokaryotes;Bacteria;Aquificae;Aquifex"
6708	hya	Hydrogenobaculum sp. Y04AAS1	"Prokaryotes;Bacteria;Aquificae;Hydrogenobaculum"
6709	hho	Hydrogenobaculum sp. HO	"Prokaryotes;Bacteria;Aquificae;Hydrogenobaculum"

6710	hys	Hydrogenobaculum sp. SN	"Prokaryotes;Bacteria;Aquificae;Hydrogenobaculum"
6711	hth	Hydrogenobacter thermophilus	"Prokaryotes;Bacteria;Aquificae;Hydrogenobacter"
6712	hte	Hydrogenobacter thermophilus	"Prokaryotes;Bacteria;Aquificae;Hydrogenobacter"
6713	tal	Thermocrinis albus	"Prokaryotes;Bacteria;Aquificae;Thermocrinis"
6714	trd	Thermocrinis ruber	"Prokaryotes;Bacteria;Aquificae;Thermocrinis"
6715	sul	Sulfurihydrogenibium sp. YO3AOP1	"Prokaryotes;Bacteria;Aquificae;Sulfurihydrogenibium"
6716	saf	Sulfurihydrogenibium azorense	"Prokaryotes;Bacteria;Aquificae;Sulfurihydrogenibium"
6717	pmx	Persephonella marina	"Prokaryotes;Bacteria;Aquificae;Persephonella"
6718	ttk	Thermosulfidibacter takaii	"Prokaryotes;Bacteria;Aquificae;Thermosulfidibacter"
6719	tam	Thermovibrio ammonificans	"Prokaryotes;Bacteria;Aquificae;Thermovibrio"
6720	dte	Desulfurobacterium thermolithotrophum	"Prokaryotes;Bacteria;Aquificae;Desulfurobacterium"
6721	tma	Thermotoga maritima MSB8	"Prokaryotes;Bacteria;Thermotogae;Thermotoga"
6722	tmm	Thermotoga maritima MSB8	"Prokaryotes;Bacteria;Thermotogae;Thermotoga"
6723	tmi	Thermotoga maritima MSB8	"Prokaryotes;Bacteria;Thermotogae;Thermotoga"
6724	tmw	Thermotoga maritima MSB8	"Prokaryotes;Bacteria;Thermotogae;Thermotoga"
6725	tmq	Thermotoga maritima Tma100	"Prokaryotes;Bacteria;Thermotogae;Thermotoga"
6726	tmx	Thermotoga maritima Tma200	"Prokaryotes;Bacteria;Thermotogae;Thermotoga"
6727	tpt	Thermotoga petrophila	"Prokaryotes;Bacteria;Thermotogae;Thermotoga"
6728	trq	Thermotoga sp. RQ2	"Prokaryotes;Bacteria;Thermotogae;Thermotoga"
6729	tna	Thermotoga neapolitana	"Prokaryotes;Bacteria;Thermotogae;Thermotoga"
6730	tnp	Thermotoga naphthophila	"Prokaryotes;Bacteria;Thermotogae;Thermotoga"
6731	thq	Thermotoga sp. 2812B	"Prokaryotes;Bacteria;Thermotogae;Thermotoga"
6732	thz	Thermotoga sp. Cell2	"Prokaryotes;Bacteria;Thermotogae;Thermotoga"
6733	thr	Thermotoga sp. RQ7	"Prokaryotes;Bacteria;Thermotogae;Thermotoga"
6734	tle	Pseudothermotoga lettingae	"Prokaryotes;Bacteria;Thermotogae;Pseudothermotoga"
6735	tta	Pseudothermotoga thermarum	"Prokaryotes;Bacteria;Thermotogae;Pseudothermotoga"
6736	phy	Pseudothermotoga hypogea	"Prokaryotes;Bacteria;Thermotogae;Pseudothermotoga"
6737	tme	Thermosipho melanesiensis	"Prokaryotes;Bacteria;Thermotogae;Thermosipho"
6738	taf	Thermosipho africanus	"Prokaryotes;Bacteria;Thermotogae;Thermosipho"
6739	thp	Thermosipho sp. 1063	"Prokaryotes;Bacteria;Thermotogae;Thermosipho"
6740	ther	Thermosipho sp. 1070	"Prokaryotes;Bacteria;Thermotogae;Thermosipho"
6741	fno	Fervidobacterium nodosum	"Prokaryotes;Bacteria;Thermotogae;Fervidobacterium"
6742	fpe	Fervidobacterium pennivorans	"Prokaryotes;Bacteria;Thermotogae;Fervidobacterium"
6743	fia	Fervidobacterium islandicum	"Prokaryotes;Bacteria;Thermotogae;Fervidobacterium"
6744	ocy	Tepiditoga spiralis	"Prokaryotes;Bacteria;Thermotogae;Tepiditoga"
6745	pmo	Petrotoga mobilis	"Prokaryotes;Bacteria;Thermotogae;Petrotoga"
6746	mpz	Marinitoga piezophila	"Prokaryotes;Bacteria;Thermotogae;Marinitoga"
6747	marn	Marinitoga sp. 1137	"Prokaryotes;Bacteria;Thermotogae;Marinitoga"
6748	dtn	Defluviitoga tunisiensis	"Prokaryotes;Bacteria;Thermotogae;Defluviitoga"
6749	kol	Kosmotoga olearia	"Prokaryotes;Bacteria;Thermotogae;Kosmotoga"
6750	kpf	Kosmotoga pacifica	"Prokaryotes;Bacteria;Thermotogae;Kosmotoga"

6751	mpg	Mesotoga prima	"Prokaryotes;Bacteria;Thermotogae;Mesotoga"
6752	minf	Mesotoga infera	"Prokaryotes;Bacteria;Thermotogae;Mesotoga"
6753	asac	Athalassotoga saccharophila	"Prokaryotes;Bacteria;Thermotogae;Athalassotoga"
6754	cpo	Coprothermobacter proteolyticus	"Prokaryotes;Bacteria;Coprothermobacterota;Coprothermobacter"
6755	cex	Caldisericum exile	"Prokaryotes;Bacteria;Caldiserica;Caldisericum"
6756	din	Desulfurispirillum indicum	"Prokaryotes;Bacteria;Chrysiogenetes;Desulfurispirillum"
6757	ddf	Deferribacter desulfuricans	"Prokaryotes;Bacteria;Deferribacteres;Deferribacter"
6758	dap	Denitrovibrio acetiphilus	"Prokaryotes;Bacteria;Deferribacteres;Denitrovibrio"
6759	cni	Calditerrivibrio nitroreducens	"Prokaryotes;Bacteria;Deferribacteres;Calditerrivibrio"
6760	fsi	Flexistipes sinusarabici	"Prokaryotes;Bacteria;Deferribacteres;Flexistipes"
6761	gtl	Geovibrio thiophilus	"Prokaryotes;Bacteria;Deferribacteres;Geovibrio"
6762	caby	Caldithrix abyssi	"Prokaryotes;Bacteria;Calditrichaeota;Caldithrix"
6763	dth	Dictyoglomus thermophilum	"Prokaryotes;Bacteria;Dictyoglomi;Dictyoglomus"
6764	dtu	Dictyoglomus turgidum	"Prokaryotes;Bacteria;Dictyoglomi;Dictyoglomus"
6765	tye	Thermodesulfovibrio yellowstonii	"Prokaryotes;Bacteria;Nitrospirae;Thermodesulfovibrio"
6766	nde	Nitrospira defluvii	"Prokaryotes;Bacteria;Nitrospirae;Nitrospira"
6767	nmv	Nitrospira moscoviensis	"Prokaryotes;Bacteria;Nitrospirae;Nitrospira"
6768	nio	Candidatus Nitrospira inopinata	"Prokaryotes;Bacteria;Nitrospirae;Nitrospira"
6769	nja	Nitrospira japonica	"Prokaryotes;Bacteria;Nitrospirae;Nitrospira"
6770	lfc	Leptospirillum ferrooxidans	"Prokaryotes;Bacteria;Nitrospirae;Leptospirillum"
6771	lfi	Leptospirillum ferriphilum ML-04	"Prokaryotes;Bacteria;Nitrospirae;Leptospirillum"
6772	lfp	Leptospirillum ferriphilum YSK	"Prokaryotes;Bacteria;Nitrospirae;Leptospirillum"
6773	leg	Leptospirillum sp. Group II	"Prokaryotes;Bacteria;Nitrospirae;Leptospirillum"
6774	tid	Thermodesulfatator indicus	"Prokaryotes;Bacteria;Thermodesulfobacteria;Thermodesulfatator"
6775	top	Thermodesulfobacterium geofontis	"Prokaryotes;Bacteria;Thermodesulfobacteria;Thermodesulfobacterium"
6776	tcm	Thermodesulfobacterium commune	"Prokaryotes;Bacteria;Thermodesulfobacteria;Thermodesulfobacterium"
6777	thet	Thermodesulfobacterium sp. TA1	"Prokaryotes;Bacteria;Thermodesulfobacteria;Thermodesulfobacterium"
6778	cthi	Caldimicrobium thiodismutans	"Prokaryotes;Bacteria;Thermodesulfobacteria;Caldimicrobium"
6779	tav	Thermosulfuriphilus ammonigenes	"Prokaryotes;Bacteria;Thermodesulfobacteria;Thermosulfuriphilus"
6780	tmai	Thermosulfurimonas marina	"Prokaryotes;Bacteria;Thermodesulfobacteria;Thermosulfurimonas"
6781	nli	Candidatus Nitronauta litoralis	"Prokaryotes;Bacteria;Nitrospinia;Nitronauta"
6782	saal	Candidatus Saccharimonas aalborgensis	"Prokaryotes;Bacteria;Saccharibacteria;Saccharimonas"
6783	sbe	Candidatus Saccharibacteria bacterium RAAC3_TM7_1	"Prokaryotes;Bacteria;Saccharibacteria;Saccharimonas"
6784	sbag	Candidatus Saccharibacteria bacterium GW2011_GWC2_44_17	"Prokaryotes;Bacteria;Saccharibacteria;Saccharimonas"
6785	sox	Candidatus Nanosynbacter lyticus	"Prokaryotes;Bacteria;Saccharibacteria;Nanosynbacter"
6786	prf	Candidatus Peribacter riflensis	"Prokaryotes;Bacteria;Peregrinibacteria;Peribacter"
6787	bana	Candidatus Bipolaricaulis anaerobius	"Prokaryotes;Bacteria;Bipolaricaulota;Bipolaricaulis"
6788	bih	Candidatus Bipolaricaulis sibiricus	"Prokaryotes;Bacteria;Bipolaricaulota;Bipolaricaulis"
6789	srb	Candidate division SR1 bacterium RAAC1_SR1_1	"Prokaryotes;Bacteria;Absconditabacteria"
6790	srg	Candidate division SR1 bacterium Aalborg_AAW-1	"Prokaryotes;Bacteria;Absconditabacteria"
6791	mox	Candidatus Methylomirabilis oxyfera	"Prokaryotes;Bacteria;Other;Methylomirabilis"

6792	caqa	Candidatus Chazhemtobacterium aquaticus	"Prokaryotes;Bacteria;Other;Chazhemtobacterium"
6793	dpb	Candidatus Babela massiliensis	"Prokaryotes;Bacteria;Other;Babela"
6794	tmg	Candidate division TM6 bacterium GW2011_GWF2_28_16	"Prokaryotes;Bacteria;unclassified Bacteria"
6795	wwe	Candidate division WWE3 bacterium RAAC2_WWE3_1	"Prokaryotes;Bacteria;unclassified Bacteria"
6796	bgw	Candidate division Kazan bacterium GW2011_GWA1_50_15	"Prokaryotes;Bacteria;unclassified Bacteria"
6797	bbgw	Berkelbacteria bacterium GW2011_GWE1_39_12	"Prokaryotes;Bacteria;unclassified Bacteria"
6798	mib	Candidatus Beckwithbacteria bacterium GW2011_GWC1_49_16	"Prokaryotes;Bacteria;unclassified Bacteria"
6799	wba	Candidatus Woesebacteria bacterium GW2011_GWF1_31_35	"Prokaryotes;Bacteria;unclassified Bacteria"
6800	pwo	Candidatus Wolfebacteria bacterium GW2011_GWB1_47_1	"Prokaryotes;Bacteria;unclassified Bacteria"
6801	cgw	Candidatus Campbellbacteria bacterium GW2011_OD1_34_28	"Prokaryotes;Bacteria;unclassified Bacteria"
6802	baab	Bacterium AB1	"Prokaryotes;Bacteria;unclassified Bacteria"
6803	mja	Methanocaldococcus jannaschii	"Prokaryotes;Archaea;Euryarchaeota;Methanocaldococcus"
6804	mfe	Methanocaldococcus fervens	"Prokaryotes;Archaea;Euryarchaeota;Methanocaldococcus"
6805	mvu	Methanocaldococcus vulcanius	"Prokaryotes;Archaea;Euryarchaeota;Methanocaldococcus"
6806	mfs	Methanocaldococcus sp. FS406-22	"Prokaryotes;Archaea;Euryarchaeota;Methanocaldococcus"
6807	mif	Methanocaldococcus infernus	"Prokaryotes;Archaea;Euryarchaeota;Methanocaldococcus"
6808	mjh	Methanocaldococcus bathoardescens	"Prokaryotes;Archaea;Euryarchaeota;Methanocaldococcus"
6809	mig	Methanotorris igneus	"Prokaryotes;Archaea;Euryarchaeota;Methanotorris"
6810	mmp	Methanococcus maripaludis S2	"Prokaryotes;Archaea;Euryarchaeota;Methanococcus"
6811	mmq	Methanococcus maripaludis C5	"Prokaryotes;Archaea;Euryarchaeota;Methanococcus"
6812	mmx	Methanococcus maripaludis C6	"Prokaryotes;Archaea;Euryarchaeota;Methanococcus"
6813	mmz	Methanococcus maripaludis C7	"Prokaryotes;Archaea;Euryarchaeota;Methanococcus"
6814	mmd	Methanococcus maripaludis X1	"Prokaryotes;Archaea;Euryarchaeota;Methanococcus"
6815	mmak	Methanococcus maripaludis KA1	"Prokaryotes;Archaea;Euryarchaeota;Methanococcus"
6816	mmao	Methanococcus maripaludis OS7	"Prokaryotes;Archaea;Euryarchaeota;Methanococcus"
6817	mmad	Methanococcus maripaludis DSM 2067	"Prokaryotes;Archaea;Euryarchaeota;Methanococcus"
6818	mae	Methanococcus aeolicus	"Prokaryotes;Archaea;Euryarchaeota;Methanococcus"
6819	mvn	Methanococcus vannielii	"Prokaryotes;Archaea;Euryarchaeota;Methanococcus"
6820	mvo	Methanococcus voltae	"Prokaryotes;Archaea;Euryarchaeota;Methanococcus"
6821	mok	Methanothermococcus okinawensis	"Prokaryotes;Archaea;Euryarchaeota;Methanothermococcus"
6822	metf	Methanofervidicoccus sp. A16	"Prokaryotes;Archaea;Euryarchaeota;Methanofervidicoccus"
6823	mth	Methanothermobacter thermautotrophicus	"Prokaryotes;Archaea;Euryarchaeota;Methanothermobacter"
6824	mmg	Methanothermobacter marburgensis	"Prokaryotes;Archaea;Euryarchaeota;Methanothermobacter"
6825	metc	Methanothermobacter sp. CaT2	"Prokaryotes;Archaea;Euryarchaeota;Methanothermobacter"
6826	mwo	Methanothermobacter wolfeii	"Prokaryotes;Archaea;Euryarchaeota;Methanothermobacter"
6827	mete	Methanothermobacter sp. EMTCatA1	"Prokaryotes;Archaea;Euryarchaeota;Methanothermobacter"
6828	metz	Methanothermobacter sp. MT-2	"Prokaryotes;Archaea;Euryarchaeota;Methanothermobacter"
6829	metk	Methanothermobacter sp. KEPCO-1	"Prokaryotes;Archaea;Euryarchaeota;Methanothermobacter"
6830	mthm	Methanothermobacter sp. THM-1	"Prokaryotes;Archaea;Euryarchaeota;Methanothermobacter"
6831	mst	Methanosphaera stadtmanae	"Prokaryotes;Archaea;Euryarchaeota;Methanosphaera"
6832	metb	Methanosphaera sp. BMS	"Prokaryotes;Archaea;Euryarchaeota;Methanosphaera"

6833	mru	Methanobrevibacter ruminantium	"Prokaryotes;Archaea;Euryarchaeota;Methanobrevibacter"
6834	msi	Methanobrevibacter smithii	"Prokaryotes;Archaea;Euryarchaeota;Methanobrevibacter"
6835	meb	Methanobrevibacter sp. AbM4	"Prokaryotes;Archaea;Euryarchaeota;Methanobrevibacter"
6836	mmil	Methanobrevibacter millerae	"Prokaryotes;Archaea;Euryarchaeota;Methanobrevibacter"
6837	meye	Methanobrevibacter sp. YE315	"Prokaryotes;Archaea;Euryarchaeota;Methanobrevibacter"
6838	mol	Methanobrevibacter olleyae	"Prokaryotes;Archaea;Euryarchaeota;Methanobrevibacter"
6839	mel	Methanobacterium lacus	"Prokaryotes;Archaea;Euryarchaeota;Methanobacterium"
6840	mew	Methanobacterium paludis	"Prokaryotes;Archaea;Euryarchaeota;Methanobacterium"
6841	meth	Methanobacterium sp. MB1	"Prokaryotes;Archaea;Euryarchaeota;Methanobacterium"
6842	mfc	Methanobacterium formicicum BRM9	"Prokaryotes;Archaea;Euryarchaeota;Methanobacterium"
6843	mfi	Methanobacterium formicicum DSM1535	"Prokaryotes;Archaea;Euryarchaeota;Methanobacterium"
6844	mcub	Methanobacterium congolense	"Prokaryotes;Archaea;Euryarchaeota;Methanobacterium"
6845	msub	Methanobacterium subterraneum	"Prokaryotes;Archaea;Euryarchaeota;Methanobacterium"
6846	metn	Methanobacterium sp. MZ-A1	"Prokaryotes;Archaea;Euryarchaeota;Methanobacterium"
6847	mett	Methanobacterium sp. BRmetb2	"Prokaryotes;Archaea;Euryarchaeota;Methanobacterium"
6848	meto	Methanobacterium sp. BAmetb5	"Prokaryotes;Archaea;Euryarchaeota;Methanobacterium"
6849	mfv	Methanothermus fervidus	"Prokaryotes;Archaea;Euryarchaeota;Methanothermus"
6850	mka	Methanopyrus kandleri	"Prokaryotes;Archaea;Euryarchaeota;Methanopyrus"
6851	afu	Archaeoglobus fulgidus DSM 4304	"Prokaryotes;Archaea;Euryarchaeota;Archaeoglobus"
6852	afg	Archaeoglobus fulgidus DSM 8774	"Prokaryotes;Archaea;Euryarchaeota;Archaeoglobus"
6853	apo	Archaeoglobus profundus	"Prokaryotes;Archaea;Euryarchaeota;Archaeoglobus"
6854	ave	Archaeoglobus veneficus	"Prokaryotes;Archaea;Euryarchaeota;Archaeoglobus"
6855	ast	Archaeoglobus sulfaticallidus	"Prokaryotes;Archaea;Euryarchaeota;Archaeoglobus"
6856	fpl	Ferroglobus placidus	"Prokaryotes;Archaea;Euryarchaeota;Ferroglobus"
6857	gac	Geoglobus acetivorans	"Prokaryotes;Archaea;Euryarchaeota;Geoglobus"
6858	gah	Geoglobus ahangari	"Prokaryotes;Archaea;Euryarchaeota;Geoglobus"
6859	pfu	Pyrococcus furiosus DSM 3638	"Prokaryotes;Archaea;Euryarchaeota;Pyrococcus"
6860	pfi	Pyrococcus furiosus COM1	"Prokaryotes;Archaea;Euryarchaeota;Pyrococcus"
6861	pho	Pyrococcus horikoshii	"Prokaryotes;Archaea;Euryarchaeota;Pyrococcus"
6862	pab	Pyrococcus abyssi	"Prokaryotes;Archaea;Euryarchaeota;Pyrococcus"
6863	pyn	Pyrococcus sp. NA2	"Prokaryotes;Archaea;Euryarchaeota;Pyrococcus"
6864	pya	Pyrococcus yayanosii	"Prokaryotes;Archaea;Euryarchaeota;Pyrococcus"
6865	pys	Pyrococcus sp. ST04	"Prokaryotes;Archaea;Euryarchaeota;Pyrococcus"
6866	pyc	Pyrococcus kukulkanii	"Prokaryotes;Archaea;Euryarchaeota;Pyrococcus"
6867	tko	Thermococcus kodakarensis	"Prokaryotes;Archaea;Euryarchaeota;Thermococcus"
6868	ton	Thermococcus onnurineus	"Prokaryotes;Archaea;Euryarchaeota;Thermococcus"
6869	tga	Thermococcus gammatolerans	"Prokaryotes;Archaea;Euryarchaeota;Thermococcus"
6870	tsi	Thermococcus sibiricus	"Prokaryotes;Archaea;Euryarchaeota;Thermococcus"
6871	tba	Thermococcus barophilus	"Prokaryotes;Archaea;Euryarchaeota;Thermococcus"
6872	the	Thermococcus sp. 4557	"Prokaryotes;Archaea;Euryarchaeota;Thermococcus"
6873	tha	Thermococcus sp. AM4	"Prokaryotes;Archaea;Euryarchaeota;Thermococcus"

6874	thm	Thermococcus cleftensis	"Prokaryotes;Archaea;Euryarchaeota;Thermococcus"
6875	tlt	Thermococcus litoralis	"Prokaryotes;Archaea;Euryarchaeota;Thermococcus"
6876	ths	Thermococcus paralvinellae	"Prokaryotes;Archaea;Euryarchaeota;Thermococcus"
6877	tnu	Thermococcus nautili	"Prokaryotes;Archaea;Euryarchaeota;Thermococcus"
6878	teu	Thermococcus eurythermalis	"Prokaryotes;Archaea;Euryarchaeota;Thermococcus"
6879	tgy	Thermococcus guaymasensis	"Prokaryotes;Archaea;Euryarchaeota;Thermococcus"
6880	thv	Thermococcus sp. 2319x1	"Prokaryotes;Archaea;Euryarchaeota;Thermococcus"
6881	tch	Thermococcus chitonophagus	"Prokaryotes;Archaea;Euryarchaeota;Thermococcus"
6882	tpep	Thermococcus peptonophilus	"Prokaryotes;Archaea;Euryarchaeota;Thermococcus"
6883	tpie	Thermococcus piezophilus	"Prokaryotes;Archaea;Euryarchaeota;Thermococcus"
6884	tgg	Thermococcus gorgonarius	"Prokaryotes;Archaea;Euryarchaeota;Thermococcus"
6885	tce	Thermococcus celer	"Prokaryotes;Archaea;Euryarchaeota;Thermococcus"
6886	tbs	Thermococcus barossii	"Prokaryotes;Archaea;Euryarchaeota;Thermococcus"
6887	thh	Thermococcus sp. 5-4	"Prokaryotes;Archaea;Euryarchaeota;Thermococcus"
6888	tsl	Thermococcus siculi	"Prokaryotes;Archaea;Euryarchaeota;Thermococcus"
6889	ttd	Thermococcus thioreducens	"Prokaryotes;Archaea;Euryarchaeota;Thermococcus"
6890	tprf	Thermococcus profundus	"Prokaryotes;Archaea;Euryarchaeota;Thermococcus"
6891	trl	Thermococcus radiotolerans	"Prokaryotes;Archaea;Euryarchaeota;Thermococcus"
6892	tpaf	Thermococcus pacificus	"Prokaryotes;Archaea;Euryarchaeota;Thermococcus"
6893	thy	Thermococcus sp. P6	"Prokaryotes;Archaea;Euryarchaeota;Thermococcus"
6894	ppac	Palaeococcus pacificus	"Prokaryotes;Archaea;Euryarchaeota;Palaeococcus"
6895	mba	Methanosarcina barkeri Fusaro	"Prokaryotes;Archaea;Euryarchaeota;Methanosarcina"
6896	mby	Methanosarcina barkeri MS	"Prokaryotes;Archaea;Euryarchaeota;Methanosarcina"
6897	mbw	Methanosarcina barkeri Wiesmoor	"Prokaryotes;Archaea;Euryarchaeota;Methanosarcina"
6898	mbar	Methanosarcina barkeri 227	"Prokaryotes;Archaea;Euryarchaeota;Methanosarcina"
6899	mbak	Methanosarcina barkeri 3	"Prokaryotes;Archaea;Euryarchaeota;Methanosarcina"
6900	mac	Methanosarcina acetivorans	"Prokaryotes;Archaea;Euryarchaeota;Methanosarcina"
6901	mma	Methanosarcina mazei Go1	"Prokaryotes;Archaea;Euryarchaeota;Methanosarcina"
6902	mmaz	Methanosarcina mazei Tuc01	"Prokaryotes;Archaea;Euryarchaeota;Methanosarcina"
6903	mmj	Methanosarcina mazei S-6	"Prokaryotes;Archaea;Euryarchaeota;Methanosarcina"
6904	mmac	Methanosarcina mazei C16	"Prokaryotes;Archaea;Euryarchaeota;Methanosarcina"
6905	mvc	Methanosarcina vacuolata	"Prokaryotes;Archaea;Euryarchaeota;Methanosarcina"
6906	mek	Methanosarcina sp. Kolksee	"Prokaryotes;Archaea;Euryarchaeota;Methanosarcina"
6907	mls	Methanosarcina lacustris	"Prokaryotes;Archaea;Euryarchaeota;Methanosarcina"
6908	metm	Methanosarcina sp. MTP4	"Prokaryotes;Archaea;Euryarchaeota;Methanosarcina"
6909	mef	Methanosarcina sp. WH1	"Prokaryotes;Archaea;Euryarchaeota;Methanosarcina"
6910	meq	Methanosarcina sp. WWM596	"Prokaryotes;Archaea;Euryarchaeota;Methanosarcina"
6911	msj	Methanosarcina siciliae C2J	"Prokaryotes;Archaea;Euryarchaeota;Methanosarcina"
6912	msz	Methanosarcina siciliae HI350	"Prokaryotes;Archaea;Euryarchaeota;Methanosarcina"
6913	msw	Methanosarcina siciliae T4/M	"Prokaryotes;Archaea;Euryarchaeota;Methanosarcina"
6914	mthr	Methanosarcina thermophila TM-1	"Prokaryotes;Archaea;Euryarchaeota;Methanosarcina"

6915	mthe	Methanosarcina thermophila CHTI-55	"Prokaryotes;Archaea;Euryarchaeota;Methanosarcina"
6916	mhor	Methanosarcina horonobensis	"Prokaryotes;Archaea;Euryarchaeota;Methanosarcina"
6917	mfz	Methanosarcina flavescens	"Prokaryotes;Archaea;Euryarchaeota;Methanosarcina"
6918	mbu	Methanococcoides burtonii	"Prokaryotes;Archaea;Euryarchaeota;Methanococcoides"
6919	mmet	Methanococcoides methylutens	"Prokaryotes;Archaea;Euryarchaeota;Methanococcoides"
6920	mmh	Methanohalophilus mahii	"Prokaryotes;Archaea;Euryarchaeota;Methanohalophilus"
6921	mhaz	Methanohalophilus halophilus	"Prokaryotes;Archaea;Euryarchaeota;Methanohalophilus"
6922	mev	Methanohalobium evestigatum	"Prokaryotes;Archaea;Euryarchaeota;Methanohalobium"
6923	mzh	Methanosalsum zhilinae	"Prokaryotes;Archaea;Euryarchaeota;Methanosalsum"
6924	mpy	Methanolobus psychrophilus	"Prokaryotes;Archaea;Euryarchaeota;Methanolobus"
6925	mzi	Methanolobus zinderi	"Prokaryotes;Archaea;Euryarchaeota;Methanolobus"
6926	mhz	Methanomethylovorans hollandica	"Prokaryotes;Archaea;Euryarchaeota;Methanomethylovorans"
6927	mtp	Methanothrix thermoacetophila	"Prokaryotes;Archaea;Euryarchaeota;Methanosaeta"
6928	mcj	Methanothrix soehngenii	"Prokaryotes;Archaea;Euryarchaeota;Methanosaeta"
6929	mhi	Methanosaeta harundinacea	"Prokaryotes;Archaea;Euryarchaeota;Methanosaeta"
6930	mhu	Methanospirillum hungatei	"Prokaryotes;Archaea;Euryarchaeota;Methanospirillum"
6931	mla	Methanocorpusculum labreanum	"Prokaryotes;Archaea;Euryarchaeota;Methanocorpusculum"
6932	mem	Methanoculleus marisnigri	"Prokaryotes;Archaea;Euryarchaeota;Methanoculleus"
6933	mbg	Methanoculleus bourgensis MS2	"Prokaryotes;Archaea;Euryarchaeota;Methanoculleus"
6934	mema	Methanoculleus bourgensis MAB1	"Prokaryotes;Archaea;Euryarchaeota;Methanoculleus"
6935	mpi	Methanolacinia petrolearia	"Prokaryotes;Archaea;Euryarchaeota;Methanolacinia"
6936	mbn	Methanoregula boonei	"Prokaryotes;Archaea;Euryarchaeota;Methanoregula"
6937	mfo	Methanoregula formicica	"Prokaryotes;Archaea;Euryarchaeota;Methanoregula"
6938	mpl	Methanosphaerula palustris	"Prokaryotes;Archaea;Euryarchaeota;Methanosphaerula"
6939	mpd	Methanocella paludicola	"Prokaryotes;Archaea;Euryarchaeota;Methanocella"
6940	mez	Methanocella conradii	"Prokaryotes;Archaea;Euryarchaeota;Methanocella"
6941	rci	Methanocella arvoryzae	"Prokaryotes;Archaea;Euryarchaeota;Methanocella"
6942	hal	Halobacterium salinarum NRC-1	"Prokaryotes;Archaea;Euryarchaeota;Halobacterium"
6943	hsl	Halobacterium salinarum	"Prokaryotes;Archaea;Euryarchaeota;Halobacterium"
6944	hdl	Halobacterium sp. DL1	"Prokaryotes;Archaea;Euryarchaeota;Halobacterium"
6945	hhb	Halobacterium hubeiense	"Prokaryotes;Archaea;Euryarchaeota;Halobacterium"
6946	hje	Halalkalicoccus jeotgali	"Prokaryotes;Archaea;Euryarchaeota;Halalkalicoccus"
6947	halh	Halodesulfurarchaeum formicicum HTSR1	"Prokaryotes;Archaea;Euryarchaeota;Halodesulfurarchaeum"
6948	hhsr	Haloarchaeon HSR6	"Prokaryotes;Archaea;Euryarchaeota;Halodesulfurarchaeum"
6949	hsu	Halanaeroarchaeum sulfurireducens HSR2	"Prokaryotes;Archaea;Euryarchaeota;Halanaeroarchaeum"
6950	hsf	Halanaeroarchaeum sulfurireducens M27-SA2	"Prokaryotes;Archaea;Euryarchaeota;Halanaeroarchaeum"
6951	salr	Salarchaeum sp. JOR-1	"Prokaryotes;Archaea;Euryarchaeota;Salarchaeum"
6952	halr	Halarchaeum sp. CBA1220	"Prokaryotes;Archaea;Euryarchaeota;Halarchaeum"
6953	hma	Haloarcula marismortui	"Prokaryotes;Archaea;Euryarchaeota;Haloarcula"
6954	hhi	Haloarcula hispanica ATCC 33960	"Prokaryotes;Archaea;Euryarchaeota;Haloarcula"
6955	hhn	Haloarcula hispanica N601	"Prokaryotes;Archaea;Euryarchaeota;Haloarcula"

6956	hab	Haloarcula sp. CBA1115	"Prokaryotes;Archaea;Euryarchaeota;Haloarcula"
6957	hta	Haloarcula taiwanensis	"Prokaryotes;Archaea;Euryarchaeota;Haloarcula"
6958	halj	Haloarcula sp. JP-L23	"Prokaryotes;Archaea;Euryarchaeota;Haloarcula"
6959	nph	Natronomonas pharaonis	"Prokaryotes;Archaea;Euryarchaeota;Natronomonas"
6960	nmo	Natronomonas moolapensis	"Prokaryotes;Archaea;Euryarchaeota;Natronomonas"
6961	hut	Halorhabdus utahensis	"Prokaryotes;Archaea;Euryarchaeota;Halorhabdus"
6962	hti	Halorhabdus tiamatea	"Prokaryotes;Archaea;Euryarchaeota;Halorhabdus"
6963	hala	Halorhabdus sp. CBA1104	"Prokaryotes;Archaea;Euryarchaeota;Halorhabdus"
6964	hmu	Halomicrobium mukohataei	"Prokaryotes;Archaea;Euryarchaeota;Halomicrobium"
6965	halz	Halomicrobium mukohataei JP60	"Prokaryotes;Archaea;Euryarchaeota;Halomicrobium"
6966	hall	Halomicrobium sp. LC1Hm	"Prokaryotes;Archaea;Euryarchaeota;Halomicrobium"
6967	hali	Halorientalis sp. IM1011	"Prokaryotes;Archaea;Euryarchaeota;Halorientalis"
6968	hsn	Halapricum salinum	"Prokaryotes;Archaea;Euryarchaeota;Halapricum"
6969	hrr	Halosimplex rubrum	"Prokaryotes;Archaea;Euryarchaeota;Halosimplex"
6970	hpel	Halosimplex pelagicum	"Prokaryotes;Archaea;Euryarchaeota;Halosimplex"
6971	hlt	Halosimplex litoreum	"Prokaryotes;Archaea;Euryarchaeota;Halosimplex"
6972	harc	Haloarculaceae archaeon HArcel1	"Prokaryotes;Archaea;Euryarchaeota;unclassified Haloarculaceae"
6973	hwa	Haloquadratum walsbyi DSM 16790	"Prokaryotes;Archaea;Euryarchaeota;Haloquadratum"
6974	hwc	Haloquadratum walsbyi C23	"Prokaryotes;Archaea;Euryarchaeota;Haloquadratum"
6975	hvo	Haloferax volcanii	"Prokaryotes;Archaea;Euryarchaeota;Haloferax"
6976	hme	Haloferax mediterranei	"Prokaryotes;Archaea;Euryarchaeota;Haloferax"
6977	hgi	Haloferax gibbonsii	"Prokaryotes;Archaea;Euryarchaeota;Haloferax"
6978	hale	Haloferax alexandrinus	"Prokaryotes;Archaea;Euryarchaeota;Haloferax"
6979	hbo	Halogeometricum borinquense	"Prokaryotes;Archaea;Euryarchaeota;Halogeometricum"
6980	haq	Haloplanus rubicundus CBA1112	"Prokaryotes;Archaea;Euryarchaeota;Haloplanus"
6981	haj	Haloplanus rubicundus CBA1113	"Prokaryotes;Archaea;Euryarchaeota;Haloplanus"
6982	haer	Haloplanus aerogenes	"Prokaryotes;Archaea;Euryarchaeota;Haloplanus"
6983	hra	Haloplanus rallus	"Prokaryotes;Archaea;Euryarchaeota;Haloplanus"
6984	hlm	Halobellus limi	"Prokaryotes;Archaea;Euryarchaeota;Halobellus"
6985	halm	Haloprofundus sp. MHR1	"Prokaryotes;Archaea;Euryarchaeota;Haloprofundus"
6986	hla	Halorubrum lacusprofundi	"Prokaryotes;Archaea;Euryarchaeota;Halorubrum"
6987	halp	Halorubrum sp. PV6	"Prokaryotes;Archaea;Euryarchaeota;Halorubrum"
6988	halb	Halorubrum sp. BOL3-1	"Prokaryotes;Archaea;Euryarchaeota;Halorubrum"
6989	hezz	Halorubrum ezzemoulense	"Prokaryotes;Archaea;Euryarchaeota;Halorubrum"
6990	halq	Halorubrum sp. CBA1229	"Prokaryotes;Archaea;Euryarchaeota;Halorubrum"
6991	srub	Salinigranum rubrum	"Prokaryotes;Archaea;Euryarchaeota;Salinigranum"
6992	hae	Halohasta litchfieldiae	"Prokaryotes;Archaea;Euryarchaeota;Halohasta"
6993	haln	Halolamina sp. CBA1230	"Prokaryotes;Archaea;Euryarchaeota;Halolamina"
6994	halg	Halobonum sp. Gai3-2	"Prokaryotes;Archaea;Euryarchaeota;Halobonum"
6995	halu	Halobonum sp. NJ-3-1	"Prokaryotes;Archaea;Euryarchaeota;Halobonum"
6996	hdf	Halalkaliarchaeum desulfuricum	"Prokaryotes;Archaea;Euryarchaeota;Halalkaliarchaeum"

6997	hah	Halophilic archaeon DL31	"Prokaryotes;Archaea;Euryarchaeota;unclassified Haloferacales"
6998	htu	Haloterrigena turkmenica	"Prokaryotes;Archaea;Euryarchaeota;Haloterrigena"
6999	hda	Haloterrigena daqingensis	"Prokaryotes;Archaea;Euryarchaeota;Haloterrigena"
7000	hjt	Haloterrigena jeotgali	"Prokaryotes;Archaea;Euryarchaeota;Haloterrigena"
7001	haly	Haloterrigena sp. YPL8	"Prokaryotes;Archaea;Euryarchaeota;Haloterrigena"
7002	nmg	Natrialba magadii	"Prokaryotes;Archaea;Euryarchaeota;Natrialba"
7003	hxa	Halopiger xanaduensis	"Prokaryotes;Archaea;Euryarchaeota;Halopiger"
7004	nat	Natrinema sp. J7-2	"Prokaryotes;Archaea;Euryarchaeota;Natrinema"
7005	npe	Natrinema pellirubrum	"Prokaryotes;Archaea;Euryarchaeota;Natrinema"
7006	nvr	Natrinema versiforme	"Prokaryotes;Archaea;Euryarchaeota;Natrinema"
7007	npl	Natrinema pallidum	"Prokaryotes;Archaea;Euryarchaeota;Natrinema"
7008	nge	Natronobacterium gregoryi	"Prokaryotes;Archaea;Euryarchaeota;Natronobacterium"
7009	hru	Halovivax ruber	"Prokaryotes;Archaea;Euryarchaeota;Halovivax"
7010	nou	Natronococcus occultus	"Prokaryotes;Archaea;Euryarchaeota;Natronococcus"
7011	sali	Salinarchaeum sp. Harcht-Bsk1	"Prokaryotes;Archaea;Euryarchaeota;Salinarchaeum"
7012	hlr	Halostagnicola larsenii	"Prokaryotes;Archaea;Euryarchaeota;Halostagnicola"
7013	hlc	Halobiforma lacisalsi	"Prokaryotes;Archaea;Euryarchaeota;Halobiforma"
7014	naj	Natrarchaeobaculum aegyptiacum	"Prokaryotes;Archaea;Euryarchaeota;Natrarchaeobaculum"
7015	nag	Natrarchaeobaculum sulfurireducens AArc-Mg	"Prokaryotes;Archaea;Euryarchaeota;Natrarchaeobaculum"
7016	nan	Natrarchaeobaculum sulfurireducens AArc1	"Prokaryotes;Archaea;Euryarchaeota;Natrarchaeobaculum"
7017	nbg	Natronorubrum bangense	"Prokaryotes;Archaea;Euryarchaeota;Natronorubrum"
7018	nas	Natronorubrum aibiense	"Prokaryotes;Archaea;Euryarchaeota;Natronorubrum"
7019	nax	Salinadaptatus halalkaliphilus	"Prokaryotes;Archaea;Euryarchaeota;Salinadaptatus"
7020	nac	Nanohaloarchaea archaeon SG9	"Prokaryotes;Archaea;Euryarchaeota;unclassified Nanohaloarchaea"
7021	tac	Thermoplasma acidophilum	"Prokaryotes;Archaea;Candidatus Thermoplasmatota;Thermoplasma"
7022	tvo	Thermoplasma volcanium	"Prokaryotes;Archaea;Candidatus Thermoplasmatota;Thermoplasma"
7023	pto	Picrophilus torridus	"Prokaryotes;Archaea;Candidatus Thermoplasmatota;Picrophilus"
7024	fac	Ferroplasma acidarmanus	"Prokaryotes;Archaea;Candidatus Thermoplasmatota;Ferroplasma"
7025	fai	Ferroplasma acidiphilum	"Prokaryotes;Archaea;Candidatus Thermoplasmatota;Ferroplasma"
7026	cdiv	Cuniculiplasma divulgatum	"Prokaryotes;Archaea;Candidatus Thermoplasmatota;Cuniculiplasma"
7027	tar	Thermoplasmatales archaeon BRNA1	"Prokaryotes;Archaea;Candidatus Thermoplasmatota;unclassified Thermoplasmatales"
7028	max	Candidatus Methanomethylophilus alvus	"Prokaryotes;Archaea;Candidatus Thermoplasmatota;Methanomethylophilus"
7029	mer	Candidatus Methanomassiliicoccus intestinalis Issoire-Mx1	"Prokaryotes;Archaea;Candidatus Thermoplasmatota;Methanomassiliicoccus"
7030	mear	Candidatus Methanoplasma termitum	"Prokaryotes;Archaea;Candidatus Thermoplasmatota;Methanoplasma"
7031	marc	Methanogenic archaeon ISO4-H5	"Prokaryotes;Archaea;Candidatus Thermoplasmatota;unclassified Thermoplasmata"
7032	abi	Aciduliprofundum boonei	"Prokaryotes;Archaea;Candidatus Thermoplasmatota;Aciduliprofundum"
7033	acf	Aciduliprofundum sp. MAR08-339	"Prokaryotes;Archaea;Candidatus Thermoplasmatota;Aciduliprofundum"
7034	ape	Aeropyrum pernix	"Prokaryotes;Archaea;Crenarchaeota;Aeropyrum"
7035	acj	Aeropyrum camini	"Prokaryotes;Archaea;Crenarchaeota;Aeropyrum"
7036	smr	Staphylothermus marinus	"Prokaryotes;Archaea;Crenarchaeota;Staphylothermus"
7037	shc	Staphylothermus hellenicus	"Prokaryotes;Archaea;Crenarchaeota;Staphylothermus"

7038	iho	Ignicoccus hospitalis	"Prokaryotes;Archaea;Crenarchaeota;Ignicoccus"
7039	iis	Ignicoccus islandicus	"Prokaryotes;Archaea;Crenarchaeota;Ignicoccus"
7040	dka	Desulfurococcus amylolyticus 1221n	"Prokaryotes;Archaea;Crenarchaeota;Desulfurococcus"
7041	dfd	Desulfurococcus amylolyticus DSM 16532	"Prokaryotes;Archaea;Crenarchaeota;Desulfurococcus"
7042	dmu	Desulfurococcus mucosus	"Prokaryotes;Archaea;Crenarchaeota;Desulfurococcus"
7043	tag	Thermosphaera aggregans	"Prokaryotes;Archaea;Crenarchaeota;Thermosphaera"
7044	iag	Ignisphaera aggregans	"Prokaryotes;Archaea;Crenarchaeota;Ignisphaera"
7045	thg	Thermogladius calderae	"Prokaryotes;Archaea;Crenarchaeota;Thermogladius"
7046	hbu	Hyperthermus butylicus	"Prokaryotes;Archaea;Crenarchaeota;Hyperthermus"
7047	pfm	Pyrolobus fumarii	"Prokaryotes;Archaea;Crenarchaeota;Pyrolobus"
7048	pdl	Pyrodictium delaneyi	"Prokaryotes;Archaea;Crenarchaeota;Pyrodictium"
7049	sto	Sulfurisphaera tokodaii	"Prokaryotes;Archaea;Crenarchaeota;Sulfurisphaera"
7050	soh	Sulfurisphaera ohwakuensis	"Prokaryotes;Archaea;Crenarchaeota;Sulfurisphaera"
7051	sso	Saccharolobus solfataricus P2	"Prokaryotes;Archaea;Crenarchaeota;Saccharolobus"
7052	sol	Saccharolobus solfataricus 98/2	"Prokaryotes;Archaea;Crenarchaeota;Saccharolobus"
7053	ssoa	Saccharolobus solfataricus SULA	"Prokaryotes;Archaea;Crenarchaeota;Saccharolobus"
7054	ssol	Saccharolobus solfataricus SARC-B	"Prokaryotes;Archaea;Crenarchaeota;Saccharolobus"
7055	ssof	Saccharolobus solfataricus SARC-C	"Prokaryotes;Archaea;Crenarchaeota;Saccharolobus"
7056	sai	Sulfolobus acidocaldarius DSM 639	"Prokaryotes;Archaea;Crenarchaeota;Sulfolobus"
7057	sacn	Sulfolobus acidocaldarius N8	"Prokaryotes;Archaea;Crenarchaeota;Sulfolobus"
7058	sacr	Sulfolobus acidocaldarius Ron12/I	"Prokaryotes;Archaea;Crenarchaeota;Sulfolobus"
7059	sacs	Sulfolobus acidocaldarius SUSAZ	"Prokaryotes;Archaea;Crenarchaeota;Sulfolobus"
7060	sis	Sulfolobus islandicus L.S.2.15	"Prokaryotes;Archaea;Crenarchaeota;Sulfolobus"
7061	sia	Sulfolobus islandicus M.14.25	"Prokaryotes;Archaea;Crenarchaeota;Sulfolobus"
7062	sim	Sulfolobus islandicus M.16.27	"Prokaryotes;Archaea;Crenarchaeota;Sulfolobus"
7063	sid	Sulfolobus islandicus M.16.4	"Prokaryotes;Archaea;Crenarchaeota;Sulfolobus"
7064	siy	Sulfolobus islandicus Y.G.57.14	"Prokaryotes;Archaea;Crenarchaeota;Sulfolobus"
7065	sin	Sulfolobus islandicus Y.N.15.51	"Prokaryotes;Archaea;Crenarchaeota;Sulfolobus"
7066	sii	Sulfolobus islandicus L.D.8.5	"Prokaryotes;Archaea;Crenarchaeota;Sulfolobus"
7067	sih	Sulfolobus islandicus HVE10/4	"Prokaryotes;Archaea;Crenarchaeota;Sulfolobus"
7068	sir	Sulfolobus islandicus REY15A	"Prokaryotes;Archaea;Crenarchaeota;Sulfolobus"
7069	sic	Sulfolobus islandicus LAL14/1	"Prokaryotes;Archaea;Crenarchaeota;Sulfolobus"
7070	sula	Sulfolobus sp. A20	"Prokaryotes;Archaea;Crenarchaeota;Sulfolobus"
7071	sule	Sulfolobus sp. E5-1-F	"Prokaryotes;Archaea;Crenarchaeota;Sulfolobus"
7072	mse	Metallosphaera sedula	"Prokaryotes;Archaea;Crenarchaeota;Metallosphaera"
7073	mcn	Metallosphaera cuprina	"Prokaryotes;Archaea;Crenarchaeota;Metallosphaera"
7074	mhk	Metallosphaera hakonensis JCM 8857 = DSM 7519	"Prokaryotes;Archaea;Crenarchaeota;Metallosphaera"
7075	mpru	Metallosphaera prunae	"Prokaryotes;Archaea;Crenarchaeota;Metallosphaera"
7076	mten	Metallosphaera tengchongensis	"Prokaryotes;Archaea;Crenarchaeota;Metallosphaera"
7077	aho	Acidianus hospitalis	"Prokaryotes;Archaea;Crenarchaeota;Acidianus"
7078	aman	Acidianus manzaensis	"Prokaryotes;Archaea;Crenarchaeota;Acidianus"

7079	abri	Acidianus brierleyi	"Prokaryotes;Archaea;Crenarchaeota;Acidianus"
7080	asul	Acidianus sulfidivorans	"Prokaryotes;Archaea;Crenarchaeota;Acidianus"
7081	aamb	Acidianus ambivalens	"Prokaryotes;Archaea;Crenarchaeota;Acidianus"
7082	sacd	Sulfodiicoccus acidiphilus	"Prokaryotes;Archaea;Crenarchaeota;Sulfodiicoccus"
7083	sazo	Stygiolobus azoricus	"Prokaryotes;Archaea;Crenarchaeota;Stygiolobus"
7084	step	Sulfuracidifex tepidarius	"Prokaryotes;Archaea;Crenarchaeota;Sulfuracidifex"
7085	pai	Pyrobaculum aerophilum	"Prokaryotes;Archaea;Crenarchaeota;Pyrobaculum"
7086	pis	Pyrobaculum islandicum	"Prokaryotes;Archaea;Crenarchaeota;Pyrobaculum"
7087	pcl	Pyrobaculum calidifontis	"Prokaryotes;Archaea;Crenarchaeota;Pyrobaculum"
7088	pas	Pyrobaculum arsenaticum	"Prokaryotes;Archaea;Crenarchaeota;Pyrobaculum"
7089	pyr	Pyrobaculum ferrireducens	"Prokaryotes;Archaea;Crenarchaeota;Pyrobaculum"
7090	pog	Pyrobaculum oguniense	"Prokaryotes;Archaea;Crenarchaeota;Pyrobaculum"
7091	tne	Pyrobaculum neutrophilum	"Prokaryotes;Archaea;Crenarchaeota;Pyrobaculum"
7092	pyw	Pyrobaculum sp. WP30	"Prokaryotes;Archaea;Crenarchaeota;Pyrobaculum"
7093	cma	Caldivirga maquilingensis	"Prokaryotes;Archaea;Crenarchaeota;Caldivirga"
7094	ttn	Thermoproteus tenax	"Prokaryotes;Archaea;Crenarchaeota;Thermoproteus"
7095	tuz	Thermoproteus uzoniensis	"Prokaryotes;Archaea;Crenarchaeota;Thermoproteus"
7096	vdi	Vulcanisaeta distributa	"Prokaryotes;Archaea;Crenarchaeota;Vulcanisaeta"
7097	vmo	Vulcanisaeta moutnovskia	"Prokaryotes;Archaea;Crenarchaeota;Vulcanisaeta"
7098	tpe	Thermofilum pendens	"Prokaryotes;Archaea;Crenarchaeota;Thermofilum"
7099	thb	Thermofilum adornatum	"Prokaryotes;Archaea;Crenarchaeota;Thermofilum"
7100	tcb	Thermofilum adornatus 1505	"Prokaryotes;Archaea;Crenarchaeota;Thermofilum"
7101	thf	Thermofilum uzonense	"Prokaryotes;Archaea;Crenarchaeota;Thermofilum"
7102	thel	Thermofilum sp. 3507LT	"Prokaryotes;Archaea;Crenarchaeota;Thermofilum"
7103	asc	Acidilobus saccharovorans	"Prokaryotes;Archaea;Crenarchaeota;Acidilobus"
7104	acia	Acidilobus sp. 7A	"Prokaryotes;Archaea;Crenarchaeota;Acidilobus"
7105	clg	Caldisphaera lagunensis	"Prokaryotes;Archaea;Crenarchaeota;Caldisphaera"
7106	ffo	Fervidicoccus fontis	"Prokaryotes;Archaea;Crenarchaeota;Fervidicoccus"
7107	nmr	Nitrosopumilus maritimus	"Prokaryotes;Archaea;Thaumarchaeota;Nitrosopumilus"
7108	nir	Candidatus Nitrosopumilus sediminis	"Prokaryotes;Archaea;Thaumarchaeota;Nitrosopumilus"
7109	nkr	Candidatus Nitrosopumilus koreensis	"Prokaryotes;Archaea;Thaumarchaeota;Nitrosopumilus"
7110	nid	Nitrosopumilus piranensis	"Prokaryotes;Archaea;Thaumarchaeota;Nitrosopumilus"
7111	nin	Nitrosopumilus adriaticus	"Prokaryotes;Archaea;Thaumarchaeota;Nitrosopumilus"
7112	niw	Candidatus Nitrosopumilus sp. SW	"Prokaryotes;Archaea;Thaumarchaeota;Nitrosopumilus"
7113	ncl	Nitrosopumilus cobalaminigenes	"Prokaryotes;Archaea;Thaumarchaeota;Nitrosopumilus"
7114	nox	Nitrosopumilus oxyclinae	"Prokaryotes;Archaea;Thaumarchaeota;Nitrosopumilus"
7115	nue	Nitrosopumilus ureiphilus	"Prokaryotes;Archaea;Thaumarchaeota;Nitrosopumilus"
7116	nct	Candidatus Nitrosomarinus catalina	"Prokaryotes;Archaea;Thaumarchaeota;Nitrosomarinus"
7117	nic	Nitrosarchaeum sp. AC2	"Prokaryotes;Archaea;Thaumarchaeota;Nitrosarchaeum"
7118	csy	Cenarchaeum symbiosum	"Prokaryotes;Archaea;Thaumarchaeota;Cenarchaeum"
7119	nga	Candidatus Nitrososphaera gargensis	"Prokaryotes;Archaea;Thaumarchaeota;Nitrososphaera"

7120	nvn	Nitrososphaera viennensis	"Prokaryotes;Archaea;Thaumarchaeota;Nitrososphaera"
7121	nev	Candidatus Nitrososphaera evergladensis	"Prokaryotes;Archaea;Thaumarchaeota;Nitrososphaera"
7122	taa	Candidatus Nitrosocosmicus oleophilus	"Prokaryotes;Archaea;Thaumarchaeota;Nitrosocosmicus"
7123	nfn	Candidatus Nitrosocosmicus franklandus	"Prokaryotes;Archaea;Thaumarchaeota;Nitrosocosmicus"
7124	ncv	Candidatus Nitrosocaldus cavascurensis	"Prokaryotes;Archaea;Thaumarchaeota;Nitrosocaldus"
7125	csu	Candidatus Caldiarchaeum subterraneum	"Prokaryotes;Archaea;Thaumarchaeota;Caldiarchaeum"
7126	nbv	Candidatus Nitrosopelagicus brevis	"Prokaryotes;Archaea;Thaumarchaeota;Nitrosopelagicus"
7127	tah	Candidatus Nitrosotenuis cloacae	"Prokaryotes;Archaea;Thaumarchaeota;Nitrosotenuis"
7128	ndv	Candidatus Nitrosotalea devanaterra	"Prokaryotes;Archaea;Thaumarchaeota;Nitrosotalea"
7129	ccai	Conexivisphaera calida	"Prokaryotes;Archaea;Geothermarchaeota;Conexivisphaera"
7130	kcr	Candidatus Korarchaeum cryptofilum	"Prokaryotes;Archaea;Korarchaeota;Korarchaeum"
7131	barc	Candidatus Bathyarchaeota archaeon BA1	"Prokaryotes;Archaea;Bathyarchaeota;unclassified Bathyarchaeota"
7132	barb	Candidatus Bathyarchaeota archaeon BA2	"Prokaryotes;Archaea;Bathyarchaeota;unclassified Bathyarchaeota"
7133	neq	Nanoarchaeum equitans	"Prokaryotes;Archaea;Nanoarchaeota;Nanoarchaeum"
7134	naa	Candidatus Nanopusillus acidilobi	"Prokaryotes;Archaea;Nanoarchaeota;Nanopusillus"
7135	marh	Candidatus Micrarchaeota archaeon Mia14	"Prokaryotes;Archaea;Micrarchaeota;Mancarchaeum"
7136	flt	Candidatus Fermentimicrarchaeum limneticum	"Prokaryotes;Archaea;Micrarchaeota;Fermentimicrarchaeum"
7137	miy	Candidatus Micrarchaeum sp. A_DKE	"Prokaryotes;Archaea;Micrarchaeota;Micrarchaeum"
7138	loki	Lokiarchaeum sp. GC14_75	"Prokaryotes;Archaea;Lokiarchaeota;Lokiarchaeum"
7139	psyt	Candidatus Prometheoarchaeum syntrophicum	"Prokaryotes;Archaea;Lokiarchaeota;Prometheoarchaeum"
7140	agw	Archaeon GW2011_AR10	"Prokaryotes;Archaea;unclassified Archaea"
7141	arg	Archaeon GW2011_AR20	"Prokaryotes;Archaea;unclassified Archaea"
