# Supplementary material for: Exploring the expressiveness of abstract metabolic networks
Source: PLoS One. 2023 Feb 9;18(2):e0281047. doi: 10.1371/journal.pone.0281047 (PMC9910719; doi:10.1371/journal.pone.0281047)
Supplement: S2 File — List of the KEGG metabolic categories and corresponding pathways. (PDF) [file pone.0281047.s002.pdf]

List of the KEGG metabolic categories and corresponding pathways

Carbohydrate metabolism:
00010 Glycolysis / Gluconeogenesis
00020 Citrate cycle (TCA cycle)
00030 Pentose phosphate pathway
00040 Pentose and glucuronate interconversions
00051 Fructose and mannose metabolism
00052 Galactose metabolism
00053 Ascorbate and aldarate metabolism
00500 Starch and sucrose metabolism
00520 Amino sugar and nucleotide sugar metabolism
00620 Pyruvate metabolism
00630 Glyoxylate and dicarboxylate metabolism
00640 Propanoate metabolism
00650 Butanoate metabolism
00660 C5-Branched dibasic acid metabolism
00562 Inositol phosphate metabolism

Energy metabolism:
00190 Oxidative phosphorylation
00195 Photosynthesis
00196 Photosynthesis - antenna proteins
00710 Carbon fixation in photosynthetic organisms
00720 Carbon fixation pathways in prokaryotes
00680 Methane metabolism
00910 Nitrogen metabolism
00920 Sulfur metabolism

Lipid metabolism:
00061 Fatty acid biosynthesis
00062 Fatty acid elongation
00071 Fatty acid degradation
00073 Cutin, suberine and wax biosynthesis
00100 Steroid biosynthesis
00120 Primary bile acid biosynthesis
00121 Secondary bile acid biosynthesis
00140 Steroid hormone biosynthesis
00561 Glycerolipid metabolism
00564 Glycerophospholipid metabolism
00565 Ether lipid metabolism
00600 Sphingolipid metabolism
00590 Arachidonic acid metabolism
00591 Linoleic acid metabolism
00592 alpha-Linolenic acid metabolism
01040 Biosynthesis of unsaturated fatty acids

Nucleotide metabolism:
00230 Purine metabolism
00240 Pyrimidine metabolism

Amino acid metabolism:
00250 Alanine, aspartate and glutamate metabolism
00260 Glycine, serine and threonine metabolism
00270 Cysteine and methionine metabolism
00280 Valine, leucine and isoleucine degradation

00290 Valine, leucine and isoleucine biosynthesis
00300 Lysine biosynthesis
00310 Lysine degradation
00220 Arginine biosynthesis
00330 Arginine and proline metabolism
00340 Histidine metabolism
00350 Tyrosine metabolism
00360 Phenylalanine metabolism
00380 Tryptophan metabolism
00400 Phenylalanine, tyrosine and tryptophan biosynthesis

Metabolism of other amino acids:
00410 beta-Alanine metabolism
00430 Taurine and hypotaurine metabolism
00440 Phosphonate and phosphinate metabolism
00450 Selenocompound metabolism
00460 Cyanoamino acid metabolism
00470 D-Amino acid metabolism
00480 Glutathione metabolism

Glycan biosynthesis and metabolism:
00510 N-Glycan biosynthesis
00513 Various types of N-glycan biosynthesis
00512 Mucin type O-glycan biosynthesis
00515 Mannose type O-glycan biosynthesis
00514 Other types of O-glycan biosynthesis
00532 Glycosaminoglycan biosynthesis - chondroitin sulfate / dermatan sulfate
00534 Glycosaminoglycan biosynthesis - heparan sulfate / heparin
00533 Glycosaminoglycan biosynthesis - keratan sulfate
00531 Glycosaminoglycan degradation
00563 Glycosylphosphatidylinositol (GPI)-anchor biosynthesis
00601 Glycosphingolipid biosynthesis - lacto and neolacto series
00603 Glycosphingolipid biosynthesis - globo and isoglobo series
00604 Glycosphingolipid biosynthesis - ganglio series
00540 Lipopolysaccharide biosynthesis
00542 O-Antigen repeat unit biosynthesis
00541 O-Antigen nucleotide sugar biosynthesis
00550 Peptidoglycan biosynthesis
00511 Other glycan degradation
00571 Lipoarabinomannan (LAM) biosynthesis
00572 Arabinogalactan biosynthesis - Mycobacterium

Metabolism of cofactors and vitamins:
00730 Thiamine metabolism
00740 Riboflavin metabolism
00750 Vitamin B6 metabolism
00760 Nicotinate and nicotinamide metabolism
00770 Pantothenate and CoA biosynthesis
00780 Biotin metabolism
00785 Lipoic acid metabolism
00790 Folate biosynthesis
00670 One carbon pool by folate
00830 Retinol metabolism
00860 Porphyrin metabolism
00130 Ubiquinone and other terpenoid-quinone biosynthesis

Metabolism of terpenoids and polyketides:
00900 Terpenoid backbone biosynthesis

00902 Monoterpenoid biosynthesis
00909 Sesquiterpenoid and triterpenoid biosynthesis
00904 Diterpenoid biosynthesis
00906 Carotenoid biosynthesis
00905 Brassinosteroid biosynthesis
00981 Insect hormone biosynthesis
00908 Zeatin biosynthesis
00903 Limonene and pinene degradation
00281 Geraniol degradation
01052 Type I polyketide structures
00522 Biosynthesis of 12-, 14- and 16-membered macrolides
01051 Biosynthesis of ansamycins
01059 Biosynthesis of enediyne antibiotics
01056 Biosynthesis of type II polyketide backbone
01057 Biosynthesis of type II polyketide products
00253 Tetracycline biosynthesis
00523 Polyketide sugar unit biosynthesis
01054 Nonribosomal peptide structures
01053 Biosynthesis of siderophore group nonribosomal peptides
01055 Biosynthesis of vancomycin group antibiotics

Biosynthesis of other secondary metabolites:
00940 Phenylpropanoid biosynthesis
00945 Stilbenoid, diarylheptanoid and gingerol biosynthesis
00941 Flavonoid biosynthesis
00944 Flavone and flavonol biosynthesis
00942 Anthocyanin biosynthesis
00943 Isoflavonoid biosynthesis
00901 Indole alkaloid biosynthesis
00403 Indole diterpene alkaloid biosynthesis
00950 Isoquinoline alkaloid biosynthesis
00960 Tropane, piperidine and pyridine alkaloid biosynthesis
00996 Biosynthesis of various alkaloids
00232 Caffeine metabolism
00965 Betalain biosynthesis
00966 Glucosinolate biosynthesis
00402 Benzoxazinoid biosynthesis
00311 Penicillin and cephalosporin biosynthesis
00332 Carbapenem biosynthesis
00261 Monobactam biosynthesis
00331 Clavulanic acid biosynthesis
00521 Streptomycin biosynthesis
00524 Neomycin, kanamycin and gentamicin biosynthesis
00525 Acarbose and validamycin biosynthesis
00401 Novobiocin biosynthesis
00404 Staurosporine biosynthesis
00405 Phenazine biosynthesis
00333 Prodigiosin biosynthesis
00254 Aflatoxin biosynthesis
00998 Biosynthesis of various antibiotics
00999 Biosynthesis of various plant secondary metabolites
00997 Biosynthesis of various other secondary metabolites

Xenobiotics biodegradation and metabolism:
00362 Benzoate degradation
00627 Aminobenzoate degradation
00364 Fluorobenzoate degradation
00625 Chloroalkane and chloroalkene degradation

00361 Chlorocyclohexane and chlorobenzene degradation
00623 Toluene degradation
00622 Xylene degradation
00633 Nitrotoluene degradation
00642 Ethylbenzene degradation
00643 Styrene degradation
00791 Atrazine degradation
00930 Caprolactam degradation
00363 Bisphenol degradation
00621 Dioxin degradation
00626 Naphthalene degradation
00624 Polycyclic aromatic hydrocarbon degradation
00365 Furfural degradation
00984 Steroid degradation
00980 Metabolism of xenobiotics by cytochrome P450
00982 Drug metabolism - cytochrome P450
00983 Drug metabolism - other enzymes
