# Supplementary material for: Exploring the expressiveness of abstract metabolic networks
Source: PLoS One. 2023 Feb 9;18(2):e0281047. doi: 10.1371/journal.pone.0281047 (PMC9910719; doi:10.1371/journal.pone.0281047)
Supplement: S3 File — Mathematical formalization of the graph kernels used in the paper. (PDF) [file pone.0281047.s003.pdf]

Graph Kernels

Supplementary Material to

"Exploring the expressiveness of Abstract Metabolic Networks"
by Irene García[1], Bessem Chouaia[2],
Mercè Llabrés[1], Marta Simeoni*[2,3]

1 Mathematics and Computer Science Department, University of the Balearic Islands, E-07122 Palma, Spain

2 Dipartimento di Scienze Ambientali, Informatica e Statistica, Università Ca' Foscari Venezia, 30172 Venice, Italy

3 European Centre for Living Technology (ECLT), 30123 Venice, Italy

Graph kernels can be intuitively understood as functions measuring the similarity of pairs of graphs. In the paper we use them to compare abstract metabolic networks, a high-level representation of the metabolism as a graph, where nodes are metabolic pathways and edges represent their connections through shared compounds. A formal introduction on graph kernels can be found, e.g., in [2, 4, 5, 7, 3]. In the following, we briefly describe first the mathematical basis of graph kernels and then the specific kernels that we apply in the paper.

Let $\mathcal{X} = \{x_1, ..., x_N\}$ be a finite set. A *kernel function* is a function $k : \mathcal{X} \times \mathcal{X} \to \mathbb{R}$, such that the matrix, $M = (m_{ij})_{i,j=1,...N}$, with $m_{ij} = k(x_i, x_j)$ is a positive semidefinite matrix. M is called a *kernel matrix*.

For every kernel function $k : \mathcal{X} \times \mathcal{X} \to \mathbb{R}$, there exists a mapping, $\phi : \mathcal{X} \to \mathcal{H}$, into a Hilbert space $\mathcal{H}$, such that the *kernel matrix* is the real matrix whose entries are the inner product of the images of ϕ, i,e, $M = (m_{ij})_{i,j=1,...N}$ and $m_{ij} = \langle \phi(x_i), \phi(x_j) \rangle_{\mathcal{H}}$ where $\langle \cdot, \cdot \rangle_{\mathcal{H}}$ is the inner product in $\mathcal{H}$.

In the graph setting, a graph kernel is a kernel function defined on graphs. Every graph is represented by a vector, and the inner product of these vectors measures the graphs' similarity. Hence, a graph kernel is a function that quantifies the similarity of two graphs in polynomial time.

We used four different kernels to compare the abstract representation of metabolisms, all implemented by the GraKel Python library [6]. Since nodes in the abstract metabolic networks are labelled with the KEGG pathways identifier, we decided to exploit such information in the comparison. For this reason, the selected kernels include node labels among the features taken into consideration for the comparison.

From now on, we consider the particular case of a labelled graph being the abstract metabolic network of a given organism. Hence, let $\mathcal{G}$ be a finite set of graphs, each one being an abstract metabolic network and let $\mathcal{L} = \{l_1, ...l_d\}$ be the set of all the KEGG pathway identifiers present in $\mathcal{G}$. For every graph $G = (V, E) \in \mathcal{G}$, let $l : V \to \mathcal{L}$ be the function that assigns to every node $u \in V$ a label, $l(u)$, which is its KEGG pathway identifier. Notice that, l is an injective function, that is, every node has only one label and different nodes have different labels.

Here follows a brief description of the four considered kernels:

- **Vertex Histogram**: This is the simplest kernel. It compares node-labelled graphs and it only considers the node labels of the graphs to compare. Given a set of node labels, the vertex label histogram of a graph counts, for each label, the number of nodes in the graph labelled with it.

  More precisely, the *vertex label histogram* of a graph $G = (V, E)$, is a vector $f_G = (f_1, ..., f_d)$ such that $f_i = |\{v \in V | l(v) = l_i\}|$. Thus, given two graphs $G(V, E)$ and $G' = (V', E')$, the vertex histogram kernel is defined by
  $$k_{VH}(G, G') = \langle f_G, f_{G'} \rangle.$$
  Note that, in the case of metabolic networks, f_G is a vector whose entries are only 1 or 0, since every node has only one label and different nodes have different labels. Thus, this kernel only considers the presence or absence of pathways (node labels) in the two organisms under comparison and sum up the number of common pathways. Since only nodes and their labels, and not edges, are considered, the way metabolites are exchanged between pathways does not affect the comparison result.

- **Shortest-Path**: This kernel compares graphs in terms of their shortest paths. It first computes the *shortest-paths graph* of every input graph. The shortest-path graph of a graph $G = (V, E)$, is a graph $G_s = (V, E_s)$,

such that $\{u, v\} \in E_s$, if and only if, there is a path connecting the nodes u, v in G. Also, every edge $e = \{u, v\} \in E_s$ is labeled by the number $d_G(u, v)$, where $d_G(u, v)$ is the length of the shortest path in G that connects u and v.

The comparison is then performed by considering each pair of shortest paths in two given graphs and comparing their length and the labels of their endpoint nodes. The resulting scores are summed up to form the final similarity score.

More precisely, let $G(V, E)$ and $G' = (V', E')$ be two graphs and let $G_s = (V, E_s)$ and $G'_s = (V', E'_s)$ be their shortest-paths graphs. Then, the shortest-paths kernel is defined by

$$k_{SP}(G, G') = \sum_{e \in E_s} \sum_{e' \in E'_s} k(e, e')$$

where $k(e, e')$ for every $e = \{u, v\}$ and $e' = \{u', v'\}$ is defined by

$$k(e, e') = \delta(e, e')(\delta(v, v')\delta(u, u') + \delta(v, u')\delta(u, v'))$$

where, $\delta(x, y) = 1$ when $x = y$ and 0 otherwise.

- **Weisfeiler-Lehman (WL) subtree kernel**: The fundamental idea of the Weisfeiler-Lehman algorithm is to replace the label of each node by a multiset of labels consisting of the original label of the node and the sorted set of labels of its neighbours. The resultant multiset is then compressed into a new short label. Such a new label reflects the knowledge of the node and its neighbourhood. This relabelling process is then repeated for h iterations. By performing this procedure simultaneously on all input graphs, it follows that two nodes from different graphs will get identical new labels if and only if they have identical multiset labels. The kernel function, in this case, compares the node labels of the graphs resulting after each iteration and summarises the comparison with a real number. It can be shown that this is equivalent to comparing the number of shared subtrees between the two input graphs (the kernel considers all subtrees up to height h).

  More precisely, let $\mathcal{G}$ be a finite set of graphs, each one being an abstract metabolic network, and assume that an order has been fixed in the set of labels $\mathcal{L}$. Thus, without loss of generality, we can assume that

$\mathcal{L} = \{1, ..., d\}$, i.e. labels are the natural numbers from 1 to d. For every $G = (V, E), G' = (V', E') \in \mathcal{G}$, let $\mathcal{L}_0 = \{l(u) \,|\, u \in V\}$ and $\mathcal{L'}_0 = \{l(u) \,|\, u \in V'\}$ be the set of node labels of G and G', respectively. For every node $u \in V$, let $\eta(u)$ be the set of neighbours nodes of u, that is, $\eta(u) = \{v \in V, \,|\, \{u, v\} \in E\}$, and let $l_1(u)$ be the set of labels of $\eta(u)$ ordered under the natural numbers ordering. Notice that $\eta(u)$ is a set, since l is an injective mapping. Let us denote by $G_1 = (V, E)$ the node labeled graph with the same set of nodes and edges as G, and such that every node $u \in V$ is labeled by $\{l(u), \{l_1(u)\}\}$. Respectively, let us denote by $G'_1 = (V', E')$ the node labeled graph with the same set of nodes and edges as G', and such that every node $u' \in V'$ is labeled by $\{l(u'), \{l'_1(u')\}\}$. Let $\mathcal{L}_1 = \{l_1(u) | u \in V\}$ be the set of node labels of G_1 (resp. $\mathcal{L'}_1 = \{l_1(u') | u' \in V'\}$) and set $m = |(\mathcal{L}_1 \cup \mathcal{L'}_1) \setminus (\mathcal{L}_0 \cup \mathcal{L'}_o)|$. Then, a label compression step is defined by considering a bijective function $\varphi : (\mathcal{L}_1 \cup \mathcal{L'}_1) \setminus (\mathcal{L}_0 \cup \mathcal{L'}_0) \to \{n+1, ..., n+m\}$ and relabelling every node u in G_1 such that $l_1(u) \in \mathcal{L}_1 \setminus \mathcal{L}_0$ by $\varphi(l_1(u))$, and by $l(u)$ when $l(u) \in \mathcal{L}_0$, and, respectively, every node u' in G'_1 such that $l'_1(u') \in \mathcal{L'}_1 \setminus \mathcal{L'}_0$ by $\varphi(l'_1(u'))$, and by $l(u')$ when $l(u') \in \mathcal{L'}_0$.

Iterating this process, we define $G_h = (V, E)$ and $G'_h = (V, E)$ the node labeled graphs with the same set of nodes and edges as G, and G' respectively, and their nodes relabeled following the process explained above. Then, the Weisfeiler-Lehman kernel with h iterations is defined as,

$$k_{WL}(G, G') = k(G, G') + k(G_1, G'_1) + \cdots + k(G_h, G'_h)$$

where $k(G_i, G'_i)$ is the vertex histogram kernel.

- **Pyramid Match**: This graph kernel is an adaptation to the graph structure of the pyramid match kernel defined on sets [1]. Described at a high level, the Pyramid Match graph kernel first embeds the graph nodes into a vector space by considering the eigenvectors of the m largest in magnitude eigenvalues of the adjacency matrix of the graph. Thus, the nodes become points of an m dimensional hypercube. Next, to assess the similarity of two sets of nodes, the kernel maps these points into multi-resolution histograms and compares them with a weighted histogram intersection function.

  In the case of node labelled graphs, as abstract metabolic networks are, the kernel restricts the matchings to occur only between vertices

that share the same label. It represents each graph as a set of sets of vectors, and matches pairs of sets of two graphs corresponding to the same label using the original pyramid match kernel defined on sets. Thus, the emerging kernel for a pair of labeled graphs G, G' corresponds to the sum of the separate kernels,

$$k(G, G') = \sum_{i=1}^{d} k^i(G, G')$$

where d is the number of distinct labels and $k^i(G, G')$ is the pyramid match kernel between the sets of vertices of the two graphs having label i.

Initially, the kernel partitions the space into regions of increasingly larger size and takes a weighted sum of the matches that occur at each level. Two points match with each other if they fall into the same partition. However, since we consider injectively node labeled graphs, there is at most one node in each graph whose label is i. Hence, $k^i(G, G')$ compares only two nodes, i.e., two points of the m dimensional hypercube. These two points produce a match when they fall into the same partition. Thus, if the points are close to each other, the match is obtained at a lower level. Since the kernel takes a weighted sum of the matches that occur at each level, and there is only one match, the kernel's result depends only on the points distance. Therefore, with this kernel, we only compare the pairs of nodes with the same label and, for every pair, their similarity depends on the distance of their corresponding points. Since the points are the eigenvectors of the m largest in magnitude eigenvalues, it intuitively measures the similarity of the topological features (e.g. connectivity) of the nodes with the same label in the two compared graphs.

References

[1] Kristen Grauman and Trevor Darrell. The pyramid match kernel: Efficient learning with sets of features. *J. Mach. Learn. Res.*, 8:725–760, May 2007.

[2] Nils M. Kriege, Fredrik D. Johansson, and Christopher Morris. A survey on graph kernels. *Applied Network Science*, 5(1):6, 2020.

[3] Giannis Nikolentzos, Polykarpos Meladianos, and Michalis Vazirgiannis. Matching node embeddings for graph similarity. In *Proceedings of the Thirty-First AAAI Conference on Artificial Intelligence*, AAAI'17, page 2429–2435. AAAI Press, 2017.

[4] Giannis Nikolentzos, Giannis Siglidis, and Michalis Vazirgiannis. Graph kernels: A survey. arXiv e-print, 2019.

[5] Nino Shervashidze, Pascal Schweitzer, Erik Jan van Leeuwen, Kurt Mehlhorn, and Karsten M Borgwardt. Weisfeiler-lehman graph kernels. *Journal of Machine Learning Research*, 12(Sep):2539–2561, 2011.

[6] G. Siglidis, G. Nikolentzos, S. Limnios, C. Giatsidis, K. Skianis, and M. Vazirgiannis. Grakel: A Graph Kernel Library in Python. *ArXiv*, abs/1806.02193, 2018.

[7] Mahito Sugiyama and Karsten Borgwardt. Halting in random walk kernels. In C. Cortes, N. D. Lawrence, D. D. Lee, M. Sugiyama, and R. Garnett, editors, *Advances in Neural Information Processing Systems 28*, pages 1639–1647. Curran Associates, Inc., 2015.
