# Supplementary material for: Exploring the expressiveness of abstract metabolic networks
Source: PLoS One. 2023 Feb 9;18(2):e0281047. doi: 10.1371/journal.pone.0281047 (PMC9910719; doi:10.1371/journal.pone.0281047)
Supplement: S4 File — Complete set of analyses considering the whole set of KEGG organisms (first experiment). (PDF) [file pone.0281047.s004.pdf]

Whole Dataset Analysis

- Vertex Hystogram (VH) kernel
  - Heatmap
  - MDS for VH
  - 6-Means clustering for VH kernel
- Shortest path (SP) kernel
  - Heatmap
  - MDS for SP
  - 6-Means clustering for SP kernel
- Weisfeiler-Lehman (WL) kernel
  - Heatmap
  - MDS for WL
  - 6-Means clustering for WL kernel
- Pyramid match (PM) kernel
  - Heatmap
  - MDS for PM
  - 6-Means clustering for PM kernel

Vertex Hystogram (VH) kernel

Heatmap

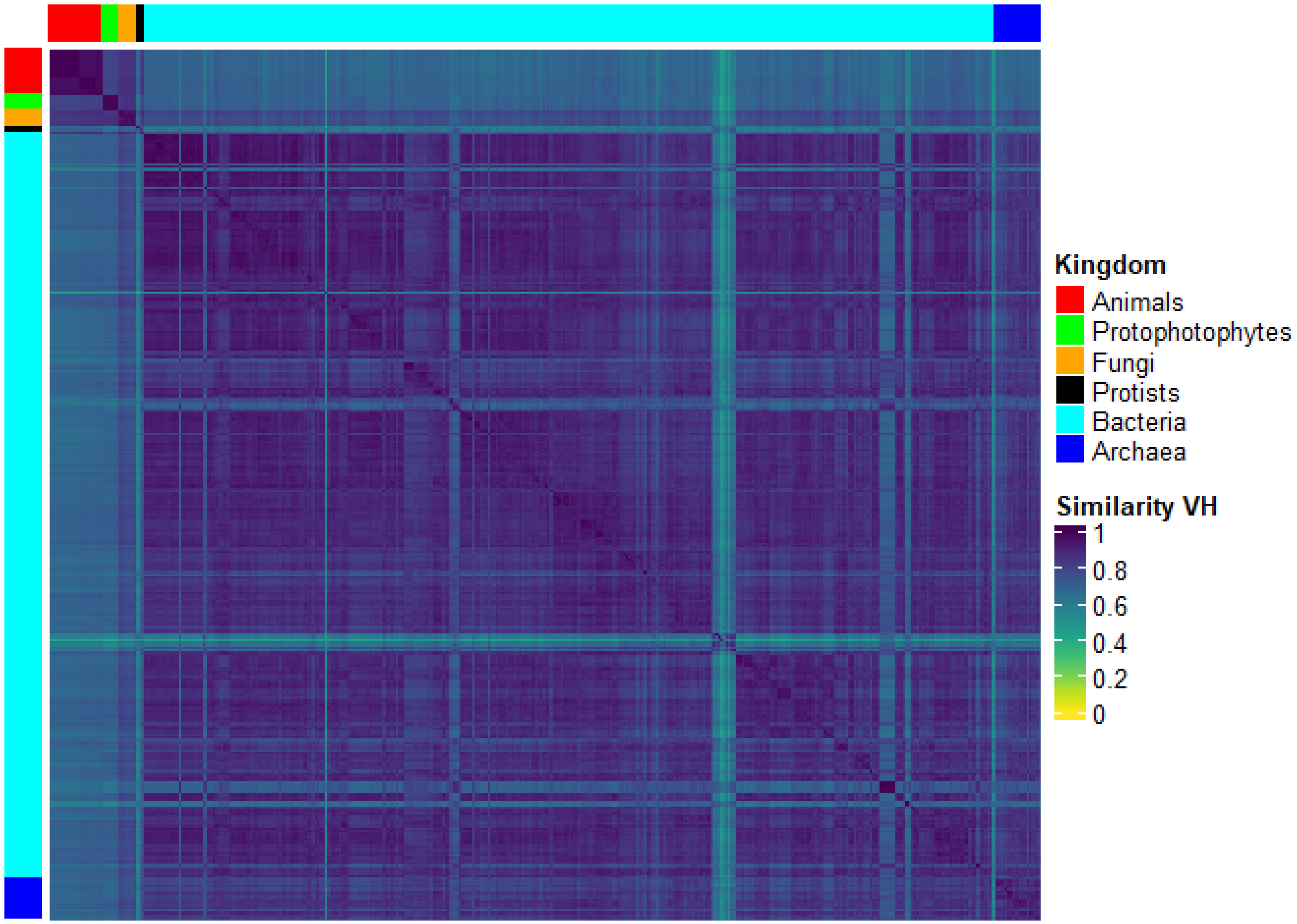

MDS for VH

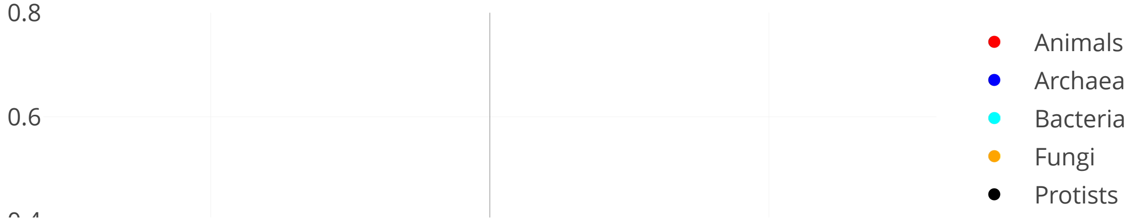

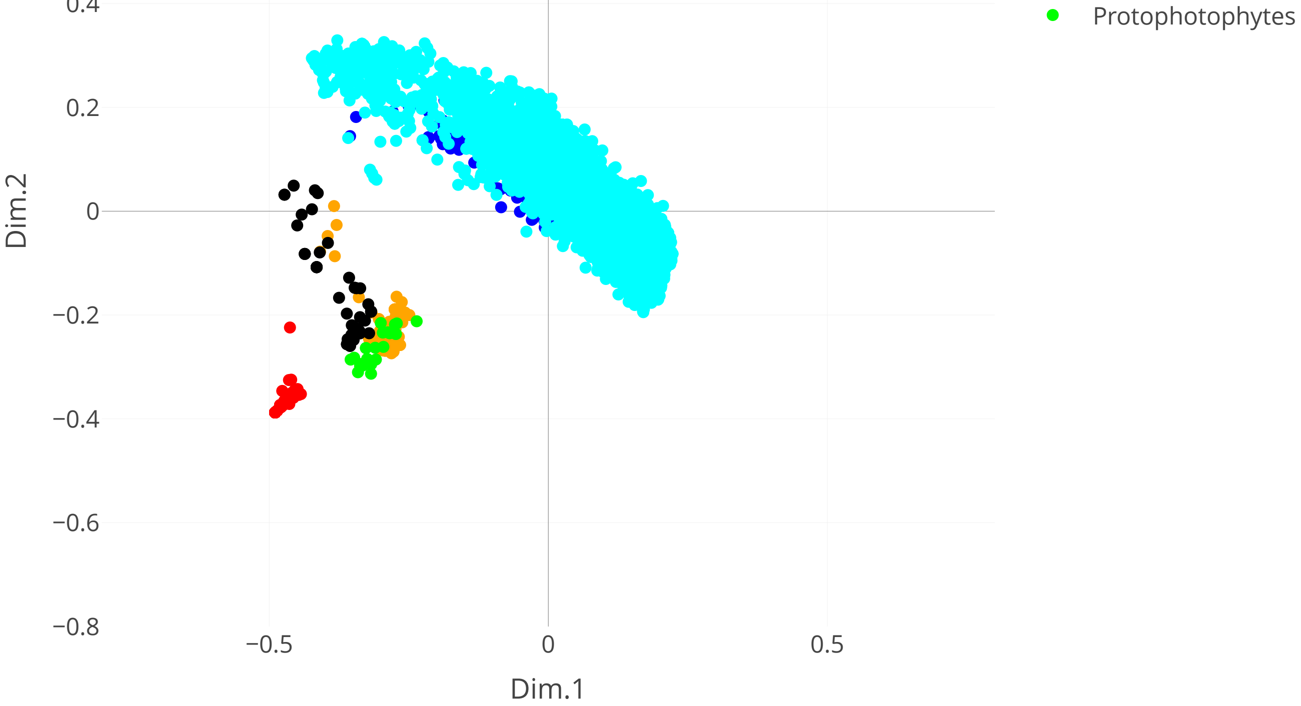

6-Means clustering for VH kernel

```
##                    Cluster
## Real group            1    2    3    4    5    6
##   Animals             0    0    1    0    0  369
##   Archaea             0  100    0   13  226    0
##   Bacteria         2501 1583    0  559 1472    0
##   Fungi               0    0  138    0    0    0
##   Protists            0    0   47    5    0    0
##   Protophotophytes    0    0  127    0    0    0
```

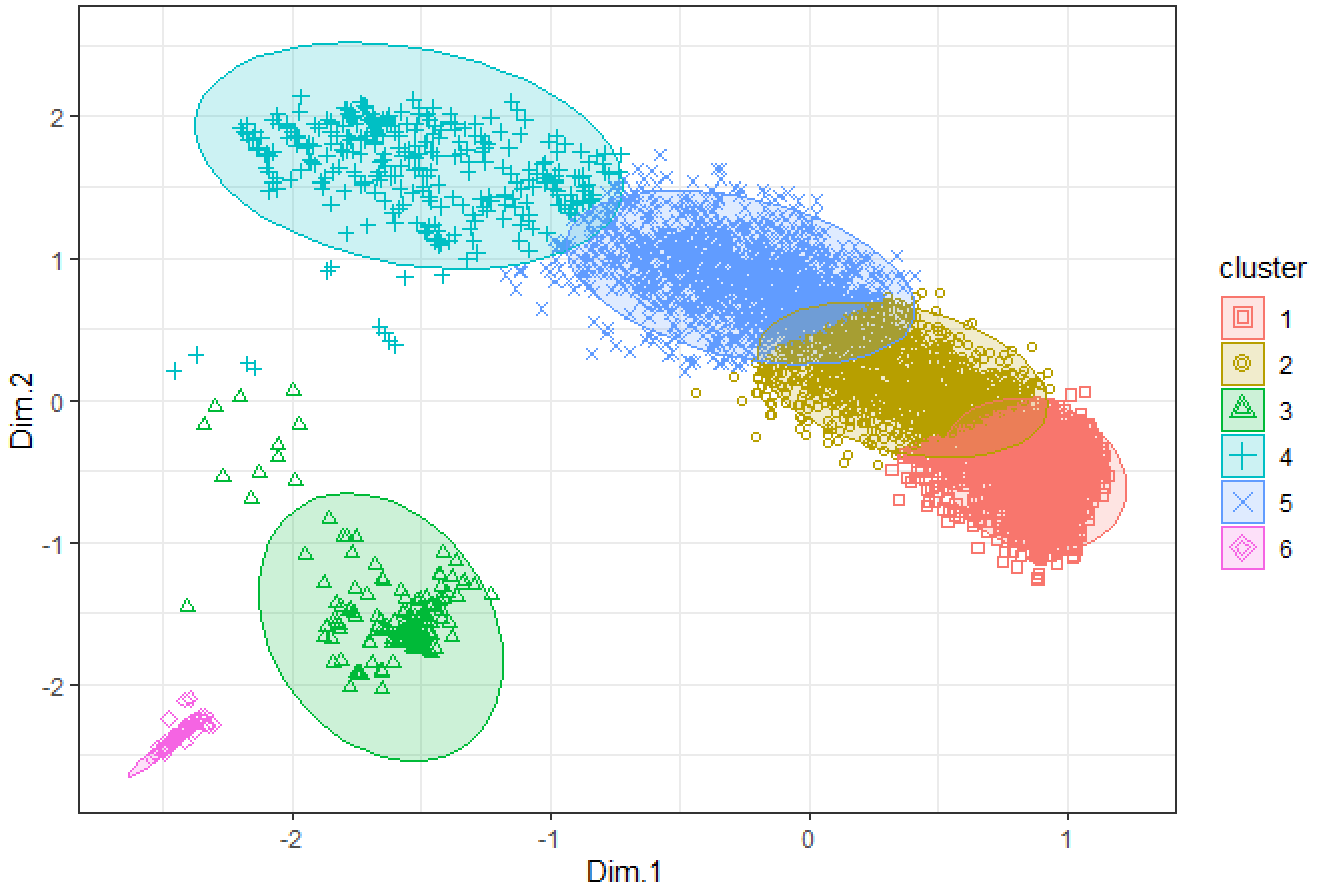

Organisms classified within cluster 1

```
##    [1] "eco"   "ecj"   "ecd"   "ebw"   "ecok" "ece"   "ecs"   "ecf"   "etw"   "elx"
##   [11] "eoi"   "eoj"   "eoh"   "ecoo" "ecoh" "esl"   "eso"   "esm"   "eck"   "ecg"
##   [21] "eok"   "elr"   "elh"   "ecw"   "eun"   "ecp"   "ena"   "ecos" "ecv"   "ecoa"
##   [31] "ecx"   "ecm"   "ecy"   "ecr"   "ecq"   "eum"   "ect"   "eoc"   "ebr"   "ebl"
##   [41] "ebe"   "ebd"   "eci"   "eih"   "ecz"   "ecc"   "elo"   "eln"   "ese"   "ecl"
##   [51] "eko"   "ekf"   "eab"   "edh"   "edj"   "elu"   "elw"   "ell"   "elc"   "eld"
##   [61] "elp"   "elf"   "ecol" "ecoi" "ecoj" "efe"   "eal"   "ema"   "esz"   "sty"
##   [71] "stt"   "sex"   "sent" "stm"   "seo"   "sev"   "sey"   "sem"   "sej"   "seb"
##   [81] "sef"   "setu" "setc" "senr" "send" "seni" "seen" "spt"   "sek"   "spq"
```

```
##  [91] "sei"  "sec"  "seh"  "shb"  "senh" "seeh" "see"  "senn" "sew"  "sea"
## [101] "sens" "seg"  "sel"  "sega" "set"  "sena" "seno" "senv" "senq" "senl"
## [111] "senj" "seec" "seeb" "seep" "senb" "sene" "senc" "ses"  "sbg"  "sbz"
## [121] "sbv"  "salz" "sfl"  "sfx"  "sfv"  "sfe"  "sfn"  "sfs"  "sft"  "ssn"
## [131] "sbo"  "sbc"  "sdy"  "sdz"  "shq"  "enc"  "enl"  "eclg" "ecle" "ecln"
## [141] "ecli" "eclx" "ecly" "eclz" "eclo" "ehm"  "exf"  "ecla" "eclc" "eau"
## [151] "ekb"  "eno"  "eec"  "elg"  "ecan" "ern"  "ecls" "echg" "esh"  "ent"
## [161] "eas"  "enr"  "enx"  "enf"  "ebg"  "end"  "esa"  "csk"  "csz"  "csj"
## [171] "ccon" "cdm"  "csi"  "cmj"  "cui"  "cmw"  "ctu"  "kpn"  "kpu"  "kpm"
## [181] "kpp"  "kph"  "kpz"  "kpv"  "kpw"  "kpy"  "kpg"  "kpc"  "kpq"  "kpt"
## [191] "kpo"  "kpr"  "kpj"  "kpi"  "kpa"  "kps"  "kpx"  "kpb"  "kpne" "kpnu"
## [201] "kpnk" "kva"  "kpe"  "kpk"  "kvd"  "kvq"  "kox"  "koe"  "koy"  "kom"
## [211] "kmi"  "kok"  "koc"  "kqu"  "eae"  "ear"  "kqv"  "kll"  "klw"  "cro"
## [221] "cko"  "cfd"  "cbra" "cwe"  "cyo"  "cpot" "cfq"  "cama" "caf"  "cif"
## [231] "cfar" "cir"  "cie"  "cpar" "ebt"  "ror"  "ron"  "rpln" "rao"  "rtg"
## [241] "ree"  "cnt"  "cem"  "cen"  "clap" "pge"  "esc"  "kle"  "ksa"  "kor"
## [251] "krd"  "kco"  "kot"  "kpse" "kgo"  "kie"  "kas"  "lax"  "lei"  "leh"
## [261] "lee"  "ler"  "lea"  "laz"  "lef"  "lni"  "lew"  "buf"  "bage" "mety"
## [271] "ahn"  "yre"  "sgoe" "kin"  "pdz"  "ebf"  "ebc"  "ebu"  "psts" "izh"
## [281] "ype"  "ypk"  "yph"  "ypa"  "ypn"  "ypm"  "ypp"  "ypg"  "ypz"  "ypt"
## [291] "ypd"  "ypx"  "ypw"  "ypj"  "ypv"  "ypl"  "yps"  "ypo"  "ypi"  "ypy"
## [301] "ypb"  "ypq"  "ypu"  "ypr"  "ypc"  "ypf"  "yen"  "yep"  "yey"  "yel"
## [311] "yew"  "yet"  "yef"  "yee"  "ysi"  "yal"  "yfr"  "yin"  "ykr"  "yro"
## [321] "yru"  "yrb"  "yak"  "yma"  "yhi"  "yca"  "ymo"  "smar" "smac" "smw"
## [331] "spe"  "srr"  "srl"  "sry"  "sply" "srs"  "sra"  "smaf" "slq"  "serf"
## [341] "sers" "sfw"  "sfg"  "srz"  "sera" "serq" "serm" "squ"  "sfj"  "sof"
## [351] "ssur" "sfo"  "rah"  "raq"  "raa"  "rox"  "gqu"  "eame" "rbad" "eca"
## [361] "patr" "pato" "pct"  "pcc"  "pcv"  "pwa"  "ppar" "pec"  "pws"  "ppoa"
## [371] "pbra" "ppuj" "ddd"  "dda"  "dze"  "ddc"  "dzc"  "dso"  "ced"  "dfn"
## [381] "ddq"  "daq"  "dic"  "bgj"  "brb"  "bng"  "lbq"  "sod"  "eam"  "eay"
## [391] "eta"  "epy"  "epr"  "ebi"  "erj"  "ege"  "epe"  "erwi" "pam"  "plf"
## [401] "paj"  "paq"  "pva"  "pagg" "pao"  "kln"  "pant" "panp" "pagc" "pstw"
## [411] "palh" "pans" "pey"  "pdis" "pgz"  "pcd"  "mint" "mthi" "tci"  "plu"
## [421] "plum" "xho"  "hav"  "hpar" "opo"  "xcc"  "xcb"  "xca"  "xcp"  "xcv"
```

```
## [431] "xax"  "xac"  "xci"  "xct"  "xcj"  "xcu"  "xcn"  "xcw"  "xcr"  "xcm"
## [441] "xcf"  "xfu"  "xao"  "xom"  "xop"  "xor"  "xoz"  "xal"  "xsa"  "xtn"
## [451] "xfr"  "xve"  "xpe"  "xhr"  "xga"  "xph"  "xva"  "xan"  "xar"  "xhy"
## [461] "xcz"  "xth"  "sml"  "smt"  "buj"  "smz"  "sacz" "stek" "srh"  "slm"
## [471] "sten" "stem" "stes" "psu"  "psuw" "psd"  "pmex" "lab"  "laq"  "lcp"
## [481] "lgu"  "lez"  "lem"  "lmb"  "lyj"  "lsol" "lum"  "lus"  "thes" "theh"
## [491] "tcn"  "tbv"  "xbc"  "fau"  "rhd"  "rgl"  "dji"  "dja"  "dtx"  "dye"
## [501] "dko"  "lrz"  "lpy"  "xba"  "rbd"  "vvu"  "vvy"  "vvm"  "vvl"  "vpa"
## [511] "vpb"  "vpk"  "vpf"  "vph"  "vha"  "vca"  "vag"  "vex"  "vdb"  "vhr"
## [521] "vna"  "vow"  "vro"  "vej"  "vfu"  "vni"  "vcy"  "vct"  "vfl"  "vmi"
## [531] "vsh"  "vaf"  "vnl"  "vcc"  "vas"  "pae"  "paev" "paei" "pau"  "pap"
## [541] "pag"  "paf"  "pnc"  "paeb" "pdk"  "psg"  "prp"  "paep" "paer" "paem"
## [551] "pael" "paes" "paeu" "paeg" "paec" "paeo" "pmy"  "pmk"  "pre"  "ppse"
## [561] "palc" "pcq"  "ppu"  "ppf"  "ppg"  "ppw"  "ppt"  "ppb"  "ppi"  "ppx"
## [571] "ppuh" "pput" "ppun" "ppud" "pfv"  "pmon" "pmot" "pmos" "ppj"  "por"
## [581] "pst"  "psb"  "psyr" "psp"  "pamg" "pci"  "pavl" "pvd"  "pfl"  "pprc"
## [591] "ppro" "pfo"  "pfs"  "pfe"  "pfc"  "pfn"  "ppz"  "pfb"  "pman" "ptv"
## [601] "pcg"  "pvr"  "pazo" "poi"  "pfw"  "pff"  "pfx"  "pen"  "psa"  "psz"
## [611] "psr"  "psc"  "psj"  "psh"  "pstu" "pstt" "pbm"  "plul" "pba"  "pbc"
## [621] "ppuu" "pdr"  "psv"  "psk"  "pkc"  "pch"  "pcz"  "pcp"  "pfz"  "plq"
## [631] "palk" "prh"  "psw"  "ppv"  "pses" "psem" "psec" "ppsy" "psos" "pkr"
## [641] "pfk"  "panr" "ppsl" "pset" "psil" "pym"  "psed" "pke"  "pall" "pum"
## [651] "poj"  "pgg"  "ppsh" "pgy"  "avn"  "avl"  "avd"  "acx"  "pagr" "pcr"
## [661] "pso"  "pali" "pspg" "psyg" "psyp" "acb"  "aby"  "abc"  "abn"  "abb"
## [671] "abx"  "abz"  "abr"  "abd"  "abh"  "abad" "abj"  "abab" "abaj" "abaz"
## [681] "abk"  "abau" "abaa" "abw"  "abal" "acc"  "ano"  "alc"  "acal" "acd"
## [691] "aci"  "att"  "aei"  "ajo"  "acw"  "acv"  "ahl"  "ajn"  "asol" "ala"
## [701] "asj"  "aid"  "adv"  "arj"  "awu"  "acum" "agu"  "alw"  "ads"  "aber"
## [711] "atn"  "achi" "alj"  "mbah" "son"  "sdn"  "sfr"  "saz"  "sbl"  "sbm"
## [721] "sbn"  "sbp"  "sbt"  "sbs"  "sbb"  "slo"  "spc"  "shp"  "sse"  "spl"
## [731] "she"  "shm"  "shn"  "shw"  "shl"  "swd"  "swp"  "svo"  "shf"  "sja"
## [741] "spsw" "sbj"  "smav" "shew" "salg" "slj"  "spol" "sbk"  "skh"  "saes"
## [751] "cps"  "com"  "coz"  "colw" "cber" "tht"  "thap" "pha"  "ptn"  "pat"
## [761] "psm"  "pseo" "pia"  "pphe" "pbw"  "prr"  "plz"  "paln" "ppis" "pea"
```

```
##  [771] "pspo" "part" "ptu"  "png"  "ptd"  "pdj"  "paga" "pcar" "pmaa" "maq"
##  [781] "mhc"  "mad"  "mbs"  "mpq"  "mari" "mlq"  "msq"  "mara" "marj" "amc"
##  [791] "amh"  "amaa" "amal" "amae" "amao" "amad" "amai" "amag" "amac" "amb"
##  [801] "amg"  "amk"  "alt"  "aal"  "aaus" "asp"  "asq"  "aaw"  "alr"  "ale"
##  [811] "alz"  "apel" "gag"  "gni"  "gps"  "pmes" "lal"  "cate" "salm" "hmi"
##  [821] "pin"  "mvs"  "mmaa" "cja"  "ceb"  "cell" "cek"  "ceg"  "sde"  "ttu"
##  [831] "saga" "spoi" "zal"  "osg"  "mthd" "micc" "mii"  "mict" "hja"  "halc"
##  [841] "kim"  "lha"  "lcd"  "lcj"  "mmai" "cyq"  "cza"  "nhl"  "tee"  "wma"
##  [851] "gai"  "hch"  "hahe" "csa"  "hel"  "hcs"  "hak"  "ham"  "hhu"  "hco"
##  [861] "hsi"  "halo" "hhh"  "hbe"  "haf"  "halk" "hvn"  "hol"  "hsr"  "hmd"
##  [871] "haxi" "htt"  "hcam" "hpiz" "haa"  "cmai" "abo"  "adi"  "apac" "aln"
##  [881] "axe"  "kak"  "mmw"  "mme"  "mpc"  "mpri" "mard" "oai"  "mars" "bsan"
##  [891] "ncu"  "nik"  "ajp"  "gsn"  "rfo"  "ome"  "aha"  "ahy"  "ahd"  "ahr"
##  [901] "ahp"  "ahj"  "ahh"  "ahi"  "aaj"  "avr"  "avo"  "adh"  "arv"  "aes"
##  [911] "ael"  "oce"  "zdf"  "sok"  "sini" "gbi"  "saln" "pspi" "gpb"  "vff"
##  [921] "cvi"  "cvc"  "chro" "chri" "chrb" "crz"  "chrm" "chae" "iod"  "ifl"
##  [931] "pse"  "jeu"  "aql"  "amah" "aqs"  "dee"  "chiz" "cfon" "rso"  "rsc"
##  [941] "rsl"  "rsn"  "rsm"  "rse"  "rsy"  "rpi"  "rpf"  "rpj"  "rmn"  "rin"
##  [951] "rpu"  "reh"  "cnc"  "cuh"  "reu"  "rme"  "cti"  "cbw"  "cgd"  "ccup"
##  [961] "cup"  "cuu"  "cpau" "cox"  "bma"  "bmv"  "bml"  "bmn"  "bmal" "bmae"
##  [971] "bmaq" "bmai" "bmaf" "bmaz" "bmab" "bps"  "bpm"  "bpl"  "bpd"  "bpr"
##  [981] "bpse" "bpsm" "bpsu" "bpsd" "bpz"  "bpq"  "bpk"  "bpsh" "bpsa" "bpso"
##  [991] "but"  "bte"  "btq"  "btj"  "btz"  "btd"  "btv"  "bthe" "bthm" "btha"
## [1001] "bthl" "bok"  "boc"  "buu"  "bvi"  "bve"  "bur"  "bcn"  "bch"  "bcm"
## [1011] "bcj"  "bcen" "bcew" "bceo" "bam"  "bac"  "bmj"  "bmu"  "bmk"  "bmul"
## [1021] "bct"  "bced" "bcep" "bdl"  "bpyr" "bcon" "bub"  "bdf"  "blat" "btei"
## [1031] "bsem" "bpsl" "bmec" "bstg" "bstl" "bgl"  "bgu"  "bug"  "bgf"  "bgd"
## [1041] "bgo"  "byi"  "buk"  "bue"  "bul"  "buq"  "bgp"  "bpla" "bud"  "bum"
## [1051] "bui"  "bxe"  "bxb"  "bph"  "bge"  "bpx"  "bpy"  "buz"  "bfn"  "bcai"
## [1061] "pspw" "para" "parb" "phs"  "pter" "pgp"  "pcj"  "pts"  "pcaf" "pmeg"
## [1071] "brh"  "ppk"  "ppno" "ppnm" "prb"  "ppul" "pspu" "papi" "pve"  "pox"
## [1081] "ptx"  "pfg"  "pnr"  "pand" "pfib" "plg"  "hyf"  "caba" "buo"  "limn"
## [1091] "cari" "bpe"  "bpc"  "bper" "bpet" "bpeu" "bpar" "bpa"  "bbh"  "bbr"
## [1101] "bbm"  "bbx"  "bpt"  "bav"  "bho"  "bhm"  "bhz"  "btrm" "bbro" "bfz"
```

```
## [1111] "bpdz" "boh"  "bgm"  "boj"  "boz"  "axy"  "axo"  "axn"  "axx"  "adt"
## [1121] "ais"  "asw"  "achr" "achb" "put"  "pus"  "pud"  "aka"  "amim" "cdn"
## [1131] "afa"  "afq"  "aaqu" "odi"  "pig"  "pacr" "kgy"  "rfr"  "rsb"  "rac"
## [1141] "rhy"  "rhf"  "rhg"  "pol"  "pna"  "pos"  "poo"  "aav"  "ajs"  "dia"
## [1151] "aaa"  "ack"  "acra" "acid" "acip" "acin" "acis" "acio" "amon" "vei"
## [1161] "dac"  "del"  "dts"  "dhk"  "dla"  "vap"  "vpe"  "vpd"  "vaa"  "vbo"
## [1171] "vam"  "ctt"  "ctes" "cke"  "cser" "cof"  "adn"  "adk"  "rta"  "otk"
## [1181] "lim"  "lih"  "hyr"  "hyb"  "hyl"  "hyc"  "hpse" "hyn"  "dpy"  "dih"
## [1191] "daer" "drg"  "simp" "melm" "mela" "sthm" "mpt"  "metp" "har"  "mms"
## [1201] "jag"  "jab"  "jaz"  "jal"  "jsv"  "jaj"  "jas"  "jlv"  "hse"  "hsz"
## [1211] "hht"  "hrb"  "hee"  "hhf"  "hfr"  "cfu"  "care" "cpra" "mnr"  "masw"
## [1221] "mass" "masz" "mtim" "masy" "mali" "mum"  "mfla" "mpli" "upv"  "nok"
## [1231] "dug"  "lch"  "thi"  "rge"  "rbn"  "rdp"  "pkt"  "miu"  "rgu"  "aon"
## [1241] "snn"  "xyk"  "pbh"  "shd"  "metr" "doe"  "uru"  "upl"  "eba"  "dsu"
## [1251] "rbu"  "otr"  "dar"  "dey"  "azo"  "aoa"  "aza"  "azi"  "atw"  "acom"
## [1261] "azd"  "azr"  "azq"  "tmz"  "thu"  "tcl"  "thk"  "app"  "beb"  "beba"
## [1271] "alk"  "dwd"  "dalk" "mxa"  "msd"  "mym"  "mfb"  "ccx"  "mfu"  "mmas"
## [1281] "sur"  "age"  "mbd"  "cfus" "scl"  "scu"  "ccro" "samy" "llu"  "mrm"
## [1291] "hoh"  "dti"  "pcay" "mlo"  "mln"  "mci"  "mop"  "mam"  "mamo" "meso"
## [1301] "mesw" "mesm" "mesp" "mhua" "mjr"  "merd" "mes"  "hoe"  "aak"  "amih"
## [1311] "pht"  "niy"  "orm"  "pla"  "pmob" "rbs"  "sme"  "smk"  "smq"  "smx"
## [1321] "smi"  "smeg" "smel" "smer" "smd"  "rhi"  "sfh"  "sfd"  "six"  "same"
## [1331] "sino" "ead"  "eah"  "esj"  "eak"  "emx"  "atu"  "ara"  "ata"  "agr"
## [1341] "atf"  "avi"  "agc"  "aro"  "agt"  "alf"  "ret"  "rec"  "rel"  "rep"
## [1351] "rei"  "rle"  "rlt"  "rlg"  "rlb"  "rlu"  "rtr"  "rir"  "rpus" "rhl"
## [1361] "rga"  "rhn"  "rpha" "rhx"  "rhv"  "rhk"  "rez"  "rjg"  "rhr"  "rgr"
## [1371] "rad"  "roy"  "rii"  "ngl"  "ngg"  "neo"  "nen"  "rht"  "shz"  "abaw"
## [1381] "kai"  "bmi"  "bmz"  "bmee" "bms"  "bsi"  "bsf"  "bsv"  "bsw"  "bcs"
## [1391] "bsk"  "bol"  "bcar" "bcas" "bmr"  "bpv"  "oin"  "oan"  "oah"  "ops"
## [1401] "och"  "bja"  "bju"  "bjp"  "bra"  "bbt"  "brs"  "aol"  "brc"  "brad"
## [1411] "bic"  "bro"  "brk"  "bot"  "brq"  "bgq"  "bgz"  "bsym" "bbet" "barh"
## [1421] "bvz"  "rpa"  "rpb"  "rpc"  "rpd"  "rpe"  "rpt"  "rpx"  "oca"  "ocg"
## [1431] "oco"  "bop"  "bos"  "bvv"  "boi"  "bof"  "vgo"  "trb"  "xau"  "azc"
## [1441] "sno"  "star" "lne"  "anc"  "apra" "mea"  "mdi"  "mex"  "mch"  "mpo"
```

```
## [1451] "mza"  "mrd"  "met"  "mno"  "mor"  "meta" "maqu" "mphy" "mee"  "metd"
## [1461] "metx" "mets" "meti" "mmes" "mtea" "moc"  "miv"  "mico" "bid"  "msl"
## [1471] "mtun" "bbar" "chel" "cdq"  "hdn"  "hdt"  "hmc"  "phl"  "deq"  "dei"
## [1481] "dea"  "rhz"  "yti"  "ntd"  "msc"  "mbry" "mros" "mhey" "mpar" "mtw"
## [1491] "pleo" "mey"  "maad" "mmed" "aua"  "brn"  "psin" "hdi"  "noh"  "rbm"
## [1501] "pphr" "lap"  "lagg" "labr" "labp" "labt" "siw"  "ccr"  "ccs"  "cak"
## [1511] "cse"  "chq"  "cmb"  "cfh"  "cauf" "pzu"  "phb"  "bsb"  "brl"  "bvc"
## [1521] "brev" "bvy"  "aex"  "tsv"  "cbot" "sil"  "sit"  "rua"  "rut"  "rmb"
## [1531] "jan"  "rpon" "pde"  "pami" "pye"  "pzh"  "paro" "parr" "pkd"  "ppan"
## [1541] "dsh"  "pga"  "pgl"  "pgd"  "php"  "ppic" "phq"  "oat"  "lmd"  "lej"
## [1551] "laqu" "cid"  "ceh"  "cmag" "malg" "con"  "rsu"  "rhm"  "rhc"  "hat"
## [1561] "daa"  "ypac" "yan"  "tpro" "suam" "spse" "suli" "suld" "spot" "tom"
## [1571] "paby" "thw"  "tec"  "rmm"  "rok"  "aht"  "rbg"  "sagu" "thaa" "geh"
## [1581] "taw"  "salo" "hml"  "pseb" "lit"  "ocd"  "maru" "rot"  "ppru" "mon"
## [1591] "malu" "tgl"  "pshq" "poz"  "palw" "ppaf" "pgv"  "thas" "faq"  "hdh"
## [1601] "gak"  "hne"  "hba"  "hbc"  "nar"  "npp"  "npn"  "nre"  "nov"  "not"
## [1611] "nor"  "ngf"  "nog"  "sal"  "sphk" "sphp" "smag" "smaz" "ster" "sgi"
## [1621] "sphq" "spho" "sphx" "sphu" "swi"  "sphd" "sphm" "stax" "sphi" "ssan"
## [1631] "snj"  "smy"  "span" "skr"  "splm" "splk" "spkc" "sphc" "sphf" "spha"
## [1641] "spau" "sech" "sjp"  "sch"  "ssy"  "syb"  "sbd"  "spmi" "sphb" "sphr"
## [1651] "sinb" "spht" "shyd" "sya"  "sclo" "spyg" "sufl" "sami" "sbar" "cij"
## [1661] "sphg" "sfla" "sphy" "blas" "bfw"  "rdi"  "sphj" "spzr" "palg" "smic"
## [1671] "sphs" "sand" "aay"  "aep"  "alb"  "alh"  "amx"  "anh"  "ado"  "cna"
## [1681] "cman" "ery"  "egn"  "eli"  "elq"  "erk"  "err"  "erf"  "emv"  "pns"
## [1691] "porl" "phz"  "pot"  "gbh"  "gbc"  "gbs"  "acr"  "amv"  "gdi"  "ksc"
## [1701] "kha"  "apf"  "apu"  "apg"  "apq"  "apx"  "apz"  "apk"  "asz"  "ato"
## [1711] "rgi"  "ros"  "rmuc" "shum" "svc"  "rru"  "rce"  "mag"  "mgy"  "mgry"
## [1721] "magx" "magn" "azl"  "ali"  "abs"  "abq"  "abf"  "ati"  "azt"  "azm"
## [1731] "azz"  "aoz"  "tmo"  "txi"  "thac" "tii"  "magq" "nao"  "ncb"  "fer"
## [1741] "htq"  "hadh" "skt"  "ecog" "phr"  "pstg" "bdz"  "sbf"  "bwd"  "bmob"
## [1751] "baci" "beo"  "bmd"  "bmeg" "bko"  "bmur" "pbut" "meku" "pjd"  "gym"
## [1761] "pms"  "pmq"  "pmw"  "pvo"  "plw"  "ppsc" "palb" "pbk"  "asoc" "aac"
## [1771] "aad"  "bts"  "eff"  "ssil" "pku"  "pmar" "pfae" "mtu"  "mtv"  "mtc"
## [1781] "mra"  "mtf"  "mtb"  "mtk"  "mtz"  "mtg"  "mti"  "mte"  "mtur" "mtl"
```

```
## [1791] "mto"  "mtd"  "mtn"  "mtj"  "mtub" "mtuc" "mtue" "mtx"  "mtuh" "mtul"
## [1801] "mtut" "mtuu" "mtq"  "mbo"  "mbb"  "mbt"  "mbm"  "mbk"  "mbx"  "maf"
## [1811] "mmic" "mce"  "mcq"  "mcv"  "mcx"  "mcz"  "mpa"  "mao"  "mavi" "mavu"
## [1821] "mav"  "mit"  "mia"  "mid"  "myo"  "mchi" "mir"  "mmal" "mlp"  "msa"
## [1831] "mul"  "mmc"  "mkm"  "mjl"  "mmi"  "mmae" "mmm"  "mli"  "mkn"  "myv"
## [1841] "mye"  "mhad" "mdx"  "mshg" "mfj"  "mgro" "mxe"  "mnv"  "mpag" "mnm"
## [1851] "mgor" "mcoo" "msm"  "msg"  "msb"  "msn"  "msh"  "mva"  "mgi"  "msp"
## [1861] "mcb"  "mne"  "myn"  "mgo"  "mft"  "mphl" "mvq"  "mll"  "mrh"  "mthn"
## [1871] "mhas" "mdu"  "mcht" "mdr"  "mauu" "mmag" "mmor" "mfx"  "maic" "mij"
## [1881] "malv" "mty"  "mpsc" "mab"  "mmv"  "mabb" "mabl" "mche" "miz"  "mste"
## [1891] "msao" "msal" "mjd"  "mter" "mmin" "mhib" "asd"  "mkr"  "cef"  "cva"
## [1901] "chn"  "cgy"  "cmq"  "clw"  "cpre" "nfa"  "nfr"  "ncy"  "nbr"  "nno"
## [1911] "nsl"  "nsr"  "ntp"  "noz"  "nod"  "nah"  "nad"  "nwl"  "rha"  "rer"
## [1921] "rey"  "reb"  "rop"  "roa"  "req"  "rpy"  "rhb"  "rav"  "rfa"  "rhw"
## [1931] "rhs"  "rrz"  "rhu"  "rqi"  "rhq"  "rhod" "rrt"  "rby"  "rcr"  "rtm"
## [1941] "gbr"  "gpo"  "gor"  "goq"  "gta"  "goc"  "git"  "gru"  "gom"  "gav"
## [1951] "god"  "tpr"  "tsm"  "srt"  "dtm"  "dit"  "diz"  "dpc"  "dlu"  "toy"
## [1961] "sco"  "salb" "sma"  "sgr"  "sgb"  "scb"  "ssx"  "svl"  "sct"  "scy"
## [1971] "sfa"  "sbh"  "shy"  "sho"  "sve"  "sdv"  "sals" "strp" "sfi"  "sci"
## [1981] "src"  "salu" "sall" "slv"  "sgu"  "svt"  "stre" "scw"  "sld"  "slc"
## [1991] "sxi"  "strm" "strc" "samb" "spri" "scz"  "scx"  "srw"  "strf" "sle"
## [2001] "srn"  "spav" "strt" "sclf" "sgs"  "stsi" "sls"  "snr"  "splu" "strd"
## [2011] "snw"  "sauo" "ssia" "svu"  "spun" "sgv"  "smal" "slau" "salf" "salj"
## [2021] "slx"  "stro" "sfk"  "snz"  "sge"  "srj"  "slk"  "sky"  "sdx"  "sgd"
## [2031] "sqz"  "scya" "sast" "snq"  "stir" "ska"  "sgz"  "svn"  "snk"  "salw"
## [2041] "shaw" "srk"  "sfic" "sgal" "sspo" "svr"  "spad" "sfy"  "saqu" "sgf"
## [2051] "scav" "sseo" "ksk"  "kab"  "kau"  "kit"  "stri" "leif" "lse"  "cmi"
## [2061] "cms"  "cmc"  "cmh"  "ccap" "mts"  "mim"  "mio"  "mip"  "mcw"  "mpal"
## [2071] "mih"  "micr" "mhos" "moo"  "mlv"  "mwa"  "mprt" "msed" "moy"  "rtn"
## [2081] "rry"  "ria"  "rfs"  "rte"  "cum"  "cub"  "mvd"  "frp"  "agy"  "agm"
## [2091] "agf"  "cart" "cry"  "cphy" "amin" "aum"  "auw"  "myl"  "salc" "sala"
## [2101] "sald" "hum"  "huw"  "gry"  "lyd"  "plap" "ltr"  "ldn"  "agg"  "mant"
## [2111] "hea"  "frn"  "gln"  "chre" "agx"  "art"  "arr"  "arm"  "arl"  "are"
## [2121] "aaq"  "arw"  "arh"  "ary"  "arz"  "aru"  "arq"  "arn"  "arx"  "acry"
```

```
## [2131] "arth" "artp" "acit" "ari"  "aau"  "pue"  "ach"  "apn"  "psul" "psni"
## [2141] "psey" "aai"  "gar"  "gcr"  "glu"  "rsa"  "kpl"  "kfv"  "krs"  "mick"
## [2151] "satk" "nae"  "bcv"  "bfa"  "brx"  "brv"  "bgg"  "brz"  "bsau" "brr"
## [2161] "lmoi" "xyl"  "ido"  "cet"  "cceu" "celh" "psei" "ica"  "jte"  "jli"
## [2171] "jme"  "phw"  "pei"  "serj" "serw" "orn"  "orz"  "blin" "bcau" "aus"
## [2181] "halt" "mph"  "mik"  "micg" "tes"  "rain" "prv"  "nca"  "ndk"  "noy"
## [2191] "noo"  "nsn"  "nbe"  "nano" "nmes" "psim" "aer"  "aez"  "aeb"  "aef"
## [2201] "mgg"  "muz"  "kfl"  "kqi"  "nda"  "nal"  "ngv"  "strr" "tcu"  "actw"
## [2211] "sro"  "noa"  "now"  "tbi"  "fra"  "fre"  "fri"  "fal"  "fsy"  "nml"
## [2221] "gob"  "bsd"  "mmar" "kra"  "sen"  "sace" "sacg" "svi"  "sacc" "amd"
## [2231] "amn"  "amm"  "amz"  "aoi"  "aja"  "amq"  "amyc" "amyb" "aab"  "amyy"
## [2241] "aori" "pdx"  "psea" "psee" "pseh" "pseq" "pecq" "phh"  "paut" "apre"
## [2251] "ami"  "sesp" "ssyi" "kal"  "kphy" "led"  "ahm"  "acti" "acad" "ahg"
## [2261] "acta" "alo"  "pmad" "stp"  "saq"  "mau"  "mil"  "micb" "mtua" "mich"
## [2271] "mtem" "mcab" "msag" "vma"  "mcra" "ase"  "ams"  "actn" "afs"  "acts"
## [2281] "plk"  "plab" "plat" "pfla" "psuu" "ver"  "cai"  "sna"  "aey"  "eke"
## [2291] "abai" "rxy"  "rub"  "bsol" "cwo"  "aym"  "atq"  "euz"  "len"  "lbo"
## [2301] "amr"  "glp"  "mvz"  "cyt"  "cyp"  "cyh"  "cyj"  "cyn"  "ter"  "mic"
## [2311] "oni"  "mpro" "nos"  "nop"  "non"  "nfl"  "noe"  "nsh"  "ned"  "calo"
## [2321] "calt" "calh" "riv"  "fis"  "toq"  "ncn"  "cthe" "plp"  "rrs"  "rca"
## [2331] "cau"  "chl"  "hau"  "cap"  "pbf"  "kbs"  "ddr"  "dez"  "dein" "tra"
## [2341] "roo"  "luo"  "rba"  "psl"  "rol"  "ahel" "lcre" "aagg" "lpav" "amuc"
## [2351] "pnd"  "pbs"  "pls"  "gmr"  "gim"  "mri"  "sdyn" "plon" "ges"  "gog"
## [2361] "gms"  "tim"  "lrs"  "ftj"  "uli"  "saci" "pbor" "agv"  "slr"  "laj"
## [2371] "lkm"  "tpx"  "aba"  "aca"  "acm"  "gma"  "grw"  "tsa"  "trs"  "talb"
## [2381] "abas" "eda"  "sus"  "pfer" "ctm"  "abac" "gau"  "gph"  "gba"  "drc"
## [2391] "sru"  "srm"  "rmr"  "rmg"  "cpi"  "cbae" "chit" "chih" "nko"  "fla"
## [2401] "fln"  "pseg" "pgin" "pgo"  "lacs" "hhy"  "phe"  "pep"  "pcm"  "psty"
## [2411] "pek"  "proe" "shg"  "sht"  "sphn" "mup"  "muc"  "mgot" "muh"  "mgk"
## [2421] "mrub" "mgos" "agd"  "oli"  "sbx"  "cmr"  "camu" "bbd"  "evi"  "est"
## [2431] "echi" "alm"  "dfe"  "sli"  "srd"  "smon" "spir" "spik" "spib" "rsi"
## [2441] "run"  "rup"  "eol"  "fae"  "fib"  "psez" "als"  "rhoz" "hsw"  "hyd"
## [2451] "hyg"  "hyp"  "hyz"  "hnv"  "hyh"  "hyj"  "hqi"  "hrs"  "pko"  "pact"
## [2461] "ruf"  "rti"  "rud"  "nib"  "add"  "aswu" "mtt"  "fpf"  "flm"  "fll"
```

```
## [2471] "fbt"  "chk"  "gfl"  "grs"  "fjo"  "ffa"  "marm" "mart" "marb" "cao"
## [2481] "cbal" "cbat" "ptq"  "ndo"  "pom"  "pob"  "prn"  "pola" "poa"  "phal"
## [2491] "win"  "salt" "aalg" "kan"  "aqb"  "aqa"  "afla" "gaa"  "fbe"  "nde"
## [2501] "nja"
```

Organisms classified within cluster 2

```
##    [1] "sed"  "lpop" "tpty" "pay"  "ptt"  "pmr"  "pmib" "pvl"  "pvg"  "phau"
##   [11] "prot" "pcol" "pcib" "xbo"  "xbv"  "xne"  "xnm"  "xdo"  "xpo"  "psi"
##   [21] "psx"  "psta" "prg"  "pala" "phei" "prq"  "prj"  "pvc"  "mmk"  "ans"
##   [31] "eic"  "etr"  "etd"  "ete"  "etc"  "edw"  "edl"  "eho"  "lpv"  "pfq"
##   [41] "prag" "lri"  "pshi" "gle"  "apl"  "apj"  "apa"  "asi"  "ass"  "aeu"
##   [51] "apor" "aio"  "alig" "gan"  "bto"  "btre" "btrh" "btra" "ooi"  "fcl"
##   [61] "paet" "xoo"  "xoy"  "lyt"  "lue"  "lug"  "vch"  "vcf"  "vcs"  "vce"
##   [71] "vcq"  "vcj"  "vci"  "vco"  "vcr"  "vcm"  "vcl"  "vcx"  "vcz"  "vsp"
##   [81] "van"  "lag"  "vau"  "vtu"  "vbr"  "vsc"  "vga"  "vqi"  "vta"  "vaq"
##   [91] "vsr"  "vfi"  "vfm"  "vsa"  "awd"  "ppr"  "pgb"  "pds"  "gho"  "pmai"
##  [101] "saly" "sks"  "scot" "pbb"  "emo"  "par"  "prw"  "pur"  "psyc" "psya"
##  [111] "psyy" "abm"  "aug"  "mct"  "mcs"  "mcat" "moi"  "mos"  "mbl"  "mboi"
##  [121] "mcun" "mnn"  "smai" "ilo"  "ili"  "ipi"  "idi"  "idt"  "cola" "cov"
##  [131] "lsd"  "psen" "msr"  "msx"  "salh" "salk" "psy"  "fbl"  "fes"  "mya"
##  [141] "maga" "mhyd" "cbu"  "cbs"  "cbd"  "cbg"  "cbc"  "alg"  "asip" "lpn"
##  [151] "lph"  "lpo"  "lpu"  "lpm"  "lpf"  "lpp"  "lpc"  "lpa"  "lpe"  "llo"
##  [161] "lfa"  "lok"  "lsh"  "llg"  "lib"  "lgt"  "ljr"  "lwa"  "lss"  "tmc"
##  [171] "mca"  "metu" "mmt"  "mdn"  "mdh"  "mko"  "metl" "mah"  "mbur" "mpsy"
##  [181] "mmob" "mein" "ftq"  "ftl"  "ftc"  "ftv"  "ftz"  "ftd"  "fty"  "fcf"
##  [191] "fcn"  "fhi"  "fph"  "fpt"  "fpi"  "fpm"  "fpx"  "fpz"  "fpj"  "frt"
##  [201] "fna"  "frf"  "fha"  "frx"  "frc"  "fad"  "fmi"  "htr"  "mej"  "mec"
##  [211] "cyy"  "psal" "tig"  "blep" "this" "noc"  "nwa"  "nwr"  "alv"  "tvi"
##  [221] "tmb"  "mpur" "tsy"  "rhh"  "ntg"  "thip" "aeh"  "hhc"  "ebs"  "tgr"
##  [231] "tkm"  "tni"  "tti"  "tvr"  "aprs" "hna"  "haz"  "woc"  "ttc"  "hag"
##  [241] "zpl"  "kus"  "kma"  "kuy"  "paur" "kko"  "kge"  "ksd"  "kpd"  "tol"
##  [251] "tor"  "bmar" "llp"  "asa"  "aeo"  "amed" "asr"  "acav" "aem"  "aea"
```

```
## [261] "tau"  "ocm"  "opf"  "orb"  "sdf"  "slim" "sva"  "acii" "tbn"  "seds"
## [271] "tsn"  "enm"  "nms"  "nmt"  "nmi"  "nel"  "nsi"  "nmj"  "nei"  "nzl"
## [281] "naq"  "nbl"  "nzo"  "nci"  "nani" "vit"  "ecor" "aff"  "cste" "nba"
## [291] "lhk"  "pnu"  "pne"  "pdq"  "poh"  "lmir" "phn"  "our"  "oto"  "cbaa"
## [301] "cbab" "upi"  "tin"  "bbag" "neu"  "net"  "nit"  "nii"  "nco"  "nur"
## [311] "nst"  "nmu"  "nlc"  "tbd"  "mfa"  "mmb"  "meh"  "mei"  "mep"  "mbac"
## [321] "meu"  "slt"  "gca"  "fam"  "nim"  "sdr"  "sulf" "splb" "sniv" "slac"
## [331] "rbh"  "tak"  "zpa"  "fmy"  "bprc" "crx"  "acib" "afc"  "ant"  "aell"
## [341] "aaqi" "asui" "aclo" "aana" "avp"  "adz"  "alp"  "amyt" "amar" "amol"
## [351] "hebr" "paco" "arc"  "smul" "shal" "suls" "sulj" "sult" "gme"  "gur"
## [361] "gbm"  "geo"  "gem"  "geb"  "gbn"  "pca"  "pef"  "ppd"  "des"  "deu"
## [371] "dpg"  "dms"  "dsd"  "dhy"  "dpr"  "dog"  "dsf"  "dol"  "dml"  "dal"
## [381] "dat"  "dto"  "dov"  "ade"  "acp"  "afw"  "ank"  "vin"  "sfu"  "dax"
## [391] "dbr"  "dav"  "bsed" "rpod" "bme"  "bmel" "bmg"  "bmw"  "bmf"  "bmb"
## [401] "bmc"  "baa"  "babo" "babr" "babt" "babb" "babu" "babs" "babc" "bsui"
## [411] "bsup" "bsuv" "bsuc" "bmt"  "bsz"  "bsg"  "bov"  "bpp"  "bcet" "bcee"
## [421] "bvl"  "bru"  "brj"  "nwi"  "nha"  "bapi" "mlg"  "rhj"  "hni"  "rva"
## [431] "fil"  "fiy"  "bvr"  "blag" "mmyr" "mcg"  "metg" "aala" "psf"  "brd"
## [441] "bne"  "brg"  "bdm"  "brf"  "bmed" "rsp"  "rsh"  "rsq"  "rsk"  "rcp"
## [451] "rhp"  "rbl"  "rde"  "rli"  "pcon" "paru" "pamn" "pmut" "pars" "kvl"
## [461] "kvu"  "kro"  "oar"  "otm"  "oct"  "red"  "ptp"  "cmar" "sulz" "don"
## [471] "rid"  "rom"  "roh"  "lvs"  "sedi" "boo"  "paed" "pamo" "rbz"  "mmr"
## [481] "hyt"  "sphl" "slut" "srhi" "efv"  "gox"  "goh"  "goy"  "gal"  "gti"
## [491] "gbe"  "gdj"  "gxy"  "gxl"  "kna"  "keu"  "kre"  "apt"  "apw"  "asv"
## [501] "aace" "aper" "apom" "aasc" "acet" "aot"  "aoy"  "abg"  "kba"  "nch"
## [511] "coq"  "comm" "ntn"  "ssam" "rrf"  "rpm"  "ahu"  "hjo"  "dex"  "dvn"
## [521] "pbr"  "mgm"  "pel"  "mai"  "man"  "apb"  "bba"  "bbat" "bbw"  "bbac"
## [531] "bex"  "bdq"  "bdc"  "bmx"  "hax"  "bsto" "afr"  "afe"  "acz"  "afi"
## [541] "afj"  "maes" "mfn"  "htl"  "bsu"  "bsr"  "bsl"  "bsh"  "bsy"  "bsut"
## [551] "bsul" "bsus" "bso"  "bsn"  "bsq"  "bsx"  "bsp"  "bss"  "bst"  "bli"
## [561] "bld"  "blh"  "bay"  "baq"  "bya"  "bamp" "baml" "bama" "bamn" "bamb"
## [571] "bamt" "bamy" "bmp"  "bao"  "baz"  "bql"  "bxh"  "bqy"  "bami" "bamc"
## [581] "bamf" "bsia" "bae"  "bvm"  "bson" "bht"  "ban"  "bar"  "bat"  "bah"
## [591] "bai"  "bax"  "bant" "banr" "bans" "banh" "banv" "bce"  "bca"  "bcz"
```

```
## [601] "bcr"  "bcb"  "bcu"  "bcg"  "bcq"  "bcx"  "bal"  "bnc"  "bcf"  "bcer"
## [611] "bcef" "bcy"  "btk"  "btl"  "btb"  "btt"  "bthr" "bthi" "btc"  "btf"
## [621] "btm"  "btg"  "bti"  "btn"  "btht" "bthu" "btw"  "bthy" "bwe"  "bww"
## [631] "bmyo" "bty"  "bmyc" "bby"  "btro" "bpu"  "bpum" "bpus" "bco"  "bjs"
## [641] "bif"  "bmet" "gst"  "bacw" "bacp" "bacb" "baco" "bacy" "bacl" "balm"
## [651] "bsm"  "bgy"  "bwh"  "bxi"  "bhk"  "bbev" "balt" "bacs" "bsaf" "bit"
## [661] "bacq" "bcir" "bfd"  "bcoh" "bda"  "bmq"  "bmh"  "bfx"  "bck"  "bag"
## [671] "bcoa" "bha"  "bcl"  "bpf"  "ble"  "bkw"  "bgi"  "bon"  "oih"  "ocn"
## [681] "gka"  "gte"  "gtk"  "gtm"  "gli"  "gtn"  "gyc"  "gya"  "gct"  "gmc"
## [691] "ggh"  "gjf"  "gel"  "gse"  "gsr"  "gej"  "gth"  "ptl"  "ptb"  "afl"
## [701] "agn"  "anm"  "aamy" "anl"  "and"  "acai" "axl"  "lsp"  "lgy"  "lfu"
## [711] "lys"  "lyb"  "lyz"  "lyg"  "lpak" "hhd"  "hmn"  "hli"  "tap"  "vir"
## [721] "vhl"  "vig"  "vil"  "vne"  "vpn"  "vim"  "lao"  "fpn"  "far"  "sje"
## [731] "apak" "bsj"  "pasa" "aqt"  "bthv" "psyh" "psyo" "prd"  "grc"  "rue"
## [741] "sale" "pof"  "nmk"  "ntm"  "aia"  "blen" "stea" "bse"  "sau"  "sav"
## [751] "saw"  "sah"  "saj"  "sam"  "sas"  "sar"  "sac"  "sax"  "saa"  "sao"
## [761] "sae"  "sad"  "suu"  "suv"  "sue"  "suj"  "suk"  "suc"  "sut"  "suq"
## [771] "suz"  "sud"  "sux"  "suw"  "sug"  "suf"  "saua" "saue" "saun" "saus"
## [781] "sauu" "saug" "sauz" "saut" "sauj" "sauk" "sauq" "sauv" "sauw" "saux"
## [791] "sauy" "sauf" "sab"  "suy"  "saub" "saum" "sauc" "saur" "saui" "saud"
## [801] "sams" "suh"  "ssp"  "sca"  "slg"  "sln"  "ssd"  "sdt"  "sdp"  "sxy"
## [811] "sxl"  "sxo"  "shu"  "sagq" "seqo" "scv"  "slz"  "snl"  "skl"  "sarl"
## [821] "spic" "ssh"  "ssim" "sscu" "sff"  "sste" "mlen" "shv"  "lmo"  "lmn"
## [831] "lmy"  "lmt"  "lmoc" "lmoe" "lmob" "lmod" "lmow" "lmoq" "lmr"  "lmom"
## [841] "lmg"  "lms"  "lmj"  "lmw"  "lmx"  "lmz"  "lmos" "lmoy" "lmot" "lsg"
## [851] "lia"  "lio"  "esi"  "eat"  "ean"  "exm"  "exu"  "bbe"  "blr"  "bfm"
## [861] "bagr" "brw"  "ppy"  "ppm"  "ppo"  "ppol" "ppq"  "ppoy" "pta"  "plv"
## [871] "psab" "pdu"  "pbd"  "pgm"  "pod"  "paen" "paef" "paeq" "pste" "paea"
## [881] "paee" "paeh" "paej" "pbj"  "pih"  "pri"  "ppeo" "pnp"  "pow"  "pbv"
## [891] "pxl"  "pyg"  "pswu" "pdh"  "pib"  "pcx"  "pkb"  "paih" "plen" "plut"
## [901] "pchi" "pprt" "pbac" "prz"  "plyc" "anx"  "coh"  "cohn" "saca" "kyr"
## [911] "tum"  "tab"  "siv"  "sob"  "pln"  "prt"  "pll"  "pana" "pdg"  "phc"
## [921] "ppla" "plx"  "pmat" "pdec" "jeo"  "kur"  "kzo"  "spsy" "spor" "spop"
## [931] "sure" "spos" "spae" "rst"  "paek" "panc" "pgq"  "play" "vij"  "ntr"
```

```
##  [941] "lfb"  "tvu"  "kpul" "keb"  "llk"  "llx"  "llj"  "llm"  "lln"  "llw"
##  [951] "lpk"  "lrn"  "sdg"  "sda"  "sdc"  "sdq"  "sik"  "siq"  "sio"  "siz"
##  [961] "lca"  "lcz"  "lcs"  "lce"  "lcw"  "lcl"  "lpap" "lrh"  "lrg"  "lrl"
##  [971] "lra"  "lro"  "lrc"  "lpl"  "lpj"  "lpz"  "lpb"  "lbh"  "lbn"  "lpar"
##  [981] "lcu"  "lkf"  "lhil" "lpd"  "lros" "oen"  "lpse" "efc"  "efu"  "ecas"
##  [991] "emu"  "ega"  "ess"  "egv"  "eav"  "esg"  "vte"  "avs"  "crn"  "cml"
## [1001] "carn" "jep"  "jeh"  "jar"  "jpo"  "cbt"  "cbe"  "cbz"  "cbei" "ccb"
## [1011] "cls"  "clb"  "csr"  "cpas" "csb"  "csq"  "cck"  "cbut" "cdrk" "cdy"
## [1021] "gfe"  "ccl"  "ruk"  "cce"  "fpla" "ral"  "cle"  "cew"  "cct"  "byl"
## [1031] "bpro" "lacy" "csh"  "anr"  "acel" "cbol" "sth"  "dsy"  "dhd"  "ddh"
## [1041] "dku"  "dgi"  "dor"  "dai"  "dmi"  "tfr"  "elm"  "emt"  "tmr"  "thef"
## [1051] "thep" "say"  "sap"  "sthr" "ibu"  "abut" "puf"  "pft"  "sted" "lpil"
## [1061] "mle"  "mlb"  "cgl"  "cgb"  "cgu"  "cgt"  "cgs"  "cgg"  "cgm"  "cgj"
## [1071] "cgq"  "cgx"  "cdi"  "cdp"  "cdh"  "cdt"  "cde"  "cdr"  "cda"  "cdz"
## [1081] "cdb"  "cds"  "cdd"  "cdw"  "cdv"  "cdip" "cjk"  "cur"  "cua"  "car"
## [1091] "ckp"  "cpl"  "cpg"  "cpp"  "cpk"  "cpq"  "cpx"  "cpz"  "cor"  "cop"
## [1101] "cod"  "cos"  "coi"  "coe"  "cou"  "cpse" "cpsu" "cpsf" "crd"  "cul"
## [1111] "cuc"  "cue"  "cun"  "cus"  "cuq"  "cuz"  "cuj"  "ccn"  "cter" "cmd"
## [1121] "caz"  "cfn"  "ccg"  "cvt"  "cii"  "coa"  "cdo"  "chm"  "csx"  "ccj"
## [1131] "cmv"  "cei"  "cted" "cdx"  "csp"  "csta" "ccjz" "cpho" "cfc"  "cgv"
## [1141] "cstr" "caqu" "csph" "camg" "cmin" "cpeg" "cxe"  "cee"  "csan" "cgk"
## [1151] "crf"  "crl"  "ccho" "cpso" "csur" "bfv"  "lxl"  "lxx"  "lxy"  "mix"
## [1161] "maur" "mfol" "rla"  "rpla" "aqg"  "rtx"  "rtc"  "cug"  "cqf"  "malk"
## [1171] "lyk"  "leu"  "leb"  "krh"  "kii"  "kod"  "kvr"  "mlu"  "rter" "rama"
## [1181] "rkr"  "aul"  "dva"  "djj"  "jde"  "kse"  "dni"  "day"  "xce"  "iva"
## [1191] "xya"  "ske"  "sanw" "cfl"  "cfi"  "cga"  "cez"  "celz" "cej"  "oek"
## [1201] "ars"  "teh"  "bly"  "bri"  "blut" "dco"  "gez"  "pac"  "paus" "ppc"
## [1211] "pbo"  "aaci" "acij" "aji"  "tfl"  "tfa"  "tez"  "tdf"  "tla"  "noi"
## [1221] "ndp"  "tfu"  "ace"  "nak"  "tbw"  "ard"  "ahw"  "asla" "fsl"  "flh"
## [1231] "plan" "plak" "plim" "psuf" "pvs"  "pvn"  "rrd"  "afo"  "erz"  "syn"
## [1241] "syz"  "syy"  "syt"  "sys"  "syq"  "syj"  "syo"  "syc"  "syf"  "syw"
## [1251] "syd"  "syg"  "syr"  "syx"  "syp"  "syne" "synp" "synk" "synr" "synd"
## [1261] "syu"  "synw" "slw"  "syv"  "syl"  "sync" "tel"  "thn"  "tvn"  "thec"
## [1271] "cgc"  "cyi"  "dsl"  "cmp"  "lep"  "let"  "hhg"  "pseu" "pser" "pmt"
```

```
## [1281] "pmf"  "theu" "gen"  "gee"  "chon" "mar"  "mpk"  "miq"  "can"  "csn" 
## [1291] "cyl"  "hao"  "enn"  "cwa"  "cyc"  "arp"  "pagh" "oxy"  "lfs"  "gei" 
## [1301] "oac"  "cep"  "gvi"  "glj"  "ana"  "npu"  "ava"  "anb"  "acy"  "awa" 
## [1311] "ann"  "csg"  "dou"  "dfs"  "ccur" "scs"  "stan" "ceo"  "cer"  "cag" 
## [1321] "tro"  "sti"  "atm"  "tbh"  "ttr"  "dra"  "dge"  "dmr"  "dpt"  "dgo" 
## [1331] "dpd"  "dsw"  "dch"  "dab"  "dpu"  "dwu"  "dfc"  "dga"  "tth"  "ttj" 
## [1341] "tts"  "ttl"  "tsc"  "thc"  "tos"  "tbc"  "mrb"  "mre"  "msv"  "mtai"
## [1351] "mhd"  "ccz"  "fgi"  "puv"  "ote"  "obg"  "vbh"  "obt"  "amu"  "min" 
## [1361] "mkc"  "meap" "vba"  "vbs"  "pir"  "rul"  "mff"  "bvo"  "ttf"  "plm" 
## [1371] "peh"  "plh"  "fmr"  "ipa"  "pcor" "vbc"  "taz"  "tpi"  "tphg" "trc" 
## [1381] "ssm"  "sbu"  "sgp"  "lil"  "lie"  "lic"  "lis"  "lbj"  "lbl"  "lbi" 
## [1391] "lbf"  "lst"  "lmay" "lwl"  "bip"  "thyd" "fva"  "ful"  "fmo"  "lgo" 
## [1401] "cpor" "fsu"  "fsc"  "bfr"  "pet"  "dys"  "ald"  "ait"  "dori" "mbas"
## [1411] "rbar" "nso"  "nia"  "fgg"  "arb"  "ark"  "agi"  "arac" "fls"  "sgn" 
## [1421] "pgs"  "pej"  "psn"  "smiz" "spsc" "sphz" "sphe" "spdr" "sdj"  "stha"
## [1431] "scn"  "mgin" "chu"  "lby"  "fli"  "hym"  "hye"  "gfo"  "grl"  "fps" 
## [1441] "fpc"  "fpy"  "fpo"  "fpq"  "fpv"  "fpw"  "fpk"  "fpsz" "fjg"  "fbr" 
## [1451] "fco"  "fin"  "fgl"  "fcm"  "fat"  "fki"  "fpal" "fmg"  "falb" "fcr" 
## [1461] "fse"  "fsn"  "fnk"  "fak"  "capq" "rbi"  "zpr"  "cat"  "fbc"  "mare"
## [1471] "cly"  "clh"  "kdi"  "dok"  "ddo"  "dod"  "lan"  "lvn"  "laci" "zga" 
## [1481] "mrs"  "mlt"  "mut"  "asl"  "aev"  "nom"  "nsd"  "nob"  "noj"  "myr" 
## [1491] "mpw"  "mod"  "myz"  "wij"  "sze"  "ahz"  "syi"  "tdi"  "ten"  "tje" 
## [1501] "tmar" "tmp"  "lut"  "lul"  "wfu"  "for"  "foh"  "fop"  "seon" "oll" 
## [1511] "oaq"  "fek"  "taj"  "aue"  "spon" "kos"  "marf" "aqd"  "emar" "mur" 
## [1521] "psyn" "anp"  "oci"  "mgel" "mesq" "cagg" "alti" "fba"  "fbu"  "rag" 
## [1531] "rat"  "wvi"  "eao"  "emn"  "een"  "elb"  "emg"  "ego"  "egm"  "elz" 
## [1541] "elt"  "chz"  "cgn"  "cih"  "chh"  "cio"  "chry" "cpip" "chrs" "chrz"
## [1551] "carh" "csha" "cnk"  "cjt"  "cil"  "ccau" "cben" "cjg"  "ccas" "cant"
## [1561] "kda"  "clac" "eva"  "ctak" "cnr"  "ebv"  "efal" "este" "fte"  "flu" 
## [1571] "oho"  "bbau" "ial"  "cprv" "caby" "nmv"  "nio"  "lfc"  "lfi"  "lfp" 
## [1581] "leg"  "nli"  "mox"  "mac"  "mhor" "hdl"  "hhb"  "hje"  "salr" "hma" 
## [1591] "hhi"  "hhn"  "hab"  "hta"  "halj" "hmu"  "halz" "hall" "hali" "hsn" 
## [1601] "hrr"  "hpel" "hlt"  "hvo"  "hgi"  "hale" "hbo"  "haq"  "haj"  "haer"
## [1611] "hra"  "hlm"  "hla"  "halb" "hezz" "halq" "srub" "hae"  "haln" "halg"
```

```
## [1621] "halu" "hdf"  "hah"  "htu"  "hda"  "hjt"  "haly" "nmg"  "hxa"  "nat"
## [1631] "npe"  "nvr"  "npl"  "nge"  "hru"  "nou"  "sali" "hlr"  "naj"  "nag"
## [1641] "nan"  "nbg"  "nas"  "nax"  "pto"  "cdiv" "sto"  "soh"  "sso"  "sol"
## [1651] "ssoa" "ssol" "ssof" "sai"  "sacn" "sacr" "sacs" "sis"  "sia"  "sim"
## [1661] "sid"  "siy"  "sin"  "sii"  "sih"  "sir"  "sic"  "sula" "sule" "mse"
## [1671] "mpru" "aman" "abri" "sacd" "pas"  "pyr"  "pog"  "cma"  "vdi"  "vmo"
## [1681] "nfn"  "loki" "psyt"
```

Organisms classified within cluster 3

```
##   [1] "shx"  "ath"  "aly"  "crb"  "csat" "eus"  "brp"  "bna"  "boe"  "rsz"
##  [11] "thj"  "cpap" "cit"  "cic"  "pvy"  "minc" "tcc"  "gra"  "ghi"  "gab"
##  [21] "dzi"  "egr"  "gmx"  "gsj"  "pvu"  "vra"  "var"  "vun"  "ccaj" "aprc"
##  [31] "mtr"  "cam"  "lja"  "adu"  "aip"  "ahf"  "lang" "fve"  "rcn"  "pper"
##  [41] "pmum" "pavi" "pdul" "mdm"  "pxb"  "zju"  "mnt"  "csv"  "cmo"  "bhj"
##  [51] "mcha" "cmax" "cmos" "cpep" "rcu"  "jcu"  "hbr"  "mesc" "pop"  "peu"
##  [61] "palz" "jre"  "qsu"  "qlo"  "twl"  "vvi"  "vri"  "sly"  "spen" "sot"
##  [71] "cann" "nta"  "nsy"  "nto"  "nau"  "ini"  "itr"  "sind" "oeu"  "egt"
##  [81] "sspl" "han"  "ecad" "lsv"  "ccav" "dcr"  "csin" "bvg"  "soe"  "cqi"
##  [91] "nnu"  "ming" "psom" "ncol" "osa"  "dosa" "obr"  "bdi"  "ats"  "tdc"
## [101] "sbi"  "zma"  "sita" "pvir" "phai" "pda"  "egu"  "mus"  "dct"  "peq"
## [111] "aof"  "atr"  "smo"  "ppp"  "cre"  "vcn"  "mng"  "csl"  "cvr"  "apro"
## [121] "olu"  "ota"  "bpg"  "mis"  "mpp"  "cme"  "gsl"  "ccp"  "sce"  "ago"
## [131] "erc"  "kla"  "kmx"  "lth"  "vpo"  "zro"  "cgr"  "ncs"  "ndi"  "tpf"
## [141] "tbl"  "tdl"  "tgb"  "kaf"  "zmk"  "ppa"  "dha"  "pic"  "pgu"  "spaa"
## [151] "lel"  "cal"  "ctp"  "cot"  "cdu"  "cten" "yli"  "clu"  "clus" "caur"
## [161] "slb"  "pkz"  "bnn"  "bbrx" "ncr"  "nte"  "smp"  "pan"  "ttt"  "mtm"
## [171] "cthr" "mgr"  "tmn"  "ssck" "fgr"  "fpu"  "fvr"  "fox"  "nhe"  "tre"
## [181] "trr"  "maw"  "maj"  "cmt"  "plj"  "val"  "vda"  "cfj"  "sapo" "ela"
## [191] "pfy"  "ssl"  "bfu"  "mbe"  "psco" "glz"  "ani"  "afm"  "act"  "nfi"
## [201] "aor"  "ang"  "afv"  "pcs"  "pdp"  "tmf"  "trg"  "cim"  "cpw"  "ure"
## [211] "pbl"  "pbn"  "abe"  "tve"  "aje"  "bgh"  "pno"  "pte"  "bze"  "bsc"
## [221] "bor"  "aalt" "ztr"  "pfj"  "bcom" "npa"  "tml"  "spo"  "cne"  "cnb"
```

```
## [231] "cgi"  "tms"  "tasa" "ppl"  "tvs"  "dsq"  "pco"  "shs"  "hir"  "psq"
## [241] "adl"  "fme"  "gtr"  "lbc"  "mpr"  "mrr"  "cci"  "scm"  "abp"  "abv"
## [251] "cput" "sla"  "wse"  "wic"  "uma"  "pfp"  "mgl"  "mrt"  "msym" "pgr"
## [261] "mlr"  "ecu"  "ein"  "ehe"  "ero"  "nce"  "mbr"  "sre"  "ddi"  "dpp"
## [271] "dfa"  "ehi"  "edi"  "eiv"  "acan" "pfa"  "pfh"  "pyo"  "pcb"  "pbe"
## [281] "pkn"  "pvx"  "pcy"  "beq"  "bbo"  "cpv"  "cho"  "tgo"  "tet"  "ptm"
## [291] "smin" "pti"  "fcy"  "tps"  "ngd"  "aaf"  "pif"  "psoj" "spar" "ehx"
## [301] "gtt"  "tbr"  "tbg"  "tcr"  "lma"  "lif"  "ldo"  "lmi"  "lbz"  "lpan"
## [311] "ngr"  "tva"  "gla"
```

Organisms classified within cluster 4

```
##   [1] "pfd"  "tan"  "tpv"  "tot"  "bmic" "bfl"  "bpn"  "bva"  "bchr" "ben"
##  [11] "bed"  "hde"  "sect" "sehc" "senm" "rip"  "rig"  "men"  "meo"  "icp"
##  [21] "sbw"  "den"  "hed"  "ged"  "cmik" "ppet" "ssz"  "seny" "ehd"  "buc"
##  [31] "bap"  "bau"  "baw"  "bajc" "bua"  "bup"  "bak"  "buh"  "bapf" "bapg"
##  [41] "bapu" "bapw" "bas"  "bab"  "bcc"  "baj"  "baph" "wbr"  "wgl"  "hhs"
##  [51] "asy"  "aen"  "hdu"  "cey"  "cea"  "cend" "les"  "ple"  "ply"  "plr"
##  [61] "plo"  "pld"  "plb"  "plc"  "pli"  "paly" "crp"  "cru"  "crc"  "crt"
##  [71] "crh"  "crv"  "cri"  "eme"  "dno"  "eof"  "bci"  "bcib" "bcig" "zin"
##  [81] "tpj"  "kbl"  "kbt"  "kga"  "kon"  "kso"  "ssdc" "ndl"  "vfg"  "hce"
##  [91] "hcm"  "het"  "hcl"  "lip"  "lir"  "rcm"  "rcc"  "rbo"  "rco"  "rri"
## [101] "rrj"  "rra"  "rrc"  "rrh"  "rrb"  "rrn"  "rrp"  "rrm"  "rrr"  "rms"
## [111] "rmi"  "rpk"  "raf"  "rhe"  "rja"  "rsv"  "rsw"  "rph"  "rmo"  "rpp"
## [121] "rre"  "rmc"  "ras"  "ots"  "ott"  "ptc"  "wol"  "wri"  "wen"  "wed"
## [131] "wpi"  "wbm"  "woo"  "wcl"  "weo"  "wpp"  "ama"  "amf"  "amw"  "amp"
## [141] "acn"  "aph"  "apy"  "apd"  "apha" "aoh"  "eru"  "erw"  "erg"  "ecn"
## [151] "ech"  "echa" "echj" "echl" "echs" "echv" "echw" "echp" "emr"  "ehh"
## [161] "nse"  "nri"  "nhm"  "nef"  "mmn"  "fso"  "eaa"  "las"  "laa"  "lat"
## [171] "lso"  "lar"  "lau"  "hci"  "hct"  "hcc"  "hcd"  "ldb"  "ldl"  "lhe"
## [181] "law"  "lje"  "lapi" "lhs"  "lsn"  "lah"  "asf"  "asm"  "aso"  "asb"
## [191] "clo"  "fsa"  "pbq"  "mdv"  "bprm" "pmic" "eri"  "mge"  "mgu"  "mgc"
## [201] "mgq"  "mgx"  "mpn"  "mpm"  "mpj"  "mpb"  "mpe"  "mga"  "mgh"  "mgf"
```

```
## [211] "mgn"  "mgs"  "mgt"  "mgv"  "mgw"  "mgac" "mgan" "mgnc" "mgz"  "mmy" 
## [221] "mmym" "mmyi" "mml"  "mcp"  "mcac" "mcap" "mcar" "mcai" "mlc"  "mlh" 
## [231] "mmo"  "mhy"  "mhj"  "mhp"  "mhn"  "mhyl" "mhyo" "mat"  "mco"  "mho" 
## [241] "mhom" "mcd"  "mhr"  "mhh"  "mhm"  "mhs"  "mhv"  "mha"  "mhf"  "mss" 
## [251] "msk"  "mpf"  "mput" "mhe"  "mwe"  "mhl"  "mhb"  "mpv"  "mov"  "mbc" 
## [261] "mgj"  "mfq"  "mcan" "myt"  "mds"  "myg"  "mpho" "mhyv" "mclo" "mamp"
## [271] "mans" "mphc" "miw"  "mane" "mnh"  "mnu"  "mstr" "mcr"  "mcm"  "mgb" 
## [281] "mgly" "mcou" "mcom" "mpu"  "msy"  "mso"  "maa"  "mal"  "mfr"  "mfm" 
## [291] "mfp"  "mbv"  "mbh"  "mbi"  "mbq"  "mcy"  "mcas" "mck"  "marg" "mpul"
## [301] "mbov" "mboh" "mani" "mphi" "uur"  "upa"  "upr"  "uue"  "hcr"  "poy" 
## [311] "ayw"  "mbp"  "pml"  "pal"  "nzs"  "psol" "pzi"  "mfl"  "mfw"  "mchc"
## [321] "mlac" "ment" "msyr" "mtab" "mcol" "elj"  "esx"  "efr"  "eml"  "scr" 
## [331] "ssyr" "sdi"  "stai" "sapi" "smir" "smia" "scq"  "ssab" "satr" "seri"
## [341] "stur" "sll"  "skn"  "scj"  "shj"  "sck"  "sfz"  "scou" "scla" "sprn"
## [351] "spit" "stab" "sphh" "smoo" "salx" "sgq"  "schi" "twh"  "tws"  "ahe" 
## [361] "ctr"  "ctd"  "ctf"  "ctrd" "ctro" "ctrt" "cta"  "cty"  "cra"  "ctrq"
## [371] "ctrx" "ctrz" "ctrp" "ctlj" "ctlx" "ctll" "ctb"  "ctrr" "ctlf" "ctli"
## [381] "ctl"  "ctru" "ctrl" "ctrv" "ctrm" "ctla" "ctlm" "ctls" "ctlz" "ctlc"
## [391] "ctln" "ctlb" "ctlq" "cto"  "ctrn" "ctj"  "ctz"  "ctg"  "ctk"  "csw" 
## [401] "ces"  "ctrb" "ctre" "ctrs" "ctec" "cfs"  "cfw"  "ctfw" "ctrf" "ctch"
## [411] "ctn"  "ctq"  "ctv"  "ctw"  "ctrg" "ctri" "ctra" "ctrh" "ctrj" "ctrk"
## [421] "ctjt" "ctcf" "ctfs" "cthf" "ctcj" "cthj" "ctmj" "cttj" "ctjs" "ctrc"
## [431] "ctrw" "ctry" "ctct" "cmu"  "cmur" "cmn"  "cmm"  "cmg"  "cmx"  "cmz" 
## [441] "cpn"  "cpa"  "cpj"  "cpt"  "clp"  "cpm"  "cpec" "cpeo" "cper" "chp" 
## [451] "chb"  "chs"  "chi"  "cht"  "chc"  "chr"  "cpsc" "cpsn" "cpsb" "cpsg"
## [461] "cpsm" "cpsi" "cpsv" "cpsw" "cpst" "cpsd" "cpsa" "cav"  "cca"  "cab" 
## [471] "cabo" "cfe"  "cgz"  "chla" "pcu"  "ney"  "psup" "bbu"  "bbz"  "bbn" 
## [481] "bbj"  "bbur" "bga"  "bgb"  "bgn"  "bgs"  "bgc"  "baf"  "bafz" "bafh"
## [491] "baft" "bafe" "bbs"  "bvt"  "bchi" "bmay" "btu"  "bhr"  "bhi"  "bdu" 
## [501] "bre"  "bcw"  "bmo"  "bmiy" "bpak" "bane" "btur" "bmat" "tpa"  "tpw" 
## [511] "tpp"  "tpu"  "tph"  "tpo"  "tpas" "tpc"  "tpg"  "tpm"  "tpb"  "tpl" 
## [521] "sns"  "sbr"  "aas"  "che"  "cec"  "cher" "smg"  "sms"  "smh"  "sum" 
## [531] "smv"  "smub" "smum" "smue" "smup" "bbl"  "bpi"  "bmm"  "bcp"  "bbg" 
## [541] "bbq"  "blp"  "blu"  "blck" "elv"  "udi"  "cex"  "saal" "sbe"  "sbag"
```

```
## [551] "sox"  "bih"  "srb"  "srg"  "caqa" "dpb"  "tmg"  "bgw"  "bbgw" "mib" 
## [561] "wba"  "pwo"  "cgw"  "baab" "the"  "thm"  "tgg"  "thy"  "nac"  "dfd" 
## [571] "thf"  "ffo"  "neq"  "naa"  "marh" "flt"  "agw"
```

Organisms classified within cluster 5

```
##   [1] "fsm"  "sgl"  "pes"  "pck"  "hin"  "hit"  "hip"  "hiq"  "hif"  "hil" 
##  [11] "hiu"  "hie"  "hiz"  "hik"  "hia"  "hih"  "hiw"  "hic"  "hix"  "hpr" 
##  [21] "hay"  "hpit" "hhz"  "haeg" "hpaa" "hap"  "hpaz" "hpas" "hpak" "hso" 
##  [31] "hsm"  "pmu"  "pmv"  "pul"  "pmp"  "pmul" "pdag" "psky" "msu"  "bsun"
##  [41] "mht"  "mhq"  "mhat" "mhx"  "mhae" "mham" "mhao" "mhal" "mhaq" "mhay"
##  [51] "mvr"  "mvi"  "mvg"  "mve"  "mann" "mgra" "asu"  "adp"  "aap"  "aaz" 
##  [61] "aat"  "aao"  "aan"  "aah"  "aacn" "aact" "aseg" "apag" "avt"  "rpne"
##  [71] "rhey" "bhud" "xfa"  "xft"  "xfm"  "xfn"  "xff"  "xfl"  "xfs"  "xfh" 
##  [81] "xtw"  "pade" "rvi"  "ftu"  "ftf"  "ftw"  "ftr"  "ftt"  "ftg"  "fth" 
##  [91] "fta"  "fts"  "fti"  "fto"  "ftm"  "ftn"  "ftx"  "fnl"  "fper" "frm" 
## [101] "foo"  "fgu"  "afri" "aii"  "tcx"  "hmar" "tcy"  "tao"  "thio" "thig"
## [111] "tse"  "tzo"  "ntt"  "ttp"  "hha"  "hhk"  "ssal" "spiu" "sros" "spiz"
## [121] "ghl"  "chj"  "gap"  "fpp"  "thin" "tho"  "rev"  "rma"  "reo"  "vok" 
## [131] "ebh"  "nme"  "nmp"  "nmh"  "nmd"  "nmm"  "nmq"  "nmz"  "nma"  "nmw" 
## [141] "nmx"  "nmc"  "nmn"  "ngo"  "ngk"  "nla"  "nwe"  "nek"  "nfv"  "nsf" 
## [151] "ncz"  "nbc"  "salv" "kki"  "koa"  "eex"  "smur" "mcys" "teq"  "tea" 
## [161] "teg"  "tas"  "tat"  "bpsi" "cbx"  "ofo"  "sutt" "sutk" "bbay" "mbat"
## [171] "fpho" "tpn"  "tpq"  "kci"  "kct"  "kde"  "hpy"  "heo"  "hpj"  "hpa" 
## [181] "hps"  "hhp"  "hhq"  "hhr"  "hpg"  "hpp"  "hpb"  "hpl"  "hpc"  "hca" 
## [191] "hpm"  "hpe"  "hpo"  "hpi"  "hpq"  "hpw"  "hpu"  "hef"  "hpf"  "heq" 
## [201] "hex"  "hpt"  "hpz"  "hpv"  "hpx"  "hen"  "hph"  "heg"  "hpn"  "hep" 
## [211] "heu"  "hes"  "hpys" "hcn"  "hpd"  "hey"  "her"  "hei"  "hpya" "hpyk"
## [221] "hpyo" "hpyl" "hpyb" "hpyc" "hpyd" "hpye" "hpyf" "hpyg" "hpyh" "hpyj"
## [231] "hpyr" "hpyi" "hpyu" "hpym" "hem"  "heb"  "hez"  "hhe"  "hac"  "hms" 
## [241] "hfe"  "hbi"  "hcp"  "hcb"  "hhm"  "hty"  "hbl"  "had"  "hwi"  "wsu" 
## [251] "tdn"  "sua"  "suln" "sulg" "sulc" "spal" "sku"  "sulr" "cje"  "cjb" 
## [261] "cjj"  "cju"  "cjn"  "cji"  "cjm"  "cjs"  "cjp"  "cjej" "cjeu" "cjen"
```

```
## [271] "cjei" "cjer" "cjv"  "cjy"  "cjq"  "cjl"  "cjw"  "cjr"  "cjd"  "cjz"
## [281] "cjx"  "cff"  "cft"  "cfv"  "cfx"  "cfz"  "camp" "cfp"  "ccv"  "cha"
## [291] "cco"  "ccoc" "cla"  "clr"  "clm"  "clq"  "cln"  "cll"  "ccol" "ccc"
## [301] "ccq"  "ccf"  "ccy"  "ccoi" "ccof" "ccoo" "caj"  "cis"  "cvo"  "cpel"
## [311] "camr" "csm"  "csf"  "cgra" "cure" "chyo" "chv"  "cspf" "cpin" "ccun"
## [321] "clx"  "cavi" "chw"  "camz" "camy" "coj"  "cux"  "cgeo" "cbla" "ccor"
## [331] "carm" "cmuc" "csho" "abu"  "abt"  "abl"  "ask"  "atp"  "acre" "alan"
## [341] "apoc" "ahs"  "acaa" "apai" "hbv"  "sdl"  "sba"  "hyo"  "nsa"  "sun"
## [351] "slh"  "nis"  "nam"  "nap"  "cmed" "cpaf" "gsu"  "gsk"  "glo"  "gpi"
## [361] "gao"  "gsb"  "pace" "dvu"  "dvl"  "dvm"  "dvg"  "dde"  "dds"  "dma"
## [371] "dgg"  "dfi"  "def"  "dtr"  "dfl"  "dcb"  "dsa"  "daf"  "das"  "dpi"
## [381] "dej"  "pprf" "psel" "ddn"  "dsx"  "dba"  "doa"  "drt"  "dps"  "dak"
## [391] "deo"  "sat"  "dao"  "hmr"  "rpr"  "rpo"  "rpw"  "rpz"  "rpg"  "rps"
## [401] "rpv"  "rpq"  "rpl"  "rpn"  "rty"  "rtt"  "rtb"  "rbe"  "rfe"  "rak"
## [411] "rau"  "ram"  "rab"  "ric"  "rbt"  "ren"  "paca" "caq"  "naf"  "lcc"
## [421] "bhe"  "bhn"  "bhs"  "bqu"  "bqr"  "bbk"  "btr"  "btx"  "bgr"  "bcd"
## [431] "baus" "bvn"  "banc" "bart" "bara" "barw" "barr" "baro" "barj" "bez"
## [441] "barn" "bky"  "bals" "thd"  "sdo"  "zmo"  "zmn"  "zmm"  "zmb"  "zmi"
## [451] "zmc"  "zmr"  "zmp"  "hgn"  "neh"  "swf"  "bob"  "bomb" "thal" "efk"
## [461] "pub"  "peg"  "apc"  "apm"  "acu"  "atx"  "ocb"  "gwc"  "gea"  "ser"
## [471] "sep"  "sepp" "seps" "sha"  "shh"  "swa"  "spas" "scap" "ssch" "sscz"
## [481] "ssif" "spet" "scoh" "sfq"  "shom" "smus" "scar" "schr" "mcl"  "mcak"
## [491] "macr" "sbac" "jea"  "lmf"  "lmc"  "lmog" "lmp"  "lmol" "lmoj" "lmoz"
## [501] "lmox" "lmh"  "lmq"  "lml"  "lmon" "lmoo" "lmoa" "lmok" "lmv"  "lin"
## [511] "lwe"  "liv"  "lii"  "liw"  "lwi"  "lgz"  "bths" "got"  "gmo"  "geq"
## [521] "gsa"  "gha"  "tco"  "lla"  "llt"  "lls"  "lld"  "llc"  "llr"  "lli"
## [531] "lgr"  "lgv"  "lact" "lack" "spy"  "spz"  "spym" "spya" "spm"  "spg"
## [541] "sps"  "sph"  "spi"  "spj"  "spk"  "spf"  "spa"  "spb"  "stg"  "stx"
## [551] "soz"  "stz"  "spyh" "spyo" "spn"  "spd"  "spr"  "spw"  "sjj"  "snv"
## [561] "spx"  "snt"  "snd"  "spnn" "sne"  "spv"  "snc"  "snm"  "spp"  "sni"
## [571] "spng" "snb"  "snp"  "snx"  "snu"  "spne" "spnu" "spnm" "spno" "sag"
## [581] "san"  "sak"  "sgc"  "sags" "sagl" "sagm" "sagi" "sagr" "sagp" "sagc"
## [591] "sagt" "sage" "sagg" "sagn" "smu"  "smc"  "smut" "smj"  "smua" "stc"
## [601] "stl"  "ste"  "stn"  "stu"  "stw"  "sthe" "sths" "ssa"  "ssb"  "ssu"
```

```
## [611] "ssv"  "ssi"  "sss"  "ssf"  "ssw"  "sup"  "ssus" "sst"  "ssuy" "ssk"
## [621] "ssq"  "sui"  "suo"  "srp"  "ssut" "ssui" "sgo"  "sez"  "seq"  "sezo"
## [631] "sequ" "seu"  "sub"  "sds"  "sga"  "sgg"  "sgt"  "smb"  "sor"  "stk"
## [641] "stb"  "scp"  "scf"  "ssr"  "stf"  "stj"  "strs" "ssah" "std"  "smn"
## [651] "sif"  "sie"  "sib"  "siu"  "sang" "sanc" "sans" "scg"  "scon" "scos"
## [661] "soi"  "slu"  "sig"  "sip"  "stv"  "spat" "stra" "strn" "ssob" "srq"
## [671] "seqi" "ski"  "spei" "srat" "sgw"  "splr" "strg" "ljo"  "ljf"  "ljh"
## [681] "ljn"  "lac"  "lad"  "laf"  "lbu"  "lde"  "lga"  "lhl"  "lhr"  "lhv"
## [691] "lhh"  "lhd"  "lcr"  "lam"  "lai"  "lay"  "lke"  "lae"  "lgl"  "lamy"
## [701] "lpw"  "lkl"  "lpq"  "lpi"  "lcb"  "lcx"  "lpt"  "lps"  "lpr"  "lpx"
## [711] "lpg"  "lre"  "lrf"  "lru"  "lrt"  "lrr"  "lfe"  "lfr"  "lff"  "lmu"
## [721] "lor"  "lva"  "lfn"  "lpon" "lng"  "lhw"  "lmal" "lle"  "lfv"  "lbr"
## [731] "lbk"  "lko"  "lzy"  "lsua" "lji"  "lsl"  "lsi"  "lsj"  "lrm"  "lagl"
## [741] "laca" "lani" "lbt"  "lcy"  "lho"  "lol"  "lnn"  "lku"  "lmae" "ppe"
## [751] "ppen" "pce"  "pdm"  "paci" "pio"  "lgn"  "lhi"  "lct"  "lalw" "lali"
## [761] "lfm"  "lzh"  "lft"  "lsa"  "lcv"  "lgm"  "lbm"  "lhb"  "ldx"  "ooe"
## [771] "osi"  "lme"  "lmm"  "lmk"  "lci"  "lki"  "lec"  "lcn"  "lgs"  "lge"
## [781] "llf"  "lgc"  "lsu"  "wko"  "wce"  "wct"  "wci"  "wcb"  "wjo"  "wpa"
## [791] "wcf"  "wso"  "whe"  "wei"  "wdi"  "wvr"  "efa"  "efl"  "efi"  "efd"
## [801] "efs"  "efn"  "efq"  "ene"  "efau" "efm"  "eft"  "ehr"  "edu"  "eth"
## [811] "mps"  "mpx"  "thl"  "tey"  "too"  "tkr"  "vpi"  "vac"  "vao"  "vah"
## [821] "vcp"  "aur"  "aun"  "aui"  "asan" "acg"  "auh"  "adc"  "abae" "caw"
## [831] "carc" "cdj"  "marr" "jda"  "dpm"  "cac"  "cae"  "cay"  "cpe"  "cpf"
## [841] "cpr"  "ctc"  "ctet" "cno"  "cbo"  "cba"  "cbh"  "cby"  "cbl"  "cbk"
## [851] "cbb"  "cbi"  "cbn"  "cbf"  "cbm"  "cbj"  "ckl"  "ckr"  "clj"  "cpat"
## [861] "cpae" "cah"  "clt"  "cbv"  "cld"  "cace" "ctyk" "ceu"  "ctae" "cfm"
## [871] "cchv" "carg" "cia"  "csep" "ccoh" "cfer" "amt"  "aoe"  "hhw"  "cale"
## [881] "crs"  "cazo" "sarj" "cth"  "ctx"  "hsc"  "rbp"  "css"  "csd"  "cthd"
## [891] "esr"  "esu"  "ccel" "eha"  "rch"  "rum"  "rus"  "ruj"  "fpr"  "fpa"
## [901] "fpra" "capr" "ova"  "obj"  "bpb"  "bfi"  "bhu"  "rho"  "rix"  "rim"
## [911] "coo"  "rob"  "bhan" "blau" "blab" "cpy"  "csci" "cso"  "bprl" "arf"
## [921] "acac" "hsd"  "cpro" "lua"  "ehl"  "pxv"  "eel"  "rto"  "rgn"  "ere"
## [931] "ert"  "era"  "lbw"  "cdf"  "pdc"  "cdc"  "cdl"  "pdf"  "eac"  "cst"
## [941] "faa"  "psor" "roc"  "phx"  "swo"  "slp"  "salq" "ddl"  "dmt"  "drm"
```

```
##  [951] "dca"  "dru"  "dfg"  "dae"  "pth"  "dau"  "tjr"  "sgy"  "ded"  "dec"
##  [961] "drs"  "hmo"  "hcv"  "elim" "awo"  "cthm" "cmiu" "amij" "amic" "euu"
##  [971] "bprs" "cbar" "tte"  "tex"  "thx"  "tpd"  "tit"  "tmt"  "tbo"  "twi"
##  [981] "tki"  "chy"  "mta"  "mtho" "mthz" "adg"  "tpz"  "csc"  "ate"  "cob"
##  [991] "chd"  "cow"  "cki"  "ckn"  "clc"  "ccha" "ttm"  "tto"  "txy"  "tsh"
## [1001] "tnr"  "taci" "mas"  "tep"  "tae"  "toc"  "nth"  "hor"  "has"  "hpk"
## [1011] "hals" "aar"  "hhl"  "aft"  "fma"  "apr"  "ped"  "phar" "piv"  "cad"
## [1021] "spoa" "kpar" "vpr"  "vat"  "vrm"  "vdn"  "vnk"  "med"  "mhw"  "meg"
## [1031] "dpn"  "dho"  "ssg"  "sri"  "sele" "selo" "selt" "mhg"  "mfun" "mana"
## [1041] "afn"  "ain"  "pfac" "erh"  "ers"  "erl"  "erd"  "eio"  "euc"  "fro"
## [1051] "aarg" "absi" "ciu"  "erm"  "fit"  "ebm"  "erb"  "tur"  "tsg"  "acl"
## [1061] "abra" "apal" "aoc"  "aaxa" "ahk"  "mbj"  "tbm"  "tbz"  "cax"  "cuv"
## [1071] "cku"  "cut"  "cfk"  "cbq"  "psai" "rmu"  "rdn"  "raj"  "cig"  "pak"
## [1081] "pav"  "pax"  "paz"  "paw"  "pad"  "pcn"  "pacc" "pach" "pacn" "cacn"
## [1091] "pra"  "cgrn" "pfr"  "pfre" "prl"  "pacd" "nfe"  "arca" "mcu"  "tpy"
## [1101] "tpyo" "asg"  "actt" "amy"  "soo"  "acq"  "aos"  "actp" "actc" "acto"
## [1111] "ane"  "actz" "air"  "avc"  "avu"  "wik"  "fvg"  "blo"  "blj"  "bln"
## [1121] "blon" "blf"  "bll"  "blb"  "blm"  "blk"  "blg"  "blz"  "blx"  "bad"
## [1131] "badl" "bado" "bla"  "blc"  "blt"  "bbb"  "bbc"  "bnm"  "blv"  "blw"
## [1141] "bls"  "bani" "banl" "bni"  "banm" "bde"  "bdn"  "bbp"  "bbi"  "bbf"
## [1151] "bbv"  "bbru" "bbre" "bbrv" "bbrj" "bbrc" "bbrn" "bbrs" "bbrd" "bast"
## [1161] "btp"  "bcor" "bka"  "bks"  "bcat" "bpsp" "bii"  "bang" "bpsc" "bsca"
## [1171] "bact" "bcho" "bgx"  "blem" "beu"  "gvg"  "gva"  "gvh"  "sij"  "pdo"
## [1181] "abam" "nhi"  "nab"  "ccu"  "shi"  "ele"  "eyy"  "gpa"  "aeq"  "ddt"
## [1191] "cbac" "apv"  "ols"  "olo"  "pcat" "cgo"  "caer" "sye"  "cya"  "cyb"
## [1201] "syh"  "pma"  "pmm"  "pmn"  "pmi"  "pmb"  "pmc"  "pmg"  "pmh"  "pmj"
## [1211] "pme"  "prc"  "prm"  "cyu"  "naz"  "nsp"  "mbf"  "det"  "deh"  "deb"
## [1221] "dev"  "deg"  "dmc"  "dmd"  "dmg"  "dmx"  "dmy"  "dmz"  "duc"  "dly"
## [1231] "dew"  "dfo"  "abat" "psub" "abao" "taq"  "tpar" "opr"  "pnl"  "wch"
## [1241] "sng"  "caa"  "agl"  "xii"  "kst"  "broc" "phm"  "pbu"  "pbp"  "pbas"
## [1251] "alus" "vbl"  "vai"  "tde"  "tsu"  "tbe"  "tped" "scd"  "tpk"  "trm"
## [1261] "trz"  "sta"  "stq"  "sfc"  "sper" "scc"  "ock"  "bhy"  "bhd"  "brm"
## [1271] "bpo"  "bpj"  "bpip" "bpw"  "bhp"  "emi"  "epo"  "eti"  "rsd"  "fnu"
## [1281] "fnc"  "fnt"  "fus"  "fne"  "fhw"  "fpd"  "fgo"  "fnf"  "fpei" "ipo"
```

```
## [1291] "lba"  "leo"  "lot"  "leq"  "lhf"  "lsz"  "lhg"  "lte"  "lwd"  "str"
## [1301] "smf"  "tai"  "aco"  "tli"  "amo"  "bth"  "btho" "bfs"  "bfg"  "bfb"
## [1311] "bhl"  "bxy"  "boa"  "bcel" "bcac" "bcae" "bzg"  "bhf"  "bis"  "bun"
## [1321] "bvu"  "bsa"  "bdo"  "bdh"  "pgi"  "pgn"  "pgt"  "pah"  "pcre" "pcag"
## [1331] "pbt"  "pmuc" "psac" "ppn"  "pdi"  "parc" "tfo"  "toh"  "pary" "dun"
## [1341] "bvs"  "copr" "osp"  "buy"  "aps"  "pru"  "pmz"  "pdn"  "pit"  "pdt"
## [1351] "pro"  "pfus" "peo"  "pje"  "poc"  "alq"  "afd"  "ash"  "aok"  "acou"
## [1361] "ada"  "rbc"  "ttz"  "blq"  "bacc" "asx"  "coc"  "ccm"  "col"  "chg"
## [1371] "capn" "cgh"  "clk"  "cspu" "ccyn" "caph" "csto" "capf" "ran"  "rai"
## [1381] "rar"  "rae"  "orh"  "ori"  "bcad" "apib" "civ"  "ise"  "cte"  "cpc"
## [1391] "clz"  "cch"  "cph"  "cpb"  "cli"  "pvi"  "plt"  "pph"  "paa"  "proc"
## [1401] "prs"  "pros" "cts"  "mro"  "caci" "aae"  "hya"  "hho"  "hys"  "hth"
## [1411] "hte"  "tal"  "trd"  "sul"  "saf"  "pmx"  "ttk"  "tam"  "dte"  "tma"
## [1421] "tmm"  "tmi"  "tmw"  "tmq"  "tmx"  "tpt"  "trq"  "tna"  "tnp"  "thq"
## [1431] "thz"  "thr"  "tle"  "tta"  "phy"  "tme"  "taf"  "thp"  "ther" "fno"
## [1441] "fpe"  "fia"  "ocy"  "pmo"  "mpz"  "marn" "dtn"  "kol"  "kpf"  "mpg"
## [1451] "minf" "asac" "cpo"  "din"  "ddf"  "dap"  "cni"  "fsi"  "gtl"  "dth"
## [1461] "dtu"  "tye"  "tid"  "top"  "tcm"  "thet" "cthi" "tav"  "tmai" "prf"
## [1471] "bana" "wwe"  "mja"  "mfe"  "mvu"  "mfs"  "mif"  "mjh"  "mig"  "mmp"
## [1481] "mmq"  "mmx"  "mmz"  "mmd"  "mmak" "mmao" "mmad" "mae"  "mvn"  "mvo"
## [1491] "mok"  "metf" "mth"  "mmg"  "metc" "mwo"  "mete" "metz" "metk" "mthm"
## [1501] "mst"  "metb" "mru"  "msi"  "meb"  "mmil" "meye" "mol"  "mel"  "mew"
## [1511] "meth" "mfc"  "mfi"  "mcub" "msub" "metn" "mett" "meto" "mfv"  "mka"
## [1521] "afu"  "afg"  "apo"  "ave"  "ast"  "fpl"  "gac"  "gah"  "pfu"  "pfi"
## [1531] "pho"  "pab"  "pyn"  "pya"  "pys"  "pyc"  "tko"  "ton"  "tga"  "tsi"
## [1541] "tba"  "tha"  "tlt"  "ths"  "tnu"  "teu"  "tgy"  "thv"  "tch"  "tpep"
## [1551] "tpie" "tce"  "tbs"  "thh"  "tsl"  "ttd"  "tprf" "trl"  "tpaf" "ppac"
## [1561] "mba"  "mby"  "mbw"  "mbar" "mbak" "mma"  "mmaz" "mmj"  "mmac" "mvc"
## [1571] "mek"  "mls"  "metm" "mef"  "meq"  "msj"  "msz"  "msw"  "mthr" "mthe"
## [1581] "mfz"  "mbu"  "mmet" "mmh"  "mhaz" "mev"  "mzh"  "mpy"  "mzi"  "mhz"
## [1591] "mtp"  "mcj"  "mhi"  "mhu"  "mla"  "mem"  "mbg"  "mema" "mpi"  "mbn"
## [1601] "mfo"  "mpl"  "mpd"  "mez"  "rci"  "hal"  "hsl"  "halh" "hhsr" "hsu"
## [1611] "hsf"  "halr" "nph"  "nmo"  "hut"  "hti"  "hala" "harc" "hwa"  "hwc"
## [1621] "hme"  "halm" "halp" "hlc"  "tac"  "tvo"  "fac"  "fai"  "tar"  "max"
```

```
## [1631] "mer"  "mear" "marc" "abi"  "acf"  "ape"  "acj"  "smr"  "shc"  "iho"
## [1641] "iis"  "dka"  "dmu"  "tag"  "iag"  "thg"  "hbu"  "pfm"  "pdl"  "mcn"
## [1651] "mhk"  "mten" "aho"  "asul" "aamb" "sazo" "step" "pai"  "pis"  "pcl"
## [1661] "tne"  "pyw"  "ttn"  "tuz"  "tpe"  "thb"  "tcb"  "thel" "asc"  "acia"
## [1671] "clg"  "nmr"  "nir"  "nkr"  "nid"  "nin"  "niw"  "ncl"  "nox"  "nue"
## [1681] "nct"  "nic"  "csy"  "nga"  "nvn"  "nev"  "taa"  "ncv"  "csu"  "nbv"
## [1691] "tah"  "ndv"  "ccai" "kcr"  "barc" "barb" "miy"  "arg"
```

Organisms classified within cluster 6

```
##   [1] "hsa"  "ptr"  "pps"  "ggo"  "pon"  "nle"  "mcc"  "mcf"  "csab" "caty"
##  [11] "panu" "rro"  "rbb"  "tfn"  "pteh" "cjc"  "sbq"  "mmur" "mmu"  "mcal"
##  [21] "mpah" "rno"  "mcoc" "mun"  "cge"  "pleu" "ngi"  "hgl"  "ccan" "ocu"
##  [31] "opi"  "tup"  "cfa"  "vvp"  "vlg"  "aml"  "umr"  "uah"  "oro"  "elk"
##  [41] "mpuf" "eju"  "mlx"  "fca"  "pyu"  "pbg"  "ptg"  "ppad" "aju"  "hhv"
##  [51] "bta"  "bom"  "biu"  "bbub" "chx"  "oas"  "oda"  "ccad" "ssc"  "cfr"
##  [61] "cbai" "cdk"  "bacu" "lve"  "oor"  "dle"  "pcad" "ecb"  "epz"  "eai"
##  [71] "myb"  "myd"  "mmyo" "mna"  "pkl"  "hai"  "dro"  "shon" "ajm"  "pdic"
##  [81] "mmf"  "rfq"  "pale" "pgig" "ray"  "mjv"  "tod"  "lav"  "tmu"  "mdo"
##  [91] "gas"  "shr"  "pcw"  "oaa"  "gga"  "pcoc" "mgp"  "cjo"  "nmel" "apla"
## [101] "acyg" "tgu"  "lsr"  "scan" "pmoa" "otc"  "pruf" "gfr"  "fab"  "phi"
## [111] "pmaj" "ccae" "ccw"  "etl"  "fpg"  "fch"  "clv"  "egz"  "nni"  "acun"
## [121] "padl" "aam"  "arow" "npd"  "dne"  "asn"  "amj"  "cpoo" "ggn"  "pss"
## [131] "cmy"  "cpic" "tst"  "cabi" "acs"  "pvt"  "sund" "pbi"  "pmur" "tsr"
## [141] "pgut" "vko"  "pmua" "zvi"  "gja"  "xla"  "xtr"  "npr"  "dre"  "srx"
## [151] "sanh" "sgh"  "ccar" "caua" "ipu"  "phyp" "amex" "eee"  "tru"  "tng"
## [161] "lco"  "ncc"  "cgob" "ely"  "plep" "sluc" "ecra" "pflv" "gat"  "ppug"
## [171] "msam" "cud"  "mze"  "onl"  "oau"  "ola"  "oml"  "xma"  "xco"  "xhe"
## [181] "pret" "cvg"  "ctul" "nfu"  "kmr"  "alim" "aoce" "csem" "pov"  "ssen"
## [191] "lcf"  "sdu"  "slal" "xgl"  "hcq"  "bpec" "malb" "sasa" "otw"  "omy"
## [201] "salp" "snh"  "els"  "sfm"  "pki"  "aang" "loc"  "pspa" "arut" "lcm"
## [211] "cmk"  "rtp"  "bfo"  "bbel" "cin"  "sclv" "spu"  "aplc" "sko"  "dme"
## [221] "der"  "dse"  "dsi"  "dya"  "dan"  "dsr"  "dpo"  "dpe"  "dmn"  "dwi"
```

```
## [231] "dgr"  "dmo"  "daz"  "dnv"  "dhe"  "dvi"  "ccat" "bod"  "mde"  "scac"
## [241] "lcq"  "aga"  "acoz" "aara" "aag"  "aalb" "cqu"  "cpii" "ame"  "acer"
## [251] "bim"  "bbif" "bvk"  "bvan" "bter" "ccal" "obb"  "mgen" "nmea" "cgig"
## [261] "soc"  "mpha" "aec"  "acep" "pbar" "vem"  "hst"  "dqu"  "cfo"  "fex"
## [271] "lhu"  "pgc"  "obo"  "pcf"  "pfuc" "vps"  "nvi"  "csol" "tpre" "mdl"
## [281] "cglo" "fas"  "dam"  "ccin" "tca"  "dpa"  "atd"  "agb"  "ldc"  "nvl"
## [291] "apln" "ppyr" "otu"  "bmor" "bman" "msex" "dpl"  "bany" "pmac" "ppot"
## [301] "pxu"  "prap" "zce"  "haw"  "tnl"  "pxy"  "api"  "dnx"  "ags"  "rmd"
## [311] "btab" "dci"  "clec" "hhal" "nlu"  "phu"  "foc"  "zne"  "csec" "fcd"
## [321] "dpx"  "dmk"  "pvm"  "pja"  "hame" "hazt" "eaf"  "isc"  "dsv"  "rsan"
## [331] "rmp"  "vde"  "vja"  "tut"  "dpte" "cscu" "ptep" "sdm"  "cel"  "cbr"
## [341] "bmy"  "loa"  "nai"  "tsp"  "hro"  "lgi"  "pcan" "bgt"  "gae"  "crg"
## [351] "myi"  "pmax" "obi"  "osn"  "lak"  "smm"  "ovi"  "egl"  "nve"  "epa"
## [361] "aten" "adf"  "amil" "pdam" "spis" "dgt"  "hmg"  "tad"  "aqu"
```

Shortest path (SP) kernel

Heatmap

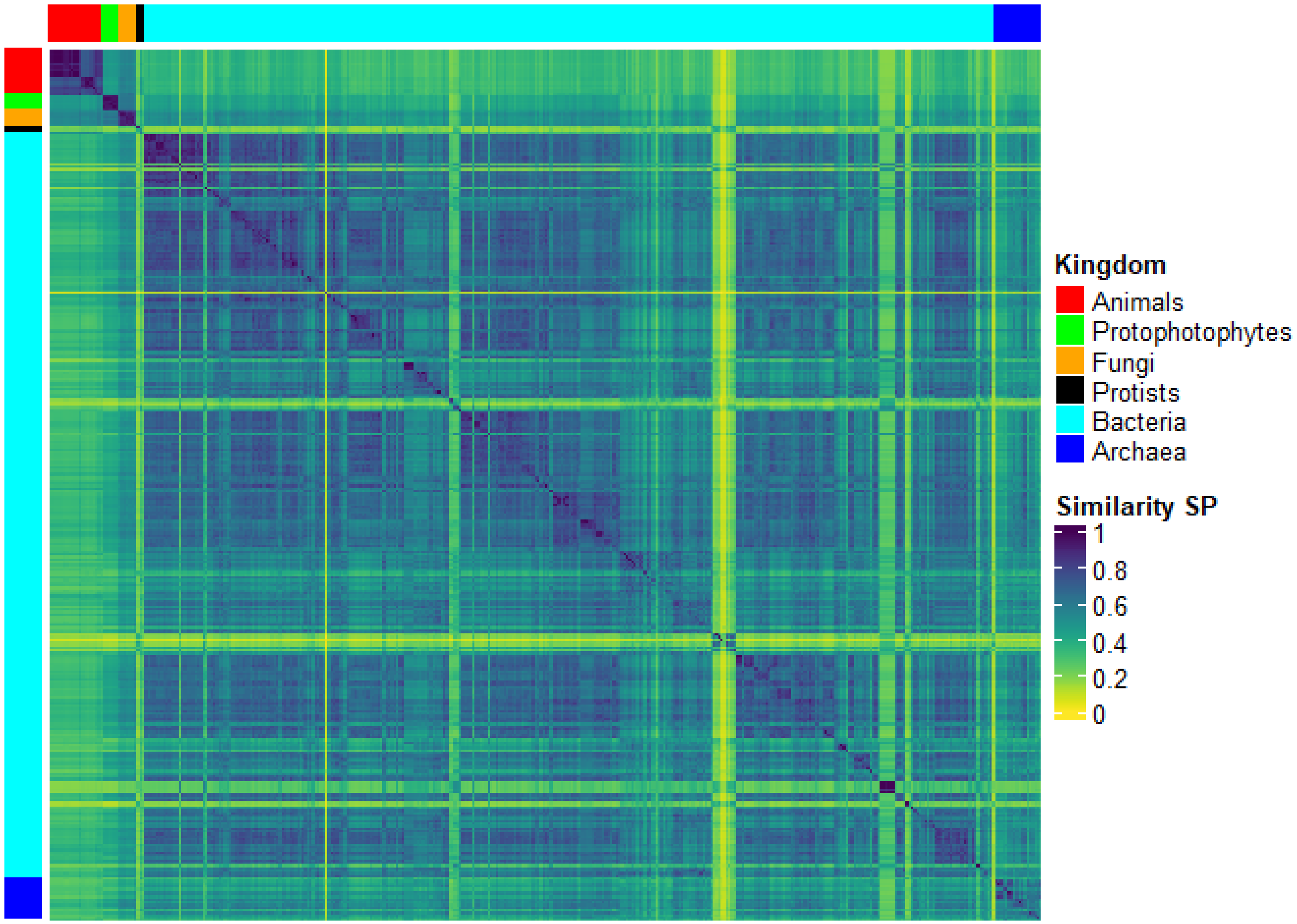
Kingdom
Animals
Protophotophytes
Fungi
Protists
Bacteria
Archaea
Similarity SP
1
0.8
0.6
0.4
0.2
0

MDS for SP

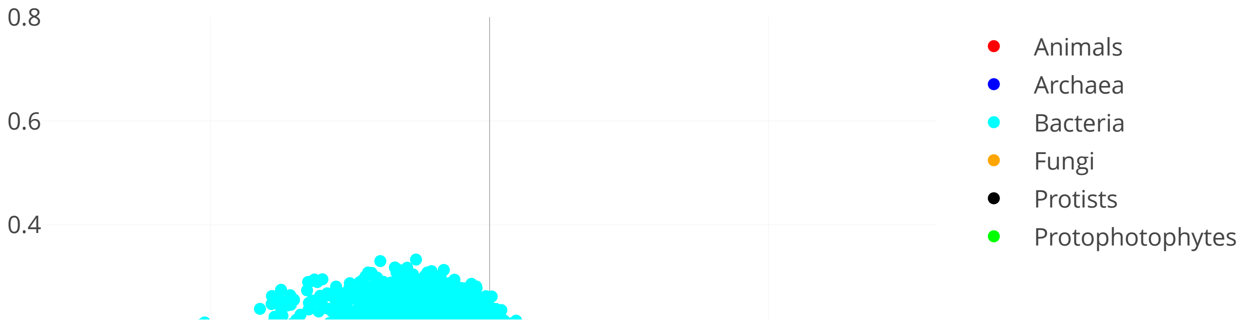
0.8
0.6
0.4
Animals
Archaea
Bacteria
Fungi
Protists
Protophotophytes

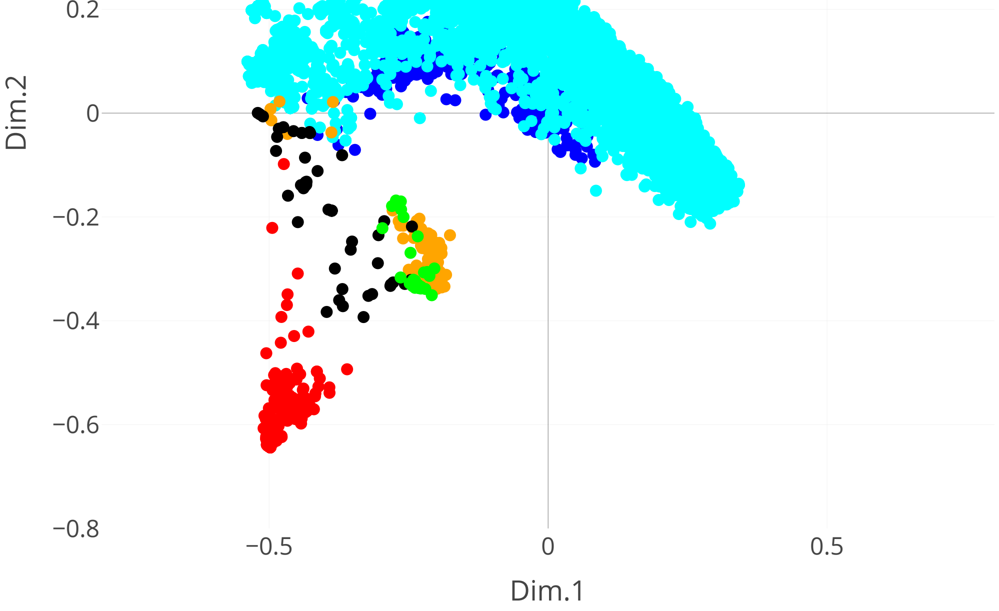

6-Means clustering for SP kernel

```
##                        Cluster
## Real group                1    2    3    4    5    6
##    Animals                1    0    4    0    0  365
##    Archaea               60   96    0  180    3    0
##    Bacteria             683 1425    0 1695 2312    0
##    Fungi                  7    0  131    0    0    0
##    Protists              15    0   37    0    0    0
##    Protophotophytes       0    0  127    0    0    0
```

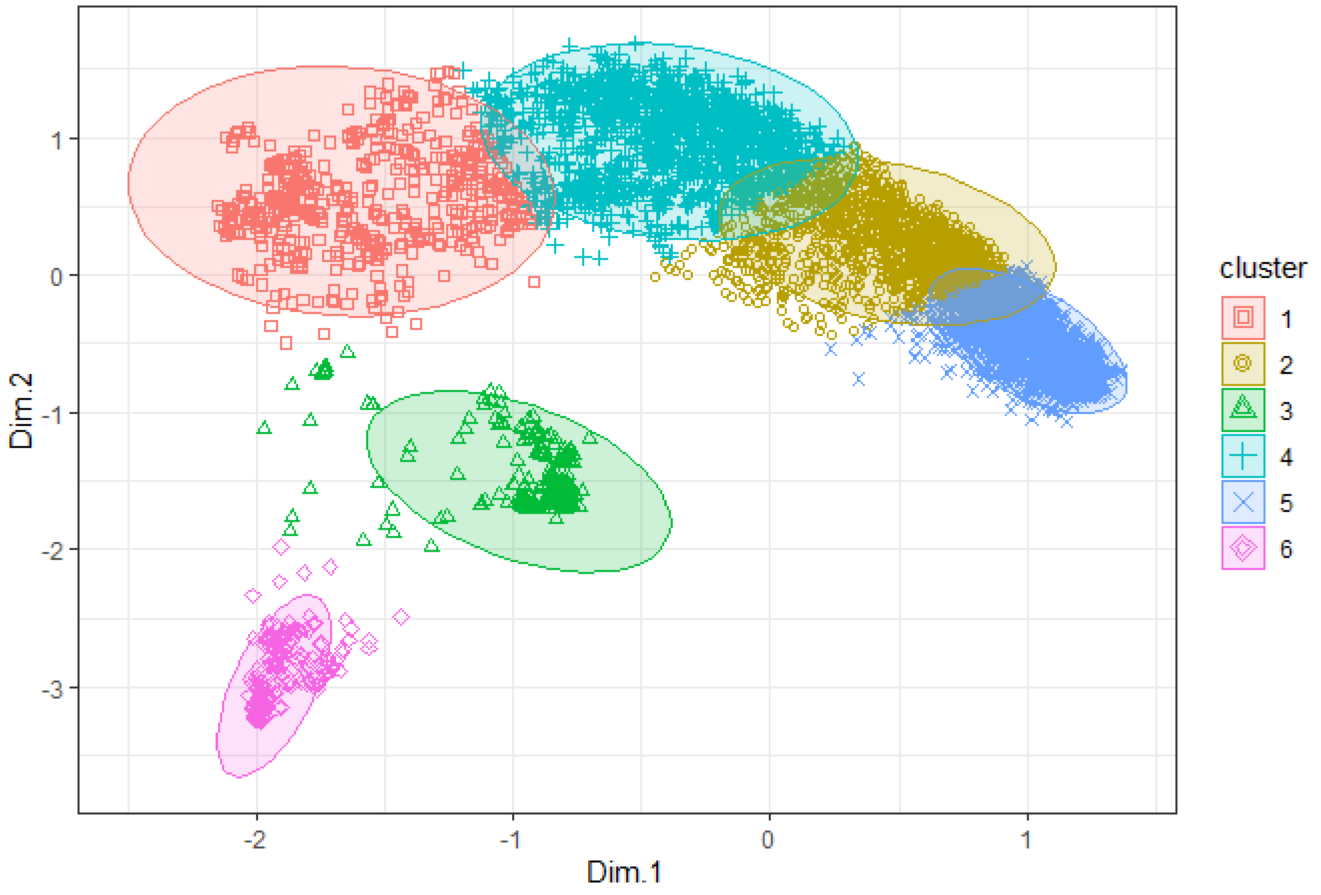

Organisms classified within cluster 1

```
##   [1] "shx"  "ppl"  "mpr"  "ecu"  "ein"  "ehe"  "ero"  "nce"  "ehi"  "edi"
##  [11] "eiv"  "pfd"  "tan"  "tpv"  "tot"  "beq"  "bbo"  "bmic" "cpv"  "cho"
##  [21] "tgo"  "tva"  "gla"  "bfl"  "bpn"  "bva"  "bchr" "ben"  "hde"  "sect"
##  [31] "sehc" "senm" "rip"  "rig"  "men"  "meo"  "icp"  "sbw"  "den"  "hed"
##  [41] "ged"  "cmik" "ppet" "ssz"  "fsm"  "seny" "ehd"  "buc"  "bap"  "bau"
##  [51] "baw"  "bajc" "bua"  "bup"  "bak"  "buh"  "bapf" "bapg" "bapu" "bapw"
##  [61] "bas"  "bab"  "bcc"  "baj"  "baph" "wbr"  "wgl"  "asy"  "aen"  "hdu"
##  [71] "adp"  "cey"  "cea"  "cend" "rvi"  "les"  "ple"  "ply"  "plr"  "plo"
##  [81] "pld"  "plb"  "plc"  "pli"  "paly" "crp"  "cru"  "crc"  "crt"  "crh"
```

```
##  [91] "crv"  "cri"  "eme"  "dno"  "eof"  "bci"  "bcib" "bcig" "zin"  "sutt"
## [101] "tpn"  "tpq"  "tpj"  "kci"  "kct"  "kbl"  "kbt"  "kde"  "kga"  "kon"
## [111] "kso"  "ssdc" "ndl"  "vfg"  "rpr"  "rpo"  "rpw"  "rpz"  "rpg"  "rps"
## [121] "rpv"  "rpq"  "rpl"  "rpn"  "rty"  "rtt"  "rtb"  "rcm"  "rcc"  "rbe"
## [131] "rbo"  "rco"  "rfe"  "rak"  "rri"  "rrj"  "rra"  "rrc"  "rrh"  "rrb"
## [141] "rrn"  "rrp"  "rrm"  "rrr"  "rms"  "rmi"  "rpk"  "raf"  "rhe"  "rja"
## [151] "rsv"  "rsw"  "rph"  "rau"  "rmo"  "rpp"  "rre"  "ram"  "rab"  "rmc"
## [161] "ras"  "ric"  "ots"  "ott"  "ptc"  "wol"  "wri"  "wen"  "wed"  "wpi"
## [171] "wbm"  "woo"  "wcl"  "weo"  "wpp"  "ama"  "amf"  "amw"  "amp"  "acn"
## [181] "aph"  "apy"  "apd"  "apha" "aoh"  "eru"  "erw"  "erg"  "ecn"  "ech"
## [191] "echa" "echj" "echl" "echs" "echv" "echw" "echp" "emr"  "ehh"  "nse"
## [201] "nri"  "nhm"  "nef"  "mmn"  "fso"  "ren"  "paca" "caq"  "naf"  "eaa"
## [211] "las"  "laa"  "lat"  "lso"  "lar"  "lau"  "bbk"  "bcd"  "baus" "banc"
## [221] "bart" "bara" "barr" "baro" "barj" "hci"  "hct"  "hcc"  "hcd"  "hgn"
## [231] "thal" "efk"  "ocb"  "got"  "gmo"  "geq"  "gsa"  "gha"  "spyo" "ljo"
## [241] "ljf"  "lac"  "lad"  "laf"  "ldb"  "lbu"  "ldl"  "lga"  "lhe"  "lhl"
## [251] "lhv"  "lhh"  "lhd"  "lam"  "lai"  "lay"  "lke"  "law"  "lae"  "lgl"
## [261] "lje"  "lamy" "lkl"  "lapi" "lhs"  "lva"  "lpon" "lsn"  "lle"  "lfv"
## [271] "lsua" "lji"  "lku"  "lmae" "ppe"  "ppen" "pio"  "lah"  "ldx"  "wko"
## [281] "wce"  "wct"  "wci"  "wcf"  "wdi"  "wvr"  "mps"  "mpx"  "vah"  "acg"
## [291] "adc"  "dpm"  "asf"  "asm"  "aso"  "asb"  "clo"  "fsa"  "lbw"  "faa"
## [301] "pbq"  "mdv"  "bprm" "fma"  "pmic" "phar" "piv"  "dpn"  "ers"  "eri"
## [311] "mge"  "mgu"  "mgc"  "mgq"  "mgx"  "mpn"  "mpm"  "mpj"  "mpb"  "mpe"
## [321] "mga"  "mgh"  "mgf"  "mgn"  "mgs"  "mgt"  "mgv"  "mgw"  "mgac" "mgan"
## [331] "mgnc" "mgz"  "mmy"  "mmym" "mmyi" "mml"  "mcp"  "mcac" "mcap" "mcar"
## [341] "mcai" "mlc"  "mlh"  "mmo"  "mhy"  "mhj"  "mhp"  "mhn"  "mhyl" "mhyo"
## [351] "mat"  "mco"  "mho"  "mhom" "mcd"  "mhr"  "mhh"  "mhm"  "mhs"  "mhv"
## [361] "mha"  "mhf"  "mss"  "msk"  "mpf"  "mput" "mhe"  "mwe"  "mhl"  "mhb"
## [371] "mpv"  "mov"  "mbc"  "mgj"  "mfq"  "mcan" "myt"  "mds"  "myg"  "mpho"
## [381] "mhyv" "mclo" "mamp" "mans" "mphc" "miw"  "mane" "mnh"  "mnu"  "mstr"
## [391] "mcr"  "mcm"  "mgb"  "mgly" "mcou" "mcom" "mpu"  "msy"  "mso"  "maa"
## [401] "mal"  "mfr"  "mfm"  "mfp"  "mbv"  "mbh"  "mbi"  "mbq"  "mcy"  "mcas"
## [411] "mck"  "marg" "mpul" "mbov" "mboh" "mani" "mphi" "uur"  "upa"  "upr"
## [421] "uue"  "hcr"  "poy"  "ayw"  "mbp"  "pml"  "pal"  "nzs"  "psol" "pzi"
```

```
## [431] "acl"  "apal" "aoc"  "aaxa" "ahk"  "mfl"  "mfw"  "mchc" "mlac" "ment"
## [441] "msyr" "mtab" "mcol" "elj"  "esx"  "efr"  "eml"  "scr"  "ssyr" "sdi"
## [451] "stai" "sapi" "smir" "smia" "scq"  "ssab" "satr" "seri" "stur" "sll"
## [461] "skn"  "scj"  "shj"  "sck"  "sfz"  "scou" "scla" "sprn" "spit" "stab"
## [471] "sphh" "smoo" "salx" "sgq"  "schi" "mbj"  "twh"  "tws"  "cig"  "nfe"
## [481] "ahe"  "arca" "fvg"  "bact" "gva"  "sij"  "pdo"  "gpa"  "apv"  "cgo"
## [491] "cyu"  "nsp"  "ctr"  "ctd"  "ctf"  "ctrd" "ctro" "ctrt" "cta"  "cty"
## [501] "cra"  "ctrq" "ctrx" "ctrz" "ctrp" "ctlj" "ctlx" "ctll" "ctb"  "ctrr"
## [511] "ctlf" "ctli" "ctl"  "ctru" "ctrl" "ctrv" "ctrm" "ctla" "ctlm" "ctls"
## [521] "ctlz" "ctlc" "ctln" "ctlb" "ctlq" "cto"  "ctrn" "ctj"  "ctz"  "ctg"
## [531] "ctk"  "csw"  "ces"  "ctrb" "ctre" "ctrs" "ctec" "cfs"  "cfw"  "ctfw"
## [541] "ctrf" "ctch" "ctn"  "ctq"  "ctv"  "ctw"  "ctrg" "ctri" "ctra" "ctrh"
## [551] "ctrj" "ctrk" "ctjt" "ctcf" "ctfs" "cthf" "ctcj" "cthj" "ctmj" "cttj"
## [561] "ctjs" "ctrc" "ctrw" "ctry" "ctct" "cmu"  "cmur" "cmn"  "cmm"  "cmg"
## [571] "cmx"  "cmz"  "cpn"  "cpa"  "cpj"  "cpt"  "clp"  "cpm"  "cpec" "cpeo"
## [581] "cper" "chp"  "chb"  "chs"  "chi"  "cht"  "chc"  "chr"  "cpsc" "cpsn"
## [591] "cpsb" "cpsg" "cpsm" "cpsi" "cpsv" "cpsw" "cpst" "cpsd" "cpsa" "cav"
## [601] "cca"  "cab"  "cabo" "cfe"  "cgz"  "chla" "pcu"  "ney"  "xii"  "psup"
## [611] "bbu"  "bbz"  "bbn"  "bbj"  "bbur" "bga"  "bgb"  "bgn"  "bgs"  "bgc"
## [621] "baf"  "bafz" "bafh" "baft" "bafe" "bbs"  "bvt"  "bchi" "bmay" "btu"
## [631] "bhr"  "bhi"  "bdu"  "bre"  "bcw"  "bmo"  "bmiy" "bpak" "bane" "btur"
## [641] "bmat" "tpa"  "tpw"  "tpp"  "tpu"  "tph"  "tpo"  "tpas" "tpc"  "tpg"
## [651] "tpm"  "tpb"  "tde"  "tpl"  "tped" "tpk"  "trm"  "trz"  "smf"  "sns"
## [661] "sbr"  "aas"  "che"  "cec"  "cher" "smg"  "sms"  "smh"  "sum"  "smv"
## [671] "smub" "smum" "smue" "smup" "bbl"  "bpi"  "bmm"  "bcp"  "bbg"  "bbq"
## [681] "blp"  "blu"  "blck" "elv"  "udi"  "cex"  "saal" "sbe"  "sbag" "sox"
## [691] "prf"  "bana" "bih"  "srb"  "srg"  "caqa" "dpb"  "tmg"  "wwe"  "bgw"
## [701] "bbgw" "mib"  "wba"  "pwo"  "cgw"  "baab" "mja"  "mvu"  "mfs"  "mif"
## [711] "mvo"  "metf" "pho"  "pya"  "pyc"  "the"  "tha"  "thm"  "ths"  "tnu"
## [721] "teu"  "tgy"  "tpep" "tpie" "tgg"  "thh"  "tsl"  "ttd"  "tprf" "trl"
## [731] "tpaf" "thy"  "ppac" "nac"  "mear" "abi"  "acf"  "smr"  "shc"  "iho"
## [741] "dka"  "dfd"  "dmu"  "tag"  "thg"  "hbu"  "tne"  "tpe"  "thb"  "tcb"
## [751] "thf"  "thel" "asc"  "acia" "ffo"  "nic"  "ccai" "barc" "barb" "neq"
## [761] "naa"  "marh" "flt"  "miy"  "agw"  "arg"
```

Organisms classified within cluster 2

```
##   [1] "sfl"  "sdy"  "sdz"  "psts" "yel"  "yru"  "yrb"  "yak"  "lbq"  "lpop"
##  [11] "sod"  "tpty" "pmr"  "pmib" "pvl"  "pvg"  "phau" "prot" "pcol" "pcib"
##  [21] "xbo"  "xbv"  "xpo"  "mmk"  "ans"  "eic"  "etr"  "etd"  "ete"  "etc"
##  [31] "edw"  "edl"  "eho"  "lpv"  "pfq"  "prag" "pshi" "mvi"  "mvg"  "mve"
##  [41] "mgra" "aio"  "gan"  "bto"  "btre" "btrh" "btra" "rhey" "fcl"  "lmb"
##  [51] "lyt"  "lue"  "lyj"  "lum"  "lus"  "lug"  "thes" "theh" "tcn"  "fau"
##  [61] "dye"  "vch"  "vcf"  "vcs"  "vce"  "vcq"  "vcj"  "vci"  "vco"  "vcr"
##  [71] "vcm"  "vcl"  "vcx"  "vcz"  "van"  "lag"  "vau"  "vbr"  "vsc"  "vqi"
##  [81] "vfi"  "vfm"  "vsa"  "awd"  "pds"  "pmai" "saly" "sks"  "scot" "pbb"
##  [91] "emo"  "par"  "pur"  "abm"  "att"  "aei"  "acv"  "ahl"  "ajn"  "asj"
## [101] "aid"  "acum" "aug"  "alj"  "mct"  "mcs"  "mcat" "moi"  "mos"  "mbl"
## [111] "mboi" "mcun" "mnn"  "mbah" "son"  "ipi"  "idi"  "coz"  "cate" "psy"
## [121] "cja"  "ceb"  "cek"  "ceg"  "sde"  "cbu"  "cbd"  "alg"  "asip" "lpn"
## [131] "lph"  "lpf"  "lpp"  "lpc"  "lpa"  "lok"  "lgt"  "lwa"  "mca"  "metu"
## [141] "mdn"  "mdh"  "mko"  "metl" "mah"  "mbur" "mpsy" "mmob" "mein" "mej"
## [151] "mec"  "cyq"  "cza"  "cyy"  "psal" "tig"  "blep" "this" "noc"  "nhl"
## [161] "nwa"  "nwr"  "alv"  "tvi"  "tmb"  "mpur" "tee"  "tsy"  "thip" "aeh"
## [171] "hha"  "hhk"  "hhc"  "ebs"  "tgr"  "tni"  "tti"  "ssal" "spiu" "sros"
## [181] "spiz" "aprs" "haz"  "wma"  "ttc"  "kus"  "kma"  "kuy"  "kak"  "kko"
## [191] "kge"  "ksd"  "kpd"  "tol"  "tor"  "oai"  "llp"  "acav" "tau"  "ocm"
## [201] "opf"  "orb"  "sdf"  "gbi"  "slim" "sva"  "acii" "tbn"  "tsn"  "thin"
## [211] "pspi" "nwe"  "nzl"  "naq"  "nbl"  "nci"  "nani" "salv" "vit"  "nba"
## [221] "lhk"  "jeu"  "dee"  "chiz" "cfon" "pnu"  "pne"  "pdq"  "poh"  "hyf"
## [231] "lmir" "pud"  "phn"  "our"  "cke"  "cbaa" "cbab" "har"  "neu"  "net"
## [241] "nit"  "nii"  "nco"  "nur"  "nst"  "nmu"  "nlc"  "doe"  "meh"  "mei"
## [251] "mep"  "meu"  "slt"  "sniv" "rbu"  "rbh"  "atw"  "tcl"  "tak"  "zpa"
## [261] "bprc" "had"  "ant"  "aell" "aaqi" "asui" "aclo" "aana" "avp"  "adz"
## [271] "alk"  "alp"  "ahs"  "amyt" "amar" "hebr" "paco" "arc"  "shal" "gme"
## [281] "gur"  "gbm"  "geo"  "geb"  "gbn"  "pef"  "des"  "deu"  "dhy"  "dol"
## [291] "dml"  "dal"  "dat"  "dto"  "vin"  "dti"  "sfu"  "dbr"  "dav"  "bsed"
```

```
## [301] "pcay" "pmob" "bcee" "nwi"  "mets" "mlg"  "rhj"  "hdn"  "hdt"  "rva"
## [311] "blag" "mcg"  "metg" "mbry" "mtw"  "phb"  "rsq"  "rcp"  "rhp"  "pcon"
## [321] "pmut" "kvl"  "kvu"  "kro"  "oct"  "cmar" "hyt"  "sphl" "spha" "slut"
## [331] "srhi" "aep"  "gti"  "gbe"  "gbh"  "gbc"  "gbs"  "gdi"  "gdj"  "gxy"
## [341] "gxl"  "kna"  "keu"  "ksc"  "kre"  "kha"  "apf"  "apu"  "apg"  "apq"
## [351] "apx"  "apz"  "apk"  "asz"  "aace" "ato"  "aoy"  "rru"  "rrf"  "rpm"
## [361] "mgy"  "mgry" "ahu"  "magq" "hjo"  "dex"  "dvn"  "pub"  "pel"  "peg"
## [371] "apc"  "apm"  "mai"  "man"  "apb"  "bba"  "bbat" "bbw"  "bbac" "bex"
## [381] "bdq"  "bdc"  "bdz"  "bmx"  "hax"  "bsto" "sbf"  "maes" "mfn"  "htl"
## [391] "bsu"  "bsr"  "bsl"  "bsh"  "bsy"  "bsut" "bsul" "bsus" "bso"  "bsn"
## [401] "bsq"  "bsx"  "bsp"  "bss"  "bst"  "bli"  "bld"  "bay"  "baq"  "bya"
## [411] "bamp" "baml" "bama" "bamn" "bamb" "bamt" "bamy" "bmp"  "bao"  "baz"
## [421] "bql"  "bxh"  "bqy"  "bami" "bamc" "bamf" "bsia" "bvm"  "bht"  "ban"
## [431] "bar"  "bat"  "bah"  "bai"  "bax"  "bant" "banr" "bans" "banh" "banv"
## [441] "bce"  "bcz"  "bcr"  "bcb"  "bcu"  "bcg"  "bcq"  "bcx"  "bal"  "bnc"
## [451] "bcf"  "bcef" "bcy"  "btk"  "btl"  "btb"  "btt"  "bthr" "bthi" "btc"
## [461] "btf"  "btm"  "btg"  "bti"  "btn"  "btht" "bthu" "btw"  "bthy" "bwe"
## [471] "bww"  "bmyo" "bty"  "bby"  "bwd"  "btro" "bmob" "bpu"  "bpum" "bpus"
## [481] "bco"  "bjs"  "baci" "bif"  "bmet" "gst"  "bacw" "bacp" "bacb" "baco"
## [491] "bacy" "bacl" "balm" "bsm"  "bwh"  "bxi"  "bhk"  "bbev" "balt" "bacs"
## [501] "bsaf" "bit"  "bcir" "bcoh" "bda"  "bfx"  "bck"  "bag"  "bcoa" "bha"
## [511] "bcl"  "bpf"  "ble"  "bkw"  "bgi"  "oih"  "ocn"  "gka"  "gte"  "gtk"
## [521] "gtm"  "gli"  "gtn"  "gwc"  "gyc"  "gea"  "gel"  "gse"  "gsr"  "gej"
## [531] "ptb"  "agn"  "anm"  "aamy" "anl"  "and"  "acai" "lsp"  "lgy"  "lfu"
## [541] "lys"  "lyb"  "lyz"  "lpak" "hhd"  "hmn"  "hli"  "vir"  "vhl"  "vim"
## [551] "lao"  "fpn"  "far"  "sje"  "apak" "pasa" "bthv" "psyh" "psyo" "prd"
## [561] "grc"  "rue"  "ntm"  "meku" "aia"  "blen" "bse"  "sue"  "suf"  "sha"
## [571] "shh"  "ssp"  "sca"  "ssd"  "sdt"  "sdp"  "swa"  "sxy"  "sxl"  "sxo"
## [581] "shu"  "ssch" "sscz" "sagq" "seqo" "ssif" "scv"  "slz"  "snl"  "skl"
## [591] "sfq"  "scar" "schr" "sarl" "spic" "sscu" "sste" "mlen" "shv"  "lmo"
## [601] "lmn"  "lmy"  "lmt"  "lmoc" "lmoe" "lmob" "lmod" "lmow" "lmoq" "lmr"
## [611] "lmom" "lmf"  "lmc"  "lmog" "lmp"  "lmol" "lmoj" "lmoz" "lmox" "lmh"
## [621] "lmq"  "lml"  "lmg"  "lms"  "lmj"  "lmw"  "lmx"  "lmz"  "lmon" "lmos"
## [631] "lmoo" "lmoy" "lmot" "lmoa" "lmok" "lmv"  "lin"  "lwe"  "lsg"  "liv"
```

```
## [641] "lii"  "liw"  "lia"  "lio"  "bths" "esi"  "ean"  "exm"  "exu"  "blr" 
## [651] "bagr" "brw"  "pjd"  "gym"  "ppy"  "ppm"  "ppo"  "ppol" "ppq"  "ppoy"
## [661] "pta"  "plv"  "pbd"  "pgm"  "pod"  "paen" "paef" "paeq" "pste" "paea"
## [671] "paee" "paeh" "paej" "pbj"  "pih"  "pri"  "ppeo" "pow"  "pbv"  "pxl" 
## [681] "pswu" "pdh"  "pib"  "pcx"  "pkb"  "paih" "pvo"  "plw"  "plen" "ppsc"
## [691] "plut" "pchi" "pbk"  "pprt" "pbac" "prz"  "plyc" "tco"  "asoc" "coh" 
## [701] "cohn" "tum"  "tab"  "eff"  "sob"  "pln"  "pku"  "prt"  "pll"  "pana"
## [711] "pdg"  "phc"  "pmar" "ppla" "pfae" "plx"  "pmat" "pdec" "jeo"  "kur" 
## [721] "kzo"  "spop" "sure" "spos" "spae" "rst"  "paek" "play" "vij"  "ntr" 
## [731] "lfb"  "tvu"  "kpul" "keb"  "lcl"  "emu"  "ess"  "egv"  "abae" "crn" 
## [741] "cml"  "cac"  "cae"  "cay"  "cbe"  "cbz"  "cbei" "csr"  "cpas" "cpat"
## [751] "cpae" "csb"  "csq"  "cace" "cck"  "cbut" "ctyk" "ceu"  "cfm"  "cdrk"
## [761] "cdy"  "amt"  "gfe"  "capr" "bpro" "lacy" "arf"  "anr"  "cbol" "cdc" 
## [771] "cdl"  "sth"  "dsy"  "dhd"  "ddh"  "ddl"  "dmt"  "dgi"  "dor"  "dai" 
## [781] "dmi"  "tfr"  "tmr"  "thef" "thep" "sthr" "abut" "chy"  "hhl"  "meg" 
## [791] "puf"  "pft"  "sted" "ciu"  "erb"  "lpil" "mtu"  "mtv"  "mtc"  "mra" 
## [801] "mtf"  "mtb"  "mtk"  "mtz"  "mtg"  "mti"  "mte"  "mtur" "mtl"  "mto" 
## [811] "mtd"  "mtn"  "mtub" "mtuc" "mtx"  "mtuh" "mtul" "mtut" "mtuu" "mtq" 
## [821] "mbo"  "mbb"  "mbt"  "mbm"  "mbk"  "mbx"  "maf"  "mmic" "mce"  "mcq" 
## [831] "mcv"  "mcx"  "mcz"  "mlp"  "mhad" "mxe"  "mdr"  "mmin" "cgl"  "cgb" 
## [841] "cgu"  "cgt"  "cgs"  "cgg"  "cgm"  "cgj"  "cgq"  "cgx"  "cef"  "cdh" 
## [851] "cds"  "cdip" "cjk"  "cur"  "cua"  "car"  "ckp"  "crd"  "cva"  "ccn" 
## [861] "cter" "cmd"  "caz"  "cfn"  "ccg"  "cvt"  "cii"  "cuv"  "coa"  "cdo" 
## [871] "chm"  "csx"  "cmq"  "ccj"  "cmv"  "cei"  "cted" "clw"  "cdx"  "csp" 
## [881] "csta" "ccjz" "cfk"  "cpho" "cfc"  "cgv"  "cstr" "caqu" "csph" "cmin"
## [891] "cpeg" "cxe"  "cee"  "csan" "cgk"  "cpre" "csur" "bfv"  "srt"  "dlu" 
## [901] "lxl"  "lxy"  "cmi"  "cms"  "cmc"  "cmh"  "ccap" "mim"  "mix"  "mpal"
## [911] "mih"  "maur" "mfol" "moo"  "msed" "rtc"  "rtn"  "rry"  "ria"  "rfs" 
## [921] "rte"  "cum"  "cub"  "cug"  "mvd"  "frp"  "agf"  "cart" "cry"  "amin"
## [931] "aum"  "auw"  "malk" "salc" "sala" "sald" "hum"  "huw"  "gry"  "lyk" 
## [941] "plap" "leu"  "leb"  "ldn"  "agg"  "mant" "frn"  "gln"  "chre" "agx" 
## [951] "ari"  "rsa"  "krh"  "kpl"  "kii"  "kod"  "kvr"  "mlu"  "mick" "rama"
## [961] "rkr"  "nae"  "aul"  "bcv"  "bfa"  "brx"  "brv"  "bgg"  "brz"  "bsau"
## [971] "brr"  "kse"  "dni"  "day"  "xce"  "xyl"  "iva"  "ido"  "cet"  "xya"
```

```
##  [981] "ske"  "sanw" "cfl"  "cfi"  "cga"  "cez"  "celz" "cej"  "celh" "oek"
##  [991] "psei" "ars"  "teh"  "phw"  "serj" "serw" "orn"  "orz"  "blin" "bri"
## [1001] "blut" "gez"  "pfr"  "pfre" "pacd" "paus" "pbo"  "aaci" "acij" "aji"
## [1011] "mik"  "micg" "tfl"  "tfa"  "tes"  "tez"  "tdf"  "tla"  "prv"  "noi"
## [1021] "ndp"  "nbe"  "aer"  "aef"  "mgg"  "tfu"  "tbi"  "fra"  "fsy"  "ace"
## [1031] "nak"  "kra"  "svi"  "fsl"  "flh"  "eke"  "abai" "rrd"  "rub"  "afo"
## [1041] "atq"  "syn"  "syz"  "syy"  "syt"  "sys"  "syq"  "syj"  "syo"  "len"
## [1051] "lbo"  "amr"  "glp"  "mar"  "mpk"  "miq"  "mvz"  "cyt"  "cyp"  "cyh"
## [1061] "cyc"  "cyj"  "cyn"  "mic"  "arp"  "pagh" "oxy"  "lfs"  "mpro" "cep"
## [1071] "gvi"  "glj"  "npu"  "non"  "nfl"  "noe"  "nsh"  "ned"  "ava"  "calh"
## [1081] "riv"  "fis"  "dfs"  "toq"  "ncn"  "cthe" "plp"  "scs"  "stan" "rrs"
## [1091] "rca"  "cau"  "chl"  "cag"  "hau"  "tro"  "sti"  "cap"  "pbf"  "tbh"
## [1101] "dra"  "dge"  "ddr"  "dmr"  "dpt"  "dgo"  "dsw"  "dch"  "dab"  "dpu"
## [1111] "dez"  "dwu"  "dfc"  "dein" "dga"  "tra"  "tth"  "ttj"  "tts"  "ttl"
## [1121] "tsc"  "thc"  "tos"  "taq"  "tbc"  "mrb"  "mre"  "msv"  "mtai" "mhd"
## [1131] "fgi"  "wch"  "obt"  "roo"  "luo"  "vbs"  "rba"  "psl"  "rul"  "mff"
## [1141] "rol"  "ahel" "lcre" "aagg" "bvo"  "lpav" "amuc" "pnd"  "pbs"  "pls"
## [1151] "plh"  "fmr"  "gmr"  "gim"  "mri"  "plon" "ges"  "gog"  "gms"  "tim"
## [1161] "lrs"  "ftj"  "uli"  "ipa"  "pbor" "agv"  "slr"  "lil"  "lie"  "lic"
## [1171] "lis"  "lbj"  "lbl"  "lbi"  "lbf"  "lst"  "laj"  "lmay" "lkm"  "lwl"
## [1181] "aba"  "aca"  "acm"  "grw"  "tsa"  "eda"  "ctm"  "fva"  "ful"  "fmo"
## [1191] "ipo"  "cpor" "gph"  "bdo"  "pet"  "dori" "drc"  "mbas" "sru"  "srm"
## [1201] "rmr"  "rmg"  "rbar" "cbae" "nso"  "nia"  "fla"  "ark"  "agi"  "arac"
## [1211] "fln"  "pseg" "pgo"  "fls"  "sgn"  "phe"  "pep"  "pcm"  "psty" "pgs"
## [1221] "pej"  "pek"  "proe" "psn"  "shg"  "sht"  "sphn" "spsc" "sphz" "sphe"
## [1231] "spdr" "sdj"  "stha" "scn"  "mup"  "mgot" "muh"  "mgin" "agd"  "oli"
## [1241] "sbx"  "cmr"  "camu" "bbd"  "evi"  "echi" "alm"  "chu"  "dfe"  "lby"
## [1251] "psez" "fli"  "hsw"  "hym"  "hyd"  "hye"  "hyg"  "hyp"  "hyz"  "hnv"
## [1261] "hyh"  "hqi"  "pko"  "ruf"  "rti"  "rud"  "nib"  "aswu" "mtt"  "fpf"
## [1271] "flm"  "fll"  "gfo"  "grl"  "gfl"  "fpc"  "fpy"  "fpo"  "fpq"  "fpv"
## [1281] "fpw"  "fpk"  "fpsz" "fjo"  "fjg"  "fbr"  "fco"  "fin"  "fgl"  "fcm"
## [1291] "ffa"  "fat"  "fki"  "fpal" "fmg"  "falb" "fcr"  "fse"  "fsn"  "fnk"
## [1301] "fak"  "rbi"  "cat"  "fbc"  "marb" "mare" "cly"  "clh"  "kdi"  "dok"
## [1311] "ddo"  "dod"  "lan"  "lvn"  "laci" "mrs"  "mlt"  "asl"  "aev"  "ndo"
```

```
## [1321] "nom"  "nsd"  "nob"  "noj"  "pom"  "pob"  "pola" "poa"  "phal" "myr"
## [1331] "mpw"  "mod"  "myz"  "win"  "wij"  "sze"  "ahz"  "syi"  "tdi"  "ten"
## [1341] "tje"  "tmar" "tmp"  "lut"  "lul"  "wfu"  "for"  "foh"  "fop"  "seon"
## [1351] "oll"  "oaq"  "fek"  "taj"  "aue"  "spon" "kos"  "kan"  "marf" "aqb"
## [1361] "aqa"  "aqd"  "emar" "mur"  "psyn" "afla" "anp"  "oci"  "mgel" "mesq"
## [1371] "gaa"  "cagg" "alti" "fba"  "fbu"  "fbe"  "wvi"  "eao"  "emn"  "een"
## [1381] "elb"  "emg"  "ego"  "egm"  "elz"  "elt"  "chz"  "cgn"  "cih"  "chh"
## [1391] "cio"  "cpip" "chrs" "chrz" "carh" "csha" "cnk"  "cjt"  "cil"  "ccau"
## [1401] "cben" "cjg"  "ccas" "cant" "kda"  "clac" "eva"  "ctak" "cnr"  "ebv"
## [1411] "efal" "este" "fte"  "flu"  "oho"  "bbau" "ial"  "mro"  "cprv" "caby"
## [1421] "nde"  "nmv"  "nio"  "nja"  "nli"  "hsl"  "hdl"  "hhb"  "hje"  "salr"
## [1431] "halr" "hma"  "hhi"  "hhn"  "hab"  "hta"  "nph"  "nmo"  "hmu"  "halz"
## [1441] "hall" "hali" "hsn"  "hrr"  "hpel" "hlt"  "hwa"  "hwc"  "hvo"  "hme"
## [1451] "hgi"  "hale" "hbo"  "haj"  "haer" "hra"  "hlm"  "halm" "hla"  "halp"
## [1461] "halb" "hezz" "halq" "srub" "hae"  "haln" "halg" "halu" "hah"  "htu"
## [1471] "hda"  "hjt"  "haly" "nmg"  "hxa"  "nat"  "npe"  "nvr"  "npl"  "nge"
## [1481] "hru"  "nou"  "sali" "hlc"  "naj"  "nag"  "nan"  "nbg"  "nas"  "fac"
## [1491] "fai"  "cdiv" "sto"  "sso"  "sol"  "ssoa" "ssol" "ssof" "sai"  "sacn"
## [1501] "sacr" "sacs" "sis"  "sid"  "sin"  "sii"  "sih"  "sir"  "sic"  "sula"
## [1511] "sule" "mhk"  "mpru" "mten" "aman" "abri" "sacd" "pog"  "vmo"  "loki"
## [1521] "psyt"
```

Organisms classified within cluster 3

```
##   [1] "bmy"  "tsp"  "smm"  "egl"  "ath"  "aly"  "crb"  "csat" "eus"  "brp"
##  [11] "bna"  "boe"  "rsz"  "thj"  "cpap" "cit"  "cic"  "pvy"  "minc" "tcc"
##  [21] "gra"  "ghi"  "gab"  "dzi"  "egr"  "gmx"  "gsj"  "pvu"  "vra"  "var"
##  [31] "vun"  "ccaj" "aprc" "mtr"  "cam"  "lja"  "adu"  "aip"  "ahf"  "lang"
##  [41] "fve"  "rcn"  "pper" "pmum" "pavi" "pdul" "mdm"  "pxb"  "zju"  "mnt"
##  [51] "csv"  "cmo"  "bhj"  "mcha" "cmax" "cmos" "cpep" "rcu"  "jcu"  "hbr"
##  [61] "mesc" "pop"  "peu"  "palz" "jre"  "qsu"  "qlo"  "twl"  "vvi"  "vri"
##  [71] "sly"  "spen" "sot"  "cann" "nta"  "nsy"  "nto"  "nau"  "ini"  "itr"
##  [81] "sind" "oeu"  "egt"  "sspl" "han"  "ecad" "lsv"  "ccav" "dcr"  "csin"
```

```
##  [91] "bvg"  "soe"  "cqi"  "nnu"  "ming" "psom" "ncol" "osa"  "dosa" "obr" 
## [101] "bdi"  "ats"  "tdc"  "sbi"  "zma"  "sita" "pvir" "phai" "pda"  "egu" 
## [111] "mus"  "dct"  "peq"  "aof"  "atr"  "smo"  "ppp"  "cre"  "vcn"  "mng" 
## [121] "csl"  "cvr"  "apro" "olu"  "ota"  "bpg"  "mis"  "mpp"  "cme"  "gsl" 
## [131] "ccp"  "sce"  "ago"  "erc"  "kla"  "kmx"  "lth"  "vpo"  "zro"  "cgr" 
## [141] "ncs"  "ndi"  "tpf"  "tbl"  "tdl"  "tgb"  "kaf"  "zmk"  "ppa"  "dha" 
## [151] "pic"  "pgu"  "spaa" "lel"  "cal"  "ctp"  "cot"  "cdu"  "cten" "yli" 
## [161] "clu"  "clus" "caur" "slb"  "pkz"  "bnn"  "bbrx" "ncr"  "nte"  "smp" 
## [171] "pan"  "ttt"  "mtm"  "cthr" "mgr"  "tmn"  "ssck" "fgr"  "fpu"  "fvr" 
## [181] "fox"  "nhe"  "tre"  "trr"  "maw"  "maj"  "cmt"  "plj"  "val"  "vda" 
## [191] "cfj"  "sapo" "ela"  "pfy"  "ssl"  "bfu"  "mbe"  "psco" "glz"  "ani" 
## [201] "afm"  "act"  "nfi"  "aor"  "ang"  "afv"  "pcs"  "pdp"  "tmf"  "trg" 
## [211] "cim"  "cpw"  "ure"  "pbl"  "pbn"  "abe"  "tve"  "aje"  "bgh"  "pno" 
## [221] "pte"  "bze"  "bsc"  "bor"  "aalt" "ztr"  "pfj"  "bcom" "npa"  "tml" 
## [231] "spo"  "cne"  "cnb"  "cgi"  "tms"  "tasa" "tvs"  "dsq"  "pco"  "shs" 
## [241] "hir"  "psq"  "adl"  "fme"  "gtr"  "lbc"  "mrr"  "cci"  "scm"  "abp" 
## [251] "abv"  "cput" "sla"  "wse"  "wic"  "uma"  "pfp"  "mgl"  "mrt"  "msym"
## [261] "pgr"  "mlr"  "mbr"  "sre"  "ddi"  "dpp"  "dfa"  "acan" "pfa"  "pfh" 
## [271] "pyo"  "pcb"  "pbe"  "pkn"  "pvx"  "pcy"  "tet"  "ptm"  "smin" "pti" 
## [281] "fcy"  "tps"  "ngd"  "aaf"  "pif"  "psoj" "spar" "ehx"  "gtt"  "tbr" 
## [291] "tbg"  "tcr"  "lma"  "lif"  "ldo"  "lmi"  "lbz"  "lpan" "ngr"
```

Organisms classified within cluster 4

```
##   [1] "bed"  "kgo"  "sgl"  "pes"  "hhs"  "pck"  "hin"  "hit"  "hip"  "hiq" 
##  [11] "hif"  "hil"  "hiu"  "hie"  "hiz"  "hik"  "hia"  "hih"  "hiw"  "hic" 
##  [21] "hix"  "hpr"  "hay"  "hpit" "hhz"  "haeg" "hpaa" "hap"  "hpaz" "hpas"
##  [31] "hpak" "gle"  "hso"  "hsm"  "pmu"  "pmv"  "pul"  "pmp"  "pmul" "pdag"
##  [41] "psky" "msu"  "bsun" "mht"  "mhq"  "mhat" "mhx"  "mhae" "mham" "mhao"
##  [51] "mhal" "mhaq" "mhay" "mvr"  "mann" "apl"  "apj"  "apa"  "asu"  "asi" 
##  [61] "ass"  "aeu"  "apor" "alig" "aap"  "aaz"  "aat"  "aao"  "aan"  "aah" 
##  [71] "aacn" "aact" "aseg" "apag" "avt"  "rpne" "ooi"  "bhud" "paet" "xfa" 
##  [81] "xft"  "xfm"  "xfn"  "xff"  "xfl"  "xfs"  "xfh"  "xtw"  "pade" "msx"
```

```
##  [91] "cbs"  "cbg"  "cbc"  "mmt"  "ftu"  "ftq"  "ftf"  "ftw"  "ftr"  "ftt"
## [101] "ftg"  "ftl"  "fth"  "fta"  "fts"  "fti"  "fto"  "ftc"  "ftv"  "ftz"
## [111] "ftm"  "ftn"  "ftx"  "ftd"  "fty"  "fcf"  "fcn"  "fhi"  "fph"  "fpt"
## [121] "fpi"  "fpm"  "fpx"  "fpz"  "fpj"  "frt"  "fna"  "fnl"  "frf"  "fper"
## [131] "fha"  "frx"  "frm"  "frc"  "fad"  "fmi"  "foo"  "fgu"  "afri" "aii"
## [141] "tcx"  "htr"  "hmar" "tcy"  "tao"  "thio" "thig" "tse"  "tzo"  "ntt"
## [151] "ttp"  "ntg"  "tkm"  "tvr"  "hna"  "ghl"  "zpl"  "chj"  "gap"  "fpp"
## [161] "tho"  "rev"  "rma"  "reo"  "vok"  "ebh"  "enm"  "nme"  "nmp"  "nmh"
## [171] "nmd"  "nmm"  "nms"  "nmq"  "nmz"  "nma"  "nmw"  "nmx"  "nmc"  "nmn"
## [181] "nmt"  "nmi"  "ngo"  "ngk"  "nla"  "nel"  "nsi"  "nmj"  "nei"  "nek"
## [191] "nfv"  "nsf"  "nzo"  "ncz"  "nbc"  "kki"  "koa"  "ecor" "eex"  "smur"
## [201] "aff"  "cste" "mcys" "teq"  "tea"  "teg"  "tas"  "tat"  "bpsi" "cbx"
## [211] "oto"  "ofo"  "sutk" "bbag" "bbay" "tbd"  "mfa"  "mmb"  "mbac" "mbat"
## [221] "gca"  "fam"  "nim"  "sdr"  "sulf" "splb" "slac" "fpho" "fmy"  "hpy"
## [231] "heo"  "hpj"  "hpa"  "hps"  "hhp"  "hhq"  "hhr"  "hpg"  "hpp"  "hpb"
## [241] "hpl"  "hpc"  "hca"  "hpm"  "hpe"  "hpo"  "hpi"  "hpq"  "hpw"  "hpu"
## [251] "hef"  "hpf"  "heq"  "hex"  "hpt"  "hpz"  "hpv"  "hpx"  "hen"  "hph"
## [261] "heg"  "hpn"  "hep"  "heu"  "hes"  "hpys" "hcn"  "hpd"  "hey"  "her"
## [271] "hei"  "hpya" "hpyk" "hpyo" "hpyl" "hpyb" "hpyc" "hpyd" "hpye" "hpyf"
## [281] "hpyg" "hpyh" "hpyj" "hpyr" "hpyi" "hpyu" "hpym" "hem"  "heb"  "hez"
## [291] "hhe"  "hac"  "hms"  "hfe"  "hbi"  "hce"  "hcm"  "hcp"  "hcb"  "hhm"
## [301] "hty"  "hbl"  "het"  "hcl"  "hwi"  "wsu"  "tdn"  "sua"  "suln" "sulg"
## [311] "sulc" "spal" "sku"  "sulr" "cje"  "cjb"  "cjj"  "cju"  "cjn"  "cji"
## [321] "cjm"  "cjs"  "cjp"  "cjej" "cjeu" "cjen" "cjei" "cjer" "cjv"  "cjy"
## [331] "cjq"  "cjl"  "cjw"  "cjr"  "cjd"  "cjz"  "cjx"  "cff"  "cft"  "cfv"
## [341] "cfx"  "cfz"  "camp" "cfp"  "ccv"  "cha"  "cco"  "ccoc" "cla"  "clr"
## [351] "clm"  "clq"  "cln"  "cll"  "ccol" "ccc"  "ccq"  "ccf"  "ccy"  "ccoi"
## [361] "ccof" "ccoo" "caj"  "cis"  "cvo"  "cpel" "camr" "csm"  "csf"  "cgra"
## [371] "cure" "chyo" "chv"  "cspf" "cpin" "ccun" "clx"  "cavi" "chw"  "camz"
## [381] "camy" "coj"  "cux"  "crx"  "cgeo" "cbla" "ccor" "carm" "cmuc" "csho"
## [391] "abu"  "abt"  "abl"  "ask"  "atp"  "acib" "acre" "alan" "apoc" "afc"
## [401] "acaa" "amol" "apai" "hbv"  "sdl"  "sba"  "smul" "suls" "sulj" "sult"
## [411] "hyo"  "nsa"  "sun"  "slh"  "nis"  "nam"  "nap"  "cmed" "cpaf" "gsu"
## [421] "gsk"  "glo"  "gem"  "gpi"  "gao"  "gsb"  "pca"  "pace" "ppd"  "dvu"
```

```
## [431] "dvl"  "dvm"  "dvg"  "dde"  "dds"  "dma"  "dgg"  "dfi"  "dpg"  "def" 
## [441] "dtr"  "dfl"  "dcb"  "dms"  "dsd"  "dsa"  "daf"  "das"  "dpi"  "dej" 
## [451] "pprf" "psel" "ddn"  "lip"  "lir"  "dsx"  "dba"  "doa"  "drt"  "dps" 
## [461] "dak"  "dpr"  "deo"  "dog"  "dsf"  "sat"  "dao"  "dax"  "hmr"  "rbt" 
## [471] "lcc"  "bhe"  "bhn"  "bhs"  "bqu"  "bqr"  "btr"  "btx"  "bgr"  "bvn" 
## [481] "barw" "bez"  "barn" "bky"  "bals" "thd"  "sdo"  "zmo"  "zmn"  "zmm" 
## [491] "zmb"  "zmi"  "zmc"  "zmr"  "zmp"  "gox"  "goh"  "goy"  "gal"  "apt" 
## [501] "apw"  "asv"  "aper" "apom" "aasc" "acet" "aot"  "abg"  "kba"  "nch" 
## [511] "coq"  "comm" "ntn"  "neh"  "ssam" "swf"  "bob"  "bomb" "pbr"  "mgm" 
## [521] "afr"  "afe"  "acu"  "acz"  "afi"  "afj"  "atx"  "afl"  "axl"  "tap" 
## [531] "aqt"  "stea" "sau"  "sav"  "saw"  "sah"  "saj"  "sam"  "sas"  "sar" 
## [541] "sac"  "sax"  "saa"  "sao"  "sae"  "sad"  "suu"  "suv"  "suj"  "suk" 
## [551] "suc"  "sut"  "suq"  "suz"  "sud"  "sux"  "suw"  "sug"  "saua" "saue"
## [561] "saun" "saus" "sauu" "saug" "sauz" "saut" "sauj" "sauk" "sauq" "sauv"
## [571] "sauw" "saux" "sauy" "sauf" "sab"  "suy"  "saub" "saum" "sauc" "saur"
## [581] "saui" "saud" "sams" "suh"  "ser"  "sep"  "sepp" "seps" "slg"  "sln" 
## [591] "spas" "scap" "spet" "scoh" "shom" "smus" "ssh"  "ssim" "sff"  "mcl" 
## [601] "mcak" "macr" "sbac" "jea"  "lwi"  "lgz"  "eat"  "psab" "pdu"  "pyg" 
## [611] "saca" "pgq"  "lla"  "llk"  "llt"  "lls"  "lld"  "llx"  "llj"  "llm" 
## [621] "llc"  "llr"  "lln"  "lli"  "llw"  "lgr"  "lgv"  "lpk"  "lrn"  "lact"
## [631] "lack" "spy"  "spz"  "spym" "spya" "spm"  "spg"  "sps"  "sph"  "spi" 
## [641] "spj"  "spk"  "spf"  "spa"  "spb"  "stg"  "stx"  "soz"  "stz"  "spyh"
## [651] "spn"  "spd"  "spr"  "spw"  "sjj"  "snv"  "spx"  "snt"  "snd"  "spnn"
## [661] "sne"  "spv"  "snc"  "snm"  "spp"  "sni"  "spng" "snb"  "snp"  "snx" 
## [671] "snu"  "spne" "spnu" "spnm" "spno" "sag"  "san"  "sak"  "sgc"  "sags"
## [681] "sagl" "sagm" "sagi" "sagr" "sagp" "sagc" "sagt" "sage" "sagg" "sagn"
## [691] "smu"  "smc"  "smut" "smj"  "smua" "stc"  "stl"  "ste"  "stn"  "stu" 
## [701] "stw"  "sthe" "sths" "ssa"  "ssb"  "ssu"  "ssv"  "ssi"  "sss"  "ssf" 
## [711] "ssw"  "sup"  "ssus" "sst"  "ssuy" "ssk"  "ssq"  "sui"  "suo"  "srp" 
## [721] "ssut" "ssui" "sgo"  "sez"  "seq"  "sezo" "sequ" "seu"  "sub"  "sds" 
## [731] "sdg"  "sda"  "sdc"  "sdq"  "sga"  "sgg"  "sgt"  "smb"  "sor"  "stk" 
## [741] "stb"  "scp"  "scf"  "ssr"  "stf"  "stj"  "strs" "ssah" "std"  "smn" 
## [751] "sif"  "sie"  "sib"  "siu"  "sang" "sanc" "sans" "scg"  "scon" "scos"
## [761] "soi"  "sik"  "siq"  "sio"  "siz"  "slu"  "sig"  "sip"  "stv"  "spat"
```

```
##  [771] "stra" "strn" "ssob" "srq"  "seqi" "ski"  "spei" "srat" "sgw"  "splr"
##  [781] "strg" "ljh"  "ljn"  "lde"  "lhr"  "lcr"  "lpw"  "lca"  "lcz"  "lcs"
##  [791] "lce"  "lcw"  "lpq"  "lpi"  "lpap" "lcb"  "lcx"  "lrh"  "lrg"  "lrl"
##  [801] "lra"  "lro"  "lrc"  "lpl"  "lpj"  "lpt"  "lps"  "lpr"  "lpz"  "lpb"
##  [811] "lpx"  "lpg"  "lre"  "lrf"  "lru"  "lrt"  "lrr"  "lfe"  "lfr"  "lff"
##  [821] "lmu"  "lor"  "lfn"  "lng"  "lhw"  "lmal" "lbh"  "lbn"  "lpar" "lcu"
##  [831] "lkf"  "lhil" "lbr"  "lbk"  "lko"  "lzy"  "lsl"  "lsi"  "lsj"  "lrm"
##  [841] "lagl" "laca" "lani" "lbt"  "lcy"  "lho"  "lol"  "lnn"  "lpd"  "pce"
##  [851] "pdm"  "paci" "lros" "lgn"  "lhi"  "lct"  "lalw" "lali" "lfm"  "lzh"
##  [861] "lft"  "lsa"  "lcv"  "lgm"  "lbm"  "lhb"  "ooe"  "oen"  "osi"  "lme"
##  [871] "lmm"  "lmk"  "lci"  "lki"  "lec"  "lcn"  "lgs"  "lge"  "llf"  "lgc"
##  [881] "lsu"  "lpse" "wcb"  "wjo"  "wpa"  "wso"  "whe"  "wei"  "efa"  "efl"
##  [891] "efi"  "efd"  "efs"  "efn"  "efq"  "ene"  "efc"  "efau" "efu"  "efm"
##  [901] "eft"  "ehr"  "ecas" "edu"  "ega"  "eth"  "eav"  "esg"  "thl"  "tey"
##  [911] "too"  "tkr"  "vte"  "vpi"  "vac"  "vao"  "vcp"  "aur"  "aun"  "aui"
##  [921] "asan" "avs"  "auh"  "caw"  "carc" "cdj"  "carn" "marr" "jep"  "jda"
##  [931] "jeh"  "jar"  "jpo"  "cpe"  "cpf"  "cpr"  "ctc"  "ctet" "cno"  "cbo"
##  [941] "cba"  "cbh"  "cby"  "cbl"  "cbk"  "cbb"  "cbi"  "cbn"  "cbt"  "cbf"
##  [951] "cbm"  "cbj"  "ckl"  "ckr"  "clj"  "ccb"  "cls"  "clb"  "cah"  "clt"
##  [961] "cbv"  "cld"  "ctae" "cchv" "carg" "cia"  "csep" "ccoh" "cfer" "aoe"
##  [971] "hhw"  "cale" "crs"  "cazo" "sarj" "cth"  "ctx"  "ccl"  "hsc"  "ruk"
##  [981] "rbp"  "cce"  "css"  "csd"  "cthd" "esr"  "esu"  "ccel" "fpla" "eha"
##  [991] "ral"  "rch"  "rum"  "rus"  "ruj"  "fpr"  "fpa"  "fpra" "ova"  "obj"
## [1001] "bpb"  "bfi"  "bhu"  "cle"  "cew"  "rho"  "rix"  "rim"  "coo"  "cct"
## [1011] "rob"  "byl"  "bhan" "blau" "blab" "cpy"  "csci" "csh"  "cso"  "bprl"
## [1021] "acac" "hsd"  "cpro" "lua"  "ehl"  "pxv"  "acel" "eel"  "rto"  "rgn"
## [1031] "ere"  "ert"  "era"  "cdf"  "pdc"  "pdf"  "eac"  "cst"  "psor" "roc"
## [1041] "phx"  "swo"  "slp"  "salq" "drm"  "dca"  "dru"  "dfg"  "dae"  "dku"
## [1051] "pth"  "dau"  "tjr"  "sgy"  "ded"  "dec"  "drs"  "hmo"  "hcv"  "elm"
## [1061] "emt"  "elim" "awo"  "cthm" "cmiu" "ibu"  "amij" "amic" "euu"  "bprs"
## [1071] "cbar" "tte"  "tex"  "thx"  "tpd"  "tit"  "tmt"  "tbo"  "twi"  "tki"
## [1081] "mta"  "mtho" "mthz" "adg"  "tpz"  "csc"  "ate"  "cob"  "chd"  "cow"
## [1091] "cki"  "ckn"  "clc"  "ccha" "ttm"  "tto"  "txy"  "tsh"  "tnr"  "taci"
## [1101] "mas"  "tep"  "tae"  "toc"  "nth"  "hor"  "has"  "hpk"  "hals" "aar"
```

```
## [1111] "aft"  "apr"  "ped"  "cad"  "spoa" "kpar" "vpr"  "vat"  "vrm"  "vdn"
## [1121] "vnk"  "med"  "mhw"  "dho"  "ssg"  "sri"  "sele" "selo" "selt" "mhg"
## [1131] "mfun" "mana" "afn"  "ain"  "pfac" "erh"  "erl"  "erd"  "eio"  "euc"
## [1141] "fro"  "aarg" "absi" "erm"  "fit"  "ebm"  "tur"  "tsg"  "abra" "tbm"
## [1151] "tbz"  "mle"  "mlb"  "cdi"  "cdp"  "cdt"  "cde"  "cdr"  "cda"  "cdz"
## [1161] "cdb"  "cdd"  "cdw"  "cdv"  "cpl"  "cpg"  "cpp"  "cpk"  "cpq"  "cpx"
## [1171] "cpz"  "cor"  "cop"  "cod"  "cos"  "coi"  "coe"  "cou"  "cpse" "cpsu"
## [1181] "cpsf" "cul"  "cuc"  "cue"  "cun"  "cus"  "cuq"  "cuz"  "cuj"  "cax"
## [1191] "cku"  "cut"  "crf"  "crl"  "ccho" "cpso" "cbq"  "lxx"  "rla"  "rpla"
## [1201] "aqg"  "rtx"  "cqf"  "psai" "rmu"  "rdn"  "raj"  "rter" "dva"  "djj"
## [1211] "jde"  "dco"  "pac"  "pak"  "pav"  "pax"  "paz"  "paw"  "pad"  "pcn"
## [1221] "pacc" "pach" "pacn" "cacn" "pra"  "cgrn" "prl"  "ppc"  "mcu"  "tpy"
## [1231] "tpyo" "tbw"  "asg"  "actt" "amy"  "soo"  "acq"  "aos"  "ard"  "actp"
## [1241] "actc" "acto" "ane"  "ahw"  "actz" "air"  "asla" "avc"  "avu"  "wik"
## [1251] "blo"  "blj"  "bln"  "blon" "blf"  "bll"  "blb"  "blm"  "blk"  "blg"
## [1261] "blz"  "blx"  "bad"  "badl" "bado" "bla"  "blc"  "blt"  "bbb"  "bbc"
## [1271] "bnm"  "blv"  "blw"  "bls"  "bani" "banl" "bni"  "banm" "bde"  "bdn"
## [1281] "bbp"  "bbi"  "bbf"  "bbv"  "bbru" "bbre" "bbrv" "bbrj" "bbrc" "bbrn"
## [1291] "bbrs" "bbrd" "bast" "btp"  "bcor" "bka"  "bks"  "bcat" "bpsp" "bii"
## [1301] "bang" "bpsc" "bsca" "bcho" "bgx"  "blem" "beu"  "gvg"  "gvh"  "plan"
## [1311] "plak" "plim" "psuf" "pvs"  "pvn"  "abam" "nhi"  "nab"  "ccu"  "shi"
## [1321] "ele"  "eyy"  "aeq"  "ddt"  "cbac" "ols"  "olo"  "pcat" "caer" "syc"
## [1331] "syf"  "syw"  "syd"  "sye"  "syg"  "syr"  "syx"  "syp"  "cya"  "cyb"
## [1341] "syne" "synp" "synk" "synr" "synd" "syu"  "syh"  "synw" "slw"  "syv"
## [1351] "syl"  "sync" "tel"  "thn"  "tvn"  "thec" "cgc"  "cyi"  "dsl"  "cmp"
## [1361] "lep"  "let"  "hhg"  "pseu" "pser" "pma"  "pmm"  "pmt"  "pmn"  "pmi"
## [1371] "pmb"  "pmc"  "pmf"  "pmg"  "pmh"  "pmj"  "pme"  "prc"  "prm"  "theu"
## [1381] "gen"  "gee"  "chon" "can"  "csn"  "cyl"  "hao"  "enn"  "cwa"  "ter"
## [1391] "gei"  "oac"  "oni"  "ana"  "nos"  "nop"  "naz"  "anb"  "acy"  "awa"
## [1401] "ann"  "csg"  "calo" "calt" "dou"  "ccur" "ceo"  "cer"  "mbf"  "det"
## [1411] "deh"  "deb"  "dev"  "deg"  "dmc"  "dmd"  "dmg"  "dmx"  "dmy"  "dmz"
## [1421] "duc"  "dly"  "dew"  "dfo"  "atm"  "abat" "psub" "abao" "ttr"  "tpar"
## [1431] "opr"  "ccz"  "pnl"  "puv"  "sng"  "ote"  "obg"  "vbh"  "caa"  "amu"
## [1441] "agl"  "min"  "mkc"  "meap" "vba"  "pir"  "ttf"  "plm"  "peh"  "kst"
```

```
## [1451] "broc" "phm"  "pcor" "pbu"  "pbp"  "pbas" "alus" "vbl"  "vbc"  "vai"
## [1461] "tsu"  "tbe"  "taz"  "tpi"  "scd"  "tphg" "trc"  "ssm"  "sta"  "stq"
## [1471] "sfc"  "sper" "sbu"  "scc"  "sgp"  "ock"  "tpx"  "bhy"  "bhd"  "brm"
## [1481] "bpo"  "bpj"  "bpip" "bpw"  "bip"  "bhp"  "trs"  "thyd" "emi"  "epo"
## [1491] "eti"  "rsd"  "fnu"  "fnc"  "fnt"  "fus"  "fne"  "fhw"  "fpd"  "fgo"
## [1501] "fnf"  "fpei" "lba"  "leo"  "lot"  "leq"  "lhf"  "lsz"  "lhg"  "lte"
## [1511] "lwd"  "lgo"  "str"  "tai"  "aco"  "tli"  "amo"  "fsu"  "fsc"  "bth"
## [1521] "btho" "bfr"  "bfs"  "bfg"  "bfb"  "bhl"  "bxy"  "boa"  "bcel" "bcac"
## [1531] "bcae" "bzg"  "bhf"  "bis"  "bun"  "bvu"  "bsa"  "bdh"  "pgi"  "pgn"
## [1541] "pgt"  "pah"  "pcre" "pcag" "pbt"  "pmuc" "psac" "dys"  "ppn"  "pdi"
## [1551] "parc" "tfo"  "toh"  "pary" "dun"  "bvs"  "copr" "osp"  "buy"  "aps"
## [1561] "pru"  "pmz"  "pdn"  "pit"  "pdt"  "pro"  "pfus" "peo"  "pje"  "poc"
## [1571] "alq"  "afd"  "ash"  "ald"  "aok"  "acou" "ada"  "ait"  "rbc"  "ttz"
## [1581] "blq"  "bacc" "asx"  "arb"  "fps"  "coc"  "ccm"  "col"  "chg"  "capn"
## [1591] "cgh"  "clk"  "cspu" "ccyn" "caph" "csto" "capq" "capf" "ran"  "rai"
## [1601] "rar"  "rag"  "rae"  "rat"  "orh"  "ori"  "bcad" "apib" "civ"  "ise"
## [1611] "cte"  "cpc"  "clz"  "cch"  "cph"  "cpb"  "cli"  "pvi"  "plt"  "pph"
## [1621] "paa"  "proc" "prs"  "pros" "cts"  "caci" "aae"  "hya"  "hho"  "hys"
## [1631] "hth"  "hte"  "tal"  "trd"  "sul"  "saf"  "pmx"  "ttk"  "tam"  "dte"
## [1641] "tma"  "tmm"  "tmi"  "tmw"  "tmq"  "tmx"  "tpt"  "trq"  "tna"  "tnp"
## [1651] "thq"  "thz"  "thr"  "tle"  "tta"  "phy"  "tme"  "taf"  "thp"  "ther"
## [1661] "fno"  "fpe"  "fia"  "ocy"  "pmo"  "mpz"  "marn" "dtn"  "kol"  "kpf"
## [1671] "mpg"  "minf" "asac" "cpo"  "din"  "ddf"  "dap"  "cni"  "fsi"  "gtl"
## [1681] "dth"  "dtu"  "tye"  "lfc"  "lfi"  "lfp"  "leg"  "tid"  "top"  "tcm"
## [1691] "thet" "cthi" "tav"  "tmai" "mox"  "mfe"  "mjh"  "mig"  "mmp"  "mmq"
## [1701] "mmx"  "mmz"  "mmd"  "mmak" "mmao" "mmad" "mae"  "mvn"  "mok"  "mth"
## [1711] "mmg"  "metc" "mwo"  "mete" "metz" "metk" "mthm" "mst"  "metb" "mru"
## [1721] "msi"  "meb"  "mmil" "meye" "mol"  "mel"  "mew"  "meth" "mfc"  "mfi"
## [1731] "mcub" "msub" "metn" "mett" "meto" "mfv"  "mka"  "afu"  "afg"  "apo"
## [1741] "ave"  "ast"  "fpl"  "gac"  "gah"  "pfu"  "pfi"  "pab"  "pyn"  "pys"
## [1751] "tko"  "ton"  "tga"  "tsi"  "tba"  "tlt"  "thv"  "tch"  "tce"  "tbs"
## [1761] "mba"  "mby"  "mbw"  "mbar" "mbak" "mac"  "mma"  "mmaz" "mmj"  "mmac"
## [1771] "mvc"  "mek"  "mls"  "metm" "mef"  "meq"  "msj"  "msz"  "msw"  "mthr"
## [1781] "mthe" "mhor" "mfz"  "mbu"  "mmet" "mmh"  "mhaz" "mev"  "mzh"  "mpy"
```

```
## [1791] "mzi"  "mhz"  "mtp"  "mcj"  "mhi"  "mhu"  "mla"  "mem"  "mbg"  "mema"
## [1801] "mpi"  "mbn"  "mfo"  "mpl"  "mpd"  "mez"  "rci"  "hal"  "halh" "hhsr"
## [1811] "hsu"  "hsf"  "hut"  "hti"  "hala" "harc" "haq"  "hdf"  "tac"  "tvo"
## [1821] "pto"  "tar"  "max"  "mer"  "marc" "ape"  "acj"  "iis"  "iag"  "pfm"
## [1831] "pdl"  "soh"  "sia"  "sim"  "siy"  "mse"  "mcn"  "aho"  "asul" "aamb"
## [1841] "sazo" "step" "pai"  "pis"  "pcl"  "pas"  "pyr"  "pyw"  "cma"  "ttn"
## [1851] "tuz"  "vdi"  "clg"  "nmr"  "nir"  "nkr"  "nid"  "nin"  "niw"  "ncl"
## [1861] "nox"  "nue"  "nct"  "csy"  "nga"  "nvn"  "nev"  "taa"  "nfn"  "ncv"
## [1871] "csu"  "nbv"  "tah"  "ndv"  "kcr"
```

Organisms classified within cluster 5

```
##    [1] "eco"  "ecj"  "ecd"  "ebw"  "ecok" "ece"  "ecs"  "ecf"  "etw"  "elx"
##   [11] "eoi"  "eoj"  "eoh"  "ecoo" "ecoh" "esl"  "eso"  "esm"  "eck"  "ecg"
##   [21] "eok"  "elr"  "elh"  "ecw"  "eun"  "ecp"  "ena"  "ecos" "ecv"  "ecoa"
##   [31] "ecx"  "ecm"  "ecy"  "ecr"  "ecq"  "eum"  "ect"  "eoc"  "ebr"  "ebl"
##   [41] "ebe"  "ebd"  "eci"  "eih"  "ecz"  "ecc"  "elo"  "eln"  "ese"  "ecl"
##   [51] "eko"  "ekf"  "eab"  "edh"  "edj"  "elu"  "elw"  "ell"  "elc"  "eld"
##   [61] "elp"  "elf"  "ecol" "ecoi" "ecoj" "efe"  "eal"  "ema"  "esz"  "sty"
##   [71] "stt"  "sex"  "sent" "stm"  "seo"  "sev"  "sey"  "sem"  "sej"  "seb"
##   [81] "sef"  "setu" "setc" "senr" "send" "seni" "seen" "spt"  "sek"  "spq"
##   [91] "sei"  "sec"  "seh"  "shb"  "senh" "seeh" "see"  "senn" "sew"  "sea"
##  [101] "sens" "sed"  "seg"  "sel"  "sega" "set"  "sena" "seno" "senv" "senq"
##  [111] "senl" "senj" "seec" "seeb" "seep" "senb" "sene" "senc" "ses"  "sbg"
##  [121] "sbz"  "sbv"  "salz" "sfx"  "sfv"  "sfe"  "sfn"  "sfs"  "sft"  "ssn"
##  [131] "sbo"  "sbc"  "shq"  "enc"  "enl"  "eclg" "ecle" "ecln" "ecli" "eclx"
##  [141] "ecly" "eclz" "eclo" "ehm"  "exf"  "ecla" "eclc" "eau"  "ekb"  "eno"
##  [151] "eec"  "elg"  "ecan" "ern"  "ecls" "echg" "esh"  "ent"  "eas"  "enr"
##  [161] "enx"  "enf"  "ebg"  "end"  "esa"  "csk"  "csz"  "csj"  "ccon" "cdm"
##  [171] "csi"  "cmj"  "cui"  "cmw"  "ctu"  "kpn"  "kpu"  "kpm"  "kpp"  "kph"
##  [181] "kpz"  "kpv"  "kpw"  "kpy"  "kpg"  "kpc"  "kpq"  "kpt"  "kpo"  "kpr"
##  [191] "kpj"  "kpi"  "kpa"  "kps"  "kpx"  "kpb"  "kpne" "kpnu" "kpnk" "kva"
##  [201] "kpe"  "kpk"  "kvd"  "kvq"  "kox"  "koe"  "koy"  "kom"  "kmi"  "kok"
```

```
##  [211] "koc"  "kqu"  "eae"  "ear"  "kqv"  "kll"  "klw"  "cro"  "cko"  "cfd"
##  [221] "cbra" "cwe"  "cyo"  "cpot" "cfq"  "cama" "caf"  "cif"  "cfar" "cir"
##  [231] "cie"  "cpar" "ebt"  "ror"  "ron"  "rpln" "rao"  "rtg"  "ree"  "cnt"
##  [241] "cem"  "cen"  "clap" "pge"  "esc"  "kle"  "ksa"  "kor"  "krd"  "kco"
##  [251] "kot"  "kpse" "kie"  "kas"  "lax"  "lei"  "leh"  "lee"  "ler"  "lea"
##  [261] "laz"  "lef"  "lni"  "lew"  "buf"  "bage" "mety" "ahn"  "yre"  "sgoe"
##  [271] "kin"  "pdz"  "ebf"  "ebc"  "ebu"  "izh"  "ype"  "ypk"  "yph"  "ypa"
##  [281] "ypn"  "ypm"  "ypp"  "ypg"  "ypz"  "ypt"  "ypd"  "ypx"  "ypw"  "ypj"
##  [291] "ypv"  "ypl"  "yps"  "ypo"  "ypi"  "ypy"  "ypb"  "ypq"  "ypu"  "ypr"
##  [301] "ypc"  "ypf"  "yen"  "yep"  "yey"  "yew"  "yet"  "yef"  "yee"  "ysi"
##  [311] "yal"  "yfr"  "yin"  "ykr"  "yro"  "yma"  "yhi"  "yca"  "ymo"  "smar"
##  [321] "smac" "smw"  "spe"  "srr"  "srl"  "sry"  "sply" "srs"  "sra"  "smaf"
##  [331] "slq"  "serf" "sers" "sfw"  "sfg"  "srz"  "sera" "serq" "serm" "squ"
##  [341] "sfj"  "sof"  "ssur" "sfo"  "rah"  "raq"  "raa"  "rox"  "gqu"  "eame"
##  [351] "rbad" "eca"  "patr" "pato" "pct"  "pcc"  "pcv"  "pwa"  "ppar" "pec"
##  [361] "pws"  "ppoa" "pbra" "ppuj" "ddd"  "dda"  "dze"  "ddc"  "dzc"  "dso"
##  [371] "ced"  "dfn"  "ddq"  "daq"  "dic"  "bgj"  "brb"  "bng"  "eam"  "eay"
##  [381] "eta"  "epy"  "epr"  "ebi"  "erj"  "ege"  "epe"  "erwi" "pam"  "plf"
##  [391] "paj"  "paq"  "pva"  "pagg" "pao"  "kln"  "pant" "panp" "pagc" "pstw"
##  [401] "palh" "pans" "pey"  "pdis" "pgz"  "pcd"  "mint" "mthi" "tci"  "plu"
##  [411] "plum" "pay"  "ptt"  "xne"  "xnm"  "xdo"  "xho"  "psi"  "psx"  "psta"
##  [421] "prg"  "pala" "phei" "prq"  "prj"  "pvc"  "hav"  "hpar" "opo"  "lri"
##  [431] "xcc"  "xcb"  "xca"  "xcp"  "xcv"  "xax"  "xac"  "xci"  "xct"  "xcj"
##  [441] "xcu"  "xcn"  "xcw"  "xcr"  "xcm"  "xcf"  "xfu"  "xao"  "xom"  "xoo"
##  [451] "xop"  "xoy"  "xor"  "xoz"  "xal"  "xsa"  "xtn"  "xfr"  "xve"  "xpe"
##  [461] "xhr"  "xga"  "xph"  "xva"  "xan"  "xar"  "xhy"  "xcz"  "xth"  "sml"
##  [471] "smt"  "buj"  "smz"  "sacz" "stek" "srh"  "slm"  "sten" "stem" "stes"
##  [481] "psu"  "psuw" "psd"  "pmex" "lab"  "laq"  "lcp"  "lgu"  "lez"  "lem"
##  [491] "lsol" "tbv"  "xbc"  "rhd"  "rgl"  "dji"  "dja"  "dtx"  "dko"  "lrz"
##  [501] "lpy"  "xba"  "rbd"  "vvu"  "vvy"  "vvm"  "vvl"  "vpa"  "vpb"  "vpk"
##  [511] "vpf"  "vph"  "vha"  "vca"  "vag"  "vex"  "vdb"  "vhr"  "vna"  "vow"
##  [521] "vro"  "vsp"  "vej"  "vfu"  "vni"  "vcy"  "vct"  "vtu"  "vfl"  "vmi"
##  [531] "vga"  "vsh"  "vta"  "vaf"  "vnl"  "vcc"  "vas"  "vaq"  "vsr"  "ppr"
##  [541] "pgb"  "gho"  "pae"  "paev" "paei" "pau"  "pap"  "pag"  "paf"  "pnc"
```

```
## [551] "paeb" "pdk"  "psg"  "prp"  "paep" "paer" "paem" "pael" "paes" "paeu"
## [561] "paeg" "paec" "paeo" "pmy"  "pmk"  "pre"  "ppse" "palc" "pcq"  "ppu"
## [571] "ppf"  "ppg"  "ppw"  "ppt"  "ppb"  "ppi"  "ppx"  "ppuh" "pput" "ppun"
## [581] "ppud" "pfv"  "pmon" "pmot" "pmos" "ppj"  "por"  "pst"  "psb"  "psyr"
## [591] "psp"  "pamg" "pci"  "pavl" "pvd"  "pfl"  "pprc" "ppro" "pfo"  "pfs"
## [601] "pfe"  "pfc"  "pfn"  "ppz"  "pfb"  "pman" "ptv"  "pcg"  "pvr"  "pazo"
## [611] "poi"  "pfw"  "pff"  "pfx"  "pen"  "psa"  "psz"  "psr"  "psc"  "psj"
## [621] "psh"  "pstu" "pstt" "pbm"  "plul" "pba"  "pbc"  "ppuu" "pdr"  "psv"
## [631] "psk"  "pkc"  "pch"  "pcz"  "pcp"  "pfz"  "plq"  "palk" "prh"  "psw"
## [641] "ppv"  "pses" "psem" "psec" "ppsy" "psos" "pkr"  "pfk"  "panr" "ppsl"
## [651] "pset" "psil" "pym"  "psed" "pke"  "pall" "pum"  "poj"  "pgg"  "ppsh"
## [661] "pgy"  "avn"  "avl"  "avd"  "acx"  "pagr" "pcr"  "prw"  "pso"  "pali"
## [671] "pspg" "psyg" "psyc" "psya" "psyy" "psyp" "acb"  "aby"  "abc"  "abn"
## [681] "abb"  "abx"  "abz"  "abr"  "abd"  "abh"  "abad" "abj"  "abab" "abaj"
## [691] "abaz" "abk"  "abau" "abaa" "abw"  "abal" "acc"  "ano"  "alc"  "acal"
## [701] "acd"  "aci"  "ajo"  "acw"  "asol" "ala"  "adv"  "arj"  "awu"  "agu"
## [711] "alw"  "ads"  "aber" "atn"  "achi" "sdn"  "sfr"  "saz"  "sbl"  "sbm"
## [721] "sbn"  "sbp"  "sbt"  "sbs"  "sbb"  "slo"  "spc"  "shp"  "sse"  "spl"
## [731] "she"  "shm"  "shn"  "shw"  "shl"  "swd"  "swp"  "svo"  "shf"  "sja"
## [741] "spsw" "sbj"  "smav" "shew" "salg" "slj"  "smai" "spol" "sbk"  "skh"
## [751] "saes" "ilo"  "ili"  "idt"  "cps"  "com"  "colw" "cola" "cber" "cov"
## [761] "lsd"  "tht"  "thap" "pha"  "ptn"  "pat"  "psm"  "pseo" "pia"  "pphe"
## [771] "pbw"  "prr"  "plz"  "paln" "ppis" "pea"  "pspo" "part" "ptu"  "png"
## [781] "ptd"  "psen" "pdj"  "paga" "pcar" "pmaa" "maq"  "mhc"  "mad"  "mbs"
## [791] "msr"  "mpq"  "mari" "mlq"  "msq"  "mara" "marj" "amc"  "amh"  "amaa"
## [801] "amal" "amae" "amao" "amad" "amai" "amag" "amac" "amb"  "amg"  "amk"
## [811] "alt"  "aal"  "aaus" "asp"  "asq"  "aaw"  "alr"  "ale"  "alz"  "apel"
## [821] "gag"  "gni"  "gps"  "pmes" "lal"  "salh" "salm" "salk" "hmi"  "pin"
## [831] "fbl"  "fes"  "mvs"  "mya"  "mmaa" "cell" "ttu"  "saga" "spoi" "zal"
## [841] "osg"  "mthd" "micc" "maga" "mii"  "mict" "mhyd" "hja"  "halc" "kim"
## [851] "lpo"  "lpu"  "lpm"  "lpe"  "llo"  "lfa"  "lha"  "lcd"  "lsh"  "llg"
## [861] "lib"  "ljr"  "lcj"  "lss"  "tmc"  "mmai" "rhh"  "woc"  "gai"  "hch"
## [871] "hahe" "csa"  "hel"  "hcs"  "hak"  "ham"  "hhu"  "hco"  "hsi"  "halo"
## [881] "hhh"  "hbe"  "hag"  "haf"  "halk" "hvn"  "hol"  "hsr"  "hmd"  "haxi"
```

```
##  [891] "htt"  "hcam" "hpiz" "haa"  "cmai" "paur" "abo"  "adi"  "apac" "aln" 
##  [901] "axe"  "mmw"  "mme"  "mpc"  "mpri" "mard" "mars" "bsan" "ncu"  "nik" 
##  [911] "bmar" "ajp"  "gsn"  "rfo"  "ome"  "aha"  "ahy"  "ahd"  "ahr"  "ahp" 
##  [921] "ahj"  "ahh"  "ahi"  "aaj"  "asa"  "aeo"  "avr"  "avo"  "amed" "asr" 
##  [931] "adh"  "aem"  "aea"  "arv"  "aes"  "ael"  "oce"  "zdf"  "sok"  "sini"
##  [941] "saln" "seds" "gpb"  "vff"  "cvi"  "cvc"  "chro" "chri" "chrb" "crz" 
##  [951] "chrm" "chae" "iod"  "ifl"  "pse"  "aql"  "amah" "aqs"  "rso"  "rsc" 
##  [961] "rsl"  "rsn"  "rsm"  "rse"  "rsy"  "rpi"  "rpf"  "rpj"  "rmn"  "rin" 
##  [971] "rpu"  "reh"  "cnc"  "cuh"  "reu"  "rme"  "cti"  "cbw"  "cgd"  "ccup"
##  [981] "cup"  "cuu"  "cpau" "cox"  "bma"  "bmv"  "bml"  "bmn"  "bmal" "bmae"
##  [991] "bmaq" "bmai" "bmaf" "bmaz" "bmab" "bps"  "bpm"  "bpl"  "bpd"  "bpr" 
## [1001] "bpse" "bpsm" "bpsu" "bpsd" "bpz"  "bpq"  "bpk"  "bpsh" "bpsa" "bpso"
## [1011] "but"  "bte"  "btq"  "btj"  "btz"  "btd"  "btv"  "bthe" "bthm" "btha"
## [1021] "bthl" "bok"  "boc"  "buu"  "bvi"  "bve"  "bur"  "bcn"  "bch"  "bcm" 
## [1031] "bcj"  "bcen" "bcew" "bceo" "bam"  "bac"  "bmj"  "bmu"  "bmk"  "bmul"
## [1041] "bct"  "bced" "bcep" "bdl"  "bpyr" "bcon" "bub"  "bdf"  "blat" "btei"
## [1051] "bsem" "bpsl" "bmec" "bstg" "bstl" "bgl"  "bgu"  "bug"  "bgf"  "bgd" 
## [1061] "bgo"  "byi"  "buk"  "bue"  "bul"  "buq"  "bgp"  "bpla" "bud"  "bum" 
## [1071] "bui"  "bxe"  "bxb"  "bph"  "bge"  "bpx"  "bpy"  "buz"  "bfn"  "bcai"
## [1081] "pspw" "para" "parb" "phs"  "pter" "pgp"  "pcj"  "pts"  "pcaf" "pmeg"
## [1091] "brh"  "ppk"  "ppno" "ppnm" "prb"  "ppul" "pspu" "papi" "pve"  "pox" 
## [1101] "ptx"  "pfg"  "pnr"  "pand" "pfib" "plg"  "caba" "buo"  "limn" "cari"
## [1111] "bpe"  "bpc"  "bper" "bpet" "bpeu" "bpar" "bpa"  "bbh"  "bbr"  "bbm" 
## [1121] "bbx"  "bpt"  "bav"  "bho"  "bhm"  "bhz"  "btrm" "bbro" "bfz"  "bpdz"
## [1131] "boh"  "bgm"  "boj"  "boz"  "axy"  "axo"  "axn"  "axx"  "adt"  "ais" 
## [1141] "asw"  "achr" "achb" "put"  "pus"  "aka"  "amim" "cdn"  "afa"  "afq" 
## [1151] "aaqu" "odi"  "pig"  "pacr" "kgy"  "rfr"  "rsb"  "rac"  "rhy"  "rhf" 
## [1161] "rhg"  "pol"  "pna"  "pos"  "poo"  "aav"  "ajs"  "dia"  "aaa"  "ack" 
## [1171] "acra" "acid" "acip" "acin" "acis" "acio" "amon" "vei"  "dac"  "del" 
## [1181] "dts"  "dhk"  "dla"  "vap"  "vpe"  "vpd"  "vaa"  "vbo"  "vam"  "ctt" 
## [1191] "ctes" "cser" "cof"  "adn"  "adk"  "rta"  "otk"  "lim"  "lih"  "hyr" 
## [1201] "hyb"  "hyl"  "hyc"  "hpse" "hyn"  "dpy"  "dih"  "daer" "drg"  "simp"
## [1211] "melm" "mela" "sthm" "mpt"  "metp" "mms"  "jag"  "jab"  "jaz"  "jal" 
## [1221] "jsv"  "jaj"  "jas"  "jlv"  "hse"  "hsz"  "hht"  "hrb"  "hee"  "hhf" 
```

```
## [1231] "hfr"  "cfu"  "care" "cpra" "mnr"  "masw" "mass" "masz" "mtim" "masy"
## [1241] "mali" "mum"  "mfla" "mpli" "upv"  "upi"  "nok"  "dug"  "lch"  "tin"
## [1251] "thi"  "rge"  "rbn"  "rdp"  "pkt"  "miu"  "rgu"  "aon"  "snn"  "xyk"
## [1261] "pbh"  "shd"  "metr" "uru"  "upl"  "eba"  "dsu"  "otr"  "dar"  "dey"
## [1271] "azo"  "aoa"  "aza"  "azi"  "acom" "azd"  "azr"  "azq"  "tmz"  "thu"
## [1281] "thk"  "app"  "beb"  "beba" "dov"  "dwd"  "dalk" "ade"  "acp"  "afw"
## [1291] "ank"  "mxa"  "msd"  "mym"  "mfb"  "ccx"  "mfu"  "mmas" "sur"  "age"
## [1301] "mbd"  "cfus" "scl"  "scu"  "ccro" "samy" "llu"  "mrm"  "hoh"  "mlo"
## [1311] "mln"  "mci"  "mop"  "mam"  "mamo" "meso" "mesw" "mesm" "mesp" "mhua"
## [1321] "mjr"  "merd" "mes"  "hoe"  "aak"  "amih" "pht"  "rpod" "niy"  "orm"
## [1331] "pla"  "rbs"  "sme"  "smk"  "smq"  "smx"  "smi"  "smeg" "smel" "smer"
## [1341] "smd"  "rhi"  "sfh"  "sfd"  "six"  "same" "sino" "ead"  "eah"  "esj"
## [1351] "eak"  "emx"  "atu"  "ara"  "ata"  "agr"  "atf"  "avi"  "agc"  "aro"
## [1361] "agt"  "alf"  "ret"  "rec"  "rel"  "rep"  "rei"  "rle"  "rlt"  "rlg"
## [1371] "rlb"  "rlu"  "rtr"  "rir"  "rpus" "rhl"  "rga"  "rhn"  "rpha" "rhx"
## [1381] "rhv"  "rhk"  "rez"  "rjg"  "rhr"  "rgr"  "rad"  "roy"  "rii"  "ngl"
## [1391] "ngg"  "neo"  "nen"  "rht"  "shz"  "abaw" "kai"  "bme"  "bmel" "bmi"
## [1401] "bmz"  "bmg"  "bmw"  "bmee" "bmf"  "bmb"  "bmc"  "baa"  "babo" "babr"
## [1411] "babt" "babb" "babu" "babs" "babc" "bms"  "bsi"  "bsf"  "bsui" "bsup"
## [1421] "bsuv" "bsuc" "bmt"  "bsz"  "bsv"  "bsw"  "bsg"  "bov"  "bcs"  "bsk"
## [1431] "bol"  "bcar" "bcas" "bmr"  "bpp"  "bpv"  "bcet" "bvl"  "bru"  "brj"
## [1441] "oin"  "oan"  "oah"  "ops"  "och"  "bja"  "bju"  "bjp"  "bra"  "bbt"
## [1451] "brs"  "aol"  "brc"  "brad" "bic"  "bro"  "brk"  "bot"  "brq"  "bgq"
## [1461] "bgz"  "bsym" "bbet" "barh" "bvz"  "rpa"  "rpb"  "rpc"  "rpd"  "rpe"
## [1471] "rpt"  "rpx"  "nha"  "oca"  "ocg"  "oco"  "bop"  "bos"  "bvv"  "boi"
## [1481] "bof"  "vgo"  "trb"  "bapi" "xau"  "azc"  "sno"  "star" "lne"  "anc"
## [1491] "apra" "mea"  "mdi"  "mex"  "mch"  "mpo"  "mza"  "mrd"  "met"  "mno"
## [1501] "mor"  "meta" "maqu" "mphy" "mee"  "metd" "metx" "meti" "mmes" "mtea"
## [1511] "moc"  "miv"  "mico" "bid"  "msl"  "mtun" "bbar" "chel" "cdq"  "hmc"
## [1521] "hni"  "phl"  "fil"  "fiy"  "deq"  "dei"  "dea"  "bvr"  "rhz"  "mmyr"
## [1531] "yti"  "ntd"  "msc"  "mros" "mhey" "mpar" "pleo" "mey"  "maad" "mmed"
## [1541] "aua"  "aala" "brn"  "psin" "hdi"  "noh"  "rbm"  "psf"  "pphr" "lap"
## [1551] "lagg" "labr" "labp" "labt" "siw"  "ccr"  "ccs"  "cak"  "cse"  "chq"
## [1561] "cmb"  "cfh"  "cauf" "pzu"  "bsb"  "brd"  "bne"  "brg"  "brl"  "bvc"
```

```
## [1571] "bdm"  "brf"  "brev" "bmed" "bvy"  "aex"  "tsv"  "cbot" "sil"  "sit"
## [1581] "rua"  "rut"  "rmb"  "rsp"  "rsh"  "rsk"  "rbl"  "jan"  "rde"  "rli"
## [1591] "rpon" "pde"  "pami" "pye"  "pzh"  "paro" "paru" "pamn" "pars" "parr"
## [1601] "pkd"  "ppan" "dsh"  "pga"  "pgl"  "pgd"  "php"  "ppic" "phq"  "oat"
## [1611] "oar"  "otm"  "lmd"  "lej"  "laqu" "red"  "ptp"  "cid"  "ceh"  "cmag"
## [1621] "malg" "con"  "rsu"  "rhm"  "rhc"  "hat"  "daa"  "ypac" "yan"  "tpro"
## [1631] "suam" "spse" "sulz" "suli" "suld" "spot" "don"  "tom"  "paby" "thw"
## [1641] "tec"  "rmm"  "rok"  "rid"  "rom"  "roh"  "lvs"  "aht"  "rbg"  "sagu"
## [1651] "thaa" "geh"  "taw"  "salo" "sedi" "hml"  "boo"  "pseb" "lit"  "ocd"
## [1661] "maru" "rot"  "ppru" "paed" "mon"  "malu" "tgl"  "pamo" "pshq" "poz"
## [1671] "palw" "ppaf" "pgv"  "rbz"  "thas" "faq"  "hdh"  "mmr"  "gak"  "hne"
## [1681] "hba"  "hbc"  "nar"  "npp"  "npn"  "nre"  "nov"  "not"  "nor"  "ngf"
## [1691] "nog"  "sal"  "sphk" "sphp" "smag" "smaz" "ster" "sgi"  "sphq" "spho"
## [1701] "sphx" "sphu" "swi"  "sphd" "sphm" "stax" "sphi" "ssan" "snj"  "smy"
## [1711] "span" "skr"  "splm" "splk" "spkc" "sphc" "sphf" "spau" "sech" "sjp"
## [1721] "sch"  "ssy"  "syb"  "sbd"  "spmi" "sphb" "sphr" "sinb" "spht" "shyd"
## [1731] "sya"  "sclo" "spyg" "sufl" "sami" "sbar" "cij"  "sphg" "sfla" "sphy"
## [1741] "blas" "bfw"  "rdi"  "sphj" "spzr" "palg" "smic" "sphs" "sand" "aay"
## [1751] "alb"  "alh"  "amx"  "anh"  "ado"  "cna"  "cman" "ery"  "egn"  "efv"
## [1761] "eli"  "elq"  "erk"  "err"  "erf"  "emv"  "pns"  "porl" "phz"  "pot"
## [1771] "acr"  "amv"  "rgi"  "ros"  "rmuc" "shum" "svc"  "rce"  "mag"  "magx"
## [1781] "magn" "azl"  "ali"  "abs"  "abq"  "abf"  "ati"  "azt"  "azm"  "azz"
## [1791] "aoz"  "tmo"  "txi"  "thac" "tii"  "nao"  "ncb"  "fer"  "htq"  "hadh"
## [1801] "skt"  "ecog" "phr"  "pstg" "blh"  "bae"  "bson" "bca"  "bcer" "bmyc"
## [1811] "bgy"  "bacq" "bfd"  "beo"  "bmq"  "bmd"  "bmh"  "bmeg" "bon"  "bko"
## [1821] "gya"  "gct"  "gmc"  "ggh"  "gjf"  "gth"  "ptl"  "lyg"  "vig"  "vil"
## [1831] "vne"  "vpn"  "bsj"  "bmur" "pbut" "sale" "pof"  "nmk"  "bbe"  "bfm"
## [1841] "pms"  "pmq"  "pmw"  "pnp"  "palb" "anx"  "aac"  "aad"  "bts"  "kyr"
## [1851] "siv"  "ssil" "spsy" "spor" "panc" "say"  "sap"  "mtj"  "mtue" "mpa"
## [1861] "mao"  "mavi" "mavu" "mav"  "mit"  "mia"  "mid"  "myo"  "mchi" "mir"
## [1871] "mmal" "msa"  "mul"  "mmc"  "mkm"  "mjl"  "mmi"  "mmae" "mmm"  "mli"
## [1881] "mkn"  "myv"  "mye"  "mdx"  "mshg" "mfj"  "mgro" "mnv"  "mpag" "mnm"
## [1891] "mgor" "mcoo" "msm"  "msg"  "msb"  "msn"  "msh"  "mva"  "mgi"  "msp"
## [1901] "mcb"  "mne"  "myn"  "mgo"  "mft"  "mphl" "mvq"  "mll"  "mrh"  "mthn"
```

```
## [1911] "mhas" "mdu"  "mcht" "mauu" "mmag" "mmor" "mfx"  "maic" "mij"  "malv"
## [1921] "mty"  "mpsc" "mab"  "mmv"  "mabb" "mabl" "mche" "miz"  "mste" "msao"
## [1931] "msal" "mjd"  "mter" "mhib" "asd"  "mkr"  "chn"  "cgy"  "camg" "nfa"
## [1941] "nfr"  "ncy"  "nbr"  "nno"  "nsl"  "nsr"  "ntp"  "noz"  "nod"  "nah"
## [1951] "nad"  "nwl"  "rha"  "rer"  "rey"  "reb"  "rop"  "roa"  "req"  "rpy"
## [1961] "rhb"  "rav"  "rfa"  "rhw"  "rhs"  "rrz"  "rhu"  "rqi"  "rhq"  "rhod"
## [1971] "rrt"  "rby"  "rcr"  "rtm"  "gbr"  "gpo"  "gor"  "goq"  "gta"  "goc"
## [1981] "git"  "gru"  "gom"  "gav"  "god"  "tpr"  "tsm"  "dtm"  "dit"  "diz"
## [1991] "dpc"  "toy"  "sco"  "salb" "sma"  "sgr"  "sgb"  "scb"  "ssx"  "svl"
## [2001] "sct"  "scy"  "sfa"  "sbh"  "shy"  "sho"  "sve"  "sdv"  "sals" "strp"
## [2011] "sfi"  "sci"  "src"  "salu" "sall" "slv"  "sgu"  "svt"  "stre" "scw"
## [2021] "sld"  "slc"  "sxi"  "strm" "strc" "samb" "spri" "scz"  "scx"  "srw"
## [2031] "strf" "sle"  "srn"  "spav" "strt" "sclf" "sgs"  "stsi" "sls"  "snr"
## [2041] "splu" "strd" "snw"  "sauo" "ssia" "svu"  "spun" "sgv"  "smal" "slau"
## [2051] "salf" "salj" "slx"  "stro" "sfk"  "snz"  "sge"  "srj"  "slk"  "sky"
## [2061] "sdx"  "sgd"  "sqz"  "scya" "sast" "snq"  "stir" "ska"  "sgz"  "svn"
## [2071] "snk"  "salw" "shaw" "srk"  "sfic" "sgal" "sspo" "svr"  "spad" "sfy"
## [2081] "saqu" "sgf"  "scav" "sseo" "ksk"  "kab"  "kau"  "kit"  "stri" "leif"
## [2091] "lse"  "mts"  "mio"  "mip"  "mcw"  "micr" "mhos" "mlv"  "mwa"  "mprt"
## [2101] "moy"  "agy"  "agm"  "cphy" "myl"  "lyd"  "ltr"  "hea"  "art"  "arr"
## [2111] "arm"  "arl"  "are"  "aaq"  "arw"  "arh"  "ary"  "arz"  "aru"  "arq"
## [2121] "arn"  "arx"  "acry" "arth" "artp" "acit" "aau"  "pue"  "ach"  "apn"
## [2131] "psul" "psni" "psey" "aai"  "gar"  "gcr"  "glu"  "kfv"  "krs"  "satk"
## [2141] "lmoi" "cceu" "ica"  "jte"  "jli"  "jme"  "pei"  "bly"  "bcau" "aus"
## [2151] "halt" "mph"  "rain" "nca"  "ndk"  "noy"  "noo"  "nsn"  "nano" "nmes"
## [2161] "psim" "aez"  "aeb"  "muz"  "kfl"  "kqi"  "nda"  "nal"  "ngv"  "strr"
## [2171] "tcu"  "actw" "sro"  "noa"  "now"  "fre"  "fri"  "fal"  "nml"  "gob"
## [2181] "bsd"  "mmar" "sen"  "sace" "sacg" "sacc" "amd"  "amn"  "amm"  "amz"
## [2191] "aoi"  "aja"  "amq"  "amyc" "amyb" "aab"  "amyy" "aori" "pdx"  "psea"
## [2201] "psee" "pseh" "pseq" "pecq" "phh"  "paut" "apre" "ami"  "sesp" "ssyi"
## [2211] "kal"  "kphy" "led"  "ahm"  "acti" "acad" "ahg"  "acta" "alo"  "pmad"
## [2221] "stp"  "saq"  "mau"  "mil"  "micb" "mtua" "mich" "mtem" "mcab" "msag"
## [2231] "vma"  "mcra" "ase"  "ams"  "actn" "afs"  "acts" "plk"  "plab" "plat"
## [2241] "pfla" "psuu" "ver"  "cai"  "sna"  "aey"  "rxy"  "bsol" "cwo"  "aym"
```

```
## [2251] "erz"  "euz"  "kbs"  "dpd"  "sdyn" "saci" "gma"  "talb" "abas" "sus" 
## [2261] "pfer" "abac" "gau"  "gba"  "cpi"  "chit" "chih" "nko"  "fgg"  "pgin"
## [2271] "lacs" "hhy"  "smiz" "muc"  "mgk"  "mrub" "mgos" "est"  "sli"  "srd" 
## [2281] "smon" "spir" "spik" "spib" "rsi"  "run"  "rup"  "eol"  "fae"  "fib" 
## [2291] "als"  "rhoz" "hyj"  "hrs"  "pact" "add"  "fbt"  "chk"  "grs"  "zpr" 
## [2301] "marm" "mart" "cao"  "cbal" "cbat" "zga"  "mut"  "ptq"  "prn"  "salt"
## [2311] "aalg" "chry" "halj" "hlr"  "nax"
```

Organisms classified within cluster 6

```
##   [1] "hsa"  "ptr"  "pps"  "ggo"  "pon"  "nle"  "mcc"  "mcf"  "csab" "caty"
##  [11] "panu" "rro"  "rbb"  "tfn"  "pteh" "cjc"  "sbq"  "mmur" "mmu"  "mcal"
##  [21] "mpah" "rno"  "mcoc" "mun"  "cge"  "pleu" "ngi"  "hgl"  "ccan" "ocu" 
##  [31] "opi"  "tup"  "cfa"  "vvp"  "vlg"  "aml"  "umr"  "uah"  "oro"  "elk" 
##  [41] "mpuf" "eju"  "mlx"  "fca"  "pyu"  "pbg"  "ptg"  "ppad" "aju"  "hhv" 
##  [51] "bta"  "bom"  "biu"  "bbub" "chx"  "oas"  "oda"  "ccad" "ssc"  "cfr" 
##  [61] "cbai" "cdk"  "bacu" "lve"  "oor"  "dle"  "pcad" "ecb"  "epz"  "eai" 
##  [71] "myb"  "myd"  "mmyo" "mna"  "pkl"  "hai"  "dro"  "shon" "ajm"  "pdic"
##  [81] "mmf"  "rfq"  "pale" "pgig" "ray"  "mjv"  "tod"  "lav"  "tmu"  "mdo" 
##  [91] "gas"  "shr"  "pcw"  "oaa"  "gga"  "pcoc" "mgp"  "cjo"  "nmel" "apla"
## [101] "acyg" "tgu"  "lsr"  "scan" "pmoa" "otc"  "pruf" "gfr"  "fab"  "phi" 
## [111] "pmaj" "ccae" "ccw"  "etl"  "fpg"  "fch"  "clv"  "egz"  "nni"  "acun"
## [121] "padl" "aam"  "arow" "npd"  "dne"  "asn"  "amj"  "cpoo" "ggn"  "pss" 
## [131] "cmy"  "cpic" "tst"  "cabi" "acs"  "pvt"  "sund" "pbi"  "pmur" "tsr" 
## [141] "pgut" "vko"  "pmua" "zvi"  "gja"  "xla"  "xtr"  "npr"  "dre"  "srx" 
## [151] "sanh" "sgh"  "ccar" "caua" "ipu"  "phyp" "amex" "eee"  "tru"  "tng" 
## [161] "lco"  "ncc"  "cgob" "ely"  "plep" "sluc" "ecra" "pflv" "gat"  "ppug"
## [171] "msam" "cud"  "mze"  "onl"  "oau"  "ola"  "oml"  "xma"  "xco"  "xhe" 
## [181] "pret" "cvg"  "ctul" "nfu"  "kmr"  "alim" "aoce" "csem" "pov"  "ssen"
## [191] "lcf"  "sdu"  "slal" "xgl"  "hcq"  "bpec" "malb" "sasa" "otw"  "omy" 
## [201] "salp" "snh"  "els"  "sfm"  "pki"  "aang" "loc"  "pspa" "arut" "lcm" 
## [211] "cmk"  "rtp"  "bfo"  "bbel" "cin"  "sclv" "spu"  "aplc" "sko"  "dme" 
## [221] "der"  "dse"  "dsi"  "dya"  "dan"  "dsr"  "dpo"  "dpe"  "dmn"  "dwi" 
```

```
## [231] "dgr"  "dmo"  "daz"  "dnv"  "dhe"  "dvi"  "ccat" "bod"  "mde"  "scac"
## [241] "lcq"  "aga"  "acoz" "aara" "aag"  "aalb" "cqu"  "cpii" "ame"  "acer"
## [251] "bim"  "bbif" "bvk"  "bvan" "bter" "ccal" "obb"  "mgen" "nmea" "cgig"
## [261] "soc"  "mpha" "aec"  "acep" "pbar" "vem"  "hst"  "dqu"  "cfo"  "fex"
## [271] "lhu"  "pgc"  "obo"  "pcf"  "pfuc" "vps"  "nvi"  "csol" "tpre" "mdl"
## [281] "cglo" "fas"  "dam"  "ccin" "tca"  "dpa"  "atd"  "agb"  "ldc"  "nvl"
## [291] "apln" "ppyr" "otu"  "bmor" "bman" "msex" "dpl"  "bany" "pmac" "ppot"
## [301] "pxu"  "prap" "zce"  "haw"  "tnl"  "pxy"  "api"  "dnx"  "ags"  "rmd"
## [311] "btab" "dci"  "clec" "hhal" "nlu"  "phu"  "foc"  "zne"  "csec" "fcd"
## [321] "dpx"  "dmk"  "pvm"  "pja"  "hame" "hazt" "eaf"  "isc"  "dsv"  "rsan"
## [331] "rmp"  "vde"  "vja"  "tut"  "dpte" "cscu" "ptep" "sdm"  "cel"  "cbr"
## [341] "loa"  "nai"  "hro"  "lgi"  "pcan" "bgt"  "gae"  "crg"  "myi"  "pmax"
## [351] "obi"  "osn"  "lak"  "ovi"  "nve"  "epa"  "aten" "adf"  "amil" "pdam"
## [361] "spis" "dgt"  "hmg"  "tad"  "aqu"
```

Weisfeiler-Lehman (WL) kernel

Heatmap

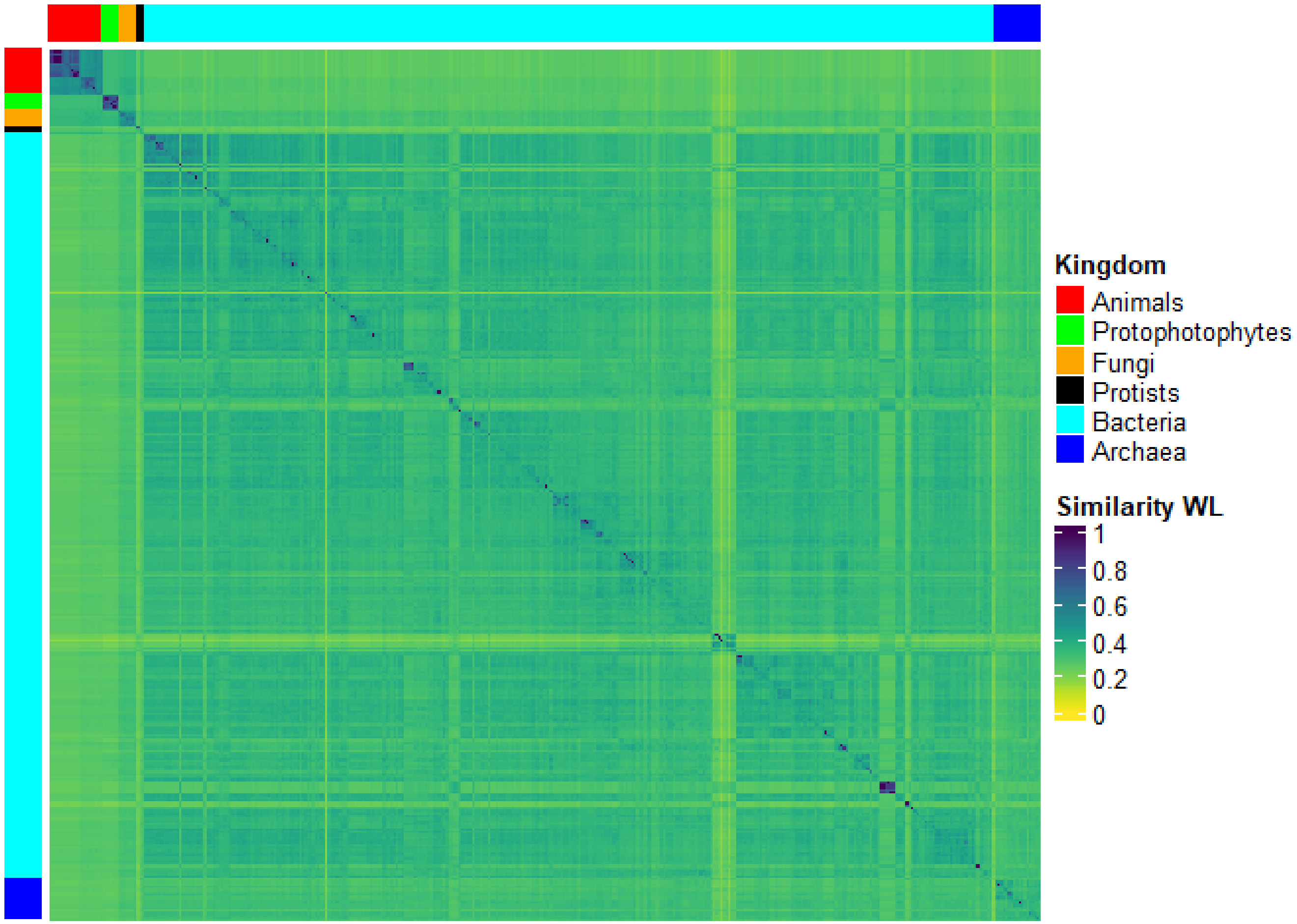
Kingdom
Animals
Protophotophytes
Fungi
Protists
Bacteria
Archaea
Similarity WL
1
0.8
0.6
0.4
0.2
0

MDS for WL

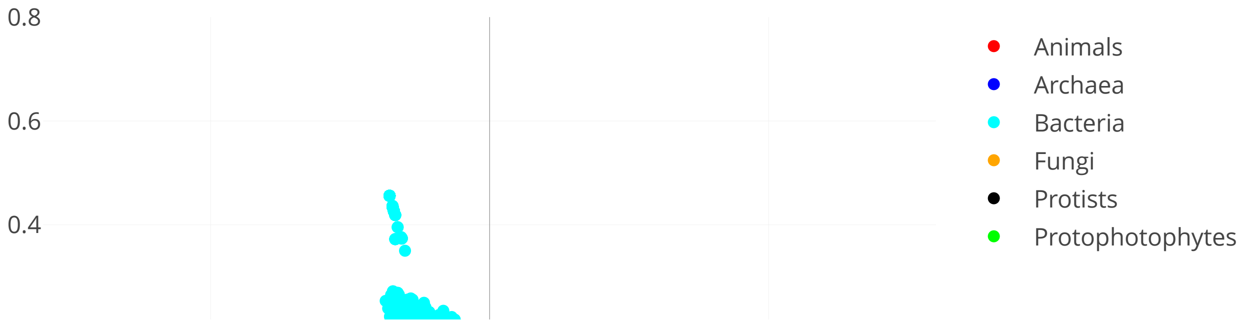
0.8
0.6
0.4
Animals
Archaea
Bacteria
Fungi
Protists
Protophotophytes

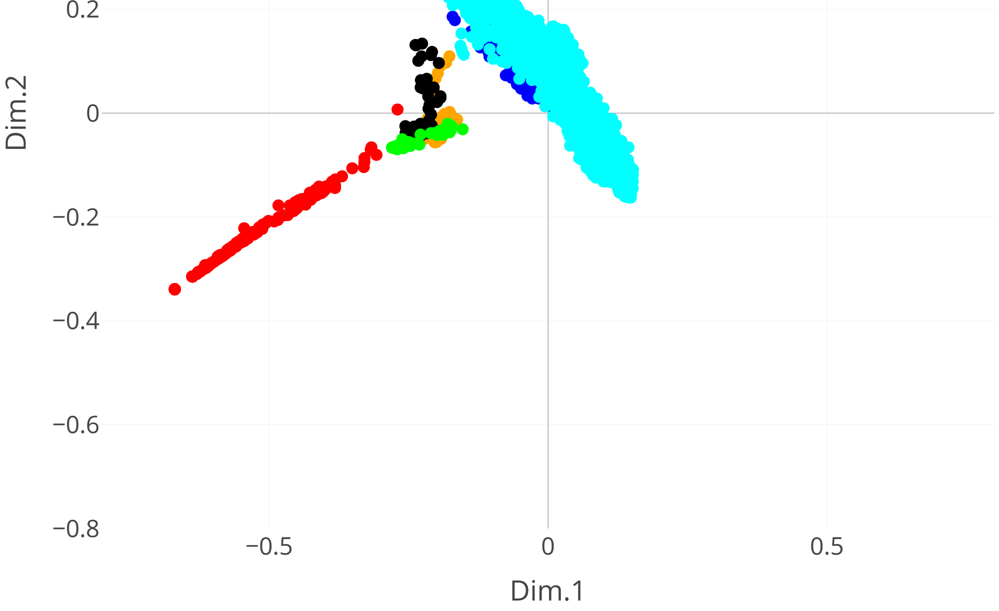

6-Means clustering for WL kernel

```
##                     Cluster
## Real group             1    2    3    4    5    6
##   Animals              0    0   19    0    0  351
##   Archaea            291    4    0    0   44    0
##   Bacteria          1683  430    0 2441 1561    0
##   Fungi                2    0  136    0    0    0
##   Protists             1    3   48    0    0    0
##   Protophotophytes     0    0  127    0    0    0
```

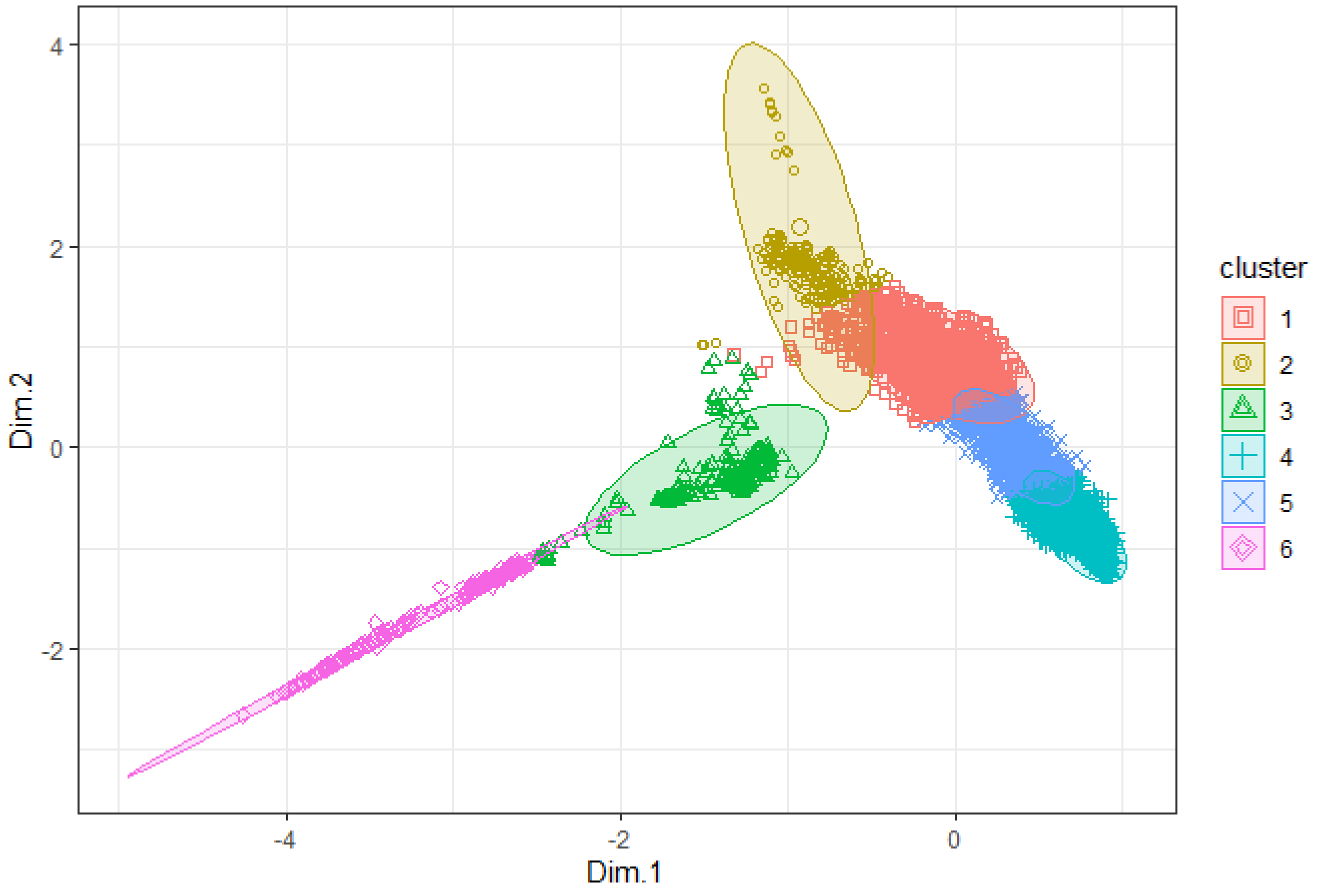

Organisms classified within cluster 1

```
##    [1] "ecu"  "ehe"  "bmic" "ben"  "bed"  "hde"  "sect" "sehc" "senm" "icp"
##   [11] "cmik" "ssz"  "fsm"  "seny" "ehd"  "baph" "hhs"  "pck"  "hin"  "hit"
##   [21] "hip"  "hiq"  "hif"  "hil"  "hiu"  "hie"  "hiz"  "hik"  "hia"  "hih"
##   [31] "hiw"  "hic"  "hix"  "hpr"  "hdu"  "hay"  "hpit" "hhz"  "haeg" "hpaa"
##   [41] "hap"  "hpaz" "hpas" "hpak" "hso"  "hsm"  "pmu"  "pmv"  "pul"  "pmp"
##   [51] "pmul" "pdag" "psky" "msu"  "bsun" "mht"  "mhq"  "mhat" "mhx"  "mhae"
##   [61] "mham" "mhao" "mhal" "mhaq" "mhay" "mvr"  "mvi"  "mvg"  "mve"  "mann"
##   [71] "mgra" "asu"  "adp"  "aap"  "aaz"  "aat"  "aan"  "aacn" "aact" "aseg"
##   [81] "apag" "avt"  "rpne" "rhey" "bhud" "xft"  "xfm"  "xfn"  "xff"  "xfl"
```

```
##   [91] "xfs"  "xtw"  "cey"  "rvi"  "fth"  "fts"  "fhi"  "fper" "fgu"  "afri"
##  [101] "aii"  "tcx"  "hmar" "tcy"  "tao"  "thig" "tzo"  "hha"  "hhk"  "ssal"
##  [111] "spiu" "sros" "spiz" "ple"  "ply"  "plr"  "plo"  "pld"  "plb"  "paly"
##  [121] "dno"  "chj"  "gap"  "fpp"  "tho"  "eof"  "rev"  "rma"  "reo"  "vok"
##  [131] "bci"  "bcib" "nmq"  "nmc"  "ngo"  "ngk"  "nek"  "ncz"  "kki"  "koa"
##  [141] "eex"  "smur" "teq"  "tea"  "teg"  "tas"  "tat"  "bpsi" "cbx"  "zin"
##  [151] "sutt" "sutk" "bbay" "tpn"  "tpq"  "tpj"  "kci"  "kct"  "kde"  "kso"
##  [161] "ndl"  "vfg"  "hpy"  "heo"  "hpj"  "hpa"  "hps"  "hhp"  "hhq"  "hhr"
##  [171] "hpg"  "hpp"  "hpb"  "hpl"  "hpc"  "hca"  "hpm"  "hpe"  "hpo"  "hpi"
##  [181] "hpq"  "hpw"  "hpu"  "hef"  "hpf"  "heq"  "hex"  "hpt"  "hpz"  "hpv"
##  [191] "hpx"  "hen"  "hph"  "heg"  "hpn"  "hep"  "heu"  "hes"  "hpys" "hcn"
##  [201] "hpd"  "hey"  "her"  "hei"  "hpya" "hpyk" "hpyo" "hpyl" "hpyb" "hpyc"
##  [211] "hpyd" "hpye" "hpyf" "hpyg" "hpyh" "hpyj" "hpyr" "hpyi" "hpyu" "hpym"
##  [221] "hem"  "heb"  "hez"  "hhe"  "hac"  "hms"  "hfe"  "hbi"  "hce"  "hcm"
##  [231] "hcp"  "hcb"  "hhm"  "hty"  "hbl"  "had"  "het"  "hcl"  "hwi"  "wsu"
##  [241] "tdn"  "sua"  "suln" "sulg" "sulc" "spal" "sulr" "cje"  "cjb"  "cjj"
##  [251] "cju"  "cjn"  "cji"  "cjm"  "cjs"  "cjp"  "cjej" "cjeu" "cjen" "cjei"
##  [261] "cjer" "cjv"  "cjy"  "cjq"  "cjl"  "cjw"  "cjr"  "cjd"  "cjz"  "cjx"
##  [271] "cff"  "cft"  "cfv"  "cfx"  "cfz"  "camp" "cfp"  "ccv"  "cha"  "cco"
##  [281] "ccoc" "cla"  "clr"  "clm"  "clq"  "cln"  "cll"  "ccol" "ccc"  "ccq"
##  [291] "ccf"  "ccy"  "ccoi" "ccof" "ccoo" "caj"  "cis"  "cvo"  "cpel" "camr"
##  [301] "csm"  "csf"  "cgra" "cure" "chyo" "chv"  "cspf" "cpin" "ccun" "clx"
##  [311] "cavi" "chw"  "camz" "camy" "coj"  "cux"  "cgeo" "cbla" "ccor" "carm"
##  [321] "cmuc" "csho" "abt"  "ask"  "atp"  "acre" "alan" "apoc" "ahs"  "hbv"
##  [331] "hyo"  "nsa"  "sun"  "slh"  "nis"  "nam"  "nap"  "cmed" "cpaf" "gsu"
##  [341] "gsk"  "gao"  "gsb"  "pace" "dvl"  "dvg"  "dds"  "dma"  "dgg"  "dfi"
##  [351] "def"  "dtr"  "dfl"  "dcb"  "lip"  "lir"  "doa"  "drt"  "dak"  "deo"
##  [361] "sat"  "dao"  "hmr"  "rpr"  "rpo"  "rpw"  "rpz"  "rpg"  "rps"  "rpv"
##  [371] "rpq"  "rpl"  "rpn"  "rty"  "rtt"  "rtb"  "rcc"  "rbe"  "rbo"  "rco"
##  [381] "rfe"  "rak"  "rri"  "rrj"  "rra"  "rrc"  "rrh"  "rrb"  "rrn"  "rrp"
##  [391] "rrm"  "rrr"  "rms"  "rpk"  "raf"  "rhe"  "rja"  "rsv"  "rsw"  "rph"
##  [401] "rau"  "rmo"  "rpp"  "ram"  "rab"  "rmc"  "ras"  "ric"  "ptc"  "mmn"
##  [411] "fso"  "rbt"  "ren"  "paca" "caq"  "naf"  "eaa"  "las"  "laa"  "lat"
##  [421] "lso"  "lcc"  "lau"  "bhe"  "bhn"  "bhs"  "bqu"  "bqr"  "bbk"  "btr"
```

```
## [431] "btx"  "bgr"  "bcd"  "baus" "bvn"  "banc" "bart" "bara" "barw" "barr"
## [441] "baro" "barj" "bez"  "barn" "bky"  "bals" "hci"  "hct"  "hcc"  "hcd"
## [451] "thd"  "pmut" "sdo"  "zmo"  "zmn"  "zmb"  "zmi"  "zmc"  "zmr"  "zmp"
## [461] "hgn"  "swf"  "bob"  "bomb" "thal" "efk"  "pub"  "peg"  "apc"  "atx"
## [471] "ocb"  "gel"  "axl"  "tap"  "aqt"  "bse"  "sau"  "sav"  "saw"  "sah"
## [481] "saj"  "sam"  "sas"  "sar"  "sac"  "sax"  "saa"  "sao"  "sae"  "sad"
## [491] "suu"  "suv"  "sue"  "suj"  "suk"  "suc"  "sut"  "suq"  "suz"  "sud"
## [501] "sux"  "suw"  "sug"  "suf"  "saua" "saue" "saun" "saus" "sauu" "saug"
## [511] "sauz" "saut" "sauj" "sauk" "sauq" "sauv" "sauw" "saux" "sauy" "sauf"
## [521] "sab"  "suy"  "saub" "saum" "sauc" "saur" "saui" "saud" "sams" "suh"
## [531] "ser"  "sep"  "sepp" "seps" "sha"  "shh"  "slg"  "sln"  "ssd"  "sdt"
## [541] "sdp"  "swa"  "spas" "shu"  "scap" "ssch" "sscz" "sagq" "ssif" "spet"
## [551] "slz"  "scoh" "sfq"  "shom" "smus" "scar" "schr" "ssh"  "ssim" "sff"
## [561] "mcl"  "mcak" "macr" "sbac" "jea"  "lmo"  "lmn"  "lmy"  "lmt"  "lmoc"
## [571] "lmoe" "lmob" "lmod" "lmow" "lmoq" "lmr"  "lmom" "lmf"  "lmc"  "lmog"
## [581] "lmp"  "lmol" "lmoj" "lmoz" "lmox" "lmh"  "lmq"  "lml"  "lmg"  "lms"
## [591] "lmj"  "lmw"  "lmx"  "lmz"  "lmon" "lmos" "lmoo" "lmoy" "lmot" "lmoa"
## [601] "lmok" "lmv"  "lin"  "lwe"  "lsg"  "liv"  "lii"  "liw"  "lia"  "lio"
## [611] "lwi"  "lgz"  "bths" "got"  "gmo"  "geq"  "gsa"  "gha"  "pyg"  "tco"
## [621] "kzo"  "paek" "pgq"  "lla"  "llk"  "llt"  "lls"  "lld"  "llx"  "llj"
## [631] "llm"  "llc"  "llr"  "lln"  "lli"  "llw"  "lgr"  "lgv"  "lpk"  "lrn"
## [641] "lact" "lack" "spy"  "spz"  "spym" "spya" "spm"  "spg"  "sps"  "sph"
## [651] "spi"  "spj"  "spk"  "spf"  "spa"  "spb"  "stg"  "stx"  "soz"  "stz"
## [661] "spyh" "spyo" "spn"  "spd"  "spr"  "spw"  "sjj"  "snv"  "spx"  "snt"
## [671] "snd"  "spnn" "sne"  "spv"  "snc"  "snm"  "spp"  "sni"  "spng" "snb"
## [681] "snp"  "snx"  "snu"  "spne" "spnu" "spnm" "spno" "sag"  "san"  "sak"
## [691] "sgc"  "sags" "sagl" "sagm" "sagi" "sagr" "sagp" "sagc" "sagt" "sage"
## [701] "sagg" "sagn" "smu"  "smc"  "smut" "smj"  "smua" "stc"  "stl"  "ste"
## [711] "stn"  "stu"  "stw"  "sthe" "sths" "ssa"  "ssb"  "ssu"  "ssv"  "ssi"
## [721] "sss"  "ssf"  "ssw"  "sup"  "ssus" "sst"  "ssuy" "ssk"  "ssq"  "sui"
## [731] "suo"  "srp"  "ssut" "ssui" "sgo"  "sez"  "seq"  "sezo" "sequ" "seu"
## [741] "sub"  "sds"  "sdg"  "sda"  "sdc"  "sdq"  "sga"  "sgg"  "sgt"  "smb"
## [751] "sor"  "stk"  "stb"  "scp"  "scf"  "ssr"  "stf"  "stj"  "strs" "ssah"
## [761] "std"  "smn"  "sif"  "sie"  "sib"  "siu"  "sang" "sanc" "sans" "scg"
```

```
##  [771] "scon" "scos" "soi"  "sik"  "siq"  "sio"  "siz"  "slu"  "sig"  "sip" 
##  [781] "stv"  "spat" "stra" "strn" "ssob" "srq"  "seqi" "ski"  "spei" "srat"
##  [791] "sgw"  "splr" "strg" "ljo"  "ljf"  "ljh"  "ljn"  "lac"  "lad"  "laf" 
##  [801] "ldb"  "lbu"  "lde"  "ldl"  "lga"  "lhe"  "lhl"  "lhr"  "lhv"  "lhh" 
##  [811] "lhd"  "lcr"  "lam"  "lai"  "lay"  "lke"  "law"  "lae"  "lgl"  "lje" 
##  [821] "lamy" "lpw"  "lkl"  "lapi" "lhs"  "lca"  "lcz"  "lcs"  "lce"  "lcw" 
##  [831] "lcl"  "lpq"  "lpi"  "lpap" "lcb"  "lcx"  "lrh"  "lrg"  "lrl"  "lra" 
##  [841] "lro"  "lrc"  "lpl"  "lpj"  "lpt"  "lps"  "lpr"  "lpz"  "lpb"  "lpx" 
##  [851] "lpg"  "lre"  "lrf"  "lru"  "lrt"  "lrr"  "lfe"  "lfr"  "lff"  "lmu" 
##  [861] "lor"  "lva"  "lfn"  "lpon" "lng"  "lhw"  "lmal" "lsn"  "lle"  "lfv" 
##  [871] "lbh"  "lbn"  "lpar" "lcu"  "lkf"  "lhil" "lbr"  "lbk"  "lko"  "lzy" 
##  [881] "lsua" "lji"  "lsl"  "lsi"  "lsj"  "lrm"  "lagl" "laca" "lani" "lbt" 
##  [891] "lcy"  "lho"  "lol"  "lnn"  "lku"  "lpd"  "lmae" "ppe"  "ppen" "pce" 
##  [901] "pdm"  "paci" "pio"  "lros" "lgn"  "lhi"  "lct"  "lalw" "lali" "lfm" 
##  [911] "lzh"  "lft"  "lsa"  "lcv"  "lgm"  "lah"  "lbm"  "lhb"  "ldx"  "ooe" 
##  [921] "oen"  "osi"  "lme"  "lmm"  "lmk"  "lci"  "lki"  "lec"  "lcn"  "lgs" 
##  [931] "lge"  "llf"  "lgc"  "lsu"  "lpse" "wko"  "wce"  "wct"  "wci"  "wcb" 
##  [941] "wjo"  "wpa"  "wcf"  "wso"  "whe"  "wei"  "wdi"  "wvr"  "efa"  "efl" 
##  [951] "efi"  "efd"  "efs"  "efn"  "efq"  "ene"  "efc"  "efau" "efu"  "efm" 
##  [961] "eft"  "ehr"  "ecas" "emu"  "edu"  "eth"  "egv"  "esg"  "mps"  "mpx" 
##  [971] "thl"  "tey"  "too"  "tkr"  "vte"  "vpi"  "vac"  "vao"  "vah"  "vcp" 
##  [981] "aur"  "aun"  "aui"  "asan" "acg"  "avs"  "auh"  "adc"  "abae" "caw" 
##  [991] "carc" "cdj"  "carn" "marr" "jep"  "jda"  "jeh"  "jar"  "jpo"  "dpm" 
## [1001] "cac"  "cae"  "cay"  "cpe"  "cpf"  "cpr"  "ctc"  "ctet" "cno"  "cbo" 
## [1011] "cba"  "cbh"  "cby"  "cbl"  "cbk"  "cbb"  "cbi"  "cbn"  "cbt"  "cbf" 
## [1021] "cbm"  "cbj"  "ckl"  "ckr"  "clj"  "ccb"  "clb"  "cah"  "clt"  "cbv" 
## [1031] "csq"  "cld"  "cace" "cck"  "cbut" "ctyk" "ceu"  "ctae" "cfm"  "cchv"
## [1041] "carg" "cdrk" "cia"  "csep" "ccoh" "cfer" "amt"  "aoe"  "asf"  "asm" 
## [1051] "aso"  "asb"  "hhw"  "cale" "crs"  "cazo" "sarj" "clo"  "fsa"  "cth" 
## [1061] "ctx"  "ccl"  "hsc"  "rbp"  "cce"  "css"  "csd"  "cthd" "esr"  "esu" 
## [1071] "ccel" "fpla" "eha"  "ral"  "rch"  "rum"  "rus"  "ruj"  "fpr"  "fpa" 
## [1081] "fpra" "capr" "ova"  "obj"  "bpb"  "bfi"  "bhu"  "cew"  "rho"  "rix" 
## [1091] "rim"  "coo"  "cct"  "rob"  "bhan" "blau" "blab" "cpy"  "lacy" "csci"
## [1101] "csh"  "cso"  "bprl" "arf"  "acac" "hsd"  "cpro" "lua"  "ehl"  "pxv" 
```

```
## [1111] "anr"  "acel" "eel"  "rto"  "rgn"  "ere"  "ert"  "era"  "lbw"  "cdf"
## [1121] "pdc"  "cdc"  "cdl"  "pdf"  "eac"  "cst"  "faa"  "psor" "roc"  "phx"
## [1131] "swo"  "slp"  "salq" "ddl"  "dmt"  "drm"  "dca"  "dru"  "dfg"  "dae"
## [1141] "dku"  "pth"  "dau"  "tjr"  "sgy"  "ded"  "dec"  "drs"  "tfr"  "hmo"
## [1151] "hcv"  "elm"  "emt"  "elim" "awo"  "cthm" "cmiu" "ibu"  "mdv"  "amij"
## [1161] "amic" "abut" "euu"  "bprm" "bprs" "cbar" "tte"  "tex"  "thx"  "tpd"
## [1171] "tit"  "tmt"  "tbo"  "twi"  "tki"  "chy"  "mta"  "mtho" "mthz" "adg"
## [1181] "tpz"  "csc"  "ate"  "cob"  "chd"  "cow"  "cki"  "ckn"  "clc"  "ccha"
## [1191] "ttm"  "tto"  "txy"  "tsh"  "tnr"  "taci" "mas"  "tep"  "tae"  "toc"
## [1201] "nth"  "hor"  "hpk"  "hals" "aar"  "aft"  "fma"  "apr"  "pmic" "ped"
## [1211] "phar" "piv"  "cad"  "spoa" "kpar" "vpr"  "vat"  "vrm"  "vdn"  "vnk"
## [1221] "med"  "mhw"  "meg"  "dpn"  "dho"  "ssg"  "sele" "selo" "selt" "mhg"
## [1231] "mfun" "afn"  "ain"  "pfac" "erh"  "ers"  "erl"  "eri"  "erd"  "eio"
## [1241] "euc"  "fro"  "aarg" "absi" "ciu"  "erm"  "fit"  "ebm"  "erb"  "tur"
## [1251] "tsg"  "acl"  "abra" "apal" "aoc"  "aaxa" "ahk"  "mbj"  "tbm"  "tbz"
## [1261] "cax"  "cuv"  "cku"  "cut"  "cfk"  "cbq"  "twh"  "tws"  "psai" "rmu"
## [1271] "rdn"  "raj"  "cig"  "djj"  "pac"  "pak"  "pav"  "pax"  "paz"  "paw"
## [1281] "pad"  "pcn"  "pacc" "pach" "pacn" "cacn" "pra"  "cgrn" "prl"  "pacd"
## [1291] "ppc"  "nfe"  "ahe"  "arca" "mcu"  "tpy"  "tpyo" "asg"  "actt" "amy"
## [1301] "soo"  "acq"  "aos"  "actp" "actc" "acto" "ane"  "actz" "air"  "asla"
## [1311] "avc"  "avu"  "wik"  "fvg"  "blo"  "blj"  "bln"  "blon" "blf"  "bll"
## [1321] "blb"  "blm"  "blk"  "blg"  "blz"  "blx"  "bad"  "badl" "bado" "bla"
## [1331] "blc"  "blt"  "bbb"  "bbc"  "bnm"  "blv"  "blw"  "bls"  "bani" "banl"
## [1341] "bni"  "banm" "bde"  "bdn"  "bbp"  "bbi"  "bbf"  "bbv"  "bbru" "bbre"
## [1351] "bbrv" "bbrj" "bbrc" "bbrn" "bbrs" "bbrd" "bast" "btp"  "bcor" "bka"
## [1361] "bks"  "bcat" "bpsp" "bii"  "bang" "bpsc" "bsca" "bact" "bcho" "bgx"
## [1371] "blem" "beu"  "gvg"  "gva"  "gvh"  "sij"  "pdo"  "plim" "pvs"  "abam"
## [1381] "nhi"  "nab"  "ccu"  "shi"  "ele"  "eyy"  "gpa"  "aeq"  "ddt"  "cbac"
## [1391] "apv"  "ols"  "olo"  "pcat" "cgo"  "caer" "sye"  "cya"  "cyb"  "syh"
## [1401] "pma"  "pmm"  "pmn"  "pmb"  "pmc"  "pmh"  "pmj"  "pme"  "prc"  "prm"
## [1411] "cyu"  "naz"  "nsp"  "mbf"  "det"  "deh"  "deb"  "dev"  "deg"  "dmc"
## [1421] "dmd"  "dmg"  "dmx"  "dmy"  "dmz"  "duc"  "dly"  "dew"  "dfo"  "atm"
## [1431] "abat" "psub" "abao" "taq"  "tpar" "opr"  "pcu"  "pnl"  "ney"  "wch"
## [1441] "sng"  "agl"  "xii"  "psup" "kst"  "broc" "pbu"  "pbp"  "pbas" "alus"
```

```
## [1451] "vbl"  "vai"  "tde"  "tsu"  "tbe"  "taz"  "tped" "scd"  "tpk"  "trm"
## [1461] "tphg" "trz"  "trc"  "ssm"  "sta"  "stq"  "sfc"  "sper" "sbu"  "scc"
## [1471] "sgp"  "ock"  "bhy"  "bhd"  "brm"  "bpo"  "bpj"  "bpip" "bpw"  "bip"
## [1481] "bhp"  "emi"  "epo"  "eti"  "rsd"  "fnu"  "fnc"  "fnt"  "fus"  "fne"
## [1491] "fhw"  "fpd"  "fgo"  "fnf"  "fpei" "lba"  "leo"  "lot"  "leq"  "lhf"
## [1501] "lsz"  "lhg"  "lte"  "lwd"  "lgo"  "str"  "smf"  "sns"  "tai"  "aco"
## [1511] "tli"  "amo"  "sbr"  "fsc"  "bth"  "btho" "bfr"  "bfs"  "bfg"  "bfb"
## [1521] "bhl"  "bxy"  "boa"  "bcel" "bcac" "bcae" "bzg"  "bhf"  "bis"  "bun"
## [1531] "bsa"  "pgi"  "pgn"  "pgt"  "pah"  "pcre" "pcag" "pmuc" "pdi"  "parc"
## [1541] "tfo"  "toh"  "pary" "dun"  "bvs"  "copr" "buy"  "aps"  "pru"  "pmz"
## [1551] "pdn"  "pit"  "pdt"  "pro"  "pfus" "peo"  "pje"  "poc"  "alq"  "afd"
## [1561] "ash"  "ald"  "aok"  "acou" "ada"  "rbc"  "bacc" "che"  "cec"  "coc"
## [1571] "ccm"  "col"  "chg"  "capn" "cgh"  "clk"  "cspu" "capf" "rar"  "orh"
## [1581] "ori"  "bcad" "apib" "smg"  "smh"  "sum"  "smv"  "smub" "smum" "smup"
## [1591] "bbl"  "bpi"  "bmm"  "bcp"  "bbg"  "bbq"  "blp"  "blu"  "blck" "ise"
## [1601] "cte"  "cpc"  "clz"  "cch"  "cph"  "cli"  "pvi"  "plt"  "pph"  "paa"
## [1611] "proc" "prs"  "pros" "cts"  "caci" "aae"  "hho"  "hys"  "hth"  "hte"
## [1621] "trd"  "sul"  "saf"  "pmx"  "tam"  "dte"  "tma"  "tmm"  "tmi"  "tmw"
## [1631] "tmq"  "tmx"  "tpt"  "trq"  "tna"  "tnp"  "thq"  "thz"  "thr"  "tle"
## [1641] "tta"  "phy"  "tme"  "taf"  "thp"  "ther" "fno"  "fpe"  "fia"  "ocy"
## [1651] "pmo"  "mpz"  "marn" "dtn"  "kol"  "kpf"  "mpg"  "minf" "asac" "cpo"
## [1661] "cex"  "ddf"  "dap"  "cni"  "gtl"  "dth"  "dtu"  "tye"  "tid"  "top"
## [1671] "tcm"  "thet" "cthi" "tav"  "tmai" "sbag" "sox"  "prf"  "bana" "bih"
## [1681] "srg"  "tmg"  "wwe"  "mib"  "wba"  "pwo"  "mja"  "mfe"  "mvu"  "mfs"
## [1691] "mif"  "mjh"  "mig"  "mmp"  "mmq"  "mmx"  "mmz"  "mmd"  "mmak" "mmao"
## [1701] "mmad" "mae"  "mvn"  "mvo"  "mok"  "metf" "mth"  "mmg"  "metc" "mwo"
## [1711] "mete" "metz" "metk" "mthm" "mst"  "metb" "mru"  "msi"  "meb"  "mmil"
## [1721] "meye" "mol"  "mel"  "mew"  "meth" "mfc"  "mfi"  "mcub" "msub" "metn"
## [1731] "mett" "meto" "mfv"  "mka"  "afu"  "afg"  "apo"  "ave"  "ast"  "fpl"
## [1741] "gac"  "gah"  "pfu"  "pfi"  "pho"  "pab"  "pyn"  "pya"  "pys"  "pyc"
## [1751] "tko"  "ton"  "tga"  "tsi"  "tba"  "the"  "tha"  "thm"  "tlt"  "ths"
## [1761] "tnu"  "teu"  "tgy"  "thv"  "tch"  "tpep" "tpie" "tgg"  "tce"  "tbs"
## [1771] "thh"  "tsl"  "ttd"  "tprf" "trl"  "tpaf" "thy"  "ppac" "mba"  "mby"
## [1781] "mbw"  "mbar" "mbak" "mac"  "mma"  "mmaz" "mmj"  "mmac" "mvc"  "mek"
```

```
## [1791] "mls"  "metm" "mef"  "meq"  "msj"  "msz"  "msw"  "mthr" "mthe" "mhor"
## [1801] "mfz"  "mbu"  "mmet" "mmh"  "mhaz" "mev"  "mzh"  "mpy"  "mzi"  "mhz"
## [1811] "mtp"  "mcj"  "mhi"  "mhu"  "mla"  "mem"  "mbg"  "mema" "mpi"  "mbn"
## [1821] "mfo"  "mpl"  "mpd"  "mez"  "rci"  "hal"  "hsl"  "hdl"  "hhb"  "halh"
## [1831] "hhsr" "hsu"  "hsf"  "salr" "halr" "hab"  "hta"  "nph"  "nmo"  "hut"
## [1841] "hti"  "hala" "hmu"  "halz" "hall" "hsn"  "hrr"  "hlt"  "harc" "hwa"
## [1851] "hwc"  "hvo"  "hme"  "hgi"  "hale" "hbo"  "haer" "hlm"  "halm" "hla"
## [1861] "halp" "halb" "hezz" "hae"  "haln" "halu" "hdf"  "hah"  "hda"  "haly"
## [1871] "nmg"  "hxa"  "nat"  "nvr"  "npl"  "nge"  "hru"  "nou"  "sali" "hlc"
## [1881] "naj"  "nag"  "nan"  "nbg"  "nas"  "tac"  "tvo"  "fac"  "fai"  "tar"
## [1891] "max"  "mer"  "mear" "marc" "abi"  "acf"  "ape"  "acj"  "smr"  "shc"
## [1901] "iho"  "iis"  "dka"  "dfd"  "dmu"  "tag"  "iag"  "thg"  "hbu"  "pfm"
## [1911] "pdl"  "sto"  "mse"  "mcn"  "mhk"  "mpru" "mten" "aho"  "abri" "asul"
## [1921] "aamb" "sazo" "step" "pai"  "pis"  "pcl"  "pas"  "pyr"  "pog"  "tne"
## [1931] "pyw"  "cma"  "ttn"  "tuz"  "vdi"  "vmo"  "tpe"  "thb"  "tcb"  "thf"
## [1941] "thel" "asc"  "acia" "clg"  "ffo"  "nmr"  "nir"  "nkr"  "nid"  "nin"
## [1951] "niw"  "ncl"  "nox"  "nue"  "nct"  "nic"  "csy"  "nga"  "nvn"  "nev"
## [1961] "taa"  "nfn"  "ncv"  "csu"  "nbv"  "tah"  "ndv"  "ccai" "kcr"  "barc"
## [1971] "barb" "flt"  "miy"  "loki" "psyt" "agw"  "arg"
```

Organisms classified within cluster 2

```
##   [1] "tan"  "tpv"  "tot"  "bfl"  "bpn"  "bva"  "bchr" "rip"  "rig"  "men"
##  [11] "meo"  "sbw"  "den"  "hed"  "ged"  "ppet" "buc"  "bap"  "bau"  "baw"
##  [21] "bajc" "bua"  "bup"  "bak"  "buh"  "bapf" "bapg" "bapu" "bapw" "bas"
##  [31] "bab"  "bcc"  "baj"  "wbr"  "wgl"  "asy"  "aen"  "cea"  "cend" "les"
##  [41] "plc"  "pli"  "crp"  "cru"  "crc"  "crt"  "crh"  "crv"  "cri"  "eme"
##  [51] "bcig" "kbl"  "kbt"  "kga"  "kon"  "ssdc" "rcm"  "rmi"  "rre"  "ots"
##  [61] "ott"  "wol"  "wri"  "wen"  "wed"  "wpi"  "wbm"  "woo"  "wcl"  "weo"
##  [71] "wpp"  "ama"  "amf"  "amw"  "amp"  "acn"  "aph"  "apy"  "apd"  "apha"
##  [81] "aoh"  "eru"  "erw"  "erg"  "ecn"  "ech"  "echa" "echj" "echl" "echs"
##  [91] "echv" "echw" "echp" "emr"  "ehh"  "nse"  "nri"  "nhm"  "nef"  "lar"
## [101] "pbq"  "mge"  "mgu"  "mgc"  "mgq"  "mgx"  "mpn"  "mpm"  "mpj"  "mpb"
```

```
## [111] "mpe"  "mga"  "mgh"  "mgf"  "mgn"  "mgs"  "mgt"  "mgv"  "mgw"  "mgac"
## [121] "mgan" "mgnc" "mgz"  "mmy"  "mmym" "mmyi" "mml"  "mcp"  "mcac" "mcap"
## [131] "mcar" "mcai" "mlc"  "mlh"  "mmo"  "mhy"  "mhj"  "mhp"  "mhn"  "mhyl"
## [141] "mhyo" "mat"  "mco"  "mho"  "mhom" "mcd"  "mhr"  "mhh"  "mhm"  "mhs"
## [151] "mhv"  "mha"  "mhf"  "mss"  "msk"  "mpf"  "mput" "mhe"  "mwe"  "mhl"
## [161] "mhb"  "mpv"  "mov"  "mbc"  "mgj"  "mfq"  "mcan" "myt"  "mds"  "myg"
## [171] "mpho" "mhyv" "mclo" "mamp" "mans" "mphc" "miw"  "mane" "mnh"  "mnu"
## [181] "mstr" "mcr"  "mcm"  "mgb"  "mgly" "mcou" "mcom" "mpu"  "msy"  "mso"
## [191] "maa"  "mal"  "mfr"  "mfm"  "mfp"  "mbv"  "mbh"  "mbi"  "mbq"  "mcy"
## [201] "mcas" "mck"  "marg" "mpul" "mbov" "mboh" "mani" "mphi" "uur"  "upa"
## [211] "upr"  "uue"  "hcr"  "poy"  "ayw"  "mbp"  "pml"  "pal"  "nzs"  "psol"
## [221] "pzi"  "mfl"  "mfw"  "mchc" "mlac" "ment" "msyr" "mtab" "mcol" "elj"
## [231] "esx"  "efr"  "eml"  "scr"  "ssyr" "sdi"  "stai" "sapi" "smir" "smia"
## [241] "scq"  "ssab" "satr" "seri" "stur" "sll"  "skn"  "scj"  "shj"  "sck"
## [251] "sfz"  "scou" "scla" "sprn" "spit" "stab" "sphh" "smoo" "salx" "sgq"
## [261] "schi" "ctr"  "ctd"  "ctf"  "ctrd" "ctro" "ctrt" "cta"  "cty"  "cra"
## [271] "ctrq" "ctrx" "ctrz" "ctrp" "ctlj" "ctlx" "ctll" "ctb"  "ctrr" "ctlf"
## [281] "ctli" "ctl"  "ctru" "ctrl" "ctrv" "ctrm" "ctla" "ctlm" "ctls" "ctlz"
## [291] "ctlc" "ctln" "ctlb" "ctlq" "cto"  "ctrn" "ctj"  "ctz"  "ctg"  "ctk"
## [301] "csw"  "ces"  "ctrb" "ctre" "ctrs" "ctec" "cfs"  "cfw"  "ctfw" "ctrf"
## [311] "ctch" "ctn"  "ctq"  "ctv"  "ctw"  "ctrg" "ctri" "ctra" "ctrh" "ctrj"
## [321] "ctrk" "ctjt" "ctcf" "ctfs" "cthf" "ctcj" "cthj" "ctmj" "cttj" "ctjs"
## [331] "ctrc" "ctrw" "ctry" "ctct" "cmu"  "cmur" "cmn"  "cmm"  "cmg"  "cmx"
## [341] "cmz"  "cpn"  "cpa"  "cpj"  "cpt"  "clp"  "cpm"  "cpec" "cpeo" "cper"
## [351] "chp"  "chb"  "chs"  "chi"  "cht"  "chc"  "chr"  "cpsc" "cpsn" "cpsb"
## [361] "cpsg" "cpsm" "cpsi" "cpsv" "cpsw" "cpst" "cpsd" "cpsa" "cav"  "cca"
## [371] "cab"  "cabo" "cfe"  "cgz"  "chla" "bbu"  "bbz"  "bbn"  "bbj"  "bbur"
## [381] "bga"  "bgb"  "bgn"  "bgs"  "bgc"  "baf"  "bafz" "bafh" "baft" "bafe"
## [391] "bbs"  "bvt"  "bchi" "bmay" "btu"  "bhr"  "bhi"  "bdu"  "bre"  "bcw"
## [401] "bmo"  "bmiy" "bpak" "bane" "btur" "bmat" "tpa"  "tpw"  "tpp"  "tpu"
## [411] "tph"  "tpo"  "tpas" "tpc"  "tpg"  "tpm"  "tpb"  "tpl"  "aas"  "cher"
## [421] "sms"  "smue" "elv"  "udi"  "saal" "sbe"  "srb"  "caqa" "dpb"  "bgw"
## [431] "bbgw" "cgw"  "baab" "nac"  "neq"  "naa"  "marh"
```

Organisms classified within cluster 3

```
##   [1] "dvi"  "fcd"  "tut"  "cel"  "cbr"  "bmy"  "loa"  "nai"  "tsp"  "lgi" 
##  [11] "crg"  "myi"  "smm"  "shx"  "ovi"  "egl"  "nve"  "tad"  "aqu"  "ath" 
##  [21] "aly"  "crb"  "csat" "eus"  "brp"  "bna"  "boe"  "rsz"  "thj"  "cpap"
##  [31] "cit"  "cic"  "pvy"  "minc" "tcc"  "gra"  "ghi"  "gab"  "dzi"  "egr" 
##  [41] "gmx"  "gsj"  "pvu"  "vra"  "var"  "vun"  "ccaj" "aprc" "mtr"  "cam" 
##  [51] "lja"  "adu"  "aip"  "ahf"  "lang" "fve"  "rcn"  "pper" "pmum" "pavi"
##  [61] "pdul" "mdm"  "pxb"  "zju"  "mnt"  "csv"  "cmo"  "bhj"  "mcha" "cmax"
##  [71] "cmos" "cpep" "rcu"  "jcu"  "hbr"  "mesc" "pop"  "peu"  "palz" "jre" 
##  [81] "qsu"  "qlo"  "twl"  "vvi"  "vri"  "sly"  "spen" "sot"  "cann" "nta" 
##  [91] "nsy"  "nto"  "nau"  "ini"  "itr"  "sind" "oeu"  "egt"  "sspl" "han" 
## [101] "ecad" "lsv"  "ccav" "dcr"  "csin" "bvg"  "soe"  "cqi"  "nnu"  "ming"
## [111] "psom" "ncol" "osa"  "dosa" "obr"  "bdi"  "ats"  "tdc"  "sbi"  "zma" 
## [121] "sita" "pvir" "phai" "pda"  "egu"  "mus"  "dct"  "peq"  "aof"  "atr" 
## [131] "smo"  "ppp"  "cre"  "vcn"  "mng"  "csl"  "cvr"  "apro" "olu"  "ota" 
## [141] "bpg"  "mis"  "mpp"  "cme"  "gsl"  "ccp"  "sce"  "ago"  "erc"  "kla" 
## [151] "kmx"  "lth"  "vpo"  "zro"  "cgr"  "ncs"  "ndi"  "tpf"  "tbl"  "tdl" 
## [161] "tgb"  "kaf"  "zmk"  "ppa"  "dha"  "pic"  "pgu"  "spaa" "lel"  "cal" 
## [171] "ctp"  "cot"  "cdu"  "cten" "yli"  "clu"  "clus" "caur" "slb"  "pkz" 
## [181] "bnn"  "bbrx" "ncr"  "nte"  "smp"  "pan"  "ttt"  "mtm"  "cthr" "mgr" 
## [191] "tmn"  "ssck" "fgr"  "fpu"  "fvr"  "fox"  "nhe"  "tre"  "trr"  "maw" 
## [201] "maj"  "cmt"  "plj"  "val"  "vda"  "cfj"  "sapo" "ela"  "pfy"  "ssl" 
## [211] "bfu"  "mbe"  "psco" "glz"  "ani"  "afm"  "act"  "nfi"  "aor"  "ang" 
## [221] "afv"  "pcs"  "pdp"  "tmf"  "trg"  "cim"  "cpw"  "ure"  "pbl"  "pbn" 
## [231] "abe"  "tve"  "aje"  "bgh"  "pno"  "pte"  "bze"  "bsc"  "bor"  "aalt"
## [241] "ztr"  "pfj"  "bcom" "npa"  "tml"  "spo"  "cne"  "cnb"  "cgi"  "tms" 
## [251] "tasa" "ppl"  "tvs"  "dsq"  "pco"  "shs"  "hir"  "psq"  "adl"  "fme" 
## [261] "gtr"  "lbc"  "mpr"  "mrr"  "cci"  "scm"  "abp"  "abv"  "cput" "sla" 
## [271] "wse"  "wic"  "uma"  "pfp"  "mgl"  "mrt"  "msym" "pgr"  "mlr"  "ein" 
## [281] "ero"  "nce"  "mbr"  "sre"  "ddi"  "dpp"  "dfa"  "ehi"  "edi"  "eiv" 
## [291] "acan" "pfa"  "pfd"  "pfh"  "pyo"  "pcb"  "pbe"  "pkn"  "pvx"  "pcy" 
## [301] "beq"  "bbo"  "cpv"  "cho"  "tgo"  "tet"  "ptm"  "smin" "pti"  "fcy"
```

```
## [311] "tps"  "ngd"  "aaf"  "pif"  "psoj" "spar" "ehx"  "gtt"  "tbr"  "tbg"
## [321] "tcr"  "lma"  "lif"  "ldo"  "lmi"  "lbz"  "lpan" "ngr"  "tva"  "gla"
```

Organisms classified within cluster 4

```
##   [1] "eco"  "ecj"  "ecd"  "ebw"  "ecok" "ece"  "ecs"  "ecf"  "etw"  "elx"
##  [11] "eoi"  "eoj"  "eoh"  "ecoo" "ecoh" "esl"  "eso"  "esm"  "eck"  "ecg"
##  [21] "eok"  "elr"  "elh"  "ecw"  "eun"  "ecp"  "ena"  "ecos" "ecv"  "ecoa"
##  [31] "ecx"  "ecm"  "ecy"  "ecr"  "ecq"  "eum"  "ect"  "eoc"  "ebr"  "ebl"
##  [41] "ebe"  "ebd"  "eci"  "eih"  "ecz"  "ecc"  "elo"  "eln"  "ese"  "ecl"
##  [51] "eko"  "ekf"  "eab"  "edh"  "edj"  "elu"  "elw"  "ell"  "elc"  "eld"
##  [61] "elp"  "elf"  "ecol" "ecoi" "ecoj" "efe"  "eal"  "ema"  "esz"  "sty"
##  [71] "stt"  "sex"  "sent" "stm"  "seo"  "sev"  "sey"  "sem"  "sej"  "seb"
##  [81] "sef"  "setu" "setc" "senr" "send" "seni" "seen" "spt"  "sek"  "spq"
##  [91] "sei"  "sec"  "seh"  "shb"  "senh" "seeh" "see"  "senn" "sew"  "sea"
## [101] "sens" "sed"  "seg"  "sel"  "sega" "set"  "sena" "seno" "senv" "senq"
## [111] "senl" "senj" "seec" "seeb" "seep" "senb" "sene" "senc" "ses"  "sbg"
## [121] "sbz"  "sbv"  "salz" "sfl"  "sfx"  "sfv"  "sfe"  "sfn"  "sfs"  "sft"
## [131] "ssn"  "sbo"  "sbc"  "sdy"  "sdz"  "shq"  "enc"  "enl"  "eclg" "ecle"
## [141] "ecln" "ecli" "eclx" "ecly" "eclz" "eclo" "ehm"  "exf"  "ecla" "eclc"
## [151] "eau"  "ekb"  "eno"  "eec"  "elg"  "ecan" "ern"  "ecls" "echg" "esh"
## [161] "ent"  "eas"  "enr"  "enx"  "enf"  "ebg"  "end"  "esa"  "csk"  "csz"
## [171] "csj"  "ccon" "cdm"  "csi"  "cmj"  "cui"  "cmw"  "ctu"  "kpn"  "kpu"
## [181] "kpm"  "kpp"  "kph"  "kpz"  "kpv"  "kpw"  "kpy"  "kpg"  "kpc"  "kpq"
## [191] "kpt"  "kpo"  "kpr"  "kpj"  "kpi"  "kpa"  "kps"  "kpx"  "kpb"  "kpne"
## [201] "kpnu" "kpnk" "kva"  "kpe"  "kpk"  "kvd"  "kvq"  "kox"  "koe"  "koy"
## [211] "kom"  "kmi"  "kok"  "koc"  "kqu"  "eae"  "ear"  "kqv"  "kll"  "klw"
## [221] "cro"  "cko"  "cfd"  "cbra" "cwe"  "cyo"  "cpot" "cfq"  "cama" "caf"
## [231] "cif"  "cfar" "cir"  "cie"  "cpar" "ebt"  "ror"  "ron"  "rpln" "rao"
## [241] "rtg"  "ree"  "cnt"  "cem"  "cen"  "clap" "pge"  "esc"  "kle"  "ksa"
## [251] "kor"  "krd"  "kco"  "kot"  "kpse" "kie"  "kas"  "lax"  "lei"  "leh"
## [261] "lee"  "ler"  "lea"  "laz"  "lef"  "lni"  "lew"  "buf"  "bage" "mety"
## [271] "ahn"  "yre"  "sgoe" "kin"  "pdz"  "ebf"  "ebc"  "ebu"  "psts" "izh"
```

```
## [281] "ype"  "ypk"  "yph"  "ypa"  "ypn"  "ypm"  "ypp"  "ypg"  "ypz"  "ypt"
## [291] "ypd"  "ypx"  "ypw"  "ypj"  "ypv"  "ypl"  "yps"  "ypo"  "ypi"  "ypy"
## [301] "ypb"  "ypq"  "ypu"  "ypr"  "ypc"  "ypf"  "yen"  "yep"  "yey"  "yel"
## [311] "yew"  "yet"  "yef"  "yee"  "ysi"  "yal"  "yfr"  "yin"  "ykr"  "yro"
## [321] "yru"  "yrb"  "yak"  "yma"  "yhi"  "yca"  "ymo"  "smar" "smac" "smw"
## [331] "spe"  "srr"  "srl"  "sry"  "sply" "srs"  "sra"  "smaf" "slq"  "serf"
## [341] "sers" "sfw"  "sfg"  "srz"  "sera" "serq" "serm" "squ"  "sfj"  "sof"
## [351] "ssur" "sfo"  "rah"  "raq"  "raa"  "rox"  "gqu"  "eame" "rbad" "eca"
## [361] "patr" "pato" "pct"  "pcc"  "pcv"  "pwa"  "ppar" "pec"  "pws"  "ppoa"
## [371] "pbra" "ppuj" "ddd"  "dda"  "dze"  "ddc"  "dzc"  "dso"  "ced"  "dfn"
## [381] "ddq"  "daq"  "dic"  "bgj"  "brb"  "bng"  "lbq"  "sod"  "eam"  "eay"
## [391] "eta"  "epy"  "epr"  "ebi"  "erj"  "ege"  "epe"  "erwi" "pam"  "plf"
## [401] "paj"  "paq"  "pva"  "pagg" "pao"  "kln"  "pant" "panp" "pagc" "pstw"
## [411] "palh" "pans" "pey"  "pdis" "pgz"  "pcd"  "mint" "mthi" "tci"  "plu"
## [421] "plum" "pay"  "ptt"  "pmr"  "phau" "xbo"  "xbv"  "xne"  "xnm"  "xdo"
## [431] "xho"  "psi"  "psx"  "psta" "prg"  "pala" "phei" "prq"  "prj"  "pvc"
## [441] "hav"  "hpar" "opo"  "pshi" "xcc"  "xcb"  "xca"  "xcp"  "xcv"  "xax"
## [451] "xac"  "xci"  "xct"  "xcj"  "xcu"  "xcn"  "xcw"  "xcr"  "xcm"  "xcf"
## [461] "xfu"  "xao"  "xom"  "xoo"  "xop"  "xoy"  "xor"  "xoz"  "xal"  "xsa"
## [471] "xtn"  "xfr"  "xve"  "xpe"  "xhr"  "xga"  "xph"  "xva"  "xan"  "xar"
## [481] "xhy"  "xcz"  "xth"  "sml"  "smt"  "buj"  "smz"  "sacz" "stek" "srh"
## [491] "slm"  "sten" "stem" "stes" "psu"  "psuw" "psd"  "pmex" "lab"  "laq"
## [501] "lcp"  "lgu"  "lez"  "lem"  "lmb"  "lyt"  "lue"  "lyj"  "lsol" "lum"
## [511] "lus"  "lug"  "thes" "theh" "tcn"  "tbv"  "xbc"  "fau"  "rhd"  "rgl"
## [521] "dji"  "dja"  "dtx"  "dye"  "dko"  "lrz"  "lpy"  "xba"  "rbd"  "vcx"
## [531] "vvu"  "vvy"  "vvm"  "vvl"  "vpa"  "vpb"  "vpk"  "vpf"  "vph"  "vha"
## [541] "vca"  "vag"  "vex"  "vdb"  "vhr"  "vna"  "vow"  "vro"  "vsp"  "vej"
## [551] "vfu"  "vni"  "van"  "lag"  "vau"  "vcy"  "vct"  "vtu"  "vfl"  "vmi"
## [561] "vga"  "vsh"  "vqi"  "vta"  "vaf"  "vnl"  "vcc"  "vas"  "vfm"  "awd"
## [571] "ppr"  "pds"  "gho"  "saly" "sks"  "pae"  "paev" "paei" "pau"  "pap"
## [581] "pag"  "paf"  "pnc"  "paeb" "pdk"  "psg"  "prp"  "paep" "paer" "paem"
## [591] "pael" "paes" "paeu" "paeg" "paec" "paeo" "pmy"  "pmk"  "pre"  "ppse"
## [601] "palc" "pcq"  "ppu"  "ppf"  "ppg"  "ppw"  "ppt"  "ppb"  "ppi"  "ppx"
## [611] "ppuh" "pput" "ppun" "ppud" "pfv"  "pmon" "pmot" "pmos" "ppj"  "por"
```

```
## [621] "pst"  "psb"  "psyr" "psp"  "pamg" "pci"  "pavl" "pvd"  "pfl"  "pprc"
## [631] "ppro" "pfo"  "pfs"  "pfe"  "pfc"  "pfn"  "ppz"  "pfb"  "pman" "ptv"
## [641] "pcg"  "pvr"  "pazo" "poi"  "pfw"  "pff"  "pfx"  "pen"  "psa"  "psz"
## [651] "psr"  "psc"  "psj"  "psh"  "pstu" "pstt" "pbm"  "plul" "pba"  "pbc"
## [661] "ppuu" "pdr"  "psv"  "psk"  "pkc"  "pch"  "pcz"  "pcp"  "pfz"  "plq"
## [671] "palk" "prh"  "psw"  "ppv"  "pses" "psem" "psec" "ppsy" "psos" "pkr"
## [681] "pfk"  "panr" "ppsl" "pset" "psil" "pym"  "psed" "pke"  "pall" "pum"
## [691] "poj"  "pgg"  "ppsh" "pgy"  "avn"  "avl"  "avd"  "acx"  "pagr" "pcr"
## [701] "pso"  "pali" "pspg" "psyg" "psyc" "psyp" "acb"  "aby"  "abc"  "abn"
## [711] "abb"  "abx"  "abz"  "abr"  "abd"  "abh"  "abad" "abj"  "abab" "abaj"
## [721] "abaz" "abk"  "abau" "abaa" "abw"  "abal" "acc"  "ano"  "alc"  "acal"
## [731] "acd"  "aci"  "att"  "aei"  "ajo"  "acw"  "acv"  "ahl"  "ajn"  "asol"
## [741] "ala"  "asj"  "adv"  "arj"  "awu"  "agu"  "alw"  "ads"  "aber" "atn"
## [751] "achi" "alj"  "mbah" "son"  "sdn"  "sfr"  "saz"  "sbl"  "sbm"  "sbn"
## [761] "sbp"  "sbt"  "sbs"  "sbb"  "slo"  "spc"  "shp"  "sse"  "spl"  "she"
## [771] "shm"  "shn"  "shw"  "shl"  "swd"  "swp"  "svo"  "shf"  "sja"  "spsw"
## [781] "sbj"  "smav" "shew" "salg" "slj"  "smai" "spol" "sbk"  "skh"  "saes"
## [791] "idt"  "cps"  "com"  "coz"  "colw" "cola" "cber" "cov"  "lsd"  "tht"
## [801] "thap" "pha"  "ptn"  "pat"  "psm"  "pseo" "pia"  "pphe" "pbw"  "prr"
## [811] "plz"  "paln" "ppis" "pea"  "pspo" "part" "ptu"  "png"  "ptd"  "psen"
## [821] "pdj"  "paga" "pcar" "pmaa" "maq"  "mhc"  "mad"  "mbs"  "mpq"  "mari"
## [831] "mlq"  "msq"  "mara" "marj" "amc"  "amh"  "amaa" "amal" "amae" "amao"
## [841] "amad" "amai" "amag" "amac" "amb"  "amg"  "amk"  "alt"  "aal"  "aaus"
## [851] "asp"  "asq"  "aaw"  "alr"  "ale"  "alz"  "apel" "gag"  "gni"  "gps"
## [861] "pmes" "lal"  "cate" "salh" "salm" "salk" "hmi"  "pin"  "fbl"  "fes"
## [871] "mvs"  "mya"  "mmaa" "cja"  "ceb"  "cell" "cek"  "ceg"  "sde"  "ttu"
## [881] "saga" "spoi" "zal"  "osg"  "mthd" "micc" "maga" "mii"  "mict" "mhyd"
## [891] "hja"  "halc" "kim"  "lpn"  "lpu"  "lpm"  "lpf"  "lpp"  "lpc"  "lpa"
## [901] "lpe"  "lha"  "lcd"  "llg"  "ljr"  "lss"  "tmc"  "mmai" "cza"  "nhl"
## [911] "tee"  "rhh"  "wma"  "woc"  "gai"  "hch"  "hahe" "csa"  "hel"  "hcs"
## [921] "hak"  "ham"  "hhu"  "hco"  "hsi"  "halo" "hhh"  "hbe"  "hag"  "haf"
## [931] "halk" "hvn"  "hol"  "hsr"  "hmd"  "haxi" "htt"  "hcam" "hpiz" "haa"
## [941] "cmai" "abo"  "adi"  "aln"  "axe"  "kak"  "mmw"  "mme"  "mpc"  "mpri"
## [951] "mard" "tol"  "tor"  "oai"  "mars" "bsan" "ncu"  "nik"  "bmar" "ajp"
```

```
##  [961] "gsn"  "rfo"  "ome"  "llp"  "aha"  "ahy"  "ahd"  "ahr"  "ahp"  "ahj" 
##  [971] "ahh"  "ahi"  "aaj"  "asa"  "aeo"  "avr"  "avo"  "amed" "asr"  "adh" 
##  [981] "aem"  "aea"  "arv"  "aes"  "ael"  "oce"  "zdf"  "sok"  "sini" "gbi" 
##  [991] "sva"  "saln" "pspi" "gpb"  "vff"  "cvi"  "cvc"  "chro" "chri" "chrb"
## [1001] "crz"  "chrm" "chae" "iod"  "ifl"  "pse"  "jeu"  "aql"  "amah" "aqs" 
## [1011] "dee"  "chiz" "cfon" "rso"  "rsc"  "rsl"  "rsn"  "rsm"  "rse"  "rsy" 
## [1021] "rpi"  "rpf"  "rpj"  "rmn"  "rin"  "rpu"  "reh"  "cnc"  "cuh"  "reu" 
## [1031] "rme"  "cti"  "cbw"  "cgd"  "ccup" "cup"  "cuu"  "cpau" "cox"  "bma" 
## [1041] "bmv"  "bml"  "bmn"  "bmal" "bmae" "bmaq" "bmai" "bmaf" "bmaz" "bmab"
## [1051] "bps"  "bpm"  "bpl"  "bpd"  "bpr"  "bpse" "bpsm" "bpsu" "bpsd" "bpz" 
## [1061] "bpq"  "bpk"  "bpsh" "bpsa" "bpso" "but"  "bte"  "btq"  "btj"  "btz" 
## [1071] "btd"  "btv"  "bthe" "bthm" "btha" "bthl" "bok"  "boc"  "buu"  "bvi" 
## [1081] "bve"  "bur"  "bcn"  "bch"  "bcm"  "bcj"  "bcen" "bcew" "bceo" "bam" 
## [1091] "bac"  "bmj"  "bmu"  "bmk"  "bmul" "bct"  "bced" "bcep" "bdl"  "bpyr"
## [1101] "bcon" "bub"  "bdf"  "blat" "btei" "bsem" "bpsl" "bmec" "bstg" "bstl"
## [1111] "bgl"  "bgu"  "bug"  "bgf"  "bgd"  "bgo"  "byi"  "buk"  "bue"  "bul" 
## [1121] "buq"  "bgp"  "bpla" "bud"  "bum"  "bui"  "bxe"  "bxb"  "bph"  "bge" 
## [1131] "bpx"  "bpy"  "buz"  "bfn"  "bcai" "pspw" "para" "parb" "phs"  "pter"
## [1141] "pgp"  "pcj"  "pts"  "pcaf" "pmeg" "brh"  "ppk"  "ppno" "ppnm" "prb" 
## [1151] "ppul" "pspu" "papi" "pve"  "pox"  "ptx"  "pfg"  "pnr"  "pand" "pfib"
## [1161] "plg"  "hyf"  "caba" "buo"  "limn" "cari" "bpe"  "bpc"  "bper" "bpet"
## [1171] "bpeu" "bpar" "bpa"  "bbh"  "bbr"  "bbm"  "bbx"  "bpt"  "bav"  "bho" 
## [1181] "bhm"  "bhz"  "btrm" "bbro" "bfz"  "bpdz" "boh"  "bgm"  "boj"  "boz" 
## [1191] "axy"  "axo"  "axn"  "axx"  "adt"  "ais"  "asw"  "achr" "achb" "put" 
## [1201] "pus"  "amim" "cdn"  "afa"  "afq"  "aaqu" "odi"  "pig"  "pacr" "kgy" 
## [1211] "rfr"  "rsb"  "rac"  "rhy"  "rhf"  "rhg"  "pol"  "pna"  "pos"  "poo" 
## [1221] "aav"  "ajs"  "dia"  "aaa"  "ack"  "acra" "acid" "acip" "acin" "acis"
## [1231] "acio" "amon" "vei"  "dac"  "del"  "dts"  "dhk"  "dla"  "vap"  "vpe" 
## [1241] "vpd"  "vaa"  "vbo"  "vam"  "ctt"  "ctes" "cke"  "cser" "cof"  "adn" 
## [1251] "adk"  "rta"  "otk"  "lim"  "lih"  "hyr"  "hyb"  "hyl"  "hyc"  "hpse"
## [1261] "hyn"  "dpy"  "dih"  "daer" "drg"  "simp" "melm" "mela" "sthm" "mpt" 
## [1271] "metp" "har"  "mms"  "jag"  "jab"  "jaz"  "jal"  "jsv"  "jaj"  "jas" 
## [1281] "jlv"  "hse"  "hsz"  "hht"  "hrb"  "hee"  "hhf"  "hfr"  "cfu"  "care"
## [1291] "cpra" "mnr"  "masw" "mass" "masz" "mtim" "masy" "mali" "mum"  "mfla"
```

```
## [1301] "mpli" "upv"  "nok"  "dug"  "lch"  "tin"  "thi"  "rge"  "rbn"  "rdp"
## [1311] "pkt"  "miu"  "rgu"  "aon"  "snn"  "xyk"  "pbh"  "shd"  "metr" "doe"
## [1321] "uru"  "upl"  "eba"  "dsu"  "otr"  "rbh"  "dar"  "dey"  "azo"  "aoa"
## [1331] "aza"  "azi"  "atw"  "acom" "azd"  "azr"  "azq"  "tmz"  "thu"  "tcl"
## [1341] "thk"  "tak"  "app"  "beb"  "beba" "dat"  "dwd"  "dalk" "afw"  "mxa"
## [1351] "msd"  "mym"  "mfb"  "ccx"  "mfu"  "mmas" "sur"  "age"  "mbd"  "cfus"
## [1361] "scl"  "scu"  "ccro" "samy" "llu"  "mrm"  "hoh"  "dti"  "pcay" "mlo"
## [1371] "mln"  "mci"  "mop"  "mam"  "mamo" "meso" "mesw" "mesm" "mesp" "mhua"
## [1381] "mjr"  "merd" "mes"  "hoe"  "aak"  "amih" "pht"  "rpod" "niy"  "orm"
## [1391] "pla"  "pmob" "rbs"  "sme"  "smk"  "smq"  "smx"  "smi"  "smeg" "smel"
## [1401] "smer" "smd"  "rhi"  "sfh"  "sfd"  "six"  "same" "sino" "ead"  "eah"
## [1411] "esj"  "eak"  "emx"  "atu"  "ara"  "ata"  "agr"  "atf"  "avi"  "agc"
## [1421] "aro"  "agt"  "alf"  "ret"  "rec"  "rel"  "rep"  "rei"  "rle"  "rlt"
## [1431] "rlg"  "rlb"  "rlu"  "rtr"  "rir"  "rpus" "rhl"  "rga"  "rhn"  "rpha"
## [1441] "rhx"  "rhv"  "rhk"  "rez"  "rjg"  "rhr"  "rgr"  "rad"  "roy"  "rii"
## [1451] "ngl"  "ngg"  "neo"  "nen"  "rht"  "shz"  "abaw" "kai"  "bmi"  "bmz"
## [1461] "bmg"  "bmee" "bms"  "bsi"  "bsf"  "bsv"  "bsw"  "bsg"  "bcs"  "bsk"
## [1471] "bol"  "bcar" "bcas" "bmr"  "bpv"  "bru"  "oin"  "oan"  "oah"  "ops"
## [1481] "och"  "bja"  "bju"  "bjp"  "bra"  "bbt"  "brs"  "aol"  "brc"  "brad"
## [1491] "bic"  "bro"  "brk"  "bot"  "brq"  "bgq"  "bgz"  "bsym" "bbet" "barh"
## [1501] "bvz"  "rpa"  "rpb"  "rpc"  "rpd"  "rpe"  "rpt"  "rpx"  "oca"  "ocg"
## [1511] "oco"  "bop"  "bos"  "bvv"  "boi"  "bof"  "vgo"  "trb"  "xau"  "azc"
## [1521] "sno"  "star" "lne"  "anc"  "apra" "mea"  "mdi"  "mex"  "mch"  "mpo"
## [1531] "mza"  "mrd"  "met"  "mno"  "mor"  "meta" "maqu" "mphy" "mee"  "metd"
## [1541] "metx" "mets" "meti" "mmes" "mtea" "moc"  "miv"  "mico" "bid"  "msl"
## [1551] "mtun" "bbar" "chel" "cdq"  "hdn"  "hdt"  "hmc"  "deq"  "rhz"  "yti"
## [1561] "ntd"  "msc"  "mbry" "mros" "mhey" "mpar" "mtw"  "pleo" "mey"  "maad"
## [1571] "mmed" "aua"  "brn"  "psin" "hdi"  "noh"  "rbm"  "pphr" "lap"  "lagg"
## [1581] "labr" "labp" "labt" "siw"  "ccr"  "ccs"  "cak"  "cse"  "chq"  "cmb"
## [1591] "cfh"  "cauf" "pzu"  "phb"  "bsb"  "bne"  "brl"  "bvc"  "brf"  "brev"
## [1601] "bvy"  "aex"  "tsv"  "cbot" "sil"  "sit"  "rua"  "rut"  "rmb"  "jan"
## [1611] "rpon" "pde"  "pami" "pye"  "pzh"  "paro" "parr" "pkd"  "ppan" "dsh"
## [1621] "pga"  "pgl"  "pgd"  "php"  "ppic" "phq"  "lmd"  "lej"  "cid"  "ceh"
## [1631] "cmag" "malg" "con"  "rsu"  "rhm"  "hat"  "daa"  "ypac" "tpro" "suam"
```

```
## [1641] "spse" "suli" "suld" "spot" "tom"  "paby" "thw"  "tec"  "rmm"  "rok"
## [1651] "aht"  "rbg"  "thaa" "geh"  "taw"  "salo" "hml"  "pseb" "lit"  "ocd"
## [1661] "maru" "rot"  "ppru" "mon"  "malu" "tgl"  "pshq" "poz"  "palw" "pgv"
## [1671] "faq"  "hdh"  "gak"  "hne"  "hba"  "hbc"  "nar"  "npp"  "npn"  "nre"
## [1681] "nov"  "nor"  "ngf"  "sphk" "sphp" "smag" "smaz" "sgi"  "sphq" "spho"
## [1691] "sphx" "sphu" "swi"  "sphd" "sphm" "stax" "sphi" "ssan" "snj"  "smy"
## [1701] "span" "skr"  "splm" "splk" "spkc" "sphc" "sphf" "spha" "spau" "sech"
## [1711] "sch"  "ssy"  "syb"  "sbd"  "spmi" "sphb" "sphr" "sinb" "spht" "shyd"
## [1721] "sya"  "sclo" "spyg" "sufl" "sami" "sbar" "cij"  "sphy" "blas" "bfw"
## [1731] "rdi"  "palg" "smic" "sphs" "sand" "alb"  "alh"  "anh"  "ado"  "cman"
## [1741] "ery"  "egn"  "elq"  "erk"  "erf"  "emv"  "pns"  "pot"  "gbe"  "gbh"
## [1751] "gbc"  "gbs"  "acr"  "amv"  "gdi"  "gxy"  "gxl"  "kna"  "keu"  "ksc"
## [1761] "kre"  "kha"  "apw"  "apf"  "apu"  "apg"  "apq"  "apx"  "apz"  "apk"
## [1771] "asz"  "asv"  "apom" "ato"  "acet" "aoy"  "rgi"  "ros"  "rmuc" "shum"
## [1781] "svc"  "rru"  "rce"  "mag"  "mgy"  "mgry" "magx" "magn" "azl"  "ali"
## [1791] "abs"  "abq"  "abf"  "ati"  "azt"  "azm"  "azz"  "aoz"  "tmo"  "txi"
## [1801] "thac" "tii"  "nao"  "ncb"  "fer"  "htq"  "hadh" "skt"  "phr"  "pstg"
## [1811] "bdc"  "bdz"  "pms"  "pmq"  "pmw"  "pbk"  "eff"  "mtu"  "mtv"  "mtc"
## [1821] "mra"  "mtf"  "mtb"  "mtk"  "mtz"  "mtg"  "mti"  "mte"  "mtur" "mtl"
## [1831] "mto"  "mtd"  "mtn"  "mtj"  "mtub" "mtuc" "mtue" "mtx"  "mtuh" "mtul"
## [1841] "mtut" "mtuu" "mtq"  "mbo"  "mbb"  "mbt"  "mbm"  "mbk"  "mbx"  "maf"
## [1851] "mmic" "mce"  "mcq"  "mcv"  "mcx"  "mcz"  "mpa"  "mao"  "mavi" "mavu"
## [1861] "mav"  "mit"  "mia"  "mid"  "myo"  "mchi" "mir"  "mmal" "mlp"  "msa"
## [1871] "mul"  "mmc"  "mkm"  "mjl"  "mmi"  "mmae" "mmm"  "mli"  "mkn"  "myv"
## [1881] "mye"  "mhad" "mdx"  "mshg" "mfj"  "mgro" "mxe"  "mnv"  "mpag" "mnm"
## [1891] "mgor" "mcoo" "msm"  "msg"  "msb"  "msn"  "msh"  "mva"  "mgi"  "msp"
## [1901] "mcb"  "mne"  "myn"  "mgo"  "mft"  "mphl" "mvq"  "mll"  "mrh"  "mthn"
## [1911] "mhas" "mdu"  "mcht" "mdr"  "mauu" "mmag" "mmor" "mfx"  "maic" "mij"
## [1921] "malv" "mty"  "mpsc" "mab"  "mmv"  "mabb" "mabl" "mche" "miz"  "mste"
## [1931] "msao" "msal" "mjd"  "mter" "mmin" "mhib" "asd"  "mkr"  "chn"  "cgy"
## [1941] "nfa"  "nfr"  "ncy"  "nbr"  "nno"  "nsl"  "nsr"  "ntp"  "noz"  "nod"
## [1951] "nah"  "nad"  "nwl"  "rha"  "rer"  "rey"  "reb"  "rop"  "roa"  "req"
## [1961] "rpy"  "rhb"  "rav"  "rfa"  "rhw"  "rhs"  "rrz"  "rhu"  "rqi"  "rhq"
## [1971] "rhod" "rrt"  "rby"  "rcr"  "rtm"  "gbr"  "gpo"  "gor"  "goq"  "gta"
```

```
## [1981] "goc"  "git"  "gru"  "gom"  "gav"  "god"  "tpr"  "tsm"  "srt"  "dtm"
## [1991] "diz"  "dpc"  "dlu"  "toy"  "sco"  "salb" "sma"  "sgr"  "sgb"  "scb"
## [2001] "ssx"  "svl"  "sct"  "scy"  "sbh"  "shy"  "sho"  "sve"  "sdv"  "sals"
## [2011] "sfi"  "sci"  "src"  "salu" "sall" "slv"  "sgu"  "svt"  "stre" "scw"
## [2021] "sld"  "slc"  "sxi"  "strm" "strc" "samb" "spri" "scz"  "scx"  "srw"
## [2031] "strf" "sle"  "srn"  "spav" "strt" "sclf" "sgs"  "stsi" "sls"  "snr"
## [2041] "splu" "strd" "snw"  "sauo" "ssia" "svu"  "spun" "sgv"  "smal" "slau"
## [2051] "salf" "salj" "slx"  "stro" "sfk"  "snz"  "sge"  "srj"  "slk"  "sky"
## [2061] "sdx"  "sgd"  "sqz"  "scya" "sast" "snq"  "stir" "ska"  "sgz"  "svn"
## [2071] "snk"  "salw" "shaw" "srk"  "sfic" "sgal" "sspo" "svr"  "spad" "sfy"
## [2081] "saqu" "sgf"  "scav" "sseo" "ksk"  "kab"  "kau"  "kit"  "stri" "leif"
## [2091] "lse"  "cmi"  "cms"  "cmc"  "cmh"  "ccap" "mts"  "mim"  "mio"  "mip"
## [2101] "mcw"  "mpal" "micr" "mhos" "mwa"  "mprt" "moy"  "rtn"  "rry"  "ria"
## [2111] "rfs"  "rte"  "agm"  "agf"  "cart" "cphy" "amin" "aum"  "auw"  "gry"
## [2121] "lyd"  "plap" "ltr"  "agg"  "mant" "hea"  "gln"  "chre" "agx"  "art"
## [2131] "arr"  "arm"  "arl"  "are"  "aaq"  "arh"  "ary"  "arz"  "aru"  "arq"
## [2141] "arn"  "arx"  "acry" "arth" "artp" "aau"  "pue"  "ach"  "apn"  "psul"
## [2151] "psni" "psey" "aai"  "gcr"  "glu"  "rsa"  "kfv"  "mick" "satk" "nae"
## [2161] "bcv"  "bfa"  "brx"  "bsau" "lmoi" "ica"  "jte"  "jli"  "jme"  "pei"
## [2171] "serw" "orn"  "orz"  "aus"  "mph"  "mik"  "micg" "rain" "nca"  "noo"
## [2181] "nsn"  "nano" "nmes" "psim" "aer"  "muz"  "kfl"  "kqi"  "nda"  "nal"
## [2191] "ngv"  "strr" "tcu"  "actw" "sro"  "noa"  "now"  "tbi"  "fra"  "fre"
## [2201] "fri"  "fal"  "fsy"  "gob"  "bsd"  "mmar" "kra"  "sen"  "sace" "sacg"
## [2211] "svi"  "sacc" "amd"  "amn"  "amm"  "amz"  "aoi"  "aja"  "amq"  "amyc"
## [2221] "amyb" "aab"  "amyy" "aori" "pdx"  "psee" "pseh" "pseq" "pecq" "phh"
## [2231] "paut" "apre" "ami"  "sesp" "ssyi" "kal"  "kphy" "led"  "ahm"  "ahg"
## [2241] "acta" "alo"  "pmad" "stp"  "saq"  "mau"  "mil"  "micb" "mtua" "mich"
## [2251] "mtem" "mcab" "msag" "vma"  "mcra" "ase"  "ams"  "actn" "afs"  "acts"
## [2261] "plk"  "plab" "plat" "pfla" "psuu" "ver"  "cai"  "sna"  "aey"  "eke"
## [2271] "bsol" "cwo"  "euz"  "amr"  "riv"  "cau"  "chl"  "cap"  "kbs"  "tra"
## [2281] "fgi"  "roo"  "luo"  "ahel" "lcre" "aagg" "pls"  "gmr"  "sdyn" "ges"
## [2291] "gog"  "gms"  "lrs"  "ftj"  "saci" "pbor" "laj"  "lkm"  "aba"  "acm"
## [2301] "gma"  "grw"  "talb" "abas" "eda"  "sus"  "pfer" "ctm"  "abac" "gau"
## [2311] "gph"  "gba"  "drc"  "sru"  "rmr"  "rmg"  "cpi"  "cbae" "chit" "chih"
```

```
## [2321] "nko"  "nso"  "fla"  "fgg"  "fln"  "pseg" "pgin" "pgo"  "lacs" "hhy" 
## [2331] "phe"  "pep"  "pcm"  "psty" "pek"  "proe" "shg"  "sht"  "sdj"  "scn" 
## [2341] "mup"  "muc"  "mgot" "muh"  "mgin" "mgk"  "mrub" "mgos" "agd"  "oli" 
## [2351] "sbx"  "cmr"  "camu" "bbd"  "evi"  "est"  "echi" "alm"  "dfe"  "sli" 
## [2361] "srd"  "smon" "spir" "spik" "spib" "lby"  "rsi"  "run"  "rup"  "eol" 
## [2371] "fae"  "fib"  "psez" "als"  "rhoz" "hsw"  "hym"  "hyd"  "hye"  "hyg" 
## [2381] "hyp"  "hyz"  "hnv"  "hyh"  "hyj"  "hrs"  "pko"  "pact" "ruf"  "rti" 
## [2391] "rud"  "nib"  "add"  "aswu" "mtt"  "flm"  "fll"  "fbt"  "chk"  "gfl" 
## [2401] "grs"  "fjo"  "fcm"  "fbc"  "marm" "mart" "marb" "mare" "cao"  "cbal"
## [2411] "cbat" "mrs"  "mut"  "ptq"  "ndo"  "nob"  "pom"  "pob"  "prn"  "pola"
## [2421] "poa"  "phal" "win"  "sze"  "syi"  "fop"  "salt" "seon" "aalg" "kan" 
## [2431] "marf" "aqb"  "aqa"  "emar" "mur"  "afla" "anp"  "fbe"  "nde"  "nmv" 
## [2441] "nja"
```

Organisms classified within cluster 5

```
##    [1] "kgo"  "lpop" "sgl"  "pes"  "tpty" "pmib" "pvl"  "pvg"  "prot" "pcol"
##   [11] "pcib" "xpo"  "mmk"  "ans"  "eic"  "etr"  "etd"  "ete"  "etc"  "edw" 
##   [21] "edl"  "eho"  "lpv"  "pfq"  "prag" "lri"  "gle"  "apl"  "apj"  "apa" 
##   [31] "asi"  "ass"  "aeu"  "apor" "aio"  "alig" "aao"  "aah"  "gan"  "bto" 
##   [41] "btre" "btrh" "btra" "ooi"  "fcl"  "paet" "xfa"  "xfh"  "vch"  "vcf" 
##   [51] "vcs"  "vce"  "vcq"  "vcj"  "vci"  "vco"  "vcr"  "vcm"  "vcl"  "vcz" 
##   [61] "vbr"  "vsc"  "vaq"  "vsr"  "vfi"  "vsa"  "pgb"  "pmai" "scot" "pade"
##   [71] "pbb"  "emo"  "par"  "prw"  "pur"  "psya" "psyy" "abm"  "aid"  "acum"
##   [81] "aug"  "mct"  "mcs"  "mcat" "moi"  "mos"  "mbl"  "mboi" "mcun" "mnn" 
##   [91] "ilo"  "ili"  "ipi"  "idi"  "msr"  "msx"  "psy"  "cbu"  "cbs"  "cbd" 
##  [101] "cbg"  "cbc"  "alg"  "asip" "lph"  "lpo"  "llo"  "lfa"  "lok"  "lsh" 
##  [111] "lib"  "lgt"  "lcj"  "lwa"  "mca"  "metu" "mmt"  "mdn"  "mdh"  "mko" 
##  [121] "metl" "mah"  "mbur" "mpsy" "mmob" "mein" "ftu"  "ftq"  "ftf"  "ftw" 
##  [131] "ftr"  "ftt"  "ftg"  "ftl"  "fta"  "fti"  "fto"  "ftc"  "ftv"  "ftz" 
##  [141] "ftm"  "ftn"  "ftx"  "ftd"  "fty"  "fcf"  "fcn"  "fph"  "fpt"  "fpi" 
##  [151] "fpm"  "fpx"  "fpz"  "fpj"  "frt"  "fna"  "fnl"  "frf"  "fha"  "frx" 
##  [161] "frm"  "frc"  "fad"  "fmi"  "foo"  "htr"  "thio" "mej"  "mec"  "cyq"
```

```
## [171] "cyy"  "psal" "tse"  "tig"  "blep" "this" "noc"  "nwa"  "nwr"  "alv"
## [181] "tvi"  "tmb"  "mpur" "ntt"  "tsy"  "ttp"  "ntg"  "thip" "aeh"  "hhc"
## [191] "ebs"  "tgr"  "tkm"  "tni"  "tti"  "tvr"  "aprs" "hna"  "haz"  "ghl"
## [201] "ttc"  "zpl"  "kus"  "kma"  "kuy"  "paur" "apac" "kko"  "kge"  "ksd"
## [211] "kpd"  "acav" "tau"  "ocm"  "opf"  "orb"  "sdf"  "slim" "acii" "tbn"
## [221] "seds" "tsn"  "thin" "ebh"  "enm"  "nme"  "nmp"  "nmh"  "nmd"  "nmm"
## [231] "nms"  "nmz"  "nma"  "nmw"  "nmx"  "nmn"  "nmt"  "nmi"  "nla"  "nel"
## [241] "nwe"  "nsi"  "nmj"  "nei"  "nfv"  "nsf"  "nzl"  "naq"  "nbl"  "nzo"
## [251] "nci"  "nani" "nbc"  "salv" "vit"  "ecor" "aff"  "cste" "nba"  "lhk"
## [261] "pnu"  "pne"  "pdq"  "poh"  "lmir" "mcys" "pud"  "aka"  "phn"  "our"
## [271] "oto"  "cbaa" "cbab" "ofo"  "upi"  "bbag" "neu"  "net"  "nit"  "nii"
## [281] "nco"  "nur"  "nst"  "nmu"  "nlc"  "tbd"  "mfa"  "mmb"  "meh"  "mei"
## [291] "mep"  "mbac" "mbat" "meu"  "slt"  "gca"  "fam"  "nim"  "sdr"  "sulf"
## [301] "splb" "sniv" "slac" "rbu"  "zpa"  "fpho" "fmy"  "bprc" "sku"  "crx"
## [311] "abu"  "abl"  "acib" "afc"  "ant"  "aell" "aaqi" "asui" "aclo" "aana"
## [321] "avp"  "adz"  "alk"  "alp"  "amyt" "amar" "acaa" "amol" "apai" "hebr"
## [331] "paco" "arc"  "sdl"  "sba"  "smul" "shal" "suls" "sulj" "sult" "gme"
## [341] "gur"  "glo"  "gbm"  "geo"  "gem"  "geb"  "gpi"  "gbn"  "pca"  "pef"
## [351] "ppd"  "des"  "deu"  "dvu"  "dvm"  "dde"  "dpg"  "dms"  "dsd"  "dsa"
## [361] "dhy"  "daf"  "das"  "dpi"  "dej"  "pprf" "psel" "ddn"  "dsx"  "dba"
## [371] "dps"  "dpr"  "dog"  "dsf"  "dol"  "dml"  "dal"  "dto"  "dov"  "ade"
## [381] "acp"  "ank"  "vin"  "sfu"  "dax"  "dbr"  "dav"  "bsed" "bme"  "bmel"
## [391] "bmw"  "bmf"  "bmb"  "bmc"  "baa"  "babo" "babr" "babt" "babb" "babu"
## [401] "babs" "babc" "bsui" "bsup" "bsuv" "bsuc" "bmt"  "bsz"  "bov"  "bpp"
## [411] "bcet" "bcee" "bvl"  "brj"  "nwi"  "nha"  "bapi" "mlg"  "rhj"  "hni"
## [421] "rva"  "phl"  "fil"  "fiy"  "dei"  "dea"  "bvr"  "blag" "mmyr" "mcg"
## [431] "metg" "aala" "psf"  "brd"  "brg"  "bdm"  "bmed" "rsp"  "rsh"  "rsq"
## [441] "rsk"  "rcp"  "rhp"  "rbl"  "rde"  "rli"  "pcon" "paru" "pamn" "pars"
## [451] "kvl"  "kvu"  "kro"  "oat"  "oar"  "otm"  "oct"  "laqu" "red"  "ptp"
## [461] "cmar" "rhc"  "yan"  "sulz" "don"  "rid"  "rom"  "roh"  "lvs"  "sagu"
## [471] "sedi" "boo"  "paed" "pamo" "ppaf" "rbz"  "thas" "mmr"  "hyt"  "zmm"
## [481] "not"  "nog"  "sal"  "ster" "sphl" "slut" "srhi" "sjp"  "sphg" "sfla"
## [491] "sphj" "spzr" "aay"  "aep"  "amx"  "cna"  "efv"  "eli"  "err"  "porl"
## [501] "phz"  "gox"  "goh"  "goy"  "gal"  "gti"  "gdj"  "apt"  "aace" "aper"
```

```
## [511] "aasc" "aot"  "abg"  "kba"  "nch"  "coq"  "comm" "ntn"  "neh"  "ssam"
## [521] "rrf"  "rpm"  "ahu"  "magq" "hjo"  "dex"  "dvn"  "pbr"  "mgm"  "pel"
## [531] "apm"  "ecog" "mai"  "man"  "apb"  "bba"  "bbat" "bbw"  "bbac" "bex"
## [541] "bdq"  "bmx"  "hax"  "bsto" "sbf"  "afr"  "afe"  "acu"  "acz"  "afi"
## [551] "afj"  "maes" "mfn"  "htl"  "bsu"  "bsr"  "bsl"  "bsh"  "bsy"  "bsut"
## [561] "bsul" "bsus" "bso"  "bsn"  "bsq"  "bsx"  "bsp"  "bss"  "bst"  "bli"
## [571] "bld"  "blh"  "bay"  "baq"  "bya"  "bamp" "baml" "bama" "bamn" "bamb"
## [581] "bamt" "bamy" "bmp"  "bao"  "baz"  "bql"  "bxh"  "bqy"  "bami" "bamc"
## [591] "bamf" "bsia" "bae"  "bvm"  "bson" "bht"  "ban"  "bar"  "bat"  "bah"
## [601] "bai"  "bax"  "bant" "banr" "bans" "banh" "banv" "bce"  "bca"  "bcz"
## [611] "bcr"  "bcb"  "bcu"  "bcg"  "bcq"  "bcx"  "bal"  "bnc"  "bcf"  "bcer"
## [621] "bcef" "bcy"  "btk"  "btl"  "btb"  "btt"  "bthr" "bthi" "btc"  "btf"
## [631] "btm"  "btg"  "bti"  "btn"  "btht" "bthu" "btw"  "bthy" "bwe"  "bww"
## [641] "bmyo" "bty"  "bmyc" "bby"  "bwd"  "btro" "bmob" "bpu"  "bpum" "bpus"
## [651] "bco"  "bjs"  "baci" "bif"  "bmet" "gst"  "bacw" "bacp" "bacb" "baco"
## [661] "bacy" "bacl" "balm" "bsm"  "bgy"  "bwh"  "bxi"  "bhk"  "bbev" "balt"
## [671] "bacs" "bsaf" "bit"  "bacq" "bcir" "bfd"  "bcoh" "bda"  "beo"  "bmq"
## [681] "bmd"  "bmh"  "bmeg" "bfx"  "bck"  "bag"  "bcoa" "bha"  "bcl"  "bpf"
## [691] "ble"  "bkw"  "bgi"  "bon"  "bko"  "oih"  "ocn"  "gka"  "gte"  "gtk"
## [701] "gtm"  "gli"  "gtn"  "gwc"  "gyc"  "gya"  "gct"  "gmc"  "ggh"  "gjf"
## [711] "gea"  "gse"  "gsr"  "gej"  "gth"  "ptl"  "ptb"  "afl"  "agn"  "anm"
## [721] "aamy" "anl"  "and"  "acai" "lsp"  "lgy"  "lfu"  "lys"  "lyb"  "lyz"
## [731] "lyg"  "lpak" "hhd"  "hmn"  "hli"  "vir"  "vhl"  "vig"  "vil"  "vne"
## [741] "vpn"  "vim"  "lao"  "fpn"  "far"  "sje"  "apak" "bsj"  "bmur" "pbut"
## [751] "pasa" "bthv" "psyh" "psyo" "prd"  "grc"  "rue"  "sale" "pof"  "nmk"
## [761] "ntm"  "meku" "aia"  "blen" "stea" "ssp"  "sca"  "sxy"  "sxl"  "sxo"
## [771] "seqo" "scv"  "snl"  "skl"  "sarl" "spic" "sscu" "sste" "mlen" "shv"
## [781] "esi"  "eat"  "ean"  "exm"  "exu"  "bbe"  "blr"  "bfm"  "bagr" "brw"
## [791] "pjd"  "gym"  "ppy"  "ppm"  "ppo"  "ppol" "ppq"  "ppoy" "pta"  "plv"
## [801] "psab" "pdu"  "pbd"  "pgm"  "pod"  "paen" "paef" "paeq" "pste" "paea"
## [811] "paee" "paeh" "paej" "pbj"  "pih"  "pri"  "ppeo" "pnp"  "pow"  "pbv"
## [821] "pxl"  "pswu" "pdh"  "pib"  "pcx"  "pkb"  "paih" "pvo"  "plw"  "plen"
## [831] "ppsc" "plut" "palb" "pchi" "pprt" "pbac" "prz"  "plyc" "anx"  "asoc"
## [841] "coh"  "cohn" "saca" "aac"  "aad"  "bts"  "kyr"  "tum"  "tab"  "siv"
```

```
##  [851] "ssil" "sob"  "pln"  "pku"  "prt"  "pll"  "pana" "pdg"  "phc"  "pmar"
##  [861] "ppla" "pfae" "plx"  "pmat" "pdec" "jeo"  "kur"  "spsy" "spor" "spop"
##  [871] "sure" "spos" "spae" "rst"  "panc" "play" "vij"  "ntr"  "lfb"  "tvu"
##  [881] "kpul" "keb"  "ega"  "ess"  "eav"  "crn"  "cml"  "cbe"  "cbz"  "cbei"
##  [891] "cls"  "csr"  "cpas" "cpat" "cpae" "csb"  "cdy"  "gfe"  "ruk"  "cle"
##  [901] "byl"  "bpro" "cbol" "sth"  "dsy"  "dhd"  "ddh"  "dgi"  "dor"  "dai"
##  [911] "dmi"  "tmr"  "thef" "thep" "say"  "sap"  "sthr" "has"  "hhl"  "sri"
##  [921] "puf"  "pft"  "mana" "sted" "lpil" "mle"  "mlb"  "cgl"  "cgb"  "cgu"
##  [931] "cgt"  "cgs"  "cgg"  "cgm"  "cgj"  "cgq"  "cgx"  "cef"  "cdi"  "cdp"
##  [941] "cdh"  "cdt"  "cde"  "cdr"  "cda"  "cdz"  "cdb"  "cds"  "cdd"  "cdw"
##  [951] "cdv"  "cdip" "cjk"  "cur"  "cua"  "car"  "ckp"  "cpl"  "cpg"  "cpp"
##  [961] "cpk"  "cpq"  "cpx"  "cpz"  "cor"  "cop"  "cod"  "cos"  "coi"  "coe"
##  [971] "cou"  "cpse" "cpsu" "cpsf" "crd"  "cul"  "cuc"  "cue"  "cun"  "cus"
##  [981] "cuq"  "cuz"  "cuj"  "cva"  "ccn"  "cter" "cmd"  "caz"  "cfn"  "ccg"
##  [991] "cvt"  "cii"  "coa"  "cdo"  "chm"  "csx"  "cmq"  "ccj"  "cmv"  "cei"
## [1001] "cted" "clw"  "cdx"  "csp"  "csta" "ccjz" "cpho" "cfc"  "cgv"  "cstr"
## [1011] "caqu" "csph" "camg" "cmin" "cpeg" "cxe"  "cee"  "csan" "cgk"  "crf"
## [1021] "crl"  "ccho" "cpre" "cpso" "csur" "bfv"  "dit"  "sfa"  "strp" "lxl"
## [1031] "lxx"  "lxy"  "mix"  "mih"  "maur" "mfol" "moo"  "mlv"  "msed" "rla"
## [1041] "rpla" "aqg"  "rtx"  "rtc"  "cum"  "cub"  "cug"  "cqf"  "mvd"  "frp"
## [1051] "agy"  "cry"  "malk" "myl"  "salc" "sala" "sald" "hum"  "huw"  "lyk"
## [1061] "leu"  "leb"  "ldn"  "frn"  "arw"  "acit" "ari"  "gar"  "krh"  "kpl"
## [1071] "kii"  "krs"  "kod"  "kvr"  "mlu"  "rter" "rama" "rkr"  "aul"  "brv"
## [1081] "bgg"  "brz"  "brr"  "dva"  "jde"  "kse"  "dni"  "day"  "xce"  "xyl"
## [1091] "iva"  "ido"  "cet"  "cceu" "xya"  "ske"  "sanw" "cfl"  "cfi"  "cga"
## [1101] "cez"  "celz" "cej"  "celh" "oek"  "psei" "ars"  "teh"  "phw"  "serj"
## [1111] "bly"  "blin" "bri"  "blut" "bcau" "dco"  "gez"  "halt" "pfr"  "pfre"
## [1121] "paus" "pbo"  "aaci" "acij" "aji"  "tfl"  "tfa"  "tes"  "tez"  "tdf"
## [1131] "tla"  "prv"  "ndk"  "noy"  "noi"  "ndp"  "nbe"  "aez"  "aeb"  "aef"
## [1141] "mgg"  "tfu"  "ace"  "nml"  "nak"  "psea" "acti" "acad" "tbw"  "ard"
## [1151] "ahw"  "fsl"  "flh"  "plan" "plak" "psuf" "pvn"  "abai" "rxy"  "rrd"
## [1161] "rub"  "afo"  "aym"  "atq"  "erz"  "syn"  "syz"  "syy"  "syt"  "sys"
## [1171] "syq"  "syj"  "syo"  "syc"  "syf"  "syw"  "syd"  "syg"  "syr"  "syx"
## [1181] "syp"  "syne" "synp" "synk" "synr" "synd" "syu"  "synw" "slw"  "syv"
```

```
## [1191] "syl"  "sync" "tel"  "thn"  "tvn"  "thec" "cgc"  "cyi"  "dsl"  "cmp"
## [1201] "lep"  "len"  "let"  "lbo"  "hhg"  "pseu" "pser" "pmt"  "pmi"  "pmf"
## [1211] "pmg"  "theu" "glp"  "gen"  "gee"  "chon" "mar"  "mpk"  "miq"  "mvz"
## [1221] "can"  "csn"  "cyl"  "hao"  "enn"  "cyt"  "cwa"  "cyp"  "cyh"  "cyc"
## [1231] "cyj"  "cyn"  "ter"  "mic"  "arp"  "pagh" "oxy"  "lfs"  "gei"  "oac"
## [1241] "oni"  "mpro" "cep"  "gvi"  "glj"  "ana"  "npu"  "nos"  "nop"  "non"
## [1251] "nfl"  "noe"  "nsh"  "ned"  "ava"  "anb"  "acy"  "awa"  "ann"  "csg"
## [1261] "calo" "calt" "calh" "fis"  "dou"  "dfs"  "ccur" "toq"  "ncn"  "cthe"
## [1271] "plp"  "scs"  "stan" "ceo"  "cer"  "rrs"  "rca"  "cag"  "hau"  "tro"
## [1281] "sti"  "pbf"  "tbh"  "ttr"  "dra"  "dge"  "ddr"  "dmr"  "dpt"  "dgo"
## [1291] "dpd"  "dsw"  "dch"  "dab"  "dpu"  "dez"  "dwu"  "dfc"  "dein" "dga"
## [1301] "tth"  "ttj"  "tts"  "ttl"  "tsc"  "thc"  "tos"  "tbc"  "mrb"  "mre"
## [1311] "msv"  "mtai" "mhd"  "ccz"  "puv"  "ote"  "obg"  "vbh"  "obt"  "caa"
## [1321] "amu"  "min"  "mkc"  "meap" "vba"  "vbs"  "rba"  "psl"  "pir"  "rul"
## [1331] "mff"  "rol"  "bvo"  "ttf"  "lpav" "amuc" "pnd"  "plm"  "peh"  "pbs"
## [1341] "plh"  "fmr"  "gim"  "mri"  "plon" "tim"  "uli"  "ipa"  "agv"  "phm"
## [1351] "pcor" "vbc"  "tpi"  "slr"  "lil"  "lie"  "lic"  "lis"  "lbj"  "lbl"
## [1361] "lbi"  "lbf"  "lst"  "lmay" "lwl"  "tpx"  "aca"  "tsa"  "trs"  "thyd"
## [1371] "fva"  "ful"  "fmo"  "ipo"  "cpor" "fsu"  "bvu"  "bdo"  "bdh"  "pbt"
## [1381] "pet"  "psac" "dys"  "ppn"  "osp"  "ait"  "ttz"  "blq"  "dori" "asx"
## [1391] "mbas" "srm"  "rbar" "nia"  "arb"  "ark"  "agi"  "arac" "fls"  "sgn"
## [1401] "pgs"  "pej"  "psn"  "sphn" "smiz" "spsc" "sphz" "sphe" "spdr" "stha"
## [1411] "chu"  "fli"  "hqi"  "fpf"  "gfo"  "grl"  "fps"  "fpc"  "fpy"  "fpo"
## [1421] "fpq"  "fpv"  "fpw"  "fpk"  "fpsz" "fjg"  "fbr"  "fco"  "fin"  "fgl"
## [1431] "ffa"  "fat"  "fki"  "fpal" "fmg"  "falb" "fcr"  "fse"  "fsn"  "fnk"
## [1441] "fak"  "ccyn" "caph" "csto" "capq" "rbi"  "zpr"  "cat"  "cly"  "clh"
## [1451] "kdi"  "dok"  "ddo"  "dod"  "lan"  "lvn"  "laci" "zga"  "mlt"  "asl"
## [1461] "aev"  "nom"  "nsd"  "noj"  "myr"  "mpw"  "mod"  "myz"  "wij"  "ahz"
## [1471] "tdi"  "ten"  "tje"  "tmar" "tmp"  "lut"  "lul"  "wfu"  "for"  "foh"
## [1481] "oll"  "oaq"  "fek"  "taj"  "aue"  "spon" "kos"  "aqd"  "psyn" "oci"
## [1491] "mgel" "mesq" "gaa"  "cagg" "alti" "fba"  "fbu"  "ran"  "rai"  "rag"
## [1501] "rae"  "rat"  "wvi"  "eao"  "emn"  "een"  "elb"  "emg"  "ego"  "egm"
## [1511] "elz"  "elt"  "chz"  "cgn"  "cih"  "chh"  "cio"  "chry" "cpip" "chrs"
## [1521] "chrz" "carh" "csha" "cnk"  "cjt"  "cil"  "ccau" "cben" "cjg"  "ccas"
```

```
## [1531] "cant" "kda"  "clac" "eva"  "ctak" "cnr"  "ebv"  "efal" "este" "civ"
## [1541] "fte"  "flu"  "oho"  "bbau" "cpb"  "ial"  "mro"  "cprv" "hya"  "tal"
## [1551] "ttk"  "din"  "fsi"  "caby" "nio"  "lfc"  "lfi"  "lfp"  "leg"  "nli"
## [1561] "mox"  "hje"  "hma"  "hhi"  "hhn"  "halj" "hali" "hpel" "haq"  "haj"
## [1571] "hra"  "halq" "srub" "halg" "htu"  "hjt"  "npe"  "hlr"  "nax"  "pto"
## [1581] "cdiv" "soh"  "sso"  "sol"  "ssoa" "ssol" "ssof" "sai"  "sacn" "sacr"
## [1591] "sacs" "sis"  "sia"  "sim"  "sid"  "siy"  "sin"  "sii"  "sih"  "sir"
## [1601] "sic"  "sula" "sule" "aman" "sacd"
```

Organisms classified within cluster 6

```
##   [1] "hsa"  "ptr"  "pps"  "ggo"  "pon"  "nle"  "mcc"  "mcf"  "csab" "caty"
##  [11] "panu" "rro"  "rbb"  "tfn"  "pteh" "cjc"  "sbq"  "mmur" "mmu"  "mcal"
##  [21] "mpah" "rno"  "mcoc" "mun"  "cge"  "pleu" "ngi"  "hgl"  "ccan" "ocu"
##  [31] "opi"  "tup"  "cfa"  "vvp"  "vlg"  "aml"  "umr"  "uah"  "oro"  "elk"
##  [41] "mpuf" "eju"  "mlx"  "fca"  "pyu"  "pbg"  "ptg"  "ppad" "aju"  "hhv"
##  [51] "bta"  "bom"  "biu"  "bbub" "chx"  "oas"  "oda"  "ccad" "ssc"  "cfr"
##  [61] "cbai" "cdk"  "bacu" "lve"  "oor"  "dle"  "pcad" "ecb"  "epz"  "eai"
##  [71] "myb"  "myd"  "mmyo" "mna"  "pkl"  "hai"  "dro"  "shon" "ajm"  "pdic"
##  [81] "mmf"  "rfq"  "pale" "pgig" "ray"  "mjv"  "tod"  "lav"  "tmu"  "mdo"
##  [91] "gas"  "shr"  "pcw"  "oaa"  "gga"  "pcoc" "mgp"  "cjo"  "nmel" "apla"
## [101] "acyg" "tgu"  "lsr"  "scan" "pmoa" "otc"  "pruf" "gfr"  "fab"  "phi"
## [111] "pmaj" "ccae" "ccw"  "etl"  "fpg"  "fch"  "clv"  "egz"  "nni"  "acun"
## [121] "padl" "aam"  "arow" "npd"  "dne"  "asn"  "amj"  "cpoo" "ggn"  "pss"
## [131] "cmy"  "cpic" "tst"  "cabi" "acs"  "pvt"  "sund" "pbi"  "pmur" "tsr"
## [141] "pgut" "vko"  "pmua" "zvi"  "gja"  "xla"  "xtr"  "npr"  "dre"  "srx"
## [151] "sanh" "sgh"  "ccar" "caua" "ipu"  "phyp" "amex" "eee"  "tru"  "tng"
## [161] "lco"  "ncc"  "cgob" "ely"  "plep" "sluc" "ecra" "pflv" "gat"  "ppug"
## [171] "msam" "cud"  "mze"  "onl"  "oau"  "ola"  "oml"  "xma"  "xco"  "xhe"
## [181] "pret" "cvg"  "ctul" "nfu"  "kmr"  "alim" "aoce" "csem" "pov"  "ssen"
## [191] "lcf"  "sdu"  "slal" "xgl"  "hcq"  "bpec" "malb" "sasa" "otw"  "omy"
## [201] "salp" "snh"  "els"  "sfm"  "pki"  "aang" "loc"  "pspa" "arut" "lcm"
## [211] "cmk"  "rtp"  "bfo"  "bbel" "cin"  "sclv" "spu"  "aplc" "sko"  "dme"
```

```
## [221] "der"  "dse"  "dsi"  "dya"  "dan"  "dsr"  "dpo"  "dpe"  "dmn"  "dwi"
## [231] "dgr"  "dmo"  "daz"  "dnv"  "dhe"  "ccat" "bod"  "mde"  "scac" "lcq"
## [241] "aga"  "acoz" "aara" "aag"  "aalb" "cqu"  "cpii" "ame"  "acer" "bim"
## [251] "bbif" "bvk"  "bvan" "bter" "ccal" "obb"  "mgen" "nmea" "cgig" "soc"
## [261] "mpha" "aec"  "acep" "pbar" "vem"  "hst"  "dqu"  "cfo"  "fex"  "lhu"
## [271] "pgc"  "obo"  "pcf"  "pfuc" "vps"  "nvi"  "csol" "tpre" "mdl"  "cglo"
## [281] "fas"  "dam"  "ccin" "tca"  "dpa"  "atd"  "agb"  "ldc"  "nvl"  "apln"
## [291] "ppyr" "otu"  "bmor" "bman" "msex" "dpl"  "bany" "pmac" "ppot" "pxu"
## [301] "prap" "zce"  "haw"  "tnl"  "pxy"  "api"  "dnx"  "ags"  "rmd"  "btab"
## [311] "dci"  "clec" "hhal" "nlu"  "phu"  "foc"  "zne"  "csec" "dpx"  "dmk"
## [321] "pvm"  "pja"  "hame" "hazt" "eaf"  "isc"  "dsv"  "rsan" "rmp"  "vde"
## [331] "vja"  "dpte" "cscu" "ptep" "sdm"  "hro"  "pcan" "bgt"  "gae"  "pmax"
## [341] "obi"  "osn"  "lak"  "epa"  "aten" "adf"  "amil" "pdam" "spis" "dgt"
## [351] "hmg"
```

Pyramid match (PM) kernel

Heatmap

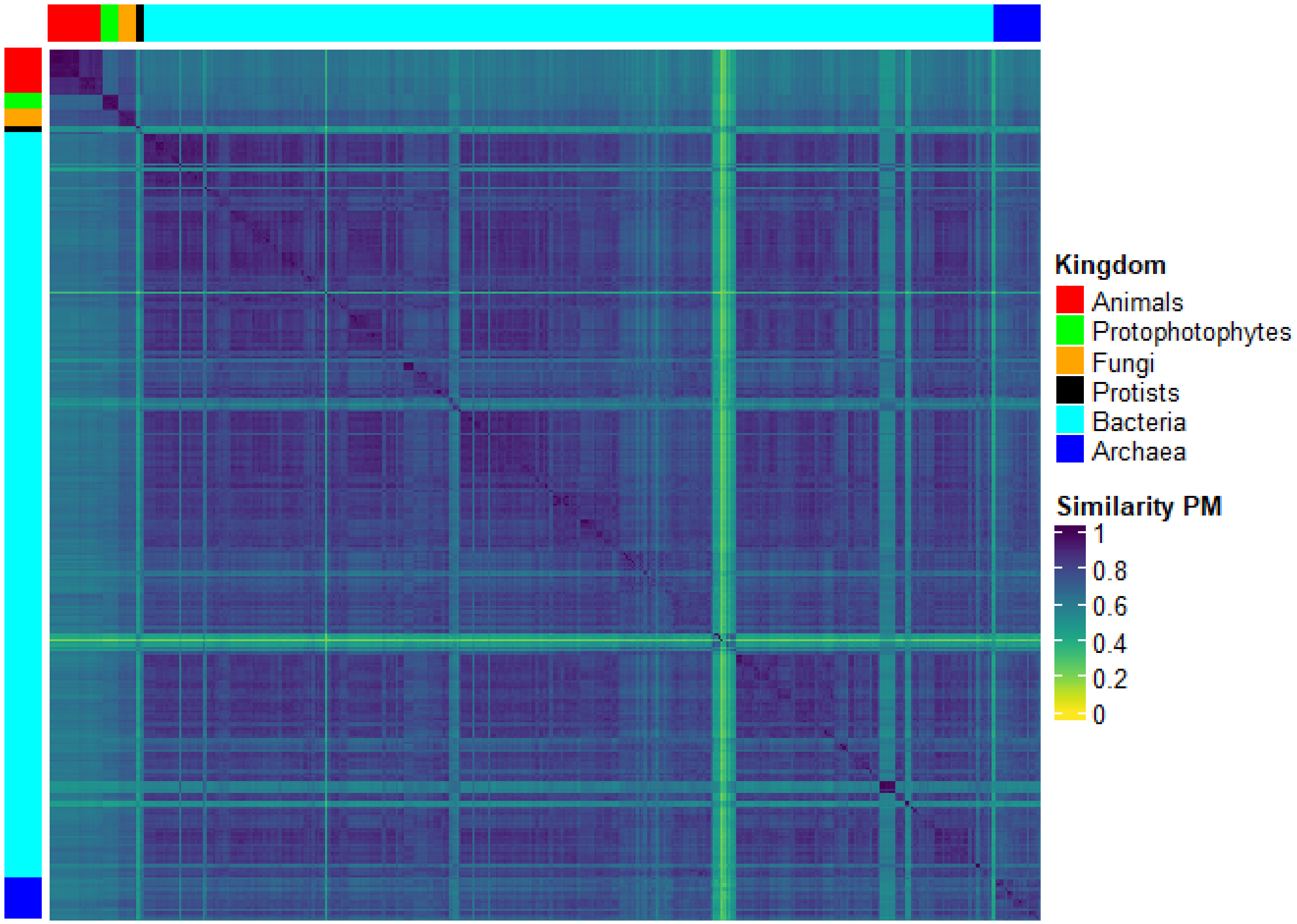
Kingdom
Animals
Protophotophytes
Fungi
Protists
Bacteria
Archaea
Similarity PM
1
0.8
0.6
0.4
0.2
0

MDS for PM

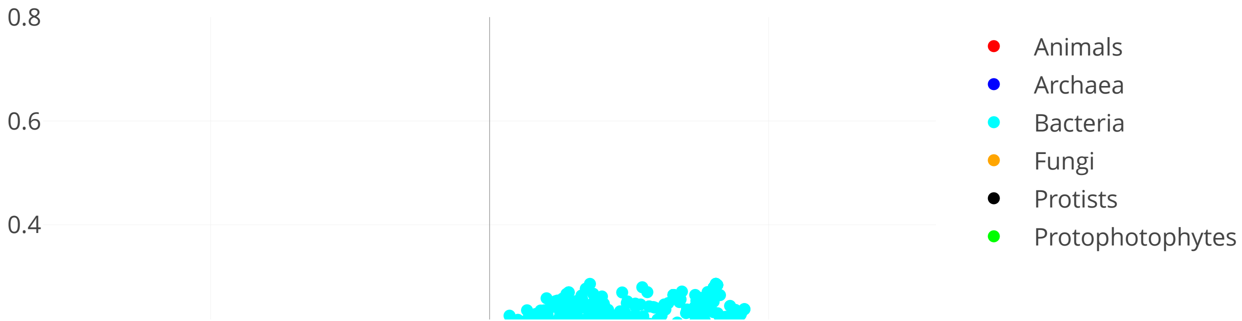
0.8
0.6
0.4
Animals
Archaea
Bacteria
Fungi
Protists
Protophotophytes

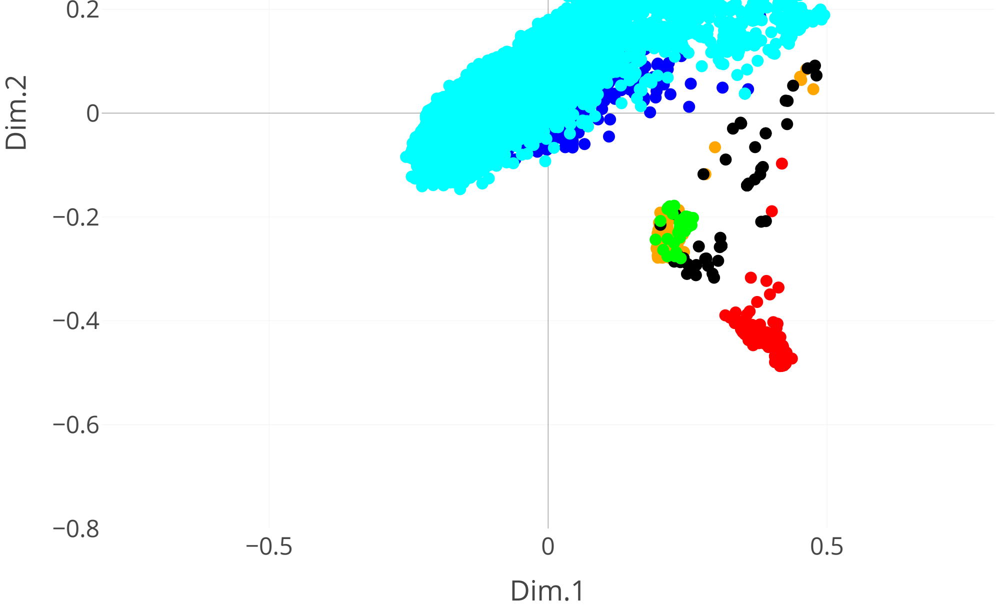

6-Means clustering for PM kernel

```
##                    Cluster
## Real group            1    2    3    4    5    6
##   Animals             3    0    0  367    0    0
##   Archaea             0   12    5    0  174  148
##   Bacteria            0  632 2553    0 1528 1402
##   Fungi             133    5    0    0    0    0
##   Protists           42   10    0    0    0    0
##   Protophotophytes  127    0    0    0    0    0
```

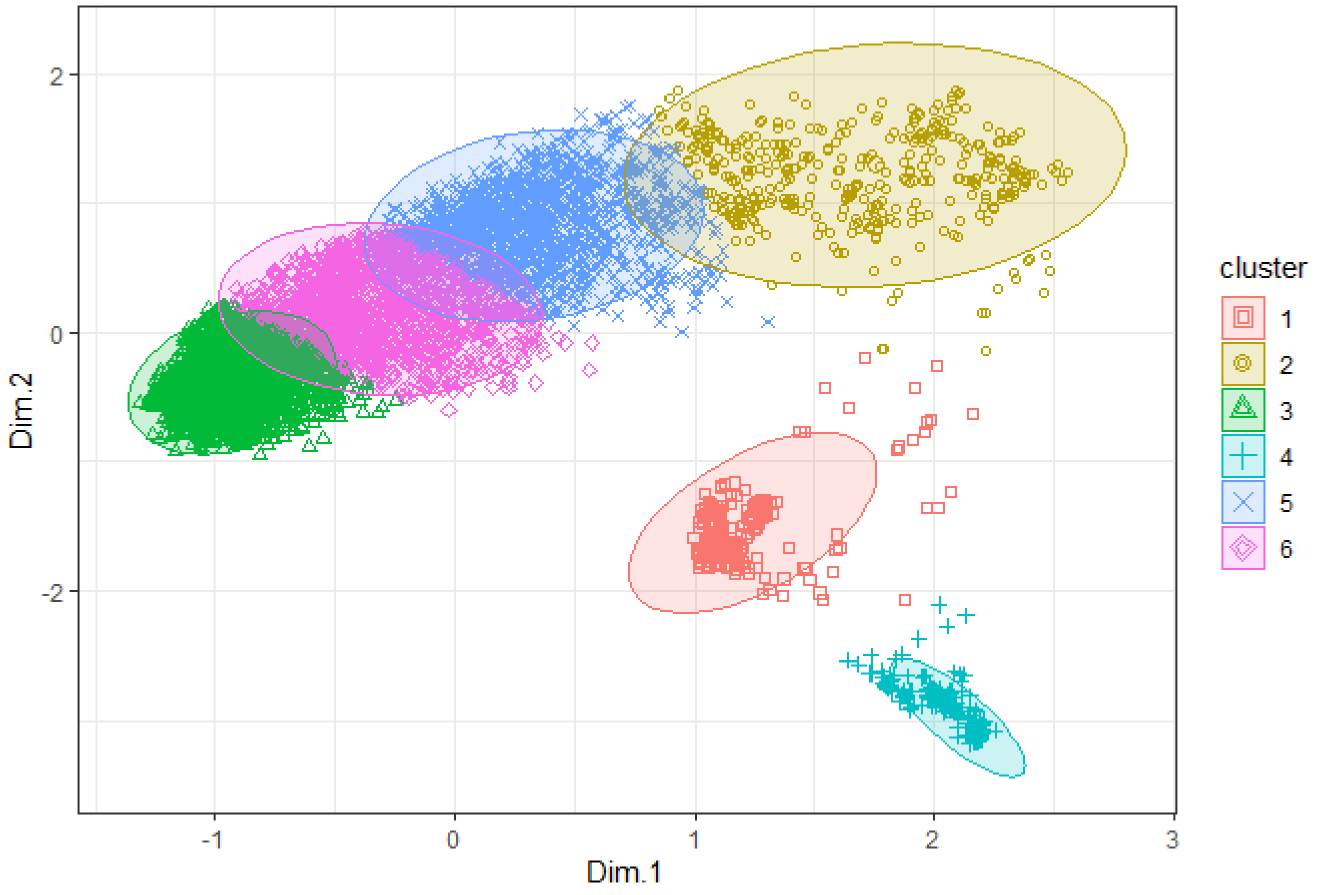

Organisms classified within cluster 1

```
##   [1] "tsp"  "shx"  "egl"  "ath"  "aly"  "crb"  "csat" "eus"  "brp"  "bna"
##  [11] "boe"  "rsz"  "thj"  "cpap" "cit"  "cic"  "pvy"  "minc" "tcc"  "gra"
##  [21] "ghi"  "gab"  "dzi"  "egr"  "gmx"  "gsj"  "pvu"  "vra"  "var"  "vun"
##  [31] "ccaj" "aprc" "mtr"  "cam"  "lja"  "adu"  "aip"  "ahf"  "lang" "fve"
##  [41] "rcn"  "pper" "pmum" "pavi" "pdul" "mdm"  "pxb"  "zju"  "mnt"  "csv"
##  [51] "cmo"  "bhj"  "mcha" "cmax" "cmos" "cpep" "rcu"  "jcu"  "hbr"  "mesc"
##  [61] "pop"  "peu"  "palz" "jre"  "qsu"  "qlo"  "twl"  "vvi"  "vri"  "sly"
##  [71] "spen" "sot"  "cann" "nta"  "nsy"  "nto"  "nau"  "ini"  "itr"  "sind"
##  [81] "oeu"  "egt"  "sspl" "han"  "ecad" "lsv"  "ccav" "dcr"  "csin" "bvg"
```

```
##  [91] "soe"  "cqi"  "nnu"  "ming" "psom" "ncol" "osa"  "dosa" "obr"  "bdi"
## [101] "ats"  "tdc"  "sbi"  "zma"  "sita" "pvir" "phai" "pda"  "egu"  "mus"
## [111] "dct"  "peq"  "aof"  "atr"  "smo"  "ppp"  "cre"  "vcn"  "mng"  "csl"
## [121] "cvr"  "apro" "olu"  "ota"  "bpg"  "mis"  "mpp"  "cme"  "gsl"  "ccp"
## [131] "sce"  "ago"  "erc"  "kla"  "kmx"  "lth"  "vpo"  "zro"  "cgr"  "ncs"
## [141] "ndi"  "tpf"  "tbl"  "tdl"  "tgb"  "kaf"  "zmk"  "ppa"  "dha"  "pic"
## [151] "pgu"  "spaa" "lel"  "cal"  "ctp"  "cot"  "cdu"  "cten" "yli"  "clu"
## [161] "clus" "caur" "slb"  "pkz"  "bnn"  "bbrx" "ncr"  "nte"  "smp"  "pan"
## [171] "ttt"  "mtm"  "cthr" "mgr"  "tmn"  "ssck" "fgr"  "fpu"  "fvr"  "fox"
## [181] "nhe"  "tre"  "trr"  "maw"  "maj"  "cmt"  "plj"  "val"  "vda"  "cfj"
## [191] "sapo" "ela"  "pfy"  "ssl"  "bfu"  "mbe"  "psco" "glz"  "ani"  "afm"
## [201] "act"  "nfi"  "aor"  "ang"  "afv"  "pcs"  "pdp"  "tmf"  "trg"  "cim"
## [211] "cpw"  "ure"  "pbl"  "pbn"  "abe"  "tve"  "aje"  "bgh"  "pno"  "pte"
## [221] "bze"  "bsc"  "bor"  "aalt" "ztr"  "pfj"  "bcom" "npa"  "tml"  "spo"
## [231] "cne"  "cnb"  "cgi"  "tms"  "tasa" "ppl"  "tvs"  "dsq"  "pco"  "shs"
## [241] "hir"  "psq"  "adl"  "fme"  "gtr"  "lbc"  "mpr"  "mrr"  "cci"  "scm"
## [251] "abp"  "abv"  "cput" "sla"  "wse"  "wic"  "uma"  "pfp"  "mgl"  "mrt"
## [261] "msym" "pgr"  "mlr"  "mbr"  "sre"  "ddi"  "dpp"  "dfa"  "acan" "pfa"
## [271] "pfd"  "pfh"  "pyo"  "pcb"  "pbe"  "pkn"  "pvx"  "pcy"  "cpv"  "cho"
## [281] "tgo"  "tet"  "ptm"  "smin" "pti"  "fcy"  "tps"  "ngd"  "aaf"  "pif"
## [291] "psoj" "spar" "ehx"  "gtt"  "tbr"  "tbg"  "tcr"  "lma"  "lif"  "ldo"
## [301] "lmi"  "lbz"  "lpan" "ngr"  "tva"
```

Organisms classified within cluster 2

```
##   [1] "ecu"  "ein"  "ehe"  "ero"  "nce"  "ehi"  "edi"  "eiv"  "tan"  "tpv"
##  [11] "tot"  "beq"  "bbo"  "bmic" "gla"  "bfl"  "bpn"  "bva"  "bchr" "ben"
##  [21] "bed"  "hde"  "sect" "sehc" "senm" "rip"  "rig"  "men"  "meo"  "sbw"
##  [31] "den"  "hed"  "ged"  "cmik" "ppet" "ssz"  "seny" "ehd"  "buc"  "bap"
##  [41] "bau"  "baw"  "bajc" "bua"  "bup"  "bak"  "buh"  "bapf" "bapg" "bapu"
##  [51] "bapw" "bas"  "bab"  "bcc"  "baj"  "baph" "wbr"  "wgl"  "asy"  "aen"
##  [61] "cey"  "cea"  "cend" "les"  "ple"  "ply"  "plr"  "plo"  "pld"  "plb"
##  [71] "plc"  "pli"  "paly" "crp"  "cru"  "crc"  "crt"  "crh"  "crv"  "cri"
```

```
##  [81] "eme"  "dno"  "eof"  "bci"  "bcib" "bcig" "zin"  "tpn"  "tpq"  "tpj" 
##  [91] "kbl"  "kbt"  "kde"  "kga"  "kon"  "kso"  "ssdc" "ndl"  "vfg"  "lip" 
## [101] "lir"  "rpr"  "rpo"  "rpw"  "rpz"  "rpg"  "rps"  "rpv"  "rpq"  "rpl" 
## [111] "rpn"  "rty"  "rtt"  "rtb"  "rcm"  "rcc"  "rbe"  "rbo"  "rco"  "rfe" 
## [121] "rak"  "rri"  "rrj"  "rra"  "rrc"  "rrh"  "rrb"  "rrn"  "rrp"  "rrm" 
## [131] "rrr"  "rms"  "rmi"  "rpk"  "raf"  "rhe"  "rja"  "rsv"  "rsw"  "rph" 
## [141] "rau"  "rmo"  "rpp"  "rre"  "ram"  "rab"  "rmc"  "ras"  "ots"  "ott" 
## [151] "ptc"  "wol"  "wri"  "wen"  "wed"  "wpi"  "wbm"  "woo"  "wcl"  "weo" 
## [161] "wpp"  "ama"  "amf"  "amw"  "amp"  "acn"  "aph"  "apy"  "apd"  "apha"
## [171] "aoh"  "eru"  "erw"  "erg"  "ecn"  "ech"  "echa" "echj" "echl" "echs"
## [181] "echv" "echw" "echp" "emr"  "ehh"  "nse"  "nri"  "nhm"  "nef"  "mmn" 
## [191] "fso"  "eaa"  "las"  "laa"  "lat"  "lso"  "lar"  "lau"  "hci"  "hct" 
## [201] "hcc"  "hcd"  "stc"  "stl"  "sths" "smb"  "std"  "sie"  "sib"  "siu" 
## [211] "sig"  "stra" "ski"  "sgw"  "ljo"  "ljf"  "ljh"  "ljn"  "lac"  "lad" 
## [221] "laf"  "ldb"  "lbu"  "ldl"  "lga"  "lhe"  "lhl"  "lhr"  "lhv"  "lhd" 
## [231] "lcr"  "lam"  "lai"  "lay"  "lke"  "law"  "lae"  "lje"  "lamy" "lpw" 
## [241] "lkl"  "lapi" "lhs"  "lsn"  "lfv"  "lji"  "ppe"  "ppen" "pdm"  "pio" 
## [251] "lsa"  "lgm"  "lah"  "ldx"  "wko"  "wce"  "wct"  "wci"  "wcf"  "wdi" 
## [261] "mps"  "mpx"  "vah"  "dpm"  "asf"  "asm"  "aso"  "asb"  "clo"  "fsa" 
## [271] "pbq"  "bprm" "fma"  "pmic" "eri"  "tur"  "mge"  "mgu"  "mgc"  "mgq" 
## [281] "mgx"  "mpn"  "mpm"  "mpj"  "mpb"  "mpe"  "mga"  "mgh"  "mgf"  "mgn" 
## [291] "mgs"  "mgt"  "mgv"  "mgw"  "mgac" "mgan" "mgnc" "mgz"  "mmy"  "mmym"
## [301] "mmyi" "mml"  "mcp"  "mcac" "mcap" "mcar" "mcai" "mlc"  "mlh"  "mmo" 
## [311] "mhy"  "mhj"  "mhp"  "mhn"  "mhyl" "mhyo" "mat"  "mco"  "mho"  "mhom"
## [321] "mcd"  "mhr"  "mhh"  "mhm"  "mhs"  "mhv"  "mha"  "mhf"  "mss"  "msk" 
## [331] "mpf"  "mput" "mhe"  "mwe"  "mhl"  "mhb"  "mpv"  "mov"  "mbc"  "mgj" 
## [341] "mfq"  "mcan" "myt"  "mds"  "myg"  "mpho" "mhyv" "mclo" "mamp" "mans"
## [351] "mphc" "miw"  "mane" "mnh"  "mnu"  "mstr" "mcr"  "mcm"  "mgb"  "mgly"
## [361] "mcou" "mcom" "mpu"  "msy"  "mso"  "maa"  "mal"  "mfr"  "mfm"  "mfp" 
## [371] "mbv"  "mbh"  "mbi"  "mbq"  "mcy"  "mcas" "mck"  "marg" "mpul" "mbov"
## [381] "mboh" "mani" "mphi" "uur"  "upa"  "upr"  "uue"  "hcr"  "poy"  "ayw" 
## [391] "mbp"  "pml"  "pal"  "nzs"  "psol" "pzi"  "abra" "apal" "aaxa" "ahk" 
## [401] "mfl"  "mfw"  "mchc" "mlac" "ment" "msyr" "mtab" "mcol" "elj"  "esx" 
## [411] "efr"  "eml"  "scr"  "ssyr" "sdi"  "stai" "sapi" "smir" "smia" "scq" 
```

```
## [421] "ssab" "satr" "seri" "stur" "sll"  "skn"  "scj"  "shj"  "sck"  "sfz"
## [431] "scou" "scla" "sprn" "spit" "stab" "sphh" "smoo" "salx" "sgq"  "schi"
## [441] "nfe"  "nsp"  "ctr"  "ctd"  "ctf"  "ctrd" "ctro" "ctrt" "cta"  "cty"
## [451] "cra"  "ctrq" "ctrx" "ctrz" "ctrp" "ctlj" "ctlx" "ctll" "ctb"  "ctrr"
## [461] "ctlf" "ctli" "ctl"  "ctru" "ctrl" "ctrv" "ctrm" "ctla" "ctlm" "ctls"
## [471] "ctlz" "ctlc" "ctln" "ctlb" "ctlq" "cto"  "ctrn" "ctj"  "ctz"  "ctg"
## [481] "ctk"  "csw"  "ces"  "ctrb" "ctre" "ctrs" "ctec" "cfs"  "cfw"  "ctfw"
## [491] "ctrf" "ctch" "ctn"  "ctq"  "ctv"  "ctw"  "ctrg" "ctri" "ctra" "ctrh"
## [501] "ctrj" "ctrk" "ctjt" "ctcf" "ctfs" "cthf" "ctcj" "cthj" "ctmj" "cttj"
## [511] "ctjs" "ctrc" "ctrw" "ctry" "ctct" "cmu"  "cmur" "cmn"  "cmm"  "cmg"
## [521] "cmx"  "cmz"  "cpn"  "cpa"  "cpj"  "cpt"  "clp"  "cpm"  "cpec" "cpeo"
## [531] "cper" "chp"  "chb"  "chs"  "chi"  "cht"  "chc"  "chr"  "cpsc" "cpsn"
## [541] "cpsb" "cpsg" "cpsm" "cpsi" "cpsv" "cpsw" "cpst" "cpsd" "cpsa" "cav"
## [551] "cca"  "cab"  "cabo" "cfe"  "cgz"  "chla" "pcu"  "ney"  "psup" "bbu"
## [561] "bbz"  "bbn"  "bbj"  "bbur" "bga"  "bgb"  "bgn"  "bgs"  "bgc"  "baf"
## [571] "bafz" "bafh" "baft" "bafe" "bbs"  "bvt"  "bchi" "bmay" "btu"  "bhr"
## [581] "bhi"  "bdu"  "bre"  "bcw"  "bmo"  "bmiy" "bpak" "bane" "btur" "bmat"
## [591] "tpa"  "tpw"  "tpp"  "tpu"  "tph"  "tpo"  "tpas" "tpc"  "tpg"  "tpm"
## [601] "tpb"  "tpl"  "trm"  "trz"  "eti"  "rsd"  "smf"  "sns"  "sbr"  "aas"
## [611] "che"  "cec"  "cher" "smg"  "sms"  "smh"  "sum"  "smv"  "smub" "smum"
## [621] "smue" "smup" "bpi"  "bmm"  "bcp"  "bbq"  "blck" "elv"  "udi"  "cex"
## [631] "saal" "sbe"  "sbag" "sox"  "srb"  "srg"  "caqa" "dpb"  "tmg"  "wwe"
## [641] "bgw"  "bbgw" "mib"  "wba"  "pwo"  "cgw"  "baab" "nac"  "dfd"  "tpe"
## [651] "thb"  "tcb"  "thf"  "thel" "neq"  "naa"  "marh" "flt"  "agw"
```

Organisms classified within cluster 3

```
##   [1] "eco"  "ecj"  "ecd"  "ebw"  "ecok" "ece"  "ecs"  "ecf"  "etw"  "elx"
##  [11] "eoi"  "eoj"  "eoh"  "ecoo" "ecoh" "esl"  "eso"  "esm"  "eck"  "ecg"
##  [21] "eok"  "elr"  "elh"  "ecw"  "eun"  "ecp"  "ena"  "ecos" "ecv"  "ecoa"
##  [31] "ecx"  "ecm"  "ecy"  "ecr"  "ecq"  "eum"  "ect"  "eoc"  "ebr"  "ebl"
##  [41] "ebe"  "ebd"  "eci"  "eih"  "ecz"  "ecc"  "elo"  "eln"  "ese"  "ecl"
##  [51] "eko"  "ekf"  "eab"  "edh"  "edj"  "elu"  "elw"  "ell"  "elc"  "eld"
```

```
##  [61] "elp"  "elf"  "ecol" "ecoi" "ecoj" "efe"  "eal"  "ema"  "esz"  "sty"
##  [71] "stt"  "sex"  "sent" "stm"  "seo"  "sev"  "sey"  "sem"  "sej"  "seb"
##  [81] "sef"  "setu" "setc" "senr" "send" "seni" "seen" "spt"  "sek"  "spq"
##  [91] "sei"  "sec"  "seh"  "shb"  "senh" "seeh" "see"  "senn" "sew"  "sea"
## [101] "sens" "sed"  "seg"  "sel"  "sega" "set"  "sena" "seno" "senv" "senq"
## [111] "senl" "senj" "seec" "seeb" "seep" "senb" "sene" "senc" "ses"  "sbg"
## [121] "sbz"  "sbv"  "salz" "sfl"  "sfx"  "sfv"  "sfe"  "sfn"  "sfs"  "sft"
## [131] "ssn"  "sbo"  "sbc"  "sdy"  "sdz"  "shq"  "enc"  "enl"  "eclg" "ecle"
## [141] "ecln" "ecli" "eclx" "ecly" "eclz" "eclo" "ehm"  "exf"  "ecla" "eclc"
## [151] "eau"  "ekb"  "eno"  "eec"  "elg"  "ecan" "ern"  "ecls" "echg" "esh"
## [161] "ent"  "eas"  "enr"  "enx"  "enf"  "ebg"  "end"  "esa"  "csk"  "csz"
## [171] "csj"  "ccon" "cdm"  "csi"  "cmj"  "cui"  "cmw"  "ctu"  "kpn"  "kpu"
## [181] "kpm"  "kpp"  "kph"  "kpz"  "kpv"  "kpw"  "kpy"  "kpg"  "kpc"  "kpq"
## [191] "kpt"  "kpo"  "kpr"  "kpj"  "kpi"  "kpa"  "kps"  "kpx"  "kpb"  "kpne"
## [201] "kpnu" "kpnk" "kva"  "kpe"  "kpk"  "kvd"  "kvq"  "kox"  "koe"  "koy"
## [211] "kom"  "kmi"  "kok"  "koc"  "kqu"  "eae"  "ear"  "kqv"  "kll"  "klw"
## [221] "cro"  "cko"  "cfd"  "cbra" "cwe"  "cyo"  "cpot" "cfq"  "cama" "caf"
## [231] "cif"  "cfar" "cir"  "cie"  "cpar" "ebt"  "ror"  "ron"  "rpln" "rao"
## [241] "rtg"  "ree"  "cnt"  "cem"  "cen"  "clap" "pge"  "esc"  "kle"  "ksa"
## [251] "kor"  "krd"  "kco"  "kot"  "kpse" "kie"  "kas"  "lax"  "lei"  "leh"
## [261] "lee"  "ler"  "lea"  "laz"  "lef"  "lni"  "lew"  "buf"  "bage" "mety"
## [271] "ahn"  "yre"  "sgoe" "kin"  "pdz"  "ebf"  "ebc"  "ebu"  "psts" "izh"
## [281] "ype"  "ypk"  "yph"  "ypa"  "ypn"  "ypm"  "ypp"  "ypg"  "ypz"  "ypt"
## [291] "ypd"  "ypx"  "ypw"  "ypj"  "ypv"  "ypl"  "yps"  "ypo"  "ypi"  "ypy"
## [301] "ypb"  "ypq"  "ypu"  "ypr"  "ypc"  "ypf"  "yen"  "yep"  "yey"  "yel"
## [311] "yew"  "yet"  "yef"  "yee"  "ysi"  "yal"  "yfr"  "yin"  "ykr"  "yro"
## [321] "yru"  "yrb"  "yak"  "yma"  "yhi"  "yca"  "ymo"  "smar" "smac" "smw"
## [331] "spe"  "srr"  "srl"  "sry"  "sply" "srs"  "sra"  "smaf" "slq"  "serf"
## [341] "sers" "sfw"  "sfg"  "srz"  "sera" "serq" "serm" "squ"  "sfj"  "sof"
## [351] "ssur" "sfo"  "rah"  "raq"  "raa"  "rox"  "gqu"  "eame" "rbad" "eca"
## [361] "patr" "pato" "pct"  "pcc"  "pcv"  "pwa"  "ppar" "pec"  "pws"  "ppoa"
## [371] "pbra" "ppuj" "ddd"  "dda"  "dze"  "ddc"  "dzc"  "dso"  "ced"  "dfn"
## [381] "ddq"  "daq"  "dic"  "bgj"  "brb"  "bng"  "sod"  "eam"  "eay"  "eta"
## [391] "epy"  "epr"  "ebi"  "erj"  "ege"  "epe"  "erwi" "pam"  "plf"  "paj"
```

```
## [401] "paq"  "pva"  "pagg" "pao"  "kln"  "pant" "panp" "pagc" "pstw" "palh"
## [411] "pans" "pey"  "pdis" "pgz"  "pcd"  "mint" "mthi" "tci"  "plu"  "plum"
## [421] "pay"  "ptt"  "xbv"  "xnm"  "xdo"  "xho"  "psi"  "psx"  "psta" "prg"
## [431] "pala" "phei" "prq"  "pvc"  "hav"  "hpar" "lri"  "pshi" "xcc"  "xcb"
## [441] "xca"  "xcp"  "xcv"  "xax"  "xac"  "xci"  "xct"  "xcj"  "xcu"  "xcn"
## [451] "xcw"  "xcr"  "xcm"  "xcf"  "xfu"  "xao"  "xom"  "xoo"  "xop"  "xoy"
## [461] "xor"  "xoz"  "xal"  "xsa"  "xtn"  "xfr"  "xve"  "xpe"  "xhr"  "xga"
## [471] "xph"  "xva"  "xan"  "xar"  "xhy"  "xcz"  "xth"  "sml"  "smt"  "buj"
## [481] "smz"  "sacz" "stek" "srh"  "slm"  "sten" "stem" "stes" "psu"  "psuw"
## [491] "psd"  "pmex" "lab"  "laq"  "lcp"  "lgu"  "lez"  "lem"  "lmb"  "lyj"
## [501] "lsol" "lus"  "lug"  "thes" "tbv"  "xbc"  "rhd"  "rgl"  "dji"  "dtx"
## [511] "dko"  "lrz"  "lpy"  "xba"  "rbd"  "vch"  "vcf"  "vcs"  "vce"  "vcq"
## [521] "vcj"  "vci"  "vco"  "vcr"  "vcm"  "vcl"  "vcz"  "vvu"  "vvy"  "vvm"
## [531] "vvl"  "vpa"  "vpb"  "vpk"  "vpf"  "vph"  "vha"  "vca"  "vag"  "vex"
## [541] "vdb"  "vhr"  "vna"  "vow"  "vro"  "vsp"  "vej"  "vfu"  "vni"  "van"
## [551] "vau"  "vcy"  "vct"  "vtu"  "vfl"  "vmi"  "vbr"  "vga"  "vsh"  "vqi"
## [561] "vta"  "vaf"  "vnl"  "vcc"  "vas"  "vsr"  "ppr"  "pgb"  "pds"  "gho"
## [571] "saly" "sks"  "scot" "pae"  "paev" "paei" "pau"  "pap"  "pag"  "paf"
## [581] "pnc"  "paeb" "pdk"  "psg"  "prp"  "paep" "paer" "paem" "pael" "paes"
## [591] "paeu" "paeg" "paec" "paeo" "pmy"  "pmk"  "pre"  "ppse" "palc" "pcq"
## [601] "ppu"  "ppf"  "ppg"  "ppw"  "ppt"  "ppb"  "ppi"  "ppx"  "ppuh" "pput"
## [611] "ppun" "ppud" "pfv"  "pmon" "pmot" "pmos" "ppj"  "por"  "pst"  "psb"
## [621] "psyr" "psp"  "pamg" "pci"  "pavl" "pvd"  "pfl"  "pprc" "ppro" "pfo"
## [631] "pfs"  "pfe"  "pfc"  "pfn"  "ppz"  "pfb"  "pman" "ptv"  "pcg"  "pvr"
## [641] "pazo" "poi"  "pfw"  "pff"  "pfx"  "pen"  "psa"  "psz"  "psr"  "psc"
## [651] "psj"  "psh"  "pstu" "pstt" "pbm"  "plul" "pba"  "pbc"  "ppuu" "pdr"
## [661] "psv"  "psk"  "pkc"  "pch"  "pcz"  "pcp"  "pfz"  "plq"  "palk" "prh"
## [671] "psw"  "ppv"  "pses" "psem" "psec" "ppsy" "psos" "pkr"  "pfk"  "panr"
## [681] "ppsl" "pset" "psil" "pym"  "psed" "pke"  "pall" "pum"  "poj"  "pgg"
## [691] "ppsh" "pgy"  "avn"  "avl"  "avd"  "acx"  "pbb"  "pagr" "par"  "pcr"
## [701] "pso"  "pur"  "pali" "pspg" "psyg" "psyc" "psya" "psyy" "psyp" "acb"
## [711] "aby"  "abc"  "abn"  "abb"  "abx"  "abz"  "abr"  "abd"  "abh"  "abad"
## [721] "abj"  "abab" "abaj" "abaz" "abk"  "abau" "abaa" "abw"  "abal" "acc"
## [731] "ano"  "alc"  "acal" "acd"  "aci"  "att"  "aei"  "ajo"  "acw"  "acv"
```

```
##  [741] "ahl"  "ajn"  "asol" "ala"  "asj"  "adv"  "arj"  "awu"  "acum" "agu" 
##  [751] "aug"  "alw"  "ads"  "aber" "atn"  "achi" "alj"  "mos"  "mbah" "son" 
##  [761] "sdn"  "sfr"  "saz"  "sbl"  "sbm"  "sbn"  "sbp"  "sbt"  "sbs"  "sbb" 
##  [771] "slo"  "spc"  "shp"  "sse"  "spl"  "she"  "shm"  "shn"  "shw"  "shl" 
##  [781] "swd"  "swp"  "svo"  "shf"  "sja"  "spsw" "sbj"  "smav" "shew" "salg"
##  [791] "slj"  "smai" "spol" "sbk"  "skh"  "saes" "ilo"  "ili"  "ipi"  "idi" 
##  [801] "idt"  "cps"  "com"  "coz"  "colw" "cola" "cber" "cov"  "lsd"  "tht" 
##  [811] "thap" "pha"  "ptn"  "pat"  "psm"  "pseo" "pia"  "pphe" "pbw"  "prr" 
##  [821] "plz"  "paln" "ppis" "pea"  "pspo" "part" "ptu"  "png"  "ptd"  "psen"
##  [831] "pdj"  "paga" "pcar" "pmaa" "maq"  "mhc"  "mad"  "mbs"  "msr"  "mpq" 
##  [841] "mari" "mlq"  "msq"  "mara" "marj" "amc"  "amh"  "amaa" "amal" "amae"
##  [851] "amao" "amad" "amai" "amag" "amac" "amb"  "amg"  "amk"  "alt"  "aal" 
##  [861] "aaus" "asp"  "asq"  "aaw"  "alr"  "ale"  "alz"  "apel" "gag"  "gni" 
##  [871] "gps"  "pmes" "lal"  "cate" "salh" "salm" "salk" "hmi"  "pin"  "fbl" 
##  [881] "fes"  "mvs"  "mya"  "mmaa" "cja"  "cell" "cek"  "ceg"  "sde"  "ttu" 
##  [891] "saga" "spoi" "zal"  "osg"  "mthd" "micc" "maga" "mii"  "mict" "mhyd"
##  [901] "hja"  "halc" "kim"  "lph"  "lpu"  "lpm"  "lpc"  "lpe"  "llo"  "lfa" 
##  [911] "lok"  "lcd"  "lsh"  "llg"  "lib"  "lgt"  "ljr"  "lcj"  "lss"  "tmc" 
##  [921] "cyq"  "cza"  "cyy"  "this" "rhh"  "aprs" "wma"  "woc"  "gai"  "hch" 
##  [931] "hahe" "csa"  "hel"  "hcs"  "hak"  "ham"  "hhu"  "hco"  "hsi"  "halo"
##  [941] "hhh"  "hbe"  "hag"  "haf"  "halk" "hvn"  "hol"  "hsr"  "hmd"  "haxi"
##  [951] "htt"  "hcam" "hpiz" "haa"  "cmai" "kus"  "kma"  "paur" "abo"  "adi" 
##  [961] "apac" "aln"  "axe"  "kak"  "kge"  "mmw"  "mme"  "mpc"  "mpri" "mard"
##  [971] "tol"  "tor"  "oai"  "mars" "bsan" "ncu"  "nik"  "ajp"  "gsn"  "rfo" 
##  [981] "ome"  "aha"  "ahd"  "ahi"  "aaj"  "asa"  "aeo"  "avr"  "avo"  "amed"
##  [991] "asr"  "adh"  "aem"  "aea"  "arv"  "aes"  "ael"  "oce"  "opf"  "zdf" 
## [1001] "sok"  "sini" "gbi"  "saln" "pspi" "gpb"  "vff"  "cvi"  "cvc"  "chro"
## [1011] "chri" "chrb" "crz"  "chrm" "chae" "iod"  "ifl"  "lhk"  "pse"  "aql" 
## [1021] "amah" "aqs"  "cfon" "rso"  "rsc"  "rsl"  "rsn"  "rsm"  "rse"  "rsy" 
## [1031] "rpi"  "rpf"  "rpj"  "rmn"  "rin"  "rpu"  "reh"  "cnc"  "cuh"  "reu" 
## [1041] "rme"  "cti"  "cbw"  "cgd"  "ccup" "cup"  "cuu"  "cpau" "cox"  "bma" 
## [1051] "bmv"  "bml"  "bmn"  "bmal" "bmae" "bmaq" "bmai" "bmaf" "bmaz" "bmab"
## [1061] "bps"  "bpm"  "bpl"  "bpd"  "bpr"  "bpse" "bpsm" "bpsu" "bpsd" "bpz" 
## [1071] "bpq"  "bpk"  "bpsh" "bpsa" "bpso" "but"  "bte"  "btq"  "btj"  "btz" 
```

```
## [1081] "btd"  "btv"  "bthe" "bthm" "btha" "bthl" "bok"  "boc"  "buu"  "bvi"
## [1091] "bve"  "bur"  "bcn"  "bch"  "bcm"  "bcj"  "bcen" "bcew" "bceo" "bam"
## [1101] "bac"  "bmj"  "bmu"  "bmk"  "bmul" "bct"  "bced" "bcep" "bdl"  "bpyr"
## [1111] "bcon" "bub"  "bdf"  "blat" "btei" "bsem" "bpsl" "bmec" "bstg" "bstl"
## [1121] "bgl"  "bgu"  "bug"  "bgf"  "bgd"  "bgo"  "byi"  "buk"  "bue"  "bul"
## [1131] "buq"  "bgp"  "bpla" "bud"  "bum"  "bui"  "bxe"  "bxb"  "bph"  "bge"
## [1141] "bpx"  "bpy"  "buz"  "bfn"  "bcai" "pspw" "para" "parb" "phs"  "pter"
## [1151] "pgp"  "pcj"  "pts"  "pcaf" "pmeg" "brh"  "pnu"  "ppk"  "ppno" "ppnm"
## [1161] "prb"  "ppul" "pspu" "papi" "pve"  "pox"  "ptx"  "pfg"  "pnr"  "pand"
## [1171] "pfib" "plg"  "hyf"  "caba" "buo"  "limn" "cari" "bpe"  "bpc"  "bper"
## [1181] "bpet" "bpeu" "bpar" "bpa"  "bbh"  "bbr"  "bbm"  "bbx"  "bpt"  "bav"
## [1191] "bho"  "bhm"  "bhz"  "btrm" "bbro" "bfz"  "bpdz" "boh"  "bgm"  "boj"
## [1201] "boz"  "axy"  "axo"  "axn"  "axx"  "adt"  "ais"  "asw"  "achr" "achb"
## [1211] "put"  "pus"  "pud"  "aka"  "amim" "cdn"  "afa"  "afq"  "aaqu" "phn"
## [1221] "odi"  "pig"  "pacr" "kgy"  "rfr"  "rsb"  "rac"  "rhy"  "rhf"  "rhg"
## [1231] "pol"  "pna"  "pos"  "poo"  "aav"  "ajs"  "dia"  "aaa"  "ack"  "acra"
## [1241] "acid" "acip" "acin" "acis" "acio" "amon" "vei"  "dac"  "del"  "dts"
## [1251] "dhk"  "dla"  "vap"  "vpe"  "vpd"  "vaa"  "vbo"  "vam"  "ctt"  "ctes"
## [1261] "cke"  "cser" "cof"  "adn"  "adk"  "rta"  "otk"  "lim"  "lih"  "hyr"
## [1271] "hyb"  "hyl"  "hyc"  "hpse" "hyn"  "dpy"  "dih"  "daer" "drg"  "simp"
## [1281] "melm" "mela" "cbaa" "cbab" "sthm" "mpt"  "metp" "har"  "mms"  "jag"
## [1291] "jab"  "jaz"  "jal"  "jsv"  "jaj"  "jas"  "jlv"  "hse"  "hsz"  "hht"
## [1301] "hrb"  "hee"  "hhf"  "hfr"  "cfu"  "care" "cpra" "mnr"  "masw" "mass"
## [1311] "masz" "mtim" "masy" "mali" "mum"  "mfla" "mpli" "upv"  "upi"  "nok"
## [1321] "dug"  "lch"  "tin"  "thi"  "rge"  "rbn"  "rdp"  "pkt"  "miu"  "rgu"
## [1331] "aon"  "snn"  "xyk"  "pbh"  "shd"  "metr" "uru"  "upl"  "eba"  "dsu"
## [1341] "otr"  "rbh"  "dar"  "azo"  "aoa"  "aza"  "azi"  "atw"  "acom" "azd"
## [1351] "azr"  "azq"  "tmz"  "thu"  "tcl"  "thk"  "tak"  "zpa"  "bprc" "beb"
## [1361] "beba" "alk"  "arc"  "des"  "dat"  "dov"  "dwd"  "dalk" "afw"  "ccx"
## [1371] "mfu"  "mmas" "sur"  "age"  "mbd"  "cfus" "scl"  "scu"  "ccro" "samy"
## [1381] "llu"  "mrm"  "hoh"  "dti"  "pcay" "mlo"  "mln"  "mci"  "mop"  "mam"
## [1391] "mamo" "meso" "mesw" "mesm" "mesp" "mhua" "mjr"  "merd" "mes"  "hoe"
## [1401] "aak"  "amih" "pht"  "rpod" "niy"  "orm"  "pla"  "rbs"  "sme"  "smk"
## [1411] "smq"  "smx"  "smi"  "smeg" "smel" "smer" "smd"  "rhi"  "sfh"  "sfd"
```

```
## [1421] "six"  "same" "sino" "ead"  "eah"  "esj"  "eak"  "emx"  "atu"  "ara"
## [1431] "ata"  "agr"  "atf"  "avi"  "agc"  "aro"  "agt"  "alf"  "ret"  "rec"
## [1441] "rel"  "rep"  "rei"  "rle"  "rlt"  "rlg"  "rlb"  "rlu"  "rtr"  "rir"
## [1451] "rpus" "rhl"  "rga"  "rhn"  "rpha" "rhx"  "rhv"  "rhk"  "rez"  "rjg"
## [1461] "rhr"  "rgr"  "rad"  "roy"  "rii"  "ngl"  "ngg"  "neo"  "nen"  "rht"
## [1471] "shz"  "abaw" "bme"  "bmel" "bmi"  "bmz"  "bmg"  "bmw"  "bmee" "bmf"
## [1481] "bmb"  "bmc"  "baa"  "babo" "babr" "babt" "babb" "babu" "babs" "babc"
## [1491] "bms"  "bsi"  "bsf"  "bsui" "bsup" "bsuv" "bsuc" "bmt"  "bsz"  "bsv"
## [1501] "bsw"  "bsg"  "bov"  "bcs"  "bsk"  "bol"  "bcar" "bcas" "bmr"  "bpp"
## [1511] "bpv"  "bcet" "bcee" "bvl"  "bru"  "brj"  "oin"  "oan"  "oah"  "ops"
## [1521] "och"  "bja"  "bju"  "bjp"  "bra"  "bbt"  "brs"  "aol"  "brc"  "brad"
## [1531] "bic"  "bro"  "brk"  "bot"  "brq"  "bgq"  "bgz"  "bsym" "bbet" "barh"
## [1541] "bvz"  "rpa"  "rpb"  "rpc"  "rpd"  "rpe"  "rpt"  "rpx"  "nha"  "oca"
## [1551] "ocg"  "oco"  "bop"  "bos"  "bvv"  "boi"  "bof"  "vgo"  "trb"  "bapi"
## [1561] "xau"  "azc"  "sno"  "star" "lne"  "anc"  "apra" "mea"  "mdi"  "mex"
## [1571] "mch"  "mpo"  "mza"  "mrd"  "met"  "mno"  "mor"  "meta" "maqu" "mphy"
## [1581] "mee"  "metd" "metx" "mets" "meti" "mmes" "mtea" "moc"  "miv"  "mico"
## [1591] "bid"  "msl"  "mtun" "rhj"  "bbar" "chel" "cdq"  "hmc"  "hni"  "phl"
## [1601] "fil"  "fiy"  "deq"  "dei"  "dea"  "bvr"  "rhz"  "mmyr" "yti"  "metg"
## [1611] "ntd"  "msc"  "mbry" "mhey" "pleo" "mey"  "maad" "mmed" "aua"  "aala"
## [1621] "brn"  "psin" "hdi"  "noh"  "rbm"  "psf"  "pphr" "lap"  "lagg" "labr"
## [1631] "labp" "labt" "siw"  "ccr"  "ccs"  "cak"  "cse"  "chq"  "cmb"  "cfh"
## [1641] "cauf" "pzu"  "phb"  "bsb"  "brd"  "bne"  "brg"  "brl"  "bvc"  "bdm"
## [1651] "brf"  "brev" "bmed" "bvy"  "aex"  "tsv"  "cbot" "sil"  "sit"  "rua"
## [1661] "rut"  "rmb"  "rsq"  "rbl"  "jan"  "rde"  "rli"  "rpon" "pde"  "pami"
## [1671] "pye"  "pzh"  "paro" "pamn" "pars" "parr" "pkd"  "ppan" "dsh"  "pga"
## [1681] "pgl"  "pgd"  "php"  "ppic" "phq"  "oat"  "oar"  "otm"  "lmd"  "lej"
## [1691] "laqu" "red"  "ptp"  "cid"  "ceh"  "cmag" "malg" "con"  "rsu"  "rhm"
## [1701] "rhc"  "hat"  "daa"  "ypac" "yan"  "tpro" "suam" "spse" "sulz" "suli"
## [1711] "suld" "spot" "tom"  "paby" "thw"  "tec"  "rmm"  "rok"  "rid"  "rom"
## [1721] "roh"  "lvs"  "aht"  "rbg"  "sagu" "thaa" "geh"  "taw"  "salo" "sedi"
## [1731] "hml"  "pseb" "lit"  "ocd"  "maru" "rot"  "ppru" "paed" "mon"  "malu"
## [1741] "tgl"  "pamo" "pshq" "poz"  "palw" "ppaf" "pgv"  "rbz"  "thas" "faq"
## [1751] "hdh"  "mmr"  "gak"  "hne"  "hba"  "hbc"  "nar"  "npp"  "npn"  "nre"
```

```
## [1761] "nov"  "not"  "nor"  "ngf"  "nog"  "sal"  "sphk" "sphp" "smag" "smaz"
## [1771] "ster" "sgi"  "sphl" "sphq" "spho" "sphx" "sphu" "swi"  "sphd" "sphm"
## [1781] "stax" "sphi" "ssan" "snj"  "smy"  "span" "skr"  "splm" "splk" "spkc"
## [1791] "sphc" "sphf" "spha" "spau" "sech" "slut" "sjp"  "sch"  "ssy"  "syb"
## [1801] "sbd"  "spmi" "sphb" "sphr" "sinb" "spht" "shyd" "sya"  "sclo" "spyg"
## [1811] "sufl" "sami" "sbar" "cij"  "sphg" "sfla" "sphy" "blas" "bfw"  "rdi"
## [1821] "sphj" "spzr" "palg" "smic" "sphs" "sand" "aay"  "aep"  "alb"  "alh"
## [1831] "amx"  "anh"  "ado"  "cna"  "cman" "ery"  "egn"  "efv"  "eli"  "elq"
## [1841] "erk"  "err"  "erf"  "emv"  "pns"  "porl" "phz"  "pot"  "gbc"  "gbs"
## [1851] "acr"  "amv"  "gdi"  "rgi"  "ros"  "rmuc" "shum" "svc"  "rrf"  "magn"
## [1861] "azl"  "ali"  "abs"  "abq"  "abf"  "ati"  "azt"  "azm"  "azz"  "aoz"
## [1871] "tmo"  "txi"  "thac" "tii"  "magq" "nao"  "ncb"  "fer"  "htq"  "hadh"
## [1881] "skt"  "phr"  "pstg" "bdc"  "bdz"  "sbf"  "blh"  "bson" "bmyc" "bgy"
## [1891] "bhk"  "beo"  "bmq"  "bmd"  "bmh"  "bmeg" "bon"  "bko"  "gtk"  "lyb"
## [1901] "lyg"  "vig"  "vil"  "bmur" "pbut" "pof"  "bbe"  "bfm"  "pms"  "pmq"
## [1911] "pmw"  "pnp"  "palb" "prz"  "anx"  "asoc" "aac"  "aad"  "bts"  "kyr"
## [1921] "eff"  "ssil" "pmar" "play" "say"  "sap"  "mtu"  "mtv"  "mtc"  "mra"
## [1931] "mtf"  "mtb"  "mtk"  "mtz"  "mtg"  "mti"  "mte"  "mtur" "mtl"  "mto"
## [1941] "mtd"  "mtn"  "mtj"  "mtub" "mtue" "mtx"  "mtul" "mtut" "mtuu" "mtq"
## [1951] "mbo"  "mbb"  "mbt"  "mbm"  "mbk"  "maf"  "mmic" "mce"  "mcq"  "mcv"
## [1961] "mcx"  "mcz"  "mpa"  "mao"  "mavi" "mavu" "mav"  "mit"  "mia"  "mid"
## [1971] "myo"  "mchi" "mir"  "mmal" "msa"  "mul"  "mmc"  "mkm"  "mjl"  "mmi"
## [1981] "mmae" "mmm"  "mli"  "mkn"  "myv"  "mye"  "mhad" "mdx"  "mshg" "mfj"
## [1991] "mgro" "mxe"  "mnv"  "mpag" "mnm"  "mgor" "mcoo" "msm"  "msg"  "msb"
## [2001] "msn"  "msh"  "mva"  "mgi"  "msp"  "mcb"  "mne"  "myn"  "mgo"  "mft"
## [2011] "mphl" "mvq"  "mll"  "mrh"  "mthn" "mhas" "mdu"  "mcht" "mdr"  "mauu"
## [2021] "mmag" "mmor" "mfx"  "maic" "mij"  "malv" "mty"  "mpsc" "mab"  "mmv"
## [2031] "mabb" "mabl" "mche" "miz"  "mste" "msao" "msal" "mjd"  "mter" "mmin"
## [2041] "mhib" "asd"  "mkr"  "cgl"  "cgb"  "cgu"  "cgt"  "cgs"  "cgg"  "cgm"
## [2051] "cgj"  "cgq"  "cef"  "cva"  "chn"  "cmd"  "ccg"  "cgy"  "chm"  "cmq"
## [2061] "ccj"  "cmv"  "cted" "clw"  "cdx"  "csta" "ccjz" "camg" "cee"  "cpre"
## [2071] "csur" "nfa"  "nfr"  "ncy"  "nbr"  "nno"  "nsl"  "nsr"  "ntp"  "noz"
## [2081] "nod"  "nah"  "nad"  "nwl"  "rha"  "rer"  "rey"  "reb"  "rop"  "roa"
## [2091] "req"  "rpy"  "rhb"  "rav"  "rfa"  "rhw"  "rhs"  "rrz"  "rhu"  "rqi"
```

```
## [2101] "rhq"  "rhod" "rrt"  "rby"  "rcr"  "rtm"  "gbr"  "gpo"  "gor"  "goq"
## [2111] "gta"  "goc"  "git"  "gru"  "gom"  "gav"  "god"  "tpr"  "tsm"  "srt"
## [2121] "dtm"  "dit"  "diz"  "dpc"  "dlu"  "toy"  "sco"  "salb" "sgr"  "sgb"
## [2131] "scb"  "ssx"  "svl"  "sct"  "scy"  "sbh"  "shy"  "sho"  "sve"  "sdv"
## [2141] "sals" "sci"  "src"  "salu" "sall" "slv"  "sgu"  "svt"  "stre" "scw"
## [2151] "sld"  "slc"  "sxi"  "strm" "strc" "samb" "spri" "scz"  "scx"  "strf"
## [2161] "sle"  "spav" "strt" "sclf" "sgs"  "stsi" "sls"  "snr"  "splu" "strd"
## [2171] "sauo" "ssia" "spun" "sgv"  "smal" "slau" "salf" "salj" "slx"  "stro"
## [2181] "sfk"  "sge"  "srj"  "slk"  "sky"  "sdx"  "sgd"  "sqz"  "scya" "sast"
## [2191] "snq"  "svn"  "salw" "shaw" "srk"  "sgal" "svr"  "spad" "sfy"  "saqu"
## [2201] "sgf"  "scav" "sseo" "ksk"  "kau"  "kit"  "leif" "lse"  "mim"  "mio"
## [2211] "mip"  "mcw"  "mpal" "mih"  "micr" "maur" "mhos" "mfol" "moo"  "mlv"
## [2221] "mwa"  "mprt" "moy"  "rry"  "ria"  "rfs"  "rte"  "mvd"  "frp"  "agy"
## [2231] "agm"  "agf"  "cphy" "aum"  "malk" "myl"  "salc" "sala" "sald" "hum"
## [2241] "huw"  "lyd"  "plap" "ltr"  "ldn"  "agg"  "mant" "gln"  "agx"  "art"
## [2251] "arr"  "arm"  "arl"  "are"  "aaq"  "arw"  "arh"  "ary"  "arz"  "aru"
## [2261] "arq"  "arn"  "arx"  "acry" "arth" "artp" "ari"  "aau"  "pue"  "ach"
## [2271] "apn"  "psul" "psni" "psey" "aai"  "gar"  "gcr"  "glu"  "rsa"  "krh"
## [2281] "kpl"  "kfv"  "krs"  "kod"  "mlu"  "mick" "satk" "nae"  "bcv"  "brx"
## [2291] "bgg"  "brz"  "bsau" "lmoi" "xyl"  "ido"  "cet"  "cceu" "xya"  "ars"
## [2301] "jte"  "jli"  "jme"  "teh"  "pei"  "bly"  "blin" "bri"  "blut" "bcau"
## [2311] "aus"  "halt" "mph"  "micg" "ndk"  "noy"  "noi"  "noo"  "ndp"  "nsn"
## [2321] "nbe"  "nano" "nmes" "psim" "aez"  "aef"  "muz"  "kfl"  "kqi"  "tfu"
## [2331] "nal"  "ngv"  "strr" "tcu"  "actw" "sro"  "noa"  "now"  "tbi"  "fre"
## [2341] "fri"  "fal"  "nml"  "gob"  "bsd"  "mmar" "kra"  "sen"  "sace" "sacg"
## [2351] "amd"  "amn"  "amm"  "amz"  "aoi"  "aja"  "amq"  "amyc" "amyb" "aab"
## [2361] "amyy" "aori" "pdx"  "psea" "psee" "pseh" "pseq" "pecq" "phh"  "paut"
## [2371] "apre" "ami"  "sesp" "ssyi" "kal"  "kphy" "led"  "ahg"  "acta" "alo"
## [2381] "pmad" "saq"  "micb" "mtua" "mich" "mtem" "mcab" "msag" "mcra" "ams"
## [2391] "actn" "afs"  "pfla" "psuu" "ver"  "cai"  "sna"  "aey"  "eke"  "abai"
## [2401] "rxy"  "rub"  "bsol" "cwo"  "aym"  "atq"  "erz"  "euz"  "cau"  "chl"
## [2411] "cag"  "hau"  "tro"  "kbs"  "dmr"  "dpd"  "dein" "tra"  "aagg" "slr"
## [2421] "lil"  "tpx"  "acm"  "gma"  "grw"  "ctm"  "gau"  "gba"  "rmr"  "rmg"
## [2431] "cpi"  "cbae" "chit" "chih" "nko"  "fla"  "fgg"  "fln"  "pseg" "pgin"
```

```
## [2441] "pgo"  "lacs" "hhy"  "phe"  "pep"  "pcm"  "psty" "pgs"  "pek"  "proe"
## [2451] "shg"  "smiz" "spsc" "sphz" "sphe" "sdj"  "scn"  "mup"  "muc"  "mgot"
## [2461] "muh"  "mgin" "mgk"  "mrub" "mgos" "agd"  "oli"  "sbx"  "cmr"  "evi"
## [2471] "est"  "echi" "alm"  "dfe"  "srd"  "spir" "spik" "spib" "run"  "rup"
## [2481] "eol"  "fae"  "fib"  "als"  "hsw"  "hym"  "hye"  "hyg"  "hyp"  "hyz"
## [2491] "hnv"  "hyj"  "hqi"  "hrs"  "pko"  "pact" "ruf"  "rti"  "rud"  "nib"
## [2501] "add"  "mtt"  "fpf"  "flm"  "fll"  "chk"  "gfl"  "grs"  "fjo"  "fcm"
## [2511] "ffa"  "fki"  "fnk"  "zpr"  "marm" "mart" "mare" "cao"  "cbal" "cbat"
## [2521] "kdi"  "dok"  "ddo"  "mrs"  "mut"  "ptq"  "ndo"  "nob"  "pom"  "pob"
## [2531] "prn"  "pola" "poa"  "phal" "win"  "sze"  "ahz"  "syi"  "tje"  "tmp"
## [2541] "fop"  "salt" "seon" "aalg" "oll"  "taj"  "marf" "aqa"  "psyn" "afla"
## [2551] "anp"  "fbe"  "nio"  "halj" "srub" "htu"  "nvr"  "nax"
```

Organisms classified within cluster 4

```
##   [1] "hsa"  "ptr"  "pps"  "ggo"  "pon"  "nle"  "mcc"  "mcf"  "csab" "caty"
##  [11] "panu" "rro"  "rbb"  "tfn"  "pteh" "cjc"  "sbq"  "mmur" "mmu"  "mcal"
##  [21] "mpah" "rno"  "mcoc" "mun"  "cge"  "pleu" "ngi"  "hgl"  "ccan" "ocu"
##  [31] "opi"  "tup"  "cfa"  "vvp"  "vlg"  "aml"  "umr"  "uah"  "oro"  "elk"
##  [41] "mpuf" "eju"  "mlx"  "fca"  "pyu"  "pbg"  "ptg"  "ppad" "aju"  "hhv"
##  [51] "bta"  "bom"  "biu"  "bbub" "chx"  "oas"  "oda"  "ccad" "ssc"  "cfr"
##  [61] "cbai" "cdk"  "bacu" "lve"  "oor"  "dle"  "pcad" "ecb"  "epz"  "eai"
##  [71] "myb"  "myd"  "mmyo" "mna"  "pkl"  "hai"  "dro"  "shon" "ajm"  "pdic"
##  [81] "mmf"  "rfq"  "pale" "pgig" "ray"  "mjv"  "tod"  "lav"  "tmu"  "mdo"
##  [91] "gas"  "shr"  "pcw"  "oaa"  "gga"  "pcoc" "mgp"  "cjo"  "nmel" "apla"
## [101] "acyg" "tgu"  "lsr"  "scan" "pmoa" "otc"  "pruf" "gfr"  "fab"  "phi"
## [111] "pmaj" "ccae" "ccw"  "etl"  "fpg"  "fch"  "clv"  "egz"  "nni"  "acun"
## [121] "padl" "aam"  "arow" "npd"  "dne"  "asn"  "amj"  "cpoo" "ggn"  "pss"
## [131] "cmy"  "cpic" "tst"  "cabi" "acs"  "pvt"  "sund" "pbi"  "pmur" "tsr"
## [141] "pgut" "vko"  "pmua" "zvi"  "gja"  "xla"  "xtr"  "npr"  "dre"  "srx"
## [151] "sanh" "sgh"  "ccar" "caua" "ipu"  "phyp" "amex" "eee"  "tru"  "tng"
## [161] "lco"  "ncc"  "cgob" "ely"  "plep" "sluc" "ecra" "pflv" "gat"  "ppug"
## [171] "msam" "cud"  "mze"  "onl"  "oau"  "ola"  "oml"  "xma"  "xco"  "xhe"
```

```
## [181] "pret" "cvg"  "ctul" "nfu"  "kmr"  "alim" "aoce" "csem" "pov"  "ssen"
## [191] "lcf"  "sdu"  "slal" "xgl"  "hcq"  "bpec" "malb" "sasa" "otw"  "omy"
## [201] "salp" "snh"  "els"  "sfm"  "pki"  "aang" "loc"  "pspa" "arut" "lcm"
## [211] "cmk"  "rtp"  "bfo"  "bbel" "cin"  "sclv" "spu"  "aplc" "sko"  "dme"
## [221] "der"  "dse"  "dsi"  "dya"  "dan"  "dsr"  "dpo"  "dpe"  "dmn"  "dwi"
## [231] "dgr"  "dmo"  "daz"  "dnv"  "dhe"  "dvi"  "ccat" "bod"  "mde"  "scac"
## [241] "lcq"  "aga"  "acoz" "aara" "aag"  "aalb" "cqu"  "cpii" "ame"  "acer"
## [251] "bim"  "bbif" "bvk"  "bvan" "bter" "ccal" "obb"  "mgen" "nmea" "cgig"
## [261] "soc"  "mpha" "aec"  "acep" "pbar" "vem"  "hst"  "dqu"  "cfo"  "fex"
## [271] "lhu"  "pgc"  "obo"  "pcf"  "pfuc" "vps"  "nvi"  "csol" "tpre" "mdl"
## [281] "cglo" "fas"  "dam"  "ccin" "tca"  "dpa"  "atd"  "agb"  "ldc"  "nvl"
## [291] "apln" "ppyr" "otu"  "bmor" "bman" "msex" "dpl"  "bany" "pmac" "ppot"
## [301] "pxu"  "prap" "zce"  "haw"  "tnl"  "pxy"  "api"  "dnx"  "ags"  "rmd"
## [311] "btab" "dci"  "clec" "hhal" "nlu"  "phu"  "foc"  "zne"  "csec" "fcd"
## [321] "dpx"  "dmk"  "pvm"  "pja"  "hame" "hazt" "eaf"  "isc"  "dsv"  "rsan"
## [331] "rmp"  "vde"  "vja"  "tut"  "dpte" "cscu" "ptep" "sdm"  "cel"  "cbr"
## [341] "bmy"  "loa"  "nai"  "hro"  "lgi"  "pcan" "bgt"  "gae"  "crg"  "myi"
## [351] "pmax" "obi"  "osn"  "lak"  "smm"  "ovi"  "nve"  "epa"  "aten" "adf"
## [361] "amil" "pdam" "spis" "dgt"  "hmg"  "tad"  "aqu"
```

Organisms classified within cluster 5

```
##   [1] "icp"  "fsm"  "sgl"  "pes"  "hhs"  "pck"  "hip"  "hiq"  "hif"  "hil"
##  [11] "hie"  "hia"  "hic"  "hpr"  "hdu"  "hay"  "hpit" "hhz"  "haeg" "hpas"
##  [21] "hso"  "psky" "msu"  "bsun" "mht"  "mhq"  "mhat" "mhx"  "mhae" "mham"
##  [31] "mhao" "mhal" "mhaq" "mhay" "mann" "asu"  "apor" "adp"  "aat"  "aao"
##  [41] "aacn" "aseg" "apag" "avt"  "bhud" "xfa"  "xft"  "xfm"  "xfn"  "xff"
##  [51] "xfl"  "xfs"  "xfh"  "xtw"  "pade" "rvi"  "mmt"  "mpsy" "ftu"  "ftq"
##  [61] "ftf"  "ftw"  "ftr"  "ftt"  "ftg"  "ftl"  "fth"  "fta"  "fts"  "fti"
##  [71] "fto"  "ftc"  "ftv"  "ftz"  "ftm"  "ftn"  "ftx"  "ftd"  "fty"  "fcf"
##  [81] "fcn"  "fhi"  "fph"  "fpi"  "fpx"  "fna"  "fnl"  "frf"  "fper" "fha"
##  [91] "frm"  "foo"  "fgu"  "afri" "aii"  "tcx"  "htr"  "tcy"  "tao"  "thio"
## [101] "thig" "tse"  "tzo"  "ntt"  "ntg"  "hna"  "ghl"  "chj"  "gap"  "tho"
```

```
## [111] "rev"  "rma"  "reo"  "vok"  "ebh"  "nme"  "nmp"  "nmh"  "nmd"  "nmm"
## [121] "nms"  "nmq"  "nmz"  "nma"  "nmw"  "nmx"  "nmc"  "nmn"  "nmt"  "nmi"
## [131] "ngo"  "ngk"  "nla"  "nsi"  "nmj"  "nei"  "nek"  "nfv"  "nsf"  "ncz"
## [141] "nbc"  "kki"  "koa"  "eex"  "smur" "nba"  "pne"  "mcys" "teq"  "tea"
## [151] "teg"  "tas"  "tat"  "bpsi" "cbx"  "ofo"  "sutt" "sutk" "bbay" "nst"
## [161] "mfa"  "mmb"  "mbac" "mbat" "fam"  "sdr"  "sulf" "splb" "fpho" "fmy"
## [171] "kci"  "kct"  "hpj"  "hpg"  "hpb"  "hpu"  "hpv"  "heg"  "hpd"  "hpyk"
## [181] "hpyh" "hpyj" "hpyu" "hhe"  "hce"  "hcm"  "hcp"  "hcb"  "hhm"  "hty"
## [191] "hbl"  "had"  "het"  "hcl"  "hwi"  "wsu"  "tdn"  "sua"  "suln" "sulg"
## [201] "sulc" "spal" "sku"  "sulr" "cje"  "cjb"  "cjj"  "cju"  "cjn"  "cji"
## [211] "cjm"  "cjs"  "cjp"  "cjej" "cjeu" "cjen" "cjei" "cjer" "cjv"  "cjy"
## [221] "cjq"  "cjl"  "cjw"  "cjr"  "cjd"  "cjz"  "cjx"  "cff"  "cft"  "cfv"
## [231] "cfx"  "cfz"  "camp" "cfp"  "ccv"  "cha"  "cco"  "ccoc" "cla"  "clr"
## [241] "clm"  "clq"  "cln"  "cll"  "ccol" "ccc"  "ccq"  "ccf"  "ccy"  "ccoi"
## [251] "ccof" "ccoo" "caj"  "cis"  "cvo"  "cpel" "camr" "csm"  "csf"  "cgra"
## [261] "cure" "chyo" "cspf" "cpin" "ccun" "clx"  "cavi" "camz" "camy" "coj"
## [271] "crx"  "cgeo" "cbla" "ccor" "carm" "cmuc" "csho" "abu"  "abt"  "abl"
## [281] "ask"  "atp"  "acre" "alan" "apoc" "ahs"  "apai" "sdl"  "sba"  "hyo"
## [291] "nsa"  "sun"  "slh"  "nis"  "nam"  "nap"  "cmed" "cpaf" "gsu"  "gsk"
## [301] "glo"  "gbm"  "gem"  "geb"  "gao"  "gbn"  "pca"  "pace" "ppd"  "dvu"
## [311] "dvl"  "dvm"  "dvg"  "dde"  "dds"  "dma"  "dgg"  "dfi"  "def"  "dtr"
## [321] "dfl"  "dcb"  "dms"  "dsd"  "dsa"  "dhy"  "daf"  "das"  "dpi"  "dej"
## [331] "pprf" "psel" "ddn"  "dsx"  "dba"  "doa"  "drt"  "dps"  "dak"  "deo"
## [341] "sat"  "dao"  "dax"  "hmr"  "ric"  "rbt"  "ren"  "paca" "caq"  "naf"
## [351] "lcc"  "bhe"  "bhn"  "bhs"  "bqu"  "bqr"  "bbk"  "btr"  "btx"  "bgr"
## [361] "bcd"  "baus" "bvn"  "banc" "bart" "bara" "barw" "barr" "baro" "barj"
## [371] "bez"  "barn" "bky"  "bals" "thd"  "sdo"  "zmo"  "zmn"  "zmm"  "zmb"
## [381] "zmi"  "zmc"  "zmr"  "zmp"  "hgn"  "gal"  "gti"  "aasc" "abg"  "kba"
## [391] "nch"  "coq"  "comm" "ntn"  "neh"  "ssam" "swf"  "bob"  "bomb" "thal"
## [401] "efk"  "apc"  "apm"  "afr"  "afe"  "acu"  "acz"  "afi"  "afj"  "atx"
## [411] "mfn"  "bcir" "ocb"  "tap"  "aqt"  "stea" "sau"  "sav"  "saw"  "sah"
## [421] "saj"  "sam"  "sas"  "sar"  "sac"  "sax"  "saa"  "sao"  "sae"  "sad"
## [431] "suu"  "suv"  "suj"  "suk"  "suc"  "sut"  "suq"  "suz"  "sud"  "sux"
## [441] "suw"  "sug"  "saua" "saue" "saun" "saus" "sauu" "saug" "sauz" "saut"
```

```
## [451] "sauj" "sauk" "sauq" "sauv" "sauw" "saux" "sauy" "sauf" "suy"  "saub"
## [461] "saum" "sauc" "saur" "saui" "saud" "sams" "suh"  "ser"  "sep"  "sepp"
## [471] "seps" "shh"  "ssd"  "sdt"  "sdp"  "spas" "scap" "ssch" "sscz" "slz"
## [481] "sfq"  "schr" "ssh"  "sff"  "sste" "mlen" "mcl"  "mcak" "macr" "sbac"
## [491] "jea"  "lmow" "lmf"  "lmc"  "lmog" "lmp"  "lmol" "lmoj" "lmoz" "lmox"
## [501] "lmh"  "lmq"  "lml"  "lmw"  "lmz"  "lmon" "lmoo" "lmot" "lmoa" "lmok"
## [511] "lmv"  "lin"  "lwe"  "liv"  "lii"  "liw"  "lwi"  "lgz"  "eat"  "got"
## [521] "gmo"  "geq"  "gsa"  "gha"  "pyg"  "pswu" "lla"  "llk"  "llt"  "lls"
## [531] "lld"  "llx"  "llj"  "llm"  "llc"  "llr"  "lln"  "lli"  "llw"  "lgr"
## [541] "lgv"  "lpk"  "lrn"  "lack" "spy"  "spz"  "spym" "spya" "spm"  "spg"
## [551] "sps"  "sph"  "spi"  "spj"  "spk"  "spf"  "spa"  "spb"  "stg"  "stx"
## [561] "soz"  "stz"  "spyh" "spyo" "spn"  "spd"  "spr"  "spw"  "sjj"  "snv"
## [571] "spx"  "snt"  "snd"  "spnn" "sne"  "spv"  "snc"  "snm"  "spp"  "sni"
## [581] "spng" "snb"  "snp"  "snx"  "snu"  "spne" "spnu" "spnm" "spno" "sag"
## [591] "san"  "sak"  "sgc"  "sags" "sagl" "sagm" "sagi" "sagr" "sagp" "sagc"
## [601] "sagt" "sage" "sagg" "sagn" "smu"  "smc"  "smut" "smj"  "smua" "ste"
## [611] "stn"  "stu"  "stw"  "sthe" "ssa"  "ssb"  "ssu"  "ssv"  "ssi"  "sss"
## [621] "ssf"  "ssw"  "sup"  "ssus" "sst"  "ssuy" "ssk"  "ssq"  "sui"  "suo"
## [631] "srp"  "ssut" "ssui" "sgo"  "sez"  "seq"  "sezo" "sequ" "seu"  "sub"
## [641] "sds"  "sdg"  "sda"  "sdc"  "sdq"  "sga"  "sgg"  "sgt"  "sor"  "stk"
## [651] "stb"  "scp"  "scf"  "ssr"  "stf"  "stj"  "strs" "ssah" "smn"  "sif"
## [661] "sang" "sanc" "sans" "scg"  "scon" "scos" "soi"  "sik"  "siq"  "sio"
## [671] "siz"  "slu"  "sip"  "stv"  "spat" "strn" "ssob" "srq"  "seqi" "spei"
## [681] "srat" "splr" "strg" "lde"  "lhh"  "lgl"  "lca"  "lcz"  "lcs"  "lce"
## [691] "lcw"  "lcl"  "lpq"  "lpap" "lcb"  "lcx"  "lrh"  "lrg"  "lrl"  "lro"
## [701] "lrc"  "lpl"  "lpj"  "lpt"  "lps"  "lpr"  "lpz"  "lpb"  "lpx"  "lpg"
## [711] "lre"  "lrf"  "lru"  "lrt"  "lrr"  "lfe"  "lfr"  "lff"  "lmu"  "lor"
## [721] "lva"  "lfn"  "lpon" "lng"  "lhw"  "lmal" "lle"  "lkf"  "lbr"  "lbk"
## [731] "lko"  "lzy"  "lsua" "lsl"  "lsi"  "lsj"  "lrm"  "lagl" "laca" "lani"
## [741] "lbt"  "lcy"  "lho"  "lol"  "lnn"  "lku"  "lmae" "pce"  "paci" "lros"
## [751] "lgn"  "lhi"  "lct"  "lalw" "lali" "lfm"  "lzh"  "lft"  "lcv"  "lbm"
## [761] "lhb"  "ooe"  "oen"  "osi"  "lme"  "lmm"  "lmk"  "lci"  "lki"  "lec"
## [771] "lcn"  "lgs"  "lge"  "llf"  "lgc"  "lsu"  "lpse" "wcb"  "wjo"  "wpa"
## [781] "wso"  "whe"  "wei"  "wvr"  "efa"  "efl"  "efi"  "efd"  "efs"  "efn"
```

```
##  [791] "efq"  "ene"  "efc"  "efau" "efu"  "efm"  "eft"  "ehr"  "ecas" "emu"
##  [801] "edu"  "ega"  "eth"  "egv"  "eav"  "esg"  "thl"  "tey"  "too"  "tkr"
##  [811] "vpi"  "vac"  "vao"  "vcp"  "aur"  "aun"  "aui"  "asan" "acg"  "avs"
##  [821] "auh"  "adc"  "crn"  "cml"  "caw"  "cdj"  "jep"  "jda"  "jeh"  "jpo"
##  [831] "cac"  "cae"  "cay"  "cpe"  "cpf"  "cpr"  "ctc"  "ctet" "cno"  "cbo"
##  [841] "cba"  "cbh"  "cby"  "cbl"  "cbk"  "cbb"  "cbi"  "cbn"  "cbt"  "cbf"
##  [851] "cbm"  "cbj"  "cbe"  "cbz"  "cbei" "clj"  "ccb"  "clb"  "cbv"  "cld"
##  [861] "cace" "cbut" "ctyk" "ceu"  "ctae" "cchv" "carg" "cia"  "csep" "ccoh"
##  [871] "cfer" "amt"  "aoe"  "hhw"  "cale" "crs"  "cazo" "sarj" "cth"  "ctx"
##  [881] "ccl"  "hsc"  "ruk"  "rbp"  "cce"  "css"  "csd"  "cthd" "esr"  "esu"
##  [891] "ccel" "eha"  "ral"  "rch"  "rum"  "rus"  "ruj"  "fpr"  "fpa"  "fpra"
##  [901] "ova"  "obj"  "bpb"  "bhu"  "cle"  "cew"  "rho"  "rix"  "rim"  "coo"
##  [911] "byl"  "bhan" "blau" "blab" "cpy"  "lacy" "csci" "cso"  "arf"  "acac"
##  [921] "hsd"  "cpro" "lua"  "pxv"  "acel" "eel"  "rto"  "rgn"  "cbol" "ere"
##  [931] "ert"  "era"  "lbw"  "cdf"  "cdc"  "cdl"  "eac"  "cst"  "faa"  "psor"
##  [941] "roc"  "phx"  "swo"  "slp"  "salq" "dsy"  "drm"  "dca"  "dru"  "dfg"
##  [951] "dae"  "dku"  "pth"  "dau"  "tjr"  "sgy"  "ded"  "dec"  "drs"  "tfr"
##  [961] "hmo"  "elm"  "emt"  "elim" "awo"  "cthm" "cmiu" "mdv"  "amij" "amic"
##  [971] "euu"  "bprs" "cbar" "tte"  "tex"  "thx"  "tpd"  "tit"  "tmt"  "tbo"
##  [981] "twi"  "tki"  "adg"  "tpz"  "csc"  "ate"  "cob"  "chd"  "cow"  "cki"
##  [991] "ckn"  "clc"  "ccha" "txy"  "tsh"  "tnr"  "taci" "mas"  "tep"  "tae"
## [1001] "toc"  "nth"  "hor"  "has"  "hpk"  "hals" "hhl"  "aft"  "apr"  "ped"
## [1011] "phar" "piv"  "cad"  "spoa" "kpar" "vat"  "med"  "dpn"  "dho"  "ssg"
## [1021] "sri"  "sele" "selo" "selt" "mhg"  "mfun" "mana" "erh"  "ers"  "erl"
## [1031] "erd"  "eio"  "euc"  "fro"  "aarg" "absi" "ciu"  "erm"  "fit"  "ebm"
## [1041] "erb"  "tsg"  "acl"  "aoc"  "mbj"  "tbm"  "tbz"  "cdi"  "cdp"  "cdt"
## [1051] "cde"  "cda"  "cdz"  "cdb"  "cdw"  "cdv"  "cpl"  "cpg"  "cpp"  "cpk"
## [1061] "cpq"  "cpx"  "cpz"  "cor"  "cop"  "cod"  "cos"  "coi"  "coe"  "cou"
## [1071] "cpse" "cpsu" "cpsf" "cul"  "cuc"  "cue"  "cun"  "cus"  "cuq"  "cuz"
## [1081] "cuj"  "cax"  "cku"  "cut"  "crf"  "crl"  "ccho" "cpso" "cbq"  "twh"
## [1091] "tws"  "cqf"  "psai" "rmu"  "rdn"  "raj"  "rter" "cig"  "dva"  "djj"
## [1101] "pav"  "pax"  "paz"  "pad"  "pacc" "pach" "pacn" "cacn" "pra"  "cgrn"
## [1111] "pfr"  "prl"  "ppc"  "pbo"  "aaci" "acij" "ahe"  "arca" "mcu"  "tpy"
## [1121] "tpyo" "asg"  "actt" "amy"  "soo"  "acq"  "aos"  "ard"  "actp" "actc"
```

```
## [1131] "acto" "ane"  "ahw"  "actz" "air"  "asla" "avc"  "avu"  "wik"  "fvg"
## [1141] "blo"  "blj"  "bln"  "blon" "blf"  "bll"  "blb"  "blm"  "blk"  "blg"
## [1151] "blz"  "blx"  "bad"  "badl" "bado" "bla"  "blc"  "blt"  "bbb"  "bbc"
## [1161] "bnm"  "blv"  "blw"  "bls"  "bani" "banl" "bni"  "banm" "bde"  "bdn"
## [1171] "bbp"  "bbi"  "bbf"  "bbv"  "bbru" "bbre" "bbrv" "bbrj" "bbrc" "bbrn"
## [1181] "bbrs" "bbrd" "bast" "btp"  "bcor" "bka"  "bks"  "bcat" "bpsp" "bii"
## [1191] "bang" "bpsc" "bsca" "bact" "bcho" "bgx"  "blem" "beu"  "gvg"  "gva"
## [1201] "gvh"  "sij"  "pdo"  "plak" "abam" "nhi"  "nab"  "ccu"  "shi"  "ele"
## [1211] "eyy"  "gpa"  "aeq"  "ddt"  "cbac" "apv"  "ols"  "olo"  "pcat" "cgo"
## [1221] "caer" "syc"  "syf"  "syw"  "syd"  "sye"  "cya"  "syne" "syh"  "synw"
## [1231] "slw"  "tel"  "thn"  "tvn"  "thec" "pma"  "pmm"  "pmn"  "pmi"  "pmb"
## [1241] "pmc"  "pmh"  "pmj"  "pme"  "prc"  "prm"  "cyu"  "naz"  "mbf"  "det"
## [1251] "deh"  "deb"  "dev"  "deg"  "dmc"  "dmd"  "dmg"  "dmx"  "dmy"  "dmz"
## [1261] "duc"  "dly"  "dew"  "dfo"  "atm"  "abat" "psub" "abao" "ttr"  "tpar"
## [1271] "pnl"  "puv"  "sng"  "ote"  "caa"  "amu"  "agl"  "xii"  "min"  "mkc"
## [1281] "meap" "vbs"  "pir"  "ttf"  "plm"  "peh"  "kst"  "broc" "phm"  "pcor"
## [1291] "pbu"  "pbp"  "pbas" "alus" "vbl"  "vbc"  "vai"  "tde"  "tsu"  "tbe"
## [1301] "taz"  "tped" "scd"  "tpk"  "tphg" "trc"  "sta"  "stq"  "sfc"  "sper"
## [1311] "sbu"  "scc"  "ock"  "bhy"  "bhd"  "brm"  "bpo"  "bpj"  "bpip" "bpw"
## [1321] "bip"  "bhp"  "thyd" "emi"  "epo"  "fnu"  "fnc"  "fnt"  "fus"  "fne"
## [1331] "fhw"  "fpd"  "ful"  "fmo"  "fgo"  "fnf"  "fpei" "lba"  "leo"  "lot"
## [1341] "leq"  "lhf"  "lsz"  "lhg"  "lte"  "lwd"  "lgo"  "str"  "tai"  "aco"
## [1351] "tli"  "amo"  "fsu"  "fsc"  "bth"  "btho" "bfr"  "bfs"  "bfg"  "bfb"
## [1361] "bhl"  "bxy"  "boa"  "bcel" "bcac" "bcae" "bzg"  "bhf"  "bis"  "bun"
## [1371] "bvu"  "bsa"  "bdo"  "bdh"  "pgi"  "pgn"  "pgt"  "pah"  "pcre" "pcag"
## [1381] "pbt"  "pmuc" "psac" "dys"  "ppn"  "pdi"  "parc" "tfo"  "toh"  "pary"
## [1391] "dun"  "bvs"  "copr" "osp"  "buy"  "aps"  "pru"  "pmz"  "pdn"  "pit"
## [1401] "pdt"  "pro"  "pfus" "peo"  "pje"  "poc"  "alq"  "afd"  "ash"  "ald"
## [1411] "aok"  "acou" "ada"  "ait"  "rbc"  "ttz"  "blq"  "bacc" "asx"  "arb"
## [1421] "coc"  "ccm"  "col"  "chg"  "capn" "cgh"  "clk"  "cspu" "ccyn" "caph"
## [1431] "csto" "capq" "capf" "orh"  "ori"  "bcad" "apib" "bbl"  "bbg"  "blp"
## [1441] "blu"  "ise"  "cte"  "cpc"  "clz"  "cch"  "cph"  "cpb"  "cli"  "pvi"
## [1451] "plt"  "pph"  "paa"  "proc" "prs"  "pros" "cts"  "mro"  "caci" "aae"
## [1461] "hya"  "hho"  "hys"  "hth"  "hte"  "tal"  "trd"  "sul"  "saf"  "pmx"
```

```
## [1471] "tam"  "dte"  "tma"  "tmm"  "tmi"  "tmw"  "tmq"  "tmx"  "tpt"  "trq"
## [1481] "tna"  "tnp"  "thq"  "thz"  "thr"  "tle"  "tta"  "phy"  "tme"  "taf"
## [1491] "thp"  "ther" "fno"  "fpe"  "fia"  "ocy"  "pmo"  "mpz"  "marn" "dtn"
## [1501] "kol"  "kpf"  "mpg"  "minf" "asac" "cpo"  "din"  "dap"  "cni"  "fsi"
## [1511] "gtl"  "dth"  "dtu"  "tye"  "lfc"  "lfi"  "lfp"  "leg"  "tid"  "top"
## [1521] "tcm"  "thet" "cthi" "tav"  "tmai" "prf"  "bana" "bih"  "mja"  "mfe"
## [1531] "mvu"  "mfs"  "mif"  "mjh"  "mig"  "mmp"  "mmq"  "mmx"  "mmz"  "mmd"
## [1541] "mmak" "mmao" "mmad" "mae"  "mvn"  "mvo"  "mok"  "metf" "mth"  "mmg"
## [1551] "metc" "mwo"  "mete" "metz" "metk" "mthm" "mst"  "metb" "mru"  "msi"
## [1561] "meb"  "mmil" "meye" "mol"  "mel"  "mew"  "meth" "mfc"  "mfi"  "mcub"
## [1571] "msub" "metn" "mett" "meto" "mfv"  "mka"  "apo"  "ave"  "ast"  "fpl"
## [1581] "gah"  "pfu"  "pfi"  "pho"  "pab"  "pyn"  "pya"  "pys"  "pyc"  "tko"
## [1591] "ton"  "tga"  "tsi"  "tba"  "the"  "tha"  "thm"  "tlt"  "ths"  "tnu"
## [1601] "teu"  "tgy"  "thv"  "tch"  "tpep" "tpie" "tgg"  "tce"  "tbs"  "thh"
## [1611] "tsl"  "ttd"  "tprf" "trl"  "tpaf" "thy"  "ppac" "mba"  "mby"  "mbw"
## [1621] "mbar" "mbak" "mvc"  "mek"  "mls"  "mef"  "meq"  "msj"  "mthr" "mthe"
## [1631] "mbu"  "mmet" "mmh"  "mhaz" "mev"  "mzh"  "mpy"  "mzi"  "mhz"  "mtp"
## [1641] "mcj"  "mhi"  "mhu"  "mla"  "mbg"  "mema" "mpi"  "mbn"  "mfo"  "mpl"
## [1651] "mez"  "rci"  "tvo"  "tar"  "max"  "mer"  "mear" "marc" "abi"  "acf"
## [1661] "smr"  "shc"  "iho"  "iis"  "dka"  "dmu"  "tag"  "iag"  "thg"  "hbu"
## [1671] "pfm"  "pdl"  "aho"  "aamb" "sazo" "step" "pai"  "tne"  "pyw"  "cma"
## [1681] "asc"  "acia" "ffo"  "nmr"  "nkr"  "nid"  "ncl"  "nct"  "nic"  "csy"
## [1691] "nga"  "nev"  "taa"  "ncv"  "nbv"  "tah"  "ndv"  "kcr"  "barc" "barb"
## [1701] "miy"  "arg"
```

Organisms classified within cluster 6

```
##    [1] "kgo"  "lbq"  "lpop" "tpty" "pmr"  "pmib" "pvl"  "pvg"  "phau" "prot"
##   [11] "pcol" "pcib" "xbo"  "xne"  "xpo"  "prj"  "mmk"  "ans"  "eic"  "etr"
##   [21] "etd"  "ete"  "etc"  "edw"  "edl"  "eho"  "opo"  "lpv"  "pfq"  "prag"
##   [31] "hin"  "hit"  "hiu"  "hiz"  "hik"  "hih"  "hiw"  "hix"  "hpaa" "hap"
##   [41] "hpaz" "hpak" "gle"  "hsm"  "pmu"  "pmv"  "pul"  "pmp"  "pmul" "pdag"
##   [51] "mvr"  "mvi"  "mvg"  "mve"  "mgra" "apl"  "apj"  "apa"  "asi"  "ass"
```

```
##  [61] "aeu"  "aio"  "alig" "aap"  "aaz"  "aan"  "aah"  "aact" "gan"  "bto"
##  [71] "btre" "btrh" "btra" "rpne" "rhey" "ooi"  "fcl"  "paet" "lyt"  "lue"
##  [81] "lum"  "theh" "tcn"  "fau"  "dja"  "dye"  "vcx"  "lag"  "vsc"  "vaq"
##  [91] "vfi"  "vfm"  "vsa"  "awd"  "pmai" "emo"  "prw"  "abm"  "aid"  "mct"
## [101] "mcs"  "mcat" "moi"  "mbl"  "mboi" "mcun" "mnn"  "msx"  "psy"  "ceb"
## [111] "cbu"  "cbs"  "cbd"  "cbg"  "cbc"  "alg"  "asip" "lpn"  "lpo"  "lpf"
## [121] "lpp"  "lpa"  "lha"  "lwa"  "mca"  "metu" "mdn"  "mdh"  "mko"  "metl"
## [131] "mah"  "mbur" "mmai" "mmob" "mein" "fpt"  "fpm"  "fpz"  "fpj"  "frt"
## [141] "frx"  "frc"  "fad"  "fmi"  "hmar" "mej"  "mec"  "psal" "tig"  "blep"
## [151] "noc"  "nhl"  "nwa"  "nwr"  "alv"  "tvi"  "tmb"  "mpur" "tee"  "tsy"
## [161] "ttp"  "thip" "aeh"  "hha"  "hhk"  "hhc"  "ebs"  "tgr"  "tkm"  "tni"
## [171] "tti"  "tvr"  "ssal" "spiu" "sros" "spiz" "haz"  "ttc"  "zpl"  "kuy"
## [181] "kko"  "ksd"  "kpd"  "bmar" "llp"  "ahy"  "ahr"  "ahp"  "ahj"  "ahh"
## [191] "acav" "tau"  "ocm"  "fpp"  "orb"  "sdf"  "slim" "sva"  "acii" "tbn"
## [201] "seds" "tsn"  "thin" "enm"  "nel"  "nwe"  "nzl"  "naq"  "nbl"  "nzo"
## [211] "nci"  "nani" "salv" "vit"  "ecor" "aff"  "cste" "jeu"  "dee"  "chiz"
## [221] "pdq"  "poh"  "lmir" "our"  "oto"  "bbag" "neu"  "net"  "nit"  "nii"
## [231] "nco"  "nur"  "nmu"  "nlc"  "doe"  "tbd"  "meh"  "mei"  "mep"  "meu"
## [241] "slt"  "gca"  "nim"  "sniv" "slac" "rbu"  "dey"  "app"  "hpy"  "heo"
## [251] "hpa"  "hps"  "hhp"  "hhq"  "hhr"  "hpp"  "hpl"  "hpc"  "hca"  "hpm"
## [261] "hpe"  "hpo"  "hpi"  "hpq"  "hpw"  "hef"  "hpf"  "heq"  "hex"  "hpt"
## [271] "hpz"  "hpx"  "hen"  "hph"  "hpn"  "hep"  "heu"  "hes"  "hpys" "hcn"
## [281] "hey"  "her"  "hei"  "hpya" "hpyo" "hpyl" "hpyb" "hpyc" "hpyd" "hpye"
## [291] "hpyf" "hpyg" "hpyr" "hpyi" "hpym" "hem"  "heb"  "hez"  "hac"  "hms"
## [301] "hfe"  "hbi"  "chv"  "chw"  "cux"  "acib" "afc"  "ant"  "aell" "aaqi"
## [311] "asui" "aclo" "aana" "avp"  "adz"  "alp"  "amyt" "amar" "acaa" "amol"
## [321] "hbv"  "hebr" "paco" "smul" "shal" "suls" "sulj" "sult" "gme"  "gur"
## [331] "geo"  "gpi"  "gsb"  "pef"  "deu"  "dpg"  "dpr"  "dog"  "dsf"  "dol"
## [341] "dml"  "dal"  "dto"  "ade"  "acp"  "ank"  "mxa"  "msd"  "mym"  "mfb"
## [351] "vin"  "sfu"  "dbr"  "dav"  "bsed" "pmob" "kai"  "nwi"  "mlg"  "hdn"
## [361] "hdt"  "rva"  "blag" "mcg"  "mros" "mpar" "mtw"  "rsp"  "rsh"  "rsk"
## [371] "rcp"  "rhp"  "pcon" "paru" "pmut" "kvl"  "kvu"  "kro"  "oct"  "cmar"
## [381] "don"  "boo"  "hyt"  "srhi" "gox"  "goh"  "goy"  "gbe"  "gbh"  "gdj"
## [391] "gxy"  "gxl"  "kna"  "keu"  "ksc"  "kre"  "kha"  "apt"  "apw"  "apf"
```

```
## [401] "apu"  "apg"  "apq"  "apx"  "apz"  "apk"  "asz"  "asv"  "aace" "aper"
## [411] "apom" "ato"  "acet" "aot"  "aoy"  "rru"  "rce"  "rpm"  "mag"  "mgy"
## [421] "mgry" "magx" "ahu"  "hjo"  "dex"  "dvn"  "pbr"  "mgm"  "pub"  "pel"
## [431] "peg"  "ecog" "mai"  "man"  "apb"  "bba"  "bbat" "bbw"  "bbac" "bex"
## [441] "bdq"  "bmx"  "hax"  "bsto" "maes" "htl"  "bsu"  "bsr"  "bsl"  "bsh"
## [451] "bsy"  "bsut" "bsul" "bsus" "bso"  "bsn"  "bsq"  "bsx"  "bsp"  "bss"
## [461] "bst"  "bli"  "bld"  "bay"  "baq"  "bya"  "bamp" "baml" "bama" "bamn"
## [471] "bamb" "bamt" "bamy" "bmp"  "bao"  "baz"  "bql"  "bxh"  "bqy"  "bami"
## [481] "bamc" "bamf" "bsia" "bae"  "bvm"  "bht"  "ban"  "bar"  "bat"  "bah"
## [491] "bai"  "bax"  "bant" "banr" "bans" "banh" "banv" "bce"  "bca"  "bcz"
## [501] "bcr"  "bcb"  "bcu"  "bcg"  "bcq"  "bcx"  "bal"  "bnc"  "bcf"  "bcer"
## [511] "bcef" "bcy"  "btk"  "btl"  "btb"  "btt"  "bthr" "bthi" "btc"  "btf"
## [521] "btm"  "btg"  "bti"  "btn"  "btht" "bthu" "btw"  "bthy" "bwe"  "bww"
## [531] "bmyo" "bty"  "bby"  "bwd"  "btro" "bmob" "bpu"  "bpum" "bpus" "bco"
## [541] "bjs"  "baci" "bif"  "bmet" "gst"  "bacw" "bacp" "bacb" "baco" "bacy"
## [551] "bacl" "balm" "bsm"  "bwh"  "bxi"  "bbev" "balt" "bacs" "bsaf" "bit"
## [561] "bacq" "bfd"  "bcoh" "bda"  "bfx"  "bck"  "bag"  "bcoa" "bha"  "bcl"
## [571] "bpf"  "ble"  "bkw"  "bgi"  "oih"  "ocn"  "gka"  "gte"  "gtm"  "gli"
## [581] "gtn"  "gwc"  "gyc"  "gya"  "gct"  "gmc"  "ggh"  "gjf"  "gea"  "gel"
## [591] "gse"  "gsr"  "gej"  "gth"  "ptl"  "ptb"  "afl"  "agn"  "anm"  "aamy"
## [601] "anl"  "and"  "acai" "axl"  "lsp"  "lgy"  "lfu"  "lys"  "lyz"  "lpak"
## [611] "hhd"  "hmn"  "hli"  "vir"  "vhl"  "vne"  "vpn"  "vim"  "lao"  "fpn"
## [621] "far"  "sje"  "apak" "bsj"  "pasa" "bthv" "psyh" "psyo" "prd"  "grc"
## [631] "rue"  "sale" "nmk"  "ntm"  "meku" "aia"  "blen" "bse"  "sue"  "suf"
## [641] "sab"  "sha"  "ssp"  "sca"  "slg"  "sln"  "swa"  "sxy"  "sxl"  "sxo"
## [651] "shu"  "sagq" "seqo" "ssif" "scv"  "spet" "scoh" "snl"  "skl"  "shom"
## [661] "smus" "scar" "sarl" "spic" "ssim" "sscu" "shv"  "lmo"  "lmn"  "lmy"
## [671] "lmt"  "lmoc" "lmoe" "lmob" "lmod" "lmoq" "lmr"  "lmom" "lmg"  "lms"
## [681] "lmj"  "lmx"  "lmos" "lmoy" "lsg"  "lia"  "lio"  "bths" "esi"  "ean"
## [691] "exm"  "exu"  "blr"  "bagr" "brw"  "pjd"  "gym"  "ppy"  "ppm"  "ppo"
## [701] "ppol" "ppq"  "ppoy" "pta"  "plv"  "psab" "pdu"  "pbd"  "pgm"  "pod"
## [711] "paen" "paef" "paeq" "pste" "paea" "paee" "paeh" "paej" "pbj"  "pih"
## [721] "pri"  "ppeo" "pow"  "pbv"  "pxl"  "pdh"  "pib"  "pcx"  "pkb"  "paih"
## [731] "pvo"  "plw"  "plen" "ppsc" "plut" "pchi" "pbk"  "pprt" "pbac" "plyc"
```

```
##  [741] "tco"  "coh"  "cohn" "saca" "tum"  "tab"  "siv"  "sob"  "pln"  "pku"
##  [751] "prt"  "pll"  "pana" "pdg"  "phc"  "ppla" "pfae" "plx"  "pmat" "pdec"
##  [761] "jeo"  "kur"  "kzo"  "spsy" "spor" "spop" "sure" "spos" "spae" "rst"
##  [771] "paek" "panc" "pgq"  "vij"  "ntr"  "lfb"  "tvu"  "kpul" "keb"  "lact"
##  [781] "lpi"  "lra"  "lbh"  "lbn"  "lpar" "lcu"  "lhil" "lpd"  "ess"  "vte"
##  [791] "abae" "carc" "carn" "marr" "jar"  "ckl"  "ckr"  "cls"  "csr"  "cpas"
##  [801] "cpat" "cpae" "csb"  "cah"  "clt"  "csq"  "cck"  "cfm"  "cdrk" "cdy"
##  [811] "gfe"  "fpla" "capr" "bfi"  "cct"  "rob"  "bpro" "csh"  "bprl" "ehl"
##  [821] "anr"  "pdc"  "pdf"  "sth"  "dhd"  "ddh"  "ddl"  "dmt"  "dgi"  "dor"
##  [831] "dai"  "dmi"  "hcv"  "tmr"  "thef" "thep" "sthr" "ibu"  "abut" "chy"
##  [841] "mta"  "mtho" "mthz" "ttm"  "tto"  "aar"  "vpr"  "vrm"  "vdn"  "vnk"
##  [851] "mhw"  "meg"  "puf"  "pft"  "sted" "afn"  "ain"  "pfac" "lpil" "mtuc"
##  [861] "mtuh" "mbx"  "mle"  "mlb"  "mlp"  "cgx"  "cdh"  "cdr"  "cds"  "cdd"
##  [871] "cdip" "cjk"  "cur"  "cua"  "car"  "ckp"  "crd"  "ccn"  "cter" "caz"
##  [881] "cfn"  "cvt"  "cii"  "cuv"  "coa"  "cdo"  "csx"  "cei"  "csp"  "cfk"
##  [891] "cpho" "cfc"  "cgv"  "cstr" "caqu" "csph" "cmin" "cpeg" "cxe"  "csan"
##  [901] "cgk"  "bfv"  "sma"  "sfa"  "strp" "sfi"  "srw"  "srn"  "snw"  "svu"
##  [911] "snz"  "stir" "ska"  "sgz"  "snk"  "sfic" "sspo" "kab"  "stri" "lxl"
##  [921] "lxx"  "lxy"  "cmi"  "cms"  "cmc"  "cmh"  "ccap" "mts"  "mix"  "msed"
##  [931] "rla"  "rpla" "aqg"  "rtx"  "rtc"  "rtn"  "cum"  "cub"  "cug"  "cart"
##  [941] "cry"  "amin" "auw"  "gry"  "lyk"  "leu"  "leb"  "hea"  "frn"  "chre"
##  [951] "acit" "kii"  "kvr"  "rama" "rkr"  "aul"  "bfa"  "brv"  "brr"  "jde"
##  [961] "kse"  "dni"  "day"  "xce"  "iva"  "ske"  "sanw" "cfl"  "cfi"  "cga"
##  [971] "cez"  "celz" "cej"  "celh" "oek"  "psei" "ica"  "phw"  "serj" "serw"
##  [981] "orn"  "orz"  "dco"  "gez"  "pac"  "pak"  "paw"  "pcn"  "pfre" "pacd"
##  [991] "paus" "aji"  "mik"  "tfl"  "tfa"  "tes"  "tez"  "tdf"  "tla"  "rain"
## [1001] "prv"  "nca"  "aer"  "aeb"  "mgg"  "nda"  "fra"  "fsy"  "ace"  "nak"
## [1011] "svi"  "sacc" "ahm"  "acti" "acad" "stp"  "mau"  "mil"  "vma"  "ase"
## [1021] "acts" "plk"  "plab" "plat" "tbw"  "fsl"  "flh"  "plan" "plim" "psuf"
## [1031] "pvs"  "pvn"  "rrd"  "afo"  "syn"  "syz"  "syy"  "syt"  "sys"  "syq"
## [1041] "syj"  "syo"  "syg"  "syr"  "syx"  "syp"  "cyb"  "synp" "synk" "synr"
## [1051] "synd" "syu"  "syv"  "syl"  "sync" "cgc"  "cyi"  "dsl"  "cmp"  "lep"
## [1061] "len"  "let"  "lbo"  "hhg"  "pseu" "pser" "pmt"  "pmf"  "pmg"  "amr"
## [1071] "theu" "glp"  "gen"  "gee"  "chon" "mar"  "mpk"  "miq"  "mvz"  "can"
```

```
## [1081] "csn"  "cyl"  "hao"  "enn"  "cyt"  "cwa"  "cyp"  "cyh"  "cyc"  "cyj"
## [1091] "cyn"  "ter"  "mic"  "arp"  "pagh" "oxy"  "lfs"  "gei"  "oac"  "oni"
## [1101] "mpro" "cep"  "gvi"  "glj"  "ana"  "npu"  "nos"  "nop"  "non"  "nfl"
## [1111] "noe"  "nsh"  "ned"  "ava"  "anb"  "acy"  "awa"  "ann"  "csg"  "calo"
## [1121] "calt" "calh" "riv"  "fis"  "dou"  "dfs"  "ccur" "toq"  "ncn"  "cthe"
## [1131] "plp"  "scs"  "stan" "ceo"  "cer"  "rrs"  "rca"  "sti"  "cap"  "pbf"
## [1141] "tbh"  "dra"  "dge"  "ddr"  "dpt"  "dgo"  "dsw"  "dch"  "dab"  "dpu"
## [1151] "dez"  "dwu"  "dfc"  "dga"  "tth"  "ttj"  "tts"  "ttl"  "tsc"  "thc"
## [1161] "tos"  "taq"  "tbc"  "mrb"  "mre"  "msv"  "mtai" "opr"  "mhd"  "ccz"
## [1171] "fgi"  "wch"  "obg"  "vbh"  "obt"  "roo"  "luo"  "vba"  "rba"  "psl"
## [1181] "rul"  "mff"  "rol"  "ahel" "lcre" "bvo"  "lpav" "amuc" "pnd"  "pbs"
## [1191] "pls"  "plh"  "fmr"  "gmr"  "gim"  "mri"  "sdyn" "plon" "ges"  "gog"
## [1201] "gms"  "tim"  "lrs"  "ftj"  "uli"  "ipa"  "saci" "pbor" "agv"  "tpi"
## [1211] "ssm"  "sgp"  "lie"  "lic"  "lis"  "lbj"  "lbl"  "lbi"  "lbf"  "lst"
## [1221] "laj"  "lmay" "lkm"  "lwl"  "aba"  "aca"  "tsa"  "trs"  "talb" "abas"
## [1231] "eda"  "sus"  "pfer" "abac" "fva"  "ipo"  "cpor" "gph"  "pet"  "dori"
## [1241] "drc"  "mbas" "sru"  "srm"  "rbar" "nso"  "nia"  "ark"  "agi"  "arac"
## [1251] "fls"  "sgn"  "pej"  "psn"  "sht"  "sphn" "spdr" "stha" "camu" "bbd"
## [1261] "chu"  "sli"  "smon" "lby"  "rsi"  "psez" "rhoz" "fli"  "hyd"  "hyh"
## [1271] "aswu" "fbt"  "gfo"  "grl"  "fps"  "fpc"  "fpy"  "fpo"  "fpq"  "fpv"
## [1281] "fpw"  "fpk"  "fpsz" "fjg"  "fbr"  "fco"  "fin"  "fgl"  "fat"  "fpal"
## [1291] "fmg"  "falb" "fcr"  "fse"  "fsn"  "fak"  "rbi"  "cat"  "fbc"  "marb"
## [1301] "cly"  "clh"  "dod"  "lan"  "lvn"  "laci" "zga"  "mlt"  "asl"  "aev"
## [1311] "nom"  "nsd"  "noj"  "myr"  "mpw"  "mod"  "myz"  "wij"  "tdi"  "ten"
## [1321] "tmar" "lut"  "lul"  "wfu"  "for"  "foh"  "oaq"  "fek"  "aue"  "spon"
## [1331] "kos"  "kan"  "aqb"  "aqd"  "emar" "mur"  "oci"  "mgel" "mesq" "gaa"
## [1341] "cagg" "alti" "fba"  "fbu"  "ran"  "rai"  "rar"  "rag"  "rae"  "rat"
## [1351] "wvi"  "eao"  "emn"  "een"  "elb"  "emg"  "ego"  "egm"  "elz"  "elt"
## [1361] "chz"  "cgn"  "cih"  "chh"  "cio"  "chry" "cpip" "chrs" "chrz" "carh"
## [1371] "csha" "cnk"  "cjt"  "cil"  "ccau" "cben" "cjg"  "ccas" "cant" "kda"
## [1381] "clac" "eva"  "ctak" "cnr"  "ebv"  "efal" "este" "civ"  "fte"  "flu"
## [1391] "oho"  "bbau" "ial"  "cprv" "ttk"  "ddf"  "caby" "nde"  "nmv"  "nja"
## [1401] "nli"  "mox"  "afu"  "afg"  "gac"  "mac"  "mma"  "mmaz" "mmj"  "mmac"
## [1411] "metm" "msz"  "msw"  "mhor" "mfz"  "mem"  "mpd"  "hal"  "hsl"  "hdl"
```

```
## [1421] "hhb"  "hje"  "halh" "hhsr" "hsu"  "hsf"  "salr" "halr" "hma"  "hhi"
## [1431] "hhn"  "hab"  "hta"  "nph"  "nmo"  "hut"  "hti"  "hala" "hmu"  "halz"
## [1441] "hall" "hali" "hsn"  "hrr"  "hpel" "hlt"  "harc" "hwa"  "hwc"  "hvo"
## [1451] "hme"  "hgi"  "hale" "hbo"  "haq"  "haj"  "haer" "hra"  "hlm"  "halm"
## [1461] "hla"  "halp" "halb" "hezz" "halq" "hae"  "haln" "halg" "halu" "hdf"
## [1471] "hah"  "hda"  "hjt"  "haly" "nmg"  "hxa"  "nat"  "npe"  "npl"  "nge"
## [1481] "hru"  "nou"  "sali" "hlr"  "hlc"  "naj"  "nag"  "nan"  "nbg"  "nas"
## [1491] "tac"  "pto"  "fac"  "fai"  "cdiv" "ape"  "acj"  "sto"  "soh"  "sso"
## [1501] "sol"  "ssoa" "ssol" "ssof" "sai"  "sacn" "sacr" "sacs" "sis"  "sia"
## [1511] "sim"  "sid"  "siy"  "sin"  "sii"  "sih"  "sir"  "sic"  "sula" "sule"
## [1521] "mse"  "mcn"  "mhk"  "mpru" "mten" "aman" "abri" "asul" "sacd" "pis"
## [1531] "pcl"  "pas"  "pyr"  "pog"  "ttn"  "tuz"  "vdi"  "vmo"  "clg"  "nir"
## [1541] "nin"  "niw"  "nox"  "nue"  "nvn"  "nfn"  "csu"  "ccai" "loki" "psyt"
```
