# Supplementary material for: Exploring the expressiveness of abstract metabolic networks
Source: PLoS One. 2023 Feb 9;18(2):e0281047. doi: 10.1371/journal.pone.0281047 (PMC9910719; doi:10.1371/journal.pone.0281047)
Supplement: S5 File — Eukaryotes analyses at kingdom level (second experiment). (PDF) [file pone.0281047.s005.pdf]

Eukaryotes Analysis

- Vertex hystogram (VH) kernel
  - Heatmap
  - MDS for VH
  - 4-means for VH
- Shortest path (SP) kernel
  - Heatmap
  - MDS for SP
  - 4-means for SP
- Weisfeiler-Lehman (WL) kernel
  - Heatmap
  - MDS for WL
  - 4-means for WL
- Pyramid match (PM) kernel
  - Heatmap
  - MDS for PM
  - 4-means for PM

Vertex hystogram (VH) kernel

Heatmap

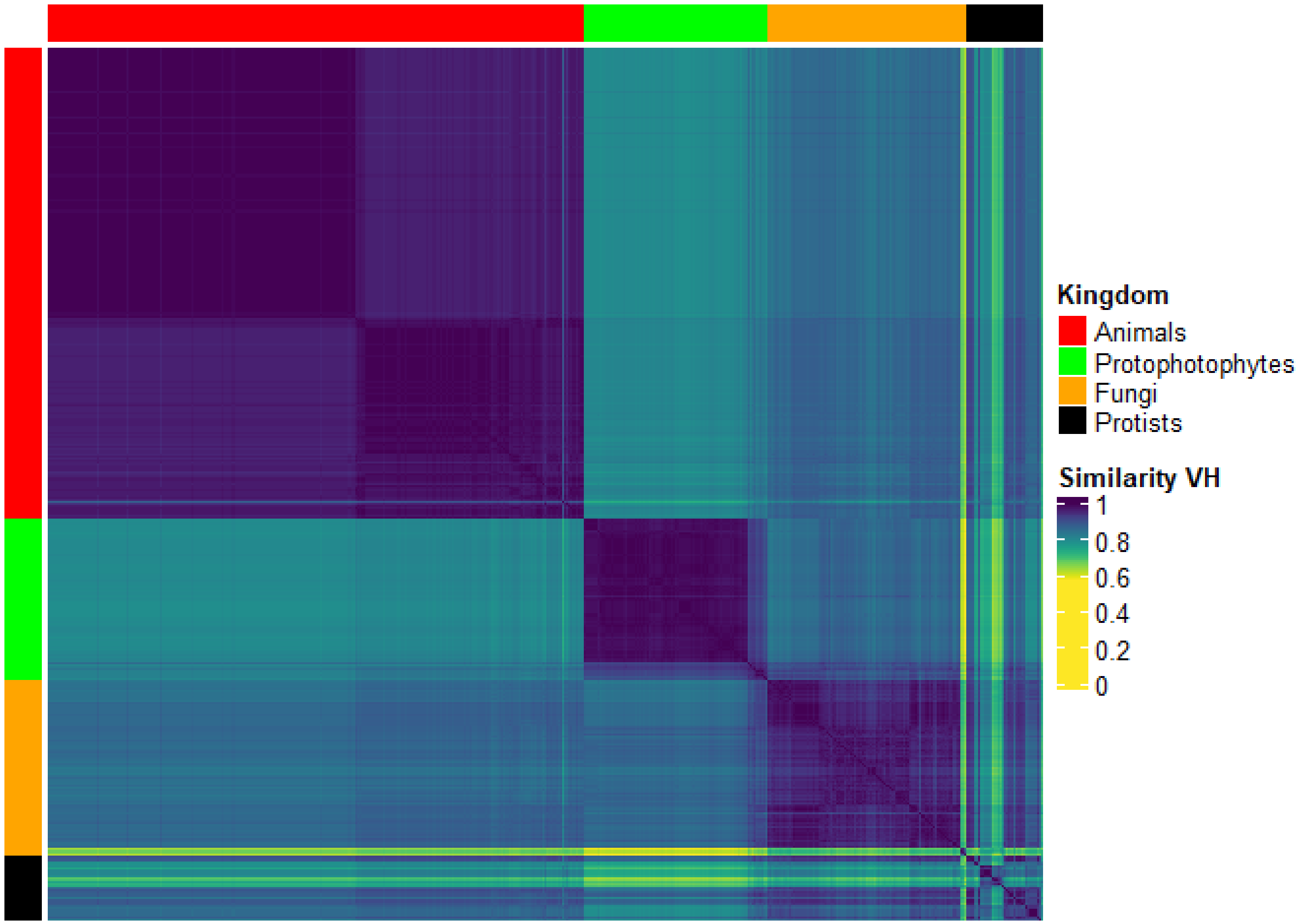

MDS for VH

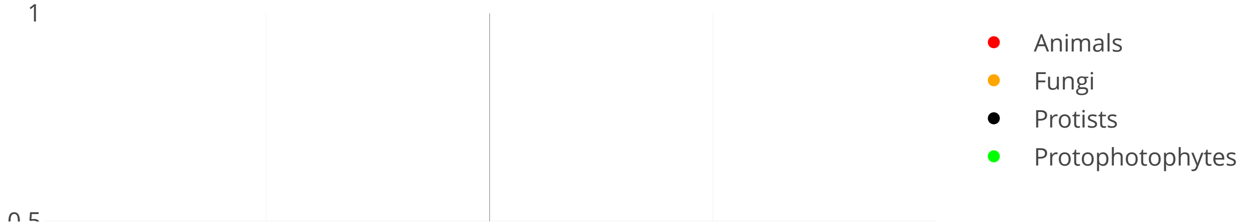

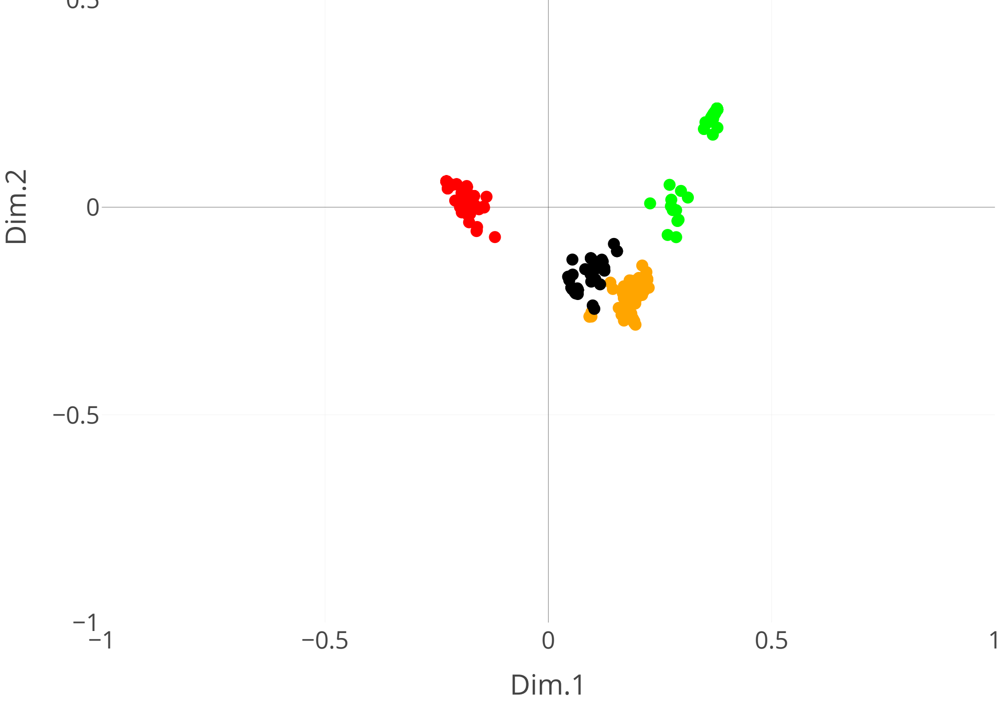

4-means for VH

```
##                      Cluster
## Real group              1   2   3   4
##   Animals               1   0   0 369
##   Fungi                 5   0 133   0
##   Protists             21   0  31   0
##   Protophotophytes      0 119   8   0
```

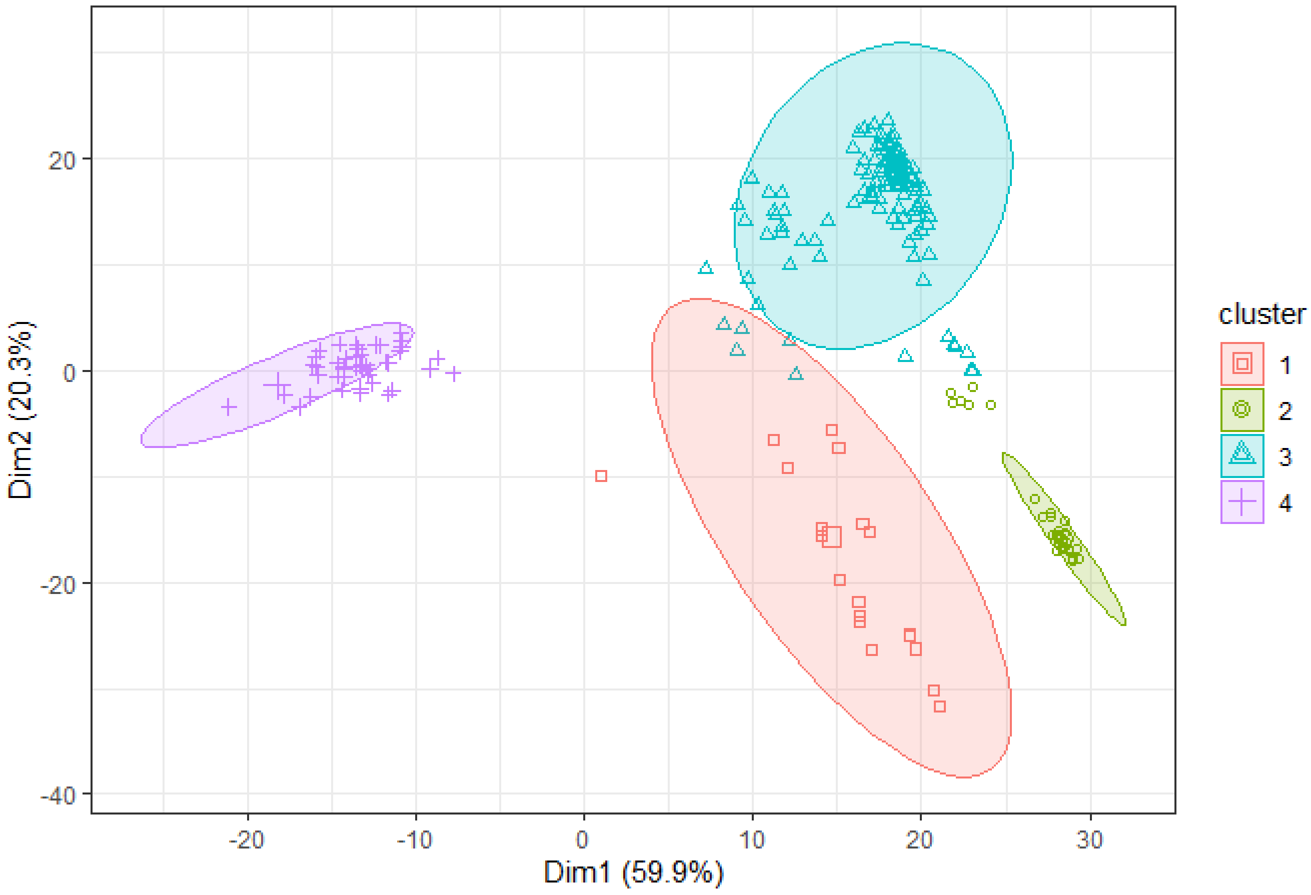

Organisms classified within cluster 1

```
##  [1] "shx"  "ecu"  "ein"  "ehe"  "ero"  "nce"  "ehi"  "edi"  "eiv"  "pfa"
## [11] "pfd"  "pfh"  "pyo"  "pcb"  "pbe"  "pkn"  "pvx"  "pcy"  "tan"  "tpv"
## [21] "tot"  "beq"  "bbo"  "bmic" "cpv"  "cho"  "gla"
```

Organisms classified within cluster 2

```
##   [1] "ath"  "aly"  "crb"  "csat" "eus"  "brp"  "bna"  "boe"  "rsz"  "thj"
##  [11] "cpap" "cit"  "cic"  "pvy"  "minc" "tcc"  "gra"  "ghi"  "gab"  "dzi"
##  [21] "egr"  "gmx"  "gsj"  "pvu"  "vra"  "var"  "vun"  "ccaj" "aprc" "mtr"
##  [31] "cam"  "lja"  "adu"  "aip"  "ahf"  "lang" "fve"  "rcn"  "pper" "pmum"
##  [41] "pavi" "pdul" "mdm"  "pxb"  "zju"  "mnt"  "csv"  "cmo"  "bhj"  "mcha"
##  [51] "cmax" "cmos" "cpep" "rcu"  "jcu"  "hbr"  "mesc" "pop"  "peu"  "palz"
##  [61] "jre"  "qsu"  "qlo"  "twl"  "vvi"  "vri"  "sly"  "spen" "sot"  "cann"
##  [71] "nta"  "nsy"  "nto"  "nau"  "ini"  "itr"  "sind" "oeu"  "egt"  "sspl"
##  [81] "han"  "ecad" "lsv"  "ccav" "dcr"  "csin" "bvg"  "soe"  "cqi"  "nnu"
##  [91] "ming" "psom" "ncol" "osa"  "dosa" "obr"  "bdi"  "ats"  "tdc"  "sbi"
## [101] "zma"  "sita" "pvir" "phai" "pda"  "egu"  "mus"  "dct"  "peq"  "aof"
## [111] "atr"  "smo"  "ppp"  "cre"  "mng"  "csl"  "cvr"  "bpg"  "ccp"
```

Organisms classified within cluster 3

```
##   [1] "vcn"  "apro" "olu"  "ota"  "mis"  "mpp"  "cme"  "gsl"  "sce"  "ago"
##  [11] "erc"  "kla"  "kmx"  "lth"  "vpo"  "zro"  "cgr"  "ncs"  "ndi"  "tpf"
##  [21] "tbl"  "tdl"  "tgb"  "kaf"  "zmk"  "ppa"  "dha"  "pic"  "pgu"  "spaa"
##  [31] "lel"  "cal"  "ctp"  "cot"  "cdu"  "cten" "yli"  "clu"  "clus" "caur"
##  [41] "slb"  "pkz"  "bnn"  "bbrx" "ncr"  "nte"  "smp"  "pan"  "ttt"  "mtm"
##  [51] "cthr" "mgr"  "tmn"  "ssck" "fgr"  "fpu"  "fvr"  "fox"  "nhe"  "tre"
##  [61] "trr"  "maw"  "maj"  "cmt"  "plj"  "val"  "vda"  "cfj"  "sapo" "ela"
##  [71] "pfy"  "ssl"  "bfu"  "mbe"  "psco" "glz"  "ani"  "afm"  "act"  "nfi"
##  [81] "aor"  "ang"  "afv"  "pcs"  "pdp"  "tmf"  "trg"  "cim"  "cpw"  "ure"
##  [91] "pbl"  "pbn"  "abe"  "tve"  "aje"  "bgh"  "pno"  "pte"  "bze"  "bsc"
## [101] "bor"  "aalt" "ztr"  "pfj"  "bcom" "npa"  "tml"  "spo"  "cne"  "cnb"
## [111] "cgi"  "tms"  "tasa" "ppl"  "tvs"  "dsq"  "pco"  "shs"  "hir"  "psq"
## [121] "adl"  "fme"  "gtr"  "lbc"  "mpr"  "mrr"  "cci"  "scm"  "abp"  "abv"
## [131] "cput" "sla"  "wse"  "wic"  "uma"  "pfp"  "mgl"  "mrt"  "msym" "pgr"
## [141] "mlr"  "mbr"  "sre"  "ddi"  "dpp"  "dfa"  "acan" "tgo"  "tet"  "ptm"
## [151] "smin" "pti"  "fcy"  "tps"  "ngd"  "aaf"  "pif"  "psoj" "spar" "ehx"
```

```
## [161] "gtt"  "tbr"  "tbg"  "tcr"  "lma"  "lif"  "ldo"  "lmi"  "lbz"  "lpan"
## [171] "ngr"  "tva"
```

Organisms classified within cluster 4

```
##   [1] "hsa"  "ptr"  "pps"  "ggo"  "pon"  "nle"  "mcc"  "mcf"  "csab" "caty"
##  [11] "panu" "rro"  "rbb"  "tfn"  "pteh" "cjc"  "sbq"  "mmur" "mmu"  "mcal"
##  [21] "mpah" "rno"  "mcoc" "mun"  "cge"  "pleu" "ngi"  "hgl"  "ccan" "ocu"
##  [31] "opi"  "tup"  "cfa"  "vvp"  "vlg"  "aml"  "umr"  "uah"  "oro"  "elk"
##  [41] "mpuf" "eju"  "mlx"  "fca"  "pyu"  "pbg"  "ptg"  "ppad" "aju"  "hhv"
##  [51] "bta"  "bom"  "biu"  "bbub" "chx"  "oas"  "oda"  "ccad" "ssc"  "cfr"
##  [61] "cbai" "cdk"  "bacu" "lve"  "oor"  "dle"  "pcad" "ecb"  "epz"  "eai"
##  [71] "myb"  "myd"  "mmyo" "mna"  "pkl"  "hai"  "dro"  "shon" "ajm"  "pdic"
##  [81] "mmf"  "rfq"  "pale" "pgig" "ray"  "mjv"  "tod"  "lav"  "tmu"  "mdo"
##  [91] "gas"  "shr"  "pcw"  "oaa"  "gga"  "pcoc" "mgp"  "cjo"  "nmel" "apla"
## [101] "acyg" "tgu"  "lsr"  "scan" "pmoa" "otc"  "pruf" "gfr"  "fab"  "phi"
## [111] "pmaj" "ccae" "ccw"  "etl"  "fpg"  "fch"  "clv"  "egz"  "nni"  "acun"
## [121] "padl" "aam"  "arow" "npd"  "dne"  "asn"  "amj"  "cpoo" "ggn"  "pss"
## [131] "cmy"  "cpic" "tst"  "cabi" "acs"  "pvt"  "sund" "pbi"  "pmur" "tsr"
## [141] "pgut" "vko"  "pmua" "zvi"  "gja"  "xla"  "xtr"  "npr"  "dre"  "srx"
## [151] "sanh" "sgh"  "ccar" "caua" "ipu"  "phyp" "amex" "eee"  "tru"  "tng"
## [161] "lco"  "ncc"  "cgob" "ely"  "plep" "sluc" "ecra" "pflv" "gat"  "ppug"
## [171] "msam" "cud"  "mze"  "onl"  "oau"  "ola"  "oml"  "xma"  "xco"  "xhe"
## [181] "pret" "cvg"  "ctul" "nfu"  "kmr"  "alim" "aoce" "csem" "pov"  "ssen"
## [191] "lcf"  "sdu"  "slal" "xgl"  "hcq"  "bpec" "malb" "sasa" "otw"  "omy"
## [201] "salp" "snh"  "els"  "sfm"  "pki"  "aang" "loc"  "pspa" "arut" "lcm"
## [211] "cmk"  "rtp"  "bfo"  "bbel" "cin"  "sclv" "spu"  "aplc" "sko"  "dme"
## [221] "der"  "dse"  "dsi"  "dya"  "dan"  "dsr"  "dpo"  "dpe"  "dmn"  "dwi"
## [231] "dgr"  "dmo"  "daz"  "dnv"  "dhe"  "dvi"  "ccat" "bod"  "mde"  "scac"
## [241] "lcq"  "aga"  "acoz" "aara" "aag"  "aalb" "cqu"  "cpii" "ame"  "acer"
## [251] "bim"  "bbif" "bvk"  "bvan" "bter" "ccal" "obb"  "mgen" "nmea" "cgig"
## [261] "soc"  "mpha" "aec"  "acep" "pbar" "vem"  "hst"  "dqu"  "cfo"  "fex"
## [271] "lhu"  "pgc"  "obo"  "pcf"  "pfuc" "vps"  "nvi"  "csol" "tpre" "mdl"
```

```
## [281] "cglo" "fas"  "dam"  "ccin" "tca"  "dpa"  "atd"  "agb"  "ldc"  "nvl"
## [291] "apln" "ppyr" "otu"  "bmor" "bman" "msex" "dpl"  "bany" "pmac" "ppot"
## [301] "pxu"  "prap" "zce"  "haw"  "tnl"  "pxy"  "api"  "dnx"  "ags"  "rmd"
## [311] "btab" "dci"  "clec" "hhal" "nlu"  "phu"  "foc"  "zne"  "csec" "fcd"
## [321] "dpx"  "dmk"  "pvm"  "pja"  "hame" "hazt" "eaf"  "isc"  "dsv"  "rsan"
## [331] "rmp"  "vde"  "vja"  "tut"  "dpte" "cscu" "ptep" "sdm"  "cel"  "cbr"
## [341] "bmy"  "loa"  "nai"  "tsp"  "hro"  "lgi"  "pcan" "bgt"  "gae"  "crg"
## [351] "myi"  "pmax" "obi"  "osn"  "lak"  "smm"  "ovi"  "egl"  "nve"  "epa"
## [361] "aten" "adf"  "amil" "pdam" "spis" "dgt"  "hmg"  "tad"  "aqu"
```

Shortest path (SP) kernel

Heatmap

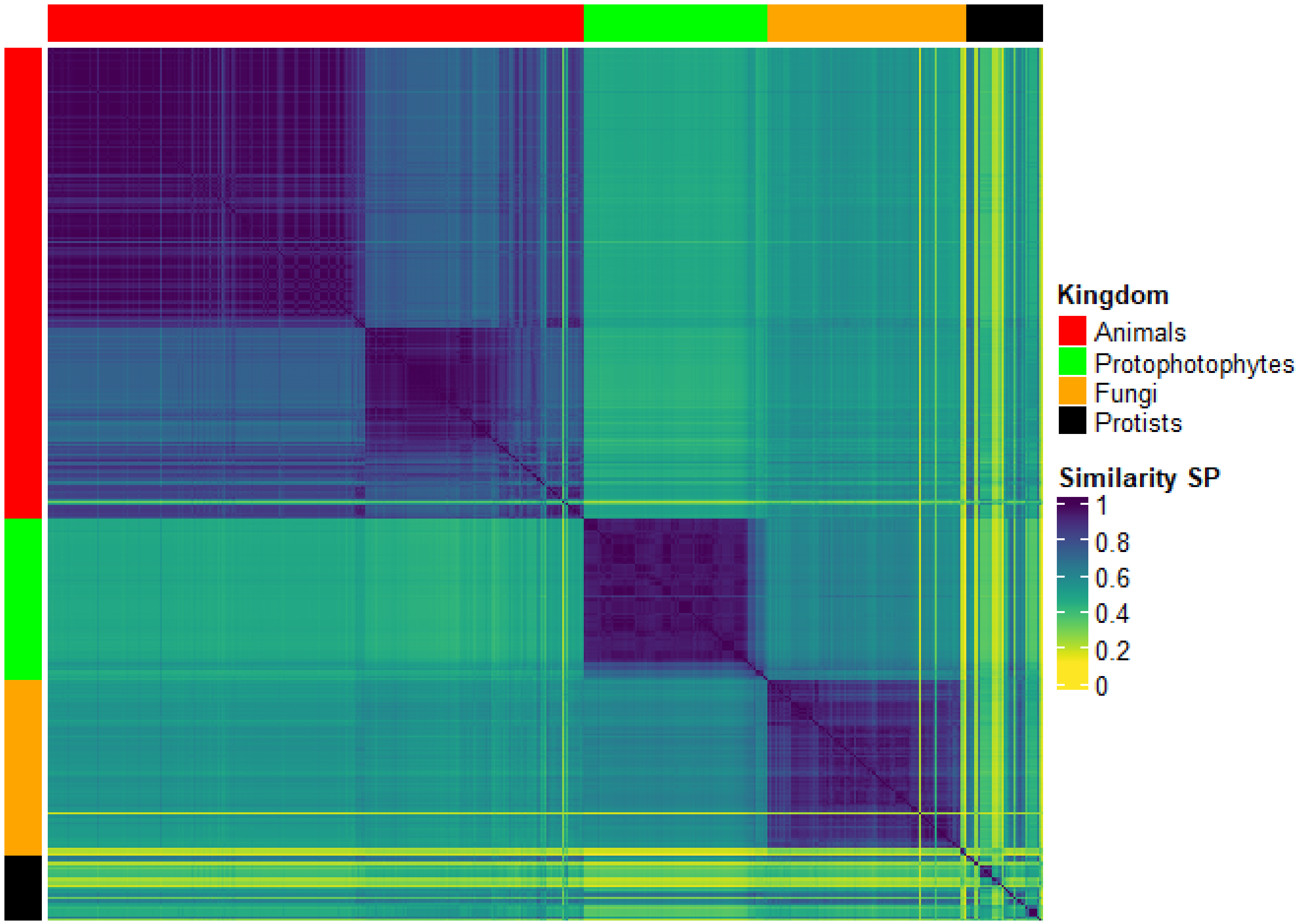
Kingdom
Animals
Protophotophytes
Fungi
Protists
Similarity SP
1
0.8
0.6
0.4
0.2
0

MDS for SP

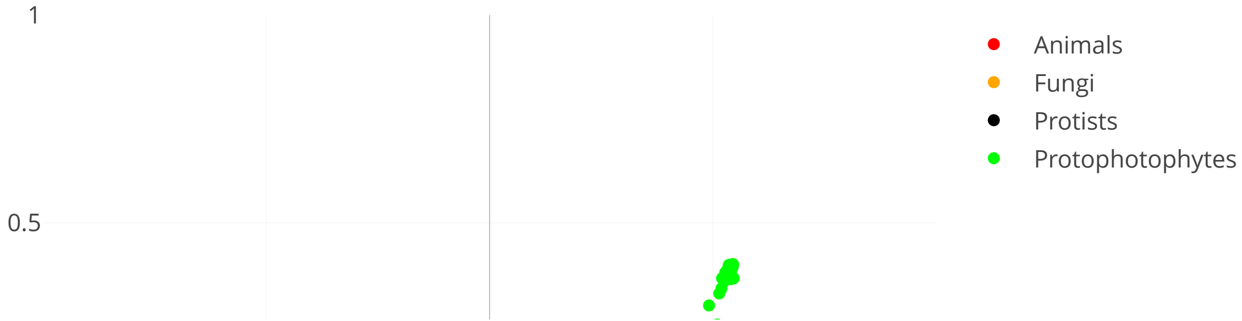
1
0.5
Animals
Fungi
Protists
Protophotophytes

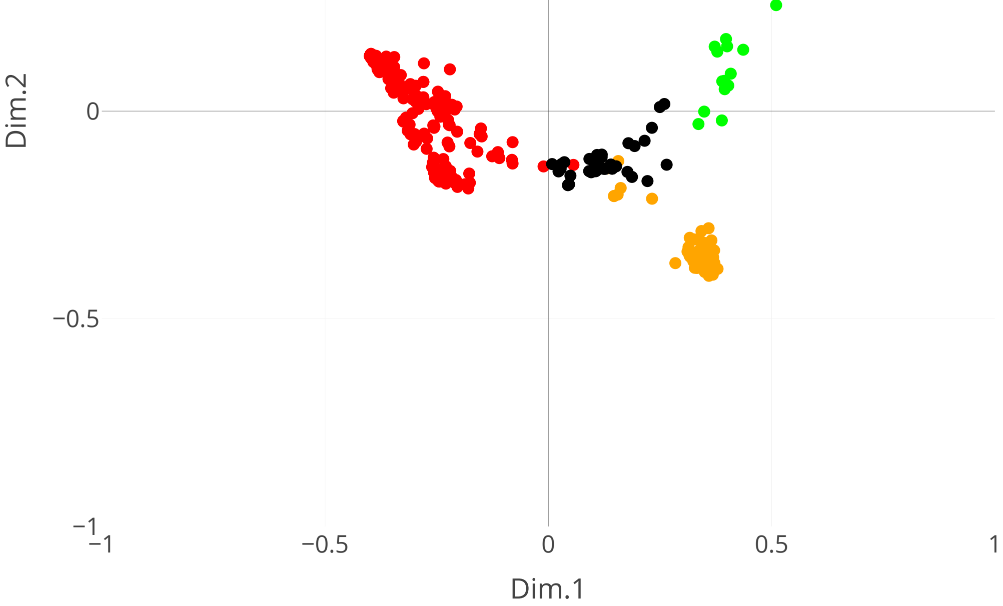

4-means for SP

```
##                      Cluster
## Real group             1   2   3   4
##   Animals              0   0   3 367
##   Fungi              131   0   7   0
##   Protists            14   0  35   3
##   Protophotophytes     0 127   0   0
```

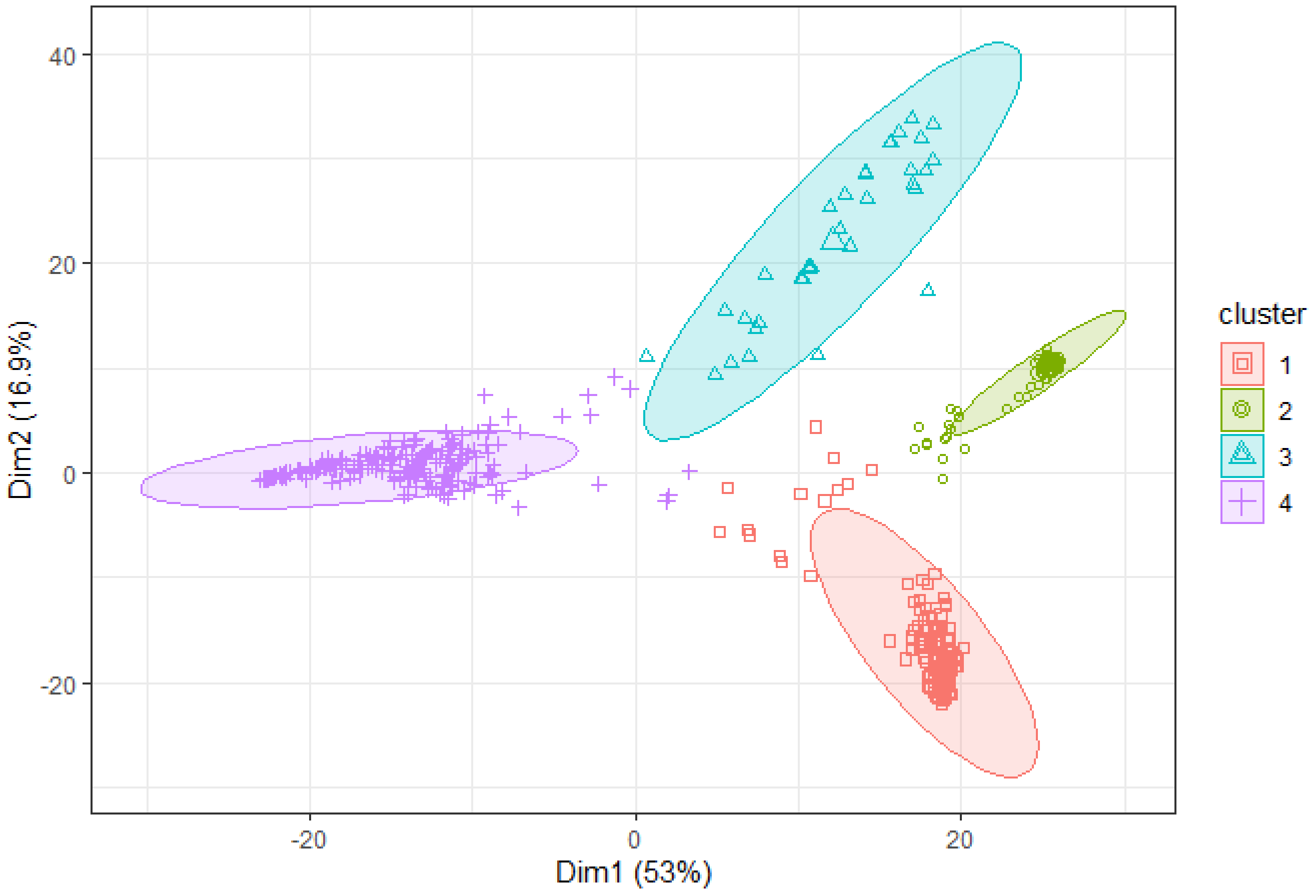

Organisms classified within cluster 1

```
##   [1] "sce"  "ago"  "erc"  "kla"  "kmx"  "lth"  "vpo"  "zro"  "cgr"  "ncs"
##  [11] "ndi"  "tpf"  "tbl"  "tdl"  "tgb"  "kaf"  "zmk"  "ppa"  "dha"  "pic"
##  [21] "pgu"  "spaa" "lel"  "cal"  "ctp"  "cot"  "cdu"  "cten" "yli"  "clu"
##  [31] "clus" "caur" "slb"  "pkz"  "bnn"  "bbrx" "ncr"  "nte"  "smp"  "pan"
##  [41] "ttt"  "mtm"  "cthr" "mgr"  "tmn"  "ssck" "fgr"  "fpu"  "fvr"  "fox"
##  [51] "nhe"  "tre"  "trr"  "maw"  "maj"  "cmt"  "plj"  "val"  "vda"  "cfj"
##  [61] "sapo" "ela"  "pfy"  "ssl"  "bfu"  "mbe"  "psco" "glz"  "ani"  "afm"
##  [71] "act"  "nfi"  "aor"  "ang"  "afv"  "pcs"  "pdp"  "tmf"  "trg"  "cim"
```

```
##  [81] "cpw"  "ure"  "pbl"  "pbn"  "abe"  "tve"  "aje"  "bgh"  "pno"  "pte" 
##  [91] "bze"  "bsc"  "bor"  "aalt" "ztr"  "pfj"  "bcom" "npa"  "tml"  "spo" 
## [101] "cne"  "cnb"  "cgi"  "tms"  "tasa" "tvs"  "dsq"  "pco"  "shs"  "hir" 
## [111] "psq"  "adl"  "fme"  "gtr"  "lbc"  "mrr"  "cci"  "scm"  "abp"  "abv" 
## [121] "cput" "sla"  "wse"  "wic"  "uma"  "pfp"  "mgl"  "mrt"  "msym" "pgr" 
## [131] "mlr"  "mbr"  "sre"  "dfa"  "acan" "smin" "pti"  "fcy"  "tps"  "aaf" 
## [141] "pif"  "psoj" "spar" "ehx"  "gtt"
```

Organisms classified within cluster 2

```
##   [1] "ath"  "aly"  "crb"  "csat" "eus"  "brp"  "bna"  "boe"  "rsz"  "thj" 
##  [11] "cpap" "cit"  "cic"  "pvy"  "minc" "tcc"  "gra"  "ghi"  "gab"  "dzi" 
##  [21] "egr"  "gmx"  "gsj"  "pvu"  "vra"  "var"  "vun"  "ccaj" "aprc" "mtr" 
##  [31] "cam"  "lja"  "adu"  "aip"  "ahf"  "lang" "fve"  "rcn"  "pper" "pmum"
##  [41] "pavi" "pdul" "mdm"  "pxb"  "zju"  "mnt"  "csv"  "cmo"  "bhj"  "mcha"
##  [51] "cmax" "cmos" "cpep" "rcu"  "jcu"  "hbr"  "mesc" "pop"  "peu"  "palz"
##  [61] "jre"  "qsu"  "qlo"  "twl"  "vvi"  "vri"  "sly"  "spen" "sot"  "cann"
##  [71] "nta"  "nsy"  "nto"  "nau"  "ini"  "itr"  "sind" "oeu"  "egt"  "sspl"
##  [81] "han"  "ecad" "lsv"  "ccav" "dcr"  "csin" "bvg"  "soe"  "cqi"  "nnu" 
##  [91] "ming" "psom" "ncol" "osa"  "dosa" "obr"  "bdi"  "ats"  "tdc"  "sbi" 
## [101] "zma"  "sita" "pvir" "phai" "pda"  "egu"  "mus"  "dct"  "peq"  "aof" 
## [111] "atr"  "smo"  "ppp"  "cre"  "vcn"  "mng"  "csl"  "cvr"  "apro" "olu" 
## [121] "ota"  "bpg"  "mis"  "mpp"  "cme"  "gsl"  "ccp"
```

Organisms classified within cluster 3

```
##  [1] "tsp"  "shx"  "egl"  "ppl"  "mpr"  "ecu"  "ein"  "ehe"  "ero"  "nce" 
## [11] "ehi"  "edi"  "eiv"  "pfa"  "pfd"  "pfh"  "pyo"  "pcb"  "pbe"  "pkn" 
## [21] "pvx"  "pcy"  "tan"  "tpv"  "tot"  "beq"  "bbo"  "bmic" "cpv"  "cho" 
## [31] "tgo"  "tet"  "ptm"  "ngd"  "tbr"  "tbg"  "tcr"  "lma"  "lif"  "ldo" 
## [41] "lmi"  "lbz"  "lpan" "tva"  "gla"
```

Organisms classified within cluster 4

```
##   [1] "hsa"  "ptr"  "pps"  "ggo"  "pon"  "nle"  "mcc"  "mcf"  "csab" "caty"
##  [11] "panu" "rro"  "rbb"  "tfn"  "pteh" "cjc"  "sbq"  "mmur" "mmu"  "mcal"
##  [21] "mpah" "rno"  "mcoc" "mun"  "cge"  "pleu" "ngi"  "hgl"  "ccan" "ocu"
##  [31] "opi"  "tup"  "cfa"  "vvp"  "vlg"  "aml"  "umr"  "uah"  "oro"  "elk"
##  [41] "mpuf" "eju"  "mlx"  "fca"  "pyu"  "pbg"  "ptg"  "ppad" "aju"  "hhv"
##  [51] "bta"  "bom"  "biu"  "bbub" "chx"  "oas"  "oda"  "ccad" "ssc"  "cfr"
##  [61] "cbai" "cdk"  "bacu" "lve"  "oor"  "dle"  "pcad" "ecb"  "epz"  "eai"
##  [71] "myb"  "myd"  "mmyo" "mna"  "pkl"  "hai"  "dro"  "shon" "ajm"  "pdic"
##  [81] "mmf"  "rfq"  "pale" "pgig" "ray"  "mjv"  "tod"  "lav"  "tmu"  "mdo"
##  [91] "gas"  "shr"  "pcw"  "oaa"  "gga"  "pcoc" "mgp"  "cjo"  "nmel" "apla"
## [101] "acyg" "tgu"  "lsr"  "scan" "pmoa" "otc"  "pruf" "gfr"  "fab"  "phi"
## [111] "pmaj" "ccae" "ccw"  "etl"  "fpg"  "fch"  "clv"  "egz"  "nni"  "acun"
## [121] "padl" "aam"  "arow" "npd"  "dne"  "asn"  "amj"  "cpoo" "ggn"  "pss"
## [131] "cmy"  "cpic" "tst"  "cabi" "acs"  "pvt"  "sund" "pbi"  "pmur" "tsr"
## [141] "pgut" "vko"  "pmua" "zvi"  "gja"  "xla"  "xtr"  "npr"  "dre"  "srx"
## [151] "sanh" "sgh"  "ccar" "caua" "ipu"  "phyp" "amex" "eee"  "tru"  "tng"
## [161] "lco"  "ncc"  "cgob" "ely"  "plep" "sluc" "ecra" "pflv" "gat"  "ppug"
## [171] "msam" "cud"  "mze"  "onl"  "oau"  "ola"  "oml"  "xma"  "xco"  "xhe"
## [181] "pret" "cvg"  "ctul" "nfu"  "kmr"  "alim" "aoce" "csem" "pov"  "ssen"
## [191] "lcf"  "sdu"  "slal" "xgl"  "hcq"  "bpec" "malb" "sasa" "otw"  "omy"
## [201] "salp" "snh"  "els"  "sfm"  "pki"  "aang" "loc"  "pspa" "arut" "lcm"
## [211] "cmk"  "rtp"  "bfo"  "bbel" "cin"  "sclv" "spu"  "aplc" "sko"  "dme"
## [221] "der"  "dse"  "dsi"  "dya"  "dan"  "dsr"  "dpo"  "dpe"  "dmn"  "dwi"
## [231] "dgr"  "dmo"  "daz"  "dnv"  "dhe"  "dvi"  "ccat" "bod"  "mde"  "scac"
## [241] "lcq"  "aga"  "acoz" "aara" "aag"  "aalb" "cqu"  "cpii" "ame"  "acer"
## [251] "bim"  "bbif" "bvk"  "bvan" "bter" "ccal" "obb"  "mgen" "nmea" "cgig"
## [261] "soc"  "mpha" "aec"  "acep" "pbar" "vem"  "hst"  "dqu"  "cfo"  "fex"
## [271] "lhu"  "pgc"  "obo"  "pcf"  "pfuc" "vps"  "nvi"  "csol" "tpre" "mdl"
## [281] "cglo" "fas"  "dam"  "ccin" "tca"  "dpa"  "atd"  "agb"  "ldc"  "nvl"
## [291] "apln" "ppyr" "otu"  "bmor" "bman" "msex" "dpl"  "bany" "pmac" "ppot"
## [301] "pxu"  "prap" "zce"  "haw"  "tnl"  "pxy"  "api"  "dnx"  "ags"  "rmd"
```

```
## [311] "btab" "dci"  "clec" "hhal" "nlu"  "phu"  "foc"  "zne"  "csec" "fcd"
## [321] "dpx"  "dmk"  "pvm"  "pja"  "hame" "hazt" "eaf"  "isc"  "dsv"  "rsan"
## [331] "rmp"  "vde"  "vja"  "tut"  "dpte" "cscu" "ptep" "sdm"  "cel"  "cbr"
## [341] "bmy"  "loa"  "nai"  "hro"  "lgi"  "pcan" "bgt"  "gae"  "crg"  "myi"
## [351] "pmax" "obi"  "osn"  "lak"  "smm"  "ovi"  "nve"  "epa"  "aten" "adf"
## [361] "amil" "pdam" "spis" "dgt"  "hmg"  "tad"  "aqu"  "ddi"  "dpp"  "ngr"
```

Weisfeiler-Lehman (WL) kernel

Heatmap

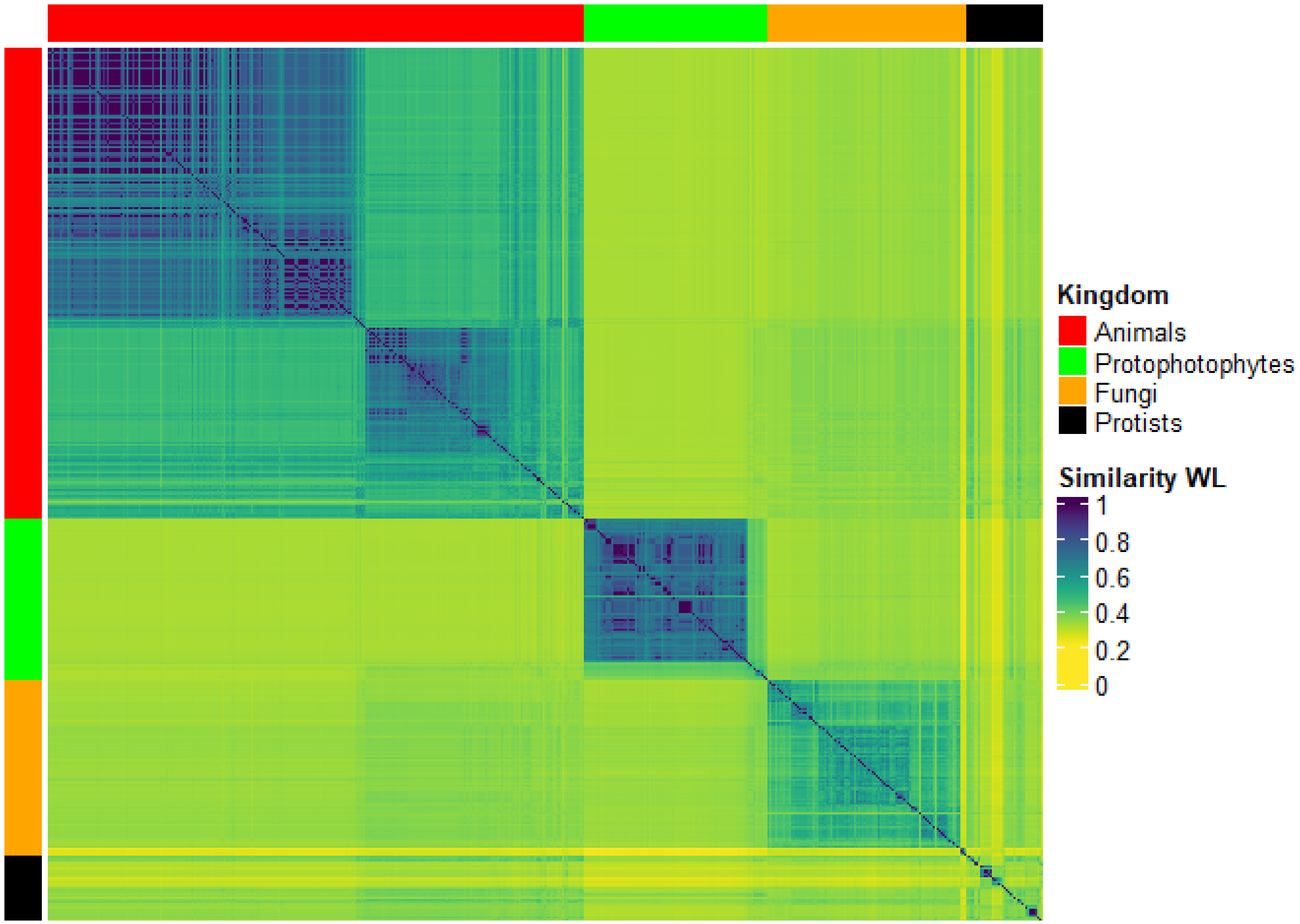
Kingdom
Animals
Protophotophytes
Fungi
Protists
Similarity WL
1
0.8
0.6
0.4
0.2
0

MDS for WL

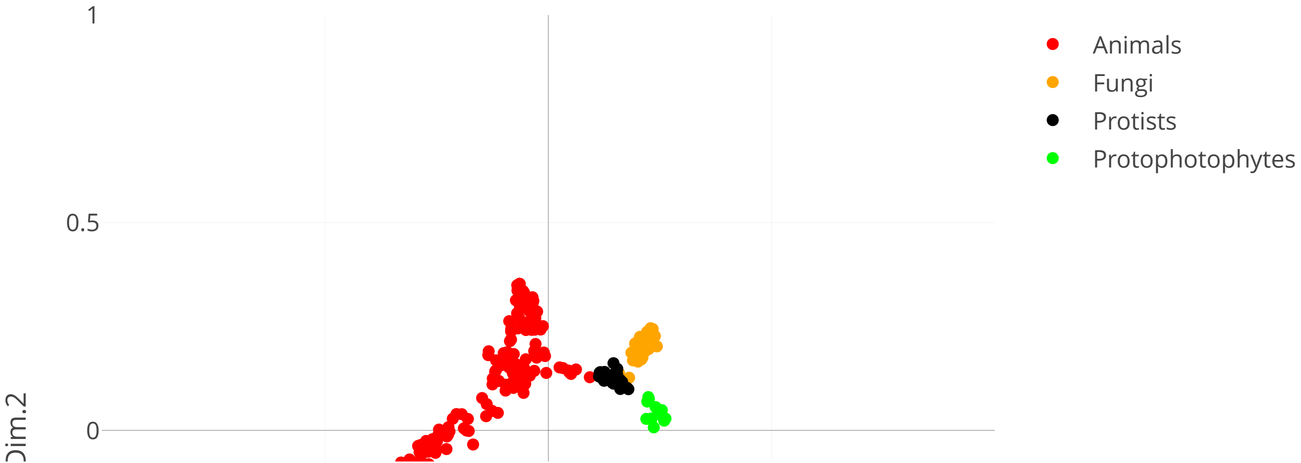
1
0.5
0
Dim.2
Animals
Fungi
Protists
Protophotophytes

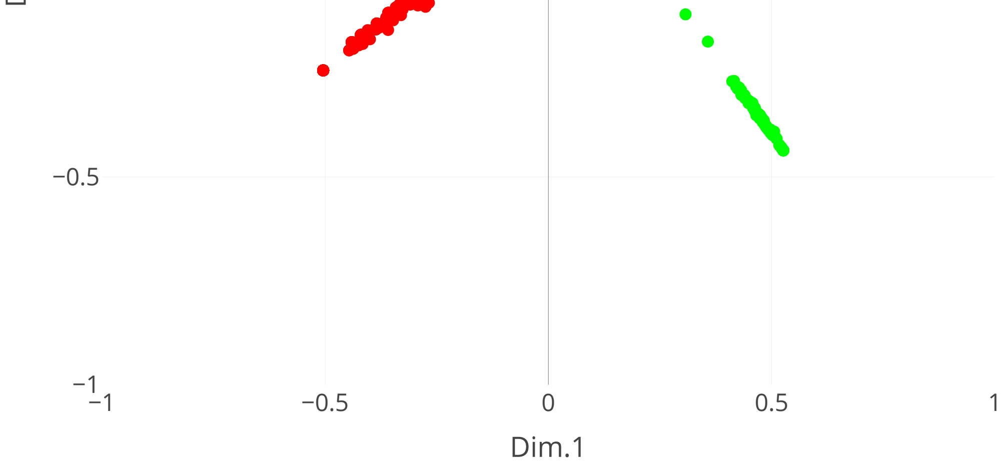

4-means for WL

```
##                         Cluster
## Real group                 1   2   3   4
##    Animals                 0 212 157   1
##    Fungi                   0   0   0 138
##    Protists                0   0   0  52
##    Protophotophytes 113   0   0  14
```

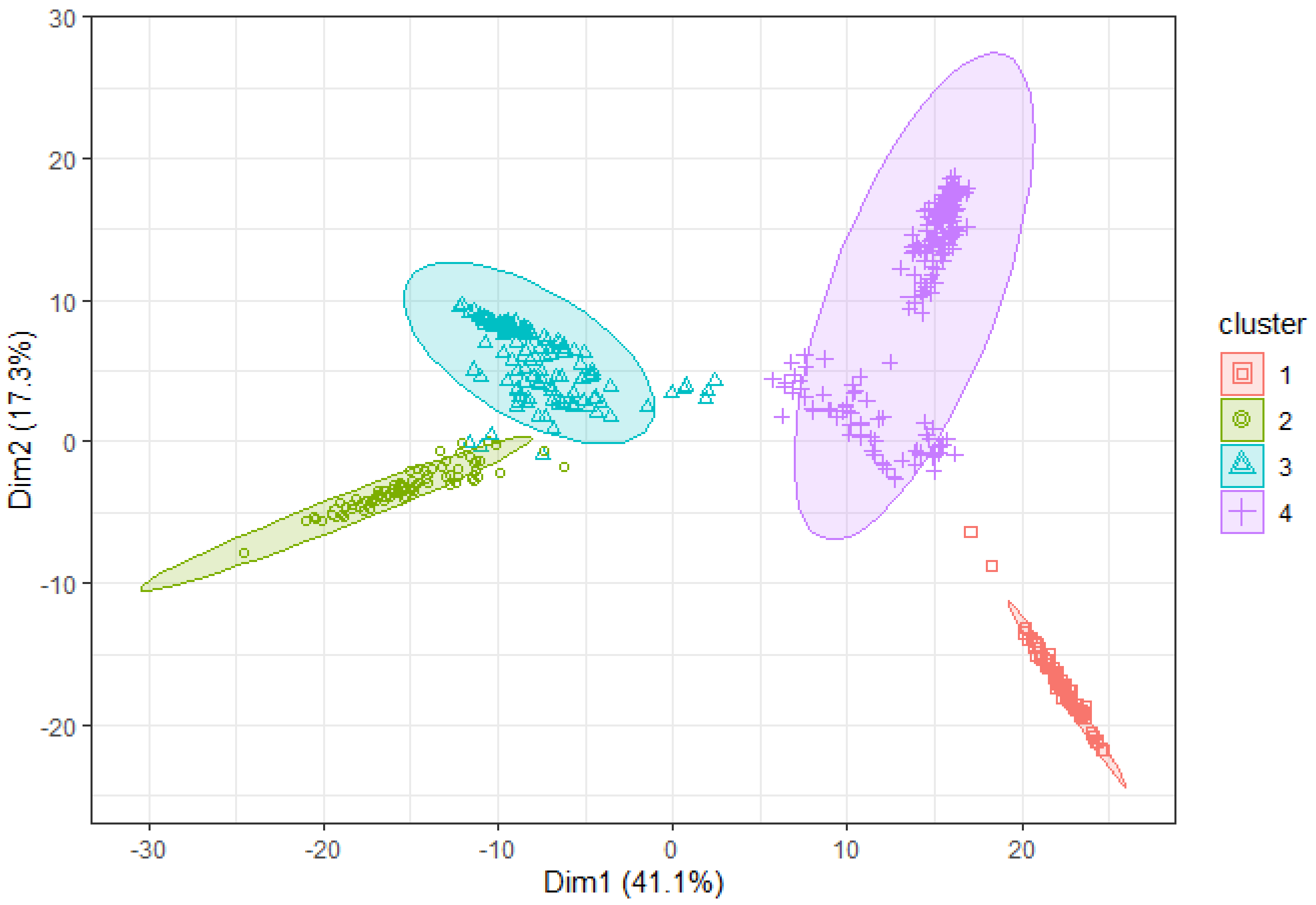

Organisms classified within cluster 1

```
##   [1] "ath"   "aly"   "crb"   "csat" "eus"   "brp"   "bna"   "boe"   "rsz"   "thj"
##  [11] "cpap" "cit"   "cic"   "pvy"   "minc" "tcc"   "gra"   "ghi"   "gab"   "dzi"
##  [21] "egr"   "gmx"   "gsj"   "pvu"   "vra"   "var"   "vun"   "ccaj" "aprc" "mtr"
##  [31] "cam"   "lja"   "adu"   "aip"   "ahf"   "lang" "fve"   "rcn"   "pper" "pmum"
##  [41] "pavi" "pdul" "mdm"   "pxb"   "zju"   "mnt"   "csv"   "cmo"   "bhj"   "mcha"
##  [51] "cmax" "cmos" "cpep" "rcu"   "jcu"   "hbr"   "mesc" "pop"   "peu"   "palz"
##  [61] "jre"   "qsu"   "qlo"   "twl"   "vvi"   "vri"   "sly"   "spen" "sot"   "cann"
##  [71] "nta"   "nsy"   "nto"   "nau"   "ini"   "itr"   "sind" "oeu"   "egt"   "sspl"
```

```
##  [81] "han"  "ecad" "lsv"  "ccav" "dcr"  "csin" "bvg"  "soe"  "cqi"  "nnu"
##  [91] "ming" "psom" "ncol" "osa"  "dosa" "obr"  "bdi"  "ats"  "tdc"  "sbi"
## [101] "zma"  "sita" "pvir" "phai" "pda"  "egu"  "mus"  "dct"  "peq"  "aof"
## [111] "atr"  "smo"  "ppp"
```

Organisms classified within cluster 2

```
##   [1] "hsa"  "ptr"  "pps"  "ggo"  "pon"  "nle"  "mcc"  "mcf"  "csab" "caty"
##  [11] "panu" "rro"  "rbb"  "tfn"  "pteh" "cjc"  "sbq"  "mmur" "mmu"  "mcal"
##  [21] "mpah" "rno"  "mcoc" "mun"  "cge"  "pleu" "ngi"  "hgl"  "ccan" "ocu"
##  [31] "opi"  "tup"  "cfa"  "vvp"  "vlg"  "aml"  "umr"  "uah"  "oro"  "elk"
##  [41] "mpuf" "eju"  "mlx"  "fca"  "pyu"  "pbg"  "ptg"  "ppad" "aju"  "hhv"
##  [51] "bta"  "bom"  "biu"  "bbub" "chx"  "oas"  "oda"  "ccad" "ssc"  "cfr"
##  [61] "cbai" "cdk"  "bacu" "lve"  "oor"  "dle"  "pcad" "ecb"  "epz"  "eai"
##  [71] "myb"  "myd"  "mmyo" "mna"  "pkl"  "hai"  "dro"  "shon" "ajm"  "pdic"
##  [81] "mmf"  "rfq"  "pale" "pgig" "ray"  "mjv"  "tod"  "lav"  "tmu"  "mdo"
##  [91] "gas"  "shr"  "pcw"  "oaa"  "gga"  "pcoc" "mgp"  "cjo"  "nmel" "acyg"
## [101] "tgu"  "lsr"  "scan" "pmoa" "otc"  "pruf" "gfr"  "fab"  "phi"  "pmaj"
## [111] "ccae" "ccw"  "etl"  "fpg"  "fch"  "clv"  "egz"  "nni"  "acun" "aam"
## [121] "arow" "npd"  "dne"  "asn"  "amj"  "cpoo" "ggn"  "pss"  "cmy"  "cpic"
## [131] "tst"  "cabi" "acs"  "pvt"  "sund" "pbi"  "pmur" "tsr"  "pgut" "vko"
## [141] "pmua" "zvi"  "gja"  "xla"  "xtr"  "npr"  "dre"  "srx"  "sanh" "sgh"
## [151] "ccar" "caua" "ipu"  "phyp" "amex" "eee"  "tru"  "tng"  "lco"  "ncc"
## [161] "cgob" "ely"  "plep" "sluc" "ecra" "pflv" "gat"  "ppug" "msam" "cud"
## [171] "mze"  "onl"  "oau"  "ola"  "oml"  "xma"  "xco"  "xhe"  "pret" "cvg"
## [181] "ctul" "nfu"  "kmr"  "alim" "aoce" "csem" "pov"  "ssen" "lcf"  "sdu"
## [191] "slal" "xgl"  "hcq"  "bpec" "malb" "sasa" "otw"  "omy"  "salp" "snh"
## [201] "els"  "sfm"  "pki"  "aang" "loc"  "pspa" "arut" "lcm"  "rtp"  "bfo"
## [211] "bbel" "sclv"
```

Organisms classified within cluster 3

```
##   [1] "apla" "padl" "cmk"  "cin"  "spu"  "aplc" "sko"  "dme"  "der"  "dse" 
##  [11] "dsi"  "dya"  "dan"  "dsr"  "dpo"  "dpe"  "dmn"  "dwi"  "dgr"  "dmo" 
##  [21] "daz"  "dnv"  "dhe"  "dvi"  "ccat" "bod"  "mde"  "scac" "lcq"  "aga" 
##  [31] "acoz" "aara" "aag"  "aalb" "cqu"  "cpii" "ame"  "acer" "bim"  "bbif"
##  [41] "bvk"  "bvan" "bter" "ccal" "obb"  "mgen" "nmea" "cgig" "soc"  "mpha"
##  [51] "aec"  "acep" "pbar" "vem"  "hst"  "dqu"  "cfo"  "fex"  "lhu"  "pgc" 
##  [61] "obo"  "pcf"  "pfuc" "vps"  "nvi"  "csol" "tpre" "mdl"  "cglo" "fas" 
##  [71] "dam"  "ccin" "tca"  "dpa"  "atd"  "agb"  "ldc"  "nvl"  "apln" "ppyr"
##  [81] "otu"  "bmor" "bman" "msex" "dpl"  "bany" "pmac" "ppot" "pxu"  "prap"
##  [91] "zce"  "haw"  "tnl"  "pxy"  "api"  "dnx"  "ags"  "rmd"  "btab" "dci" 
## [101] "clec" "hhal" "nlu"  "phu"  "foc"  "zne"  "csec" "fcd"  "dpx"  "dmk" 
## [111] "pvm"  "pja"  "hame" "hazt" "eaf"  "isc"  "dsv"  "rsan" "rmp"  "vde" 
## [121] "vja"  "tut"  "dpte" "cscu" "ptep" "sdm"  "cel"  "cbr"  "bmy"  "loa" 
## [131] "nai"  "tsp"  "hro"  "lgi"  "pcan" "bgt"  "gae"  "crg"  "myi"  "pmax"
## [141] "obi"  "osn"  "lak"  "smm"  "ovi"  "egl"  "nve"  "epa"  "aten" "adf" 
## [151] "amil" "pdam" "spis" "dgt"  "hmg"  "tad"  "aqu"
```

Organisms classified within cluster 4

```
##   [1] "shx"  "cre"  "vcn"  "mng"  "csl"  "cvr"  "apro" "olu"  "ota"  "bpg" 
##  [11] "mis"  "mpp"  "cme"  "gsl"  "ccp"  "sce"  "ago"  "erc"  "kla"  "kmx" 
##  [21] "lth"  "vpo"  "zro"  "cgr"  "ncs"  "ndi"  "tpf"  "tbl"  "tdl"  "tgb" 
##  [31] "kaf"  "zmk"  "ppa"  "dha"  "pic"  "pgu"  "spaa" "lel"  "cal"  "ctp" 
##  [41] "cot"  "cdu"  "cten" "yli"  "clu"  "clus" "caur" "slb"  "pkz"  "bnn" 
##  [51] "bbrx" "ncr"  "nte"  "smp"  "pan"  "ttt"  "mtm"  "cthr" "mgr"  "tmn" 
##  [61] "ssck" "fgr"  "fpu"  "fvr"  "fox"  "nhe"  "tre"  "trr"  "maw"  "maj" 
##  [71] "cmt"  "plj"  "val"  "vda"  "cfj"  "sapo" "ela"  "pfy"  "ssl"  "bfu" 
##  [81] "mbe"  "psco" "glz"  "ani"  "afm"  "act"  "nfi"  "aor"  "ang"  "afv" 
##  [91] "pcs"  "pdp"  "tmf"  "trg"  "cim"  "cpw"  "ure"  "pbl"  "pbn"  "abe" 
## [101] "tve"  "aje"  "bgh"  "pno"  "pte"  "bze"  "bsc"  "bor"  "aalt" "ztr" 
## [111] "pfj"  "bcom" "npa"  "tml"  "spo"  "cne"  "cnb"  "cgi"  "tms"  "tasa"
## [121] "ppl"  "tvs"  "dsq"  "pco"  "shs"  "hir"  "psq"  "adl"  "fme"  "gtr"
```

```
## [131] "lbc"  "mpr"  "mrr"  "cci"  "scm"  "abp"  "abv"  "cput" "sla"  "wse"
## [141] "wic"  "uma"  "pfp"  "mgl"  "mrt"  "msym" "pgr"  "mlr"  "ecu"  "ein"
## [151] "ehe"  "ero"  "nce"  "mbr"  "sre"  "ddi"  "dpp"  "dfa"  "ehi"  "edi"
## [161] "eiv"  "acan" "pfa"  "pfd"  "pfh"  "pyo"  "pcb"  "pbe"  "pkn"  "pvx"
## [171] "pcy"  "tan"  "tpv"  "tot"  "beq"  "bbo"  "bmic" "cpv"  "cho"  "tgo"
## [181] "tet"  "ptm"  "smin" "pti"  "fcy"  "tps"  "ngd"  "aaf"  "pif"  "psoj"
## [191] "spar" "ehx"  "gtt"  "tbr"  "tbg"  "tcr"  "lma"  "lif"  "ldo"  "lmi"
## [201] "lbz"  "lpan" "ngr"  "tva"  "gla"
```

Pyramid match (PM) kernel

Heatmap

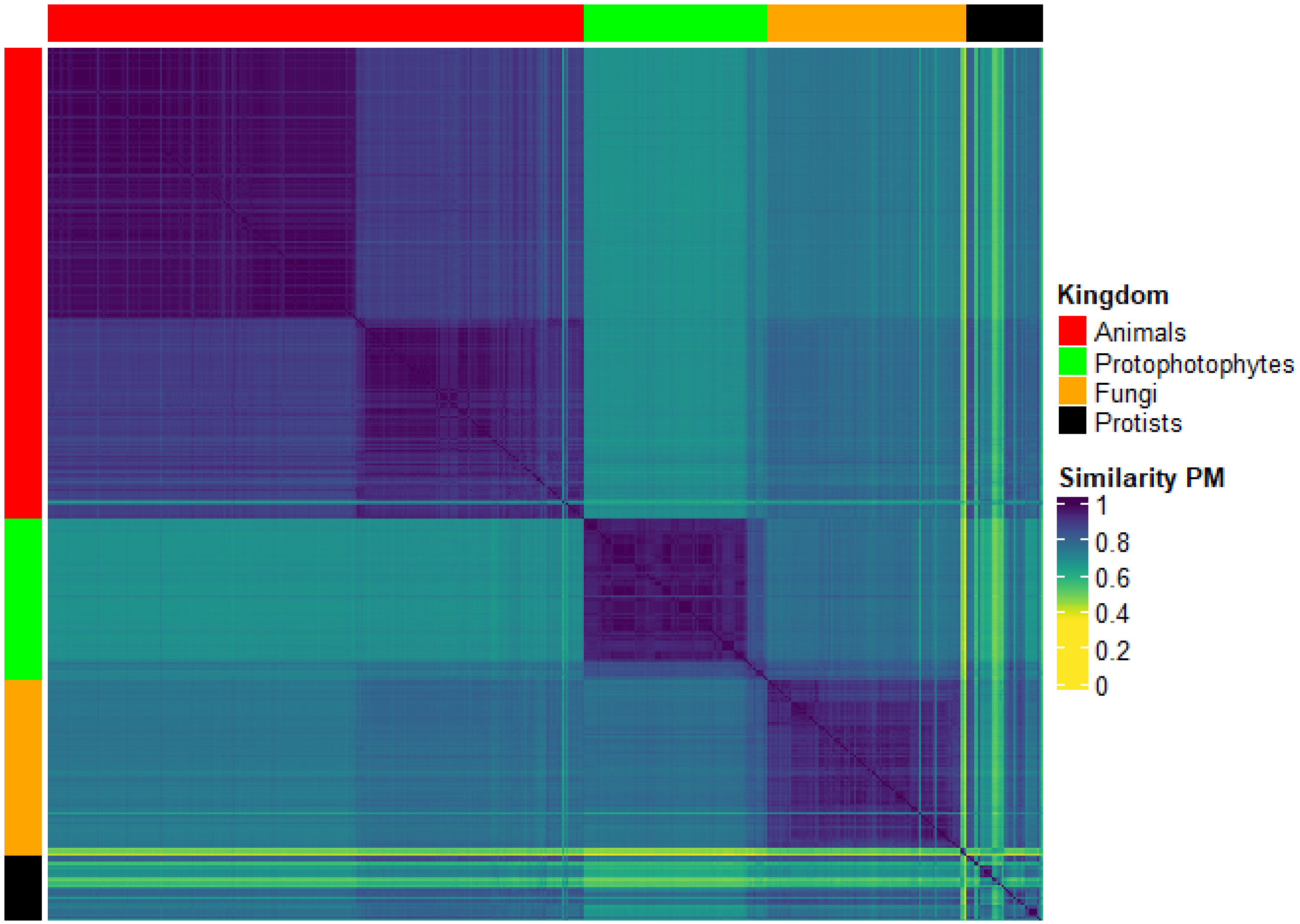

Kingdom
Animals
Protophotophytes
Fungi
Protists
Similarity PM
1
0.8
0.6
0.4
0.2
0

MDS for PM

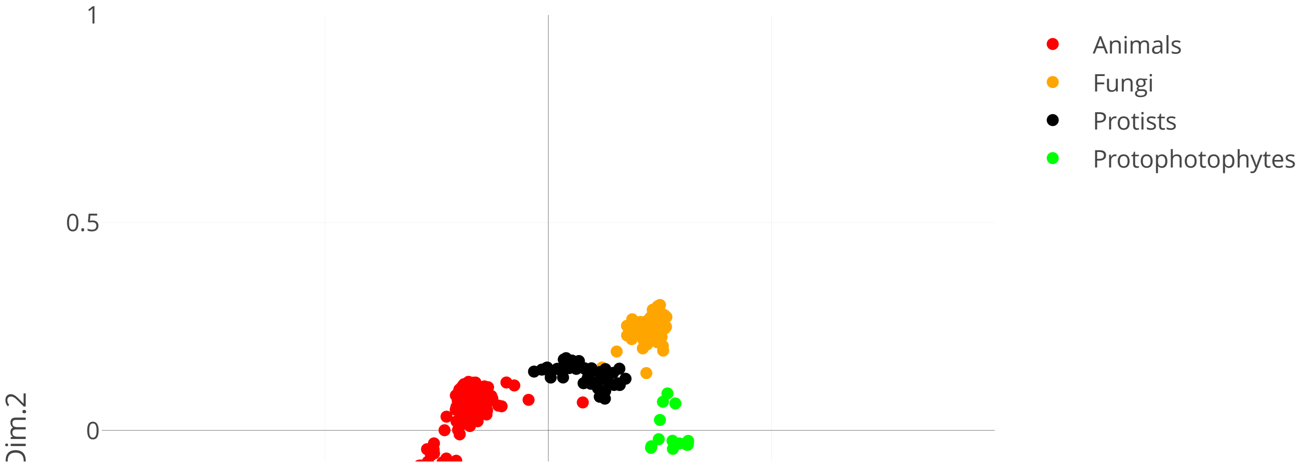

1
0.5
0
Dim.2
Animals
Fungi
Protists
Protophotophytes

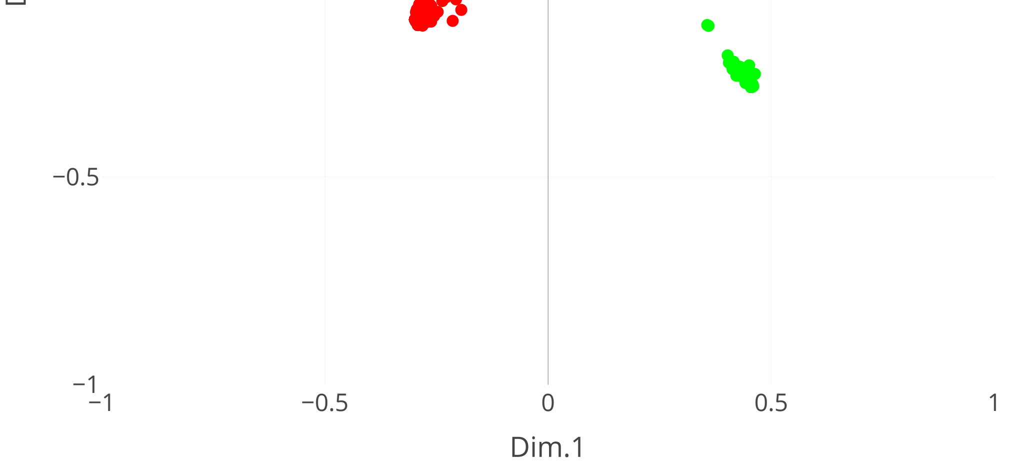

4-means for PM

```
##                       Cluster
## Real group              1   2   3   4
##   Animals               0   0 368   2
##   Fungi               132   0   0   6
##   Protists             26   0   1  25
##   Protophotophytes      9 118   0   0
```

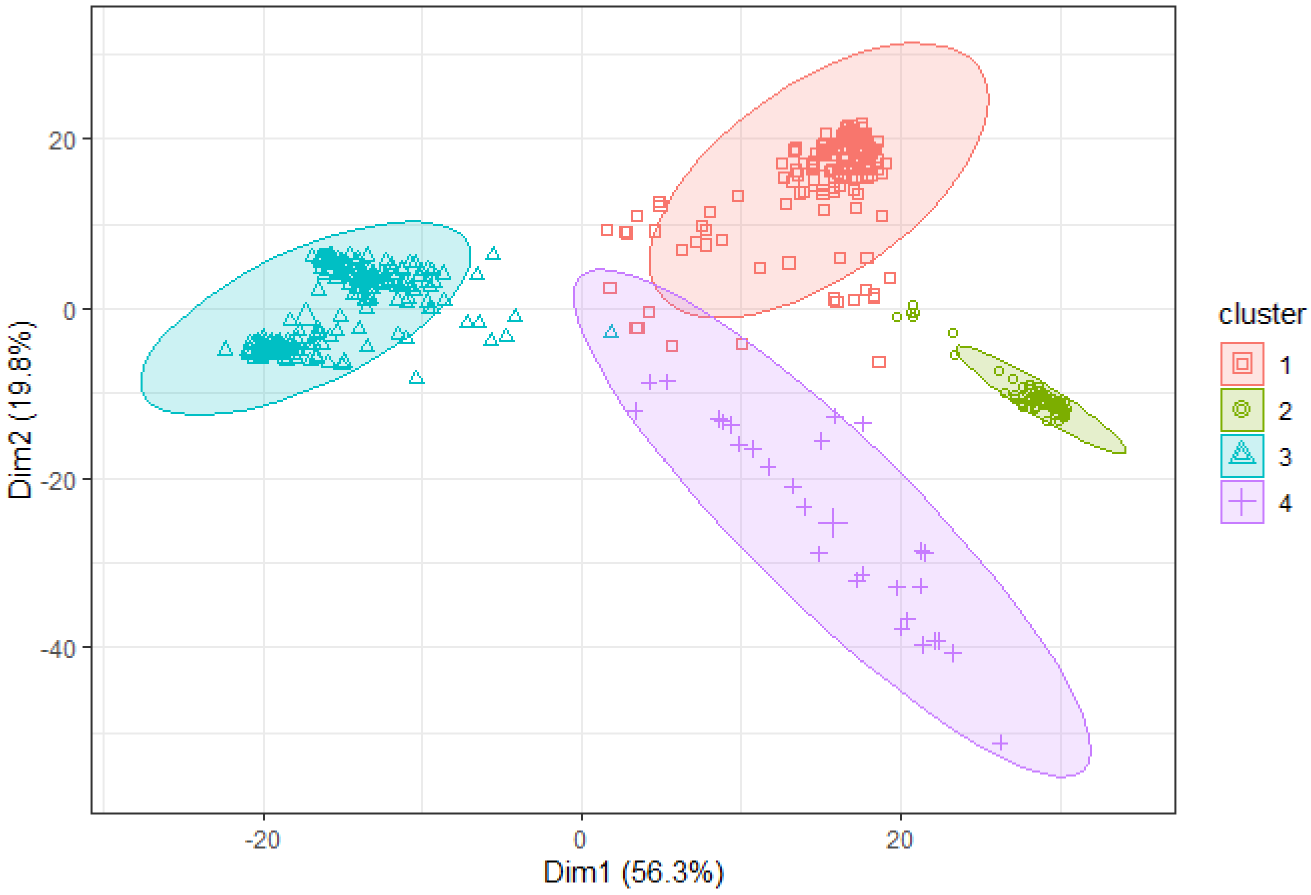

Organisms classified within cluster 1

```
##   [1] "cre"  "vcn"  "mng"  "csl"  "cvr"  "apro" "cme"  "gsl"  "ccp"  "sce"
##  [11] "ago"  "erc"  "kla"  "kmx"  "lth"  "vpo"  "zro"  "cgr"  "ncs"  "ndi"
##  [21] "tpf"  "tbl"  "tdl"  "tgb"  "kaf"  "zmk"  "ppa"  "dha"  "pic"  "pgu"
##  [31] "spaa" "lel"  "cal"  "ctp"  "cot"  "cdu"  "cten" "yli"  "clu"  "clus"
##  [41] "caur" "slb"  "pkz"  "bnn"  "bbrx" "ncr"  "nte"  "smp"  "pan"  "ttt"
##  [51] "mtm"  "cthr" "mgr"  "tmn"  "ssck" "fgr"  "fpu"  "fvr"  "fox"  "nhe"
##  [61] "tre"  "trr"  "maw"  "maj"  "cmt"  "plj"  "val"  "vda"  "cfj"  "sapo"
##  [71] "ela"  "pfy"  "ssl"  "bfu"  "mbe"  "psco" "glz"  "ani"  "afm"  "act"
```

```
##  [81] "nfi"  "aor"  "ang"  "afv"  "pcs"  "pdp"  "tmf"  "trg"  "cim"  "cpw" 
##  [91] "ure"  "pbl"  "pbn"  "abe"  "tve"  "aje"  "bgh"  "pno"  "pte"  "bze" 
## [101] "bsc"  "bor"  "aalt" "ztr"  "pfj"  "bcom" "npa"  "tml"  "spo"  "cne" 
## [111] "cnb"  "cgi"  "tms"  "tasa" "tvs"  "dsq"  "pco"  "shs"  "hir"  "psq" 
## [121] "adl"  "fme"  "gtr"  "lbc"  "mpr"  "mrr"  "cci"  "scm"  "abp"  "abv" 
## [131] "cput" "sla"  "wse"  "wic"  "uma"  "pfp"  "mgl"  "mrt"  "msym" "pgr" 
## [141] "mlr"  "mbr"  "sre"  "ddi"  "dpp"  "dfa"  "acan" "tgo"  "tet"  "ptm" 
## [151] "smin" "pti"  "fcy"  "tps"  "aaf"  "pif"  "psoj" "spar" "ehx"  "gtt" 
## [161] "lma"  "lif"  "ldo"  "lmi"  "lbz"  "lpan" "ngr"
```

Organisms classified within cluster 2

```
##   [1] "ath"  "aly"  "crb"  "csat" "eus"  "brp"  "bna"  "boe"  "rsz"  "thj" 
##  [11] "cpap" "cit"  "cic"  "pvy"  "minc" "tcc"  "gra"  "ghi"  "gab"  "dzi" 
##  [21] "egr"  "gmx"  "gsj"  "pvu"  "vra"  "var"  "vun"  "ccaj" "aprc" "mtr" 
##  [31] "cam"  "lja"  "adu"  "aip"  "ahf"  "lang" "fve"  "rcn"  "pper" "pmum"
##  [41] "pavi" "pdul" "mdm"  "pxb"  "zju"  "mnt"  "csv"  "cmo"  "bhj"  "mcha"
##  [51] "cmax" "cmos" "cpep" "rcu"  "jcu"  "hbr"  "mesc" "pop"  "peu"  "palz"
##  [61] "jre"  "qsu"  "qlo"  "twl"  "vvi"  "vri"  "sly"  "spen" "sot"  "cann"
##  [71] "nta"  "nsy"  "nto"  "nau"  "ini"  "itr"  "sind" "oeu"  "egt"  "sspl"
##  [81] "han"  "ecad" "lsv"  "ccav" "dcr"  "csin" "bvg"  "soe"  "cqi"  "nnu" 
##  [91] "ming" "psom" "ncol" "osa"  "dosa" "obr"  "bdi"  "ats"  "tdc"  "sbi" 
## [101] "zma"  "sita" "pvir" "phai" "pda"  "egu"  "mus"  "dct"  "peq"  "aof" 
## [111] "atr"  "smo"  "ppp"  "olu"  "ota"  "bpg"  "mis"  "mpp"
```

Organisms classified within cluster 3

```
##   [1] "hsa"  "ptr"  "pps"  "ggo"  "pon"  "nle"  "mcc"  "mcf"  "csab" "caty"
##  [11] "panu" "rro"  "rbb"  "tfn"  "pteh" "cjc"  "sbq"  "mmur" "mmu"  "mcal"
##  [21] "mpah" "rno"  "mcoc" "mun"  "cge"  "pleu" "ngi"  "hgl"  "ccan" "ocu" 
##  [31] "opi"  "tup"  "cfa"  "vvp"  "vlg"  "aml"  "umr"  "uah"  "oro"  "elk" 
##  [41] "mpuf" "eju"  "mlx"  "fca"  "pyu"  "pbg"  "ptg"  "ppad" "aju"  "hhv"
```

```
##  [51] "bta"  "bom"  "biu"  "bbub" "chx"  "oas"  "oda"  "ccad" "ssc"  "cfr"
##  [61] "cbai" "cdk"  "bacu" "lve"  "oor"  "dle"  "pcad" "ecb"  "epz"  "eai"
##  [71] "myb"  "myd"  "mmyo" "mna"  "pkl"  "hai"  "dro"  "shon" "ajm"  "pdic"
##  [81] "mmf"  "rfq"  "pale" "pgig" "ray"  "mjv"  "tod"  "lav"  "tmu"  "mdo"
##  [91] "gas"  "shr"  "pcw"  "oaa"  "gga"  "pcoc" "mgp"  "cjo"  "nmel" "apla"
## [101] "acyg" "tgu"  "lsr"  "scan" "pmoa" "otc"  "pruf" "gfr"  "fab"  "phi"
## [111] "pmaj" "ccae" "ccw"  "etl"  "fpg"  "fch"  "clv"  "egz"  "nni"  "acun"
## [121] "padl" "aam"  "arow" "npd"  "dne"  "asn"  "amj"  "cpoo" "ggn"  "pss"
## [131] "cmy"  "cpic" "tst"  "cabi" "acs"  "pvt"  "sund" "pbi"  "pmur" "tsr"
## [141] "pgut" "vko"  "pmua" "zvi"  "gja"  "xla"  "xtr"  "npr"  "dre"  "srx"
## [151] "sanh" "sgh"  "ccar" "caua" "ipu"  "phyp" "amex" "eee"  "tru"  "tng"
## [161] "lco"  "ncc"  "cgob" "ely"  "plep" "sluc" "ecra" "pflv" "gat"  "ppug"
## [171] "msam" "cud"  "mze"  "onl"  "oau"  "ola"  "oml"  "xma"  "xco"  "xhe"
## [181] "pret" "cvg"  "ctul" "nfu"  "kmr"  "alim" "aoce" "csem" "pov"  "ssen"
## [191] "lcf"  "sdu"  "slal" "xgl"  "hcq"  "bpec" "malb" "sasa" "otw"  "omy"
## [201] "salp" "snh"  "els"  "sfm"  "pki"  "aang" "loc"  "pspa" "arut" "lcm"
## [211] "cmk"  "rtp"  "bfo"  "bbel" "cin"  "sclv" "spu"  "aplc" "sko"  "dme"
## [221] "der"  "dse"  "dsi"  "dya"  "dan"  "dsr"  "dpo"  "dpe"  "dmn"  "dwi"
## [231] "dgr"  "dmo"  "daz"  "dnv"  "dhe"  "dvi"  "ccat" "bod"  "mde"  "scac"
## [241] "lcq"  "aga"  "acoz" "aara" "aag"  "aalb" "cqu"  "cpii" "ame"  "acer"
## [251] "bim"  "bbif" "bvk"  "bvan" "bter" "ccal" "obb"  "mgen" "nmea" "cgig"
## [261] "soc"  "mpha" "aec"  "acep" "pbar" "vem"  "hst"  "dqu"  "cfo"  "fex"
## [271] "lhu"  "pgc"  "obo"  "pcf"  "pfuc" "vps"  "nvi"  "csol" "tpre" "mdl"
## [281] "cglo" "fas"  "dam"  "ccin" "tca"  "dpa"  "atd"  "agb"  "ldc"  "nvl"
## [291] "apln" "ppyr" "otu"  "bmor" "bman" "msex" "dpl"  "bany" "pmac" "ppot"
## [301] "pxu"  "prap" "zce"  "haw"  "tnl"  "pxy"  "api"  "dnx"  "ags"  "rmd"
## [311] "btab" "dci"  "clec" "hhal" "nlu"  "phu"  "foc"  "zne"  "csec" "fcd"
## [321] "dpx"  "dmk"  "pvm"  "pja"  "hame" "hazt" "eaf"  "isc"  "dsv"  "rsan"
## [331] "rmp"  "vde"  "vja"  "tut"  "dpte" "cscu" "ptep" "sdm"  "cel"  "cbr"
## [341] "bmy"  "loa"  "nai"  "tsp"  "hro"  "lgi"  "pcan" "bgt"  "gae"  "crg"
## [351] "myi"  "pmax" "obi"  "osn"  "lak"  "smm"  "ovi"  "nve"  "epa"  "aten"
## [361] "adf"  "amil" "pdam" "spis" "dgt"  "hmg"  "tad"  "aqu"  "tcr"
```

Organisms classified within cluster 4

```
##  [1] "shx"  "egl"  "ppl"  "ecu"  "ein"  "ehe"  "ero"  "nce"  "ehi"  "edi"
## [11] "eiv"  "pfa"  "pfd"  "pfh"  "pyo"  "pcb"  "pbe"  "pkn"  "pvx"  "pcy"
## [21] "tan"  "tpv"  "tot"  "beq"  "bbo"  "bmic" "cpv"  "cho"  "ngd"  "tbr"
## [31] "tbg"  "tva"  "gla"
```
