# Supplementary material for: Exploring the expressiveness of abstract metabolic networks
Source: PLoS One. 2023 Feb 9;18(2):e0281047. doi: 10.1371/journal.pone.0281047 (PMC9910719; doi:10.1371/journal.pone.0281047)
Supplement: S6 File — Animals analyses at phylum level (second experiment). (PDF) [file pone.0281047.s006.pdf]

Animals Analysis

- Vertex hystogram (VH) kernel
  - Heatmap
  - MDS for VH
  - 14-Means clustering for VH Kernel
  - Optimal number of clusters for VH
- Shortest path (SP) kernel
  - Heatmap
  - MDS for SP
  - 14-Means clustering for SP Kernel
  - Optimal number of clusters for SP
- Weisfeiler-Lehman (WL) kernel
  - Heatmap
  - MDS for WL
  - 14-Means clustering for WL Kernel
  - Optimal number of clusters for WL
- Pyramid match (PM) kernel
  - Heatmap
  - MDS for PM
  - 14-Means clustering for PM Kernel
  - Optimal number of clusters for PM

Vertex hystogram (VH) kernel

Heatmap

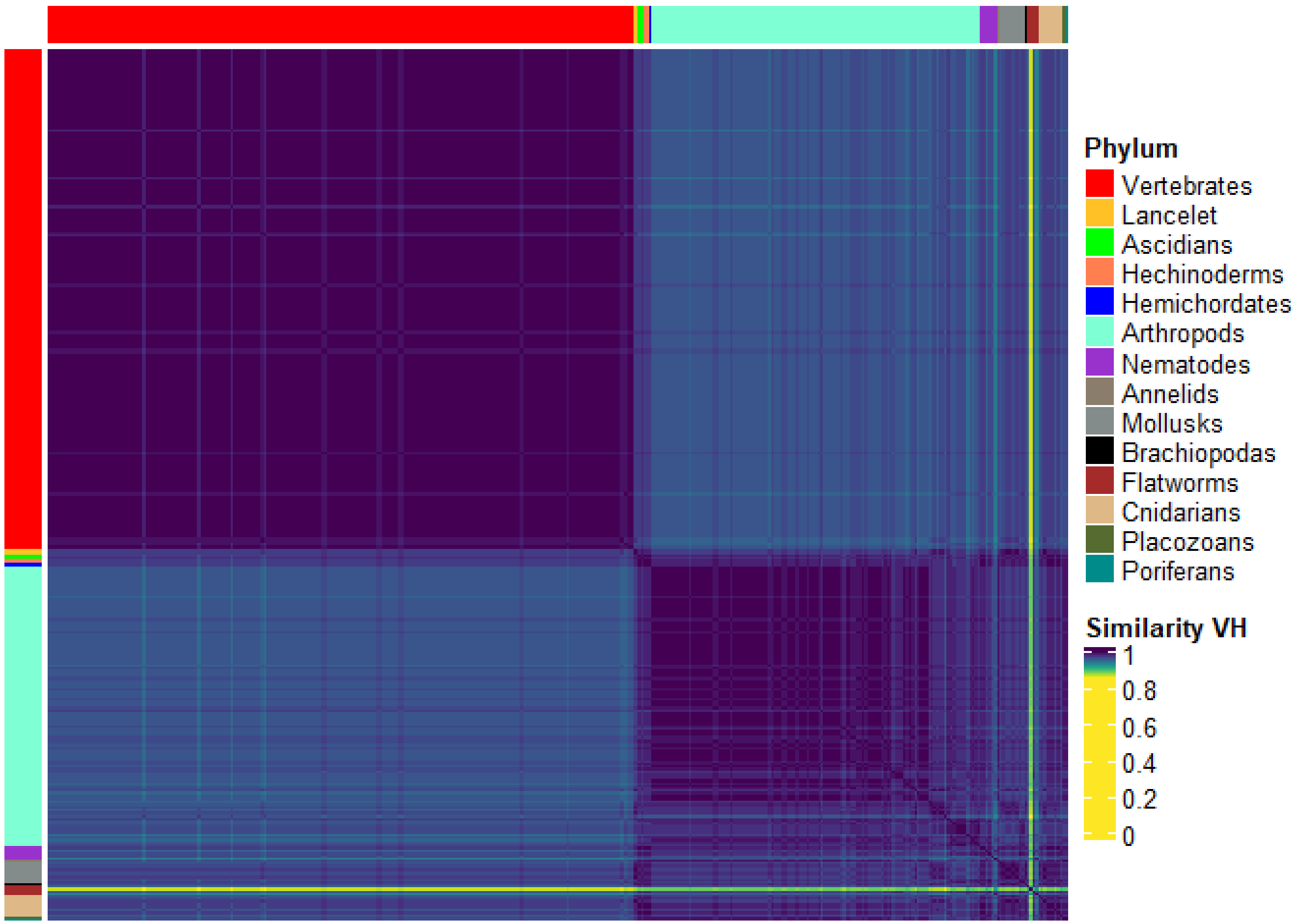

MDS for VH

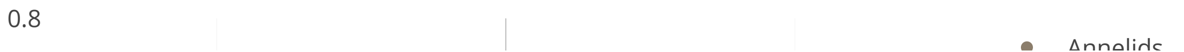

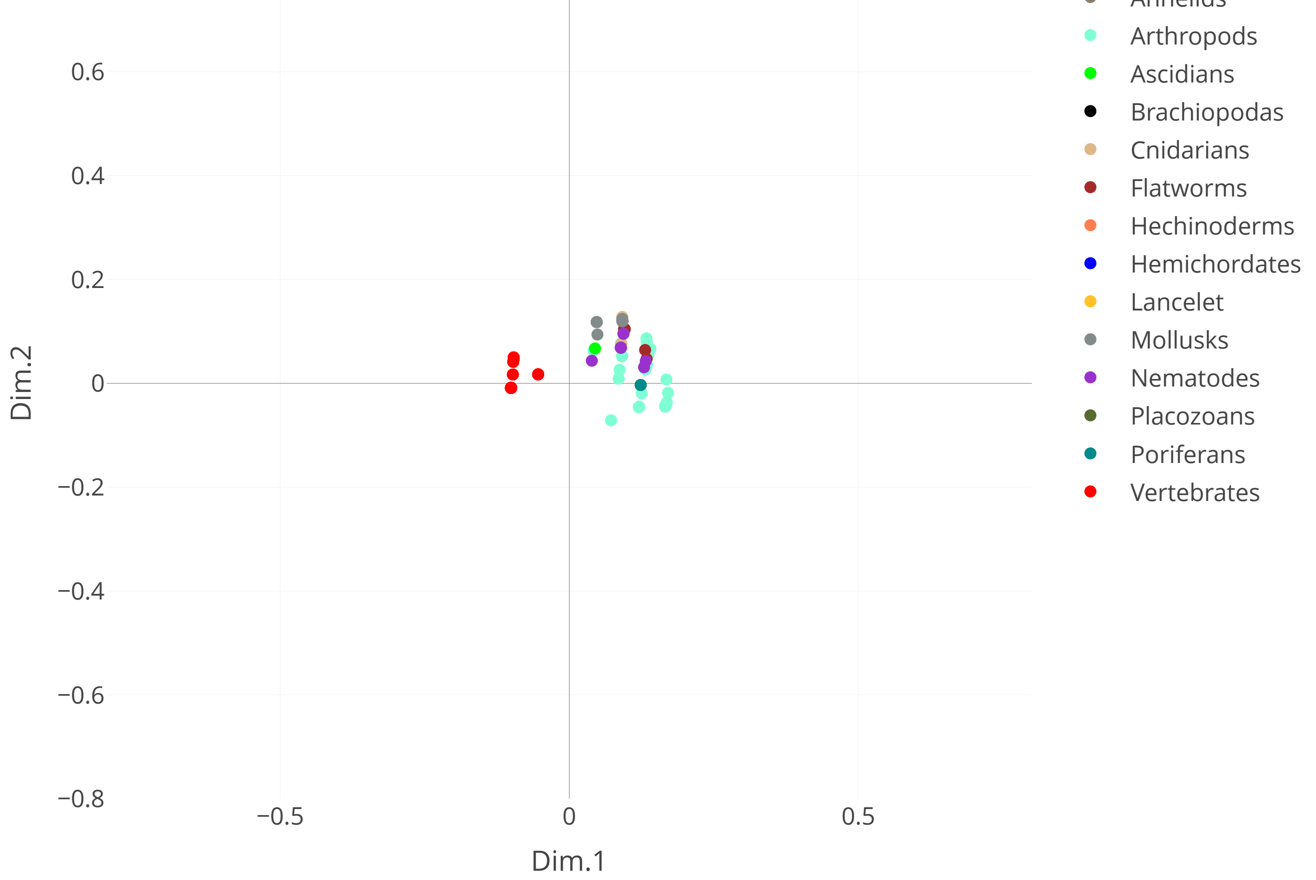

14-Means clustering for VH Kernel

```
##                  Cluster
## Real group         1   2   3   4   5   6   7   8   9  10  11  12  13  14
##   Annelids         0   0   0   0   0   0   0   0   1   0   0   0   0   0
##   Arthropods      70   6   0   2   3  14   0   5   7   0   0   0   4   8
##   Ascidians        0   0   0   0   0   0   0   0   0   0   1   0   1   0
##   Brachiopodas     0   0   0   0   1   0   0   0   0   0   0   0   0   0
##   Cnidarians       0   0   0   0   2   0   0   0   2   0   4   0   1   0
##   Flatworms        0   0   0   2   0   0   0   0   1   1   0   0   0   0
##   Hechinoderms     0   0   0   0   0   0   0   0   0   0   2   0   0   0
##   Hemichordates    0   0   0   0   0   0   0   0   0   0   1   0   0   0
##   Lancelet         0   0   0   0   0   0   0   0   0   0   0   0   2   0
##   Mollusks         0   1   0   0   4   0   0   0   1   0   0   0   3   0
```

```
##    Nematodes         0    0    0    3    0    0    0    0    1    0    2    0    0    0
##    Placozoans        0    0    0    0    0    0    0    0    1    0    0    0    0    0
##    Poriferans        0    0    0    0    0    0    0    0    1    0    0    0    0    0
##    Vertebrates       0    0    7    0    0    0    9    0    0    0    0  196    0    0
```

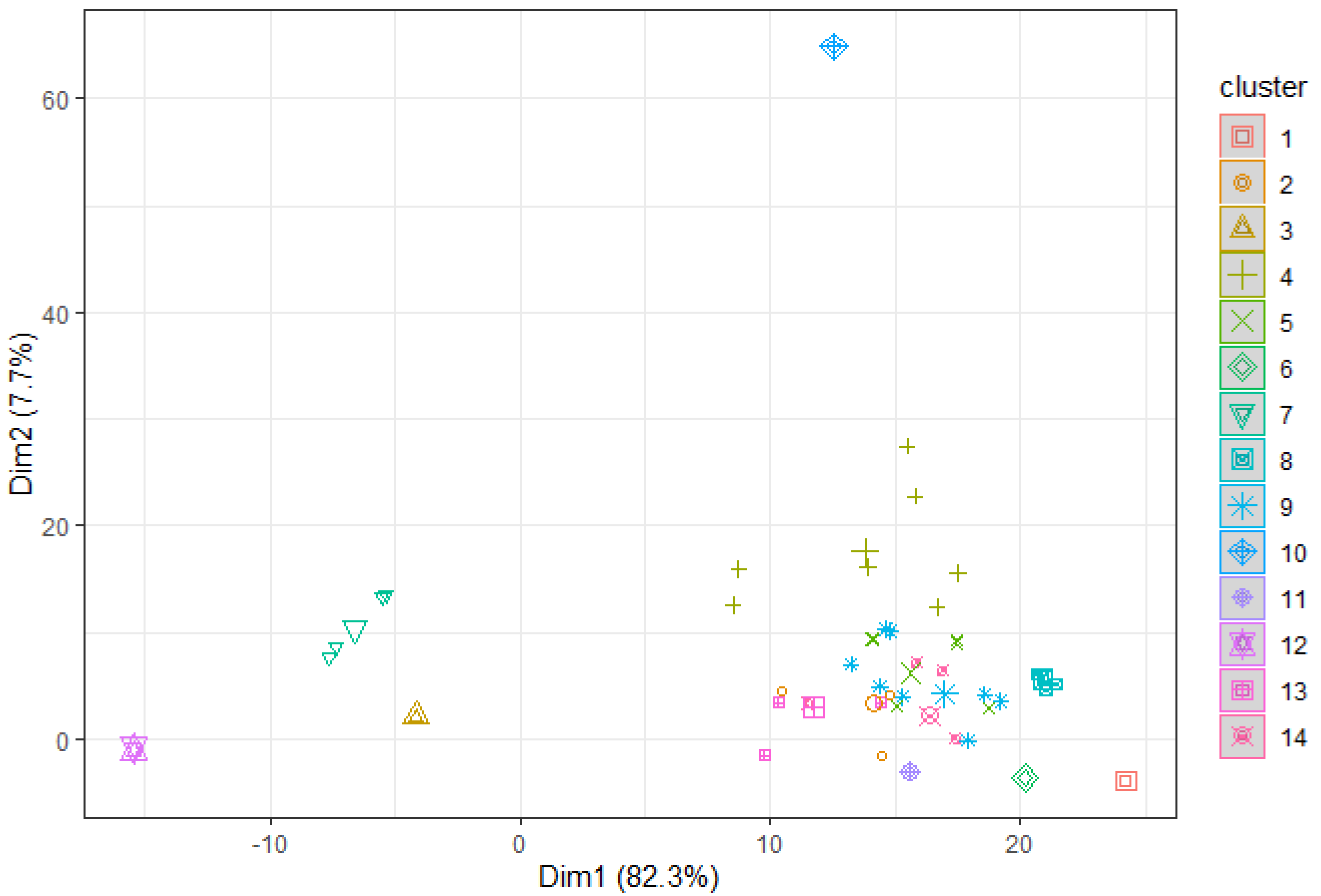

Organisms classified within cluster 1

```
##  [1] "dme"  "der"  "dse"  "dsi"  "dya"  "dan"  "dsr"  "dpo"  "dpe"  "dmn"
## [11] "dwi"  "dgr"  "dmo"  "dnv"  "dhe"  "dvi"  "ccat" "bod"  "mde"  "scac"
```

```
## [21] "lcq"  "aara" "aag"  "aalb" "cqu"  "ame"  "acer" "bim"  "bbif" "bvk"
## [31] "bvan" "bter" "ccal" "obb"  "mgen" "nmea" "cgig" "soc"  "acep" "pbar"
## [41] "vem"  "cfo"  "fex"  "lhu"  "obo"  "pcf"  "pfuc" "nvi"  "csol" "tpre"
## [51] "fas"  "dam"  "ccin" "tca"  "dpa"  "atd"  "nvl"  "apln" "msex" "bany"
## [61] "pmac" "ppot" "pxu"  "prap" "tnl"  "btab" "clec" "foc"  "zne"  "csec"
```

Organisms classified within cluster 2

```
## [1] "eaf"  "rsan" "rmp"  "vde"  "vja"  "sdm"  "osn"
```

Organisms classified within cluster 3

```
## [1] "apla" "acyg" "acun" "padl" "cpoo" "ggn"  "cud"
```

Organisms classified within cluster 4

```
## [1] "hazt" "tut"  "bmy"  "nai"  "tsp"  "smm"  "egl"
```

Organisms classified within cluster 5

```
##  [1] "fcd"  "dpx"  "pvm"  "lgi"  "pcan" "crg"  "myi"  "lak"  "nve"  "spis"
```

Organisms classified within cluster 6

```
##  [1] "aga"  "acoz" "cpii" "aec"  "hst"  "dqu"  "pgc"  "vps"  "mdl"  "bmor"
## [11] "bman" "dpl"  "haw"  "pxy"
```

Organisms classified within cluster 7

```
## [1] "vlg"  "chx"  "pcad" "shon" "ajm"  "pov"  "pspa" "arut" "lcm"
```

Organisms classified within cluster 8

```
## [1] "daz"  "mpha" "dci"  "phu"  "cscu"
```

Organisms classified within cluster 9

```
##  [1] "api"  "dnx"  "ags"  "rmd"  "isc"  "dpte" "ptep" "loa"  "hro"  "obi"
## [11] "ovi"  "epa"  "hmg"  "tad"  "aqu"
```

Organisms classified within cluster 10

```
## [1] "shx"
```

Organisms classified within cluster 11

```
##  [1] "cin"  "spu"  "aplc" "sko"  "cel"  "cbr"  "adf"  "amil" "pdam" "dgt"
```

Organisms classified within cluster 12

```
##   [1] "hsa"  "ptr"  "pps"  "ggo"  "pon"  "nle"  "mcc"  "mcf"  "csab" "caty"
##  [11] "panu" "rro"  "rbb"  "tfn"  "pteh" "cjc"  "sbq"  "mmur" "mmu"  "mcal"
##  [21] "mpah" "rno"  "mcoc" "mun"  "cge"  "pleu" "ngi"  "hgl"  "ccan" "ocu"
##  [31] "opi"  "tup"  "cfa"  "vvp"  "aml"  "umr"  "uah"  "oro"  "elk"  "mpuf"
##  [41] "eju"  "mlx"  "fca"  "pyu"  "pbg"  "ptg"  "ppad" "aju"  "hhv"  "bta"
##  [51] "bom"  "biu"  "bbub" "oas"  "oda"  "ccad" "ssc"  "cfr"  "cbai" "cdk"
##  [61] "bacu" "lve"  "oor"  "dle"  "ecb"  "epz"  "eai"  "myb"  "myd"  "mmyo"
##  [71] "mna"  "pkl"  "hai"  "dro"  "pdic" "mmf"  "rfq"  "pale" "pgig" "ray"
##  [81] "mjv"  "tod"  "lav"  "tmu"  "mdo"  "gas"  "shr"  "pcw"  "oaa"  "gga"
```

```
##  [91] "pcoc" "mgp"  "cjo"  "nmel" "tgu"  "lsr"  "scan" "pmoa" "otc"  "pruf"
## [101] "gfr"  "fab"  "phi"  "pmaj" "ccae" "ccw"  "etl"  "fpg"  "fch"  "clv"
## [111] "egz"  "nni"  "aam"  "arow" "npd"  "dne"  "asn"  "amj"  "pss"  "cmy"
## [121] "cpic" "tst"  "cabi" "acs"  "pvt"  "sund" "pbi"  "pmur" "tsr"  "pgut"
## [131] "vko"  "pmua" "zvi"  "gja"  "xla"  "xtr"  "npr"  "dre"  "srx"  "sanh"
## [141] "sgh"  "ccar" "caua" "ipu"  "phyp" "amex" "eee"  "tru"  "tng"  "lco"
## [151] "ncc"  "cgob" "ely"  "plep" "sluc" "ecra" "pflv" "gat"  "ppug" "msam"
## [161] "mze"  "onl"  "oau"  "ola"  "oml"  "xma"  "xco"  "xhe"  "pret" "cvg"
## [171] "ctul" "nfu"  "kmr"  "alim" "aoce" "csem" "ssen" "lcf"  "sdu"  "slal"
## [181] "xgl"  "hcq"  "bpec" "malb" "sasa" "otw"  "omy"  "salp" "snh"  "els"
## [191] "sfm"  "pki"  "aang" "loc"  "cmk"  "rtp"
```

Organisms classified within cluster 13

```
##  [1] "bfo"  "bbel" "sclv" "nlu"  "dmk"  "pja"  "hame" "bgt"  "gae"  "pmax"
## [11] "aten"
```

Organisms classified within cluster 14

```
## [1] "cglo" "agb"  "ldc"  "ppyr" "otu"  "zce"  "hhal" "dsv"
```

Optimal number of clusters for VH

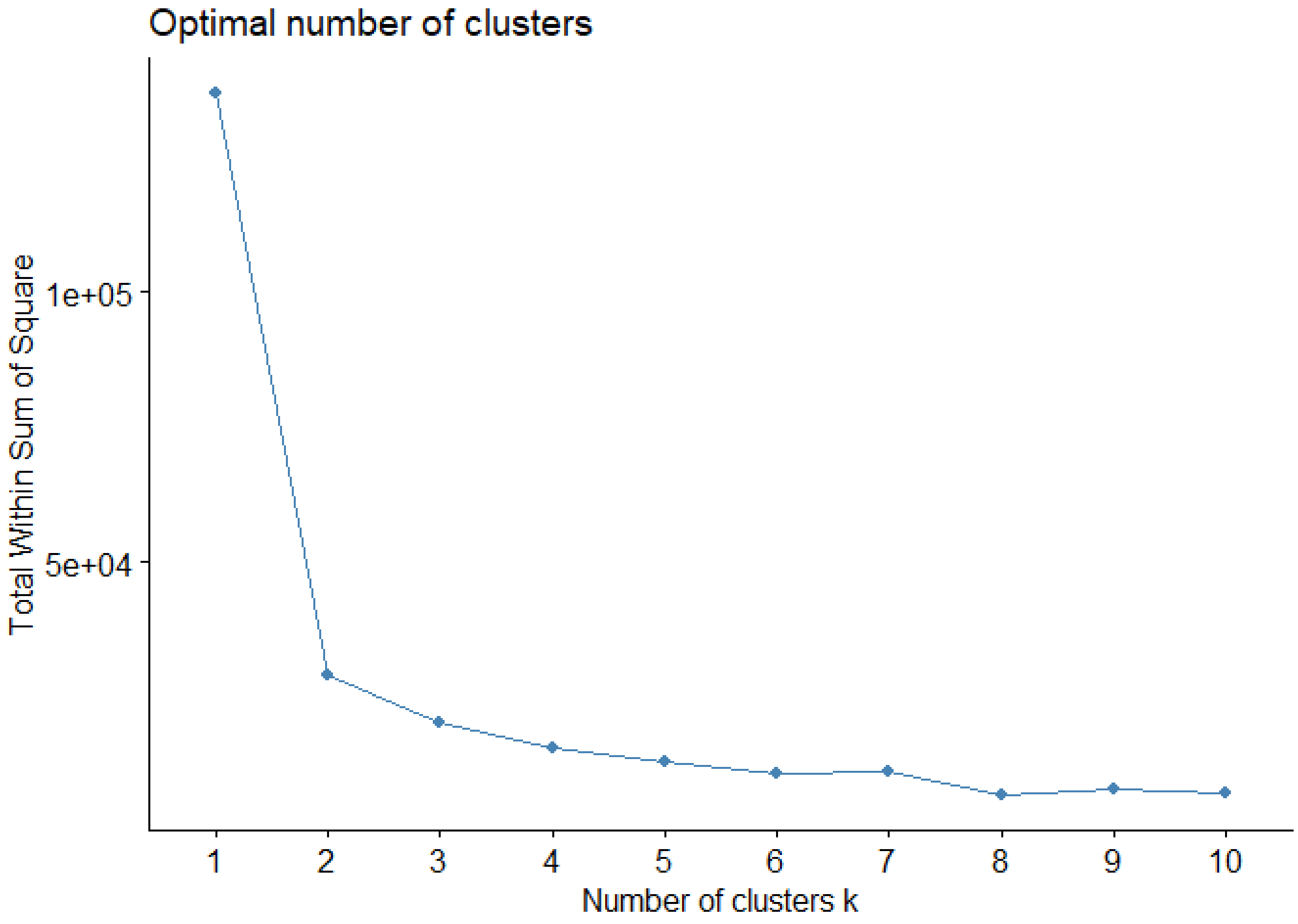


```
##                 Cluster
## Real group        1   2   3   4
##   Annelids        1   0   0   0
##   Arthropods     39  80   0   0
##   Ascidians       2   0   0   0
##   Brachiopodas    1   0   0   0
##   Cnidarians      9   0   0   0
##   Flatworms       2   0   0   2
##   Hechinoderms    2   0   0   0
##   Hemichordates   1   0   0   0
##   Lancelet        2   0   0   0
```

```
##    Mollusks         9    0    0    0
##    Nematodes        5    0    0    1
##    Placozoans       1    0    0    0
##    Poriferans       1    0    0    0
##    Vertebrates      0    0  212    0
```

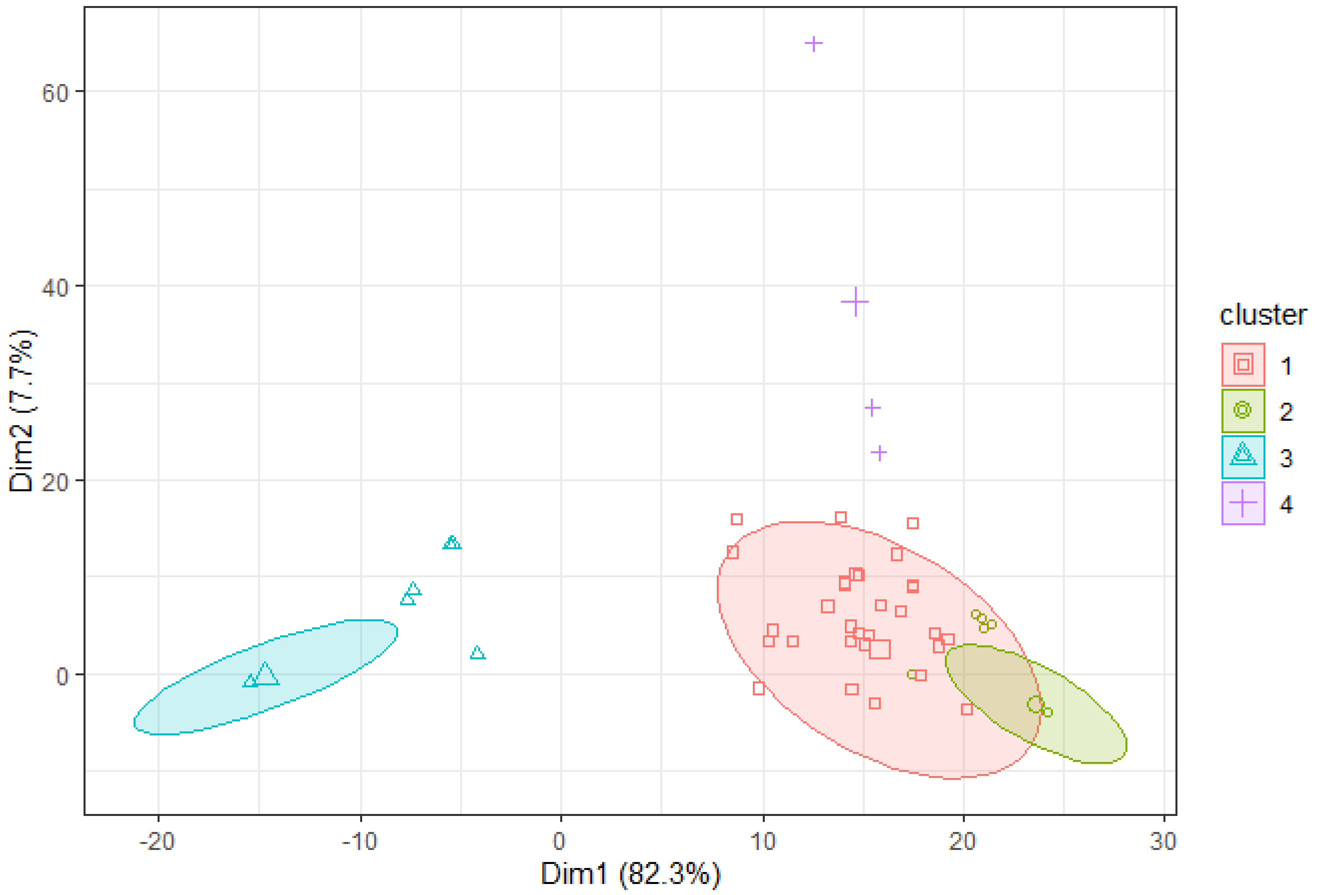

Organisms classified within cluster 1

```
##  [1] "bfo"  "bbel" "cin"  "sclv" "spu"  "aplc" "sko"  "aga"  "acoz" "cpii"
## [11] "aec"  "hst"  "dqu"  "pgc"  "vps"  "mdl"  "cglo" "ldc"  "bmor" "bman"
## [21] "dpl"  "haw"  "pxy"  "api"  "dnx"  "ags"  "rmd"  "nlu"  "fcd"  "dpx"
## [31] "dmk"  "pvm"  "pja"  "hame" "hazt" "eaf"  "isc"  "dsv"  "rsan" "rmp"
## [41] "vde"  "vja"  "tut"  "dpte" "ptep" "sdm"  "cel"  "cbr"  "bmy"  "loa"
## [51] "nai"  "hro"  "lgi"  "pcan" "bgt"  "gae"  "crg"  "myi"  "pmax" "obi"
## [61] "osn"  "lak"  "smm"  "ovi"  "nve"  "epa"  "aten" "adf"  "amil" "pdam"
## [71] "spis" "dgt"  "hmg"  "tad"  "aqu"
```

Organisms classified within cluster 2

```
##  [1] "dme"  "der"  "dse"  "dsi"  "dya"  "dan"  "dsr"  "dpo"  "dpe"  "dmn"
## [11] "dwi"  "dgr"  "dmo"  "daz"  "dnv"  "dhe"  "dvi"  "ccat" "bod"  "mde"
## [21] "scac" "lcq"  "aara" "aag"  "aalb" "cqu"  "ame"  "acer" "bim"  "bbif"
## [31] "bvk"  "bvan" "bter" "ccal" "obb"  "mgen" "nmea" "cgig" "soc"  "mpha"
## [41] "acep" "pbar" "vem"  "cfo"  "fex"  "lhu"  "obo"  "pcf"  "pfuc" "nvi"
## [51] "csol" "tpre" "fas"  "dam"  "ccin" "tca"  "dpa"  "atd"  "agb"  "nvl"
## [61] "apln" "ppyr" "otu"  "msex" "bany" "pmac" "ppot" "pxu"  "prap" "zce"
## [71] "tnl"  "btab" "dci"  "clec" "hhal" "phu"  "foc"  "zne"  "csec" "cscu"
```

Organisms classified within cluster 3

```
##   [1] "hsa"  "ptr"  "pps"  "ggo"  "pon"  "nle"  "mcc"  "mcf"  "csab" "caty"
##  [11] "panu" "rro"  "rbb"  "tfn"  "pteh" "cjc"  "sbq"  "mmur" "mmu"  "mcal"
##  [21] "mpah" "rno"  "mcoc" "mun"  "cge"  "pleu" "ngi"  "hgl"  "ccan" "ocu"
##  [31] "opi"  "tup"  "cfa"  "vvp"  "vlg"  "aml"  "umr"  "uah"  "oro"  "elk"
##  [41] "mpuf" "eju"  "mlx"  "fca"  "pyu"  "pbg"  "ptg"  "ppad" "aju"  "hhv"
##  [51] "bta"  "bom"  "biu"  "bbub" "chx"  "oas"  "oda"  "ccad" "ssc"  "cfr"
##  [61] "cbai" "cdk"  "bacu" "lve"  "oor"  "dle"  "pcad" "ecb"  "epz"  "eai"
##  [71] "myb"  "myd"  "mmyo" "mna"  "pkl"  "hai"  "dro"  "shon" "ajm"  "pdic"
##  [81] "mmf"  "rfq"  "pale" "pgig" "ray"  "mjv"  "tod"  "lav"  "tmu"  "mdo"
##  [91] "gas"  "shr"  "pcw"  "oaa"  "gga"  "pcoc" "mgp"  "cjo"  "nmel" "apla"
```

```
## [101] "acyg" "tgu"  "lsr"  "scan" "pmoa" "otc"  "pruf" "gfr"  "fab"  "phi"
## [111] "pmaj" "ccae" "ccw"  "etl"  "fpg"  "fch"  "clv"  "egz"  "nni"  "acun"
## [121] "padl" "aam"  "arow" "npd"  "dne"  "asn"  "amj"  "cpoo" "ggn"  "pss"
## [131] "cmy"  "cpic" "tst"  "cabi" "acs"  "pvt"  "sund" "pbi"  "pmur" "tsr"
## [141] "pgut" "vko"  "pmua" "zvi"  "gja"  "xla"  "xtr"  "npr"  "dre"  "srx"
## [151] "sanh" "sgh"  "ccar" "caua" "ipu"  "phyp" "amex" "eee"  "tru"  "tng"
## [161] "lco"  "ncc"  "cgob" "ely"  "plep" "sluc" "ecra" "pflv" "gat"  "ppug"
## [171] "msam" "cud"  "mze"  "onl"  "oau"  "ola"  "oml"  "xma"  "xco"  "xhe"
## [181] "pret" "cvg"  "ctul" "nfu"  "kmr"  "alim" "aoce" "csem" "pov"  "ssen"
## [191] "lcf"  "sdu"  "slal" "xgl"  "hcq"  "bpec" "malb" "sasa" "otw"  "omy"
## [201] "salp" "snh"  "els"  "sfm"  "pki"  "aang" "loc"  "pspa" "arut" "lcm"
## [211] "cmk"  "rtp"
```

Organisms classified within cluster 4

```
## [1] "tsp" "shx" "egl"
```

Shortest path (SP) kernel

Heatmap

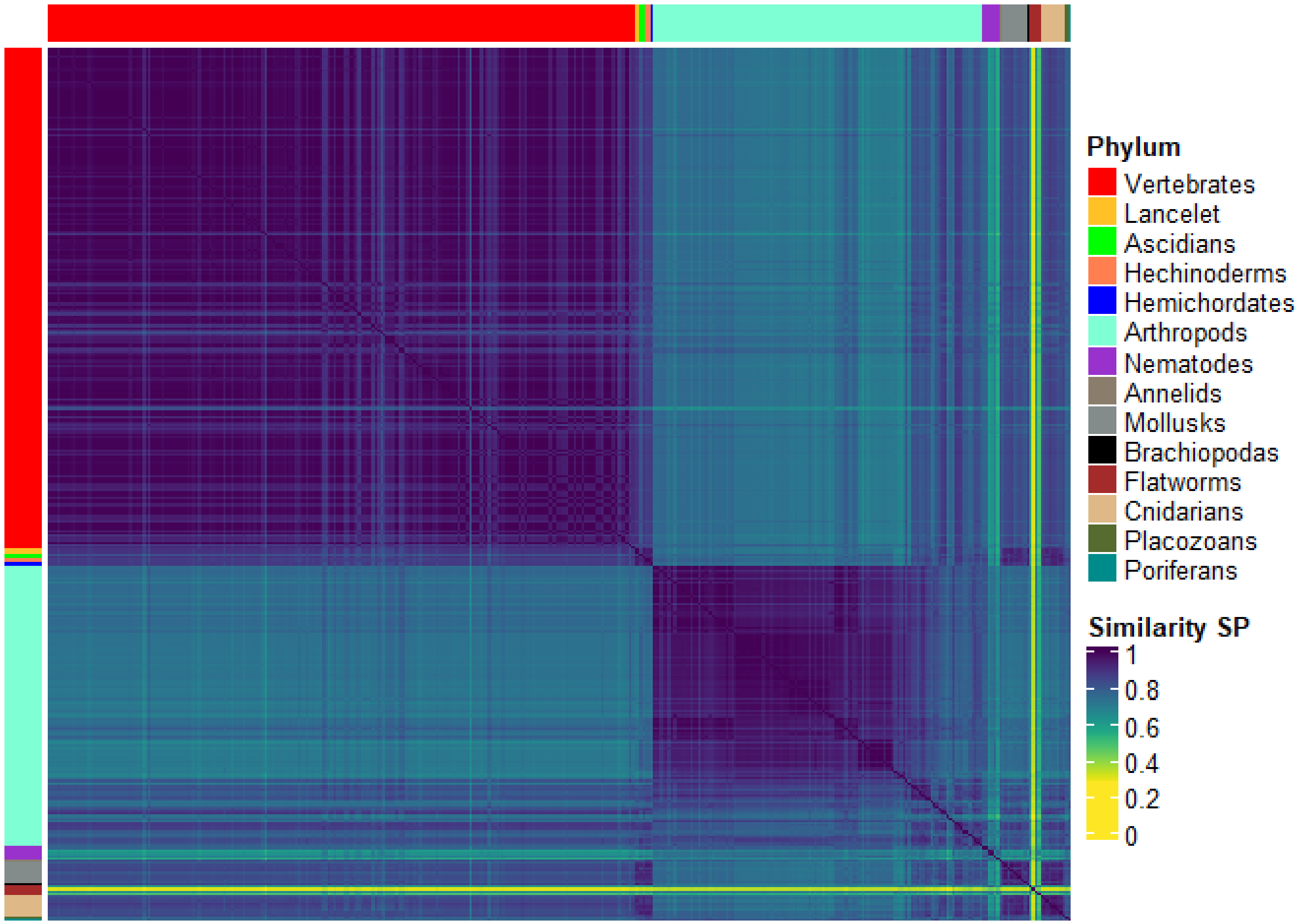
Phylum
Vertebrates
Lancelet
Ascidians
Hechinoderms
Hemichordates
Arthropods
Nematodes
Annelids
Mollusks
Brachiopodas
Flatworms
Cnidarians
Placozoans
Poriferans
Similarity SP
1
0.8
0.6
0.4
0.2
0

MDS for SP

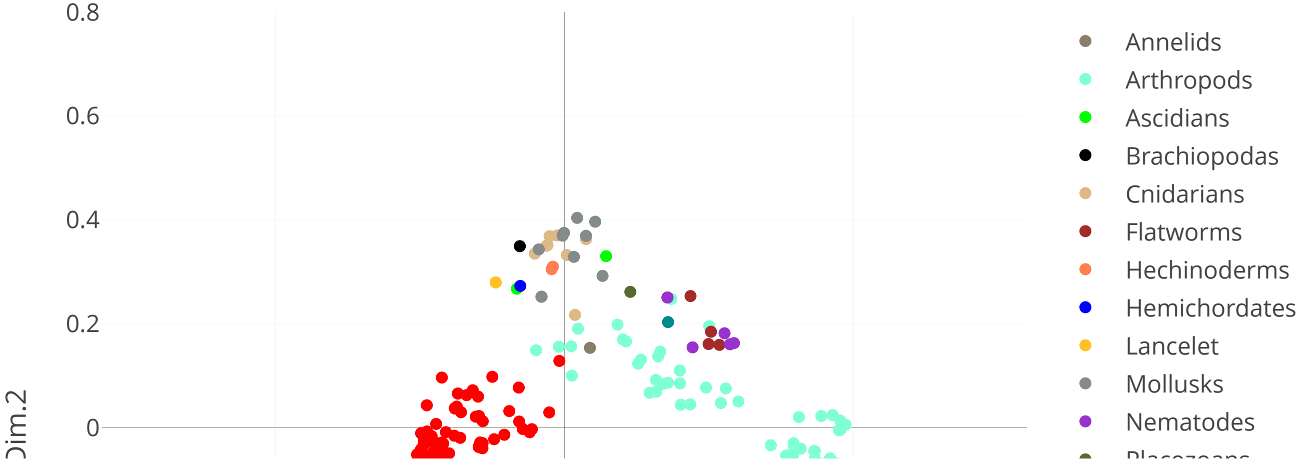
0.8
0.6
0.4
0.2
0
Dim.2
Annelids
Arthropods
Ascidians
Brachiopodas
Cnidarians
Flatworms
Hechinoderms
Hemichordates
Lancelet
Mollusks
Nematodes

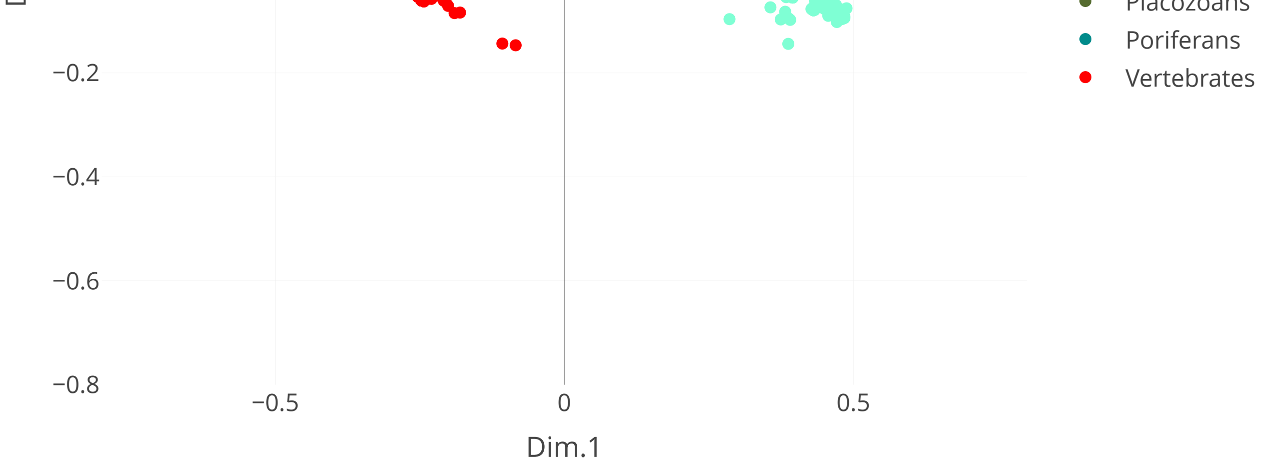

14-Means clustering for SP Kernel

```
##                  Cluster
## Real group         1   2   3   4   5   6   7   8   9  10  11  12  13  14
##   Annelids         0   0   0   0   0   0   1   0   0   0   0   0   0   0
##   Arthropods       0   0   0   0   0   1   1  16   5   0  44  19   0  33
##   Ascidians        0   0   0   0   0   2   0   0   0   0   0   0   0   0
##   Brachiopodas     0   0   0   0   0   1   0   0   0   0   0   0   0   0
##   Cnidarians       0   0   0   0   0   8   0   0   0   0   0   1   0   0
##   Flatworms        0   0   0   2   0   0   0   0   0   0   0   0   2   0
##   Hechinoderms     0   0   0   0   0   2   0   0   0   0   0   0   0   0
##   Hemichordates    0   0   0   0   0   1   0   0   0   0   0   0   0   0
##   Lancelet         0   0   0   0   0   2   0   0   0   0   0   0   0   0
##   Mollusks         0   0   0   0   0   9   0   0   0   0   0   0   0   0
##   Nematodes        0   0   0   4   0   0   2   0   0   0   0   0   0   0
##   Placozoans       0   0   0   0   0   0   1   0   0   0   0   0   0   0
##   Poriferans       0   0   0   0   0   0   1   0   0   0   0   0   0   0
##   Vertebrates      8  21 132   0  16   0   0   0   0  35   0   0   0   0
```

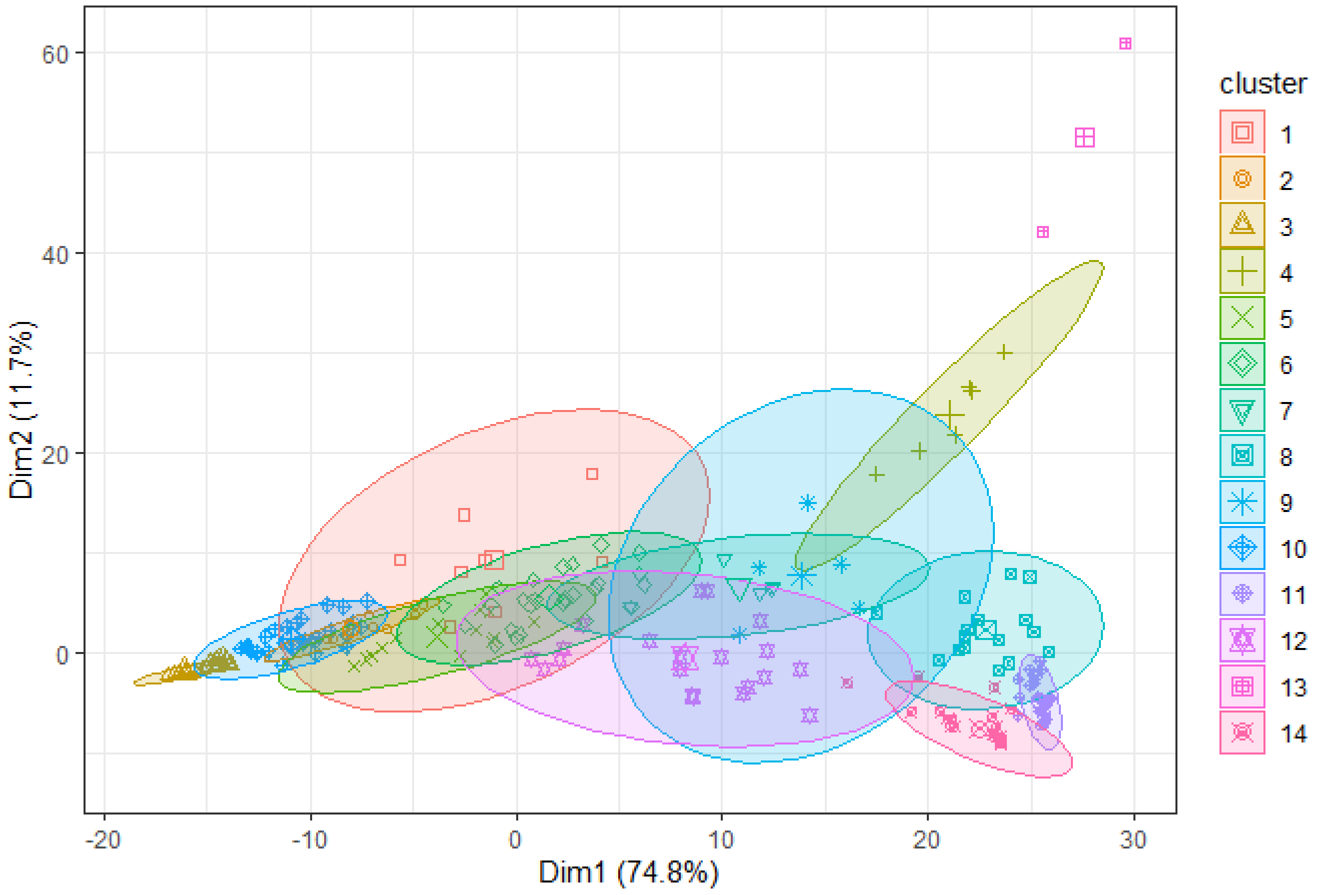

Organisms classified within cluster 1

```
## [1] "vlg"  "umr"  "ajm"  "padl" "aam"  "ccar" "tng"  "loc"
```

Organisms classified within cluster 2

```
##  [1] "dre"  "sgh"  "amex" "tru"  "lco"  "ncc"  "msam" "mze"  "cvg"  "kmr"
## [11] "alim" "aoce" "csem" "lcf"  "xgl"  "otw"  "omy"  "salp" "pki"  "pspa"
## [21] "arut"
```

Organisms classified within cluster 3

```
##   [1] "hsa"  "ptr"  "pps"  "pon"  "nle"  "mcc"  "mcf"  "csab" "panu" "rro" 
##  [11] "rbb"  "tfn"  "cjc"  "sbq"  "mmur" "mmu"  "mcal" "mpah" "rno"  "mcoc"
##  [21] "mun"  "cge"  "pleu" "ngi"  "hgl"  "ccan" "opi"  "tup"  "cfa"  "vvp" 
##  [31] "aml"  "uah"  "oro"  "elk"  "mpuf" "mlx"  "fca"  "pyu"  "pbg"  "ptg" 
##  [41] "ppad" "aju"  "hhv"  "bta"  "bom"  "bbub" "oas"  "oda"  "ccad" "cfr" 
##  [51] "cbai" "cdk"  "bacu" "oor"  "dle"  "ecb"  "epz"  "eai"  "myd"  "mna" 
##  [61] "hai"  "dro"  "pdic" "mmf"  "pale" "pgig" "ray"  "tod"  "lav"  "tmu" 
##  [71] "pcw"  "gga"  "pcoc" "mgp"  "cjo"  "lsr"  "pmoa" "otc"  "pruf" "phi" 
##  [81] "fch"  "clv"  "asn"  "amj"  "cmy"  "cpic" "cabi" "pvt"  "sund" "pbi" 
##  [91] "pmur" "vko"  "pmua" "zvi"  "gja"  "xla"  "xtr"  "npr"  "srx"  "sanh"
## [101] "caua" "ipu"  "phyp" "eee"  "ely"  "sluc" "ecra" "pflv" "gat"  "ppug"
## [111] "onl"  "oau"  "oml"  "xma"  "xco"  "xhe"  "pret" "ctul" "nfu"  "pov" 
## [121] "ssen" "sdu"  "slal" "hcq"  "bpec" "malb" "sasa" "snh"  "els"  "sfm" 
## [131] "aang" "lcm"
```

Organisms classified within cluster 4

```
## [1] "bmy" "loa" "nai" "tsp" "smm" "ovi"
```

Organisms classified within cluster 5

```
##  [1] "apla" "acyg" "scan" "gfr"  "fab"  "ccae" "ccw"  "egz"  "acun" "arow"
## [11] "npd"  "dne"  "cpoo" "ggn"  "cmk"  "rtp"
```

Organisms classified within cluster 6

```
##  [1] "bfo"  "bbel" "cin"  "sclv" "spu"  "aplc" "sko"  "hame" "lgi"  "pcan"
## [11] "bgt"  "gae"  "crg"  "myi"  "pmax" "obi"  "osn"  "lak"  "nve"  "epa"
```

```
## [21] "aten" "adf"  "amil" "pdam" "spis" "dgt"
```

Organisms classified within cluster 7

```
## [1] "tut" "cel" "cbr" "hro" "tad" "aqu"
```

Organisms classified within cluster 8

```
##  [1] "dan"  "daz"  "dvi"  "vps"  "cglo" "dam"  "bman" "dpl"  "zce"  "pxy"
## [11] "api"  "dnx"  "ags"  "rmd"  "dci"  "isc"
```

Organisms classified within cluster 9

```
## [1] "fcd"  "dpx"  "dmk"  "hazt" "eaf"
```

Organisms classified within cluster 10

```
##  [1] "ggo"  "caty" "pteh" "ocu"  "eju"  "biu"  "chx"  "ssc"  "lve"  "pcad"
## [11] "myb"  "mmyo" "pkl"  "shon" "rfq"  "mjv"  "mdo"  "gas"  "shr"  "oaa"
## [21] "nmel" "tgu"  "pmaj" "etl"  "fpg"  "nni"  "pss"  "tst"  "acs"  "tsr"
## [31] "pgut" "cgob" "plep" "cud"  "ola"
```

Organisms classified within cluster 11

```
##  [1] "dpo"  "aalb" "ame"  "acer" "bim"  "bbif" "bvk"  "bvan" "bter" "ccal"
## [11] "obb"  "mgen" "nmea" "cgig" "soc"  "mpha" "aec"  "acep" "pbar" "vem"
## [21] "hst"  "dqu"  "cfo"  "fex"  "lhu"  "pgc"  "obo"  "pcf"  "pfuc" "nvi"
## [31] "csol" "tpre" "mdl"  "fas"  "ccin" "bmor" "msex" "bany" "pmac" "ppot"
## [41] "pxu"  "prap" "haw"  "tnl"
```

Organisms classified within cluster 12

```
##  [1] "btab" "clec" "hhal" "nlu"  "phu"  "foc"  "zne"  "csec" "pvm"  "pja"
## [11] "dsv"  "rsan" "rmp"  "vde"  "vja"  "dpte" "cscu" "ptep" "sdm"  "hmg"
```

Organisms classified within cluster 13

```
## [1] "shx" "egl"
```

Organisms classified within cluster 14

```
##  [1] "dme"  "der"  "dse"  "dsi"  "dya"  "dsr"  "dpe"  "dmn"  "dwi"  "dgr"
## [11] "dmo"  "dnv"  "dhe"  "ccat" "bod"  "mde"  "scac" "lcq"  "aga"  "acoz"
## [21] "aara" "aag"  "cqu"  "cpii" "tca"  "dpa"  "atd"  "agb"  "ldc"  "nvl"
## [31] "apln" "ppyr" "otu"
```

Optimal number of clusters for SP

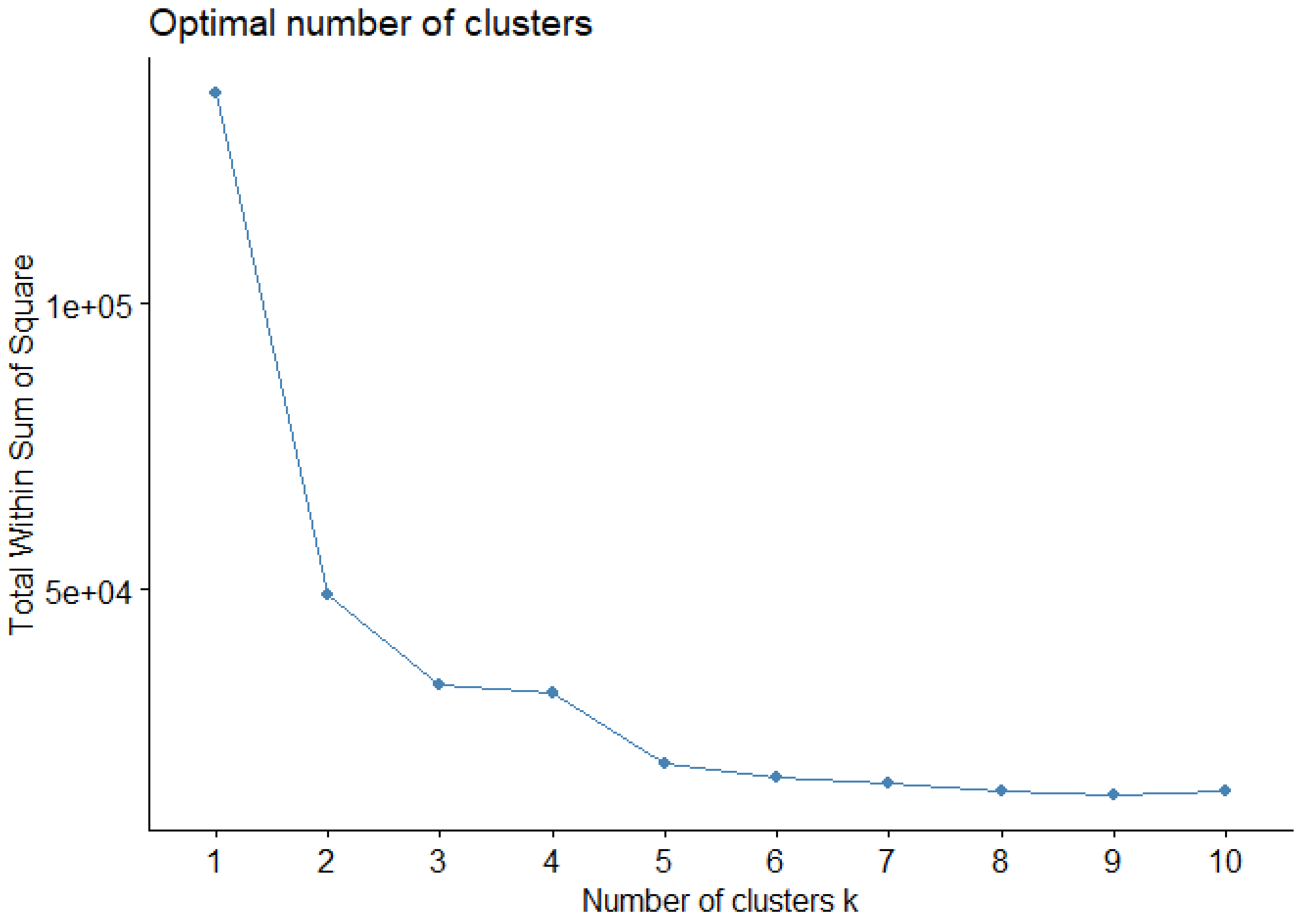


```
##                 Cluster
## Real group        1   2   3   4
##   Annelids        0   1   0   0
##   Arthropods     95  24   0   0
##   Ascidians       0   2   0   0
##   Brachiopodas    0   1   0   0
##   Cnidarians      0   9   0   0
##   Flatworms       0   0   4   0
##   Hechinoderms    0   2   0   0
##   Hemichordates   0   1   0   0
##   Lancelet        0   2   0   0
```

```
##    Mollusks         0   9   0   0
##    Nematodes        0   2   4   0
##    Placozoans       0   1   0   0
##    Poriferans       0   1   0   0
##    Vertebrates      0  17   0 195
```

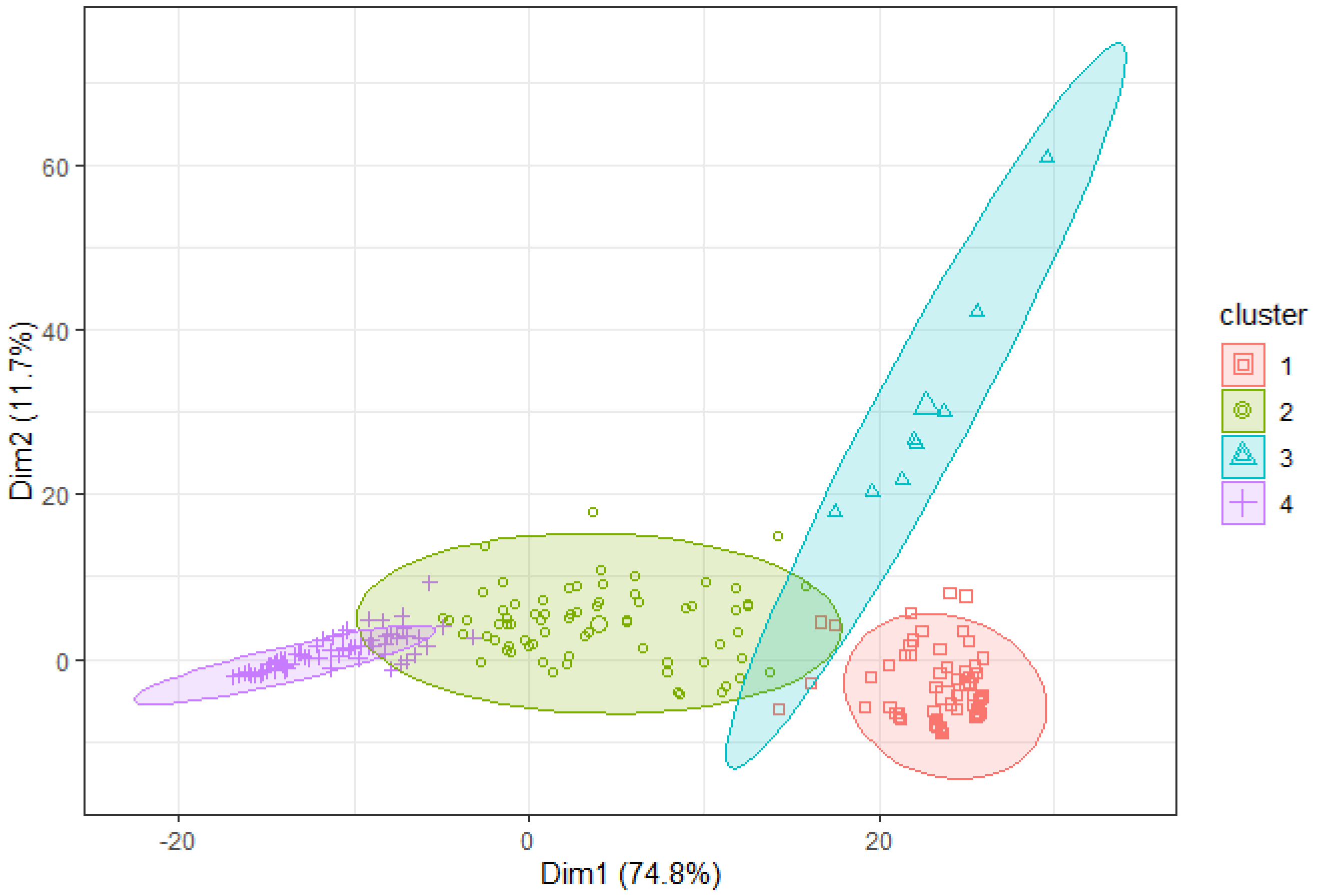

Organisms classified within cluster 1

```
##  [1] "dme"  "der"  "dse"  "dsi"  "dya"  "dan"  "dsr"  "dpo"  "dpe"  "dmn"
## [11] "dwi"  "dgr"  "dmo"  "daz"  "dnv"  "dhe"  "dvi"  "ccat" "bod"  "mde"
## [21] "scac" "lcq"  "aga"  "acoz" "aara" "aag"  "aalb" "cqu"  "cpii" "ame"
## [31] "acer" "bim"  "bbif" "bvk"  "bvan" "bter" "ccal" "obb"  "mgen" "nmea"
## [41] "cgig" "soc"  "mpha" "aec"  "acep" "pbar" "vem"  "hst"  "dqu"  "cfo"
## [51] "fex"  "lhu"  "pgc"  "obo"  "pcf"  "pfuc" "vps"  "nvi"  "csol" "tpre"
## [61] "mdl"  "cglo" "fas"  "dam"  "ccin" "tca"  "dpa"  "atd"  "agb"  "ldc"
## [71] "nvl"  "apln" "ppyr" "otu"  "bmor" "bman" "msex" "dpl"  "bany" "pmac"
## [81] "ppot" "pxu"  "prap" "zce"  "haw"  "tnl"  "pxy"  "api"  "dnx"  "ags"
## [91] "rmd"  "dci"  "foc"  "dpx"  "isc"
```

Organisms classified within cluster 2

```
##  [1] "ajm"  "apla" "acyg" "ccw"  "egz"  "acun" "padl" "aam"  "cpoo" "ggn"
## [11] "ccar" "amex" "tng"  "ncc"  "loc"  "cmk"  "rtp"  "bfo"  "bbel" "cin"
## [21] "sclv" "spu"  "aplc" "sko"  "btab" "clec" "hhal" "nlu"  "phu"  "zne"
## [31] "csec" "fcd"  "dmk"  "pvm"  "pja"  "hame" "hazt" "eaf"  "dsv"  "rsan"
## [41] "rmp"  "vde"  "vja"  "tut"  "dpte" "cscu" "ptep" "sdm"  "cel"  "cbr"
## [51] "hro"  "lgi"  "pcan" "bgt"  "gae"  "crg"  "myi"  "pmax" "obi"  "osn"
## [61] "lak"  "nve"  "epa"  "aten" "adf"  "amil" "pdam" "spis" "dgt"  "hmg"
## [71] "tad"  "aqu"
```

Organisms classified within cluster 3

```
## [1] "bmy" "loa" "nai" "tsp" "smm" "shx" "ovi" "egl"
```

Organisms classified within cluster 4

```
##   [1] "hsa"  "ptr"  "pps"  "ggo"  "pon"  "nle"  "mcc"  "mcf"  "csab" "caty"
##  [11] "panu" "rro"  "rbb"  "tfn"  "pteh" "cjc"  "sbq"  "mmur" "mmu"  "mcal"
##  [21] "mpah" "rno"  "mcoc" "mun"  "cge"  "pleu" "ngi"  "hgl"  "ccan" "ocu"
```

```
##  [31] "opi"  "tup"  "cfa"  "vvp"  "vlg"  "aml"  "umr"  "uah"  "oro"  "elk" 
##  [41] "mpuf" "eju"  "mlx"  "fca"  "pyu"  "pbg"  "ptg"  "ppad" "aju"  "hhv" 
##  [51] "bta"  "bom"  "biu"  "bbub" "chx"  "oas"  "oda"  "ccad" "ssc"  "cfr" 
##  [61] "cbai" "cdk"  "bacu" "lve"  "oor"  "dle"  "pcad" "ecb"  "epz"  "eai" 
##  [71] "myb"  "myd"  "mmyo" "mna"  "pkl"  "hai"  "dro"  "shon" "pdic" "mmf" 
##  [81] "rfq"  "pale" "pgig" "ray"  "mjv"  "tod"  "lav"  "tmu"  "mdo"  "gas" 
##  [91] "shr"  "pcw"  "oaa"  "gga"  "pcoc" "mgp"  "cjo"  "nmel" "tgu"  "lsr" 
## [101] "scan" "pmoa" "otc"  "pruf" "gfr"  "fab"  "phi"  "pmaj" "ccae" "etl" 
## [111] "fpg"  "fch"  "clv"  "nni"  "arow" "npd"  "dne"  "asn"  "amj"  "pss" 
## [121] "cmy"  "cpic" "tst"  "cabi" "acs"  "pvt"  "sund" "pbi"  "pmur" "tsr" 
## [131] "pgut" "vko"  "pmua" "zvi"  "gja"  "xla"  "xtr"  "npr"  "dre"  "srx" 
## [141] "sanh" "sgh"  "caua" "ipu"  "phyp" "eee"  "tru"  "lco"  "cgob" "ely" 
## [151] "plep" "sluc" "ecra" "pflv" "gat"  "ppug" "msam" "cud"  "mze"  "onl" 
## [161] "oau"  "ola"  "oml"  "xma"  "xco"  "xhe"  "pret" "cvg"  "ctul" "nfu" 
## [171] "kmr"  "alim" "aoce" "csem" "pov"  "ssen" "lcf"  "sdu"  "slal" "xgl" 
## [181] "hcq"  "bpec" "malb" "sasa" "otw"  "omy"  "salp" "snh"  "els"  "sfm" 
## [191] "pki"  "aang" "pspa" "arut" "lcm"
```

>br>

Weisfeiler-Lehman (WL) kernel

Heatmap

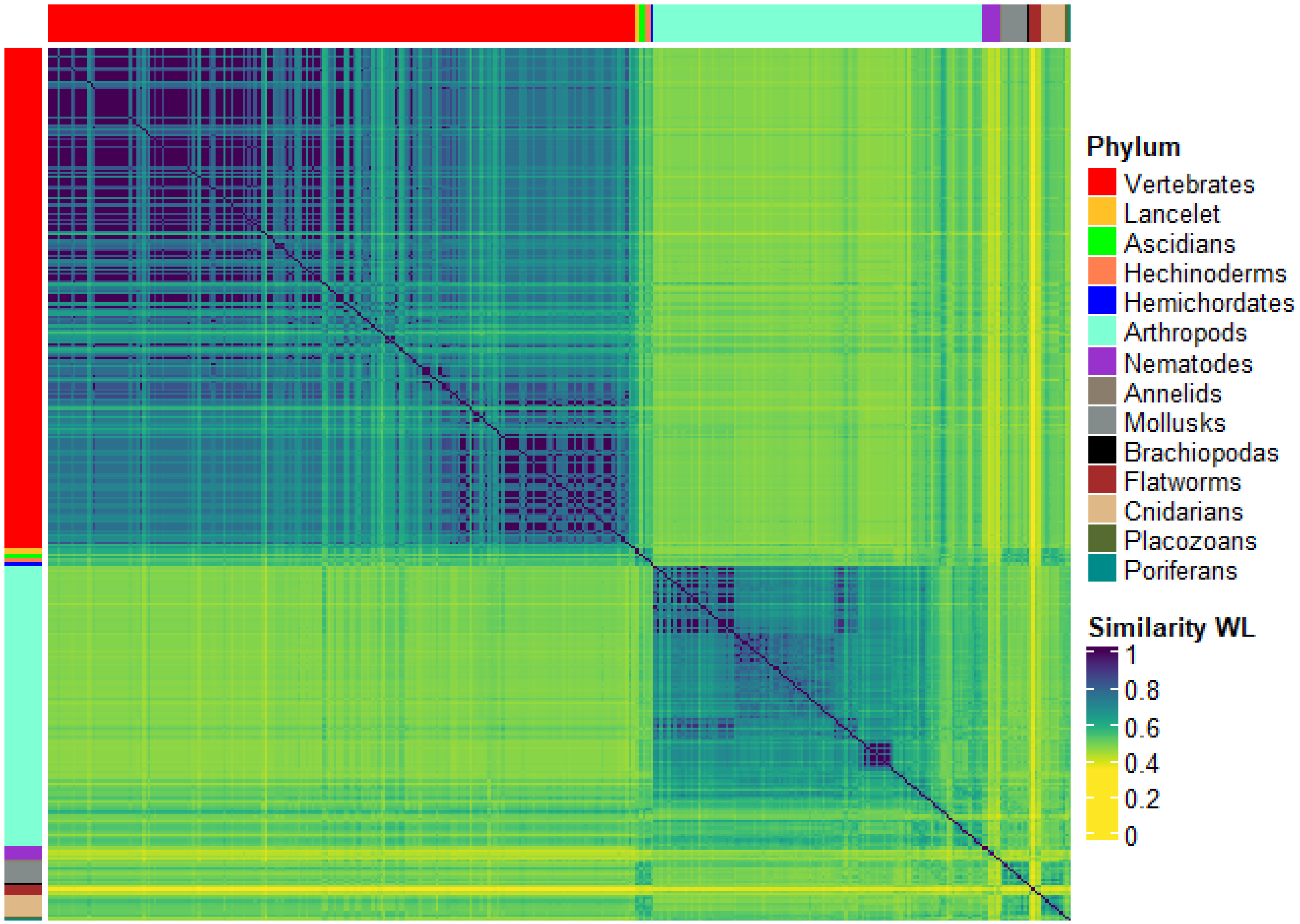

Phylum
Vertebrates
Lancelet
Ascidians
Hechinoderms
Hemichordates
Arthropods
Nematodes
Annelids
Mollusks
Brachiopodas
Flatworms
Cnidarians
Placozoans
Poriferans
Similarity WL
1
0.8
0.6
0.4
0.2
0

MDS for WL

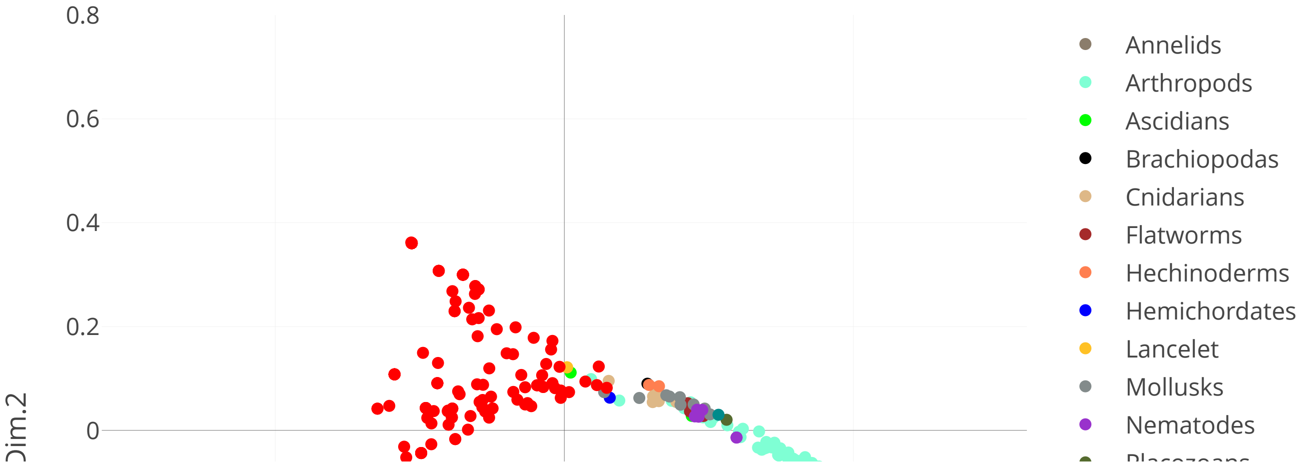

0.8
0.6
0.4
0.2
0
Annelids
Arthropods
Ascidians
Brachiopodas
Cnidarians
Flatworms
Hechinoderms
Hemichordates
Lancelet
Mollusks
Nematodes

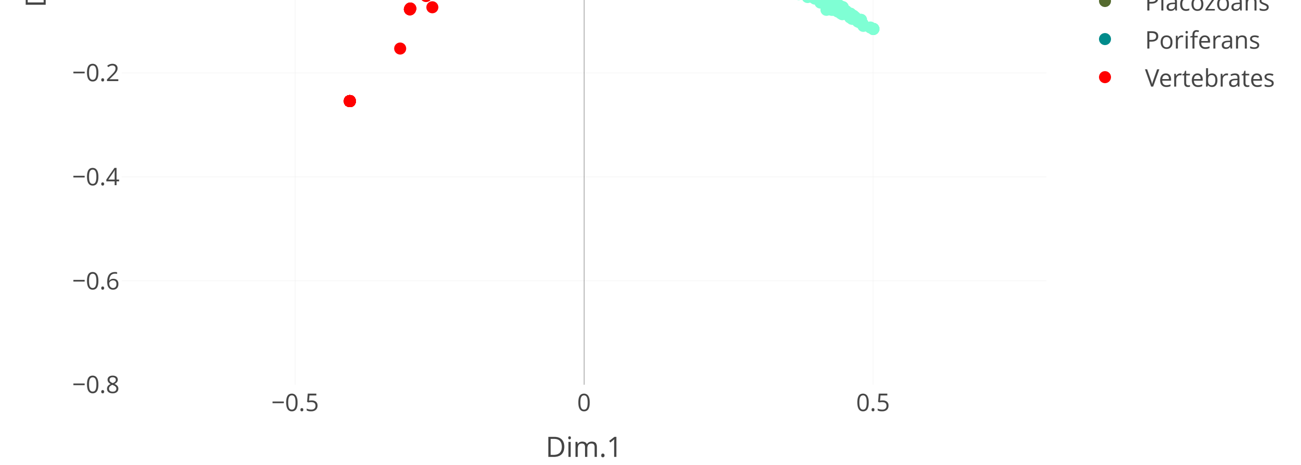

14-Means clustering for WL Kernel

```
##                  Cluster
## Real group         1  2  3  4  5  6  7  8  9 10 11 12 13 14
##   Annelids         0  0  0  0  0  0  0  0  0  0  0  1  0  0
##   Arthropods       2  0 54  0 19  0  0 18  0  0  9 17  0  0
##   Ascidians        1  0  0  0  0  0  0  0  1  0  0  0  0  0
##   Brachiopodas     0  1  0  0  0  0  0  0  0  0  0  0  0  0
##   Cnidarians       0  7  0  0  0  0  0  0  1  0  0  1  0  0
##   Flatworms        0  0  0  0  0  0  0  0  0  0  0  0  4  0
##   Hechinoderms     0  0  0  0  0  0  0  0  2  0  0  0  0  0
##   Hemichordates    1  0  0  0  0  0  0  0  0  0  0  0  0  0
##   Lancelet         2  0  0  0  0  0  0  0  0  0  0  0  0  0
##   Mollusks         1  1  0  0  0  0  0  0  7  0  0  0  0  0
##   Nematodes        0  0  0  0  0  0  0  2  0  0  0  0  4  0
##   Placozoans       0  0  0  0  0  0  0  1  0  0  0  0  0  0
##   Poriferans       0  0  0  0  0  0  0  0  1  0  0  0  0  0
##   Vertebrates     15  0  0 28  0 67 67  0  0 17  0  0  0 18
```

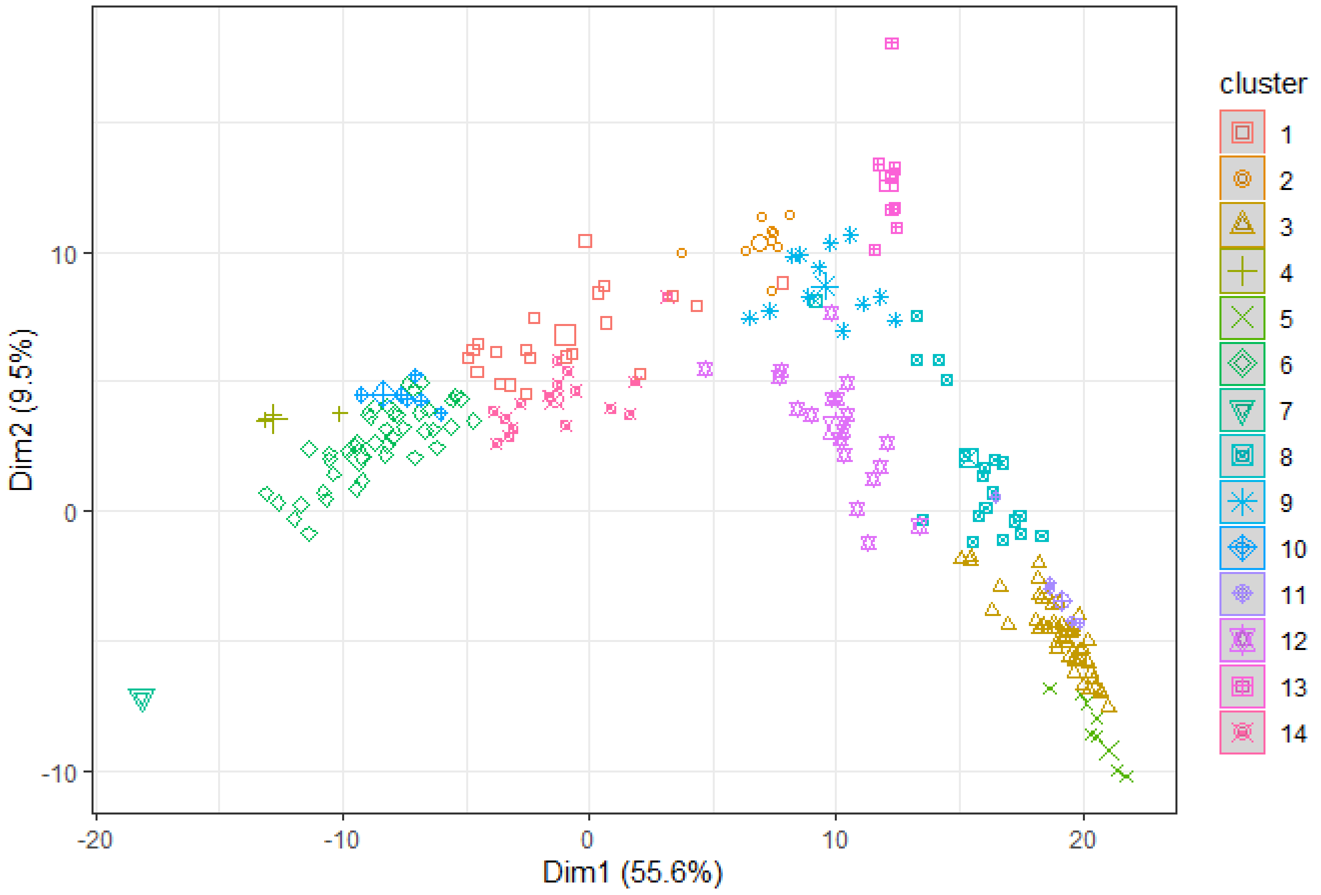

Organisms classified within cluster 1

```
##  [1] "pteh" "vlg"  "chx"  "shon" "ajm"  "aam"  "acs"  "ccar" "amex" "tng"
## [11] "ncc"  "cgob" "plep" "loc"  "rtp"  "bfo"  "bbel" "sclv" "sko"  "pja"
## [21] "hazt" "bgt"
```

Organisms classified within cluster 2

```
## [1] "obi"  "lak"  "nve"  "epa"  "aten" "adf"  "amil" "pdam" "spis"
```

Organisms classified within cluster 3

```
##  [1] "dme"  "dpo"  "dwi"  "dgr"  "daz"  "ccat" "bod"  "scac" "lcq"  "aga"
## [11] "aalb" "ame"  "acer" "bim"  "bbif" "bvk"  "bvan" "bter" "ccal" "obb"
## [21] "mgen" "nmea" "cgig" "soc"  "mpha" "aec"  "acep" "pbar" "vem"  "hst"
## [31] "dqu"  "cfo"  "fex"  "lhu"  "pgc"  "obo"  "pcf"  "pfuc" "nvi"  "tpre"
## [41] "mdl"  "fas"  "dam"  "ccin" "dpa"  "apln" "ppyr" "otu"  "bmor" "rmd"
## [51] "btab" "clec" "phu"  "foc"
```

Organisms classified within cluster 4

```
##  [1] "srx"  "sanh" "caua" "ely"  "sluc" "ecra" "pflv" "gat"  "ppug" "onl"
## [11] "oau"  "oml"  "xma"  "xco"  "xhe"  "ctul" "nfu"  "pov"  "ssen" "sdu"
## [21] "slal" "bpec" "malb" "sasa" "snh"  "els"  "sfm"  "aang"
```

Organisms classified within cluster 5

```
##  [1] "der"  "dsi"  "dya"  "dsr"  "dpe"  "dmn"  "dmo"  "dnv"  "dhe"  "mde"
## [11] "acoz" "aara" "aag"  "cqu"  "cpii" "tca"  "atd"  "agb"  "nvl"
```

Organisms classified within cluster 6

```
##  [1] "ggo"  "csab" "caty" "cjc"  "sbq"  "ocu"  "cfa"  "vvp"  "aml"  "umr"
## [11] "oro"  "eju"  "bta"  "biu"  "ssc"  "cfr"  "pcad" "myb"  "mmyo" "pkl"
## [21] "rfq"  "pale" "pgig" "ray"  "mjv"  "lav"  "mdo"  "gas"  "shr"  "oaa"
## [31] "pcoc" "nmel" "tgu"  "lsr"  "pmaj" "etl"  "fpg"  "fch"  "clv"  "nni"
## [41] "amj"  "pss"  "cmy"  "tst"  "cabi" "pvt"  "sund" "pbi"  "pmur" "tsr"
## [51] "vko"  "pmua" "zvi"  "gja"  "xla"  "xtr"  "npr"  "ipu"  "phyp" "eee"
## [61] "cud"  "ola"  "pret" "aoce" "hcq"  "salp" "lcm"
```

Organisms classified within cluster 7

```
##  [1] "hsa"   "ptr"   "pps"   "pon"   "nle"   "mcc"   "mcf"   "panu"  "rro"   "rbb"  
## [11] "tfn"   "mmur"  "mmu"   "mcal"  "mpah"  "rno"   "mcoc"  "mun"   "cge"   "pleu" 
## [21] "ngi"   "hgl"   "ccan"  "opi"   "tup"   "uah"   "elk"   "mpuf"  "mlx"   "fca"  
## [31] "pyu"   "pbg"   "ptg"   "ppad"  "aju"   "hhv"   "bom"   "bbub"  "oas"   "oda"  
## [41] "ccad"  "cbai"  "cdk"   "bacu"  "oor"   "dle"   "ecb"   "epz"   "eai"   "myd"  
## [51] "mna"   "hai"   "dro"   "pdic"  "mmf"   "tod"   "tmu"   "pcw"   "gga"   "mgp"  
## [61] "cjo"   "pmoa"  "otc"   "pruf"  "phi"   "asn"   "cpic"
```

Organisms classified within cluster 8

```
##  [1] "dse"   "dan"   "dvi"   "vps"   "csol"  "cglo"  "ldc"   "bman"  "dpl"   "pxy"  
## [11] "api"   "dnx"   "ags"   "dci"   "fcd"   "eaf"   "isc"   "tut"   "cel"   "cbr"  
## [21] "tad"
```

Organisms classified within cluster 9

```
##  [1] "cin"   "spu"   "aplc"  "lgi"   "pcan"  "gae"   "crg"   "myi"   "pmax"  "osn"  
## [11] "dgt"   "aqu"
```

Organisms classified within cluster 10

```
##  [1] "dre"   "sgh"   "tru"   "lco"   "msam"  "mze"   "cvg"   "kmr"   "alim"  "csem" 
## [11] "lcf"   "xgl"   "otw"   "omy"   "pki"   "pspa"  "arut"
```

Organisms classified within cluster 11

```
## [1] "msex" "bany" "pmac" "ppot" "pxu"  "prap" "zce"  "haw"  "tnl"
```

Organisms classified within cluster 12

```
##  [1] "hhal" "nlu"  "zne"  "csec" "dpx"  "dmk"  "pvm"  "hame" "dsv"  "rsan"
## [11] "rmp"  "vde"  "vja"  "dpte" "cscu" "ptep" "sdm"  "hro"  "hmg"
```

Organisms classified within cluster 13

```
## [1] "bmy" "loa" "nai" "tsp" "smm" "shx" "ovi" "egl"
```

Organisms classified within cluster 14

```
##  [1] "lve"  "apla" "acyg" "scan" "gfr"  "fab"  "ccae" "ccw"  "egz"  "acun"
## [11] "padl" "arow" "npd"  "dne"  "cpoo" "ggn"  "pgut" "cmk"
```

Optimal number of clusters for WL

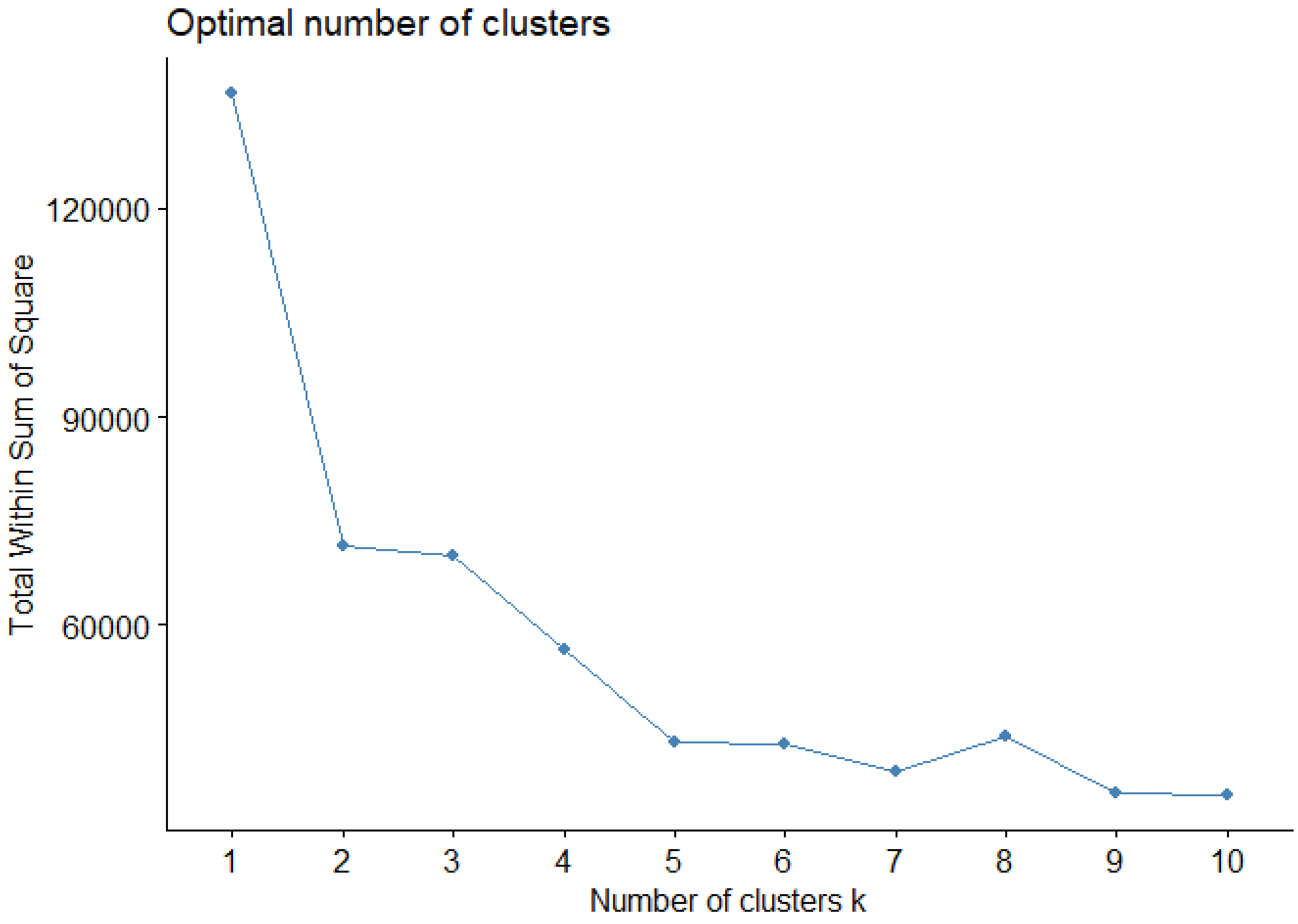


```
##                 Cluster
## Real group        1   2   3   4
##   Annelids        1   0   0   0
##   Arthropods     20   0   0  99
##   Ascidians       2   0   0   0
##   Brachiopodas    1   0   0   0
##   Cnidarians      9   0   0   0
##   Flatworms       4   0   0   0
##   Hechinoderms    2   0   0   0
##   Hemichordates   1   0   0   0
##   Lancelet        2   0   0   0
```

```
##    Mollusks        9   0   0   0
##    Nematodes       6   0   0   0
##    Placozoans      1   0   0   0
##    Poriferans      1   0   0   0
##    Vertebrates     4 141  67   0
```

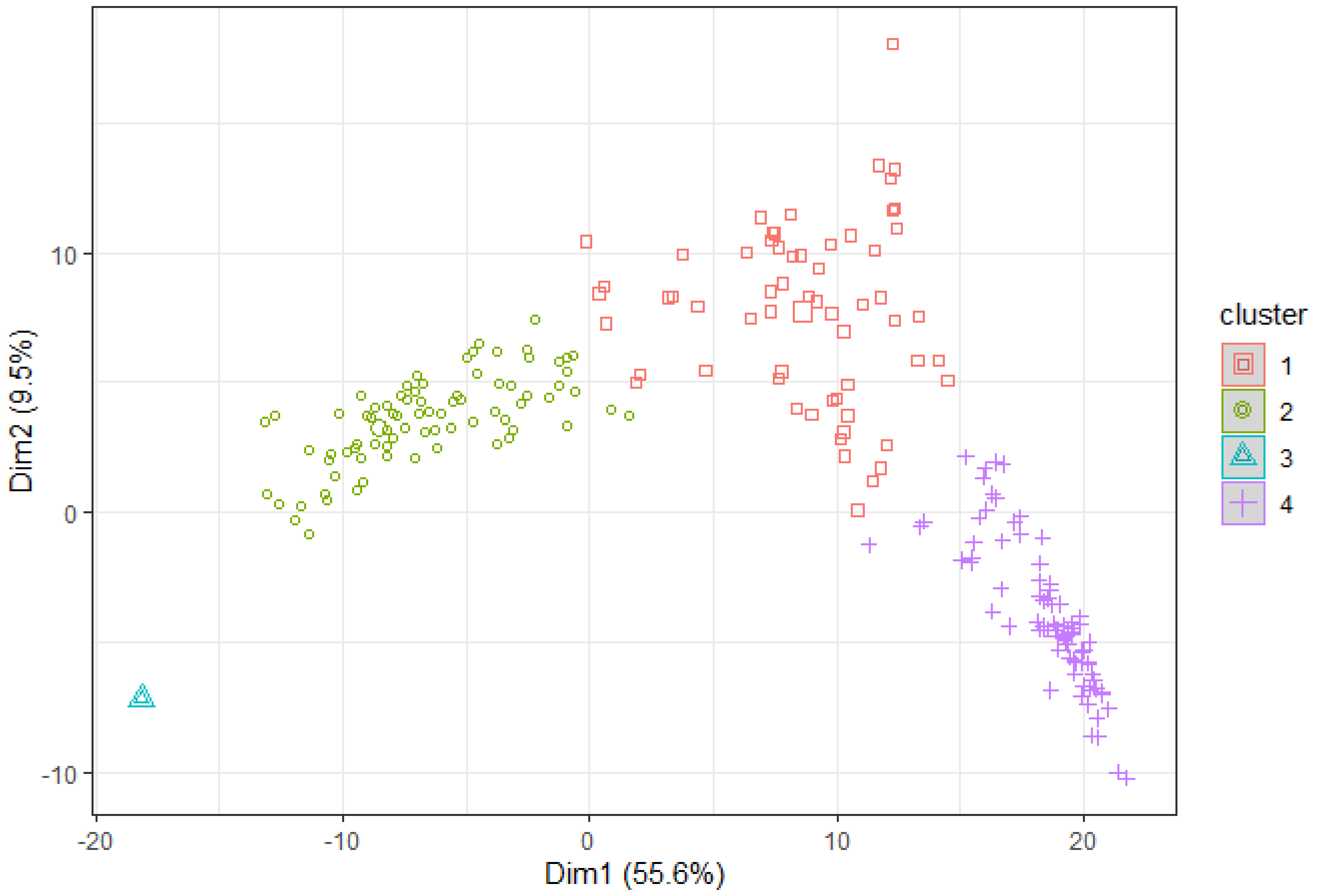

Organisms classified within cluster 1

```
##  [1] "ajm"  "padl" "ccar" "cmk"  "bfo"  "bbel" "cin"  "sclv" "spu"  "aplc"
## [11] "sko"  "nlu"  "csec" "fcd"  "dpx"  "dmk"  "pvm"  "pja"  "hame" "hazt"
## [21] "eaf"  "dsv"  "rsan" "rmp"  "vde"  "vja"  "tut"  "dpte" "cscu" "ptep"
## [31] "sdm"  "cel"  "cbr"  "bmy"  "loa"  "nai"  "tsp"  "hro"  "lgi"  "pcan"
## [41] "bgt"  "gae"  "crg"  "myi"  "pmax" "obi"  "osn"  "lak"  "smm"  "shx"
## [51] "ovi"  "egl"  "nve"  "epa"  "aten" "adf"  "amil" "pdam" "spis" "dgt"
## [61] "hmg"  "tad"  "aqu"
```

Organisms classified within cluster 2

```
##   [1] "ggo"  "csab" "caty" "pteh" "cjc"  "sbq"  "ocu"  "cfa"  "vvp"  "vlg"
##  [11] "aml"  "umr"  "oro"  "eju"  "bta"  "biu"  "chx"  "ssc"  "cfr"  "lve"
##  [21] "pcad" "myb"  "mmyo" "pkl"  "shon" "rfq"  "pale" "pgig" "ray"  "mjv"
##  [31] "lav"  "mdo"  "gas"  "shr"  "oaa"  "pcoc" "nmel" "apla" "acyg" "tgu"
##  [41] "lsr"  "scan" "gfr"  "fab"  "pmaj" "ccae" "ccw"  "etl"  "fpg"  "fch"
##  [51] "clv"  "egz"  "nni"  "acun" "aam"  "arow" "npd"  "dne"  "amj"  "cpoo"
##  [61] "ggn"  "pss"  "cmy"  "tst"  "cabi" "acs"  "pvt"  "sund" "pbi"  "pmur"
##  [71] "tsr"  "pgut" "vko"  "pmua" "zvi"  "gja"  "xla"  "xtr"  "npr"  "dre"
##  [81] "srx"  "sanh" "sgh"  "caua" "ipu"  "phyp" "amex" "eee"  "tru"  "tng"
##  [91] "lco"  "ncc"  "cgob" "ely"  "plep" "sluc" "ecra" "pflv" "gat"  "ppug"
## [101] "msam" "cud"  "mze"  "onl"  "oau"  "ola"  "oml"  "xma"  "xco"  "xhe"
## [111] "pret" "cvg"  "ctul" "nfu"  "kmr"  "alim" "aoce" "csem" "pov"  "ssen"
## [121] "lcf"  "sdu"  "slal" "xgl"  "hcq"  "bpec" "malb" "sasa" "otw"  "omy"
## [131] "salp" "snh"  "els"  "sfm"  "pki"  "aang" "loc"  "pspa" "arut" "lcm"
## [141] "rtp"
```

Organisms classified within cluster 3

```
##  [1] "hsa"  "ptr"  "pps"  "pon"  "nle"  "mcc"  "mcf"  "panu" "rro"  "rbb"
## [11] "tfn"  "mmur" "mmu"  "mcal" "mpah" "rno"  "mcoc" "mun"  "cge"  "pleu"
## [21] "ngi"  "hgl"  "ccan" "opi"  "tup"  "uah"  "elk"  "mpuf" "mlx"  "fca"
## [31] "pyu"  "pbg"  "ptg"  "ppad" "aju"  "hhv"  "bom"  "bbub" "oas"  "oda"
```

```
## [41] "ccad" "cbai" "cdk"  "bacu" "oor"  "dle"  "ecb"  "epz"  "eai"  "myd"
## [51] "mna"  "hai"  "dro"  "pdic" "mmf"  "tod"  "tmu"  "pcw"  "gga"  "mgp"
## [61] "cjo"  "pmoa" "otc"  "pruf" "phi"  "asn"  "cpic"
```

Organisms classified within cluster 4

```
##  [1] "dme"  "der"  "dse"  "dsi"  "dya"  "dan"  "dsr"  "dpo"  "dpe"  "dmn"
## [11] "dwi"  "dgr"  "dmo"  "daz"  "dnv"  "dhe"  "dvi"  "ccat" "bod"  "mde"
## [21] "scac" "lcq"  "aga"  "acoz" "aara" "aag"  "aalb" "cqu"  "cpii" "ame"
## [31] "acer" "bim"  "bbif" "bvk"  "bvan" "bter" "ccal" "obb"  "mgen" "nmea"
## [41] "cgig" "soc"  "mpha" "aec"  "acep" "pbar" "vem"  "hst"  "dqu"  "cfo"
## [51] "fex"  "lhu"  "pgc"  "obo"  "pcf"  "pfuc" "vps"  "nvi"  "csol" "tpre"
## [61] "mdl"  "cglo" "fas"  "dam"  "ccin" "tca"  "dpa"  "atd"  "agb"  "ldc"
## [71] "nvl"  "apln" "ppyr" "otu"  "bmor" "bman" "msex" "dpl"  "bany" "pmac"
## [81] "ppot" "pxu"  "prap" "zce"  "haw"  "tnl"  "pxy"  "api"  "dnx"  "ags"
## [91] "rmd"  "btab" "dci"  "clec" "hhal" "phu"  "foc"  "zne"  "isc"
```

Pyramid match (PM) kernel

Heatmap

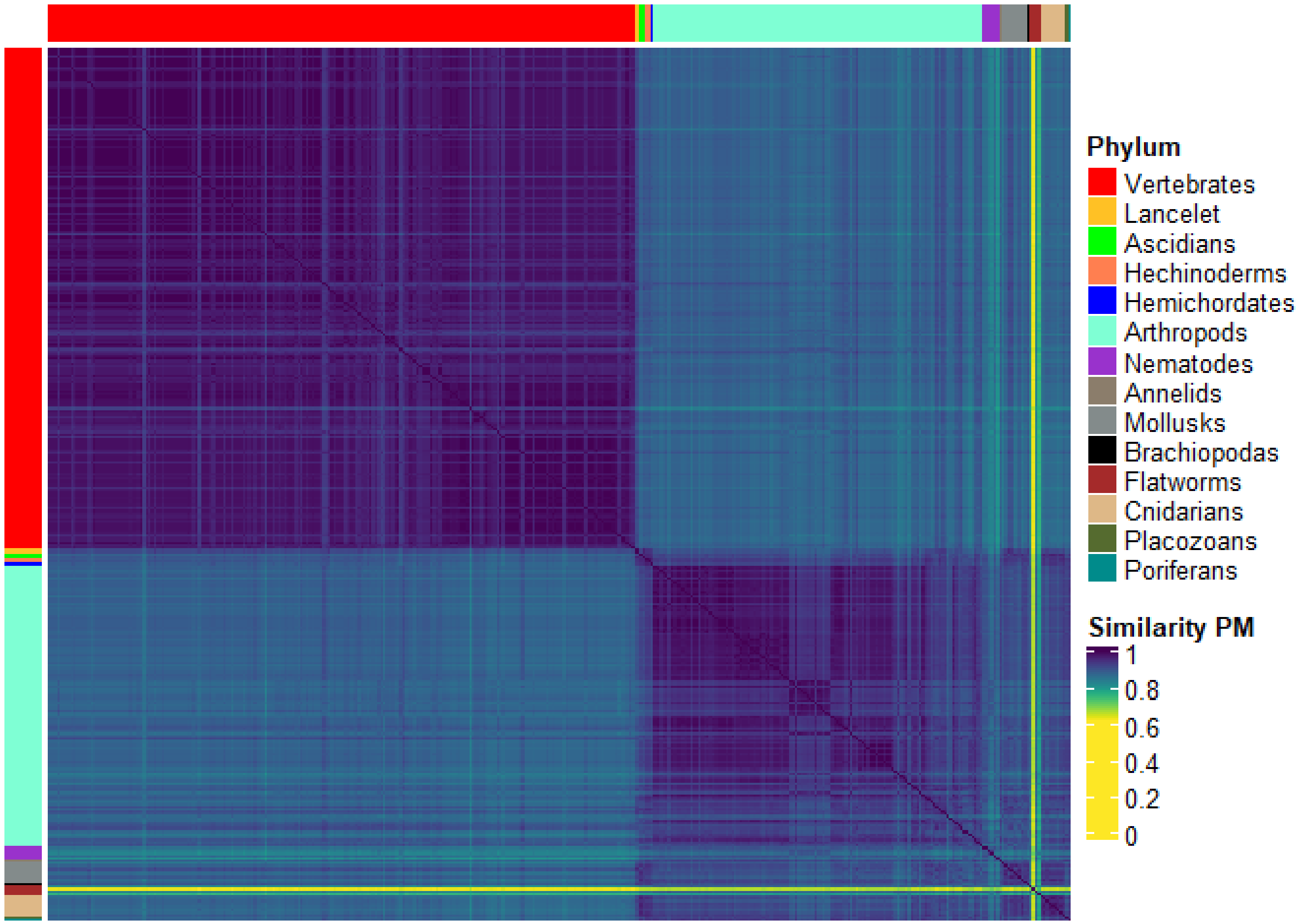
Phylum
Vertebrates
Lancelet
Ascidians
Hechinoderms
Hemichordates
Arthropods
Nematodes
Annelids
Mollusks
Brachiopodas
Flatworms
Cnidarians
Placozoans
Poriferans
Similarity PM
1
0.8
0.6
0.4
0.2
0

MDS for PM

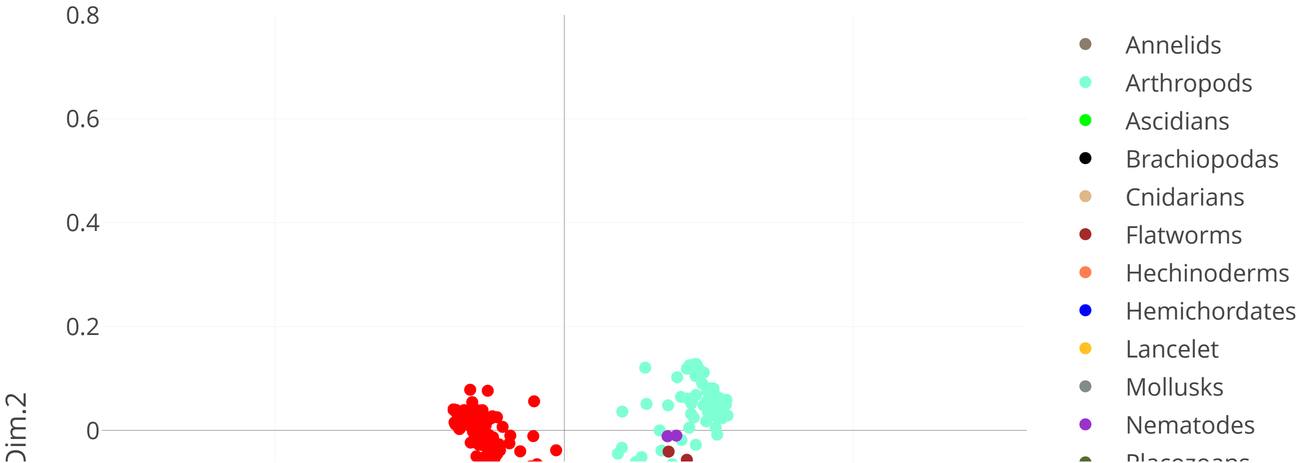
0.8
0.6
0.4
0.2
0
Dim.2
Annelids
Arthropods
Ascidians
Brachiopodas
Cnidarians
Flatworms
Hechinoderms
Hemichordates
Lancelet
Mollusks
Nematodes

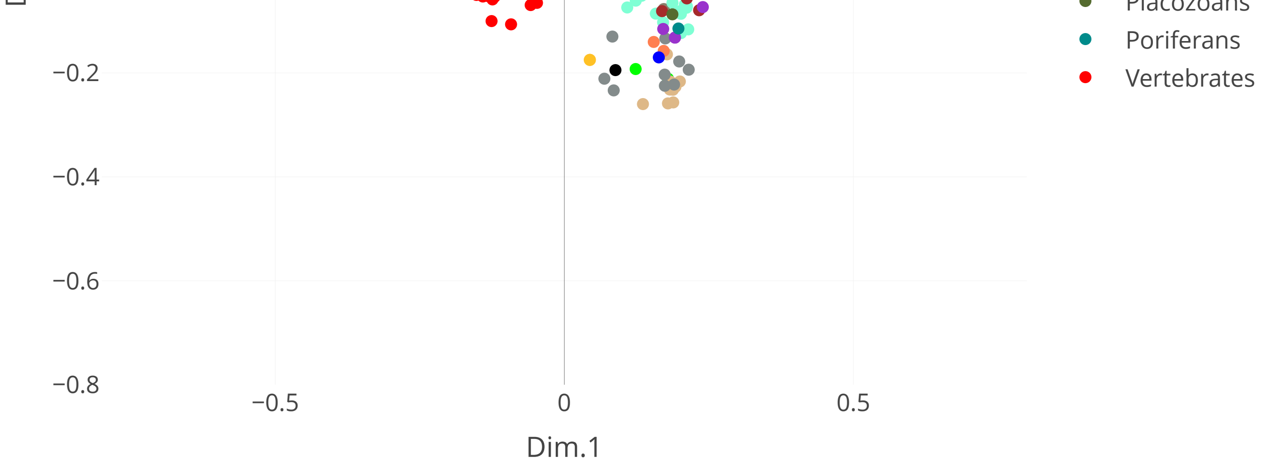

14-Means clustering for PM Kernel

```
##                  Cluster
## Real group         1  2  3  4  5  6  7  8  9 10 11 12 13 14
##   Annelids         0  0  0  0  0  0  1  0  0  0  0  0  0  0
##   Arthropods       0  0  1  0  0  0  8  0  0 30  9  0 16 55
##   Ascidians        0  0  2  0  0  0  0  0  0  0  0  0  0  0
##   Brachiopodas     0  0  1  0  0  0  0  0  0  0  0  0  0  0
##   Cnidarians       0  0  9  0  0  0  0  0  0  0  0  0  0  0
##   Flatworms        2  1  0  0  0  1  0  0  0  0  0  0  0  0
##   Hechinoderms     0  0  2  0  0  0  0  0  0  0  0  0  0  0
##   Hemichordates    0  0  1  0  0  0  0  0  0  0  0  0  0  0
##   Lancelet         0  0  0  0  0  0  0  0  0  0  2  0  0  0
##   Mollusks         0  0  6  0  0  0  1  0  0  0  2  0  0  0
##   Nematodes        3  0  0  0  0  0  3  0  0  0  0  0  0  0
##   Placozoans       0  0  0  0  0  0  1  0  0  0  0  0  0  0
##   Poriferans       0  0  1  0  0  0  0  0  0  0  0  0  0  0
##   Vertebrates      0  0  0 18 44  0  0 70 44  0  0 36  0  0
```

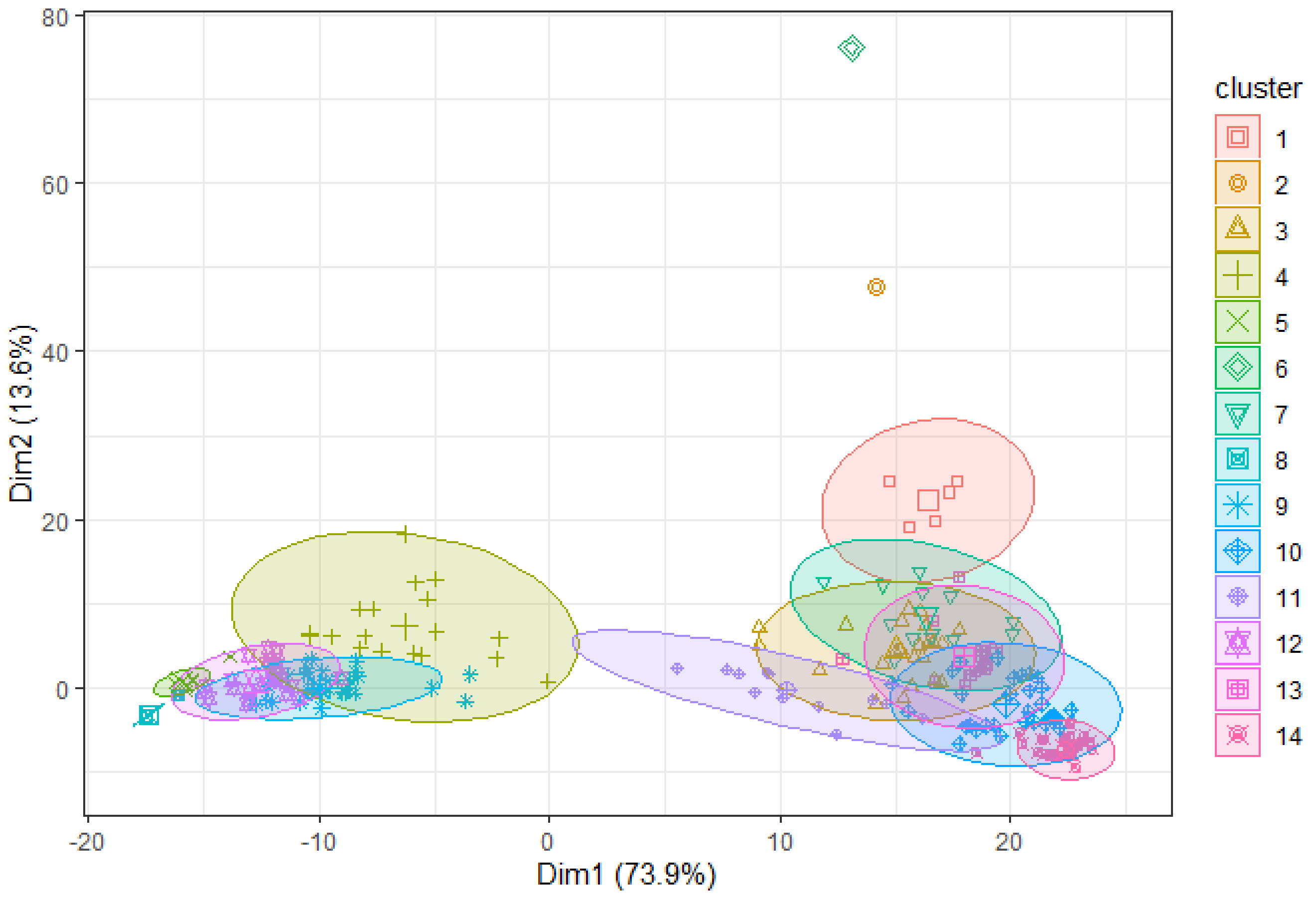

Organisms classified within cluster 1

```
## [1] "bmy" "loa" "tsp" "smm" "ovi"
```

Organisms classified within cluster 2

```
## [1] "egl"
```

Organisms classified within cluster 3

```
##  [1] "cin"  "sclv" "spu"  "aplc" "sko"  "pvm"  "pcan" "crg"  "myi"  "pmax"
## [11] "obi"  "osn"  "lak"  "nve"  "epa"  "aten" "adf"  "amil" "pdam" "spis"
## [21] "dgt"  "hmg"  "aqu"
```

Organisms classified within cluster 4

```
##  [1] "ggo"  "vlg"  "chx"  "ajm"  "apla" "acun" "padl" "aam"  "cpoo" "ccar"
## [11] "amex" "lco"  "ncc"  "cgob" "plep" "ola"  "aoce" "loc"
```

Organisms classified within cluster 5

```
##  [1] "sbq"  "vvp"  "amj"  "pmur" "pmua" "zvi"  "gja"  "xtr"  "srx"  "sanh"
## [11] "sgh"  "caua" "tru"  "ely"  "sluc" "ecra" "pflv" "gat"  "ppug" "mze"
## [21] "onl"  "oau"  "oml"  "xma"  "xco"  "xhe"  "cvg"  "nfu"  "kmr"  "alim"
## [31] "pov"  "ssen" "lcf"  "sdu"  "slal" "xgl"  "bpec" "malb" "omy"  "salp"
## [41] "snh"  "els"  "sfm"  "pki"
```

Organisms classified within cluster 6

```
## [1] "shx"
```

Organisms classified within cluster 7

```
##  [1] "ags"  "dci"  "fcd"  "hazt" "eaf"  "vde"  "vja"  "tut"  "cel"  "cbr"
## [11] "nai"  "hro"  "lgi"  "tad"
```

Organisms classified within cluster 8

```
##  [1] "hsa"  "ptr"  "pps"  "pon"  "nle"  "mcc"  "mcf"  "panu" "rro"  "rbb"
## [11] "tfn"  "mmur" "mmu"  "mcal" "mpah" "rno"  "mcoc" "mun"  "cge"  "pleu"
## [21] "ngi"  "hgl"  "ccan" "opi"  "tup"  "uah"  "elk"  "mpuf" "mlx"  "fca"
## [31] "pyu"  "pbg"  "ptg"  "ppad" "aju"  "hhv"  "bom"  "bbub" "oas"  "oda"
## [41] "ccad" "cbai" "cdk"  "bacu" "oor"  "dle"  "ecb"  "epz"  "eai"  "myd"
## [51] "mna"  "hai"  "dro"  "pdic" "mmf"  "tod"  "tmu"  "pcw"  "gga"  "mgp"
## [61] "cjo"  "pmoa" "otc"  "pruf" "phi"  "asn"  "cpic" "xla"  "npr"  "lcm"
```

Organisms classified within cluster 9

```
##  [1] "csab" "caty" "pteh" "umr"  "oro"  "eju"  "ssc"  "cfr"  "lve"  "pcad"
## [11] "myb"  "mmyo" "pkl"  "rfq"  "mjv"  "mdo"  "shr"  "nmel" "acyg" "tgu"
## [21] "gfr"  "fab"  "ccae" "ccw"  "etl"  "fpg"  "fch"  "egz"  "nni"  "arow"
## [31] "npd"  "dne"  "ggn"  "pss"  "cabi" "acs"  "pvt"  "sund" "pbi"  "tsr"
## [41] "vko"  "cud"  "hcq"  "rtp"
```

Organisms classified within cluster 10

```
##  [1] "dse"  "dan"  "daz"  "dvi"  "scac" "ame"  "nmea" "mpha" "vem"  "csol"
## [11] "ldc"  "ppyr" "bmor" "bman" "dpl"  "ppot" "zce"  "pxy"  "api"  "rmd"
## [21] "btab" "clec" "hhal" "zne"  "csec" "dpx"  "isc"  "dpte" "cscu" "ptep"
```

Organisms classified within cluster 11

```
##  [1] "bfo"  "bbel" "otu"  "nlu"  "dmk"  "pja"  "hame" "dsv"  "rsan" "rmp"
## [11] "sdm"  "bgt"  "gae"
```

Organisms classified within cluster 12

```
##  [1] "cjc"  "ocu"  "cfa"  "aml"  "bta"  "biu"  "shon" "pale" "pgig" "ray" 
## [11] "lav"  "gas"  "oaa"  "pcoc" "lsr"  "scan" "pmaj" "clv"  "cmy"  "tst" 
## [21] "pgut" "dre"  "ipu"  "phyp" "eee"  "tng"  "msam" "pret" "ctul" "csem"
## [31] "sasa" "otw"  "aang" "pspa" "arut" "cmk"
```

Organisms classified within cluster 13

```
##  [1] "cfo"  "fex"  "pgc"  "obo"  "pcf"  "pfuc" "vps"  "nvi"  "tpre" "mdl" 
## [11] "cglo" "fas"  "dam"  "apln" "dnx"  "phu"
```

Organisms classified within cluster 14

```
##  [1] "dme"  "der"  "dsi"  "dya"  "dsr"  "dpo"  "dpe"  "dmn"  "dwi"  "dgr" 
## [11] "dmo"  "dnv"  "dhe"  "ccat" "bod"  "mde"  "lcq"  "aga"  "acoz" "aara"
## [21] "aag"  "aalb" "cqu"  "cpii" "acer" "bim"  "bbif" "bvk"  "bvan" "bter"
## [31] "ccal" "obb"  "mgen" "cgig" "soc"  "aec"  "acep" "pbar" "hst"  "dqu" 
## [41] "lhu"  "ccin" "tca"  "dpa"  "atd"  "agb"  "nvl"  "msex" "bany" "pmac"
## [51] "pxu"  "prap" "haw"  "tnl"  "foc"
```

Optimal number of clusters for PM

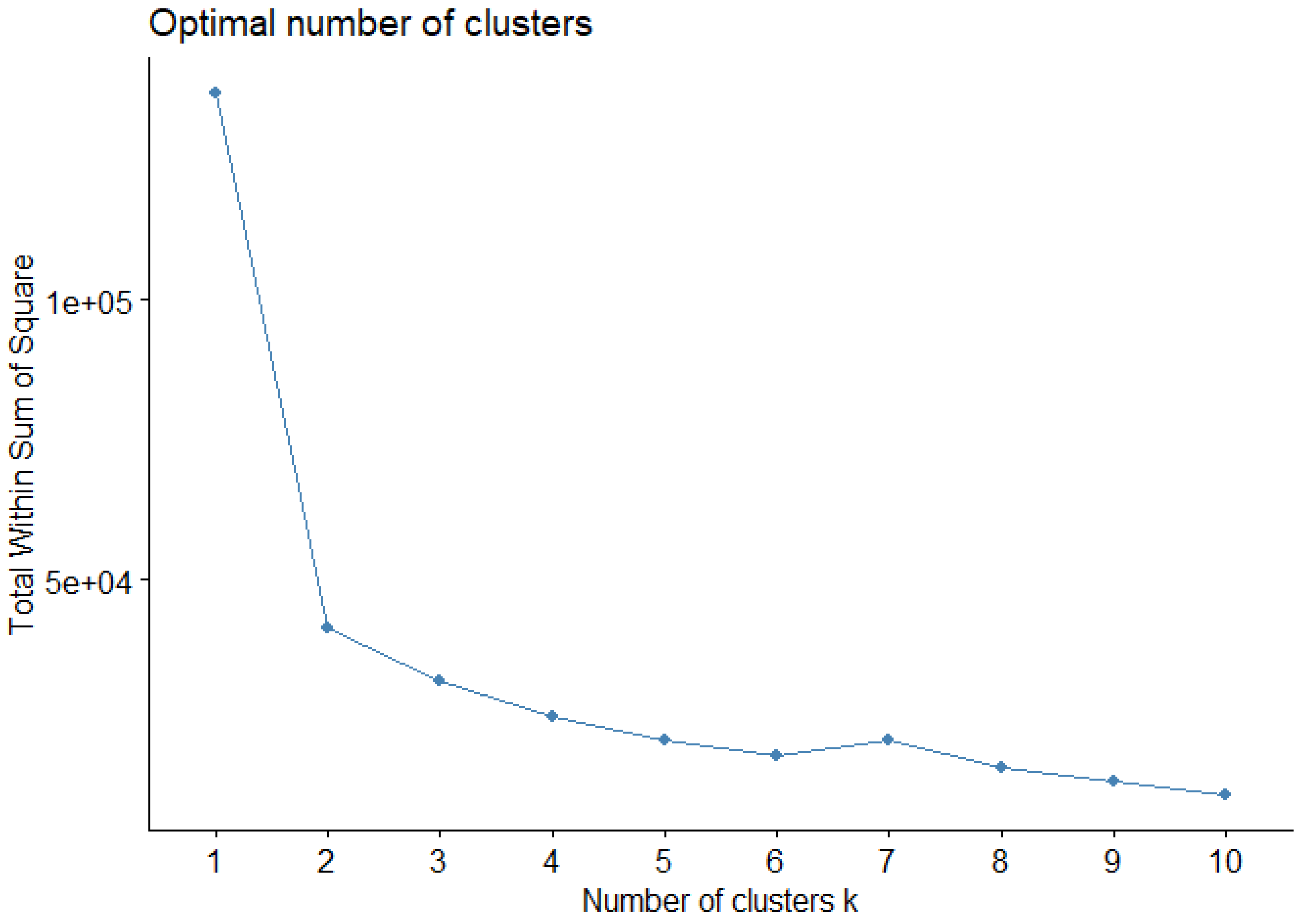


```
##                  Cluster
## Real group         1   2   3   4
##   Annelids         0   0   1   0
##   Arthropods       0   0  22  97
##   Ascidians        0   0   2   0
##   Brachiopodas     0   0   1   0
##   Cnidarians       0   0   9   0
##   Flatworms        0   2   2   0
##   Hechinoderms     0   0   2   0
##   Hemichordates    0   0   1   0
##   Lancelet         0   0   2   0
```

```
##    Mollusks       0   0   9   0
##    Nematodes      0   0   6   0
##    Placozoans     0   0   1   0
##    Poriferans     0   0   1   0
##    Vertebrates  212   0   0   0
```

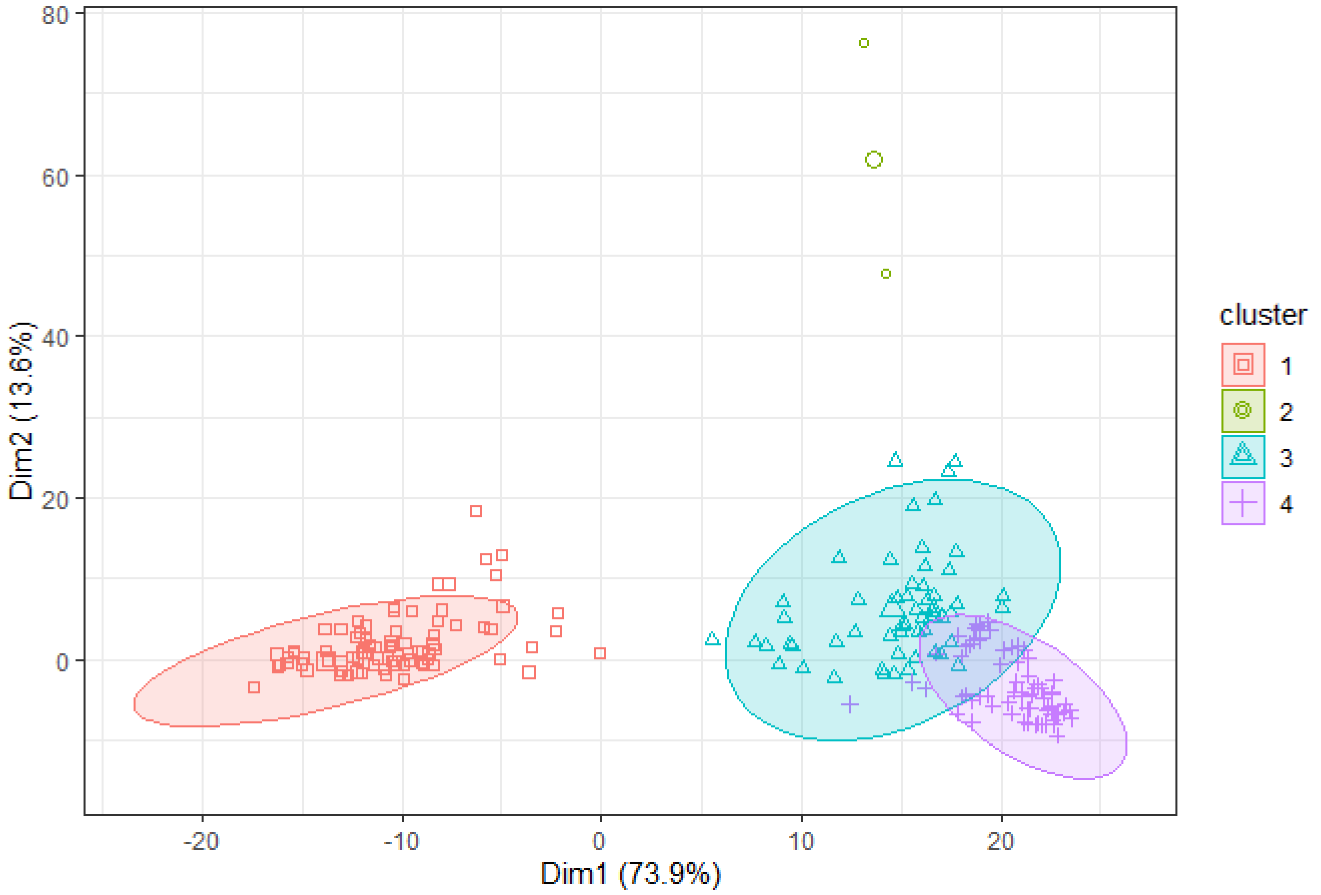

Organisms classified within cluster 1

```
##   [1] "hsa"  "ptr"  "pps"  "ggo"  "pon"  "nle"  "mcc"  "mcf"  "csab" "caty"
##  [11] "panu" "rro"  "rbb"  "tfn"  "pteh" "cjc"  "sbq"  "mmur" "mmu"  "mcal"
##  [21] "mpah" "rno"  "mcoc" "mun"  "cge"  "pleu" "ngi"  "hgl"  "ccan" "ocu"
##  [31] "opi"  "tup"  "cfa"  "vvp"  "vlg"  "aml"  "umr"  "uah"  "oro"  "elk"
##  [41] "mpuf" "eju"  "mlx"  "fca"  "pyu"  "pbg"  "ptg"  "ppad" "aju"  "hhv"
##  [51] "bta"  "bom"  "biu"  "bbub" "chx"  "oas"  "oda"  "ccad" "ssc"  "cfr"
##  [61] "cbai" "cdk"  "bacu" "lve"  "oor"  "dle"  "pcad" "ecb"  "epz"  "eai"
##  [71] "myb"  "myd"  "mmyo" "mna"  "pkl"  "hai"  "dro"  "shon" "ajm"  "pdic"
##  [81] "mmf"  "rfq"  "pale" "pgig" "ray"  "mjv"  "tod"  "lav"  "tmu"  "mdo"
##  [91] "gas"  "shr"  "pcw"  "oaa"  "gga"  "pcoc" "mgp"  "cjo"  "nmel" "apla"
## [101] "acyg" "tgu"  "lsr"  "scan" "pmoa" "otc"  "pruf" "gfr"  "fab"  "phi"
## [111] "pmaj" "ccae" "ccw"  "etl"  "fpg"  "fch"  "clv"  "egz"  "nni"  "acun"
## [121] "padl" "aam"  "arow" "npd"  "dne"  "asn"  "amj"  "cpoo" "ggn"  "pss"
## [131] "cmy"  "cpic" "tst"  "cabi" "acs"  "pvt"  "sund" "pbi"  "pmur" "tsr"
## [141] "pgut" "vko"  "pmua" "zvi"  "gja"  "xla"  "xtr"  "npr"  "dre"  "srx"
## [151] "sanh" "sgh"  "ccar" "caua" "ipu"  "phyp" "amex" "eee"  "tru"  "tng"
## [161] "lco"  "ncc"  "cgob" "ely"  "plep" "sluc" "ecra" "pflv" "gat"  "ppug"
## [171] "msam" "cud"  "mze"  "onl"  "oau"  "ola"  "oml"  "xma"  "xco"  "xhe"
## [181] "pret" "cvg"  "ctul" "nfu"  "kmr"  "alim" "aoce" "csem" "pov"  "ssen"
## [191] "lcf"  "sdu"  "slal" "xgl"  "hcq"  "bpec" "malb" "sasa" "otw"  "omy"
## [201] "salp" "snh"  "els"  "sfm"  "pki"  "aang" "loc"  "pspa" "arut" "lcm"
## [211] "cmk"  "rtp"
```

Organisms classified within cluster 2

```
## [1] "shx" "egl"
```

Organisms classified within cluster 3

```
##  [1] "bfo"  "bbel" "cin"  "sclv" "spu"  "aplc" "sko"  "cglo" "dnx"  "ags"
## [11] "dci"  "phu"  "csec" "fcd"  "dpx"  "dmk"  "pvm"  "pja"  "hame" "hazt"
## [21] "eaf"  "dsv"  "rsan" "rmp"  "vde"  "vja"  "tut"  "ptep" "sdm"  "cel"
```

```
## [31] "cbr"  "bmy"  "loa"  "nai"  "tsp"  "hro"  "lgi"  "pcan" "bgt"  "gae"
## [41] "crg"  "myi"  "pmax" "obi"  "osn"  "lak"  "smm"  "ovi"  "nve"  "epa"
## [51] "aten" "adf"  "amil" "pdam" "spis" "dgt"  "hmg"  "tad"  "aqu"
```

Organisms classified within cluster 4

```
##  [1] "dme"  "der"  "dse"  "dsi"  "dya"  "dan"  "dsr"  "dpo"  "dpe"  "dmn"
## [11] "dwi"  "dgr"  "dmo"  "daz"  "dnv"  "dhe"  "dvi"  "ccat" "bod"  "mde"
## [21] "scac" "lcq"  "aga"  "acoz" "aara" "aag"  "aalb" "cqu"  "cpii" "ame"
## [31] "acer" "bim"  "bbif" "bvk"  "bvan" "bter" "ccal" "obb"  "mgen" "nmea"
## [41] "cgig" "soc"  "mpha" "aec"  "acep" "pbar" "vem"  "hst"  "dqu"  "cfo"
## [51] "fex"  "lhu"  "pgc"  "obo"  "pcf"  "pfuc" "vps"  "nvi"  "csol" "tpre"
## [61] "mdl"  "fas"  "dam"  "ccin" "tca"  "dpa"  "atd"  "agb"  "ldc"  "nvl"
## [71] "apln" "ppyr" "otu"  "bmor" "bman" "msex" "dpl"  "bany" "pmac" "ppot"
## [81] "pxu"  "prap" "zce"  "haw"  "tnl"  "pxy"  "api"  "rmd"  "btab" "clec"
## [91] "hhal" "nlu"  "foc"  "zne"  "isc"  "dpte" "cscu"
```
