# Supplementary material for: Exploring the expressiveness of abstract metabolic networks
Source: PLoS One. 2023 Feb 9;18(2):e0281047. doi: 10.1371/journal.pone.0281047 (PMC9910719; doi:10.1371/journal.pone.0281047)
Supplement: S7 File — Vertebrates analyses at class level (second experiment). (PDF) [file pone.0281047.s007.pdf]

Vetebrates Analysis

- Heatmaps
  - Vertex Hystogram (VH) kernel
  - Shortest path (SP) kernel
  - Weisfeiler-Lehman (WL) kernel
  - Pyramid match (PM) kernel
- MDS
  - MDS for VH
  - MDS for SP
  - MDS for WL
  - MDS for PM
- Clustering
  - 6-means clustering for VH
  - 6-means clustering for SP
  - 6-means clustering for WL
  - 6-means clustering for PM

Heatmaps

Vertex Hystogram (VH) kernel

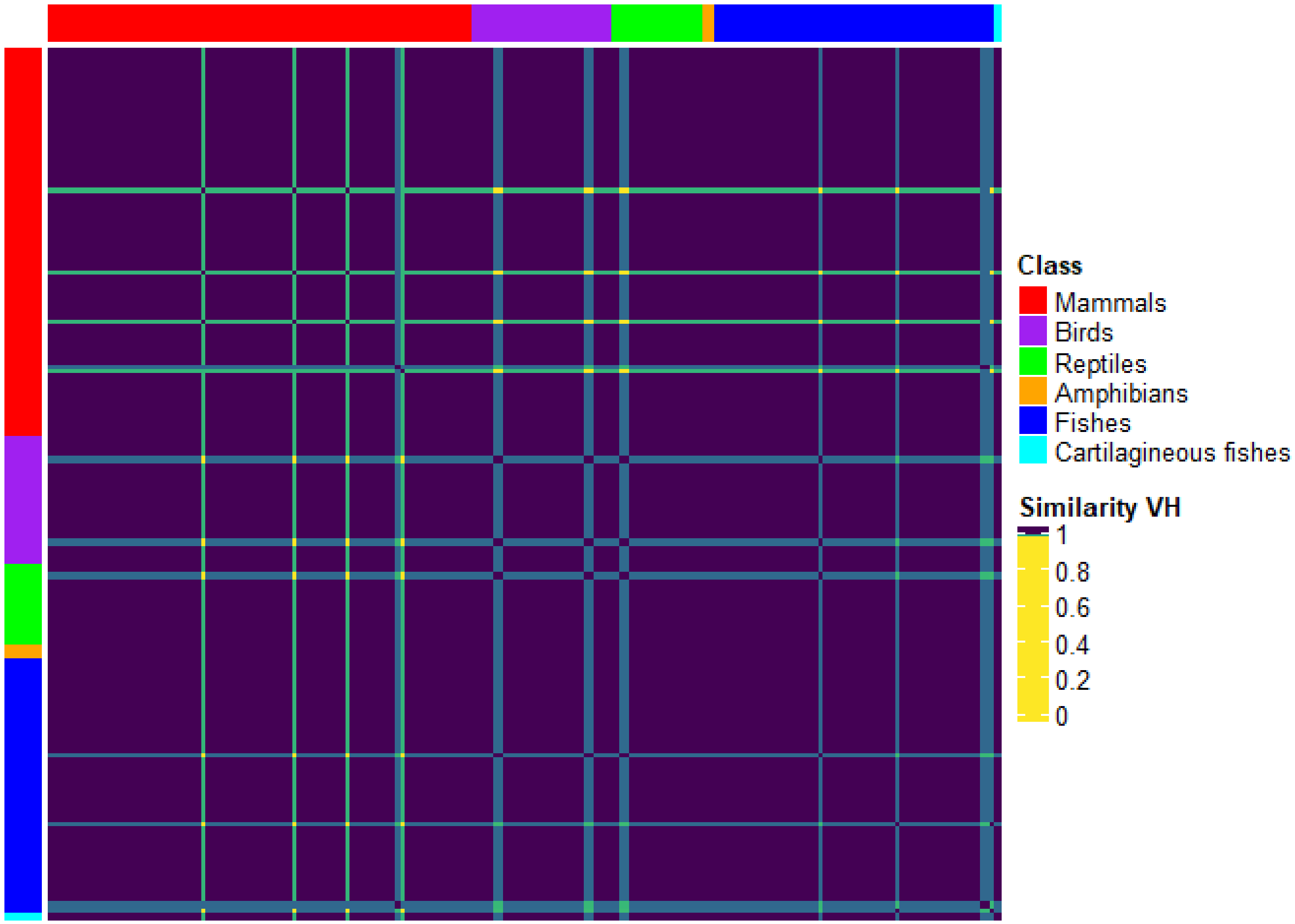

Shortest path (SP) kernel

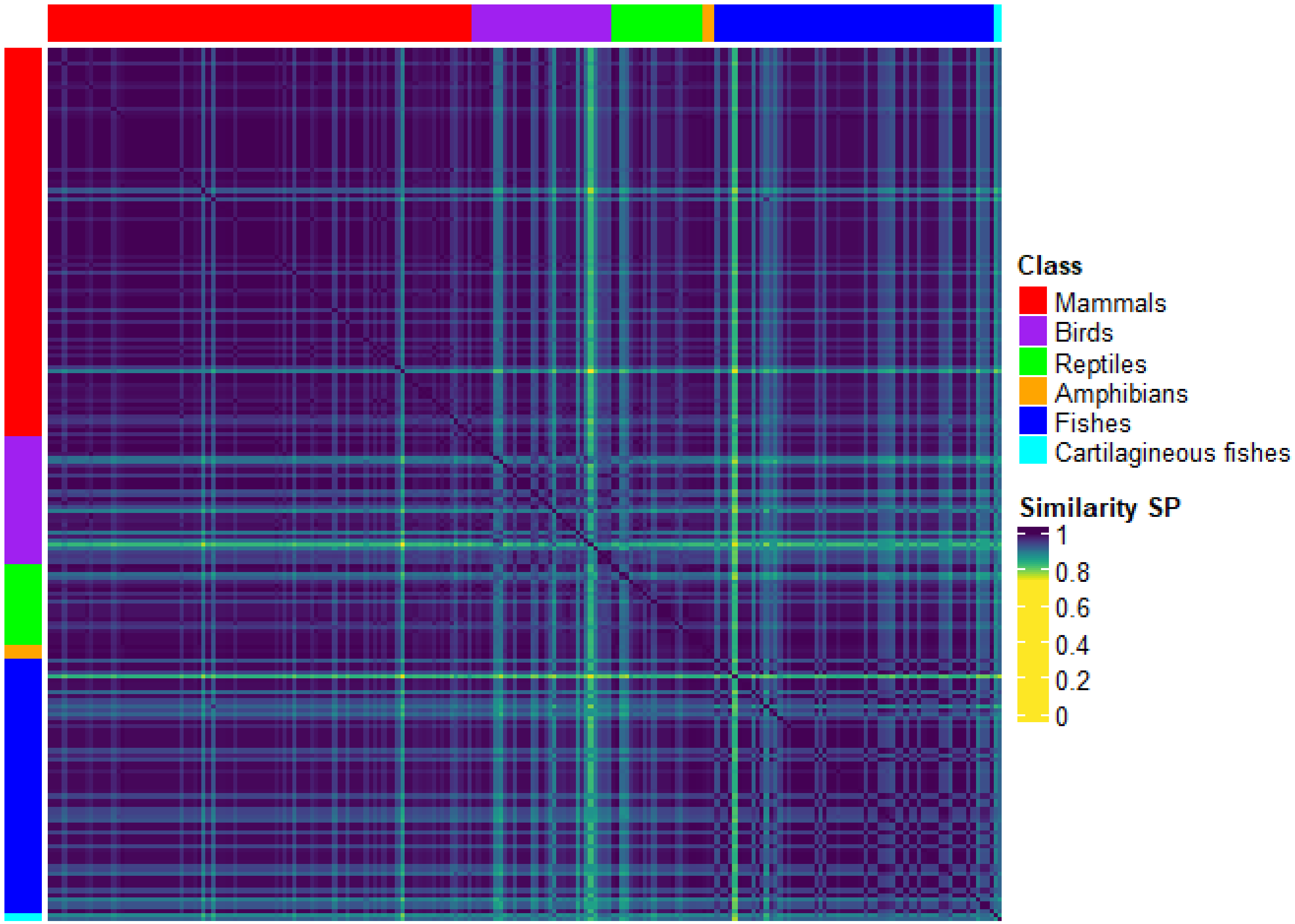

Weisfeiler-Lehman (WL) kernel

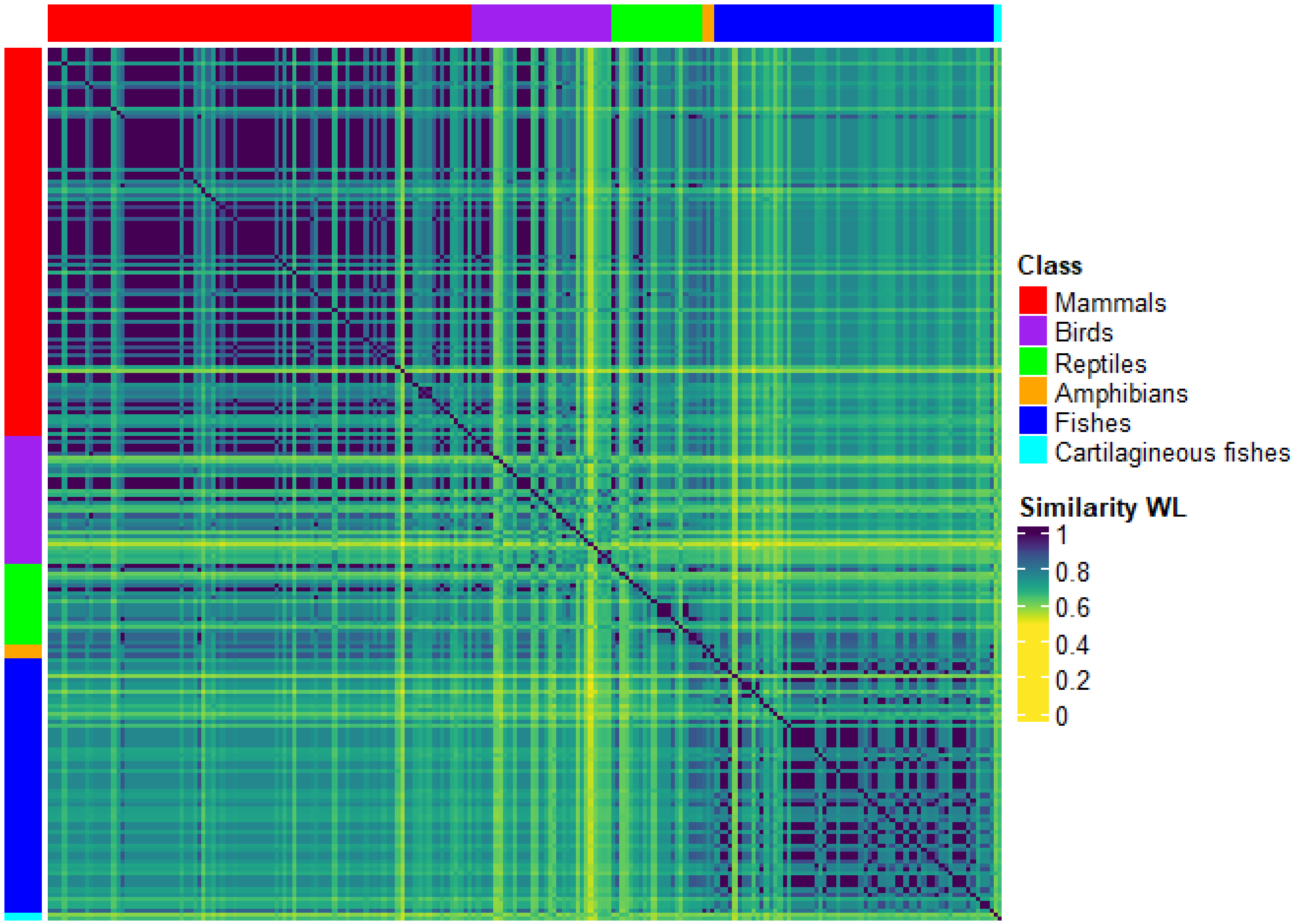

Pyramid match (PM) kernel

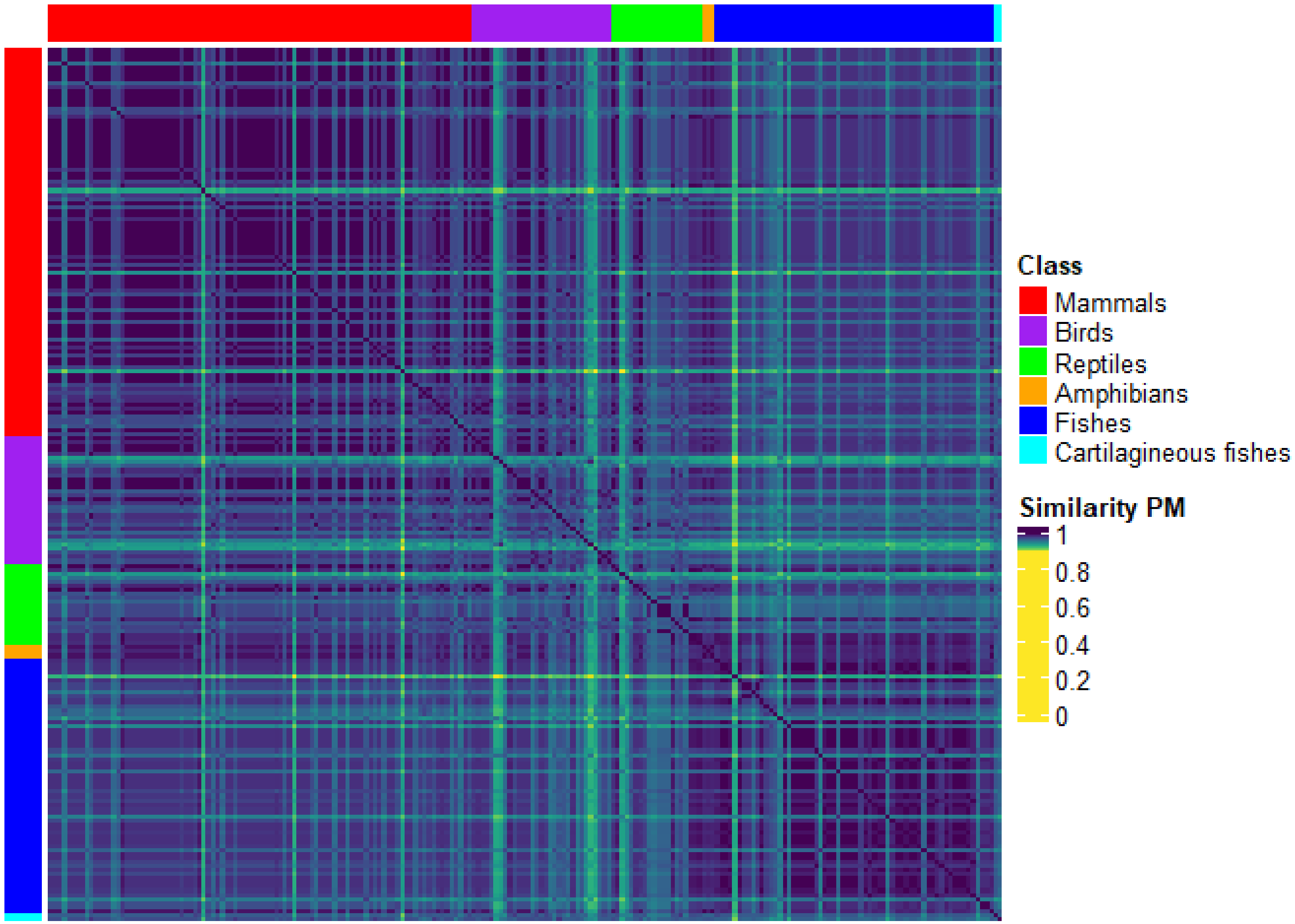

MDS

MDS for VH

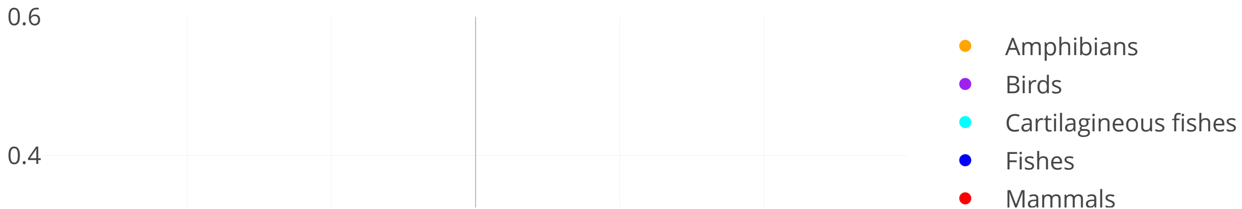

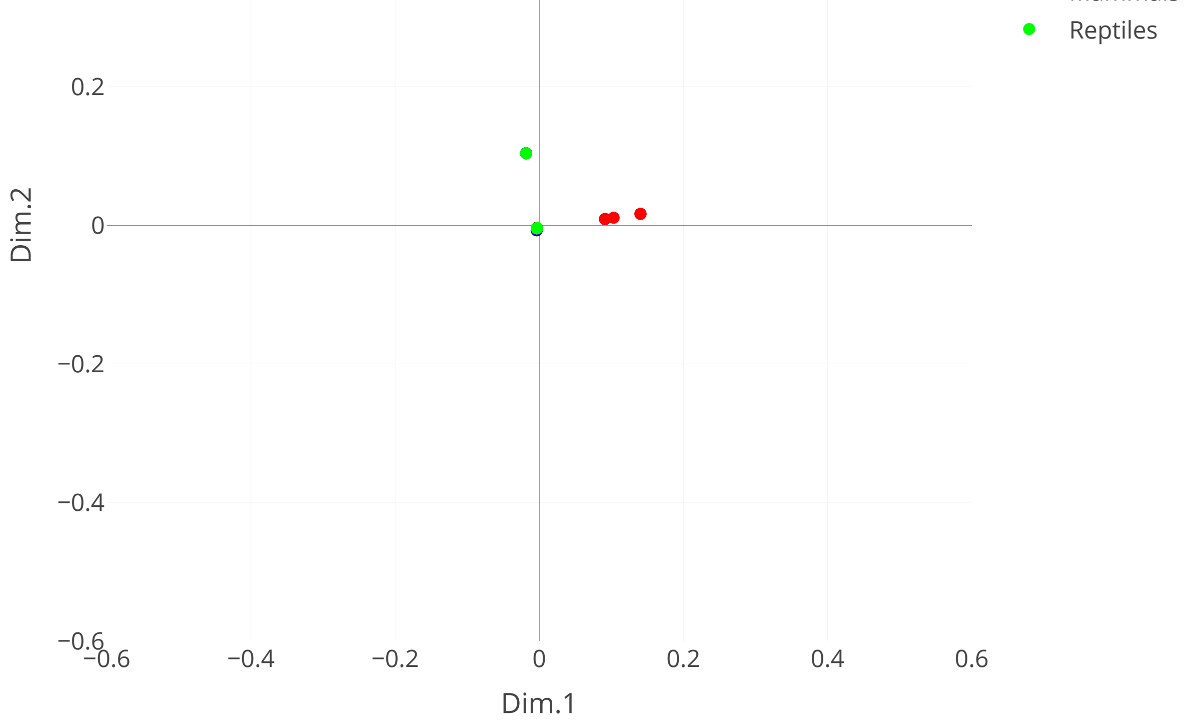
Reptiles
0.2
0
−0.2
−0.4
−0.6
Dim.2
−0.6
−0.4
−0.2
0
0.2
0.4
0.6
Dim.1

MDS for SP

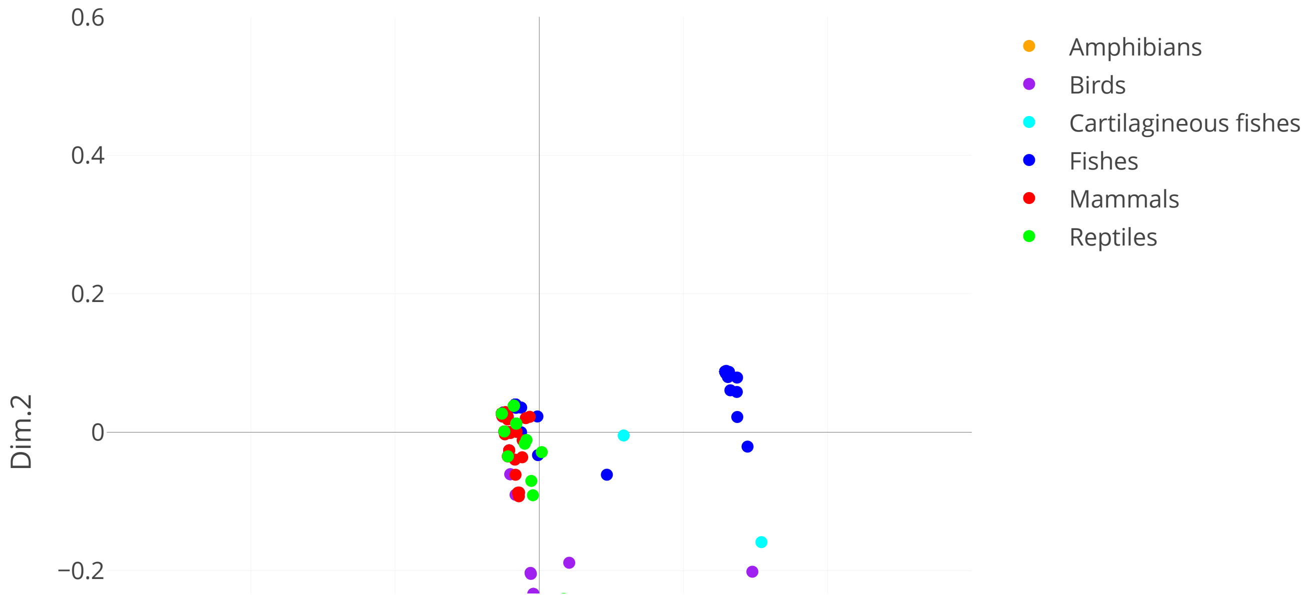
Amphibians
Birds
Cartilagineous fishes
Fishes
Mammals
Reptiles
0.6
0.4
0.2
0
−0.2
Dim.2

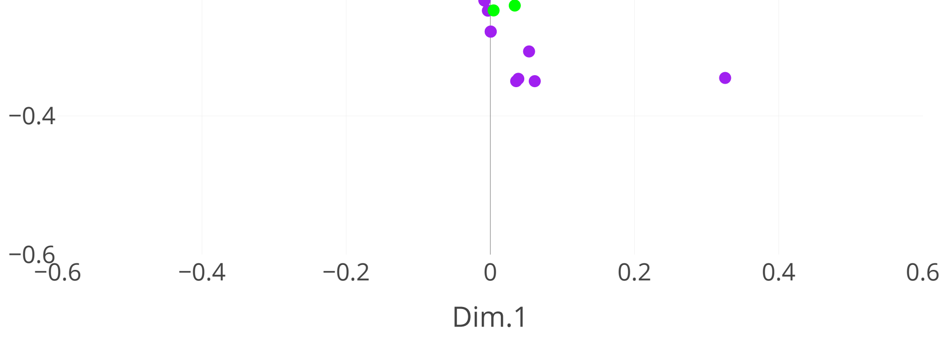

−0.4
−0.6
−0.6
−0.4
−0.2
0
0.2
0.4
0.6
Dim.1

MDS for WL

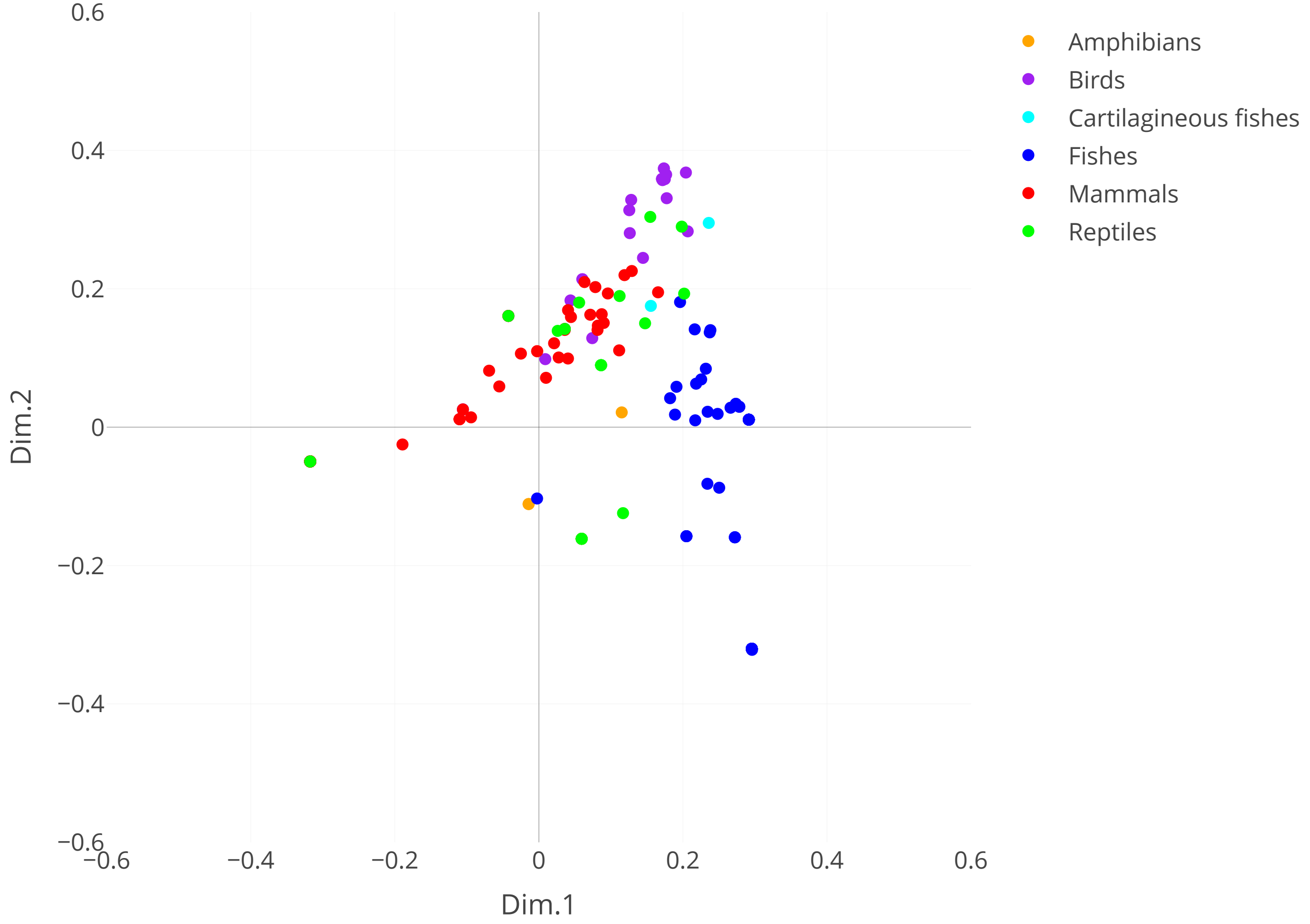

0.6
0.4
0.2
0
−0.2
−0.4
−0.6
Dim.2
−0.6
−0.4
−0.2
0
0.2
0.4
0.6
Dim.1
Amphibians
Birds
Cartilagineous fishes
Fishes
Mammals
Reptiles

MDS for PM

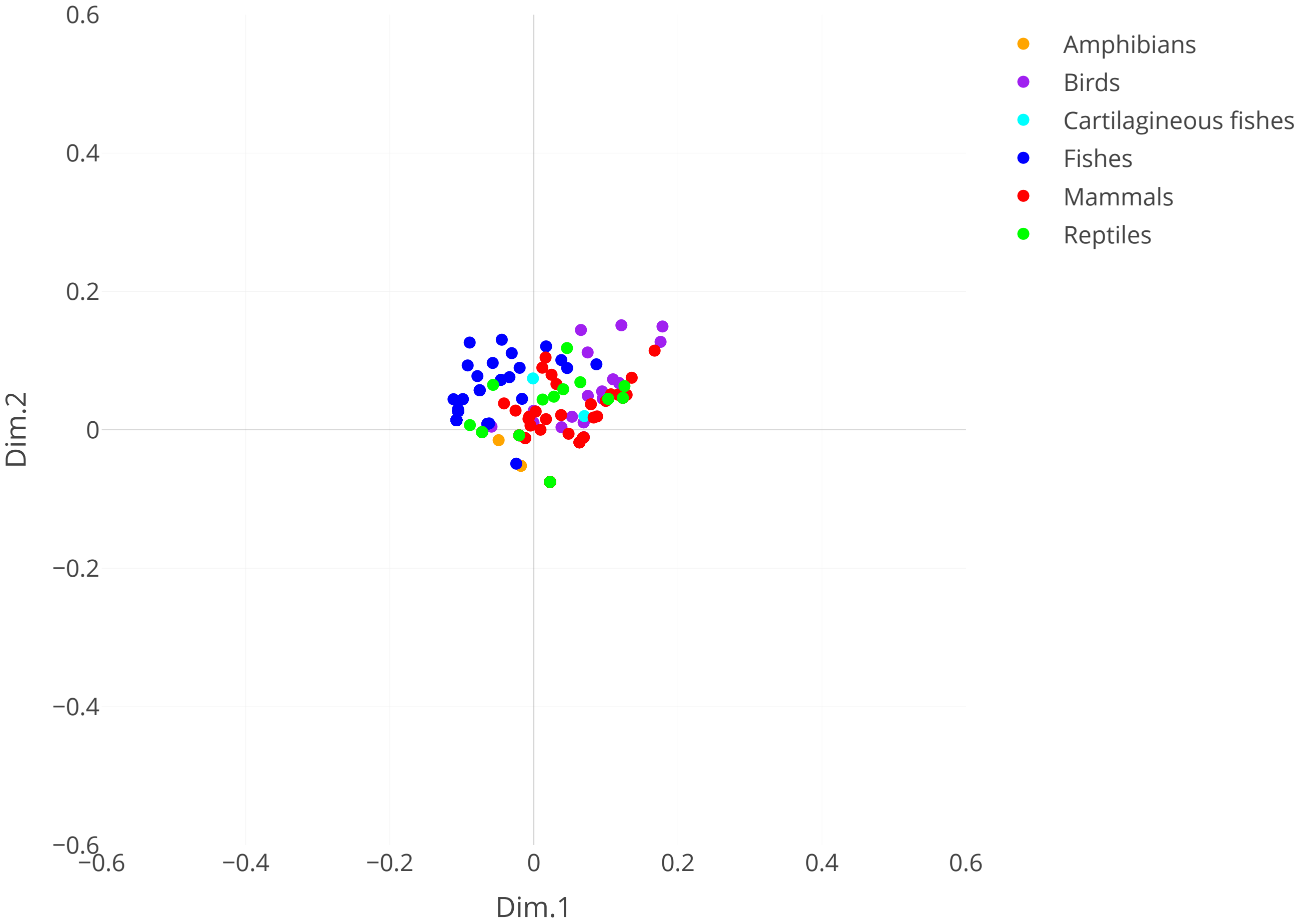

Clustering

6-means clustering for VH

```
##                          Cluster
## Real group                 1  2  3  4  5  6
##   Amphibians               0  0  0  1  0  2
```

```
##    Birds                   5  0  6  1 12  7
##    Cartilagineous fishes   0  1  1  0  0  0
##    Fishes                  5 15  2 39  0  1
##    Mammals                 3  4 11  2 16 58
##    Reptiles                2  1  7  5  3  2
```

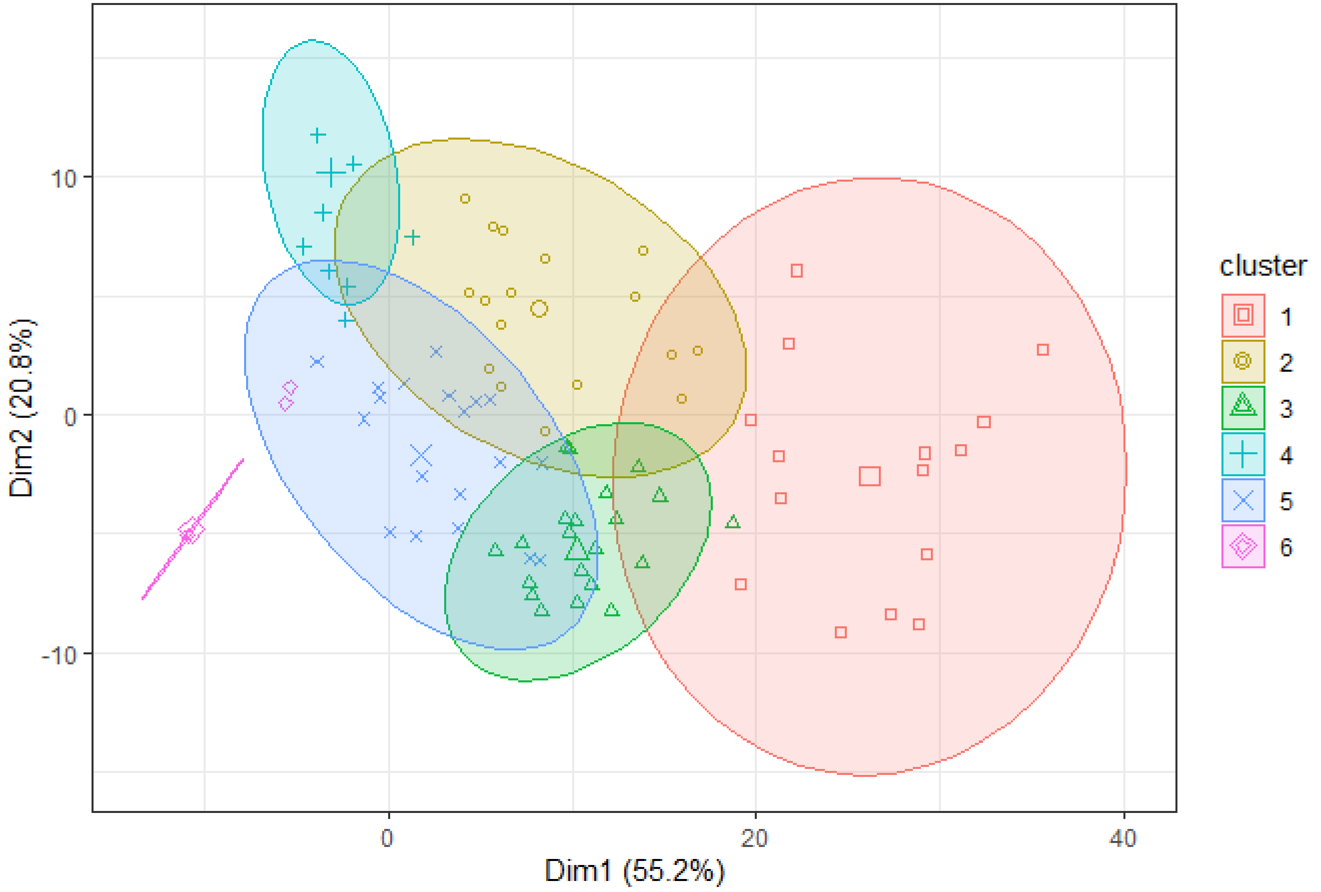

Organisms classified within cluster 1

```
##  [1] "vlg"  "chx"  "ajm"  "apla" "acyg" "acun" "padl" "aam"  "cpoo" "ggn"
## [11] "ccar" "cgob" "plep" "aoce" "loc"
```

Organisms classified within cluster 2

```
##  [1] "ggo"  "cjc"  "pgig" "gas"  "tst"  "ipu"  "phyp" "amex" "eee"  "tng" 
## [11] "lco"  "ncc"  "msam" "ola"  "pret" "csem" "otw"  "salp" "pspa" "arut"
## [21] "cmk"
```

Organisms classified within cluster 3

```
##  [1] "csab" "pteh" "umr"  "oro"  "cfr"  "lve"  "pcad" "myb"  "pkl"  "rfq" 
## [11] "shr"  "tgu"  "gfr"  "ccae" "ccw"  "fch"  "egz"  "pss"  "cabi" "acs" 
## [21] "pvt"  "sund" "pbi"  "vko"  "cud"  "hcq"  "rtp"
```

Organisms classified within cluster 4

```
##  [1] "sbq"  "vvp"  "lsr"  "amj"  "pmur" "pmua" "zvi"  "gja"  "xtr"  "dre" 
## [11] "srx"  "sanh" "sgh"  "caua" "tru"  "ely"  "sluc" "ecra" "pflv" "gat" 
## [21] "ppug" "mze"  "onl"  "oau"  "oml"  "xma"  "xco"  "xhe"  "cvg"  "ctul"
## [31] "nfu"  "kmr"  "alim" "pov"  "ssen" "lcf"  "sdu"  "slal" "xgl"  "bpec"
## [41] "malb" "sasa" "omy"  "snh"  "els"  "sfm"  "pki"  "aang"
```

Organisms classified within cluster 5

```
##  [1] "caty" "ocu"  "cfa"  "aml"  "eju"  "bta"  "biu"  "ssc"  "mmyo" "shon"
## [11] "pale" "ray"  "mjv"  "lav"  "mdo"  "oaa"  "pcoc" "nmel" "scan" "fab" 
## [21] "pmaj" "etl"  "fpg"  "clv"  "nni"  "arow" "npd"  "dne"  "cmy"  "tsr" 
## [31] "pgut"
```

Organisms classified within cluster 6

```
##  [1] "hsa"  "ptr"  "pps"  "pon"  "nle"  "mcc"  "mcf"  "panu" "rro"  "rbb"
## [11] "tfn"  "mmur" "mmu"  "mcal" "mpah" "rno"  "mcoc" "mun"  "cge"  "pleu"
## [21] "ngi"  "hgl"  "ccan" "opi"  "tup"  "uah"  "elk"  "mpuf" "mlx"  "fca"
## [31] "pyu"  "pbg"  "ptg"  "ppad" "aju"  "hhv"  "bom"  "bbub" "oas"  "oda"
## [41] "ccad" "cbai" "cdk"  "bacu" "oor"  "dle"  "ecb"  "epz"  "eai"  "myd"
## [51] "mna"  "hai"  "dro"  "pdic" "mmf"  "tod"  "tmu"  "pcw"  "gga"  "mgp"
## [61] "cjo"  "pmoa" "otc"  "pruf" "phi"  "asn"  "cpic" "xla"  "npr"  "lcm"
```

6-means clustering for SP

```
##                         Cluster
## Real group               1  2  3  4  5  6
##   Amphibians             0  0  0  0  1  2
##   Birds                 10  5  8  0  1  7
##   Cartilagineous fishes  1  0  0  1  0  0
##   Fishes                 1  6  0 15 39  1
##   Mammals               12  3 15  4  2 58
##   Reptiles               8  2  2  1  5  2
```

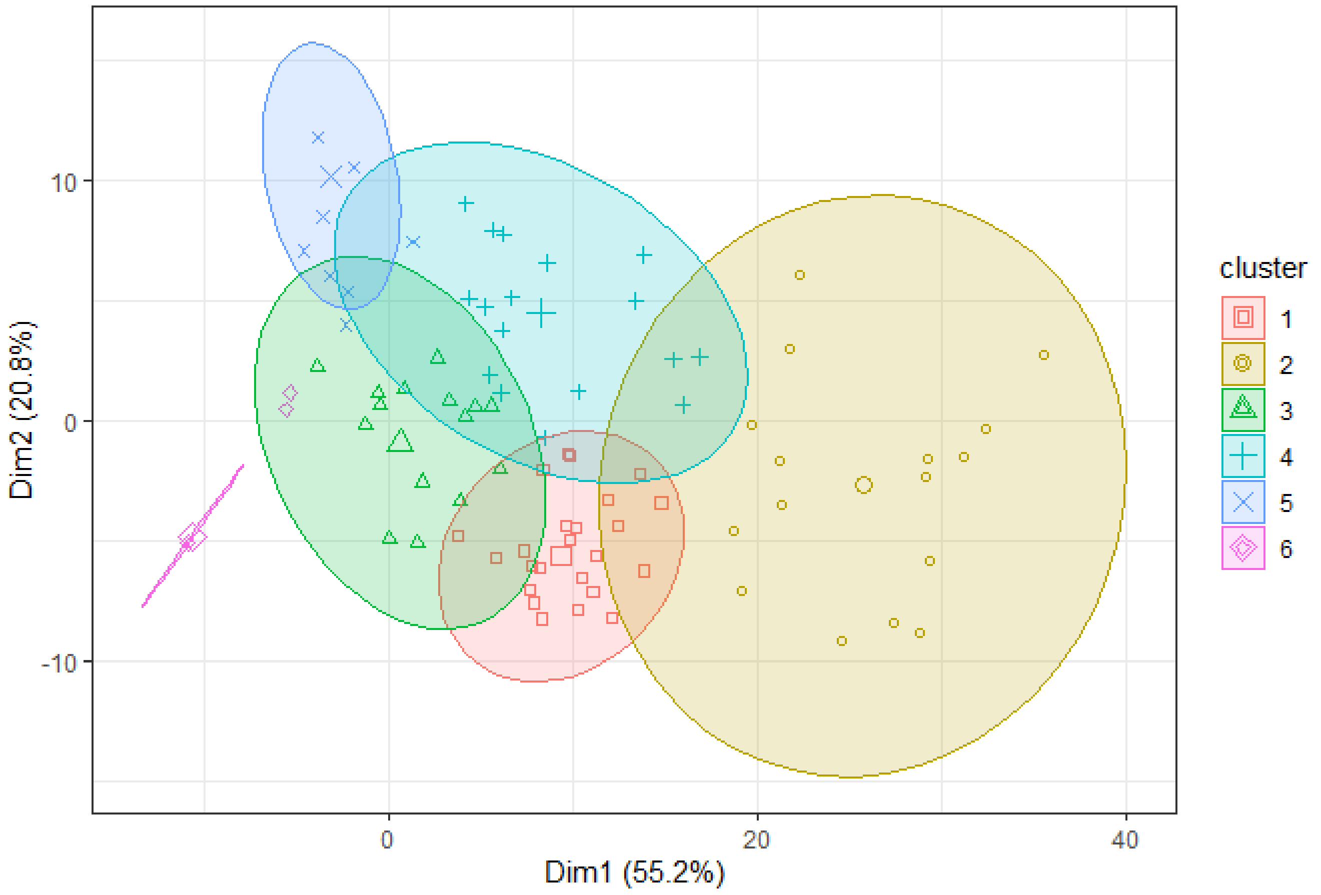

Organisms classified within cluster 1

```
##  [1] "csab" "pteh" "umr"  "oro"  "cfr"  "lve"  "pcad" "myb"  "pkl"  "rfq"
## [11] "mdo"  "shr"  "tgu"  "gfr"  "ccae" "ccw"  "fpg"  "fch"  "egz"  "nni"
## [21] "arow" "dne"  "pss"  "cabi" "acs"  "pvt"  "sund" "pbi"  "tsr"  "vko"
## [31] "hcq"  "rtp"
```

Organisms classified within cluster 2

```
##  [1] "vlg"  "chx"  "ajm"  "apla" "acyg" "acun" "padl" "aam"  "cpoo" "ggn"
## [11] "ccar" "cgob" "plep" "cud"  "aoce" "loc"
```

Organisms classified within cluster 3

```
##  [1] "caty" "ocu"  "cfa"  "aml"  "eju"  "bta"  "biu"  "ssc"  "mmyo" "shon"
## [11] "pale" "ray"  "mjv"  "lav"  "oaa"  "pcoc" "nmel" "scan" "fab"  "pmaj"
## [21] "etl"  "clv"  "npd"  "cmy"  "pgut"
```

Organisms classified within cluster 4

```
##  [1] "ggo"  "cjc"  "pgig" "gas"  "tst"  "ipu"  "phyp" "amex" "eee"  "tng"
## [11] "lco"  "ncc"  "msam" "ola"  "pret" "csem" "otw"  "salp" "pspa" "arut"
## [21] "cmk"
```

Organisms classified within cluster 5

```
##  [1] "sbq"  "vvp"  "lsr"  "amj"  "pmur" "pmua" "zvi"  "gja"  "xtr"  "dre"
## [11] "srx"  "sanh" "sgh"  "caua" "tru"  "ely"  "sluc" "ecra" "pflv" "gat"
## [21] "ppug" "mze"  "onl"  "oau"  "oml"  "xma"  "xco"  "xhe"  "cvg"  "ctul"
## [31] "nfu"  "kmr"  "alim" "pov"  "ssen" "lcf"  "sdu"  "slal" "xgl"  "bpec"
## [41] "malb" "sasa" "omy"  "snh"  "els"  "sfm"  "pki"  "aang"
```

Organisms classified within cluster 6

```
##  [1] "hsa"  "ptr"  "pps"  "pon"  "nle"  "mcc"  "mcf"  "panu" "rro"  "rbb"
## [11] "tfn"  "mmur" "mmu"  "mcal" "mpah" "rno"  "mcoc" "mun"  "cge"  "pleu"
## [21] "ngi"  "hgl"  "ccan" "opi"  "tup"  "uah"  "elk"  "mpuf" "mlx"  "fca"
## [31] "pyu"  "pbg"  "ptg"  "ppad" "aju"  "hhv"  "bom"  "bbub" "oas"  "oda"
## [41] "ccad" "cbai" "cdk"  "bacu" "oor"  "dle"  "ecb"  "epz"  "eai"  "myd"
## [51] "mna"  "hai"  "dro"  "pdic" "mmf"  "tod"  "tmu"  "pcw"  "gga"  "mgp"
## [61] "cjo"  "pmoa" "otc"  "pruf" "phi"  "asn"  "cpic" "xla"  "npr"  "lcm"
```

6-means clustering for WL

```
##                          Cluster
## Real group                1  2  3  4  5  6
##   Amphibians              0  0  0  0  2  1
##   Birds                   4  0 12  5  7  3
##   Cartilagineous fishes   0  1  1  0  0  0
##   Fishes                  2 15  0  5  1 39
##   Mammals                10  4 15  3 58  4
##   Reptiles                7  1  2  2  2  6
```

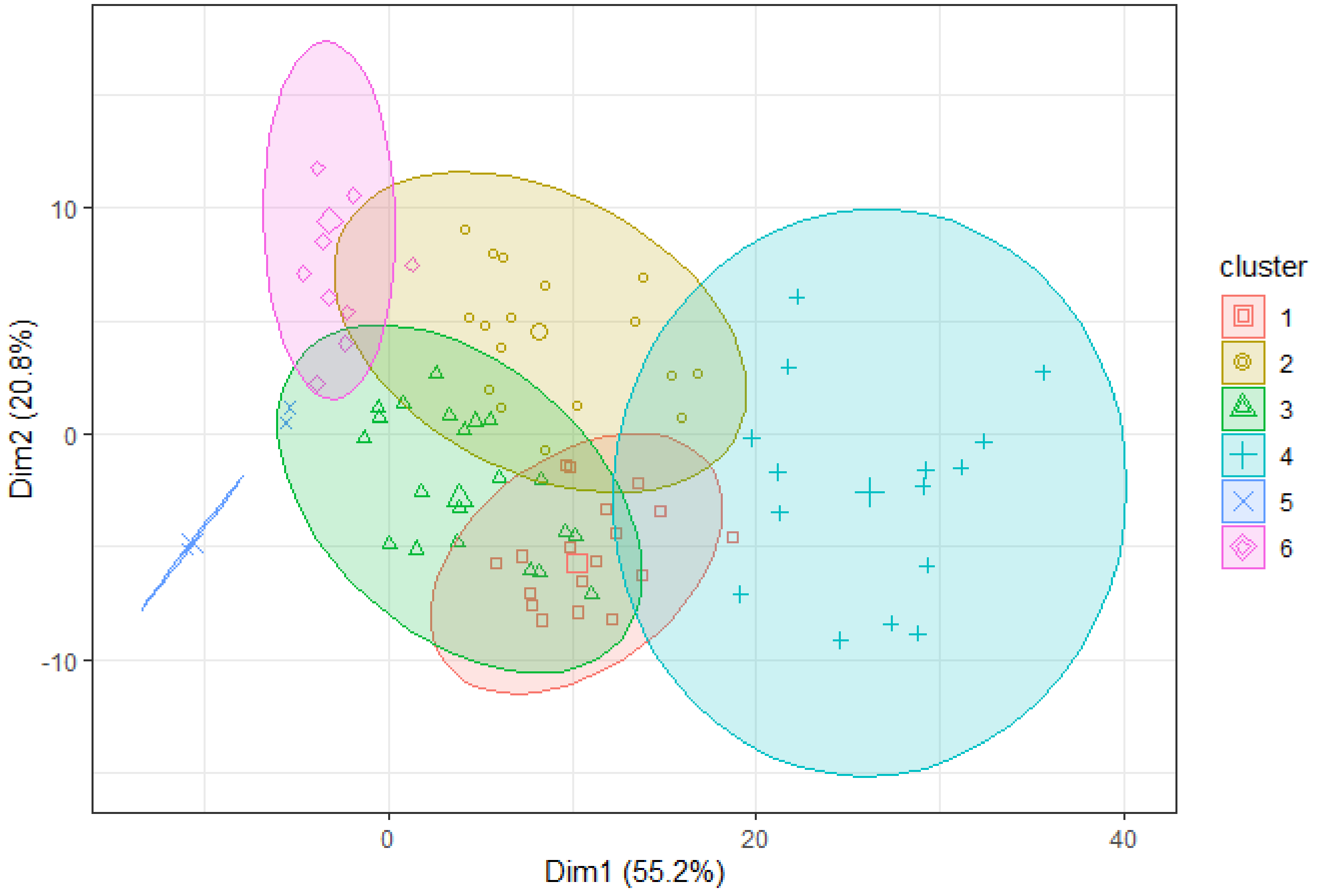

Organisms classified within cluster 1

```
##  [1] "csab" "umr"  "oro"  "cfr"  "lve"  "pcad" "myb"  "pkl"  "rfq"  "shr" 
## [11] "tgu"  "ccw"  "fch"  "egz"  "pss"  "cabi" "acs"  "pvt"  "sund" "pbi" 
## [21] "vko"  "cud"  "hcq"
```

Organisms classified within cluster 2

```
##  [1] "ggo"  "cjc"  "pgig" "gas"  "tst"  "ipu"  "phyp" "amex" "eee"  "tng" 
## [11] "lco"  "ncc"  "msam" "ola"  "pret" "csem" "otw"  "salp" "pspa" "arut"
## [21] "cmk"
```

Organisms classified within cluster 3

```
##  [1] "caty" "pteh" "ocu"  "cfa"  "aml"  "eju"  "biu"  "ssc"  "mmyo" "shon"
## [11] "pale" "ray"  "mjv"  "mdo"  "oaa"  "nmel" "scan" "gfr"  "fab"  "pmaj"
## [21] "ccae" "etl"  "fpg"  "nni"  "arow" "npd"  "dne"  "tsr"  "pgut" "rtp"
```

Organisms classified within cluster 4

```
##  [1] "vlg"  "chx"  "ajm"  "apla" "acyg" "acun" "padl" "aam"  "cpoo" "ggn" 
## [11] "ccar" "cgob" "plep" "aoce" "loc"
```

Organisms classified within cluster 5

```
##  [1] "hsa"  "ptr"  "pps"  "pon"  "nle"  "mcc"  "mcf"  "panu" "rro"  "rbb" 
## [11] "tfn"  "mmur" "mmu"  "mcal" "mpah" "rno"  "mcoc" "mun"  "cge"  "pleu"
## [21] "ngi"  "hgl"  "ccan" "opi"  "tup"  "uah"  "elk"  "mpuf" "mlx"  "fca" 
## [31] "pyu"  "pbg"  "ptg"  "ppad" "aju"  "hhv"  "bom"  "bbub" "oas"  "oda" 
## [41] "ccad" "cbai" "cdk"  "bacu" "oor"  "dle"  "ecb"  "epz"  "eai"  "myd"
```

```
## [51] "mna"  "hai"  "dro"  "pdic" "mmf"  "tod"  "tmu"  "pcw"  "gga"  "mgp"
## [61] "cjo"  "pmoa" "otc"  "pruf" "phi"  "asn"  "cpic" "xla"  "npr"  "lcm"
```

Organisms classified within cluster 6

```
##  [1] "sbq"  "vvp"  "bta"  "lav"  "pcoc" "lsr"  "clv"  "amj"  "cmy"  "pmur"
## [11] "pmua" "zvi"  "gja"  "xtr"  "dre"  "srx"  "sanh" "sgh"  "caua" "tru"
## [21] "ely"  "sluc" "ecra" "pflv" "gat"  "ppug" "mze"  "onl"  "oau"  "oml"
## [31] "xma"  "xco"  "xhe"  "cvg"  "ctul" "nfu"  "kmr"  "alim" "pov"  "ssen"
## [41] "lcf"  "sdu"  "slal" "xgl"  "bpec" "malb" "sasa" "omy"  "snh"  "els"
## [51] "sfm"  "pki"  "aang"
```

6-means clustering for PM

```
##                        Cluster
## Real group               1  2  3  4  5  6
##   Amphibians             2  1  0  0  0  0
##   Birds                  7  1 10  0  5  8
##   Cartilagineous fishes  0  0  1  1  0  0
##   Fishes                 1 39  1 15  6  0
##   Mammals               58  2 12  4  3 15
##   Reptiles               2  5  8  1  2  2
```

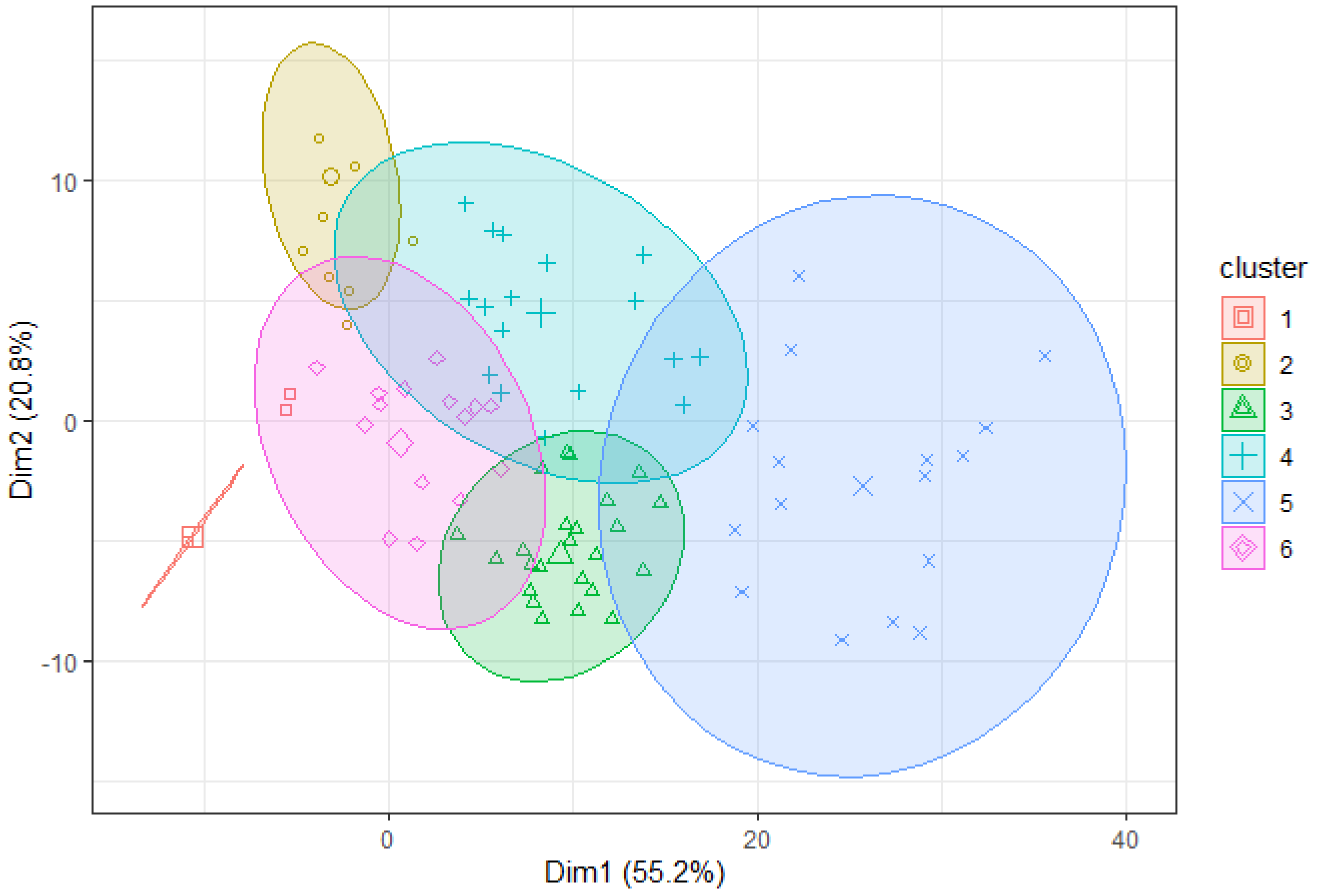

Organisms classified within cluster 1

```
##  [1] "hsa"  "ptr"  "pps"  "pon"  "nle"  "mcc"  "mcf"  "panu" "rro"  "rbb"
## [11] "tfn"  "mmur" "mmu"  "mcal" "mpah" "rno"  "mcoc" "mun"  "cge"  "pleu"
## [21] "ngi"  "hgl"  "ccan" "opi"  "tup"  "uah"  "elk"  "mpuf" "mlx"  "fca"
## [31] "pyu"  "pbg"  "ptg"  "ppad" "aju"  "hhv"  "bom"  "bbub" "oas"  "oda"
## [41] "ccad" "cbai" "cdk"  "bacu" "oor"  "dle"  "ecb"  "epz"  "eai"  "myd"
## [51] "mna"  "hai"  "dro"  "pdic" "mmf"  "tod"  "tmu"  "pcw"  "gga"  "mgp"
## [61] "cjo"  "pmoa" "otc"  "pruf" "phi"  "asn"  "cpic" "xla"  "npr"  "lcm"
```

Organisms classified within cluster 2

```
##  [1] "sbq"  "vvp"  "lsr"  "amj"  "pmur" "pmua" "zvi"  "gja"  "xtr"  "dre" 
## [11] "srx"  "sanh" "sgh"  "caua" "tru"  "ely"  "sluc" "ecra" "pflv" "gat" 
## [21] "ppug" "mze"  "onl"  "oau"  "oml"  "xma"  "xco"  "xhe"  "cvg"  "ctul"
## [31] "nfu"  "kmr"  "alim" "pov"  "ssen" "lcf"  "sdu"  "slal" "xgl"  "bpec"
## [41] "malb" "sasa" "omy"  "snh"  "els"  "sfm"  "pki"  "aang"
```

Organisms classified within cluster 3

```
##  [1] "csab" "pteh" "umr"  "oro"  "cfr"  "lve"  "pcad" "myb"  "pkl"  "rfq" 
## [11] "mdo"  "shr"  "tgu"  "gfr"  "ccae" "ccw"  "fpg"  "fch"  "egz"  "nni" 
## [21] "arow" "dne"  "pss"  "cabi" "acs"  "pvt"  "sund" "pbi"  "tsr"  "vko" 
## [31] "hcq"  "rtp"
```

Organisms classified within cluster 4

```
##  [1] "ggo"  "cjc"  "pgig" "gas"  "tst"  "ipu"  "phyp" "amex" "eee"  "tng" 
## [11] "lco"  "ncc"  "msam" "ola"  "pret" "csem" "otw"  "salp" "pspa" "arut"
## [21] "cmk"
```

Organisms classified within cluster 5

```
##  [1] "vlg"  "chx"  "ajm"  "apla" "acyg" "acun" "padl" "aam"  "cpoo" "ggn" 
## [11] "ccar" "cgob" "plep" "cud"  "aoce" "loc"
```

Organisms classified within cluster 6

```
##  [1] "caty" "ocu"  "cfa"  "aml"  "eju"  "bta"  "biu"  "ssc"  "mmyo" "shon"
## [11] "pale" "ray"  "mjv"  "lav"  "oaa"  "pcoc" "nmel" "scan" "fab"  "pmaj"
## [21] "etl"  "clv"  "npd"  "cmy"  "pgut"
```
