# Supplementary material for: Exploring the expressiveness of abstract metabolic networks
Source: PLoS One. 2023 Feb 9;18(2):e0281047. doi: 10.1371/journal.pone.0281047 (PMC9910719; doi:10.1371/journal.pone.0281047)
Supplement: S8 File — Protophotophytes analyses at phylum level (second experiment). (PDF) [file pone.0281047.s008.pdf]

Protophotophytes Analysis

- Vertex hystogram (VH) kernel
  - Heatmap
  - MDS for VH
  - 3-means clustering for VH
- Shortest Path (SP) kernel
  - Heatmap
  - MDS for SP
  - 3-means clustering for SP
- Weisfeiler-Lehman (WL) kernel
  - Heatmap
  - MDS for WL
  - 3-means clustering for WL
- Pyramid match (PM) kernel
  - Heatmap
  - MDS for WL
  - 3-means clustering for PM

Vertex hystogram (VH) kernel

Heatmap

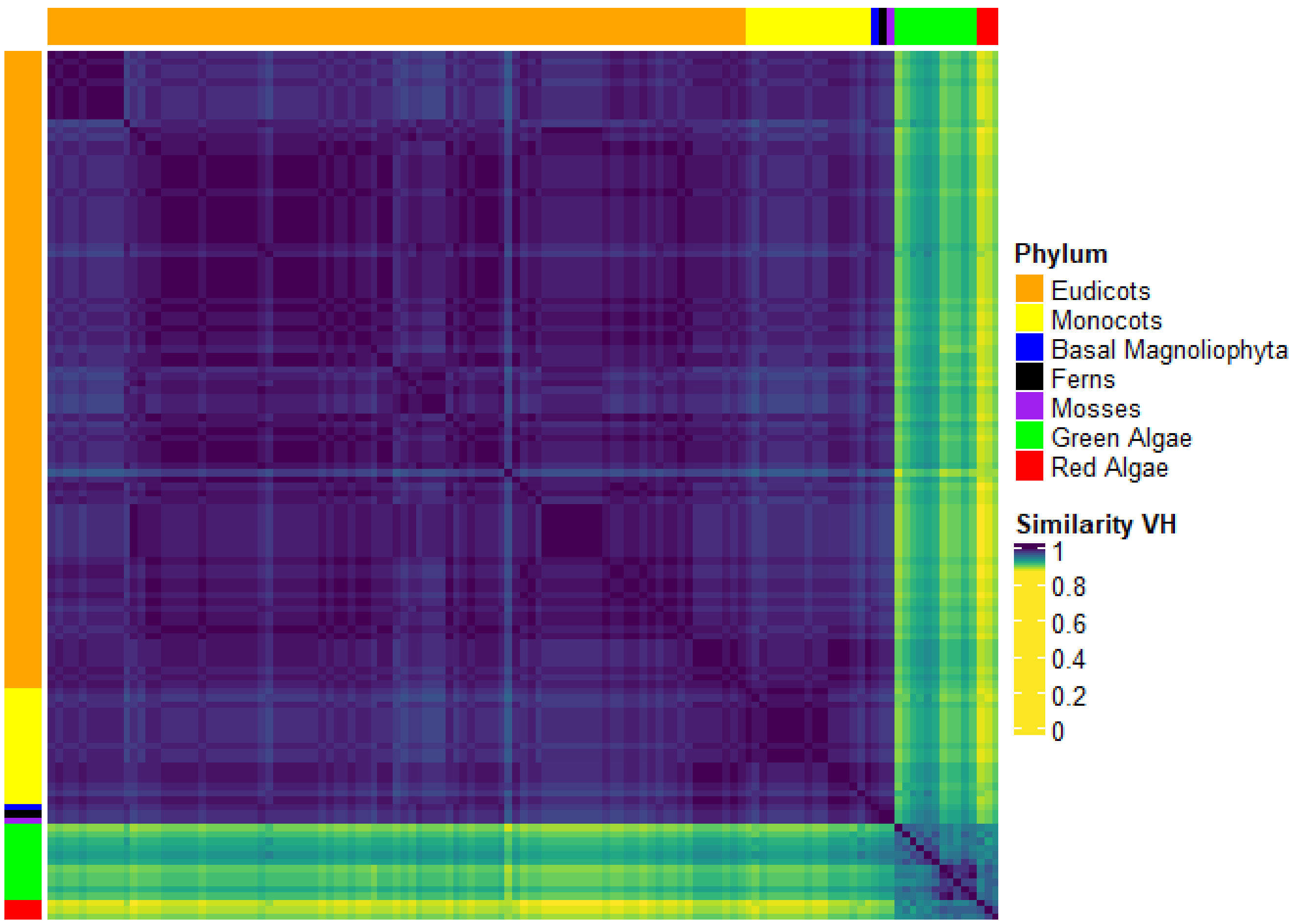

MDS for VH

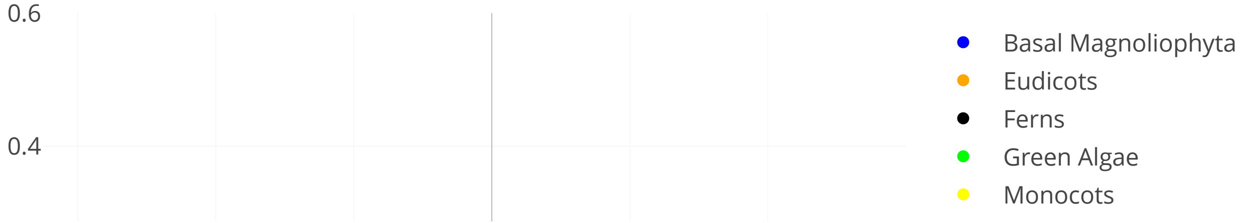

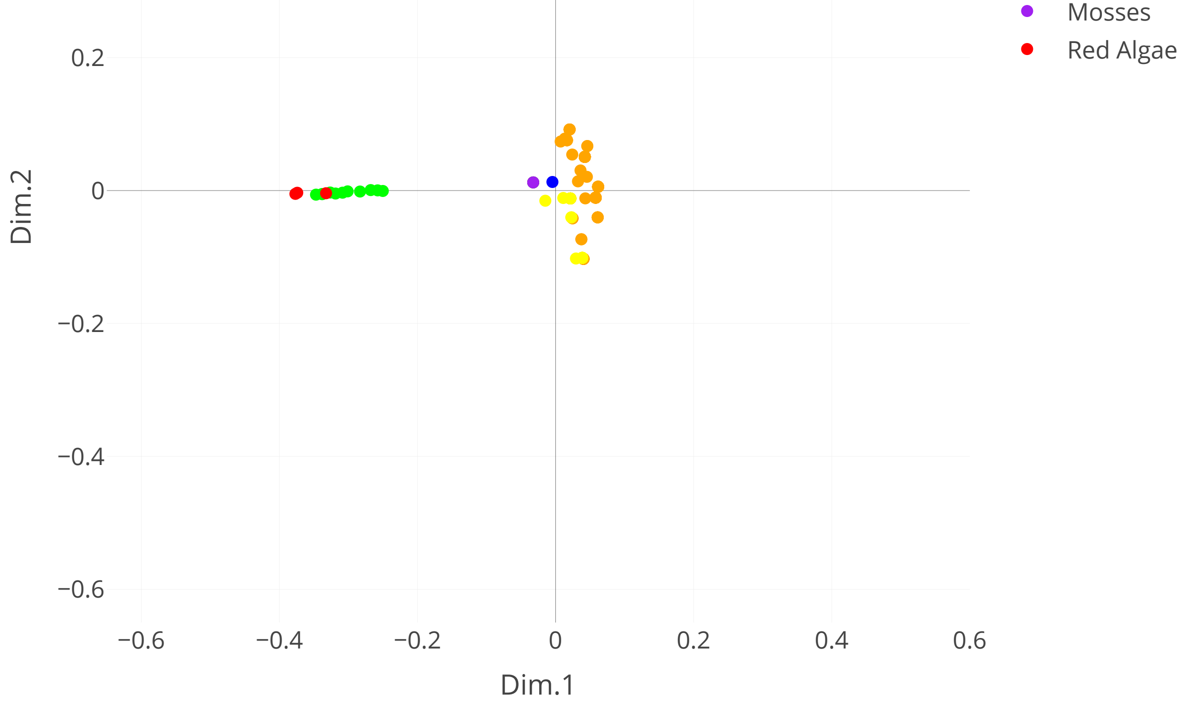

3-means clustering for VH

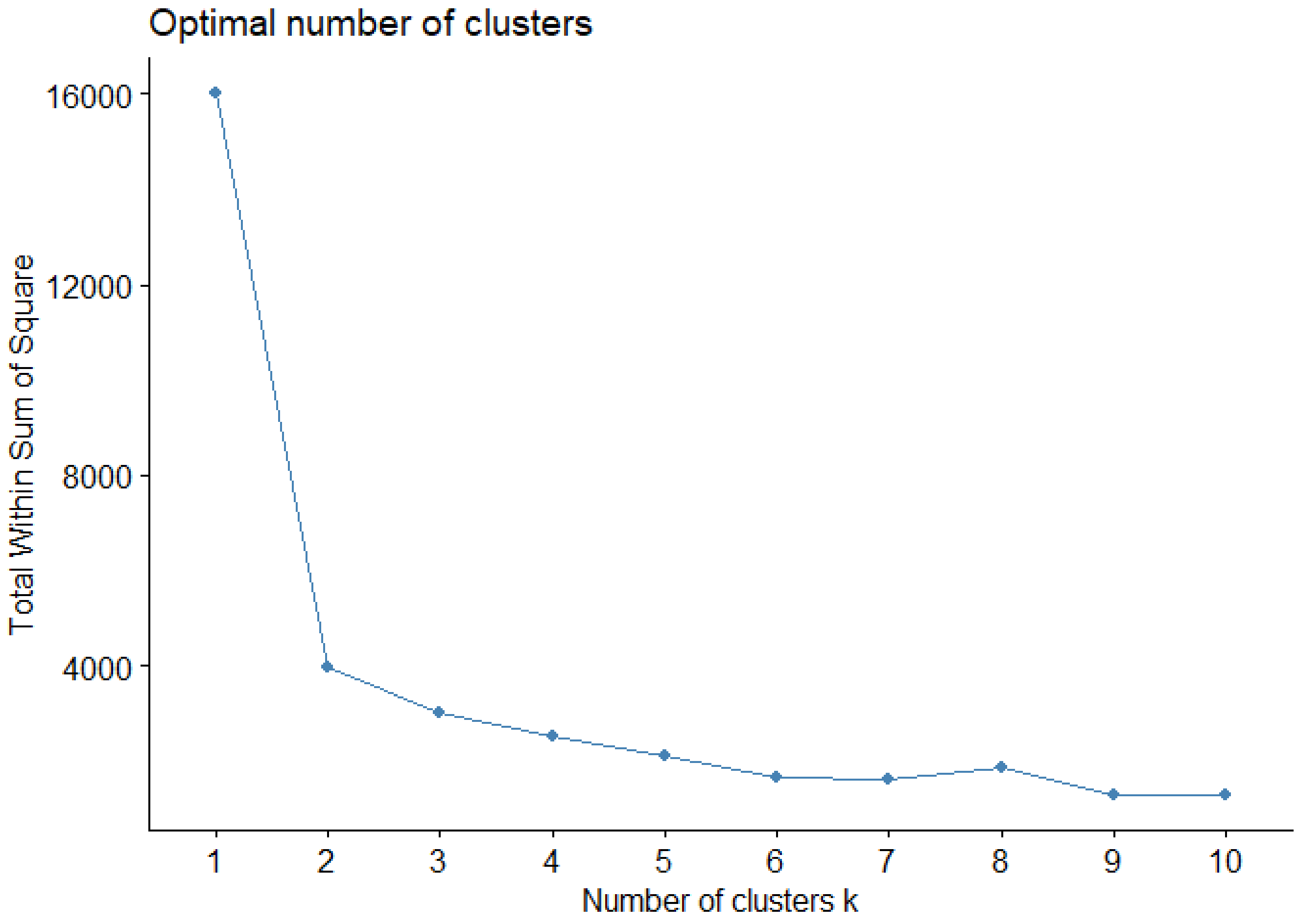


```
##                         Cluster
## Real group                1  2  3
##   Basal Magnoliophyta     0  1  0
##   Eudicots               46 47  0
##   Ferns                   0  1  0
##   Green Algae             0  0 11
##   Monocots                0 17  0
##   Mosses                  0  1  0
##   Red Algae               0  0  3
```

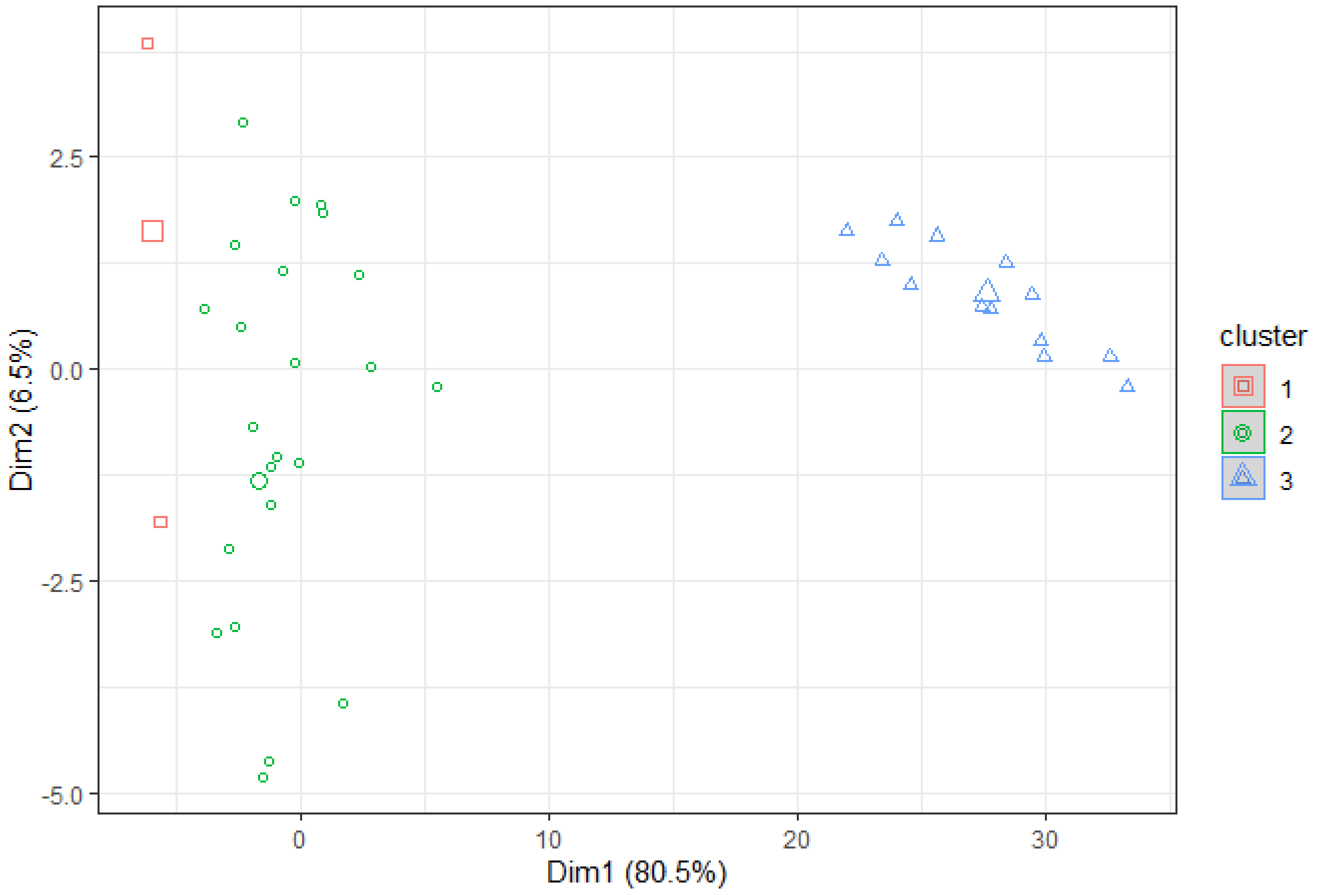

Organisms classified within cluster 1

```
##  [1] "pvy"  "minc" "tcc"  "gra"  "ghi"  "gab"  "dzi"  "egr"  "gmx"  "gsj"
## [11] "pvu"  "vra"  "var"  "vun"  "ccaj" "cam"  "lja"  "adu"  "aip"  "ahf"
## [21] "lang" "fve"  "rcn"  "pper" "pmum" "pavi" "pdul" "mdm"  "zju"  "mnt"
## [31] "rcu"  "hbr"  "mesc" "pop"  "peu"  "palz" "qlo"  "vvi"  "ini"  "oeu"
## [41] "egt"  "han"  "lsv"  "ccav" "dcr"  "csin"
```

Organisms classified within cluster 2

```
##  [1] "ath"  "aly"  "crb"  "csat" "eus"  "brp"  "bna"  "boe"  "rsz"  "thj"
## [11] "cpap" "cit"  "cic"  "aprc" "mtr"  "pxb"  "csv"  "cmo"  "bhj"  "mcha"
## [21] "cmax" "cmos" "cpep" "jcu"  "jre"  "qsu"  "twl"  "vri"  "sly"  "spen"
## [31] "sot"  "cann" "nta"  "nsy"  "nto"  "nau"  "itr"  "sind" "sspl" "ecad"
## [41] "bvg"  "soe"  "cqi"  "nnu"  "ming" "psom" "ncol" "osa"  "dosa" "obr"
## [51] "bdi"  "ats"  "tdc"  "sbi"  "zma"  "sita" "pvir" "phai" "pda"  "egu"
## [61] "mus"  "dct"  "peq"  "aof"  "atr"  "smo"  "ppp"
```

Organisms classified within cluster 3

```
##  [1] "cre"  "vcn"  "mng"  "csl"  "cvr"  "apro" "olu"  "ota"  "bpg"  "mis"
## [11] "mpp"  "cme"  "gsl"  "ccp"
```

Shortest Path (SP) kernel

Heatmap

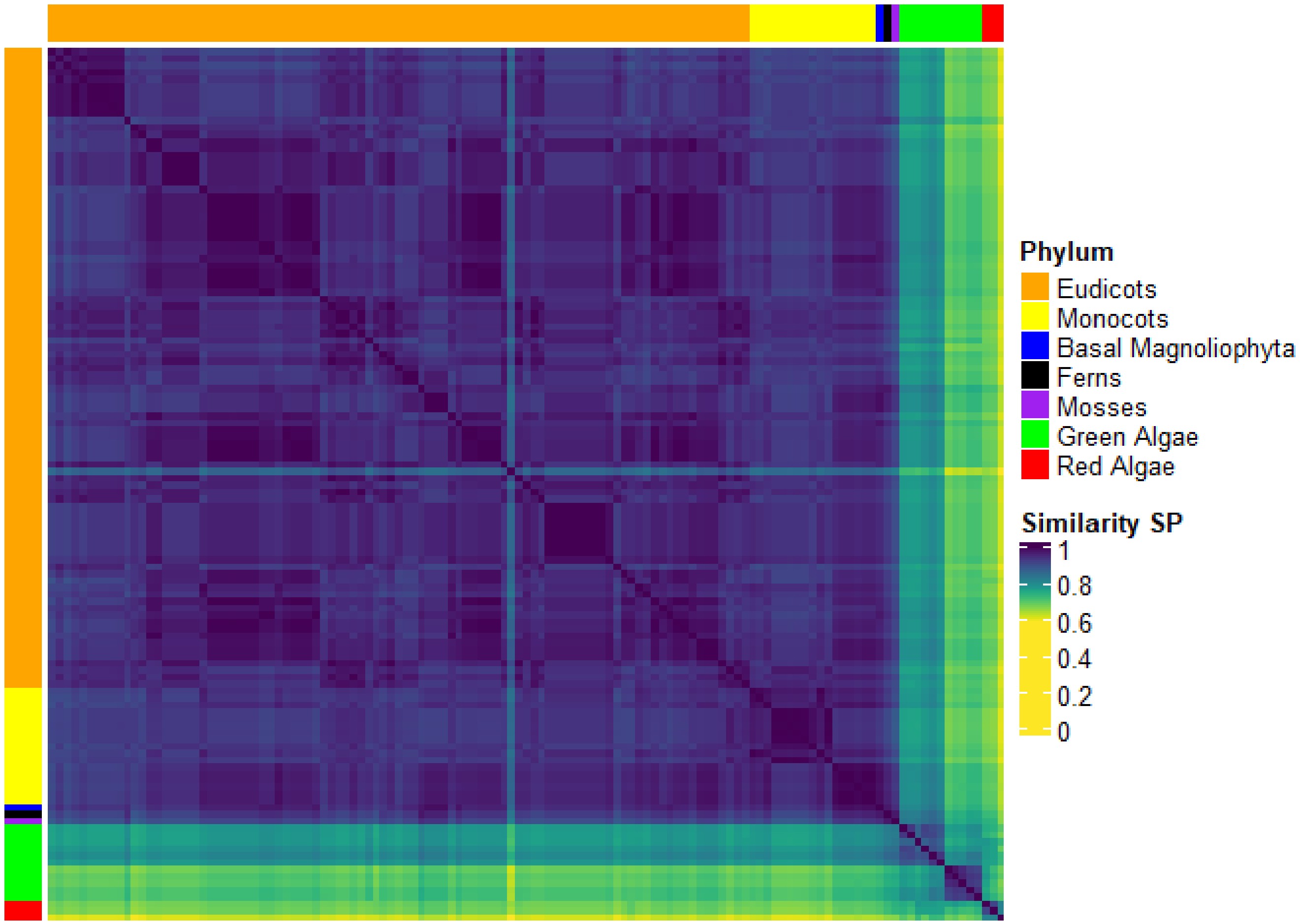
Phylum
Eudicots
Monocots
Basal Magnoliophyta
Ferns
Mosses
Green Algae
Red Algae
Similarity SP
1
0.8
0.6
0.4
0.2
0

MDS for SP

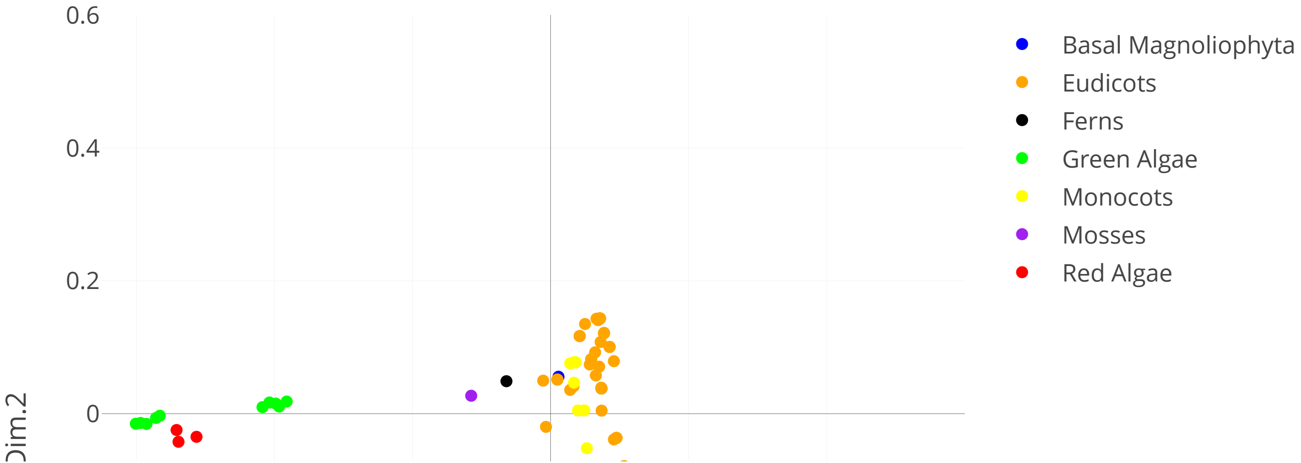
0.6
0.4
0.2
0
Dim.2
Basal Magnoliophyta
Eudicots
Ferns
Green Algae
Monocots
Mosses
Red Algae

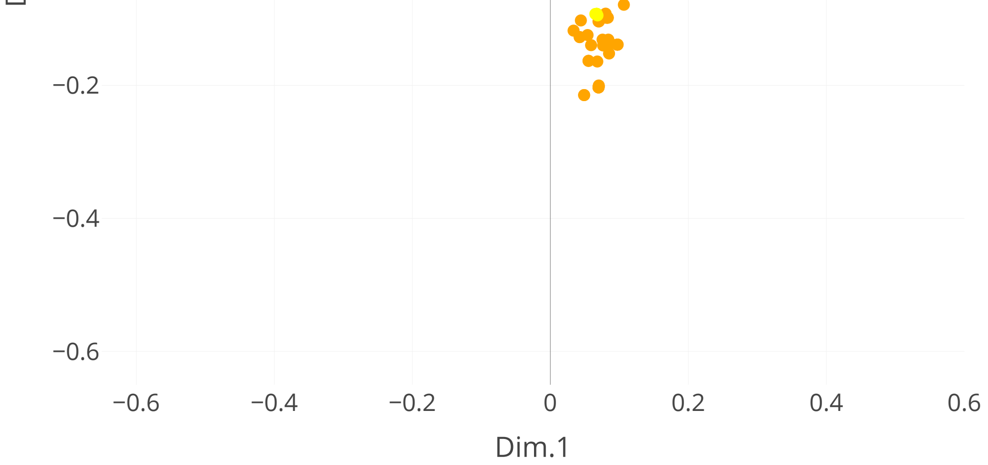

3-means clustering for SP

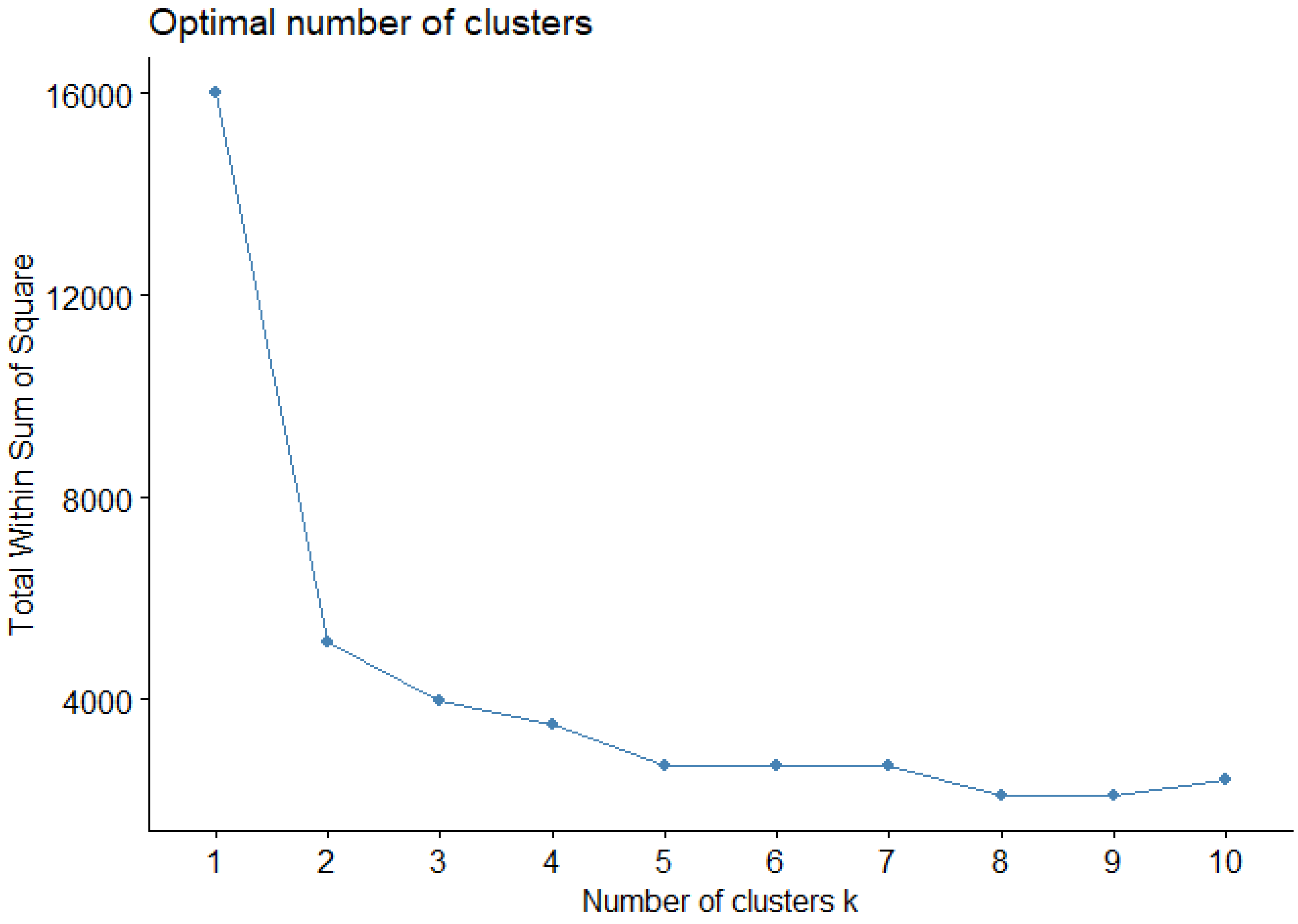

```
##                          Cluster
## Real group                1  2  3
##   Basal Magnoliophyta     0  0  1
##   Eudicots               40  0 53
##   Ferns                   0  0  1
##   Green Algae             0 11  0
##   Monocots               10  0  7
##   Mosses                  1  0  0
##   Red Algae               0  3  0
```

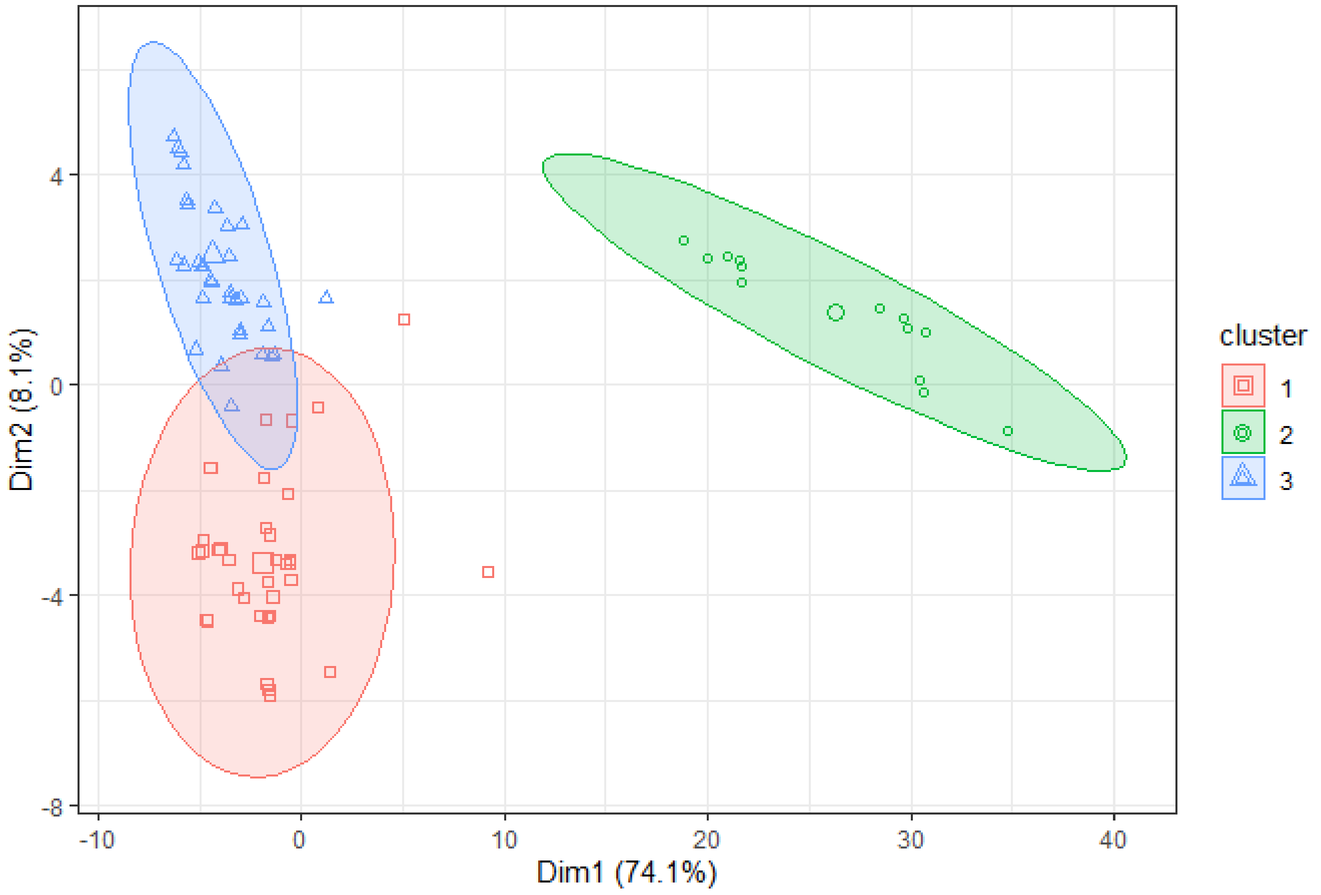

Organisms classified within cluster 1

```
##  [1] "ath"  "aly"  "crb"  "csat" "eus"  "brp"  "bna"  "boe"  "rsz"  "thj"
## [11] "cpap" "cit"  "cic"  "tcc"  "gra"  "ghi"  "gab"  "dzi"  "fve"  "rcn"
## [21] "pper" "pmum" "pavi" "pdul" "pxb"  "zju"  "mnt"  "csv"  "cmo"  "bhj"
## [31] "jre"  "qsu"  "qlo"  "vvi"  "vri"  "itr"  "nnu"  "ming" "psom" "ncol"
## [41] "osa"  "dosa" "bdi"  "ats"  "tdc"  "sbi"  "zma"  "sita" "pvir" "phai"
## [51] "ppp"
```

Organisms classified within cluster 2

```
##  [1] "cre"  "vcn"  "mng"  "csl"  "cvr"  "apro" "olu"  "ota"  "bpg"  "mis"
## [11] "mpp"  "cme"  "gsl"  "ccp"
```

Organisms classified within cluster 3

```
##  [1] "pvy"  "minc" "egr"  "gmx"  "gsj"  "pvu"  "vra"  "var"  "vun"  "ccaj"
## [11] "aprc" "mtr"  "cam"  "lja"  "adu"  "aip"  "ahf"  "lang" "mdm"  "mcha"
## [21] "cmax" "cmos" "cpep" "rcu"  "jcu"  "hbr"  "mesc" "pop"  "peu"  "palz"
## [31] "twl"  "sly"  "spen" "sot"  "cann" "nta"  "nsy"  "nto"  "nau"  "ini"
## [41] "sind" "oeu"  "egt"  "sspl" "han"  "ecad" "lsv"  "ccav" "dcr"  "csin"
## [51] "bvg"  "soe"  "cqi"  "obr"  "pda"  "egu"  "mus"  "dct"  "peq"  "aof"
## [61] "atr"  "smo"
```

Weisfeiler-Lehman (WL) kernel

Heatmap

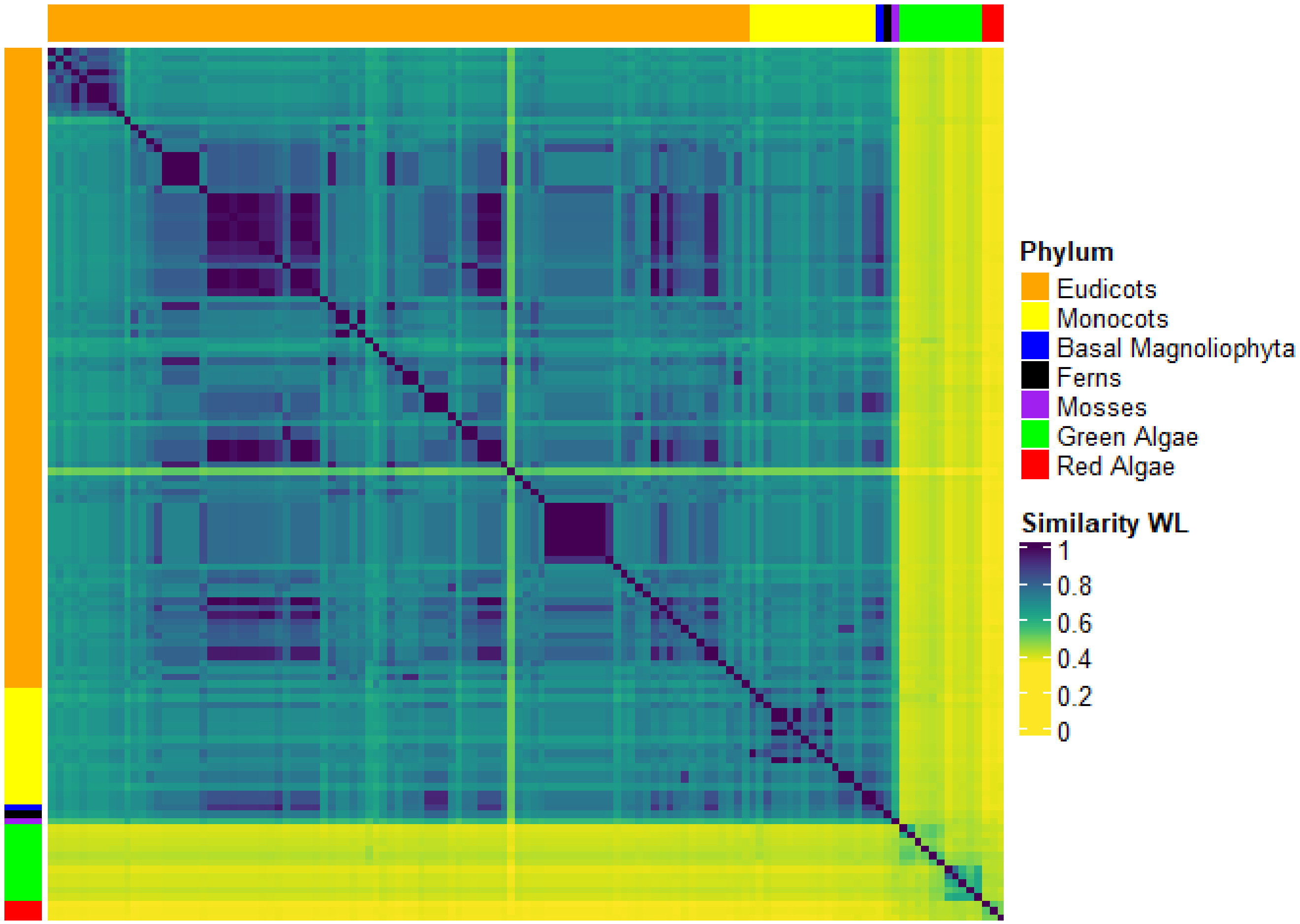
Phylum
Eudicots
Monocots
Basal Magnoliophyta
Ferns
Mosses
Green Algae
Red Algae
Similarity WL
1
0.8
0.6
0.4
0.2
0

MDS for WL

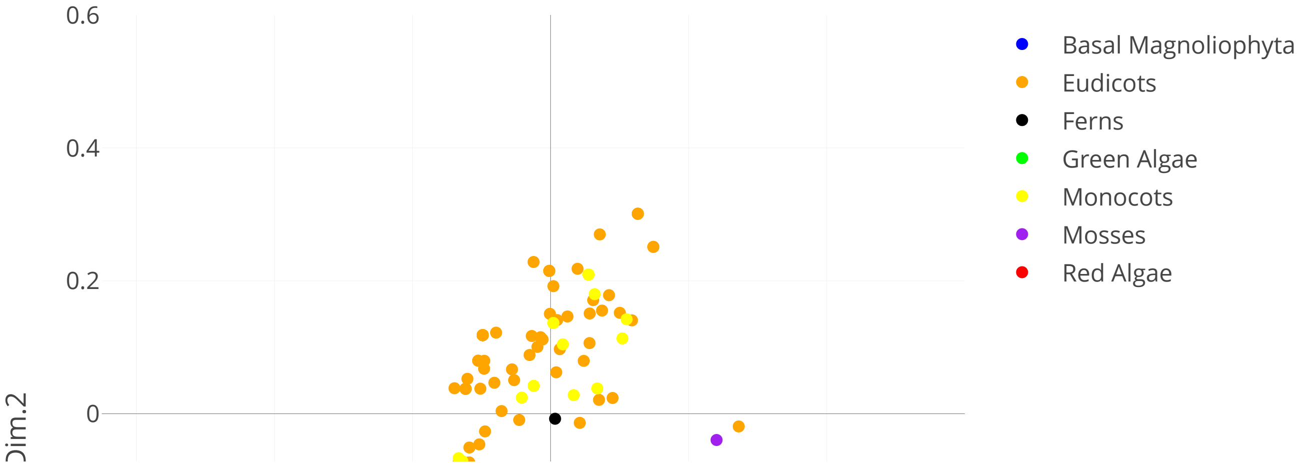
0.6
0.4
0.2
0
Dim.2
Basal Magnoliophyta
Eudicots
Ferns
Green Algae
Monocots
Mosses
Red Algae

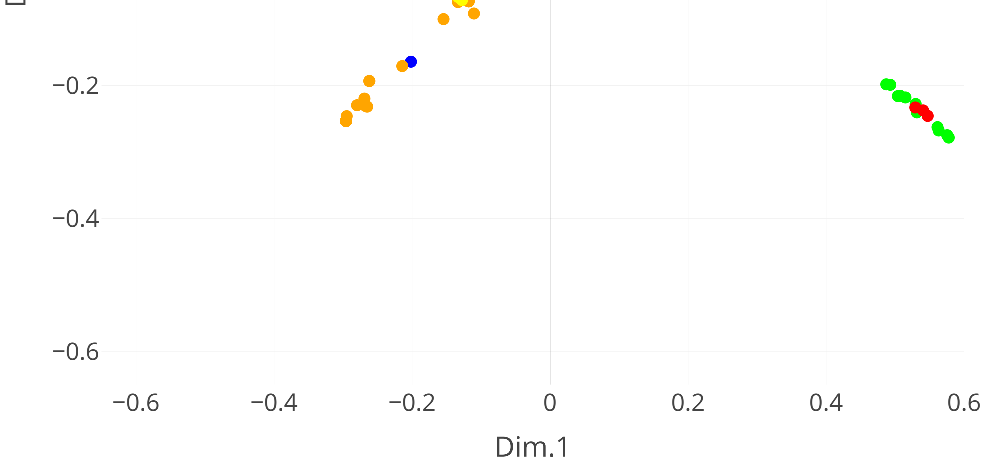

3-means clustering for WL

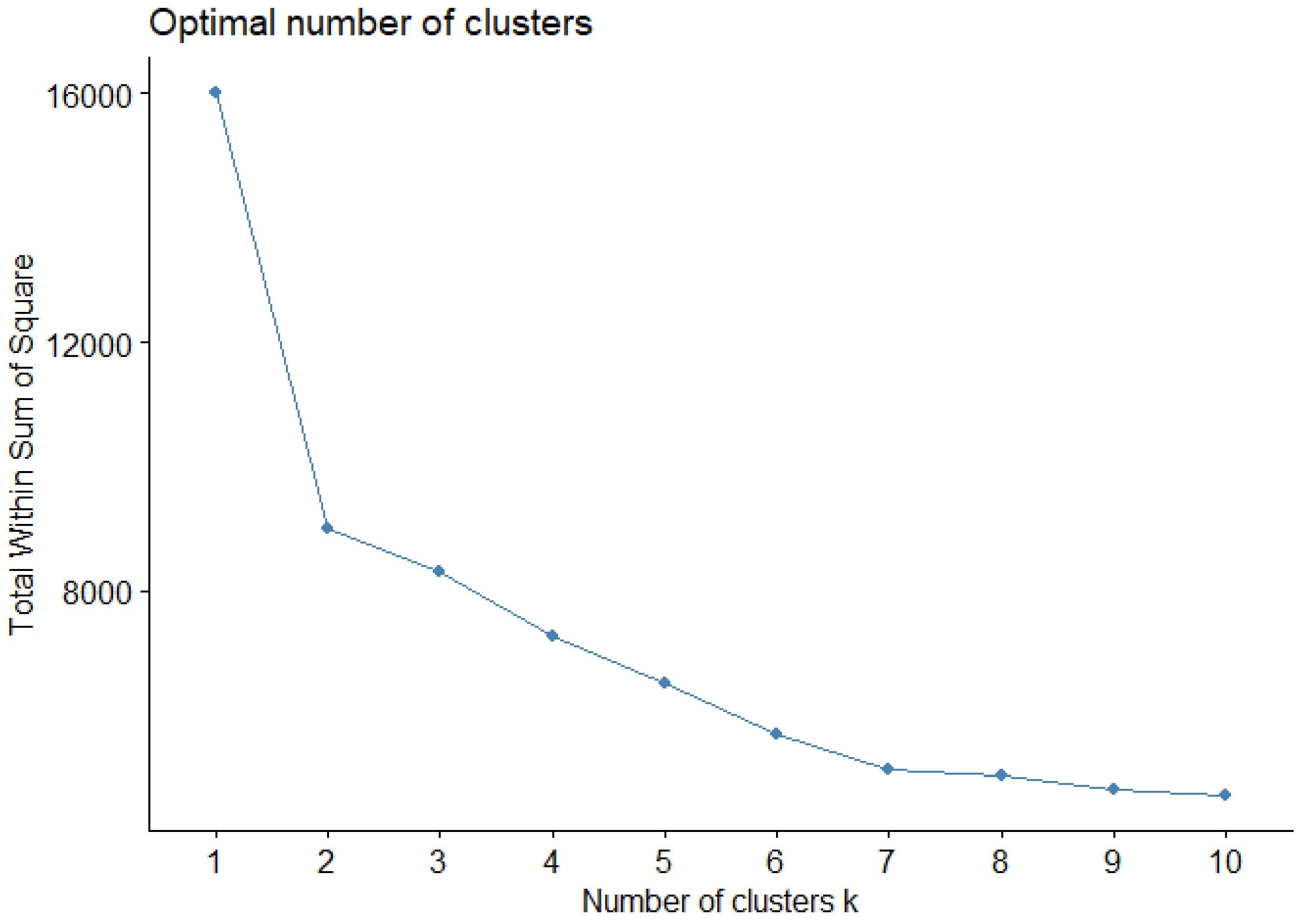

```
##                         Cluster
## Real group               1  2  3
##    Basal Magnoliophyta   1  0  0
##    Eudicots             21  1 71
##    Ferns                 0  0  1
##    Green Algae           0 11  0
##    Monocots              0  0 17
##    Mosses                0  0  1
##    Red Algae             0  3  0
```

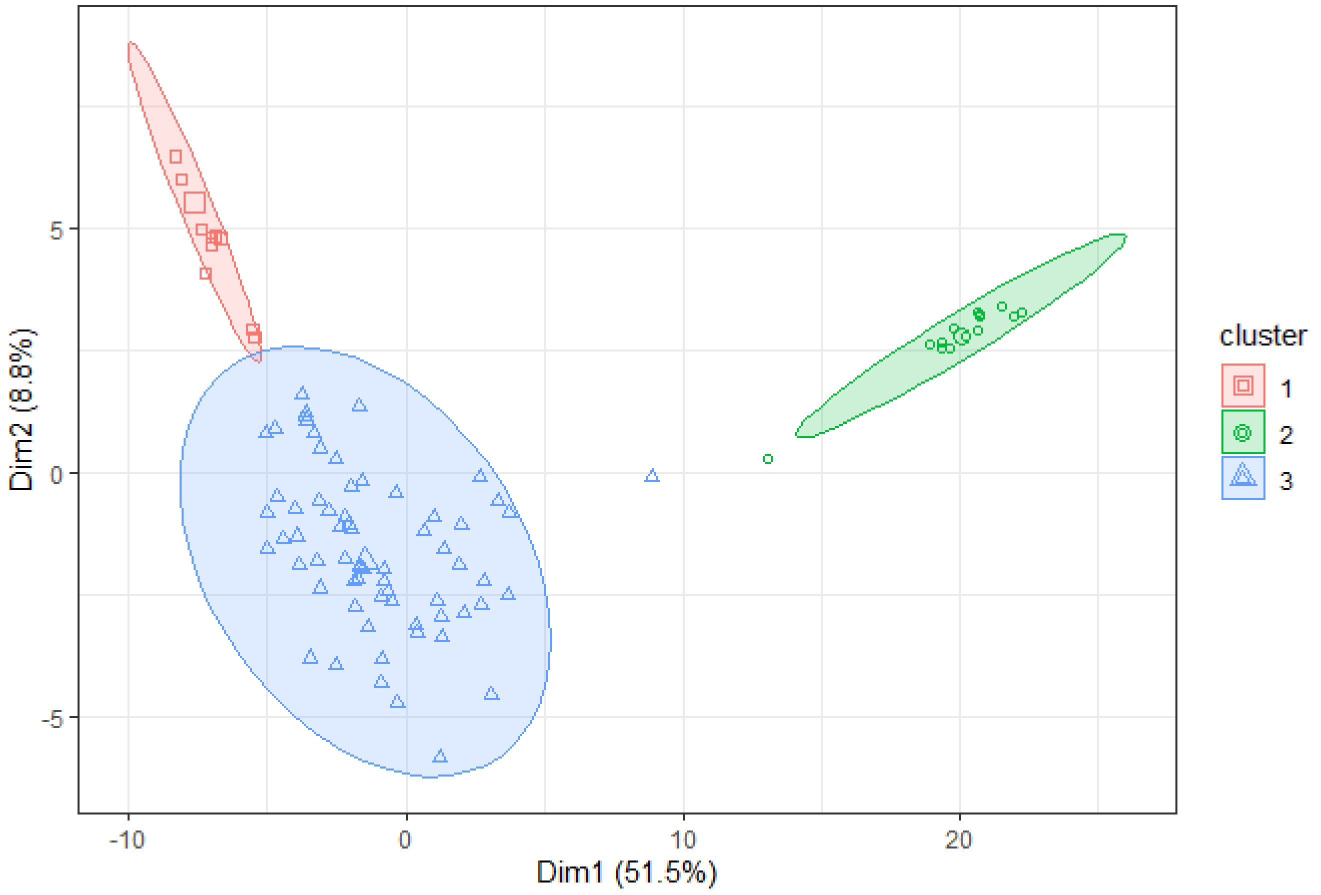

Organisms classified within cluster 1

```
##  [1] "gmx"   "gsj"   "pvu"   "vra"   "var"   "vun"   "ccaj" "aprc" "mtr"   "cam"
## [11] "adu"   "aip"   "ahf"   "lang" "pop"   "peu"   "palz" "han"   "lsv"   "soe"
## [21] "cqi"   "atr"
```

Organisms classified within cluster 2

```
##  [1] "qsu"   "cre"   "vcn"   "mng"   "csl"   "cvr"   "apro" "olu"   "ota"   "bpg"
## [11] "mis"   "mpp"   "cme"   "gsl"   "ccp"
```

Organisms classified within cluster 3

```
##  [1] "ath"   "aly"   "crb"   "csat" "eus"   "brp"   "bna"   "boe"   "rsz"   "thj"
## [11] "cpap" "cit"   "cic"   "pvy"   "minc" "tcc"   "gra"   "ghi"   "gab"   "dzi"
## [21] "egr"   "lja"   "fve"   "rcn"   "pper" "pmum" "pavi" "pdul" "mdm"   "pxb"
## [31] "zju"   "mnt"   "csv"   "cmo"   "bhj"   "mcha" "cmax" "cmos" "cpep" "rcu"
## [41] "jcu"   "hbr"   "mesc" "jre"   "qlo"   "twl"   "vvi"   "vri"   "sly"   "spen"
## [51] "sot"   "cann" "nta"   "nsy"   "nto"   "nau"   "ini"   "itr"   "sind" "oeu"
## [61] "egt"   "sspl" "ecad" "ccav" "dcr"   "csin" "bvg"   "nnu"   "ming" "psom"
## [71] "ncol" "osa"   "dosa" "obr"   "bdi"   "ats"   "tdc"   "sbi"   "zma"   "sita"
## [81] "pvir" "phai" "pda"   "egu"   "mus"   "dct"   "peq"   "aof"   "smo"   "ppp"
```

Pyramid match (PM) kernel

Heatmap

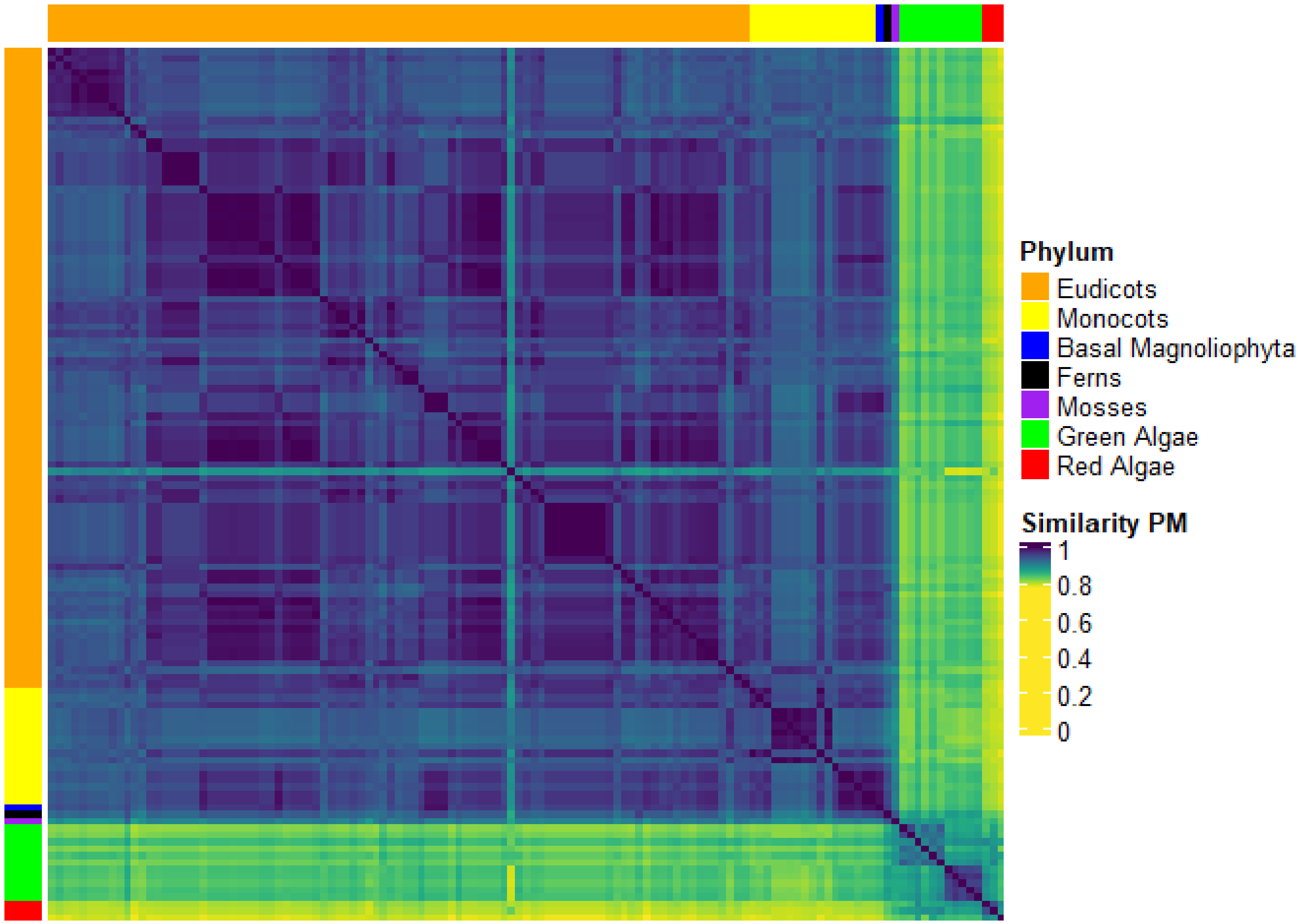
Phylum
Eudicots
Monocots
Basal Magnoliophyta
Ferns
Mosses
Green Algae
Red Algae
Similarity PM
1
0.8
0.6
0.4
0.2
0

MDS for WL

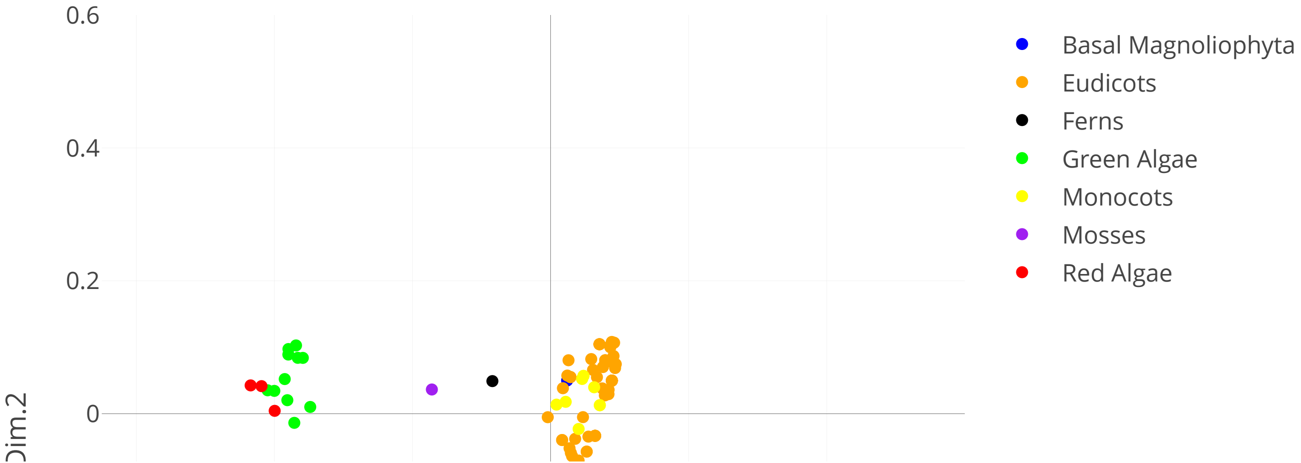
0.6
0.4
0.2
0
Dim.2
Basal Magnoliophyta
Eudicots
Ferns
Green Algae
Monocots
Mosses
Red Algae

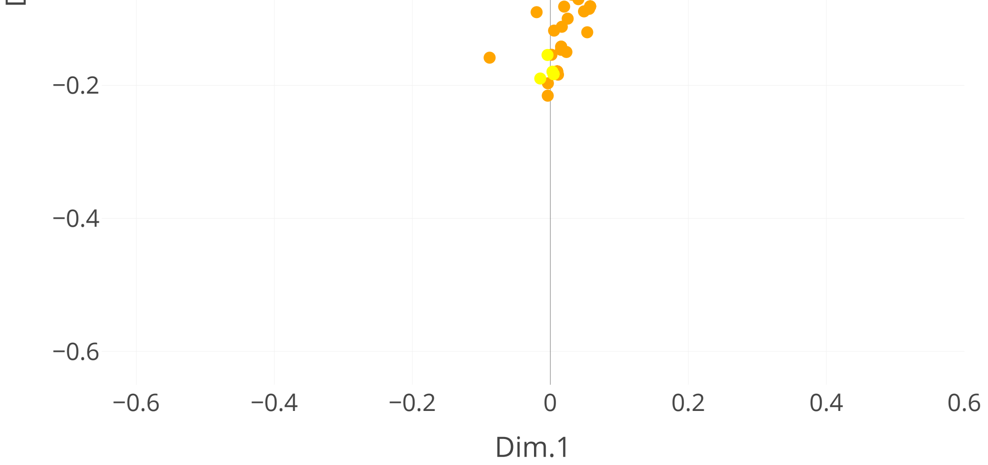

3-means clustering for PM

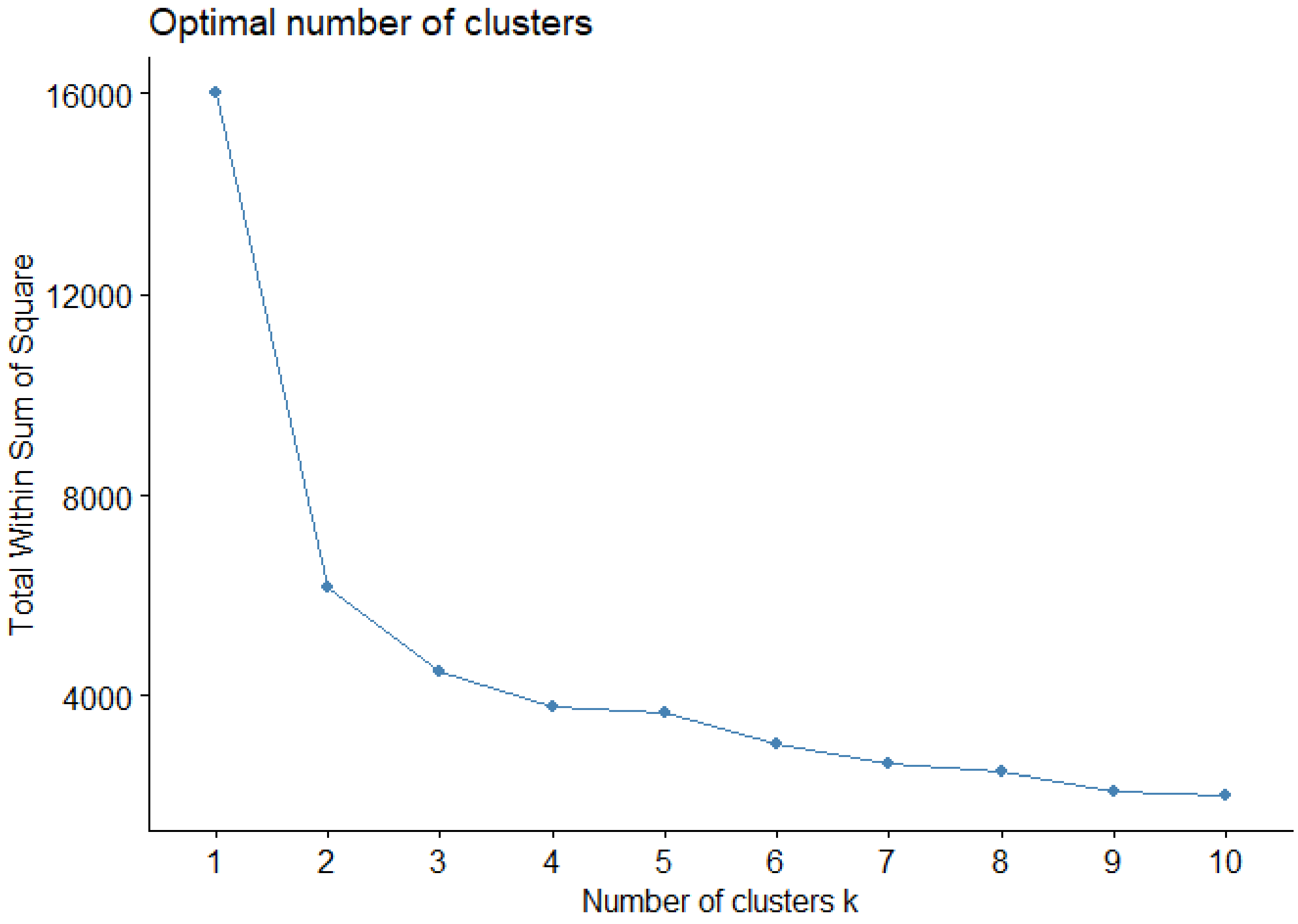

```
##                          Cluster
## Real group                1  2  3
##    Basal Magnoliophyta    0  0  1
##    Eudicots              27  0 66
##    Ferns                  1  0  0
##    Green Algae            0 11  0
##    Monocots               9  0  8
##    Mosses                 1  0  0
##    Red Algae              0  3  0
```

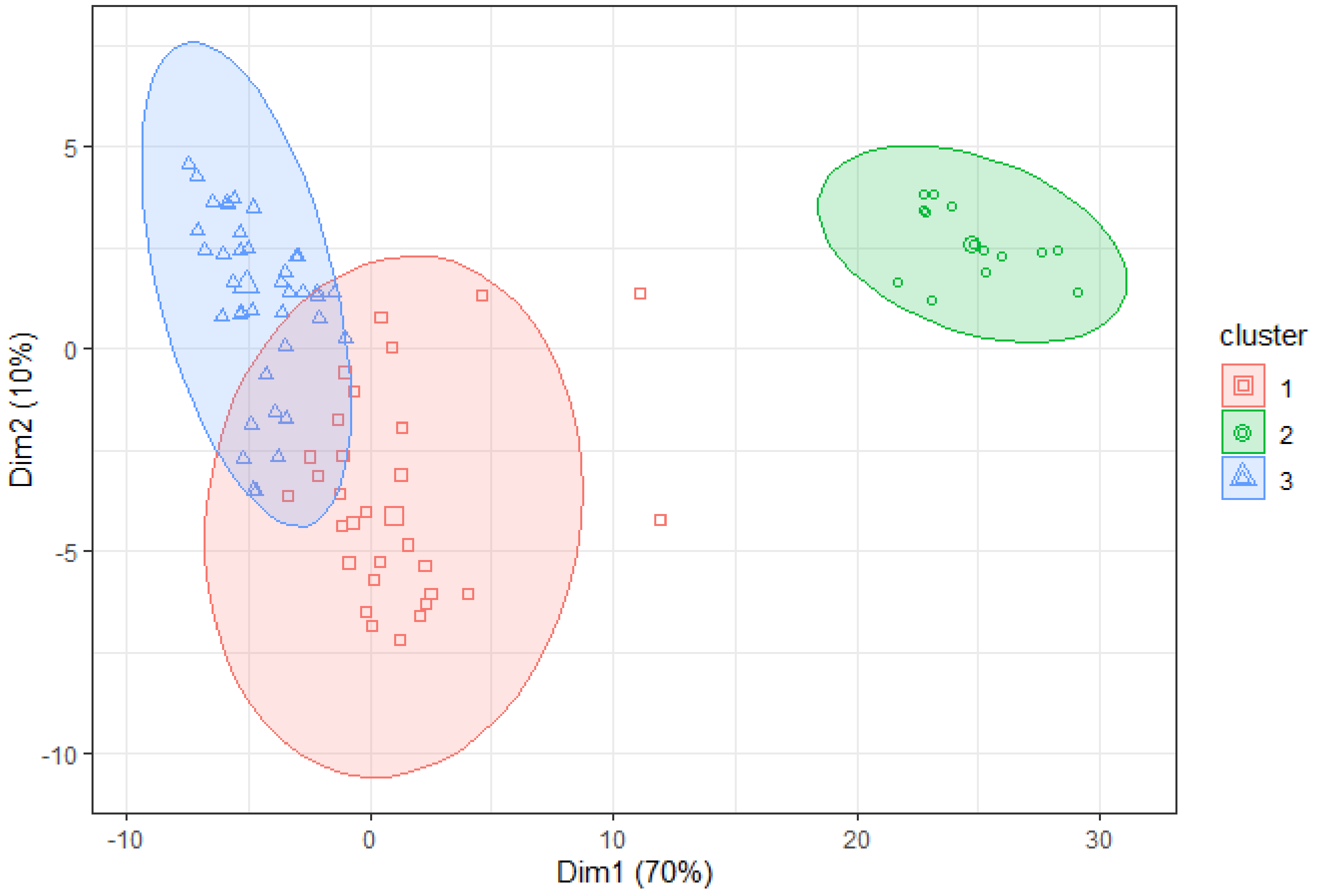

Organisms classified within cluster 1

```
##  [1] "ath"  "aly"  "crb"  "csat" "eus"  "brp"  "bna"  "boe"  "rsz"  "thj"
## [11] "cpap" "cit"  "cic"  "fve"  "mdm"  "pxb"  "zju"  "csv"  "cmo"  "bhj"
## [21] "qsu"  "qlo"  "vri"  "itr"  "egt"  "nnu"  "ming" "dosa" "bdi"  "ats"
## [31] "tdc"  "sbi"  "zma"  "sita" "phai" "pda"  "smo"  "ppp"
```

Organisms classified within cluster 2

```
##  [1] "cre"  "vcn"  "mng"  "csl"  "cvr"  "apro" "olu"  "ota"  "bpg"  "mis"
## [11] "mpp"  "cme"  "gsl"  "ccp"
```

Organisms classified within cluster 3

```
##  [1] "pvy"  "minc" "tcc"  "gra"  "ghi"  "gab"  "dzi"  "egr"  "gmx"  "gsj"
## [11] "pvu"  "vra"  "var"  "vun"  "ccaj" "aprc" "mtr"  "cam"  "lja"  "adu"
## [21] "aip"  "ahf"  "lang" "rcn"  "pper" "pmum" "pavi" "pdul" "mnt"  "mcha"
## [31] "cmax" "cmos" "cpep" "rcu"  "jcu"  "hbr"  "mesc" "pop"  "peu"  "palz"
## [41] "jre"  "twl"  "vvi"  "sly"  "spen" "sot"  "cann" "nta"  "nsy"  "nto"
## [51] "nau"  "ini"  "sind" "oeu"  "sspl" "han"  "ecad" "lsv"  "ccav" "dcr"
## [61] "csin" "bvg"  "soe"  "cqi"  "psom" "ncol" "osa"  "obr"  "pvir" "egu"
## [71] "mus"  "dct"  "peq"  "aof"  "atr"
```
