# Supplementary material for: Exploring the expressiveness of abstract metabolic networks
Source: PLoS One. 2023 Feb 9;18(2):e0281047. doi: 10.1371/journal.pone.0281047 (PMC9910719; doi:10.1371/journal.pone.0281047)
Supplement: S9 File — Fungi analyses at phylum level (second experiment). (PDF) [file pone.0281047.s009.pdf]

# Fungi Analysis


- Vertex hystogram (VH) kernel
  - Heatmap
  - MDS for VH
  - 3-Means clustering for VH Kernel
- Shortest Path (SP) kernel
  - Heatmap
  - MDS for SP
  - 3-Means clustering for SP
- Weisfeiler-Lehman (WL) kernel
  - Heatmap
  - MDS for WL
- 3-Means clustering for WL Kernel
- Pyramid match (PM) kernel
  - Heatmap
  - MDS for PM
  - 3-Means clustering for PM kernel


## Vertex hystogram (VH) kernel

## Heatmap

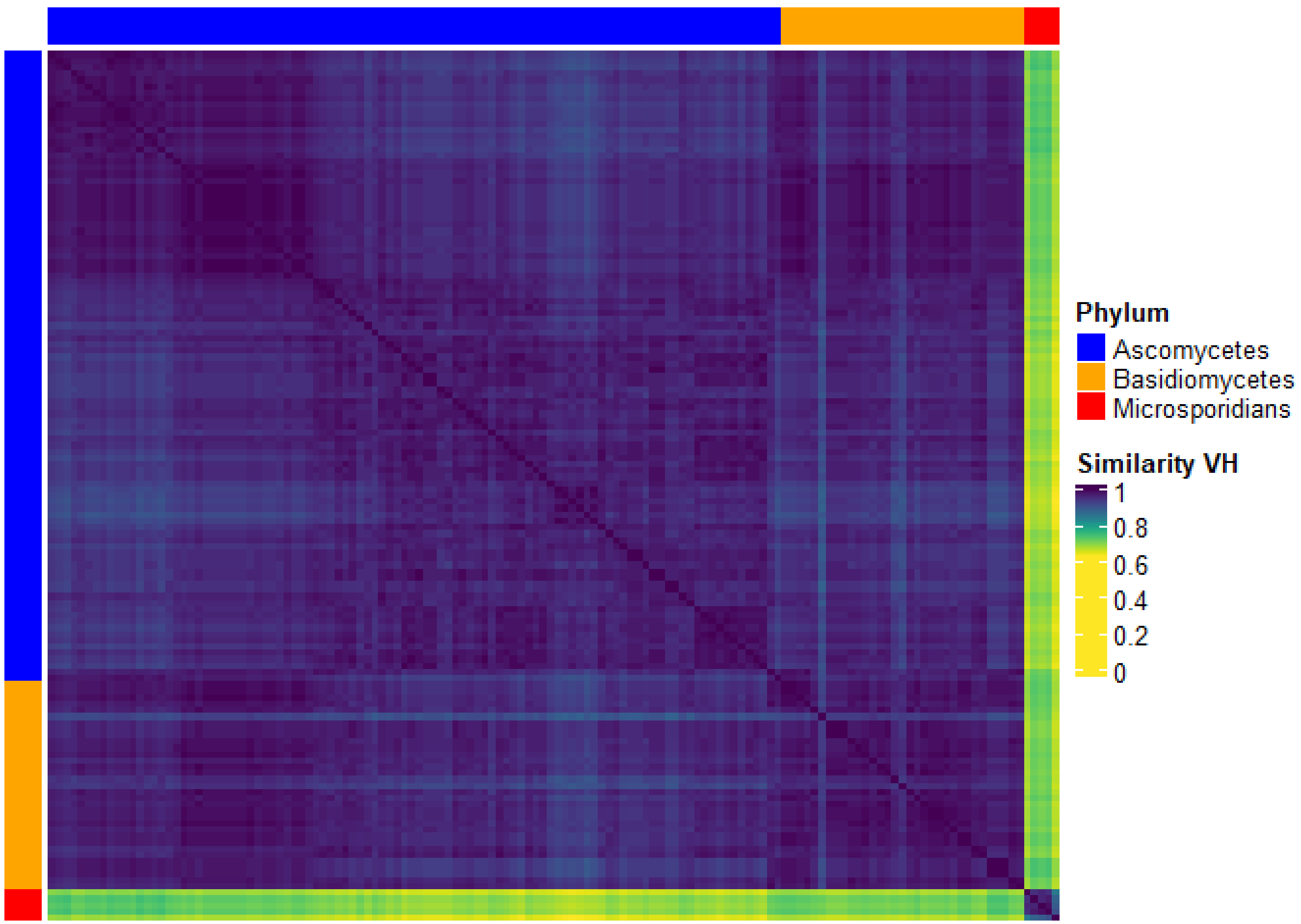


## MDS for VH

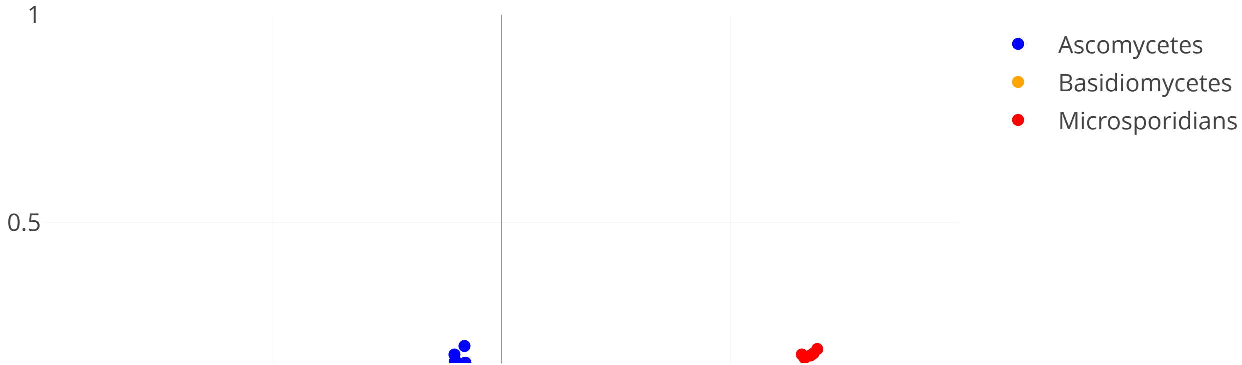

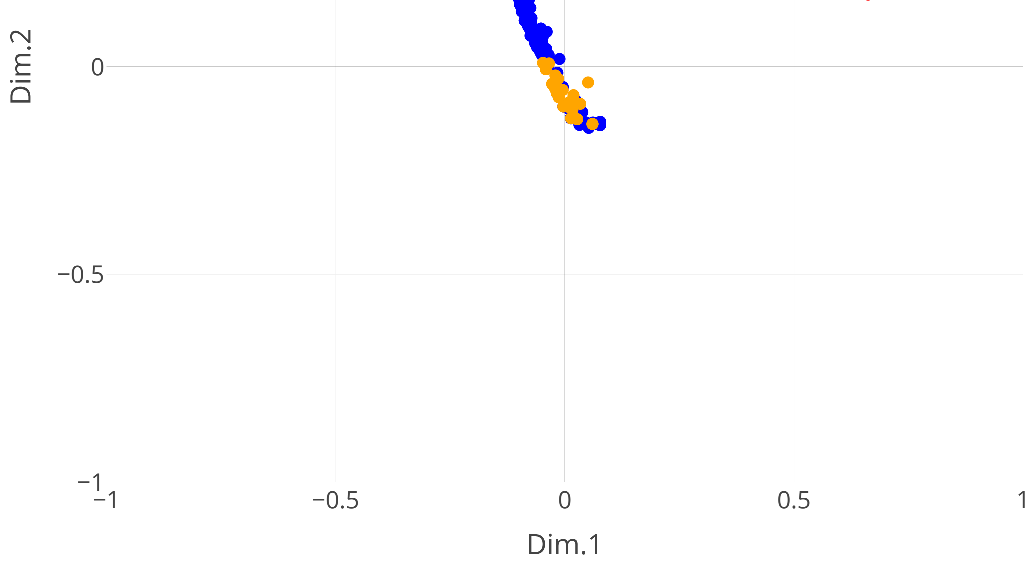


## 3-Means clustering for VH Kernel

```
##                    Cluster
## Real group           1  2  3
##   Ascomycetes       38 62  0
##   Basidiomycetes    30  3  0
##   Microsporidians    0  0  5
```

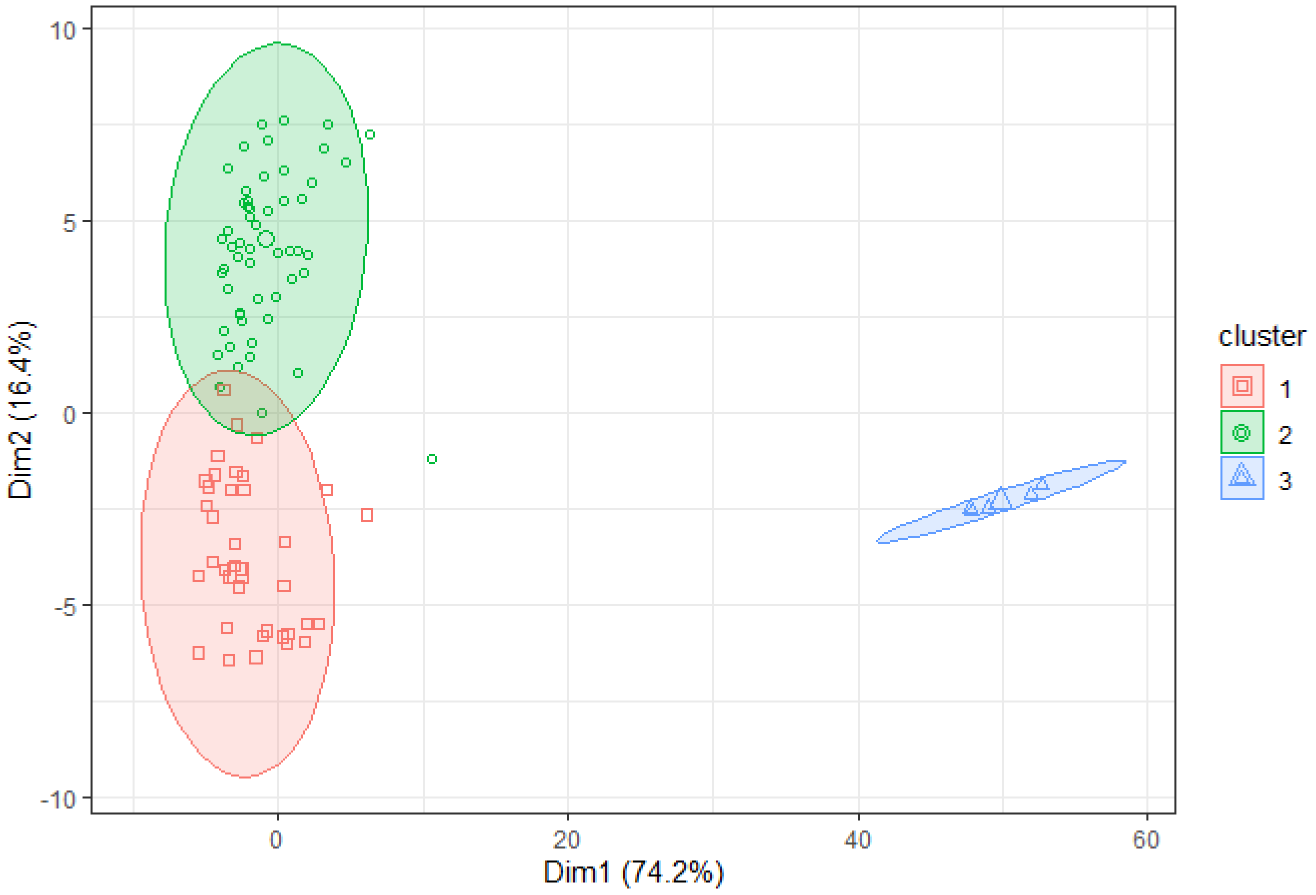


## Organisms classified within cluster 1

```
##  [1] "sce"  "ago"  "erc"  "kla"  "kmx"  "lth"  "vpo"  "zro"  "cgr"  "ncs"
## [11] "ndi"  "tpf"  "tbl"  "tdl"  "tgb"  "kaf"  "zmk"  "ppa"  "dha"  "pic"
## [21] "pgu"  "spaa" "lel"  "cal"  "ctp"  "cot"  "cdu"  "cten" "yli"  "clu"
## [31] "clus" "caur" "slb"  "pkz"  "bnn"  "bbrx" "tml"  "spo"  "cne"  "cnb"
## [41] "cgi"  "tms"  "tasa" "tvs"  "dsq"  "pco"  "shs"  "hir"  "psq"  "adl"
## [51] "fme"  "gtr"  "lbc"  "mpr"  "mrr"  "cci"  "scm"  "abp"  "abv"  "cput"
## [61] "sla"  "wse"  "wic"  "mgl"  "mrt"  "msym" "pgr"  "mlr"
```

### Organisms classified within cluster 2

```
##  [1] "ncr"  "nte"  "smp"  "pan"  "ttt"  "mtm"  "cthr" "mgr"  "tmn"  "ssck"
## [11] "fgr"  "fpu"  "fvr"  "fox"  "nhe"  "tre"  "trr"  "maw"  "maj"  "cmt"
## [21] "plj"  "val"  "vda"  "cfj"  "sapo" "ela"  "pfy"  "ssl"  "bfu"  "mbe"
## [31] "psco" "glz"  "ani"  "afm"  "act"  "nfi"  "aor"  "ang"  "afv"  "pcs"
## [41] "pdp"  "tmf"  "trg"  "cim"  "cpw"  "ure"  "pbl"  "pbn"  "abe"  "tve"
## [51] "aje"  "bgh"  "pno"  "pte"  "bze"  "bsc"  "bor"  "aalt" "ztr"  "pfj"
## [61] "bcom" "npa"  "ppl"  "uma"  "pfp"
```

### Organisms classified within cluster 3

```
## [1] "ecu" "ein" "ehe" "ero" "nce"
```

# Shortest Path (SP) kernel

## Heatmap

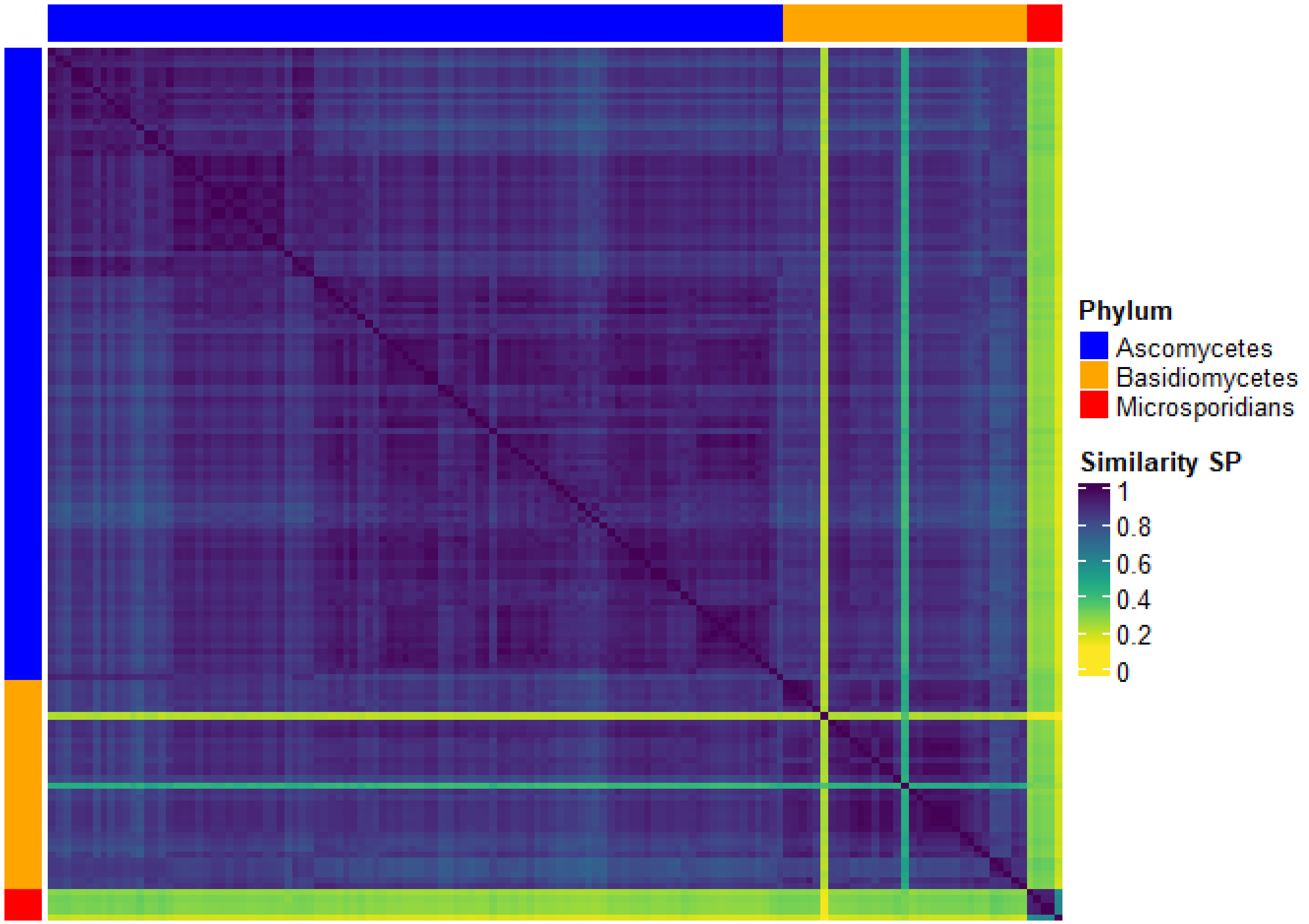
Phylum
Ascomycetes
Basidiomycetes
Microsporidians
Similarity SP
1
0.8
0.6
0.4
0.2
0

## MDS for SP

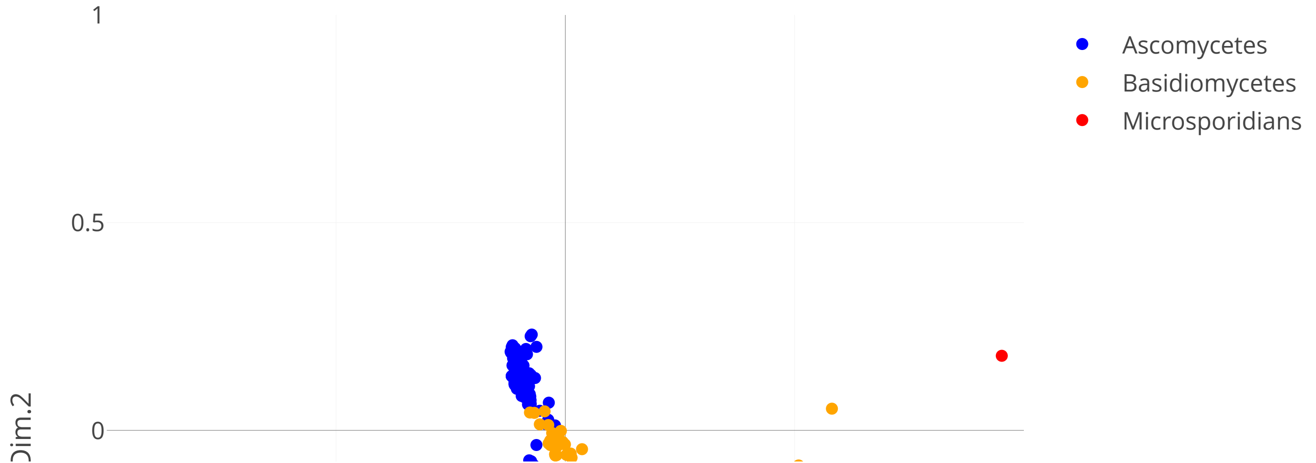
1
0.5
0
Ascomycetes
Basidiomycetes
Microsporidians

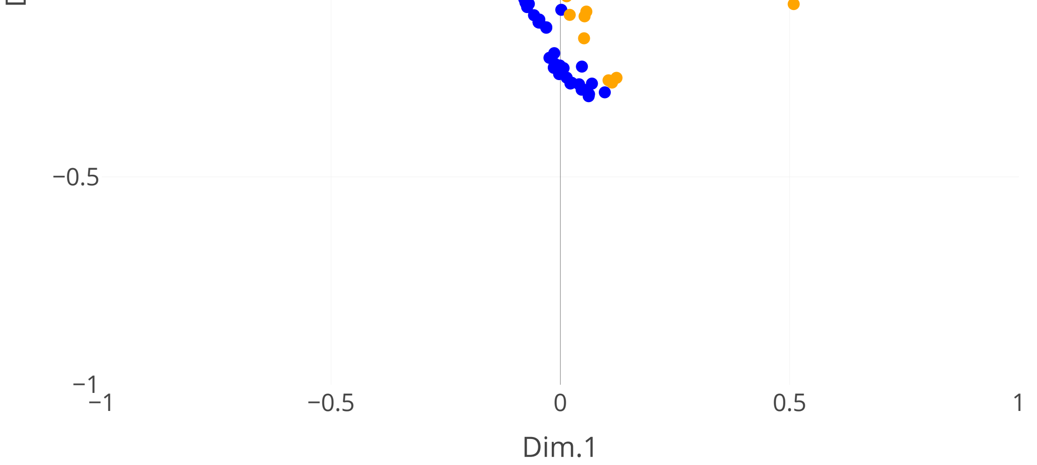


## 3-Means clustering for SP

```
##                   Cluster
## Real group          1  2  3
##   Ascomycetes      30  0 70
##   Basidiomycetes   27  2  4
##   Microsporidians   0  5  0
```

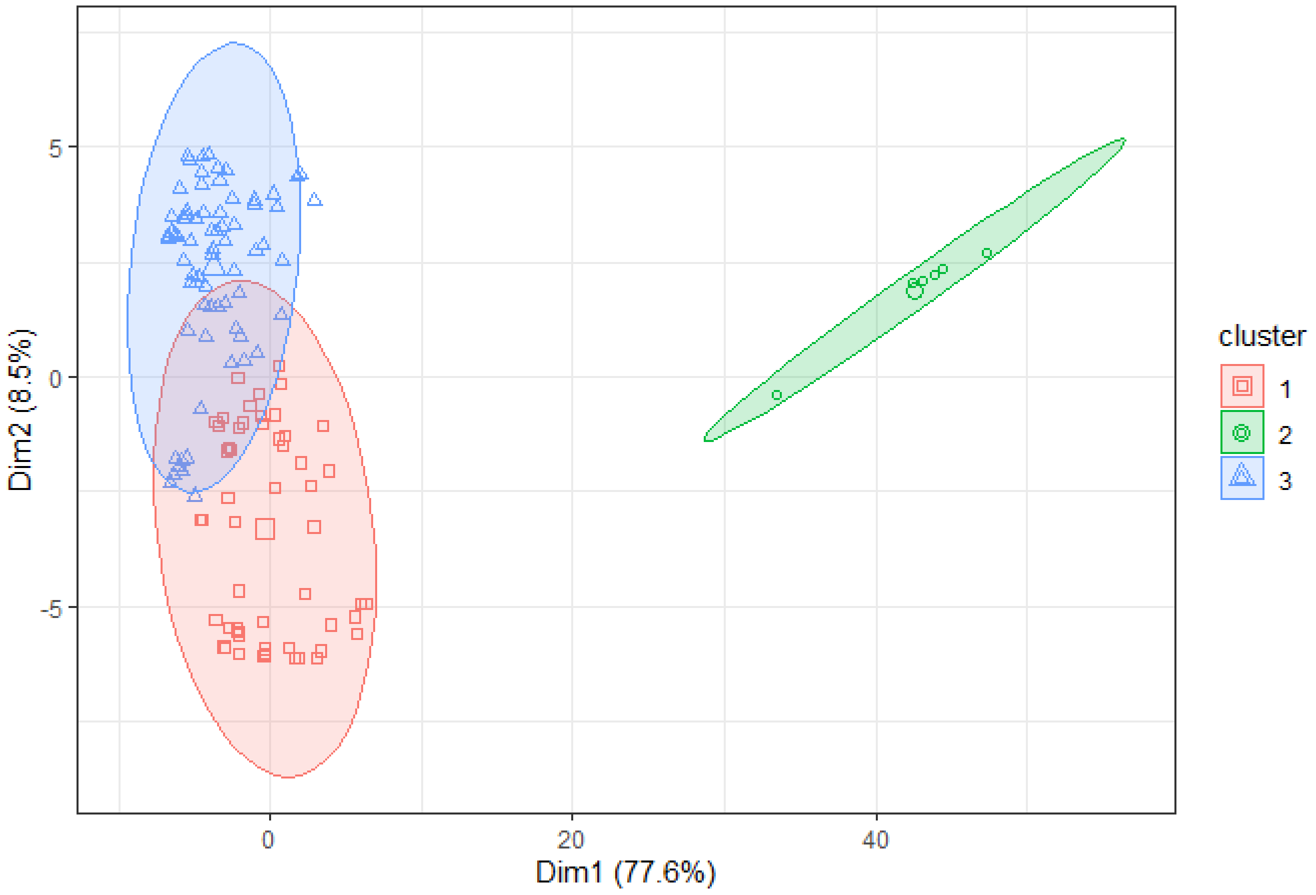


### Organisms classified within cluster 1

```
##  [1] "sce"   "ago"   "erc"   "kla"   "kmx"   "lth"   "vpo"   "zro"   "cgr"   "ncs"
## [11] "ndi"   "tpf"   "tbl"   "tdl"   "tgb"   "kaf"   "zmk"   "pgu"   "lel"   "cal"
## [21] "cot"   "cdu"   "clu"   "clus" "slb"   "pkz"   "bnn"   "bbrx" "sapo" "spo"
## [31] "cne"   "cnb"   "cgi"   "tms"   "tasa" "shs"   "hir"   "psq"   "fme"   "gtr"
## [41] "lbc"   "mrr"   "cci"   "scm"   "abp"   "abv"   "cput" "sla"   "wse"   "wic"
## [51] "uma"   "pfp"   "mgl"   "mrt"   "msym" "pgr"   "mlr"
```

### Organisms classified within cluster 2

```
## [1] "ppl" "mpr" "ecu" "ein" "ehe" "ero" "nce"
```

### Organisms classified within cluster 3

```
##  [1] "ppa"  "dha"  "pic"  "spaa" "ctp"  "cten" "yli"  "caur" "ncr"  "nte"
## [11] "smp"  "pan"  "ttt"  "mtm"  "cthr" "mgr"  "tmn"  "ssck" "fgr"  "fpu"
## [21] "fvr"  "fox"  "nhe"  "tre"  "trr"  "maw"  "maj"  "cmt"  "plj"  "val"
## [31] "vda"  "cfj"  "ela"  "pfy"  "ssl"  "bfu"  "mbe"  "psco" "glz"  "ani"
## [41] "afm"  "act"  "nfi"  "aor"  "ang"  "afv"  "pcs"  "pdp"  "tmf"  "trg"
## [51] "cim"  "cpw"  "ure"  "pbl"  "pbn"  "abe"  "tve"  "aje"  "bgh"  "pno"
## [61] "pte"  "bze"  "bsc"  "bor"  "aalt" "ztr"  "pfj"  "bcom" "npa"  "tml"
## [71] "tvs"  "dsq"  "pco"  "adl"
```

# Weisfeiler-Lehman (WL) kernel

## Heatmap

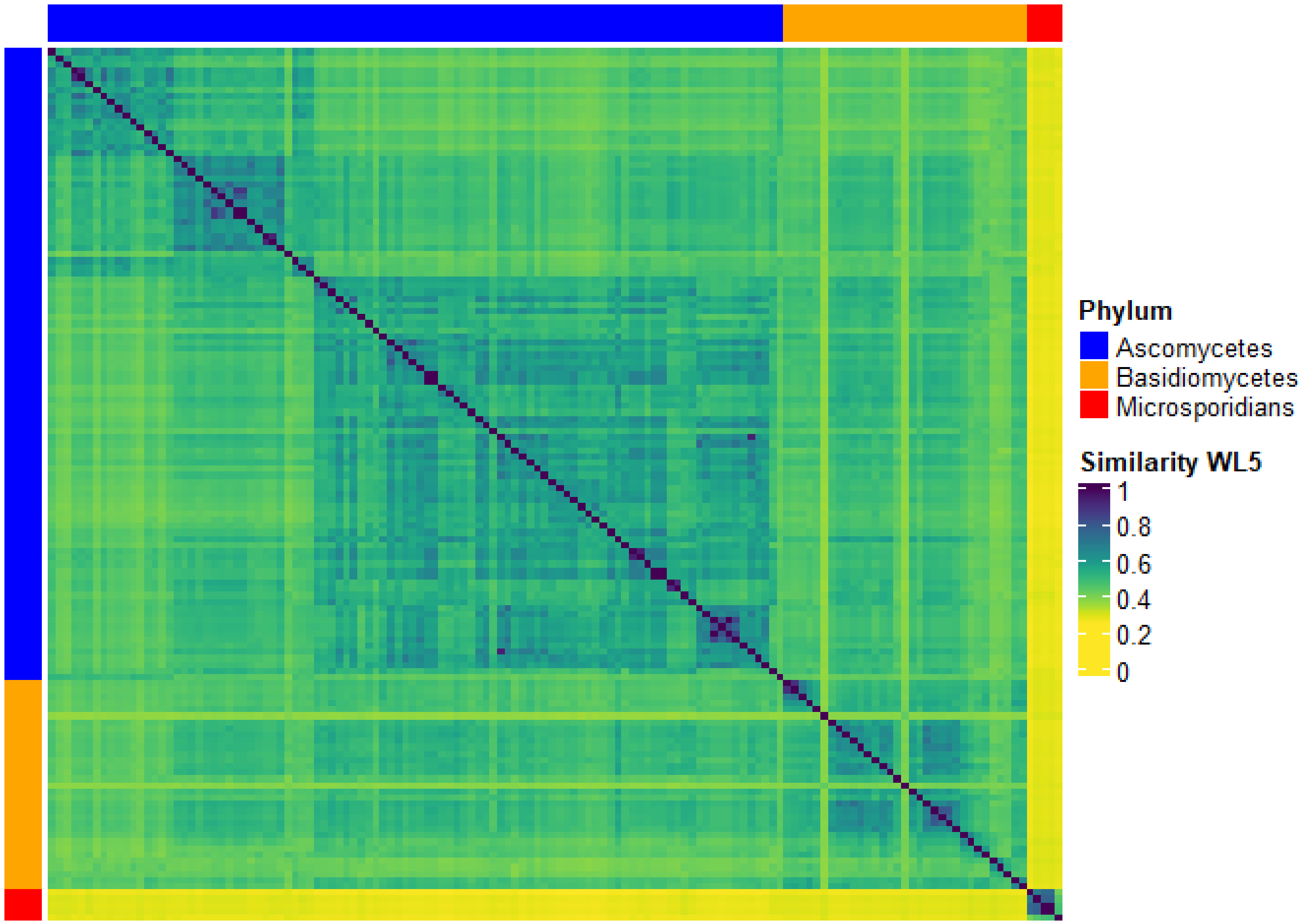
Phylum
Ascomycetes
Basidiomycetes
Microsporidians
Similarity WL5
1
0.8
0.6
0.4
0.2
0

MDS for WL

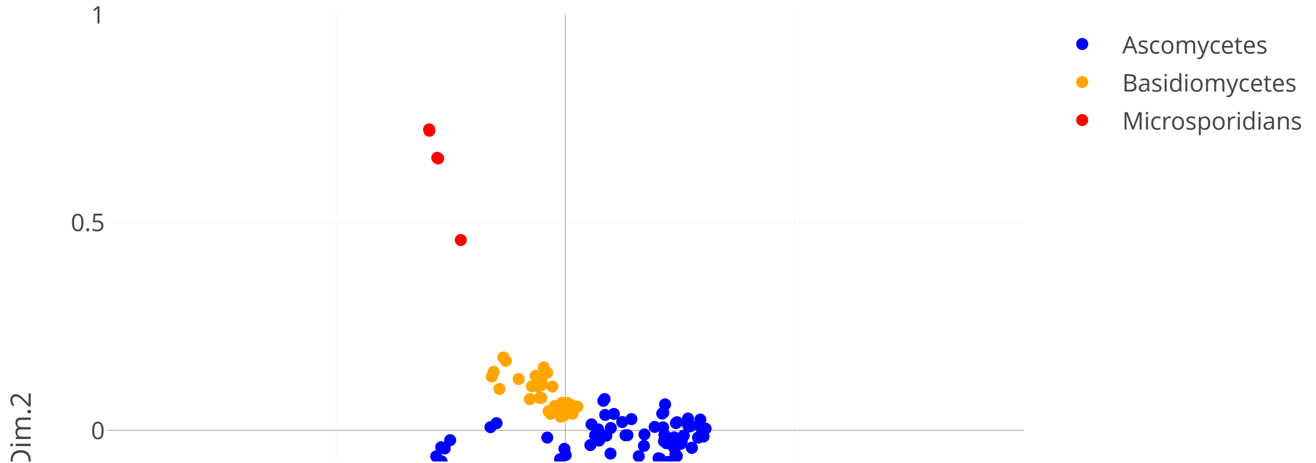
1
0.5
0
Dim.2
Ascomycetes
Basidiomycetes
Microsporidians

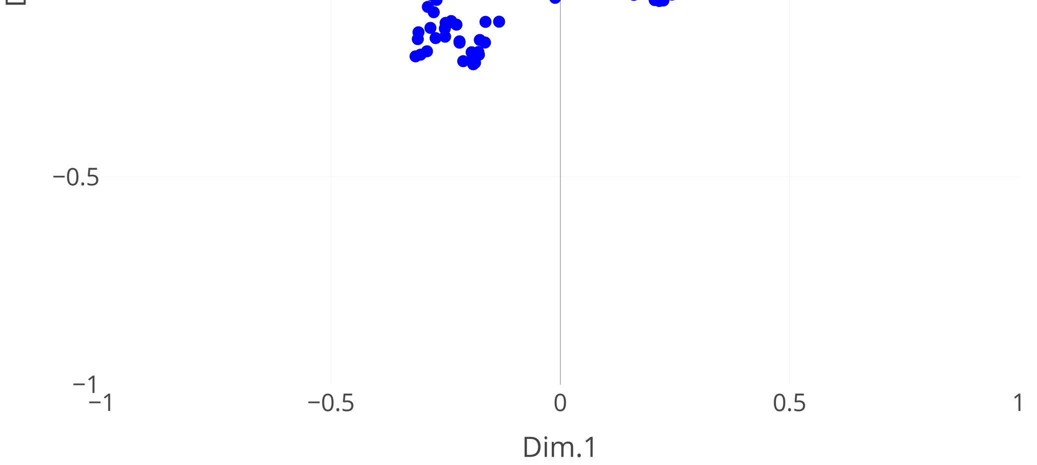


## 3-Means clustering for WL Kernel

```
##                    Cluster
## Real group          1  2  3
##   Ascomycetes      42  0 58
##   Basidiomycetes   33  0  0
##   Microsporidians   0  5  0
```

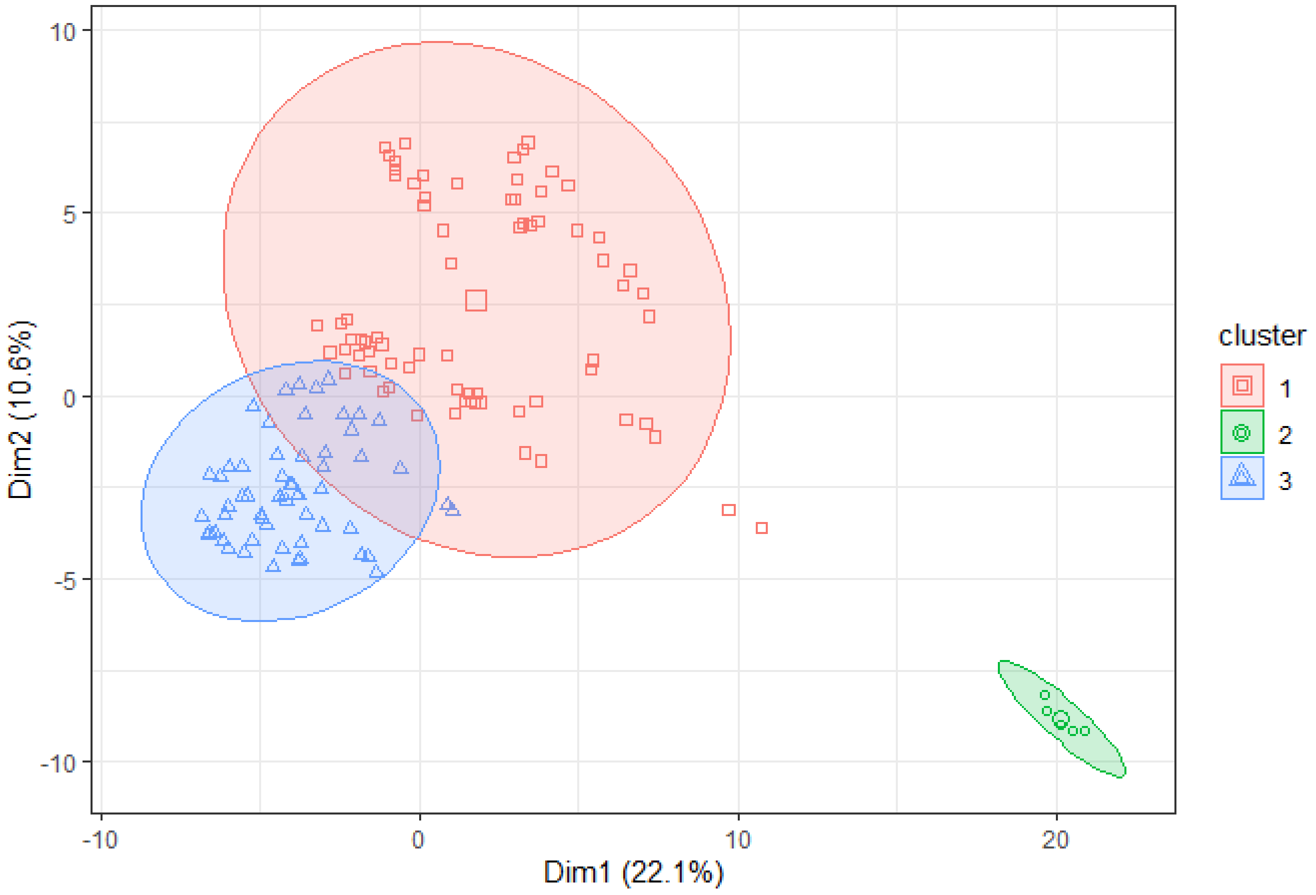


### Organisms classified within cluster 1

```
##  [1] "sce"  "ago"  "erc"  "kla"  "kmx"  "lth"  "vpo"  "zro"  "cgr"  "ncs"
## [11] "ndi"  "tpf"  "tbl"  "tdl"  "tgb"  "kaf"  "zmk"  "ppa"  "dha"  "pic"
## [21] "pgu"  "spaa" "lel"  "cal"  "ctp"  "cot"  "cdu"  "cten" "yli"  "clu"
## [31] "clus" "caur" "slb"  "pkz"  "bnn"  "bbrx" "ttt"  "cthr" "abe"  "tve"
## [41] "tml"  "spo"  "cne"  "cnb"  "cgi"  "tms"  "tasa" "ppl"  "tvs"  "dsq"
## [51] "pco"  "shs"  "hir"  "psq"  "adl"  "fme"  "gtr"  "lbc"  "mpr"  "mrr"
## [61] "cci"  "scm"  "abp"  "abv"  "cput" "sla"  "wse"  "wic"  "uma"  "pfp"
## [71] "mgl"  "mrt"  "msym" "pgr"  "mlr"
```

### Organisms classified within cluster 2

```
## [1] "ecu" "ein" "ehe" "ero" "nce"
```

### Organisms classified within cluster 3

```
##  [1] "ncr"  "nte"  "smp"  "pan"  "mtm"  "mgr"  "tmn"  "ssck" "fgr"  "fpu"
## [11] "fvr"  "fox"  "nhe"  "tre"  "trr"  "maw"  "maj"  "cmt"  "plj"  "val"
## [21] "vda"  "cfj"  "sapo" "ela"  "pfy"  "ssl"  "bfu"  "mbe"  "psco" "glz"
## [31] "ani"  "afm"  "act"  "nfi"  "aor"  "ang"  "afv"  "pcs"  "pdp"  "tmf"
## [41] "trg"  "cim"  "cpw"  "ure"  "pbl"  "pbn"  "aje"  "bgh"  "pno"  "pte"
## [51] "bze"  "bsc"  "bor"  "aalt" "ztr"  "pfj"  "bcom" "npa"
```

## Pyramid match (PM) kernel

### Heatmap

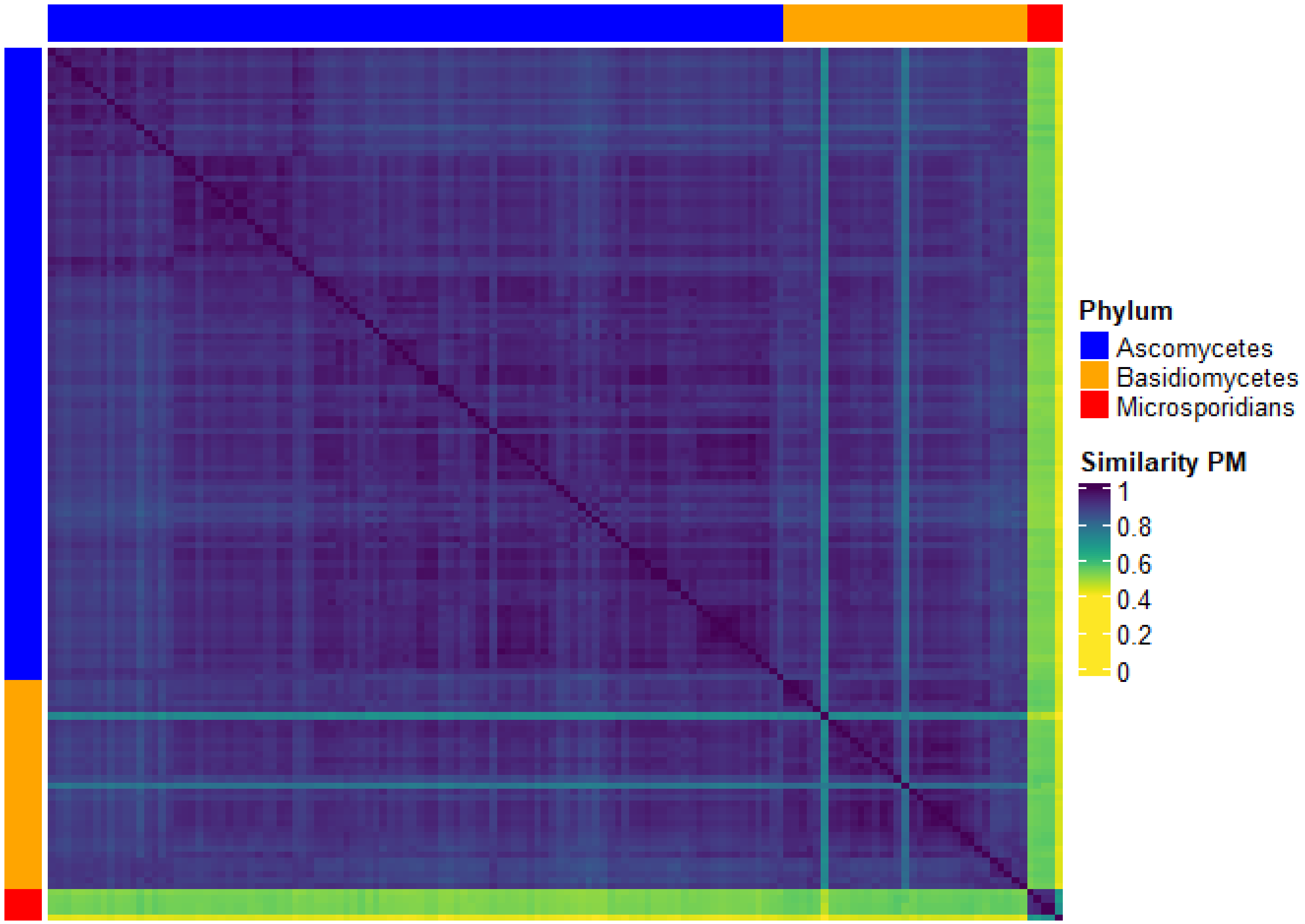
Phylum
Ascomycetes
Basidiomycetes
Microsporidians
Similarity PM
1
0.8
0.6
0.4
0.2
0

MDS for PM

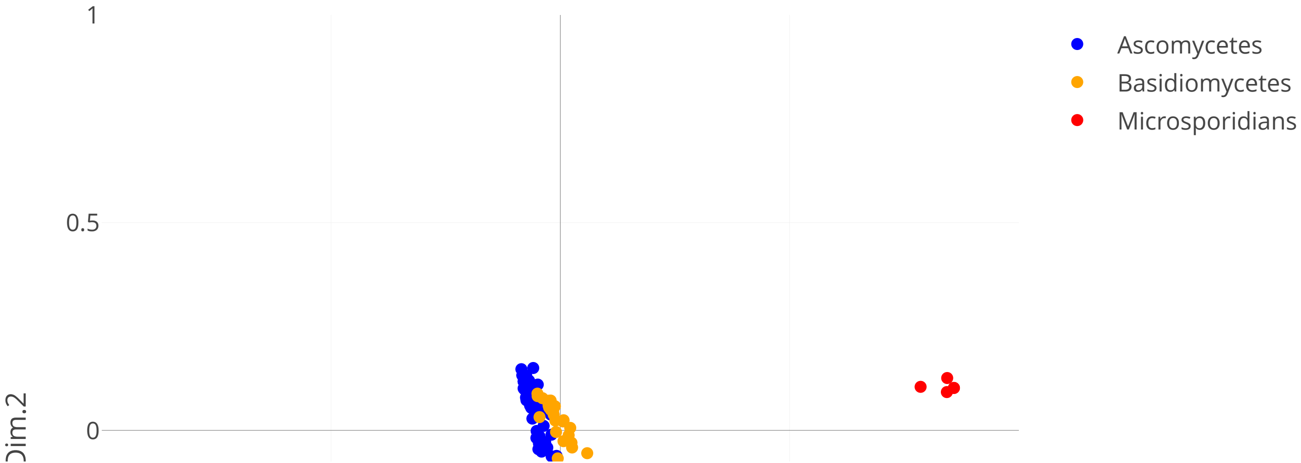
1
0.5
0
Dim.2
Ascomycetes
Basidiomycetes
Microsporidians

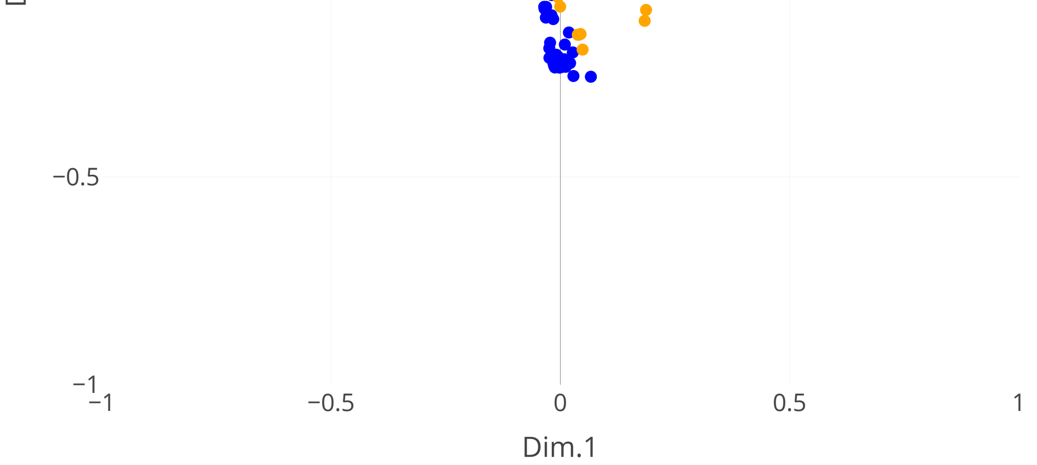


## 3-Means clustering for PM kernel

```
##                     Cluster
## Real group            1  2  3
##   Ascomycetes        28 72  0
##   Basidiomycetes     12 20  1
##   Microsporidians     0  0  5
```

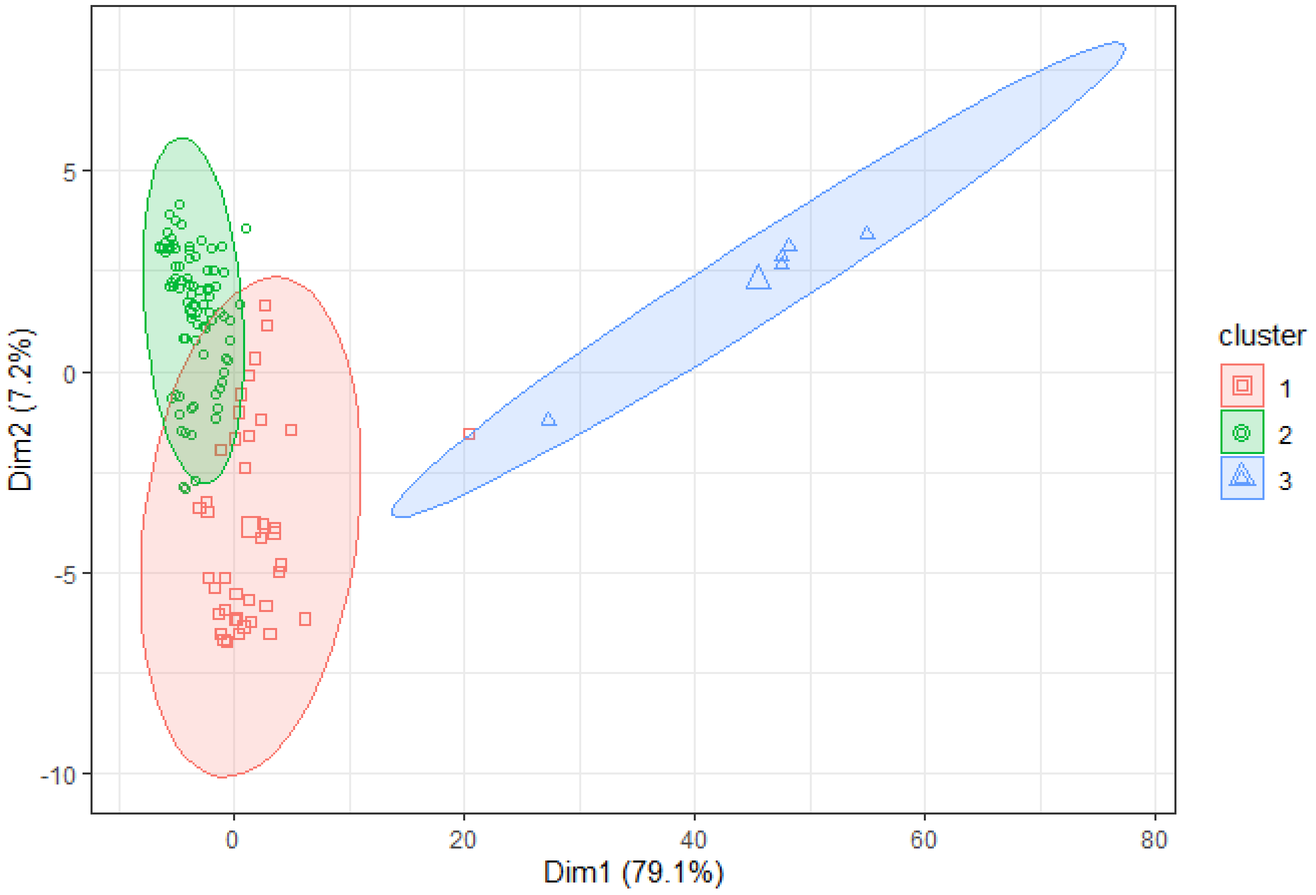


## Organisms classified within cluster 1

```
##  [1] "sce"  "ago"  "erc"  "kla"  "kmx"  "lth"  "vpo"  "zro"  "cgr"  "ncs"
## [11] "ndi"  "tpf"  "tbl"  "tdl"  "tgb"  "kaf"  "zmk"  "pgu"  "clu"  "clus"
## [21] "pkz"  "bnn"  "bbrx" "sapo" "afm"  "aor"  "afv"  "spo"  "tms"  "tasa"
## [31] "lbc"  "mpr"  "cci"  "wic"  "uma"  "mgl"  "mrt"  "msym" "pgr"  "mlr"
```

## Organisms classified within cluster 2

```
##  [1] "ppa"  "dha"  "pic"  "spaa" "lel"  "cal"  "ctp"  "cot"  "cdu"  "cten"
## [11] "yli"  "caur" "slb"  "ncr"  "nte"  "smp"  "pan"  "ttt"  "mtm"  "cthr"
## [21] "mgr"  "tmn"  "ssck" "fgr"  "fpu"  "fvr"  "fox"  "nhe"  "tre"  "trr"
## [31] "maw"  "maj"  "cmt"  "plj"  "val"  "vda"  "cfj"  "ela"  "pfy"  "ssl"
## [41] "bfu"  "mbe"  "psco" "glz"  "ani"  "act"  "nfi"  "ang"  "pcs"  "pdp"
## [51] "tmf"  "trg"  "cim"  "cpw"  "ure"  "pbl"  "pbn"  "abe"  "tve"  "aje"
## [61] "bgh"  "pno"  "pte"  "bze"  "bsc"  "bor"  "aalt" "ztr"  "pfj"  "bcom"
## [71] "npa"  "tml"  "cne"  "cnb"  "cgi"  "tvs"  "dsq"  "pco"  "shs"  "hir"
## [81] "psq"  "adl"  "fme"  "gtr"  "mrr"  "scm"  "abp"  "abv"  "cput" "sla"
## [91] "wse"  "pfp"
```

### Organisms classified within cluster 3

```
## [1] "ppl" "ecu" "ein" "ehe" "ero" "nce"
```
