# Supplementary material for: Exploring the expressiveness of abstract metabolic networks
Source: PLoS One. 2023 Feb 9;18(2):e0281047. doi: 10.1371/journal.pone.0281047 (PMC9910719; doi:10.1371/journal.pone.0281047)
Supplement: S10 File — Protists analyses at phylum level (second experiment). (PDF) [file pone.0281047.s010.pdf]

Protists Analysis

- Vertex hystogram (VH) kernel
  - Heatmap
  - MDS for VH
  - 9-Means clustering for VH Kernel
  - Optimal number of clusters for VH
- Shortest Path (SP) kernel
  - Heatmap
  - MDS for SP
  - 9-Means clustering for SP Kernel
  - Optimal number of clusters for SP
- Weisfeiler-Lehman (WL) kernel
  - Heatmap
  - MDS for WL
  - 9-Means clustering for WL Kernel
  - Optimal number of clusters for WL
- Pyramid match (PM) kernel
  - Heatmap
  - MDS for PM
  - 9-Means clustering for PM Kernel
  - Optimal number of clusters for PM

Vertex hystogram (VH) kernel

Heatmap

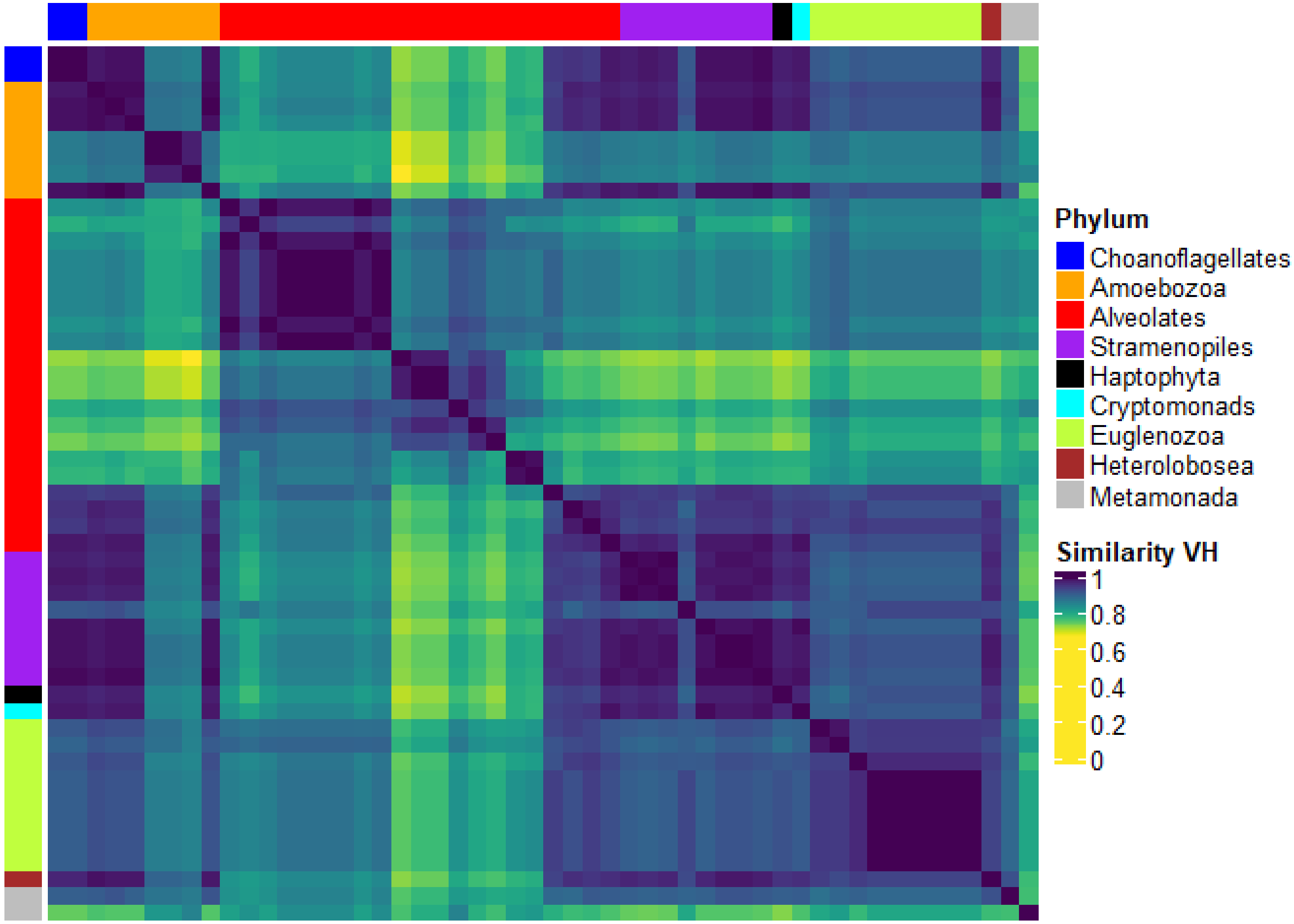

MDS for VH

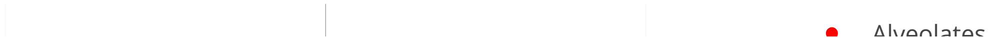

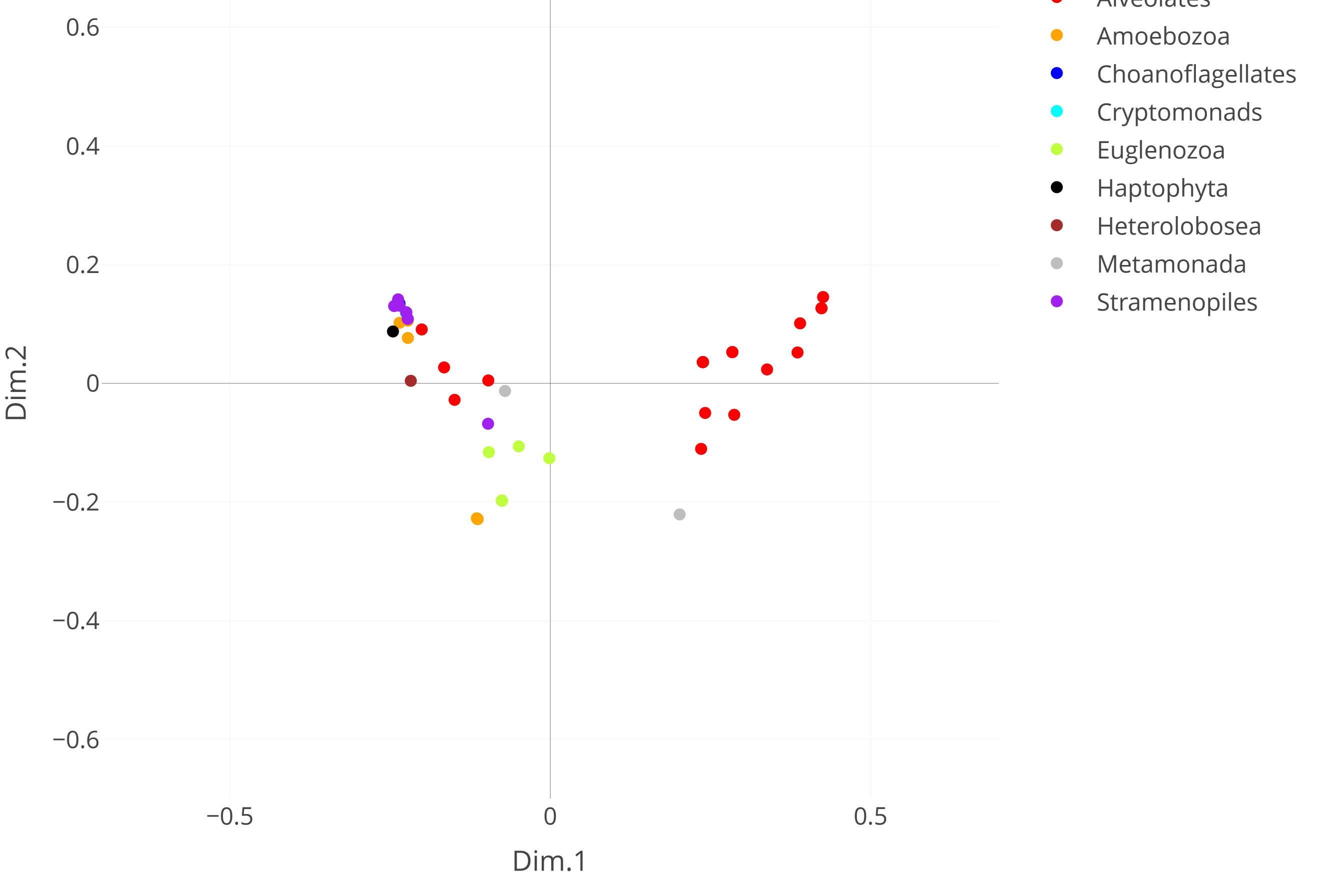

9-Means clustering for VH Kernel

```
##                     Cluster
## Real group          1 2 3 4 5 6 7 8 9
##   Alveolates        2 8 5 2 3 1 0 0 0
##   Amoebozoa         0 0 0 0 0 4 0 0 3
##   Choanoflagellates 0 0 0 0 0 2 0 0 0
##   Cryptomonads      0 0 0 0 0 1 0 0 0
##   Euglenozoa        0 0 0 0 3 0 6 0 0
##   Haptophyta        0 0 0 0 0 1 0 0 0
##   Heterolobosea     0 0 0 0 0 1 0 0 0
##   Metamonada        0 0 0 0 1 0 0 1 0
##   Stramenopiles     0 0 0 0 1 7 0 0 0
```

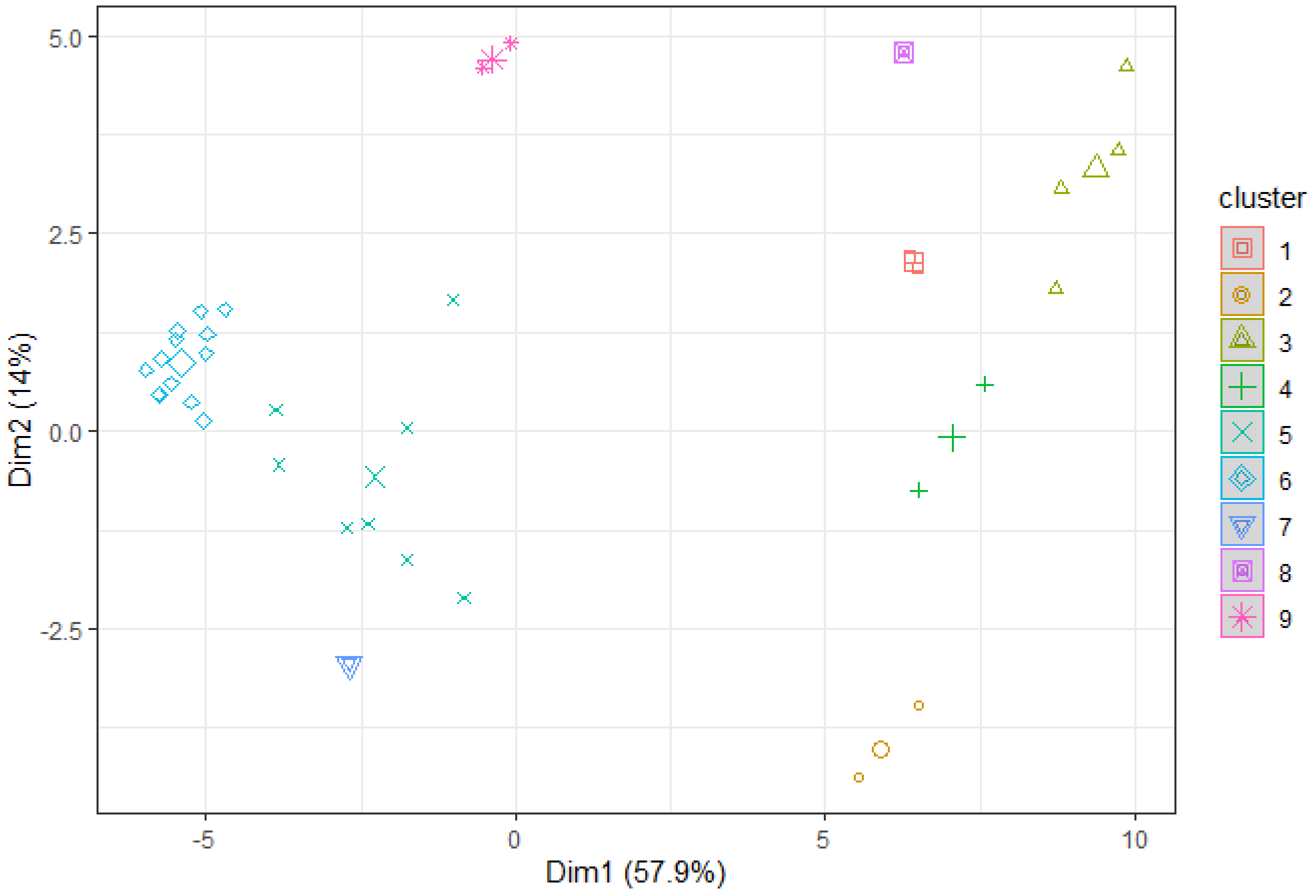

Organisms classified within cluster 1

```
## [1] "cpv" "cho"
```

Organisms classified within cluster 2

```
## [1] "pfa" "pfh" "pyo" "pcb" "pbe" "pkn" "pvx" "pcy"
```

Organisms classified within cluster 3

```
## [1] "tan"  "tpv"  "tot"  "bbo"  "bmic"
```

Organisms classified within cluster 4

```
## [1] "pfd" "beq"
```

Organisms classified within cluster 5

```
## [1] "tgo" "tet" "ptm" "ngd" "tbr" "tbg" "tcr" "tva"
```

Organisms classified within cluster 6

```
##  [1] "mbr"  "sre"  "ddi"  "dpp"  "dfa"  "acan" "smin" "pti"  "fcy"  "tps"
## [11] "aaf"  "pif"  "psoj" "spar" "ehx"  "gtt"  "ngr"
```

Organisms classified within cluster 7

```
## [1] "lma"  "lif"  "ldo"  "lmi"  "lbz"  "lpan"
```

Organisms classified within cluster 8

```
## [1] "gla"
```

Organisms classified within cluster 9

```
## [1] "ehi" "edi" "eiv"
```

Optimal number of clusters for VH

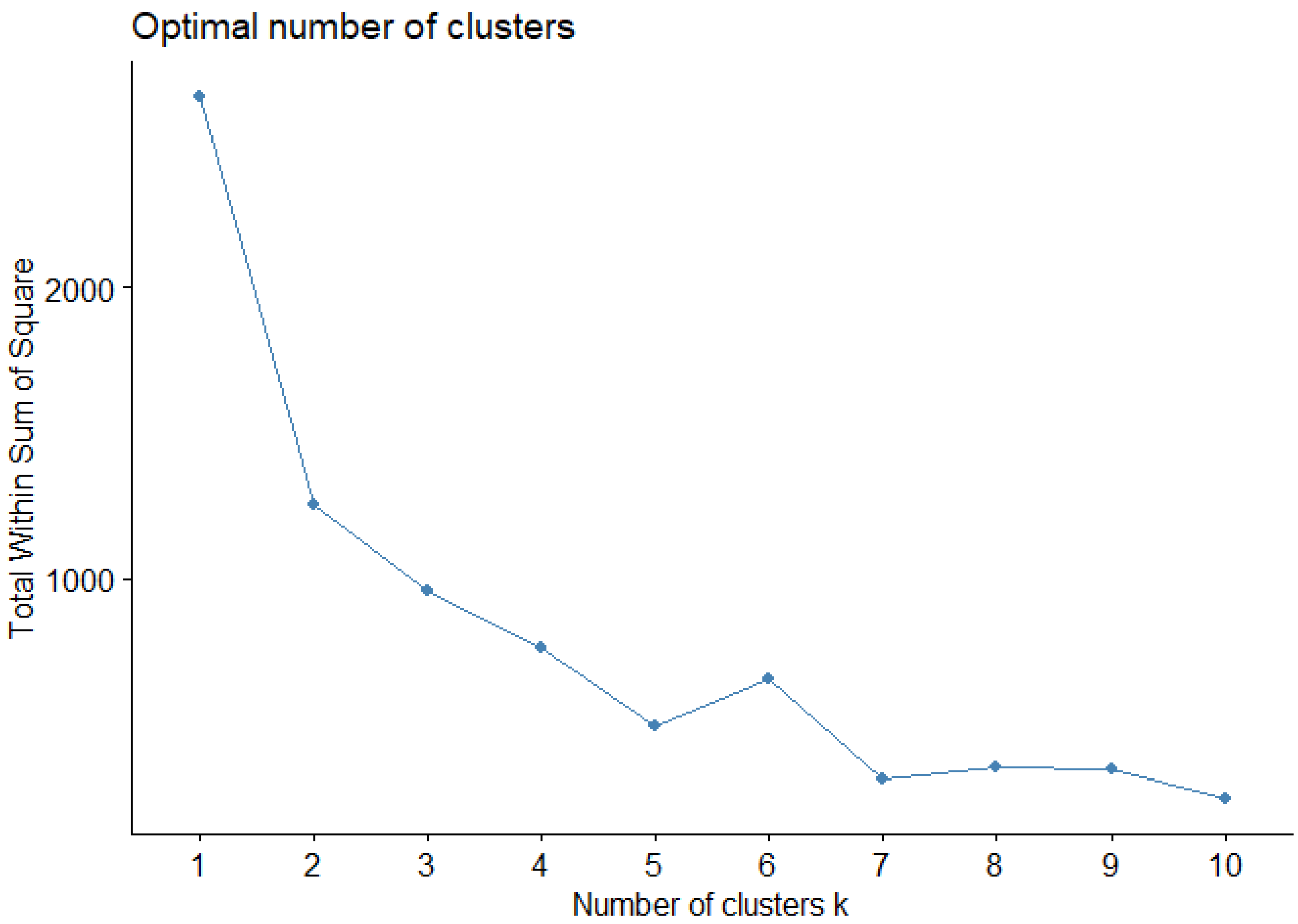


```
##                      Cluster
## Real group             1 2 3 4
##   Alveolates           1 9 8 3
##   Amoebozoa            3 0 0 4
##   Choanoflagellates 0 0 0 2
##   Cryptomonads         0 0 0 1
```

```
##    Euglenozoa        9 0 0 0
##    Haptophyta        0 0 0 1
##    Heterolobosea     0 0 0 1
##    Metamonada        1 0 1 0
##    Stramenopiles     1 0 0 7
```

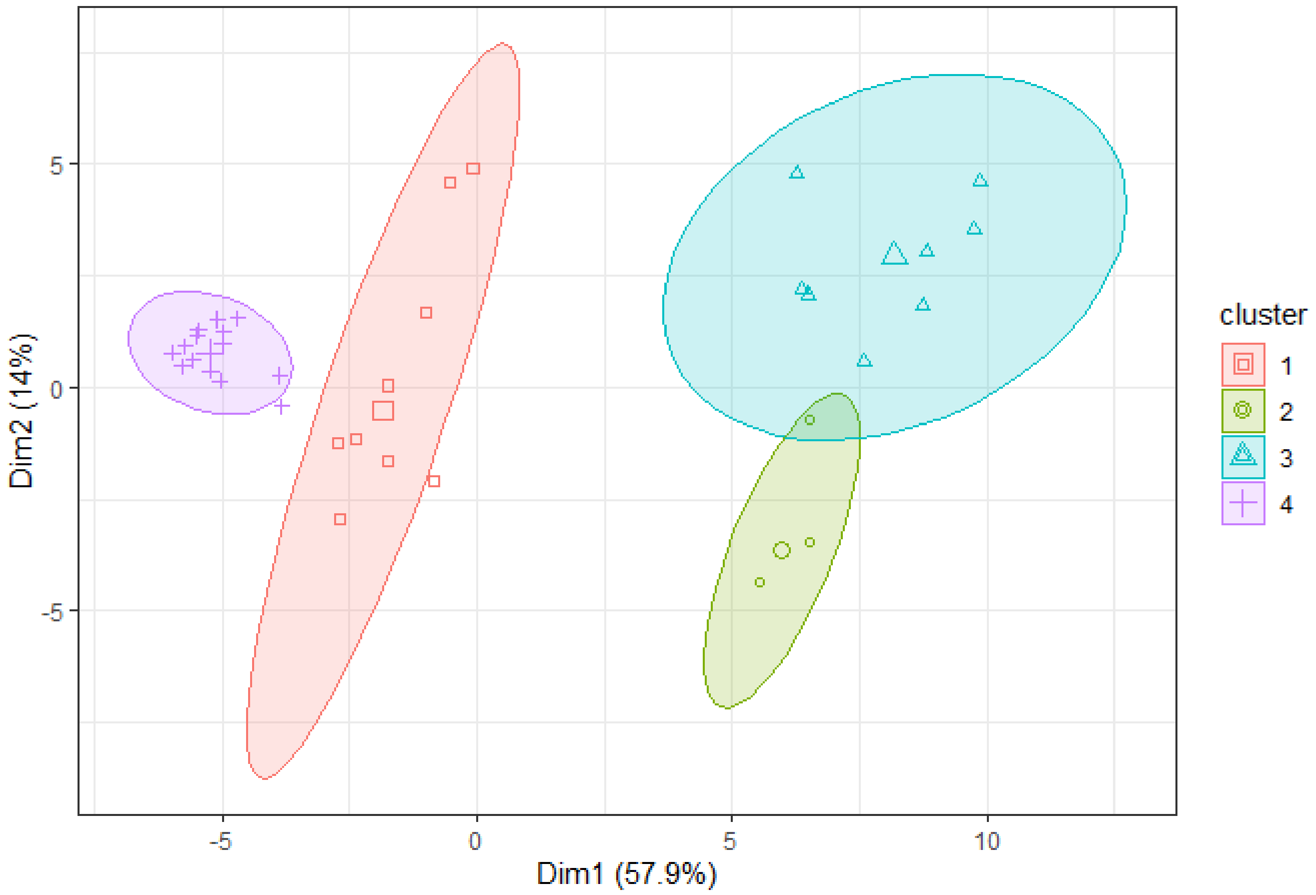

Organisms classified within cluster 1

```
##  [1] "ehi"  "edi"  "eiv"  "tgo"  "ngd"  "tbr"  "tbg"  "tcr"  "lma"  "lif"
## [11] "ldo"  "lmi"  "lbz"  "lpan" "tva"
```

Organisms classified within cluster 2

```
## [1] "pfa" "pfd" "pfh" "pyo" "pcb" "pbe" "pkn" "pvx" "pcy"
```

Organisms classified within cluster 3

```
## [1] "tan"  "tpv"  "tot"  "beq"  "bbo"  "bmic" "cpv"  "cho"  "gla"
```

Organisms classified within cluster 4

```
##  [1] "mbr"  "sre"  "ddi"  "dpp"  "dfa"  "acan" "tet"  "ptm"  "smin" "pti"
## [11] "fcy"  "tps"  "aaf"  "pif"  "psoj" "spar" "ehx"  "gtt"  "ngr"
```

Shortest Path (SP) kernel

Heatmap

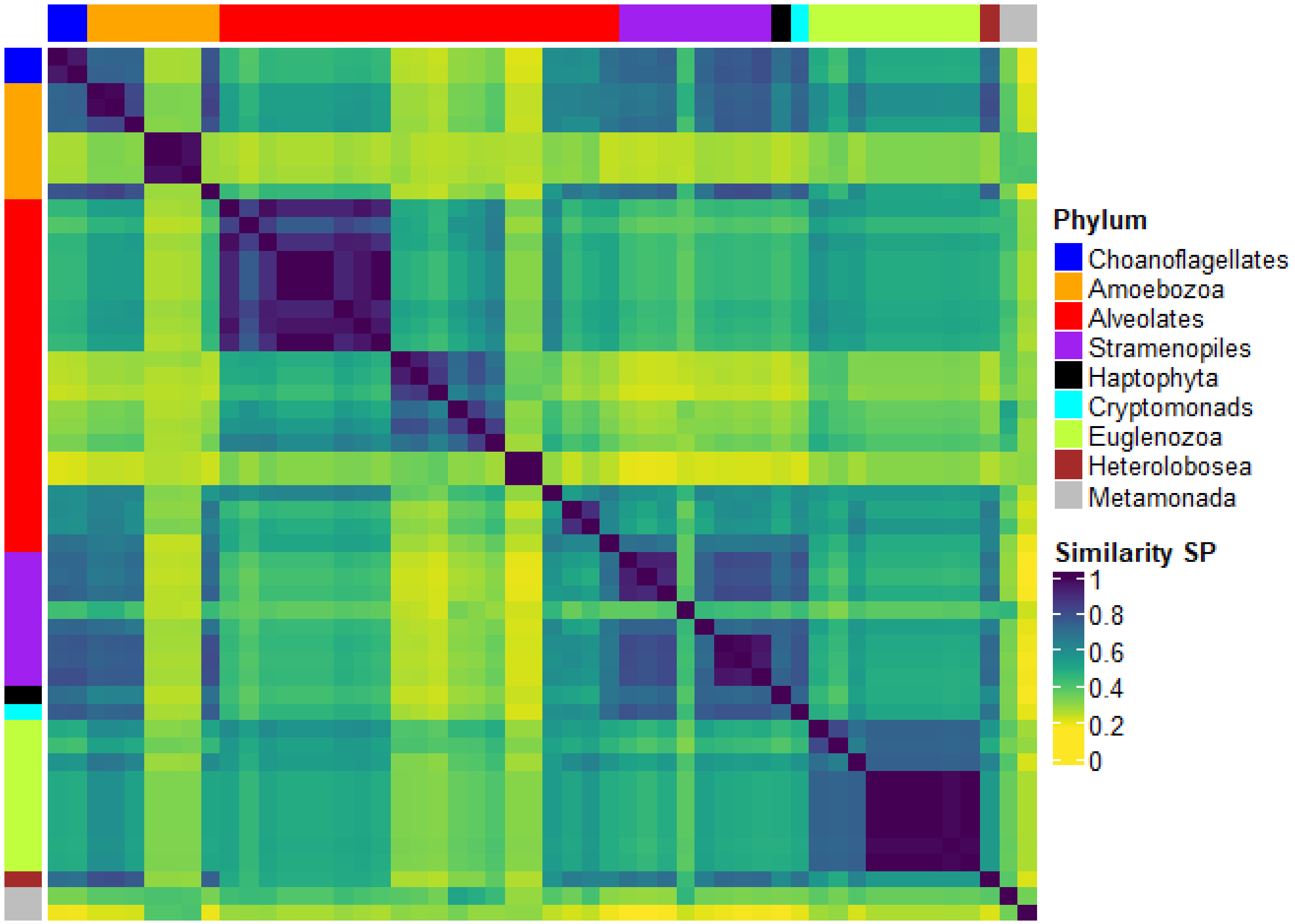
Phylum
Choanoflagellates
Amoebozoa
Alveolates
Stramenopiles
Haptophyta
Cryptomonads
Euglenozoa
Heterolobosea
Metamonada
Similarity SP
1
0.8
0.6
0.4
0.2
0

MDS for SP

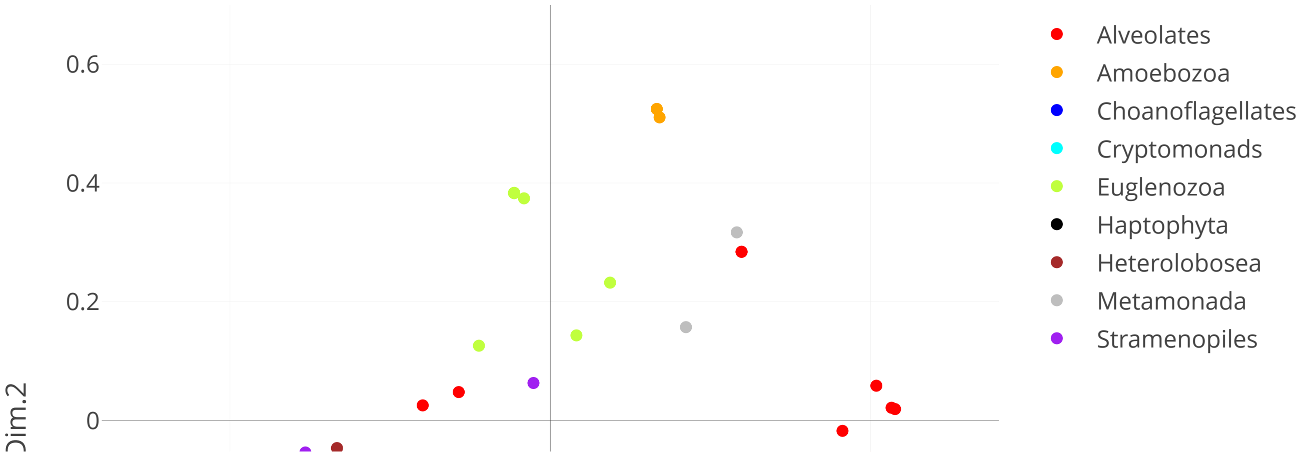
0.6
0.4
0.2
0
Dim.2
Alveolates
Amoebozoa
Choanoflagellates
Cryptomonads
Euglenozoa
Haptophyta
Heterolobosea
Metamonada
Stramenopiles

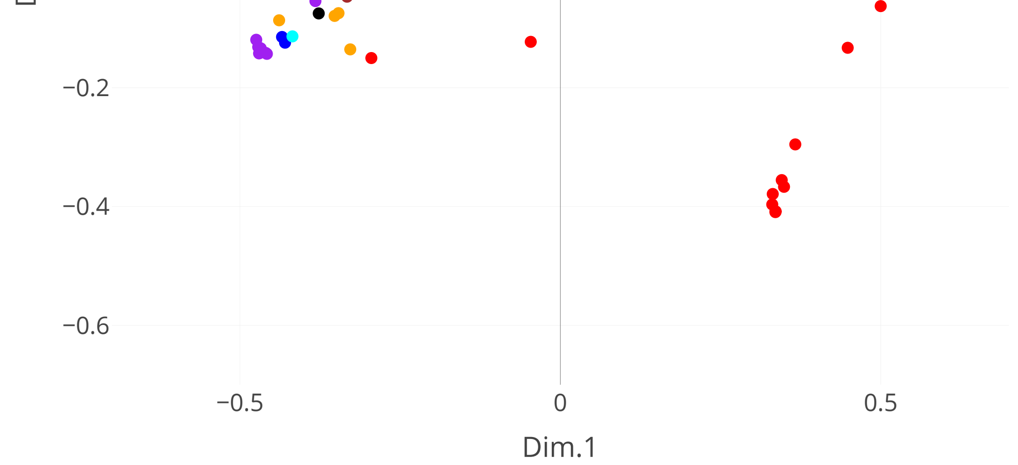

9-Means clustering for SP Kernel

```
##                      Cluster
## Real group            1 2 3 4 5 6 7 8 9
##   Alveolates          2 6 9 0 4 0 0 0 0
##   Amoebozoa           0 0 0 0 3 0 1 3 0
##   Choanoflagellates 0 0 0 0 0 0 2 0 0
##   Cryptomonads        0 0 0 0 0 0 1 0 0
##   Euglenozoa          0 0 0 0 0 6 0 0 3
##   Haptophyta          0 0 0 0 0 0 1 0 0
##   Heterolobosea       0 0 0 0 1 0 0 0 0
##   Metamonada          0 0 0 2 0 0 0 0 0
##   Stramenopiles       0 0 0 1 0 0 7 0 0
```

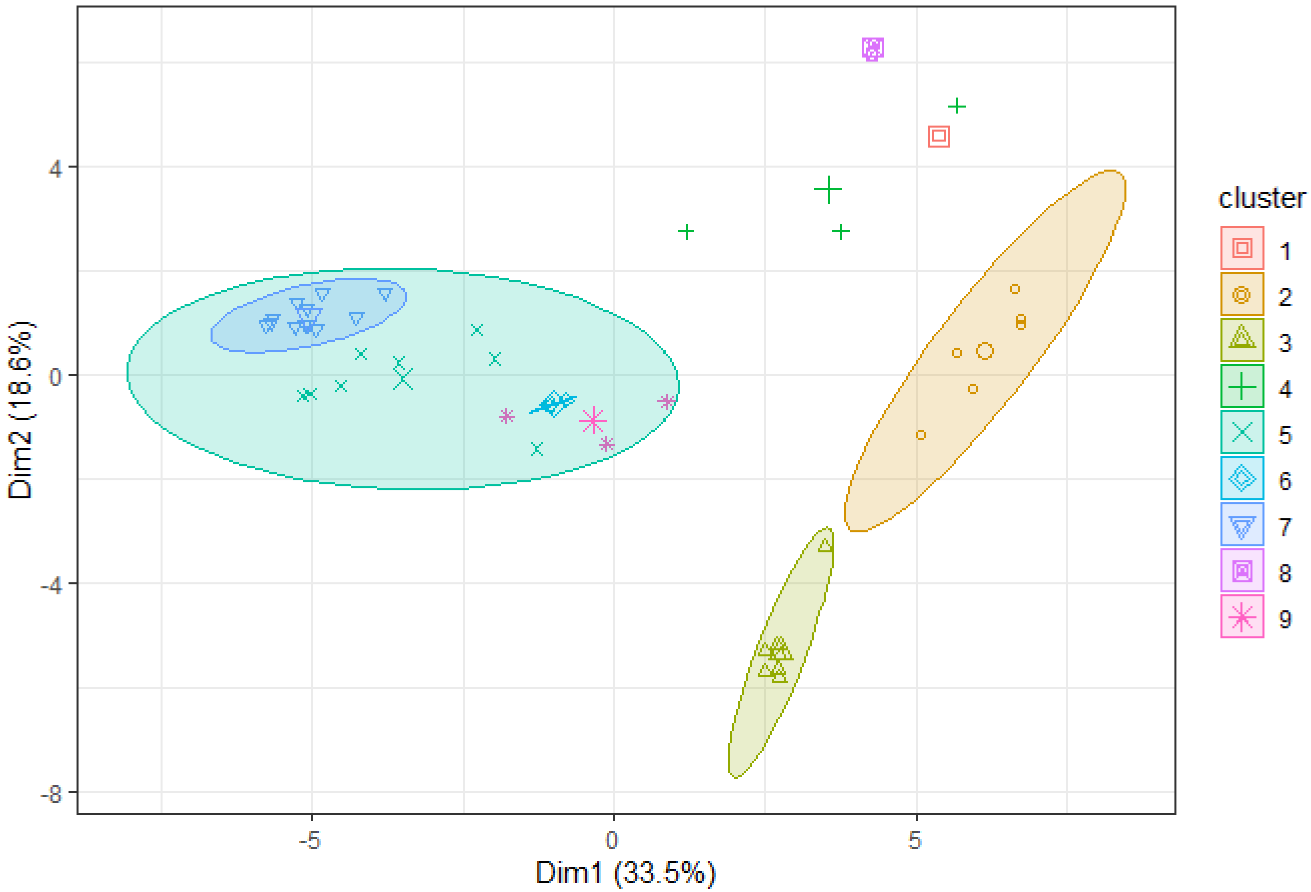

Organisms classified within cluster 1

```
## [1] "cpv" "cho"
```

Organisms classified within cluster 2

```
## [1] "tan"  "tpv"  "tot"  "beq"  "bbo"  "bmic"
```

Organisms classified within cluster 3

```
## [1] "pfa" "pfd" "pfh" "pyo" "pcb" "pbe" "pkn" "pvx" "pcy"
```

Organisms classified within cluster 4

```
## [1] "ngd" "tva" "gla"
```

Organisms classified within cluster 5

```
## [1] "ddi"  "dpp"  "dfa"  "tgo"  "tet"  "ptm"  "smin" "ngr"
```

Organisms classified within cluster 6

```
## [1] "lma"  "lif"  "ldo"  "lmi"  "lbz"  "lpan"
```

Organisms classified within cluster 7

```
##  [1] "mbr"  "sre"  "acan" "pti"  "fcy"  "tps"  "aaf"  "pif"  "psoj" "spar"
## [11] "ehx"  "gtt"
```

Organisms classified within cluster 8

```
## [1] "ehi" "edi" "eiv"
```

Organisms classified within cluster 9

```
## [1] "tbr" "tbg" "tcr"
```

Optimal number of clusters for SP

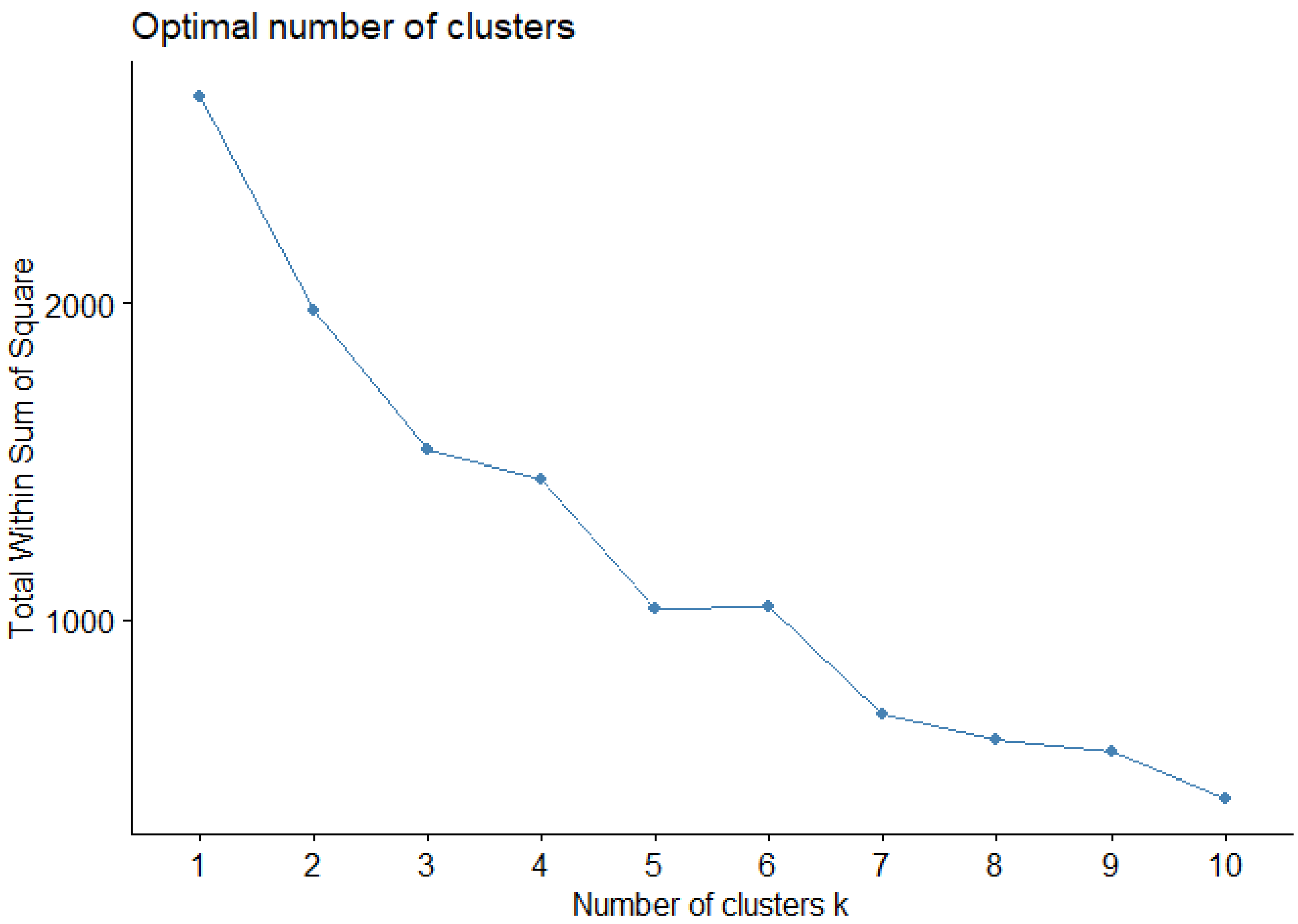


```
##                     Cluster
## Real group           1 2 3 4
##   Alveolates         8 9 4 0
##   Amoebozoa          3 0 4 0
##   Choanoflagellates  0 0 2 0
##   Cryptomonads       0 0 1 0
```

```
##    Euglenozoa         0 0 0 9
##    Haptophyta         0 0 1 0
##    Heterolobosea      0 0 1 0
##    Metamonada         2 0 0 0
##    Stramenopiles      1 0 7 0
```

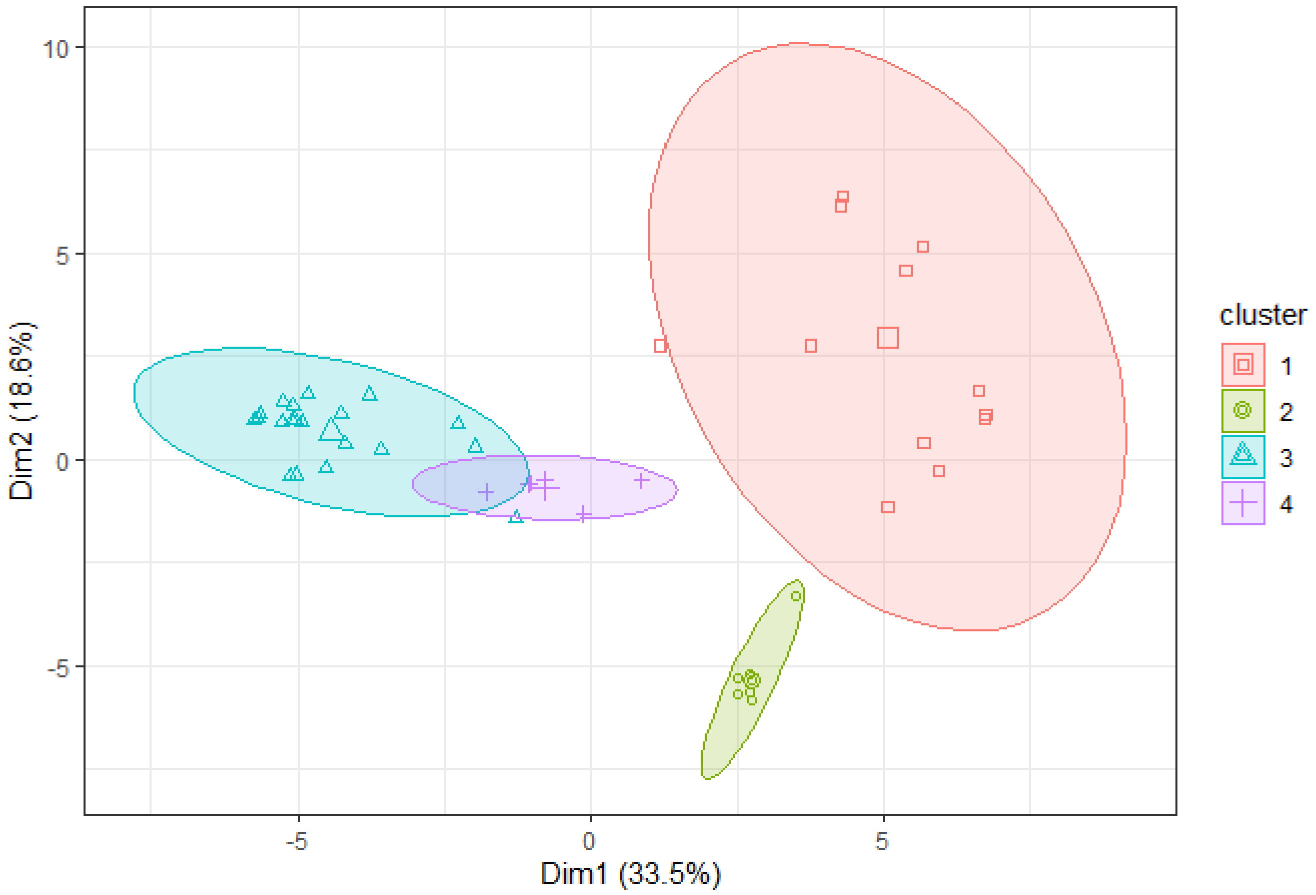

Organisms classified within cluster 1

```
##  [1] "ehi"   "edi"   "eiv"   "tan"   "tpv"   "tot"   "beq"   "bbo"   "bmic" "cpv" 
## [11] "cho"   "ngd"   "tva"   "gla"
```

Organisms classified within cluster 2

```
## [1] "pfa" "pfd" "pfh" "pyo" "pcb" "pbe" "pkn" "pvx" "pcy"
```

Organisms classified within cluster 3

```
##  [1] "mbr"   "sre"   "ddi"   "dpp"   "dfa"   "acan" "tgo"   "tet"   "ptm"   "smin"
## [11] "pti"   "fcy"   "tps"   "aaf"   "pif"   "psoj" "spar" "ehx"   "gtt"   "ngr"
```

Organisms classified within cluster 4

```
## [1] "tbr"   "tbg"   "tcr"   "lma"   "lif"   "ldo"   "lmi"   "lbz"   "lpan"
```

Weisfeiler-Lehman (WL) kernel

Heatmap

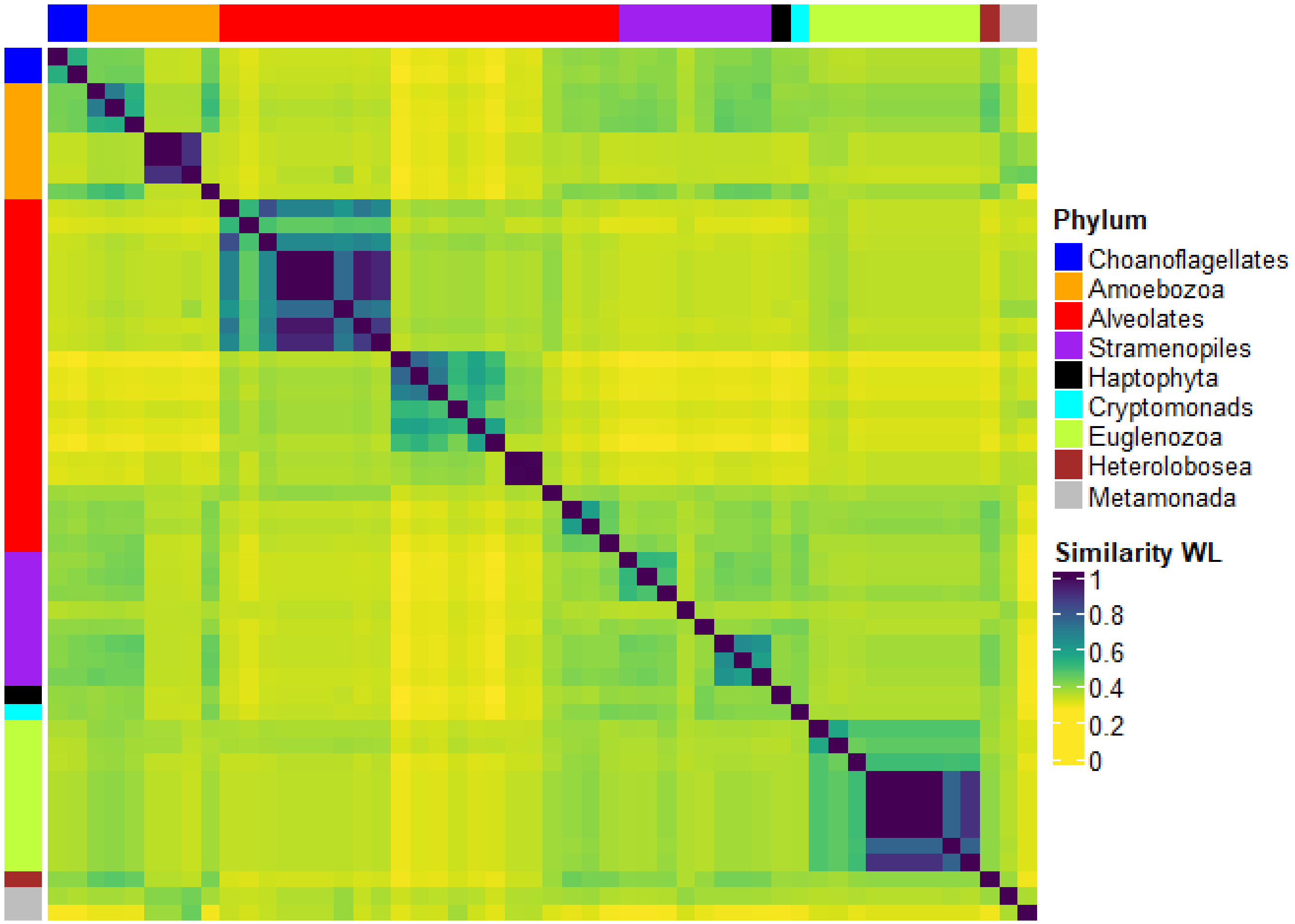
Phylum
Choanoflagellates
Amoebozoa
Alveolates
Stramenopiles
Haptophyta
Cryptomonads
Euglenozoa
Heterolobosea
Metamonada
Similarity WL
1
0.8
0.6
0.4
0.2
0

MDS for WL

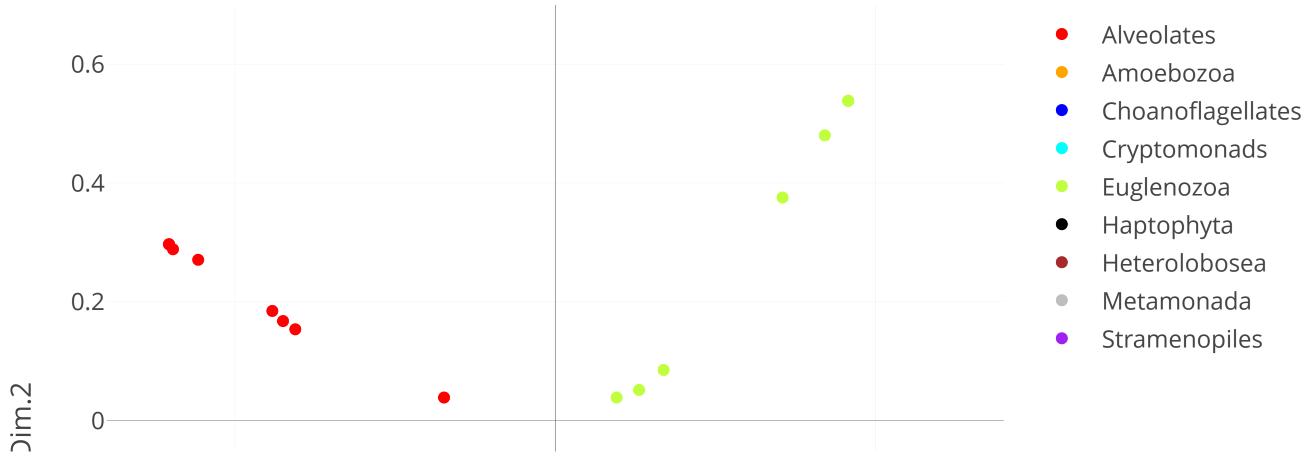
0.6
0.4
0.2
0
Dim.2
Alveolates
Amoebozoa
Choanoflagellates
Cryptomonads
Euglenozoa
Haptophyta
Heterolobosea
Metamonada
Stramenopiles

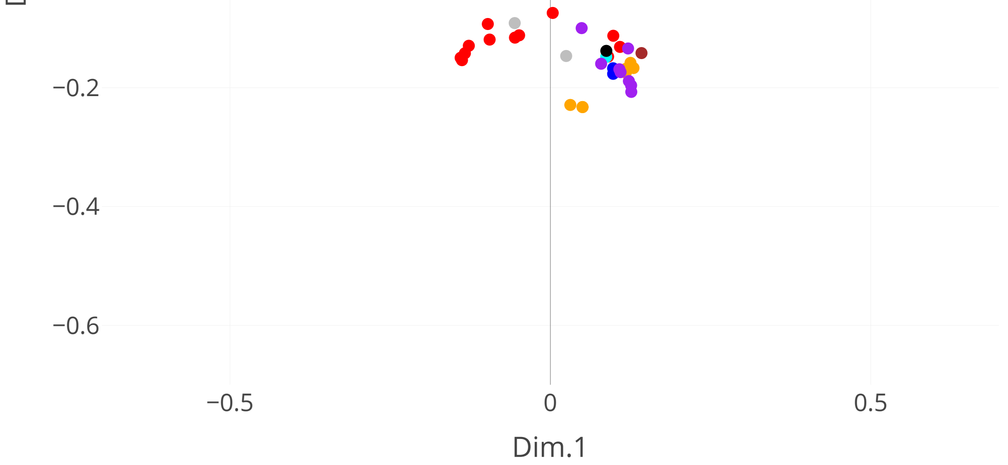

9-Means clustering for WL Kernel

```
##                        Cluster
## Real group              1 2 3 4 5 6 7 8 9
##    Alveolates           2 0 2 0 8 6 3 0 0
##    Amoebozoa            0 0 0 3 0 0 4 0 0
##    Choanoflagellates 0 0 0 0 0 0 2 0 0
##    Cryptomonads         0 0 0 0 0 0 0 1 0
##    Euglenozoa           3 6 0 0 0 0 0 0 0
##    Haptophyta           0 0 0 0 0 0 0 1 0
##    Heterolobosea        0 0 0 0 0 0 1 0 0
##    Metamonada           2 0 0 0 0 0 0 0 0
##    Stramenopiles        1 0 0 0 0 0 0 4 3
```

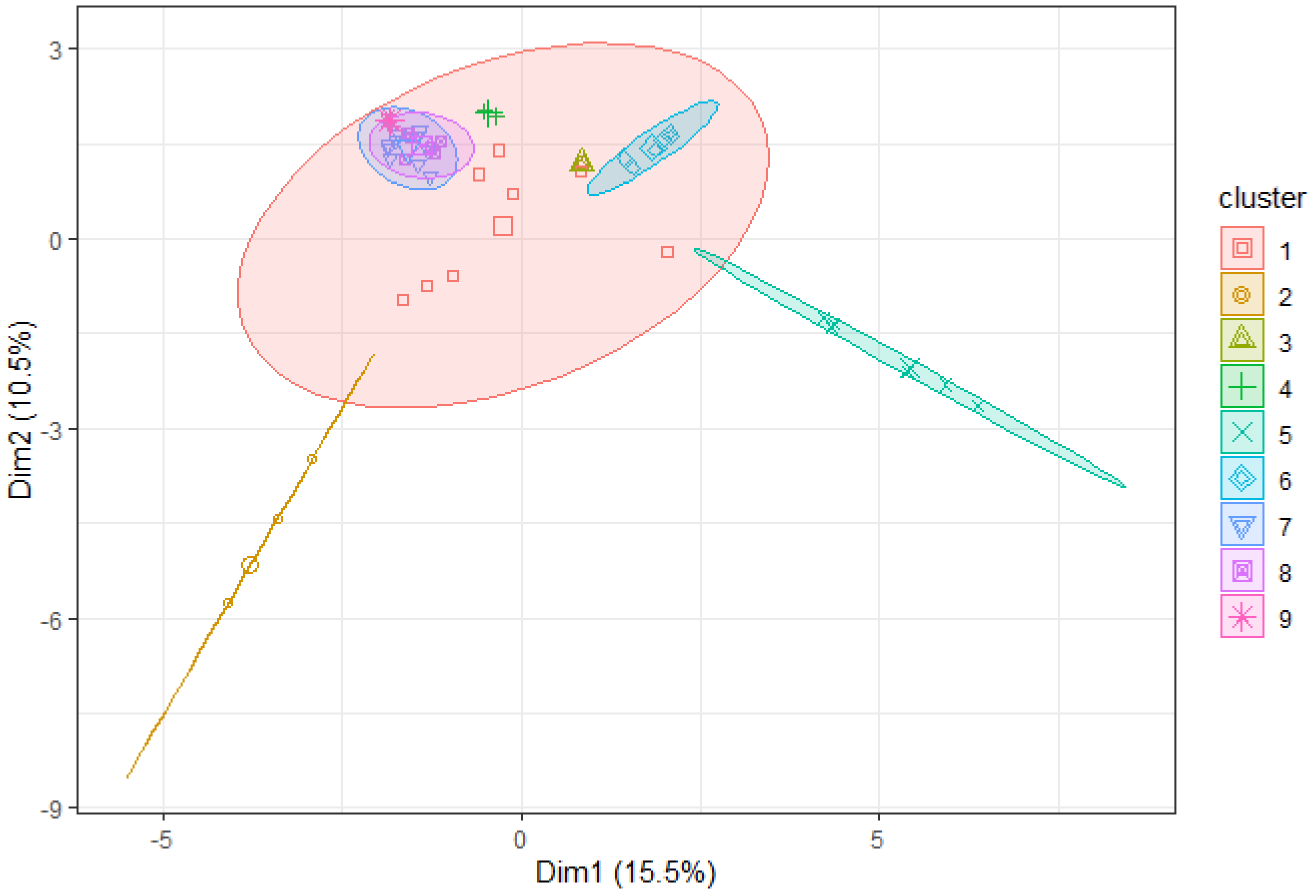

Organisms classified within cluster 1

```
## [1] "pfd" "tgo" "ngd" "tbr" "tbg" "tcr" "tva" "gla"
```

Organisms classified within cluster 2

```
## [1] "lma"  "lif"  "ldo"  "lmi"  "lbz"  "lpan"
```

Organisms classified within cluster 3

```
## [1] "cpv" "cho"
```

Organisms classified within cluster 4

```
## [1] "ehi" "edi" "eiv"
```

Organisms classified within cluster 5

```
## [1] "pfa" "pfh" "pyo" "pcb" "pbe" "pkn" "pvx" "pcy"
```

Organisms classified within cluster 6

```
## [1] "tan"  "tpv"  "tot"  "beq"  "bbo"  "bmic"
```

Organisms classified within cluster 7

```
##  [1] "mbr"  "sre"  "ddi"  "dpp"  "dfa"  "acan" "tet"  "ptm"  "smin" "ngr"
```

Organisms classified within cluster 8

```
## [1] "pti" "fcy" "tps" "aaf" "ehx" "gtt"
```

Organisms classified within cluster 9

```
## [1] "pif"  "psoj" "spar"
```

Optimal number of clusters for WL

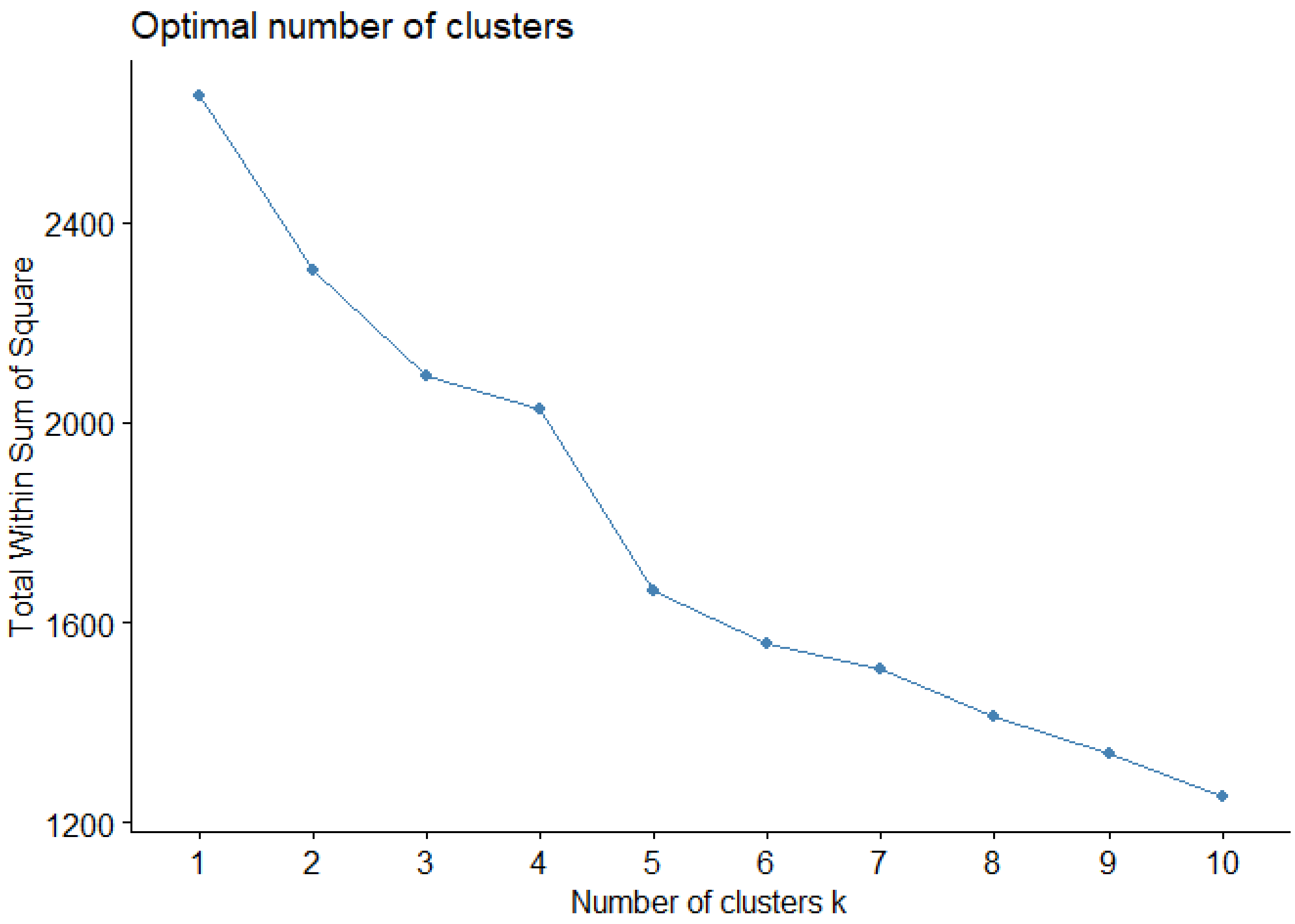


```
##                       Cluster
## Real group             1 2 3 4
##   Alveolates           8 4 0 9
##   Amoebozoa            0 7 0 0
##   Choanoflagellates 0 2 0 0
##   Cryptomonads         0 1 0 0
##   Euglenozoa           0 3 6 0
##   Haptophyta           0 1 0 0
##   Heterolobosea        0 1 0 0
```

```
##    Metamonada        0 1 0 1
##    Stramenopiles     0 8 0 0
```

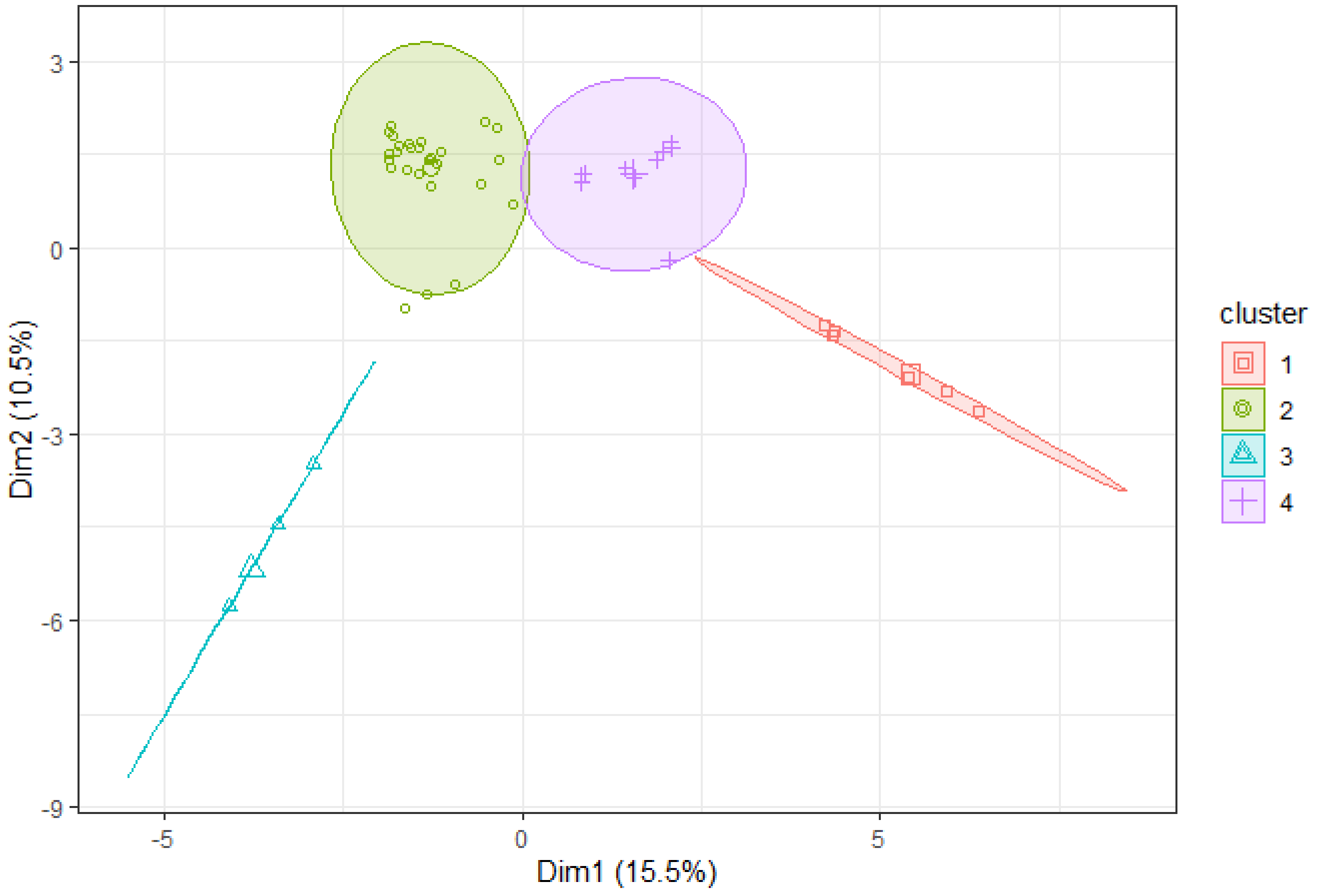

Organisms classified within cluster 1

```
## [1] "pfa" "pfh" "pyo" "pcb" "pbe" "pkn" "pvx" "pcy"
```

Organisms classified within cluster 2

```
##  [1] "mbr"  "sre"  "ddi"  "dpp"  "dfa"  "ehi"  "edi"  "eiv"  "acan" "tgo"
## [11] "tet"  "ptm"  "smin" "pti"  "fcy"  "tps"  "ngd"  "aaf"  "pif"  "psoj"
## [21] "spar" "ehx"  "gtt"  "tbr"  "tbg"  "tcr"  "ngr"  "tva"
```

Organisms classified within cluster 3

```
## [1] "lma"  "lif"  "ldo"  "lmi"  "lbz"  "lpan"
```

Organisms classified within cluster 4

```
##  [1] "pfd"  "tan"  "tpv"  "tot"  "beq"  "bbo"  "bmic" "cpv"  "cho"  "gla"
```

Pyramid match (PM) kernel

Heatmap

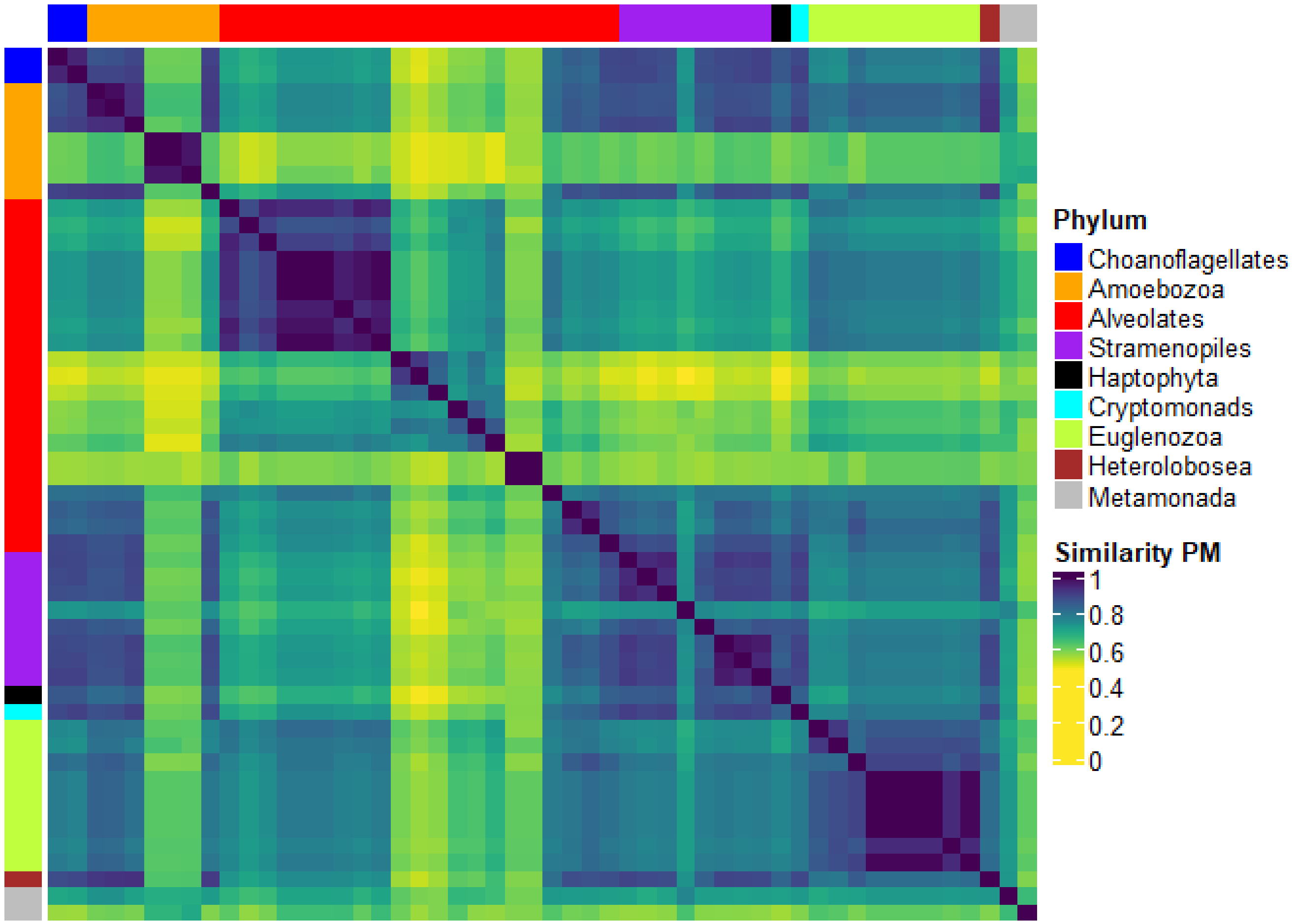
Phylum
Choanoflagellates
Amoebozoa
Alveolates
Stramenopiles
Haptophyta
Cryptomonads
Euglenozoa
Heterolobosea
Metamonada
Similarity PM
1
0.8
0.6
0.4
0.2
0

MDS for PM

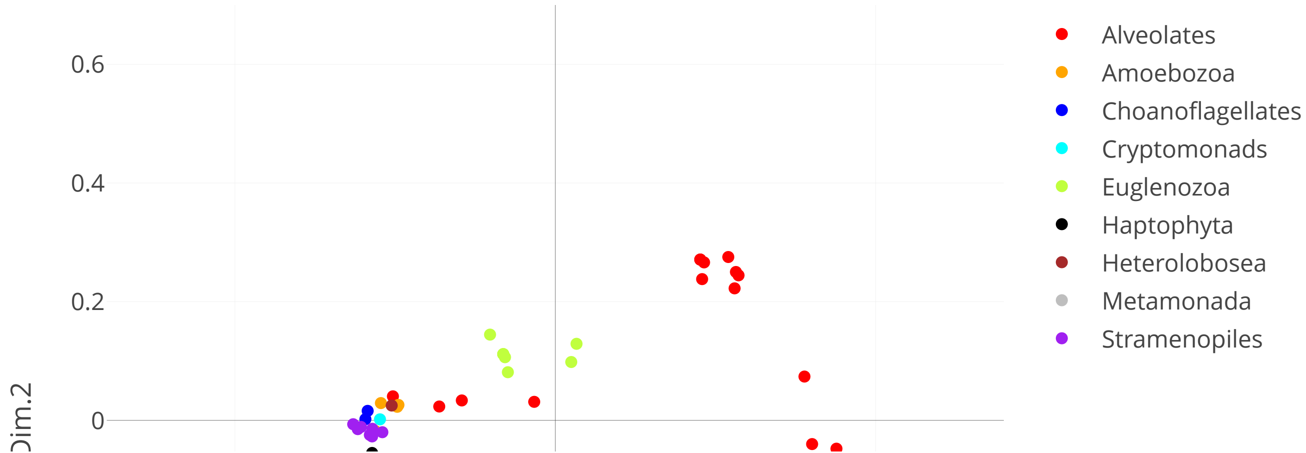
0.6
0.4
0.2
0
Dim.2
Alveolates
Amoebozoa
Choanoflagellates
Cryptomonads
Euglenozoa
Haptophyta
Heterolobosea
Metamonada
Stramenopiles

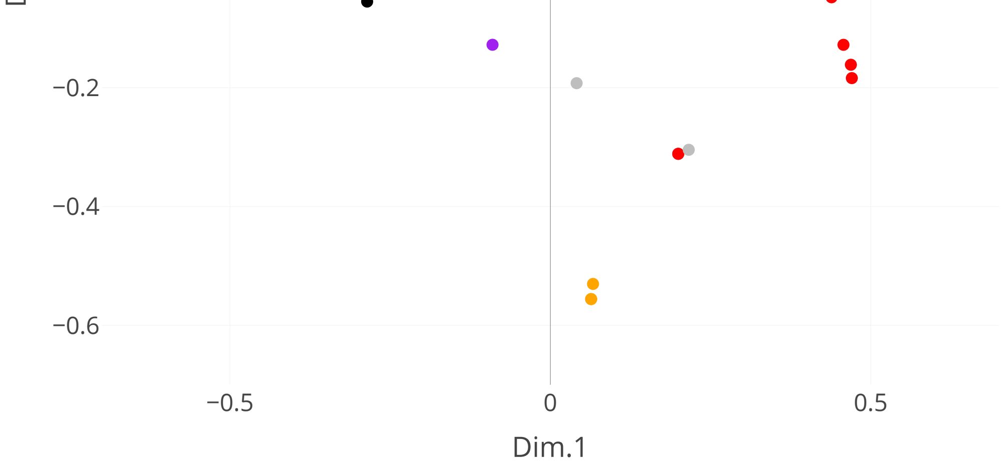

9-Means clustering for PM Kernel

```
##                     Cluster
## Real group           1 2 3 4 5 6 7 8 9
##   Alveolates         0 2 9 3 4 0 0 3 0
##   Amoebozoa          3 0 0 0 4 0 0 0 0
##   Choanoflagellates  0 0 0 0 2 0 0 0 0
##   Cryptomonads       0 0 0 0 1 0 0 0 0
##   Euglenozoa         0 0 0 0 0 9 0 0 0
##   Haptophyta         0 0 0 0 1 0 0 0 0
##   Heterolobosea      0 0 0 0 1 0 0 0 0
##   Metamonada         0 0 0 0 0 0 1 0 1
##   Stramenopiles      0 0 0 0 7 0 1 0 0
```

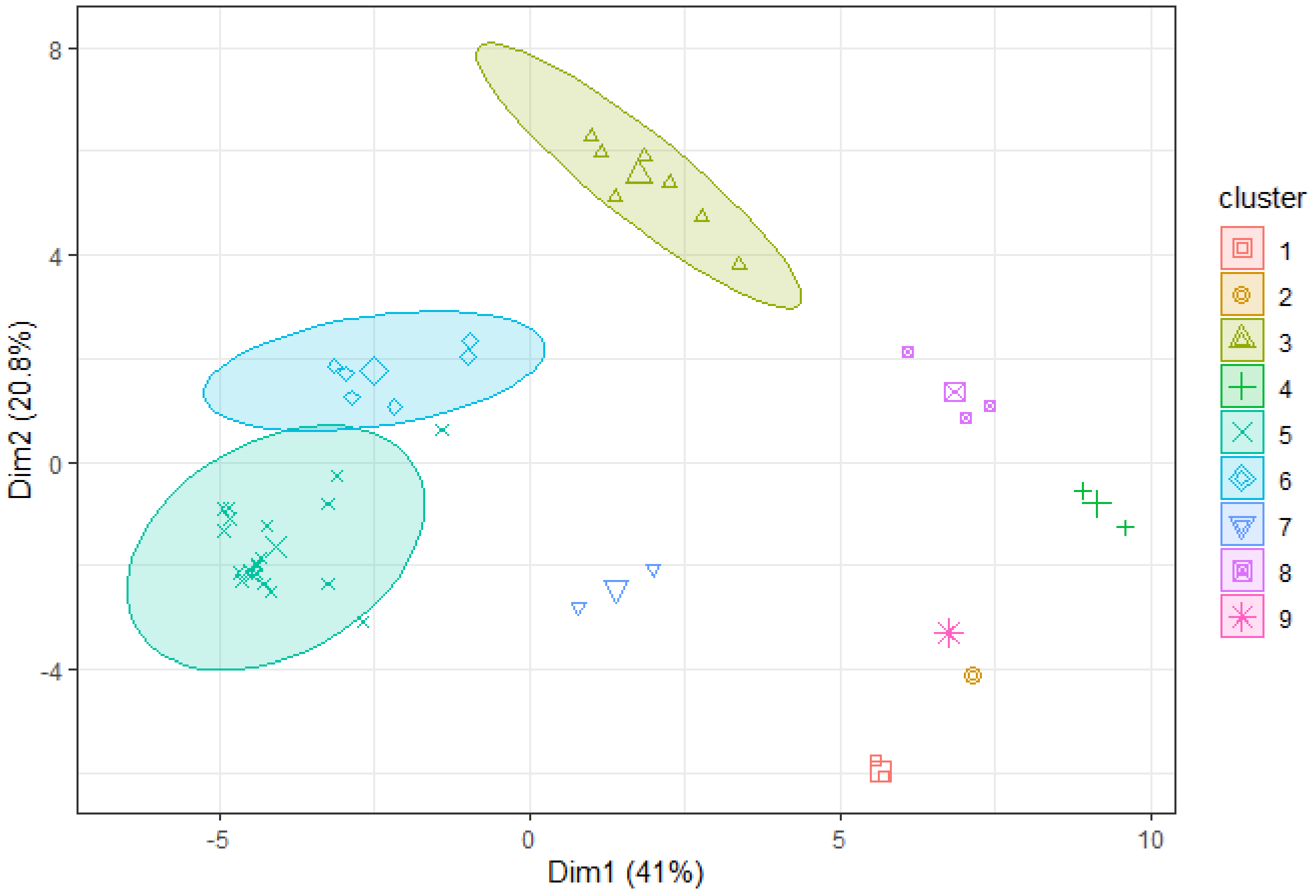

Organisms classified within cluster 1

```
## [1] "ehi" "edi" "eiv"
```

Organisms classified within cluster 2

```
## [1] "cpv" "cho"
```

Organisms classified within cluster 3

```
## [1] "pfa" "pfd" "pfh" "pyo" "pcb" "pbe" "pkn" "pvx" "pcy"
```

Organisms classified within cluster 4

```
## [1] "tan" "tpv" "tot"
```

Organisms classified within cluster 5

```
##  [1] "mbr"  "sre"  "ddi"  "dpp"  "dfa"  "acan" "tgo"  "tet"  "ptm"  "smin"
## [11] "pti"  "fcy"  "tps"  "aaf"  "pif"  "psoj" "spar" "ehx"  "gtt"  "ngr"
```

Organisms classified within cluster 6

```
## [1] "tbr"  "tbg"  "tcr"  "lma"  "lif"  "ldo"  "lmi"  "lbz"  "lpan"
```

Organisms classified within cluster 7

```
## [1] "ngd" "tva"
```

Organisms classified within cluster 8

```
## [1] "beq"  "bbo"  "bmic"
```

Organisms classified within cluster 9

```
## [1] "gla"
```

Optimal number of clusters for PM

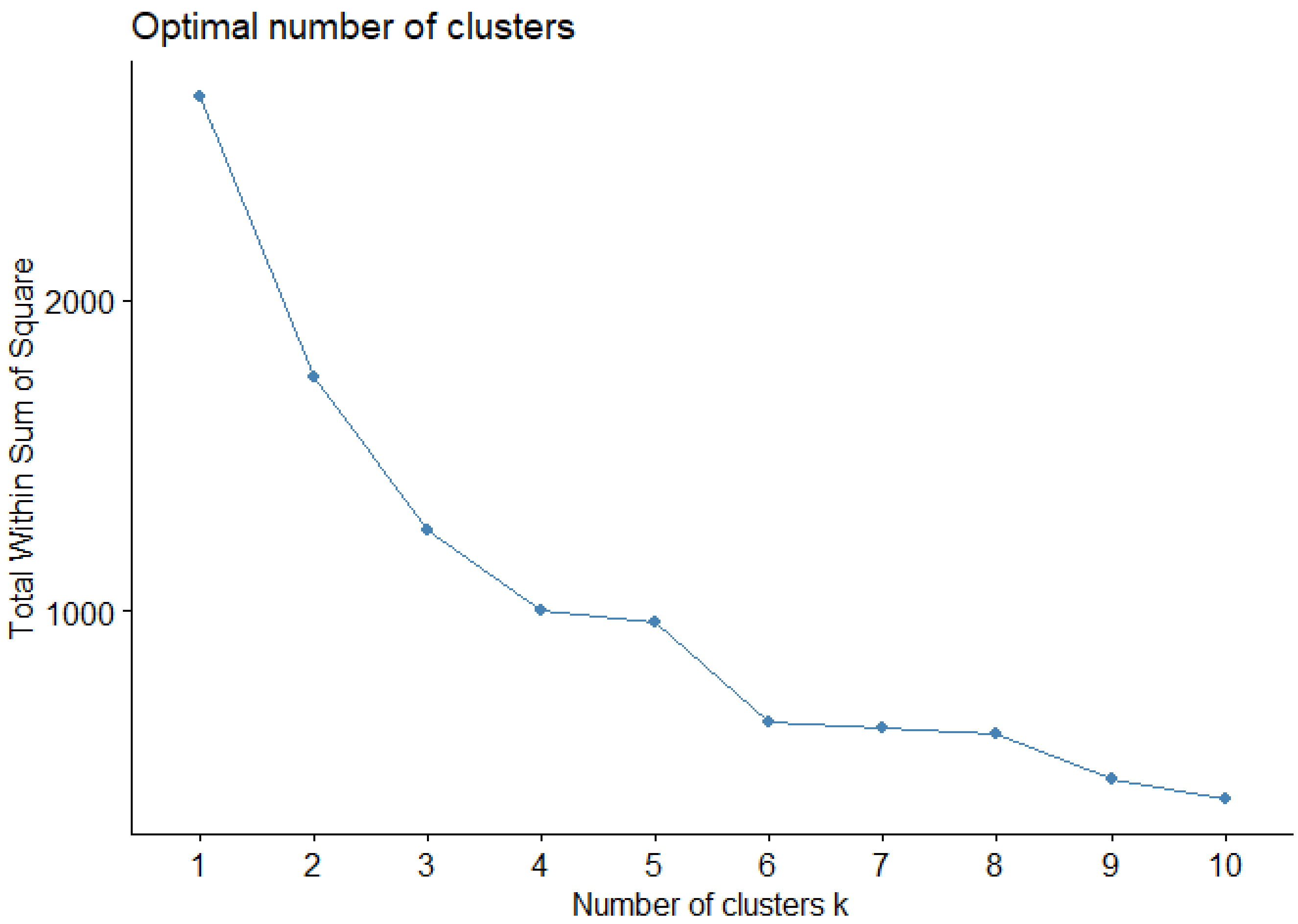


```
##                      Cluster
## Real group            1 2 3 4
##   Alveolates          0 4 8 9
##   Amoebozoa           0 4 3 0
##   Choanoflagellates 0 2 0 0
##   Cryptomonads        0 1 0 0
```

```
##    Euglenozoa        9 0 0 0
##    Haptophyta        0 1 0 0
##    Heterolobosea     0 1 0 0
##    Metamonada        0 0 2 0
##    Stramenopiles     0 8 0 0
```

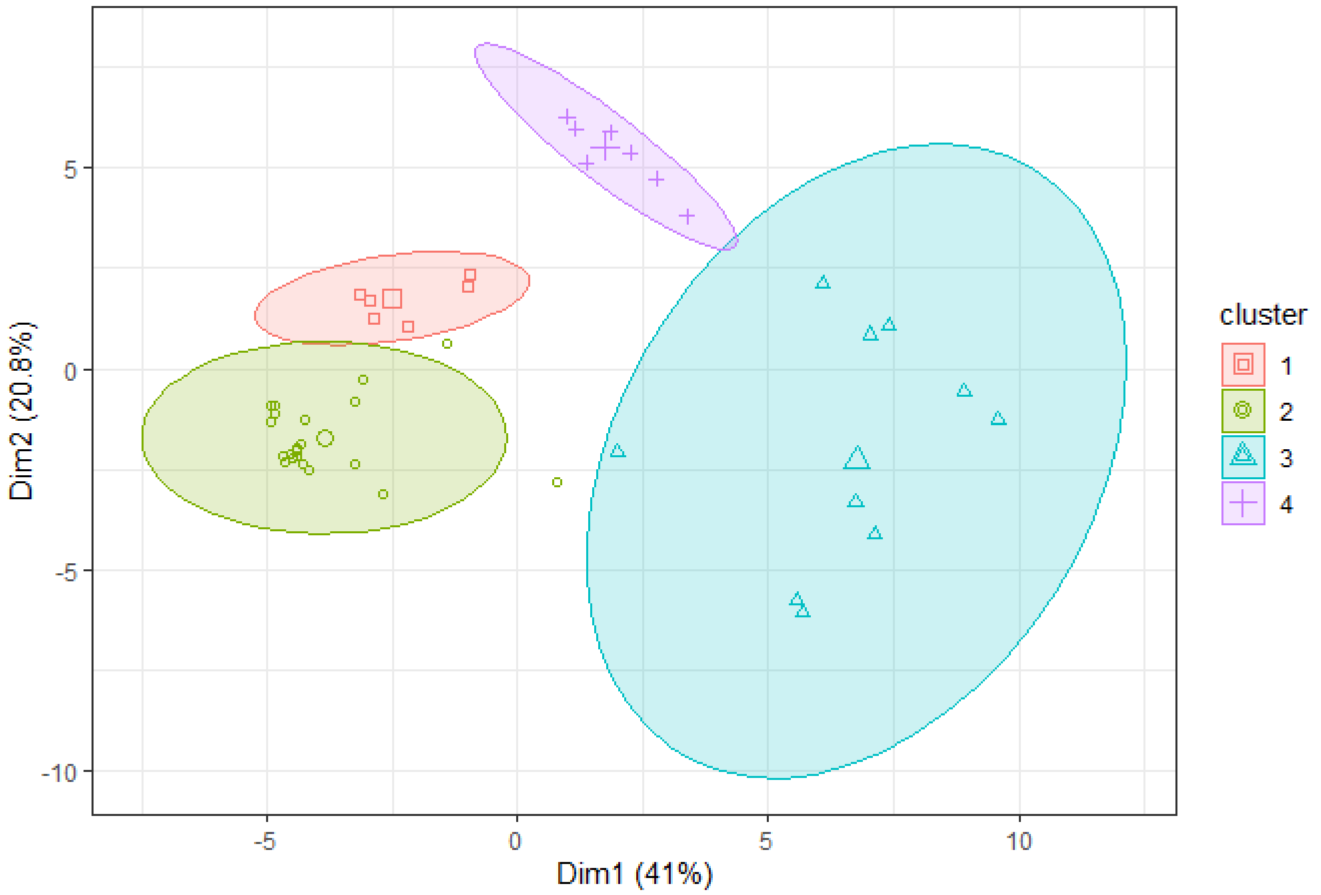

Organisms classified within cluster 1

```
## [1] "tbr"  "tbg"  "tcr"  "lma"  "lif"  "ldo"  "lmi"  "lbz"  "lpan"
```

Organisms classified within cluster 2

```
##  [1] "mbr"  "sre"  "ddi"  "dpp"  "dfa"  "acan" "tgo"  "tet"  "ptm"  "smin"
## [11] "pti"  "fcy"  "tps"  "ngd"  "aaf"  "pif"  "psoj" "spar" "ehx"  "gtt"
## [21] "ngr"
```

Organisms classified within cluster 3

```
##  [1] "ehi"  "edi"  "eiv"  "tan"  "tpv"  "tot"  "beq"  "bbo"  "bmic" "cpv"
## [11] "cho"  "tva"  "gla"
```

Organisms classified within cluster 4

```
## [1] "pfa" "pfd" "pfh" "pyo" "pcb" "pbe" "pkn" "pvx" "pcy"
```
