# Supplementary material for: Exploring the expressiveness of abstract metabolic networks
Source: PLoS One. 2023 Feb 9;18(2):e0281047. doi: 10.1371/journal.pone.0281047 (PMC9910719; doi:10.1371/journal.pone.0281047)
Supplement: S11 File — Bacteria analyses at phylum level (third experiment). Only the phyla Actinobacteria, Bacteroidetes, Firmicutes, Proteobacteria and Tenericutes present in the list S1 File have been considered. (PDF) [file pone.0281047.s011.pdf]

Bacteria Restricted Analysis

- Vertex hystogram (VH) kernel
  - Heatmap
  - MDS for VH
  - 5-means clustering for VH
- Shortest Path (SP) kernel
  - Heatmap
  - MDS for SP
  - 5-means clustering for SP
- Weisfeiler-Lehman (WL) kernel
  - Heatmap
  - MDS for WL
  - 5-means clustering for WL
- Pyramid match (PM) kernel
  - Heatmap
  - MDS for PM
  - 5-means clustering for PM

Vertex hystogram (VH) kernel

Heatmap

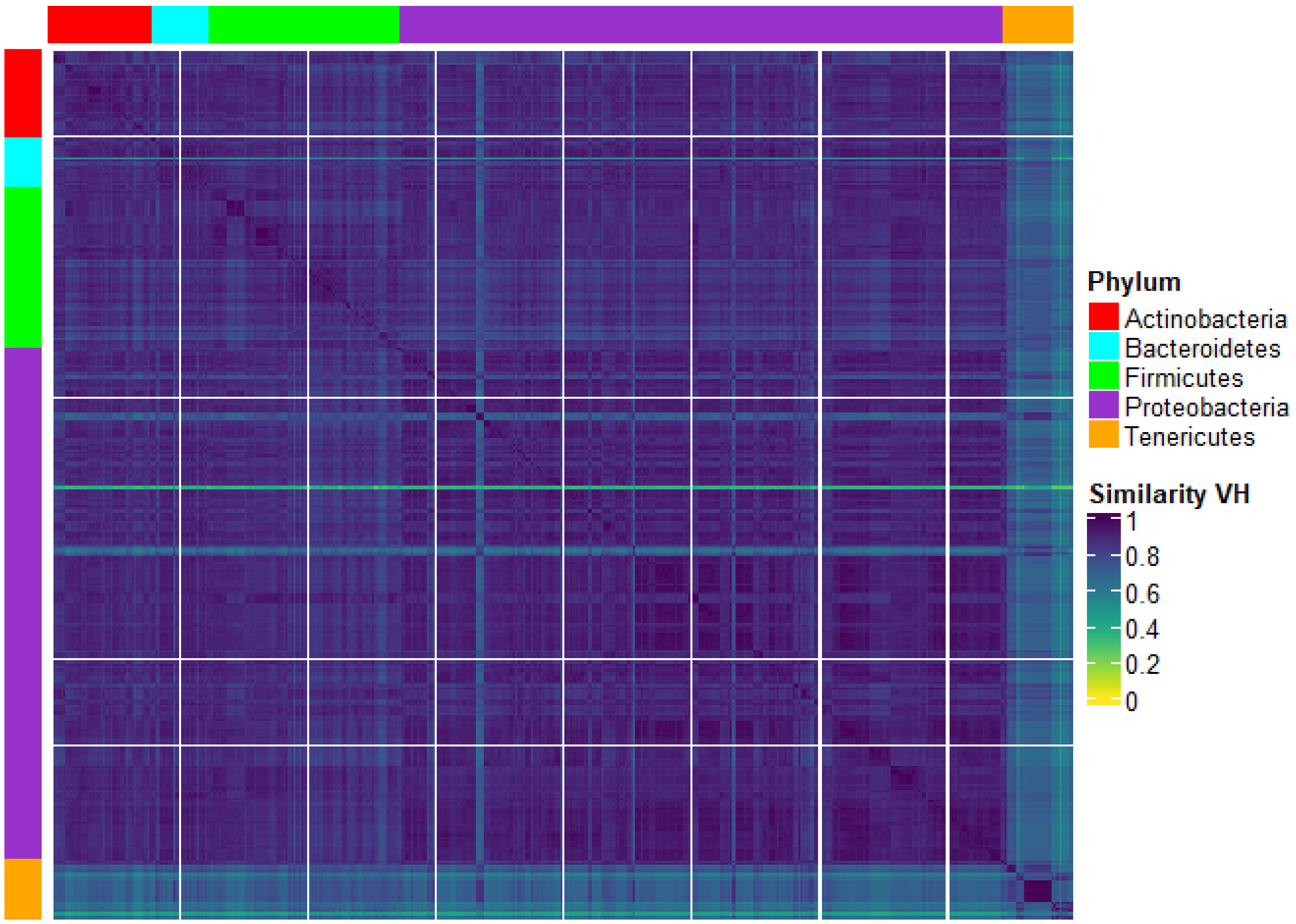

MDS for VH

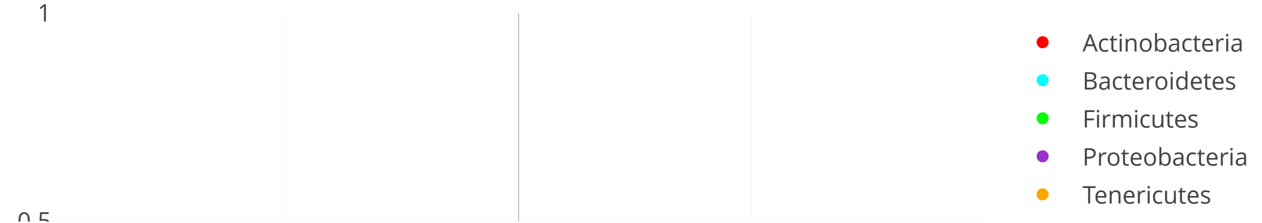

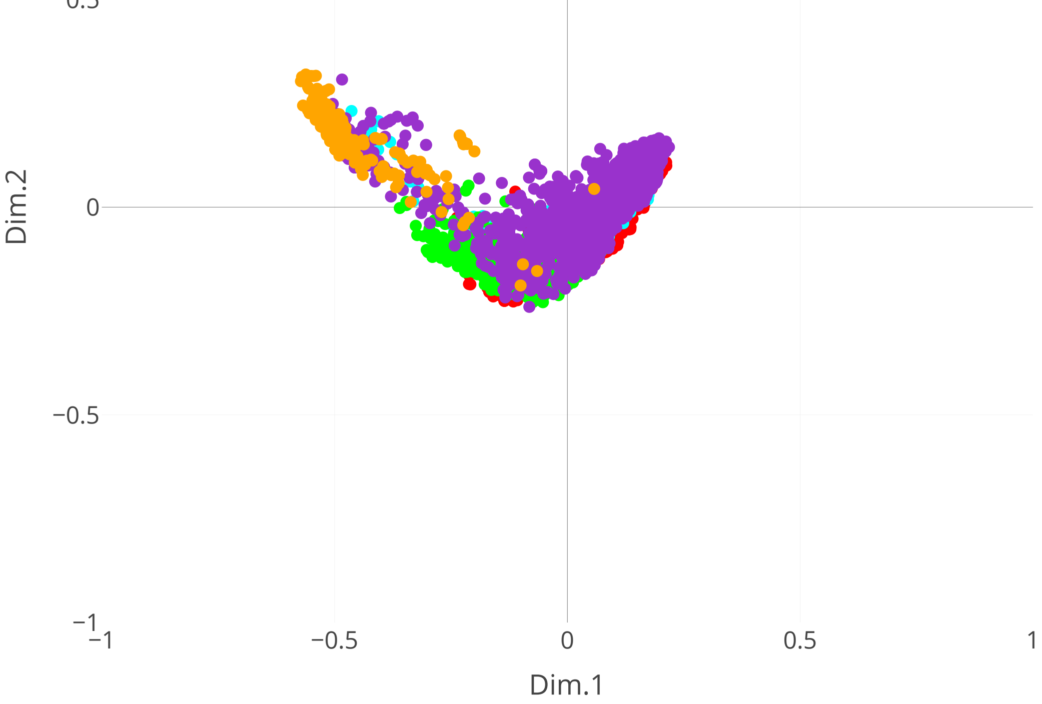

5-means clustering for VH

```
##                  Cluster
## Real group          1    2    3    4    5
##   Actinobacteria    1  168    1  195  106
##   Bacteroidetes    12   97    9   95   47
##   Firmicutes        0  398   11  102  358
##   Proteobacteria   34  651   93 1810  160
##   Tenericutes     125    2  192    1    4
```

Organisms classified within cluster 1

```
##  [1] "nfe"  "che"  "cec"  "cher" "smg"  "sms"  "smh"  "sum"  "smv"  "smub"
## [11] "smum" "smue" "smup" "hci"  "hct"  "hcc"  "hcd"  "ndl"  "tpn"  "tpq"
## [21] "tpj"  "vfg"  "zin"  "bcc"  "baj"  "men"  "meo"  "ppet" "aen"  "cmik"
## [31] "crp"  "cru"  "crc"  "crt"  "crh"  "crv"  "cri"  "ple"  "ply"  "plr"
## [41] "plo"  "pld"  "plb"  "plc"  "pli"  "paly" "baab" "elj"  "esx"  "efr"
```

```
##  [51] "eml"  "mfl"  "mfw"  "mchc" "mlac" "ment" "msyr" "mtab" "mcol" "sdi" 
##  [61] "sll"  "skn"  "scj"  "sck"  "sphh" "smoo" "pml"  "pal"  "psol" "pzi" 
##  [71] "mge"  "mgu"  "mgc"  "mgq"  "mgx"  "mpn"  "mpm"  "mpj"  "mpb"  "mga" 
##  [81] "mgh"  "mgf"  "mgn"  "mgs"  "mgt"  "mgv"  "mgw"  "mgac" "mgan" "mgnc"
##  [91] "mgz"  "mmy"  "mmym" "mmyi" "mml"  "mcp"  "mcac" "mcap" "mcar" "mcai"
## [101] "mlc"  "mlh"  "mmo"  "mhy"  "mhj"  "mhp"  "mhn"  "mhyl" "mhyo" "mat" 
## [111] "mco"  "mho"  "mhom" "mcd"  "mhr"  "mhh"  "mhm"  "mhs"  "mhv"  "mha" 
## [121] "mhf"  "mss"  "msk"  "mpf"  "mput" "mhe"  "mwe"  "mhl"  "mhb"  "mpv" 
## [131] "mov"  "mbc"  "mgj"  "mfq"  "mcan" "myt"  "mds"  "myg"  "mpho" "mhyv"
## [141] "mclo" "mamp" "mans" "mphc" "mane" "mnh"  "mnu"  "mstr" "mgly" "mcou"
## [151] "mcom" "mpu"  "msy"  "mso"  "maa"  "mal"  "mbv"  "mbh"  "mbi"  "mbq" 
## [161] "mcy"  "mcas" "mck"  "marg" "mbov" "mboh" "mani" "mphi" "uur"  "upa" 
## [171] "upr"  "uue"
```

Organisms classified within cluster 2

```
##   [1] "afo"  "tbw"  "bde"  "bdn"  "bsca" "beu"  "nak"  "gez"  "oek"  "bfa" 
##  [11] "brv"  "brr"  "ars"  "dni"  "day"  "aqg"  "cry"  "lxx"  "lxy"  "leif"
##  [21] "leu"  "leb"  "ldn"  "mvd"  "rtx"  "rtc"  "cgl"  "cgb"  "cgu"  "cgt" 
##  [31] "cgs"  "cgg"  "cgm"  "cgj"  "cgq"  "cgx"  "cdi"  "cdp"  "cdh"  "cdt" 
##  [41] "cde"  "cdr"  "cda"  "cdz"  "cdb"  "cds"  "cdd"  "cdw"  "cdv"  "cdip"
##  [51] "cjk"  "cur"  "cua"  "car"  "ckp"  "cpl"  "cpg"  "cpp"  "cpk"  "cpq" 
##  [61] "cpx"  "cpz"  "cor"  "cop"  "cod"  "cos"  "coi"  "coe"  "cou"  "cpse"
##  [71] "cpsu" "cpsf" "crd"  "cul"  "cuc"  "cue"  "cun"  "cus"  "cuq"  "cuz" 
##  [81] "cuj"  "cva"  "ccn"  "cter" "cmd"  "caz"  "cfn"  "ccg"  "cvt"  "cii" 
##  [91] "cuv"  "coa"  "cdo"  "chm"  "csx"  "cku"  "ccj"  "cmv"  "cei"  "cted"
## [101] "clw"  "cdx"  "csp"  "csta" "ccjz" "cfk"  "cpho" "cfc"  "cgv"  "cstr"
## [111] "caqu" "csph" "camg" "cmin" "cpeg" "cxe"  "cee"  "csan" "cgk"  "crf" 
## [121] "crl"  "ccho" "cpso" "rkr"  "mix"  "mip"  "mcw"  "mih"  "maur" "mfol"
## [131] "moo"  "mlv"  "iva"  "ido"  "cfl"  "cfi"  "cga"  "cez"  "celz" "cej" 
## [141] "celh" "halt" "ske"  "sanw" "cet"  "cceu" "noy"  "noi"  "ndp"  "nsn" 
## [151] "pak"  "pfr"  "pfre" "prl"  "paus" "tfl"  "tfa"  "tes"  "tez"  "tdf" 
## [161] "tla"  "kii"  "kod"  "kvr"  "tfu"  "ard"  "erz"  "rrd"  "pmuc" "pet" 
```

```
## [171] "buy"  "osp"  "ald"  "aok"  "acou" "ait"  "blq"  "nso"  "nia"  "fgg" 
## [181] "sgn"  "lby"  "flu"  "aqd"  "aue"  "ccyn" "caph" "csto" "capq" "cagg"
## [191] "emar" "gfo"  "grl"  "mgel" "mesq" "mrs"  "mlt"  "myr"  "mpw"  "mod" 
## [201] "myz"  "nom"  "nsd"  "rbi"  "lan"  "lvn"  "laci" "for"  "foh"  "fop" 
## [211] "taj"  "marf" "ahz"  "tdi"  "ten"  "tje"  "tmar" "tmp"  "lut"  "fek" 
## [221] "asl"  "aev"  "wfu"  "wij"  "psyn" "zpr"  "cnr"  "eao"  "emn"  "een" 
## [231] "elb"  "emg"  "ego"  "egm"  "elz"  "elt"  "wvi"  "ebv"  "efal" "este"
## [241] "ran"  "rai"  "rar"  "rag"  "rae"  "rat"  "fba"  "fbu"  "oho"  "mgin"
## [251] "oli"  "psn"  "pgs"  "pej"  "proe" "cte"  "cpc"  "clz"  "pros" "cpb" 
## [261] "cli"  "ial"  "cbae" "chih" "rbar" "apak" "axl"  "aia"  "afl"  "agn" 
## [271] "anm"  "aamy" "anl"  "and"  "acai" "bfv"  "bri"  "blut" "fpn"  "gth" 
## [281] "ptl"  "ptb"  "grc"  "oih"  "ocn"  "pof"  "sje"  "tap"  "hhd"  "hmn" 
## [291] "hli"  "lao"  "gka"  "gte"  "gtk"  "gtm"  "gli"  "gtn"  "gwc"  "gyc" 
## [301] "gya"  "gct"  "gmc"  "ggh"  "gjf"  "gea"  "gel"  "gse"  "gsr"  "gej" 
## [311] "bths" "anx"  "coh"  "cohn" "esi"  "eat"  "ean"  "exm"  "exu"  "saca"
## [321] "tco"  "bbe"  "blr"  "bfm"  "bagr" "brw"  "jeo"  "lsp"  "lgy"  "lfu" 
## [331] "lys"  "lyz"  "lpak" "pln"  "pku"  "prt"  "pll"  "pana" "pdg"  "phc" 
## [341] "ppla" "pfae" "plx"  "pmat" "pdec" "psyh" "psyo" "rst"  "siv"  "sob" 
## [351] "spsy" "spor" "spop" "sure" "spos" "spae" "pgq"  "play" "panc" "vir" 
## [361] "vhl"  "vig"  "vil"  "vne"  "vpn"  "vim"  "stea" "macr" "shv"  "sau" 
## [371] "sav"  "saw"  "sah"  "saj"  "sam"  "sas"  "sar"  "sac"  "sax"  "saa" 
## [381] "sao"  "sae"  "sad"  "suu"  "suv"  "sue"  "suj"  "suk"  "suc"  "sut" 
## [391] "suq"  "suz"  "sud"  "sux"  "suw"  "sug"  "suf"  "saua" "saue" "saun"
## [401] "saus" "sauu" "saug" "sauz" "saut" "sauj" "sauk" "sauq" "sauv" "sauw"
## [411] "saux" "sauy" "sauf" "sab"  "suy"  "saub" "saum" "sauc" "saur" "saui"
## [421] "saud" "sams" "suh"  "ser"  "sep"  "sepp" "seps" "sha"  "shh"  "ssp" 
## [431] "sca"  "slg"  "sln"  "ssd"  "sdt"  "sdp"  "swa"  "sxy"  "sxl"  "sxo" 
## [441] "shu"  "ssch" "sscz" "sagq" "seqo" "ssif" "scv"  "slz"  "snl"  "skl" 
## [451] "sfq"  "shom" "smus" "scar" "schr" "sarl" "spic" "ssh"  "ssim" "kpul"
## [461] "keb"  "lfb"  "pjd"  "gym"  "ppy"  "ppm"  "ppo"  "ppol" "ppq"  "ppoy"
## [471] "pta"  "plv"  "psab" "pdu"  "pbd"  "pgm"  "pod"  "paen" "paef" "paeq"
## [481] "pste" "paea" "paee" "paeh" "paej" "pbj"  "pih"  "pri"  "ppeo" "pow" 
## [491] "pbv"  "pxl"  "pyg"  "pswu" "pdh"  "pib"  "pcx"  "pkb"  "paih" "pvo" 
## [501] "plw"  "plen" "plut" "pchi" "pprt" "pbac" "prz"  "plyc" "tvu"  "aui" 
```

```
## [511] "avs"  "jep"  "jeh"  "jar"  "jpo"  "thl"  "tey"  "tkr"  "vte"  "oen"
## [521] "lci"  "lki"  "lec"  "lge"  "lsu"  "lpse" "lla"  "llk"  "llt"  "lls"
## [531] "lld"  "llx"  "llj"  "llm"  "llc"  "llr"  "lln"  "lli"  "llw"  "lpk"
## [541] "lrn"  "lact" "smu"  "smc"  "smut" "smj"  "smua" "ssb"  "ssu"  "ssv"
## [551] "ssi"  "sss"  "ssw"  "sup"  "ssus" "ssuy" "ssk"  "ssq"  "sui"  "suo"
## [561] "srp"  "ssut" "ssui" "sub"  "sds"  "sdg"  "sda"  "sdc"  "sdq"  "stk"
## [571] "ssr"  "stj"  "strs" "ssah" "sik"  "siq"  "sio"  "siz"  "spat" "seqi"
## [581] "srat" "efl"  "efi"  "efd"  "efs"  "efn"  "efq"  "emu"  "ega"  "ess"
## [591] "egv"  "eav"  "sarj" "cale" "amt"  "gfe"  "arf"  "acac" "cpy"  "lacy"
## [601] "bpb"  "bfi"  "bhu"  "cct"  "byl"  "bpro" "dsy"  "dhd"  "ddh"  "ddl"
## [611] "dmt"  "dor"  "dai"  "dmi"  "pth"  "drm"  "dca"  "dru"  "ded"  "dec"
## [621] "drs"  "cdf"  "pdc"  "cdc"  "cdl"  "pdf"  "capr" "ova"  "rum"  "bacc"
## [631] "sth"  "ttm"  "tto"  "txy"  "tsh"  "chy"  "fro"  "afn"  "lca"  "lcs"
## [641] "lce"  "lcl"  "lpq"  "lpi"  "lpap" "lcx"  "lrh"  "lrg"  "lrl"  "lra"
## [651] "lro"  "lrc"  "lpl"  "lpj"  "lpz"  "lfe"  "lbh"  "lbn"  "lpar" "med"
## [661] "mhw"  "meg"  "sted" "abg"  "kba"  "gox"  "goy"  "gal"  "gti"  "gdj"
## [671] "nch"  "mlu"  "pbr"  "mai"  "man"  "dra"  "dge"  "dmr"  "dpt"  "dgo"
## [681] "dsw"  "dch"  "dab"  "dpu"  "dwu"  "dfc"  "dga"  "nwi"  "blag" "paru"
## [691] "oct"  "dex"  "rpm"  "sphl" "srhi" "bba"  "bbat" "bbac" "bex"  "bsed"
## [701] "dto"  "dsf"  "dpr"  "deo"  "dog"  "drt"  "dba"  "doa"  "des"  "deu"
## [711] "pca"  "ppd"  "gsu"  "gsk"  "gme"  "gur"  "glo"  "gbm"  "geo"  "gem"
## [721] "geb"  "gpi"  "gao"  "gbn"  "ade"  "acp"  "afw"  "ank"  "gsb"  "sat"
## [731] "sfu"  "tth"  "ttj"  "tts"  "ttl"  "tsc"  "thc"  "tos"  "taq"  "tbc"
## [741] "afi"  "afj"  "tau"  "crn"  "cml"  "caw"  "carn" "ocm"  "opf"  "cly"
## [751] "clh"  "salk" "psy"  "fbl"  "nio"  "tig"  "aeh"  "lhk"  "rsh"  "rsq"
## [761] "rcp"  "rhp"  "rbl"  "lmir" "ofo"  "pne"  "poh"  "arw"  "upi"  "fmy"
## [771] "slt"  "gca"  "mfa"  "cste" "nme"  "nmp"  "nmh"  "nmd"  "nmm"  "nms"
## [781] "nmq"  "nmz"  "nma"  "nmw"  "nmx"  "nmc"  "nmn"  "nmt"  "nmi"  "ngo"
## [791] "ngk"  "nla"  "nel"  "nwe"  "nsi"  "nmj"  "nei"  "nek"  "nfv"  "nsf"
## [801] "nzl"  "naq"  "nbl"  "nzo"  "nci"  "ncz"  "nani" "nbc"  "koa"  "ecor"
## [811] "salv" "nba"  "neu"  "net"  "nit"  "nii"  "nco"  "nur"  "nst"  "nmu"
## [821] "nlc"  "sphn" "smiz" "spsc" "sphz" "sphe" "spdr" "sdj"  "stha" "sdr"
## [831] "rbh"  "maga" "mhyd" "alv"  "mpur" "ttc"  "thip" "aprs" "hhc"  "ebs"
## [841] "eic"  "etr"  "etd"  "ete"  "etc"  "edw"  "edl"  "eho"  "ans"  "pfq"
```

```
##  [851] "asa"  "amed" "asr"  "acav" "aem"  "aea"  "apt"  "aper" "aasc" "lpop"
##  [861] "mmk"  "pshi" "psi"  "psx"  "psta" "prg"  "prq"  "prj"  "pvc"  "lmo"
##  [871] "lmn"  "lmy"  "lmt"  "lmoc" "lmoe" "lmob" "lmod" "lmow" "lmoq" "lmr"
##  [881] "lmom" "lmf"  "lmc"  "lmog" "lmp"  "lmol" "lmoj" "lmoz" "lmox" "lmh"
##  [891] "lmq"  "lml"  "lmg"  "lms"  "lmj"  "lmw"  "lmx"  "lmz"  "lmon" "lmos"
##  [901] "lmoo" "lmoy" "lmot" "lmoa" "lmok" "lmv"  "lin"  "lwe"  "lsg"  "liv"
##  [911] "lii"  "liw"  "lia"  "lio"  "lwi"  "lgz"  "fps"  "fpc"  "fpy"  "fpo"
##  [921] "fpq"  "fpv"  "fpw"  "fpk"  "fpsz" "fjg"  "fbr"  "fco"  "fin"  "fgl"
##  [931] "fcm"  "fat"  "fki"  "fpal" "fmg"  "falb" "fcr"  "fsn"  "fnk"  "fak"
##  [941] "tpty" "sgl"  "pmr"  "pmib" "pvl"  "pvg"  "phau" "prot" "pcol" "pcib"
##  [951] "ftu"  "ftq"  "ftf"  "ftw"  "ftr"  "ftt"  "ftg"  "ftl"  "fta"  "fts"
##  [961] "ftc"  "ftv"  "ftz"  "ftn"  "ftx"  "ftd"  "fty"  "fcf"  "fcn"  "fhi"
##  [971] "fph"  "fpt"  "fpi"  "fpm"  "fpx"  "fpz"  "fpj"  "frt"  "fna"  "fnl"
##  [981] "frf"  "fha"  "frx"  "frm"  "frc"  "fad"  "fmi"  "foo"  "hna"  "haz"
##  [991] "tgr"  "tkm"  "tni"  "tvr"  "mmob" "mca"  "metu" "mein" "mmt"  "mdn"
## [1001] "mdh"  "mko"  "metl" "hhk"  "mej"  "ntt"  "ilo"  "ili"  "ipi"  "idi"
## [1011] "idt"  "kus"  "kma"  "kko"  "kge"  "ksd"  "kpd"  "fpp"  "gap"  "orb"
## [1021] "apl"  "apj"  "apa"  "asu"  "asi"  "ass"  "aeu"  "apor" "aio"  "alig"
## [1031] "bhud" "gan"  "aaz"  "aat"  "aao"  "aan"  "aah"  "aacn" "aact" "msu"
## [1041] "mham" "mvr"  "mvi"  "mvg"  "mve"  "mgra" "ooi"  "pmu"  "pmv"  "pul"
## [1051] "pmp"  "pdag" "avt"  "paet" "rpne" "rhey" "hin"  "hpaa" "lph"  "lpo"
## [1061] "llo"  "lok"  "lsh"  "lgt"  "ljr"  "lwa"  "psal" "cac"  "cae"  "cay"
## [1071] "cbo"  "cba"  "cbh"  "cby"  "cbk"  "cbb"  "cbi"  "cbt"  "cbe"  "cbz"
## [1081] "cbei" "ckl"  "ckr"  "clj"  "ccb"  "cls"  "csr"  "cpas" "cpat" "cpae"
## [1091] "csb"  "cah"  "clt"  "cbv"  "csq"  "cace" "cck"  "cbut" "ceu"  "cfm"
## [1101] "carg" "cdrk" "csep" "cdy"  "csh"  "mct"  "mcs"  "mcat" "mbl"  "mboi"
## [1111] "mcun" "mnn"  "par"  "prw"  "psya" "pay"  "ptt"  "xbo"  "xbv"  "xpo"
## [1121] "msx"  "bsu"  "bsr"  "bsl"  "bsh"  "bsy"  "bsut" "bsul" "bsus" "bso"
## [1131] "bsn"  "bsq"  "bsx"  "bsp"  "bss"  "bst"  "bli"  "bld"  "blh"  "bay"
## [1141] "baq"  "bya"  "bamp" "baml" "bama" "bamn" "bamb" "bamt" "bamy" "bmp"
## [1151] "bao"  "baz"  "bql"  "bxh"  "bqy"  "bami" "bamc" "bamf" "bsia" "bae"
## [1161] "bvm"  "bson" "bht"  "ban"  "bar"  "bat"  "bah"  "bai"  "bax"  "bant"
## [1171] "banr" "bans" "banh" "banv" "bce"  "bca"  "bcz"  "bcr"  "bcb"  "bcu"
## [1181] "bcg"  "bcq"  "bcx"  "bal"  "bnc"  "bcf"  "bcer" "bcef" "bcy"  "btk"
```

```
## [1191] "btl"  "btb"  "btt"  "bthr" "bthi" "btc"  "btf"  "btm"  "btg"  "bti"
## [1201] "btn"  "btht" "bthu" "btw"  "bthy" "bwe"  "bww"  "bmyo" "bty"  "bmyc"
## [1211] "bby"  "bwd"  "btro" "bmob" "bpu"  "bpum" "bpus" "bco"  "bjs"  "bif"
## [1221] "bmet" "gst"  "bacw" "bacp" "bacb" "bacy" "bacl" "balm" "bsm"  "bgy"
## [1231] "bwh"  "bxi"  "bhk"  "bbev" "balt" "bacs" "bsaf" "bit"  "bacq" "bcir"
## [1241] "bfd"  "bcoh" "bda"  "bmq"  "bmd"  "bmh"  "bmeg" "bck"  "bag"  "bcoa"
## [1251] "bha"  "bcl"  "bpf"  "ble"  "bon"  "bse"  "cum"  "cub"  "cug"  "chz"
## [1261] "cgn"  "cih"  "chh"  "cio"  "chry" "cpip" "chrs" "chrz" "carh" "csha"
## [1271] "cnk"  "cjt"  "cil"  "ccau" "cben" "ccas" "clac" "ctak" "rbz"  "sdf"
## [1281] "gho"  "ppr"  "pgb"  "pds"  "vch"  "vcf"  "vcs"  "vce"  "vcq"  "vcj"
## [1291] "vci"  "vco"  "vcr"  "vcm"  "vcl"  "vcz"  "vsp"  "van"  "lag"  "vau"
## [1301] "vbr"  "vsc"  "vqi"  "vta"  "saly" "sks"  "scot" "smai" "xoo"  "xoy"
## [1311] "xfh"  "mgm"  "maes" "mfn"  "tbm"  "tbz"
```

Organisms classified within cluster 3

```
##   [1] "ahe"  "bbl"  "bpi"  "bmm"  "bcp"  "bbg"  "bbq"  "blp"  "blu"  "blck"
##  [11] "vah"  "pmic" "pbq"  "eri"  "dpn"  "ldb"  "lae"  "lje"  "lapi" "lhs"
##  [21] "lsn"  "las"  "laa"  "lat"  "lso"  "lar"  "lau"  "ama"  "amf"  "amw"
##  [31] "amp"  "acn"  "aph"  "apy"  "apd"  "apha" "aoh"  "eru"  "erw"  "erg"
##  [41] "ecn"  "ech"  "echa" "echj" "echl" "echs" "echv" "echw" "echp" "emr"
##  [51] "ehh"  "nse"  "nri"  "nhm"  "nef"  "wol"  "wri"  "wen"  "wed"  "wpi"
##  [61] "wbm"  "woo"  "wcl"  "weo"  "wpp"  "ots"  "ott"  "kci"  "kct"  "kbl"
##  [71] "kbt"  "kga"  "kon"  "kso"  "cey"  "cea"  "cend" "buc"  "bap"  "bau"
##  [81] "baw"  "bajc" "bua"  "bup"  "bak"  "buh"  "bapf" "bapg" "bapu" "bapw"
##  [91] "bas"  "bab"  "baph" "bci"  "bcib" "bcig" "hde"  "icp"  "rip"  "rig"
## [101] "bfl"  "bpn"  "bva"  "bchr" "asy"  "hed"  "ssz"  "den"  "ged"  "seny"
## [111] "ehd"  "wbr"  "wgl"  "les"  "acl"  "apal" "aaxa" "rpr"  "rpo"  "rpw"
## [121] "rpz"  "rpg"  "rps"  "rpv"  "rpq"  "rpl"  "rpn"  "rty"  "rtt"  "rtb"
## [131] "rcm"  "rcc"  "rbe"  "rbo"  "rco"  "rfe"  "rak"  "rri"  "rrj"  "rra"
## [141] "rrc"  "rrh"  "rrb"  "rrn"  "rrp"  "rrm"  "rrr"  "rms"  "rmi"  "rpk"
## [151] "raf"  "rhe"  "rja"  "rsv"  "rsw"  "rph"  "rau"  "rmo"  "rpp"  "rre"
## [161] "ram"  "rab"  "rmc"  "ras"  "ric"  "scr"  "ssyr" "stai" "sapi" "smir"
```

```
## [171] "smia" "scq"  "ssab" "satr" "seri" "stur" "shj"  "sfz"  "scou" "scla"
## [181] "sprn" "spit" "stab" "salx" "sgq"  "schi" "ctr"  "ctd"  "ctf"  "ctrd"
## [191] "ctro" "ctrt" "cta"  "cty"  "cra"  "ctrq" "ctrx" "ctrz" "ctrp" "ctlj"
## [201] "ctlx" "ctll" "ctb"  "ctrr" "ctlf" "ctli" "ctl"  "ctru" "ctrl" "ctrv"
## [211] "ctrm" "ctla" "ctlm" "ctls" "ctlz" "ctlc" "ctln" "ctlb" "ctlq" "cto"
## [221] "ctrn" "ctj"  "ctz"  "ctg"  "ctk"  "csw"  "ces"  "ctrb" "ctre" "ctrs"
## [231] "ctec" "cfs"  "cfw"  "ctfw" "ctrf" "ctch" "ctn"  "ctq"  "ctv"  "ctw"
## [241] "ctrg" "ctri" "ctra" "ctrh" "ctrj" "ctrk" "ctjt" "ctcf" "ctfs" "cthf"
## [251] "ctcj" "cthj" "ctmj" "cttj" "ctjs" "ctrc" "ctrw" "ctry" "ctct" "cmu"
## [261] "cmur" "cmn"  "cmm"  "cmg"  "cmx"  "cmz"  "cpn"  "cpa"  "cpj"  "cpt"
## [271] "clp"  "cpm"  "cpec" "cpeo" "cper" "chp"  "chb"  "chs"  "chi"  "cht"
## [281] "chc"  "chr"  "cpsc" "cpsn" "cpsb" "cpsg" "cpsm" "cpsi" "cpsv" "cpsw"
## [291] "cpst" "cpsd" "cpsa" "cav"  "cca"  "cab"  "cabo" "cfe"  "cgz"  "chla"
## [301] "mpe"  "miw"  "mfr"  "mfm"  "mfp"  "mpul"
```

Organisms classified within cluster 4

```
##   [1] "aym"  "toy"  "dtm"  "dit"  "diz"  "dpc"  "dlu"  "asd"  "gbr"  "gpo"
##  [11] "gor"  "goq"  "gta"  "goc"  "git"  "gru"  "gom"  "gav"  "god"  "nfa"
##  [21] "nfr"  "ncy"  "nbr"  "nno"  "nsl"  "nsr"  "ntp"  "noz"  "nod"  "nah"
##  [31] "nad"  "nwl"  "tpr"  "tsm"  "bsd"  "gob"  "mmar" "nml"  "kra"  "brx"
##  [41] "bgg"  "brz"  "bsau" "aus"  "ica"  "jte"  "jli"  "jme"  "agg"  "cmi"
##  [51] "cms"  "cmc"  "cmh"  "ccap" "cart" "frn"  "hea"  "hum"  "huw"  "lse"
##  [61] "ltr"  "plap" "rtn"  "rry"  "ria"  "rfs"  "rte"  "salc" "sala" "sald"
##  [71] "aai"  "gar"  "gcr"  "glu"  "cef"  "chn"  "cgy"  "cmq"  "cpre" "csur"
##  [81] "aau"  "pue"  "ach"  "apn"  "psul" "psni" "psey" "satk" "agy"  "agm"
##  [91] "agf"  "mts"  "mim"  "mio"  "mpal" "micr" "mhos" "mwa"  "mprt" "msed"
## [101] "moy"  "ver"  "fra"  "fre"  "fri"  "fal"  "fsy"  "plk"  "plab" "plat"
## [111] "stp"  "saq"  "sna"  "ase"  "ams"  "actn" "afs"  "acts" "nca"  "ndk"
## [121] "noo"  "nbe"  "nano" "nmes" "prv"  "ahm"  "acti" "acad" "ahg"  "acta"
## [131] "apre" "ami"  "led"  "aey"  "pmad" "pdx"  "psea" "psee" "pseh" "pseq"
## [141] "pecq" "phh"  "paut" "svi"  "sacc" "sen"  "sace" "sacg" "sesp" "ssyi"
## [151] "amd"  "amn"  "amm"  "amz"  "aoi"  "aja"  "amq"  "amyc" "amyb" "aab"
```

```
## [161] "amyy" "aori" "stri" "ksk"  "kab"  "kau"  "kit"  "krh"  "kpl"  "kfv"
## [171] "krs"  "nda"  "nal"  "ngv"  "strr" "noa"  "now"  "sro"  "actw" "tcu"
## [181] "cai"  "mau"  "mil"  "micb" "mtua" "mich" "mtem" "mcab" "msag" "vma"
## [191] "mcra" "euz"  "rxy"  "rub"  "cwo"  "fln"  "nko"  "fla"  "hhy"  "bbd"
## [201] "cmr"  "camu" "evi"  "est"  "echi" "rhoz" "flm"  "fll"  "mtt"  "add"
## [211] "aswu" "hsw"  "hym"  "hyd"  "hye"  "hyg"  "hyp"  "hyz"  "hnv"  "hyh"
## [221] "hyj"  "hqi"  "hrs"  "pko"  "pact" "ruf"  "rti"  "rud"  "alm"  "fbt"
## [231] "eol"  "rsi"  "run"  "rup"  "sli"  "srd"  "smon" "spir" "spik" "spib"
## [241] "dfe"  "aqb"  "aqa"  "aalg" "gfl"  "grs"  "kos"  "kan"  "fbc"  "marm"
## [251] "mart" "marb" "mare" "mut"  "ndo"  "nob"  "noj"  "pom"  "pob"  "prn"
## [261] "pola" "poa"  "phal" "ptq"  "seon" "sze"  "win"  "salt" "fbe"  "agd"
## [271] "mup"  "muc"  "mgot" "muh"  "mgk"  "mrub" "mgos" "scn"  "phe"  "pep"
## [281] "pcm"  "psty" "pek"  "sbx"  "cpi"  "chit" "rmr"  "rmg"  "sru"  "srm"
## [291] "eff"  "aac"  "aad"  "bts"  "kyr"  "bly"  "blin" "bcau" "far"  "asoc"
## [301] "mtu"  "mtv"  "mtc"  "mra"  "mtf"  "mtb"  "mtk"  "mtz"  "mtg"  "mti"
## [311] "mte"  "mtur" "mtl"  "mto"  "mtd"  "mtn"  "mtj"  "mtub" "mtuc" "mtue"
## [321] "mtx"  "mtuh" "mtul" "mtut" "mtuu" "mtq"  "mbo"  "mbb"  "mbt"  "mbm"
## [331] "mbk"  "mbx"  "maf"  "mmic" "mce"  "mcq"  "mcv"  "mcx"  "mcz"  "mle"
## [341] "mlb"  "mpa"  "mao"  "mavi" "mavu" "mav"  "mit"  "mia"  "mid"  "myo"
## [351] "mchi" "mir"  "mmal" "mlp"  "msa"  "mul"  "mmc"  "mkm"  "mjl"  "mmi"
## [361] "mmae" "mmm"  "mli"  "mkn"  "myv"  "mye"  "mhad" "mdx"  "mshg" "mfj"
## [371] "mgro" "mxe"  "mnv"  "mpag" "mnm"  "mgor" "mcoo" "mste" "lyb"  "lyg"
## [381] "pmar" "ssil" "pms"  "pmq"  "pmw"  "pnp"  "ppsc" "palb" "pbk"  "say"
## [391] "sap"  "sthr" "acr"  "amv"  "goh"  "gbe"  "gbh"  "gbc"  "gbs"  "gdi"
## [401] "mick" "rgi"  "ros"  "rmuc" "nao"  "skt"  "aex"  "ccr"  "ccs"  "cak"
## [411] "cse"  "chq"  "cmb"  "cfh"  "cauf" "pzu"  "phb"  "bsb"  "brd"  "bne"
## [421] "brg"  "brl"  "bvc"  "bdm"  "brf"  "brev" "bmed" "bvy"  "hba"  "hne"
## [431] "mmr"  "hbc"  "bop"  "bos"  "bvv"  "boi"  "bof"  "msc"  "mbry" "mros"
## [441] "mhey" "mpar" "bid"  "moc"  "miv"  "mico" "ddr"  "dpd"  "dez"  "dein"
## [451] "msl"  "mtun" "chel" "cdq"  "mtw"  "bbar" "deq"  "dei"  "dea"  "cbot"
## [461] "phl"  "hdn"  "hdt"  "hmc"  "hni"  "lne"  "rbs"  "kai"  "atu"  "ara"
## [471] "ata"  "agr"  "atf"  "avi"  "agc"  "aro"  "agt"  "alf"  "aua"  "aala"
## [481] "bapi" "mes"  "aak"  "amih" "ngl"  "ngg"  "neo"  "nen"  "niy"  "shz"
## [491] "hoe"  "pht"  "rva"  "lap"  "lagg" "labr" "labp" "labt" "pphr" "psf"
```

```
## [501] "sme"  "smk"  "smq"  "smx"  "smi"  "smeg" "smel" "smer" "smd"  "rhi"
## [511] "sfh"  "sfd"  "six"  "same" "sino" "siw"  "ead"  "eah"  "esj"  "eak"
## [521] "emx"  "nha"  "oca"  "ocg"  "oco"  "rhz"  "rpa"  "rpb"  "rpc"  "rpd"
## [531] "rpe"  "rpt"  "rpx"  "anc"  "apra" "bvr"  "boo"  "aht"  "con"  "dsh"
## [541] "rmb"  "geh"  "hml"  "jan"  "pde"  "pami" "pye"  "pcon" "pzh"  "paro"
## [551] "pamn" "pars" "parr" "pkd"  "ppan" "rha"  "rer"  "rey"  "reb"  "rop"
## [561] "roa"  "req"  "rpy"  "rhb"  "rav"  "rfa"  "rhw"  "rhs"  "rrz"  "rhu"
## [571] "rqi"  "rhq"  "rhod" "rrt"  "rby"  "rcr"  "rtm"  "palw" "pga"  "pgl"
## [581] "pgd"  "php"  "ppic" "phq"  "sagu" "pseb" "red"  "ypac" "tpro" "rmm"
## [591] "rok"  "rid"  "rom"  "roh"  "sil"  "sit"  "rua"  "rut"  "oat"  "oar"
## [601] "otm"  "tgl"  "thw"  "tec"  "rde"  "rli"  "rpon" "rsu"  "rhm"  "rhc"
## [611] "thaa" "ocd"  "maru" "magq" "mag"  "mgy"  "mgry" "magx" "magn" "dvn"
## [621] "txi"  "thac" "tii"  "bme"  "bmel" "bmi"  "bmz"  "bmg"  "bmw"  "bmee"
## [631] "bmf"  "bmb"  "bmc"  "baa"  "babo" "babr" "babt" "babb" "babu" "babs"
## [641] "babc" "bms"  "bsi"  "bsf"  "bsui" "bsup" "bsuv" "bsuc" "bmt"  "bsz"
## [651] "bsv"  "bsw"  "bsg"  "bov"  "bcs"  "bsk"  "bol"  "bcar" "bcas" "bmr"
## [661] "bpp"  "bpv"  "bcet" "bcee" "bvl"  "bru"  "brj"  "oin"  "oah"  "ops"
## [671] "bja"  "bju"  "bjp"  "bra"  "bbt"  "brs"  "aol"  "brc"  "brad" "bic"
## [681] "bro"  "brk"  "bot"  "brq"  "bgq"  "bgz"  "bsym" "bbet" "barh" "bvz"
## [691] "aay"  "aep"  "alb"  "alh"  "sjp"  "sch"  "ssy"  "syb"  "sbd"  "spmi"
## [701] "sphb" "sphr" "sinb" "spht" "shyd" "sya"  "sclo" "spyg" "sufl" "sami"
## [711] "sbar" "sal"  "sphk" "sphp" "smag" "smaz" "ster" "sgi"  "sphq" "spho"
## [721] "sphx" "blas" "bfw"  "swi"  "sphd" "sphm" "stax" "sphi" "ssan" "snj"
## [731] "smy"  "span" "skr"  "splm" "splk" "spkc" "sphc" "sphf" "spha" "spau"
## [741] "sech" "slut" "smic" "sphs" "eli"  "elq"  "erk"  "err"  "erf"  "emv"
## [751] "pns"  "porl" "phz"  "pot"  "ntd"  "nar"  "npp"  "npn"  "nre"  "nov"
## [761] "not"  "nor"  "ngf"  "nog"  "tmo"  "pla"  "rbm"  "shum" "svc"  "bbw"
## [771] "bdc"  "bdz"  "dov"  "dwd"  "dalk" "dal"  "dat"  "abai" "hoh"  "ccx"
## [781] "mfu"  "mmas" "mxa"  "msd"  "mym"  "mfb"  "cfus" "sur"  "ccro" "llu"
## [791] "mrm"  "scl"  "scu"  "samy" "vin"  "bsto" "dti"  "ret"  "rec"  "rel"
## [801] "rep"  "rei"  "rle"  "rlt"  "rlg"  "rlb"  "rlu"  "rtr"  "rir"  "rpus"
## [811] "rhl"  "rga"  "rhn"  "rpha" "rhx"  "rhv"  "rhk"  "rez"  "rjg"  "rhr"
## [821] "rgr"  "rad"  "roy"  "rii"  "sva"  "acy"  "oce"  "zdf"  "cate" "cao"
## [831] "cbal" "cbat" "gag"  "gni"  "rhh"  "salm" "lsd"  "mvs"  "mya"  "mmaa"
```

```
##  [841] "pin"  "fes"  "suam" "spse" "sulz" "suli" "suld" "spot" "cps"  "com"
##  [851] "coz"  "colw" "cola" "cber" "cov"  "nde"  "nmv"  "nja"  "blep" "beb"
##  [861] "beba" "simp" "aka"  "amim" "oan"  "och"  "rsp"  "rsk"  "aon"  "bpe"
##  [871] "bpc"  "bper" "bpet" "bpeu" "bpar" "bpa"  "bbh"  "bbr"  "bbm"  "bbx"
##  [881] "bpt"  "bav"  "bho"  "bhm"  "bhz"  "btrm" "bbro" "bfz"  "bpdz" "boh"
##  [891] "bgm"  "boj"  "bxe"  "bxb"  "bph"  "bge"  "bpx"  "bpy"  "buz"  "bfn"
##  [901] "bcai" "pspw" "para" "parb" "phs"  "pter" "pgp"  "pcj"  "pts"  "pcaf"
##  [911] "pmeg" "caba" "buo"  "cdn"  "dac"  "del"  "dts"  "dhk"  "dla"  "dpy"
##  [921] "dih"  "daer" "drg"  "dug"  "adn"  "adk"  "mms"  "jag"  "jab"  "jaz"
##  [931] "jal"  "jsv"  "jaj"  "jas"  "jlv"  "limn" "lim"  "lih"  "mnr"  "masw"
##  [941] "mass" "masz" "mtim" "masy" "mali" "mum"  "mfla" "mpli" "miu"  "hse"
##  [951] "hsz"  "hht"  "hrb"  "hee"  "hhf"  "hfr"  "nok"  "ppk"  "ppno" "ppnm"
##  [961] "prb"  "ppul" "pspu" "papi" "pve"  "pox"  "ptx"  "pfg"  "pnr"  "pand"
##  [971] "pfib" "pkt"  "mpt"  "metp" "pnu"  "pdq"  "har"  "put"  "pus"  "pud"
##  [981] "afa"  "afq"  "aaqu" "rso"  "rsc"  "rsl"  "rsn"  "rsm"  "rse"  "rsy"
##  [991] "rpi"  "rpf"  "rpj"  "rmn"  "rin"  "rpu"  "art"  "arr"  "arm"  "arl"
## [1001] "are"  "aaq"  "arh"  "ary"  "arz"  "aru"  "arq"  "arn"  "arx"  "acry"
## [1011] "arth" "artp" "acit" "rta"  "rfr"  "rsb"  "rac"  "rhy"  "rhf"  "rhg"
## [1021] "rdp"  "rge"  "rbn"  "sthm" "tin"  "thi"  "pol"  "pna"  "pos"  "poo"
## [1031] "aav"  "ajs"  "dia"  "aaa"  "ack"  "acra" "acid" "acip" "acin" "acis"
## [1041] "acio" "amon" "ctt"  "ctes" "cke"  "cser" "cof"  "hyr"  "hyb"  "hyl"
## [1051] "hyc"  "hpse" "hyn"  "lch"  "upv"  "vap"  "vpe"  "vpd"  "vaa"  "vbo"
## [1061] "vam"  "chiz" "cfon" "cari" "amah" "aqs"  "cvi"  "cvc"  "chro" "chri"
## [1071] "chrb" "crz"  "chrm" "chae" "pse"  "aql"  "htl"  "meu"  "meh"  "mei"
## [1081] "mep"  "azo"  "aoa"  "aza"  "azi"  "atw"  "acom" "azd"  "azr"  "azq"
## [1091] "dsu"  "app"  "doe"  "shg"  "sht"  "metr" "tmz"  "thu"  "tcl"  "thk"
## [1101] "tak"  "shd"  "dar"  "dey"  "reh"  "cnc"  "cuh"  "reu"  "rme"  "cti"
## [1111] "cbw"  "cgd"  "ccup" "cup"  "cuu"  "cpau" "cox"  "ttu"  "mthd" "micc"
## [1121] "mii"  "mict" "cja"  "ceb"  "cell" "cek"  "ceg"  "spoi" "osg"  "alg"
## [1131] "asip" "buf"  "bage" "cnt"  "cem"  "cen"  "clap" "sfo"  "ahn"  "aha"
## [1141] "ahy"  "ahd"  "ahr"  "ahp"  "ahj"  "ahh"  "ahi"  "aaj"  "aeo"  "avr"
## [1151] "avo"  "adh"  "arv"  "aes"  "ael"  "apw"  "apf"  "apu"  "apg"  "apq"
## [1161] "apx"  "apz"  "apk"  "asz"  "asv"  "aace" "apom" "ato"  "acet" "aot"
## [1171] "aoy"  "eco"  "ecj"  "ecd"  "ebw"  "ecok" "ece"  "ecs"  "ecf"  "etw"
```

```
## [1181] "elx"  "eoi"  "eoj"  "eoh"  "ecoo" "ecoh" "esl"  "eso"  "esm"  "eck" 
## [1191] "ecg"  "eok"  "elr"  "elh"  "ecw"  "eun"  "ecp"  "ena"  "ecos" "ecv" 
## [1201] "ecoa" "ecx"  "ecm"  "ecy"  "ecr"  "ecq"  "eum"  "ect"  "eoc"  "ebr" 
## [1211] "ebl"  "ebe"  "ebd"  "eci"  "eih"  "ecz"  "ecc"  "elo"  "eln"  "ese" 
## [1221] "ecl"  "eko"  "ekf"  "eab"  "edh"  "edj"  "elu"  "elw"  "ell"  "elc" 
## [1231] "eld"  "elp"  "elf"  "ecol" "ecoi" "ecoj" "efe"  "eal"  "ema"  "esz" 
## [1241] "sfl"  "sfx"  "sfv"  "sfe"  "sfn"  "sfs"  "sft"  "ssn"  "sbo"  "sbc" 
## [1251] "sdy"  "sdz"  "shq"  "esa"  "csk"  "csz"  "csj"  "ccon" "cdm"  "csi" 
## [1261] "cmj"  "cui"  "cmw"  "ctu"  "ksa"  "kor"  "krd"  "kco"  "kot"  "kpse"
## [1271] "gxy"  "gxl"  "kna"  "keu"  "ksc"  "kre"  "kha"  "gqu"  "hav"  "hpar"
## [1281] "opo"  "ddd"  "dda"  "dze"  "ddc"  "dzc"  "dso"  "ced"  "dfn"  "ddq" 
## [1291] "daq"  "dic"  "lax"  "lei"  "leh"  "lee"  "ler"  "lea"  "laz"  "lef" 
## [1301] "lni"  "lew"  "bgj"  "brb"  "bng"  "lbq"  "kgo"  "kie"  "kas"  "psts"
## [1311] "pge"  "pala" "phei" "sty"  "stt"  "sex"  "sent" "stm"  "seo"  "sev" 
## [1321] "sey"  "sem"  "sej"  "seb"  "sef"  "setu" "setc" "senr" "send" "seni"
## [1331] "seen" "spt"  "sek"  "spq"  "sei"  "sec"  "seh"  "shb"  "senh" "seeh"
## [1341] "see"  "senn" "sew"  "sea"  "sens" "sed"  "seg"  "sel"  "sega" "set" 
## [1351] "sena" "seno" "senv" "senq" "senl" "senj" "seec" "seeb" "seep" "senb"
## [1361] "sene" "senc" "ses"  "sbg"  "sbz"  "sbv"  "salz" "smar" "smac" "smw" 
## [1371] "spe"  "srr"  "srl"  "sry"  "sply" "srs"  "sra"  "smaf" "slq"  "serf"
## [1381] "sers" "sfw"  "sfg"  "srz"  "sera" "serq" "serm" "squ"  "sfj"  "sof" 
## [1391] "ssur" "rah"  "raq"  "raa"  "rox"  "eame" "pam"  "plf"  "paj"  "paq" 
## [1401] "pva"  "pagg" "pao"  "kln"  "pant" "panp" "pagc" "pstw" "palh" "pans"
## [1411] "pey"  "pdis" "fjo"  "ffa"  "fse"  "ebt"  "tci"  "ebf"  "ebc"  "ebu" 
## [1421] "sod"  "eam"  "eay"  "eta"  "epy"  "epr"  "ebi"  "erj"  "ege"  "epe" 
## [1431] "erwi" "ype"  "ypk"  "yph"  "ypa"  "ypn"  "ypm"  "ypp"  "ypg"  "ypz" 
## [1441] "ypt"  "ypd"  "ypx"  "ypw"  "ypj"  "ypv"  "ypl"  "yps"  "ypo"  "ypi" 
## [1451] "ypy"  "ypb"  "ypq"  "ypu"  "ypr"  "ypc"  "ypf"  "yen"  "yep"  "yey" 
## [1461] "yel"  "yew"  "yet"  "yef"  "yee"  "ysi"  "yal"  "yfr"  "yin"  "ykr" 
## [1471] "yro"  "yru"  "yrb"  "yak"  "yma"  "yhi"  "yca"  "ymo"  "eca"  "patr"
## [1481] "pato" "pct"  "pcc"  "pcv"  "pwa"  "ppar" "pec"  "pws"  "ppoa" "pbra"
## [1491] "ppuj" "cro"  "cko"  "cfd"  "cbra" "cwe"  "cyo"  "cpot" "cfq"  "cama"
## [1501] "caf"  "cif"  "cfar" "cir"  "cie"  "cpar" "ror"  "ron"  "rpln" "rao" 
## [1511] "rtg"  "ree"  "yre"  "sgoe" "kin"  "pdz"  "izh"  "pgz"  "pcd"  "mint"
```

```
## [1521] "mthi" "gbi"  "tti"  "mmai" "amc"  "amh"  "amaa" "amal" "amae" "amao"
## [1531] "amad" "amai" "amag" "amac" "amb"  "amg"  "amk"  "alt"  "aal"  "aaus"
## [1541] "asp"  "asq"  "aaw"  "alr"  "ale"  "alz"  "apel" "abo"  "adi"  "apac"
## [1551] "aln"  "axe"  "cmai" "kuy"  "csa"  "haa"  "hel"  "hcs"  "hak"  "ham"
## [1561] "hhu"  "hco"  "hsi"  "halo" "hhh"  "hbe"  "hag"  "haf"  "halk" "hvn"
## [1571] "hol"  "hsr"  "hmd"  "haxi" "htt"  "hcam" "hpiz" "mlo"  "mln"  "mci"
## [1581] "mop"  "mam"  "mamo" "meso" "mesw" "mesm" "mesp" "mhua" "mjr"  "merd"
## [1591] "llp"  "axy"  "axo"  "axn"  "axx"  "adt"  "ais"  "asw"  "achr" "achb"
## [1601] "mars" "nik"  "ncu"  "ome"  "mmw"  "mme"  "mpc"  "mpri" "mard" "bmar"
## [1611] "gsn"  "tol"  "tor"  "rfo"  "azl"  "ali"  "abs"  "abq"  "abf"  "ati"
## [1621] "ahu"  "azt"  "azm"  "azz"  "aoz"  "lpn"  "lpu"  "lpm"  "lpf"  "lpp"
## [1631] "lpc"  "lpa"  "lpe"  "lfa"  "lha"  "lcd"  "llg"  "lib"  "lcj"  "lss"
## [1641] "moi"  "mos"  "pcr"  "pso"  "pur"  "pali" "pspg" "psyg" "psyc" "psyy"
## [1651] "psyp" "plu"  "plum" "xne"  "xnm"  "xdo"  "xho"  "enc"  "enl"  "eclg"
## [1661] "ecle" "ecln" "ecli" "eclx" "ecly" "eclz" "eclo" "ehm"  "exf"  "ecla"
## [1671] "eclc" "eau"  "ekb"  "eno"  "eec"  "elg"  "ecan" "ern"  "ecls" "echg"
## [1681] "esh"  "ent"  "eas"  "enr"  "enx"  "enf"  "ebg"  "end"  "kpn"  "kpu"
## [1691] "kpm"  "kpp"  "kph"  "kpz"  "kpv"  "kpw"  "kpy"  "kpg"  "kpc"  "kpq"
## [1701] "kpt"  "kpo"  "kpr"  "kpj"  "kpi"  "kpa"  "kps"  "kpx"  "kpb"  "kpne"
## [1711] "kpnu" "kpnk" "kva"  "kpe"  "kpk"  "kvd"  "kvq"  "kox"  "koe"  "koy"
## [1721] "kom"  "kmi"  "kok"  "koc"  "kqu"  "eae"  "ear"  "kqv"  "kll"  "klw"
## [1731] "esc"  "kle"  "acb"  "abm"  "aby"  "abc"  "abn"  "abb"  "abx"  "abz"
## [1741] "abr"  "abd"  "abh"  "abad" "abj"  "abab" "abaj" "abaz" "abk"  "abau"
## [1751] "abaa" "abw"  "abal" "acc"  "ano"  "alc"  "acal" "acd"  "aci"  "att"
## [1761] "aei"  "ajo"  "acw"  "acv"  "ahl"  "ajn"  "asol" "ala"  "asj"  "aid"
## [1771] "adv"  "arj"  "awu"  "acum" "agu"  "aug"  "alw"  "ads"  "aber" "atn"
## [1781] "achi" "alj"  "maq"  "mhc"  "mad"  "mbs"  "msr"  "mpq"  "mari" "mlq"
## [1791] "msq"  "mara" "marj" "bma"  "bmv"  "bml"  "bmn"  "bmal" "bmae" "bmaq"
## [1801] "bmai" "bmaf" "bmaz" "bmab" "bps"  "bpm"  "bpl"  "bpd"  "bpr"  "bpse"
## [1811] "bpsm" "bpsu" "bpsd" "bpz"  "bpq"  "bpk"  "bpsh" "bpsa" "bpso" "but"
## [1821] "bte"  "btq"  "btj"  "btz"  "btd"  "btv"  "bthe" "bthm" "btha" "bthl"
## [1831] "bok"  "boc"  "buu"  "bvi"  "bve"  "bur"  "bcn"  "bch"  "bcm"  "bcj"
## [1841] "bcen" "bcew" "bceo" "bam"  "bac"  "bmj"  "bmu"  "bmk"  "bmul" "bct"
## [1851] "bced" "bcep" "bdl"  "bpyr" "bcon" "bub"  "bdf"  "blat" "btei" "bsem"
```

```
## [1861] "bpsl" "bmec" "bstg" "bstl" "bgl"  "bgu"  "bug"  "bgf"  "bgd"  "bgo"
## [1871] "byi"  "buk"  "bue"  "bul"  "buq"  "bgp"  "bpla" "bud"  "bum"  "bui"
## [1881] "mrd"  "met"  "mno"  "mor"  "meta" "maqu" "mphy" "mee"  "metd" "metx"
## [1891] "mets" "meti" "mmes" "mtea" "baci" "baco" "saln" "sok"  "thas" "faq"
## [1901] "hdh"  "tee"  "this" "vcx"  "vvu"  "vvy"  "vvm"  "vvl"  "vpa"  "vpb"
## [1911] "vpk"  "vpf"  "vph"  "vha"  "vca"  "vag"  "vex"  "vdb"  "vhr"  "vna"
## [1921] "vow"  "vro"  "vej"  "vfu"  "vni"  "vcy"  "vct"  "vtu"  "vfl"  "vmi"
## [1931] "vga"  "vsh"  "vaf"  "vnl"  "vcc"  "vas"  "vaq"  "vsr"  "son"  "sdn"
## [1941] "sfr"  "saz"  "sbl"  "sbm"  "sbn"  "sbp"  "sbt"  "sbs"  "sbb"  "slo"
## [1951] "spc"  "shp"  "sse"  "spl"  "she"  "shm"  "shn"  "shw"  "shl"  "swd"
## [1961] "swp"  "svo"  "shf"  "sja"  "spsw" "sbj"  "smav" "shew" "salg" "slj"
## [1971] "spol" "sbk"  "skh"  "saes" "tht"  "thap" "pha"  "ptn"  "pat"  "psm"
## [1981] "pseo" "pia"  "pphe" "pbw"  "prr"  "plz"  "paln" "ppis" "pea"  "pspo"
## [1991] "part" "ptu"  "png"  "ptd"  "psen" "pdj"  "paga" "pcar" "pmaa" "fau"
## [2001] "dji"  "dja"  "dtx"  "dye"  "lrz"  "lpy"  "rhd"  "rgl"  "dko"  "lab"
## [2011] "laq"  "lcp"  "lgu"  "lez"  "lem"  "lmb"  "lyt"  "lue"  "lyj"  "lsol"
## [2021] "psu"  "psuw" "psd"  "pmex" "avn"  "avl"  "avd"  "acx"  "pae"  "paev"
## [2031] "paei" "pau"  "pap"  "pag"  "paf"  "pnc"  "paeb" "pdk"  "psg"  "prp"
## [2041] "paep" "paer" "paem" "pael" "paes" "paeu" "paeg" "paec" "paeo" "pmy"
## [2051] "pmk"  "pre"  "ppse" "palc" "pcq"  "ppu"  "ppf"  "ppg"  "ppw"  "ppt"
## [2061] "ppb"  "ppi"  "ppx"  "ppuh" "pput" "ppun" "ppud" "pfv"  "pmon" "pmot"
## [2071] "pmos" "ppj"  "por"  "pst"  "psb"  "psyr" "psp"  "pamg" "pci"  "pavl"
## [2081] "pvd"  "pfl"  "pprc" "ppro" "pfo"  "pfs"  "pfe"  "pfc"  "pfn"  "ppz"
## [2091] "pfb"  "pman" "ptv"  "pcg"  "pvr"  "pazo" "poi"  "pfw"  "pff"  "pfx"
## [2101] "pen"  "psa"  "psz"  "psr"  "psc"  "psj"  "psh"  "pstu" "pstt" "pbm"
## [2111] "plul" "pba"  "pbc"  "ppuu" "pdr"  "psv"  "psk"  "pkc"  "pch"  "pcz"
## [2121] "pcp"  "pfz"  "plq"  "palk" "prh"  "psw"  "ppv"  "pses" "psem" "psec"
## [2131] "ppsy" "psos" "pkr"  "pfk"  "panr" "ppsl" "pset" "psil" "pym"  "psed"
## [2141] "pke"  "pall" "pum"  "poj"  "pgg"  "ppsh" "pgy"  "thes" "theh" "tcn"
## [2151] "tbv"  "lum"  "lus"  "lug"  "sml"  "smt"  "buj"  "smz"  "sacz" "stek"
## [2161] "srh"  "slm"  "sten" "stem" "stes" "xcc"  "xcb"  "xca"  "xcp"  "xcv"
## [2171] "xax"  "xac"  "xci"  "xct"  "xcj"  "xcu"  "xcn"  "xcw"  "xcr"  "xcm"
## [2181] "xcf"  "xfu"  "xao"  "xom"  "xop"  "xor"  "xoz"  "xal"  "xsa"  "xtn"
```

```
## [2191] "xfr"  "xve"  "xpe"  "xhr"  "xga"  "xph"  "xva"  "xan"  "xar"  "xhy"
## [2201] "xcz"  "xth"  "chu"
```

Organisms classified within cluster 5

```
##   [1] "asg"  "arca" "actt" "mcu"  "tpy"  "tpyo" "blo"  "blj"  "bln"  "blon"
##  [11] "blf"  "bll"  "blb"  "blm"  "blk"  "blg"  "blz"  "blx"  "bad"  "badl"
##  [21] "bado" "bla"  "blc"  "blt"  "bbb"  "bbc"  "bnm"  "blv"  "blw"  "bls"
##  [31] "bani" "banl" "bni"  "banm" "bbp"  "bbi"  "bbf"  "bbv"  "bbru" "bbre"
##  [41] "bbrv" "bbrj" "bbrc" "bbrn" "bbrs" "bbrd" "bast" "btp"  "bcor" "bka"
##  [51] "bks"  "bcat" "bpsp" "bii"  "bang" "bpsc" "bact" "bcho" "bgx"  "blem"
##  [61] "cbq"  "dva"  "djj"  "dco"  "cax"  "cut"  "rmu"  "rdn"  "raj"  "rter"
##  [71] "rama" "cig"  "pac"  "pav"  "pax"  "paz"  "paw"  "pad"  "pcn"  "pacc"
##  [81] "pach" "pacn" "cacn" "pra"  "cgrn" "pacd" "acq"  "aos"  "actp" "actc"
##  [91] "acto" "ane"  "ahw"  "actz" "air"  "asla" "avc"  "apv"  "ols"  "olo"
## [101] "caer" "cgo"  "ele"  "eyy"  "ddt"  "gpa"  "copr" "ppn"  "dys"  "pgi"
## [111] "pgn"  "pgt"  "pah"  "pcre" "pcag" "alq"  "pru"  "pmz"  "pdn"  "pit"
## [121] "pdt"  "pro"  "pfus" "peo"  "pje"  "poc"  "afd"  "ash"  "ada"  "tfo"
## [131] "toh"  "fte"  "coc"  "ccm"  "col"  "chg"  "capn" "cgh"  "clk"  "cspu"
## [141] "capf" "apib" "orh"  "ori"  "bcad" "paa"  "proc" "prs"  "cts"  "cch"
## [151] "cph"  "pvi"  "plt"  "ocb"  "got"  "gmo"  "geq"  "gsa"  "gha"  "kur"
## [161] "kzo"  "paek" "jea"  "mcl"  "mcak" "spas" "scap" "spet" "scoh" "aur"
## [171] "aun"  "asan" "acg"  "auh"  "dpm"  "adc"  "jda"  "too"  "vpi"  "vac"
## [181] "vao"  "vcp"  "ppe"  "ppen" "pce"  "pdm"  "paci" "pio"  "ooe"  "osi"
## [191] "lme"  "lmm"  "lmk"  "lcn"  "lgs"  "llf"  "lgc"  "wko"  "wce"  "wct"
## [201] "wci"  "wcb"  "wjo"  "wpa"  "wcf"  "wso"  "whe"  "wei"  "wdi"  "wvr"
## [211] "lgr"  "lgv"  "lack" "spy"  "spz"  "spym" "spya" "spm"  "spg"  "sps"
## [221] "sph"  "spi"  "spj"  "spk"  "spf"  "spa"  "spb"  "stg"  "stx"  "soz"
## [231] "stz"  "spyh" "spyo" "spn"  "spd"  "spr"  "spw"  "sjj"  "snv"  "spx"
## [241] "snt"  "snd"  "spnn" "sne"  "spv"  "snc"  "snm"  "spp"  "sni"  "spng"
## [251] "snb"  "snp"  "snx"  "snu"  "spne" "spnu" "spnm" "spno" "sag"  "san"
## [261] "sak"  "sgc"  "sags" "sagl" "sagm" "sagi" "sagr" "sagp" "sagc" "sagt"
## [271] "sage" "sagg" "sagn" "stc"  "stl"  "ste"  "stn"  "stu"  "stw"  "sthe"
```

```
## [281] "sths" "ssa"  "ssf"  "sst"  "sgo"  "sez"  "seq"  "sezo" "sequ" "seu" 
## [291] "sga"  "sgg"  "sgt"  "smb"  "sor"  "stb"  "scp"  "scf"  "stf"  "std" 
## [301] "smn"  "sif"  "sie"  "sib"  "siu"  "sang" "sanc" "sans" "scg"  "scon"
## [311] "scos" "soi"  "slu"  "sig"  "sip"  "stv"  "stra" "strn" "ssob" "srq" 
## [321] "ski"  "spei" "sgw"  "splr" "strg" "efa"  "ene"  "efc"  "efau" "efu" 
## [331] "efm"  "eft"  "ehr"  "ecas" "edu"  "eth"  "esg"  "dau"  "cmiu" "asf" 
## [341] "asm"  "aso"  "asb"  "aoe"  "mba"  "mby"  "mbw"  "mbar" "mbak" "mac" 
## [351] "mma"  "mmaz" "mmj"  "mmac" "mvc"  "mek"  "mls"  "metm" "mef"  "meq" 
## [361] "msj"  "msz"  "msw"  "mthr" "mthe" "mhor" "mfz"  "spoa" "fma"  "ped" 
## [371] "phar" "piv"  "cthm" "hmo"  "hcv"  "bprl" "cle"  "cew"  "pxv"  "obj" 
## [381] "rho"  "rix"  "rim"  "coo"  "rob"  "bhan" "blau" "blab" "dfg"  "tjr" 
## [391] "faa"  "apr"  "roc"  "psor" "eha"  "fpr"  "fpa"  "fpra" "cce"  "ral" 
## [401] "rch"  "rus"  "ruj"  "rto"  "rgn"  "hsc"  "rbp"  "lbw"  "ibu"  "swo" 
## [411] "csc"  "ate"  "cob"  "chd"  "cow"  "cki"  "ckn"  "clc"  "ccha" "toc" 
## [421] "tte"  "tpz"  "mta"  "mtho" "mthz" "erh"  "ers"  "erl"  "erd"  "eio" 
## [431] "esr"  "esu"  "eel"  "ere"  "ert"  "era"  "elm"  "emt"  "elim" "euu" 
## [441] "tur"  "tsg"  "ain"  "awo"  "pfac" "dho"  "mhg"  "mfun" "ljo"  "ljf" 
## [451] "ljh"  "ljn"  "lac"  "lad"  "laf"  "lbu"  "lde"  "ldl"  "lga"  "lhe" 
## [461] "lhl"  "lhr"  "lhv"  "lhh"  "lhd"  "lcr"  "lam"  "lai"  "lay"  "lke" 
## [471] "law"  "lgl"  "lamy" "lpw"  "lkl"  "lcb"  "lpt"  "lps"  "lpr"  "lre" 
## [481] "lrf"  "lru"  "lrt"  "lrr"  "lfr"  "lff"  "lmu"  "lbr"  "lbk"  "lzy" 
## [491] "lsl"  "lsi"  "lrm"  "laca" "lcy"  "lho"  "lol"  "lku"  "lali" "lfm" 
## [501] "lsa"  "ssg"  "sri"  "sele" "selo" "selt" "vpr"  "vat"  "vrm"  "vdn" 
## [511] "vnk"  "bhe"  "bhn"  "bhs"  "bqu"  "bqr"  "bbk"  "btr"  "btx"  "bgr" 
## [521] "bcd"  "baus" "bvn"  "banc" "bart" "bara" "barw" "barr" "baro" "barj"
## [531] "bez"  "barn" "bky"  "bals" "lcc"  "pmut" "rbt"  "ren"  "zmo"  "zmn" 
## [541] "zmm"  "zmb"  "zmi"  "zmc"  "zmr"  "zmp"  "bdq"  "dak"  "tpar" "cthi"
## [551] "top"  "tcm"  "thet" "tmai" "afr"  "afe"  "acu"  "acz"  "atx"  "carc"
## [561] "cdj"  "anb"  "awa"  "ann"  "sdo"  "bpsi" "kde"  "sutt" "sutk" "teq" 
## [571] "tea"  "teg"  "tas"  "tat"  "mbac" "mbat" "mmb"  "kki"  "eex"  "chj" 
## [581] "cbu"  "cbs"  "cbd"  "cbg"  "cbc"  "rvi"  "ben"  "bed"  "pdi"  "parc"
## [591] "hhs"  "pck"  "pes"  "fth"  "fti"  "fto"  "ftm"  "fper" "hha"  "mec" 
## [601] "adp"  "aap"  "aseg" "hso"  "hsm"  "mht"  "mhq"  "mhat" "mhx"  "mhae"
## [611] "mhao" "mhal" "mhaq" "mhay" "mann" "pmul" "psky" "apag" "hit"  "hip" 
```

```
## [621] "hiq"  "hif"  "hil"  "hiu"  "hie"  "hiz"  "hik"  "hia"  "hih"  "hiw"
## [631] "hic"  "hix"  "hpr"  "hdu"  "hay"  "hpit" "hhz"  "haeg" "cpe"  "cpf"
## [641] "cpr"  "ctc"  "ctet" "cno"  "cbl"  "cbn"  "cbf"  "cbm"  "cbj"  "clb"
## [651] "cld"  "ctyk" "ctae" "cchv" "cia"  "ccoh" "cfer" "ccel" "csci" "cso"
## [661] "ciu"  "cqf"  "pade" "xfa"  "xft"  "xfm"  "xfn"  "xff"  "xfl"  "xfs"
## [671] "xtw"  "abra" "aoc"  "ahk"  "erb"
```

Shortest Path (SP) kernel

Heatmap

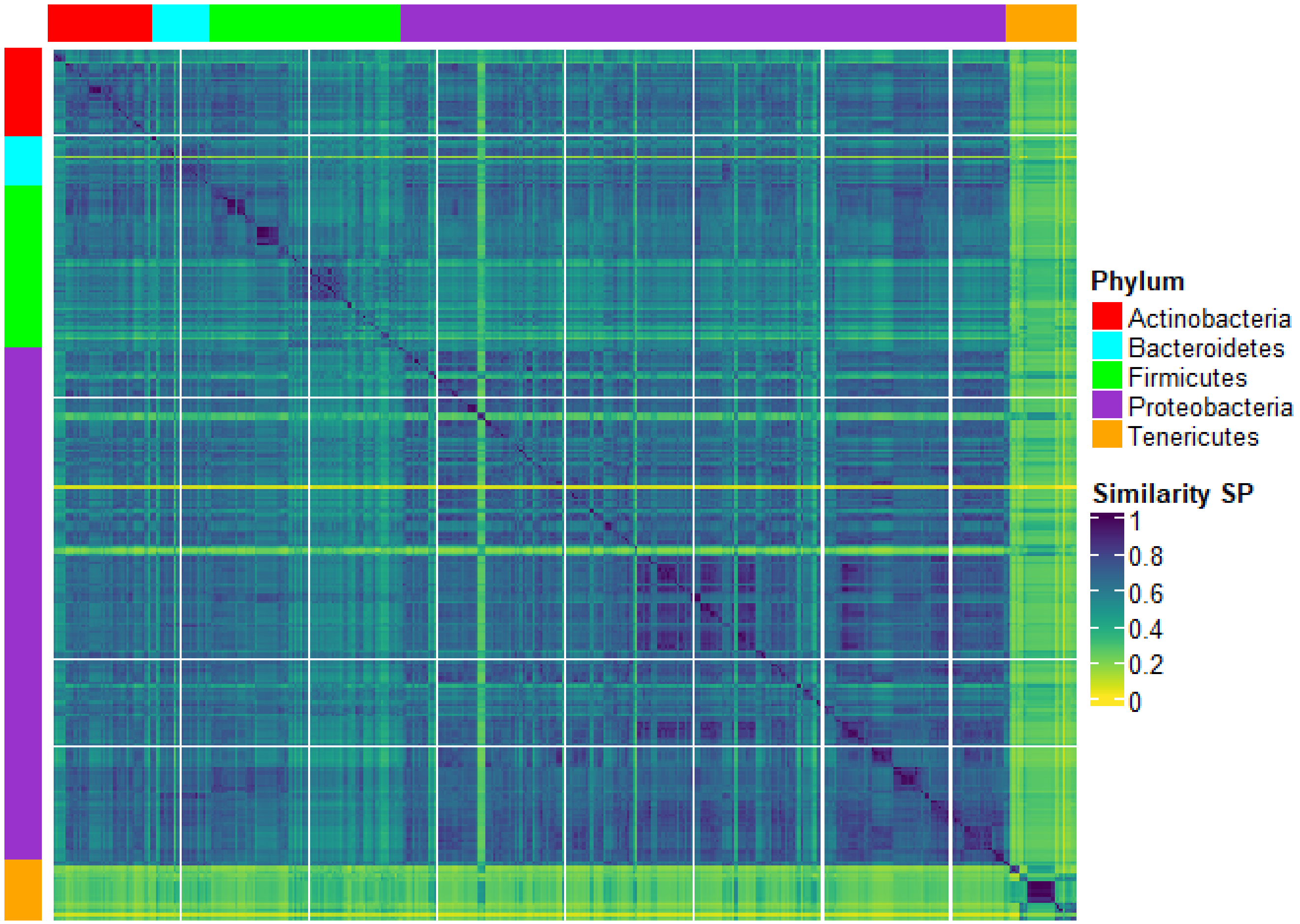
Phylum
Actinobacteria
Bacteroidetes
Firmicutes
Proteobacteria
Tenericutes
Similarity SP
1
0.8
0.6
0.4
0.2
0

MDS for SP

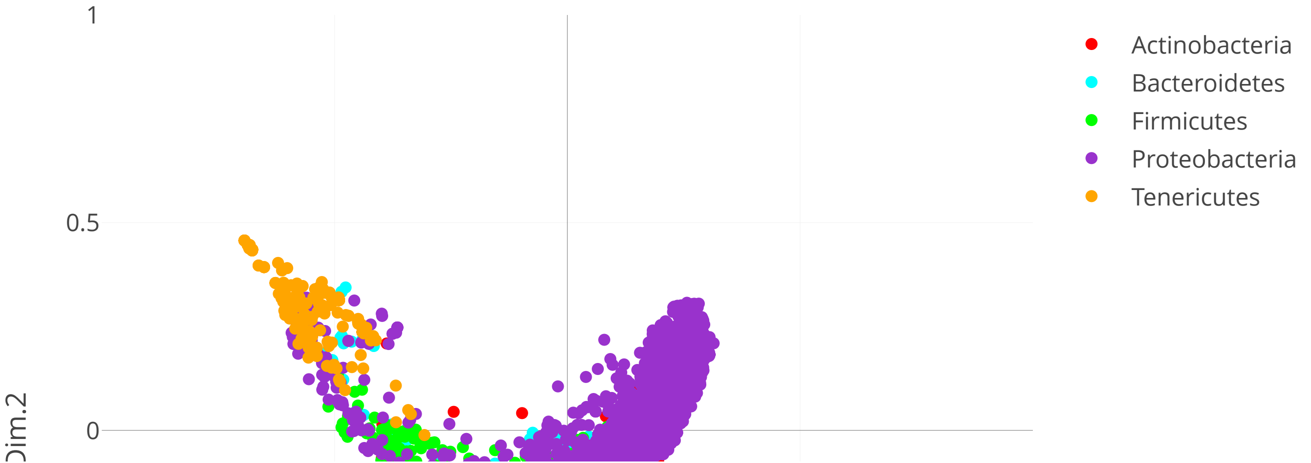
1
0.5
0
Dim.2
Actinobacteria
Bacteroidetes
Firmicutes
Proteobacteria
Tenericutes

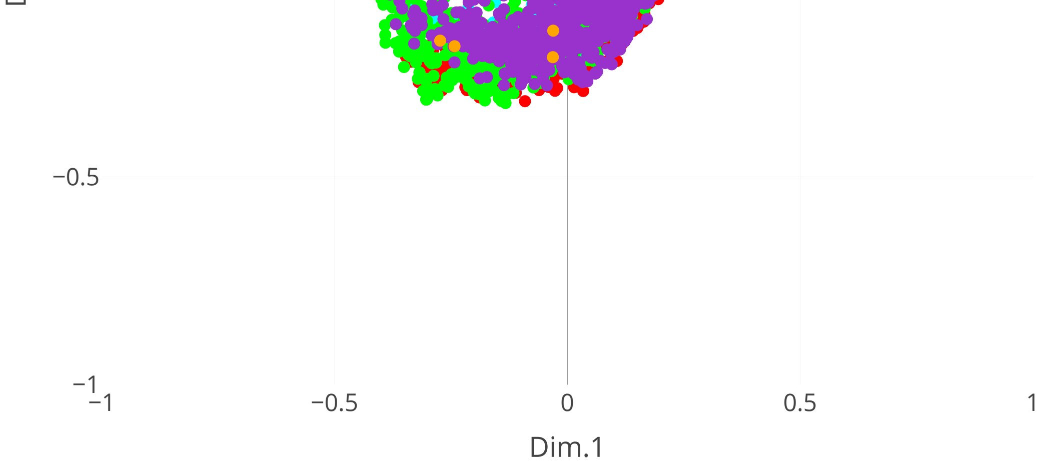

5-means clustering for SP

```
##                    Cluster
## Real group            1    2    3    4    5
##   Actinobacteria    126    5  125  138   77
##   Bacteroidetes       0   21   65  136   38
##   Firmicutes         60   10  348  140  311
##   Proteobacteria    962  107  435 1127  117
##   Tenericutes         0  315    2    0    7
```

Organisms classified within cluster 1

```
##    [1] "aym"  "toy"  "dit"  "diz"  "dpc"  "dlu"  "asd"  "gbr"  "gpo"  "gor"
##   [11] "goq"  "gta"  "gom"  "nfa"  "nfr"  "ncy"  "nbr"  "nno"  "nsl"  "nsr"
##   [21] "ntp"  "noz"  "nod"  "nah"  "nad"  "nwl"  "tpr"  "tsm"  "bsd"  "gob"
##   [31] "mmar" "nml"  "aus"  "ica"  "jte"  "jli"  "jme"  "salc" "gcr"  "glu"
##   [41] "chn"  "cgy"  "ach"  "apn"  "psul" "psni" "ver"  "fre"  "fri"  "fal"
##   [51] "plk"  "plab" "plat" "stp"  "saq"  "sna"  "ase"  "ams"  "actn" "afs"
##   [61] "acts" "nca"  "ndk"  "nbe"  "nano" "acti" "acta" "apre" "ami"  "led"
##   [71] "aey"  "pmad" "pdx"  "psea" "psee" "pseh" "pseq" "pecq" "phh"  "paut"
##   [81] "svi"  "sen"  "sace" "sacg" "sesp" "ssyi" "amd"  "amn"  "amm"  "amz"
##   [91] "aoi"  "aja"  "amq"  "amyc" "amyb" "aab"  "amyy" "aori" "stri" "ksk"
```

```
## [101] "kab"  "kau"  "kit"  "krs"  "nda"  "ngv"  "strr" "noa"  "now"  "sro" 
## [111] "actw" "tcu"  "cai"  "mau"  "mil"  "micb" "mtua" "mich" "mtem" "mcab"
## [121] "msag" "vma"  "mcra" "rxy"  "rub"  "cwo"  "eff"  "aac"  "aad"  "bts" 
## [131] "kyr"  "bcau" "mpa"  "mao"  "mavi" "mavu" "mav"  "mit"  "mia"  "mid" 
## [141] "myo"  "mchi" "mir"  "mmal" "msa"  "mul"  "mmc"  "mkm"  "mjl"  "mmi" 
## [151] "mmae" "mmm"  "mli"  "mkn"  "myv"  "mye"  "mdx"  "mshg" "mfj"  "mgro"
## [161] "mnv"  "mpag" "mnm"  "mgor" "mcoo" "mste" "bbe"  "bfm"  "lgy"  "lyb" 
## [171] "lyg"  "pmar" "siv"  "ssil" "spsy" "spor" "sure" "panc" "vig"  "vil" 
## [181] "pms"  "pmq"  "pmw"  "pnp"  "say"  "sap"  "acr"  "amv"  "rgi"  "ros" 
## [191] "rmuc" "nao"  "skt"  "ccr"  "ccs"  "cak"  "cse"  "cmb"  "cfh"  "cauf"
## [201] "bvy"  "hba"  "bop"  "bos"  "bvv"  "boi"  "bof"  "mpar" "bid"  "moc" 
## [211] "miv"  "mico" "msl"  "mtun" "chel" "cdq"  "bbar" "deq"  "dei"  "dea" 
## [221] "cbot" "phl"  "hni"  "lne"  "kai"  "atu"  "ara"  "ata"  "agr"  "atf" 
## [231] "avi"  "agc"  "aro"  "agt"  "alf"  "aua"  "aala" "bapi" "mes"  "aak" 
## [241] "amih" "ngl"  "ngg"  "neo"  "nen"  "niy"  "shz"  "hoe"  "pht"  "lap" 
## [251] "lagg" "labr" "labp" "labt" "pphr" "psf"  "sme"  "smk"  "smq"  "smx" 
## [261] "smi"  "smeg" "smel" "smer" "smd"  "rhi"  "sfh"  "sfd"  "six"  "same"
## [271] "sino" "siw"  "ead"  "eah"  "esj"  "eak"  "emx"  "rhz"  "rpa"  "rpb" 
## [281] "rpc"  "rpd"  "rpe"  "rpt"  "rpx"  "anc"  "apra" "bvr"  "boo"  "aht" 
## [291] "con"  "dsh"  "rmb"  "geh"  "hml"  "jan"  "pde"  "pami" "pye"  "pcon"
## [301] "pzh"  "paro" "pamn" "pars" "parr" "pkd"  "ppan" "rha"  "rer"  "rey" 
## [311] "reb"  "rop"  "roa"  "req"  "rpy"  "rhb"  "rav"  "rfa"  "rhw"  "rhs" 
## [321] "rrz"  "rqi"  "rhq"  "rhod" "rrt"  "rby"  "rcr"  "rtm"  "palw" "pga" 
## [331] "pgl"  "pgd"  "php"  "ppic" "phq"  "sagu" "red"  "ypac" "tpro" "rmm" 
## [341] "rok"  "rid"  "rom"  "roh"  "sil"  "sit"  "rua"  "rut"  "oat"  "oar" 
## [351] "oct"  "tgl"  "thw"  "tec"  "rde"  "rli"  "rpon" "rsu"  "rhm"  "rhc" 
## [361] "thaa" "ocd"  "maru" "magq" "txi"  "thac" "tii"  "bme"  "bmel" "bmi" 
## [371] "bmz"  "bmg"  "bmw"  "bmee" "bmf"  "bmb"  "bmc"  "baa"  "babo" "babr"
## [381] "babt" "babb" "babu" "babs" "babc" "bms"  "bsi"  "bsf"  "bsui" "bsup"
## [391] "bsuv" "bsuc" "bmt"  "bsz"  "bsv"  "bsw"  "bsg"  "bov"  "bcs"  "bsk" 
## [401] "bol"  "bcar" "bcas" "bmr"  "bpp"  "bpv"  "bcet" "bcee" "bvl"  "bru" 
## [411] "brj"  "oin"  "oah"  "ops"  "bja"  "bju"  "bjp"  "bra"  "bbt"  "brs" 
## [421] "aol"  "brc"  "brad" "bic"  "bro"  "brk"  "bot"  "brq"  "bgq"  "bgz" 
## [431] "bsym" "bbet" "barh" "bvz"  "aay"  "alb"  "alh"  "sjp"  "sch"  "ssy" 
```

```
## [441] "syb"  "sbd"  "sphb" "sphr" "sinb" "spht" "shyd" "sya"  "spyg" "sufl"
## [451] "sbar" "sal"  "sphk" "smag" "smaz" "ster" "sgi"  "sphq" "sphx" "blas"
## [461] "bfw"  "swi"  "sphd" "sphm" "stax" "sphi" "ssan" "snj"  "smy"  "span"
## [471] "skr"  "spkc" "sphc" "spau" "sech" "smic" "sphs" "erk"  "phz"  "ntd"
## [481] "nar"  "npp"  "npn"  "nre"  "nov"  "not"  "nor"  "nog"  "tmo"  "rbm"
## [491] "shum" "svc"  "bdc"  "hoh"  "mfb"  "ccro" "mrm"  "scl"  "scu"  "ret"
## [501] "rec"  "rel"  "rep"  "rei"  "rle"  "rlt"  "rlg"  "rlb"  "rlu"  "rtr"
## [511] "rir"  "rpus" "rhl"  "rga"  "rhn"  "rpha" "rhx"  "rhv"  "rhk"  "rez"
## [521] "rjg"  "rhr"  "rgr"  "rad"  "roy"  "rii"  "zdf"  "suam" "spse" "sulz"
## [531] "suli" "suld" "spot" "cps"  "beba" "simp" "aka"  "amim" "oan"  "och"
## [541] "rsk"  "aon"  "bpe"  "bpc"  "bper" "bpet" "bpeu" "bpar" "bpa"  "bbh"
## [551] "bbr"  "bbm"  "bbx"  "bpt"  "bho"  "bhm"  "bhz"  "btrm" "bbro" "bfz"
## [561] "bpdz" "boh"  "bgm"  "boj"  "bxe"  "bxb"  "bph"  "bge"  "bpx"  "bpy"
## [571] "buz"  "bfn"  "bcai" "pspw" "para" "parb" "phs"  "pter" "pgp"  "pcj"
## [581] "pts"  "pcaf" "pmeg" "caba" "buo"  "cdn"  "dac"  "del"  "dts"  "dhk"
## [591] "dla"  "dpy"  "dih"  "daer" "drg"  "adn"  "adk"  "mms"  "jag"  "jaz"
## [601] "jal"  "jsv"  "jas"  "jlv"  "limn" "lim"  "lih"  "mnr"  "masw" "mass"
## [611] "masz" "masy" "mali" "mum"  "mfla" "miu"  "hse"  "hsz"  "hht"  "hrb"
## [621] "hee"  "hhf"  "hfr"  "ppk"  "ppno" "ppnm" "prb"  "ppul" "pspu" "papi"
## [631] "pve"  "pox"  "ptx"  "pfg"  "pnr"  "pand" "pfib" "pkt"  "mpt"  "metp"
## [641] "put"  "pus"  "pud"  "afa"  "afq"  "aaqu" "rso"  "rsl"  "rsn"  "rsm"
## [651] "rse"  "rsy"  "rpi"  "rpf"  "rpj"  "rmn"  "rin"  "rpu"  "aaq"  "arh"
## [661] "arz"  "arq"  "acry" "artp" "rta"  "rfr"  "rsb"  "rhy"  "rhf"  "rhg"
## [671] "rdp"  "sthm" "pol"  "pna"  "pos"  "poo"  "aav"  "ajs"  "dia"  "aaa"
## [681] "ack"  "acra" "acid" "acip" "acin" "acis" "acio" "amon" "ctt"  "ctes"
## [691] "cke"  "cser" "cof"  "hyr"  "hyb"  "hyl"  "hyc"  "hpse" "hyn"  "lch"
## [701] "upi"  "vap"  "vpe"  "vpd"  "vaa"  "vbo"  "vam"  "aqs"  "cvi"  "cvc"
## [711] "chro" "crz"  "chae" "pse"  "azo"  "aoa"  "aza"  "azi"  "acom" "azd"
## [721] "azr"  "azq"  "tmz"  "thu"  "thk"  "dar"  "reh"  "cnc"  "cuh"  "reu"
## [731] "rme"  "cti"  "cbw"  "cgd"  "ccup" "cup"  "cuu"  "cpau" "cox"  "spoi"
## [741] "gqu"  "kas"  "pge"  "smac" "spe"  "srr"  "srl"  "sry"  "sply" "srs"
## [751] "sra"  "smaf" "slq"  "serf" "sfw"  "sfg"  "sera" "serq" "serm" "squ"
## [761] "ssur" "paj"  "pva"  "pao"  "pagc" "pans" "ebt"  "ebf"  "ebu"  "ebi"
## [771] "eca"  "patr" "pato" "pcc"  "ppoa" "cyo"  "cpot" "cif"  "ror"  "ron"
```

```
##  [781] "rao"  "rtg"  "ree"  "kin"  "izh"  "alr"  "csa"  "hel"  "hcs"  "hak" 
##  [791] "ham"  "halo" "hhh"  "hbe"  "halk" "hvn"  "hol"  "hsr"  "htt"  "hcam"
##  [801] "hpiz" "mlo"  "mln"  "mci"  "mop"  "mam"  "mamo" "meso" "mesw" "mesm"
##  [811] "mesp" "mhua" "mjr"  "merd" "kko"  "ksd"  "axy"  "axo"  "axn"  "axx" 
##  [821] "adt"  "ais"  "asw"  "achr" "achb" "mars" "mmw"  "mme"  "mpc"  "mpri"
##  [831] "mard" "rfo"  "azl"  "ali"  "abs"  "abq"  "abf"  "ati"  "azt"  "azm" 
##  [841] "azz"  "aoz"  "plu"  "plum" "kpn"  "kpu"  "kpm"  "kpp"  "kph"  "kpz" 
##  [851] "kpv"  "kpy"  "kpg"  "kpc"  "kpt"  "kpo"  "kpr"  "kpj"  "kpi"  "kpa" 
##  [861] "kps"  "kpx"  "kpb"  "kpne" "kpnu" "kpnk" "kva"  "kpe"  "kpk"  "kvd" 
##  [871] "kvq"  "kox"  "koe"  "koy"  "kom"  "kmi"  "kok"  "koc"  "kqu"  "kqv" 
##  [881] "kll"  "klw"  "aby"  "abab" "abk"  "abau" "acc"  "alc"  "acal" "acd" 
##  [891] "aci"  "ajo"  "acw"  "asol" "adv"  "arj"  "agu"  "ads"  "aber" "atn" 
##  [901] "msr"  "mpq"  "mlq"  "msq"  "marj" "bma"  "bmv"  "bml"  "bmn"  "bmal"
##  [911] "bmae" "bmaq" "bmai" "bmaf" "bmaz" "bmab" "bps"  "bpm"  "bpl"  "bpd" 
##  [921] "bpr"  "bpse" "bpsm" "bpsu" "bpsd" "bpz"  "bpq"  "bpk"  "bpsh" "bpsa"
##  [931] "bpso" "but"  "bte"  "btq"  "btj"  "btz"  "btd"  "btv"  "bthe" "bthm"
##  [941] "btha" "bthl" "bok"  "boc"  "buu"  "bvi"  "bve"  "bur"  "bcn"  "bch" 
##  [951] "bcm"  "bcj"  "bcen" "bcew" "bceo" "bam"  "bac"  "bmj"  "bmu"  "bmk" 
##  [961] "bmul" "bct"  "bced" "bcep" "bdl"  "bpyr" "bcon" "bub"  "bdf"  "blat"
##  [971] "btei" "bsem" "bpsl" "bmec" "bstg" "bstl" "bgl"  "bgu"  "bug"  "bgf" 
##  [981] "bgd"  "bgo"  "byi"  "buk"  "bue"  "bul"  "buq"  "bgp"  "bpla" "bud" 
##  [991] "bum"  "bui"  "mrd"  "met"  "mno"  "mor"  "meta" "maqu" "mphy" "mee" 
## [1001] "metd" "metx" "mets" "meti" "mmes" "mtea" "baco" "bacq" "bfd"  "bmq" 
## [1011] "bmd"  "bmh"  "bmeg" "saln" "sok"  "thas" "hdh"  "vna"  "vej"  "ptn" 
## [1021] "rgl"  "psd"  "avn"  "avl"  "avd"  "acx"  "pae"  "paev" "paei" "pau" 
## [1031] "pap"  "pag"  "paf"  "pnc"  "paeb" "pdk"  "psg"  "prp"  "paep" "paer"
## [1041] "paem" "pael" "paes" "paeu" "paeg" "paec" "paeo" "pre"  "ppse" "pcq" 
## [1051] "ppu"  "ppf"  "ppg"  "ppw"  "ppt"  "ppb"  "ppi"  "ppx"  "ppuh" "pput"
## [1061] "ppun" "ppud" "pfv"  "pmon" "pmot" "pmos" "ppj"  "por"  "pst"  "psb" 
## [1071] "psyr" "psp"  "pamg" "pavl" "pvd"  "pfl"  "pprc" "ppro" "pfo"  "pfs" 
## [1081] "pfe"  "pfc"  "pfn"  "ppz"  "pfb"  "pman" "ptv"  "pcg"  "pvr"  "pazo"
## [1091] "pfw"  "pff"  "pfx"  "pen"  "psc"  "pstu" "pstt" "pbm"  "plul" "pba" 
## [1101] "pbc"  "ppuu" "pdr"  "psv"  "psk"  "pkc"  "pch"  "pcz"  "pcp"  "pfz" 
## [1111] "plq"  "palk" "pses" "psem" "ppsy" "psos" "pkr"  "pfk"  "panr" "ppsl"
```

```
## [1121] "pset" "psil" "pym"  "pke"  "pall" "pum"  "poj"  "pgg"  "ppsh" "pgy" 
## [1131] "xac"  "xci"  "xct"  "xcj"  "xcu"  "xcn"  "xcw"  "xcr"  "xcm"  "xcf" 
## [1141] "xao"  "xal"  "xsa"  "xpe"  "xph"  "xva"  "xan"  "xar"
```

Organisms classified within cluster 2

```
##   [1] "bact" "cig"  "nfe"  "apv"  "gpa"  "che"  "cec"  "cher" "bbl"  "bpi" 
##  [11] "bmm"  "bcp"  "bbg"  "bbq"  "blp"  "blu"  "blck" "smg"  "sms"  "smh" 
##  [21] "sum"  "smv"  "smub" "smum" "smue" "smup" "ocb"  "pbq"  "lbw"  "dpn" 
##  [31] "ldb"  "lbu"  "lje"  "lapi" "lhs"  "lsn"  "hci"  "hct"  "hcc"  "hcd" 
##  [41] "lar"  "ama"  "amf"  "amw"  "amp"  "acn"  "aph"  "apy"  "apd"  "apha"
##  [51] "aoh"  "eru"  "erw"  "erg"  "ecn"  "ech"  "echa" "echj" "echl" "echs"
##  [61] "echv" "echw" "echp" "emr"  "ehh"  "nse"  "nri"  "nhm"  "nef"  "wol" 
##  [71] "wri"  "wen"  "wed"  "wpi"  "wbm"  "woo"  "wcl"  "weo"  "wpp"  "ots" 
##  [81] "ott"  "kci"  "kct"  "kbl"  "kbt"  "kga"  "kon"  "ndl"  "tpn"  "tpq" 
##  [91] "tpj"  "vfg"  "zin"  "cea"  "cend" "buc"  "bap"  "bau"  "baw"  "bajc"
## [101] "bua"  "bup"  "bak"  "buh"  "bapf" "bapg" "bapu" "bapw" "bas"  "bab" 
## [111] "bcc"  "baj"  "baph" "bci"  "bcig" "men"  "meo"  "ppet" "rip"  "rig" 
## [121] "aen"  "hed"  "cmik" "den"  "ged"  "crp"  "cru"  "crc"  "crt"  "crh" 
## [131] "crv"  "cri"  "ple"  "ply"  "plr"  "plo"  "pld"  "plb"  "plc"  "pli" 
## [141] "paly" "les"  "baab" "aaxa" "rpr"  "rpo"  "rpw"  "rpz"  "rpg"  "rps" 
## [151] "rpv"  "rpq"  "rpl"  "rpn"  "rty"  "rtt"  "rtb"  "rcm"  "rcc"  "rbe" 
## [161] "rbo"  "rco"  "rfe"  "rak"  "rri"  "rrj"  "rra"  "rrc"  "rrh"  "rrb" 
## [171] "rrn"  "rrp"  "rrm"  "rrr"  "rms"  "rmi"  "rpk"  "raf"  "rhe"  "rja" 
## [181] "rsv"  "rsw"  "rph"  "rau"  "rmo"  "rpp"  "rre"  "ram"  "rab"  "rmc" 
## [191] "ras"  "ric"  "elj"  "esx"  "efr"  "eml"  "mfl"  "mfw"  "mchc" "mlac"
## [201] "ment" "msyr" "mtab" "mcol" "scr"  "ssyr" "sdi"  "stai" "sapi" "smir"
## [211] "smia" "scq"  "ssab" "satr" "seri" "stur" "sll"  "skn"  "scj"  "shj" 
## [221] "sck"  "sfz"  "scou" "scla" "sprn" "spit" "stab" "sphh" "smoo" "salx"
## [231] "sgq"  "schi" "pml"  "pal"  "psol" "pzi"  "ctr"  "ctd"  "ctf"  "ctrd"
## [241] "ctro" "ctrt" "cta"  "cty"  "cra"  "ctrq" "ctrx" "ctrz" "ctrp" "ctlj"
## [251] "ctlx" "ctll" "ctb"  "ctrr" "ctlf" "ctli" "ctl"  "ctru" "ctrl" "ctrv"
## [261] "ctrm" "ctla" "ctlm" "ctls" "ctlz" "ctlc" "ctln" "ctlb" "ctlq" "cto"
```

```
## [271] "ctrn" "ctj"  "ctz"  "ctg"  "ctk"  "csw"  "ces"  "ctrb" "ctre" "ctrs"
## [281] "ctec" "cfs"  "cfw"  "ctfw" "ctrf" "ctch" "ctn"  "ctq"  "ctv"  "ctw"
## [291] "ctrg" "ctri" "ctra" "ctrh" "ctrj" "ctrk" "ctjt" "ctcf" "ctfs" "cthf"
## [301] "ctcj" "cthj" "ctmj" "cttj" "ctjs" "ctrc" "ctrw" "ctry" "ctct" "cmu"
## [311] "cmur" "cmn"  "cmm"  "cmg"  "cmx"  "cmz"  "cpn"  "cpa"  "cpj"  "cpt"
## [321] "clp"  "cpm"  "cpec" "cpeo" "cper" "chp"  "chb"  "chs"  "chi"  "cht"
## [331] "chc"  "chr"  "cpsc" "cpsn" "cpsb" "cpsg" "cpsm" "cpsi" "cpsv" "cpsw"
## [341] "cpst" "cpsd" "cpsa" "cav"  "cca"  "cab"  "cabo" "cfe"  "cgz"  "chla"
## [351] "mge"  "mgu"  "mgc"  "mgq"  "mgx"  "mpn"  "mpm"  "mpj"  "mpb"  "mpe"
## [361] "mga"  "mgh"  "mgf"  "mgn"  "mgs"  "mgt"  "mgv"  "mgw"  "mgac" "mgan"
## [371] "mgnc" "mgz"  "mmy"  "mmym" "mmyi" "mml"  "mcp"  "mcac" "mcap" "mcar"
## [381] "mcai" "mlc"  "mlh"  "mmo"  "mhy"  "mhj"  "mhp"  "mhn"  "mhyl" "mhyo"
## [391] "mat"  "mco"  "mho"  "mhom" "mcd"  "mhr"  "mhh"  "mhm"  "mhs"  "mhv"
## [401] "mha"  "mhf"  "mss"  "msk"  "mpf"  "mput" "mhe"  "mwe"  "mhl"  "mhb"
## [411] "mpv"  "mov"  "mbc"  "mgj"  "mfq"  "mcan" "myt"  "mds"  "myg"  "mpho"
## [421] "mhyv" "mclo" "mamp" "mans" "mphc" "miw"  "mane" "mnh"  "mnu"  "mstr"
## [431] "mgly" "mcou" "mcom" "mpu"  "msy"  "mso"  "maa"  "mal"  "mfr"  "mfm"
## [441] "mfp"  "mbv"  "mbh"  "mbi"  "mbq"  "mcy"  "mcas" "mck"  "marg" "mpul"
## [451] "mbov" "mboh" "mani" "mphi" "uur"  "upa"  "upr"  "uue"
```

Organisms classified within cluster 3

```
##   [1] "afo"  "actt" "tbw"  "bde"  "bdn"  "cbq"  "brv"  "bgg"  "dco"  "aqg"
##  [11] "cmi"  "cms"  "cmc"  "ccap" "frn"  "lxx"  "lxy"  "rtx"  "rtc"  "cef"
##  [21] "cdi"  "cdp"  "cdh"  "cdt"  "cde"  "cdr"  "cda"  "cdz"  "cdb"  "cds"
##  [31] "cdd"  "cdw"  "cdv"  "cdip" "cur"  "cua"  "car"  "ckp"  "cpl"  "cpg"
##  [41] "cpp"  "cpk"  "cpq"  "cpx"  "cpz"  "cor"  "cop"  "cod"  "cos"  "coi"
##  [51] "coe"  "cou"  "cpse" "cpsu" "cpsf" "cul"  "cuc"  "cue"  "cun"  "cus"
##  [61] "cuq"  "cuz"  "cuj"  "ccn"  "cter" "cmd"  "caz"  "cax"  "cii"  "cuv"
##  [71] "coa"  "cdo"  "csx"  "cku"  "cut"  "cfk"  "cgv"  "caqu" "csph" "cpeg"
##  [81] "csan" "cgk"  "crf"  "crl"  "ccho" "cpso" "rter" "rama" "rkr"  "mix"
##  [91] "cga"  "sanw" "fra"  "noi"  "ndp"  "pac"  "pak"  "pav"  "pax"  "paz"
## [101] "paw"  "pcn"  "pacc" "pach" "pacn" "pra"  "prv"  "prl"  "pacd" "tes"
```

```
## [111] "tdf"  "kpl"  "acq"  "aos"  "ard"  "actp" "actc" "acto" "ane"  "ahw"
## [121] "air"  "asla" "avc"  "olo"  "rrd"  "copr" "pmuc" "pet"  "buy"  "osp"
## [131] "ppn"  "dys"  "pru"  "pdt"  "afd"  "ald"  "ait"  "blq"  "sgn"  "hyd"
## [141] "hye"  "hyh"  "hqi"  "rud"  "fte"  "flu"  "aue"  "ccyn" "caph" "csto"
## [151] "capq" "mgel" "ndo"  "nom"  "nsd"  "nob"  "noj"  "for"  "foh"  "tdi"
## [161] "asl"  "aev"  "wij"  "apib" "cnr"  "emg"  "elt"  "wvi"  "este" "ran"
## [171] "rai"  "rar"  "rag"  "rae"  "rat"  "fba"  "oho"  "psn"  "pej"  "cpc"
## [181] "clz"  "pros" "cts"  "cph"  "cpb"  "cli"  "pvi"  "plt"  "ial"  "rbar"
## [191] "axl"  "afl"  "agn"  "bfv"  "ocn"  "sje"  "tap"  "gel"  "bths" "esi"
## [201] "eat"  "ean"  "exm"  "exu"  "mti"  "mtuc" "mtuh" "mle"  "mlb"  "saca"
## [211] "tco"  "kur"  "kzo"  "lsp"  "lfu"  "lys"  "ppla" "plx"  "pdec" "psyh"
## [221] "rst"  "spop" "spos" "paek" "stea" "mcak" "macr" "shv"  "sau"  "sav"
## [231] "saw"  "sah"  "saj"  "sam"  "sas"  "sar"  "sac"  "sax"  "saa"  "sao"
## [241] "sae"  "sad"  "suu"  "suv"  "sue"  "suj"  "suk"  "suc"  "sut"  "suq"
## [251] "suz"  "sud"  "sux"  "suw"  "sug"  "suf"  "saua" "saue" "saun" "saus"
## [261] "sauu" "saug" "sauz" "saut" "sauj" "sauk" "sauq" "sauv" "sauw" "saux"
## [271] "sauy" "sauf" "sab"  "suy"  "saub" "saum" "sauc" "saur" "saui" "saud"
## [281] "sams" "suh"  "ser"  "sep"  "sepp" "seps" "sha"  "shh"  "ssp"  "sca"
## [291] "slg"  "sln"  "ssd"  "sdt"  "sdp"  "swa"  "spas" "sxy"  "sxl"  "sxo"
## [301] "shu"  "scap" "ssch" "sscz" "sagq" "seqo" "ssif" "scv"  "spet" "slz"
## [311] "scoh" "snl"  "skl"  "sfq"  "shom" "smus" "scar" "schr" "sarl" "spic"
## [321] "ssh"  "ssim" "keb"  "lfb"  "ppy"  "ppm"  "ppo"  "ppol" "ppoy" "pta"
## [331] "plv"  "psab" "pdu"  "paea" "paee" "pih"  "pow"  "pbv"  "pxl"  "pyg"
## [341] "pswu" "pdh"  "pcx"  "paih" "plen" "plut" "pprt" "pbac" "aui"  "avs"
## [351] "jep"  "jeh"  "jar"  "vpi"  "ooe"  "oen"  "osi"  "lci"  "lge"  "lsu"
## [361] "lla"  "llk"  "llt"  "lls"  "lld"  "llx"  "llj"  "llm"  "llc"  "llr"
## [371] "lln"  "lli"  "llw"  "lgr"  "lgv"  "lpk"  "lact" "sag"  "sak"  "sgc"
## [381] "sags" "sagl" "sagm" "sagi" "sagr" "sagp" "sagc" "sagt" "sage" "sagg"
## [391] "sagn" "smu"  "ssa"  "ssb"  "ssu"  "ssv"  "ssi"  "sss"  "ssw"  "sup"
## [401] "ssus" "sst"  "ssuy" "ssk"  "ssq"  "sui"  "suo"  "ssut" "ssui" "sgo"
## [411] "sub"  "sga"  "sgg"  "sgt"  "sor"  "stk"  "stb"  "smn"  "sif"  "sang"
## [421] "sans" "soi"  "stv"  "spat" "strn" "ssob" "srq"  "spei" "srat" "strg"
## [431] "efl"  "efd"  "ehr"  "ecas" "emu"  "ega"  "ess"  "eth"  "egv"  "eav"
## [441] "cmiu" "sarj" "cale" "amt"  "cthm" "hmo"  "hcv"  "bprl" "arf"  "acac"
```

```
## [451] "cle"  "cew"  "cpy"  "lacy" "bpb"  "bfi"  "bhu"  "pxv"  "rho"  "cct"
## [461] "rob"  "byl"  "bpro" "dsy"  "ddh"  "ddl"  "dmt"  "dai"  "dmi"  "pth"
## [471] "drm"  "dca"  "cdf"  "pdc"  "cdc"  "cdl"  "pdf"  "capr" "fpa"  "ova"
## [481] "ral"  "rto"  "ibu"  "bacc" "swo"  "tte"  "ttm"  "tto"  "txy"  "tsh"
## [491] "chy"  "eel"  "ere"  "ert"  "era"  "elm"  "emt"  "elim" "afn"  "pfac"
## [501] "mhg"  "mfun" "lca"  "lcs"  "lce"  "lcl"  "lpq"  "lpi"  "lpap" "lcb"
## [511] "lcx"  "lrh"  "lrg"  "lrl"  "lra"  "lro"  "lrc"  "lpl"  "lpj"  "lpt"
## [521] "lps"  "lpr"  "lpz"  "lfe"  "lff"  "lbh"  "lbn"  "lpar" "lfm"  "med"
## [531] "mhw"  "meg"  "ssg"  "sri"  "vpr"  "vrm"  "vdn"  "vnk"  "abg"  "kba"
## [541] "gox"  "goh"  "goy"  "gal"  "gti"  "nch"  "mlu"  "pbr"  "mai"  "man"
## [551] "dra"  "dpt"  "dch"  "dab"  "dpu"  "dez"  "dwu"  "dvn"  "spha" "srhi"
## [561] "zmo"  "zmn"  "zmm"  "zmb"  "zmi"  "zmc"  "zmr"  "zmp"  "bba"  "bbat"
## [571] "bbw"  "bbac" "bex"  "bdq"  "bsed" "dal"  "dto"  "dsf"  "dpr"  "dog"
## [581] "drt"  "dba"  "doa"  "pca"  "ppd"  "abai" "vin"  "gsu"  "gsk"  "gme"
## [591] "gur"  "glo"  "gbm"  "geo"  "gem"  "geb"  "gpi"  "gbn"  "gsb"  "sat"
## [601] "tth"  "tsc"  "thc"  "tos"  "taq"  "tbc"  "afr"  "afe"  "acu"  "acz"
## [611] "afi"  "afj"  "atx"  "tau"  "crn"  "cml"  "cdj"  "carn" "anb"  "acy"
## [621] "ann"  "psy"  "sdo"  "nde"  "nmv"  "nio"  "nja"  "tig"  "ofo"  "pne"
## [631] "pdq"  "poh"  "fmy"  "slt"  "gca"  "mbac" "mbat" "meu"  "mmb"  "meh"
## [641] "mfa"  "mei"  "mep"  "cste" "nme"  "nmp"  "nmh"  "nmd"  "nmm"  "nms"
## [651] "nmq"  "nmz"  "nma"  "nmw"  "nmx"  "nmc"  "nmn"  "nmt"  "nmi"  "ngo"
## [661] "ngk"  "nla"  "nel"  "nsi"  "nmj"  "nei"  "nek"  "nfv"  "nsf"  "nzl"
## [671] "naq"  "nbl"  "nzo"  "nci"  "ncz"  "nani" "nbc"  "ecor" "salv" "nit"
## [681] "nur"  "nmu"  "nlc"  "sdr"  "chj"  "cja"  "ttc"  "cbu"  "cbs"  "cbd"
## [691] "cbg"  "cbc"  "alg"  "asip" "hhc"  "ans"  "apt"  "apw"  "apf"  "apu"
## [701] "apg"  "apq"  "apx"  "apz"  "apk"  "asz"  "asv"  "aace" "aper" "apom"
## [711] "ato"  "aasc" "acet" "aot"  "aoy"  "gxy"  "gxl"  "kna"  "keu"  "ksc"
## [721] "kre"  "kha"  "pdi"  "parc" "lbq"  "lmoe" "lmob" "lmf"  "lmc"  "lmog"
## [731] "lmp"  "lmol" "lmoj" "lmoz" "lmox" "lmh"  "lmq"  "lml"  "lmon" "lmoo"
## [741] "lmoa" "lmok" "lmv"  "lin"  "lwe"  "liv"  "lii"  "liw"  "lia"  "lio"
## [751] "lwi"  "lgz"  "fps"  "fpc"  "fpy"  "fpo"  "fpq"  "fpv"  "fpw"  "fpk"
## [761] "fpsz" "fco"  "fin"  "fat"  "fki"  "fpal" "fmg"  "falb" "fse"  "fak"
## [771] "sgl"  "ftq"  "ftw"  "ftr"  "ftl"  "fta"  "ftc"  "ftv"  "ftz"  "ftm"
## [781] "ftn"  "ftx"  "ftd"  "fty"  "fcf"  "fcn"  "fhi"  "fph"  "fpt"  "fpi"
```

```
## [791] "fpm"  "fpx"  "fpz"  "fpj"  "frt"  "fna"  "fnl"  "fha"  "frx"  "frm" 
## [801] "frc"  "fad"  "fmi"  "foo"  "hna"  "haz"  "tkm"  "tni"  "tti"  "tvr" 
## [811] "mca"  "metu" "mein" "mmt"  "mdn"  "mdh"  "mko"  "metl" "hha"  "hhk" 
## [821] "mej"  "mec"  "ntt"  "kpd"  "fpp"  "gap"  "orb"  "apl"  "apj"  "apa" 
## [831] "asu"  "asi"  "ass"  "aeu"  "apor" "aio"  "alig" "bhud" "gan"  "aaz" 
## [841] "aat"  "aao"  "aan"  "aah"  "aacn" "aact" "hso"  "hsm"  "msu"  "mht" 
## [851] "mhq"  "mhat" "mhx"  "mhae" "mham" "mhao" "mhal" "mhaq" "mhay" "mvr" 
## [861] "mvi"  "mvg"  "mve"  "mann" "mgra" "ooi"  "pmu"  "pmv"  "pul"  "pmp" 
## [871] "pmul" "pdag" "apag" "avt"  "paet" "rpne" "rhey" "hin"  "hit"  "hiu" 
## [881] "hiz"  "hik"  "hih"  "hiw"  "hix"  "hpit" "lgt"  "psal" "cac"  "cae" 
## [891] "cay"  "cpe"  "cpf"  "cpr"  "cno"  "cbo"  "cba"  "cbh"  "cby"  "cbl" 
## [901] "cbk"  "cbb"  "cbi"  "cbn"  "cbt"  "cbf"  "cbm"  "cbj"  "cbe"  "cbz" 
## [911] "cbei" "ckl"  "ckr"  "clj"  "ccb"  "cls"  "clb"  "cpas" "cpat" "cpae"
## [921] "csb"  "cah"  "clt"  "cbv"  "csq"  "cld"  "cace" "cck"  "cbut" "ctyk"
## [931] "ceu"  "ctae" "cfm"  "cchv" "carg" "cdrk" "csep" "cdy"  "cfer" "csh" 
## [941] "ciu"  "mct"  "mcs"  "mcat" "moi"  "mbl"  "mboi" "mcun" "mnn"  "bcef"
## [951] "bcir" "bse"  "cih"  "chh"  "chrs" "chrz" "csha" "cil"  "ccas" "ctak"
## [961] "lue"  "xfa"  "xft"  "xfm"  "xfn"  "xff"  "xfl"  "xfs"  "xfh"  "xtw" 
## [971] "mgm"  "maes" "mfn"  "chu"  "erb"
```

Organisms classified within cluster 4

```
##    [1] "dtm"  "goc"  "git"  "gru"  "gav"  "god"  "nak"  "kra"  "gez"  "oek" 
##   [11] "bfa"  "brx"  "brz"  "bsau" "brr"  "ars"  "dni"  "day"  "agg"  "cmh" 
##   [21] "cart" "cry"  "hea"  "hum"  "huw"  "leif" "lse"  "leu"  "ltr"  "ldn" 
##   [31] "mvd"  "plap" "rtn"  "rry"  "ria"  "rfs"  "rte"  "sala" "sald" "aai" 
##   [41] "gar"  "cgl"  "cgb"  "cgu"  "cgt"  "cgs"  "cgg"  "cgm"  "cgj"  "cgq" 
##   [51] "cgx"  "cjk"  "crd"  "cva"  "cfn"  "ccg"  "cvt"  "chm"  "cmq"  "ccj" 
##   [61] "cmv"  "cei"  "cted" "clw"  "cdx"  "csp"  "csta" "ccjz" "cpho" "cfc" 
##   [71] "cstr" "camg" "cmin" "cxe"  "cee"  "cpre" "csur" "aau"  "pue"  "psey"
##   [81] "satk" "agy"  "agm"  "agf"  "mts"  "mim"  "mio"  "mip"  "mcw"  "mpal"
##   [91] "mih"  "micr" "maur" "mhos" "mfol" "moo"  "mlv"  "mwa"  "mprt" "msed"
##  [101] "moy"  "iva"  "ido"  "cfl"  "cfi"  "cez"  "celz" "cej"  "celh" "halt"
```

```
## [111] "ske"  "cet"  "cceu" "fsy"  "noy"  "noo"  "nsn"  "nmes" "pfr"  "pfre"
## [121] "paus" "tfl"  "tfa"  "tez"  "tla"  "ahm"  "acad" "ahg"  "sacc" "krh"
## [131] "kfv"  "kii"  "kod"  "kvr"  "nal"  "tfu"  "erz"  "euz"  "fln"  "nso"
## [141] "nia"  "nko"  "fla"  "fgg"  "hhy"  "bbd"  "cmr"  "camu" "evi"  "est"
## [151] "echi" "rhoz" "flm"  "fll"  "mtt"  "add"  "aswu" "hsw"  "hym"  "hyg"
## [161] "hyp"  "hyz"  "hnv"  "hyj"  "hrs"  "pko"  "pact" "ruf"  "rti"  "alm"
## [171] "fbt"  "eol"  "lby"  "rsi"  "run"  "rup"  "sli"  "srd"  "smon" "spir"
## [181] "spik" "spib" "dfe"  "aqb"  "aqa"  "aqd"  "aalg" "cagg" "emar" "gfo"
## [191] "grl"  "gfl"  "grs"  "kos"  "kan"  "fbc"  "marm" "mart" "marb" "mare"
## [201] "mesq" "mrs"  "mlt"  "mut"  "myr"  "mpw"  "mod"  "myz"  "pom"  "pob"
## [211] "prn"  "pola" "poa"  "phal" "ptq"  "rbi"  "seon" "sze"  "lan"  "lvn"
## [221] "laci" "fop"  "taj"  "marf" "ahz"  "ten"  "tje"  "tmar" "tmp"  "lut"
## [231] "fek"  "wfu"  "win"  "psyn" "zpr"  "salt" "eao"  "emn"  "een"  "elb"
## [241] "ego"  "egm"  "elz"  "ebv"  "efal" "fbu"  "fbe"  "agd"  "mup"  "muc"
## [251] "mgot" "muh"  "mgin" "mgk"  "mrub" "mgos" "oli"  "scn"  "phe"  "pep"
## [261] "pcm"  "psty" "pgs"  "pek"  "proe" "sbx"  "cpi"  "cbae" "chit" "chih"
## [271] "rmr"  "rmg"  "sru"  "srm"  "apak" "aia"  "anm"  "aamy" "anl"  "and"
## [281] "acai" "bly"  "blin" "bri"  "blut" "fpn"  "far"  "gth"  "ptl"  "ptb"
## [291] "grc"  "oih"  "pof"  "hhd"  "hmn"  "hli"  "lao"  "gka"  "gte"  "gtk"
## [301] "gtm"  "gli"  "gtn"  "gwc"  "gyc"  "gya"  "gct"  "gmc"  "ggh"  "gjf"
## [311] "gea"  "gse"  "gsr"  "gej"  "anx"  "asoc" "coh"  "cohn" "mtu"  "mtv"
## [321] "mtc"  "mra"  "mtf"  "mtb"  "mtk"  "mtz"  "mtg"  "mte"  "mtur" "mtl"
## [331] "mto"  "mtd"  "mtn"  "mtj"  "mtub" "mtue" "mtx"  "mtul" "mtut" "mtuu"
## [341] "mtq"  "mbo"  "mbb"  "mbt"  "mbm"  "mbk"  "mbx"  "maf"  "mmic" "mce"
## [351] "mcq"  "mcv"  "mcx"  "mcz"  "mlp"  "mhad" "mxe"  "blr"  "bagr" "brw"
## [361] "jeo"  "lyz"  "lpak" "pln"  "pku"  "prt"  "pll"  "pana" "pdg"  "phc"
## [371] "pfae" "pmat" "psyo" "sob"  "spae" "play" "vir"  "vhl"  "vne"  "vpn"
## [381] "vim"  "kpul" "pjd"  "gym"  "ppq"  "pbd"  "pgm"  "pod"  "paen" "paef"
## [391] "paeq" "pste" "paeh" "paej" "pbj"  "pri"  "ppeo" "pib"  "pkb"  "pvo"
## [401] "plw"  "ppsc" "palb" "pchi" "pbk"  "prz"  "plyc" "tvu"  "gfe"  "sthr"
## [411] "dhd"  "dor"  "sth"  "sted" "gbe"  "gbh"  "gbc"  "gbs"  "gdi"  "gdj"
## [421] "mick" "aex"  "chq"  "pzu"  "phb"  "bsb"  "brd"  "bne"  "brg"  "brl"
## [431] "bvc"  "bdm"  "brf"  "brev" "bmed" "hne"  "mmr"  "hbc"  "msc"  "mbry"
## [441] "mros" "mhey" "dge"  "ddr"  "dmr"  "dgo"  "dpd"  "dsw"  "dfc"  "dein"
```

```
## [451] "dga"  "mtw"  "hdn"  "hdt"  "hmc"  "rbs"  "rva"  "nwi"  "nha"  "oca"
## [461] "ocg"  "oco"  "blag" "paru" "rhu"  "pseb" "otm"  "mag"  "mgy"  "mgry"
## [471] "magx" "magn" "dex"  "rpm"  "aep"  "spmi" "sclo" "sami" "sphp" "sphl"
## [481] "spho" "splm" "splk" "sphf" "slut" "eli"  "elq"  "err"  "erf"  "emv"
## [491] "pns"  "porl" "pot"  "ngf"  "pla"  "bdz"  "dov"  "dwd"  "dalk" "dat"
## [501] "des"  "deu"  "ccx"  "mfu"  "mmas" "mxa"  "msd"  "mym"  "cfus" "sur"
## [511] "llu"  "samy" "ade"  "acp"  "afw"  "ank"  "dti"  "sfu"  "ttj"  "tts"
## [521] "ttl"  "sva"  "oce"  "ocm"  "opf"  "cate" "cao"  "cly"  "clh"  "cbal"
## [531] "cbat" "gag"  "gni"  "rhh"  "salm" "salk" "lsd"  "mvs"  "mya"  "mmaa"
## [541] "pin"  "fbl"  "fes"  "com"  "coz"  "colw" "cola" "cber" "cov"  "blep"
## [551] "aeh"  "lhk"  "beb"  "rsp"  "rsh"  "rsq"  "rcp"  "rhp"  "rbl"  "bav"
## [561] "dug"  "jab"  "jaj"  "lmir" "mtim" "mpli" "nok"  "pnu"  "har"  "rsc"
## [571] "art"  "arr"  "arm"  "arl"  "are"  "arw"  "ary"  "aru"  "arn"  "arx"
## [581] "arth" "acit" "rac"  "rge"  "rbn"  "tin"  "thi"  "upv"  "chiz" "cfon"
## [591] "cari" "amah" "chri" "chrb" "chrm" "aql"  "htl"  "nwe"  "nba"  "neu"
## [601] "net"  "nii"  "nco"  "nst"  "atw"  "dsu"  "app"  "doe"  "shg"  "sht"
## [611] "sphn" "smiz" "spsc" "sphz" "sphe" "spdr" "sdj"  "stha" "metr" "tcl"
## [621] "tak"  "shd"  "dey"  "rbh"  "ttu"  "mthd" "micc" "maga" "mii"  "mict"
## [631] "mhyd" "ceb"  "cell" "cek"  "ceg"  "osg"  "alv"  "mpur" "thip" "aprs"
## [641] "ebs"  "buf"  "bage" "cnt"  "cem"  "cen"  "clap" "sfo"  "eic"  "etr"
## [651] "etd"  "ete"  "etc"  "edw"  "edl"  "eho"  "ahn"  "pfq"  "aha"  "ahy"
## [661] "ahd"  "ahr"  "ahp"  "ahj"  "ahh"  "ahi"  "aaj"  "asa"  "aeo"  "avr"
## [671] "avo"  "amed" "asr"  "adh"  "acav" "aem"  "aea"  "arv"  "aes"  "ael"
## [681] "eco"  "ecj"  "ecd"  "ebw"  "ecok" "ece"  "ecs"  "ecf"  "etw"  "elx"
## [691] "eoi"  "eoj"  "eoh"  "ecoo" "ecoh" "esl"  "eso"  "esm"  "eck"  "ecg"
## [701] "eok"  "elr"  "elh"  "ecw"  "eun"  "ecp"  "ena"  "ecos" "ecv"  "ecoa"
## [711] "ecx"  "ecm"  "ecy"  "ecr"  "ecq"  "eum"  "ect"  "eoc"  "ebr"  "ebl"
## [721] "ebe"  "ebd"  "eci"  "eih"  "ecz"  "ecc"  "elo"  "eln"  "ese"  "ecl"
## [731] "eko"  "ekf"  "eab"  "edh"  "edj"  "elu"  "elw"  "ell"  "elc"  "eld"
## [741] "elp"  "elf"  "ecol" "ecoi" "ecoj" "efe"  "eal"  "ema"  "esz"  "sfl"
## [751] "sfx"  "sfv"  "sfe"  "sfn"  "sfs"  "sft"  "ssn"  "sbo"  "sbc"  "sdy"
## [761] "sdz"  "shq"  "esa"  "csk"  "csz"  "csj"  "ccon" "cdm"  "csi"  "cmj"
## [771] "cui"  "cmw"  "ctu"  "ksa"  "kor"  "krd"  "kco"  "kot"  "kpse" "hav"
## [781] "hpar" "opo"  "ddd"  "dda"  "dze"  "ddc"  "dzc"  "dso"  "ced"  "dfn"
```

```
## [791] "ddq"  "daq"  "dic"  "lax"  "lei"  "leh"  "lee"  "ler"  "lea"  "laz"
## [801] "lef"  "lni"  "lew"  "bgj"  "brb"  "bng"  "lpop" "mmk"  "kie"  "psts"
## [811] "pshi" "psi"  "psx"  "psta" "prg"  "pala" "phei" "prq"  "prj"  "pvc"
## [821] "lmo"  "lmn"  "lmy"  "lmt"  "lmoc" "lmod" "lmow" "lmoq" "lmr"  "lmom"
## [831] "lmg"  "lms"  "lmj"  "lmw"  "lmx"  "lmz"  "lmos" "lmoy" "lmot" "lsg"
## [841] "sty"  "stt"  "sex"  "sent" "stm"  "seo"  "sev"  "sey"  "sem"  "sej"
## [851] "seb"  "sef"  "setu" "setc" "senr" "send" "seni" "seen" "spt"  "sek"
## [861] "spq"  "sei"  "sec"  "seh"  "shb"  "senh" "seeh" "see"  "senn" "sew"
## [871] "sea"  "sens" "sed"  "seg"  "sel"  "sega" "set"  "sena" "seno" "senv"
## [881] "senq" "senl" "senj" "seec" "seeb" "seep" "senb" "sene" "senc" "ses"
## [891] "sbg"  "sbz"  "sbv"  "salz" "smar" "smw"  "sers" "srz"  "sfj"  "sof"
## [901] "rah"  "raq"  "raa"  "rox"  "eame" "pam"  "plf"  "paq"  "pagg" "kln"
## [911] "pant" "panp" "pstw" "palh" "pey"  "pdis" "fjo"  "fjg"  "fbr"  "fgl"
## [921] "fcm"  "ffa"  "fcr"  "fsn"  "fnk"  "tci"  "tpty" "ebc"  "sod"  "eam"
## [931] "eay"  "eta"  "epy"  "epr"  "erj"  "ege"  "epe"  "erwi" "pmr"  "pmib"
## [941] "pvl"  "pvg"  "phau" "prot" "pcol" "pcib" "ype"  "ypk"  "yph"  "ypa"
## [951] "ypn"  "ypm"  "ypp"  "ypg"  "ypz"  "ypt"  "ypd"  "ypx"  "ypw"  "ypj"
## [961] "ypv"  "ypl"  "yps"  "ypo"  "ypi"  "ypy"  "ypb"  "ypq"  "ypu"  "ypr"
## [971] "ypc"  "ypf"  "yen"  "yep"  "yey"  "yel"  "yew"  "yet"  "yef"  "yee"
## [981] "ysi"  "yal"  "yfr"  "yin"  "ykr"  "yro"  "yru"  "yrb"  "yak"  "yma"
## [991] "yhi"  "yca"  "ymo"  "pct"  "pcv"  "pwa"  "ppar" "pec"  "pws"  "pbra"
## [1001] "ppuj" "cro"  "cko"  "cfd"  "cbra" "cwe"  "cfq"  "cama" "caf"  "cfar"
## [1011] "cir"  "cie"  "cpar" "rpln" "yre"  "sgoe" "pdz"  "pgz"  "pcd"  "mint"
## [1021] "mthi" "gbi"  "tgr"  "mmob" "mmai" "amc"  "amh"  "amaa" "amal" "amae"
## [1031] "amao" "amad" "amai" "amag" "amac" "amb"  "amg"  "amk"  "alt"  "aal"
## [1041] "aaus" "asp"  "asq"  "aaw"  "ale"  "alz"  "apel" "abo"  "adi"  "apac"
## [1051] "aln"  "axe"  "ilo"  "ili"  "ipi"  "idi"  "idt"  "cmai" "kus"  "kma"
## [1061] "kuy"  "haa"  "hhu"  "hco"  "hsi"  "hag"  "haf"  "hmd"  "haxi" "kge"
## [1071] "llp"  "nik"  "ncu"  "ome"  "bmar" "gsn"  "tol"  "tor"  "lpn"  "lph"
## [1081] "lpo"  "lpu"  "lpm"  "lpf"  "lpp"  "lpc"  "lpa"  "lpe"  "llo"  "lfa"
## [1091] "lha"  "lok"  "lcd"  "lsh"  "llg"  "lib"  "ljr"  "lcj"  "lwa"  "lss"
## [1101] "csr"  "mos"  "par"  "pcr"  "prw"  "pso"  "pur"  "pali" "pspg" "psyg"
## [1111] "psyc" "psya" "psyy" "psyp" "pay"  "ptt"  "xbo"  "xbv"  "xne"  "xnm"
## [1121] "xdo"  "xpo"  "xho"  "enc"  "enl"  "eclg" "ecle" "ecln" "ecli" "eclx"
```

```
## [1131] "ecly" "eclz" "eclo" "ehm"  "exf"  "ecla" "eclc" "eau"  "ekb"  "eno"
## [1141] "eec"  "elg"  "ecan" "ern"  "ecls" "echg" "esh"  "ent"  "eas"  "enr"
## [1151] "enx"  "enf"  "ebg"  "end"  "kpw"  "kpq"  "eae"  "ear"  "esc"  "kle"
## [1161] "acb"  "abm"  "abc"  "abn"  "abb"  "abx"  "abz"  "abr"  "abd"  "abh"
## [1171] "abad" "abj"  "abaj" "abaz" "abaa" "abw"  "abal" "ano"  "att"  "aei"
## [1181] "acv"  "ahl"  "ajn"  "ala"  "asj"  "aid"  "awu"  "acum" "aug"  "alw"
## [1191] "achi" "alj"  "maq"  "mhc"  "mad"  "mbs"  "mari" "mara" "bsu"  "bsr"
## [1201] "bsl"  "bsh"  "bsy"  "bsut" "bsul" "bsus" "bso"  "bsn"  "bsq"  "bsx"
## [1211] "bsp"  "bss"  "bst"  "bli"  "bld"  "blh"  "bay"  "baq"  "bya"  "bamp"
## [1221] "baml" "bama" "bamn" "bamb" "bamt" "bamy" "bmp"  "bao"  "baz"  "bql"
## [1231] "bxh"  "bqy"  "bami" "bamc" "bamf" "bsia" "bae"  "bvm"  "bson" "bht"
## [1241] "ban"  "bar"  "bat"  "bah"  "bai"  "bax"  "bant" "banr" "bans" "banh"
## [1251] "banv" "bce"  "bca"  "bcz"  "bcr"  "bcb"  "bcu"  "bcg"  "bcq"  "bcx"
## [1261] "bal"  "bnc"  "bcf"  "bcer" "bcy"  "btk"  "btl"  "btb"  "btt"  "bthr"
## [1271] "bthi" "btc"  "btf"  "btm"  "btg"  "bti"  "btn"  "btht" "bthu" "btw"
## [1281] "bthy" "bwe"  "bww"  "bmyo" "bty"  "bmyc" "bby"  "bwd"  "btro" "bmob"
## [1291] "bpu"  "bpum" "bpus" "bco"  "bjs"  "baci" "bif"  "bmet" "gst"  "bacw"
## [1301] "bacp" "bacb" "bacy" "bacl" "balm" "bsm"  "bgy"  "bwh"  "bxi"  "bhk"
## [1311] "bbev" "balt" "bacs" "bsaf" "bit"  "bcoh" "bda"  "bck"  "bag"  "bcoa"
## [1321] "bha"  "bcl"  "bpf"  "ble"  "bon"  "cum"  "cub"  "cug"  "chz"  "cgn"
## [1331] "cio"  "chry" "cpip" "carh" "cnk"  "cjt"  "ccau" "cben" "clac" "rbz"
## [1341] "faq"  "sdf"  "tee"  "this" "gho"  "ppr"  "pgb"  "pds"  "vch"  "vcf"
## [1351] "vcs"  "vce"  "vcq"  "vcj"  "vci"  "vco"  "vcr"  "vcm"  "vcl"  "vcx"
## [1361] "vcz"  "vvu"  "vvy"  "vvm"  "vvl"  "vpa"  "vpb"  "vpk"  "vpf"  "vph"
## [1371] "vha"  "vca"  "vag"  "vex"  "vdb"  "vhr"  "vow"  "vro"  "vsp"  "vfu"
## [1381] "vni"  "van"  "lag"  "vau"  "vcy"  "vct"  "vtu"  "vfl"  "vmi"  "vbr"
## [1391] "vsc"  "vga"  "vsh"  "vqi"  "vta"  "vaf"  "vnl"  "vcc"  "vas"  "vaq"
## [1401] "vsr"  "saly" "sks"  "scot" "son"  "sdn"  "sfr"  "saz"  "sbl"  "sbm"
## [1411] "sbn"  "sbp"  "sbt"  "sbs"  "sbb"  "slo"  "spc"  "shp"  "sse"  "spl"
## [1421] "she"  "shm"  "shn"  "shw"  "shl"  "swd"  "swp"  "svo"  "shf"  "sja"
## [1431] "spsw" "sbj"  "smav" "shew" "salg" "slj"  "smai" "spol" "sbk"  "skh"
## [1441] "saes" "tht"  "thap" "pha"  "pat"  "psm"  "pseo" "pia"  "pphe" "pbw"
## [1451] "prr"  "plz"  "paln" "ppis" "pea"  "pspo" "part" "ptu"  "png"  "ptd"
## [1461] "psen" "pdj"  "paga" "pcar" "pmaa" "fau"  "dji"  "dja"  "dtx"  "dye"
```

```
## [1471] "lrz"  "lpy"  "rhd"  "dko"  "lab"  "laq"  "lcp"  "lgu"  "lez"  "lem"
## [1481] "lmb"  "lyt"  "lyj"  "lsol" "psu"  "psuw" "pmex" "pmy"  "pmk"  "palc"
## [1491] "pci"  "poi"  "psa"  "psz"  "psr"  "psj"  "psh"  "prh"  "psw"  "ppv"
## [1501] "psec" "psed" "thes" "theh" "tcn"  "tbv"  "lum"  "lus"  "lug"  "sml"
## [1511] "smt"  "buj"  "smz"  "sacz" "stek" "srh"  "slm"  "sten" "stem" "stes"
## [1521] "xcc"  "xcb"  "xca"  "xcp"  "xcv"  "xax"  "xfu"  "xom"  "xoo"  "xop"
## [1531] "xoy"  "xor"  "xoz"  "xtn"  "xfr"  "xve"  "xhr"  "xga"  "xhy"  "xcz"
## [1541] "xth"
```

Organisms classified within cluster 5

```
##   [1] "asg"  "ahe"  "arca" "mcu"  "tpy"  "tpyo" "blo"  "blj"  "bln"  "blon"
##  [11] "blf"  "bll"  "blb"  "blm"  "blk"  "blg"  "blz"  "blx"  "bad"  "badl"
##  [21] "bado" "bla"  "blc"  "blt"  "bbb"  "bbc"  "bnm"  "blv"  "blw"  "bls"
##  [31] "bani" "banl" "bni"  "banm" "bbp"  "bbi"  "bbf"  "bbv"  "bbru" "bbre"
##  [41] "bbrv" "bbrj" "bbrc" "bbrn" "bbrs" "bbrd" "bast" "btp"  "bcor" "bka"
##  [51] "bks"  "bcat" "bpsp" "bii"  "bang" "bpsc" "bsca" "bcho" "bgx"  "blem"
##  [61] "beu"  "dva"  "djj"  "leb"  "rmu"  "rdn"  "raj"  "pad"  "cacn" "cgrn"
##  [71] "actz" "ols"  "caer" "cgo"  "ele"  "eyy"  "ddt"  "pgi"  "pgn"  "pgt"
##  [81] "pah"  "pcre" "pcag" "alq"  "pmz"  "pdn"  "pit"  "pro"  "pfus" "peo"
##  [91] "pje"  "poc"  "ash"  "aok"  "acou" "ada"  "tfo"  "toh"  "coc"  "ccm"
## [101] "col"  "chg"  "capn" "cgh"  "clk"  "cspu" "capf" "orh"  "ori"  "bcad"
## [111] "cte"  "paa"  "proc" "prs"  "cch"  "got"  "gmo"  "geq"  "gsa"  "gha"
## [121] "pgq"  "jea"  "mcl"  "aur"  "aun"  "asan" "acg"  "auh"  "dpm"  "adc"
## [131] "jda"  "jpo"  "thl"  "tey"  "too"  "tkr"  "vte"  "vac"  "vao"  "vah"
## [141] "vcp"  "ppe"  "ppen" "pce"  "pdm"  "paci" "pio"  "lme"  "lmm"  "lmk"
## [151] "lki"  "lec"  "lcn"  "lgs"  "llf"  "lgc"  "lpse" "wko"  "wce"  "wct"
## [161] "wci"  "wcb"  "wjo"  "wpa"  "wcf"  "wso"  "whe"  "wei"  "wdi"  "wvr"
## [171] "lrn"  "lack" "spy"  "spz"  "spym" "spya" "spm"  "spg"  "sps"  "sph"
## [181] "spi"  "spj"  "spk"  "spf"  "spa"  "spb"  "stg"  "stx"  "soz"  "stz"
## [191] "spyh" "spyo" "spn"  "spd"  "spr"  "spw"  "sjj"  "snv"  "spx"  "snt"
## [201] "snd"  "spnn" "sne"  "spv"  "snc"  "snm"  "spp"  "sni"  "spng" "snb"
## [211] "snp"  "snx"  "snu"  "spne" "spnu" "spnm" "spno" "san"  "smc"  "smut"
```

```
## [221] "smj"  "smua" "stc"  "stl"  "ste"  "stn"  "stu"  "stw"  "sthe" "sths"
## [231] "ssf"  "srp"  "sez"  "seq"  "sezo" "sequ" "seu"  "sds"  "sdg"  "sda"
## [241] "sdc"  "sdq"  "smb"  "scp"  "scf"  "ssr"  "stf"  "stj"  "strs" "ssah"
## [251] "std"  "sie"  "sib"  "siu"  "sanc" "scg"  "scon" "scos" "sik"  "siq"
## [261] "sio"  "siz"  "slu"  "sig"  "sip"  "stra" "seqi" "ski"  "sgw"  "splr"
## [271] "efa"  "efi"  "efs"  "efn"  "efq"  "ene"  "efc"  "efau" "efu"  "efm"
## [281] "eft"  "edu"  "esg"  "dau"  "asf"  "asm"  "aso"  "asb"  "aoe"  "mba"
## [291] "mby"  "mbw"  "mbar" "mbak" "mac"  "mma"  "mmaz" "mmj"  "mmac" "mvc"
## [301] "mek"  "mls"  "metm" "mef"  "meq"  "msj"  "msz"  "msw"  "mthr" "mthe"
## [311] "mhor" "mfz"  "spoa" "fma"  "pmic" "ped"  "phar" "piv"  "obj"  "rix"
## [321] "rim"  "coo"  "bhan" "blau" "blab" "dru"  "dfg"  "tjr"  "ded"  "dec"
## [331] "drs"  "faa"  "apr"  "roc"  "psor" "eha"  "fpr"  "fpra" "cce"  "rch"
## [341] "rum"  "rus"  "ruj"  "rgn"  "hsc"  "rbp"  "csc"  "ate"  "cob"  "chd"
## [351] "cow"  "cki"  "ckn"  "clc"  "ccha" "toc"  "tpz"  "mta"  "mtho" "mthz"
## [361] "erh"  "ers"  "erl"  "eri"  "erd"  "eio"  "fro"  "esr"  "esu"  "euu"
## [371] "tur"  "tsg"  "ain"  "awo"  "dho"  "ljo"  "ljf"  "ljh"  "ljn"  "lac"
## [381] "lad"  "laf"  "lde"  "ldl"  "lga"  "lhe"  "lhl"  "lhr"  "lhv"  "lhh"
## [391] "lhd"  "lcr"  "lam"  "lai"  "lay"  "lke"  "law"  "lae"  "lgl"  "lamy"
## [401] "lpw"  "lkl"  "lre"  "lrf"  "lru"  "lrt"  "lrr"  "lfr"  "lmu"  "lbr"
## [411] "lbk"  "lzy"  "lsl"  "lsi"  "lrm"  "laca" "lcy"  "lho"  "lol"  "lku"
## [421] "lali" "lsa"  "sele" "selo" "selt" "vat"  "bhe"  "bhn"  "bhs"  "bqu"
## [431] "bqr"  "bbk"  "btr"  "btx"  "bgr"  "bcd"  "baus" "bvn"  "banc" "bart"
## [441] "bara" "barw" "barr" "baro" "barj" "bez"  "barn" "bky"  "bals" "las"
## [451] "laa"  "lat"  "lso"  "lcc"  "lau"  "pmut" "rbt"  "ren"  "dak"  "deo"
## [461] "gao"  "bsto" "tpar" "cthi" "top"  "tcm"  "thet" "tmai" "caw"  "carc"
## [471] "awa"  "bpsi" "kde"  "kso"  "sutt" "sutk" "teq"  "tea"  "teg"  "tas"
## [481] "tat"  "kki"  "koa"  "eex"  "cey"  "rvi"  "bcib" "hde"  "icp"  "bfl"
## [491] "bpn"  "bva"  "bchr" "ben"  "bed"  "asy"  "kgo"  "ssz"  "hhs"  "pck"
## [501] "pes"  "seny" "ehd"  "wbr"  "wgl"  "ftu"  "ftf"  "ftt"  "ftg"  "fth"
## [511] "fts"  "fti"  "fto"  "frf"  "fper" "adp"  "ahu"  "aap"  "aseg" "psky"
## [521] "hip"  "hiq"  "hif"  "hil"  "hie"  "hia"  "hic"  "hpr"  "hdu"  "hay"
## [531] "hhz"  "haeg" "hpaa" "ctc"  "ctet" "cia"  "ccoh" "ccel" "csci" "cso"
## [541] "msx"  "cqf"  "pade" "acl"  "abra" "apal" "aoc"  "ahk"  "tbm"  "tbz"
```

Weisfeiler-Lehman (WL) kernel

Heatmap

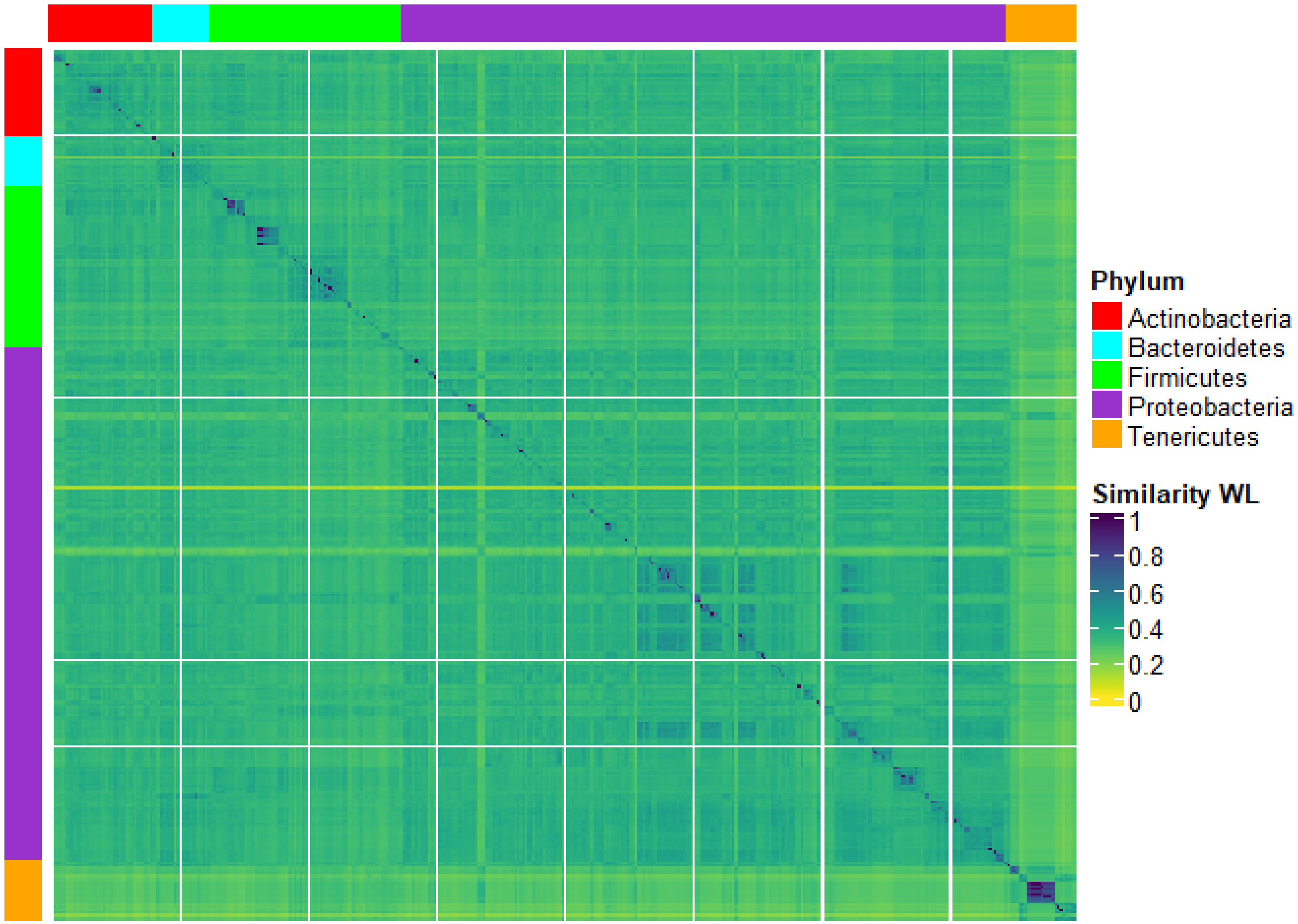

MDS for WL

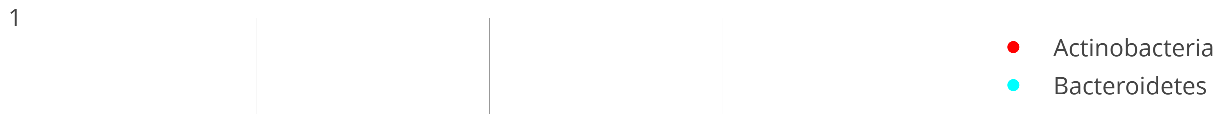

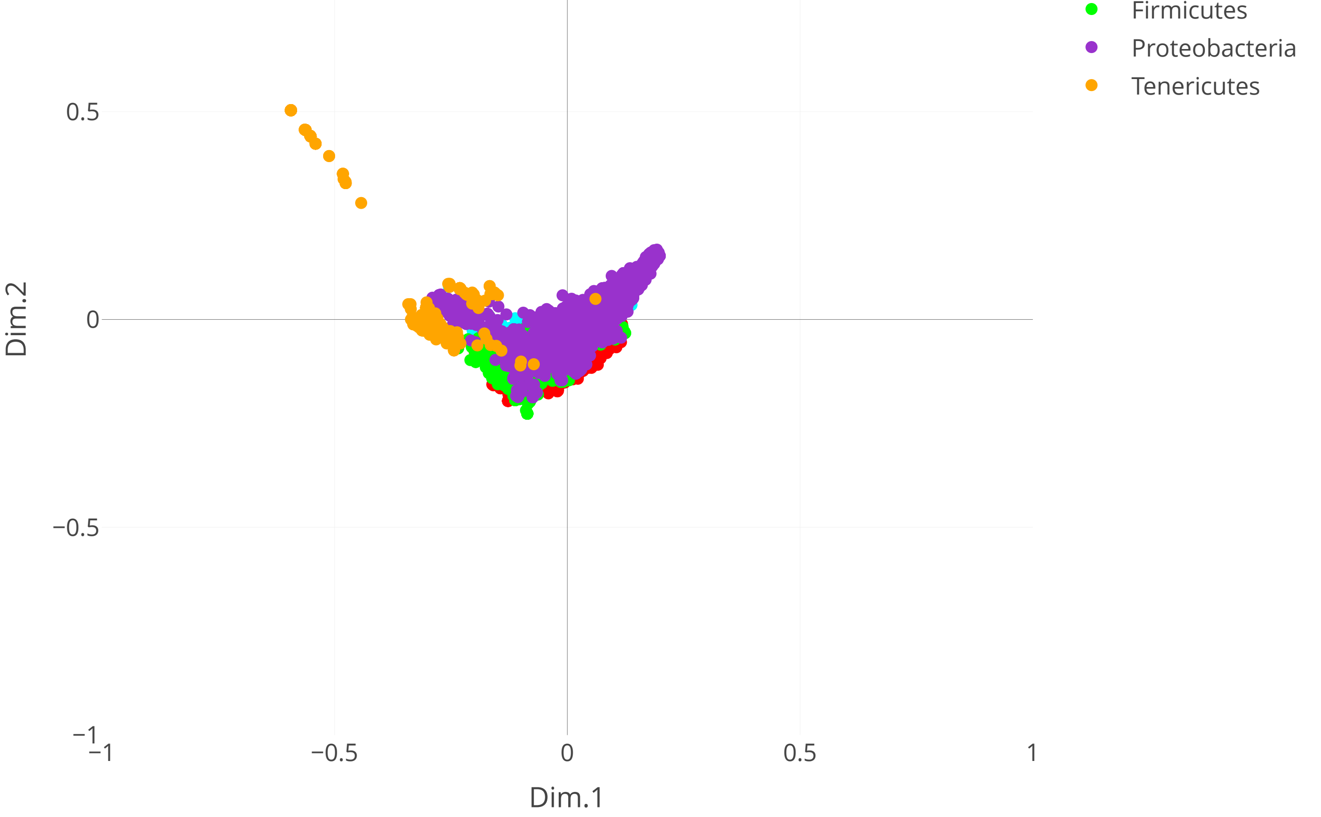

5-means clustering for WL

```
##                  Cluster
## Real group          1    2    3    4    5
##   Actinobacteria    2    0  237    8  224
##   Bacteroidetes    45   12   79    9  115
##   Firmicutes        0    0  686   75  108
##   Proteobacteria  877   34  546  109 1182
##   Tenericutes       0  130    7  186    1
```

Organisms classified within cluster 1

```
##   [1] "cmc"  "cmh"  "nso"  "nia"  "nko"  "fla"  "fgg"  "hhy"  "bbd"  "evi"
##  [11] "echi" "mtt"  "hsw"  "hyd"  "hyz"  "hyj"  "hrs"  "ruf"  "rti"  "rud"
##  [21] "fbt"  "eol"  "lby"  "rsi"  "rup"  "sli"  "srd"  "smon" "spir" "dfe"
```

```
##  [31] "gfl"  "mare" "nob"  "pob"  "prn"  "pola" "muc"  "mgk"  "oli"  "scn" 
##  [41] "phe"  "pcm"  "psty" "pek"  "proe" "sbx"  "chit" "abg"  "gal"  "nch" 
##  [51] "aex"  "ccr"  "chq"  "cfh"  "cauf" "phb"  "tpro" "sech" "bdz"  "ccx" 
##  [61] "mfu"  "mmas" "mxa"  "mym"  "dti"  "sva"  "tau"  "zdf"  "gag"  "gni" 
##  [71] "rhh"  "salm" "salk" "lsd"  "mvs"  "mya"  "mmaa" "pin"  "fbl"  "com" 
##  [81] "coz"  "colw" "cola" "cber" "beb"  "beba" "btrm" "dug"  "jag"  "jab" 
##  [91] "jsv"  "jaj"  "jas"  "jlv"  "limn" "masz" "mtim" "masy" "mfla" "mpli"
## [101] "nok"  "pkt"  "upv"  "chiz" "cfon" "cari" "amah" "aqs"  "cvi"  "cvc" 
## [111] "chri" "chrb" "crz"  "chrm" "chae" "atw"  "dsu"  "app"  "shg"  "sphe"
## [121] "tmz"  "tcl"  "shd"  "dar"  "dey"  "sdr"  "ttu"  "micc" "maga" "mii" 
## [131] "mhyd" "cja"  "ceb"  "cell" "cek"  "ceg"  "osg"  "buf"  "bage" "cnt" 
## [141] "cem"  "cen"  "clap" "sfo"  "etr"  "etd"  "ete"  "etc"  "edl"  "eho" 
## [151] "ans"  "ahn"  "pfq"  "aha"  "ahy"  "ahd"  "ahr"  "ahp"  "ahj"  "ahh" 
## [161] "ahi"  "aaj"  "asa"  "aeo"  "avr"  "avo"  "amed" "asr"  "adh"  "aem" 
## [171] "aea"  "arv"  "aes"  "ael"  "apt"  "apw"  "apf"  "apu"  "apg"  "apq" 
## [181] "apx"  "apz"  "apk"  "asz"  "asv"  "apom" "aasc" "acet" "aoy"  "eco" 
## [191] "ecj"  "ecd"  "ebw"  "ecok" "ece"  "ecs"  "ecf"  "etw"  "elx"  "eoi" 
## [201] "eoj"  "eoh"  "ecoo" "ecoh" "esl"  "eso"  "esm"  "eck"  "ecg"  "eok" 
## [211] "elr"  "elh"  "ecw"  "eun"  "ecp"  "ena"  "ecos" "ecv"  "ecoa" "ecx" 
## [221] "ecm"  "ecy"  "ecr"  "ecq"  "eum"  "ect"  "eoc"  "ebr"  "ebl"  "ebe" 
## [231] "ebd"  "eci"  "eih"  "ecz"  "ecc"  "elo"  "eln"  "ese"  "ecl"  "eko" 
## [241] "ekf"  "eab"  "edh"  "edj"  "elu"  "elw"  "ell"  "elc"  "eld"  "elp" 
## [251] "elf"  "ecol" "ecoi" "ecoj" "efe"  "eal"  "ema"  "esz"  "sfl"  "sfx" 
## [261] "sfv"  "sfe"  "sfn"  "sfs"  "sft"  "ssn"  "sbo"  "sbc"  "sdy"  "sdz" 
## [271] "shq"  "esa"  "csk"  "csz"  "csj"  "ccon" "cdm"  "csi"  "cmj"  "cui" 
## [281] "cmw"  "ctu"  "ksa"  "kor"  "krd"  "kco"  "kot"  "kpse" "gxl"  "keu" 
## [291] "kha"  "gqu"  "hav"  "hpar" "opo"  "ddd"  "dda"  "dze"  "ddc"  "dzc" 
## [301] "dso"  "ced"  "dfn"  "ddq"  "daq"  "dic"  "lax"  "lei"  "leh"  "lee" 
## [311] "ler"  "lea"  "laz"  "lef"  "lni"  "lew"  "bgj"  "brb"  "bng"  "lbq" 
## [321] "mmk"  "kie"  "kas"  "psts" "pshi" "pge"  "psi"  "psx"  "psta" "prg" 
## [331] "pala" "phei" "prq"  "prj"  "pvc"  "sty"  "stt"  "sex"  "sent" "stm" 
## [341] "seo"  "sev"  "sey"  "sem"  "sej"  "seb"  "sef"  "setu" "setc" "senr"
## [351] "send" "seni" "seen" "spt"  "sek"  "spq"  "sei"  "sec"  "seh"  "shb" 
## [361] "senh" "seeh" "see"  "senn" "sew"  "sea"  "sens" "sed"  "seg"  "sel" 
```

```
## [371] "sega" "set"  "sena" "seno" "senv" "senq" "senl" "senj" "seec" "seeb"
## [381] "seep" "senb" "sene" "senc" "ses"  "sbg"  "sbz"  "sbv"  "salz" "smar"
## [391] "smac" "smw"  "spe"  "srr"  "srl"  "sry"  "sply" "srs"  "sra"  "smaf"
## [401] "slq"  "serf" "sers" "sfw"  "sfg"  "srz"  "sera" "serq" "serm" "squ"
## [411] "sfj"  "sof"  "ssur" "rah"  "raq"  "raa"  "rox"  "eame" "pam"  "plf"
## [421] "paj"  "paq"  "pva"  "pagg" "pao"  "kln"  "pant" "panp" "pagc" "pstw"
## [431] "palh" "pans" "pey"  "pdis" "fcm"  "ebt"  "tci"  "tpty" "ebf"  "ebc"
## [441] "ebu"  "sod"  "eam"  "eay"  "eta"  "epy"  "epr"  "ebi"  "erj"  "ege"
## [451] "epe"  "erwi" "pmr"  "pmib" "pvl"  "pvg"  "phau" "prot" "pcol" "pcib"
## [461] "ype"  "ypk"  "yph"  "ypa"  "ypn"  "ypm"  "ypp"  "ypg"  "ypz"  "ypt"
## [471] "ypd"  "ypx"  "ypw"  "ypj"  "ypv"  "ypl"  "yps"  "ypo"  "ypi"  "ypy"
## [481] "ypb"  "ypq"  "ypu"  "ypr"  "ypc"  "ypf"  "yen"  "yep"  "yey"  "yel"
## [491] "yew"  "yet"  "yef"  "yee"  "ysi"  "yal"  "yfr"  "yin"  "ykr"  "yro"
## [501] "yru"  "yrb"  "yak"  "yma"  "yhi"  "yca"  "ymo"  "eca"  "patr" "pato"
## [511] "pct"  "pcc"  "pcv"  "pwa"  "ppar" "pec"  "pws"  "ppoa" "pbra" "ppuj"
## [521] "cro"  "cko"  "cfd"  "cbra" "cwe"  "cyo"  "cpot" "cfq"  "cama" "caf"
## [531] "cif"  "cfar" "cir"  "cie"  "cpar" "ror"  "ron"  "rpln" "rao"  "rtg"
## [541] "ree"  "yre"  "sgoe" "kin"  "pdz"  "izh"  "pgz"  "pcd"  "mint" "mthi"
## [551] "ftd"  "fty"  "fpm"  "fpz"  "fpj"  "amc"  "amh"  "amaa" "amal" "amae"
## [561] "amao" "amad" "amai" "amag" "amac" "amb"  "amg"  "amk"  "alt"  "aal"
## [571] "aaus" "asp"  "asq"  "aaw"  "alr"  "ale"  "alz"  "apel" "abo"  "aln"
## [581] "ili"  "idi"  "idt"  "cmai" "hel"  "hhu"  "hsi"  "hbe"  "haf"  "halk"
## [591] "hvn"  "haxi" "llp"  "mard" "bmar" "lcd"  "llg"  "ljr"  "pcr"  "pso"
## [601] "psyc" "pay"  "ptt"  "xbo"  "xbv"  "xne"  "xnm"  "xdo"  "xho"  "enc"
## [611] "enl"  "eclg" "ecle" "ecln" "ecli" "eclx" "ecly" "eclz" "eclo" "ehm"
## [621] "exf"  "ecla" "eclc" "eau"  "ekb"  "eno"  "eec"  "elg"  "ecan" "ern"
## [631] "ecls" "echg" "esh"  "ent"  "eas"  "enr"  "enx"  "enf"  "ebg"  "end"
## [641] "kpn"  "kpu"  "kpm"  "kpp"  "kph"  "kpz"  "kpv"  "kpw"  "kpy"  "kpg"
## [651] "kpc"  "kpq"  "kpt"  "kpo"  "kpr"  "kpj"  "kpi"  "kpa"  "kps"  "kpx"
## [661] "kpb"  "kpne" "kpnu" "kpnk" "kva"  "kpe"  "kpk"  "kvd"  "kvq"  "kox"
## [671] "koe"  "koy"  "kom"  "kmi"  "kok"  "koc"  "kqu"  "eae"  "ear"  "kqv"
## [681] "kll"  "klw"  "esc"  "kle"  "abh"  "abaz" "ano"  "aci"  "acv"  "ajn"
## [691] "asol" "ala"  "atn"  "alj"  "maq"  "mhc"  "mbs"  "mara" "tee"  "gho"
## [701] "ppr"  "pds"  "vcx"  "vvu"  "vvy"  "vvm"  "vvl"  "vpa"  "vpb"  "vpk"
```

```
## [711] "vpf"  "vph"  "vha"  "vca"  "vag"  "vex"  "vdb"  "vhr"  "vna"  "vow"
## [721] "vro"  "vsp"  "vej"  "vfu"  "vni"  "van"  "lag"  "vau"  "vcy"  "vtu"
## [731] "vfl"  "vmi"  "vga"  "vsh"  "vqi"  "vta"  "vaf"  "vnl"  "vcc"  "vas"
## [741] "saly" "sks"  "son"  "sdn"  "sfr"  "saz"  "sbl"  "sbm"  "sbn"  "sbp"
## [751] "sbt"  "sbs"  "sbb"  "slo"  "spc"  "shp"  "sse"  "spl"  "she"  "shm"
## [761] "shn"  "shw"  "shl"  "swd"  "swp"  "svo"  "shf"  "sja"  "spsw" "sbj"
## [771] "smav" "shew" "salg" "slj"  "smai" "spol" "sbk"  "skh"  "saes" "tht"
## [781] "thap" "pat"  "psm"  "pseo" "pia"  "pphe" "pbw"  "plz"  "paln" "ppis"
## [791] "pea"  "pspo" "part" "ptu"  "ptd"  "pdj"  "paga" "pcar" "pmaa" "fau"
## [801] "dji"  "dja"  "dtx"  "dye"  "lrz"  "lpy"  "rhd"  "lab"  "laq"  "lcp"
## [811] "lgu"  "lez"  "lem"  "lmb"  "lyt"  "lyj"  "lsol" "psu"  "psuw" "psd"
## [821] "pmex" "pae"  "paev" "paei" "pau"  "pap"  "pag"  "paf"  "pnc"  "paeb"
## [831] "pdk"  "psg"  "prp"  "paep" "paem" "pael" "paes" "paeu" "paeg" "paec"
## [841] "paeo" "pmk"  "palc" "ppb"  "pfv"  "por"  "pst"  "pci"  "pfo"  "pfs"
## [851] "pfb"  "pman" "ptv"  "pazo" "poi"  "pfw"  "pff"  "psa"  "psz"  "psr"
## [861] "psj"  "pstu" "pfz"  "psw"  "ppv"  "ppsy" "panr" "pgg"  "thes" "theh"
## [871] "tcn"  "tbv"  "lum"  "lus"  "lug"  "sml"  "smt"  "buj"  "smz"  "sacz"
## [881] "stek" "srh"  "slm"  "sten" "stem" "stes" "xcc"  "xcb"  "xca"  "xcp"
## [891] "xcv"  "xax"  "xac"  "xci"  "xct"  "xcj"  "xcu"  "xcn"  "xcw"  "xcr"
## [901] "xcm"  "xcf"  "xfu"  "xao"  "xom"  "xoo"  "xop"  "xoy"  "xor"  "xoz"
## [911] "xsa"  "xtn"  "xfr"  "xve"  "xpe"  "xhr"  "xga"  "xph"  "xva"  "xan"
## [921] "xar"  "xhy"  "xcz"  "xth"
```

Organisms classified within cluster 2

```
##   [1] "che"  "cec"  "cher" "smg"  "sms"  "smh"  "sum"  "smv"  "smub" "smum"
##  [11] "smue" "smup" "hci"  "hct"  "hcc"  "hcd"  "ndl"  "tpn"  "tpq"  "tpj"
##  [21] "vfg"  "zin"  "bcc"  "baj"  "men"  "meo"  "ppet" "aen"  "cmik" "crp"
##  [31] "cru"  "crc"  "crt"  "crh"  "crv"  "cri"  "ple"  "ply"  "plr"  "plo"
##  [41] "pld"  "plb"  "plc"  "pli"  "paly" "baab" "elj"  "esx"  "efr"  "eml"
##  [51] "mfl"  "mfw"  "mchc" "mlac" "ment" "msyr" "mtab" "mcol" "scr"  "ssyr"
##  [61] "sdi"  "ssab" "satr" "stur" "sll"  "skn"  "scj"  "shj"  "sck"  "sfz"
##  [71] "scla" "sphh" "smoo" "pml"  "pal"  "psol" "pzi"  "mge"  "mgu"  "mgc"
```

```
##  [81] "mgq"  "mgx"  "mpn"  "mpm"  "mpj"  "mpb"  "mga"  "mgh"  "mgf"  "mgn" 
##  [91] "mgs"  "mgt"  "mgv"  "mgw"  "mgac" "mgan" "mgnc" "mgz"  "mmy"  "mmym"
## [101] "mmyi" "mml"  "mcp"  "mcac" "mcap" "mcar" "mcai" "mlc"  "mlh"  "mmo" 
## [111] "mhy"  "mhj"  "mhp"  "mhn"  "mhyl" "mhyo" "mat"  "mco"  "mho"  "mhom"
## [121] "mhr"  "mhh"  "mhm"  "mhs"  "mhv"  "mha"  "mhf"  "mss"  "msk"  "mpf" 
## [131] "mput" "mhe"  "mwe"  "mhl"  "mhb"  "mpv"  "mov"  "mbc"  "mgj"  "mfq" 
## [141] "mcan" "myt"  "mds"  "myg"  "mpho" "mhyv" "mclo" "mamp" "mphc" "mane"
## [151] "mnh"  "mnu"  "mstr" "mgly" "mcou" "mcom" "mpu"  "msy"  "mso"  "maa" 
## [161] "mal"  "mbv"  "mbh"  "mbi"  "mbq"  "mcy"  "mcas" "mck"  "marg" "mbov"
## [171] "mboh" "mphi" "uur"  "upa"  "upr"  "uue"
```

Organisms classified within cluster 3

```
##   [1] "asg"  "actt" "mcu"  "tpy"  "tbw"  "blo"  "blj"  "bln"  "blon" "blf" 
##  [11] "bll"  "blb"  "blm"  "blk"  "blg"  "blz"  "blx"  "bad"  "badl" "bado"
##  [21] "bla"  "blc"  "blt"  "bbb"  "bbc"  "bnm"  "blv"  "blw"  "bls"  "bani"
##  [31] "banl" "bni"  "banm" "bde"  "bdn"  "bbp"  "bbi"  "bbf"  "bbv"  "bbru"
##  [41] "bbre" "bbrv" "bbrj" "bbrc" "bbrn" "bbrs" "bbrd" "bast" "btp"  "bcor"
##  [51] "bka"  "bks"  "bcat" "bpsp" "bii"  "bang" "bpsc" "bsca" "bcho" "bgx" 
##  [61] "blem" "beu"  "cbq"  "gez"  "oek"  "brr"  "dva"  "djj"  "dni"  "day" 
##  [71] "dco"  "aqg"  "lxx"  "lxy"  "leb"  "mvd"  "rtx"  "rtc"  "cgl"  "cgb" 
##  [81] "cgu"  "cgt"  "cgs"  "cgg"  "cgm"  "cgj"  "cgq"  "cgx"  "cdi"  "cdp" 
##  [91] "cdh"  "cdt"  "cde"  "cdr"  "cda"  "cdz"  "cdb"  "cds"  "cdd"  "cdw" 
## [101] "cdv"  "cdip" "cjk"  "cur"  "cua"  "car"  "ckp"  "cpl"  "cpg"  "cpp" 
## [111] "cpk"  "cpq"  "cpx"  "cpz"  "cor"  "cop"  "cod"  "cos"  "coi"  "coe" 
## [121] "cou"  "cpse" "cpsu" "cpsf" "crd"  "cul"  "cuc"  "cue"  "cun"  "cus" 
## [131] "cuq"  "cuz"  "cuj"  "ccn"  "cter" "cmd"  "caz"  "cfn"  "ccg"  "cvt" 
## [141] "cax"  "cii"  "cuv"  "coa"  "cdo"  "csx"  "cku"  "ccj"  "cmv"  "cei" 
## [151] "cted" "cut"  "cdx"  "csp"  "ccjz" "cfk"  "cpho" "cfc"  "cgv"  "cstr"
## [161] "caqu" "csph" "camg" "cmin" "cpeg" "cxe"  "csan" "cgk"  "crf"  "crl" 
## [171] "ccho" "cpso" "rmu"  "rdn"  "raj"  "rter" "rama" "rkr"  "mix"  "iva" 
## [181] "cfl"  "cfi"  "cga"  "cez"  "celz" "cej"  "ske"  "sanw" "pac"  "pak" 
## [191] "pav"  "pax"  "paz"  "paw"  "pad"  "pcn"  "pacc" "pach" "pacn" "cacn"
```

```
## [201] "pra"  "cgrn" "prv"  "pfr"  "pfre" "prl"  "pacd" "paus" "tfl"  "tfa"
## [211] "tez"  "tdf"  "tla"  "kii"  "kod"  "kvr"  "acq"  "aos"  "ard"  "actp"
## [221] "actc" "acto" "ane"  "ahw"  "actz" "air"  "asla" "avc"  "ols"  "olo"
## [231] "caer" "ele"  "eyy"  "ddt"  "gpa"  "erz"  "rrd"  "copr" "pmuc" "pet"
## [241] "buy"  "osp"  "ppn"  "dys"  "pgi"  "pgn"  "pgt"  "pah"  "pcre" "pcag"
## [251] "alq"  "pru"  "pmz"  "pdn"  "pit"  "pdt"  "pro"  "pfus" "peo"  "pje"
## [261] "poc"  "afd"  "ash"  "ald"  "aok"  "acou" "ada"  "ait"  "blq"  "tfo"
## [271] "toh"  "fte"  "flu"  "coc"  "ccm"  "col"  "chg"  "capn" "cgh"  "clk"
## [281] "cspu" "ccyn" "caph" "csto" "capq" "capf" "for"  "foh"  "aev"  "apib"
## [291] "wvi"  "orh"  "ori"  "ran"  "rai"  "rar"  "rag"  "rae"  "rat"  "bcad"
## [301] "oho"  "psn"  "cte"  "cpc"  "clz"  "paa"  "proc" "prs"  "pros" "cts"
## [311] "cch"  "cph"  "cpb"  "cli"  "pvi"  "plt"  "apak" "axl"  "aia"  "afl"
## [321] "agn"  "anm"  "aamy" "anl"  "and"  "acai" "bfv"  "blut" "fpn"  "ptl"
## [331] "ptb"  "grc"  "oih"  "ocn"  "ocb"  "pof"  "sje"  "tap"  "hhd"  "hmn"
## [341] "hli"  "lao"  "gka"  "gte"  "gtk"  "gtm"  "gli"  "gtn"  "gwc"  "gyc"
## [351] "gya"  "gct"  "gmc"  "ggh"  "gjf"  "gea"  "gel"  "gse"  "gsr"  "gej"
## [361] "gmo"  "geq"  "bths" "coh"  "cohn" "esi"  "eat"  "ean"  "exm"  "exu"
## [371] "saca" "tco"  "bbe"  "blr"  "bfm"  "bagr" "brw"  "jeo"  "kur"  "kzo"
## [381] "lsp"  "lgy"  "lfu"  "lys"  "lyb"  "lyz"  "lpak" "pln"  "prt"  "pll"
## [391] "pana" "pdg"  "phc"  "ppla" "plx"  "pmat" "pdec" "psyh" "psyo" "rst"
## [401] "sob"  "spsy" "spop" "sure" "spos" "spae" "pgq"  "paek" "panc" "vir"
## [411] "vhl"  "vig"  "vil"  "vne"  "vpn"  "vim"  "stea" "jea"  "mcl"  "mcak"
## [421] "macr" "shv"  "sau"  "sav"  "saw"  "sah"  "saj"  "sam"  "sas"  "sar"
## [431] "sac"  "sax"  "saa"  "sao"  "sae"  "sad"  "suu"  "suv"  "sue"  "suj"
## [441] "suk"  "suc"  "sut"  "suq"  "suz"  "sud"  "sux"  "suw"  "sug"  "suf"
## [451] "saua" "saue" "saun" "saus" "sauu" "saug" "sauz" "saut" "sauj" "sauk"
## [461] "sauq" "sauv" "sauw" "saux" "sauy" "sauf" "sab"  "suy"  "saub" "saum"
## [471] "sauc" "saur" "saui" "saud" "sams" "suh"  "ser"  "sep"  "sepp" "seps"
## [481] "sha"  "shh"  "ssp"  "sca"  "slg"  "sln"  "ssd"  "sdt"  "sdp"  "swa"
## [491] "spas" "sxy"  "sxl"  "sxo"  "shu"  "scap" "ssch" "sscz" "sagq" "seqo"
## [501] "ssif" "scv"  "spet" "slz"  "scoh" "snl"  "skl"  "sfq"  "shom" "smus"
## [511] "scar" "schr" "sarl" "spic" "ssh"  "ssim" "kpul" "keb"  "lfb"  "pjd"
## [521] "gym"  "ppy"  "ppm"  "ppo"  "ppol" "ppq"  "ppoy" "pta"  "plv"  "psab"
## [531] "pdu"  "pbd"  "pgm"  "pod"  "paen" "paef" "paeq" "pste" "paea" "paee"
```

```
## [541] "paeh" "paej" "pbj"  "pih"  "pri"  "ppeo" "pow"  "pbv"  "pxl"  "pyg" 
## [551] "pswu" "pdh"  "pib"  "pcx"  "pkb"  "paih" "pvo"  "plw"  "plen" "ppsc"
## [561] "plut" "pchi" "pprt" "pbac" "prz"  "plyc" "tvu"  "aur"  "aun"  "aui" 
## [571] "asan" "avs"  "auh"  "dpm"  "adc"  "jep"  "jda"  "jeh"  "jar"  "jpo" 
## [581] "thl"  "tey"  "too"  "tkr"  "vte"  "vpi"  "vac"  "vao"  "vcp"  "pce" 
## [591] "paci" "ooe"  "oen"  "osi"  "lme"  "lmm"  "lmk"  "lci"  "lki"  "lec" 
## [601] "lcn"  "lgs"  "lge"  "llf"  "lgc"  "lsu"  "lpse" "wko"  "wce"  "wct" 
## [611] "wci"  "wcb"  "wjo"  "wpa"  "wcf"  "wso"  "whe"  "wei"  "wvr"  "lla" 
## [621] "llk"  "llt"  "lls"  "lld"  "llx"  "llj"  "llm"  "llc"  "llr"  "lln" 
## [631] "lli"  "llw"  "lgr"  "lgv"  "lpk"  "lrn"  "lact" "lack" "spy"  "spz" 
## [641] "spym" "spya" "spm"  "spg"  "sps"  "sph"  "spi"  "spj"  "spk"  "spf" 
## [651] "spa"  "spb"  "stg"  "stx"  "soz"  "stz"  "spyh" "spyo" "spn"  "spd" 
## [661] "spr"  "spw"  "sjj"  "snv"  "spx"  "snt"  "snd"  "spnn" "sne"  "spv" 
## [671] "snc"  "snm"  "spp"  "sni"  "spng" "snb"  "snp"  "snx"  "snu"  "spne"
## [681] "spnu" "spnm" "spno" "sag"  "san"  "sak"  "sgc"  "sags" "sagl" "sagm"
## [691] "sagi" "sagr" "sagp" "sagc" "sagt" "sage" "sagg" "sagn" "smu"  "smc" 
## [701] "smut" "smj"  "smua" "stc"  "stl"  "ste"  "stn"  "stu"  "stw"  "sthe"
## [711] "sths" "ssa"  "ssb"  "ssu"  "ssv"  "ssi"  "sss"  "ssf"  "ssw"  "sup" 
## [721] "ssus" "sst"  "ssuy" "ssk"  "ssq"  "sui"  "suo"  "srp"  "ssut" "ssui"
## [731] "sgo"  "sez"  "seq"  "sezo" "sequ" "seu"  "sub"  "sds"  "sdg"  "sda" 
## [741] "sdc"  "sdq"  "sga"  "sgg"  "sgt"  "sor"  "stk"  "stb"  "scp"  "scf" 
## [751] "ssr"  "stf"  "stj"  "strs" "ssah" "std"  "smn"  "sif"  "sie"  "sib" 
## [761] "siu"  "sang" "sanc" "sans" "scg"  "scon" "scos" "soi"  "sik"  "siq" 
## [771] "sio"  "siz"  "slu"  "sig"  "sip"  "stv"  "spat" "stra" "strn" "ssob"
## [781] "srq"  "seqi" "ski"  "spei" "srat" "sgw"  "splr" "strg" "efa"  "efl" 
## [791] "efi"  "efd"  "efs"  "efn"  "efq"  "ene"  "efc"  "efau" "efu"  "efm" 
## [801] "eft"  "ehr"  "ecas" "emu"  "edu"  "ega"  "ess"  "eth"  "egv"  "eav" 
## [811] "esg"  "dau"  "cmiu" "sarj" "cale" "amt"  "mac"  "mhor" "gfe"  "spoa"
## [821] "ped"  "cthm" "hmo"  "hcv"  "bprl" "arf"  "acac" "cle"  "cew"  "cpy" 
## [831] "lacy" "bpb"  "bfi"  "bhu"  "pxv"  "rho"  "rix"  "rim"  "cct"  "rob" 
## [841] "byl"  "blau" "bpro" "blab" "dsy"  "dhd"  "ddh"  "ddl"  "dmt"  "dor" 
## [851] "dai"  "dmi"  "pth"  "drm"  "dca"  "dru"  "dfg"  "tjr"  "ded"  "dec" 
## [861] "drs"  "cdf"  "pdc"  "cdc"  "cdl"  "pdf"  "roc"  "psor" "capr" "eha" 
## [871] "fpr"  "fpa"  "fpra" "ova"  "cce"  "ral"  "rch"  "rum"  "rus"  "ruj"
```

```
## [881] "rto"  "rgn"  "hsc"  "lbw"  "ibu"  "bacc" "swo"  "csc"  "ate"  "cob"
## [891] "chd"  "cow"  "cki"  "ckn"  "clc"  "ccha" "toc"  "sth"  "tte"  "ttm"
## [901] "tto"  "txy"  "tsh"  "chy"  "tpz"  "mta"  "mtho" "mthz" "erl"  "erd"
## [911] "eio"  "fro"  "eel"  "ere"  "ert"  "era"  "elm"  "emt"  "elim" "tur"
## [921] "tsg"  "afn"  "ain"  "awo"  "pfac" "dho"  "mfun" "ljo"  "ljh"  "ljn"
## [931] "lde"  "lga"  "lhr"  "lhv"  "lhh"  "lcr"  "lam"  "lai"  "lay"  "lgl"
## [941] "lpw"  "lca"  "lcs"  "lce"  "lcl"  "lpq"  "lpi"  "lpap" "lcb"  "lcx"
## [951] "lrh"  "lrg"  "lrl"  "lra"  "lro"  "lrc"  "lpl"  "lpj"  "lpt"  "lps"
## [961] "lpr"  "lpz"  "lre"  "lrf"  "lru"  "lrt"  "lrr"  "lfe"  "lfr"  "lff"
## [971] "lmu"  "lbh"  "lbn"  "lpar" "lbr"  "lbk"  "lzy"  "lsl"  "lsi"  "lrm"
## [981] "laca" "lcy"  "lho"  "lol"  "lku"  "lali" "lfm"  "lsa"  "med"  "mhw"
## [991] "meg"  "ssg"  "sri"  "sele" "selo" "selt" "sted" "vpr"  "vat"  "vrm"
## [1001] "vdn"  "vnk"  "gox"  "pbr"  "mai"  "man"  "dra"  "dge"  "dmr"  "dpt"
## [1011] "dgo"  "dsw"  "dch"  "dab"  "dpu"  "dwu"  "bhe"  "bhn"  "bhs"  "bqu"
## [1021] "bqr"  "btr"  "btx"  "bgr"  "bvn"  "barw" "bez"  "barn" "bky"  "bals"
## [1031] "lcc"  "pmut" "oct"  "rbt"  "srhi" "zmo"  "zmn"  "zmm"  "zmb"  "zmi"
## [1041] "zmc"  "zmr"  "zmp"  "dto"  "dak"  "dpr"  "deo"  "dog"  "drt"  "dba"
## [1051] "doa"  "pca"  "ppd"  "gsu"  "gsk"  "gur"  "glo"  "gbm"  "gem"  "geb"
## [1061] "gpi"  "gao"  "gsb"  "sat"  "sfu"  "tth"  "ttj"  "tts"  "ttl"  "tsc"
## [1071] "tos"  "taq"  "tpar" "tbc"  "cthi" "top"  "tcm"  "thet" "tmai" "afr"
## [1081] "afe"  "acu"  "acz"  "afi"  "afj"  "atx"  "crn"  "cml"  "caw"  "carc"
## [1091] "cdj"  "carn" "awa"  "ann"  "sdo"  "bpsi" "lmir" "ofo"  "sutt" "sutk"
## [1101] "teq"  "tea"  "teg"  "tas"  "tat"  "fmy"  "slt"  "gca"  "mbac" "mbat"
## [1111] "mmb"  "mfa"  "nme"  "nmp"  "nmh"  "nmd"  "nmm"  "nms"  "nmq"  "nmz"
## [1121] "nma"  "nmw"  "nmx"  "nmc"  "nmn"  "nmt"  "nmi"  "ngo"  "ngk"  "nla"
## [1131] "nel"  "nwe"  "nsi"  "nmj"  "nei"  "nek"  "nfv"  "nsf"  "nzl"  "naq"
## [1141] "nbl"  "nzo"  "ncz"  "nani" "nbc"  "kki"  "koa"  "ecor" "eex"  "salv"
## [1151] "nba"  "net"  "nit"  "nii"  "nur"  "nst"  "nlc"  "chj"  "alv"  "cbu"
## [1161] "cbs"  "cbd"  "cbg"  "cbc"  "rvi"  "hhc"  "eic"  "edw"  "pdi"  "parc"
## [1171] "lmo"  "lmn"  "lmy"  "lmt"  "lmoc" "lmoe" "lmob" "lmod" "lmow" "lmoq"
## [1181] "lmr"  "lmom" "lmf"  "lmc"  "lmog" "lmp"  "lmol" "lmoj" "lmoz" "lmox"
## [1191] "lmh"  "lmq"  "lml"  "lmg"  "lms"  "lmj"  "lmw"  "lmx"  "lmz"  "lmon"
## [1201] "lmos" "lmoo" "lmoy" "lmot" "lmoa" "lmok" "lmv"  "lin"  "lwe"  "lsg"
## [1211] "liv"  "lii"  "liw"  "lia"  "lio"  "lwi"  "lgz"  "pck"  "fps"  "sgl"
```

```
## [1221] "pes"  "ftu"  "ftq"  "ftf"  "ftw"  "ftr"  "ftt"  "ftg"  "ftl"  "fth" 
## [1231] "fta"  "fts"  "fti"  "fto"  "ftc"  "ftv"  "ftz"  "ftm"  "ftn"  "ftx" 
## [1241] "fcf"  "fcn"  "fhi"  "fph"  "fpt"  "fpi"  "fpx"  "frt"  "fna"  "fnl" 
## [1251] "frf"  "fper" "fha"  "frx"  "frm"  "frc"  "fad"  "fmi"  "foo"  "hna" 
## [1261] "tkm"  "tvr"  "mmt"  "hha"  "hhk"  "ntt"  "fpp"  "gap"  "orb"  "apl" 
## [1271] "apj"  "apa"  "asu"  "asi"  "ass"  "aeu"  "apor" "aio"  "adp"  "alig"
## [1281] "bhud" "aap"  "aaz"  "aat"  "aao"  "aan"  "aah"  "aacn" "aact" "aseg"
## [1291] "hso"  "hsm"  "msu"  "mht"  "mhq"  "mhat" "mhx"  "mhae" "mham" "mhao"
## [1301] "mhal" "mhaq" "mhay" "mvr"  "mvi"  "mvg"  "mve"  "mann" "mgra" "ooi" 
## [1311] "pmu"  "pmv"  "pul"  "pmp"  "pmul" "pdag" "psky" "apag" "avt"  "paet"
## [1321] "rpne" "rhey" "hin"  "hit"  "hip"  "hiq"  "hif"  "hil"  "hiu"  "hie" 
## [1331] "hiz"  "hik"  "hia"  "hih"  "hiw"  "hic"  "hix"  "hpr"  "hay"  "hpit"
## [1341] "hhz"  "haeg" "hpaa" "cac"  "cae"  "cay"  "cpe"  "cpf"  "cpr"  "ctc" 
## [1351] "ctet" "cno"  "cbo"  "cba"  "cbh"  "cby"  "cbl"  "cbk"  "cbb"  "cbi" 
## [1361] "cbn"  "cbt"  "cbf"  "cbm"  "cbj"  "cbe"  "cbz"  "cbei" "ckl"  "ckr" 
## [1371] "clj"  "ccb"  "cls"  "clb"  "csr"  "cpas" "cpat" "cpae" "csb"  "cah" 
## [1381] "clt"  "cbv"  "csq"  "cld"  "cace" "cck"  "cbut" "ctyk" "ceu"  "ctae"
## [1391] "cfm"  "cchv" "carg" "cdrk" "cia"  "csep" "cdy"  "ccoh" "cfer" "ccel"
## [1401] "csci" "csh"  "ciu"  "mnn"  "msx"  "bsu"  "bsr"  "bsl"  "bsh"  "bsy" 
## [1411] "bsut" "bsul" "bsus" "bso"  "bsn"  "bsq"  "bsx"  "bsp"  "bss"  "bst" 
## [1421] "bli"  "bld"  "blh"  "bay"  "baq"  "bya"  "bamp" "baml" "bama" "bamn"
## [1431] "bamb" "bamt" "bamy" "bmp"  "bao"  "baz"  "bql"  "bxh"  "bqy"  "bami"
## [1441] "bamc" "bamf" "bsia" "bae"  "bvm"  "bson" "bht"  "ban"  "bar"  "bat" 
## [1451] "bah"  "bai"  "bax"  "bant" "banr" "bans" "banh" "banv" "bce"  "bcz" 
## [1461] "bcr"  "bcb"  "bcu"  "bcq"  "bcx"  "bal"  "bnc"  "bcf"  "bcer" "bcef"
## [1471] "bcy"  "btk"  "btl"  "btb"  "btt"  "bthr" "bthi" "btc"  "btf"  "btm" 
## [1481] "btg"  "btn"  "btht" "bthu" "btw"  "bthy" "bwe"  "bww"  "bmyo" "bty" 
## [1491] "bby"  "bwd"  "btro" "bmob" "bpu"  "bpum" "bpus" "bco"  "bjs"  "bif" 
## [1501] "bmet" "gst"  "bacw" "bacp" "bacb" "bacy" "bacl" "balm" "bsm"  "bgy" 
## [1511] "bwh"  "bxi"  "bbev" "balt" "bacs" "bsaf" "bit"  "bacq" "bcir" "bfd" 
## [1521] "bcoh" "bda"  "bmq"  "bmd"  "bmh"  "bck"  "bag"  "bcoa" "bha"  "bcl" 
## [1531] "bpf"  "ble"  "bse"  "cug"  "cqf"  "pade" "xfa"  "xft"  "xfm"  "xfn" 
## [1541] "xff"  "xfl"  "xfs"  "xfh"  "xtw"  "mgm"  "maes" "mfn"  "abra" "aoc" 
## [1551] "aaxa" "ahk"  "tbm"  "tbz"  "erb"
```

Organisms classified within cluster 4

```
##   [1] "ahe"  "arca" "tpyo" "bact" "cig"  "nfe"  "apv"  "cgo"  "bbl"  "bpi" 
##  [11] "bmm"  "bcp"  "bbg"  "bbq"  "blp"  "blu"  "blck" "got"  "gsa"  "gha" 
##  [21] "acg"  "vah"  "ppe"  "ppen" "pdm"  "pio"  "wdi"  "smb"  "asf"  "asm" 
##  [31] "aso"  "asb"  "aoe"  "mba"  "mby"  "mbw"  "mbar" "mbak" "mma"  "mmaz"
##  [41] "mmj"  "mmac" "mvc"  "mek"  "mls"  "metm" "mef"  "meq"  "msj"  "msz" 
##  [51] "msw"  "mthr" "mthe" "mfz"  "fma"  "pmic" "phar" "piv"  "obj"  "coo" 
##  [61] "bhan" "faa"  "apr"  "pbq"  "rbp"  "erh"  "ers"  "eri"  "esr"  "esu" 
##  [71] "euu"  "dpn"  "mhg"  "ljf"  "lac"  "lad"  "laf"  "ldb"  "lbu"  "ldl" 
##  [81] "lhe"  "lhl"  "lhd"  "lke"  "law"  "lae"  "lje"  "lamy" "lkl"  "lapi"
##  [91] "lhs"  "lsn"  "bbk"  "bcd"  "baus" "banc" "bart" "bara" "barr" "baro"
## [101] "barj" "las"  "laa"  "lat"  "lso"  "lar"  "lau"  "ama"  "amf"  "amw" 
## [111] "amp"  "acn"  "aph"  "apy"  "apd"  "apha" "aoh"  "eru"  "erw"  "erg" 
## [121] "ecn"  "ech"  "echa" "echj" "echl" "echs" "echv" "echw" "echp" "emr" 
## [131] "ehh"  "nse"  "nri"  "nhm"  "nef"  "wol"  "wri"  "wen"  "wed"  "wpi" 
## [141] "wbm"  "woo"  "wcl"  "weo"  "wpp"  "ots"  "ott"  "ren"  "kci"  "kct" 
## [151] "kbl"  "kbt"  "kde"  "kga"  "kon"  "kso"  "cey"  "cea"  "cend" "buc" 
## [161] "bap"  "bau"  "baw"  "bajc" "bua"  "bup"  "bak"  "buh"  "bapf" "bapg"
## [171] "bapu" "bapw" "bas"  "bab"  "baph" "bci"  "bcib" "bcig" "hde"  "icp" 
## [181] "rip"  "rig"  "bfl"  "bpn"  "bva"  "bchr" "ben"  "bed"  "asy"  "hed" 
## [191] "ssz"  "hhs"  "den"  "ged"  "seny" "ehd"  "wbr"  "wgl"  "hdu"  "les" 
## [201] "cso"  "acl"  "apal" "rpr"  "rpo"  "rpw"  "rpz"  "rpg"  "rps"  "rpv" 
## [211] "rpq"  "rpl"  "rpn"  "rty"  "rtt"  "rtb"  "rcm"  "rcc"  "rbe"  "rbo" 
## [221] "rco"  "rfe"  "rak"  "rri"  "rrj"  "rra"  "rrc"  "rrh"  "rrb"  "rrn" 
## [231] "rrp"  "rrm"  "rrr"  "rms"  "rmi"  "rpk"  "raf"  "rhe"  "rja"  "rsv" 
## [241] "rsw"  "rph"  "rau"  "rmo"  "rpp"  "rre"  "ram"  "rab"  "rmc"  "ras" 
## [251] "ric"  "stai" "sapi" "smir" "smia" "scq"  "seri" "scou" "sprn" "spit"
## [261] "stab" "salx" "sgq"  "schi" "ctr"  "ctd"  "ctf"  "ctrd" "ctro" "ctrt"
## [271] "cta"  "cty"  "cra"  "ctrq" "ctrx" "ctrz" "ctrp" "ctlj" "ctlx" "ctll"
## [281] "ctb"  "ctrr" "ctlf" "ctli" "ctl"  "ctru" "ctrl" "ctrv" "ctrm" "ctla"
## [291] "ctlm" "ctls" "ctlz" "ctlc" "ctln" "ctlb" "ctlq" "cto"  "ctrn" "ctj"
```

```
## [301] "ctz"  "ctg"  "ctk"  "csw"  "ces"  "ctrb" "ctre" "ctrs" "ctec" "cfs"
## [311] "cfw"  "ctfw" "ctrf" "ctch" "ctn"  "ctq"  "ctv"  "ctw"  "ctrg" "ctri"
## [321] "ctra" "ctrh" "ctrj" "ctrk" "ctjt" "ctcf" "ctfs" "cthf" "ctcj" "cthj"
## [331] "ctmj" "cttj" "ctjs" "ctrc" "ctrw" "ctry" "ctct" "cmu"  "cmur" "cmn"
## [341] "cmm"  "cmg"  "cmx"  "cmz"  "cpn"  "cpa"  "cpj"  "cpt"  "clp"  "cpm"
## [351] "cpec" "cpeo" "cper" "chp"  "chb"  "chs"  "chi"  "cht"  "chc"  "chr"
## [361] "cpsc" "cpsn" "cpsb" "cpsg" "cpsm" "cpsi" "cpsv" "cpsw" "cpst" "cpsd"
## [371] "cpsa" "cav"  "cca"  "cab"  "cabo" "cfe"  "cgz"  "chla" "mpe"  "mcd"
## [381] "mans" "miw"  "mfr"  "mfm"  "mfp"  "mpul" "mani"
```

Organisms classified within cluster 5

```
##   [1] "afo"  "aym"  "toy"  "dtm"  "dit"  "diz"  "dpc"  "dlu"  "asd"  "gbr"
##  [11] "gpo"  "gor"  "goq"  "gta"  "goc"  "git"  "gru"  "gom"  "gav"  "god"
##  [21] "nfa"  "nfr"  "ncy"  "nbr"  "nno"  "nsl"  "nsr"  "ntp"  "noz"  "nod"
##  [31] "nah"  "nad"  "nwl"  "tpr"  "tsm"  "bsd"  "gob"  "mmar" "nml"  "nak"
##  [41] "kra"  "bfa"  "brx"  "brv"  "bgg"  "brz"  "bsau" "ars"  "aus"  "ica"
##  [51] "jte"  "jli"  "jme"  "agg"  "cmi"  "cms"  "ccap" "cart" "cry"  "frn"
##  [61] "hea"  "hum"  "huw"  "leif" "lse"  "leu"  "ltr"  "ldn"  "plap" "rtn"
##  [71] "rry"  "ria"  "rfs"  "rte"  "salc" "sala" "sald" "aai"  "gar"  "gcr"
##  [81] "glu"  "cef"  "cva"  "chn"  "cgy"  "chm"  "cmq"  "clw"  "csta" "cee"
##  [91] "cpre" "csur" "aau"  "pue"  "ach"  "apn"  "psul" "psni" "psey" "satk"
## [101] "agy"  "agm"  "agf"  "mts"  "mim"  "mio"  "mip"  "mcw"  "mpal" "mih"
## [111] "micr" "maur" "mhos" "mfol" "moo"  "mlv"  "mwa"  "mprt" "msed" "moy"
## [121] "ido"  "celh" "halt" "cet"  "cceu" "ver"  "fra"  "fre"  "fri"  "fal"
## [131] "fsy"  "plk"  "plab" "plat" "stp"  "saq"  "sna"  "ase"  "ams"  "actn"
## [141] "afs"  "acts" "nca"  "ndk"  "noy"  "noi"  "noo"  "ndp"  "nsn"  "nbe"
## [151] "nano" "nmes" "tes"  "ahm"  "acti" "acad" "ahg"  "acta" "apre" "ami"
## [161] "led"  "aey"  "pmad" "pdx"  "psea" "psee" "pseh" "pseq" "pecq" "phh"
## [171] "paut" "svi"  "sacc" "sen"  "sace" "sacg" "sesp" "ssyi" "amd"  "amn"
## [181] "amm"  "amz"  "aoi"  "aja"  "amq"  "amyc" "amyb" "aab"  "amyy" "aori"
## [191] "stri" "ksk"  "kab"  "kau"  "kit"  "krh"  "kpl"  "kfv"  "krs"  "nda"
## [201] "nal"  "ngv"  "strr" "tfu"  "noa"  "now"  "sro"  "actw" "tcu"  "cai"
```

```
## [211] "mau"  "mil"  "micb" "mtua" "mich" "mtem" "mcab" "msag" "vma"  "mcra"
## [221] "euz"  "rxy"  "rub"  "cwo"  "fln"  "sgn"  "cmr"  "camu" "est"  "rhoz"
## [231] "flm"  "fll"  "add"  "aswu" "hym"  "hye"  "hyg"  "hyp"  "hnv"  "hyh"
## [241] "hqi"  "pko"  "pact" "alm"  "run"  "spik" "spib" "aqb"  "aqa"  "aqd"
## [251] "aalg" "aue"  "cagg" "emar" "gfo"  "grl"  "grs"  "kos"  "kan"  "fbc"
## [261] "marm" "mart" "marb" "mgel" "mesq" "mrs"  "mlt"  "mut"  "myr"  "mpw"
## [271] "mod"  "myz"  "ndo"  "nom"  "nsd"  "noj"  "pom"  "poa"  "phal" "ptq"
## [281] "rbi"  "seon" "sze"  "lan"  "lvn"  "laci" "fop"  "taj"  "marf" "ahz"
## [291] "tdi"  "ten"  "tje"  "tmar" "tmp"  "lut"  "fek"  "asl"  "wfu"  "win"
## [301] "wij"  "psyn" "zpr"  "salt" "cnr"  "eao"  "emn"  "een"  "elb"  "emg"
## [311] "ego"  "egm"  "elz"  "elt"  "ebv"  "efal" "este" "fba"  "fbu"  "fbe"
## [321] "agd"  "mup"  "mgot" "muh"  "mgin" "mrub" "mgos" "pep"  "pgs"  "pej"
## [331] "ial"  "cpi"  "cbae" "chih" "rmr"  "rmg"  "sru"  "srm"  "rbar" "eff"
## [341] "aac"  "aad"  "bts"  "kyr"  "bly"  "blin" "bri"  "bcau" "far"  "gth"
## [351] "anx"  "asoc" "mtu"  "mtv"  "mtc"  "mra"  "mtf"  "mtb"  "mtk"  "mtz"
## [361] "mtg"  "mti"  "mte"  "mtur" "mtl"  "mto"  "mtd"  "mtn"  "mtj"  "mtub"
## [371] "mtuc" "mtue" "mtx"  "mtuh" "mtul" "mtut" "mtuu" "mtq"  "mbo"  "mbb"
## [381] "mbt"  "mbm"  "mbk"  "mbx"  "maf"  "mmic" "mce"  "mcq"  "mcv"  "mcx"
## [391] "mcz"  "mle"  "mlb"  "mpa"  "mao"  "mavi" "mavu" "mav"  "mit"  "mia"
## [401] "mid"  "myo"  "mchi" "mir"  "mmal" "mlp"  "msa"  "mul"  "mmc"  "mkm"
## [411] "mjl"  "mmi"  "mmae" "mmm"  "mli"  "mkn"  "myv"  "mye"  "mhad" "mdx"
## [421] "mshg" "mfj"  "mgro" "mxe"  "mnv"  "mpag" "mnm"  "mgor" "mcoo" "mste"
## [431] "lyg"  "pku"  "pmar" "pfae" "siv"  "ssil" "spor" "play" "pms"  "pmq"
## [441] "pmw"  "pnp"  "palb" "pbk"  "say"  "sap"  "sthr" "acr"  "amv"  "kba"
## [451] "goh"  "goy"  "gti"  "gbe"  "gbh"  "gbc"  "gbs"  "gdi"  "gdj"  "mlu"
## [461] "mick" "rgi"  "ros"  "rmuc" "nao"  "skt"  "ccs"  "cak"  "cse"  "cmb"
## [471] "pzu"  "bsb"  "brd"  "bne"  "brg"  "brl"  "bvc"  "bdm"  "brf"  "brev"
## [481] "bmed" "bvy"  "hba"  "hne"  "mmr"  "hbc"  "bop"  "bos"  "bvv"  "boi"
## [491] "bof"  "msc"  "mbry" "mros" "mhey" "mpar" "bid"  "moc"  "miv"  "mico"
## [501] "ddr"  "dpd"  "dez"  "dfc"  "dein" "dga"  "msl"  "mtun" "chel" "cdq"
## [511] "mtw"  "bbar" "deq"  "dei"  "dea"  "cbot" "phl"  "hdn"  "hdt"  "hmc"
## [521] "hni"  "lne"  "rbs"  "kai"  "atu"  "ara"  "ata"  "agr"  "atf"  "avi"
## [531] "agc"  "aro"  "agt"  "alf"  "aua"  "aala" "bapi" "mes"  "aak"  "amih"
## [541] "ngl"  "ngg"  "neo"  "nen"  "niy"  "shz"  "hoe"  "pht"  "rva"  "lap"
```

```
## [551] "lagg" "labr" "labp" "labt" "pphr" "psf"  "sme"  "smk"  "smq"  "smx" 
## [561] "smi"  "smeg" "smel" "smer" "smd"  "rhi"  "sfh"  "sfd"  "six"  "same"
## [571] "sino" "siw"  "ead"  "eah"  "esj"  "eak"  "emx"  "nwi"  "nha"  "oca" 
## [581] "ocg"  "oco"  "rhz"  "rpa"  "rpb"  "rpc"  "rpd"  "rpe"  "rpt"  "rpx" 
## [591] "anc"  "apra" "bvr"  "blag" "boo"  "aht"  "con"  "dsh"  "rmb"  "geh" 
## [601] "hml"  "jan"  "pde"  "pami" "pye"  "pcon" "pzh"  "paro" "paru" "pamn"
## [611] "pars" "parr" "pkd"  "ppan" "rha"  "rer"  "rey"  "reb"  "rop"  "roa" 
## [621] "req"  "rpy"  "rhb"  "rav"  "rfa"  "rhw"  "rhs"  "rrz"  "rhu"  "rqi" 
## [631] "rhq"  "rhod" "rrt"  "rby"  "rcr"  "rtm"  "palw" "pga"  "pgl"  "pgd" 
## [641] "php"  "ppic" "phq"  "sagu" "pseb" "red"  "ypac" "rmm"  "rok"  "rid" 
## [651] "rom"  "roh"  "sil"  "sit"  "rua"  "rut"  "oat"  "oar"  "otm"  "tgl" 
## [661] "thw"  "tec"  "rde"  "rli"  "rpon" "rsu"  "rhm"  "rhc"  "thaa" "ocd" 
## [671] "maru" "magq" "mag"  "mgy"  "mgry" "magx" "magn" "dex"  "dvn"  "rpm" 
## [681] "txi"  "thac" "tii"  "bme"  "bmel" "bmi"  "bmz"  "bmg"  "bmw"  "bmee"
## [691] "bmf"  "bmb"  "bmc"  "baa"  "babo" "babr" "babt" "babb" "babu" "babs"
## [701] "babc" "bms"  "bsi"  "bsf"  "bsui" "bsup" "bsuv" "bsuc" "bmt"  "bsz" 
## [711] "bsv"  "bsw"  "bsg"  "bov"  "bcs"  "bsk"  "bol"  "bcar" "bcas" "bmr" 
## [721] "bpp"  "bpv"  "bcet" "bcee" "bvl"  "bru"  "brj"  "oin"  "oah"  "ops" 
## [731] "bja"  "bju"  "bjp"  "bra"  "bbt"  "brs"  "aol"  "brc"  "brad" "bic" 
## [741] "bro"  "brk"  "bot"  "brq"  "bgq"  "bgz"  "bsym" "bbet" "barh" "bvz" 
## [751] "aay"  "aep"  "alb"  "alh"  "sjp"  "sch"  "ssy"  "syb"  "sbd"  "spmi"
## [761] "sphb" "sphr" "sinb" "spht" "shyd" "sya"  "sclo" "spyg" "sufl" "sami"
## [771] "sbar" "sal"  "sphk" "sphp" "smag" "smaz" "ster" "sgi"  "sphl" "sphq"
## [781] "spho" "sphx" "blas" "bfw"  "swi"  "sphd" "sphm" "stax" "sphi" "ssan"
## [791] "snj"  "smy"  "span" "skr"  "splm" "splk" "spkc" "sphc" "sphf" "spha"
## [801] "spau" "slut" "smic" "sphs" "eli"  "elq"  "erk"  "err"  "erf"  "emv" 
## [811] "pns"  "porl" "phz"  "pot"  "ntd"  "nar"  "npp"  "npn"  "nre"  "nov" 
## [821] "not"  "nor"  "ngf"  "nog"  "tmo"  "pla"  "rbm"  "shum" "svc"  "bba" 
## [831] "bbat" "bbw"  "bbac" "bex"  "bdq"  "bdc"  "bsed" "dov"  "dwd"  "dalk"
## [841] "dal"  "dat"  "dsf"  "des"  "deu"  "abai" "hoh"  "msd"  "mfb"  "cfus"
## [851] "sur"  "ccro" "llu"  "mrm"  "scl"  "scu"  "samy" "vin"  "gme"  "geo" 
## [861] "gbn"  "ade"  "acp"  "afw"  "ank"  "bsto" "thc"  "ret"  "rec"  "rel" 
## [871] "rep"  "rei"  "rle"  "rlt"  "rlg"  "rlb"  "rlu"  "rtr"  "rir"  "rpus"
## [881] "rhl"  "rga"  "rhn"  "rpha" "rhx"  "rhv"  "rhk"  "rez"  "rjg"  "rhr" 
```

```
##  [891] "rgr"  "rad"  "roy"  "rii"  "anb"  "acy"  "oce"  "ocm"  "opf"  "cate"
##  [901] "cao"  "cly"  "clh"  "cbal" "cbat" "psy"  "fes"  "suam" "spse" "sulz"
##  [911] "suli" "suld" "spot" "cps"  "cov"  "nde"  "nmv"  "nio"  "nja"  "tig"
##  [921] "blep" "aeh"  "lhk"  "simp" "aka"  "amim" "oan"  "och"  "rsp"  "rsh"
##  [931] "rsq"  "rsk"  "rcp"  "rhp"  "rbl"  "aon"  "bpe"  "bpc"  "bper" "bpet"
##  [941] "bpeu" "bpar" "bpa"  "bbh"  "bbr"  "bbm"  "bbx"  "bpt"  "bav"  "bho"
##  [951] "bhm"  "bhz"  "bbro" "bfz"  "bpdz" "boh"  "bgm"  "boj"  "bxe"  "bxb"
##  [961] "bph"  "bge"  "bpx"  "bpy"  "buz"  "bfn"  "bcai" "pspw" "para" "parb"
##  [971] "phs"  "pter" "pgp"  "pcj"  "pts"  "pcaf" "pmeg" "caba" "buo"  "cdn"
##  [981] "dac"  "del"  "dts"  "dhk"  "dla"  "dpy"  "dih"  "daer" "drg"  "adn"
##  [991] "adk"  "mms"  "jaz"  "jal"  "lim"  "lih"  "mnr"  "masw" "mass" "mali"
## [1001] "mum"  "miu"  "hse"  "hsz"  "hht"  "hrb"  "hee"  "hhf"  "hfr"  "ppk"
## [1011] "ppno" "ppnm" "prb"  "ppul" "pspu" "papi" "pve"  "pox"  "ptx"  "pfg"
## [1021] "pnr"  "pand" "pfib" "mpt"  "metp" "pnu"  "pne"  "pdq"  "poh"  "har"
## [1031] "put"  "pus"  "pud"  "afa"  "afq"  "aaqu" "rso"  "rsc"  "rsl"  "rsn"
## [1041] "rsm"  "rse"  "rsy"  "rpi"  "rpf"  "rpj"  "rmn"  "rin"  "rpu"  "art"
## [1051] "arr"  "arm"  "arl"  "are"  "aaq"  "arw"  "arh"  "ary"  "arz"  "aru"
## [1061] "arq"  "arn"  "arx"  "acry" "arth" "artp" "acit" "rta"  "rfr"  "rsb"
## [1071] "rac"  "rhy"  "rhf"  "rhg"  "rdp"  "rge"  "rbn"  "sthm" "tin"  "thi"
## [1081] "pol"  "pna"  "pos"  "poo"  "aav"  "ajs"  "dia"  "aaa"  "ack"  "acra"
## [1091] "acid" "acip" "acin" "acis" "acio" "amon" "ctt"  "ctes" "cke"  "cser"
## [1101] "cof"  "hyr"  "hyb"  "hyl"  "hyc"  "hpse" "hyn"  "lch"  "upi"  "vap"
## [1111] "vpe"  "vpd"  "vaa"  "vbo"  "vam"  "chro" "pse"  "aql"  "htl"  "meu"
## [1121] "meh"  "mei"  "mep"  "cste" "nci"  "neu"  "nco"  "nmu"  "azo"  "aoa"
## [1131] "aza"  "azi"  "acom" "azd"  "azr"  "azq"  "doe"  "sht"  "sphn" "smiz"
## [1141] "spsc" "sphz" "spdr" "sdj"  "stha" "metr" "thu"  "thk"  "tak"  "reh"
## [1151] "cnc"  "cuh"  "reu"  "rme"  "cti"  "cbw"  "cgd"  "ccup" "cup"  "cuu"
## [1161] "cpau" "cox"  "rbh"  "mthd" "mict" "spoi" "mpur" "ttc"  "thip" "alg"
## [1171] "asip" "aprs" "ebs"  "acav" "aace" "aper" "ato"  "aot"  "gxy"  "kna"
## [1181] "ksc"  "kre"  "lpop" "kgo"  "fpc"  "fpy"  "fpo"  "fpq"  "fpv"  "fpw"
## [1191] "fpk"  "fpsz" "fjo"  "fjg"  "fbr"  "fco"  "fin"  "fgl"  "ffa"  "fat"
## [1201] "fki"  "fpal" "fmg"  "falb" "fcr"  "fse"  "fsn"  "fnk"  "fak"  "haz"
## [1211] "gbi"  "tgr"  "tni"  "tti"  "mmob" "mca"  "metu" "mmai" "mein" "mdn"
## [1221] "mdh"  "mko"  "metl" "mej"  "mec"  "adi"  "apac" "axe"  "ilo"  "ipi"
```

```
## [1231] "kus"  "kma"  "kuy"  "csa"  "haa"  "hcs"  "hak"  "ham"  "hco"  "halo"
## [1241] "hhh"  "hag"  "hol"  "hsr"  "hmd"  "htt"  "hcam" "hpiz" "mlo"  "mln"
## [1251] "mci"  "mop"  "mam"  "mamo" "meso" "mesw" "mesm" "mesp" "mhua" "mjr"
## [1261] "merd" "kko"  "kge"  "ksd"  "kpd"  "axy"  "axo"  "axn"  "axx"  "adt"
## [1271] "ais"  "asw"  "achr" "achb" "mars" "nik"  "ncu"  "ome"  "mmw"  "mme"
## [1281] "mpc"  "mpri" "gsn"  "tol"  "tor"  "rfo"  "azl"  "ali"  "abs"  "abq"
## [1291] "abf"  "ati"  "ahu"  "azt"  "azm"  "azz"  "aoz"  "gan"  "lpn"  "lph"
## [1301] "lpo"  "lpu"  "lpm"  "lpf"  "lpp"  "lpc"  "lpa"  "lpe"  "llo"  "lfa"
## [1311] "lha"  "lok"  "lsh"  "lib"  "lgt"  "lcj"  "lwa"  "lss"  "psal" "mct"
## [1321] "mcs"  "mcat" "moi"  "mos"  "mbl"  "mboi" "mcun" "par"  "prw"  "pur"
## [1331] "pali" "pspg" "psyg" "psya" "psyy" "psyp" "plu"  "plum" "xpo"  "acb"
## [1341] "abm"  "aby"  "abc"  "abn"  "abb"  "abx"  "abz"  "abr"  "abd"  "abad"
## [1351] "abj"  "abab" "abaj" "abk"  "abau" "abaa" "abw"  "abal" "acc"  "alc"
## [1361] "acal" "acd"  "att"  "aei"  "ajo"  "acw"  "ahl"  "asj"  "aid"  "adv"
## [1371] "arj"  "awu"  "acum" "agu"  "aug"  "alw"  "ads"  "aber" "achi" "mad"
## [1381] "msr"  "mpq"  "mari" "mlq"  "msq"  "marj" "bma"  "bmv"  "bml"  "bmn"
## [1391] "bmal" "bmae" "bmaq" "bmai" "bmaf" "bmaz" "bmab" "bps"  "bpm"  "bpl"
## [1401] "bpd"  "bpr"  "bpse" "bpsm" "bpsu" "bpsd" "bpz"  "bpq"  "bpk"  "bpsh"
## [1411] "bpsa" "bpso" "but"  "bte"  "btq"  "btj"  "btz"  "btd"  "btv"  "bthe"
## [1421] "bthm" "btha" "bthl" "bok"  "boc"  "buu"  "bvi"  "bve"  "bur"  "bcn"
## [1431] "bch"  "bcm"  "bcj"  "bcen" "bcew" "bceo" "bam"  "bac"  "bmj"  "bmu"
## [1441] "bmk"  "bmul" "bct"  "bced" "bcep" "bdl"  "bpyr" "bcon" "bub"  "bdf"
## [1451] "blat" "btei" "bsem" "bpsl" "bmec" "bstg" "bstl" "bgl"  "bgu"  "bug"
## [1461] "bgf"  "bgd"  "bgo"  "byi"  "buk"  "bue"  "bul"  "buq"  "bgp"  "bpla"
## [1471] "bud"  "bum"  "bui"  "mrd"  "met"  "mno"  "mor"  "meta" "maqu" "mphy"
## [1481] "mee"  "metd" "metx" "mets" "meti" "mmes" "mtea" "bca"  "bcg"  "bti"
## [1491] "bmyc" "baci" "baco" "bhk"  "bmeg" "bon"  "cum"  "cub"  "chz"  "cgn"
## [1501] "cih"  "chh"  "cio"  "chry" "cpip" "chrs" "chrz" "carh" "csha" "cnk"
## [1511] "cjt"  "cil"  "ccau" "cben" "ccas" "clac" "ctak" "saln" "sok"  "rbz"
## [1521] "thas" "faq"  "hdh"  "sdf"  "this" "pgb"  "vch"  "vcf"  "vcs"  "vce"
## [1531] "vcq"  "vcj"  "vci"  "vco"  "vcr"  "vcm"  "vcl"  "vcz"  "vct"  "vbr"
## [1541] "vsc"  "vaq"  "vsr"  "scot" "pha"  "ptn"  "prr"  "png"  "psen" "rgl"
## [1551] "dko"  "lue"  "avn"  "avl"  "avd"  "acx"  "paer" "pmy"  "pre"  "ppse"
## [1561] "pcq"  "ppu"  "ppf"  "ppg"  "ppw"  "ppt"  "ppi"  "ppx"  "ppuh" "pput"
```

```
## [1571] "ppun" "ppud" "pmon" "pmot" "pmos" "ppj"  "psb"  "psyr" "psp"  "pamg"
## [1581] "pavl" "pvd"  "pfl"  "pprc" "ppro" "pfe"  "pfc"  "pfn"  "ppz"  "pcg"
## [1591] "pvr"  "pfx"  "pen"  "psc"  "psh"  "pstt" "pbm"  "plul" "pba"  "pbc"
## [1601] "ppuu" "pdr"  "psv"  "psk"  "pkc"  "pch"  "pcz"  "pcp"  "plq"  "palk"
## [1611] "prh"  "pses" "psem" "psec" "psos" "pkr"  "pfk"  "ppsl" "pset" "psil"
## [1621] "pym"  "psed" "pke"  "pall" "pum"  "poj"  "ppsh" "pgy"  "xal"  "chu"
```

Pyramid match (PM) kernel

Heatmap

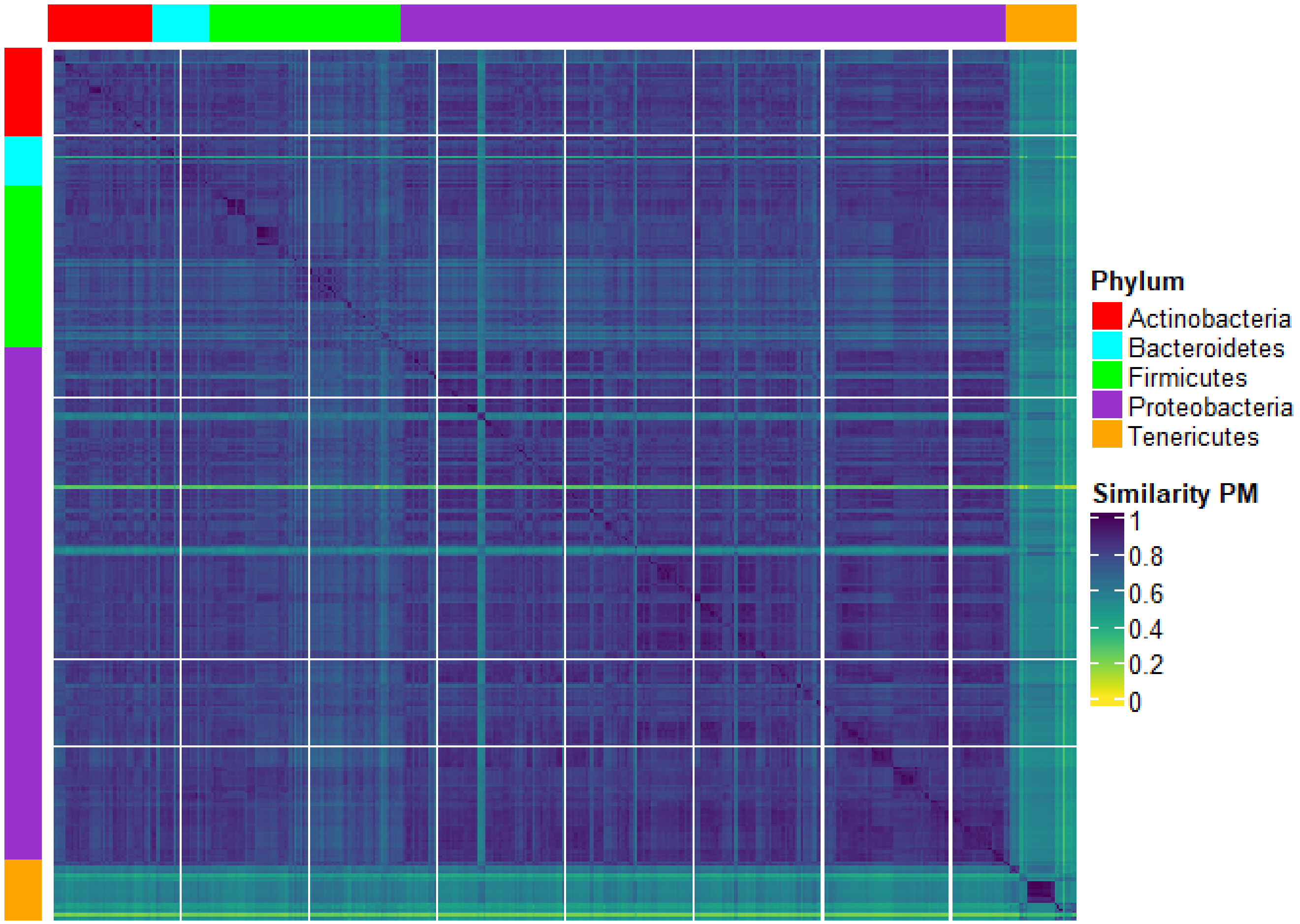
Phylum
Actinobacteria
Bacteroidetes
Firmicutes
Proteobacteria
Tenericutes
Similarity PM
1
0.8
0.6
0.4
0.2
0

MDS for PM

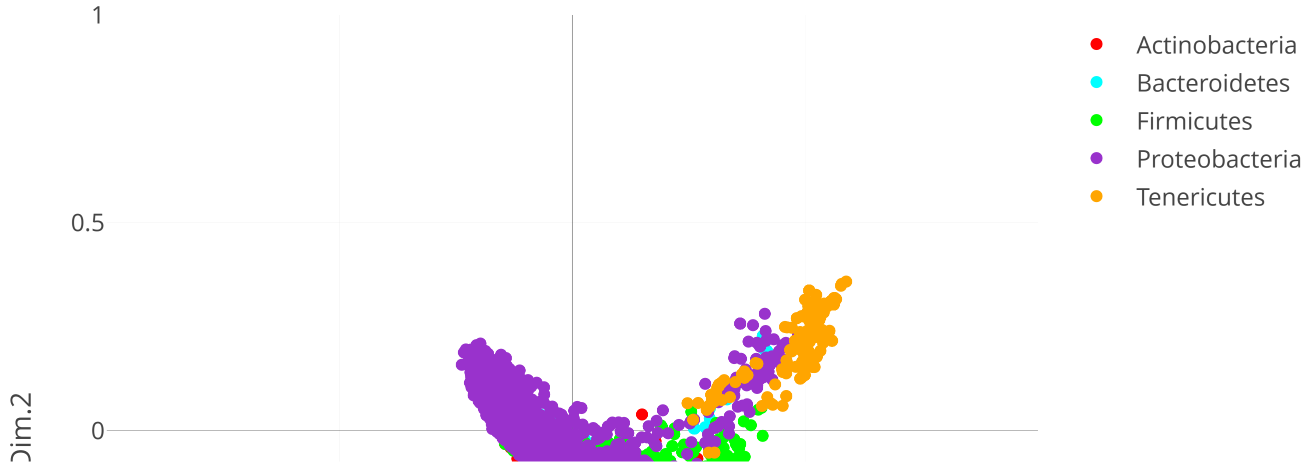
1
0.5
0
Dim.2
Actinobacteria
Bacteroidetes
Firmicutes
Proteobacteria
Tenericutes

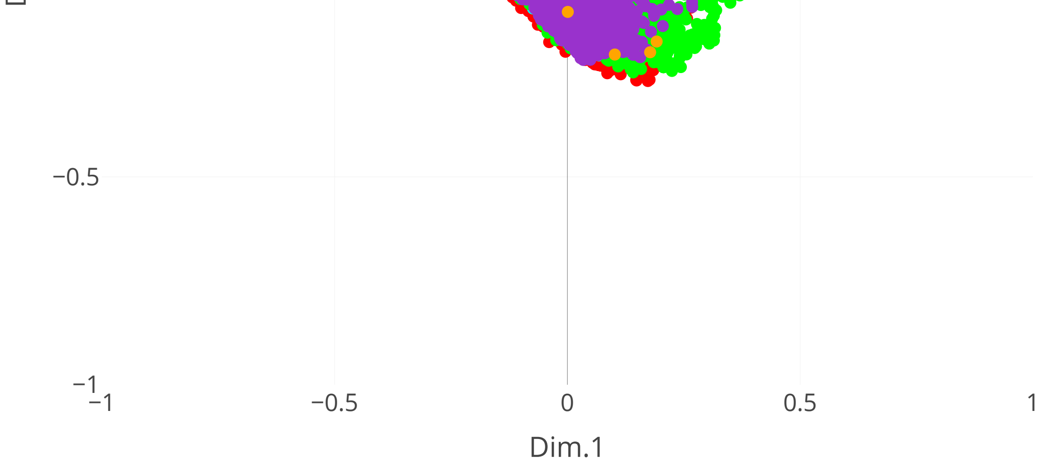

5-means clustering for PM

```
##                  Cluster
## Real group          1    2    3    4    5
##   Actinobacteria    1  230  156    2   82
##   Bacteroidetes    12  114   89    9   36
##   Firmicutes        0  115  380   21  353
##   Proteobacteria   31 1876  638   90  113
##   Tenericutes     127    0    2  191    4
```

Organisms classified within cluster 1

```
##   [1] "nfe"   "che"   "cec"   "cher" "smg"   "sms"   "smh"   "sum"   "smv"   "smub"
##  [11] "smum" "smue" "smup" "hci"   "hct"   "hcc"   "hcd"   "ndl"   "tpn"   "tpq"
##  [21] "tpj"   "vfg"   "zin"   "men"   "meo"   "aen"   "cmik" "crp"   "cru"   "crc"
##  [31] "crt"   "crh"   "crv"   "cri"   "ple"   "ply"   "plr"   "plo"   "pld"   "plb"
##  [41] "plc"   "pli"   "paly" "baab" "elj"   "esx"   "efr"   "eml"   "mfl"   "mfw"
##  [51] "mchc" "mlac" "ment" "msyr" "mtab" "mcol" "sdi"   "stai" "skn"   "sphh"
##  [61] "smoo" "pml"   "pal"   "psol" "pzi"   "mge"   "mgu"   "mgc"   "mgq"   "mgx"
##  [71] "mpn"   "mpm"   "mpj"   "mpb"   "mga"   "mgh"   "mgf"   "mgn"   "mgs"   "mgt"
##  [81] "mgv"   "mgw"   "mgac" "mgan" "mgnc" "mgz"   "mmy"   "mmym" "mmyi" "mml"
##  [91] "mcp"   "mcac" "mcap" "mcar" "mcai" "mlc"   "mlh"   "mmo"   "mhy"   "mhj"
```

```
## [101] "mhp"   "mhn"   "mhyl"  "mhyo"  "mat"   "mco"   "mho"   "mhom"  "mcd"   "mhr"
## [111] "mhh"   "mhm"   "mhs"   "mhv"   "mha"   "mhf"   "mss"   "msk"   "mpf"   "mput"
## [121] "mhe"   "mwe"   "mhl"   "mhb"   "mpv"   "mov"   "mbc"   "mgj"   "mfq"   "mcan"
## [131] "myt"   "mds"   "myg"   "mpho"  "mhyv"  "mclo"  "mamp"  "mans"  "mphc"  "mane"
## [141] "mnh"   "mnu"   "mstr"  "mgly"  "mcou"  "mcom"  "mpu"   "msy"   "mso"   "maa"
## [151] "mal"   "mfr"   "mfm"   "mfp"   "mbv"   "mbh"   "mbi"   "mbq"   "mcy"   "mcas"
## [161] "mck"   "marg"  "mpul"  "mbov"  "mboh"  "mani"  "mphi"  "uur"   "upa"   "upr"
## [171] "uue"
```

Organisms classified within cluster 2

```
##   [1] "aym"  "toy"  "dtm"  "dit"  "diz"  "dpc"  "dlu"  "asd"  "gbr"  "gpo"
##  [11] "gor"  "goq"  "gta"  "goc"  "git"  "gru"  "gom"  "gav"  "god"  "nfa"
##  [21] "nfr"  "ncy"  "nbr"  "nno"  "nsl"  "nsr"  "ntp"  "noz"  "nod"  "nah"
##  [31] "nad"  "nwl"  "tpr"  "tsm"  "bsd"  "gob"  "mmar" "nml"  "kra"  "brx"
##  [41] "bgg"  "brz"  "bsau" "ars"  "dni"  "day"  "aus"  "ica"  "jte"  "jli"
##  [51] "jme"  "agg"  "cmc"  "cart" "hum"  "huw"  "lxy"  "leif" "lse"  "leu"
##  [61] "ltr"  "ldn"  "mvd"  "plap" "rry"  "ria"  "rfs"  "rte"  "salc" "sala"
##  [71] "sald" "aai"  "gar"  "gcr"  "glu"  "cgl"  "cgb"  "cgu"  "cgt"  "cgs"
##  [81] "cgg"  "cgm"  "cgj"  "cgq"  "cef"  "cjk"  "cva"  "chn"  "cmd"  "ccg"
##  [91] "cgy"  "chm"  "cmq"  "cmv"  "cted" "clw"  "cdx"  "csta" "ccjz" "camg"
## [101] "cee"  "cpre" "csur" "aau"  "pue"  "ach"  "apn"  "psul" "psni" "psey"
## [111] "satk" "agy"  "agm"  "agf"  "mts"  "mim"  "mio"  "mip"  "mcw"  "mpal"
## [121] "mih"  "micr" "maur" "mhos" "mfol" "moo"  "mlv"  "mwa"  "mprt" "msed"
## [131] "moy"  "ido"  "halt" "cet"  "cceu" "ver"  "fre"  "fri"  "fal"  "plk"
## [141] "plab" "plat" "stp"  "saq"  "sna"  "ase"  "ams"  "actn" "afs"  "acts"
## [151] "nca"  "ndk"  "noy"  "noi"  "noo"  "ndp"  "nsn"  "nbe"  "nano" "nmes"
## [161] "ahg"  "acta" "apre" "ami"  "led"  "aey"  "pmad" "pdx"  "psea" "psee"
## [171] "pseh" "pseq" "pecq" "phh"  "paut" "svi"  "sacc" "sen"  "sace" "sacg"
## [181] "sesp" "ssyi" "amd"  "amn"  "amm"  "amz"  "aoi"  "aja"  "amq"  "amyc"
## [191] "amyb" "aab"  "amyy" "aori" "stri" "ksk"  "kau"  "kit"  "krh"  "kpl"
## [201] "kfv"  "krs"  "kod"  "nda"  "nal"  "ngv"  "strr" "tfu"  "noa"  "now"
## [211] "sro"  "actw" "tcu"  "cai"  "mau"  "mil"  "micb" "mtua" "mich" "mtem"
```

```
## [221] "mcab" "msag" "vma"  "mcra" "erz"  "euz"  "rxy"  "rrd"  "rub"  "cwo" 
## [231] "fln"  "nso"  "nia"  "nko"  "fla"  "fgg"  "hhy"  "bbd"  "cmr"  "camu"
## [241] "evi"  "est"  "echi" "flm"  "fll"  "mtt"  "add"  "hsw"  "hym"  "hyd" 
## [251] "hye"  "hyg"  "hyp"  "hyz"  "hnv"  "hyh"  "hyj"  "hqi"  "hrs"  "pko" 
## [261] "pact" "ruf"  "rti"  "rud"  "alm"  "fbt"  "eol"  "lby"  "rsi"  "run" 
## [271] "rup"  "sli"  "srd"  "smon" "spir" "spik" "spib" "dfe"  "aqa"  "aqd" 
## [281] "aalg" "gfo"  "gfl"  "grs"  "kos"  "kan"  "fbc"  "marm" "mart" "marb"
## [291] "mare" "mrs"  "mut"  "ndo"  "nob"  "pom"  "pob"  "prn"  "pola" "poa" 
## [301] "phal" "ptq"  "seon" "sze"  "lan"  "lvn"  "fop"  "taj"  "marf" "ahz" 
## [311] "ten"  "tje"  "tmp"  "win"  "psyn" "zpr"  "salt" "fbe"  "agd"  "mup" 
## [321] "muc"  "mgot" "muh"  "mgin" "mgk"  "mrub" "mgos" "oli"  "scn"  "phe" 
## [331] "pep"  "pcm"  "psty" "pgs"  "pek"  "proe" "sbx"  "cpi"  "cbae" "chit"
## [341] "chih" "rmr"  "rmg"  "srm"  "eff"  "aac"  "aad"  "bts"  "kyr"  "bfv" 
## [351] "bly"  "blin" "bri"  "blut" "bcau" "pof"  "gtk"  "gtm"  "gmc"  "ggh" 
## [361] "gjf"  "anx"  "asoc" "mtu"  "mtv"  "mtc"  "mra"  "mtf"  "mtb"  "mtk" 
## [371] "mtz"  "mtg"  "mti"  "mte"  "mtur" "mtl"  "mto"  "mtd"  "mtn"  "mtj" 
## [381] "mtub" "mtue" "mtx"  "mtuh" "mtul" "mtut" "mtuu" "mtq"  "mbo"  "mbb" 
## [391] "mbt"  "mbm"  "mbk"  "mbx"  "maf"  "mmic" "mce"  "mcq"  "mcv"  "mcx" 
## [401] "mcz"  "mle"  "mlb"  "mpa"  "mao"  "mavi" "mavu" "mav"  "mit"  "mia" 
## [411] "mid"  "myo"  "mchi" "mir"  "mmal" "mlp"  "msa"  "mul"  "mmc"  "mkm" 
## [421] "mjl"  "mmi"  "mmae" "mmm"  "mli"  "mkn"  "myv"  "mye"  "mhad" "mdx" 
## [431] "mshg" "mfj"  "mgro" "mxe"  "mnv"  "mpag" "mnm"  "mgor" "mcoo" "mste"
## [441] "bbe"  "blr"  "bfm"  "brw"  "lyb"  "lyg"  "pmar" "ssil" "vig"  "vil" 
## [451] "pms"  "pmq"  "pmw"  "pnp"  "palb" "prz"  "plyc" "say"  "sap"  "acr" 
## [461] "amv"  "gbe"  "gbh"  "gbc"  "gbs"  "gdi"  "mlu"  "mick" "rgi"  "ros" 
## [471] "rmuc" "nao"  "skt"  "aex"  "ccr"  "ccs"  "cak"  "cse"  "chq"  "cmb" 
## [481] "cfh"  "cauf" "pzu"  "phb"  "bsb"  "brd"  "bne"  "brg"  "brl"  "bvc" 
## [491] "bdm"  "brf"  "brev" "bmed" "bvy"  "hba"  "hne"  "mmr"  "hbc"  "bop" 
## [501] "bos"  "bvv"  "boi"  "bof"  "msc"  "mbry" "mros" "mhey" "mpar" "bid" 
## [511] "moc"  "miv"  "mico" "dmr"  "dpd"  "dsw"  "dez"  "dein" "msl"  "mtun"
## [521] "chel" "cdq"  "mtw"  "bbar" "deq"  "dei"  "dea"  "cbot" "phl"  "hdn" 
## [531] "hdt"  "hmc"  "hni"  "lne"  "rbs"  "kai"  "atu"  "ara"  "ata"  "agr" 
## [541] "atf"  "avi"  "agc"  "aro"  "agt"  "alf"  "aua"  "aala" "bapi" "mes" 
## [551] "aak"  "amih" "ngl"  "ngg"  "neo"  "nen"  "niy"  "shz"  "hoe"  "pht" 
```

```
## [561] "rva"  "lap"  "lagg" "labr" "labp" "labt" "pphr" "psf"  "sme"  "smk" 
## [571] "smq"  "smx"  "smi"  "smeg" "smel" "smer" "smd"  "rhi"  "sfh"  "sfd" 
## [581] "six"  "same" "sino" "siw"  "ead"  "eah"  "esj"  "eak"  "emx"  "nha" 
## [591] "oca"  "ocg"  "oco"  "rhz"  "rpa"  "rpb"  "rpc"  "rpd"  "rpe"  "rpt" 
## [601] "rpx"  "anc"  "apra" "bvr"  "blag" "boo"  "aht"  "con"  "dsh"  "rmb" 
## [611] "geh"  "hml"  "jan"  "pde"  "pami" "pye"  "pzh"  "paro" "paru" "pamn"
## [621] "pars" "parr" "pkd"  "ppan" "rha"  "rer"  "rey"  "reb"  "rop"  "roa" 
## [631] "req"  "rpy"  "rhb"  "rav"  "rfa"  "rhw"  "rhs"  "rrz"  "rhu"  "rqi" 
## [641] "rhq"  "rhod" "rrt"  "rby"  "rcr"  "rtm"  "palw" "pga"  "pgl"  "pgd" 
## [651] "php"  "ppic" "phq"  "sagu" "pseb" "red"  "ypac" "tpro" "rmm"  "rok" 
## [661] "rid"  "rom"  "roh"  "sil"  "sit"  "rua"  "rut"  "oat"  "oar"  "otm" 
## [671] "tgl"  "thw"  "tec"  "rde"  "rli"  "rpon" "rsu"  "rhm"  "rhc"  "thaa"
## [681] "ocd"  "maru" "magq" "mag"  "mgy"  "mgry" "magx" "magn" "dvn"  "rpm" 
## [691] "txi"  "thac" "tii"  "bme"  "bmel" "bmi"  "bmz"  "bmg"  "bmw"  "bmee"
## [701] "bmf"  "bmb"  "bmc"  "baa"  "babo" "babr" "babt" "babb" "babu" "babs"
## [711] "babc" "bms"  "bsi"  "bsf"  "bsui" "bsup" "bsuv" "bsuc" "bmt"  "bsz" 
## [721] "bsv"  "bsw"  "bsg"  "bov"  "bcs"  "bsk"  "bol"  "bcar" "bcas" "bmr" 
## [731] "bpp"  "bpv"  "bcet" "bcee" "bvl"  "bru"  "brj"  "oin"  "oah"  "ops" 
## [741] "bja"  "bju"  "bjp"  "bra"  "bbt"  "brs"  "aol"  "brc"  "brad" "bic" 
## [751] "bro"  "brk"  "bot"  "brq"  "bgq"  "bgz"  "bsym" "bbet" "barh" "bvz" 
## [761] "aay"  "aep"  "alb"  "alh"  "sjp"  "sch"  "ssy"  "syb"  "sbd"  "spmi"
## [771] "sphb" "sphr" "sinb" "spht" "shyd" "sya"  "sclo" "spyg" "sufl" "sami"
## [781] "sbar" "sal"  "sphk" "sphp" "smag" "smaz" "ster" "sgi"  "sphl" "sphq"
## [791] "spho" "sphx" "blas" "bfw"  "swi"  "sphd" "sphm" "stax" "sphi" "ssan"
## [801] "snj"  "smy"  "span" "skr"  "splm" "splk" "spkc" "sphc" "sphf" "spha"
## [811] "spau" "sech" "slut" "smic" "sphs" "eli"  "elq"  "erk"  "err"  "erf" 
## [821] "emv"  "pns"  "porl" "phz"  "pot"  "ntd"  "nar"  "npp"  "npn"  "nre" 
## [831] "nov"  "not"  "nor"  "ngf"  "nog"  "tmo"  "pla"  "rbm"  "shum" "svc" 
## [841] "bdc"  "bdz"  "dov"  "dwd"  "dalk" "dal"  "dat"  "des"  "deu"  "abai"
## [851] "hoh"  "ccx"  "mfu"  "mmas" "mxa"  "msd"  "mym"  "mfb"  "cfus" "sur" 
## [861] "ccro" "llu"  "mrm"  "scl"  "scu"  "samy" "vin"  "afw"  "dti"  "ret" 
## [871] "rec"  "rel"  "rep"  "rei"  "rle"  "rlt"  "rlg"  "rlb"  "rlu"  "rtr" 
## [881] "rir"  "rpus" "rhl"  "rga"  "rhn"  "rpha" "rhx"  "rhv"  "rhk"  "rez" 
## [891] "rjg"  "rhr"  "rgr"  "rad"  "roy"  "rii"  "oce"  "opf"  "zdf"  "cate"
```

```
##  [901] "cao"  "cly"  "clh"  "cbal" "cbat" "gag"  "gni"  "rhh"  "salm" "salk"
##  [911] "lsd"  "mvs"  "mya"  "mmaa" "pin"  "fbl"  "fes"  "suam" "spse" "sulz"
##  [921] "suli" "suld" "spot" "cps"  "com"  "coz"  "colw" "cola" "cber" "cov"
##  [931] "nde"  "nio"  "blep" "lhk"  "beb"  "beba" "simp" "aka"  "amim" "oan"
##  [941] "och"  "rsq"  "rsk"  "rbl"  "aon"  "bpe"  "bpc"  "bper" "bpet" "bpeu"
##  [951] "bpar" "bpa"  "bbh"  "bbr"  "bbm"  "bbx"  "bpt"  "bav"  "bho"  "bhm"
##  [961] "bhz"  "btrm" "bbro" "bfz"  "bpdz" "boh"  "bgm"  "boj"  "bxe"  "bxb"
##  [971] "bph"  "bge"  "bpx"  "bpy"  "buz"  "bfn"  "bcai" "pspw" "para" "parb"
##  [981] "phs"  "pter" "pgp"  "pcj"  "pts"  "pcaf" "pmeg" "caba" "buo"  "cdn"
##  [991] "dac"  "del"  "dts"  "dhk"  "dla"  "dpy"  "dih"  "daer" "drg"  "dug"
## [1001] "adn"  "adk"  "mms"  "jag"  "jab"  "jaz"  "jal"  "jsv"  "jaj"  "jas"
## [1011] "jlv"  "limn" "lim"  "lih"  "mnr"  "masw" "mass" "masz" "mtim" "masy"
## [1021] "mali" "mum"  "mfla" "mpli" "miu"  "hse"  "hsz"  "hht"  "hrb"  "hee"
## [1031] "hhf"  "hfr"  "nok"  "ppk"  "ppno" "ppnm" "prb"  "ppul" "pspu" "papi"
## [1041] "pve"  "pox"  "ptx"  "pfg"  "pnr"  "pand" "pfib" "pkt"  "mpt"  "metp"
## [1051] "pnu"  "pdq"  "har"  "put"  "pus"  "pud"  "afa"  "afq"  "aaqu" "rso"
## [1061] "rsc"  "rsl"  "rsn"  "rsm"  "rse"  "rsy"  "rpi"  "rpf"  "rpj"  "rmn"
## [1071] "rin"  "rpu"  "art"  "arr"  "arm"  "arl"  "are"  "aaq"  "arw"  "arh"
## [1081] "ary"  "arz"  "aru"  "arq"  "arn"  "arx"  "acry" "arth" "artp" "rta"
## [1091] "rfr"  "rsb"  "rac"  "rhy"  "rhf"  "rhg"  "rdp"  "rge"  "rbn"  "sthm"
## [1101] "tin"  "thi"  "pol"  "pna"  "pos"  "poo"  "aav"  "ajs"  "dia"  "aaa"
## [1111] "ack"  "acra" "acid" "acip" "acin" "acis" "acio" "amon" "ctt"  "ctes"
## [1121] "cke"  "cser" "cof"  "hyr"  "hyb"  "hyl"  "hyc"  "hpse" "hyn"  "lch"
## [1131] "upv"  "upi"  "vap"  "vpe"  "vpd"  "vaa"  "vbo"  "vam"  "cfon" "cari"
## [1141] "amah" "aqs"  "cvi"  "cvc"  "chro" "chri" "chrb" "crz"  "chrm" "chae"
## [1151] "pse"  "aql"  "htl"  "nci"  "azo"  "aoa"  "aza"  "azi"  "atw"  "acom"
## [1161] "azd"  "azr"  "azq"  "dsu"  "app"  "shg"  "sht"  "smiz" "spsc" "sphz"
## [1171] "sphe" "spdr" "sdj"  "stha" "metr" "tmz"  "thu"  "tcl"  "thk"  "tak"
## [1181] "shd"  "dar"  "dey"  "reh"  "cnc"  "cuh"  "reu"  "rme"  "cti"  "cbw"
## [1191] "cgd"  "ccup" "cup"  "cuu"  "cpau" "cox"  "rbh"  "ttu"  "mthd" "micc"
## [1201] "maga" "mii"  "mict" "mhyd" "cja"  "ceb"  "cell" "cek"  "ceg"  "spoi"
## [1211] "osg"  "alg"  "aprs" "buf"  "bage" "cnt"  "cem"  "cen"  "clap" "sfo"
## [1221] "ahn"  "pfq"  "aha"  "ahy"  "ahd"  "ahr"  "ahp"  "ahj"  "ahh"  "ahi"
## [1231] "aaj"  "asa"  "aeo"  "avr"  "avo"  "amed" "asr"  "adh"  "acav" "aem"
```

```
## [1241] "aea"  "arv"  "aes"  "ael"  "ato"  "eco"  "ecj"  "ecd"  "ebw"  "ecok"
## [1251] "ece"  "ecs"  "ecf"  "etw"  "elx"  "eoi"  "eoj"  "eoh"  "ecoo" "ecoh"
## [1261] "esl"  "eso"  "esm"  "eck"  "ecg"  "eok"  "elr"  "elh"  "ecw"  "eun"
## [1271] "ecp"  "ena"  "ecos" "ecv"  "ecoa" "ecx"  "ecm"  "ecy"  "ecr"  "ecq"
## [1281] "eum"  "ect"  "eoc"  "ebr"  "ebl"  "ebe"  "ebd"  "eci"  "eih"  "ecz"
## [1291] "ecc"  "elo"  "eln"  "ese"  "ecl"  "eko"  "ekf"  "eab"  "edh"  "edj"
## [1301] "elu"  "elw"  "ell"  "elc"  "eld"  "elp"  "elf"  "ecol" "ecoi" "ecoj"
## [1311] "efe"  "eal"  "ema"  "esz"  "sfl"  "sfx"  "sfv"  "sfe"  "sfn"  "sfs"
## [1321] "sft"  "ssn"  "sbo"  "sbc"  "sdy"  "sdz"  "shq"  "esa"  "csk"  "csz"
## [1331] "csj"  "ccon" "cdm"  "csi"  "cmj"  "cui"  "cmw"  "ctu"  "ksa"  "kor"
## [1341] "krd"  "kco"  "kot"  "kpse" "ksc"  "gqu"  "hav"  "hpar" "opo"  "ddd"
## [1351] "dda"  "dze"  "ddc"  "dzc"  "dso"  "ced"  "dfn"  "ddq"  "daq"  "dic"
## [1361] "lax"  "lei"  "leh"  "lee"  "ler"  "lea"  "laz"  "lef"  "lni"  "lew"
## [1371] "bgj"  "brb"  "bng"  "kie"  "kas"  "psts" "pshi" "pge"  "psi"  "psx"
## [1381] "psta" "prg"  "pala" "phei" "prq"  "prj"  "pvc"  "sty"  "stt"  "sex"
## [1391] "sent" "stm"  "seo"  "sev"  "sey"  "sem"  "sej"  "seb"  "sef"  "setu"
## [1401] "setc" "senr" "send" "seni" "seen" "spt"  "sek"  "spq"  "sei"  "sec"
## [1411] "seh"  "shb"  "senh" "seeh" "see"  "senn" "sew"  "sea"  "sens" "sed"
## [1421] "seg"  "sel"  "sega" "set"  "sena" "seno" "senv" "senq" "senl" "senj"
## [1431] "seec" "seeb" "seep" "senb" "sene" "senc" "ses"  "sbg"  "sbz"  "sbv"
## [1441] "salz" "smar" "smac" "smw"  "spe"  "srr"  "srl"  "sry"  "sply" "srs"
## [1451] "sra"  "smaf" "slq"  "serf" "sers" "sfw"  "sfg"  "srz"  "sera" "serq"
## [1461] "serm" "squ"  "sfj"  "sof"  "ssur" "rah"  "raq"  "raa"  "rox"  "eame"
## [1471] "pam"  "plf"  "paj"  "paq"  "pva"  "pagg" "pao"  "kln"  "pant" "panp"
## [1481] "pagc" "pstw" "palh" "pans" "pey"  "pdis" "fjo"  "fgl"  "fcm"  "ffa"
## [1491] "fnk"  "ebt"  "tci"  "ebf"  "ebc"  "ebu"  "sod"  "eam"  "eay"  "eta"
## [1501] "epy"  "epr"  "ebi"  "erj"  "ege"  "epe"  "erwi" "ype"  "ypk"  "yph"
## [1511] "ypa"  "ypn"  "ypm"  "ypp"  "ypg"  "ypz"  "ypt"  "ypd"  "ypx"  "ypw"
## [1521] "ypj"  "ypv"  "ypl"  "yps"  "ypo"  "ypi"  "ypy"  "ypb"  "ypq"  "ypu"
## [1531] "ypr"  "ypc"  "ypf"  "yen"  "yep"  "yey"  "yel"  "yew"  "yet"  "yef"
## [1541] "yee"  "ysi"  "yal"  "yfr"  "yin"  "ykr"  "yro"  "yru"  "yrb"  "yak"
## [1551] "yma"  "yhi"  "yca"  "ymo"  "eca"  "patr" "pato" "pct"  "pcc"  "pcv"
## [1561] "pwa"  "ppar" "pec"  "pws"  "ppoa" "pbra" "ppuj" "cro"  "cko"  "cfd"
## [1571] "cbra" "cwe"  "cyo"  "cpot" "cfq"  "cama" "caf"  "cif"  "cfar" "cir"
```

```
## [1581] "cie"  "cpar" "ror"  "ron"  "rpln" "rao"  "rtg"  "ree"  "yre"  "sgoe"
## [1591] "kin"  "pdz"  "izh"  "pgz"  "pcd"  "mint" "mthi" "gbi"  "amc"  "amh"
## [1601] "amaa" "amal" "amae" "amao" "amad" "amai" "amag" "amac" "amb"  "amg"
## [1611] "amk"  "alt"  "aal"  "aaus" "asp"  "asq"  "aaw"  "alr"  "ale"  "alz"
## [1621] "apel" "abo"  "adi"  "apac" "aln"  "axe"  "ilo"  "ili"  "ipi"  "idi"
## [1631] "idt"  "cmai" "kus"  "kma"  "csa"  "haa"  "hel"  "hcs"  "hak"  "ham"
## [1641] "hhu"  "hco"  "hsi"  "halo" "hhh"  "hbe"  "hag"  "haf"  "halk" "hvn"
## [1651] "hol"  "hsr"  "hmd"  "haxi" "htt"  "hcam" "hpiz" "mlo"  "mln"  "mci"
## [1661] "mop"  "mam"  "mamo" "meso" "mesw" "mesm" "mesp" "mhua" "mjr"  "merd"
## [1671] "kge"  "llp"  "axy"  "axo"  "axn"  "axx"  "adt"  "ais"  "asw"  "achr"
## [1681] "achb" "mars" "nik"  "ncu"  "ome"  "mmw"  "mme"  "mpc"  "mpri" "mard"
## [1691] "bmar" "gsn"  "tol"  "tor"  "rfo"  "azl"  "ali"  "abs"  "abq"  "abf"
## [1701] "ati"  "ahu"  "azt"  "azm"  "azz"  "aoz"  "lpn"  "lph"  "lpo"  "lpu"
## [1711] "lpm"  "lpf"  "lpp"  "lpc"  "lpa"  "lpe"  "llo"  "lfa"  "lha"  "lok"
## [1721] "lcd"  "lsh"  "llg"  "lib"  "lgt"  "ljr"  "lcj"  "lwa"  "lss"  "mos"
## [1731] "par"  "pcr"  "pso"  "pur"  "pali" "pspg" "psyg" "psyc" "psya" "psyy"
## [1741] "psyp" "plu"  "plum" "pay"  "ptt"  "xbo"  "xbv"  "xne"  "xnm"  "xdo"
## [1751] "xho"  "enc"  "enl"  "eclg" "ecle" "ecln" "ecli" "eclx" "ecly" "eclz"
## [1761] "eclo" "ehm"  "exf"  "ecla" "eclc" "eau"  "ekb"  "eno"  "eec"  "elg"
## [1771] "ecan" "ern"  "ecls" "echg" "esh"  "ent"  "eas"  "enr"  "enx"  "enf"
## [1781] "ebg"  "end"  "kpn"  "kpu"  "kpm"  "kpp"  "kph"  "kpz"  "kpv"  "kpw"
## [1791] "kpy"  "kpg"  "kpc"  "kpq"  "kpt"  "kpo"  "kpr"  "kpj"  "kpi"  "kpa"
## [1801] "kps"  "kpx"  "kpb"  "kpne" "kpnu" "kpnk" "kva"  "kpe"  "kpk"  "kvd"
## [1811] "kvq"  "kox"  "koe"  "koy"  "kom"  "kmi"  "kok"  "koc"  "kqu"  "eae"
## [1821] "ear"  "kqv"  "kll"  "klw"  "esc"  "kle"  "acb"  "abm"  "aby"  "abc"
## [1831] "abn"  "abb"  "abx"  "abz"  "abr"  "abd"  "abh"  "abad" "abj"  "abab"
## [1841] "abaj" "abaz" "abk"  "abau" "abaa" "abw"  "abal" "acc"  "ano"  "alc"
## [1851] "acal" "acd"  "aci"  "att"  "aei"  "ajo"  "acw"  "acv"  "ahl"  "ajn"
## [1861] "asol" "ala"  "asj"  "aid"  "adv"  "arj"  "awu"  "acum" "agu"  "aug"
## [1871] "alw"  "ads"  "aber" "atn"  "achi" "alj"  "maq"  "mhc"  "mad"  "mbs"
## [1881] "msr"  "mpq"  "mari" "mlq"  "msq"  "mara" "marj" "bma"  "bmv"  "bml"
## [1891] "bmn"  "bmal" "bmae" "bmaq" "bmai" "bmaf" "bmaz" "bmab" "bps"  "bpm"
## [1901] "bpl"  "bpd"  "bpr"  "bpse" "bpsm" "bpsu" "bpsd" "bpz"  "bpq"  "bpk"
## [1911] "bpsh" "bpsa" "bpso" "but"  "bte"  "btq"  "btj"  "btz"  "btd"  "btv"
```

```
## [1921] "bthe" "bthm" "btha" "bthl" "bok"  "boc"  "buu"  "bvi"  "bve"  "bur" 
## [1931] "bcn"  "bch"  "bcm"  "bcj"  "bcen" "bcew" "bceo" "bam"  "bac"  "bmj" 
## [1941] "bmu"  "bmk"  "bmul" "bct"  "bced" "bcep" "bdl"  "bpyr" "bcon" "bub" 
## [1951] "bdf"  "blat" "btei" "bsem" "bpsl" "bmec" "bstg" "bstl" "bgl"  "bgu" 
## [1961] "bug"  "bgf"  "bgd"  "bgo"  "byi"  "buk"  "bue"  "bul"  "buq"  "bgp" 
## [1971] "bpla" "bud"  "bum"  "bui"  "mrd"  "met"  "mno"  "mor"  "meta" "maqu"
## [1981] "mphy" "mee"  "metd" "metx" "mets" "meti" "mmes" "mtea" "blh"  "bae" 
## [1991] "bson" "bmyc" "bgy"  "bhk"  "bmq"  "bmd"  "bmh"  "bmeg" "bon"  "chry"
## [2001] "cben" "saln" "sok"  "rbz"  "thas" "faq"  "hdh"  "this" "gho"  "ppr" 
## [2011] "pgb"  "pds"  "vch"  "vcf"  "vcs"  "vce"  "vcq"  "vcj"  "vci"  "vco" 
## [2021] "vcr"  "vcm"  "vcl"  "vcx"  "vcz"  "vvu"  "vvy"  "vvm"  "vvl"  "vpa" 
## [2031] "vpb"  "vpk"  "vpf"  "vph"  "vha"  "vca"  "vag"  "vex"  "vdb"  "vhr" 
## [2041] "vna"  "vow"  "vro"  "vsp"  "vej"  "vfu"  "vni"  "van"  "lag"  "vau" 
## [2051] "vcy"  "vct"  "vtu"  "vfl"  "vmi"  "vbr"  "vsc"  "vga"  "vsh"  "vqi" 
## [2061] "vta"  "vaf"  "vnl"  "vcc"  "vas"  "vaq"  "vsr"  "saly" "sks"  "scot"
## [2071] "son"  "sdn"  "sfr"  "saz"  "sbl"  "sbm"  "sbn"  "sbp"  "sbt"  "sbs" 
## [2081] "sbb"  "slo"  "spc"  "shp"  "sse"  "spl"  "she"  "shm"  "shn"  "shw" 
## [2091] "shl"  "swd"  "swp"  "svo"  "shf"  "sja"  "spsw" "sbj"  "smav" "shew"
## [2101] "salg" "slj"  "smai" "spol" "sbk"  "skh"  "saes" "tht"  "thap" "pha" 
## [2111] "ptn"  "pat"  "psm"  "pseo" "pia"  "pphe" "pbw"  "prr"  "plz"  "paln"
## [2121] "ppis" "pea"  "pspo" "part" "ptu"  "png"  "ptd"  "psen" "pdj"  "paga"
## [2131] "pcar" "pmaa" "dji"  "dja"  "dtx"  "dye"  "lrz"  "lpy"  "rhd"  "rgl" 
## [2141] "dko"  "lab"  "laq"  "lcp"  "lgu"  "lez"  "lem"  "lmb"  "lyt"  "lyj" 
## [2151] "lsol" "psu"  "psuw" "psd"  "pmex" "avn"  "avl"  "avd"  "acx"  "pae" 
## [2161] "paev" "paei" "pau"  "pap"  "pag"  "paf"  "pnc"  "paeb" "pdk"  "psg" 
## [2171] "prp"  "paep" "paer" "paem" "pael" "paes" "paeu" "paeg" "paec" "paeo"
## [2181] "pmy"  "pmk"  "pre"  "ppse" "palc" "pcq"  "ppu"  "ppf"  "ppg"  "ppw" 
## [2191] "ppt"  "ppb"  "ppi"  "ppx"  "ppuh" "pput" "ppun" "ppud" "pfv"  "pmon"
## [2201] "pmot" "pmos" "ppj"  "por"  "pst"  "psb"  "psyr" "psp"  "pamg" "pci" 
## [2211] "pavl" "pvd"  "pfl"  "pprc" "ppro" "pfo"  "pfs"  "pfe"  "pfc"  "pfn" 
## [2221] "ppz"  "pfb"  "pman" "ptv"  "pcg"  "pvr"  "pazo" "poi"  "pfw"  "pff" 
## [2231] "pfx"  "pen"  "psa"  "psz"  "psr"  "psc"  "psj"  "psh"  "pstu" "pstt"
## [2241] "pbm"  "plul" "pba"  "pbc"  "ppuu" "pdr"  "psv"  "psk"  "pkc"  "pch" 
## [2251] "pcz"  "pcp"  "pfz"  "plq"  "palk" "prh"  "psw"  "ppv"  "pses" "psem"
```

```
## [2261] "psec" "ppsy" "psos" "pkr"  "pfk"  "panr" "ppsl" "pset" "psil" "pym"
## [2271] "psed" "pke"  "pall" "pum"  "poj"  "pgg"  "ppsh" "pgy"  "thes" "theh"
## [2281] "tcn"  "tbv"  "lum"  "lus"  "lug"  "sml"  "smt"  "buj"  "smz"  "sacz"
## [2291] "stek" "srh"  "slm"  "sten" "stem" "stes" "xcc"  "xcb"  "xca"  "xcp"
## [2301] "xcv"  "xax"  "xac"  "xci"  "xct"  "xcj"  "xcu"  "xcn"  "xcw"  "xcr"
## [2311] "xcm"  "xcf"  "xfu"  "xao"  "xom"  "xoo"  "xop"  "xoy"  "xor"  "xoz"
## [2321] "xal"  "xsa"  "xtn"  "xfr"  "xve"  "xpe"  "xhr"  "xga"  "xph"  "xva"
## [2331] "xan"  "xar"  "xhy"  "xcz"  "xth"
```

Organisms classified within cluster 3

```
##    [1] "afo"  "actt" "tbw"  "bde"  "nak"  "gez"  "oek"  "bfa"  "brv"  "brr"
##   [11] "dco"  "aqg"  "cmi"  "cms"  "cmh"  "ccap" "cry"  "frn"  "hea"  "lxx"
##   [21] "rtx"  "rtc"  "rtn"  "cgx"  "cdi"  "cdp"  "cdh"  "cdt"  "cde"  "cdr"
##   [31] "cda"  "cdz"  "cdb"  "cds"  "cdd"  "cdw"  "cdv"  "cdip" "cur"  "cua"
##   [41] "car"  "ckp"  "cpl"  "cpg"  "cpp"  "cpk"  "cpq"  "cpx"  "cpz"  "cor"
##   [51] "cop"  "cod"  "cos"  "coi"  "coe"  "cou"  "cpse" "cpsu" "cpsf" "crd"
##   [61] "cul"  "cuc"  "cue"  "cun"  "cus"  "cuq"  "cuz"  "cuj"  "ccn"  "cter"
##   [71] "caz"  "cfn"  "cvt"  "cax"  "cii"  "cuv"  "coa"  "cdo"  "csx"  "cku"
##   [81] "ccj"  "cei"  "csp"  "cfk"  "cpho" "cfc"  "cgv"  "cstr" "caqu" "csph"
##   [91] "cmin" "cpeg" "cxe"  "csan" "cgk"  "crf"  "crl"  "ccho" "cpso" "raj"
##  [101] "rter" "rama" "rkr"  "mix"  "iva"  "cfl"  "cfi"  "cga"  "cez"  "celz"
##  [111] "cej"  "celh" "ske"  "sanw" "fra"  "fsy"  "pac"  "pak"  "pav"  "pax"
##  [121] "paz"  "paw"  "pad"  "pcn"  "pacc" "pach" "pacn" "cacn" "pra"  "prv"
##  [131] "pfr"  "pfre" "prl"  "pacd" "paus" "tfl"  "tfa"  "tes"  "tez"  "tdf"
##  [141] "tla"  "ahm"  "acti" "acad" "kab"  "kii"  "kvr"  "aos"  "ard"  "actp"
##  [151] "actc" "ane"  "ahw"  "air"  "asla" "olo"  "copr" "pmuc" "pet"  "buy"
##  [161] "osp"  "ppn"  "dys"  "alq"  "pru"  "afd"  "ald"  "aok"  "acou" "ait"
##  [171] "blq"  "sgn"  "rhoz" "aswu" "flu"  "aqb"  "aue"  "ccyn" "caph" "csto"
##  [181] "capq" "cagg" "emar" "grl"  "mgel" "mesq" "mlt"  "myr"  "mpw"  "mod"
##  [191] "myz"  "nom"  "nsd"  "noj"  "rbi"  "laci" "for"  "foh"  "tdi"  "tmar"
##  [201] "lut"  "fek"  "asl"  "aev"  "wfu"  "wij"  "apib" "cnr"  "eao"  "emn"
##  [211] "een"  "elb"  "emg"  "ego"  "egm"  "elz"  "elt"  "wvi"  "ebv"  "efal"
```

```
## [221] "este" "ran"  "rai"  "rar"  "rag"  "rae"  "rat"  "fba"  "fbu"  "oho"
## [231] "psn"  "pej"  "cte"  "cpc"  "clz"  "pros" "cts"  "cch"  "cph"  "cpb"
## [241] "cli"  "plt"  "ial"  "sru"  "rbar" "apak" "axl"  "aia"  "afl"  "agn"
## [251] "anm"  "aamy" "anl"  "and"  "acai" "fpn"  "far"  "gth"  "ptl"  "ptb"
## [261] "grc"  "oih"  "ocn"  "sje"  "tap"  "hhd"  "hmn"  "hli"  "lao"  "gka"
## [271] "gte"  "gli"  "gtn"  "gwc"  "gyc"  "gya"  "gct"  "gea"  "gel"  "gse"
## [281] "gsr"  "gej"  "bths" "coh"  "cohn" "esi"  "eat"  "ean"  "exm"  "exu"
## [291] "mtuc" "saca" "tco"  "bagr" "jeo"  "lsp"  "lgy"  "lfu"  "lys"  "lyz"
## [301] "lpak" "pln"  "pku"  "prt"  "pll"  "pana" "pdg"  "phc"  "ppla" "pfae"
## [311] "plx"  "pmat" "pdec" "psyh" "psyo" "rst"  "siv"  "sob"  "spsy" "spor"
## [321] "spop" "sure" "spos" "spae" "play" "paek" "panc" "vir"  "vhl"  "vne"
## [331] "vpn"  "vim"  "stea" "shv"  "sau"  "sav"  "saw"  "sah"  "saj"  "sam"
## [341] "sas"  "sar"  "sac"  "sax"  "saa"  "sao"  "sae"  "sad"  "suu"  "suv"
## [351] "sue"  "suj"  "suk"  "suc"  "sut"  "suq"  "suz"  "sud"  "sux"  "suw"
## [361] "sug"  "suf"  "saua" "saue" "saun" "saus" "sauu" "saug" "sauz" "saut"
## [371] "sauj" "sauk" "sauq" "sauv" "sauw" "saux" "sauy" "sauf" "sab"  "suy"
## [381] "saub" "saum" "sauc" "saur" "saui" "saud" "sams" "suh"  "ser"  "sep"
## [391] "sepp" "seps" "sha"  "shh"  "ssp"  "sca"  "slg"  "sln"  "ssd"  "sdt"
## [401] "sdp"  "swa"  "spas" "sxy"  "sxl"  "sxo"  "shu"  "scap" "ssch" "sscz"
## [411] "sagq" "seqo" "ssif" "scv"  "spet" "slz"  "snl"  "skl"  "sfq"  "shom"
## [421] "smus" "scar" "schr" "sarl" "spic" "ssh"  "ssim" "kpul" "keb"  "lfb"
## [431] "pjd"  "gym"  "ppy"  "ppm"  "ppo"  "ppol" "ppq"  "ppoy" "pta"  "plv"
## [441] "psab" "pdu"  "pbd"  "pgm"  "pod"  "paen" "paef" "paeq" "pste" "paea"
## [451] "paee" "paeh" "paej" "pbj"  "pih"  "pri"  "ppeo" "pow"  "pbv"  "pxl"
## [461] "pyg"  "pswu" "pdh"  "pib"  "pcx"  "pkb"  "paih" "pvo"  "plw"  "plen"
## [471] "ppsc" "plut" "pchi" "pbk"  "pprt" "pbac" "tvu"  "aui"  "avs"  "jep"
## [481] "jeh"  "jar"  "vte"  "oen"  "lsu"  "lla"  "llk"  "llt"  "lls"  "lld"
## [491] "llx"  "llj"  "llm"  "llc"  "llr"  "lln"  "lli"  "llw"  "lpk"  "lact"
## [501] "sak"  "smut" "ssb"  "ssu"  "ssi"  "sss"  "ssw"  "sup"  "ssus" "ssuy"
## [511] "ssk"  "ssq"  "sui"  "suo"  "ssut" "ssui" "stk"  "spat" "srat" "efl"
## [521] "ecas" "emu"  "ega"  "ess"  "egv"  "eav"  "cmiu" "cale" "amt"  "gfe"
## [531] "cthm" "sthr" "hmo"  "hcv"  "bprl" "arf"  "acac" "cle"  "cew"  "lacy"
## [541] "bpb"  "bfi"  "bhu"  "pxv"  "rho"  "cct"  "rob"  "byl"  "bpro" "dsy"
## [551] "dhd"  "ddh"  "ddl"  "dmt"  "dor"  "dai"  "dmi"  "pth"  "drm"  "dca"
```

```
## [561] "dec"  "pdc"  "cdc"  "cdl"  "pdf"  "capr" "fpa"  "ova"  "cce"  "ral"
## [571] "rto"  "ibu"  "bacc" "swo"  "ate"  "sth"  "tte"  "ttm"  "tto"  "txy"
## [581] "tsh"  "chy"  "tpz"  "mta"  "mtho" "mthz" "ere"  "ert"  "era"  "elm"
## [591] "emt"  "elim" "afn"  "ain"  "pfac" "mfun" "lca"  "lcs"  "lce"  "lcl"
## [601] "lpi"  "lpap" "lcb"  "lcx"  "lrh"  "lrg"  "lrl"  "lra"  "lro"  "lrc"
## [611] "lpl"  "lpj"  "lff"  "lbh"  "lbn"  "lpar" "med"  "mhw"  "meg"  "sri"
## [621] "sted" "vpr"  "vrm"  "vdn"  "vnk"  "abg"  "kba"  "gox"  "goh"  "goy"
## [631] "gal"  "gti"  "gdj"  "nch"  "pbr"  "mai"  "man"  "dra"  "dge"  "ddr"
## [641] "dpt"  "dgo"  "dch"  "dab"  "dpu"  "dwu"  "dfc"  "dga"  "nwi"  "pcon"
## [651] "oct"  "dex"  "srhi" "zmm"  "zmb"  "zmi"  "zmc"  "zmr"  "bba"  "bbat"
## [661] "bbw"  "bbac" "bex"  "bdq"  "bsed" "dto"  "dsf"  "dpr"  "deo"  "dog"
## [671] "drt"  "dba"  "doa"  "pca"  "ppd"  "gsu"  "gsk"  "gme"  "gur"  "glo"
## [681] "gbm"  "geo"  "gem"  "geb"  "gpi"  "gao"  "gbn"  "ade"  "acp"  "ank"
## [691] "bsto" "gsb"  "sat"  "sfu"  "tth"  "ttj"  "tts"  "ttl"  "tsc"  "thc"
## [701] "tos"  "taq"  "tbc"  "sva"  "acz"  "afi"  "afj"  "tau"  "cml"  "carn"
## [711] "anb"  "acy"  "awa"  "ann"  "ocm"  "psy"  "sdo"  "nmv"  "nja"  "tig"
## [721] "aeh"  "rsp"  "rsh"  "rcp"  "rhp"  "lmir" "ofo"  "pne"  "poh"  "acit"
## [731] "chiz" "fmy"  "slt"  "gca"  "meh"  "mfa"  "mei"  "mep"  "cste" "nme"
## [741] "nmp"  "nmh"  "nmd"  "nmm"  "nms"  "nmq"  "nmz"  "nma"  "nmw"  "nmx"
## [751] "nmc"  "nmn"  "nmt"  "nmi"  "ngo"  "ngk"  "nla"  "nel"  "nwe"  "nsi"
## [761] "nmj"  "nei"  "nek"  "nfv"  "nsf"  "nzl"  "naq"  "nbl"  "nzo"  "ncz"
## [771] "nani" "nbc"  "kki"  "koa"  "ecor" "salv" "nba"  "neu"  "net"  "nit"
## [781] "nii"  "nco"  "nur"  "nst"  "nmu"  "nlc"  "doe"  "sphn" "sdr"  "alv"
## [791] "mpur" "ttc"  "thip" "cbu"  "cbd"  "cbg"  "cbc"  "asip" "hhc"  "ebs"
## [801] "eic"  "etr"  "etd"  "ete"  "etc"  "edw"  "edl"  "eho"  "ans"  "apt"
## [811] "apw"  "apf"  "apu"  "apg"  "apq"  "apx"  "apz"  "apk"  "asz"  "asv"
## [821] "aace" "aper" "apom" "aasc" "acet" "aot"  "aoy"  "gxy"  "gxl"  "kna"
## [831] "keu"  "kre"  "kha"  "pdi"  "parc" "lbq"  "lpop" "mmk"  "lmo"  "lmn"
## [841] "lmy"  "lmt"  "lmoc" "lmoe" "lmob" "lmod" "lmow" "lmoq" "lmr"  "lmom"
## [851] "lmf"  "lmc"  "lmog" "lmp"  "lmol" "lmoj" "lmoz" "lmox" "lmh"  "lmq"
## [861] "lml"  "lmg"  "lms"  "lmj"  "lmw"  "lmx"  "lmz"  "lmon" "lmos" "lmoo"
## [871] "lmoy" "lmot" "lmoa" "lmok" "lmv"  "lin"  "lwe"  "lsg"  "liv"  "lii"
## [881] "liw"  "lia"  "lio"  "fps"  "fpc"  "fpy"  "fpo"  "fpq"  "fpv"  "fpw"
## [891] "fpk"  "fpsz" "fjg"  "fbr"  "fco"  "fin"  "fat"  "fki"  "fpal" "fmg"
```

```
##  [901] "falb" "fcr"  "fse"  "fsn"  "fak"  "tpty" "sgl"  "pmr"  "pmib" "pvl" 
##  [911] "pvg"  "phau" "prot" "pcol" "pcib" "ftu"  "ftq"  "ftf"  "ftw"  "ftr" 
##  [921] "ftt"  "ftg"  "ftl"  "fta"  "fts"  "ftc"  "ftv"  "ftz"  "ftm"  "ftn" 
##  [931] "ftx"  "ftd"  "fty"  "fcf"  "fcn"  "fhi"  "fph"  "fpt"  "fpi"  "fpm" 
##  [941] "fpx"  "fpz"  "fpj"  "frt"  "fna"  "fnl"  "frf"  "fha"  "frx"  "frm" 
##  [951] "frc"  "fad"  "fmi"  "foo"  "hna"  "haz"  "tgr"  "tkm"  "tni"  "tti" 
##  [961] "tvr"  "mmob" "mca"  "metu" "mmai" "mein" "mmt"  "mdn"  "mdh"  "mko" 
##  [971] "metl" "hha"  "hhk"  "mej"  "mec"  "ntt"  "kuy"  "kko"  "ksd"  "kpd" 
##  [981] "fpp"  "gap"  "orb"  "apl"  "apj"  "apa"  "asu"  "asi"  "ass"  "aeu" 
##  [991] "apor" "aio"  "alig" "gan"  "aap"  "aaz"  "aat"  "aao"  "aan"  "aah" 
## [1001] "aacn" "aact" "aseg" "hsm"  "msu"  "mht"  "mhq"  "mhat" "mhx"  "mhae"
## [1011] "mham" "mhao" "mhal" "mhaq" "mhay" "mvr"  "mvi"  "mvg"  "mve"  "mann"
## [1021] "mgra" "ooi"  "pmu"  "pmv"  "pul"  "pmp"  "pmul" "pdag" "psky" "apag"
## [1031] "avt"  "paet" "rpne" "rhey" "hin"  "hit"  "hiq"  "hiu"  "hiz"  "hik" 
## [1041] "hia"  "hih"  "hiw"  "hic"  "hix"  "hpaa" "psal" "cac"  "cae"  "cay" 
## [1051] "cno"  "cbo"  "cba"  "cbh"  "cby"  "cbk"  "cbb"  "cbi"  "cbt"  "cbf" 
## [1061] "cbm"  "cbe"  "cbz"  "cbei" "ckl"  "ckr"  "clj"  "ccb"  "cls"  "csr" 
## [1071] "cpas" "cpat" "cpae" "csb"  "cah"  "clt"  "cbv"  "csq"  "cace" "cck" 
## [1081] "cbut" "ctyk" "ceu"  "cfm"  "carg" "cdrk" "cia"  "csep" "cdy"  "cfer"
## [1091] "csh"  "ciu"  "mct"  "mcs"  "mcat" "moi"  "mbl"  "mboi" "mcun" "mnn" 
## [1101] "prw"  "xpo"  "msx"  "bsu"  "bsr"  "bsl"  "bsh"  "bsy"  "bsut" "bsul"
## [1111] "bsus" "bso"  "bsn"  "bsq"  "bsx"  "bsp"  "bss"  "bst"  "bli"  "bld" 
## [1121] "bay"  "baq"  "bya"  "bamp" "baml" "bama" "bamn" "bamb" "bamt" "bamy"
## [1131] "bmp"  "bao"  "baz"  "bql"  "bxh"  "bqy"  "bami" "bamc" "bamf" "bsia"
## [1141] "bvm"  "bht"  "ban"  "bar"  "bat"  "bah"  "bai"  "bax"  "bant" "banr"
## [1151] "bans" "banh" "banv" "bce"  "bca"  "bcz"  "bcr"  "bcb"  "bcu"  "bcg" 
## [1161] "bcq"  "bcx"  "bal"  "bnc"  "bcf"  "bcer" "bcef" "bcy"  "btk"  "btl" 
## [1171] "btb"  "btt"  "bthr" "bthi" "btc"  "btf"  "btm"  "btg"  "bti"  "btn" 
## [1181] "btht" "bthu" "btw"  "bthy" "bwe"  "bww"  "bmyo" "bty"  "bby"  "bwd" 
## [1191] "btro" "bmob" "bpu"  "bpum" "bpus" "bco"  "bjs"  "baci" "bif"  "bmet"
## [1201] "gst"  "bacw" "bacp" "bacb" "baco" "bacy" "bacl" "balm" "bsm"  "bwh" 
## [1211] "bxi"  "bbev" "balt" "bacs" "bsaf" "bit"  "bacq" "bcir" "bfd"  "bcoh"
## [1221] "bda"  "bck"  "bag"  "bcoa" "bha"  "bcl"  "bpf"  "ble"  "bse"  "cum" 
## [1231] "cub"  "cug"  "chz"  "cgn"  "cih"  "chh"  "cio"  "cpip" "chrs" "chrz"
```

```
## [1241] "carh" "csha" "cnk"  "cjt"  "cil"  "ccau" "ccas" "clac" "ctak" "sdf"
## [1251] "tee"  "fau"  "lue"  "xfa"  "xft"  "xfm"  "xfn"  "xff"  "xfh"  "xtw"
## [1261] "mgm"  "maes" "mfn"  "chu"  "erb"
```

Organisms classified within cluster 4

```
##   [1] "bact" "cig"  "bbl"  "bpi"  "bmm"  "bcp"  "bbg"  "bbq"  "blp"  "blu"
##  [11] "blck" "vah"  "wko"  "wdi"  "fma"  "pmic" "pbq"  "dpn"  "ljo"  "ljf"
##  [21] "ljn"  "lac"  "lad"  "laf"  "lga"  "lcr"  "lam"  "lae"  "lje"  "lapi"
##  [31] "lhs"  "lsn"  "las"  "laa"  "lat"  "lso"  "lar"  "lau"  "ama"  "amf"
##  [41] "amw"  "amp"  "acn"  "aph"  "apy"  "apd"  "apha" "aoh"  "eru"  "erw"
##  [51] "erg"  "ecn"  "ech"  "echa" "echj" "echl" "echs" "echv" "echw" "echp"
##  [61] "emr"  "ehh"  "nse"  "nri"  "nhm"  "nef"  "wol"  "wri"  "wen"  "wed"
##  [71] "wpi"  "wbm"  "woo"  "wcl"  "weo"  "wpp"  "ots"  "ott"  "kci"  "kct"
##  [81] "kbl"  "kbt"  "kga"  "kon"  "kso"  "cea"  "cend" "buc"  "bap"  "bau"
##  [91] "baw"  "bajc" "bua"  "bup"  "bak"  "buh"  "bapf" "bapg" "bapu" "bapw"
## [101] "bas"  "bab"  "bcc"  "baj"  "baph" "bci"  "bcig" "hde"  "ppet" "rip"
## [111] "rig"  "bpn"  "bva"  "bchr" "asy"  "hed"  "ssz"  "den"  "ged"  "seny"
## [121] "wbr"  "les"  "acl"  "apal" "aaxa" "ahk"  "rpr"  "rpo"  "rpw"  "rpz"
## [131] "rpg"  "rps"  "rpv"  "rpq"  "rpl"  "rpn"  "rty"  "rtt"  "rtb"  "rcm"
## [141] "rcc"  "rbe"  "rbo"  "rco"  "rfe"  "rak"  "rri"  "rrj"  "rra"  "rrc"
## [151] "rrh"  "rrb"  "rrn"  "rrp"  "rrm"  "rrr"  "rms"  "rmi"  "rpk"  "raf"
## [161] "rhe"  "rja"  "rsv"  "rsw"  "rph"  "rau"  "rmo"  "rpp"  "rre"  "ram"
## [171] "rab"  "rmc"  "ras"  "ric"  "scr"  "ssyr" "sapi" "smir" "smia" "scq"
## [181] "ssab" "satr" "seri" "stur" "sll"  "scj"  "shj"  "sck"  "sfz"  "scou"
## [191] "scla" "sprn" "spit" "stab" "salx" "sgq"  "schi" "ctr"  "ctd"  "ctf"
## [201] "ctrd" "ctro" "ctrt" "cta"  "cty"  "cra"  "ctrq" "ctrx" "ctrz" "ctrp"
## [211] "ctlj" "ctlx" "ctll" "ctb"  "ctrr" "ctlf" "ctli" "ctl"  "ctru" "ctrl"
## [221] "ctrv" "ctrm" "ctla" "ctlm" "ctls" "ctlz" "ctlc" "ctln" "ctlb" "ctlq"
## [231] "cto"  "ctrn" "ctj"  "ctz"  "ctg"  "ctk"  "csw"  "ces"  "ctrb" "ctre"
## [241] "ctrs" "ctec" "cfs"  "cfw"  "ctfw" "ctrf" "ctch" "ctn"  "ctq"  "ctv"
## [251] "ctw"  "ctrg" "ctri" "ctra" "ctrh" "ctrj" "ctrk" "ctjt" "ctcf" "ctfs"
## [261] "cthf" "ctcj" "cthj" "ctmj" "cttj" "ctjs" "ctrc" "ctrw" "ctry" "ctct"
```

```
## [271] "cmu"  "cmur" "cmn"  "cmm"  "cmg"  "cmx"  "cmz"  "cpn"  "cpa"  "cpj" 
## [281] "cpt"  "clp"  "cpm"  "cpec" "cpeo" "cper" "chp"  "chb"  "chs"  "chi" 
## [291] "cht"  "chc"  "chr"  "cpsc" "cpsn" "cpsb" "cpsg" "cpsm" "cpsi" "cpsv"
## [301] "cpsw" "cpst" "cpsd" "cpsa" "cav"  "cca"  "cab"  "cabo" "cfe"  "cgz" 
## [311] "chla" "mpe"  "miw"
```

Organisms classified within cluster 5

```
##   [1] "asg"  "ahe"  "arca" "mcu"  "tpy"  "tpyo" "blo"  "blj"  "bln"  "blon"
##  [11] "blf"  "bll"  "blb"  "blm"  "blk"  "blg"  "blz"  "blx"  "bad"  "badl"
##  [21] "bado" "bla"  "blc"  "blt"  "bbb"  "bbc"  "bnm"  "blv"  "blw"  "bls" 
##  [31] "bani" "banl" "bni"  "banm" "bdn"  "bbp"  "bbi"  "bbf"  "bbv"  "bbru"
##  [41] "bbre" "bbrv" "bbrj" "bbrc" "bbrn" "bbrs" "bbrd" "bast" "btp"  "bcor"
##  [51] "bka"  "bks"  "bcat" "bpsp" "bii"  "bang" "bpsc" "bsca" "bcho" "bgx" 
##  [61] "blem" "beu"  "cbq"  "dva"  "djj"  "leb"  "cut"  "rmu"  "rdn"  "cgrn"
##  [71] "acq"  "acto" "actz" "avc"  "apv"  "ols"  "caer" "cgo"  "ele"  "eyy" 
##  [81] "ddt"  "gpa"  "pgi"  "pgn"  "pgt"  "pah"  "pcre" "pcag" "pmz"  "pdn" 
##  [91] "pit"  "pdt"  "pro"  "pfus" "peo"  "pje"  "poc"  "ash"  "ada"  "tfo" 
## [101] "toh"  "fte"  "coc"  "ccm"  "col"  "chg"  "capn" "cgh"  "clk"  "cspu"
## [111] "capf" "orh"  "ori"  "bcad" "paa"  "proc" "prs"  "pvi"  "ocb"  "got" 
## [121] "gmo"  "geq"  "gsa"  "gha"  "kur"  "kzo"  "pgq"  "jea"  "mcl"  "mcak"
## [131] "macr" "scoh" "aur"  "aun"  "asan" "acg"  "auh"  "dpm"  "adc"  "jda" 
## [141] "jpo"  "thl"  "tey"  "too"  "tkr"  "vpi"  "vac"  "vao"  "vcp"  "ppe" 
## [151] "ppen" "pce"  "pdm"  "paci" "pio"  "ooe"  "osi"  "lme"  "lmm"  "lmk" 
## [161] "lci"  "lki"  "lec"  "lcn"  "lgs"  "lge"  "llf"  "lgc"  "lpse" "wce" 
## [171] "wct"  "wci"  "wcb"  "wjo"  "wpa"  "wcf"  "wso"  "whe"  "wei"  "wvr" 
## [181] "lgr"  "lgv"  "lrn"  "lack" "spy"  "spz"  "spym" "spya" "spm"  "spg" 
## [191] "sps"  "sph"  "spi"  "spj"  "spk"  "spf"  "spa"  "spb"  "stg"  "stx" 
## [201] "soz"  "stz"  "spyh" "spyo" "spn"  "spd"  "spr"  "spw"  "sjj"  "snv" 
## [211] "spx"  "snt"  "snd"  "spnn" "sne"  "spv"  "snc"  "snm"  "spp"  "sni" 
## [221] "spng" "snb"  "snp"  "snx"  "snu"  "spne" "spnu" "spnm" "spno" "sag" 
## [231] "san"  "sgc"  "sags" "sagl" "sagm" "sagi" "sagr" "sagp" "sagc" "sagt"
## [241] "sage" "sagg" "sagn" "smu"  "smc"  "smj"  "smua" "stc"  "stl"  "ste" 
```

```
## [251] "stn"  "stu"  "stw"  "sthe" "sths" "ssa"  "ssv"  "ssf"  "sst"  "srp"
## [261] "sgo"  "sez"  "seq"  "sezo" "sequ" "seu"  "sub"  "sds"  "sdg"  "sda"
## [271] "sdc"  "sdq"  "sga"  "sgg"  "sgt"  "smb"  "sor"  "stb"  "scp"  "scf"
## [281] "ssr"  "stf"  "stj"  "strs" "ssah" "std"  "smn"  "sif"  "sie"  "sib"
## [291] "siu"  "sang" "sanc" "sans" "scg"  "scon" "scos" "soi"  "sik"  "siq"
## [301] "sio"  "siz"  "slu"  "sig"  "sip"  "stv"  "stra" "strn" "ssob" "srq"
## [311] "seqi" "ski"  "spei" "sgw"  "splr" "strg" "efa"  "efi"  "efd"  "efs"
## [321] "efn"  "efq"  "ene"  "efc"  "efau" "efu"  "efm"  "eft"  "ehr"  "edu"
## [331] "eth"  "esg"  "dau"  "asf"  "asm"  "aso"  "asb"  "sarj" "aoe"  "mba"
## [341] "mby"  "mbw"  "mbar" "mbak" "mac"  "mma"  "mmaz" "mmj"  "mmac" "mvc"
## [351] "mek"  "mls"  "metm" "mef"  "meq"  "msj"  "msz"  "msw"  "mthr" "mthe"
## [361] "mhor" "mfz"  "spoa" "ped"  "phar" "piv"  "cpy"  "obj"  "rix"  "rim"
## [371] "coo"  "bhan" "blau" "blab" "dru"  "dfg"  "tjr"  "ded"  "drs"  "cdf"
## [381] "faa"  "apr"  "roc"  "psor" "eha"  "fpr"  "fpra" "rch"  "rum"  "rus"
## [391] "ruj"  "rgn"  "hsc"  "rbp"  "lbw"  "csc"  "cob"  "chd"  "cow"  "cki"
## [401] "ckn"  "clc"  "ccha" "toc"  "erh"  "ers"  "erl"  "eri"  "erd"  "eio"
## [411] "fro"  "esr"  "esu"  "eel"  "euu"  "tur"  "tsg"  "awo"  "dho"  "mhg"
## [421] "ljh"  "ldb"  "lbu"  "lde"  "ldl"  "lhe"  "lhl"  "lhr"  "lhv"  "lhh"
## [431] "lhd"  "lai"  "lay"  "lke"  "law"  "lgl"  "lamy" "lpw"  "lkl"  "lpq"
## [441] "lpt"  "lps"  "lpr"  "lpz"  "lre"  "lrf"  "lru"  "lrt"  "lrr"  "lfe"
## [451] "lfr"  "lmu"  "lbr"  "lbk"  "lzy"  "lsl"  "lsi"  "lrm"  "laca" "lcy"
## [461] "lho"  "lol"  "lku"  "lali" "lfm"  "lsa"  "ssg"  "sele" "selo" "selt"
## [471] "vat"  "bhe"  "bhn"  "bhs"  "bqu"  "bqr"  "bbk"  "btr"  "btx"  "bgr"
## [481] "bcd"  "baus" "bvn"  "banc" "bart" "bara" "barw" "barr" "baro" "barj"
## [491] "bez"  "barn" "bky"  "bals" "lcc"  "pmut" "rbt"  "ren"  "zmo"  "zmn"
## [501] "zmp"  "dak"  "tpar" "cthi" "top"  "tcm"  "thet" "tmai" "afr"  "afe"
## [511] "acu"  "atx"  "crn"  "caw"  "carc" "cdj"  "bpsi" "kde"  "sutt" "sutk"
## [521] "teq"  "tea"  "teg"  "tas"  "tat"  "mbac" "mbat" "meu"  "mmb"  "eex"
## [531] "chj"  "cbs"  "cey"  "rvi"  "bcib" "icp"  "bfl"  "ben"  "bed"  "kgo"
## [541] "lwi"  "lgz"  "hhs"  "pck"  "pes"  "ehd"  "wgl"  "fth"  "fti"  "fto"
## [551] "fper" "adp"  "bhud" "hso"  "hip"  "hif"  "hil"  "hie"  "hpr"  "hdu"
## [561] "hay"  "hpit" "hhz"  "haeg" "cpe"  "cpf"  "cpr"  "ctc"  "ctet" "cbl"
## [571] "cbn"  "cbj"  "clb"  "cld"  "ctae" "cchv" "ccoh" "ccel" "csci" "cso"
## [581] "cqf"  "pade" "xfl"  "xfs"  "abra" "aoc"  "tbm"  "tbz"
```
