# Supplementary material for: Exploring the expressiveness of abstract metabolic networks
Source: PLoS One. 2023 Feb 9;18(2):e0281047. doi: 10.1371/journal.pone.0281047 (PMC9910719; doi:10.1371/journal.pone.0281047)
Supplement: S12 File — Pseudomonas analyses at species level (third experiment). Comparison between the Pseudomonas phylogenetic tree and the dendrograms resulting by using the VH, SP, WL and PM graph kernels. (PDF) [file pone.0281047.s012.pdf]

Pseudomonas

KEGG ID	ORGANISM NAME	SPECIES
pae	Pseudomonas aeruginosa PAO1	P. aeruginosa
paev	Pseudomonas aeruginosa PAO1-VE13	P. aeruginosa
paei	Pseudomonas aeruginosa PAO1-VE2	P. aeruginosa
pau	Pseudomonas aeruginosa UCBPP-PA14	P. aeruginosa
pap	Pseudomonas aeruginosa PA7	P. aeruginosa
pag	Pseudomonas aeruginosa LESB58	P. aeruginosa
paf	Pseudomonas aeruginosa M18	P. aeruginosa
pnc	Pseudomonas aeruginosa NCGM2.S1	P. aeruginosa
paeb	Pseudomonas aeruginosa NCGM 1900	P. aeruginosa
pdk	Pseudomonas aeruginosa DK2	P. aeruginosa
psg	Pseudomonas aeruginosa B136-33	P. aeruginosa
prp	Pseudomonas aeruginosa RP73	P. aeruginosa
paep	Pseudomonas aeruginosa PA1	P. aeruginosa
paer	Pseudomonas aeruginosa PA1R	P. aeruginosa
paem	Pseudomonas aeruginosa MTB-1	P. aeruginosa
pael	Pseudomonas aeruginosa LES431	P. aeruginosa
paes	Pseudomonas aeruginosa SCV20265	P. aeruginosa
paeu	Pseudomonas aeruginosa PA38182	P. aeruginosa
paeg	Pseudomonas aeruginosa YL84	P. aeruginosa
paec	Pseudomonas aeruginosa c7447m	P. aeruginosa
paeo	Pseudomonas aeruginosa PAO581	P. aeruginosa
pmy	Pseudomonas mendocina ymp	P. mendocina
pmk	Pseudomonas mendocina NK-01	P. mendocina
pre	Pseudomonas resinovorans	P. resinovorans
ppse	Pseudomonas pseudoalcaligenes	P. pseudoalcaligenes
palc	Pseudomonas alcaligenes	P. alcaligenes
pcq	Pseudomonas citronellolis	P. citronellolis
ppu	Pseudomonas putida KT2440	P. putida

ppf	Pseudomonas putida F1	P. putida
ppg	Pseudomonas putida GB-1	P. putida
ppw	Pseudomonas putida W619	P. putida
ppt	Pseudomonas putida S16	P. putida
ppb	Pseudomonas putida BIRD-1	P. putida
ppi	Pseudomonas putida ND6	P. putida
ppx	Pseudomonas putida DOT-T1E	P. putida
ppuh	Pseudomonas putida HB3267	P. putida
pput	Pseudomonas putida H8234	P. putida
ppun	Pseudomonas putida NBRC 14164	P. putida
ppud	Pseudomonas putida DLL-E4	P. putida
pfv	Pseudomonas fulva	P. fulva
pmon	Pseudomonas monteilii SB3078	P. montelli
pmot	Pseudomonas monteilii SB3101	P. montelli
pmos	Pseudomonas soli	P. montelli
ppj	Pseudomonas plecoglossicida	P. plecoglossicida
por	Pseudomonas oryzihabitans	P. oryzihabitans
pst	Pseudomonas syringae pv. tomato DC3000	P. syringae
psb	Pseudomonas syringae pv. syringae B728a	P. syringae
psyr	Pseudomonas syringae CC1557	P. syringae
psp	Pseudomonas savastanoi pv. phaseolicola 1448A	P. savastanoi
pamg	Pseudomonas amygdali	P. amygdali
pci	Pseudomonas cichorii	P. cichorii
pavl	Pseudomonas avellanae	P. avellanae
pvd	Pseudomonas viridiflava	P. viridiflava
pfl	Pseudomonas protegens Pf-5	P. protegens
pprc	Pseudomonas protegens CHA0	P. protegens
ppro	Pseudomonas protegens Cab57	P. protegens
pfo	Pseudomonas fluorescens Pf0-1	P. fluorescens
pfs	Pseudomonas fluorescens SBW25	P. fluorescens
pfe	Pseudomonas fluorescens F113	P. fluorescens

pfc	Pseudomonas fluorescens A506	P. fluorescens
pfn	Pseudomonas fluorescens UK4	P. fluorescens
ppz	Pseudomonas poae	P. poae
pfb	Pseudomonas synxantha LBUM223	P. synxantha
pman	Pseudomonas mandelii	P. mandelii
ptv	Pseudomonas trivialis	P. trivialis
pcg	Pseudomonas corrugata	P. corrugata
pvr	Pseudomonas veronii	P. veronii
pazo	Pseudomonas azotoformans	P. azotoformans
poi	Pseudomonas orientalis	P. orientalis
pfw	Pseudomonas simiae PCL1751	P. simiae
pff	Pseudomonas simiae PICF7	P. simiae
pfx	Pseudomonas lurida	P. lurida
pen	Pseudomonas entomophila	P. entomophila
psa	Pseudomonas stutzeri A1501	P. stutzeri
psz	Pseudomonas stutzeri ATCC 17588	P. stutzeri
psr	Pseudomonas stutzeri DSM 4166	P. stutzeri
psc	Pseudomonas stutzeri CCUG 29243	P. stutzeri
psj	Pseudomonas stutzeri DSM 10701	P. stutzeri
psh	Pseudomonas stutzeri RCH2	P. stutzeri
pstu	Pseudomonas stutzeri 19SMN4	P. stutzeri
pstt	Pseudomonas stutzeri 28a24	P. stutzeri
pbm	Pseudomonas balearica	P. balearica
plul	Pseudomonas luteola	P. luteola
pba	Pseudomonas brassicacearum subsp. brassicacearum NFM421	P. brassicacearum
pbc	Pseudomonas brassicacearum DF41	P. brassicacearum
ppuu	Pseudomonas sp. UW4	P. sp.
pdr	Pseudomonas sp. ATCC 13867	P. sp.
psv	Pseudomonas sp. VLB120	P. sp.
psk	Pseudomonas sp. TKP	P. sp.
pkc	Pseudomonas knackmussii	P. furukawaii

pch	Pseudomonas chlororaphis PA23	P. chlororaphis
pcz	Pseudomonas chlororaphis PCL1606	P. chlororaphis
pcp	Pseudomonas chlororaphis subsp. aurantiaca	P. chlororaphis
pfz	Pseudomonas fragi	P. fragi
plq	Pseudomonas lundensis	P. lundensis
palk	Pseudomonas alkylphenolica	P. alkylphenolica
prh	Pseudomonas rhizosphaerae	P. rhizosphaerae
psw	Pseudomonas cremoricolorata	P. cremoricolorata
ppv	Pseudomonas parafulva	P. parafulva
pses	Pseudomonas sp. StFLB209	P. sp.
psem	Pseudomonas sp. MRSN12121	P. sp.
psec	Pseudomonas sp. CCOS 191	P. sp.
ppsy	Pseudomonas versuta	P. versuta
psos	Pseudomonas sp. Os17	P. sp.
pkr	Pseudomonas koreensis	P. koreensis
pfk	Pseudomonas frederiksbergensis	P. frederiksbergensis
panr	Pseudomonas antarctica	P. antarctica
ppsl	Pseudomonas psychrotolerans	P. psychrotolerans
pset	Pseudomonas sp. TCU-HL1	P. sp.
psil	Pseudomonas silesiensis	P. silesiensis
pym	Pseudomonas yamanorum	P. yamanorum
pade	Candidatus Pseudomonas adelgestsugas	Candidatus P. adelgestsugas
psed	Pseudomonas sp. R2A2	P. sp.
pke	Pseudomonas kribbensis	P. kribbensis
pall	Pseudomonas alcaliphila	P. alcaliphila
pum	Pseudomonas umsongensis	P. umsongensis
poj	Pseudomonas otitidis	P. otitidis
pgg	Pseudomonas graminis	P. graminis
ppsh	Pseudomonas psychrophila	P. psychrophila
pgy	Pseudomonas glycinae	P. glycinae

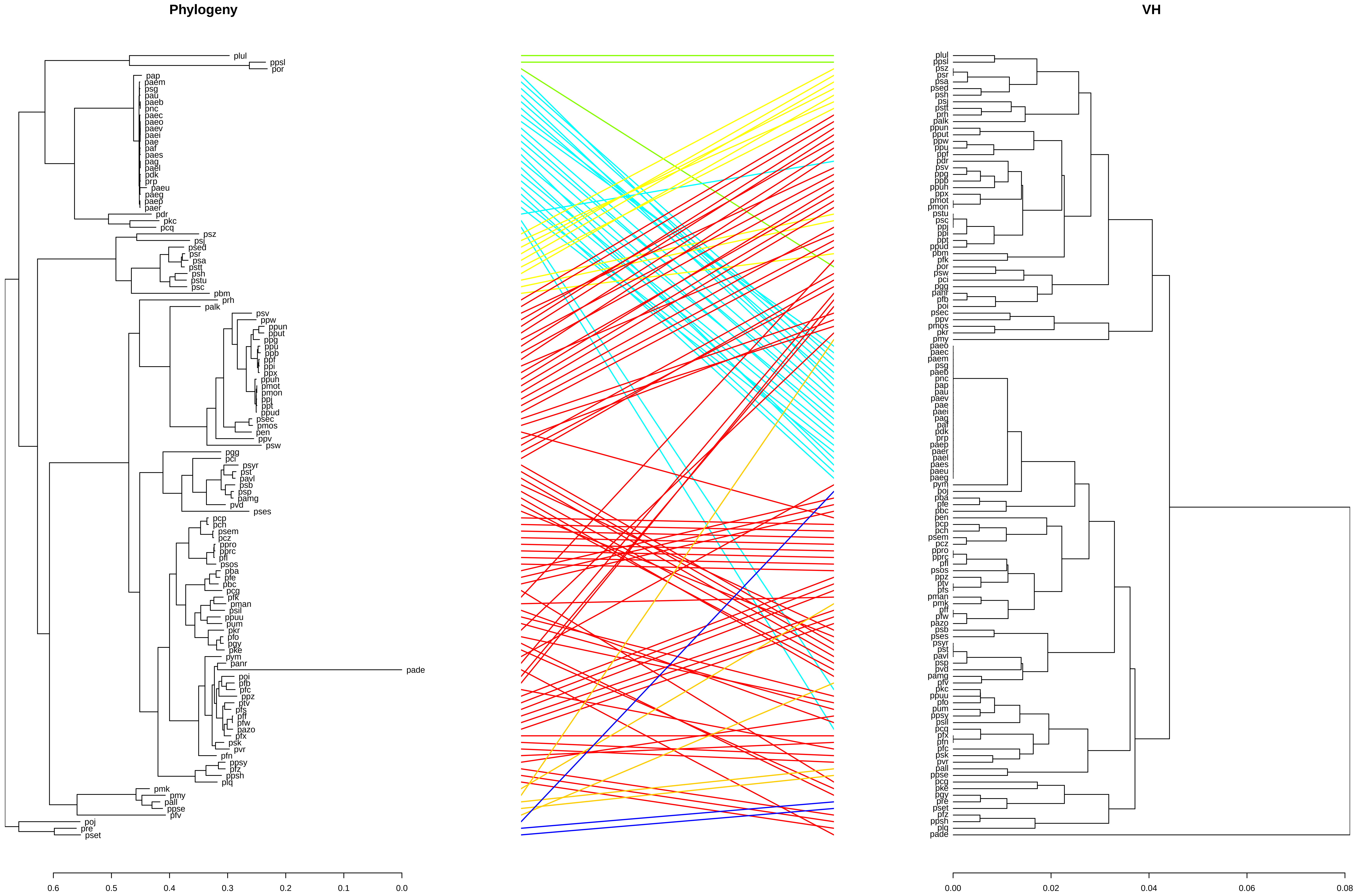
Phylogeny
VH
plul
ppsl
por
pap
paem
psg
pau
paeb
pnc
paec
paeo
paev
paei
pae
paf
paes
pag
pael
pdk
prp
paeu
paeg
paep
paer
pdr
pkc
pcq
psz
psj
psed
psr
psa
pstt
psh
pstu
psc
pbm
prh
palk
psv
ppw
ppun
pput
ppg
ppu
ppb
ppf
ppi
ppx
ppuh
pmot
pmon
ppj
ppt
ppud
psec
pmos
pen
ppv
psw
pgg
pci
psyr
pst
pavl
psb
psp
pamg
pvd
pses
pcp
pch
psem
pcz
ppro
pprc
pfl
psos
pba
pfe
pbc
pcg
pfk
pman
psil
ppuu
pum
pkr
pfo
pgy
pke
pym
panr
pade
poi
pfb
pfc
ppz
ptv
pfs
pff
pfw
pazo
pfx
psk
pvr
pfn
ppsy
pfz
ppsh
plq
pmk
pmy
pall
ppse
pfv
poj
pre
pset
0.6
0.5
0.4
0.3
0.2
0.1
0.0
plul
ppsl
psz
psr
psa
psed
psh
psj
pstt
prh
palk
ppun
pput
ppw
ppu
ppf
pdr
psv
ppg
ppb
ppuh
ppx
pmot
pmon
pstu
psc
ppj
ppi
ppt
ppud
pbm
pfk
por
psw
pci
pgg
panr
pfb
poi
psec
ppv
pmos
pkr
pmy
paeo
paec
paem
psg
paeb
pnc
pap
pau
paev
pae
paei
pag
paf
pdk
prp
paep
paer
pael
paes
paeu
paeg
pym
poj
pba
pfe
pbc
pen
pcp
pch
psem
pcz
ppro
pprc
pfl
psos
ppz
ptv
pfs
pman
pmk
pff
pfw
pazo
psb
pses
psyr
pst
pavl
psp
pvd
pamg
pfv
pkc
ppuu
pfo
pum
ppsy
psil
pcq
pfx
pfn
pfc
psk
pvr
pall
ppse
pcg
pke
pgy
pre
pset
pfz
ppsh
plq
pade
0.00
0.02
0.04
0.06
0.08

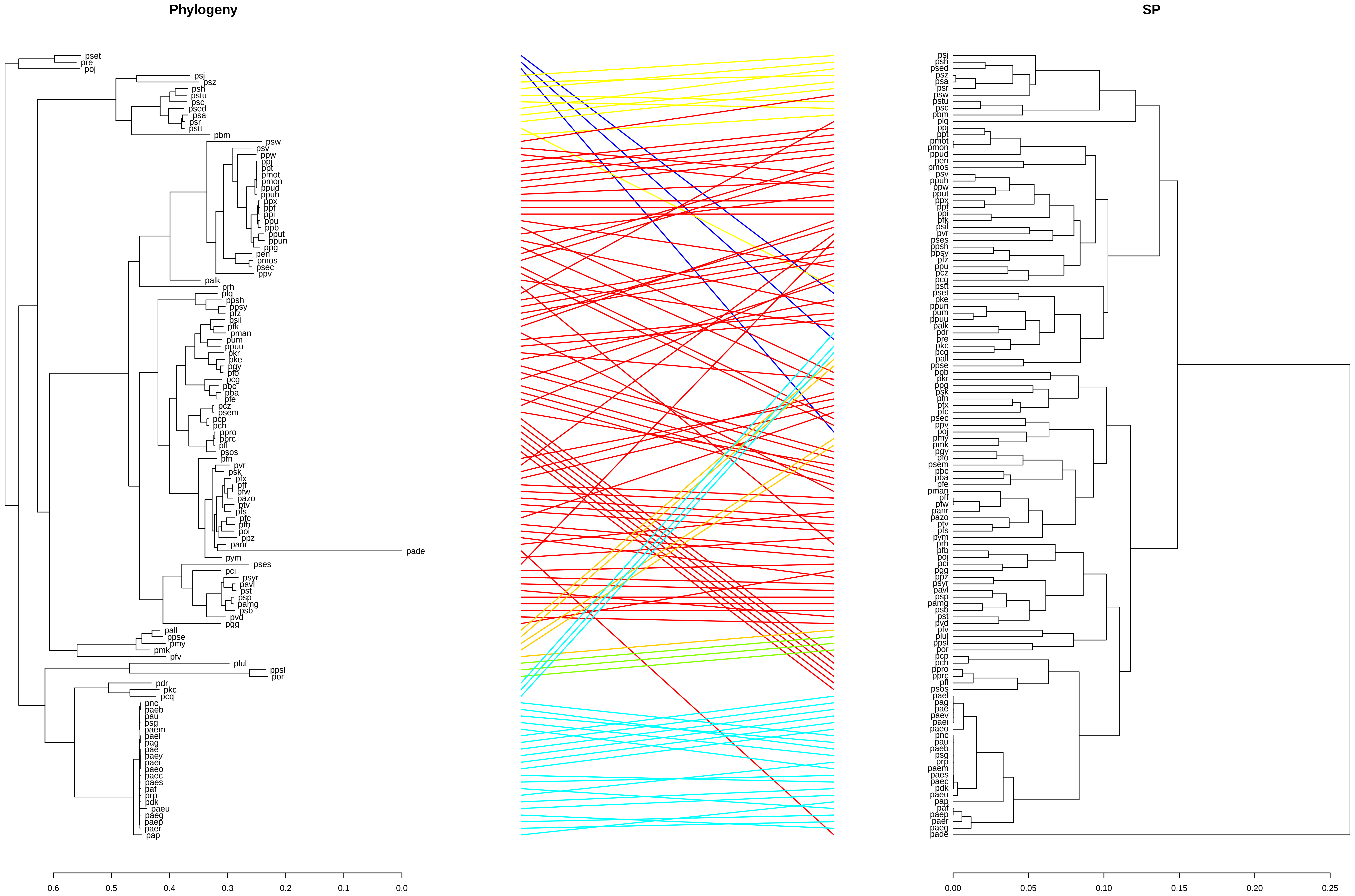
Phylogeny
SP
pset
pre
poj
psj
psz
psh
pstu
psc
psed
psa
psr
pstt
pbm
psw
psv
ppw
ppj
ppt
pmot
pmon
ppud
ppuh
ppx
ppf
ppi
ppu
ppb
pput
ppun
ppg
pen
pmos
psec
ppv
palk
prh
plq
ppsh
ppsy
pfz
psil
pfk
pman
pum
ppuu
pkr
pke
pgy
pfo
pcg
pbc
pba
pfe
pcz
psem
pcp
pch
ppro
pprc
pfl
psos
pfn
pvr
psk
pfx
pff
pfw
pazo
ptv
pfs
pfc
pfb
poi
ppz
panr
pade
pym
pses
pci
psyr
pavl
pst
psp
pamg
psb
pvd
pgg
pall
ppse
pmy
pmk
pfv
plul
ppsl
por
pdr
pkc
pcq
pnc
paeb
pau
psg
paem
pael
pag
pae
paev
paei
paeo
paec
paes
paf
prp
pdk
paeu
paeg
paep
paer
pap
0.6
0.5
0.4
0.3
0.2
0.1
0.0
psj
psh
psed
psz
psa
psr
psw
pstu
psc
pbm
plq
ppj
ppt
pmot
pmon
ppud
pen
pmos
psv
ppuh
ppw
pput
ppx
ppf
ppi
pfk
psil
pvr
pses
ppsh
ppsy
pfz
ppu
pcz
pcg
pstt
pset
pke
ppun
pum
ppuu
palk
pdr
pre
pkc
pcq
pall
ppse
ppb
pkr
ppg
psk
pfn
pfx
pfc
psec
ppv
poj
pmy
pmk
pgy
pfo
psem
pbc
pba
pfe
pman
pff
pfw
panr
pazo
ptv
pfs
pym
prh
pfb
poi
pci
pgg
ppz
psyr
pavl
psp
pamg
psb
pst
pvd
pfv
plul
ppsl
por
pcp
pch
ppro
pprc
pfl
psos
pael
pag
pae
paev
paei
paeo
pnc
pau
paeb
psg
prp
paem
paes
paec
pdk
paeu
pap
paf
paep
paer
paeg
pade
0.00
0.05
0.10
0.15
0.20
0.25

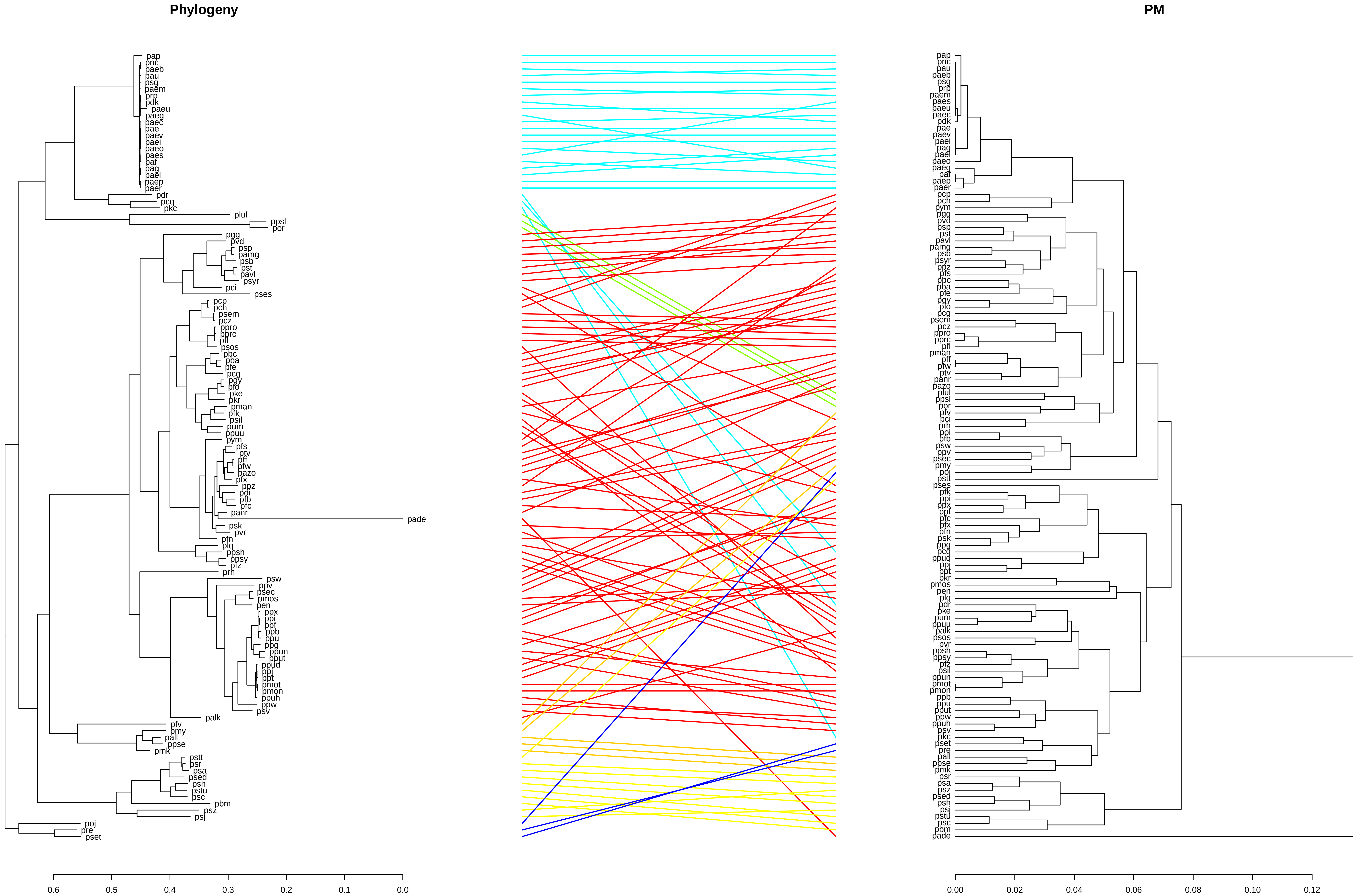
Phylogeny
PM
pap
pnc
paeb
pau
psg
paem
prp
pdk
paeu
paeg
paec
pae
paev
paei
paeo
paes
paf
pag
pael
paep
paer
pdr
pcq
pkc
plul
ppsl
por
pgg
pvd
psp
pamg
psb
pst
pavl
psyr
pci
pses
pcp
pch
psem
pcz
ppro
pprc
pfl
psos
pbc
pba
pfe
pcg
pgy
pfo
pke
pkr
pman
pfk
psil
pum
ppuu
pym
pfs
ptv
pff
pfw
pazo
pfx
ppz
poi
pfb
pfc
panr
pade
psk
pvr
pfn
plq
ppsh
ppsy
pfz
prh
psw
ppv
psec
pmos
pen
ppx
ppi
ppf
ppb
ppu
ppg
ppun
pput
ppud
ppj
ppt
pmot
pmon
ppuh
ppw
psv
palk
pfv
pmy
pall
ppse
pmk
pstt
psr
psa
psed
psh
pstu
psc
pbm
psz
psj
poj
pre
pset
0.6
0.5
0.4
0.3
0.2
0.1
0.0
pap
pnc
pau
paeb
psg
prp
paem
paes
paeu
paec
pdk
pae
paev
paei
pag
pael
paeo
paeg
paf
paep
paer
pcp
pch
pym
pgg
pvd
psp
pst
pavl
pamg
psb
psyr
ppz
pfs
pbc
pba
pfe
pgy
pfo
pcg
psem
pcz
ppro
pprc
pfl
pman
pff
pfw
ptv
panr
pazo
plul
ppsl
por
pfv
pci
prh
poi
pfb
psw
ppv
psec
pmy
poj
pstt
pses
pfk
ppi
ppx
ppf
pfc
pfx
pfn
psk
ppg
pcq
ppud
ppj
ppt
pkr
pmos
pen
plq
pdr
pke
pum
ppuu
palk
psos
pvr
ppsh
ppsy
pfz
psil
ppun
pmot
pmon
ppb
ppu
pput
ppw
ppuh
psv
pkc
pset
pre
pall
ppse
pmk
psr
psa
psz
psed
psh
psj
pstu
psc
pbm
pade
0.00
0.02
0.04
0.06
0.08
0.10
0.12

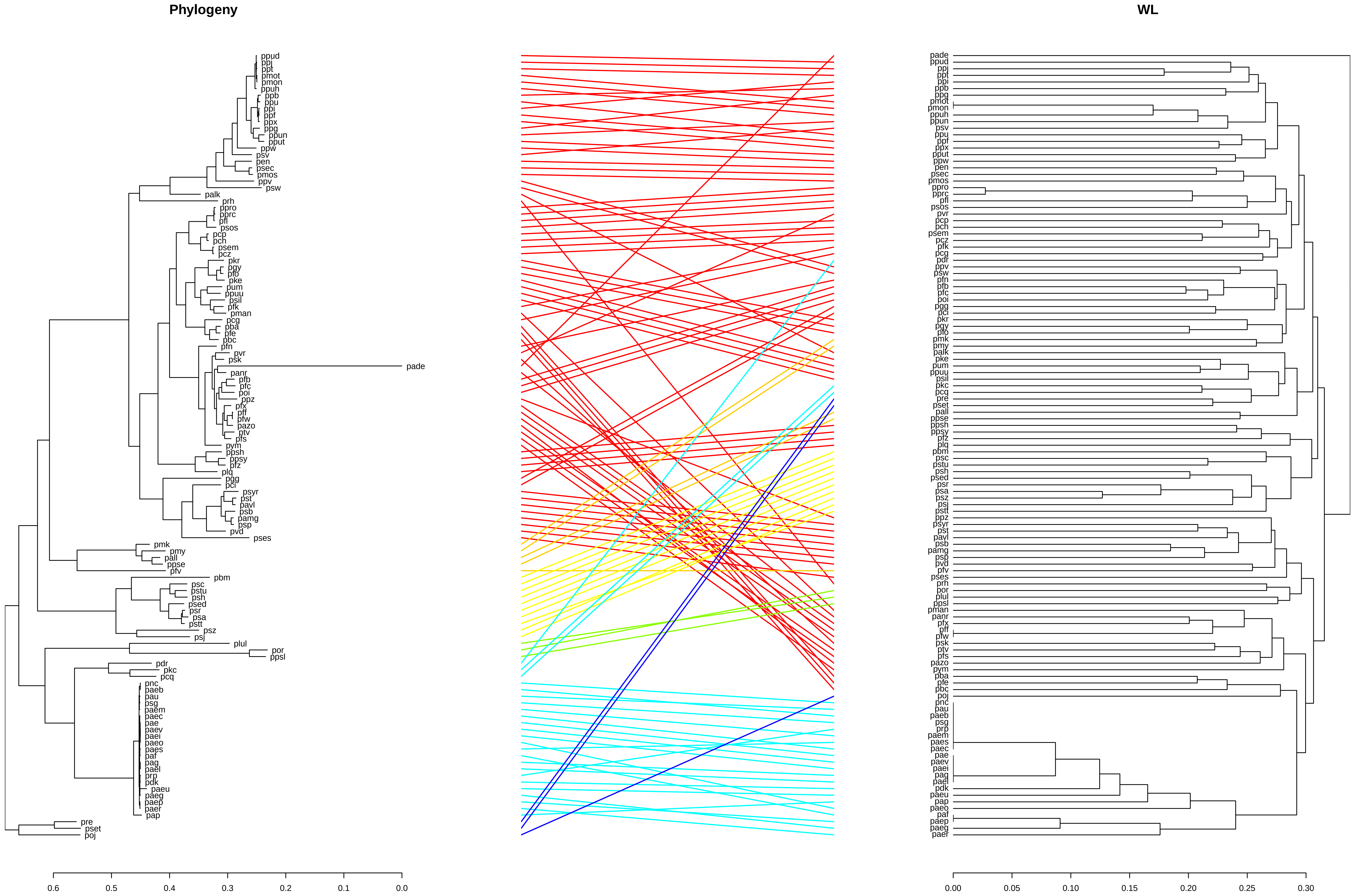
Phylogeny
ppud
ppj
ppt
pmot
pmon
ppuh
ppb
ppu
ppi
ppf
ppx
ppg
ppun
pput
ppw
psv
pen
psec
pmos
ppv
psw
palk
prh
ppro
pprc
pfl
psos
pcp
pch
psem
pcz
pkr
pgy
pfo
pke
pum
ppuu
psil
pfk
pman
pcg
pba
pfe
pbc
pfn
pvr
psk
pade
panr
pfb
pfc
poi
ppz
pfx
pff
pfw
pazo
ptv
pfs
pym
ppsh
ppsy
pfz
plq
pgg
pci
psyr
pst
pavl
psb
pamg
psp
pvd
pses
pmk
pmy
pall
ppse
pfv
pbm
psc
pstu
psh
psed
psr
psa
pstt
psz
psj
plul
por
ppsl
pdr
pkc
pcq
pnc
paeb
pau
psg
paem
paec
pae
paev
paei
paeo
paes
paf
pag
pael
prp
pdk
paeu
paeg
paep
paer
pap
pre
pset
poj
0.6
0.5
0.4
0.3
0.2
0.1
0.0
WL
pade
ppud
ppj
ppt
ppi
ppb
ppg
pmot
pmon
ppuh
ppun
psv
ppu
ppf
ppx
pput
ppw
pen
psec
pmos
ppro
pprc
pfl
psos
pvr
pcp
pch
psem
pcz
pfk
pcg
pdr
ppv
psw
pfn
pfb
pfc
poi
pgg
pci
pkr
pgy
pfo
pmk
pmy
palk
pke
pum
ppuu
psil
pkc
pcq
pre
pset
pall
ppse
ppsh
ppsy
pfz
plq
pbm
psc
pstu
psh
psed
psr
psa
psz
psj
pstt
ppz
psyr
pst
pavl
psb
pamg
psp
pvd
pfv
pses
prh
por
plul
ppsl
pman
panr
pfx
pff
pfw
psk
ptv
pfs
pazo
pym
pba
pfe
pbc
poj
pnc
pau
paeb
psg
prp
paem
paes
paec
pae
paev
paei
pag
pael
pdk
paeu
pap
paeo
paf
paep
paeg
paer
0.00
0.05
0.10
0.15
0.20
0.25
0.30
