# Supplementary material for: Exploring the expressiveness of abstract metabolic networks
Source: PLoS One. 2023 Feb 9;18(2):e0281047. doi: 10.1371/journal.pone.0281047 (PMC9910719; doi:10.1371/journal.pone.0281047)
Supplement: S13 File — Analyses of the considered experiments after removing the pathways harbouring only one reaction (threshold 1) or at most two reactions (threshold 2). The results can be directly compared with the ones presented in the Results section. (PDF) [file pone.0281047.s013.pdf]

Whole Dataset Analysis

Different thresholds

- Vertex hystogram (VH) kernel
  - Heatmap of the original matrix
  - Heatmaps with threshold 1 (left side) and threshold 2 (right side)
  - 6-means clusters for the original matrix
  - 6-means clusters for threshold 1 matrix
  - 6-means clusters for threshold 2 matrix
- Shortest Path (SP) kernel
  - Heatmap of the original matrix
  - Heatmaps with threshold 1 (left side) and threshold 2 (right side)
  - 6-means clusters for the original matrix
  - 6-means clusters for threshold 1 matrix
  - 6-means clusters for threshold 2 matrix
- Weisfeiler-Lehman (WL) kernel
  - Heatmap of the original matrix
  - Heatmaps with threshold 1 (left side) and threshold 2 (right side)
  - 6-means clusters for the original matrix
  - 6-means clusters for threshold 1 matrix
  - 6-means clusters for threshold 2 matrix
- Pyramid match (PM) kernel
  - Heatmap of the original matrix
  - Heatmaps with threshold 1 (left side) and threshold 2 (right side)
  - 6-means clusters for the original matrix
  - 6-means clusters for threshold 1 matrix
  - 6-means clusters for threshold 2 matrix

Vertex hystogram (VH) kernel

Heatmap of the original matrix

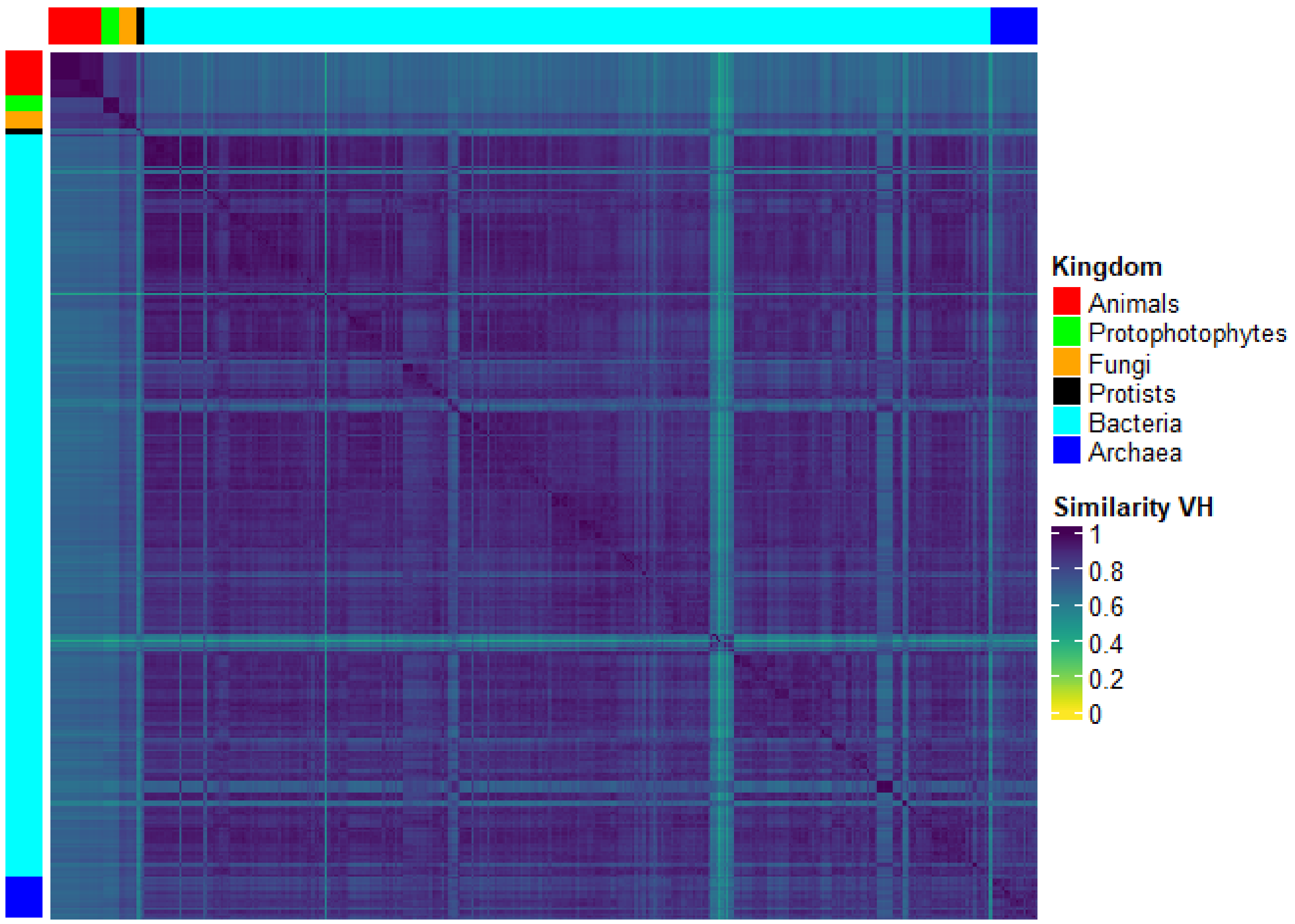

Heatmaps with threshold 1 (left side) and threshold 2 (right side)

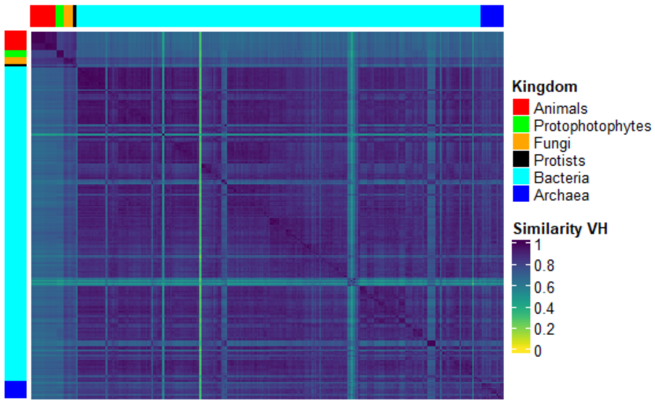

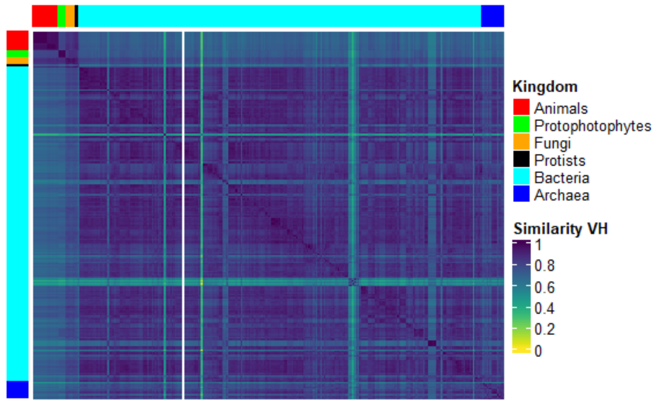

- Threshold 1: for each organism, pathways containing only one reaction are not included in the corresponding abstract metabolic network.
- Threshold 2: for each organism, pathways with only one or two reactions are not included in the corresponding abstract metabolic network.

6-means clusters for the original matrix

```
##                    Cluster
## Real group            1    2    3    4    5    6
##   Animals             0    0    1    0    0  369
##   Archaea             0  100    0   13  226    0
```

```
##    Bacteria          2501 1583    0  559 1472    0
##    Fungi                0    0  138    0    0    0
##    Protists             0    0   47    5    0    0
##    Protophotophytes     0    0  127    0    0    0
```

6-means clusters for threshold 1 matrix

```
##                     Cluster
## Real group             1    2    3    4    5    6
##    Animals             0    0    1    0    0  369
##    Archaea             0  100    0   13  226    0
##    Bacteria          2512 1576    0  561 1466    0
##    Fungi                0    0  138    0    0    0
##    Protists             0    0   47    5    0    0
##    Protophotophytes     0    0  127    0    0    0
```

6-means clusters for threshold 2 matrix

```
##                     Cluster
## Real group             1    2    3    4    5    6
##    Animals             0    0    1    0    0  369
##    Archaea             0  101    0   68  170    0
##    Bacteria          2358 1568    0  699 1487    0
##    Fungi                0    0  138    0    0    0
##    Protists             0    0   44    8    0    0
##    Protophotophytes     0    0  127    0    0    0
```

Shortest Path (SP) kernel

Heatmap of the original matrix

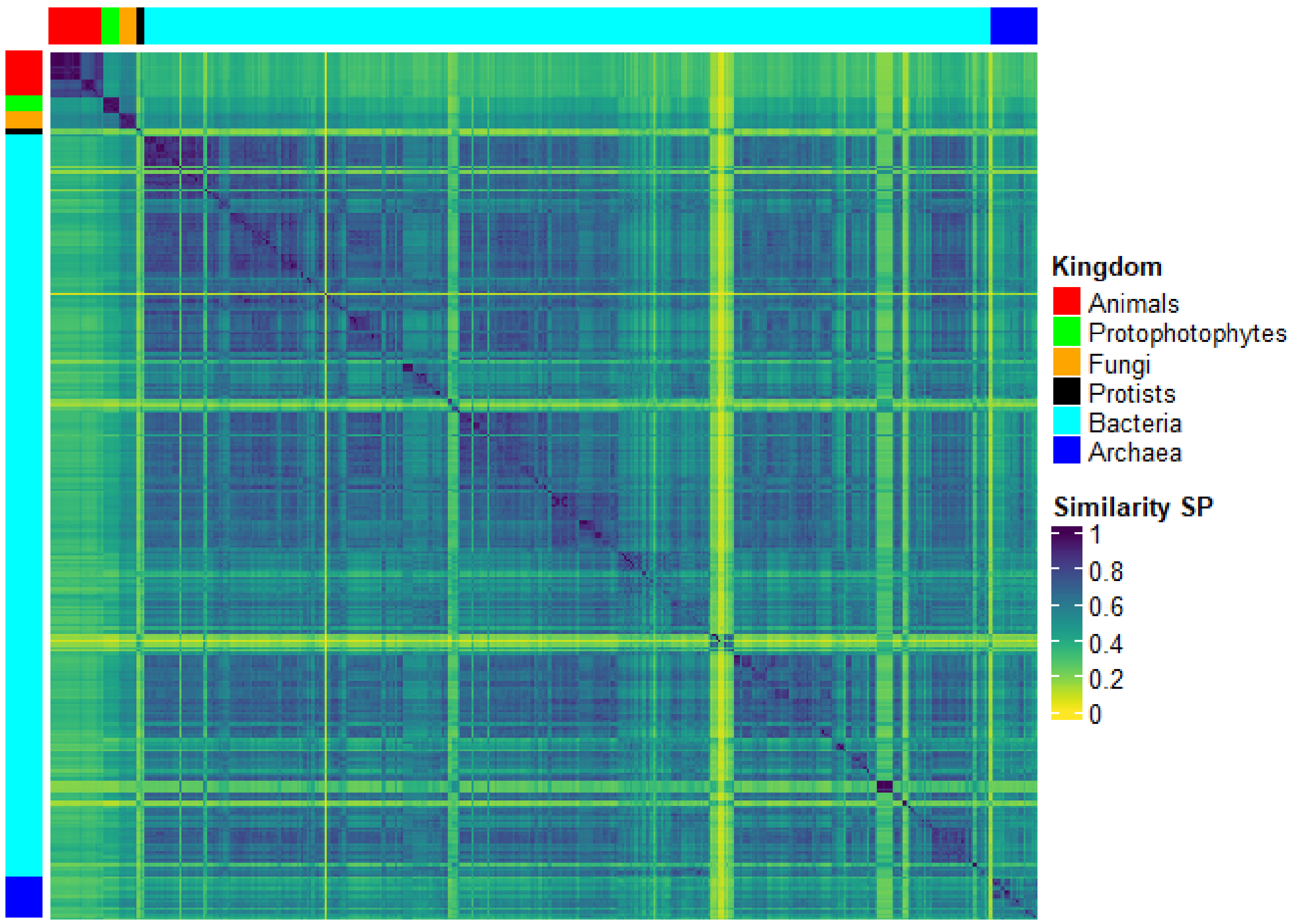

Heatmaps with threshold 1 (left side) and threshold 2 (right side)

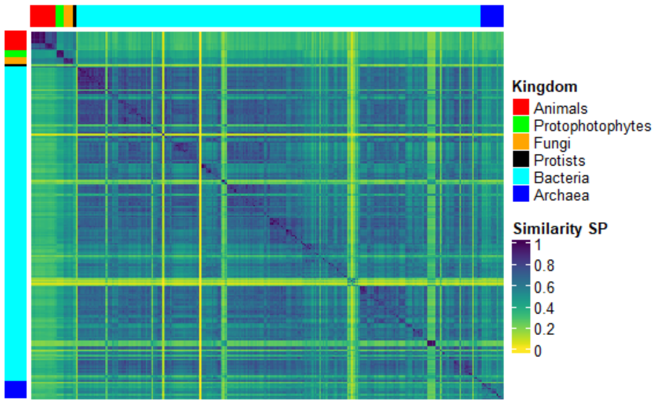

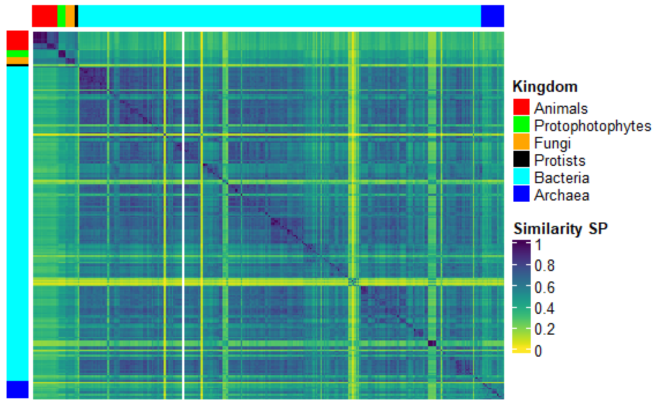

- Threshold 1: for each organism, pathways containing only one reaction are not included in the corresponding abstract metabolic network.
- Threshold 2: for each organism, pathways with only one or two reactions are not included in the corresponding abstract metabolic network.

6-means clusters for the original matrix

```
##                     Cluster
## Real group              1    2    3    4    5    6
##    Animals              1    0    4    0    0  365
##    Archaea             60   96    0  180    3    0
```

```
##    Bacteria           683 1425    0 1695 2312    0
##    Fungi                7    0  131    0    0    0
##    Protists            15    0   37    0    0    0
##    Protophotophytes     0    0  127    0    0    0
```

6-means clusters for threshold 1 matrix

```
##                      Cluster
## Real group              1    2    3    4    5    6
##    Animals              0    4    0    0  365    1
##    Archaea             95    0  180    3    0   61
##    Bacteria          1429    0 1710 2292    0  684
##    Fungi                0  131    0    0    0    7
##    Protists             0   37    0    0    0   15
##    Protophotophytes     0  127    0    0    0    0
```

6-means clusters for threshold 2 matrix

```
##                      Cluster
## Real group              1    2    3    4    5    6
##    Animals              1  363    0    6    0    0
##    Archaea             74    0   99    0  161    5
##    Bacteria           703    0 1435    0 1500 2474
##    Fungi                7    0    0  131    0    0
##    Protists            24    0    0   28    0    0
##    Protophotophytes     0    0    0  127    0    0
```

Weisfeiler-Lehman (WL) kernel

Heatmap of the original matrix

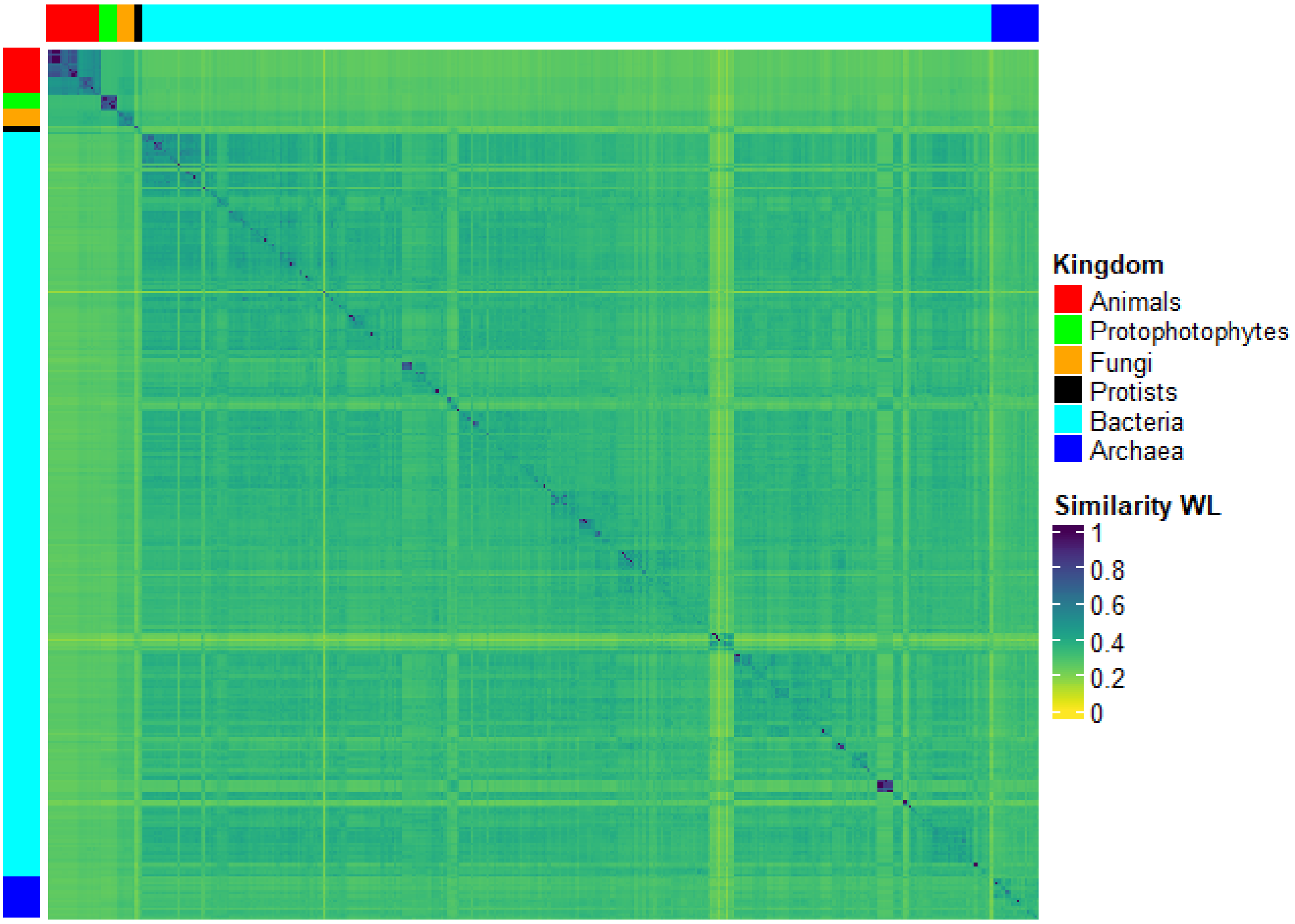

Heatmaps with threshold 1 (left side) and threshold 2 (right side)

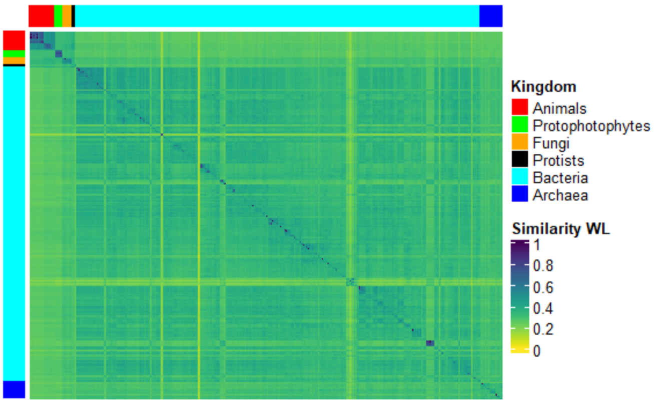

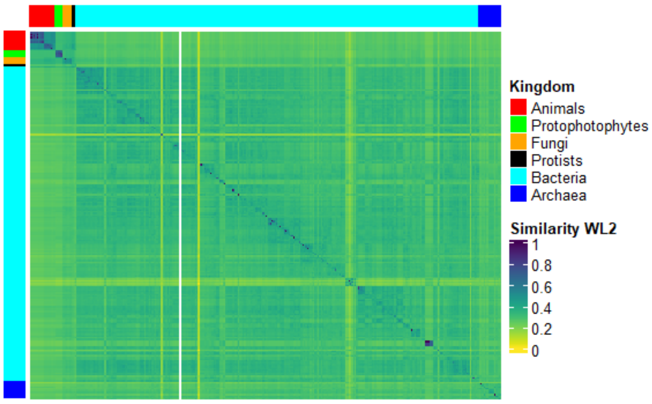

- Threshold 1: for each organism, pathways containing only one reaction are not included in the corresponding abstract metabolic network.
- Threshold 2: for each organism, pathways with only one or two reactions are not included in the corresponding abstract metabolic network.

6-means clusters for the original matrix

```
##                    Cluster
## Real group            1    2    3    4    5    6
##   Animals             0    0   19    0    0  351
##   Archaea           291    4    0    0   44    0
```

```
##    Bacteria          1683  430     0 2441 1561    0
##    Fungi                2    0   136    0    0    0
##    Protists             1    3    48    0    0    0
##    Protophotophytes     0    0   127    0    0    0
```

6-means clusters for threshold 1 matrix

```
##                     Cluster
## Real group             1    2    3    4    5    6
##    Animals             0   16    0    0    0  354
##    Archaea            43    0    4  292    0    0
##    Bacteria         1566    0  430 1683 2436    0
##    Fungi               0  136    0    2    0    0
##    Protists            0   48    3    1    0    0
##    Protophotophytes    0  127    0    0    0    0
```

6-means clusters for threshold 2 matrix

```
##                     Cluster
## Real group             1    2    3    4    5    6
##    Animals             0   17    0    0  353    0
##    Archaea           107    0  230    0    0    2
##    Bacteria         1605    0 1397 2777    0  333
##    Fungi               0  133    5    0    0    0
##    Protists            0   36   15    0    0    1
##    Protophotophytes    0  127    0    0    0    0
```

Pyramid match (PM) kernel

Heatmap of the original matrix

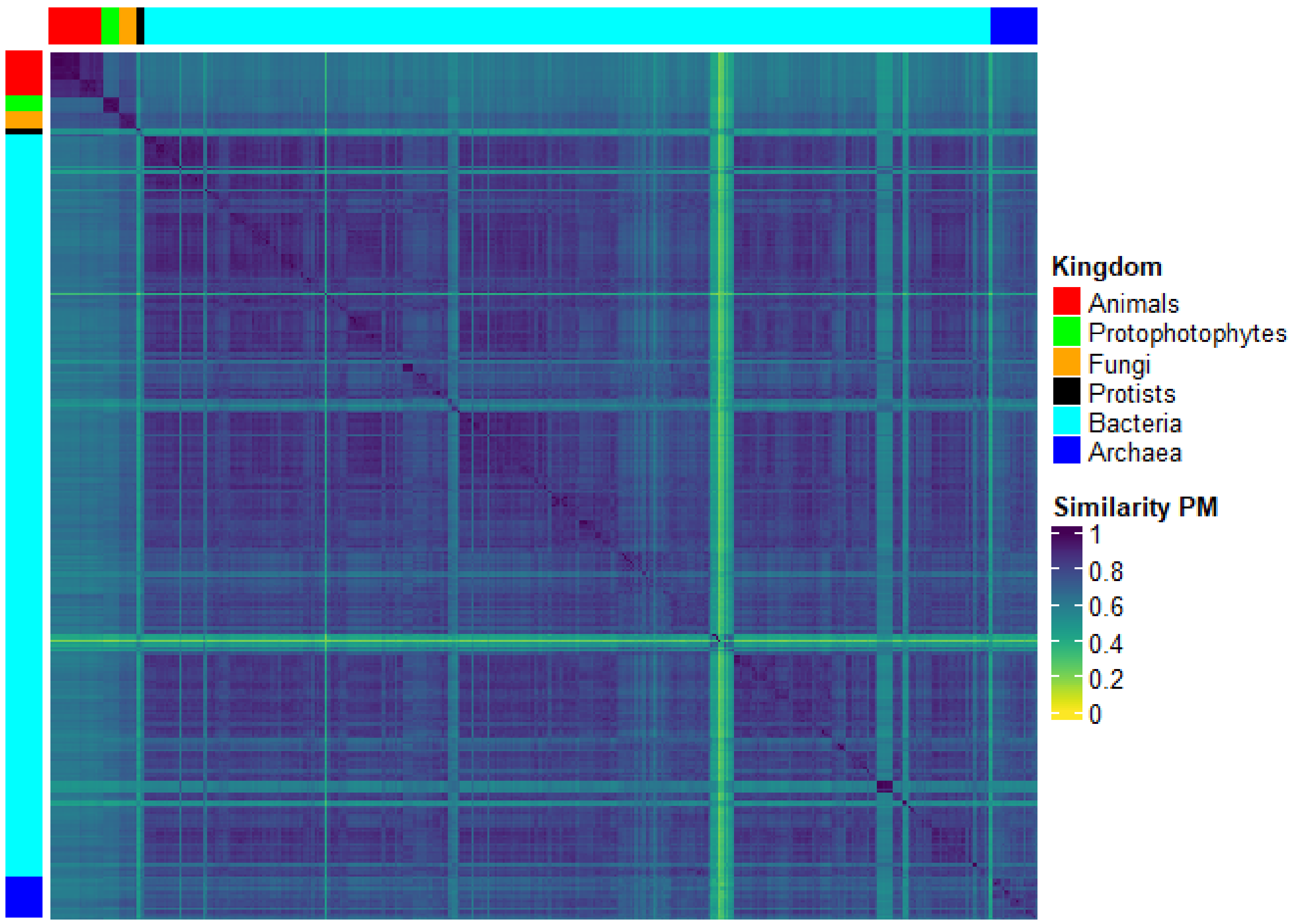

Heatmaps with threshold 1 (left side) and threshold 2 (right side)

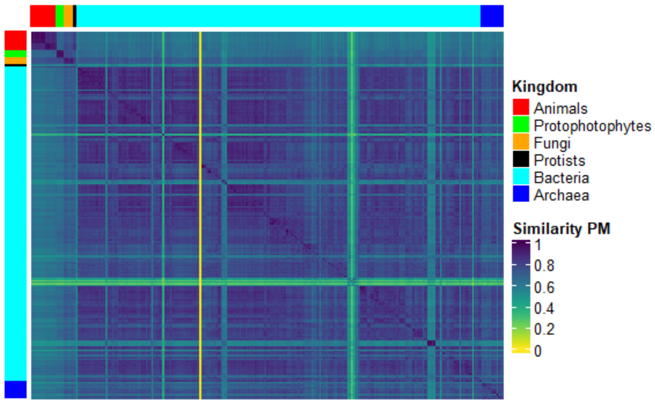

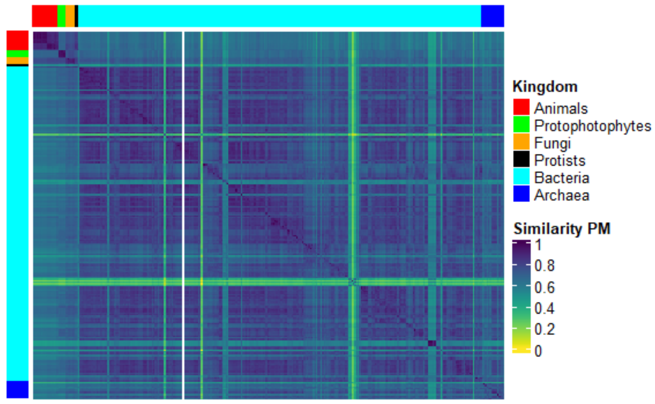

- Threshold 1: for each organism, pathways containing only one reaction are not included in the corresponding abstract metabolic network.
- Threshold 2: for each organism, pathways with only one or two reactions are not included in the corresponding abstract metabolic network.

6-means clusters for the original matrix

```
##                     Cluster
## Real group              1    2    3    4    5    6
##    Animals              3    0    0  367    0    0
##    Archaea              0   12    5    0  174  148
```

```
##    Bacteria             0  632 2553    0 1528 1402
##    Fungi              133    5    0    0    0    0
##    Protists            42   10    0    0    0    0
##    Protophotophytes   127    0    0    0    0    0
```

6-means clusters for threshold 1 matrix

```
##                      Cluster
## Real group              1    2    3    4    5    6
##    Animals              0    0    0  367    0    3
##    Archaea             12  151    5    0  171    0
##    Bacteria           634 1395 2561    0 1525    0
##    Fungi                5    0    0    0    0  133
##    Protists            10    0    0    0    0   42
##    Protophotophytes     0    0    0    0    0  127
```

6-means clusters for threshold 2 matrix

```
##                      Cluster
## Real group              1    2    3    4    5    6
##    Animals              0    3    0    0  367    0
##    Archaea            129    0  164   26    0   20
##    Bacteria          1305    0 1390 2855    0  562
##    Fungi                0  133    0    0    0    5
##    Protists             0   38    0    0    0   14
##    Protophotophytes     0  127    0    0    0    0
```

Eukaryotes Analysis

Different thresholds

- Vertex hystogram (VH) kernel
  - Heatmap for the original matrix
  - Heatmaps with threshold 1 (left side) and threshold 2 (right side)
  - 4-means clusters for the original matrix
  - 4-means clusters for threshold 1 matrix
  - 4-means clusters for threshold 2 matrix
- Shortest path (SP) kernel
  - Heatmap for the original matrix
  - Heatmaps with threshold 1 (left side) and threshold 2 (right side)
  - 4-means clusters for the original matrix
  - 4-means clusters for threshold 1 matrix
  - 4-means clusters for threshold 2 matrix
- Weisfeiler-Lehman (WL) kernel
  - Heatmap for the original matrix
  - Heatmaps with threshold 1 (left side) and threshold 2 (right side)
  - 4-means clusters for the original matrix
  - 4-means clusters for threshold 1 matrix
  - 4-means clusters for threshold 2 matrix
- Pyramid match (PM) kernel
  - Heatmap for the original matrix
  - Heatmaps with threshold 1 (left side) and threshold 2 (right side)
  - 4-means clusters for the original matrix
  - 4-means clusters for threshold 1 matrix
  - 4-means clusters for threshold 2 matrix

Vertex hystogram (VH) kernel

Heatmap for the original matrix

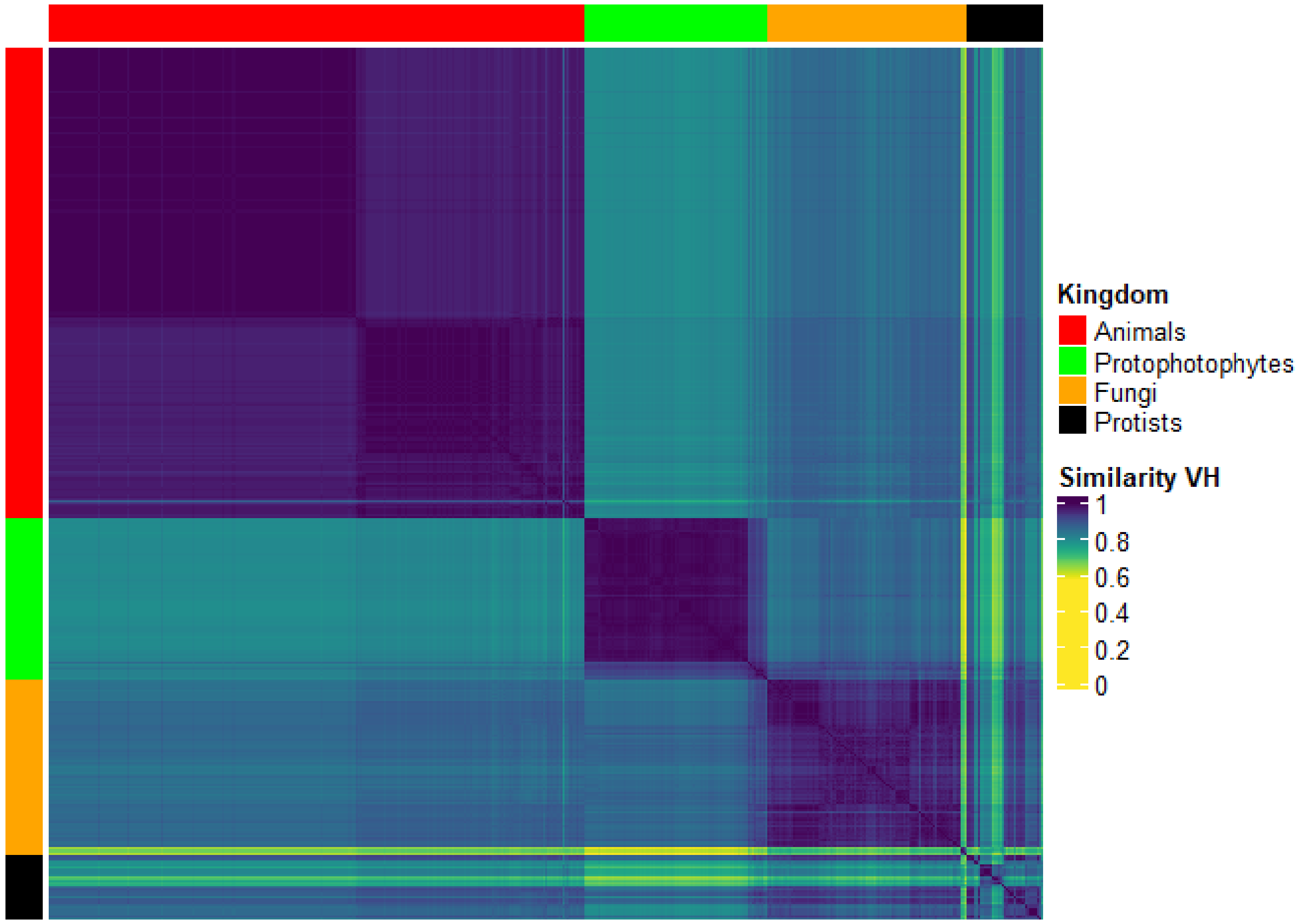

Heatmaps with threshold 1 (left side) and threshold 2 (right side)

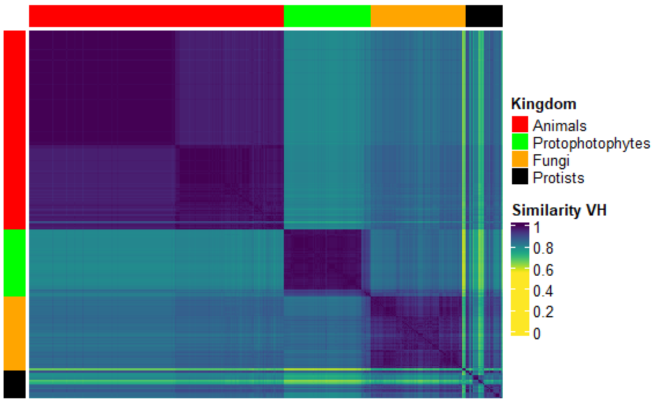

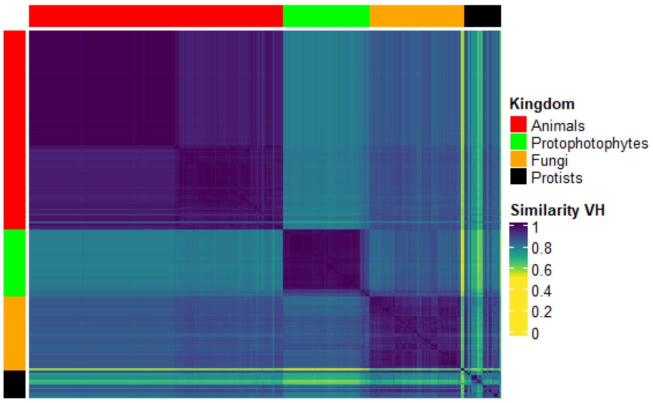

- Threshold 1: for each organism, pathways containing only one reaction are not included in the corresponding abstract metabolic network.
- Threshold 2: for each organism, pathways with only one or two reactions are not included in the corresponding abstract metabolic network.

4-means clusters for the original matrix

```
##                     Cluster
## Real group             1   2   3   4
##    Animals             1   0   0 369
##    Fungi               5   0 133   0
```

```
##   Protists           21   0  31   0
##   Protophotophytes    0 119   8   0
```

4-means clusters for threshold 1 matrix

```
##                     Cluster
## Real group             1   2   3   4
##   Animals              0   0   1 369
##   Fungi                0 133   5   0
##   Protists             0  31  21   0
##   Protophotophytes 120   7   0   0
```

4-means clusters for threshold 2 matrix

```
##                     Cluster
## Real group             1   2   3   4
##   Animals              0 369   0   1
##   Fungi                0   0 133   5
##   Protists             0   0  29  23
##   Protophotophytes 125   0   2   0
```

Shortest path (SP) kernel

Heatmap for the original matrix

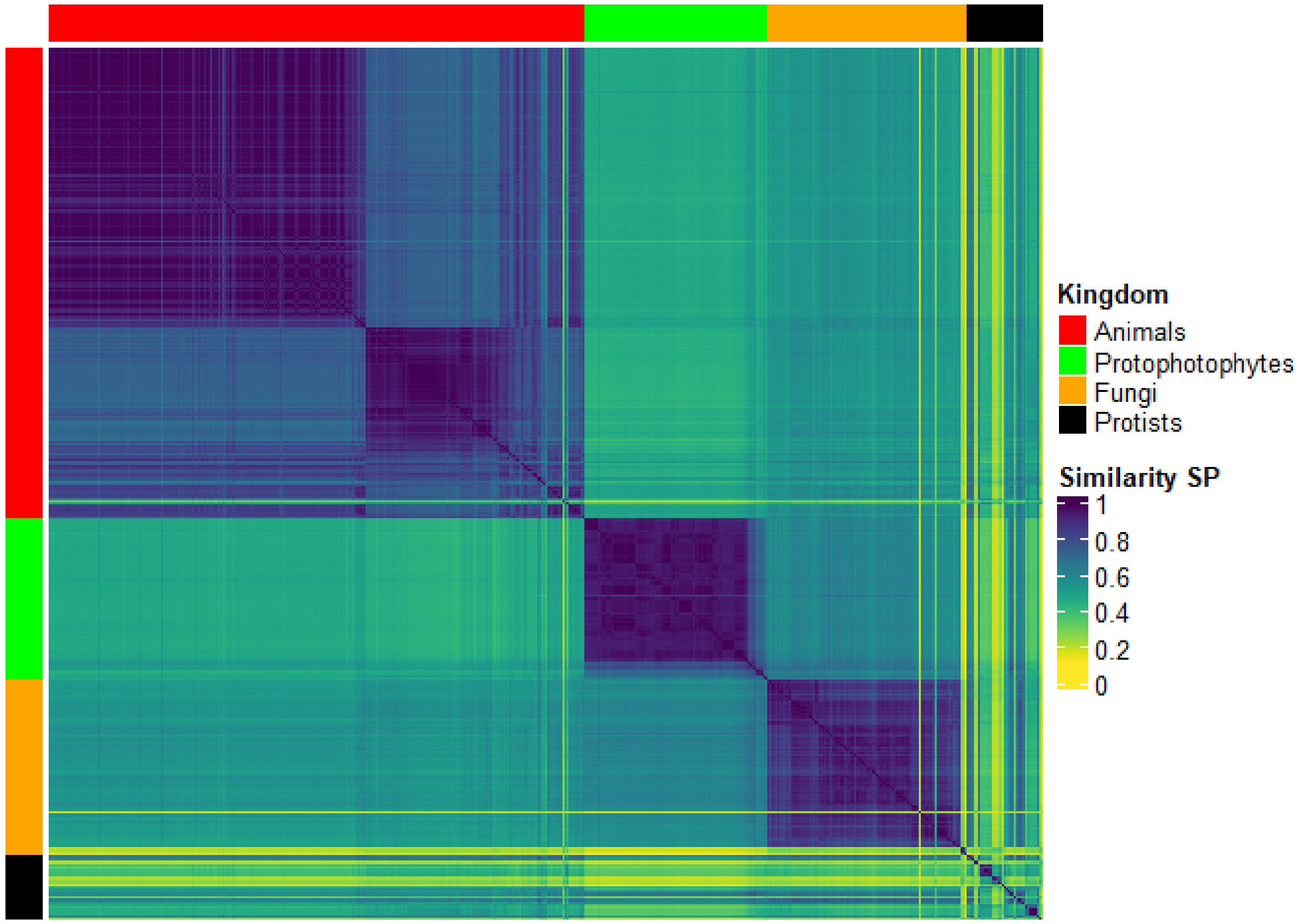

Heatmaps with threshold 1 (left side) and threshold 2 (right side)

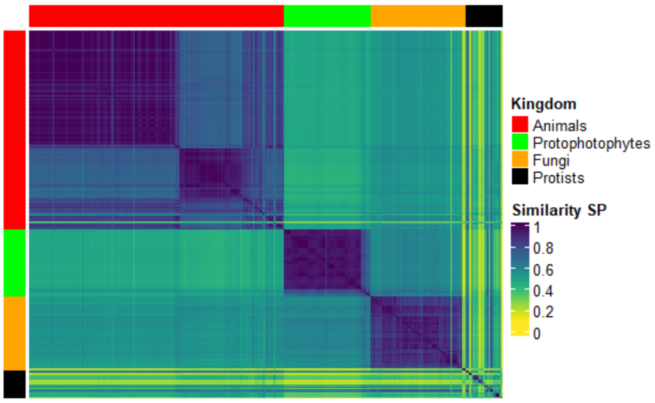

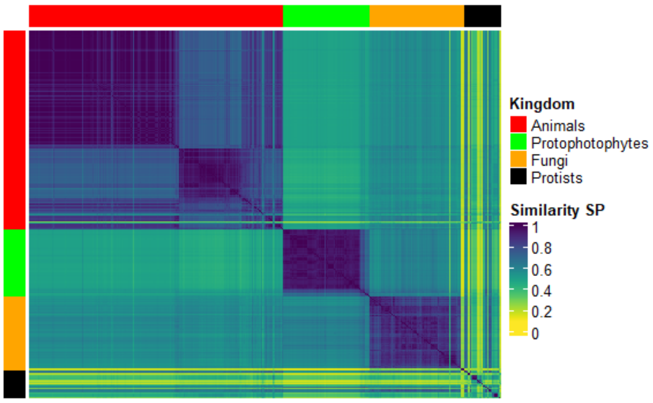

- Threshold 1: for each organism, pathways containing only one reaction are not included in the corresponding abstract metabolic network.
- Threshold 2: for each organism, pathways with only one or two reactions are not included in the corresponding abstract metabolic network.

4-means clusters for the original matrix

```
##                     Cluster
## Real group             1   2   3   4
##   Animals              0   0   3 367
##   Fungi              131   0   7   0
```

```
##   Protists           14   0  35   3
##   Protophotophytes    0 127   0   0
```

4-means clusters for threshold 1 matrix

```
##                     Cluster
## Real group             1   2   3   4
##   Animals              0   0   3 367
##   Fungi              131   0   7   0
##   Protists            14   0  35   3
##   Protophotophytes     0 127   0   0
```

4-means clusters for threshold 2 matrix

```
##                     Cluster
## Real group             1   2   3   4
##   Animals              5   0 365   0
##   Fungi                7 131   0   0
##   Protists            35  17   0   0
##   Protophotophytes     0   1   0 126
```

Weisfeiler-Lehman (WL) kernel

Heatmap for the original matrix

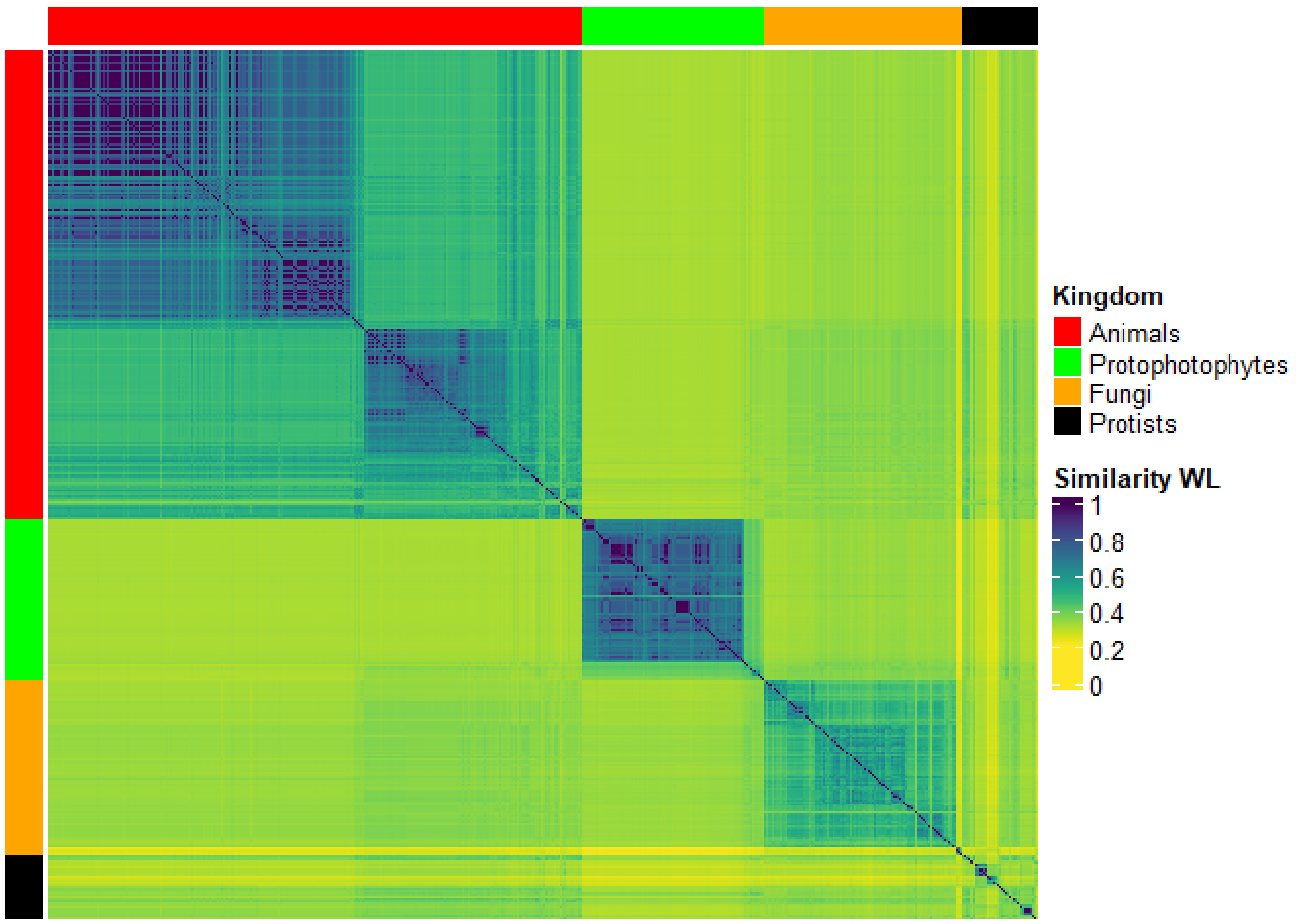

Heatmaps with threshold 1 (left side) and threshold 2 (right side)

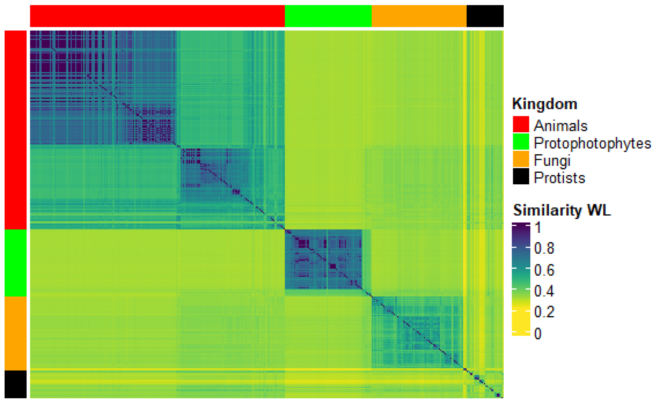

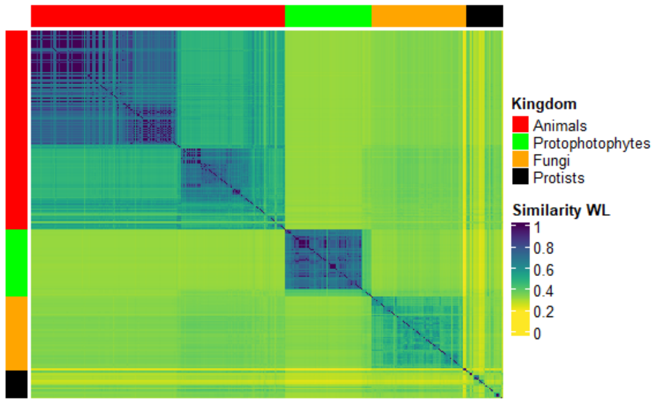

- Threshold 1: for each organism, pathways containing only one reaction are not included in the corresponding abstract metabolic network.
- Threshold 2: for each organism, pathways with only one or two reactions are not included in the corresponding abstract metabolic network.

4-means clusters for the original matrix

```
##                     Cluster
## Real group             1   2   3   4
##    Animals             0 212 157   1
##    Fungi               0   0   0 138
```

```
##    Protists             0    0    0   52
##    Protophotophytes 113    0    0   14
```

4-means clusters for threshold 1 matrix

```
##                         Cluster
## Real group               1    2    3    4
##    Animals               1  212  157    0
##    Fungi               138    0    0    0
##    Protists             52    0    0    0
##    Protophotophytes     14    0    0  113
```

4-means clusters for threshold 2 matrix

```
##                         Cluster
## Real group               1    2    3    4
##    Animals             152    4  214    0
##    Fungi                 0  138    0    0
##    Protists              0   52    0    0
##    Protophotophytes      0   14    0  113
```

Pyramid match (PM) kernel

Heatmap for the original matrix

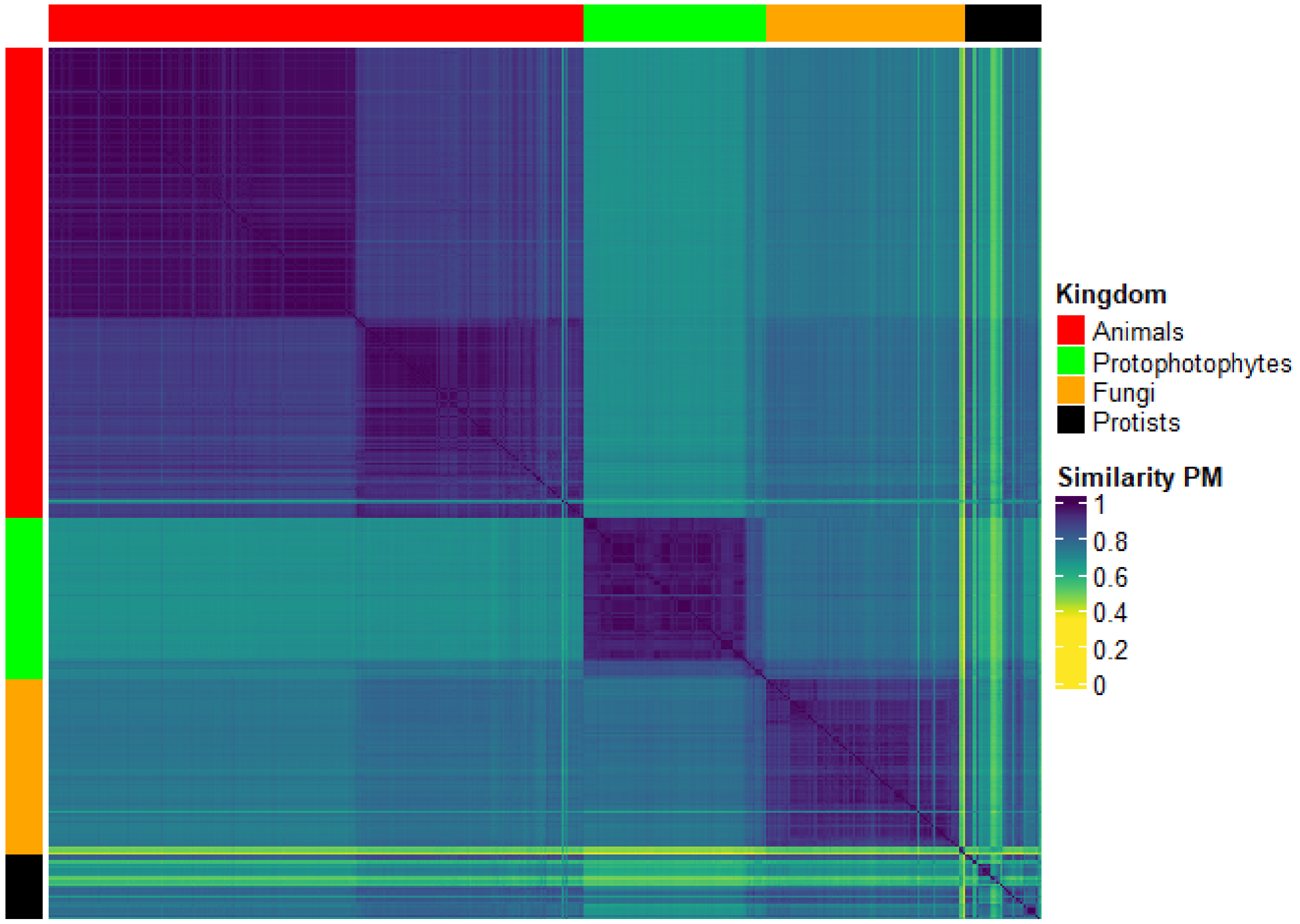

Heatmaps with threshold 1 (left side) and threshold 2 (right side)

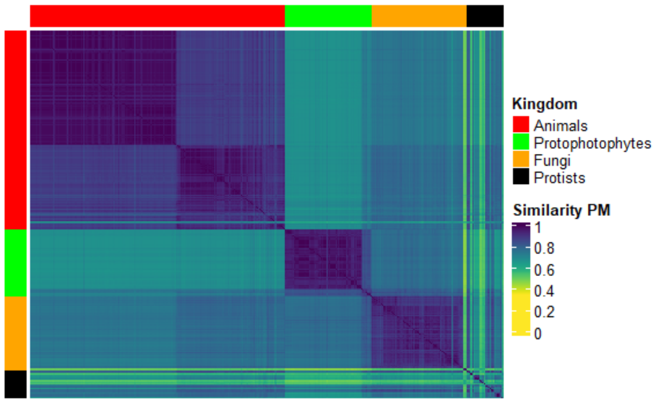

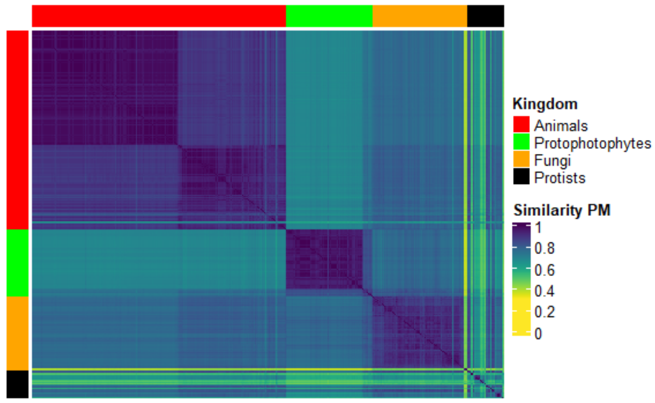

- Threshold 1: for each organism, pathways containing only one reaction are not included in the corresponding abstract metabolic network.
- Threshold 2: for each organism, pathways with only one or two reactions are not included in the corresponding abstract metabolic network.

4-means clusters for the original matrix

```
##                     Cluster
## Real group             1   2   3   4
##    Animals             0   0 368   2
##    Fungi             132   0   0   6
```

```
##    Protists           26   0   1  25
##    Protophotophytes    9 118   0   0
```

4-means clusters for threshold 1 matrix

```
##                      Cluster
## Real group             1   2   3   4
##    Animals             0   0 368   2
##    Fungi             132   0   0   6
##    Protists           26   0   1  25
##    Protophotophytes    9 118   0   0
```

4-means clusters for threshold 2 matrix

```
##                      Cluster
## Real group             1   2   3   4
##    Animals             2   0 368   0
##    Fungi               6 132   0   0
##    Protists           26  26   0   0
##    Protophotophytes    0   9   0 118
```

Animals Phylum Analysis

Different thresholds

- Vertex hystogram (VH) kernel
  - Heatmap of the original matrix
  - Heatmaps with threshold 1 (left side) and threshold 2 (right side)
  - Optimal number of clusters for the original matrix
  - Optimal number of clusters for threshold 1 matrix
  - Optimal number of clusters for threshold 2 matrix
- Shortest path (SP) kernel
  - Heatmap of the original matrix
  - Heatmaps with threshold 1 (left side) and threshold 2 (right side)
  - Optimal number of clusters for the original matrix
  - Optimal number of clusters for threshold 1 matrix
  - Optimal number of clusters for threshold 2 matrix
- Weisfeiler-Lehman (WL) kernel
  - Heatmap of the original matrix
  - Heatmaps with threshold 1 (left side) and threshold 2 (right side)
  - Optimal number of clusters for the original matrix
  - Optimal number of clusters for threshold 1 matrix
  - Optimal number of clusters for threshold 2 matrix
- Pyramid match (PM) kernel
  - Heatmap of the original matrix
  - Heatmaps with threshold 1 (left side) and threshold 2 (right side)
  - Optimal number of clusters for the original matrix
  - Optimal number of clusters for threshold 1 matrix
  - Optimal number of clusters for threshold 2 matrix

Vertex hystogram (VH) kernel

Heatmap of the original matrix

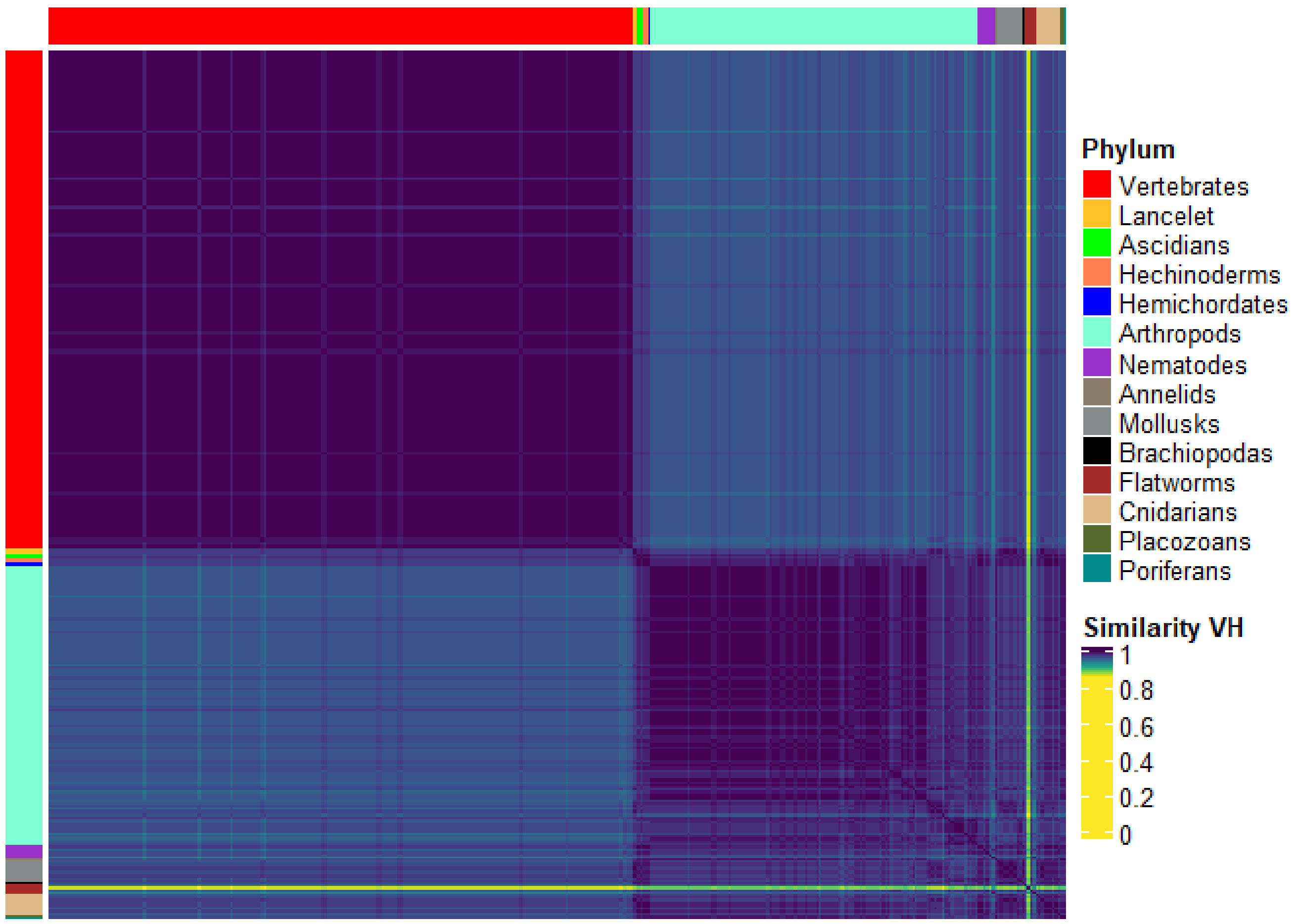

Heatmaps with threshold 1 (left side) and threshold 2 (right side)

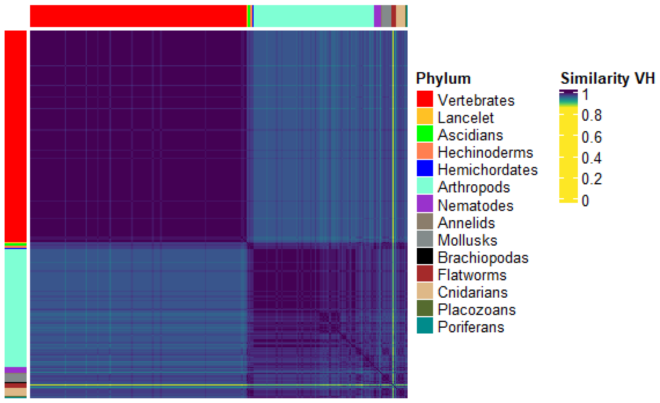

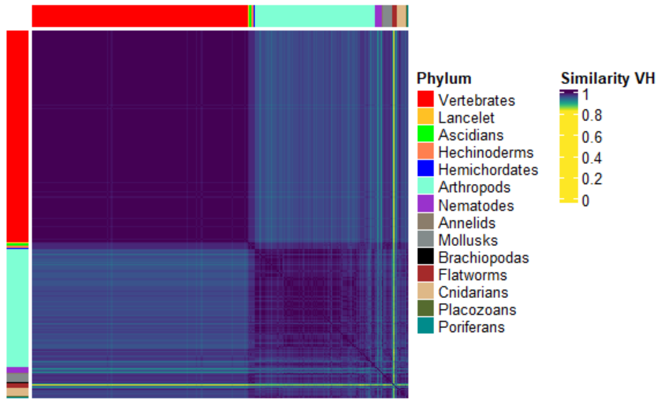

- Threshold 1: for each organism, pathways containing only one reaction are not included in the corresponding abstract metabolic network.
- Threshold 2: for each organism, pathways with only one or two reactions are not included in the corresponding abstract metabolic network.

Optimal number of clusters for the original matrix

```
##                  Cluster
## Real group          1   2   3   4
##   Annelids          1   0   0   0
##   Arthropods       39   0   0  80
```

```
##    Ascidians        2   0   0   0
##    Brachiopodas     1   0   0   0
##    Cnidarians       9   0   0   0
##    Flatworms        2   0   2   0
##    Hechinoderms     2   0   0   0
##    Hemichordates    1   0   0   0
##    Lancelet         2   0   0   0
##    Mollusks         9   0   0   0
##    Nematodes        5   0   1   0
##    Placozoans       1   0   0   0
##    Poriferans       1   0   0   0
##    Vertebrates      0 212   0   0
```

Optimal number of clusters for threshold 1 matrix

```
##                  Cluster
## Real group         1   2   3   4
##    Annelids        0   0   1   0
##    Arthropods      0  81  38   0
##    Ascidians       0   0   2   0
##    Brachiopodas    0   0   1   0
##    Cnidarians      0   0   9   0
##    Flatworms       2   0   2   0
##    Hechinoderms    0   0   2   0
##    Hemichordates   0   0   1   0
##    Lancelet        0   0   2   0
##    Mollusks        0   0   9   0
##    Nematodes       1   0   5   0
##    Placozoans      0   0   1   0
##    Poriferans      0   0   1   0
##    Vertebrates     0   0   0 212
```

Optimal number of clusters for threshold 2 matrix

```
##                  Cluster
## Real group         1   2   3   4
##    Annelids        0   0   1   0
##    Arthropods      0   0  99  20
##    Ascidians       0   0   0   2
##    Brachiopodas    0   0   0   1
##    Cnidarians      0   0   0   9
##    Flatworms       0   3   0   1
##    Hechinoderms    0   0   0   2
##    Hemichordates   0   0   0   1
```

```
##    Lancelet        0   0   0   2
##    Mollusks        0   0   0   9
##    Nematodes       0   2   0   4
##    Placozoans      0   0   1   0
##    Poriferans      0   0   1   0
##    Vertebrates   212   0   0   0
```

Shortest path (SP) kernel

Heatmap of the original matrix

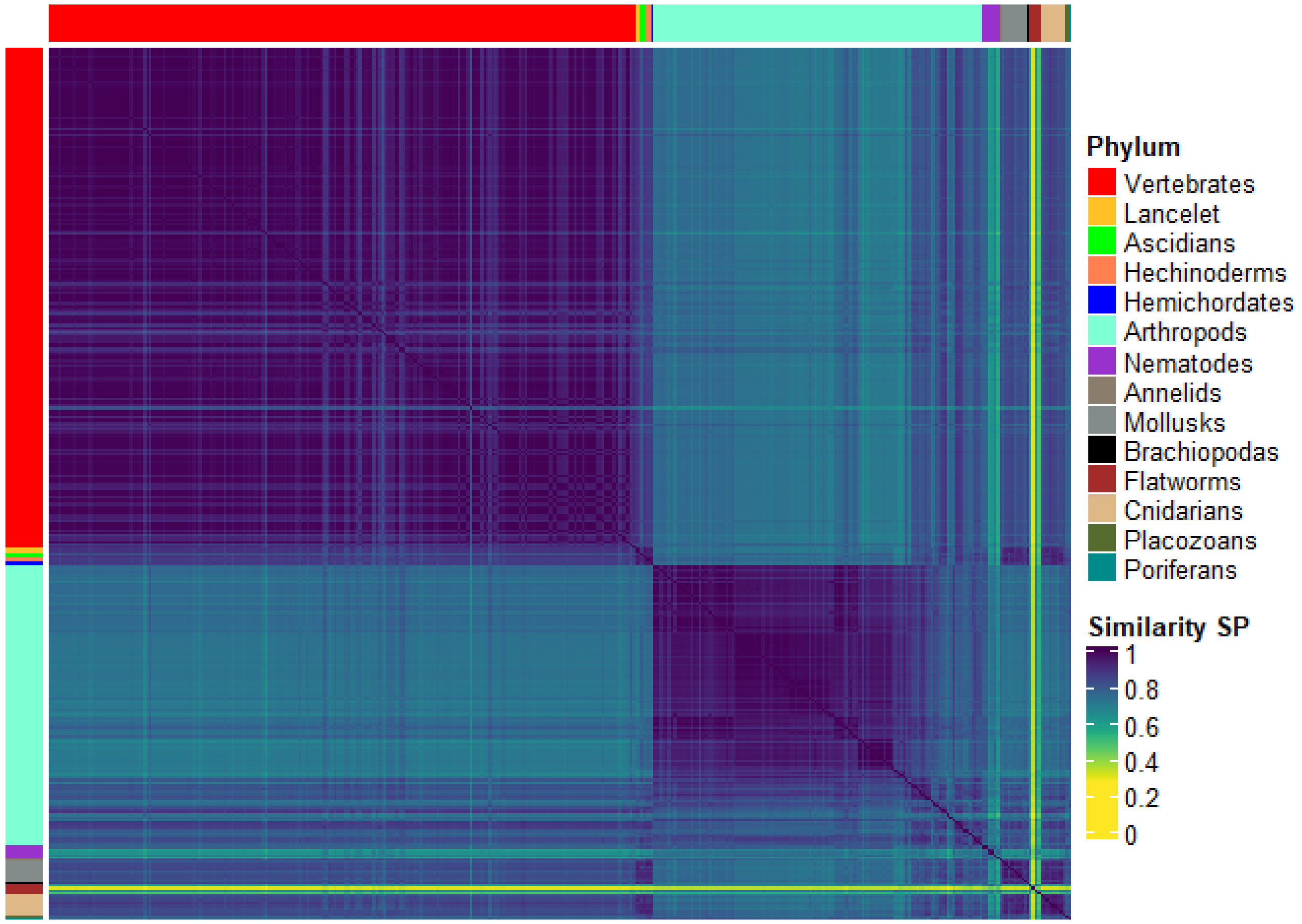

Heatmaps with threshold 1 (left side) and threshold 2 (right side)

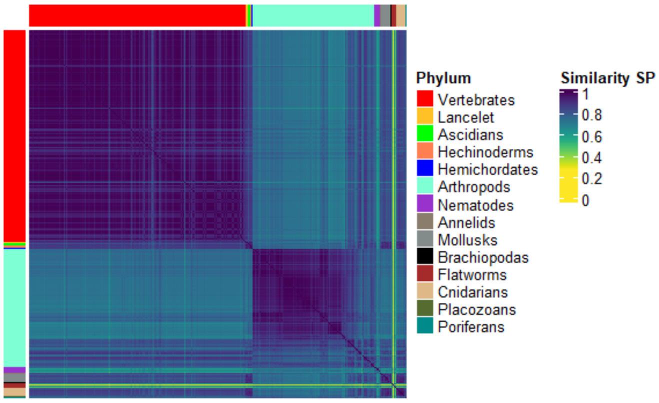

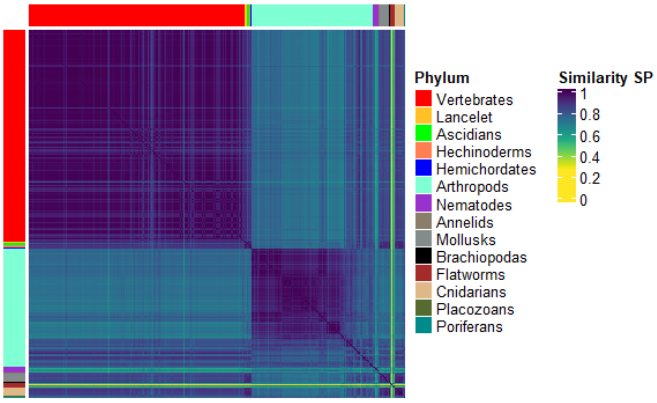

- Threshold 1: for each organism, pathways containing only one reaction are not included in the corresponding abstract metabolic network.
- Threshold 2: for each organism, pathways with only one or two reactions are not included in the corresponding abstract metabolic network.

Optimal number of clusters for the original matrix

```
##                  Cluster
## Real group          1   2   3   4
##   Annelids          0   0   0   1
##   Arthropods        0  95   0  24
##   Ascidians         0   0   0   2
##   Brachiopodas      0   0   0   1
##   Cnidarians        0   0   0   9
##   Flatworms         0   0   4   0
##   Hechinoderms      0   0   0   2
##   Hemichordates     0   0   0   1
##   Lancelet          0   0   0   2
##   Mollusks          0   0   0   9
##   Nematodes         0   0   4   2
##   Placozoans        0   0   0   1
##   Poriferans        0   0   0   1
##   Vertebrates     195   0   0  17
```

Optimal number of clusters for threshold 1 matrix

```
##                  Cluster
## Real group          1   2   3   4
##   Annelids          0   0   0   1
##   Arthropods        0  95   0  24
##   Ascidians         0   0   0   2
##   Brachiopodas      0   0   0   1
##   Cnidarians        0   0   0   9
##   Flatworms         4   0   0   0
```

```
##    Hechinoderms    0   0   0   2
##    Hemichordates   0   0   0   1
##    Lancelet        0   0   0   2
##    Mollusks        0   0   0   9
##    Nematodes       4   0   0   2
##    Placozoans      0   0   0   1
##    Poriferans      0   0   0   1
##    Vertebrates     0   0 195  17
```

Optimal number of clusters for threshold 2 matrix

```
##                 Cluster
## Real group        1   2   3   4
##    Annelids       0   0   0   1
##    Arthropods     0   0  95  24
##    Ascidians      0   0   0   2
##    Brachiopodas   0   0   0   1
##    Cnidarians     0   0   0   9
##    Flatworms      0   4   0   0
##    Hechinoderms   0   0   0   2
##    Hemichordates  0   0   0   1
##    Lancelet       0   0   0   2
##    Mollusks       0   0   0   9
##    Nematodes      0   4   0   2
##    Placozoans     0   0   0   1
##    Poriferans     0   0   0   1
##    Vertebrates  193   0   0  19
```

Weisfeiler-Lehman (WL) kernel

Heatmap of the original matrix

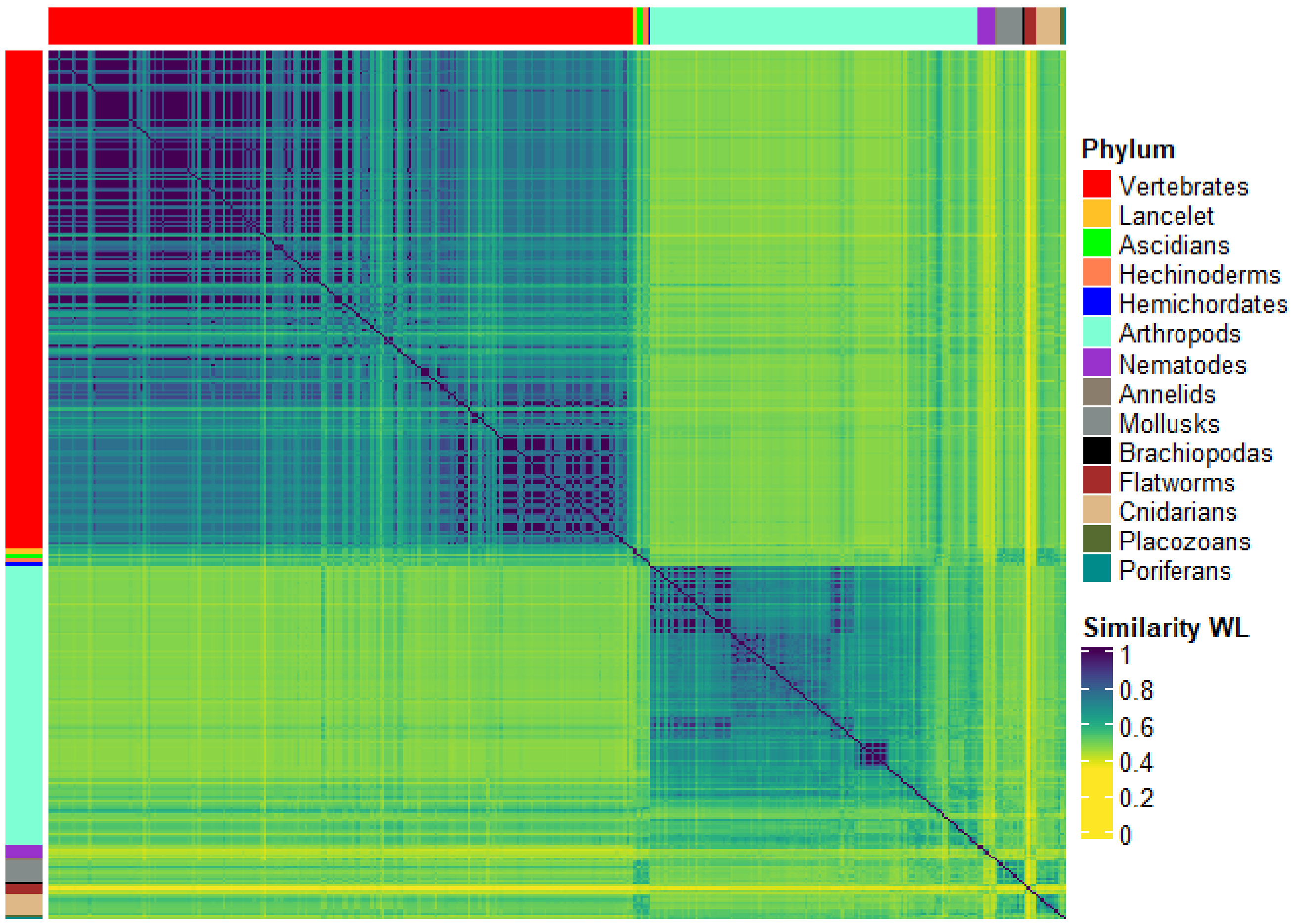

Heatmaps with threshold 1 (left side) and threshold 2 (right side)

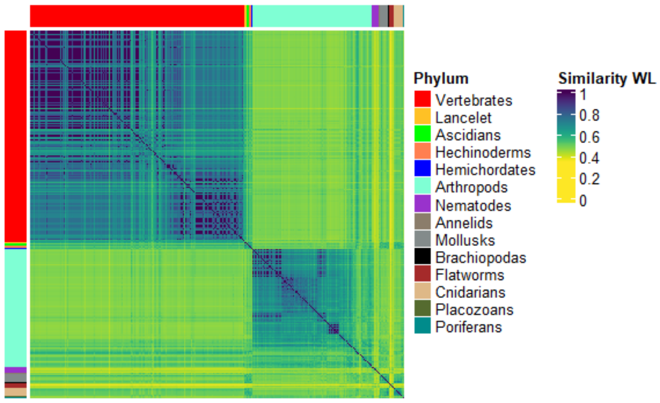

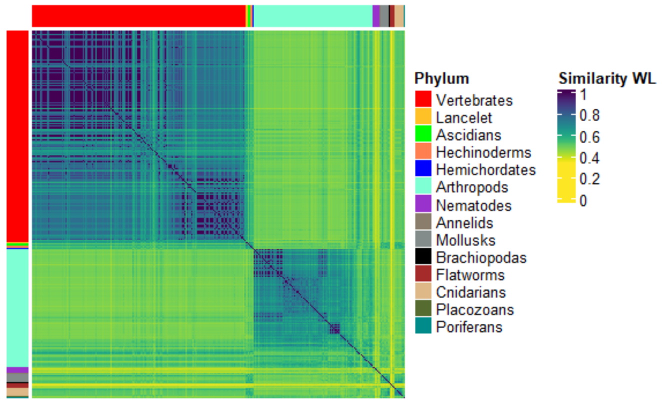

- Threshold 1: for each organism, pathways containing only one reaction are not included in the corresponding abstract metabolic network.
- Threshold 2: for each organism, pathways with only one or two reactions are not included in the corresponding abstract metabolic network.

Optimal number of clusters for the original matrix

```
##                  Cluster
## Real group          1    2    3    4
##    Annelids         0    0    0    1
##    Arthropods       0    0   99   20
```

```
##    Ascidians        0    0    0    2
##    Brachiopodas     0    0    0    1
##    Cnidarians       0    0    0    9
##    Flatworms        0    0    0    4
##    Hechinoderms     0    0    0    2
##    Hemichordates    0    0    0    1
##    Lancelet         0    0    0    2
##    Mollusks         0    0    0    9
##    Nematodes        0    0    0    6
##    Placozoans       0    0    0    1
##    Poriferans       0    0    0    1
##    Vertebrates    141   67    0    4
```

Optimal number of clusters for threshold 1 matrix

```
##                  Cluster
## Real group          1    2    3    4
##    Annelids         0    1    0    0
##    Arthropods      99   20    0    0
##    Ascidians        0    2    0    0
##    Brachiopodas     0    1    0    0
##    Cnidarians       0    9    0    0
##    Flatworms        0    4    0    0
##    Hechinoderms     0    2    0    0
##    Hemichordates    0    1    0    0
##    Lancelet         0    2    0    0
##    Mollusks         0    9    0    0
##    Nematodes        0    6    0    0
##    Placozoans       0    1    0    0
##    Poriferans       0    1    0    0
##    Vertebrates      0    4   67  141
```

Optimal number of clusters for threshold 2 matrix

```
##                  Cluster
## Real group          1    2    3    4
##    Annelids         1    0    0    0
##    Arthropods      20   99    0    0
##    Ascidians        2    0    0    0
##    Brachiopodas     1    0    0    0
##    Cnidarians       9    0    0    0
##    Flatworms        4    0    0    0
##    Hechinoderms     2    0    0    0
##    Hemichordates    1    0    0    0
```

```
##    Lancelet        2   0   0   0
##    Mollusks        9   0   0   0
##    Nematodes       6   0   0   0
##    Placozoans      1   0   0   0
##    Poriferans      1   0   0   0
##    Vertebrates     4   0 140  68
```

Pyramid match (PM) kernel

Heatmap of the original matrix

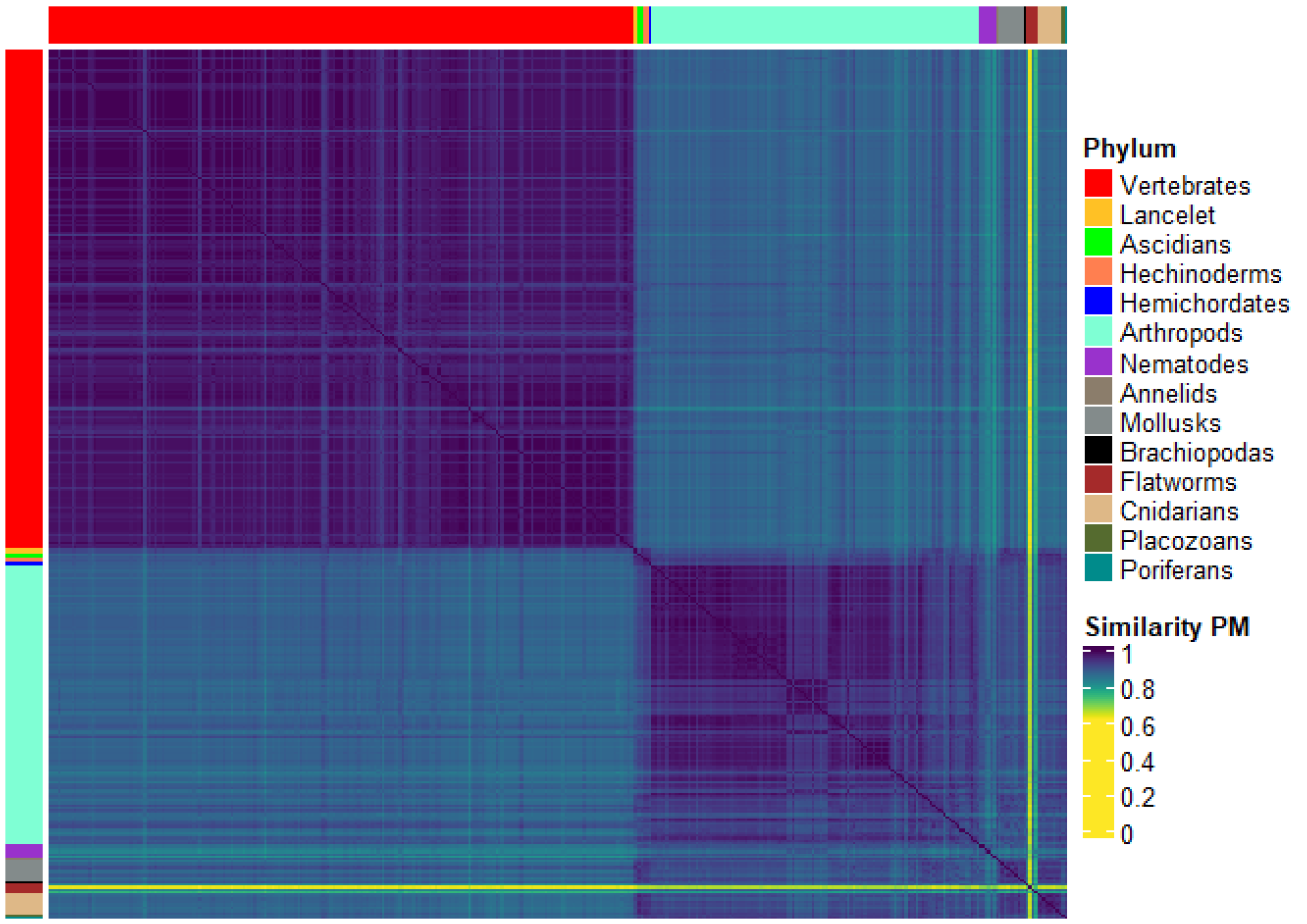

Heatmaps with threshold 1 (left side) and threshold 2 (right side)

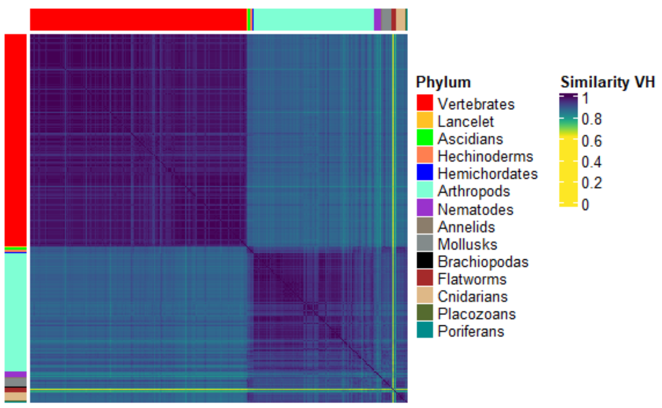

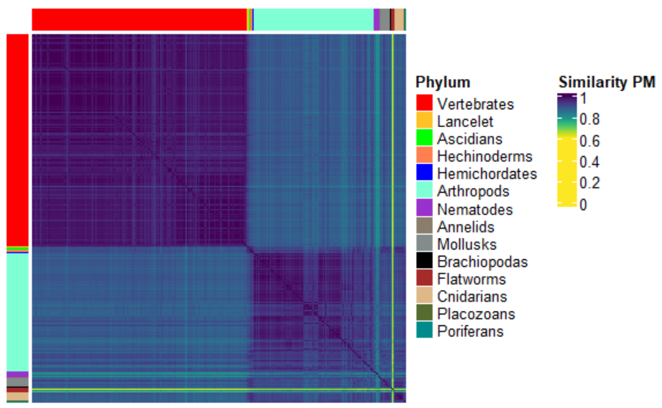

- Threshold 1: for each organism, pathways containing only one reaction are not included in the corresponding abstract metabolic network.
- Threshold 2: for each organism, pathways with only one or two reactions are not included in the corresponding abstract metabolic network.

Optimal number of clusters for the original matrix

```
##                  Cluster
## Real group         1   2   3   4
##    Annelids        1   0   0   0
##    Arthropods     22   0   0  97
##    Ascidians       2   0   0   0
##    Brachiopodas    1   0   0   0
##    Cnidarians      9   0   0   0
##    Flatworms       2   2   0   0
##    Hechinoderms    2   0   0   0
##    Hemichordates   1   0   0   0
##    Lancelet        2   0   0   0
##    Mollusks        9   0   0   0
##    Nematodes       6   0   0   0
##    Placozoans      1   0   0   0
##    Poriferans      1   0   0   0
##    Vertebrates     0   0 212   0
```

Optimal number of clusters for threshold 1 matrix

```
##                  Cluster
## Real group         1   2   3   4
##    Annelids        0   0   0   1
##    Arthropods      0   0  97  22
##    Ascidians       0   0   0   2
##    Brachiopodas    0   0   0   1
##    Cnidarians      0   0   0   9
##    Flatworms       0   2   0   2
##    Hechinoderms    0   0   0   2
##    Hemichordates   0   0   0   1
##    Lancelet        0   0   0   2
##    Mollusks        0   0   0   9
##    Nematodes       0   0   0   6
##    Placozoans      0   0   0   1
##    Poriferans      0   0   0   1
##    Vertebrates   212   0   0   0
```

Optimal number of clusters for threshold 2 matrix

```
##                 Cluster
## Real group        1   2   3   4
##   Annelids        1   0   0   0
##   Arthropods     40   0   0  79
##   Ascidians       2   0   0   0
##   Brachiopodas    1   0   0   0
##   Cnidarians      9   0   0   0
##   Flatworms       1   3   0   0
##   Hechinoderms    2   0   0   0
##   Hemichordates   1   0   0   0
##   Lancelet        2   0   0   0
##   Mollusks        9   0   0   0
##   Nematodes       3   3   0   0
##   Placozoans      1   0   0   0
##   Poriferans      1   0   0   0
##   Vertebrates     0   0 212   0
```

Vetebrates Analysis

Different thresholds

- Vertex hystogram (VH) kernel
    - Heatmap of the original matrix
    - Heatmaps with threshold 1 (left side) and threshold 2 (right side)
    - Optimal number of clusters for the original matrix
    - Optimal number of clusters for threshold 1 matrix
    - Optimal number of clusters for threshold 2 matrix
- Shortest path (SP) kernel
    - Heatmap of the original matrix
    - Heatmaps with threshold 1 (left side) and threshold 2 (right side)
    - Optimal number of clusters for the original matrix
    - Optimal number of clusters for threshold 1 matrix
    - Optimal number of clusters for threshold 2 matrix
- Weisfeiler-Lehman (WL) kernel
    - Heatmap of the original matrix
    - Heatmaps with threshold 1 (left side) and threshold 2 (right side)
    - Optimal number of clusters for the original matrix
    - Optimal number of clusters for threshold 1 matrix
    - Optimal number of clusters for threshold 2 matrix
- Pyramid match (PM) kernel
    - Heatmap of the original matrix
    - Heatmaps with threshold 1 (left side) and threshold 2 (right side)
    - Optimal number of clusters for the original matrix
    - Optimal number of clusters for threshold 1 matrix
    - Optimal number of clusters for threshold 2 matrix

Vertex hystogram (VH) kernel

Heatmap of the original matrix

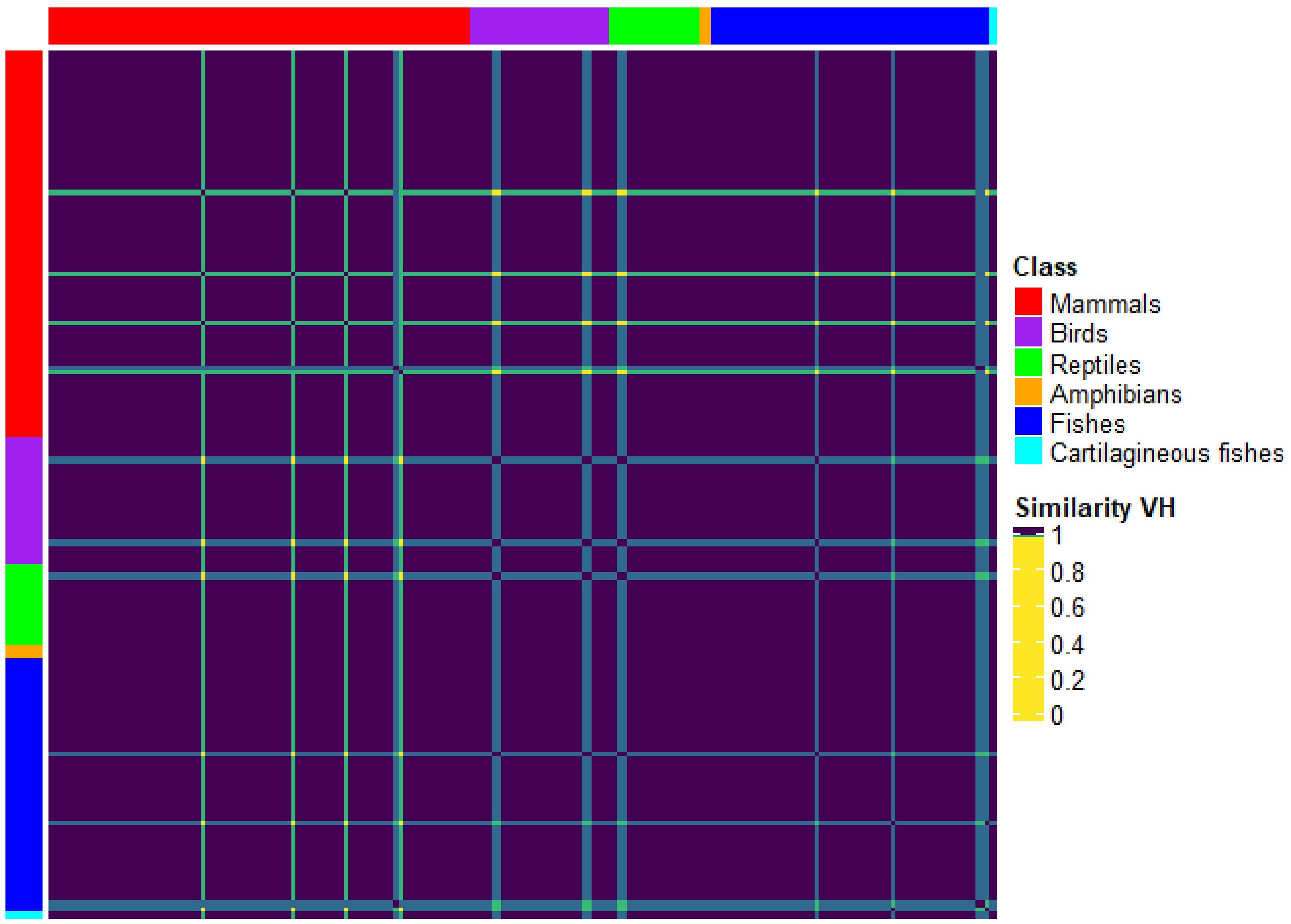

Heatmaps with threshold 1 (left side) and threshold 2 (right side)

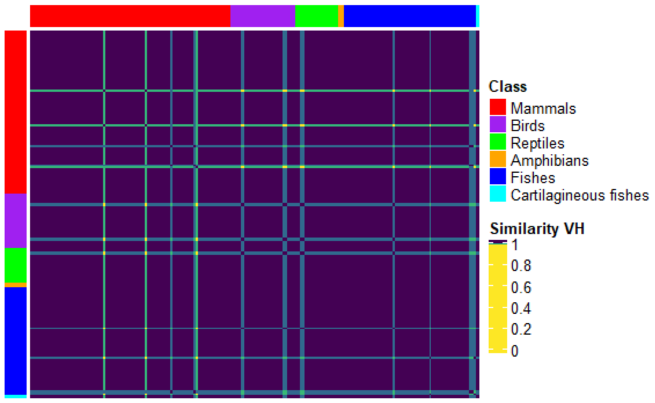

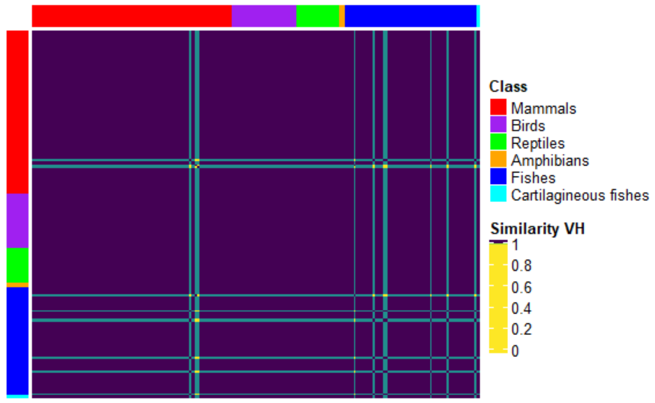

- Threshold 1: for each organism, pathways containing only one reaction are not included in the corresponding abstract metabolic network.
- Threshold 2: for each organism, pathways with only one or two reactions are not included in the corresponding abstract metabolic network.

Optimal number of clusters for the original matrix

```
##                          Cluster
## Real group                 1  2  3  4  5  6
##   Amphibians               0  3  0  0  0  0
##   Birds                    0 27  0  0  4  0
```

```
##    Cartilagineous fishes  0  2  0  0  0  0
##    Fishes                 0 57  2  0  1  2
##    Mammals                3 89  1  1  0  0
##    Reptiles               0 18  0  0  2  0
```

Optimal number of clusters for threshold 1 matrix

```
##                          Cluster
## Real group                 1  2  3  4  5  6
##    Amphibians              0  0  0  0  3  0
##    Birds                   0  0  0  4 27  0
##    Cartilagineous fishes   0  0  0  0  2  0
##    Fishes                  2  0  2  1 57  0
##    Mammals                 0  1  2  0 89  2
##    Reptiles                0  0  0  2 18  0
```

Optimal number of clusters for threshold 2 matrix

```
##                          Cluster
## Real group                 1  2  3  4
##    Amphibians              0  3  0  0
##    Birds                   0 31  0  0
##    Cartilagineous fishes   0  2  0  0
##    Fishes                  6 55  1  0
##    Mammals                 1 91  1  1
##    Reptiles                0 20  0  0
```

Shortest path (SP) kernel

Heatmap of the original matrix

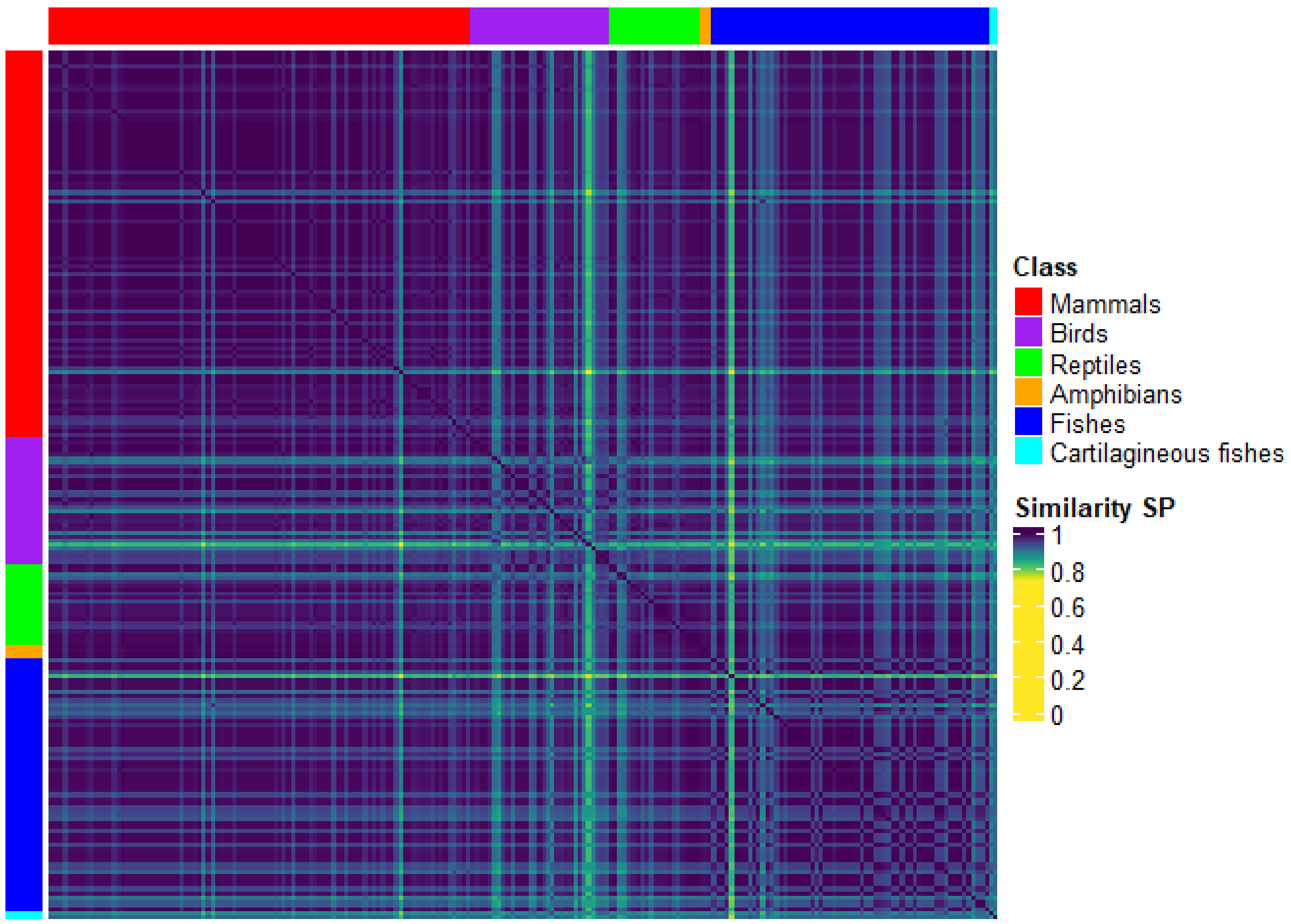

Heatmaps with threshold 1 (left side) and threshold 2 (right side)

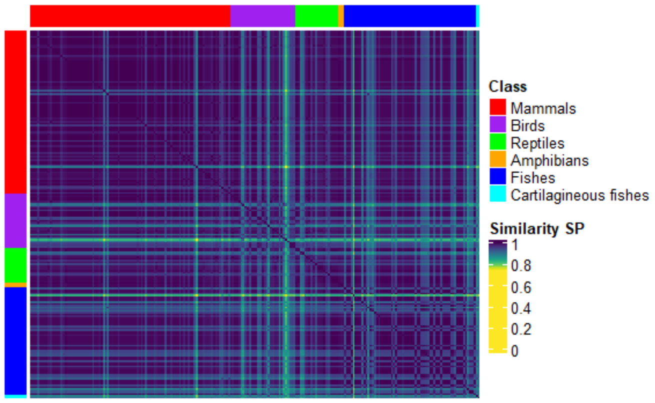

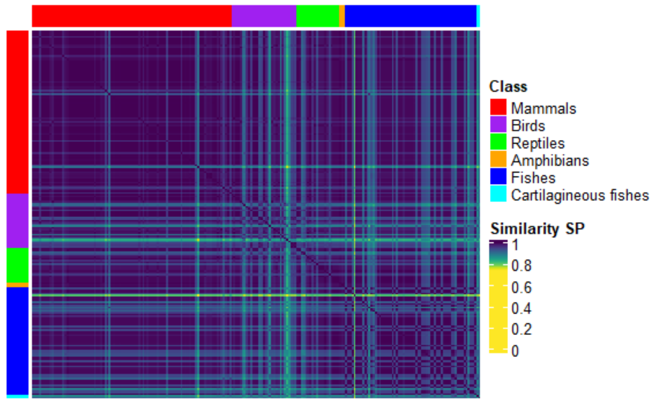

- Threshold 1: for each organism, pathways containing only one reaction are not included in the corresponding abstract metabolic network.
- Threshold 2: for each organism, pathways with only one or two reactions are not included in the corresponding abstract metabolic network.

Optimal number of clusters for the original matrix

```
##                         Cluster
## Real group                1  2  3  4  5  6
##   Amphibians              1  2  0  0  0  0
##   Birds                   9  7  7  8  0  0
```

```
##   Cartilagineous fishes  0  0  2  0  0  0
##   Fishes                 3  1  3  1 33 21
##   Mammals               21 60  2  9  2  0
##   Reptiles               8  2  2  3  5  0
```

Optimal number of clusters for threshold 1 matrix

```
##                         Cluster
## Real group               1  2  3  4  5  6
##   Amphibians             1  0  0  0  2  0
##   Birds                  9  7  0  8  7  0
##   Cartilagineous fishes  0  2  0  0  0  0
##   Fishes                 3  3 33  1  1 21
##   Mammals               22  2  2  8 60  0
##   Reptiles               8  2  5  3  2  0
```

Optimal number of clusters for threshold 2 matrix

```
##                         Cluster
## Real group               1  2  3  4  5  6
##   Amphibians             0  2  1  0  0  0
##   Birds                  0  7  9 11  4  0
##   Cartilagineous fishes  0  0  0  0  2  0
##   Fishes                34  1  2  1  3 21
##   Mammals                2 66 19  6  1  0
##   Reptiles               5  2  8  5  0  0
```

Weisfeiler-Lehman (WL) kernel

Heatmap of the original matrix

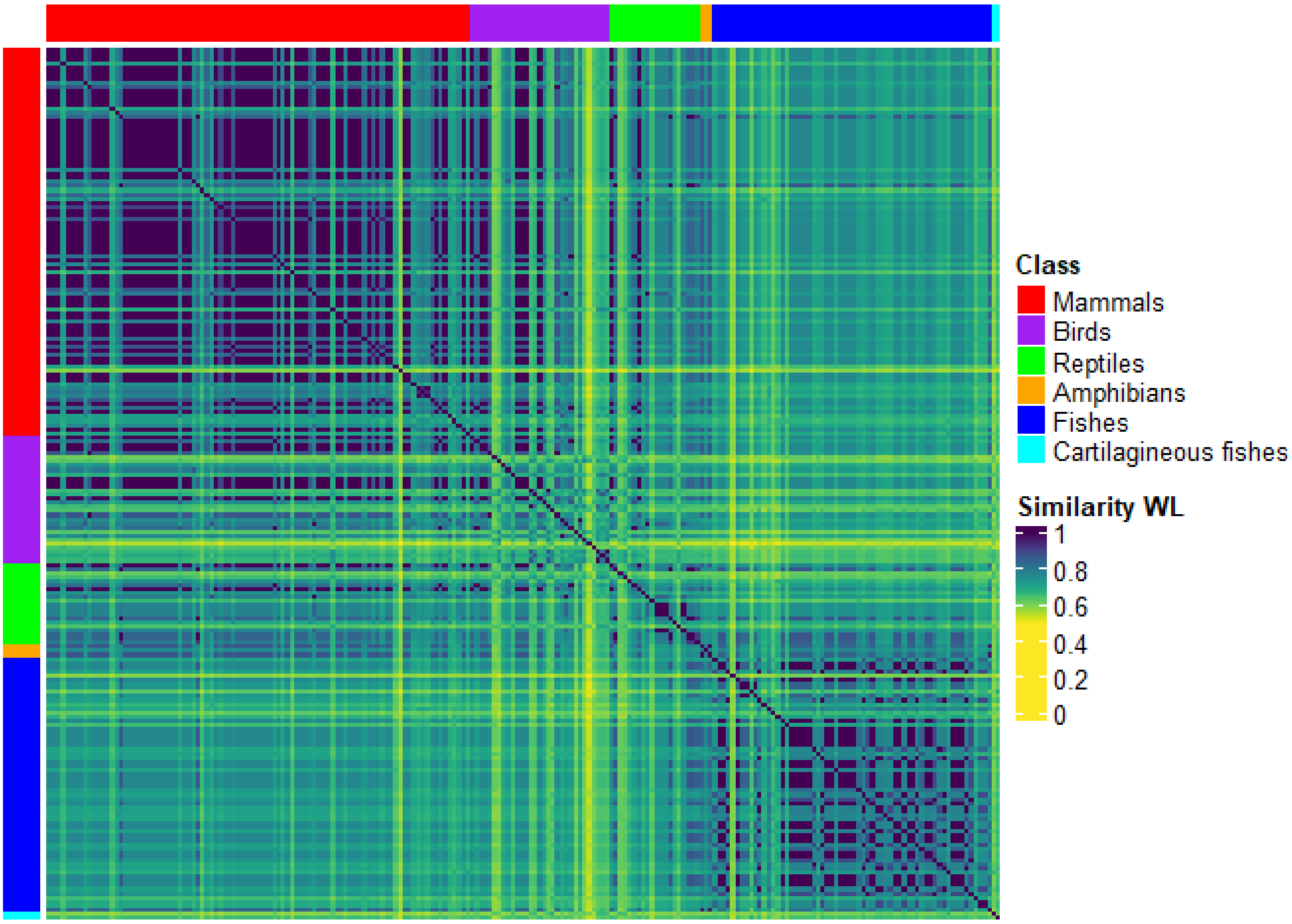

Heatmaps with threshold 1 (left side) and threshold 2 (right side)

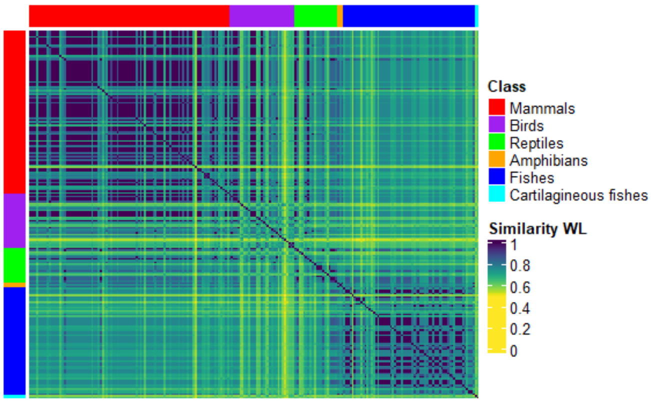

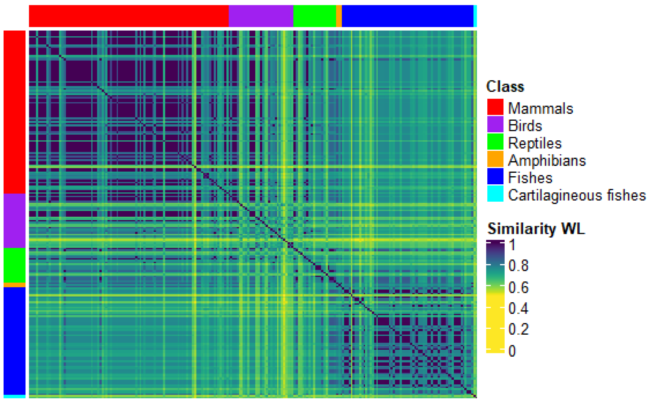

- Threshold 1: for each organism, pathways containing only one reaction are not included in the corresponding abstract metabolic network.
- Threshold 2: for each organism, pathways with only one or two reactions are not included in the corresponding abstract metabolic network.

Optimal number of clusters for the original matrix

```
##                         Cluster
## Real group                1  2  3  4  5  6
##   Amphibians              0  3  0  0  0  0
##   Birds                   0 10  0  7 13  1
```

```
##    Cartilagineous fishes  0  0  0  0  1  1
##    Fishes                18  7 28  0  0  9
##    Mammals                0 28  0 58  1  7
##    Reptiles               0 12  0  2  3  3
```

Optimal number of clusters for threshold 1 matrix

```
##                          Cluster
## Real group                1  2  3  4  5  6
##    Amphibians             3  0  0  0  0  0
##    Birds                 10 13  0  0  1  7
##    Cartilagineous fishes  0  1  0  0  1  0
##    Fishes                 7  0 28 18  9  0
##    Mammals               28  1  0  0  7 58
##    Reptiles              12  3  0  0  3  2
```

Optimal number of clusters for threshold 2 matrix

```
##                          Cluster
## Real group                1  2  3  4  5  6
##    Amphibians             0  3  0  0  0  0
##    Birds                  7 10  0  1  0 13
##    Cartilagineous fishes  0  0  0  1  0  1
##    Fishes                 0  7 18  9 28  0
##    Mammals               59 28  0  6  0  1
##    Reptiles               2 13  0  2  0  3
```

Pyramid match (PM) kernel

Heatmap of the original matrix

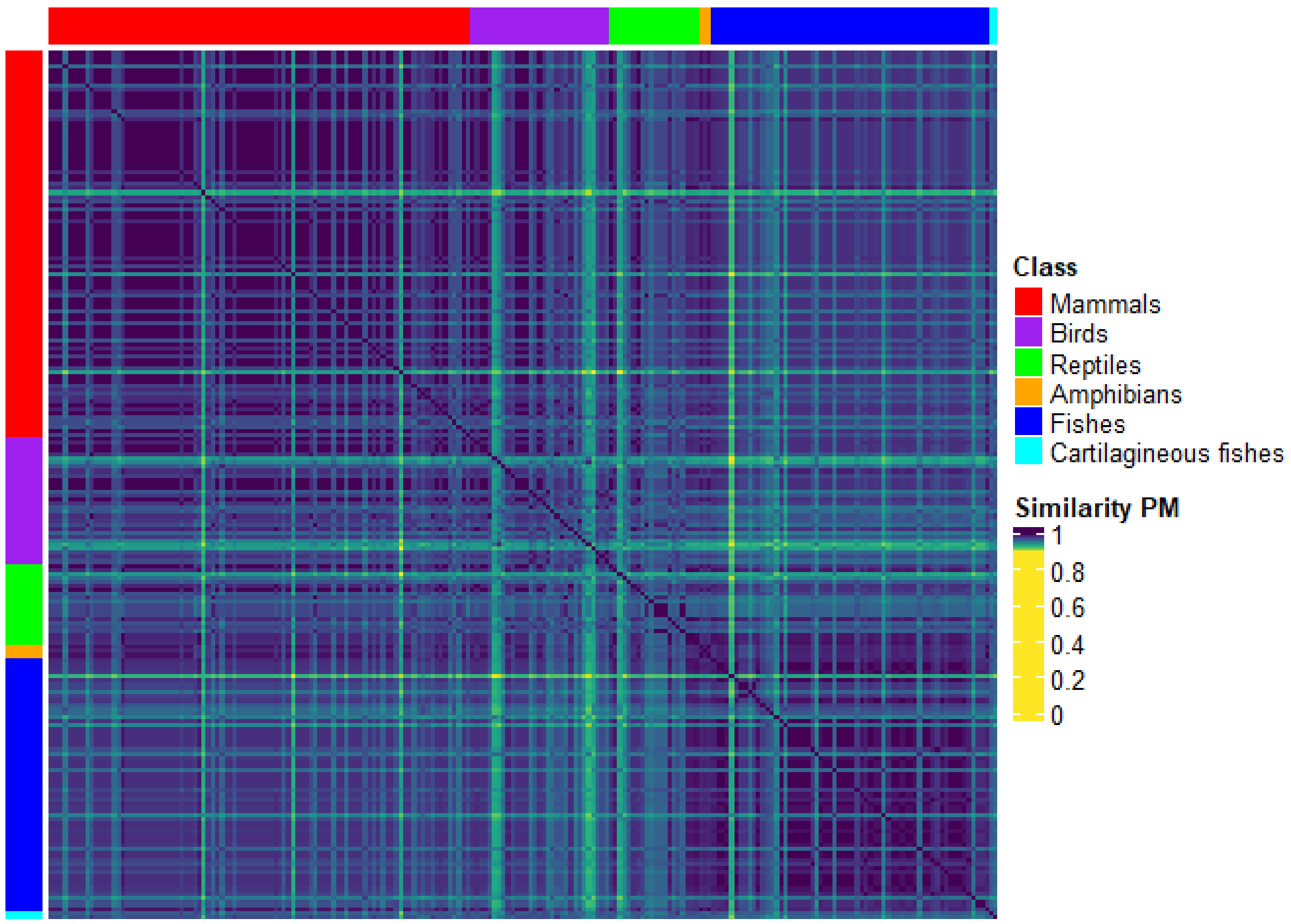

Heatmaps with threshold 1 (left side) and threshold 2 (right side)

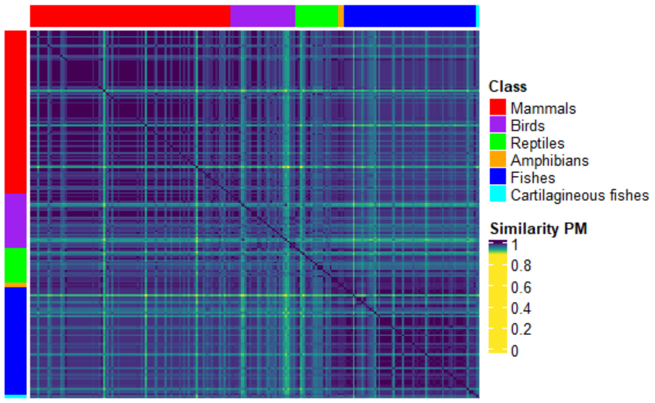

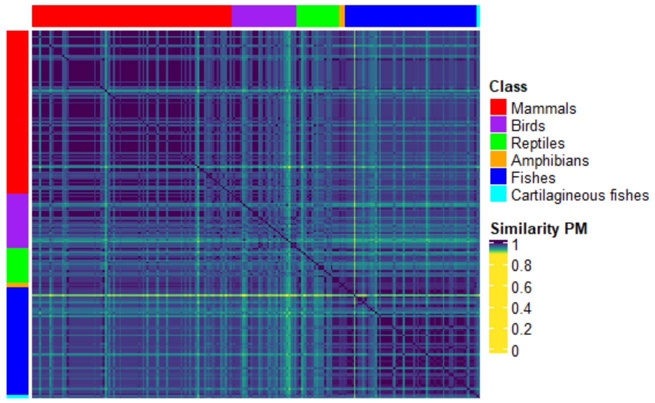

- Threshold 1: for each organism, pathways containing only one reaction are not included in the corresponding abstract metabolic network.
- Threshold 2: for each organism, pathways with only one or two reactions are not included in the corresponding abstract metabolic network.

Optimal number of clusters for the original matrix

```
##                         Cluster
## Real group                1  2  3  4  5  6
##   Amphibians              0  1  2  0  0  0
##   Birds                  10  1  7  0  8  5
```

```
##   Cartilagineous fishes  1  0  0  1  0  0
##   Fishes                 1 39  1 15  0  6
##   Mammals               12  2 58  4 15  3
##   Reptiles               8  5  2  1  2  2
```

Optimal number of clusters for threshold 1 matrix

```
##                          Cluster
## Real group                1  2  3  4  5  6
##   Amphibians              0  1  2  0  0  0
##   Birds                   0  1  7  8  5 10
##   Cartilagineous fishes   1  0  0  0  0  1
##   Fishes                 15 39  1  0  6  1
##   Mammals                 4  2 58 15  3 12
##   Reptiles                1  5  2  2  2  8
```

Optimal number of clusters for threshold 2 matrix

```
##                          Cluster
## Real group                1  2  3  4  5  6
##   Amphibians              1  0  0  0  2  0
##   Birds                   5  5  0  2  7 12
##   Cartilagineous fishes   1  0  0  0  0  1
##   Fishes                 12  3 38  8  1  0
##   Mammals                12  9  2  3 59  9
##   Reptiles                3  8  5  1  2  1
```

Protophotophytes Analysis

Different thresholds

- Vertex hystogram (VH) kernel
  - Heatmap of the original matrix
  - Heatmaps with threshold 1 (left side) and threshold 2 (right side)
  - Optimal number of clustering for the original matrix
  - Optimal number of clustering for threshold 1 matrix
  - Optimal number of clustering for threshold 2 matrix
- Shortest Path (SP) kernel
  - Heatmap of the original matrix
  - Heatmaps with threshold 1 (left side) and threshold 2 (right side)
  - Optimal number of clustering for the original matrix
  - Optimal number of clustering for threshold 1 matrix
  - Optimal number of clustering for threshold 2 matrix
- Weisfeiler-Lehman (WL) kernel
  - Heatmap of the original matrix
  - Heatmaps with threshold 1 (left side) and threshold 2 (right side)
  - Optimal number of clustering for the original matrix
  - Optimal number of clustering for threshold 1 matrix
  - Optimal number of clustering for threshold 2 matrix
- Pyramid match (PM) kernel
  - Heatmap of the original matrix
  - Heatmaps with threshold 1 (left side) and threshold 2 (right side)
  - Optimal number of clustering for the original matrix
  - Optimal number of clustering for threshold 1 matrix
  - Optimal number of clustering for threshold 2 matrix

Vertex hystogram (VH) kernel

Heatmap of the original matrix

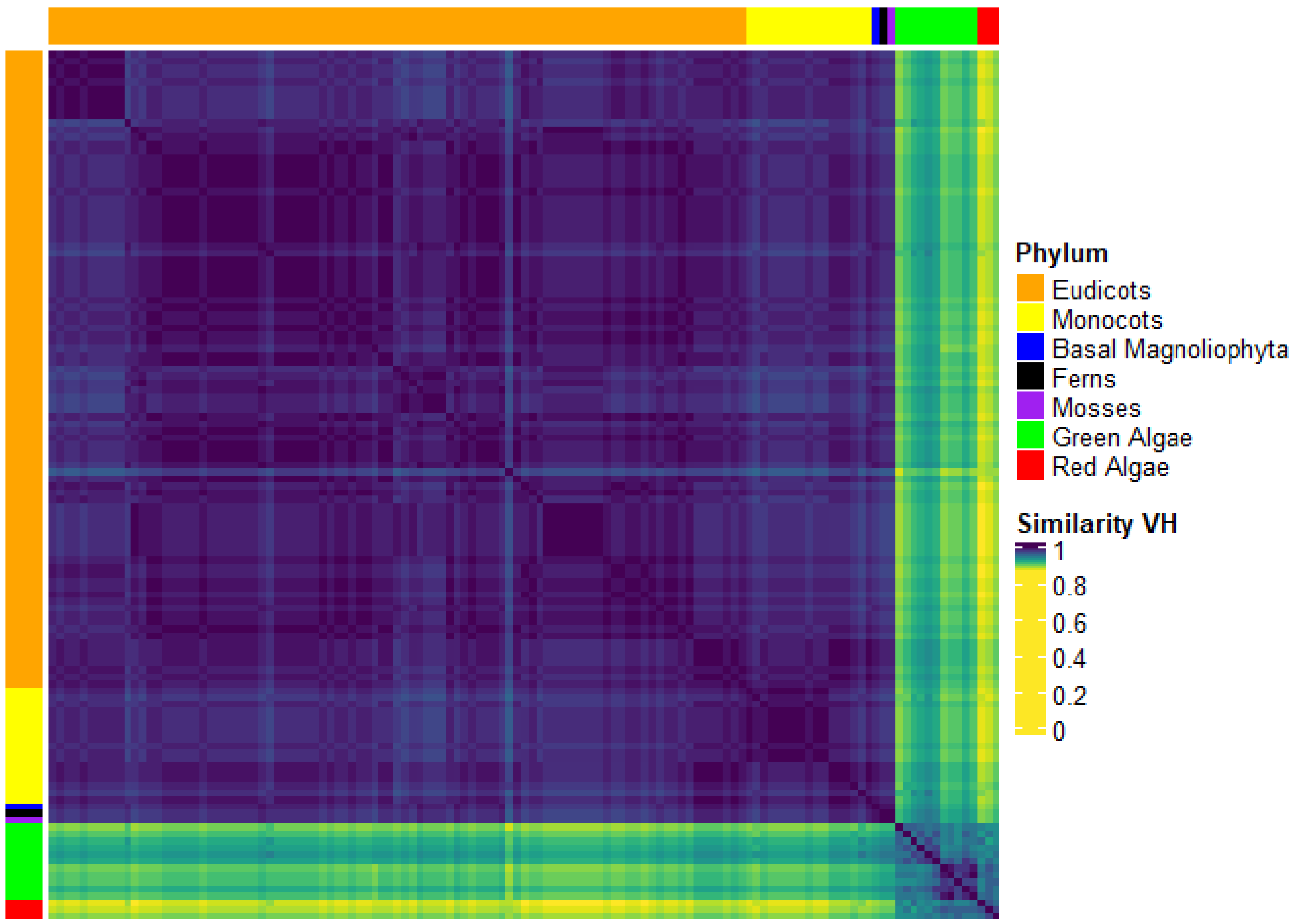

Heatmaps with threshold 1 (left side) and threshold 2 (right side)

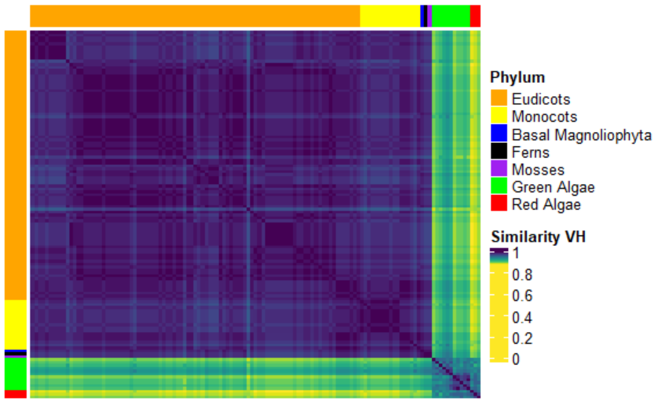

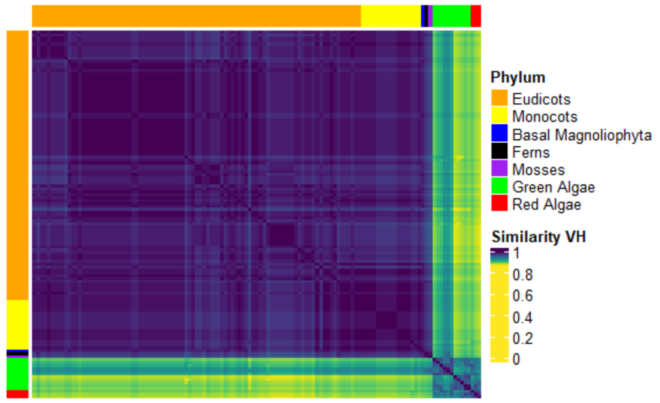

- Threshold 1: for each organism, pathways containing only one reaction are not included in the corresponding abstract metabolic network.
- Threshold 2: for each organism, pathways with only one or two reactions are not included in the corresponding abstract metabolic network.

Optimal number of clustering for the original matrix

```
##                         Cluster
## Real group                1  2  3
##   Basal Magnoliophyta     0  1  0
##   Eudicots                0 38 55
```

```
##   Ferns                 0  1  0
##   Green Algae          11  0  0
##   Monocots              0 13  4
##   Mosses                0  1  0
##   Red Algae             3  0  0
```

Optimal number of clustering for threshold 1 matrix

```
##                        Cluster
## Real group              1  2  3
##   Basal Magnoliophyta  0  0  1
##   Eudicots              0 55 38
##   Ferns                 0  0  1
##   Green Algae          11  0  0
##   Monocots              0  4 13
##   Mosses                0  0  1
##   Red Algae             3  0  0
```

Optimal number of clustering for threshold 2 matrix

```
##                        Cluster
## Real group              1  2  3
##   Basal Magnoliophyta  0  1  0
##   Eudicots              0 57 36
##   Ferns                 0  1  0
##   Green Algae          11  0  0
##   Monocots              0 17  0
##   Mosses                0  1  0
##   Red Algae             3  0  0
```

Shortest Path (SP) kernel

Heatmap of the original matrix

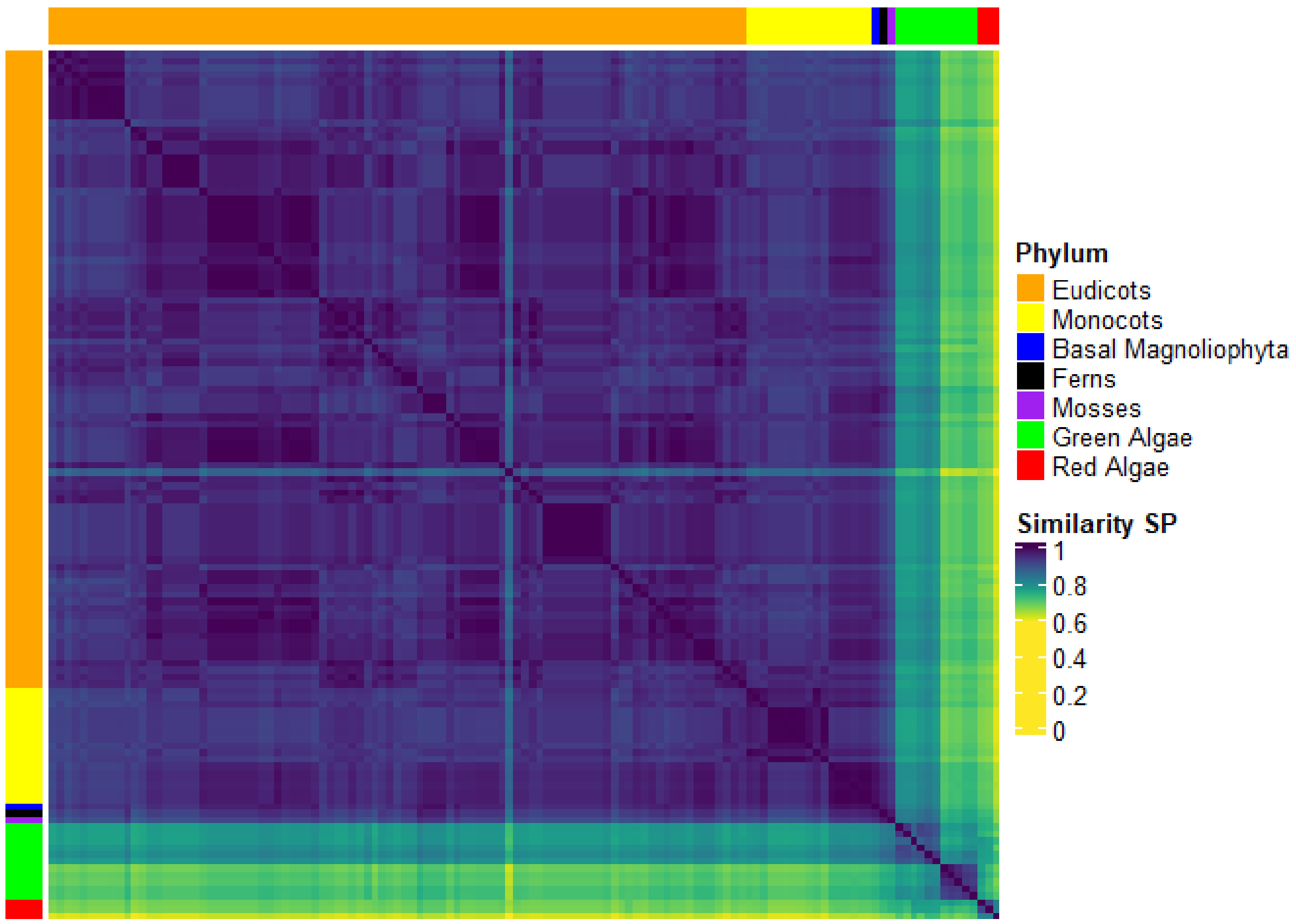

Heatmaps with threshold 1 (left side) and threshold 2 (right side)

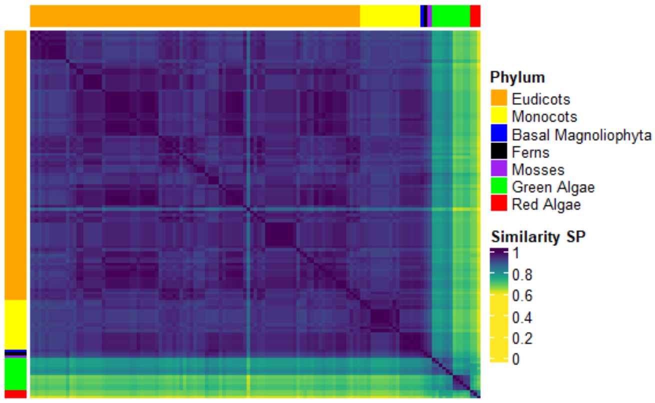

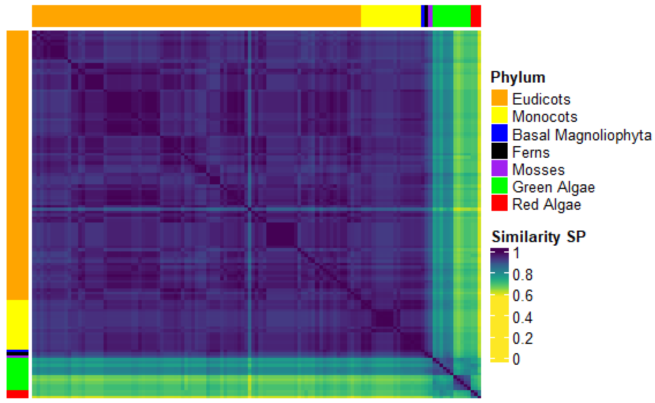

- Threshold 1: for each organism, pathways containing only one reaction are not included in the corresponding abstract metabolic network.
- Threshold 2: for each organism, pathways with only one or two reactions are not included in the corresponding abstract metabolic network.

Optimal number of clustering for the original matrix

```
##                        Cluster
## Real group               1  2  3
##   Basal Magnoliophyta    1  0  0
##   Eudicots              53 40  0
```

```
##    Ferns                  1  0  0
##    Green Algae            0  0 11
##    Monocots               7 10  0
##    Mosses                 0  1  0
##    Red Algae              0  0  3
```

Optimal number of clustering for threshold 1 matrix

```
##                         Cluster
## Real group               1  2  3
##    Basal Magnoliophyta  1  0  0
##    Eudicots             53 40  0
##    Ferns                 1  0  0
##    Green Algae           0  0 11
##    Monocots              7 10  0
##    Mosses                0  1  0
##    Red Algae             0  0  3
```

Optimal number of clustering for threshold 2 matrix

```
##                         Cluster
## Real group               1  2  3
##    Basal Magnoliophyta  1  0  0
##    Eudicots             53  0 40
##    Ferns                 0  0  1
##    Green Algae           0 11  0
##    Monocots             10  0  7
##    Mosses                0  0  1
##    Red Algae             0  3  0
```

Weisfeiler-Lehman (WL) kernel

Heatmap of the original matrix

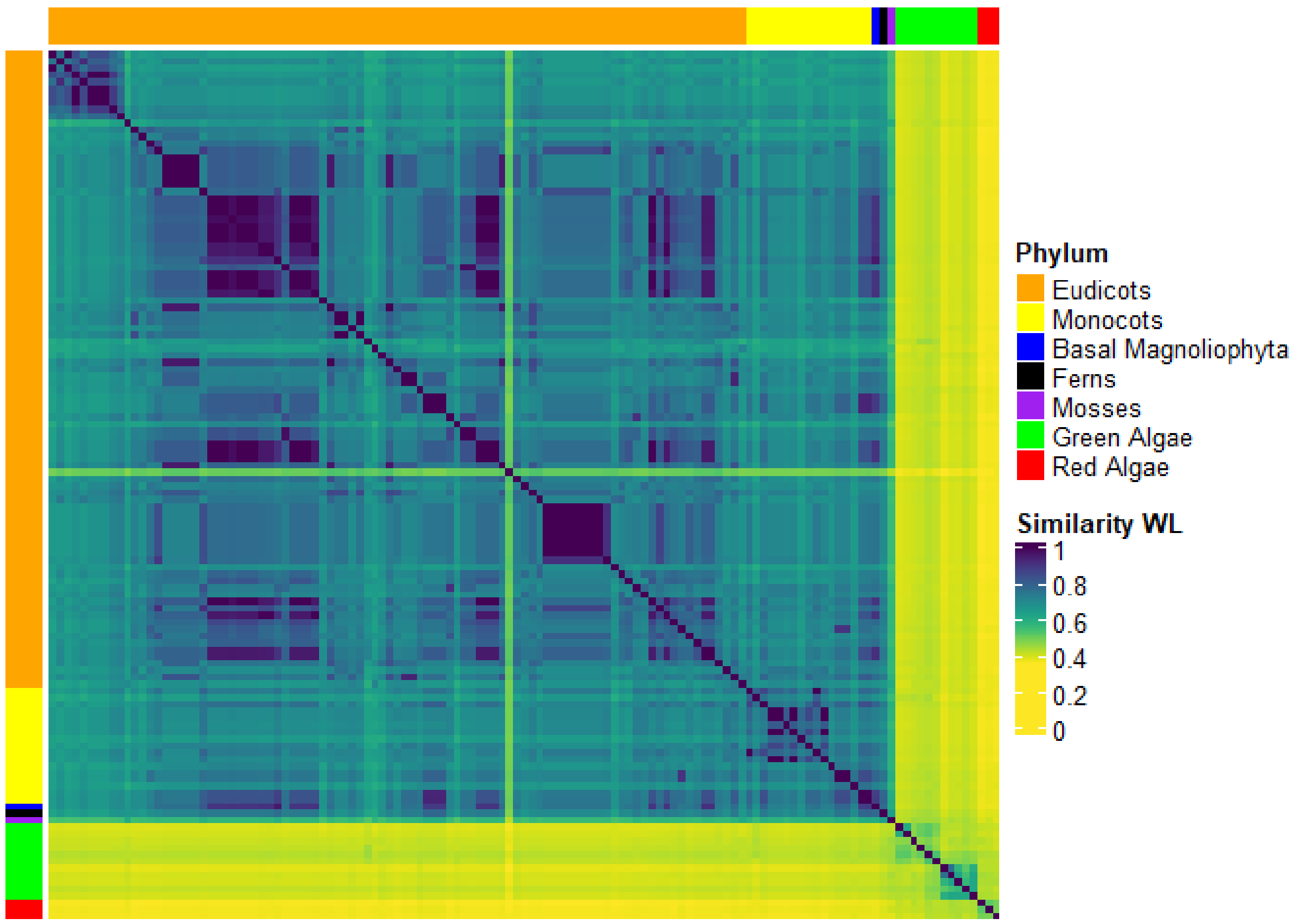

Heatmaps with threshold 1 (left side) and threshold 2 (right side)

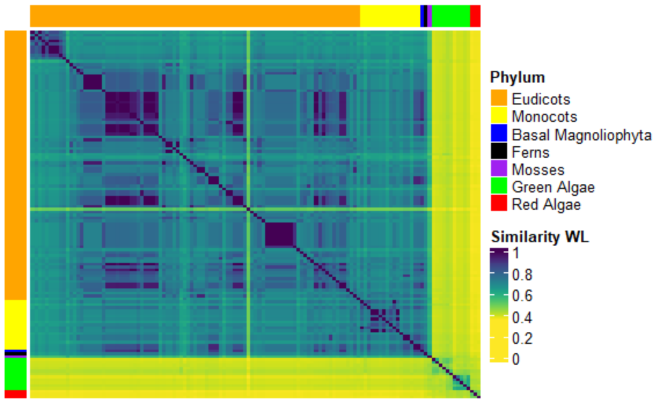

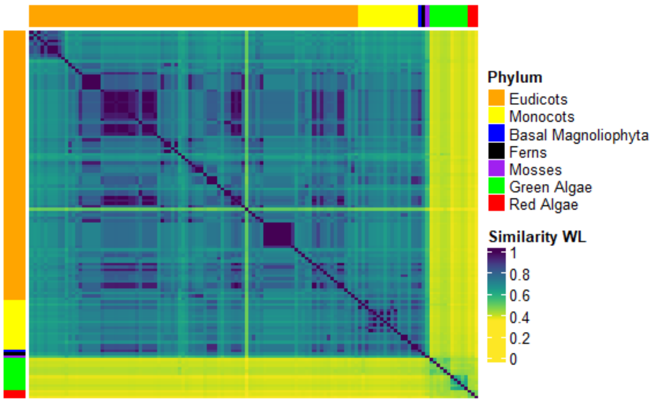

- Threshold 1: for each organism, pathways containing only one reaction are not included in the corresponding abstract metabolic network.
- Threshold 2: for each organism, pathways with only one or two reactions are not included in the corresponding abstract metabolic network.

Optimal number of clustering for the original matrix

```
##                          Cluster
## Real group                 1  2  3
##   Basal Magnoliophyta      0  1  0
##   Eudicots                 1 21 71
```

```
##    Ferns                  0  0  1
##    Green Algae           11  0  0
##    Monocots               0  0 17
##    Mosses                 0  0  1
##    Red Algae              3  0  0
```

Optimal number of clustering for threshold 1 matrix

```
##                         Cluster
## Real group               1  2  3
##    Basal Magnoliophyta  0  1  0
##    Eudicots              1 21 71
##    Ferns                 0  0  1
##    Green Algae          11  0  0
##    Monocots              0  0 17
##    Mosses                0  0  1
##    Red Algae             3  0  0
```

Optimal number of clustering for threshold 2 matrix

```
##                         Cluster
## Real group               1  2  3
##    Basal Magnoliophyta  0  1  0
##    Eudicots              1 23 69
##    Ferns                 0  0  1
##    Green Algae          11  0  0
##    Monocots              0  0 17
##    Mosses                1  0  0
##    Red Algae             3  0  0
```

Pyramid match (PM) kernel

Heatmap of the original matrix

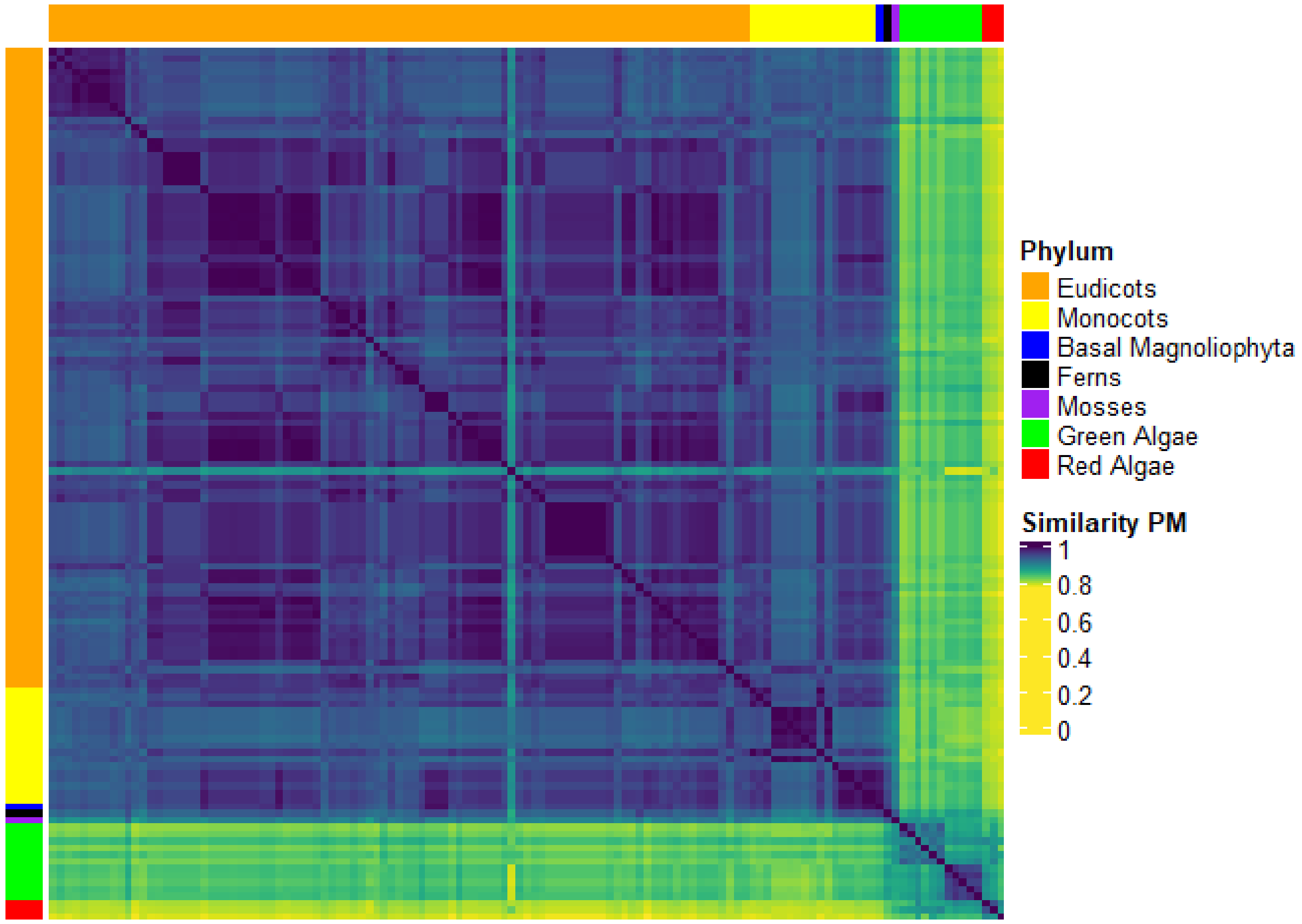

Heatmaps with threshold 1 (left side) and threshold 2 (right side)

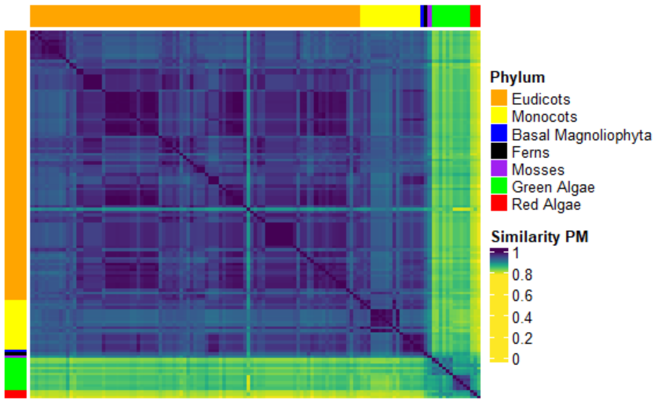

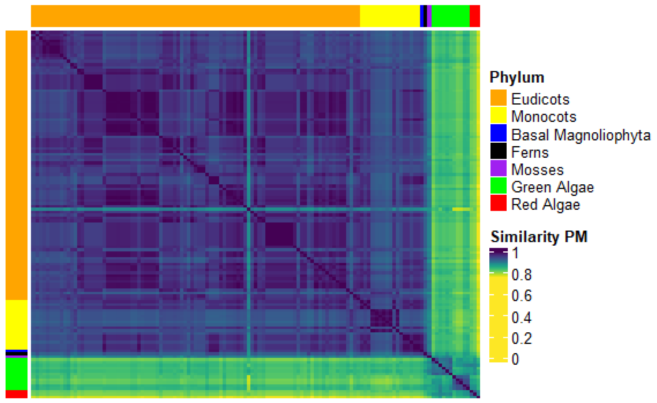

- Threshold 1: for each organism, pathways containing only one reaction are not included in the corresponding abstract metabolic network.
- Threshold 2: for each organism, pathways with only one or two reactions are not included in the corresponding abstract metabolic network.

Optimal number of clustering for the original matrix

```
##                        Cluster
## Real group               1  2  3
##   Basal Magnoliophyta    0  1  0
##   Eudicots               0 66 27
```

```
##    Ferns                  0  0  1
##    Green Algae           11  0  0
##    Monocots               0  8  9
##    Mosses                 0  0  1
##    Red Algae              3  0  0
```

Optimal number of clustering for threshold 1 matrix

```
##                         Cluster
## Real group               1  2  3
##    Basal Magnoliophyta   0  1  0
##    Eudicots              0 66 27
##    Ferns                 0  0  1
##    Green Algae          11  0  0
##    Monocots              0  8  9
##    Mosses                0  0  1
##    Red Algae             3  0  0
```

Optimal number of clustering for threshold 2 matrix

```
##                         Cluster
## Real group               1  2  3
##    Basal Magnoliophyta   0  0  1
##    Eudicots              1 41 51
##    Ferns                 0  1  0
##    Green Algae          11  0  0
##    Monocots              0 10  7
##    Mosses                1  0  0
##    Red Algae             3  0  0
```

Fungi Analysis

Different thresholds

- Vertex hystogram (VH) kernel
  - Heatmap of the original matrix
  - Heatmaps with threshold 1 (left side) and threshold 2 (right side)
  - 3-means clusters for the original matrix
  - 3-means clusters for threshold 1 matrix
  - 3-means clusters for threshold 2 matrix
- Shortest Path (SP) kernel
  - Heatmap of the original matrix
  - Heatmaps with threshold 1 (left side) and threshold 2 (right side)
  - 3-means clusters for the original matrix
  - 3-means clusters for threshold 1 matrix
  - 3-means clusters for threshold 2 matrix
- Weisfeiler-Lehman (WL) kernel
  - Heatmap of the original matrix
  - Heatmaps with threshold 1 (left side) and threshold 2 (right side)
  - 3-means clusters for the original matrix
  - 3-means clusters for threshold 1 matrix
  - 3-means clusters for threshold 2 matrix
- Pyramid match (PM) kernel
  - Heatmap of the original matrix
  - Heatmaps with threshold 1 (left side) and threshold 2 (right side)
  - 3-means clusters for the original matrix
  - 3-means clusters for threshold 1 matrix
  - 3-means clusters for threshold 2 matrix

Vertex hystogram (VH) kernel

Heatmap of the original matrix

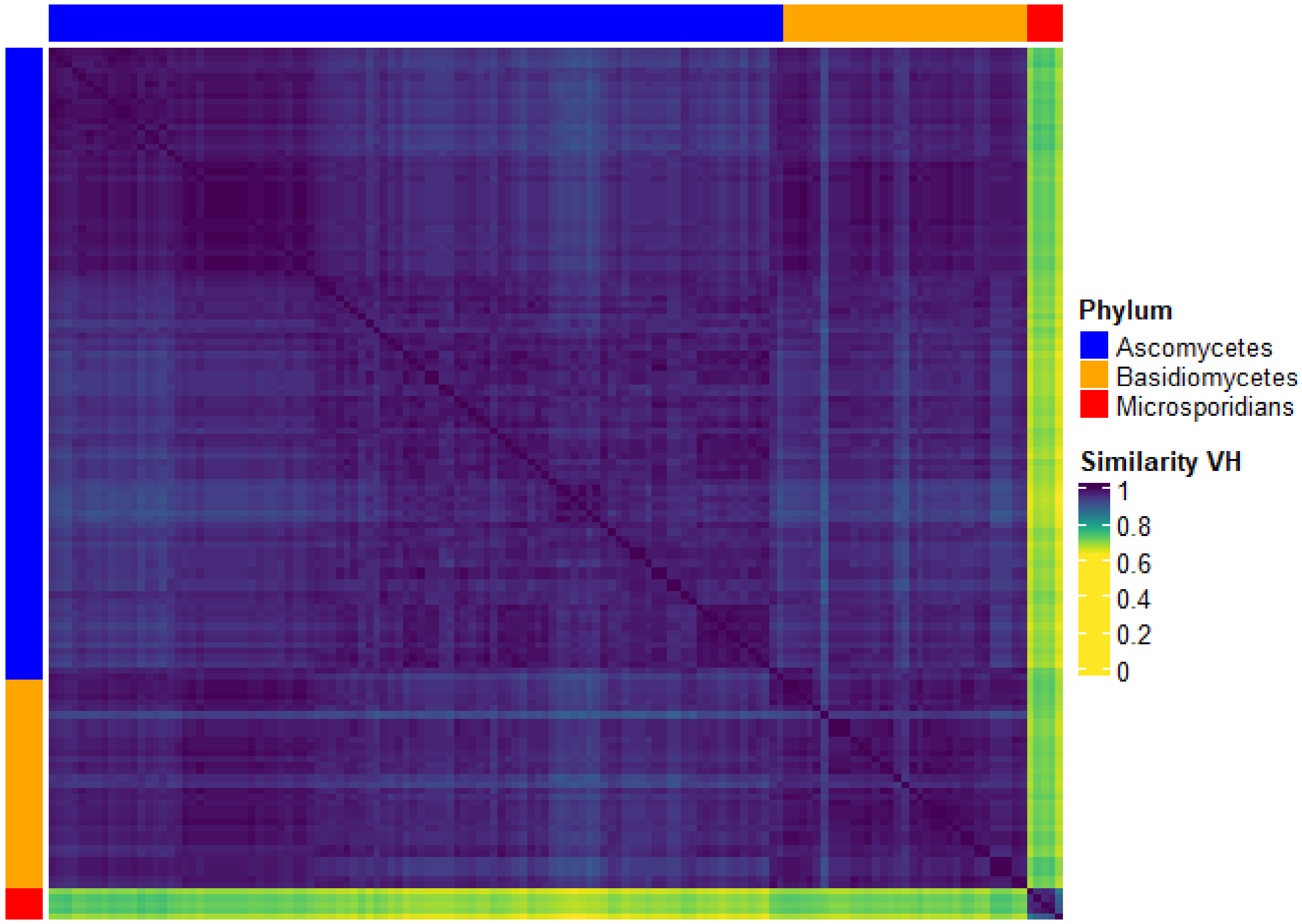

Heatmaps with threshold 1 (left side) and threshold 2 (right side)

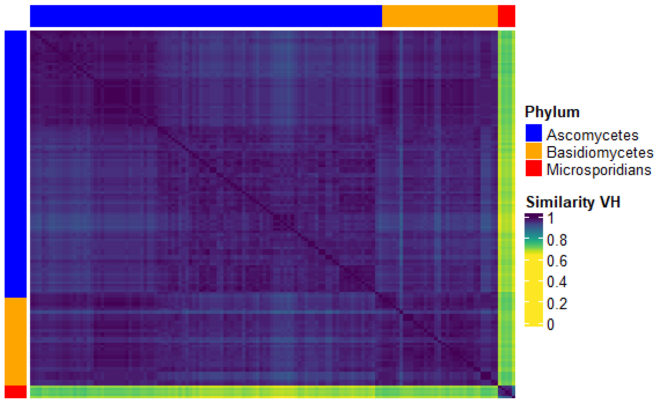

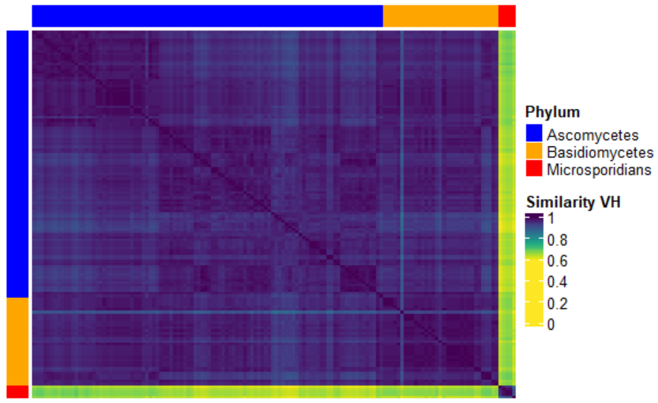

- Threshold 1: for each organism, pathways containing only one reaction are not included in the corresponding abstract metabolic network.
- Threshold 2: for each organism, pathways with only one or two reactions are not included in the corresponding abstract metabolic network.

3-means clusters for the original matrix

```
##                    Cluster
## Real group           1  2  3
##   Ascomycetes       62  0 38
```

```
##   Basidiomycetes   3  0 30
##   Microsporidians  0  5  0
```

3-means clusters for threshold 1 matrix

```
##                     Cluster
## Real group            1  2  3
##   Ascomycetes        62  0 38
##   Basidiomycetes      6  0 27
##   Microsporidians     0  5  0
```

3-means clusters for threshold 2 matrix

```
##                     Cluster
## Real group            1  2  3
##   Ascomycetes        61 39  0
##   Basidiomycetes      3 30  0
##   Microsporidians     0  0  5
```

Shortest Path (SP) kernel

Heatmap of the original matrix

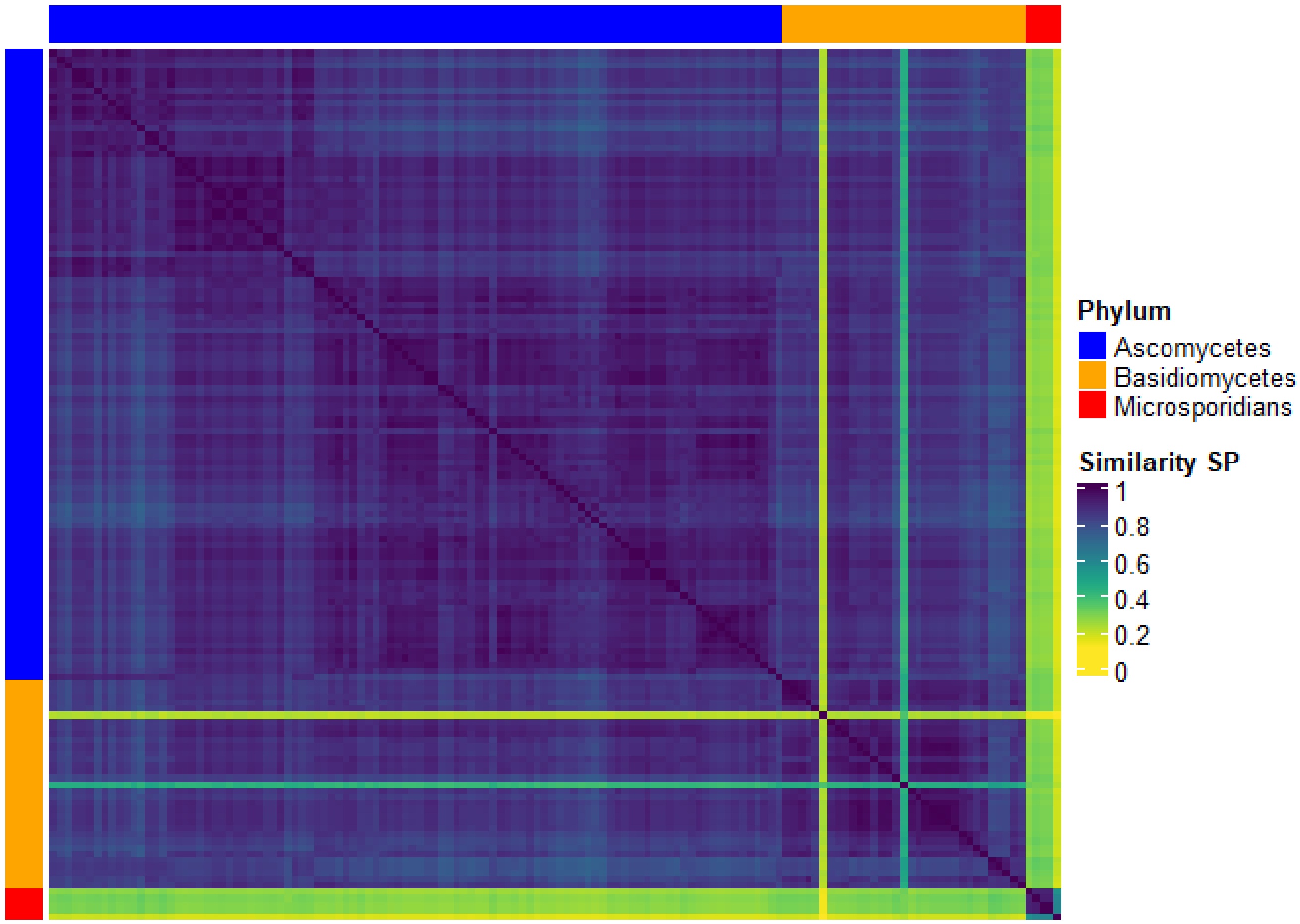

Heatmaps with threshold 1 (left side) and threshold 2 (right side)

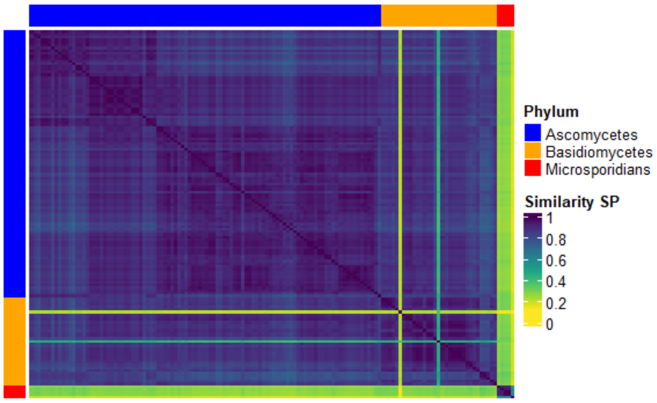

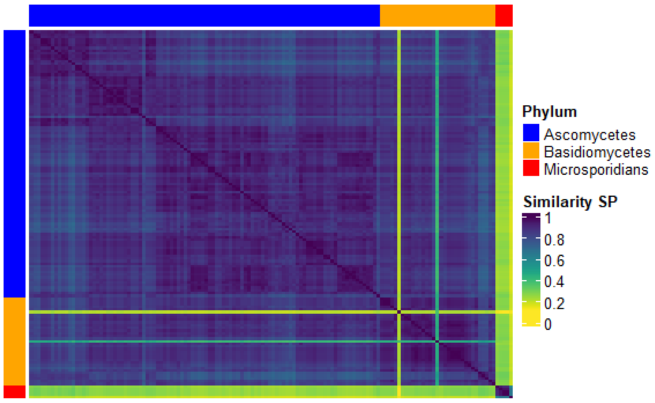

- Threshold 1: for each organism, pathways containing only one reaction are not included in the corresponding abstract metabolic network.
- Threshold 2: for each organism, pathways with only one or two reactions are not included in the corresponding abstract metabolic network.

3-means clusters for the original matrix

```
##                   Cluster
## Real group          1  2  3
##   Ascomycetes       0 70 30
##   Basidiomycetes    2  4 27
##   Microsporidians   5  0  0
```

3-means clusters for threshold 1 matrix

```
##                   Cluster
## Real group          1  2  3
##   Ascomycetes      31  0 69
##   Basidiomycetes   27  2  4
##   Microsporidians   0  5  0
```

3-means clusters for threshold 2 matrix

```
##                   Cluster
## Real group          1  2  3
##   Ascomycetes       0 25 75
##   Basidiomycetes    2  8 23
##   Microsporidians   5  0  0
```

Weisfeiler-Lehman (WL) kernel

Heatmap of the original matrix

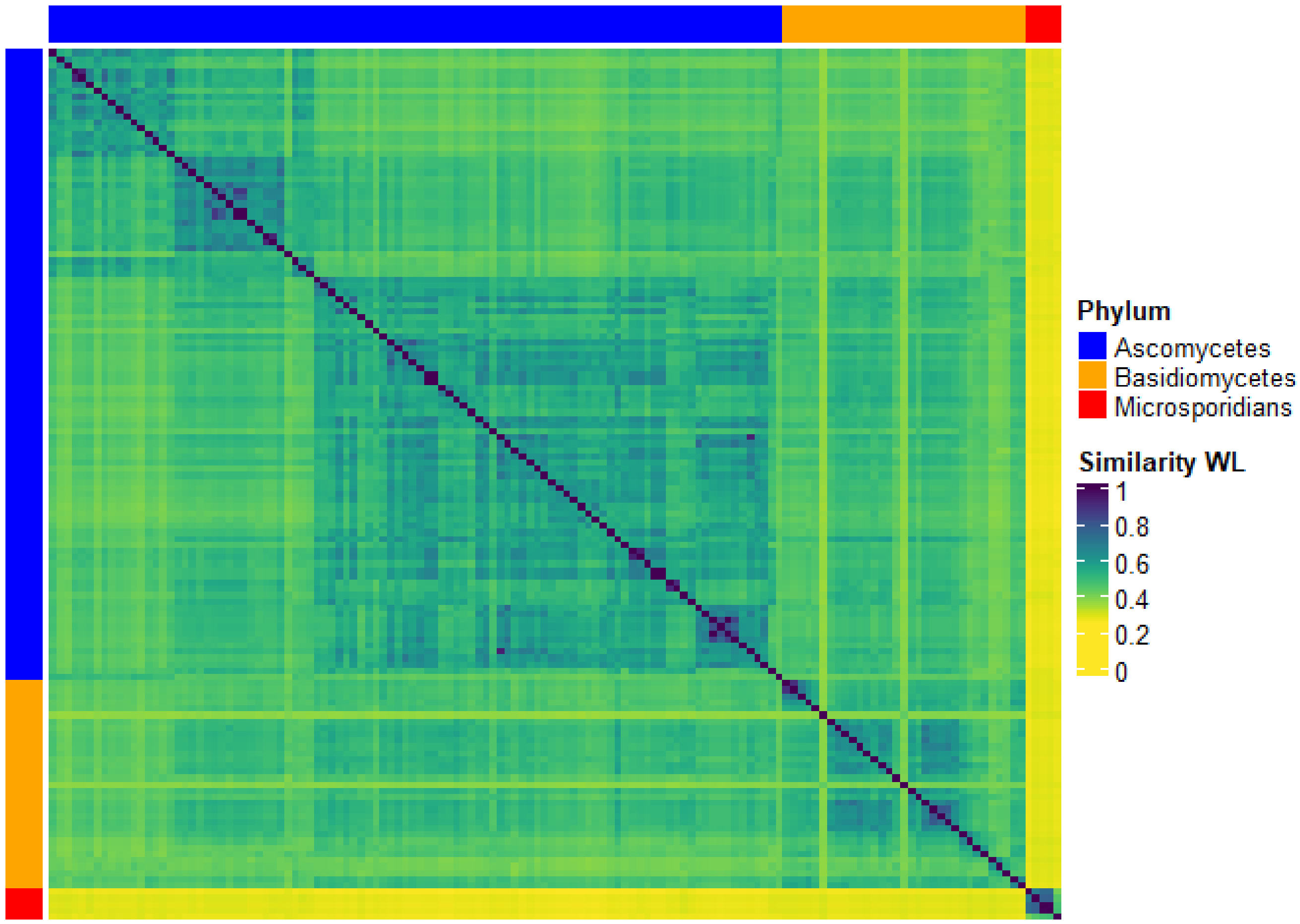

Heatmaps with threshold 1 (left side) and threshold 2 (right side)

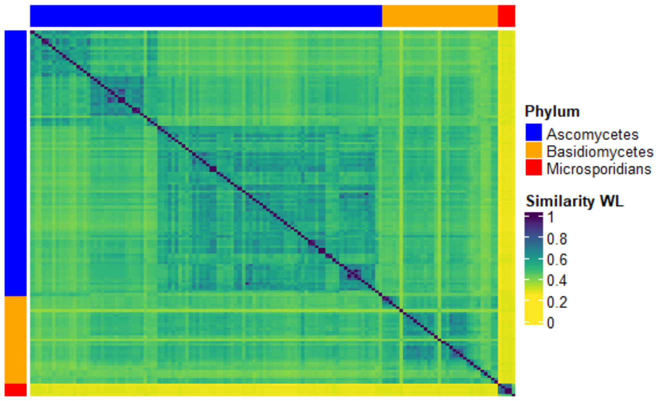

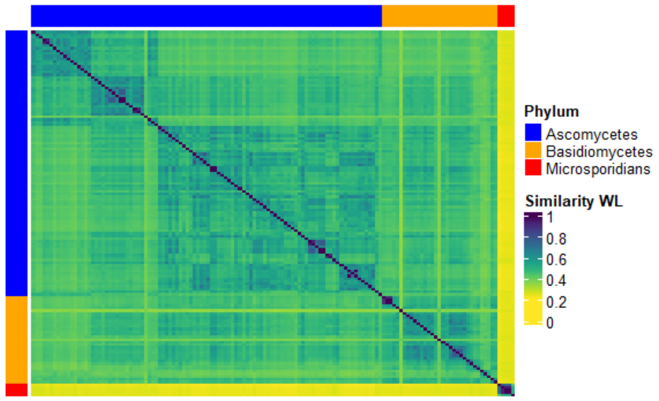

- Threshold 1: for each organism, pathways containing only one reaction are not included in the corresponding abstract metabolic network.
- Threshold 2: for each organism, pathways with only one or two reactions are not included in the corresponding abstract metabolic network.

3-means clusters for the original matrix

```
##                    Cluster
## Real group           1   2   3
```

```
##   Ascomycetes       0 42 58
##   Basidiomycetes    0 33  0
##   Microsporidians   5  0  0
```

3-means clusters for threshold 1 matrix

```
##                    Cluster
## Real group           1  2  3
##   Ascomycetes       0 58 42
##   Basidiomycetes    0  0 33
##   Microsporidians   5  0  0
```

3-means clusters for threshold 2 matrix

```
##                    Cluster
## Real group           1  2  3
##   Ascomycetes       0 64 36
##   Basidiomycetes    0 28  5
##   Microsporidians   5  0  0
```

Pyramid match (PM) kernel

Heatmap of the original matrix

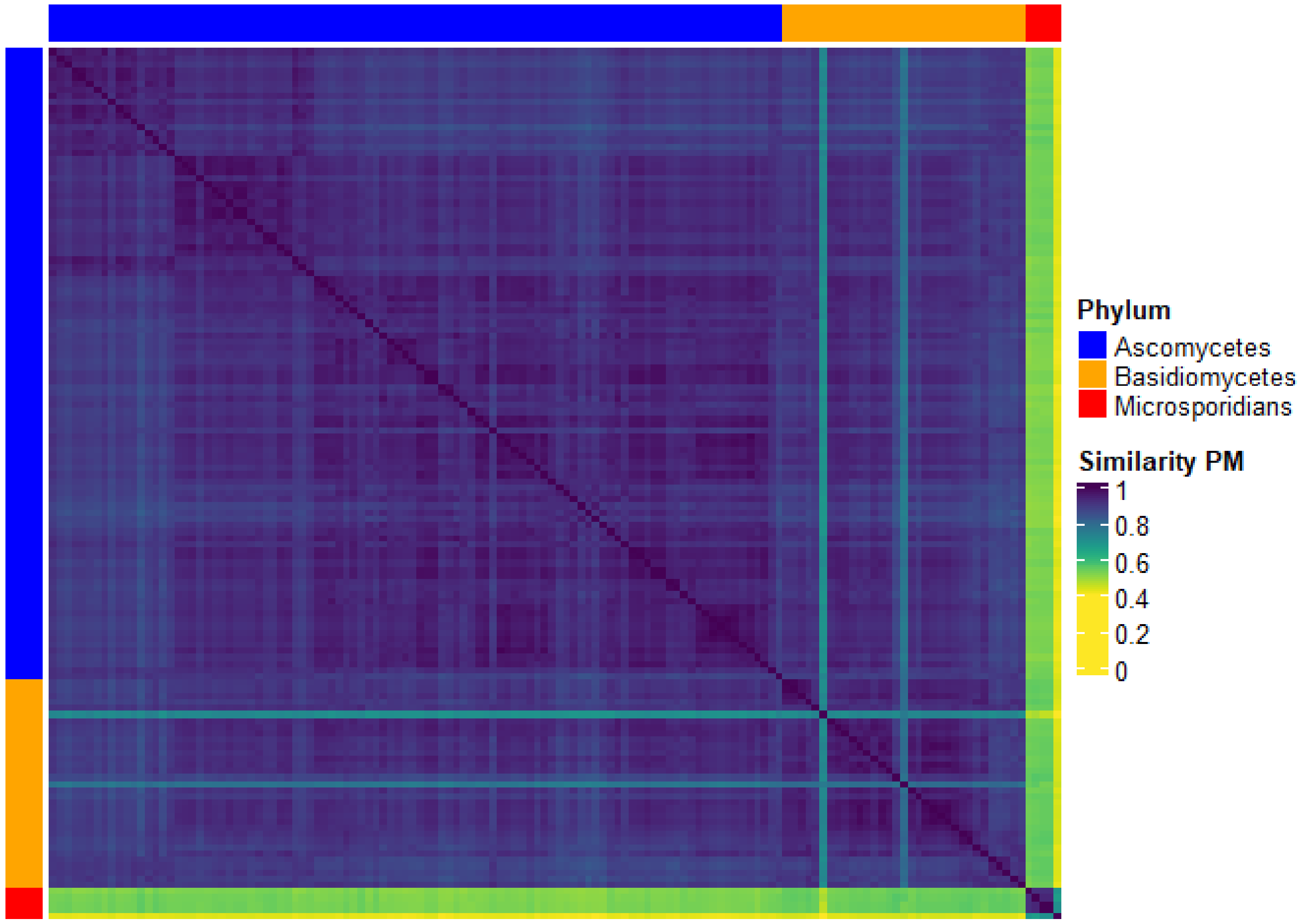

Heatmaps with threshold 1 (left side) and threshold 2 (right side)

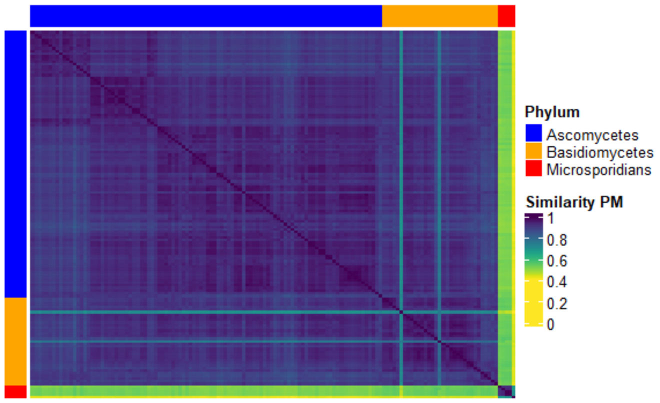

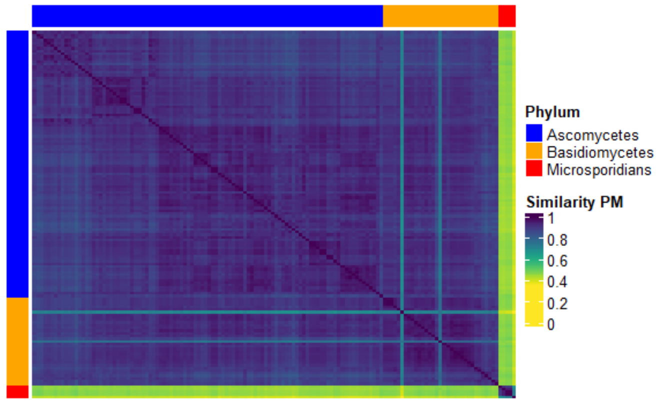

- Threshold 1: for each organism, pathways containing only one reaction are not included in the corresponding abstract metabolic network.
- Threshold 2: for each organism, pathways with only one or two reactions are not included in the corresponding abstract metabolic network.

3-means clusters for the original matrix

```
##                  Cluster
## Real group         1  2  3
##   Ascomycetes     28  0 72
```

```
##   Basidiomycetes  12  1 20
##   Microsporidians  0  5  0
```

3-means clusters for threshold 1 matrix

```
##                  Cluster
## Real group         1  2  3
##   Ascomycetes     71 29  0
##   Basidiomycetes  20 12  1
##   Microsporidians  0  0  5
```

3-means clusters for threshold 2 matrix

```
##                  Cluster
## Real group         1  2  3
##   Ascomycetes     22 78  0
##   Basidiomycetes   8 25  0
##   Microsporidians  0  0  5
```

Protists Analysis

Different thresholds

- Vertex hystogram (VH) kernel
  - Heatmap of the original matrix
  - Heatmaps with threshold 1 (left side) and threshold 2 (right side)
  - Optimal number of clusters for the original matrix
  - Optimal number of clusters for threshold 1 matrix
  - Optimal number of clusters for threshold 2 matrix
- Shortest Path (SP) kernel
  - Heatmap of the original matrix
  - Heatmaps with threshold 1 (left side) and threshold 2 (right side)
  - Optimal number of clusters for the original matrix
  - Optimal number of clusters for threshold 1 matrix
  - Optimal number of clusters for threshold 2 matrix
- Weisfeiler-Lehman (WL) kernel
  - Heatmap of the original matrix
  - Heatmaps with threshold 1 (left side) and threshold 2 (right side)
  - Optimal number of clusters for the original matrix
  - Optimal number of clusters for threshold 1 matrix
  - Optimal number of clusters for threshold 2 matrix
- Pyramid match (PM) kernel
  - Heatmap of the original matrix
  - Heatmaps with threshold 1 (left side) and threshold 2 (right side)
  - Optimal number of clusters for the original matrix
  - Optimal number of clusters for threshold 1 matrix
  - Optimal number of clusters for threshold 2 matrix

Vertex hystogram (VH) kernel

Heatmap of the original matrix

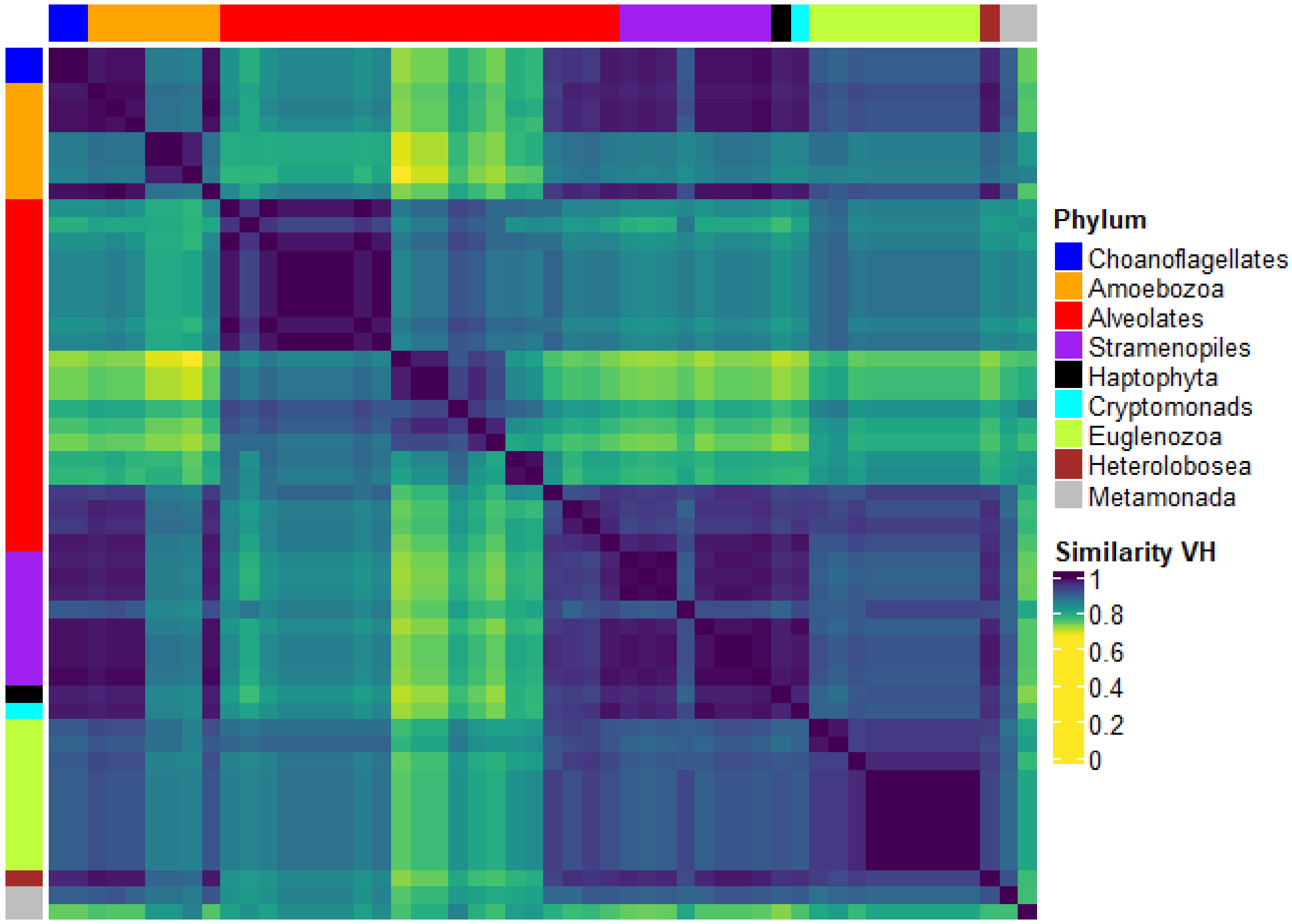

Heatmaps with threshold 1 (left side) and threshold 2 (right side)

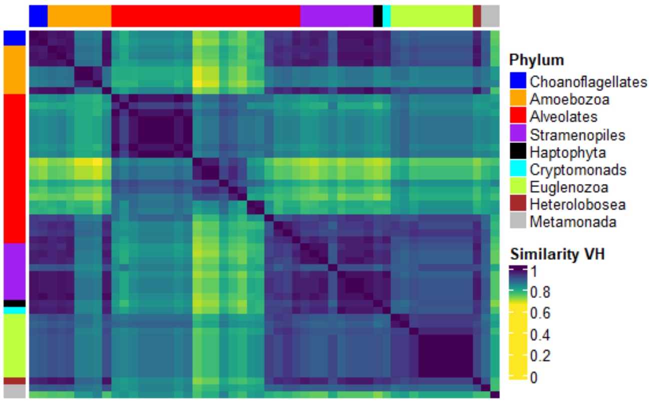

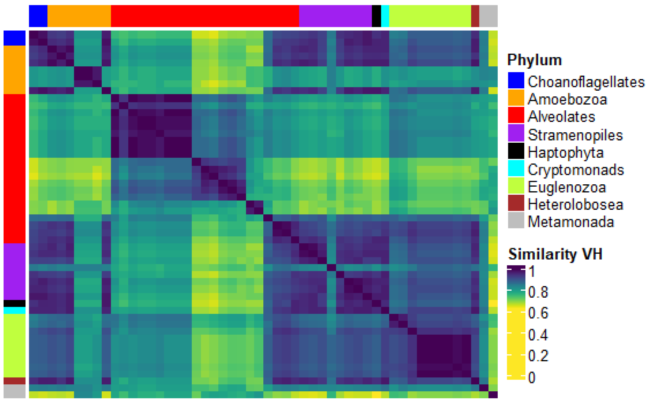

- Threshold 1: for each organism, pathways containing only one reaction are not included in the corresponding abstract metabolic network.
- Threshold 2: for each organism, pathways with only one or two reactions are not included in the corresponding abstract metabolic network.

Optimal number of clusters for the original matrix

```
##                      Cluster
## Real group            1  2  3  4
##    Alveolates         3  9  8  1
##    Amoebozoa          4  0  0  3
```

```
##   Choanoflagellates 2 0 0 0
##   Cryptomonads      1 0 0 0
##   Euglenozoa        0 0 0 9
##   Haptophyta        1 0 0 0
##   Heterolobosea     1 0 0 0
##   Metamonada        0 0 1 1
##   Stramenopiles     7 0 0 1
```

Optimal number of clusters for threshold 1 matrix

```
##                    Cluster
## Real group          1 2 3 4
##   Alveolates        9 2 2 8
##   Amoebozoa         0 4 3 0
##   Choanoflagellates 0 2 0 0
##   Cryptomonads      0 1 0 0
##   Euglenozoa        0 0 9 0
##   Haptophyta        0 1 0 0
##   Heterolobosea     0 1 0 0
##   Metamonada        0 0 1 1
##   Stramenopiles     0 7 1 0
```

Optimal number of clusters for threshold 2 matrix

```
##                    Cluster
## Real group          1 2 3 4
##   Alveolates        0 4 9 8
##   Amoebozoa         3 4 0 0
##   Choanoflagellates 0 2 0 0
##   Cryptomonads      0 1 0 0
##   Euglenozoa        0 9 0 0
##   Haptophyta        0 1 0 0
##   Heterolobosea     0 1 0 0
##   Metamonada        1 0 0 1
##   Stramenopiles     1 7 0 0
```

Shortest Path (SP) kernel

Heatmap of the original matrix

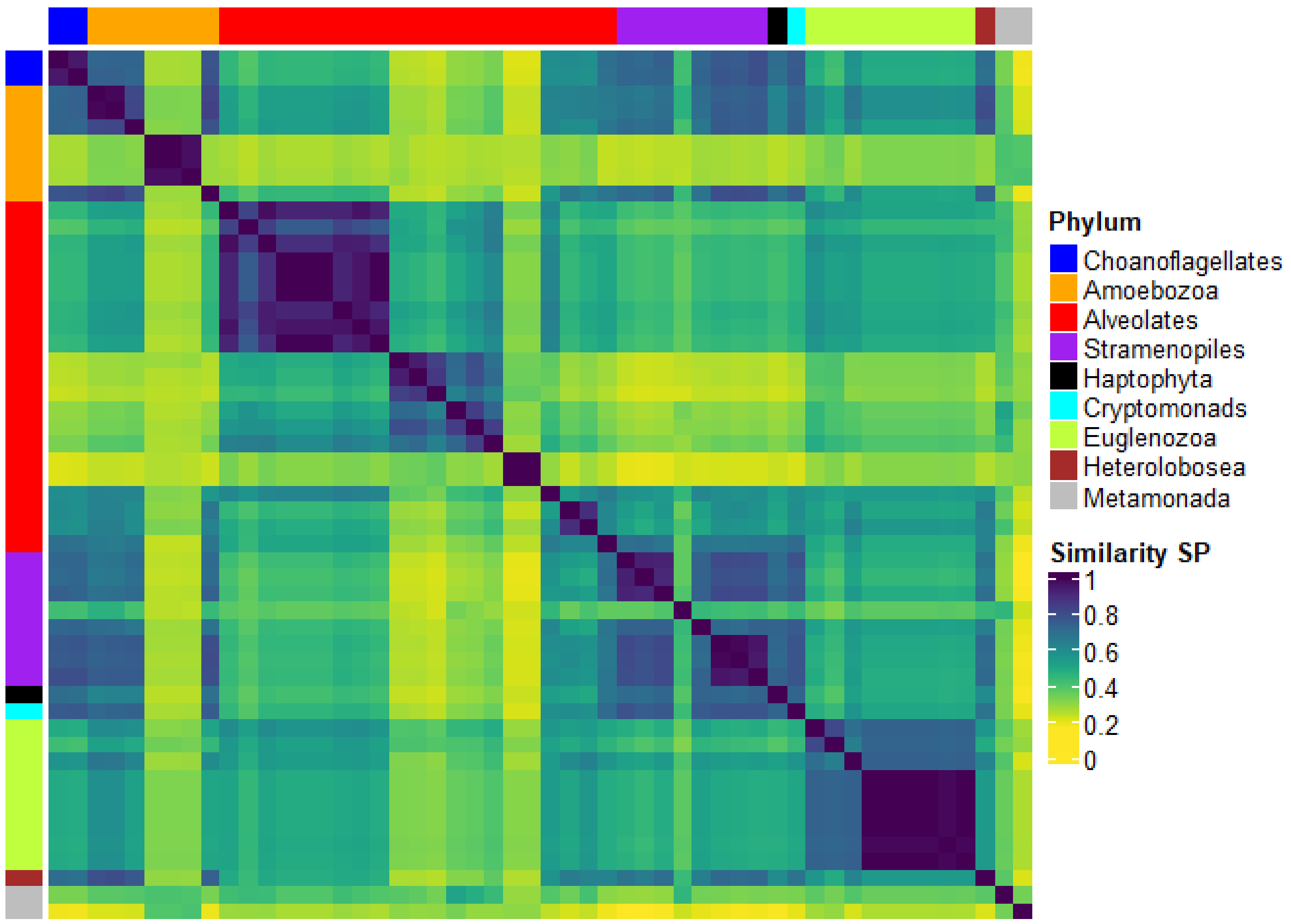

Heatmaps with threshold 1 (left side) and threshold 2 (right side)

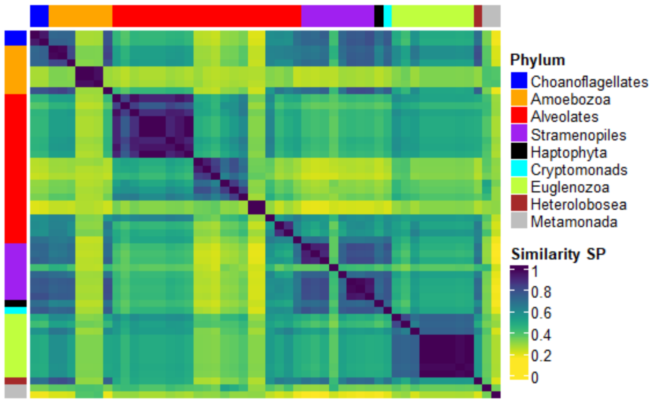

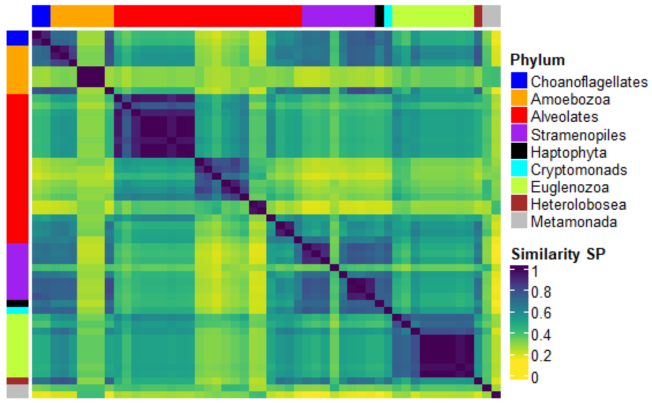

- Threshold 1: for each organism, pathways containing only one reaction are not included in the corresponding abstract metabolic network.
- Threshold 2: for each organism, pathways with only one or two reactions are not included in the corresponding abstract metabolic network.

Optimal number of clusters for the original matrix

```
##                      Cluster
## Real group            1 2 3 4
##   Alveolates          0 4 8 9
##   Amoebozoa           0 4 3 0
```

```
##   Choanoflagellates 0 2 0 0
##   Cryptomonads      0 1 0 0
##   Euglenozoa        9 0 0 0
##   Haptophyta        0 1 0 0
##   Heterolobosea     0 1 0 0
##   Metamonada        0 0 2 0
##   Stramenopiles     0 7 1 0
```

Optimal number of clusters for threshold 1 matrix

```
##                     Cluster
## Real group           1 2 3 4
##   Alveolates         4 0 8 9
##   Amoebozoa          4 0 3 0
##   Choanoflagellates 2 0 0 0
##   Cryptomonads      1 0 0 0
##   Euglenozoa        0 9 0 0
##   Haptophyta        1 0 0 0
##   Heterolobosea     1 0 0 0
##   Metamonada        0 0 2 0
##   Stramenopiles     7 0 1 0
```

Optimal number of clusters for threshold 2 matrix

```
##                     Cluster
## Real group           1 2 3 4
##   Alveolates         8 0 9 4
##   Amoebozoa          3 0 0 4
##   Choanoflagellates 0 0 0 2
##   Cryptomonads      0 0 0 1
##   Euglenozoa        0 9 0 0
##   Haptophyta        0 0 0 1
##   Heterolobosea     0 0 0 1
##   Metamonada        2 0 0 0
##   Stramenopiles     1 0 0 7
```

Weisfeiler-Lehman (WL) kernel

Heatmap of the original matrix

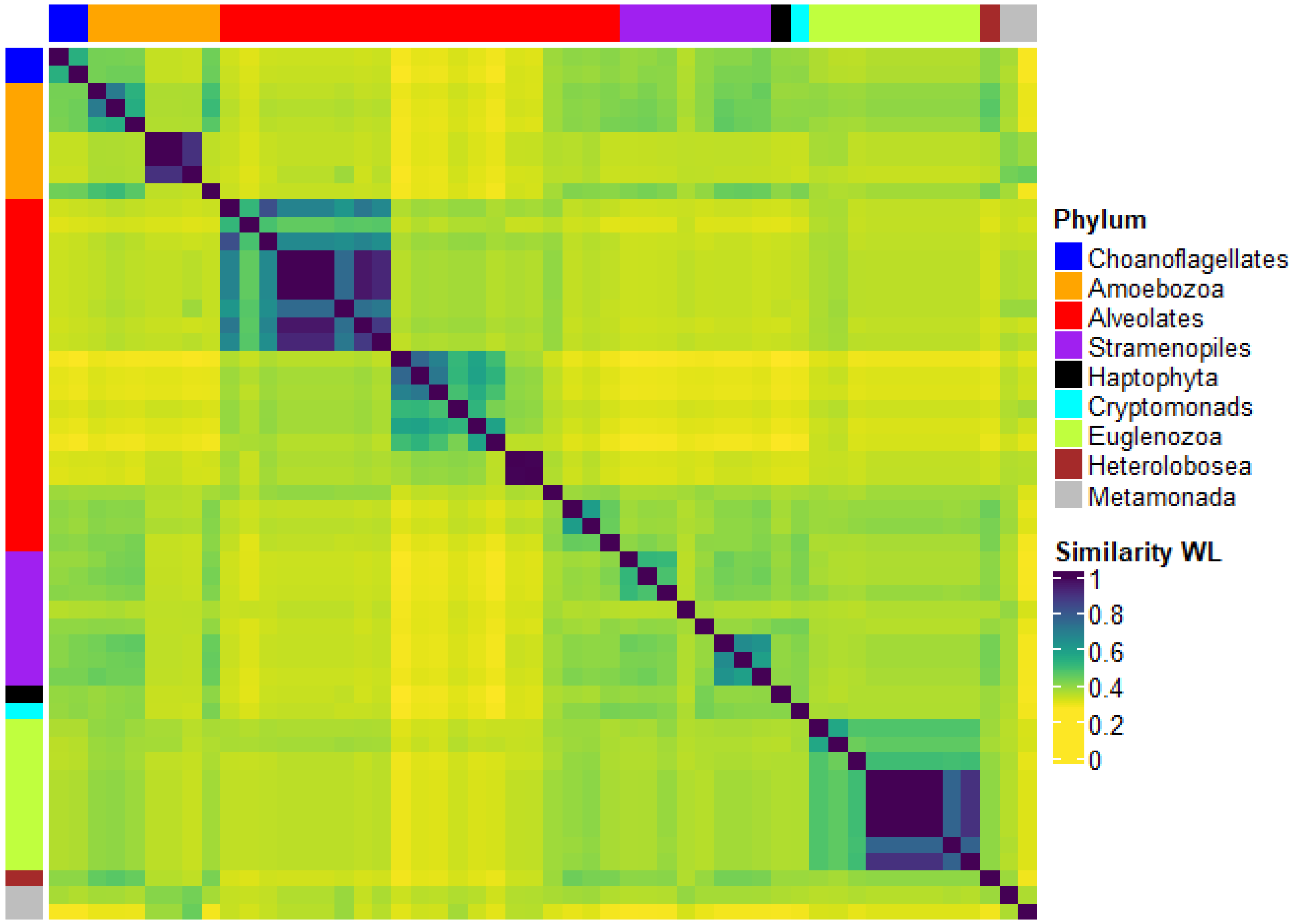

Heatmaps with threshold 1 (left side) and threshold 2 (right side)

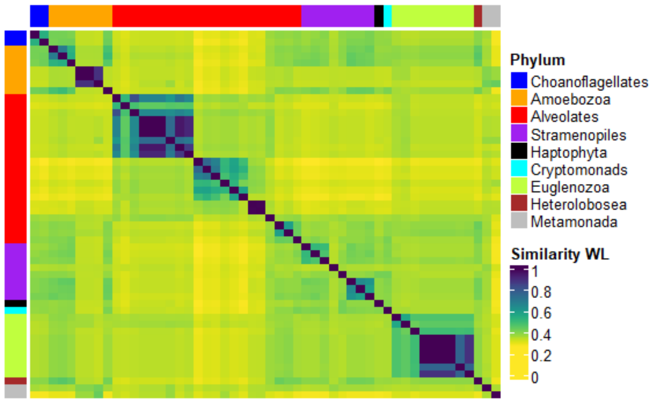

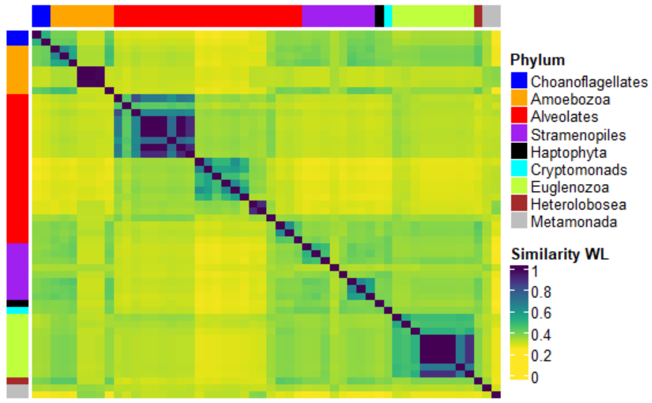

- Threshold 1: for each organism, pathways containing only one reaction are not included in the corresponding abstract metabolic network.
- Threshold 2: for each organism, pathways with only one or two reactions are not included in the corresponding abstract metabolic network.

Optimal number of clusters for the original matrix

```
##                      Cluster
## Real group            1  2  3  4
##    Alveolates         0  4  8  9
##    Amoebozoa          0  7  0  0
```

```
##    Choanoflagellates 0 2 0 0
##    Cryptomonads      0 1 0 0
##    Euglenozoa        6 3 0 0
##    Haptophyta        0 1 0 0
##    Heterolobosea     0 1 0 0
##    Metamonada        0 1 0 1
##    Stramenopiles     0 8 0 0
```

Optimal number of clusters for threshold 1 matrix

```
##                      Cluster
## Real group            1 2 3 4
##    Alveolates         0 4 8 9
##    Amoebozoa          0 7 0 0
##    Choanoflagellates 0 2 0 0
##    Cryptomonads      0 1 0 0
##    Euglenozoa        6 3 0 0
##    Haptophyta        0 1 0 0
##    Heterolobosea     0 1 0 0
##    Metamonada        0 1 0 1
##    Stramenopiles     0 8 0 0
```

Optimal number of clusters for threshold 2 matrix

```
##                      Cluster
## Real group            1 2 3 4
##    Alveolates         0 8 4 9
##    Amoebozoa          0 0 4 3
##    Choanoflagellates 0 0 2 0
##    Cryptomonads      0 0 1 0
##    Euglenozoa        6 0 3 0
##    Haptophyta        0 0 1 0
##    Heterolobosea     0 0 1 0
##    Metamonada        0 0 1 1
##    Stramenopiles     0 0 8 0
```

Pyramid match (PM) kernel

Heatmap of the original matrix

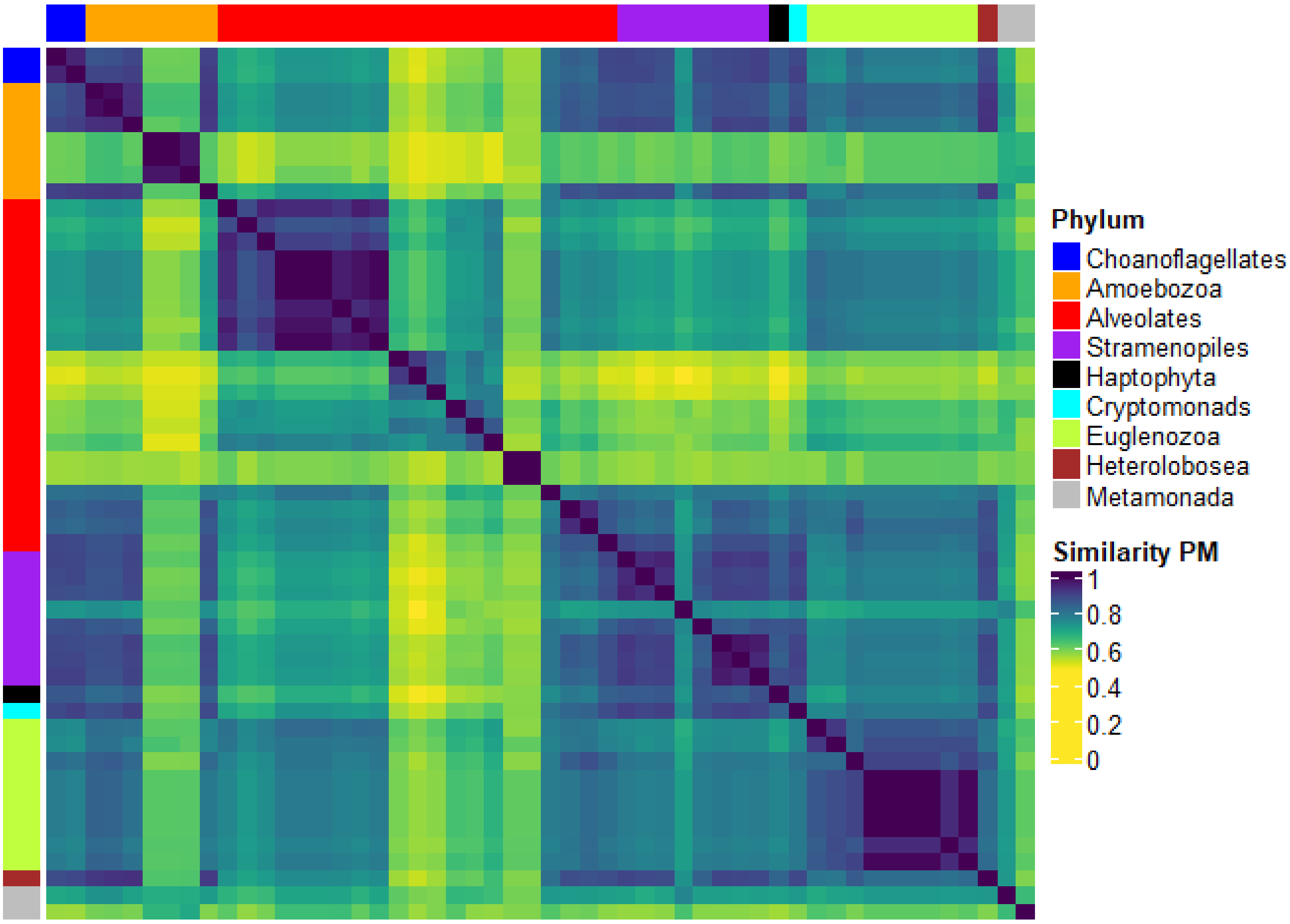

Heatmaps with threshold 1 (left side) and threshold 2 (right side)

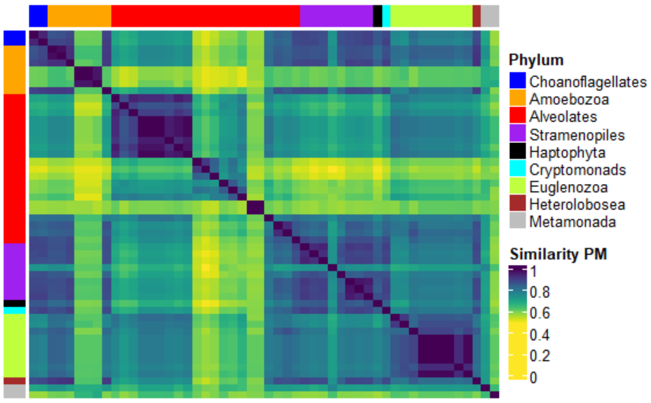

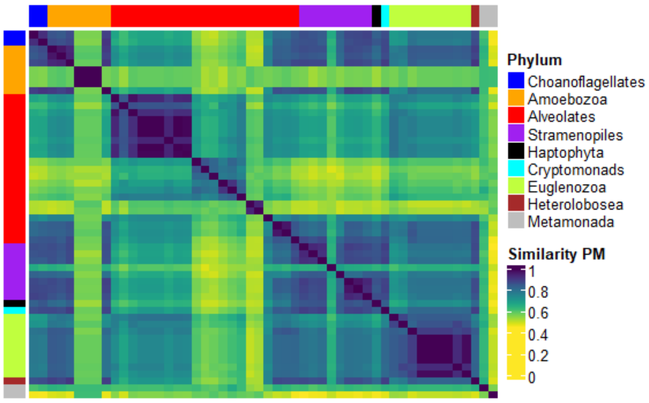

- Threshold 1: for each organism, pathways containing only one reaction are not included in the corresponding abstract metabolic network.
- Threshold 2: for each organism, pathways with only one or two reactions are not included in the corresponding abstract metabolic network.

Optimal number of clusters for the original matrix

```
##                      Cluster
## Real group            1  2  3  4
##   Alveolates          4  0  9  8
##   Amoebozoa           4  0  0  3
```

```
##    Choanoflagellates 2 0 0 0
##    Cryptomonads      1 0 0 0
##    Euglenozoa        0 9 0 0
##    Haptophyta        1 0 0 0
##    Heterolobosea     1 0 0 0
##    Metamonada        0 0 0 2
##    Stramenopiles     8 0 0 0
```

Optimal number of clusters for threshold 1 matrix

```
##                      Cluster
## Real group            1 2 3 4
##    Alveolates        8 4 9 0
##    Amoebozoa         3 4 0 0
##    Choanoflagellates 0 2 0 0
##    Cryptomonads      0 1 0 0
##    Euglenozoa        0 0 0 9
##    Haptophyta        0 1 0 0
##    Heterolobosea     0 1 0 0
##    Metamonada        2 0 0 0
##    Stramenopiles     0 8 0 0
```

Optimal number of clusters for threshold 2 matrix

```
##                      Cluster
## Real group            1 2 3 4
##    Alveolates        8 4 0 9
##    Amoebozoa         3 4 0 0
##    Choanoflagellates 0 2 0 0
##    Cryptomonads      0 1 0 0
##    Euglenozoa        0 0 9 0
##    Haptophyta        0 1 0 0
##    Heterolobosea     0 1 0 0
##    Metamonada        2 0 0 0
##    Stramenopiles     1 7 0 0
```

Bacteria Restricted Analysis

Different thresholds

- Vertex hystogram (VH) kernel
  - Heatmap of the original matrix
  - Heatmaps with threshold 1 (left side) and threshold 2 (right side)
  - 5-means clusters forh the original matrix
  - 5-means clusters for threshold 1 matrix
  - 5-means clusters for threshold 2 matrix
- Shortest Path (SP) kernel
  - Heatmap of the original matrix
  - Heatmaps with threshold 1 (left side) and threshold 2 (right side)
  - 5-means clusters for the original matrix
  - 5-means clusters for threshold 1 matrix
  - 5-means clusters for threshold 2 matrix
- Weisfeiler-Lehman (WL) kernel
  - Heatmap of the original matrix
  - Heatmaps with threshold 1 (left side) and threshold 2 (right side)
  - 5-means clusters for the original matrix
  - 5-means clusters for threshold 1 matrix
  - 5-means clusters for threshold 2 matrix
- Pyramid match (PM) kernel
  - Heatmap of the original matrix
  - Heatmaps with threshold 1 (left side) and threshold 2 (right side)
  - 5-means clusters for the original matrix
  - 5-means clusters for threshold 1 matrix
  - 5-means clusters for threshold 2 matrix

Vertex hystogram (VH) kernel

Heatmap of the original matrix

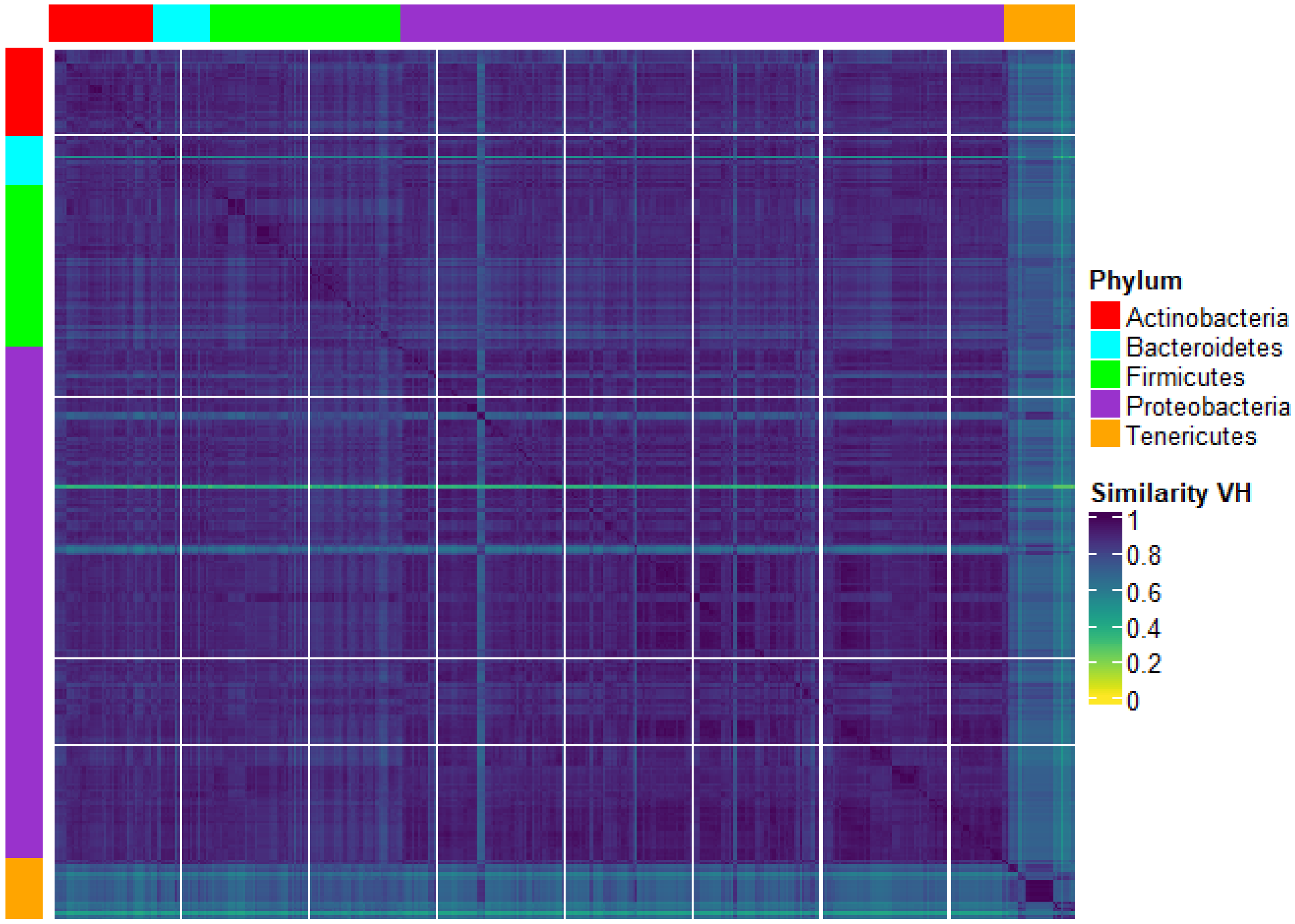

Heatmaps with threshold 1 (left side) and threshold 2 (right side)

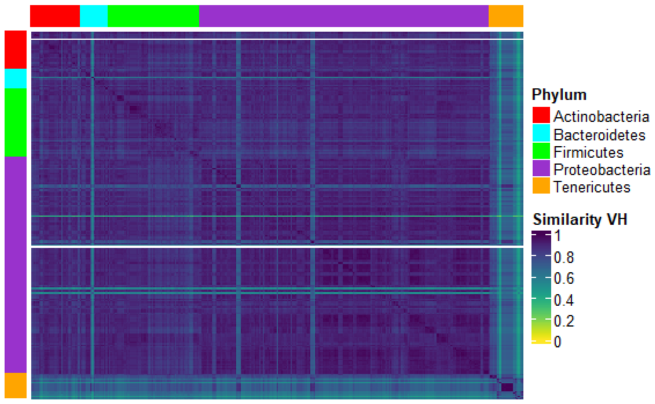

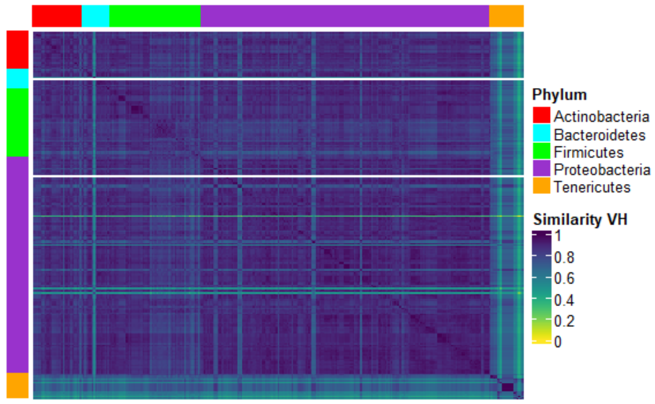

- Threshold 1: for each organism, pathways containing only one reaction are not included in the corresponding abstract metabolic network.
- Threshold 2: for each organism, pathways with only one or two reactions are not included in the corresponding abstract metabolic network.

5-means clusters forh the original matrix

```
##                    Cluster
## Real group           1    2    3    4    5
##   Actinobacteria   195    1    1  168  106
##   Bacteroidetes     95   12    9   97   47
```

```
##    Firmicutes       102    0   11  398  358
##    Proteobacteria 1810   34   93  651  160
##    Tenericutes        1  125  192    2    4
```

5-means clusters for threshold 1 matrix

```
##                   Cluster
## Real group           1    2    3    4    5
##    Actinobacteria  164  104    1    1  201
##    Bacteroidetes    95   47    9   12   97
##    Firmicutes      405  351   11    0  102
##    Proteobacteria  654  151   93   34 1816
##    Tenericutes       2    5  191  125    1
```

5-means clusters for threshold 2 matrix

```
##                   Cluster
## Real group           1    2    3    4    5
##    Actinobacteria    1    4  166  150  150
##    Bacteroidetes    12    9   65  168    6
##    Firmicutes        0   47  518  217   87
##    Proteobacteria   35  102  300 1324  985
##    Tenericutes     145  173    5    1    0
```

Shortest Path (SP) kernel

Heatmap of the original matrix

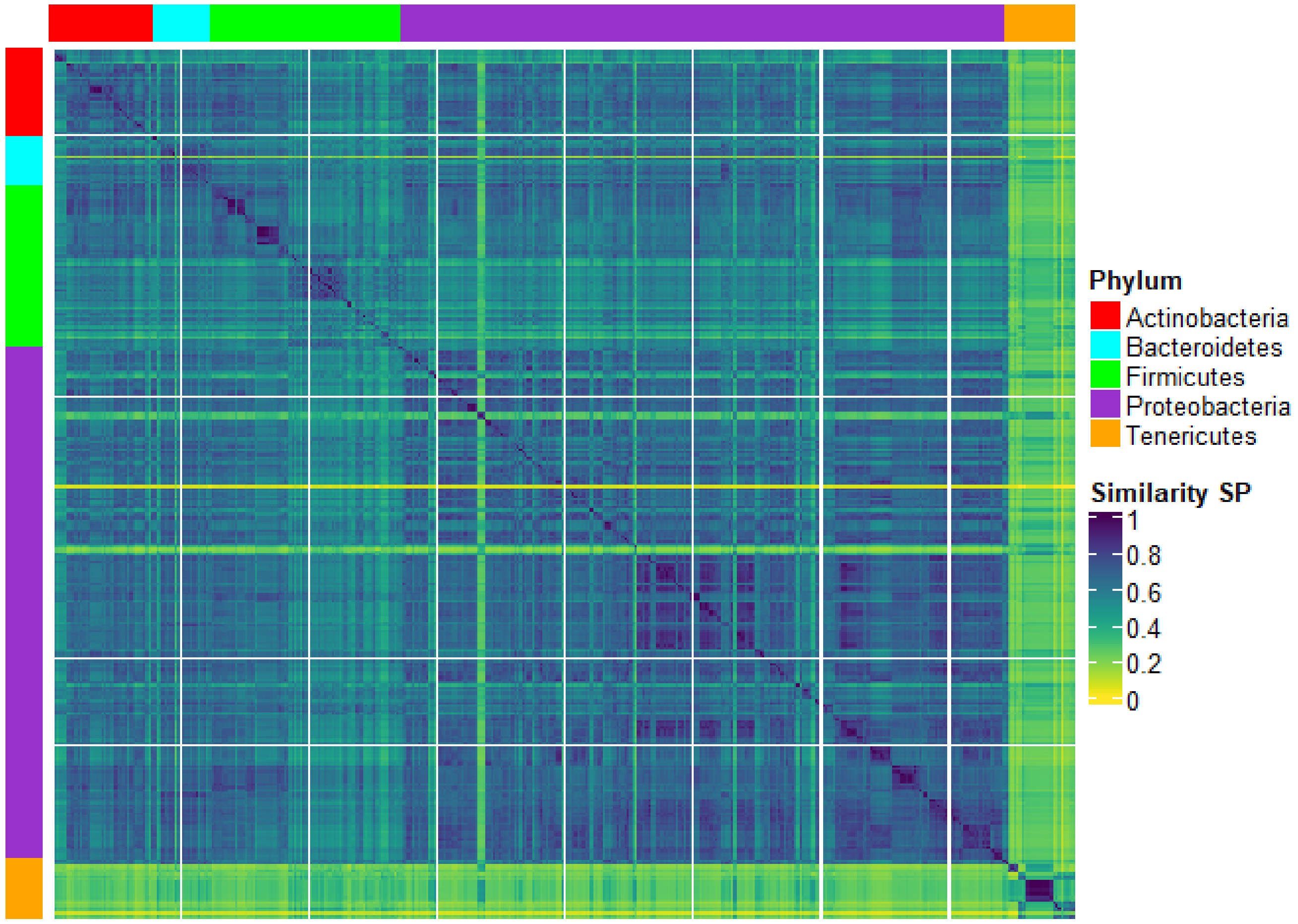

Heatmaps with threshold 1 (left side) and threshold 2 (right side)

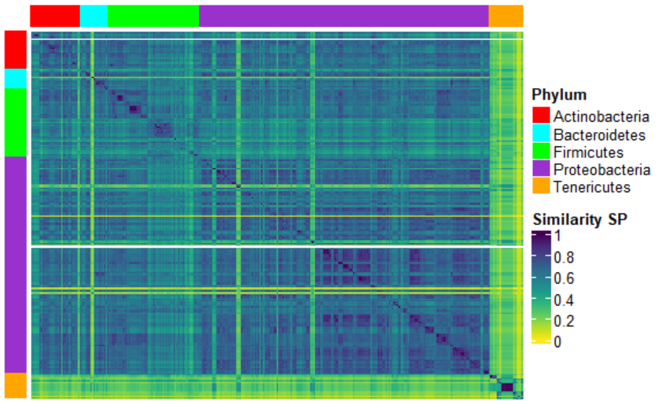

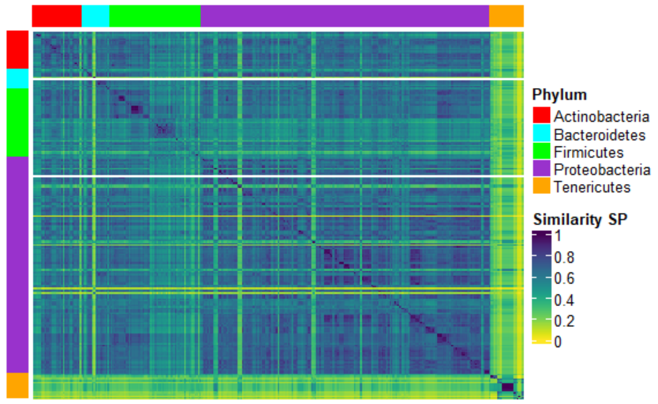

- Threshold 1: for each organism, pathways containing only one reaction are not included in the corresponding abstract metabolic network.
- Threshold 2: for each organism, pathways with only one or two reactions are not included in the corresponding abstract metabolic network.

5-means clusters for the original matrix

```
##                    Cluster
## Real group           1    2    3    4    5
##   Actinobacteria    77  126  125    5  138
##   Bacteroidetes     38    0   65   21  136
```

```
##    Firmicutes       311   60  348   10  140
##    Proteobacteria  117  962  435  107 1127
##    Tenericutes       7    0    2  315    0
```

5-means clusters for threshold 1 matrix

```
##                    Cluster
## Real group            1    2    3    4    5
##    Actinobacteria   136   77  126    5  127
##    Bacteroidetes    136   38    0   21   65
##    Firmicutes       141  311   59   10  348
##    Proteobacteria  1122  117  965  107  437
##    Tenericutes        0    7    0  315    2
```

5-means clusters for threshold 2 matrix

```
##                    Cluster
## Real group            1    2    3    4    5
##    Actinobacteria     8  142  167    1  153
##    Bacteroidetes     15   14  154   12   65
##    Firmicutes        66   49  313    0  441
##    Proteobacteria   105 1518  781   40  302
##    Tenericutes      167    0    2  153    2
```

Weisfeiler-Lehman (WL) kernel

Heatmap of the original matrix

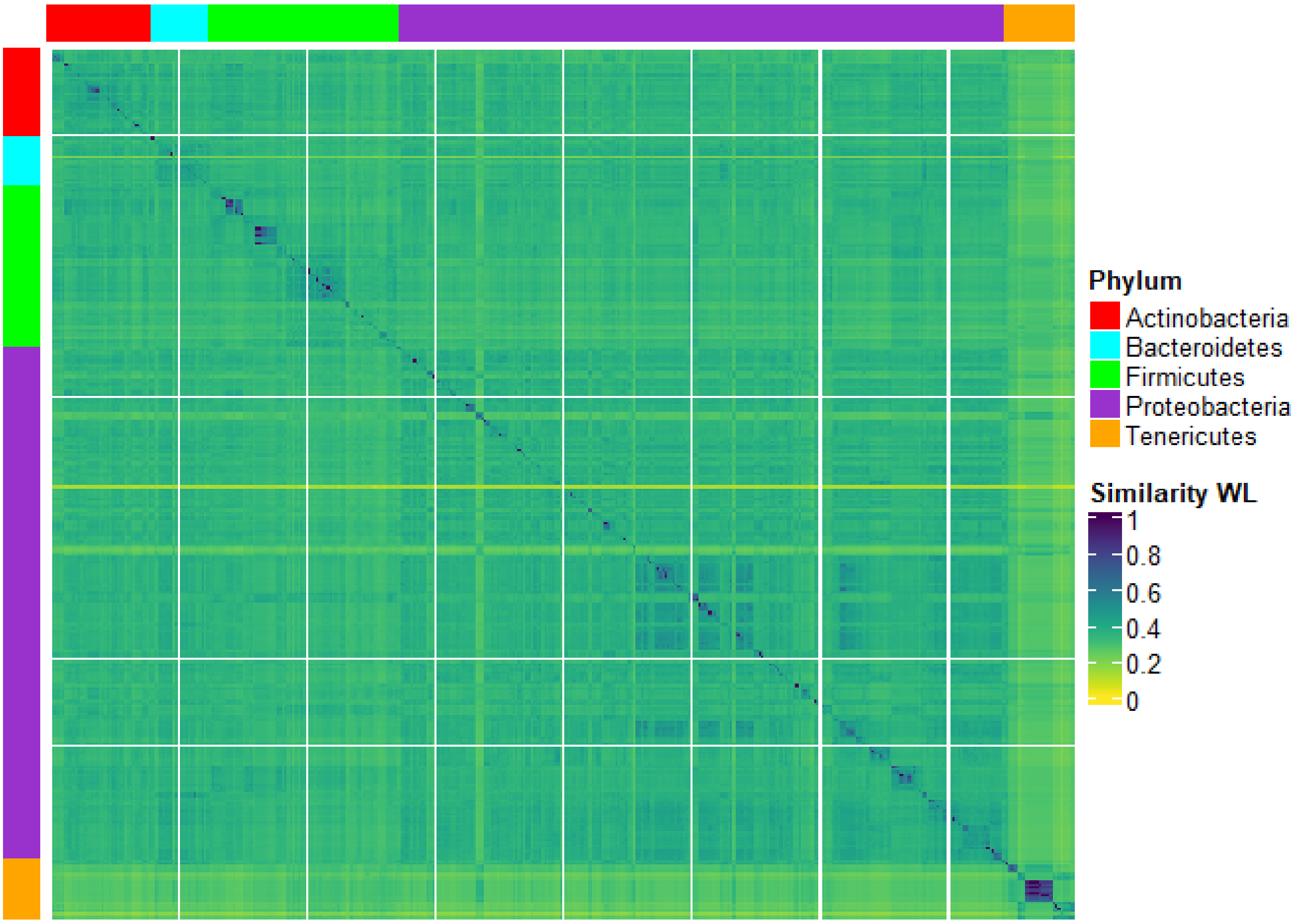

Heatmaps with threshold 1 (left side) and threshold 2 (right side)

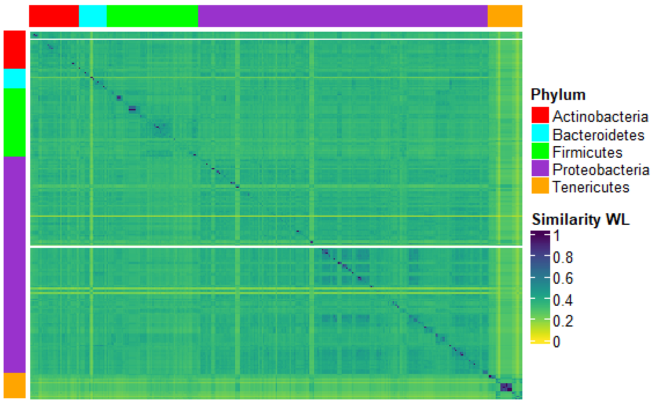

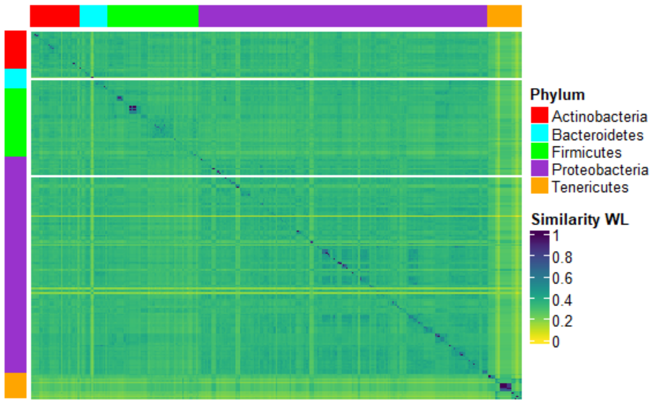

- Threshold 1: for each organism, pathways containing only one reaction are not included in the corresponding abstract metabolic network.
- Threshold 2: for each organism, pathways with only one or two reactions are not included in the corresponding abstract metabolic network.

5-means clusters for the original matrix

```
##                   Cluster
## Real group           1    2    3    4    5
##   Actinobacteria   224    0    2    8  237
##   Bacteroidetes    115   12   45    9   79
```

```
##   Firmicutes       108    0    0   75  686
##   Proteobacteria 1182   34  877  109  546
##   Tenericutes        1  130    0  186    7
```

5-means clusters for threshold 1 matrix

```
##                 Cluster
## Real group          1    2    3    4    5
##   Actinobacteria  236    1    7  226    1
##   Bacteroidetes    79   45    9  115   12
##   Firmicutes      685    0   75  109    0
##   Proteobacteria  547  876  109 1182   34
##   Tenericutes       7    0  186    1  130
```

5-means clusters for threshold 2 matrix

```
##                 Cluster
## Real group          1    2    3    4    5
##   Actinobacteria    6    1   80  222  162
##   Bacteroidetes    13   12  167   67    1
##   Firmicutes       95    0   90  593   91
##   Proteobacteria  117   34 1298  416  881
##   Tenericutes     176  143    1    4    0
```

Pyramid match (PM) kernel

Heatmap of the original matrix

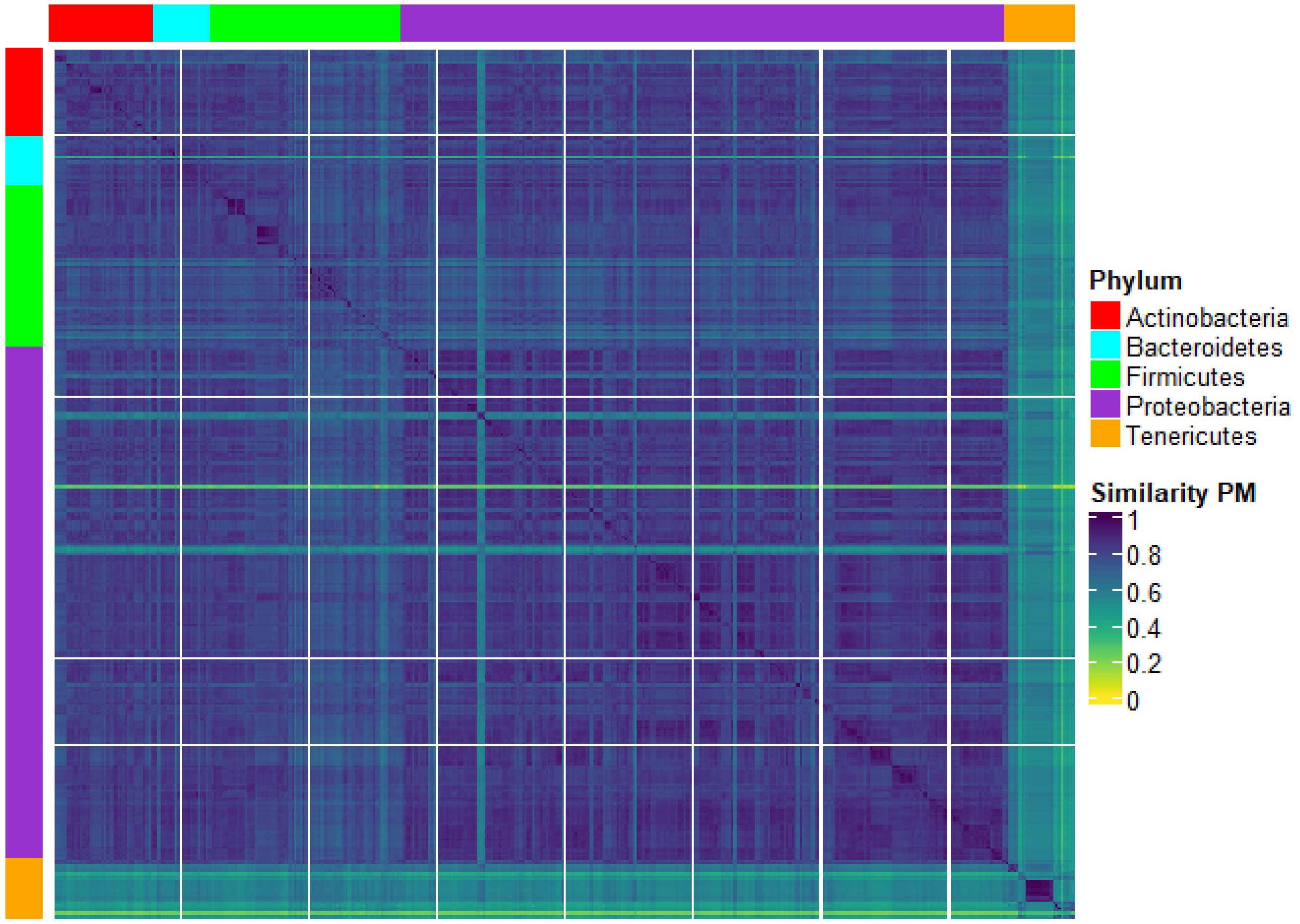

Heatmaps with threshold 1 (left side) and threshold 2 (right side)

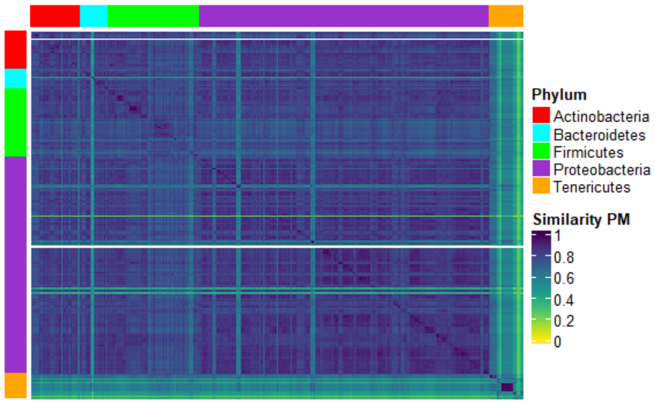

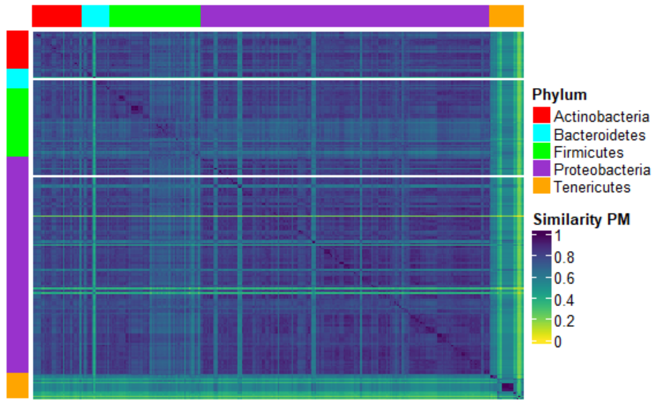

- Threshold 1: for each organism, pathways containing only one reaction are not included in the corresponding abstract metabolic network.
- Threshold 2: for each organism, pathways with only one or two reactions are not included in the corresponding abstract metabolic network.

5-means clusters for the original matrix

```
##                    Cluster
## Real group           1    2    3    4    5
##    Actinobacteria  230  157    1    1   82
##    Bacteroidetes   115   88    9   12   36
```

```
##   Firmicutes       115  381   20    0  353
##   Proteobacteria 1877  639   88   31  113
##   Tenericutes       0    2  191  127    4
```

5-means clusters for threshold 1 matrix

```
##                  Cluster
## Real group          1    2    3    4    5
##   Actinobacteria    1    1  157   82  230
##   Bacteroidetes     9   12   88   36  115
##   Firmicutes       19    0  382  353  115
##   Proteobacteria   88   31  640  112 1877
##   Tenericutes     191  127    2    4    0
```

5-means clusters for threshold 2 matrix

```
##                  Cluster
## Real group          1    2    3    4    5
##   Actinobacteria  156  160    5  149    1
##   Bacteroidetes    69  165    9    5   12
##   Firmicutes      518  213   57   81    0
##   Proteobacteria  325 1245   99 1042   35
##   Tenericutes       3    1  177    0  143
```

Pseudomonas Analysis

Threshold 1

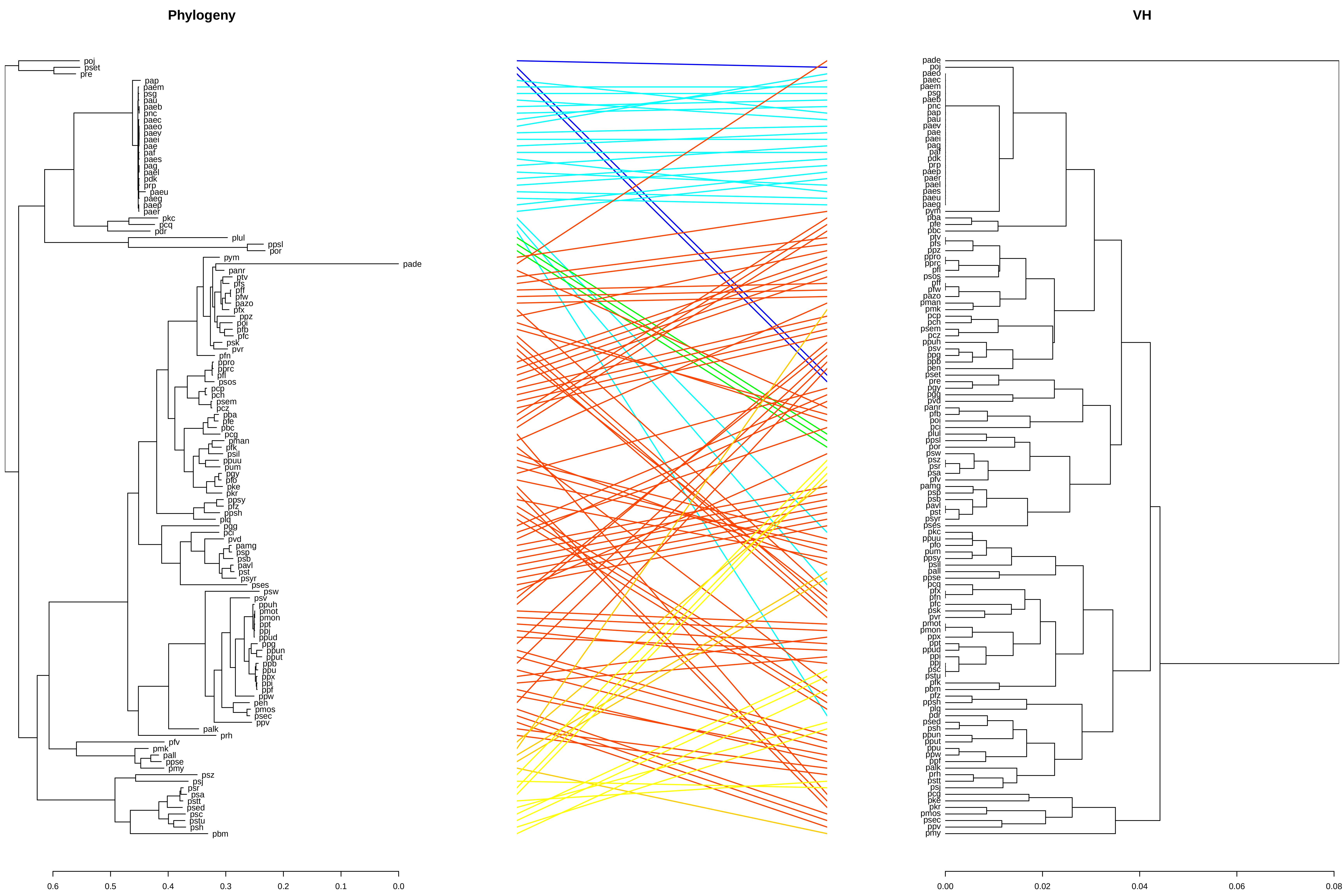
Phylogeny
VH
0.6
0.5
0.4
0.3
0.2
0.1
0.0
0.00
0.02
0.04
0.06
0.08

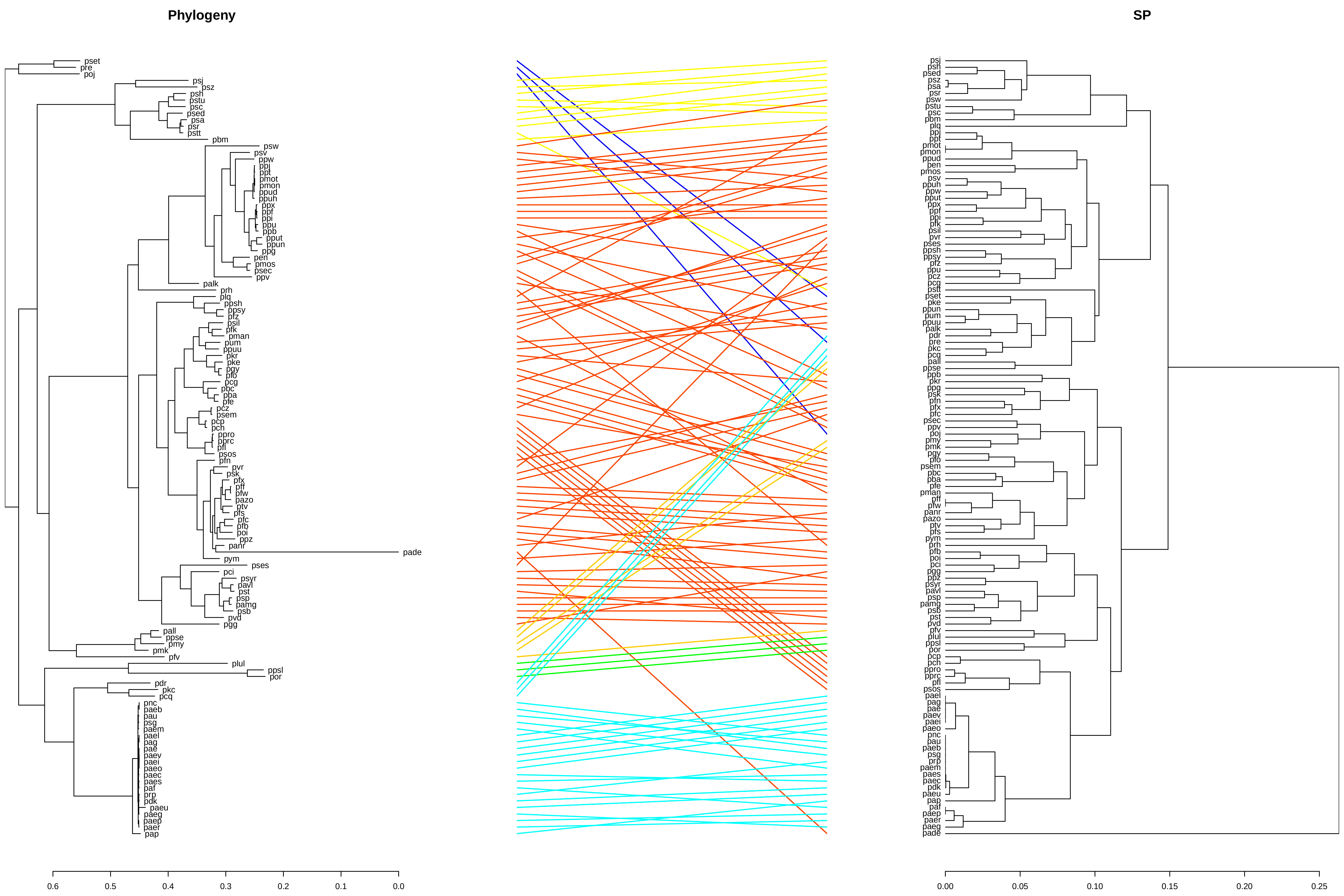
Phylogeny
SP
0.6 0.5 0.4 0.3 0.2 0.1 0.0
0.00 0.05 0.10 0.15 0.20 0.25

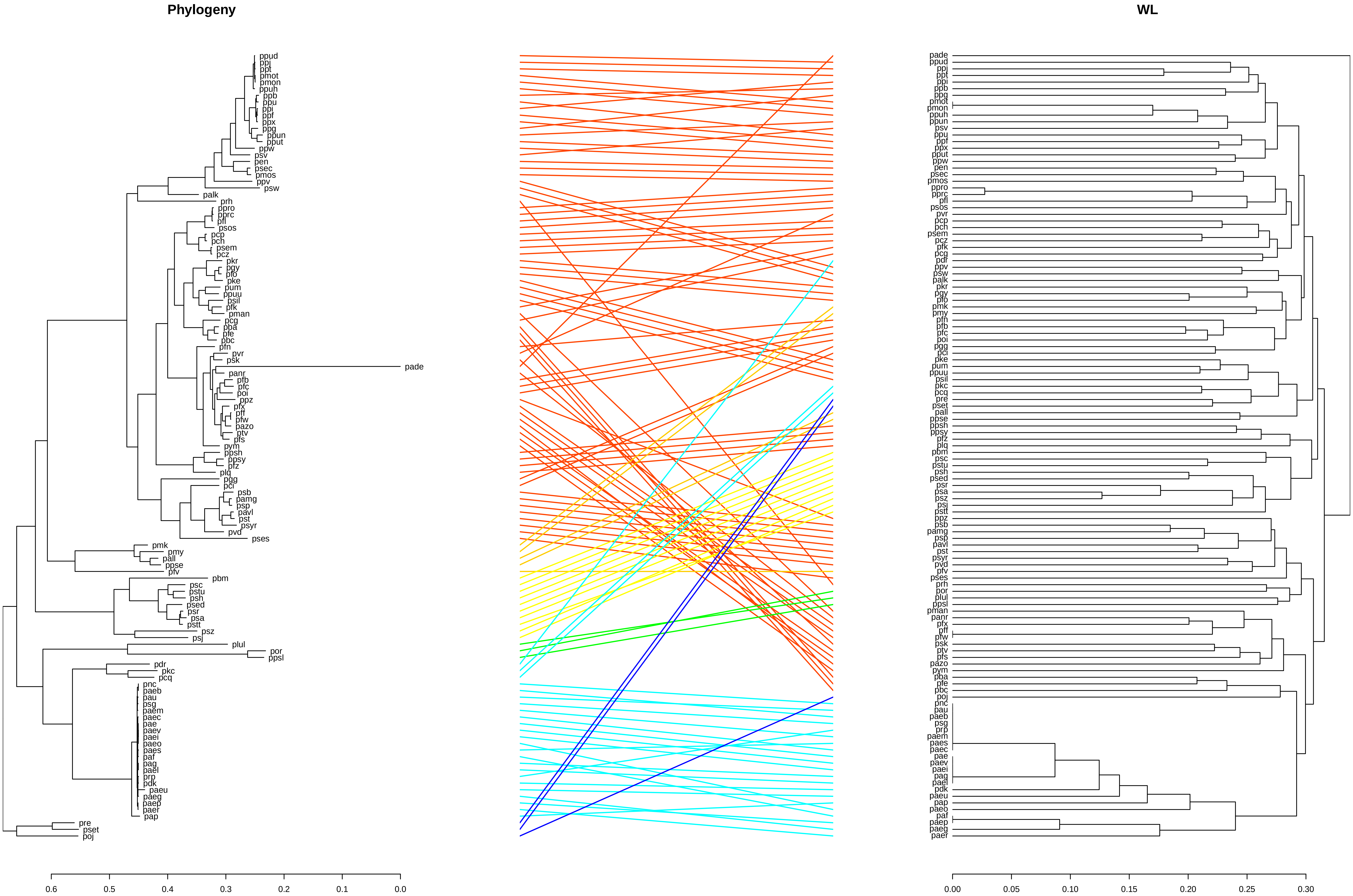
Phylogeny
WL
0.6
0.5
0.4
0.3
0.2
0.1
0.0
0.00
0.05
0.10
0.15
0.20
0.25
0.30

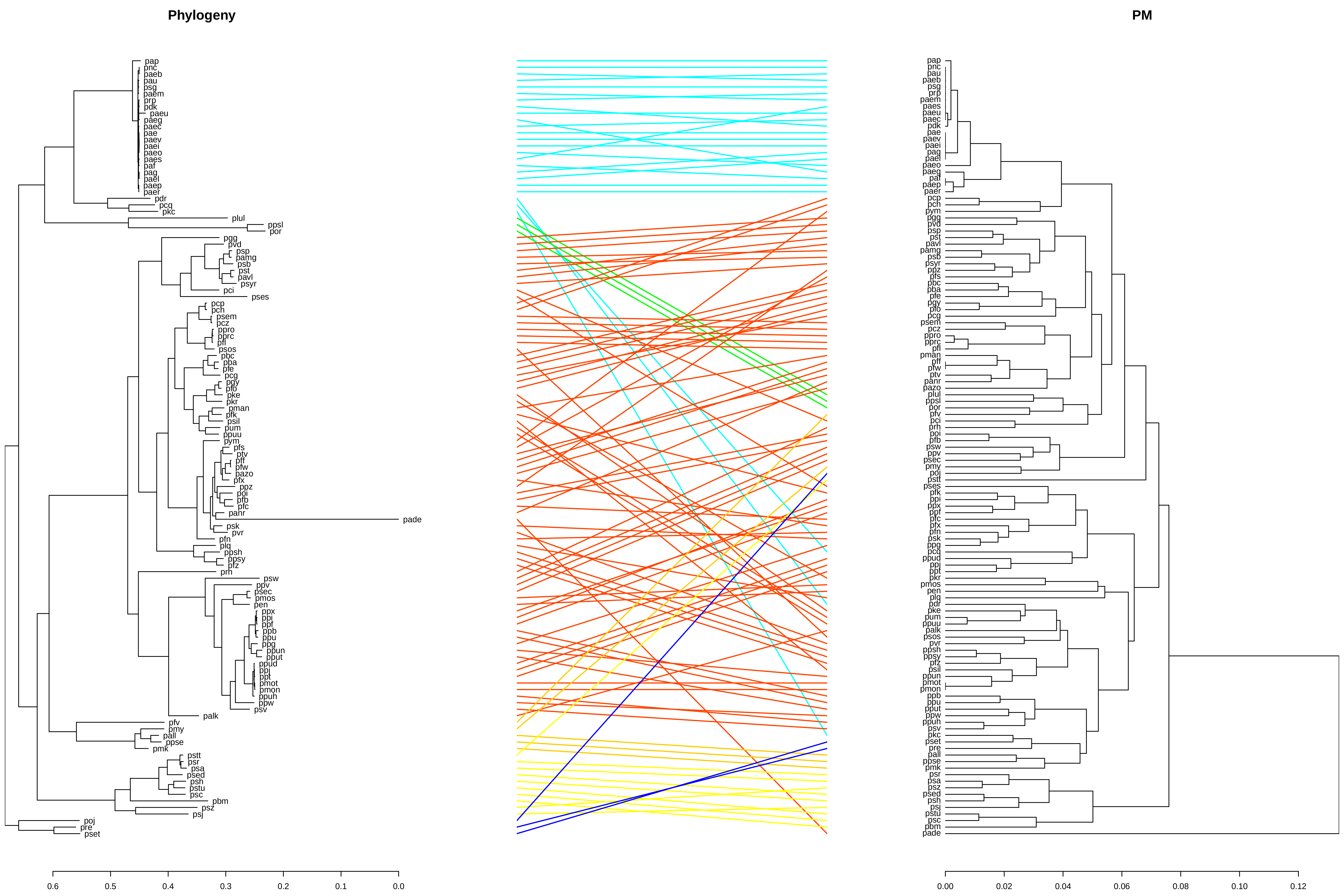

Phylogeny
PM
0.6 0.5 0.4 0.3 0.2 0.1 0.0
0.00 0.02 0.04 0.06 0.08 0.10 0.12

Pseudomonas Analysis

Threshold 2

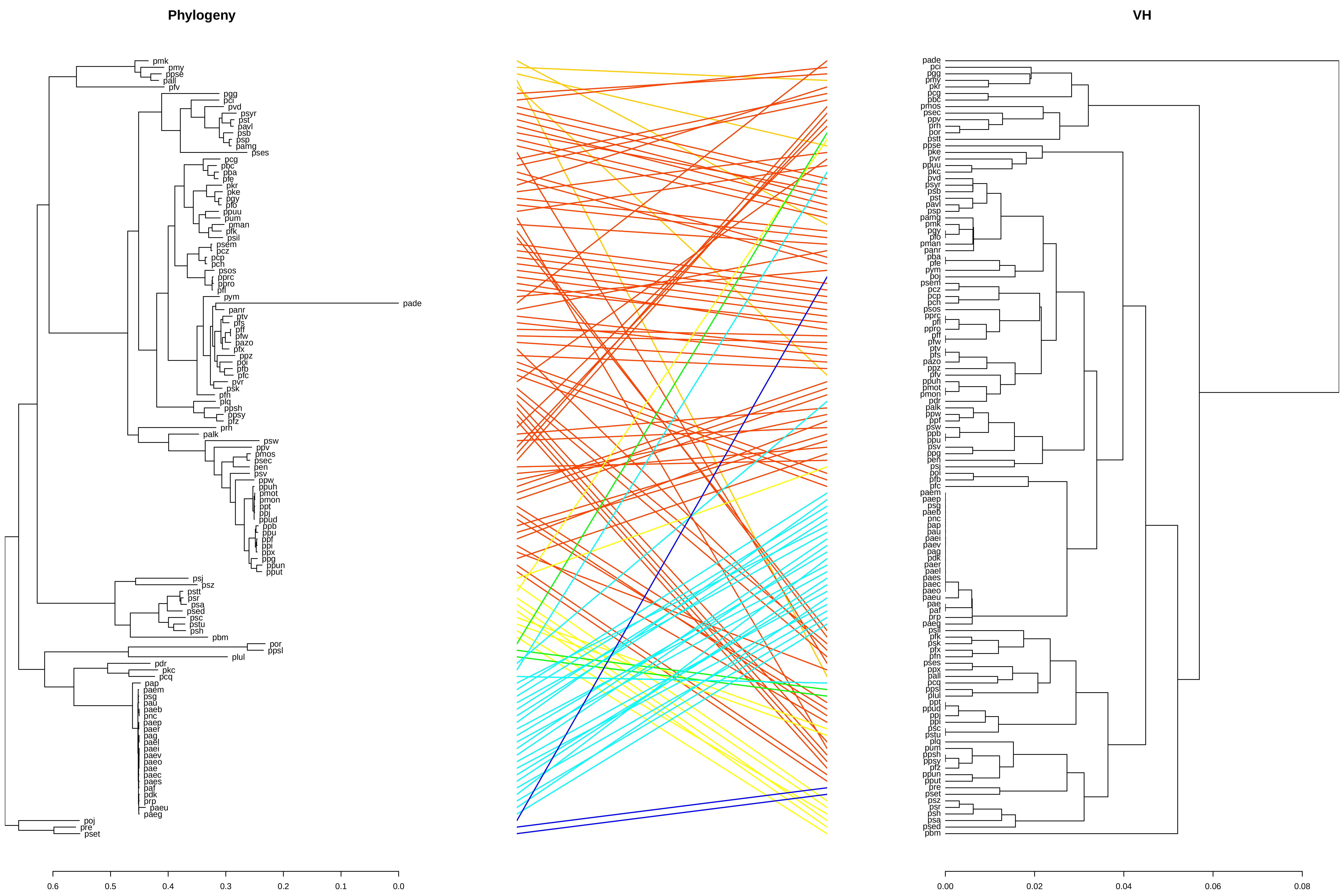
Phylogeny
VH
0.6
0.5
0.4
0.3
0.2
0.1
0.0
0.00
0.02
0.04
0.06
0.08

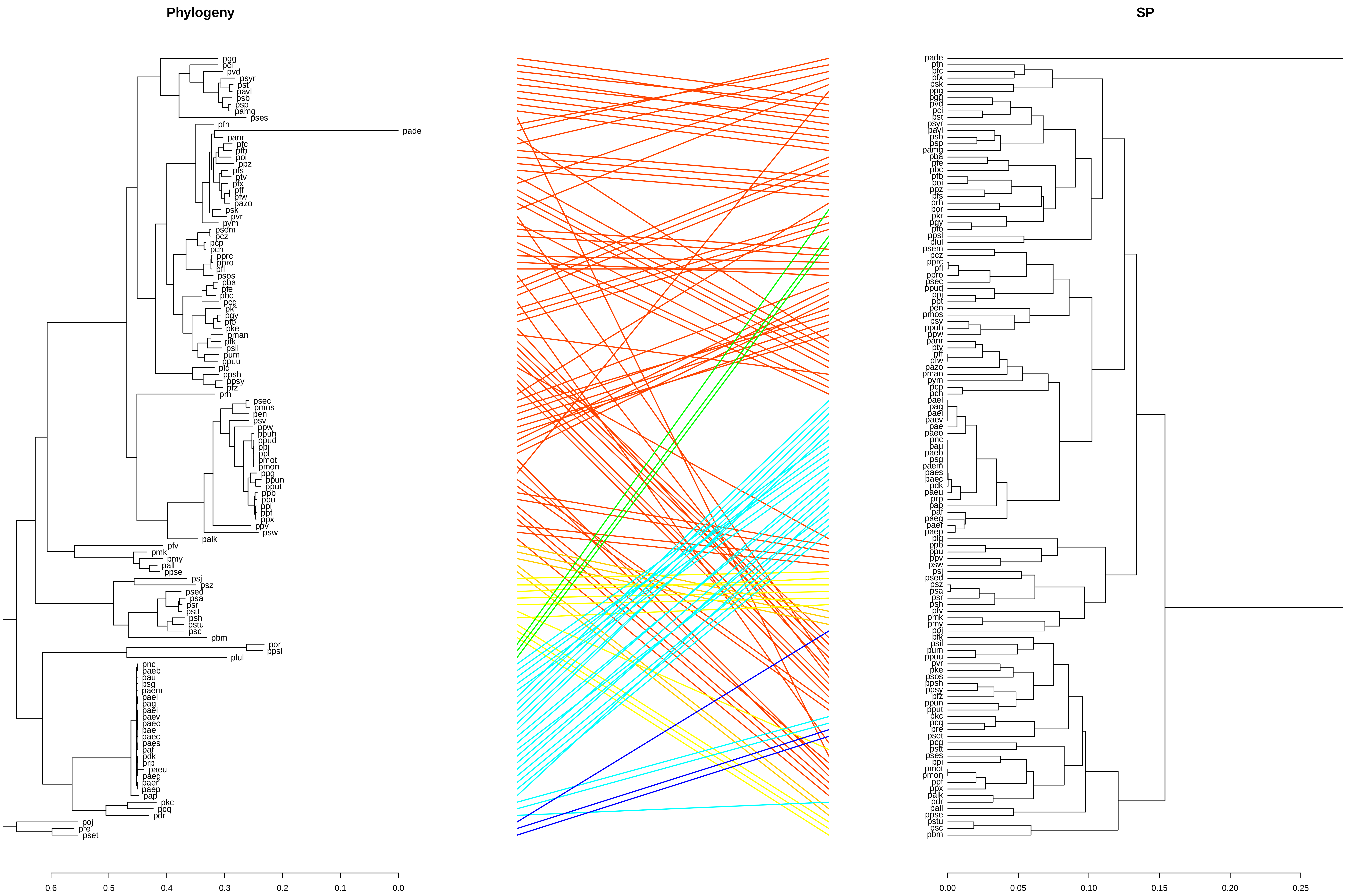

Phylogeny
SP
0.6
0.5
0.4
0.3
0.2
0.1
0.0
0.00
0.05
0.10
0.15
0.20
0.25

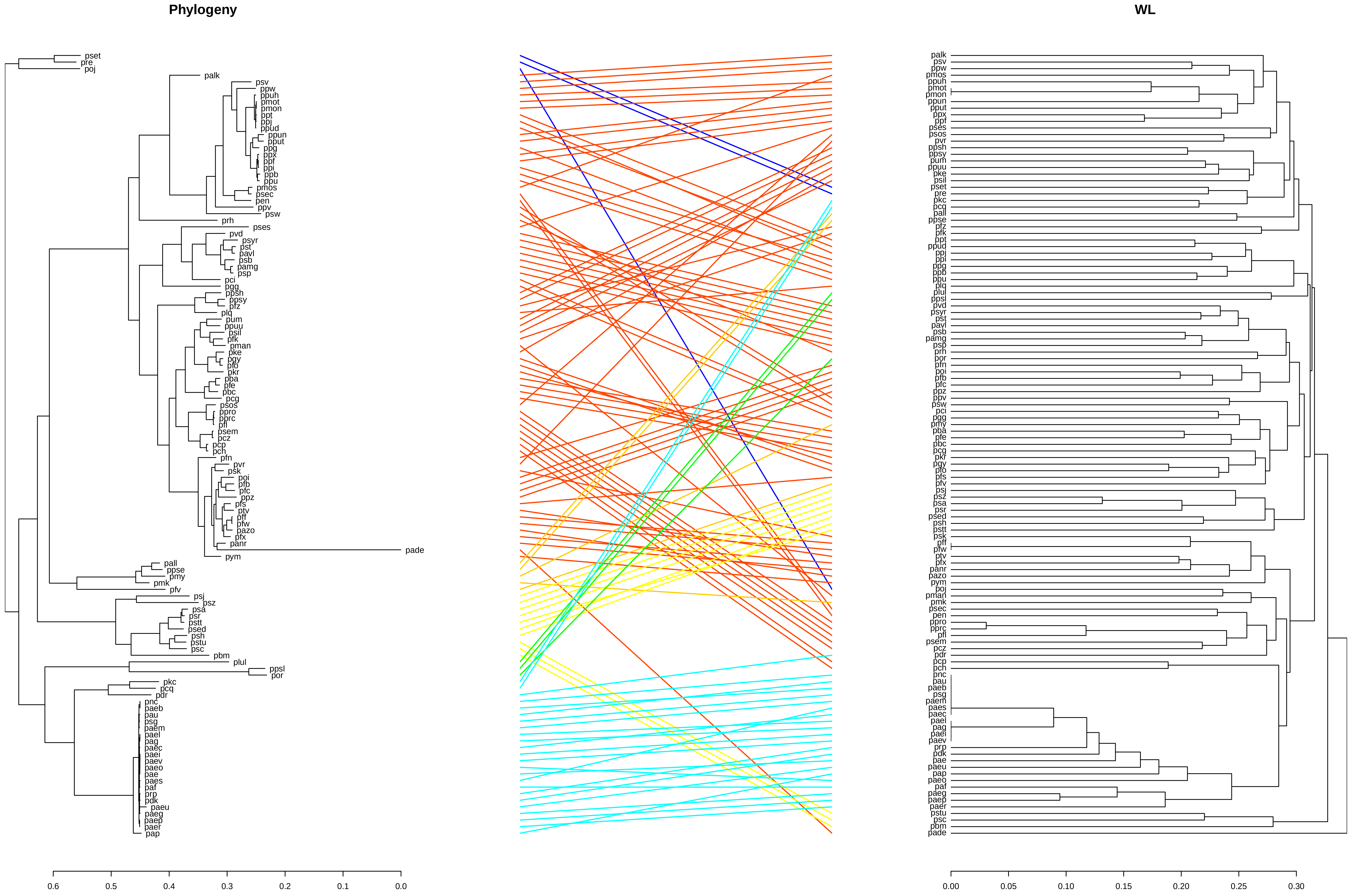
Phylogeny
WL
pset pre poj palk psv ppw ppuh pmot pmon ppt ppj ppud ppun pput ppg ppx ppf ppi ppb ppu pmos psec pen ppv psw prh pses pvd psyr pst pavl psb pamg psp pci pgg ppsh ppsy pfz plq pum ppuu psil pfk pman pke pgy pfo pkr pba pfe pbc pcg psos ppro pprc pfl psem pcz pcp pch pfn pvr psk poi pfb pfc ppz pfs ptv pff pfw pazo pfx panr pade pym pall ppse pmy pmk pfv psj psz psa psr pstt psed psh pstu psc pbm plul ppsl por pkc pcq pdr pnc paeb pau psg paem pael pag paec paei paev paeo pae paes paf prp pdk paeu paeg paep paer pap
palk psv ppw pmos ppuh pmot pmon ppun pput ppx ppf pses psos pvr ppsh ppsy pum ppuu pke psil pset pre pkc pcq pall ppse pfz pfk ppt ppud ppj ppi ppg ppb ppu plq plul ppsl pvd psyr pst pavl psb pamg psp prh por pfn poi pfb pfc ppz ppv psw pci pgg pmy pba pfe pbc pcg pkr pgy pfo pfs pfv psj psz psa psr psed psh pstt psk pff pfw ptv pfx panr pazo pym poj pman pmk psec pen ppro pprc pfl psem pcz pdr pcp pch pnc pau paeb psg paem paes paec pael pag paei paev prp pdk pae paeu pap paeo paf paeg paep paer pstu psc pbm pade
0.6 0.5 0.4 0.3 0.2 0.1 0.0
0.00 0.05 0.10 0.15 0.20 0.25 0.30

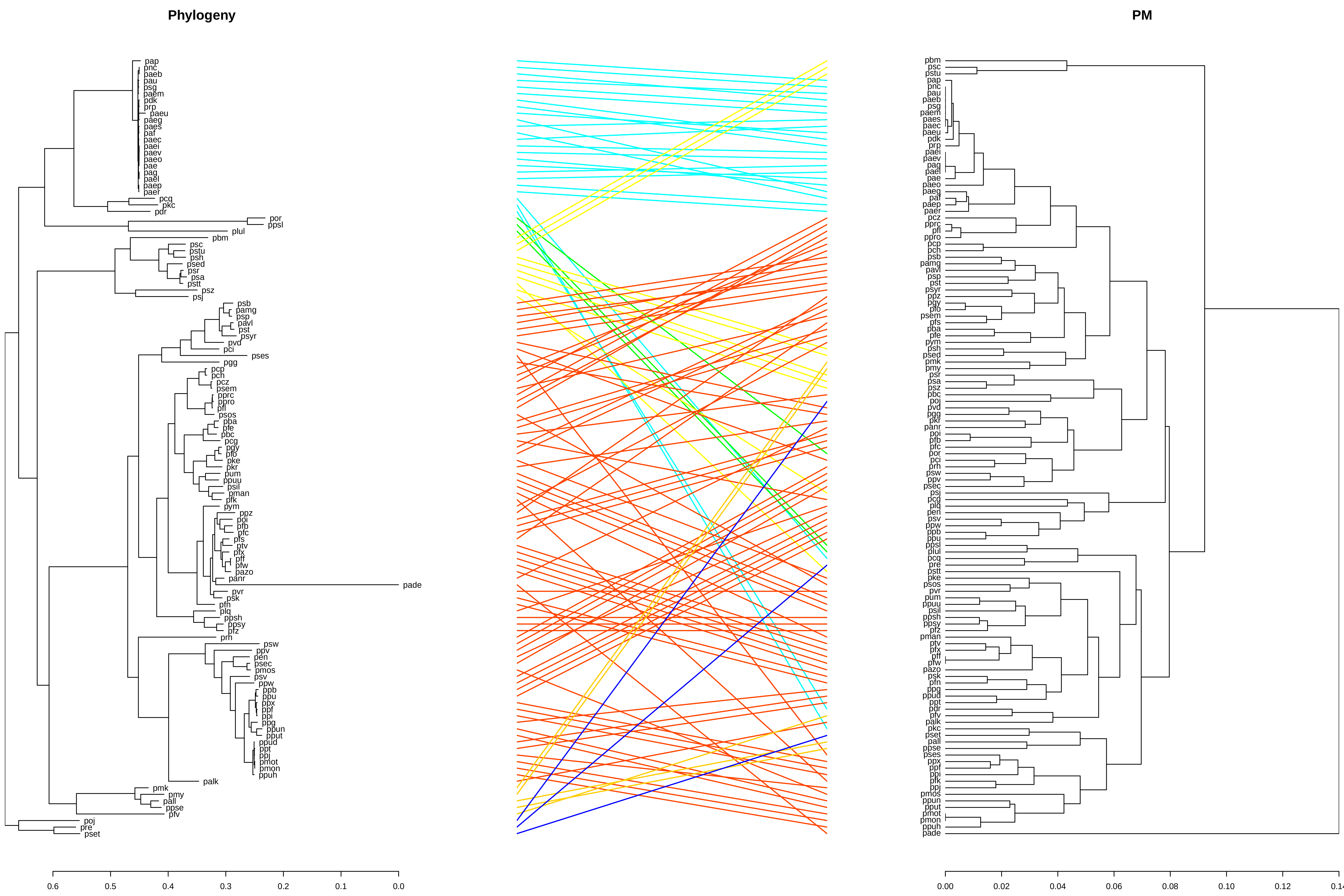
Phylogeny
PM
0.6
0.5
0.4
0.3
0.2
0.1
0.0
0.00
0.02
0.04
0.06
0.08
0.10
0.12
0.14

Pseudomonas Analysis -- three distance comparison Phylogeny vs Dendrogram

Graph Kernel	non filtered	Threshold 1	Threshold 2
PM	0.7144284	0.6249406	0.6552115
SP	0.6331594	0.6012156	0.6335984
VH	0.6298671	0.6312053	0.6603908
WL2	0.5296399	0.5339407	0.5996786
